# Maxillofacial Injuries as Markers of Interpersonal Violence in Belo Horizonte-Brazil: Analysis of the Socio-Spatial Vulnerability of the Location of Victim’s Residences

**DOI:** 10.1371/journal.pone.0134577

**Published:** 2015-08-14

**Authors:** Carlos José de Paula Silva, Ana Clara Mourão Moura, Paula Cristina Pelli Paiva, Raquel Conceição Ferreira, Rafaella Almeida Silvestrini, Andréa Maria Duarte Vargas, Liliam Pacheco Pinto de Paula, Marcelo Drummond Naves, Efigênia Ferreira e Ferreira

**Affiliations:** 1 Department of Dentistry, Federal University of Jequitinhonha and Mucuri Valleys, Diamantina, Minas Gerais, Brazil; 2 Laboratory of Geographic Information System, School of Architecture, Federal University of Minas Gerais Belo Horizonte, Minas Gerais, Brazil; 3 Department of Oral Public Health, School of Dentistry, Federal University of Minas Gerais, Belo Horizonte, Minas Gerais, Brazil; 4 Department of Spatial Statistics and Geostatistics, University of Belo Horizonte, Belo Horizonte, Minas Gerais, Brazil; 5 Department of Psychology, Catholic University of Minas, Belo Horizonte, Minas Gerais, Brazil; 6 Department of Oral and Maxillofacial Surgery, School of Dentistry, Federal University of Minas, Belo Horizonte, Minas Gerais, Brazil; Örebro University, SWEDEN

## Abstract

The aim of the present study was to analyze the spatial pattern of cases of maxillofacial injuries caused by interpersonal violence, based on the location of the victim’s residence, and to investigate the existence of conditions of socio-spatial vulnerability in these areas. This is a cross-sectional study, using the data of victims attended in three emergency hospitals in Belo Horizonte-Brazil between January 2008 and December 2010. Based on the process of spatial signature, the socio-spatial condition of the victims was identified according to data from census tracts. The spatial distribution trends of the addresses of victims were analyzed using Kernel maps and Ripley’s K function. Multicriteria analysis was used to analyze the territorial insertion of victims, using a combination of variables to obtain the degree of socio-spatial vulnerability. The residences of the victims were distributed in an aggregated manner in urban areas, with a confidence level of 99%. The highest densities were found in areas of unfavorable socioeconomic conditions and, to a lesser extent, areas with worse residential and neighborhood infrastructure. Spatial clusters of households formed in slums with a significant level of socio-spatial vulnerability. Explanations of the living conditions in segregated urban areas and analysis of the concentration of more vulnerable populations should be a priority in the development of public health and safety policies.

## Introduction

Violence in Brazil has become increasingly evident and causes alarm among many sectors of society. Manifestations of violence are often liked to poverty, social inequality and urban segregation [1–10], which appear to be explanatory elements in the social profile of both victims and aggressors. As a social phenomenon that can result from multiple causes, violence should be addressed from different perspectives and with the use of appropriate methods. Thus, researchers in different fields of knowledge have sought to understand how and in what social contexts violence emerges.

Considering the significant increase in violence, Brazilian society seems to be undergoing a substantial transformation in the forms of interactions among individuals, who experience isolation, fear and a lack of security. These characteristics are more readily seen in larger cities. In Belo Horizonte, which is the capital of the state of Minas Gerais, violent crime increased more than 11% between 2010 and 2011. In absolute numbers, 17,369 cases of the most varied forms of violence were recorded in 2010 [11]. Among such cases, the homicide rate reached as high as 30.1/100,000 inhabitants, surpassing the rate in other capital cities in the same region of the country, such as Rio de Janeiro and São Paulo, for which the homicide rate was 23.5/100,00 and 10.4/100,000 [12].

The abovementioned cases of violence could be the result of the interaction of individual attributes and a set of variables that permeate the urban context, including political, institutional, social and economic factors [13]. Thus, urban areas serve as a mediator in relationship between individuals and society, producing social problems, segregation and violence [6, 14]. Studies have highlighted that contextual aspects may explain the heterogeneous distribution of violence and victims in different areas of the city [13]. Studies have reported that the use of Geographic Information Systems could help to explain the phenomena by combining cartographic and epidemiological data [15–17].

Violence can materialize in many forms, the most common of which are cases of physical aggression resulting from interpersonal violence. It is estimated that for every homicide recorded, approximately ten times more non-fatal cases of physical aggression occur, many of which are not even recorded by public health and safety authorities [18].

Aggressive strikes to the face can cause a specific type of injury known as maxillofacial injury. Studies indicate that this type of injury has increased significantly in cases of interpersonal violence mainly due to fact that this is the most exposed, unprotected part of the body. Maxillofacial injuries often have emotional and functional repercussions [19, 20]. The selection of this part of the body as the focus of the aggression could be motivated by the role the face plays in interactions between individuals and the conveyance of emotions. This area of the body is one of the most unique characteristics of an individual and is the part of the body that most represents one’s identity [21]. According to Tucherman [22], the relationship between identity and the human face is so close that mutilation of this area is known as disfiguration, since it marks an attempt to destroy that which most represents the individual.

Using such a singular type of injury as a marker of violence could reveal a modality of insidious violence, which is often repeated silently without getting attention, and may represent the starting point for a fatal outcome. Therefore, the aim of the present study was to analyze the spatial pattern of cases of maxillofacial injuries caused by interpersonal violence, based on the location of the victim’s residence, and to investigate the existence of socio-spatial vulnerability in these areas.

## Materials and Methods

### Study Area

The present study was conducted in the city of Belo Horizonte, capital city of the state of Minas Gerais. The city is located in the southeast of Brazil and has 2,375,151 inhabitants. Together with São Paulo and Rio de Janeiro, Belo Horizonte is part of the most important economic hub in Brazil [23].

### Location of data collection

This is a cross-sectional study developed using secondary data collected from the Traumatology and Surgery services of three public hospitals that specialize in medium to highly complex injury and are a reference in Belo Horizonte in terms of treating victims of maxillofacial injuries. Data were collected from the Odilon Behrens Hospital, the Pronto Socorro João XXIII Hospital and the Maria Amélia Lins Hospital.

### Characteristics of the study

The present study included male and female victims of all ages. The records of fatal and non-fatal victims attended to in Belo Horizonte hospitals between January 2008 and December 2010 were analyzed. All cases of maxillofacial injuries were included, regardless of whether they were associated with injuries in other parts of the body. A single researcher extracted the data from the medical records and transcribed them to a form designed specifically for the present study. The data were collected between October and December of the three years included in the research (2008, 2009 and 2010).

Cases stemming from interpersonal violence were considered relevant to the study and were grouped as follows: aggression using a part of the body such as slapping, punching or kicking; aggression with a firearm, such as a revolver or pistol; aggression with a cutting weapon such as knife or dagger; and aggression using other means such as rock, iron rod, bottle, glass or blunt object.

### Exclusion criteria

The following types of cases were excluded from the research: accidental discharge of a firearm; domestic accidents; suicide attempts; falls from a height and falls not caused by interpersonal violence; victims who did not reside in the city of Belo Horizonte.

### Organization of spatial databases

The cases were recorded on a map by georeferencing, adopting the victim’s address as the reference point. The georeferencing was conducted through geocoding associated with an alphanumeric table containing the addresses of the households and a digital map base. A cartographic address base was adopted from the Empresa de Informática e Informação of Belo Horizonte (PRODABEL), containing sections of roads with the initial and final numbers of each block, separated according to the left and right sides of each stretch of all streets and avenues in the city. The socio-demographic characterization was conducted based on the census sectors scale of the Instituto Brasileiro de Geografia e Estatística [23]. This was done by associating alphanumerical tables containing the variables of interest and the cartographic basis of the census sectors of the city. The socio-demographic variables used were related to the Brazilian demographic census conducted in 2010 [23]. A system of projections and UTM coordinates was adopted to structure the information plans and South American Datum 69 was used for the 23S time zone (S1 Data).

### Analysis of the randomness of specific standards

The levels of spatial aggregation and the models of the density of points were analyzed. Ripley’s K function [24] was used during the analysis of the level of aggregation to examine cases in an aggregate, random or regular manner. When the data exhibited a pattern of spatial aggregation, the data curve was above the confidence envelope. The statistical significance of the test was confirmed by Monte Carlo simulations, with 99% confidence intervals. In the test involving Ripley’s K function, a distance or area of influence of 3000 meters was adopted [25, 26].

### Analysis of the density of points

Kernel’s function was used to investigate the spatial density of the cases [25]. Kernel density is a method of spatial interpolation that provides estimates of the intensity or density of the points along the entire surface. This enables the identification of regions of greater aggregation, also known as hotspots. The density of cases was obtained through Kernel analysis, which was weighted for the socio-demographic variables of the points analyzed. The radius of influence used was 500 meters [25–27]. A spatial resolution with pixel size of 25 x 25 meters was defined for the raster maps.

### Variables

Socio-demographic indicators cited in the literature an important to the relationship between violence and both poverty and spatial segregation were selected. The variables that most often contributed to the explanation of this phenomenon were identified. As the aim of the present study was to analyze the existence of socio-spatial vulnerability, variables that evidenced poorer socioeconomic indicators, residential infrastructure and neighborhood infrastructure of the residences of victims were highlighted [8, 17, 28–32]. The following were analyzed: percentage of homes with eight residents; percentage of homes with no monthly income; percentage of heads of households with no monthly income; percentage of homes with no exclusive bathroom or with no bathroom; percentage of homes with electricity of an unknown origin and unofficial connection; percentage of homes with no connection to the water supply or sewage supply and no trash pickup; percentage of homes with no streetlights in the neighborhood; and percentage of homes with open-air sewage in the neighborhood. For variables related to income, the minimum monthly salary in the period (US$ 290) was used for the basis of analysis [23].

### Multi-criteria analysis

Multi-criteria analysis is a procedure that combines variables using an algebra map process and weighted means [27]. The most significant socio-demographic variables were associated and grouped within their respective analysis categories. These were then combined to compose the synthesis maps, defined as the synthesis of socioeconomic conditions, of the infrastructure of the households and the neighborhoods. Each variable was represented on an information plan (matrix map) and received a weight according to the degree of significance in terms of the composition of the final synthesis. Therefore, the variables received a weight according to their degree of interference in the socio-spatial vulnerability conditions in the area of the victim’s homes.

Once the residences of the victims had been marked in the territory and the layers of data related to the variables (socioeconomic condition, infrastructure of the residences and the neighborhoods) had been confirmed, the spatial signature process was initiated [33]. This process enables a characterization of each unit record (address of the victim’s home) with its spatial conditions, recorded as attributes for the census sector in the area. After the signature, the most significant variables, in terms of the vulnerability of victims, were identified. This process is known as a Data Driven Evaluation, which investigates the course of events based on its variable components.

Once the hierarchy of the variables was clarified according to their representations at the investigation points by the signature process, this hierarchy was translated into weights by a heuristic procedure: a bibliographic review and a Knowledge Driven Evaluation, based on the opinion of experts. In order to maintain maximal equilibrium and pertinence between the variables and their respective weights, a specialist in urbanism and a specialist in public health indicated the weights. Heuristics is defined as a method of problem-solving through successive approximation. In this process, the possibility of inappropriate weighting is the inverse of the number of weights attributed [27]. Thus, a first approximation was performed by the spatial signature and the final attribution of weights for the multi-criteria analysis was based on expert opinion.

**Fig 1:**
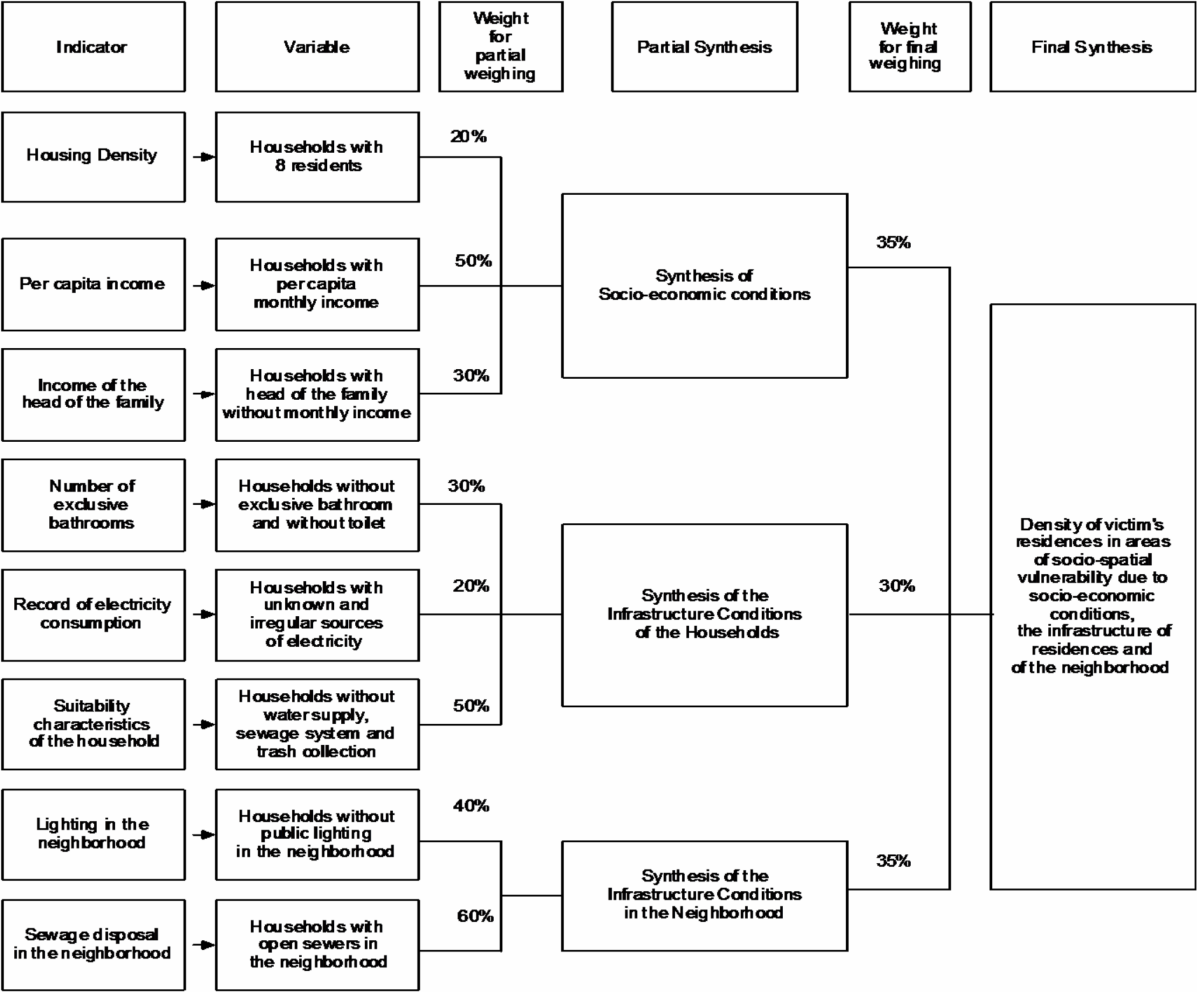
Decision tree in the assessment of the synthesis of socio-spatial vulnerability of victims of maxillofacial injuries caused by interpersonal violence, according to the location of their residence. Belo Horizonte, January 2008 to December 2010.

Fig 1 displays the decision tree that constituted the analysis of multi-criteria, according to the analysis of socioeconomic condition, the infrastructure of the residences and the infrastructure of the neighborhood. The sum of the weights of the plans involved should equal 100%, which defines the degree of pertinence of each variable. The weight of each variable, represented in information plans, was attributed through a heuristic approach, initially supported by a bibliographic review to identify the most important variables when characterizing conditions of socio-spatial vulnerability.

As well as the weight of each variable or information plan, grades (from 0 to 10) were also attributed for the components of the legend of each map, indicating the degree of pertinence for the phenomenon investigated (socio-spatial vulnerability). The variables combined were quantitative and indicated the percentage of households or the percentage of people who exhibited a certain characteristic. The maps were structured with five components portraying the lowest to the highest ranges (high, upper middle, middle, lower middle and low), which respectively received a score of 10, 7, 5, 3 and 1. This was performed for all of the maps or information plans.

Five bands were chosen due to the principles of graphical semiology and the analysis of mapping data. Five is a logical number for humans to perform syntheses and rank their analysis results. This scale avoids excessive simplification (high, medium and low) and excessive information (a higher number of bands are difficult to compose on the mental map) [34]. It is also recommended that the division of the map classes into five legend components should not involve bands of large concentrations or large absences of occurrences, which led to the selection of the natural breaks method.

Weights were attributed to the variables or information plans and grades were given to their respective legend components. Integration was performed through advanced analysis, which is the application of the weighted mean in the algebra of maps, according to the rule:

$$Aij\;=\;\sum_{k=1}^{n}\left(Pk\;x\;Nk\right)$$

Given that:

I–Lines of the matrix

J–Columns of the matrix

Sum of 1 to n matrices of information plans

P–Weight attributed to each matrix or information plan

N–Grade attributed to each cell of the matrix or information plan

R software (version 2.15.1) was used to analyze the spatial pattern. ArcGis software (version 9.3) was used to investigate the spatial density of cases and advanced analysis.

### Ethical Aspects

The present study was approved by the Ethics Research Committee of the Universidade Federal de Minas Gerais (ETIC 352/ 08), in association with the Hospital Odilon Behrens (352/ 08) and the Fundação Hospitalar do Estado de Minas Gerais (CEP 125/ 08). The secondary data used in this research come from routine clinical care records at different times of the year. In such cases, the hospitals do not require consent from patients. However, to ensure confidentiality and anonymity, the Ethics Committee determined that the names and registration numbers of the victims would be deleted before analysis.

## Results

**Fig 2:**
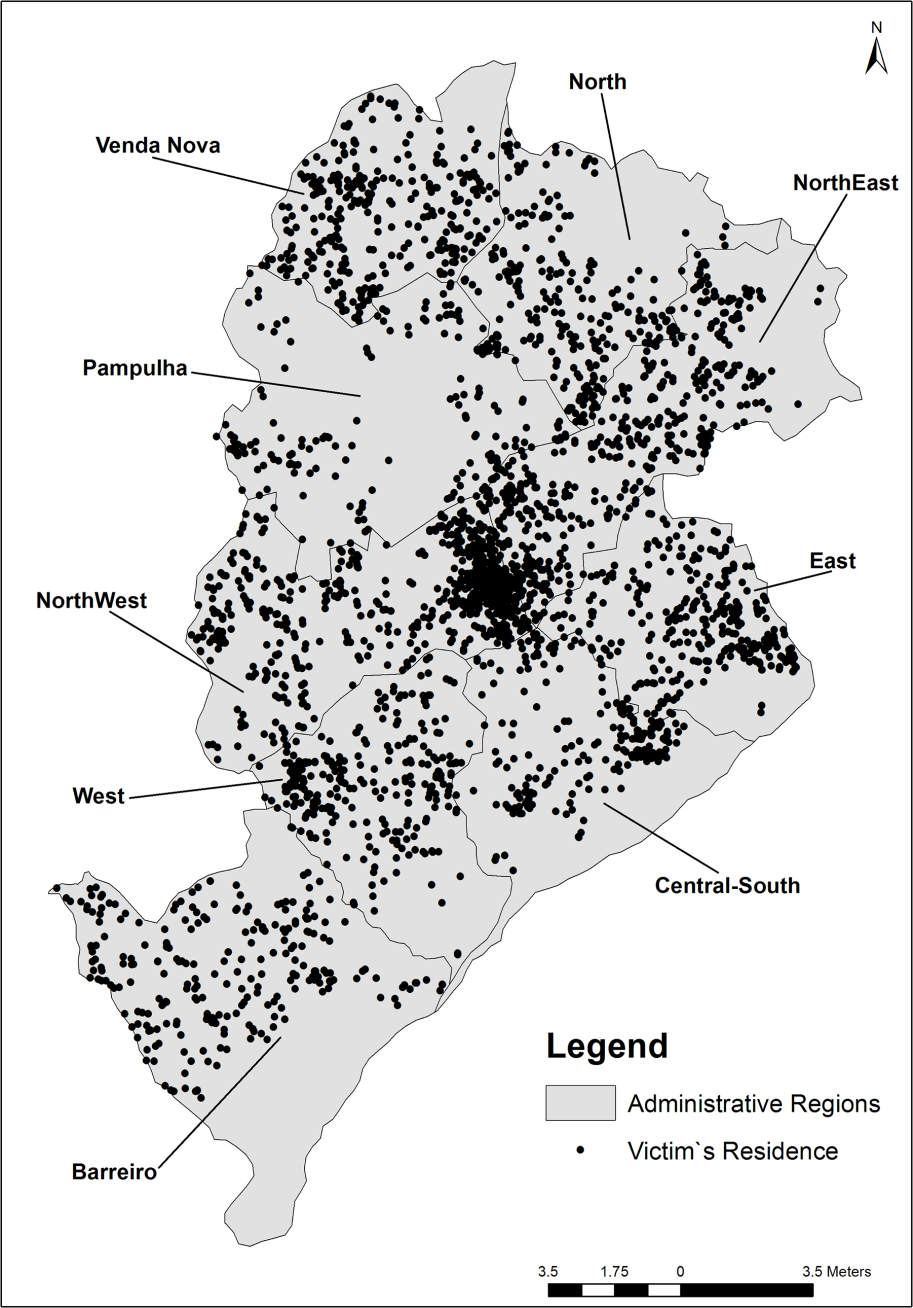
Distribution of cases of maxillofacial injuries caused by interpersonal violence according to the victim’s residence. Belo Horizonte- Brazil, January 2008 to December 2010.

In total, 3,202 records of victims of maxillofacial injuries caused by interpersonal violence were found. From the total number, 63 cases were excluded from the study due to inconsistent data for the addresses of victims. The losses represented 1.97% of the cases, which was considered acceptable for the development of the study. Fig 2 displays the distribution of cases according to the location of the victim’s residence.

**Fig 3:**
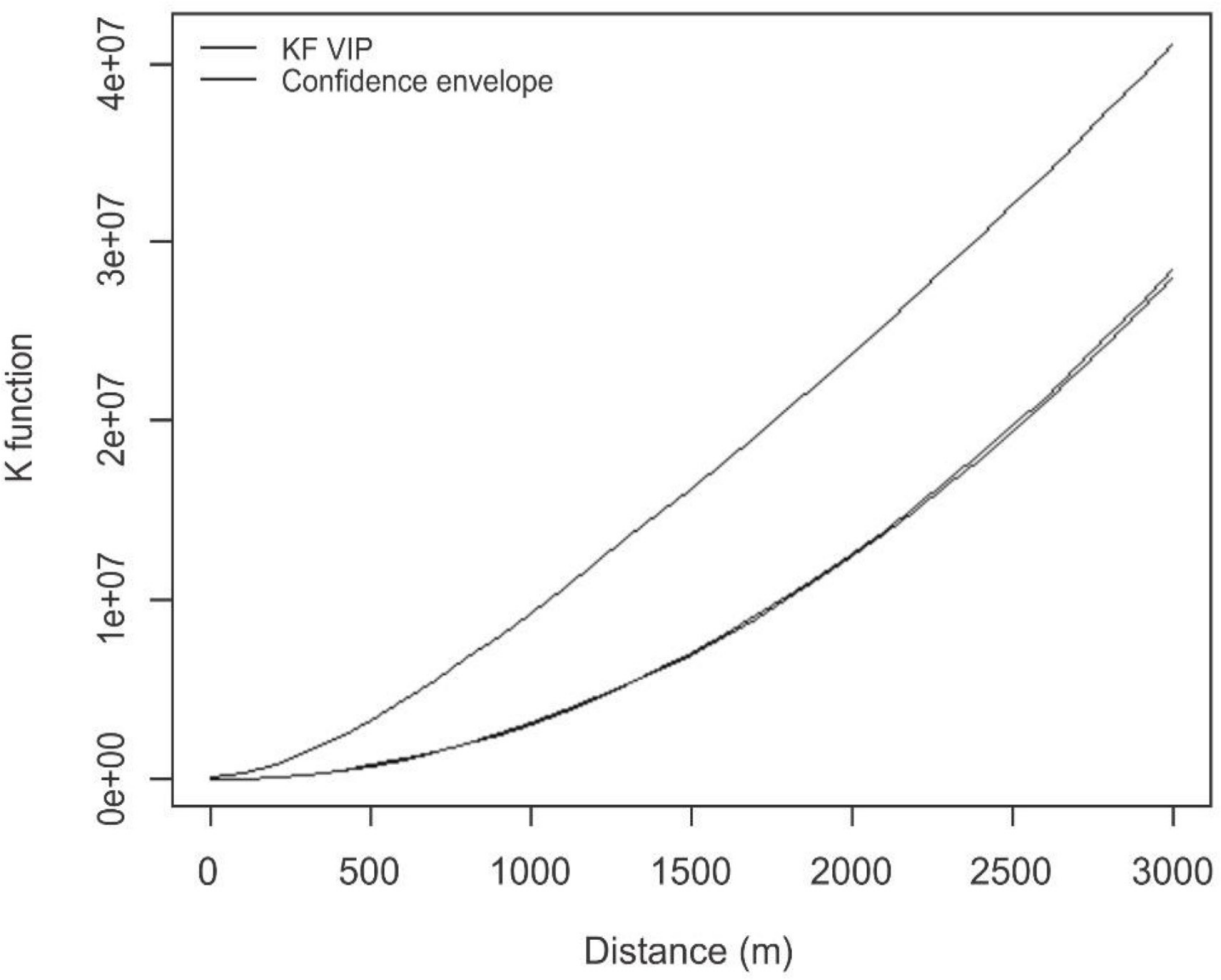
K function of victims of maxillofacial injuries caused by interpersonal violence according to the location of their residence. Belo Horizonte-Brazil, January 2008 to December 2010.

Fig 3 displays the results of Ripley’s K function for the randomness test. Considering a 99% confidence interval, the homes of the victims exhibited an aggregated spatial pattern, as the curve of the data remained above the confidence envelope. Thus, the test revealed that the distribution of the residences of victims did not occur randomly and clusters formed in specific areas of the city.

**Fig 4:**
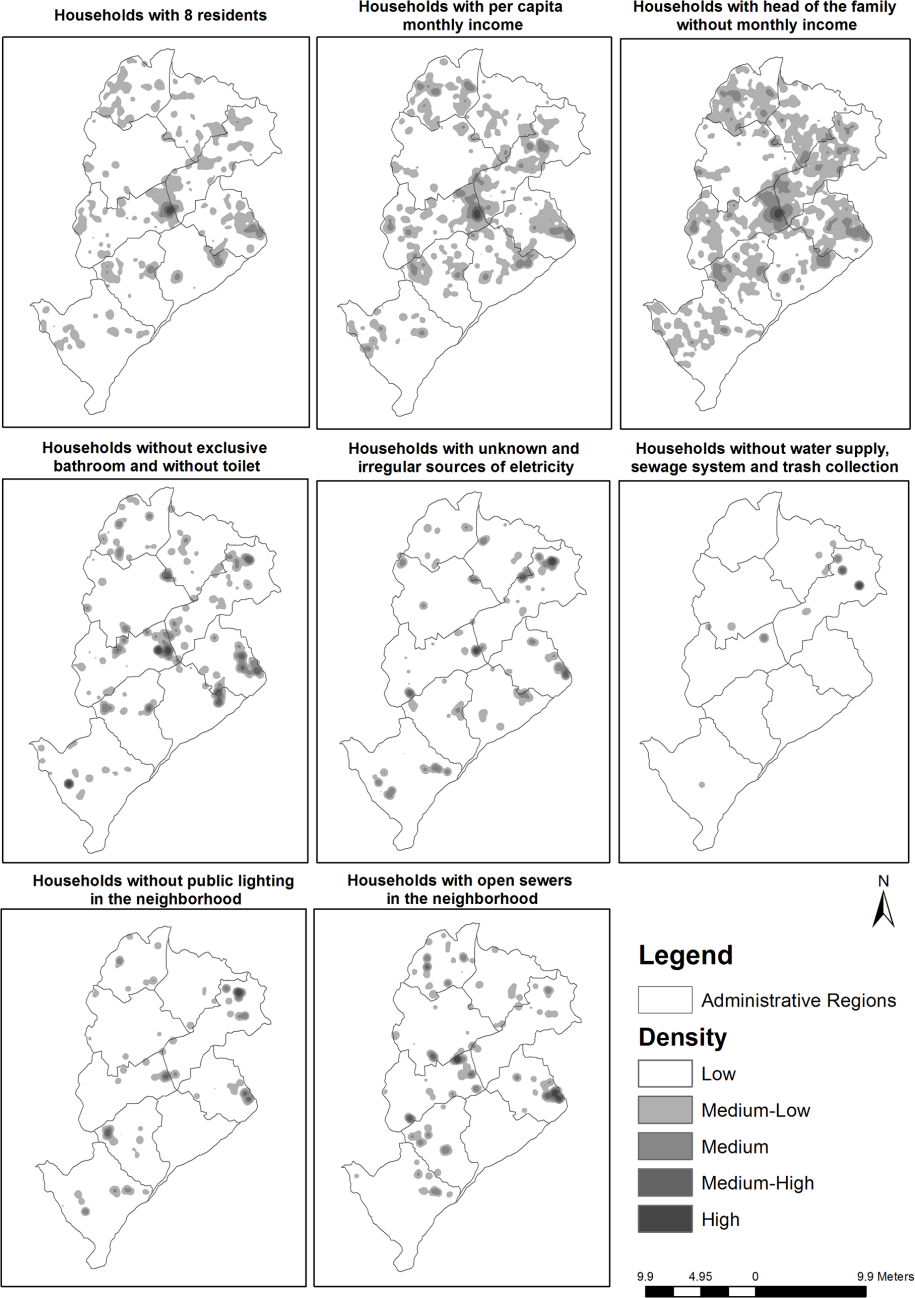
Concentration of victims of maxillofacial injuries caused by interpersonal violence, weighted for socioeconomic variables, the infrastructure of the residences and the infrastructure of the neighborhoods, according to the location of the residence. Belo Horizonte, January 2008 to December 2010.

Fig 4 displays the variables that composed the levels of information used in the multi-criteria analysis. The variables analyzed exhibited a high density of cases in which the victims lived in a specific and spatially well-defined region. These were mainly located in the following areas of the city: Barreiro; the West; the Northwest and the Northeast. The only variable that typified households with the worst characteristics of suitability (no water supply, no sewage system and no trash collection) exhibited the formation of isolated clusters in peripheral areas of the city. With regards to most of the variables studied, the Pedreira Prado Lopes slum concentrated the most significant cluster.

**Fig 5:**
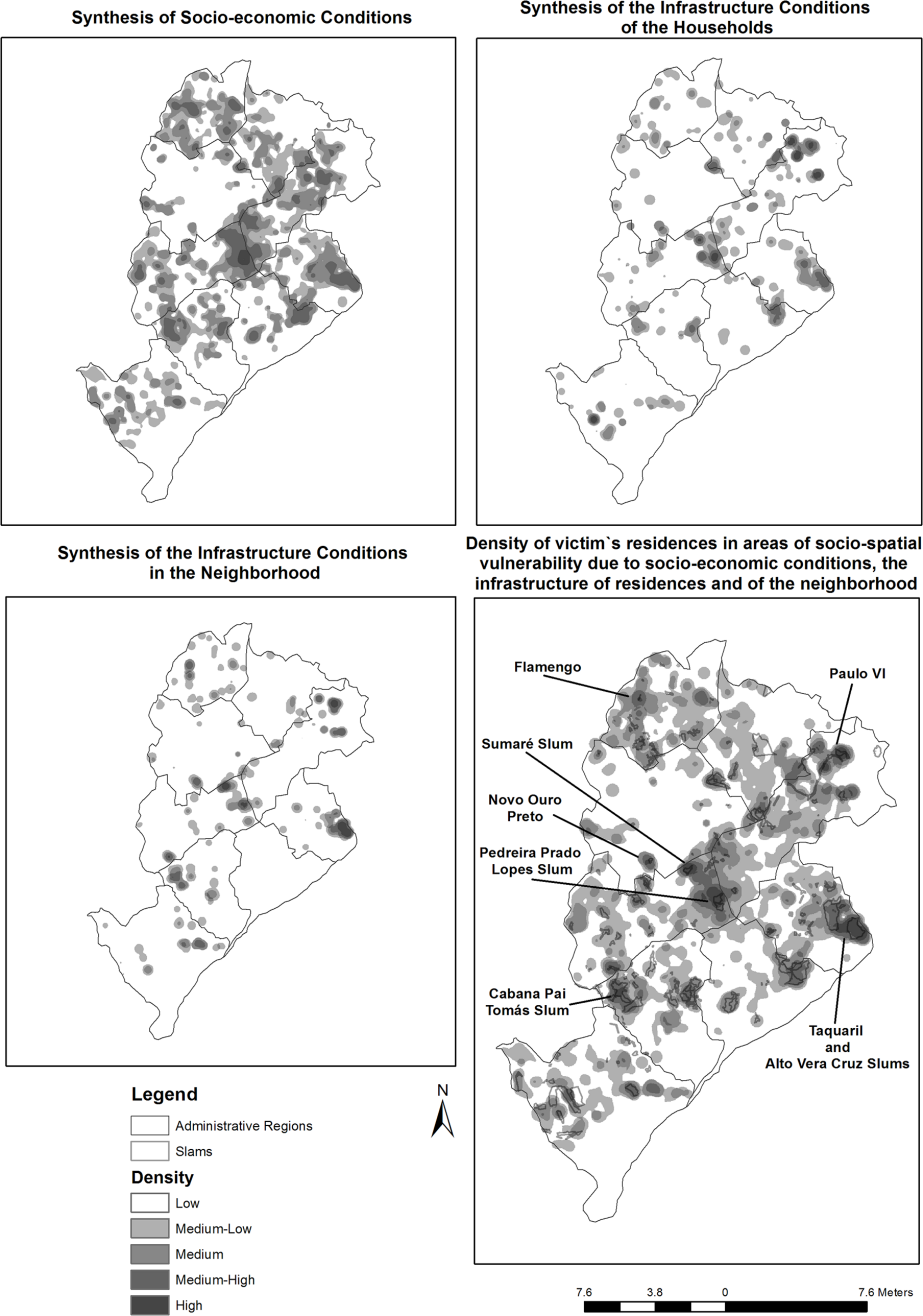
Synthesis maps of socioeconomic conditions, the infrastructure of the residences and the infrastructure of the neighborhoods and the final synthesis of victims of interpersonal violence, weighted for conditions of socio-spatial vulnerability in the location of the residence. Belo Horizonte, January 2008 to December 2010.

Fig 5 displays the synthesis maps of socioeconomic conditions, the infrastructure of residences and the infrastructure of neighborhoods based on the residences of the victims. The variable that best characterized the profile of the location of the residence was a low socioeconomic condition, which was most relevant in the following areas of the city: the Northwest, the West, the East, the Northeast and Venda Nova. Fig 5 also displays the final synthesis map of the concentration of victims of interpersonal violence, weighted for the socio-spatial vulnerability conditions of the location of the residence. This map revealed the formation of clusters of high socio-spatial vulnerability in seven regions of the city: the Northwest (Pedreira Prado Lopes and Sumaré slums); the East (Alto Vera Cruz and Taquaril slums); the Northeast (Paulo VI and Vila Maria); the West (Cabana Pai Tomás slum); Venda Nova (Flamengo); the North (Ribeiro de Abreu) and Pampulha (Novo Ouro Preto).

## Discussion

When investigating cases based on the location of the residence of victims, bearing in mind that they were not necessarily the location of the injury event, the authors of the present study intentionally used non-traditional methods to approach violence. The exclusive analysis of the location of the event is often only associated with criminal behavior, regardless of the social profile of the victims. The proposed approach allowed the investigation of possible exposure factors on the part of victims based on local living conditions. This was significant because the victim was included in a context permeated by numerous attributes of a family, social, community, economic, historical and cultural nature, which can obviously effect or model individual and collective components of behavior or exposure. These attributes, acting either alone or in conjunction, can contribute to the resolution of interpersonal conflicts, serving as a protection factor or, in the worst of hypotheses, as a risk factor for violence.

This argument is based on several studies developed in Brazil and in other countries that considered the location of the residence to be a powerful indicator during investigations of the determining factors of a population’s health [2,29,35–40]. The results of the study seem to confirm the importance of the adoption of this type of data. The randomness test indicated that the homes were distributed in an aggregated and unequal fashion in the city, demonstrating that the victims lived in areas with a certain degree of specificity. It is possible that these areas contain components that have contributed, to a greater or lesser degree, to the materialization of cases of maxillofacial injury caused by interpersonal violence.

The results of the analysis of density in Fig 4 reinforce the issues raised above. The concentration of residences of victims, weighted for socioeconomic variables, the infrastructure of the residences and of the neighborhood, clearly revealed a sui generis spatial pattern. The areas that exhibited a convergence of greater density were associated with a number of basic variables of infrastructure: open sewers in the neighborhood; no public lighting; the absence of exclusive bathrooms; a precarious and irregular electricity supply, portraying a polarization of cases in well-defined areas. A number of studies have indicated that areas with greater levels of disorder and degradation are more prone to the occurrence of violence and a larger concentration of victims [28, 31].

As seen, habitation density, household income and income of the head of the household seemed to contribute evenly to the formation of clusters in the same regions. Numerous studies indicate that these variables can express diminished affection and an increase in stress among family members caused by various economic limitations and uncertainties [3, 8, 29, 31]. According to the authors, a high number of residents per household can increase the risk of economic deprivation and family instability. Furthermore, they highlight the possibility of households in which the head of the family does not earn enough to sustain the family and consequently, their authority is questioned, generating a tense environment prone to episodes of violence and the disruption of family cohesion.

The analysis of socioeconomic variables, residential infrastructure and neighborhood infrastructure revealed the importance of these indicators to the characterization of the place of residence of the victims. The spatial patterns found in the study showed that the factors related to these variables were more significant, from a spatial point of view, than the infrastructure conditions of the residences and neighborhoods, with more scope in the territory. Based on the results of the present study, the worst infrastructure conditions of the residences and neighborhoods seem to have exerted less influence on the characterization than the location of the victim’s residence. The greater densities exhibited a reduced scope in the configuration of urban space. This could be explained by the transformation that has occurred in the city in recent years, through government interventions related to urbanization, social inclusion policies and the regularization of precarious urban settlements, as well as interventions in the road system, improvements in housing and better basic sanitation [27].

In terms of the analysis of the conditions of victimization, the authors of the present study agree with the findings of Adorno [41] and Barbosa et al. [32]. These authors considered that areas with socioeconomic inequality, allied to segregation and social exclusion, exhibit higher numbers of violent cases due to the deterioration of physical and social infrastructure. In this context, one may experience difficulty in protecting oneself from violent events and influencing aggressive behavior.

Although the present study addressed interpersonal violence as a unique entity, it is important to highlight that several forms of aggression were grouped within this entity (aggression using part of the body, firearm or cutting weapon or other object) and victims of all ages and both genders were considered. Aggression probably occurs for different reasons and can be potentiated by different relational mechanisms. However, all of the elements cited possess the same context and characteristics of the community where the victim resides. Several studies have recognized the effect of context on the health of individuals, behavior patterns and socialization, particularly when associated with factors such as poverty, crime and social disorganization [2, 6, 29, 35–40].

The analysis of socioeconomic conditions, the infrastructure of the residences and the infrastructure of the neighborhoods and the final synthesis of victims of interpersonal violence, weighted for conditions of socio-spatial vulnerability in the location of the residence shows that the context may have been important in the profile of victimization. When considering the conditions of greater socio-spatial vulnerability, clusters are confirmed in pockets of poverty, revealing the existence of social groups that are vulnerable to the type of event analyzed. Clusters formed in some regions characterized by the presence of shanties. Some of these areas have been indicated in previous studies as locations of many types of violence and criminality including aggression and homicide [30,42,43]. Based on the results of the present study, these areas exhibit homogenous social, economic and infrastructure characteristics and contain a high density of victims of interpersonal violence.

However, when analyzing the socioeconomic variables, residential infrastructure and neighborhood infrastructure, the concentration of cases did not indistinctly affect all areas with the same urban characteristics. The fact that high densities were concentrated in only some of the shantytowns in the city reveals that the relationship between poverty and violence cannot be analyzed in an isolated manner. One must also consider the fact that violence is associated with different degrees of the overlap of socioeconomic needs and other implicit social factors rather than merely economic deprivation. This shows the complexity of the associations established between violence, poverty, social inequality and urban segregation. The mechanisms that mediate interpersonal conflicts may not exist, or may be reduced, in these areas. In addition, other harmful coexisting factors must be recognized as causes of social instability and violence.

With regards to the establishment of comparative analysis, other studies have identified that criminal activity associated with drug trafficking seems to be the most similar characteristic between slums and the location of victim’s residences. Beato Filho and Reis [30] reported that these slums are areas with a concentration of violent events such as aggression and homicide, which are associated with criminal activity related to drug trafficking. The presence of drug trafficking in these areas could explain the great concentration of victims of interpersonal violence. Beato Filho [44] synthesized the characteristics of violence in Belo Horizonte and reported that feelings of insecurity and fear are uniformly distributed, although the victimization is highly concentrated in small locations and among specific social groups. These groups are mainly formed by individuals who live in areas with social inequities and spatial segregation, which are stigmatized and controlled by organized crime connected to illegal drug trafficking. The connection between the high incidences of violence in areas controlled by drug traffickers is a consensus in many studies [6, 45, 46, 47].

The results of the present study suggest the formation of spatial agglomerates of victim’s residences in areas with unfavorable socio-demographic indicators. Furthermore, a combination of factors could, in association with these indicators, have found fertile ground for increased exposure to interpersonal violence and victimization in areas with a more vulnerable population. This in turn could have directly or indirectly contributed to the maintenance of the risk of maxillofacial injury. This fact demonstrates that such areas should receive financial and social investments and should be priority in public health and safety policies. In the present study, the spatial approach enabled fusion between the events, space and territory that could be the mediators between life in society and the types of violence manifested.

The present study has a number of limitations related to the nature of the data. Errors and imprecise data are common in the maintenance of hospital records. In addition, there may have been an issue related to underreporting cases. These records can be even more complex in cases of violence, which can involve a wide variety of causes, including gender issues, vulnerable people, gang wars, drug use, drug trafficking and homicides. A number of victims may have omitted the cause of the violence in their case or given a false address due to the fear of some form of retaliation from the aggressors, who in some cases accompany the victim in the hospital, or further investigation by public security authorities or the press. Another limitation resides in the fact only part of the universe of victims of interpersonal violence was analyzed, as the sample was restricted to victims of maxillofacial injury, with no investigation of victims of injury to other parts of the body. Another limitation too regards the absence of information on individual aspects of the victims.

It is also worth noting that, despite the fact that these are well-renowned public hospitals that specialize in maxillofacial injury, they may not tend to all victims of interpersonal violence. Some victims could have received care in private units and as such, were not identified in the present study.

## Conclusions

The location of the victim’s residence exhibited a pattern of spatial aggregation, revealing the existence of a polarization of cases in areas with socio-economic disadvantages and, to a lesser extent, areas with the worst infrastructure. A high density of victims of interpersonal violence was found in areas of considerable socio-spatial vulnerability. These areas were limited to a number of slums with a history of violence connected to drug trafficking. Understanding this dynamic could direct the efforts of managers to reducing violence and the impact of these events on the health of the population through public policies focusing on more vulnerable groups. Explanations of the living conditions in segregated urban areas, as well as analysis of the concentration of more vulnerable populations, should be prioritized during the development of these policies.

## Supporting Information

S1 Data Dataset for Study. (ZIP)
