# Supplementary material for: Maxillofacial Injuries as Markers of Interpersonal Violence in Belo Horizonte-Brazil: Analysis of the Socio-Spatial Vulnerability of the Location of Victim’s Residences
Source: PLoS One. 2015 Aug 14;10(8):e0134577. doi: 10.1371/journal.pone.0134577 (PMC4537271; doi:10.1371/journal.pone.0134577)
Supplement: S1 Data — (ZIP) [file pone.0134577.s001.zip › DATA_PLOS.pdf]

ID	CD_GEOCODI	TIPO	CD_GEOCODB	NM_BAIRRO	CD_GEOCODS
978	310620005620091	URBANO	310620005088	Boa Viagem	31062000562
979	310620005620092	URBANO	310620005083	Centro	31062000562
980	310620005620093	URBANO	310620005010	Floresta	31062000562
981	310620005620094	URBANO	310620005010	Floresta	31062000562
982	310620005620095	URBANO	310620005010	Floresta	31062000562
983	310620005620096	URBANO	310620005093	Nossa Senhora da A	31062000562
984	310620005620097	URBANO	310620005093	Nossa Senhora da A	31062000562
985	310620005620415	URBANO	310620005090	Santo Agostinho	31062000562
986	310620005620416	URBANO	310620005102	Santa L·cia	31062000562
987	310620005620417	URBANO	310620005102	Santa L·cia	31062000562
2097	310620005620062	URBANO	310620005098	Serra	31062000562
2098	310620005620063	URBANO	310620005098	Serra	31062000562
2099	310620005620064	URBANO	310620005098	Serra	31062000562
2100	310620005620065	URBANO	310620005098	Serra	31062000562
2101	310620005620066	URBANO	310620005098	Serra	31062000562
2102	310620005620067	URBANO	310620005098	Serra	31062000562
2103	310620005620068	URBANO	310620005098	Serra	31062000562
2104	310620005620069	URBANO	310620005099	Mangabeiras	31062000562
2105	310620005620070	URBANO	310620005100	Comiteco	31062000562
2106	310620005620071	URBANO	310620005119	Cruzeiro	31062000562
2107	310620005620072	URBANO	310620005098	Serra	31062000562
977	310620005620090	URBANO	310620005088	Boa Viagem	31062000562
988	310620005620418	URBANO	310620005110	Vila Paris	31062000562
989	310620005620419	URBANO	310620005090	Santo Agostinho	31062000562
990	310620005620420	URBANO	310620005090	Santo Agostinho	31062000562
991	310620005620421	URBANO	310620005101	Belvedere	31062000562
992	310620005620422	URBANO	310620005101	Belvedere	31062000562
993	310620005630005	URBANO	310620005021	Esplanada	31062000563
994	310620005630006	URBANO	310620005021	Esplanada	31062000563
995	310620005630007	URBANO	310620005021	Esplanada	31062000563
996	310620005630008	URBANO	310620005021	Esplanada	31062000563
997	310620005630009	URBANO	310620005021	Esplanada	31062000563
998	310620005630010	URBANO	310620005023	PompÚia	31062000563
999	310620005630011	URBANO	310620005023	PompÚia	31062000563
1000	310620005630012	URBANO	310620005023	PompÚia	31062000563
1001	310620005630013	URBANO	310620005023	PompÚia	31062000563
1002	310620005630116	URBANO	310620005004	Sagrada FamÝlia	31062000563
1003	310620005630117	URBANO	310620005001	Santa InÛs	31062000563
1004	310620005630118	URBANO	310620005006	Boa Vista	31062000563
1005	310620005630119	URBANO	310620005006	Boa Vista	31062000563
1006	310620005630120	URBANO	310620005006	Boa Vista	31062000563
1007	310620005630121	URBANO	310620005006	Boa Vista	31062000563
1008	310620005630122	URBANO	310620005016	SÒo Geraldo	31062000563
1009	310620005630123	URBANO	310620005016	SÒo Geraldo	31062000563
1010	310620005630124	URBANO	310620005020	Mariano de Abreu	31062000563
1011	310620005640014	URBANO	310620005069	Ipiranga	31062000564
1012	310620005640015	URBANO	310620005069	Ipiranga	31062000564
1013	310620005640016	URBANO	310620005077	Cidade Nova	31062000564
1014	310620005640017	URBANO	310620005077	Cidade Nova	31062000564

1015	310620005640018	URBANO	310620005077	Cidade Nova	31062000564
1016	310620005640019	URBANO	310620005077	Cidade Nova	31062000564
1017	310620005640020	URBANO	310620005063	Silveira	31062000564
1018	310620005640021	URBANO	310620005063	Silveira	31062000564
1019	310620005650006	URBANO	310620005232	Padre Eustßquio	31062000565
1020	310620005650007	URBANO	310620005232	Padre Eustßquio	31062000565
1021	310620005650008	URBANO	310620005226	Minas Brasil	31062000565
1022	310620005650009	URBANO	310620005232	Padre Eustßquio	31062000565
1023	310620005650010	URBANO	310620005219	Jardim MontanhÛs	31062000565
1024	310620005650011	URBANO	310620005219	Jardim MontanhÛs	31062000565
1025	310620005650012	URBANO	310620005219	Jardim MontanhÛs	31062000565
1026	310620005650013	URBANO	310620005219	Jardim MontanhÛs	31062000565
1027	310620005650014	URBANO	310620005199	Caiþara - Adelaide	31062000565
1028	310620005650015	URBANO	310620005199	Caiþara - Adelaide	31062000565
1029	310620005650016	URBANO	310620005199	Caiþara - Adelaide	31062000565
1030	310620005650017	URBANO	310620005219	Jardim MontanhÛs	31062000565
1031	310620005650018	URBANO	310620005199	Caiþara - Adelaide	31062000565
1032	310620005650019	URBANO	310620005199	Caiþara - Adelaide	31062000565
1033	310620005650020	URBANO	310620005227	Monsenhor Messias	31062000565
1034	310620005650021	URBANO	310620005227	Monsenhor Messias	31062000565
2036	310620005620001	URBANO	310620005086	Funcionßrios	31062000562
2037	310620005620002	URBANO	310620005086	Funcionßrios	31062000562
2038	310620005620003	URBANO	310620005086	Funcionßrios	31062000562
2039	310620005620004	URBANO	310620005098	Serra	31062000562
2040	310620005620005	URBANO	310620005098	Serra	31062000562
2041	310620005620006	URBANO	310620005098	Serra	31062000562
2042	310620005620007	URBANO	310620005098	Serra	31062000562
2043	310620005620008	URBANO	310620005098	Serra	31062000562
2044	310620005620009	URBANO	310620005098	Serra	31062000562
2045	310620005620010	URBANO	310620005119	Cruzeiro	31062000562
2046	310620005620011	URBANO	310620005119	Cruzeiro	31062000562
2047	310620005620012	URBANO	310620005087	Savassi	31062000562
2048	310620005620013	URBANO	310620005087	Savassi	31062000562
2049	310620005620014	URBANO	310620005087	Savassi	31062000562
2050	310620005620015	URBANO	310620005087	Savassi	31062000562
2051	310620005620016	URBANO	310620005086	Funcionßrios	31062000562
2052	310620005620017	URBANO	310620005086	Funcionßrios	31062000562
2053	310620005620018	URBANO	310620005086	Funcionßrios	31062000562
2054	310620005620019	URBANO	310620005086	Funcionßrios	31062000562
2055	310620005620020	URBANO	310620005092	SÒo Lucas	31062000562
2056	310620005620021	URBANO	310620005092	SÒo Lucas	31062000562
2057	310620005620022	URBANO	310620005092	SÒo Lucas	31062000562
2058	310620005620023	URBANO	310620005098	Serra	31062000562
2059	310620005620024	URBANO	310620005098	Serra	31062000562
2060	310620005620025	URBANO	310620005098	Serra	31062000562
2061	310620005620026	URBANO	310620005098	Serra	31062000562
2062	310620005620027	URBANO	310620005098	Serra	31062000562
2063	310620005620028	URBANO	310620005098	Serra	31062000562
2064	310620005620029	URBANO	310620005098	Serra	31062000562
2065	310620005620030	URBANO	310620005098	Serra	31062000562

2066	310620005620031	URBANO	310620005098	Serra	31062000562
2067	310620005620032	URBANO	310620005098	Serra	31062000562
2068	310620005620033	URBANO	310620005098	Serra	31062000562
2069	310620005620034	URBANO	310620005119	Cruzeiro	31062000562
2070	310620005620035	URBANO	310620005119	Cruzeiro	31062000562
2071	310620005620036	URBANO	310620005119	Cruzeiro	31062000562
2072	310620005620037	URBANO	310620005119	Cruzeiro	31062000562
2073	310620005620038	URBANO	310620005119	Cruzeiro	31062000562
2074	310620005620039	URBANO	310620005119	Cruzeiro	31062000562
2075	310620005620040	URBANO	310620005118	Carmo	31062000562
2076	310620005620041	URBANO	310620005118	Carmo	31062000562
2077	310620005620042	URBANO	310620005087	Savassi	31062000562
2078	310620005620043	URBANO	310620005087	Savassi	31062000562
2079	310620005620044	URBANO	310620005087	Savassi	31062000562
2080	310620005620045	URBANO	310620005087	Savassi	31062000562
2081	310620005620046	URBANO	310620005087	Savassi	31062000562
2082	310620005620047	URBANO	310620005087	Savassi	31062000562
2083	310620005620048	URBANO	310620005087	Savassi	31062000562
2084	310620005620049	URBANO	310620005086	Funcionßrios	31062000562
2085	310620005620050	URBANO	310620005086	Funcionßrios	31062000562
2086	310620005620051	URBANO	310620005086	Funcionßrios	31062000562
2087	310620005620052	URBANO	310620005043	Santa EfigÛnia	31062000562
2088	310620005620053	URBANO	310620005043	Santa EfigÛnia	31062000562
2089	310620005620054	URBANO	310620005043	Santa EfigÛnia	31062000562
2090	310620005620055	URBANO	310620005043	Santa EfigÛnia	31062000562
2091	310620005620056	URBANO	310620005043	Santa EfigÛnia	31062000562
2092	310620005620057	URBANO	310620005043	Santa EfigÛnia	31062000562
2093	310620005620058	URBANO	310620005043	Santa EfigÛnia	31062000562
2094	310620005620059	URBANO	310620005043	Santa EfigÛnia	31062000562
2095	310620005620060	URBANO	310620005098	Serra	31062000562
2096	310620005620061	URBANO	310620005098	Serra	31062000562
2108	310620005620073	URBANO	310620005119	Cruzeiro	31062000562
2109	310620005620074	URBANO	310620005119	Cruzeiro	31062000562
2110	310620005620075	URBANO	310620005120	Anchieta	31062000562
2111	310620005620076	URBANO	310620005120	Anchieta	31062000562
2112	310620005620077	URBANO	310620005119	Cruzeiro	31062000562
2113	310620005620078	URBANO	310620005118	Carmo	31062000562
2114	310620005620079	URBANO	310620005118	Carmo	31062000562
2115	310620005620080	URBANO	310620005117	SÒo Pedro	31062000562
2116	310620005620081	URBANO	310620005087	Savassi	31062000562
2117	310620005620082	URBANO	310620005087	Savassi	31062000562
2118	310620005620083	URBANO	310620005087	Savassi	31062000562
2119	310620005620084	URBANO	310620005087	Savassi	31062000562
2120	310620005620085	URBANO	310620005087	Savassi	31062000562
2121	310620005620086	URBANO	310620005087	Savassi	31062000562
2122	310620005620087	URBANO	310620005088	Boa Viagem	31062000562
2123	310620005620088	URBANO	310620005088	Boa Viagem	31062000562
2124	310620005620089	URBANO	310620005088	Boa Viagem	31062000562
2125	310620005620098	URBANO	310620005093	Nossa Senhora da A	31062000562
2126	310620005620099	URBANO	310620005093	Nossa Senhora da A	31062000562

2127	310620005620100	URBANO	310620005093	Nossa Senhora da A	31062000562
2128	310620005620101	URBANO	310620005093	Nossa Senhora da A	31062000562
2129	310620005620102	URBANO	310620005093	Nossa Senhora da A	31062000562
2130	310620005620103	URBANO	310620005094	Nossa Senhora da C	31062000562
2131	310620005620104	URBANO	310620005094	Nossa Senhora da C	31062000562
2132	310620005620105	URBANO	310620005096	Nossa Senhora de F	31062000562
2133	310620005620106	URBANO	310620005090	Santo Agostinho	31062000562
2134	310620005620107	URBANO	310620005095	Santana do Cafezal	31062000562
2135	310620005620108	URBANO	310620005095	Santana do Cafezal	31062000562
2136	310620005620109	URBANO	310620005095	Santana do Cafezal	31062000562
2137	310620005620110	URBANO	310620005096	Nossa Senhora de F	31062000562
2138	310620005620111	URBANO	310620005096	Nossa Senhora de F	31062000562
2139	310620005620112	URBANO	310620005096	Nossa Senhora de F	31062000562
2140	310620005620113	URBANO	310620005096	Nossa Senhora de F	31062000562
2141	310620005620114	URBANO	310620005096	Nossa Senhora de F	31062000562
2142	310620005620115	URBANO	310620005095	Santana do Cafezal	31062000562
2143	310620005620116	URBANO	310620005095	Santana do Cafezal	31062000562
2144	310620005620117	URBANO	310620005095	Santana do Cafezal	31062000562
2145	310620005620118	URBANO	310620005096	Nossa Senhora de F	31062000562
2146	310620005620119	URBANO	310620005096	Nossa Senhora de F	31062000562
2147	310620005620120	URBANO	310620005096	Nossa Senhora de F	31062000562
2148	310620005620121	URBANO	310620005096	Nossa Senhora de F	31062000562
2149	310620005620122	URBANO	310620005097	Marþola	31062000562
2150	310620005620123	URBANO	310620005096	Nossa Senhora de F	31062000562
2151	310620005620124	URBANO	310620005097	Marþola	31062000562
2152	310620005620125	URBANO	310620005097	Marþola	31062000562
2153	310620005620126	URBANO	310620005097	Marþola	31062000562
2154	310620005620127	URBANO	310620005097	Marþola	31062000562
2155	310620005620128	URBANO	310620005097	Marþola	31062000562
2156	310620005620129	URBANO	310620005097	Marþola	31062000562
2157	310620005620130	URBANO	310620005097	Marþola	31062000562
2158	310620005620131	URBANO	310620005097	Marþola	31062000562
2159	310620005620132	URBANO	310620005096	Nossa Senhora de F	31062000562
2160	310620005620133	URBANO	310620005097	Marþola	31062000562
2161	310620005620134	URBANO	310620005096	Nossa Senhora de F	31062000562
2162	310620005620135	URBANO	310620005096	Nossa Senhora de F	31062000562
2163	310620005620136	URBANO	310620005094	Nossa Senhora da C	31062000562
2164	310620005620137	URBANO	310620005094	Nossa Senhora da C	31062000562
2165	310620005620138	URBANO	310620005094	Nossa Senhora da C	31062000562
2166	310620005620139	URBANO	310620005094	Nossa Senhora da C	31062000562
2167	310620005620140	URBANO	310620005094	Nossa Senhora da C	31062000562
2168	310620005620141	URBANO	310620005094	Nossa Senhora da C	31062000562
2169	310620005620142	URBANO	310620005094	Nossa Senhora da C	31062000562
2170	310620005620143	URBANO	310620005103	Acaba Mundo	31062000562
2171	310620005620144	URBANO	310620005100	Comiteco	31062000562
2172	310620005620145	URBANO	310620005100	Comiteco	31062000562
2173	310620005620146	URBANO	310620005120	Anchieta	31062000562
2174	310620005620147	URBANO	310620005120	Anchieta	31062000562
2175	310620005620148	URBANO	310620005120	Anchieta	31062000562
2176	310620005620149	URBANO	310620005120	Anchieta	31062000562

2177	310620005620150	URBANO	310620005120	Anchieta	31062000562
2178	310620005620151	URBANO	310620005120	Anchieta	31062000562
2179	310620005620152	URBANO	310620005120	Anchieta	31062000562
2180	310620005620153	URBANO	310620005120	Anchieta	31062000562
2181	310620005620154	URBANO	310620005120	Anchieta	31062000562
2182	310620005620155	URBANO	310620005120	Anchieta	31062000562
2183	310620005620156	URBANO	310620005120	Anchieta	31062000562
2184	310620005620157	URBANO	310620005120	Anchieta	31062000562
2185	310620005620158	URBANO	310620005118	Carmo	31062000562
2186	310620005620159	URBANO	310620005118	Carmo	31062000562
2187	310620005620160	URBANO	310620005117	SÒo Pedro	31062000562
2188	310620005620161	URBANO	310620005117	SÒo Pedro	31062000562
2189	310620005620162	URBANO	310620005113	Santo Ant¶nio	31062000562
2190	310620005620163	URBANO	310620005113	Santo Ant¶nio	31062000562
2191	310620005620164	URBANO	310620005113	Santo Ant¶nio	31062000562
2192	310620005620165	URBANO	310620005087	Savassi	31062000562
2193	310620005620166	URBANO	310620005087	Savassi	31062000562
2194	310620005620167	URBANO	310620005087	Savassi	31062000562
2195	310620005620168	URBANO	310620005089	Lourdes	31062000562
2196	310620005620169	URBANO	310620005089	Lourdes	31062000562
2197	310620005620170	URBANO	310620005089	Lourdes	31062000562
2198	310620005620171	URBANO	310620005089	Lourdes	31062000562
2199	310620005620172	URBANO	310620005089	Lourdes	31062000562
2200	310620005620173	URBANO	310620005083	Centro	31062000562
2201	310620005620174	URBANO	310620005083	Centro	31062000562
2202	310620005620175	URBANO	310620005083	Centro	31062000562
2203	310620005620176	URBANO	310620005083	Centro	31062000562
2204	310620005620177	URBANO	310620005083	Centro	31062000562
2205	310620005620178	URBANO	310620005083	Centro	31062000562
2206	310620005620179	URBANO	310620005083	Centro	31062000562
2207	310620005620180	URBANO	310620005083	Centro	31062000562
2208	310620005620181	URBANO	310620005083	Centro	31062000562
2209	310620005620182	URBANO	310620005083	Centro	31062000562
2210	310620005620183	URBANO	310620005083	Centro	31062000562
2211	310620005620184	URBANO	310620005083	Centro	31062000562
2212	310620005620185	URBANO	310620005083	Centro	31062000562
2213	310620005620186	URBANO	310620005083	Centro	31062000562
2214	310620005620187	URBANO	310620005010	Floresta	31062000562
2215	310620005620188	URBANO	310620005010	Floresta	31062000562
2216	310620005620189	URBANO	310620005010	Floresta	31062000562
2217	310620005620190	URBANO	310620005010	Floresta	31062000562
2218	310620005620191	URBANO	310620005121	Sion	31062000562
2219	310620005620192	URBANO	310620005120	Anchieta	31062000562
2220	310620005620193	URBANO	310620005120	Anchieta	31062000562
2221	310620005620194	URBANO	310620005120	Anchieta	31062000562
2222	310620005620195	URBANO	310620005121	Sion	31062000562
2223	310620005620196	URBANO	310620005121	Sion	31062000562
2224	310620005620197	URBANO	310620005121	Sion	31062000562
2225	310620005620198	URBANO	310620005117	SÒo Pedro	31062000562
2226	310620005620199	URBANO	310620005117	SÒo Pedro	31062000562

2227	310620005620200	URBANO	310620005113	Santo Ant¶nio	31062000562
2228	310620005620201	URBANO	310620005113	Santo Ant¶nio	31062000562
2229	310620005620202	URBANO	310620005113	Santo Ant¶nio	31062000562
2230	310620005620203	URBANO	310620005089	Lourdes	31062000562
2231	310620005620204	URBANO	310620005087	Savassi	31062000562
2232	310620005620205	URBANO	310620005089	Lourdes	31062000562
2233	310620005620206	URBANO	310620005089	Lourdes	31062000562
2234	310620005620207	URBANO	310620005089	Lourdes	31062000562
2235	310620005620208	URBANO	310620005083	Centro	31062000562
2236	310620005620209	URBANO	310620005089	Lourdes	31062000562
2237	310620005620210	URBANO	310620005089	Lourdes	31062000562
2238	310620005620211	URBANO	310620005083	Centro	31062000562
2239	310620005620212	URBANO	310620005083	Centro	31062000562
2240	310620005620213	URBANO	310620005083	Centro	31062000562
2241	310620005620214	URBANO	310620005083	Centro	31062000562
2242	310620005620215	URBANO	310620005083	Centro	31062000562
2243	310620005620216	URBANO	310620005083	Centro	31062000562
2244	310620005620217	URBANO	310620005083	Centro	31062000562
2245	310620005620218	URBANO	310620005083	Centro	31062000562
2246	310620005620219	URBANO	310620005083	Centro	31062000562
2247	310620005620220	URBANO	310620005083	Centro	31062000562
2248	310620005620221	URBANO	310620005121	Sion	31062000562
2249	310620005620222	URBANO	310620005121	Sion	31062000562
2250	310620005620223	URBANO	310620005121	Sion	31062000562
2251	310620005620224	URBANO	310620005121	Sion	31062000562
2252	310620005620225	URBANO	310620005121	Sion	31062000562
2253	310620005620226	URBANO	310620005121	Sion	31062000562
2254	310620005620227	URBANO	310620005121	Sion	31062000562
2255	310620005620228	URBANO	310620005121	Sion	31062000562
2256	310620005620229	URBANO	310620005121	Sion	31062000562
2257	310620005620230	URBANO	310620005117	SÒo Pedro	31062000562
2258	310620005620231	URBANO	310620005117	SÒo Pedro	31062000562
2259	310620005620232	URBANO	310620005117	SÒo Pedro	31062000562
2260	310620005620233	URBANO	310620005113	Santo Ant¶nio	31062000562
2261	310620005620234	URBANO	310620005113	Santo Ant¶nio	31062000562
2262	310620005620235	URBANO	310620005113	Santo Ant¶nio	31062000562
2263	310620005620236	URBANO	310620005113	Santo Ant¶nio	31062000562
2264	310620005620237	URBANO	310620005113	Santo Ant¶nio	31062000562
2265	310620005620238	URBANO	310620005089	Lourdes	31062000562
2266	310620005620239	URBANO	310620005089	Lourdes	31062000562
2267	310620005620240	URBANO	310620005089	Lourdes	31062000562
2268	310620005620241	URBANO	310620005089	Lourdes	31062000562
2269	310620005620242	URBANO	310620005089	Lourdes	31062000562
2270	310620005620243	URBANO	310620005089	Lourdes	31062000562
2271	310620005620244	URBANO	310620005089	Lourdes	31062000562
2272	310620005620245	URBANO	310620005089	Lourdes	31062000562
2273	310620005620246	URBANO	310620005090	Santo Agostinho	31062000562
2274	310620005620247	URBANO	310620005083	Centro	31062000562
2275	310620005620248	URBANO	310620005083	Centro	31062000562
2276	310620005620249	URBANO	310620005083	Centro	31062000562

2277	310620005620250	URBANO	310620005083	Centro	31062000562
2278	310620005620251	URBANO	310620005083	Centro	31062000562
2279	310620005620252	URBANO	310620005083	Centro	31062000562
2280	310620005620253	URBANO	310620005101	Belvedere	31062000562
2281	310620005620254	URBANO	310620005121	Sion	31062000562
2282	310620005620255	URBANO	310620005121	Sion	31062000562
2283	310620005620256	URBANO	310620005121	Sion	31062000562
2284	310620005620257	URBANO	310620005121	Sion	31062000562
2285	310620005620258	URBANO	310620005121	Sion	31062000562
2286	310620005620259	URBANO	310620005121	Sion	31062000562
2287	310620005620260	URBANO	310620005121	Sion	31062000562
2288	310620005620261	URBANO	310620005121	Sion	31062000562
2289	310620005620262	URBANO	310620005121	Sion	31062000562
2290	310620005620263	URBANO	310620005121	Sion	31062000562
2291	310620005620264	URBANO	310620005113	Santo Ant¶nio	31062000562
2292	310620005620265	URBANO	310620005113	Santo Ant¶nio	31062000562
2293	310620005620266	URBANO	310620005113	Santo Ant¶nio	31062000562
2294	310620005620267	URBANO	310620005113	Santo Ant¶nio	31062000562
2295	310620005620268	URBANO	310620005113	Santo Ant¶nio	31062000562
2296	310620005620269	URBANO	310620005113	Santo Ant¶nio	31062000562
2297	310620005620270	URBANO	310620005113	Santo Ant¶nio	31062000562
2298	310620005620271	URBANO	310620005089	Lourdes	31062000562
2299	310620005620272	URBANO	310620005089	Lourdes	31062000562
2300	310620005620273	URBANO	310620005089	Lourdes	31062000562
2301	310620005620274	URBANO	310620005089	Lourdes	31062000562
2302	310620005620275	URBANO	310620005089	Lourdes	31062000562
2303	310620005620276	URBANO	310620005090	Santo Agostinho	31062000562
2304	310620005620277	URBANO	310620005090	Santo Agostinho	31062000562
2305	310620005620278	URBANO	310620005090	Santo Agostinho	31062000562
2306	310620005620279	URBANO	310620005090	Santo Agostinho	31062000562
2307	310620005620280	URBANO	310620005090	Santo Agostinho	31062000562
2308	310620005620281	URBANO	310620005090	Santo Agostinho	31062000562
2309	310620005620282	URBANO	310620005091	Barro Preto	31062000562
2310	310620005620283	URBANO	310620005091	Barro Preto	31062000562
2311	310620005620284	URBANO	310620005091	Barro Preto	31062000562
2312	310620005620285	URBANO	310620005091	Barro Preto	31062000562
2313	310620005620286	URBANO	310620005091	Barro Preto	31062000562
2314	310620005620287	URBANO	310620005121	Sion	31062000562
2315	310620005620288	URBANO	310620005115	Santa Rita de Cßssia	31062000562
2316	310620005620289	URBANO	310620005115	Santa Rita de Cßssia	31062000562
2317	310620005620290	URBANO	310620005114	Vila Barragem Santa	31062000562
2318	310620005620291	URBANO	310620005114	Vila Barragem Santa	31062000562
2319	310620005620292	URBANO	310620005114	Vila Barragem Santa	31062000562
2320	310620005620293	URBANO	310620005114	Vila Barragem Santa	31062000562
2321	310620005620294	URBANO	310620005114	Vila Barragem Santa	31062000562
2322	310620005620295	URBANO	310620005114	Vila Barragem Santa	31062000562
2323	310620005620296	URBANO	310620005115	Santa Rita de Cßssia	31062000562
2324	310620005620297	URBANO	310620005115	Santa Rita de Cßssia	31062000562
2325	310620005620298	URBANO	310620005115	Santa Rita de Cßssia	31062000562
2326	310620005620299	URBANO	310620005115	Santa Rita de Cßssia	31062000562

2327	310620005620300	URBANO	310620005115	Santa Rita de Cßssia	31062000562
2328	310620005620301	URBANO	310620005114	Vila Barragem Santa	31062000562
2329	310620005620302	URBANO	310620005114	Vila Barragem Santa	31062000562
2330	310620005620303	URBANO	310620005114	Vila Barragem Santa	31062000562
2331	310620005620304	URBANO	310620005114	Vila Barragem Santa	31062000562
2332	310620005620305	URBANO	310620005114	Vila Barragem Santa	31062000562
2333	310620005620306	URBANO	310620005116	Estrela	31062000562
2334	310620005620307	URBANO	310620005113	Santo Ant¶nio	31062000562
2335	310620005620308	URBANO	310620005113	Santo Ant¶nio	31062000562
2336	310620005620309	URBANO	310620005113	Santo Ant¶nio	31062000562
2337	310620005620310	URBANO	310620005113	Santo Ant¶nio	31062000562
2338	310620005620311	URBANO	310620005113	Santo Ant¶nio	31062000562
2339	310620005620312	URBANO	310620005113	Santo Ant¶nio	31062000562
2340	310620005620313	URBANO	310620005113	Santo Ant¶nio	31062000562
2341	310620005620314	URBANO	310620005113	Santo Ant¶nio	31062000562
2342	310620005620315	URBANO	310620005113	Santo Ant¶nio	31062000562
2343	310620005620316	URBANO	310620005113	Santo Ant¶nio	31062000562
2344	310620005620317	URBANO	310620005113	Santo Ant¶nio	31062000562
2345	310620005620318	URBANO	310620005112	Cidade Jardim	31062000562
2346	310620005620319	URBANO	310620005112	Cidade Jardim	31062000562
2347	310620005620320	URBANO	310620005090	Santo Agostinho	31062000562
2348	310620005620321	URBANO	310620005090	Santo Agostinho	31062000562
2349	310620005620322	URBANO	310620005090	Santo Agostinho	31062000562
2350	310620005620323	URBANO	310620005090	Santo Agostinho	31062000562
2351	310620005620324	URBANO	310620005091	Barro Preto	31062000562
2352	310620005620325	URBANO	310620005091	Barro Preto	31062000562
2353	310620005620326	URBANO	310620005091	Barro Preto	31062000562
2354	310620005620327	URBANO	310620005091	Barro Preto	31062000562
2355	310620005620328	URBANO	310620005091	Barro Preto	31062000562
2356	310620005620329	URBANO	310620005091	Barro Preto	31062000562
2357	310620005620330	URBANO	310620005083	Centro	31062000562
2358	310620005620331	URBANO	310620005083	Centro	31062000562
2359	310620005620332	URBANO	310620005102	Santa L·cia	31062000562
2360	310620005620333	URBANO	310620005102	Santa L·cia	31062000562
2361	310620005620334	URBANO	310620005102	Santa L·cia	31062000562
2362	310620005620335	URBANO	310620005102	Santa L·cia	31062000562
2363	310620005620336	URBANO	310620005104	SÒo Bento	31062000562
2364	310620005620337	URBANO	310620005102	Santa L·cia	31062000562
2365	310620005620338	URBANO	310620005104	SÒo Bento	31062000562
2366	310620005620339	URBANO	310620005104	SÒo Bento	31062000562
2367	310620005620340	URBANO	310620005110	Vila Paris	31062000562
2368	310620005620341	URBANO	310620005110	Vila Paris	31062000562
2369	310620005620342	URBANO	310620005110	Vila Paris	31062000562
2370	310620005620343	URBANO	310620005109	CoraþÒo de Jesus	31062000562
2371	310620005620344	URBANO	310620005109	CoraþÒo de Jesus	31062000562
2372	310620005620345	URBANO	310620005109	CoraþÒo de Jesus	31062000562
2373	310620005620346	URBANO	310620005109	CoraþÒo de Jesus	31062000562
2374	310620005620347	URBANO	310620005109	CoraþÒo de Jesus	31062000562
2375	310620005620348	URBANO	310620005109	CoraþÒo de Jesus	31062000562
2376	310620005620349	URBANO	310620005112	Cidade Jardim	31062000562

2377	310620005620350	URBANO	310620005090	Santo Agostinho	31062000562
2378	310620005620351	URBANO	310620005090	Santo Agostinho	31062000562
2379	310620005620352	URBANO	310620005090	Santo Agostinho	31062000562
2380	310620005620353	URBANO	310620005091	Barro Preto	31062000562
2381	310620005620354	URBANO	310620005091	Barro Preto	31062000562
2382	310620005620355	URBANO	310620005091	Barro Preto	31062000562
2383	310620005620356	URBANO	310620005091	Barro Preto	31062000562
2384	310620005620357	URBANO	310620005101	Belvedere	31062000562
2385	310620005620358	URBANO	310620005101	Belvedere	31062000562
2386	310620005620359	URBANO	310620005101	Belvedere	31062000562
2387	310620005620360	URBANO	310620005101	Belvedere	31062000562
2388	310620005620361	URBANO	310620005101	Belvedere	31062000562
2389	310620005620362	URBANO	310620005102	Santa L·cia	31062000562
2390	310620005620363	URBANO	310620005104	SÒo Bento	31062000562
2391	310620005620364	URBANO	310620005104	SÒo Bento	31062000562
2392	310620005620365	URBANO	310620005104	SÒo Bento	31062000562
2393	310620005620366	URBANO	310620005104	SÒo Bento	31062000562
2394	310620005620367	URBANO	310620005108	Luxemburgo	31062000562
2395	310620005620368	URBANO	310620005108	Luxemburgo	31062000562
2396	310620005620369	URBANO	310620005108	Luxemburgo	31062000562
2397	310620005620370	URBANO	310620005108	Luxemburgo	31062000562
2398	310620005620371	URBANO	310620005108	Luxemburgo	31062000562
2399	310620005620372	URBANO	310620005108	Luxemburgo	31062000562
2400	310620005620373	URBANO	310620005108	Luxemburgo	31062000562
2401	310620005620374	URBANO	310620005109	CoraþÒo de Jesus	31062000562
2402	310620005620375	URBANO	310620005109	CoraþÒo de Jesus	31062000562
2403	310620005620376	URBANO	310620005108	Luxemburgo	31062000562
2404	310620005620377	URBANO	310620005111	Monte SÒo JosÚ	31062000562
2405	310620005620378	URBANO	310620005086	Funcionßrios	31062000562
2406	310620005620379	URBANO	310620005087	Savassi	31062000562
2407	310620005620380	URBANO	310620005087	Savassi	31062000562
2408	310620005620381	URBANO	310620005086	Funcionßrios	31062000562
2409	310620005620382	URBANO	310620005086	Funcionßrios	31062000562
2410	310620005620383	URBANO	310620005098	Serra	31062000562
2411	310620005620384	URBANO	310620005098	Serra	31062000562
2412	310620005620385	URBANO	310620005118	Carmo	31062000562
2413	310620005620386	URBANO	310620005086	Funcionßrios	31062000562
2414	310620005620387	URBANO	310620005098	Serra	31062000562
2415	310620005620388	URBANO	310620005119	Cruzeiro	31062000562
2416	310620005620389	URBANO	310620005098	Serra	31062000562
2417	310620005620390	URBANO	310620005118	Carmo	31062000562
2418	310620005620391	URBANO	310620005088	Boa Viagem	31062000562
2419	310620005620392	URBANO	310620005010	Floresta	31062000562
2420	310620005620393	URBANO	310620005120	Anchieta	31062000562
2421	310620005620394	URBANO	310620005120	Anchieta	31062000562
2422	310620005620395	URBANO	310620005117	SÒo Pedro	31062000562
2423	310620005620396	URBANO	310620005089	Lourdes	31062000562
2424	310620005620397	URBANO	310620005083	Centro	31062000562
2425	310620005620398	URBANO	310620005121	Sion	31062000562
2426	310620005620399	URBANO	310620005089	Lourdes	31062000562

2427	310620005620400	URBANO	310620005089	Lourdes	31062000562
2428	310620005620401	URBANO	310620005089	Lourdes	31062000562
2429	310620005620402	URBANO	310620005121	Sion	31062000562
2430	310620005620403	URBANO	310620005089	Lourdes	31062000562
2431	310620005620404	URBANO	310620005089	Lourdes	31062000562
2432	310620005620405	URBANO	310620005089	Lourdes	31062000562
2433	310620005620406	URBANO	310620005089	Lourdes	31062000562
2434	310620005620407	URBANO	310620005121	Sion	31062000562
2435	310620005620408	URBANO	310620005113	Santo Ant¶nio	31062000562
2436	310620005620409	URBANO	310620005113	Santo Ant¶nio	31062000562
2437	310620005620410	URBANO	310620005089	Lourdes	31062000562
2438	310620005620411	URBANO	310620005090	Santo Agostinho	31062000562
2439	310620005620412	URBANO	310620005090	Santo Agostinho	31062000562
2440	310620005620413	URBANO	310620005113	Santo Ant¶nio	31062000562
2441	310620005620414	URBANO	310620005113	Santo Ant¶nio	31062000562
2442	310620005620423	URBANO	310620005101	Belvedere	31062000562
2443	310620005620424	URBANO	310620005101	Belvedere	31062000562
2444	310620005620425	URBANO	310620005086	Funcionßrios	31062000562
2445	310620005620426	URBANO	310620005121	Sion	31062000562
2446	310620005620427	URBANO	310620005121	Sion	31062000562
2447	310620005620428	URBANO	310620005120	Anchieta	31062000562
2448	310620005620429	URBANO	310620005121	Sion	31062000562
2449	310620005620430	URBANO	310620005120	Anchieta	31062000562
2450	310620005620431	URBANO	310620005110	Vila Paris	31062000562
2451	310620005620432	URBANO	310620005110	Vila Paris	31062000562
2452	310620005620433	URBANO	310620005109	CoraþÒo de Jesus	31062000562
2453	310620005620434	URBANO	310620005109	CoraþÒo de Jesus	31062000562
2454	310620005620435	URBANO	310620005102	Santa L·cia	31062000562
2455	310620005620436	URBANO	310620005086	Funcionßrios	31062000562
2456	310620005620437	URBANO	310620005088	Boa Viagem	31062000562
2457	310620005620438	URBANO	310620005089	Lourdes	31062000562
2458	310620005620439	URBANO	310620005089	Lourdes	31062000562
2459	310620005620440	URBANO	310620005087	Savassi	31062000562
2460	310620005620441	URBANO	310620005089	Lourdes	31062000562
2461	310620005620442	URBANO	310620005090	Santo Agostinho	31062000562
2462	310620005620443	URBANO	310620005087	Savassi	31062000562
2463	310620005620444	URBANO	310620005086	Funcionßrios	31062000562
2464	310620005620445	URBANO	310620005119	Cruzeiro	31062000562
2465	310620005620446	URBANO	310620005110	Vila Paris	31062000562
2466	310620005620447	URBANO	310620005108	Luxemburgo	31062000562
2467	310620005620448	URBANO	310620005109	CoraþÒo de Jesus	31062000562
2468	310620005620449	URBANO	310620005120	Anchieta	31062000562
2469	310620005620450	URBANO	310620005121	Sion	31062000562
2470	310620005620451	URBANO	310620005100	Comiteco	31062000562
2471	310620005620452	URBANO	310620005117	SÒo Pedro	31062000562
2472	310620005620453	URBANO	310620005102	Santa L·cia	31062000562
2473	310620005620454	URBANO	310620005102	Santa L·cia	31062000562
2474	310620005620455	URBANO	310620005101	Belvedere	31062000562
2475	310620005620456	URBANO	310620005099	Mangabeiras	31062000562
2476	310620005620457	URBANO	310620005121	Sion	31062000562

2477	310620005620458	URBANO	310620005096	Nossa Senhora de F	31062000562
2478	310620005620459	URBANO	310620005083	Centro	31062000562
2479	310620005620460	URBANO	310620005099	Mangabeiras	31062000562
2480	310620005620461	URBANO	310620005102	Santa L·cia	31062000562
2481	310620005620462	URBANO	310620005096	Nossa Senhora de F	31062000562
2482	310620005620463	URBANO	310620005113	Santo Ant¶nio	31062000562
2483	310620005620464	URBANO	310620005095	Santana do Cafezal	31062000562
2484	310620005620465	URBANO	310620005097	Marþola	31062000562
2485	310620005620466	URBANO	310620005092	SÒo Lucas	31062000562
2486	310620005620467	URBANO	310620005097	Marþola	31062000562
2487	310620005620468	URBANO	310620005116	Estrela	31062000562
2488	310620005620469	URBANO	310620005099	Mangabeiras	31062000562
2489	310620005620470	URBANO	310620005095	Santana do Cafezal	31062000562
2490	310620005620471	URBANO	310620005095	Santana do Cafezal	31062000562
2491	310620005620472	URBANO	310620005096	Nossa Senhora de F	31062000562
2492	310620005620473	URBANO	310620005099	Mangabeiras	31062000562
2493	310620005620474	URBANO	310620005101	Belvedere	31062000562
2494	310620005620475	URBANO	310620005089	Lourdes	31062000562
2495	310620005620476	URBANO	310620005107	Vila Bandeirantes	31062000562
2496	310620005620477	URBANO	310620005107	Vila Bandeirantes	31062000562
2497	310620005620478	URBANO	310620005105	┴pia	31062000562
2498	310620005620479	URBANO	310620005106	Conjunto Santa Mar	31062000562
2499	310620005620480	URBANO	310620005109	CoraþÒo de Jesus	31062000562
2500	310620005620481	URBANO	310620005088	Boa Viagem	31062000562
2501	310620005620482	URBANO	310620005086	Funcionßrios	31062000562
2502	310620005620483	URBANO	310620005087	Savassi	31062000562
2503	310620005620484	URBANO	310620005099	Mangabeiras	31062000562
2504	310620005620485	URBANO	310620005087	Savassi	31062000562
2505	310620005620486	URBANO	310620005091	Barro Preto	31062000562
2506	310620005620487	URBANO	310620005122	Santa Isabel	31062000562
2507	310620005620488	URBANO	310620005123	Vila Fumec	31062000562
2508	310620005620489	URBANO	310620005124	Pindura Saia	31062000562
2509	310620005620490	URBANO	310620005125	Mala e Cuia	31062000562
2510	310620005620491	URBANO	310620005104	SÒo Bento	31062000562
2511	310620005620492	URBANO	310620005117	SÒo Pedro	31062000562
2512	310620005620493	URBANO	310620005043	Santa EfigÛnia	31062000562
2513	310620005620494	URBANO	310620005113	Santo Ant¶nio	31062000562
2514	310620005620495	URBANO	310620005086	Funcionßrios	31062000562
2515	310620005620496	URBANO	310620005109	CoraþÒo de Jesus	31062000562
2516	310620005620497	URBANO	310620005090	Santo Agostinho	31062000562
2517	310620005620498	URBANO	310620005108	Luxemburgo	31062000562
2518	310620005630001	URBANO	310620005021	Esplanada	31062000563
2519	310620005630002	URBANO	310620005021	Esplanada	31062000563
2520	310620005630003	URBANO	310620005022	Vila Nossa Senhora	31062000563
2521	310620005630004	URBANO	310620005021	Esplanada	31062000563
2522	310620005630014	URBANO	310620005021	Esplanada	31062000563
2523	310620005630015	URBANO	310620005021	Esplanada	31062000563
2524	310620005630016	URBANO	310620005021	Esplanada	31062000563
2525	310620005630017	URBANO	310620005025	Vera Cruz	31062000563
2526	310620005630018	URBANO	310620005025	Vera Cruz	31062000563

2527	310620005630019	URBANO	310620005023	PompÚia	31062000563
2528	310620005630020	URBANO	310620005014	Horto	31062000563
2529	310620005630021	URBANO	310620005014	Horto	31062000563
2530	310620005630022	URBANO	310620005002	Horto Florestal	31062000563
2531	310620005630023	URBANO	310620005016	SÒo Geraldo	31062000563
2532	310620005630024	URBANO	310620005016	SÒo Geraldo	31062000563
2533	310620005630025	URBANO	310620005016	SÒo Geraldo	31062000563
2534	310620005630026	URBANO	310620005016	SÒo Geraldo	31062000563
2535	310620005630027	URBANO	310620005032	Alto Vera Cruz	31062000563
2536	310620005630028	URBANO	310620005025	Vera Cruz	31062000563
2537	310620005630029	URBANO	310620005025	Vera Cruz	31062000563
2538	310620005630030	URBANO	310620005029	Saudade	31062000563
2539	310620005630031	URBANO	310620005029	Saudade	31062000563
2540	310620005630032	URBANO	310620005023	PompÚia	31062000563
2541	310620005630033	URBANO	310620005023	PompÚia	31062000563
2542	310620005630034	URBANO	310620005044	ParaÝso	31062000563
2543	310620005630035	URBANO	310620005011	Santa Tereza	31062000563
2544	310620005630036	URBANO	310620005011	Santa Tereza	31062000563
2545	310620005630037	URBANO	310620005011	Santa Tereza	31062000563
2546	310620005630038	URBANO	310620005011	Santa Tereza	31062000563
2547	310620005630039	URBANO	310620005011	Santa Tereza	31062000563
2548	310620005630040	URBANO	310620005014	Horto	31062000563
2549	310620005630041	URBANO	310620005004	Sagrada FamÝlia	31062000563
2550	310620005630042	URBANO	310620005004	Sagrada FamÝlia	31062000563
2551	310620005630043	URBANO	310620005002	Horto Florestal	31062000563
2552	310620005630044	URBANO	310620005002	Horto Florestal	31062000563
2553	310620005630045	URBANO	310620005002	Horto Florestal	31062000563
2554	310620005630046	URBANO	310620005002	Horto Florestal	31062000563
2555	310620005630047	URBANO	310620005001	Santa InÛs	31062000563
2556	310620005630048	URBANO	310620005006	Boa Vista	31062000563
2557	310620005630049	URBANO	310620005006	Boa Vista	31062000563
2558	310620005630050	URBANO	310620005016	SÒo Geraldo	31062000563
2559	310620005630051	URBANO	310620005016	SÒo Geraldo	31062000563
2560	310620005630052	URBANO	310620005016	SÒo Geraldo	31062000563
2561	310620005630053	URBANO	310620005008	Casa Branca	31062000563
2562	310620005630054	URBANO	310620005032	Alto Vera Cruz	31062000563
2563	310620005630055	URBANO	310620005032	Alto Vera Cruz	31062000563
2564	310620005630056	URBANO	310620005032	Alto Vera Cruz	31062000563
2565	310620005630057	URBANO	310620005032	Alto Vera Cruz	31062000563
2566	310620005630058	URBANO	310620005025	Vera Cruz	31062000563
2567	310620005630059	URBANO	310620005025	Vera Cruz	31062000563
2568	310620005630060	URBANO	310620005029	Saudade	31062000563
2569	310620005630061	URBANO	310620005029	Saudade	31062000563
2570	310620005630062	URBANO	310620005044	ParaÝso	31062000563
2571	310620005630063	URBANO	310620005044	ParaÝso	31062000563
2572	310620005630064	URBANO	310620005043	Santa EfigÛnia	31062000563
2573	310620005630065	URBANO	310620005011	Santa Tereza	31062000563
2574	310620005630066	URBANO	310620005014	Horto	31062000563
2575	310620005630067	URBANO	310620005011	Santa Tereza	31062000563
2576	310620005630068	URBANO	310620005011	Santa Tereza	31062000563

2577	310620005630069	URBANO	310620005014	Horto	31062000563
2578	310620005630070	URBANO	310620005004	Sagrada FamÝlia	31062000563
2579	310620005630071	URBANO	310620005004	Sagrada FamÝlia	31062000563
2580	310620005630072	URBANO	310620005004	Sagrada FamÝlia	31062000563
2581	310620005630073	URBANO	310620005004	Sagrada FamÝlia	31062000563
2582	310620005630074	URBANO	310620005004	Sagrada FamÝlia	31062000563
2583	310620005630075	URBANO	310620005004	Sagrada FamÝlia	31062000563
2584	310620005630076	URBANO	310620005004	Sagrada FamÝlia	31062000563
2585	310620005630077	URBANO	310620005002	Horto Florestal	31062000563
2586	310620005630078	URBANO	310620005002	Horto Florestal	31062000563
2587	310620005630079	URBANO	310620005001	Santa InÛs	31062000563
2588	310620005630080	URBANO	310620005001	Santa InÛs	31062000563
2589	310620005630081	URBANO	310620005006	Boa Vista	31062000563
2590	310620005630082	URBANO	310620005006	Boa Vista	31062000563
2591	310620005630083	URBANO	310620005006	Boa Vista	31062000563
2592	310620005630084	URBANO	310620005006	Boa Vista	31062000563
2593	310620005630085	URBANO	310620005016	SÒo Geraldo	31062000563
2594	310620005630086	URBANO	310620005016	SÒo Geraldo	31062000563
2595	310620005630087	URBANO	310620005016	SÒo Geraldo	31062000563
2596	310620005630088	URBANO	310620005032	Alto Vera Cruz	31062000563
2597	310620005630089	URBANO	310620005032	Alto Vera Cruz	31062000563
2598	310620005630090	URBANO	310620005032	Alto Vera Cruz	31062000563
2599	310620005630091	URBANO	310620005032	Alto Vera Cruz	31062000563
2600	310620005630092	URBANO	310620005032	Alto Vera Cruz	31062000563
2601	310620005630093	URBANO	310620005032	Alto Vera Cruz	31062000563
2602	310620005630094	URBANO	310620005029	Saudade	31062000563
2603	310620005630095	URBANO	310620005029	Saudade	31062000563
2604	310620005630096	URBANO	310620005044	ParaÝso	31062000563
2605	310620005630097	URBANO	310620005044	ParaÝso	31062000563
2606	310620005630098	URBANO	310620005044	ParaÝso	31062000563
2607	310620005630099	URBANO	310620005046	C¶nego Pinheiro 1¬	31062000563
2608	310620005630100	URBANO	310620005044	ParaÝso	31062000563
2609	310620005630101	URBANO	310620005043	Santa EfigÛnia	31062000563
2610	310620005630102	URBANO	310620005011	Santa Tereza	31062000563
2611	310620005630103	URBANO	310620005011	Santa Tereza	31062000563
2612	310620005630104	URBANO	310620005011	Santa Tereza	31062000563
2613	310620005630105	URBANO	310620005011	Santa Tereza	31062000563
2614	310620005630106	URBANO	310620005011	Santa Tereza	31062000563
2615	310620005630107	URBANO	310620005011	Santa Tereza	31062000563
2616	310620005630108	URBANO	310620005004	Sagrada FamÝlia	31062000563
2617	310620005630109	URBANO	310620005004	Sagrada FamÝlia	31062000563
2618	310620005630110	URBANO	310620005004	Sagrada FamÝlia	31062000563
2619	310620005630111	URBANO	310620005004	Sagrada FamÝlia	31062000563
2620	310620005630112	URBANO	310620005004	Sagrada FamÝlia	31062000563
2621	310620005630113	URBANO	310620005004	Sagrada FamÝlia	31062000563
2622	310620005630114	URBANO	310620005004	Sagrada FamÝlia	31062000563
2623	310620005630115	URBANO	310620005004	Sagrada FamÝlia	31062000563
2624	310620005630125	URBANO	310620005008	Casa Branca	31062000563
2625	310620005630126	URBANO	310620005008	Casa Branca	31062000563
2626	310620005630127	URBANO	310620005032	Alto Vera Cruz	31062000563

2627	310620005630128	URBANO	310620005032	Alto Vera Cruz	31062000563
2628	310620005630129	URBANO	310620005032	Alto Vera Cruz	31062000563
2629	310620005630130	URBANO	310620005032	Alto Vera Cruz	31062000563
2630	310620005630131	URBANO	310620005032	Alto Vera Cruz	31062000563
2631	310620005630132	URBANO	310620005044	ParaÝso	31062000563
2632	310620005630133	URBANO	310620005044	ParaÝso	31062000563
2633	310620005630134	URBANO	310620005044	ParaÝso	31062000563
2634	310620005630135	URBANO	310620005044	ParaÝso	31062000563
2635	310620005630136	URBANO	310620005044	ParaÝso	31062000563
2636	310620005630137	URBANO	310620005043	Santa EfigÛnia	31062000563
2637	310620005630138	URBANO	310620005043	Santa EfigÛnia	31062000563
2638	310620005630139	URBANO	310620005043	Santa EfigÛnia	31062000563
2639	310620005630140	URBANO	310620005045	Vila UniÒo	31062000563
2640	310620005630141	URBANO	310620005011	Santa Tereza	31062000563
2641	310620005630142	URBANO	310620005011	Santa Tereza	31062000563
2642	310620005630143	URBANO	310620005004	Sagrada FamÝlia	31062000563
2643	310620005630144	URBANO	310620005004	Sagrada FamÝlia	31062000563
2644	310620005630145	URBANO	310620005004	Sagrada FamÝlia	31062000563
2645	310620005630146	URBANO	310620005004	Sagrada FamÝlia	31062000563
2646	310620005630147	URBANO	310620005004	Sagrada FamÝlia	31062000563
2647	310620005630148	URBANO	310620005004	Sagrada FamÝlia	31062000563
2648	310620005630149	URBANO	310620005004	Sagrada FamÝlia	31062000563
2649	310620005630150	URBANO	310620005001	Santa InÛs	31062000563
2650	310620005630151	URBANO	310620005001	Santa InÛs	31062000563
2651	310620005630152	URBANO	310620005005	Nova Vista	31062000563
2652	310620005630153	URBANO	310620005005	Nova Vista	31062000563
2653	310620005630154	URBANO	310620005005	Nova Vista	31062000563
2654	310620005630155	URBANO	310620005005	Nova Vista	31062000563
2655	310620005630156	URBANO	310620005006	Boa Vista	31062000563
2656	310620005630157	URBANO	310620005006	Boa Vista	31062000563
2657	310620005630158	URBANO	310620005006	Boa Vista	31062000563
2658	310620005630159	URBANO	310620005006	Boa Vista	31062000563
2659	310620005630160	URBANO	310620005006	Boa Vista	31062000563
2660	310620005630161	URBANO	310620005016	SÒo Geraldo	31062000563
2661	310620005630162	URBANO	310620005007	Vila Boa Vista	31062000563
2662	310620005630163	URBANO	310620005007	Vila Boa Vista	31062000563
2663	310620005630164	URBANO	310620005020	Mariano de Abreu	31062000563
2664	310620005630165	URBANO	310620005020	Mariano de Abreu	31062000563
2665	310620005630166	URBANO	310620005008	Casa Branca	31062000563
2666	310620005630167	URBANO	310620005032	Alto Vera Cruz	31062000563
2667	310620005630168	URBANO	310620005032	Alto Vera Cruz	31062000563
2668	310620005630169	URBANO	310620005032	Alto Vera Cruz	31062000563
2669	310620005630170	URBANO	310620005032	Alto Vera Cruz	31062000563
2670	310620005630171	URBANO	310620005032	Alto Vera Cruz	31062000563
2671	310620005630172	URBANO	310620005035	Taquaril	31062000563
2672	310620005630173	URBANO	310620005032	Alto Vera Cruz	31062000563
2673	310620005630174	URBANO	310620005039	Jonas Veiga	31062000563
2674	310620005630175	URBANO	310620005044	ParaÝso	31062000563
2675	310620005630176	URBANO	310620005044	ParaÝso	31062000563
2676	310620005630177	URBANO	310620005044	ParaÝso	31062000563

2677	310620005630178	URBANO	310620005044	ParaÝso	31062000563
2678	310620005630179	URBANO	310620005043	Santa EfigÛnia	31062000563
2679	310620005630180	URBANO	310620005043	Santa EfigÛnia	31062000563
2680	310620005630181	URBANO	310620005043	Santa EfigÛnia	31062000563
2681	310620005630182	URBANO	310620005043	Santa EfigÛnia	31062000563
2682	310620005630183	URBANO	310620005043	Santa EfigÛnia	31062000563
2683	310620005630184	URBANO	310620005043	Santa EfigÛnia	31062000563
2684	310620005630185	URBANO	310620005013	Buraco Quente	31062000563
2685	310620005630186	URBANO	310620005011	Santa Tereza	31062000563
2686	310620005630187	URBANO	310620005012	SÒo Vicente	31062000563
2687	310620005630188	URBANO	310620005011	Santa Tereza	31062000563
2688	310620005630189	URBANO	310620005011	Santa Tereza	31062000563
2689	310620005630190	URBANO	310620005010	Floresta	31062000563
2690	310620005630191	URBANO	310620005011	Santa Tereza	31062000563
2691	310620005630192	URBANO	310620005010	Floresta	31062000563
2692	310620005630193	URBANO	310620005004	Sagrada FamÝlia	31062000563
2693	310620005630194	URBANO	310620005004	Sagrada FamÝlia	31062000563
2694	310620005630195	URBANO	310620005004	Sagrada FamÝlia	31062000563
2695	310620005630196	URBANO	310620005004	Sagrada FamÝlia	31062000563
2696	310620005630197	URBANO	310620005004	Sagrada FamÝlia	31062000563
2697	310620005630198	URBANO	310620005004	Sagrada FamÝlia	31062000563
2698	310620005630199	URBANO	310620005004	Sagrada FamÝlia	31062000563
2699	310620005630200	URBANO	310620005004	Sagrada FamÝlia	31062000563
2700	310620005630201	URBANO	310620005001	Santa InÛs	31062000563
2701	310620005630202	URBANO	310620005001	Santa InÛs	31062000563
2702	310620005630203	URBANO	310620005005	Nova Vista	31062000563
2703	310620005630204	URBANO	310620005005	Nova Vista	31062000563
2704	310620005630205	URBANO	310620005005	Nova Vista	31062000563
2705	310620005630206	URBANO	310620005006	Boa Vista	31062000563
2706	310620005630207	URBANO	310620005040	Pirineus	31062000563
2707	310620005630208	URBANO	310620005042	Baleia	31062000563
2708	310620005630209	URBANO	310620005051	Fazendinha	31062000563
2709	310620005630210	URBANO	310620005051	Fazendinha	31062000563
2710	310620005630211	URBANO	310620005044	ParaÝso	31062000563
2711	310620005630212	URBANO	310620005044	ParaÝso	31062000563
2712	310620005630213	URBANO	310620005043	Santa EfigÛnia	31062000563
2713	310620005630214	URBANO	310620005010	Floresta	31062000563
2714	310620005630215	URBANO	310620005010	Floresta	31062000563
2715	310620005630216	URBANO	310620005010	Floresta	31062000563
2716	310620005630217	URBANO	310620005010	Floresta	31062000563
2717	310620005630218	URBANO	310620005010	Floresta	31062000563
2718	310620005630219	URBANO	310620005010	Floresta	31062000563
2719	310620005630220	URBANO	310620005010	Floresta	31062000563
2720	310620005630221	URBANO	310620005010	Floresta	31062000563
2721	310620005630222	URBANO	310620005004	Sagrada FamÝlia	31062000563
2722	310620005630223	URBANO	310620005004	Sagrada FamÝlia	31062000563
2723	310620005630224	URBANO	310620005004	Sagrada FamÝlia	31062000563
2724	310620005630225	URBANO	310620005004	Sagrada FamÝlia	31062000563
2725	310620005630226	URBANO	310620005004	Sagrada FamÝlia	31062000563
2726	310620005630227	URBANO	310620005001	Santa InÛs	31062000563

2727	310620005630228	URBANO	310620005005	Nova Vista	31062000563
2728	310620005630229	URBANO	310620005006	Boa Vista	31062000563
2729	310620005630230	URBANO	310620005006	Boa Vista	31062000563
2730	310620005630231	URBANO	310620005008	Casa Branca	31062000563
2731	310620005630232	URBANO	310620005050	Vila Novo SÒo Lucas	31062000563
2732	310620005630233	URBANO	310620005049	Novo SÒo Lucas	31062000563
2733	310620005630234	URBANO	310620005043	Santa EfigÛnia	31062000563
2734	310620005630235	URBANO	310620005049	Novo SÒo Lucas	31062000563
2735	310620005630236	URBANO	310620005049	Novo SÒo Lucas	31062000563
2736	310620005630237	URBANO	310620005049	Novo SÒo Lucas	31062000563
2737	310620005630238	URBANO	310620005043	Santa EfigÛnia	31062000563
2738	310620005630239	URBANO	310620005036	Granja de Freitas	31062000563
2739	310620005630240	URBANO	310620005043	Santa EfigÛnia	31062000563
2740	310620005630241	URBANO	310620005010	Floresta	31062000563
2741	310620005630242	URBANO	310620005009	ColÚgio Batista	31062000563
2742	310620005630243	URBANO	310620005009	ColÚgio Batista	31062000563
2743	310620005630244	URBANO	310620005009	ColÚgio Batista	31062000563
2744	310620005630245	URBANO	310620005009	ColÚgio Batista	31062000563
2745	310620005630246	URBANO	310620005009	ColÚgio Batista	31062000563
2746	310620005630247	URBANO	310620005009	ColÚgio Batista	31062000563
2747	310620005630248	URBANO	310620005009	ColÚgio Batista	31062000563
2748	310620005630249	URBANO	310620005009	ColÚgio Batista	31062000563
2749	310620005630250	URBANO	310620005009	ColÚgio Batista	31062000563
2750	310620005630251	URBANO	310620005009	ColÚgio Batista	31062000563
2751	310620005630252	URBANO	310620005044	ParaÝso	31062000563
2752	310620005630253	URBANO	310620005036	Granja de Freitas	31062000563
2753	310620005630254	URBANO	310620005035	Taquaril	31062000563
2754	310620005630255	URBANO	310620005035	Taquaril	31062000563
2755	310620005630256	URBANO	310620005041	Cidade Jardim Taqu	31062000563
2756	310620005630257	URBANO	310620005038	Conjunto Taquaril	31062000563
2757	310620005630258	URBANO	310620005038	Conjunto Taquaril	31062000563
2758	310620005630259	URBANO	310620005038	Conjunto Taquaril	31062000563
2759	310620005630260	URBANO	310620005038	Conjunto Taquaril	31062000563
2760	310620005630261	URBANO	310620005038	Conjunto Taquaril	31062000563
2761	310620005630262	URBANO	310620005038	Conjunto Taquaril	31062000563
2762	310620005630263	URBANO	310620005038	Conjunto Taquaril	31062000563
2763	310620005630264	URBANO	310620005038	Conjunto Taquaril	31062000563
2764	310620005630265	URBANO	310620005038	Conjunto Taquaril	31062000563
2765	310620005630266	URBANO	310620005038	Conjunto Taquaril	31062000563
2766	310620005630267	URBANO	310620005038	Conjunto Taquaril	31062000563
2767	310620005630268	URBANO	310620005038	Conjunto Taquaril	31062000563
2768	310620005630269	URBANO	310620005038	Conjunto Taquaril	31062000563
2769	310620005630270	URBANO	310620005038	Conjunto Taquaril	31062000563
2770	310620005630271	URBANO	310620005038	Conjunto Taquaril	31062000563
2771	310620005630272	URBANO	310620005043	Santa EfigÛnia	31062000563
2772	310620005630273	URBANO	310620005043	Santa EfigÛnia	31062000563
2773	310620005630274	URBANO	310620005043	Santa EfigÛnia	31062000563
2774	310620005630275	URBANO	310620005043	Santa EfigÛnia	31062000563
2775	310620005630276	URBANO	310620005009	ColÚgio Batista	31062000563
2776	310620005630277	URBANO	310620005009	ColÚgio Batista	31062000563

2777	310620005630278	URBANO	310620005010	Floresta	31062000563
2778	310620005630279	URBANO	310620005004	Sagrada FamÝlia	31062000563
2779	310620005630280	URBANO	310620005009	ColÚgio Batista	31062000563
2780	310620005630281	URBANO	310620005010	Floresta	31062000563
2781	310620005630282	URBANO	310620005010	Floresta	31062000563
2782	310620005630283	URBANO	310620005004	Sagrada FamÝlia	31062000563
2783	310620005630284	URBANO	310620005004	Sagrada FamÝlia	31062000563
2784	310620005630285	URBANO	310620005004	Sagrada FamÝlia	31062000563
2785	310620005630286	URBANO	310620005004	Sagrada FamÝlia	31062000563
2786	310620005630287	URBANO	310620005004	Sagrada FamÝlia	31062000563
2787	310620005630288	URBANO	310620005004	Sagrada FamÝlia	31062000563
2788	310620005630289	URBANO	310620005004	Sagrada FamÝlia	31062000563
2789	310620005630290	URBANO	310620005004	Sagrada FamÝlia	31062000563
2790	310620005630291	URBANO	310620005004	Sagrada FamÝlia	31062000563
2791	310620005630292	URBANO	310620005004	Sagrada FamÝlia	31062000563
2792	310620005630293	URBANO	310620005004	Sagrada FamÝlia	31062000563
2793	310620005630294	URBANO	310620005004	Sagrada FamÝlia	31062000563
2794	310620005630295	URBANO	310620005010	Floresta	31062000563
2795	310620005630296	URBANO	310620005011	Santa Tereza	31062000563
2796	310620005630297	URBANO	310620005014	Horto	31062000563
2797	310620005630298	URBANO	310620005014	Horto	31062000563
2798	310620005630299	URBANO	310620005014	Horto	31062000563
2799	310620005630300	URBANO	310620005004	Sagrada FamÝlia	31062000563
2800	310620005630301	URBANO	310620005014	Horto	31062000563
2801	310620005630302	URBANO	310620005004	Sagrada FamÝlia	31062000563
2802	310620005630303	URBANO	310620005004	Sagrada FamÝlia	31062000563
2803	310620005630304	URBANO	310620005004	Sagrada FamÝlia	31062000563
2804	310620005630305	URBANO	310620005002	Horto Florestal	31062000563
2805	310620005630306	URBANO	310620005004	Sagrada FamÝlia	31062000563
2806	310620005630307	URBANO	310620005004	Sagrada FamÝlia	31062000563
2807	310620005630308	URBANO	310620005014	Horto	31062000563
2808	310620005630309	URBANO	310620005002	Horto Florestal	31062000563
2809	310620005630310	URBANO	310620005011	Santa Tereza	31062000563
2810	310620005630311	URBANO	310620005014	Horto	31062000563
2811	310620005630312	URBANO	310620005011	Santa Tereza	31062000563
2812	310620005630313	URBANO	310620005011	Santa Tereza	31062000563
2813	310620005630314	URBANO	310620005015	JoÒo Alfredo	31062000563
2814	310620005630315	URBANO	310620005017	Camponesa 2¬ Seþ	31062000563
2815	310620005630316	URBANO	310620005014	Horto	31062000563
2816	310620005630317	URBANO	310620005016	SÒo Geraldo	31062000563
2817	310620005630318	URBANO	310620005006	Boa Vista	31062000563
2818	310620005630319	URBANO	310620005002	Horto Florestal	31062000563
2819	310620005630320	URBANO	310620005003	Camponesa 1¬ Seþ	31062000563
2820	310620005630321	URBANO	310620005016	SÒo Geraldo	31062000563
2821	310620005630322	URBANO	310620005019	Grota	31062000563
2822	310620005630323	URBANO	310620005016	SÒo Geraldo	31062000563
2823	310620005630324	URBANO	310620005016	SÒo Geraldo	31062000563
2824	310620005630325	URBANO	310620005016	SÒo Geraldo	31062000563
2825	310620005630326	URBANO	310620005023	PompÚia	31062000563
2826	310620005630327	URBANO	310620005023	PompÚia	31062000563

2827	310620005630328	URBANO	310620005024	Vila SÒo Rafael	31062000563
2828	310620005630329	URBANO	310620005024	Vila SÒo Rafael	31062000563
2829	310620005630330	URBANO	310620005023	PompÚia	31062000563
2830	310620005630331	URBANO	310620005023	PompÚia	31062000563
2831	310620005630332	URBANO	310620005029	Saudade	31062000563
2832	310620005630333	URBANO	310620005023	PompÚia	31062000563
2833	310620005630334	URBANO	310620005002	Horto Florestal	31062000563
2834	310620005630335	URBANO	310620005002	Horto Florestal	31062000563
2835	310620005630336	URBANO	310620005016	SÒo Geraldo	31062000563
2836	310620005630337	URBANO	310620005034	Caetano Furquim	31062000563
2837	310620005630338	URBANO	310620005008	Casa Branca	31062000563
2838	310620005630339	URBANO	310620005039	Jonas Veiga	31062000563
2839	310620005630340	URBANO	310620005032	Alto Vera Cruz	31062000563
2840	310620005630341	URBANO	310620005029	Saudade	31062000563
2841	310620005630342	URBANO	310620005044	ParaÝso	31062000563
2842	310620005630343	URBANO	310620005004	Sagrada FamÝlia	31062000563
2843	310620005630344	URBANO	310620005016	SÒo Geraldo	31062000563
2844	310620005630345	URBANO	310620005008	Casa Branca	31062000563
2845	310620005630346	URBANO	310620005032	Alto Vera Cruz	31062000563
2846	310620005630347	URBANO	310620005032	Alto Vera Cruz	31062000563
2847	310620005630348	URBANO	310620005032	Alto Vera Cruz	31062000563
2848	310620005630349	URBANO	310620005032	Alto Vera Cruz	31062000563
2849	310620005630350	URBANO	310620005025	Vera Cruz	31062000563
2850	310620005630351	URBANO	310620005039	Jonas Veiga	31062000563
2851	310620005630352	URBANO	310620005048	Vila ParaÝso	31062000563
2852	310620005630353	URBANO	310620005047	C¶nego Pinheiro 2¬	31062000563
2853	310620005630354	URBANO	310620005043	Santa EfigÛnia	31062000563
2854	310620005630355	URBANO	310620005044	ParaÝso	31062000563
2855	310620005630356	URBANO	310620005044	ParaÝso	31062000563
2856	310620005630357	URBANO	310620005005	Nova Vista	31062000563
2857	310620005630358	URBANO	310620005018	Vila SÒo Geraldo	31062000563
2858	310620005630359	URBANO	310620005006	Boa Vista	31062000563
2859	310620005630360	URBANO	310620005020	Mariano de Abreu	31062000563
2860	310620005630361	URBANO	310620005008	Casa Branca	31062000563
2861	310620005630362	URBANO	310620005016	SÒo Geraldo	31062000563
2862	310620005630363	URBANO	310620005008	Casa Branca	31062000563
2863	310620005630364	URBANO	310620005032	Alto Vera Cruz	31062000563
2864	310620005630365	URBANO	310620005032	Alto Vera Cruz	31062000563
2865	310620005630366	URBANO	310620005039	Jonas Veiga	31062000563
2866	310620005630367	URBANO	310620005043	Santa EfigÛnia	31062000563
2867	310620005630368	URBANO	310620005001	Santa InÛs	31062000563
2868	310620005630369	URBANO	310620005005	Nova Vista	31062000563
2869	310620005630370	URBANO	310620005006	Boa Vista	31062000563
2870	310620005630371	URBANO	310620005032	Alto Vera Cruz	31062000563
2871	310620005630372	URBANO	310620005039	Jonas Veiga	31062000563
2872	310620005630373	URBANO	310620005032	Alto Vera Cruz	31062000563
2873	310620005630374	URBANO	310620005039	Jonas Veiga	31062000563
2874	310620005630375	URBANO	310620005043	Santa EfigÛnia	31062000563
2875	310620005630376	URBANO	310620005011	Santa Tereza	31062000563
2876	310620005630377	URBANO	310620005011	Santa Tereza	31062000563

2877	310620005630378	URBANO	310620005011	Santa Tereza	31062000563
2878	310620005630379	URBANO	310620005001	Santa InÛs	31062000563
2879	310620005630380	URBANO	310620005001	Santa InÛs	31062000563
2880	310620005630381	URBANO	310620005005	Nova Vista	31062000563
2881	310620005630382	URBANO	310620005041	Cidade Jardim Taqu	31062000563
2882	310620005630383	URBANO	310620005042	Baleia	31062000563
2883	310620005630384	URBANO	310620005051	Fazendinha	31062000563
2884	310620005630385	URBANO	310620005044	ParaÝso	31062000563
2885	310620005630386	URBANO	310620005001	Santa InÛs	31062000563
2886	310620005630387	URBANO	310620005006	Boa Vista	31062000563
2887	310620005630388	URBANO	310620005006	Boa Vista	31062000563
2888	310620005630389	URBANO	310620005008	Casa Branca	31062000563
2889	310620005630390	URBANO	310620005049	Novo SÒo Lucas	31062000563
2890	310620005630391	URBANO	310620005035	Taquaril	31062000563
2891	310620005630392	URBANO	310620005038	Conjunto Taquaril	31062000563
2892	310620005630393	URBANO	310620005037	Vila da ┴ria	31062000563
2893	310620005630394	URBANO	310620005038	Conjunto Taquaril	31062000563
2894	310620005630395	URBANO	310620005040	Pirineus	31062000563
2895	310620005630396	URBANO	310620005036	Granja de Freitas	31062000563
2896	310620005630397	URBANO	310620005038	Conjunto Taquaril	31062000563
2897	310620005630398	URBANO	310620005038	Conjunto Taquaril	31062000563
3214	310620005650098	URBANO	310620005221	JoÒo Pinheiro	31062000565
3215	310620005650099	URBANO	310620005211	Delta	31062000565
3216	310620005650100	URBANO	310620005193	Alto dos Pinheiros	31062000565
3217	310620005650101	URBANO	310620005201	Calif¾rnia	31062000565
3218	310620005650102	URBANO	310620005212	Dom Bosco	31062000565
3219	310620005650103	URBANO	310620005204	Conjunto Calif¾rnia	31062000565
3220	310620005650104	URBANO	310620005204	Conjunto Calif¾rnia	31062000565
3221	310620005650105	URBANO	310620005204	Conjunto Calif¾rnia	31062000565
3222	310620005650106	URBANO	310620005212	Dom Bosco	31062000565
3223	310620005650107	URBANO	310620005212	Dom Bosco	31062000565
3337	310620005650163	URBANO	310620005232	Padre Eustßquio	31062000565
3338	310620005650164	URBANO	310620005232	Padre Eustßquio	31062000565
3339	310620005650165	URBANO	310620005232	Padre Eustßquio	31062000565
3340	310620005650166	URBANO	310620005232	Padre Eustßquio	31062000565
3341	310620005650167	URBANO	310620005232	Padre Eustßquio	31062000565
3342	310620005650168	URBANO	310620005225	Marmiteiros	31062000565
3343	310620005650169	URBANO	310620005210	CoraþÒo EucarÝstic	31062000565
3344	310620005650170	URBANO	310620005210	CoraþÒo EucarÝstic	31062000565
3345	310620005650171	URBANO	310620005210	CoraþÒo EucarÝstic	31062000565
3346	310620005650172	URBANO	310620005210	CoraþÒo EucarÝstic	31062000565
3433	310620005650259	URBANO	310620005216	Gl¾ria	31062000565
3434	310620005650260	URBANO	310620005216	Gl¾ria	31062000565
3435	310620005650261	URBANO	310620005191	AlÝpio de Melo	31062000565
3436	310620005650262	URBANO	310620005191	AlÝpio de Melo	31062000565
3437	310620005650263	URBANO	310620005239	SÒo Salvador	31062000565
3438	310620005650264	URBANO	310620005239	SÒo Salvador	31062000565
3439	310620005650265	URBANO	310620005239	SÒo Salvador	31062000565
3440	310620005650266	URBANO	310620005254	Vila Santo Ant¶nio I	31062000565
3441	310620005650267	URBANO	310620005241	Serrano	31062000565

3442	310620005650268	URBANO	310620005239	SÒo Salvador	31062000565
4208	310620005670471	URBANO	310620005127	Vila Calafate	31062000567
4209	310620005680003	URBANO	310620005261	Campos UFMG	31062000568
4210	310620005680004	URBANO	310620005277	Ouro Preto	31062000568
4211	310620005680005	URBANO	310620005277	Ouro Preto	31062000568
4212	310620005680006	URBANO	310620005277	Ouro Preto	31062000568
4213	310620005680007	URBANO	310620005277	Ouro Preto	31062000568
4214	310620005680008	URBANO	310620005277	Ouro Preto	31062000568
4215	310620005680009	URBANO	310620005277	Ouro Preto	31062000568
4216	310620005680010	URBANO	310620005277	Ouro Preto	31062000568
4217	310620005680011	URBANO	310620005278	Paquetß	31062000568
4218	310620005680012	URBANO	310620005278	Paquetß	31062000568
4498	310620025610019	URBANO	310620025347	Diamante	31062002561
4499	310620025610020	URBANO	310620025347	Diamante	31062002561
4500	310620025610021	URBANO	310620025347	Diamante	31062002561
4501	310620025610022	URBANO	310620025355	Teixeira Dias	31062002561
4502	310620025610023	URBANO	310620025347	Diamante	31062002561
4503	310620025610024	URBANO	310620025355	Teixeira Dias	31062002561
4504	310620025610025	URBANO	310620025354	Santa Helena	31062002561
4505	310620025610026	URBANO	310620025353	Miramar	31062002561
4506	310620025610027	URBANO	310620025353	Miramar	31062002561
4507	310620025610028	URBANO	310620025346	Brasil Industrial	31062002561
4508	310620025610029	URBANO	310620025346	Brasil Industrial	31062002561
4795	310620025610325	URBANO	310620025331	Vila Independencia	31062002561
4796	310620025610326	URBANO	310620025334	MineirÒo	31062002561
4797	310620025610327	URBANO	310620025334	MineirÒo	31062002561
4798	310620025610328	URBANO	310620025331	Vila Independencia	31062002561
4799	310620025610329	URBANO	310620025318	Conjunto Bonsucess	31062002561
4800	310620025610330	URBANO	310620025492	Itaipu	31062002561
4801	310620025610331	URBANO	310620025310	Vila Cemig	31062002561
4802	310620025610332	URBANO	310620025303	Vila Pilar	31062002561
4803	310620025610333	URBANO	310620025189	Pilar	31062002561
4804	310620025610334	URBANO	310620025344	Novo Santa Cecilia	31062002561
4805	310620025610335	URBANO	310620025329	Vitoria da Conquista	31062002561
4993	310620060640086	URBANO	310620060381	Maria Goretti	31062006064
4994	310620060640087	URBANO	310620060390	SÒo Gabriel	31062006064
4995	310620060640088	URBANO	310620060390	SÒo Gabriel	31062006064
4996	310620060640089	URBANO	310620060378	SÒo Benedito	31062006064
4997	310620060640090	URBANO	310620060378	SÒo Benedito	31062006064
4998	310620060640091	URBANO	310620060378	SÒo Benedito	31062006064
4999	310620060640092	URBANO	310620060376	Goiania	31062006064
5000	310620060640093	URBANO	310620060375	Ipe	31062006064
5001	310620060640094	URBANO	310620060375	Ipe	31062006064
5002	310620060640095	URBANO	310620060381	Maria Goretti	31062006064
5003	310620060640096	URBANO	310620060381	Maria Goretti	31062006064
2898	310620005630399	URBANO	310620005038	Conjunto Taquaril	31062000563
2899	310620005630400	URBANO	310620005038	Conjunto Taquaril	31062000563
2900	310620005630401	URBANO	310620005049	Novo SÒo Lucas	31062000563
2901	310620005630402	URBANO	310620005052	Nossa Senhora do R	31062000563
2902	310620005630403	URBANO	310620005052	Nossa Senhora do R	31062000563

2903	310620005630404	URBANO	310620005050	Vila Novo SÒo Lucas	31062000563
2904	310620005630405	URBANO	310620005049	Novo SÒo Lucas	31062000563
2905	310620005630406	URBANO	310620005050	Vila Novo SÒo Lucas	31062000563
2906	310620005630407	URBANO	310620005050	Vila Novo SÒo Lucas	31062000563
2907	310620005630408	URBANO	310620005050	Vila Novo SÒo Lucas	31062000563
2908	310620005630409	URBANO	310620005050	Vila Novo SÒo Lucas	31062000563
2909	310620005630410	URBANO	310620005051	Fazendinha	31062000563
2910	310620005640001	URBANO	310620005069	Ipiranga	31062000564
2911	310620005640002	URBANO	310620005069	Ipiranga	31062000564
2912	310620005640003	URBANO	310620005069	Ipiranga	31062000564
2913	310620005640004	URBANO	310620005069	Ipiranga	31062000564
2914	310620005640005	URBANO	310620005069	Ipiranga	31062000564
2915	310620005640006	URBANO	310620005069	Ipiranga	31062000564
2916	310620005640007	URBANO	310620005063	Silveira	31062000564
2917	310620005640008	URBANO	310620005064	Nova Floresta	31062000564
2918	310620005640009	URBANO	310620005065	Renascenþa	31062000564
2919	310620005640010	URBANO	310620005065	Renascenþa	31062000564
2920	310620005640011	URBANO	310620005065	Renascenþa	31062000564
2921	310620005640012	URBANO	310620005065	Renascenþa	31062000564
2922	310620005640013	URBANO	310620005066	Santa Cruz	31062000564
2923	310620005640022	URBANO	310620005064	Nova Floresta	31062000564
2924	310620005640023	URBANO	310620005065	Renascenþa	31062000564
2925	310620005640024	URBANO	310620005065	Renascenþa	31062000564
2926	310620005640025	URBANO	310620005065	Renascenþa	31062000564
2927	310620005640026	URBANO	310620005056	Cachoeirinha	31062000564
2928	310620005640027	URBANO	310620005066	Santa Cruz	31062000564
2929	310620005640028	URBANO	310620005066	Santa Cruz	31062000564
2930	310620005640029	URBANO	310620005066	Santa Cruz	31062000564
2931	310620005640030	URBANO	310620005069	Ipiranga	31062000564
2932	310620005640031	URBANO	310620005069	Ipiranga	31062000564
2933	310620005640032	URBANO	310620005077	Cidade Nova	31062000564
2934	310620005640033	URBANO	310620005077	Cidade Nova	31062000564
2935	310620005640034	URBANO	310620005064	Nova Floresta	31062000564
2936	310620005640035	URBANO	310620005065	Renascenþa	31062000564
2937	310620005640036	URBANO	310620005065	Renascenþa	31062000564
2938	310620005640037	URBANO	310620005065	Renascenþa	31062000564
2939	310620005640038	URBANO	310620005066	Santa Cruz	31062000564
2940	310620005640039	URBANO	310620005066	Santa Cruz	31062000564
2941	310620005640040	URBANO	310620005066	Santa Cruz	31062000564
2942	310620005640041	URBANO	310620005066	Santa Cruz	31062000564
2943	310620005640042	URBANO	310620005069	Ipiranga	31062000564
2944	310620005640043	URBANO	310620005069	Ipiranga	31062000564
2945	310620005640044	URBANO	310620005077	Cidade Nova	31062000564
2946	310620005640045	URBANO	310620005077	Cidade Nova	31062000564
2947	310620005640046	URBANO	310620005077	Cidade Nova	31062000564
2948	310620005640047	URBANO	310620005077	Cidade Nova	31062000564
2949	310620005640048	URBANO	310620005063	Silveira	31062000564
2950	310620005640049	URBANO	310620005063	Silveira	31062000564
2951	310620005640050	URBANO	310620005064	Nova Floresta	31062000564
2952	310620005640051	URBANO	310620005065	Renascenþa	31062000564

2953	310620005640052	URBANO	310620005065	Renascenþa	31062000564
2954	310620005640053	URBANO	310620005056	Cachoeirinha	31062000564
2955	310620005640054	URBANO	310620005056	Cachoeirinha	31062000564
2956	310620005640055	URBANO	310620005056	Cachoeirinha	31062000564
2957	310620005640056	URBANO	310620005066	Santa Cruz	31062000564
2958	310620005640057	URBANO	310620005066	Santa Cruz	31062000564
2959	310620005640058	URBANO	310620005067	Vila da Paz	31062000564
2960	310620005640059	URBANO	310620005066	Santa Cruz	31062000564
2961	310620005640060	URBANO	310620005066	Santa Cruz	31062000564
2962	310620005640061	URBANO	310620005066	Santa Cruz	31062000564
2963	310620005640062	URBANO	310620005066	Santa Cruz	31062000564
2964	310620005640063	URBANO	310620005066	Santa Cruz	31062000564
2965	310620005640064	URBANO	310620005066	Santa Cruz	31062000564
2966	310620005640065	URBANO	310620005066	Santa Cruz	31062000564
2967	310620005640066	URBANO	310620005069	Ipiranga	31062000564
2968	310620005640067	URBANO	310620005069	Ipiranga	31062000564
2969	310620005640068	URBANO	310620005069	Ipiranga	31062000564
2970	310620005640069	URBANO	310620005077	Cidade Nova	31062000564
2971	310620005640070	URBANO	310620005077	Cidade Nova	31062000564
2972	310620005640071	URBANO	310620005077	Cidade Nova	31062000564
2973	310620005640072	URBANO	310620005077	Cidade Nova	31062000564
2974	310620005640073	URBANO	310620005077	Cidade Nova	31062000564
2975	310620005640074	URBANO	310620005063	Silveira	31062000564
2976	310620005640075	URBANO	310620005078	UniÒo	31062000564
2977	310620005640076	URBANO	310620005064	Nova Floresta	31062000564
2978	310620005640077	URBANO	310620005064	Nova Floresta	31062000564
2979	310620005640078	URBANO	310620005059	Conc¾rdia	31062000564
2980	310620005640079	URBANO	310620005059	Conc¾rdia	31062000564
2981	310620005640080	URBANO	310620005065	Renascenþa	31062000564
2982	310620005640081	URBANO	310620005059	Conc¾rdia	31062000564
2983	310620005640082	URBANO	310620005056	Cachoeirinha	31062000564
2984	310620005640083	URBANO	310620005057	Vila Nova Cachoeiri	31062000564
2985	310620005640084	URBANO	310620005066	Santa Cruz	31062000564
2986	310620005640085	URBANO	310620005066	Santa Cruz	31062000564
2987	310620005640086	URBANO	310620005071	Palmares	31062000564
2988	310620005640087	URBANO	310620005071	Palmares	31062000564
2989	310620005640088	URBANO	310620005078	UniÒo	31062000564
2990	310620005640089	URBANO	310620005078	UniÒo	31062000564
2991	310620005640090	URBANO	310620005078	UniÒo	31062000564
2992	310620005640091	URBANO	310620005078	UniÒo	31062000564
2993	310620005640092	URBANO	310620005078	UniÒo	31062000564
2994	310620005640093	URBANO	310620005077	Cidade Nova	31062000564
2995	310620005640094	URBANO	310620005077	Cidade Nova	31062000564
2996	310620005640095	URBANO	310620005077	Cidade Nova	31062000564
2997	310620005640096	URBANO	310620005062	Graþa	31062000564
2998	310620005640097	URBANO	310620005062	Graþa	31062000564
2999	310620005640098	URBANO	310620005062	Graþa	31062000564
3000	310620005640099	URBANO	310620005059	Conc¾rdia	31062000564
3001	310620005640100	URBANO	310620005059	Conc¾rdia	31062000564
3002	310620005640101	URBANO	310620005059	Conc¾rdia	31062000564

3003	310620005640102	URBANO	310620005059	Conc¾rdia	31062000564
3004	310620005640103	URBANO	310620005059	Conc¾rdia	31062000564
3005	310620005640104	URBANO	310620005060	Tiradentes	31062000564
3006	310620005640105	URBANO	310620005060	Tiradentes	31062000564
3007	310620005640106	URBANO	310620005059	Conc¾rdia	31062000564
3008	310620005640107	URBANO	310620005058	Canadß	31062000564
3009	310620005640108	URBANO	310620005056	Cachoeirinha	31062000564
3010	310620005640109	URBANO	310620005056	Cachoeirinha	31062000564
3011	310620005640110	URBANO	310620005056	Cachoeirinha	31062000564
3012	310620005640111	URBANO	310620005056	Cachoeirinha	31062000564
3013	310620005640112	URBANO	310620005056	Cachoeirinha	31062000564
3014	310620005640113	URBANO	310620005056	Cachoeirinha	31062000564
3015	310620005640114	URBANO	310620005056	Cachoeirinha	31062000564
3016	310620005640115	URBANO	310620005066	Santa Cruz	31062000564
3017	310620005640116	URBANO	310620005066	Santa Cruz	31062000564
3018	310620005640117	URBANO	310620005066	Santa Cruz	31062000564
3019	310620005640118	URBANO	310620005071	Palmares	31062000564
3020	310620005640119	URBANO	310620005074	SÒo Paulo	31062000564
3021	310620005640120	URBANO	310620005078	UniÒo	31062000564
3022	310620005640121	URBANO	310620005078	UniÒo	31062000564
3023	310620005640122	URBANO	310620005078	UniÒo	31062000564
3024	310620005640123	URBANO	310620005078	UniÒo	31062000564
3025	310620005640124	URBANO	310620005078	UniÒo	31062000564
3026	310620005640125	URBANO	310620005078	UniÒo	31062000564
3027	310620005640126	URBANO	310620005055	SÒo Crist¾vÒo	31062000564
3028	310620005640127	URBANO	310620005066	Santa Cruz	31062000564
3029	310620005640128	URBANO	310620005066	Santa Cruz	31062000564
3030	310620005640129	URBANO	310620005072	Maria VirgÝnia	31062000564
3031	310620005640130	URBANO	310620005073	SÒo SebastiÒo	31062000564
3032	310620005640131	URBANO	310620005072	Maria VirgÝnia	31062000564
3033	310620005640132	URBANO	310620005072	Maria VirgÝnia	31062000564
3034	310620005640133	URBANO	310620005078	UniÒo	31062000564
3035	310620005640134	URBANO	310620005062	Graþa	31062000564
3036	310620005640135	URBANO	310620005059	Conc¾rdia	31062000564
3037	310620005640136	URBANO	310620005059	Conc¾rdia	31062000564
3038	310620005640137	URBANO	310620005059	Conc¾rdia	31062000564
3039	310620005640138	URBANO	310620005009	ColÚgio Batista	31062000564
3040	310620005640139	URBANO	310620005055	SÒo Crist¾vÒo	31062000564
3041	310620005640140	URBANO	310620005055	SÒo Crist¾vÒo	31062000564
3042	310620005640141	URBANO	310620005072	Maria VirgÝnia	31062000564
3043	310620005640142	URBANO	310620005072	Maria VirgÝnia	31062000564
3044	310620005640143	URBANO	310620005075	Andiroba	31062000564
3045	310620005640144	URBANO	310620005074	SÒo Paulo	31062000564
3046	310620005640145	URBANO	310620005074	SÒo Paulo	31062000564
3047	310620005640146	URBANO	310620005074	SÒo Paulo	31062000564
3048	310620005640147	URBANO	310620005074	SÒo Paulo	31062000564
3049	310620005640148	URBANO	310620005082	FernÒo Dias	31062000564
3050	310620005640149	URBANO	310620005082	FernÒo Dias	31062000564
3051	310620005640150	URBANO	310620005082	FernÒo Dias	31062000564
3052	310620005640151	URBANO	310620005080	Dom Joaquim	31062000564

3053	310620005640152	URBANO	310620005080	Dom Joaquim	31062000564
3054	310620005640153	URBANO	310620005080	Dom Joaquim	31062000564
3055	310620005640154	URBANO	310620005080	Dom Joaquim	31062000564
3056	310620005640155	URBANO	310620005080	Dom Joaquim	31062000564
3057	310620005640156	URBANO	310620005009	ColÚgio Batista	31062000564
3058	310620005640157	URBANO	310620005009	ColÚgio Batista	31062000564
3059	310620005640158	URBANO	310620005054	Lagoinha	31062000564
3060	310620005640159	URBANO	310620005054	Lagoinha	31062000564
3061	310620005640160	URBANO	310620005074	SÒo Paulo	31062000564
3062	310620005640161	URBANO	310620005081	Penha	31062000564
3063	310620005640162	URBANO	310620005081	Penha	31062000564
3064	310620005640163	URBANO	310620005081	Penha	31062000564
3065	310620005640164	URBANO	310620005077	Cidade Nova	31062000564
3066	310620005640165	URBANO	310620005063	Silveira	31062000564
3067	310620005640166	URBANO	310620005054	Lagoinha	31062000564
3068	310620005640167	URBANO	310620005009	ColÚgio Batista	31062000564
3069	310620005640168	URBANO	310620005059	Conc¾rdia	31062000564
3070	310620005640169	URBANO	310620005062	Graþa	31062000564
3071	310620005640170	URBANO	310620005062	Graþa	31062000564
3072	310620005640171	URBANO	310620005062	Graþa	31062000564
3073	310620005640172	URBANO	310620005062	Graþa	31062000564
3074	310620005640173	URBANO	310620005062	Graþa	31062000564
3075	310620005640174	URBANO	310620005064	Nova Floresta	31062000564
3076	310620005640175	URBANO	310620005064	Nova Floresta	31062000564
3077	310620005640176	URBANO	310620005062	Graþa	31062000564
3078	310620005640177	URBANO	310620005063	Silveira	31062000564
3079	310620005640178	URBANO	310620005068	Vila Inestan	31062000564
3080	310620005640179	URBANO	310620005066	Santa Cruz	31062000564
3081	310620005640180	URBANO	310620005056	Cachoeirinha	31062000564
3082	310620005640181	URBANO	310620005065	Renascenþa	31062000564
3083	310620005640182	URBANO	310620005070	Vila Ipiranga	31062000564
3084	310620005640183	URBANO	310620005069	Ipiranga	31062000564
3085	310620005640184	URBANO	310620005069	Ipiranga	31062000564
3086	310620005640185	URBANO	310620005069	Ipiranga	31062000564
3087	310620005640186	URBANO	310620005063	Silveira	31062000564
3088	310620005640187	URBANO	310620005063	Silveira	31062000564
3089	310620005640188	URBANO	310620005063	Silveira	31062000564
3090	310620005640189	URBANO	310620005064	Nova Floresta	31062000564
3091	310620005640190	URBANO	310620005078	UniÒo	31062000564
3092	310620005640191	URBANO	310620005064	Nova Floresta	31062000564
3093	310620005640192	URBANO	310620005059	Conc¾rdia	31062000564
3094	310620005640193	URBANO	310620005065	Renascenþa	31062000564
3095	310620005640194	URBANO	310620005056	Cachoeirinha	31062000564
3096	310620005640195	URBANO	310620005077	Cidade Nova	31062000564
3097	310620005640196	URBANO	310620005065	Renascenþa	31062000564
3098	310620005640197	URBANO	310620005066	Santa Cruz	31062000564
3099	310620005640198	URBANO	310620005071	Palmares	31062000564
3100	310620005640199	URBANO	310620005071	Palmares	31062000564
3101	310620005640200	URBANO	310620005078	UniÒo	31062000564
3102	310620005640201	URBANO	310620005078	UniÒo	31062000564

3103	310620005640202	URBANO	310620005077	Cidade Nova	31062000564
3104	310620005640203	URBANO	310620005078	UniÒo	31062000564
3105	310620005640204	URBANO	310620005059	Conc¾rdia	31062000564
3106	310620005640205	URBANO	310620005059	Conc¾rdia	31062000564
3107	310620005640206	URBANO	310620005061	Vila do Pombal	31062000564
3108	310620005640207	URBANO	310620005059	Conc¾rdia	31062000564
3109	310620005640208	URBANO	310620005065	Renascenþa	31062000564
3110	310620005640209	URBANO	310620005059	Conc¾rdia	31062000564
3111	310620005640210	URBANO	310620005056	Cachoeirinha	31062000564
3112	310620005640211	URBANO	310620005056	Cachoeirinha	31062000564
3113	310620005640212	URBANO	310620005059	Conc¾rdia	31062000564
3114	310620005640213	URBANO	310620005078	UniÒo	31062000564
3115	310620005640214	URBANO	310620005078	UniÒo	31062000564
3116	310620005640215	URBANO	310620005078	UniÒo	31062000564
3117	310620005640216	URBANO	310620005056	Cachoeirinha	31062000564
3118	310620005640217	URBANO	310620005066	Santa Cruz	31062000564
3119	310620005640218	URBANO	310620005071	Palmares	31062000564
3120	310620005640219	URBANO	310620005072	Maria VirgÝnia	31062000564
3121	310620005640220	URBANO	310620005071	Palmares	31062000564
3122	310620005640221	URBANO	310620005071	Palmares	31062000564
3123	310620005640222	URBANO	310620005072	Maria VirgÝnia	31062000564
3124	310620005640223	URBANO	310620005076	Vila SÒo Paulo	31062000564
3125	310620005640224	URBANO	310620005082	FernÒo Dias	31062000564
3126	310620005640225	URBANO	310620005074	SÒo Paulo	31062000564
3127	310620005640226	URBANO	310620005080	Dom Joaquim	31062000564
3128	310620005640227	URBANO	310620005080	Dom Joaquim	31062000564
3129	310620005640228	URBANO	310620005079	Vila de Sß	31062000564
3130	310620005640229	URBANO	310620005076	Vila SÒo Paulo	31062000564
3131	310620005640230	URBANO	310620005080	Dom Joaquim	31062000564
3132	310620005640231	URBANO	310620005059	Conc¾rdia	31062000564
3133	310620005650001	URBANO	310620005227	Monsenhor Messias	31062000565
3134	310620005650002	URBANO	310620005232	Padre Eustßquio	31062000565
3135	310620005650003	URBANO	310620005232	Padre Eustßquio	31062000565
3136	310620005650004	URBANO	310620005232	Padre Eustßquio	31062000565
3137	310620005650005	URBANO	310620005232	Padre Eustßquio	31062000565
3138	310620005650022	URBANO	310620005232	Padre Eustßquio	31062000565
3139	310620005650023	URBANO	310620005232	Padre Eustßquio	31062000565
3140	310620005650024	URBANO	310620005232	Padre Eustßquio	31062000565
3141	310620005650025	URBANO	310620005232	Padre Eustßquio	31062000565
3142	310620005650026	URBANO	310620005232	Padre Eustßquio	31062000565
3143	310620005650027	URBANO	310620005232	Padre Eustßquio	31062000565
3144	310620005650028	URBANO	310620005226	Minas Brasil	31062000565
3145	310620005650029	URBANO	310620005226	Minas Brasil	31062000565
3146	310620005650030	URBANO	310620005213	Dom Cabral	31062000565
3147	310620005650031	URBANO	310620005213	Dom Cabral	31062000565
3148	310620005650032	URBANO	310620005213	Dom Cabral	31062000565
3149	310620005650033	URBANO	310620005213	Dom Cabral	31062000565
3150	310620005650034	URBANO	310620005256	Vila Trinta e Um de	31062000565
3151	310620005650035	URBANO	310620005256	Vila Trinta e Um de	31062000565
3152	310620005650036	URBANO	310620005212	Dom Bosco	31062000565

3153	310620005650037	URBANO	310620005210	CoraþÒo EucarÝstic	31062000565
3154	310620005650038	URBANO	310620005226	Minas Brasil	31062000565
3155	310620005650039	URBANO	310620005232	Padre Eustßquio	31062000565
3156	310620005650040	URBANO	310620005232	Padre Eustßquio	31062000565
3157	310620005650041	URBANO	310620005212	Dom Bosco	31062000565
3158	310620005650042	URBANO	310620005212	Dom Bosco	31062000565
3159	310620005650043	URBANO	310620005217	InconfidÛncia	31062000565
3160	310620005650044	URBANO	310620005212	Dom Bosco	31062000565
3161	310620005650045	URBANO	310620005212	Dom Bosco	31062000565
3162	310620005650046	URBANO	310620005217	InconfidÛncia	31062000565
3163	310620005650047	URBANO	310620005220	Jardim SÒo JosÚ	31062000565
3164	310620005650048	URBANO	310620005220	Jardim SÒo JosÚ	31062000565
3165	310620005650049	URBANO	310620005220	Jardim SÒo JosÚ	31062000565
3166	310620005650050	URBANO	310620005220	Jardim SÒo JosÚ	31062000565
3167	310620005650051	URBANO	310620005220	Jardim SÒo JosÚ	31062000565
3168	310620005650052	URBANO	310620005220	Jardim SÒo JosÚ	31062000565
3169	310620005650053	URBANO	310620005232	Padre Eustßquio	31062000565
3170	310620005650054	URBANO	310620005219	Jardim MontanhÛs	31062000565
3171	310620005650055	URBANO	310620005219	Jardim MontanhÛs	31062000565
3172	310620005650056	URBANO	310620005219	Jardim MontanhÛs	31062000565
3173	310620005650057	URBANO	310620005199	Caiþara - Adelaide	31062000565
3174	310620005650058	URBANO	310620005199	Caiþara - Adelaide	31062000565
3175	310620005650059	URBANO	310620005199	Caiþara - Adelaide	31062000565
3176	310620005650060	URBANO	310620005192	Alto Caiþaras	31062000565
3177	310620005650061	URBANO	310620005199	Caiþara - Adelaide	31062000565
3178	310620005650062	URBANO	310620005192	Alto Caiþaras	31062000565
3179	310620005650063	URBANO	310620005200	Caiþaras	31062000565
3180	310620005650064	URBANO	310620005200	Caiþaras	31062000565
3181	310620005650065	URBANO	310620005200	Caiþaras	31062000565
3182	310620005650066	URBANO	310620005200	Caiþaras	31062000565
3183	310620005650067	URBANO	310620005200	Caiþaras	31062000565
3184	310620005650068	URBANO	310620005200	Caiþaras	31062000565
3185	310620005650069	URBANO	310620005200	Caiþaras	31062000565
3186	310620005650070	URBANO	310620005200	Caiþaras	31062000565
3187	310620005650071	URBANO	310620005199	Caiþara - Adelaide	31062000565
3188	310620005650072	URBANO	310620005200	Caiþaras	31062000565
3189	310620005650073	URBANO	310620005200	Caiþaras	31062000565
3190	310620005650074	URBANO	310620005200	Caiþaras	31062000565
3191	310620005650075	URBANO	310620005199	Caiþara - Adelaide	31062000565
3192	310620005650076	URBANO	310620005200	Caiþaras	31062000565
3193	310620005650077	URBANO	310620005227	Monsenhor Messias	31062000565
3194	310620005650078	URBANO	310620005232	Padre Eustßquio	31062000565
3195	310620005650079	URBANO	310620005232	Padre Eustßquio	31062000565
3196	310620005650080	URBANO	310620005232	Padre Eustßquio	31062000565
3197	310620005650081	URBANO	310620005232	Padre Eustßquio	31062000565
3198	310620005650082	URBANO	310620005232	Padre Eustßquio	31062000565
3199	310620005650083	URBANO	310620005210	CoraþÒo EucarÝstic	31062000565
3200	310620005650084	URBANO	310620005210	CoraþÒo EucarÝstic	31062000565
3201	310620005650085	URBANO	310620005210	CoraþÒo EucarÝstic	31062000565
3202	310620005650086	URBANO	310620005210	CoraþÒo EucarÝstic	31062000565

3203	310620005650087	URBANO	310620005210	CoraþÒo EucarÝstic	31062000565
3204	310620005650088	URBANO	310620005213	Dom Cabral	31062000565
3205	310620005650089	URBANO	310620005221	JoÒo Pinheiro	31062000565
3206	310620005650090	URBANO	310620005213	Dom Cabral	31062000565
3207	310620005650091	URBANO	310620005221	JoÒo Pinheiro	31062000565
3208	310620005650092	URBANO	310620005221	JoÒo Pinheiro	31062000565
3209	310620005650093	URBANO	310620005256	Vila Trinta e Um de	31062000565
3210	310620005650094	URBANO	310620005221	JoÒo Pinheiro	31062000565
3211	310620005650095	URBANO	310620005221	JoÒo Pinheiro	31062000565
3212	310620005650096	URBANO	310620005221	JoÒo Pinheiro	31062000565
3213	310620005650097	URBANO	310620005221	JoÒo Pinheiro	31062000565
3224	310620005650108	URBANO	310620005212	Dom Bosco	31062000565
3225	310620005650109	URBANO	310620005212	Dom Bosco	31062000565
3226	310620005650110	URBANO	310620005212	Dom Bosco	31062000565
3227	310620005650111	URBANO	310620005194	┴lvaro Camargos	31062000565
3228	310620005650112	URBANO	310620005216	Gl¾ria	31062000565
3229	310620005650113	URBANO	310620005216	Gl¾ria	31062000565
3230	310620005650114	URBANO	310620005207	Conjunto Jardim Fila	31062000565
3231	310620005650115	URBANO	310620005216	Gl¾ria	31062000565
3232	310620005650116	URBANO	310620005216	Gl¾ria	31062000565
3233	310620005650117	URBANO	310620005216	Gl¾ria	31062000565
3234	310620005650118	URBANO	310620005207	Conjunto Jardim Fila	31062000565
3235	310620005650119	URBANO	310620005207	Conjunto Jardim Fila	31062000565
3236	310620005650120	URBANO	310620005230	Novo Gl¾ria	31062000565
3237	310620005650121	URBANO	310620005230	Novo Gl¾ria	31062000565
3238	310620005650122	URBANO	310620005230	Novo Gl¾ria	31062000565
3239	310620005650123	URBANO	310620005230	Novo Gl¾ria	31062000565
3240	310620005650124	URBANO	310620005230	Novo Gl¾ria	31062000565
3241	310620005650125	URBANO	310620005230	Novo Gl¾ria	31062000565
3242	310620005650126	URBANO	310620005230	Novo Gl¾ria	31062000565
3243	310620005650127	URBANO	310620005216	Gl¾ria	31062000565
3244	310620005650128	URBANO	310620005216	Gl¾ria	31062000565
3245	310620005650129	URBANO	310620005216	Gl¾ria	31062000565
3246	310620005650130	URBANO	310620005217	InconfidÛncia	31062000565
3247	310620005650131	URBANO	310620005249	Vila Jardim SÒo Jos	31062000565
3248	310620005650132	URBANO	310620005249	Vila Jardim SÒo Jos	31062000565
3249	310620005650133	URBANO	310620005199	Caiþara - Adelaide	31062000565
3250	310620005650134	URBANO	310620005192	Alto Caiþaras	31062000565
3251	310620005650396	URBANO	310620005203	Carlos Prates	31062000565
3252	310620005650397	URBANO	310620005203	Carlos Prates	31062000565
3253	310620005650398	URBANO	310620005203	Carlos Prates	31062000565
3254	310620005650399	URBANO	310620005203	Carlos Prates	31062000565
3255	310620005650400	URBANO	310620005232	Padre Eustßquio	31062000565
3256	310620005650401	URBANO	310620005232	Padre Eustßquio	31062000565
3257	310620005650402	URBANO	310620005232	Padre Eustßquio	31062000565
3258	310620005650403	URBANO	310620005232	Padre Eustßquio	31062000565
3259	310620005650404	URBANO	310620005232	Padre Eustßquio	31062000565
3260	310620005650405	URBANO	310620005232	Padre Eustßquio	31062000565
3261	310620005650406	URBANO	310620005232	Padre Eustßquio	31062000565
3262	310620005650407	URBANO	310620005232	Padre Eustßquio	31062000565

3263	310620005650408	URBANO	310620005232	Padre Eustßquio	31062000565
3264	310620005650409	URBANO	310620005232	Padre Eustßquio	31062000565
3265	310620005650410	URBANO	310620005232	Padre Eustßquio	31062000565
3266	310620005650411	URBANO	310620005232	Padre Eustßquio	31062000565
3267	310620005650549	URBANO	310620005196	Aparecida 7¬ SeþÒc	31062000565
3268	310620005650550	URBANO	310620005196	Aparecida 7¬ SeþÒc	31062000565
3269	310620005650551	URBANO	310620005196	Aparecida 7¬ SeþÒc	31062000565
3270	310620005650552	URBANO	310620005228	Nova Cachoeirinha	31062000565
3271	310620005650553	URBANO	310620005215	Ermelinda	31062000565
3272	310620005650554	URBANO	310620005228	Nova Cachoeirinha	31062000565
3273	310620005650555	URBANO	310620005228	Nova Cachoeirinha	31062000565
3274	310620005650556	URBANO	310620005251	Vila Nova Cachoeiri	31062000565
3275	310620005670355	URBANO	310620005188	Vila BetÔnia	31062000567
3276	310620005670356	URBANO	310620005152	Bairro das Ind·strias	31062000567
3277	310620005670357	URBANO	310620005143	Vila Vista Alegre	31062000567
3278	310620005670358	URBANO	310620005151	Vista Alegre	31062000567
3279	310620005670359	URBANO	310620005145	Emba·bas	31062000567
3280	310620005670360	URBANO	310620005146	Nova Gameleira	31062000567
3281	310620005670361	URBANO	310620005145	Emba·bas	31062000567
3282	310620005670362	URBANO	310620005146	Nova Gameleira	31062000567
3283	310620005670472	URBANO	310620005162	Vila da Amizade	31062000567
3284	310620005670473	URBANO	310620005135	Nova SuÝþa	31062000567
3285	310620005670474	URBANO	310620005135	Nova SuÝþa	31062000567
3286	310620005670475	URBANO	310620005175	SÒo Jorge 1¬ SeþÒc	31062000567
3287	310620005670476	URBANO	310620005175	SÒo Jorge 1¬ SeþÒc	31062000567
3288	310620005670477	URBANO	310620005177	SÒo Jorge 3¬ SeþÒc	31062000567
3289	310620005670478	URBANO	310620005126	Prado	31062000567
3290	310620005670479	URBANO	310620005131	Graja·	31062000567
3291	310620025610174	URBANO	310620025357	Santa Margarida	31062002561
3292	310620025610175	URBANO	310620025357	Santa Margarida	31062002561
3293	310620025610176	URBANO	310620025356	Barreiro	31062002561
3294	310620025610177	URBANO	310620025356	Barreiro	31062002561
3295	310620025610178	URBANO	310620025322	das Industrias I	31062002561
3296	310620025610179	URBANO	310620025322	das Industrias I	31062002561
3297	310620025610180	URBANO	310620025322	das Industrias I	31062002561
3298	310620025610181	URBANO	310620025322	das Industrias I	31062002561
3299	310620025610182	URBANO	310620025322	das Industrias I	31062002561
3300	310620060640197	URBANO	310620060376	Goiania	31062006064
3301	310620060640198	URBANO	310620060376	Goiania	31062006064
3302	310620060640199	URBANO	310620060381	Maria Goretti	31062006064
3303	310620060640200	URBANO	310620060381	Maria Goretti	31062006064
3304	310620060640201	URBANO	310620060382	Piraja	31062006064
3305	310620060640202	URBANO	310620060411	CapitÒo Eduardo	31062006064
3306	310620060640203	URBANO	310620060377	Morro dos Macacos	31062006064
3307	310620060640204	URBANO	310620060375	Ipe	31062006064
3308	310620060640205	URBANO	310620060374	SÒo Marcos	31062006064
3309	310620005650135	URBANO	310620005192	Alto Caiþaras	31062000565
3310	310620005650136	URBANO	310620005192	Alto Caiþaras	31062000565
3311	310620005650137	URBANO	310620005192	Alto Caiþaras	31062000565
3312	310620005650138	URBANO	310620005200	Caiþaras	31062000565

3313	310620005650139	URBANO	310620005200	Caiþaras	31062000565
3314	310620005650140	URBANO	310620005200	Caiþaras	31062000565
3315	310620005650141	URBANO	310620005196	Aparecida 7¬ SeþÒc	31062000565
3316	310620005650142	URBANO	310620005196	Aparecida 7¬ SeþÒc	31062000565
3317	310620005650143	URBANO	310620005196	Aparecida 7¬ SeþÒc	31062000565
3318	310620005650144	URBANO	310620005195	Aparecida	31062000565
3319	310620005650145	URBANO	310620005195	Aparecida	31062000565
3320	310620005650146	URBANO	310620005197	Bom Jesus	31062000565
3321	310620005650147	URBANO	310620005229	Nova Esperanþa	31062000565
3322	310620005650148	URBANO	310620005229	Nova Esperanþa	31062000565
3323	310620005650149	URBANO	310620005236	Santo AndrÚ	31062000565
3324	310620005650150	URBANO	310620005236	Santo AndrÚ	31062000565
3325	310620005650151	URBANO	310620005229	Nova Esperanþa	31062000565
3326	310620005650152	URBANO	310620005229	Nova Esperanþa	31062000565
3327	310620005650153	URBANO	310620005229	Nova Esperanþa	31062000565
3328	310620005650154	URBANO	310620005236	Santo AndrÚ	31062000565
3329	310620005650155	URBANO	310620005236	Santo AndrÚ	31062000565
3330	310620005650156	URBANO	310620005236	Santo AndrÚ	31062000565
3331	310620005650157	URBANO	310620005203	Carlos Prates	31062000565
3332	310620005650158	URBANO	310620005200	Caiþaras	31062000565
3333	310620005650159	URBANO	310620005203	Carlos Prates	31062000565
3334	310620005650160	URBANO	310620005203	Carlos Prates	31062000565
3335	310620005650161	URBANO	310620005203	Carlos Prates	31062000565
3336	310620005650162	URBANO	310620005232	Padre Eustßquio	31062000565
3347	310620005650173	URBANO	310620005210	CoraþÒo EucarÝstic	31062000565
3348	310620005650174	URBANO	310620005210	CoraþÒo EucarÝstic	31062000565
3349	310620005650175	URBANO	310620005210	CoraþÒo EucarÝstic	31062000565
3350	310620005650176	URBANO	310620005231	Oeste	31062000565
3351	310620005650177	URBANO	310620005231	Oeste	31062000565
3352	310620005650178	URBANO	310620005221	JoÒo Pinheiro	31062000565
3353	310620005650179	URBANO	310620005221	JoÒo Pinheiro	31062000565
3354	310620005650180	URBANO	310620005221	JoÒo Pinheiro	31062000565
3355	310620005650181	URBANO	310620005221	JoÒo Pinheiro	31062000565
3356	310620005650182	URBANO	310620005193	Alto dos Pinheiros	31062000565
3357	310620005650183	URBANO	310620005221	JoÒo Pinheiro	31062000565
3358	310620005650184	URBANO	310620005193	Alto dos Pinheiros	31062000565
3359	310620005650185	URBANO	310620005231	Oeste	31062000565
3360	310620005650186	URBANO	310620005231	Oeste	31062000565
3361	310620005650187	URBANO	310620005231	Oeste	31062000565
3362	310620005650188	URBANO	310620005159	Camargos	31062000565
3363	310620005650189	URBANO	310620005235	Santa Maria	31062000565
3364	310620005650190	URBANO	310620005159	Camargos	31062000565
3365	310620005650191	URBANO	310620005159	Camargos	31062000565
3366	310620005650192	URBANO	310620005159	Camargos	31062000565
3367	310620005650193	URBANO	310620005159	Camargos	31062000565
3368	310620005650194	URBANO	310620005159	Camargos	31062000565
3369	310620005650195	URBANO	310620005159	Camargos	31062000565
3370	310620005650196	URBANO	310620005159	Camargos	31062000565
3371	310620005650197	URBANO	310620005159	Camargos	31062000565
3372	310620005650198	URBANO	310620005159	Camargos	31062000565

3373	310620005650199	URBANO	310620005159	Camargos	31062000565
3374	310620005650200	URBANO	310620005235	Santa Maria	31062000565
3375	310620005650201	URBANO	310620005235	Santa Maria	31062000565
3376	310620005650202	URBANO	310620005235	Santa Maria	31062000565
3377	310620005650203	URBANO	310620005235	Santa Maria	31062000565
3378	310620005650204	URBANO	310620005235	Santa Maria	31062000565
3379	310620005650205	URBANO	310620005235	Santa Maria	31062000565
3380	310620005650206	URBANO	310620005201	Calif¾rnia	31062000565
3381	310620005650207	URBANO	310620005204	Conjunto Calif¾rnia	31062000565
3382	310620005650208	URBANO	310620005201	Calif¾rnia	31062000565
3383	310620005650209	URBANO	310620005205	Conjunto Calif¾rnia	31062000565
3384	310620005650210	URBANO	310620005205	Conjunto Calif¾rnia	31062000565
3385	310620005650211	URBANO	310620005244	Vila Calif¾rnia	31062000565
3386	310620005650212	URBANO	310620005244	Vila Calif¾rnia	31062000565
3387	310620005650213	URBANO	310620005244	Vila Calif¾rnia	31062000565
3388	310620005650214	URBANO	310620005244	Vila Calif¾rnia	31062000565
3389	310620005650215	URBANO	310620005244	Vila Calif¾rnia	31062000565
3390	310620005650216	URBANO	310620005201	Calif¾rnia	31062000565
3391	310620005650217	URBANO	310620005201	Calif¾rnia	31062000565
3392	310620005650218	URBANO	310620005201	Calif¾rnia	31062000565
3393	310620005650219	URBANO	310620005234	Pindorama	31062000565
3394	310620005650220	URBANO	310620005234	Pindorama	31062000565
3395	310620005650221	URBANO	310620005234	Pindorama	31062000565
3396	310620005650222	URBANO	310620005234	Pindorama	31062000565
3397	310620005650223	URBANO	310620005245	Vila Coqueiral	31062000565
3398	310620005650224	URBANO	310620005245	Vila Coqueiral	31062000565
3399	310620005650225	URBANO	310620005234	Pindorama	31062000565
3400	310620005650226	URBANO	310620005245	Vila Coqueiral	31062000565
3401	310620005650227	URBANO	310620005234	Pindorama	31062000565
3402	310620005650228	URBANO	310620005234	Pindorama	31062000565
3403	310620005650229	URBANO	310620005234	Pindorama	31062000565
3404	310620005650230	URBANO	310620005234	Pindorama	31062000565
3405	310620005650231	URBANO	310620005234	Pindorama	31062000565
3406	310620005650232	URBANO	310620005234	Pindorama	31062000565
3407	310620005650233	URBANO	310620005234	Pindorama	31062000565
3408	310620005650234	URBANO	310620005234	Pindorama	31062000565
3409	310620005650235	URBANO	310620005234	Pindorama	31062000565
3410	310620005650236	URBANO	310620005234	Pindorama	31062000565
3411	310620005650237	URBANO	310620005234	Pindorama	31062000565
3412	310620005650238	URBANO	310620005234	Pindorama	31062000565
3413	310620005650239	URBANO	310620005234	Pindorama	31062000565
3414	310620005650240	URBANO	310620005234	Pindorama	31062000565
3415	310620005650241	URBANO	310620005230	Novo Gl¾ria	31062000565
3416	310620005650242	URBANO	310620005230	Novo Gl¾ria	31062000565
3417	310620005650243	URBANO	310620005216	Gl¾ria	31062000565
3418	310620005650244	URBANO	310620005209	Coqueiros	31062000565
3419	310620005650245	URBANO	310620005209	Coqueiros	31062000565
3420	310620005650246	URBANO	310620005209	Coqueiros	31062000565
3421	310620005650247	URBANO	310620005209	Coqueiros	31062000565
3422	310620005650248	URBANO	310620005209	Coqueiros	31062000565

3423	310620005650249	URBANO	310620005209	Coqueiros	31062000565
3424	310620005650250	URBANO	310620005209	Coqueiros	31062000565
3425	310620005650251	URBANO	310620005239	SÒo Salvador	31062000565
3426	310620005650252	URBANO	310620005239	SÒo Salvador	31062000565
3427	310620005650253	URBANO	310620005239	SÒo Salvador	31062000565
3428	310620005650254	URBANO	310620005216	Gl¾ria	31062000565
3429	310620005650255	URBANO	310620005216	Gl¾ria	31062000565
3430	310620005650256	URBANO	310620005216	Gl¾ria	31062000565
3431	310620005650257	URBANO	310620005216	Gl¾ria	31062000565
3432	310620005650258	URBANO	310620005216	Gl¾ria	31062000565
3443	310620005650269	URBANO	310620005241	Serrano	31062000565
3444	310620005650270	URBANO	310620005241	Serrano	31062000565
3445	310620005650271	URBANO	310620005206	Conjunto Celso Mac	31062000565
3446	310620005650272	URBANO	310620005241	Serrano	31062000565
3447	310620005650273	URBANO	310620005206	Conjunto Celso Mac	31062000565
3448	310620005650274	URBANO	310620005206	Conjunto Celso Mac	31062000565
3449	310620005650275	URBANO	310620005191	AlÝpio de Melo	31062000565
3450	310620005650276	URBANO	310620005191	AlÝpio de Melo	31062000565
3451	310620005650277	URBANO	310620005191	AlÝpio de Melo	31062000565
3452	310620005650278	URBANO	310620005191	AlÝpio de Melo	31062000565
3453	310620005650279	URBANO	310620005191	AlÝpio de Melo	31062000565
3454	310620005650280	URBANO	310620005191	AlÝpio de Melo	31062000565
3455	310620005650281	URBANO	310620005191	AlÝpio de Melo	31062000565
3456	310620005650282	URBANO	310620005191	AlÝpio de Melo	31062000565
3457	310620005650283	URBANO	310620005191	AlÝpio de Melo	31062000565
3458	310620005650284	URBANO	310620005191	AlÝpio de Melo	31062000565
3459	310620005650285	URBANO	310620005191	AlÝpio de Melo	31062000565
3460	310620005650286	URBANO	310620005217	InconfidÛncia	31062000565
3461	310620005650287	URBANO	310620005220	Jardim SÒo JosÚ	31062000565
3462	310620005650288	URBANO	310620005220	Jardim SÒo JosÚ	31062000565
3463	310620005650289	URBANO	310620005220	Jardim SÒo JosÚ	31062000565
3464	310620005650290	URBANO	310620005249	Vila Jardim SÒo Jos	31062000565
3465	310620005650291	URBANO	310620005194	┴lvaro Camargos	31062000565
3466	310620005650292	URBANO	310620005208	Conjunto Novo Dom	31062000565
3467	310620005650293	URBANO	310620005249	Vila Jardim SÒo Jos	31062000565
3468	310620005650294	URBANO	310620005249	Vila Jardim SÒo Jos	31062000565
3469	310620005650295	URBANO	310620005249	Vila Jardim SÒo Jos	31062000565
3470	310620005650296	URBANO	310620005218	Jardim Alvorada	31062000565
3471	310620005650297	URBANO	310620005218	Jardim Alvorada	31062000565
3472	310620005650298	URBANO	310620005218	Jardim Alvorada	31062000565
3473	310620005650299	URBANO	310620005218	Jardim Alvorada	31062000565
3474	310620005650300	URBANO	310620005218	Jardim Alvorada	31062000565
3475	310620005650301	URBANO	310620005218	Jardim Alvorada	31062000565
3476	310620005650302	URBANO	310620005218	Jardim Alvorada	31062000565
3477	310620005650303	URBANO	310620005218	Jardim Alvorada	31062000565
3478	310620005650304	URBANO	310620005247	Vila Jardim Alvorada	31062000565
3479	310620005650305	URBANO	310620005218	Jardim Alvorada	31062000565
3480	310620005650306	URBANO	310620005247	Vila Jardim Alvorada	31062000565
3481	310620005650307	URBANO	310620005247	Vila Jardim Alvorada	31062000565
3482	310620005650308	URBANO	310620005243	Vila Antena Montar	31062000565

3483	310620005650309	URBANO	310620005218	Jardim Alvorada	31062000565
3484	310620005650310	URBANO	310620005218	Jardim Alvorada	31062000565
3485	310620005650311	URBANO	310620005214	Engenho Nogueira	31062000565
3486	310620005650312	URBANO	310620005192	Alto Caiþaras	31062000565
3487	310620005650313	URBANO	310620005200	Caiþaras	31062000565
3488	310620005650314	URBANO	310620005200	Caiþaras	31062000565
3489	310620005650315	URBANO	310620005200	Caiþaras	31062000565
3490	310620005650316	URBANO	310620005196	Aparecida 7¬ SeþÒc	31062000565
3491	310620005650317	URBANO	310620005196	Aparecida 7¬ SeþÒc	31062000565
3492	310620005650318	URBANO	310620005196	Aparecida 7¬ SeþÒc	31062000565
3493	310620005650319	URBANO	310620005215	Ermelinda	31062000565
3494	310620005650320	URBANO	310620005255	Vila SumarÚ	31062000565
3495	310620005650321	URBANO	310620005255	Vila SumarÚ	31062000565
3496	310620005650322	URBANO	310620005255	Vila SumarÚ	31062000565
3497	310620005650323	URBANO	310620005255	Vila SumarÚ	31062000565
3498	310620005650324	URBANO	310620005215	Ermelinda	31062000565
3499	310620005650325	URBANO	310620005215	Ermelinda	31062000565
3500	310620005650326	URBANO	310620005228	Nova Cachoeirinha	31062000565
3501	310620005650327	URBANO	310620005228	Nova Cachoeirinha	31062000565
3502	310620005650328	URBANO	310620005215	Ermelinda	31062000565
3503	310620005650329	URBANO	310620005228	Nova Cachoeirinha	31062000565
3504	310620005650330	URBANO	310620005228	Nova Cachoeirinha	31062000565
3505	310620005650331	URBANO	310620005228	Nova Cachoeirinha	31062000565
3506	310620005650332	URBANO	310620005222	Vila Maloca	31062000565
3507	310620005650333	URBANO	310620005250	Vila Nova Cachoeiri	31062000565
3508	310620005650334	URBANO	310620005250	Vila Nova Cachoeiri	31062000565
3509	310620005650335	URBANO	310620005250	Vila Nova Cachoeiri	31062000565
3510	310620005650336	URBANO	310620005228	Nova Cachoeirinha	31062000565
3511	310620005650337	URBANO	310620005195	Aparecida	31062000565
3512	310620005650338	URBANO	310620005195	Aparecida	31062000565
3513	310620005650339	URBANO	310620005195	Aparecida	31062000565
3514	310620005650340	URBANO	310620005195	Aparecida	31062000565
3515	310620005650341	URBANO	310620005195	Aparecida	31062000565
3516	310620005650342	URBANO	310620005195	Aparecida	31062000565
3517	310620005650343	URBANO	310620005195	Aparecida	31062000565
3518	310620005650344	URBANO	310620005195	Aparecida	31062000565
3519	310620005650345	URBANO	310620005195	Aparecida	31062000565
3520	310620005650346	URBANO	310620005197	Bom Jesus	31062000565
3521	310620005650347	URBANO	310620005197	Bom Jesus	31062000565
3522	310620005650348	URBANO	310620005197	Bom Jesus	31062000565
3523	310620005650349	URBANO	310620005197	Bom Jesus	31062000565
3524	310620005650350	URBANO	310620005195	Aparecida	31062000565
3525	310620005650351	URBANO	310620005195	Aparecida	31062000565
3526	310620005650352	URBANO	310620005236	Santo AndrÚ	31062000565
3527	310620005650353	URBANO	310620005236	Santo AndrÚ	31062000565
3528	310620005650354	URBANO	310620005233	Pedreira Padro Lope	31062000565
3529	310620005650355	URBANO	310620005233	Pedreira Padro Lope	31062000565
3530	310620005650356	URBANO	310620005233	Pedreira Padro Lope	31062000565
3531	310620005650357	URBANO	310620005233	Pedreira Padro Lope	31062000565
3532	310620005650358	URBANO	310620005233	Pedreira Padro Lope	31062000565

3533	310620005650359	URBANO	310620005055	SÒo Crist¾vÒo	31062000565
3534	310620005650360	URBANO	310620005055	SÒo Crist¾vÒo	31062000565
3535	310620005650361	URBANO	310620005055	SÒo Crist¾vÒo	31062000565
3536	310620005650362	URBANO	310620005054	Lagoinha	31062000565
3537	310620005650363	URBANO	310620005233	Pedreira Padro Lope	31062000565
3538	310620005650364	URBANO	310620005233	Pedreira Padro Lope	31062000565
3539	310620005650365	URBANO	310620005240	Senhor dos Passos	31062000565
3540	310620005650366	URBANO	310620005240	Senhor dos Passos	31062000565
3541	310620005650367	URBANO	310620005240	Senhor dos Passos	31062000565
3542	310620005650368	URBANO	310620005054	Lagoinha	31062000565
3543	310620005650369	URBANO	310620005240	Senhor dos Passos	31062000565
3544	310620005650370	URBANO	310620005198	Bonfim	31062000565
3545	310620005650371	URBANO	310620005198	Bonfim	31062000565
3546	310620005650372	URBANO	310620005236	Santo AndrÚ	31062000565
3547	310620005650373	URBANO	310620005198	Bonfim	31062000565
3548	310620005650374	URBANO	310620005198	Bonfim	31062000565
3549	310620005650375	URBANO	310620005198	Bonfim	31062000565
3550	310620005650376	URBANO	310620005054	Lagoinha	31062000565
3551	310620005650377	URBANO	310620005198	Bonfim	31062000565
3552	310620005650378	URBANO	310620005054	Lagoinha	31062000565
3553	310620005650379	URBANO	310620005198	Bonfim	31062000565
3554	310620005650380	URBANO	310620005203	Carlos Prates	31062000565
3555	310620005650381	URBANO	310620005203	Carlos Prates	31062000565
3556	310620005650382	URBANO	310620005203	Carlos Prates	31062000565
3557	310620005650383	URBANO	310620005203	Carlos Prates	31062000565
3558	310620005650384	URBANO	310620005203	Carlos Prates	31062000565
3559	310620005650385	URBANO	310620005203	Carlos Prates	31062000565
3560	310620005650386	URBANO	310620005203	Carlos Prates	31062000565
3561	310620005650387	URBANO	310620005198	Bonfim	31062000565
3562	310620005650388	URBANO	310620005203	Carlos Prates	31062000565
3563	310620005650389	URBANO	310620005203	Carlos Prates	31062000565
3564	310620005650390	URBANO	310620005203	Carlos Prates	31062000565
3565	310620005650391	URBANO	310620005203	Carlos Prates	31062000565
3566	310620005650392	URBANO	310620005203	Carlos Prates	31062000565
3567	310620005650393	URBANO	310620005203	Carlos Prates	31062000565
3568	310620005650394	URBANO	310620005238	SÒo Francisco das C	31062000565
3569	310620005650395	URBANO	310620005203	Carlos Prates	31062000565
3570	310620005650412	URBANO	310620005232	Padre Eustßquio	31062000565
3571	310620005650413	URBANO	310620005232	Padre Eustßquio	31062000565
3572	310620005650414	URBANO	310620005227	Monsenhor Messias	31062000565
3573	310620005650415	URBANO	310620005219	Jardim MontanhÛs	31062000565
3574	310620005650416	URBANO	310620005223	Lorena	31062000565
3575	310620005650417	URBANO	310620005226	Minas Brasil	31062000565
3576	310620005650418	URBANO	310620005226	Minas Brasil	31062000565
3577	310620005650419	URBANO	310620005226	Minas Brasil	31062000565
3578	310620005650420	URBANO	310620005227	Monsenhor Messias	31062000565
3579	310620005650421	URBANO	310620005219	Jardim MontanhÛs	31062000565
3580	310620005650422	URBANO	310620005199	Caiþara - Adelaide	31062000565
3581	310620005650423	URBANO	310620005227	Monsenhor Messias	31062000565
3582	310620005650424	URBANO	310620005227	Monsenhor Messias	31062000565

3583	310620005650425	URBANO	310620005199	Caiþara - Adelaide	31062000565
3584	310620005650426	URBANO	310620005232	Padre Eustßquio	31062000565
3585	310620005650427	URBANO	310620005213	Dom Cabral	31062000565
3586	310620005650428	URBANO	310620005213	Dom Cabral	31062000565
3587	310620005650429	URBANO	310620005253	Vila Puc	31062000565
3588	310620005650430	URBANO	310620005213	Dom Cabral	31062000565
3589	310620005650431	URBANO	310620005226	Minas Brasil	31062000565
3590	310620005650432	URBANO	310620005216	Gl¾ria	31062000565
3591	310620005650433	URBANO	310620005249	Vila Jardim SÒo JosÚ	31062000565
3592	310620005650434	URBANO	310620005220	Jardim SÒo JosÚ	31062000565
3593	310620005650435	URBANO	310620005249	Vila Jardim SÒo JosÚ	31062000565
3594	310620005650436	URBANO	310620005219	Jardim MontanhÛs	31062000565
3595	310620005650437	URBANO	310620005199	Caiþara - Adelaide	31062000565
3596	310620005650438	URBANO	310620005199	Caiþara - Adelaide	31062000565
3597	310620005650439	URBANO	310620005192	Alto Caiþaras	31062000565
3598	310620005650440	URBANO	310620005200	Caiþaras	31062000565
3599	310620005650441	URBANO	310620005229	Nova Esperanþa	31062000565
3600	310620005650442	URBANO	310620005199	Caiþara - Adelaide	31062000565
3601	310620005650443	URBANO	310620005200	Caiþaras	31062000565
3602	310620005650444	URBANO	310620005199	Caiþara - Adelaide	31062000565
3603	310620005650445	URBANO	310620005227	Monsenhor Messias	31062000565
3604	310620005650446	URBANO	310620005199	Caiþara - Adelaide	31062000565
3605	310620005650447	URBANO	310620005232	Padre Eustßquio	31062000565
3606	310620005650448	URBANO	310620005225	Marmiteiros	31062000565
3607	310620005650449	URBANO	310620005232	Padre Eustßquio	31062000565
3608	310620005650450	URBANO	310620005210	CoraþÒo EucarÝstic	31062000565
3609	310620005650451	URBANO	310620005221	JoÒo Pinheiro	31062000565
3610	310620005650452	URBANO	310620005221	JoÒo Pinheiro	31062000565
3611	310620005650453	URBANO	310620005211	Delta	31062000565
3612	310620005650454	URBANO	310620005221	JoÒo Pinheiro	31062000565
3613	310620005650455	URBANO	310620005204	Conjunto Calif¾rnia	31062000565
3614	310620005650456	URBANO	310620005212	Dom Bosco	31062000565
3615	310620005650457	URBANO	310620005212	Dom Bosco	31062000565
3616	310620005650458	URBANO	310620005216	Gl¾ria	31062000565
3617	310620005650459	URBANO	310620005230	Novo Gl¾ria	31062000565
3618	310620005650460	URBANO	310620005194	┴lvaro Camargos	31062000565
3619	310620005650461	URBANO	310620005230	Novo Gl¾ria	31062000565
3620	310620005650462	URBANO	310620005216	Gl¾ria	31062000565
3621	310620005650463	URBANO	310620005216	Gl¾ria	31062000565
3622	310620005650464	URBANO	310620005216	Gl¾ria	31062000565
3623	310620005650465	URBANO	310620005220	Jardim SÒo JosÚ	31062000565
3624	310620005650466	URBANO	310620005199	Caiþara - Adelaide	31062000565
3625	310620005650467	URBANO	310620005192	Alto Caiþaras	31062000565
3626	310620005650468	URBANO	310620005192	Alto Caiþaras	31062000565
3627	310620005650469	URBANO	310620005196	Aparecida 7¬ SeþÒc	31062000565
3628	310620005650470	URBANO	310620005229	Nova Esperanþa	31062000565
3629	310620005650471	URBANO	310620005195	Aparecida	31062000565
3630	310620005650472	URBANO	310620005236	Santo AndrÚ	31062000565
3631	310620005650473	URBANO	310620005197	Bom Jesus	31062000565
3632	310620005650474	URBANO	310620005236	Santo AndrÚ	31062000565

3633	310620005650475	URBANO	310620005236	Santo AndrÚ	31062000565
3634	310620005650476	URBANO	310620005198	Bonfim	31062000565
3635	310620005650477	URBANO	310620005198	Bonfim	31062000565
3636	310620005650478	URBANO	310620005236	Santo AndrÚ	31062000565
3637	310620005650479	URBANO	310620005203	Carlos Prates	31062000565
3638	310620005650480	URBANO	310620005232	Padre Eustßquio	31062000565
3639	310620005650481	URBANO	310620005210	CoraþÒo EucarÝstic	31062000565
3640	310620005650482	URBANO	310620005232	Padre Eustßquio	31062000565
3641	310620005650483	URBANO	310620005246	Vila das Oliveiras	31062000565
3642	310620005650484	URBANO	310620005221	JoÒo Pinheiro	31062000565
3643	310620005650485	URBANO	310620005252	Vila Oeste	31062000565
3644	310620005650486	URBANO	310620005231	Oeste	31062000565
3645	310620005650487	URBANO	310620005221	JoÒo Pinheiro	31062000565
3646	310620005650488	URBANO	310620005221	JoÒo Pinheiro	31062000565
3647	310620005650489	URBANO	310620005193	Alto dos Pinheiros	31062000565
3648	310620005650490	URBANO	310620005231	Oeste	31062000565
3649	310620005650491	URBANO	310620005231	Oeste	31062000565
3650	310620005650492	URBANO	310620005231	Oeste	31062000565
3651	310620005650493	URBANO	310620005252	Vila Oeste	31062000565
3652	310620005650494	URBANO	310620005252	Vila Oeste	31062000565
3653	310620005650495	URBANO	310620005159	Camargos	31062000565
3654	310620005650496	URBANO	310620005159	Camargos	31062000565
3655	310620005650497	URBANO	310620005159	Camargos	31062000565
3656	310620005650498	URBANO	310620005159	Camargos	31062000565
3657	310620005650499	URBANO	310620005159	Camargos	31062000565
3658	310620005650500	URBANO	310620005159	Camargos	31062000565
3659	310620005650501	URBANO	310620005224	Maravilha	31062000565
3660	310620005650502	URBANO	310620005235	Santa Maria	31062000565
3661	310620005650503	URBANO	310620005159	Camargos	31062000565
3662	310620005650504	URBANO	310620005204	Conjunto Calif¾rnia	31062000565
3663	310620005650505	URBANO	310620005208	Conjunto Novo Don	31062000565
3664	310620005650506	URBANO	310620005217	InconfidÛncia	31062000565
3665	310620005650507	URBANO	310620005221	JoÒo Pinheiro	31062000565
3666	310620005650508	URBANO	310620005208	Conjunto Novo Don	31062000565
3667	310620005650509	URBANO	310620005201	Calif¾rnia	31062000565
3668	310620005650510	URBANO	310620005201	Calif¾rnia	31062000565
3669	310620005650511	URBANO	310620005234	Pindorama	31062000565
3670	310620005650512	URBANO	310620005234	Pindorama	31062000565
3671	310620005650513	URBANO	310620005234	Pindorama	31062000565
3672	310620005650514	URBANO	310620005234	Pindorama	31062000565
3673	310620005650515	URBANO	310620005234	Pindorama	31062000565
3674	310620005650516	URBANO	310620005245	Vila Coqueiral	31062000565
3675	310620005650517	URBANO	310620005234	Pindorama	31062000565
3676	310620005650518	URBANO	310620005245	Vila Coqueiral	31062000565
3677	310620005650519	URBANO	310620005230	Novo Gl¾ria	31062000565
3678	310620005650520	URBANO	310620005230	Novo Gl¾ria	31062000565
3679	310620005650521	URBANO	310620005216	Gl¾ria	31062000565
3680	310620005650522	URBANO	310620005209	Coqueiros	31062000565
3681	310620005650523	URBANO	310620005239	SÒo Salvador	31062000565
3682	310620005650524	URBANO	310620005230	Novo Gl¾ria	31062000565

3683	310620005650525	URBANO	310620005241	Serrano	31062000565
3684	310620005650526	URBANO	310620005191	AlÝpio de Melo	31062000565
3685	310620005650527	URBANO	310620005241	Serrano	31062000565
3686	310620005650528	URBANO	310620005206	Conjunto Celso Mac	31062000565
3687	310620005650529	URBANO	310620005241	Serrano	31062000565
3688	310620005650530	URBANO	310620005216	Gl¾ria	31062000565
3689	310620005650531	URBANO	310620005217	InconfidÛncia	31062000565
3690	310620005650532	URBANO	310620005217	InconfidÛncia	31062000565
3691	310620005650533	URBANO	310620005220	Jardim SÒo JosÚ	31062000565
3692	310620005650534	URBANO	310620005218	Jardim Alvorada	31062000565
3693	310620005650535	URBANO	310620005247	Vila Jardim Alvorada	31062000565
3694	310620005650536	URBANO	310620005218	Jardim Alvorada	31062000565
3695	310620005650537	URBANO	310620005218	Jardim Alvorada	31062000565
3696	310620005650538	URBANO	310620005218	Jardim Alvorada	31062000565
3697	310620005650539	URBANO	310620005247	Vila Jardim Alvorada	31062000565
3698	310620005650540	URBANO	310620005218	Jardim Alvorada	31062000565
3699	310620005650541	URBANO	310620005218	Jardim Alvorada	31062000565
3700	310620005650542	URBANO	310620005218	Jardim Alvorada	31062000565
3701	310620005650543	URBANO	310620005247	Vila Jardim Alvorada	31062000565
3702	310620005650544	URBANO	310620005218	Jardim Alvorada	31062000565
3703	310620005650545	URBANO	310620005218	Jardim Alvorada	31062000565
3704	310620005650546	URBANO	310620005248	Vila Jardim Montan	31062000565
3705	310620005650547	URBANO	310620005214	Engenho Nogueira	31062000565
3706	310620005650548	URBANO	310620005192	Alto Caiþaras	31062000565
3707	310620005650557	URBANO	310620005197	Bom Jesus	31062000565
3708	310620005650558	URBANO	310620005195	Aparecida	31062000565
3709	310620005650559	URBANO	310620005236	Santo AndrÚ	31062000565
3710	310620005650560	URBANO	310620005195	Aparecida	31062000565
3711	310620005650561	URBANO	310620005236	Santo AndrÚ	31062000565
3712	310620005650562	URBANO	310620005055	SÒo Crist¾vÒo	31062000565
3713	310620005650563	URBANO	310620005240	Senhor dos Passos	31062000565
3714	310620005650564	URBANO	310620005236	Santo AndrÚ	31062000565
3715	310620005650565	URBANO	310620005242	SumarÚ	31062000565
3716	310620005650566	URBANO	310620005236	Santo AndrÚ	31062000565
3717	310620005650567	URBANO	310620005203	Carlos Prates	31062000565
3718	310620005650568	URBANO	310620005203	Carlos Prates	31062000565
3719	310620005650569	URBANO	310620005203	Carlos Prates	31062000565
3720	310620005650570	URBANO	310620005232	Padre Eustßquio	31062000565
3721	310620005650571	URBANO	310620005203	Carlos Prates	31062000565
3722	310620005650572	URBANO	310620005232	Padre Eustßquio	31062000565
3723	310620005650573	URBANO	310620005232	Padre Eustßquio	31062000565
3724	310620005650574	URBANO	310620005232	Padre Eustßquio	31062000565
3725	310620005650575	URBANO	310620005232	Padre Eustßquio	31062000565
3726	310620005650576	URBANO	310620005248	Vila Jardim Montan	31062000565
3727	310620005650577	URBANO	310620005218	Jardim Alvorada	31062000565
3728	310620005650578	URBANO	310620005241	Serrano	31062000565
3729	310620005670001	URBANO	310620005139	Cinquentenßrio	31062000567
3730	310620005670002	URBANO	310620005139	Cinquentenßrio	31062000567
3731	310620005670003	URBANO	310620005140	Maraj¾	31062000567
3732	310620005670004	URBANO	310620005139	Cinquentenßrio	31062000567

3733	310620005670005	URBANO	310620005141	Estrela do Oriente	31062000567
3734	310620005670006	URBANO	310620005142	BetÔnia	31062000567
3735	310620005670007	URBANO	310620005139	Cinquentenßrio	31062000567
3736	310620005670008	URBANO	310620005142	BetÔnia	31062000567
3737	310620005670009	URBANO	310620005144	Nova Cintra	31062000567
3738	310620005670010	URBANO	310620005144	Nova Cintra	31062000567
3739	310620005670011	URBANO	310620005142	BetÔnia	31062000567
3740	310620005670012	URBANO	310620005136	Salgado Filho	31062000567
3741	310620005670013	URBANO	310620005136	Salgado Filho	31062000567
3742	310620005670014	URBANO	310620005136	Salgado Filho	31062000567
3743	310620005670015	URBANO	310620005173	HavaÝ	31062000567
3744	310620005670016	URBANO	310620005173	HavaÝ	31062000567
3745	310620005670017	URBANO	310620005173	HavaÝ	31062000567
3746	310620005670018	URBANO	310620005173	HavaÝ	31062000567
3747	310620005670019	URBANO	310620005173	HavaÝ	31062000567
3748	310620005670020	URBANO	310620005173	HavaÝ	31062000567
3749	310620005670021	URBANO	310620005173	HavaÝ	31062000567
3750	310620005670022	URBANO	310620005172	Parque SÒo JosÚ	31062000567
3751	310620005670023	URBANO	310620005140	Maraj¾	31062000567
3752	310620005670024	URBANO	310620005139	Cinquentenßrio	31062000567
3753	310620005670025	URBANO	310620005141	Estrela do Oriente	31062000567
3754	310620005670026	URBANO	310620005141	Estrela do Oriente	31062000567
3755	310620005670027	URBANO	310620005142	BetÔnia	31062000567
3756	310620005670028	URBANO	310620005142	BetÔnia	31062000567
3757	310620005670029	URBANO	310620005142	BetÔnia	31062000567
3758	310620005670030	URBANO	310620005143	Vila Vista Alegre	31062000567
3759	310620005670031	URBANO	310620005143	Vila Vista Alegre	31062000567
3760	310620005670032	URBANO	310620005143	Vila Vista Alegre	31062000567
3761	310620005670033	URBANO	310620005143	Vila Vista Alegre	31062000567
3762	310620005670034	URBANO	310620005144	Nova Cintra	31062000567
3763	310620005670035	URBANO	310620005144	Nova Cintra	31062000567
3764	310620005670036	URBANO	310620005146	Nova Gameleira	31062000567
3765	310620005670037	URBANO	310620005136	Salgado Filho	31062000567
3766	310620005670038	URBANO	310620005136	Salgado Filho	31062000567
3767	310620005670039	URBANO	310620005136	Salgado Filho	31062000567
3768	310620005670040	URBANO	310620005136	Salgado Filho	31062000567
3769	310620005670041	URBANO	310620005136	Salgado Filho	31062000567
3770	310620005670042	URBANO	310620005136	Salgado Filho	31062000567
3771	310620005670043	URBANO	310620005170	Ventosa	31062000567
3772	310620005670044	URBANO	310620005170	Ventosa	31062000567
3773	310620005670045	URBANO	310620005170	Ventosa	31062000567
3774	310620005670046	URBANO	310620005170	Ventosa	31062000567
3775	310620005670047	URBANO	310620005170	Ventosa	31062000567
3776	310620005670048	URBANO	310620005170	Ventosa	31062000567
3777	310620005670049	URBANO	310620005170	Ventosa	31062000567
3778	310620005670050	URBANO	310620005170	Ventosa	31062000567
3779	310620005670051	URBANO	310620005134	Jardim AmÚrica	31062000567
3780	310620005670052	URBANO	310620005134	Jardim AmÚrica	31062000567
3781	310620005670053	URBANO	310620005184	Estoril	31062000567
3782	310620005670054	URBANO	310620005184	Estoril	31062000567

3783	310620005670055	URBANO	310620005184	Estoril	31062000567
3784	310620005670056	URBANO	310620005184	Estoril	31062000567
3785	310620005670057	URBANO	310620005173	HavaÝ	31062000567
3786	310620005670058	URBANO	310620005173	HavaÝ	31062000567
3787	310620005670059	URBANO	310620005173	HavaÝ	31062000567
3788	310620005670060	URBANO	310620005173	HavaÝ	31062000567
3789	310620005670061	URBANO	310620005173	HavaÝ	31062000567
3790	310620005670062	URBANO	310620005172	Parque SÒo JosÚ	31062000567
3791	310620005670063	URBANO	310620005141	Estrela do Oriente	31062000567
3792	310620005670064	URBANO	310620005141	Estrela do Oriente	31062000567
3793	310620005670065	URBANO	310620005142	BetÔnia	31062000567
3794	310620005670066	URBANO	310620005142	BetÔnia	31062000567
3795	310620005670067	URBANO	310620005142	BetÔnia	31062000567
3796	310620005670068	URBANO	310620005142	BetÔnia	31062000567
3797	310620005670069	URBANO	310620005143	Vila Vista Alegre	31062000567
3798	310620005670070	URBANO	310620005143	Vila Vista Alegre	31062000567
3799	310620005670071	URBANO	310620005143	Vila Vista Alegre	31062000567
3800	310620005670072	URBANO	310620005143	Vila Vista Alegre	31062000567
3801	310620005670073	URBANO	310620005143	Vila Vista Alegre	31062000567
3802	310620005670074	URBANO	310620005143	Vila Vista Alegre	31062000567
3803	310620005670075	URBANO	310620005151	Vista Alegre	31062000567
3804	310620005670076	URBANO	310620005144	Nova Cintra	31062000567
3805	310620005670077	URBANO	310620005145	Emba·bas	31062000567
3806	310620005670078	URBANO	310620005146	Nova Gameleira	31062000567
3807	310620005670079	URBANO	310620005146	Nova Gameleira	31062000567
3808	310620005670080	URBANO	310620005136	Salgado Filho	31062000567
3809	310620005670081	URBANO	310620005136	Salgado Filho	31062000567
3810	310620005670082	URBANO	310620005136	Salgado Filho	31062000567
3811	310620005670083	URBANO	310620005136	Salgado Filho	31062000567
3812	310620005670084	URBANO	310620005136	Salgado Filho	31062000567
3813	310620005670085	URBANO	310620005136	Salgado Filho	31062000567
3814	310620005670086	URBANO	310620005134	Jardim AmÚrica	31062000567
3815	310620005670087	URBANO	310620005134	Jardim AmÚrica	31062000567
3816	310620005670088	URBANO	310620005134	Jardim AmÚrica	31062000567
3817	310620005670089	URBANO	310620005134	Jardim AmÚrica	31062000567
3818	310620005670090	URBANO	310620005134	Jardim AmÚrica	31062000567
3819	310620005670091	URBANO	310620005134	Jardim AmÚrica	31062000567
3820	310620005670092	URBANO	310620005134	Jardim AmÚrica	31062000567
3821	310620005670093	URBANO	310620005184	Estoril	31062000567
3822	310620005670094	URBANO	310620005184	Estoril	31062000567
3823	310620005670095	URBANO	310620005173	HavaÝ	31062000567
3824	310620005670096	URBANO	310620005186	Buritis	31062000567
3825	310620005670097	URBANO	310620005186	Buritis	31062000567
3826	310620005670098	URBANO	310620005186	Buritis	31062000567
3827	310620005670099	URBANO	310620005186	Buritis	31062000567
3828	310620005670100	URBANO	310620005186	Buritis	31062000567
3829	310620005670101	URBANO	310620005186	Buritis	31062000567
3830	310620005670102	URBANO	310620005186	Buritis	31062000567
3831	310620005670103	URBANO	310620005186	Buritis	31062000567
3832	310620005670104	URBANO	310620005186	Buritis	31062000567

3833	310620005670105	URBANO	310620005186	Buritis	31062000567
3834	310620005670106	URBANO	310620005186	Buritis	31062000567
3835	310620005670107	URBANO	310620005185	Palmeiras	31062000567
3836	310620005670108	URBANO	310620005185	Palmeiras	31062000567
3837	310620005670109	URBANO	310620005185	Palmeiras	31062000567
3838	310620005670110	URBANO	310620005185	Palmeiras	31062000567
3839	310620005670111	URBANO	310620005185	Palmeiras	31062000567
3840	310620005670112	URBANO	310620005185	Palmeiras	31062000567
3841	310620005670113	URBANO	310620005142	BetÔnia	31062000567
3842	310620005670114	URBANO	310620005152	Bairro das Ind·strias	31062000567
3843	310620005670115	URBANO	310620005151	Vista Alegre	31062000567
3844	310620005670116	URBANO	310620005143	Vila Vista Alegre	31062000567
3845	310620005670117	URBANO	310620005151	Vista Alegre	31062000567
3846	310620005670118	URBANO	310620005150	Cabana do Pai Tom	31062000567
3847	310620005670119	URBANO	310620005150	Cabana do Pai Tom	31062000567
3848	310620005670120	URBANO	310620005150	Cabana do Pai Tom	31062000567
3849	310620005670121	URBANO	310620005150	Cabana do Pai Tom	31062000567
3850	310620005670122	URBANO	310620005150	Cabana do Pai Tom	31062000567
3851	310620005670123	URBANO	310620005151	Vista Alegre	31062000567
3852	310620005670124	URBANO	310620005150	Cabana do Pai Tom	31062000567
3853	310620005670125	URBANO	310620005150	Cabana do Pai Tom	31062000567
3854	310620005670126	URBANO	310620005150	Cabana do Pai Tom	31062000567
3855	310620005670127	URBANO	310620005150	Cabana do Pai Tom	31062000567
3856	310620005670128	URBANO	310620005150	Cabana do Pai Tom	31062000567
3857	310620005670129	URBANO	310620005150	Cabana do Pai Tom	31062000567
3858	310620005670130	URBANO	310620005150	Cabana do Pai Tom	31062000567
3859	310620005670131	URBANO	310620005150	Cabana do Pai Tom	31062000567
3860	310620005670132	URBANO	310620005150	Cabana do Pai Tom	31062000567
3861	310620005670133	URBANO	310620005150	Cabana do Pai Tom	31062000567
3862	310620005670134	URBANO	310620005150	Cabana do Pai Tom	31062000567
3863	310620005670135	URBANO	310620005150	Cabana do Pai Tom	31062000567
3864	310620005670136	URBANO	310620005150	Cabana do Pai Tom	31062000567
3865	310620005670137	URBANO	310620005150	Cabana do Pai Tom	31062000567
3866	310620005670138	URBANO	310620005150	Cabana do Pai Tom	31062000567
3867	310620005670139	URBANO	310620005150	Cabana do Pai Tom	31062000567
3868	310620005670140	URBANO	310620005150	Cabana do Pai Tom	31062000567
3869	310620005670141	URBANO	310620005150	Cabana do Pai Tom	31062000567
3870	310620005670142	URBANO	310620005150	Cabana do Pai Tom	31062000567
3871	310620005670143	URBANO	310620005150	Cabana do Pai Tom	31062000567
3872	310620005670144	URBANO	310620005146	Nova Gameleira	31062000567
3873	310620005670145	URBANO	310620005146	Nova Gameleira	31062000567
3874	310620005670146	URBANO	310620005146	Nova Gameleira	31062000567
3875	310620005670147	URBANO	310620005146	Nova Gameleira	31062000567
3876	310620005670148	URBANO	310620005146	Nova Gameleira	31062000567
3877	310620005670149	URBANO	310620005166	Gameleira	31062000567
3878	310620005670150	URBANO	310620005146	Nova Gameleira	31062000567
3879	310620005670151	URBANO	310620005146	Nova Gameleira	31062000567
3880	310620005670152	URBANO	310620005146	Nova Gameleira	31062000567
3881	310620005670153	URBANO	310620005138	GuaratÒ	31062000567
3882	310620005670154	URBANO	310620005135	Nova SuÝþa	31062000567

3883	310620005670155	URBANO	310620005135	Nova SuÝþa	31062000567
3884	310620005670156	URBANO	310620005163	Ambrosina	31062000567
3885	310620005670157	URBANO	310620005136	Salgado Filho	31062000567
3886	310620005670158	URBANO	310620005136	Salgado Filho	31062000567
3887	310620005670159	URBANO	310620005136	Salgado Filho	31062000567
3888	310620005670160	URBANO	310620005135	Nova SuÝþa	31062000567
3889	310620005670161	URBANO	310620005134	Jardim AmÚrica	31062000567
3890	310620005670162	URBANO	310620005134	Jardim AmÚrica	31062000567
3891	310620005670163	URBANO	310620005134	Jardim AmÚrica	31062000567
3892	310620005670164	URBANO	310620005134	Jardim AmÚrica	31062000567
3893	310620005670165	URBANO	310620005167	BarÒo Homem de M	31062000567
3894	310620005670166	URBANO	310620005167	BarÒo Homem de M	31062000567
3895	310620005670167	URBANO	310620005179	Santa Sofia	31062000567
3896	310620005670168	URBANO	310620005179	Santa Sofia	31062000567
3897	310620005670169	URBANO	310620005179	Santa Sofia	31062000567
3898	310620005670170	URBANO	310620005180	Leonina	31062000567
3899	310620005670171	URBANO	310620005182	Alpes	31062000567
3900	310620005670172	URBANO	310620005182	Alpes	31062000567
3901	310620005670173	URBANO	310620005182	Alpes	31062000567
3902	310620005670174	URBANO	310620005187	Olhos D'┴gua	31062000567
3903	310620005670175	URBANO	310620005186	Buritis	31062000567
3904	310620005670176	URBANO	310620005186	Buritis	31062000567
3905	310620005670177	URBANO	310620005186	Buritis	31062000567
3906	310620005670178	URBANO	310620005186	Buritis	31062000567
3907	310620005670179	URBANO	310620005186	Buritis	31062000567
3908	310620005670180	URBANO	310620005186	Buritis	31062000567
3909	310620005670181	URBANO	310620005186	Buritis	31062000567
3910	310620005670182	URBANO	310620005186	Buritis	31062000567
3911	310620005670183	URBANO	310620005186	Buritis	31062000567
3912	310620005670184	URBANO	310620005185	Palmeiras	31062000567
3913	310620005670185	URBANO	310620005186	Buritis	31062000567
3914	310620005670186	URBANO	310620005151	Vista Alegre	31062000567
3915	310620005670187	URBANO	310620005154	Madre Gertrudes	31062000567
3916	310620005670188	URBANO	310620005151	Vista Alegre	31062000567
3917	310620005670189	URBANO	310620005155	Vila Madre Gertrud	31062000567
3918	310620005670190	URBANO	310620005154	Madre Gertrudes	31062000567
3919	310620005670191	URBANO	310620005154	Madre Gertrudes	31062000567
3920	310620005670192	URBANO	310620005154	Madre Gertrudes	31062000567
3921	310620005670193	URBANO	310620005150	Cabana do Pai Tom	31062000567
3922	310620005670194	URBANO	310620005150	Cabana do Pai Tom	31062000567
3923	310620005670195	URBANO	310620005150	Cabana do Pai Tom	31062000567
3924	310620005670196	URBANO	310620005149	Jardin¾polis	31062000567
3925	310620005670197	URBANO	310620005149	Jardin¾polis	31062000567
3926	310620005670198	URBANO	310620005146	Nova Gameleira	31062000567
3927	310620005670199	URBANO	310620005147	Vila Nova Gameleira	31062000567
3928	310620005670200	URBANO	310620005149	Jardin¾polis	31062000567
3929	310620005670201	URBANO	310620005128	Calafate	31062000567
3930	310620005670202	URBANO	310620005128	Calafate	31062000567
3931	310620005670203	URBANO	310620005135	Nova SuÝþa	31062000567
3932	310620005670204	URBANO	310620005135	Nova SuÝþa	31062000567

3933	310620005670205	URBANO	310620005135	Nova SuÝþa	31062000567
3934	310620005670206	URBANO	310620005135	Nova SuÝþa	31062000567
3935	310620005670207	URBANO	310620005135	Nova SuÝþa	31062000567
3936	310620005670208	URBANO	310620005135	Nova SuÝþa	31062000567
3937	310620005670209	URBANO	310620005135	Nova SuÝþa	31062000567
3938	310620005670210	URBANO	310620005134	Jardim AmÚrica	31062000567
3939	310620005670211	URBANO	310620005132	Nova Granada	31062000567
3940	310620005670212	URBANO	310620005132	Nova Granada	31062000567
3941	310620005670213	URBANO	310620005132	Nova Granada	31062000567
3942	310620005670214	URBANO	310620005177	SÒo Jorge 3¬ SeþÒc	31062000567
3943	310620005670215	URBANO	310620005179	Santa Sofia	31062000567
3944	310620005670216	URBANO	310620005180	Leonina	31062000567
3945	310620005670217	URBANO	310620005154	Madre Gertrudes	31062000567
3946	310620005670218	URBANO	310620005154	Madre Gertrudes	31062000567
3947	310620005670219	URBANO	310620005154	Madre Gertrudes	31062000567
3948	310620005670220	URBANO	310620005161	Sport Club	31062000567
3949	310620005670221	URBANO	310620005161	Sport Club	31062000567
3950	310620005670222	URBANO	310620005160	VirgÝnia	31062000567
3951	310620005670223	URBANO	310620005128	Calafate	31062000567
3952	310620005670224	URBANO	310620005128	Calafate	31062000567
3953	310620005670225	URBANO	310620005128	Calafate	31062000567
3954	310620005670226	URBANO	310620005135	Nova SuÝþa	31062000567
3955	310620005670227	URBANO	310620005135	Nova SuÝþa	31062000567
3956	310620005670228	URBANO	310620005135	Nova SuÝþa	31062000567
3957	310620005670229	URBANO	310620005135	Nova SuÝþa	31062000567
3958	310620005670230	URBANO	310620005135	Nova SuÝþa	31062000567
3959	310620005670231	URBANO	310620005135	Nova SuÝþa	31062000567
3960	310620005670232	URBANO	310620005132	Nova Granada	31062000567
3961	310620005670233	URBANO	310620005132	Nova Granada	31062000567
3962	310620005670234	URBANO	310620005132	Nova Granada	31062000567
3963	310620005670235	URBANO	310620005132	Nova Granada	31062000567
3964	310620005670236	URBANO	310620005132	Nova Granada	31062000567
3965	310620005670237	URBANO	310620005132	Nova Granada	31062000567
3966	310620005670238	URBANO	310620005175	SÒo Jorge 1¬ SeþÒc	31062000567
3967	310620005670239	URBANO	310620005175	SÒo Jorge 1¬ SeþÒc	31062000567
3968	310620005670240	URBANO	310620005175	SÒo Jorge 1¬ SeþÒc	31062000567
3969	310620005670241	URBANO	310620005177	SÒo Jorge 3¬ SeþÒc	31062000567
3970	310620005670242	URBANO	310620005178	Vila Antena	31062000567
3971	310620005670243	URBANO	310620005128	Calafate	31062000567
3972	310620005670244	URBANO	310620005128	Calafate	31062000567
3973	310620005670245	URBANO	310620005128	Calafate	31062000567
3974	310620005670246	URBANO	310620005126	Prado	31062000567
3975	310620005670247	URBANO	310620005126	Prado	31062000567
3976	310620005670248	URBANO	310620005126	Prado	31062000567
3977	310620005670249	URBANO	310620005130	Alto Barroca	31062000567
3978	310620005670250	URBANO	310620005130	Alto Barroca	31062000567
3979	310620005670251	URBANO	310620005130	Alto Barroca	31062000567
3980	310620005670252	URBANO	310620005130	Alto Barroca	31062000567
3981	310620005670253	URBANO	310620005130	Alto Barroca	31062000567
3982	310620005670254	URBANO	310620005130	Alto Barroca	31062000567

3983	310620005670255	URBANO	310620005131	Graja·	31062000567
3984	310620005670256	URBANO	310620005131	Graja·	31062000567
3985	310620005670257	URBANO	310620005131	Graja·	31062000567
3986	310620005670258	URBANO	310620005131	Graja·	31062000567
3987	310620005670259	URBANO	310620005131	Graja·	31062000567
3988	310620005670260	URBANO	310620005176	SÒo Jorge 2¬ SeþÒc	31062000567
3989	310620005670261	URBANO	310620005176	SÒo Jorge 2¬ SeþÒc	31062000567
3990	310620005670262	URBANO	310620005177	SÒo Jorge 3¬ SeþÒc	31062000567
3991	310620005670263	URBANO	310620005177	SÒo Jorge 3¬ SeþÒc	31062000567
3992	310620005670264	URBANO	310620005177	SÒo Jorge 3¬ SeþÒc	31062000567
3993	310620005670265	URBANO	310620005178	Vila Antena	31062000567
3994	310620005670266	URBANO	310620005178	Vila Antena	31062000567
3995	310620005670267	URBANO	310620005128	Calafate	31062000567
3996	310620005670268	URBANO	310620005128	Calafate	31062000567
3997	310620005670269	URBANO	310620005128	Calafate	31062000567
3998	310620005670270	URBANO	310620005128	Calafate	31062000567
3999	310620005670271	URBANO	310620005126	Prado	31062000567
4000	310620005670272	URBANO	310620005129	Barroca	31062000567
4001	310620005670273	URBANO	310620005130	Alto Barroca	31062000567
4002	310620005670274	URBANO	310620005131	Graja·	31062000567
4003	310620005670275	URBANO	310620005137	Gutierrez	31062000567
4004	310620005670276	URBANO	310620005126	Prado	31062000567
4005	310620005670277	URBANO	310620005126	Prado	31062000567
4006	310620005670278	URBANO	310620005126	Prado	31062000567
4007	310620005670279	URBANO	310620005126	Prado	31062000567
4008	310620005670280	URBANO	310620005126	Prado	31062000567
4009	310620005670281	URBANO	310620005126	Prado	31062000567
4010	310620005670282	URBANO	310620005129	Barroca	31062000567
4011	310620005670283	URBANO	310620005129	Barroca	31062000567
4012	310620005670284	URBANO	310620005137	Gutierrez	31062000567
4013	310620005670285	URBANO	310620005137	Gutierrez	31062000567
4014	310620005670286	URBANO	310620005137	Gutierrez	31062000567
4015	310620005670287	URBANO	310620005137	Gutierrez	31062000567
4016	310620005670288	URBANO	310620005137	Gutierrez	31062000567
4017	310620005670289	URBANO	310620005137	Gutierrez	31062000567
4018	310620005670290	URBANO	310620005176	SÒo Jorge 2¬ SeþÒc	31062000567
4019	310620005670291	URBANO	310620005137	Gutierrez	31062000567
4020	310620005670292	URBANO	310620005137	Gutierrez	31062000567
4021	310620005670293	URBANO	310620005176	SÒo Jorge 2¬ SeþÒc	31062000567
4022	310620005670294	URBANO	310620005176	SÒo Jorge 2¬ SeþÒc	31062000567
4023	310620005670295	URBANO	310620005126	Prado	31062000567
4024	310620005670296	URBANO	310620005126	Prado	31062000567
4025	310620005670297	URBANO	310620005126	Prado	31062000567
4026	310620005670298	URBANO	310620005126	Prado	31062000567
4027	310620005670299	URBANO	310620005126	Prado	31062000567
4028	310620005670300	URBANO	310620005126	Prado	31062000567
4029	310620005670301	URBANO	310620005126	Prado	31062000567
4030	310620005670302	URBANO	310620005126	Prado	31062000567
4031	310620005670303	URBANO	310620005137	Gutierrez	31062000567
4032	310620005670304	URBANO	310620005137	Gutierrez	31062000567

4033	310620005670305	URBANO	310620005137	Gutierrez	31062000567
4034	310620005670306	URBANO	310620005129	Barroca	31062000567
4035	310620005670307	URBANO	310620005137	Gutierrez	31062000567
4036	310620005670308	URBANO	310620005137	Gutierrez	31062000567
4037	310620005670309	URBANO	310620005137	Gutierrez	31062000567
4038	310620005670310	URBANO	310620005137	Gutierrez	31062000567
4039	310620005670311	URBANO	310620005137	Gutierrez	31062000567
4040	310620005670312	URBANO	310620005137	Gutierrez	31062000567
4041	310620005670313	URBANO	310620005137	Gutierrez	31062000567
4042	310620005670314	URBANO	310620005137	Gutierrez	31062000567
4043	310620005670315	URBANO	310620005137	Gutierrez	31062000567
4044	310620005670316	URBANO	310620005137	Gutierrez	31062000567
4045	310620005670317	URBANO	310620005139	Cinquentenßrio	31062000567
4046	310620005670318	URBANO	310620005173	HavaÝ	31062000567
4047	310620005670319	URBANO	310620005142	BetÔnia	31062000567
4048	310620005670320	URBANO	310620005141	Estrela do Oriente	31062000567
4049	310620005670321	URBANO	310620005139	Cinquentenßrio	31062000567
4050	310620005670322	URBANO	310620005143	Vila Vista Alegre	31062000567
4051	310620005670323	URBANO	310620005144	Nova Cintra	31062000567
4052	310620005670324	URBANO	310620005173	HavaÝ	31062000567
4053	310620005670325	URBANO	310620005170	Ventosa	31062000567
4054	310620005670326	URBANO	310620005173	HavaÝ	31062000567
4055	310620005670327	URBANO	310620005140	Maraj¾	31062000567
4056	310620005670328	URBANO	310620005170	Ventosa	31062000567
4057	310620005670329	URBANO	310620005173	HavaÝ	31062000567
4058	310620005670330	URBANO	310620005170	Ventosa	31062000567
4059	310620005670331	URBANO	310620005184	Estoril	31062000567
4060	310620005670332	URBANO	310620005173	HavaÝ	31062000567
4061	310620005670333	URBANO	310620005174	Vila HavaÝ	31062000567
4062	310620005670334	URBANO	310620005173	HavaÝ	31062000567
4063	310620005670335	URBANO	310620005173	HavaÝ	31062000567
4064	310620005670336	URBANO	310620005140	Maraj¾	31062000567
4065	310620005670337	URBANO	310620005185	Palmeiras	31062000567
4066	310620005670338	URBANO	310620005172	Parque SÒo JosÚ	31062000567
4067	310620005670339	URBANO	310620005139	Cinquentenßrio	31062000567
4068	310620005670340	URBANO	310620005144	Nova Cintra	31062000567
4069	310620005670341	URBANO	310620005146	Nova Gameleira	31062000567
4070	310620005670342	URBANO	310620005144	Nova Cintra	31062000567
4071	310620005670343	URBANO	310620005136	Salgado Filho	31062000567
4072	310620005670344	URBANO	310620005170	Ventosa	31062000567
4073	310620005670345	URBANO	310620005173	HavaÝ	31062000567
4074	310620005670346	URBANO	310620005173	HavaÝ	31062000567
4075	310620005670347	URBANO	310620005170	Ventosa	31062000567
4076	310620005670348	URBANO	310620005184	Estoril	31062000567
4077	310620005670349	URBANO	310620005173	HavaÝ	31062000567
4078	310620005670350	URBANO	310620005172	Parque SÒo JosÚ	31062000567
4079	310620005670351	URBANO	310620005185	Palmeiras	31062000567
4080	310620005670352	URBANO	310620005141	Estrela do Oriente	31062000567
4081	310620005670353	URBANO	310620005142	BetÔnia	31062000567
4082	310620005670354	URBANO	310620005142	BetÔnia	31062000567

4083	310620005670480	URBANO	310620005131	Graja·	31062000567
4084	310620005670481	URBANO	310620005137	Gutierrez	31062000567
4085	310620005670482	URBANO	310620005137	Gutierrez	31062000567
4086	310620005670483	URBANO	310620005126	Prado	31062000567
4087	310620005670484	URBANO	310620005129	Barroca	31062000567
4088	310620005670485	URBANO	310620005131	Graja·	31062000567
4089	310620005670486	URBANO	310620005131	Graja·	31062000567
4090	310620005670487	URBANO	310620005176	SÒo Jorge 2¬ SeþÒc	31062000567
4091	310620005670488	URBANO	310620005176	SÒo Jorge 2¬ SeþÒc	31062000567
4092	310620005670489	URBANO	310620005129	Barroca	31062000567
4093	310620005670490	URBANO	310620005161	Sport Club	31062000567
4094	310620005670491	URBANO	310620005159	Camargos	31062000567
4095	310620005670492	URBANO	310620005149	Jardin¾polis	31062000567
4096	310620005670493	URBANO	310620005165	Vila Nova Gameleira	31062000567
4097	310620005670494	URBANO	310620005139	Cinquentenßrio	31062000567
4098	310620005680001	URBANO	310620005283	SÒo Luiz	31062000568
4099	310620005680002	URBANO	310620005282	SÒo JosÚ	31062000568
4100	310620005670363	URBANO	310620005163	Ambrosina	31062000567
4101	310620005670364	URBANO	310620005136	Salgado Filho	31062000567
4102	310620005670365	URBANO	310620005136	Salgado Filho	31062000567
4103	310620005670366	URBANO	310620005134	Jardim AmÚrica	31062000567
4104	310620005670367	URBANO	310620005170	Ventosa	31062000567
4105	310620005670368	URBANO	310620005170	Ventosa	31062000567
4106	310620005670369	URBANO	310620005134	Jardim AmÚrica	31062000567
4107	310620005670370	URBANO	310620005134	Jardim AmÚrica	31062000567
4108	310620005670371	URBANO	310620005170	Ventosa	31062000567
4109	310620005670372	URBANO	310620005134	Jardim AmÚrica	31062000567
4110	310620005670373	URBANO	310620005170	Ventosa	31062000567
4111	310620005670374	URBANO	310620005170	Ventosa	31062000567
4112	310620005670375	URBANO	310620005182	Alpes	31062000567
4113	310620005670376	URBANO	310620005183	Pantanal	31062000567
4114	310620005670377	URBANO	310620005182	Alpes	31062000567
4115	310620005670378	URBANO	310620005173	HavaÝ	31062000567
4116	310620005670379	URBANO	310620005184	Estoril	31062000567
4117	310620005670380	URBANO	310620005186	Buritis	31062000567
4118	310620005670381	URBANO	310620005186	Buritis	31062000567
4119	310620005670382	URBANO	310620005186	Buritis	31062000567
4120	310620005670383	URBANO	310620005186	Buritis	31062000567
4121	310620005670384	URBANO	310620005186	Buritis	31062000567
4122	310620005670385	URBANO	310620005186	Buritis	31062000567
4123	310620005670386	URBANO	310620005186	Buritis	31062000567
4124	310620005670387	URBANO	310620005186	Buritis	31062000567
4125	310620005670388	URBANO	310620005186	Buritis	31062000567
4126	310620005670389	URBANO	310620005186	Buritis	31062000567
4127	310620005670390	URBANO	310620005186	Buritis	31062000567
4128	310620005670391	URBANO	310620005186	Buritis	31062000567
4129	310620005670392	URBANO	310620005186	Buritis	31062000567
4130	310620005670393	URBANO	310620005185	Palmeiras	31062000567
4131	310620005670394	URBANO	310620005173	HavaÝ	31062000567
4132	310620005670395	URBANO	310620005185	Palmeiras	31062000567

4133	310620005670396	URBANO	310620005169	Vila Nova ParaÝso	31062000567
4134	310620005670397	URBANO	310620005141	Estrela do Oriente	31062000567
4135	310620005670398	URBANO	310620005152	Bairro das Ind·strias	31062000567
4136	310620005670399	URBANO	310620005188	Vila BetÔnia	31062000567
4137	310620005670400	URBANO	310620005143	Vila Vista Alegre	31062000567
4138	310620005670401	URBANO	310620005150	Cabana do Pai Toml	31062000567
4139	310620005670402	URBANO	310620005150	Cabana do Pai Toml	31062000567
4140	310620005670403	URBANO	310620005151	Vista Alegre	31062000567
4141	310620005670404	URBANO	310620005150	Cabana do Pai Toml	31062000567
4142	310620005670405	URBANO	310620005151	Vista Alegre	31062000567
4143	310620005670406	URBANO	310620005150	Cabana do Pai Toml	31062000567
4144	310620005670407	URBANO	310620005146	Nova Gameleira	31062000567
4145	310620005670408	URBANO	310620005145	Emba·bas	31062000567
4146	310620005670409	URBANO	310620005148	Vila Nova Gameleira	31062000567
4147	310620005670410	URBANO	310620005146	Nova Gameleira	31062000567
4148	310620005670411	URBANO	310620005146	Nova Gameleira	31062000567
4149	310620005670412	URBANO	310620005148	Vila Nova Gameleira	31062000567
4150	310620005670413	URBANO	310620005166	Gameleira	31062000567
4151	310620005670414	URBANO	310620005138	GuaratÒ	31062000567
4152	310620005670415	URBANO	310620005135	Nova SuÝþa	31062000567
4153	310620005670416	URBANO	310620005136	Salgado Filho	31062000567
4154	310620005670417	URBANO	310620005136	Salgado Filho	31062000567
4155	310620005670418	URBANO	310620005135	Nova SuÝþa	31062000567
4156	310620005670419	URBANO	310620005135	Nova SuÝþa	31062000567
4157	310620005670420	URBANO	310620005134	Jardim AmÚrica	31062000567
4158	310620005670421	URBANO	310620005135	Nova SuÝþa	31062000567
4159	310620005670422	URBANO	310620005134	Jardim AmÚrica	31062000567
4160	310620005670423	URBANO	310620005134	Jardim AmÚrica	31062000567
4161	310620005670424	URBANO	310620005168	BarÒo Homem de M	31062000567
4162	310620005670425	URBANO	310620005167	BarÒo Homem de M	31062000567
4163	310620005670426	URBANO	310620005134	Jardim AmÚrica	31062000567
4164	310620005670427	URBANO	310620005134	Jardim AmÚrica	31062000567
4165	310620005670428	URBANO	310620005134	Jardim AmÚrica	31062000567
4166	310620005670429	URBANO	310620005182	Alpes	31062000567
4167	310620005670430	URBANO	310620005179	Santa Sofia	31062000567
4168	310620005670431	URBANO	310620005180	Leonina	31062000567
4169	310620005670432	URBANO	310620005182	Alpes	31062000567
4170	310620005670433	URBANO	310620005180	Leonina	31062000567
4171	310620005670434	URBANO	310620005181	Chßcara Leonina	31062000567
4172	310620005670435	URBANO	310620005181	Chßcara Leonina	31062000567
4173	310620005670436	URBANO	310620005186	Buritis	31062000567
4174	310620005670437	URBANO	310620005102	Santa L·cia	31062000567
4175	310620005670438	URBANO	310620005184	Estoril	31062000567
4176	310620005670439	URBANO	310620005187	Olhos D'┴gua	31062000567
4177	310620005670440	URBANO	310620005186	Buritis	31062000567
4178	310620005670441	URBANO	310620005185	Palmeiras	31062000567
4179	310620005670442	URBANO	310620005151	Vista Alegre	31062000567
4180	310620005670443	URBANO	310620005151	Vista Alegre	31062000567
4181	310620005670444	URBANO	310620005155	Vila Madre Gertrude	31062000567
4182	310620005670445	URBANO	310620005154	Madre Gertrudes	31062000567

4183	310620005670446	URBANO	310620005155	Vila Madre Gertrud	31062000567
4184	310620005670447	URBANO	310620005155	Vila Madre Gertrud	31062000567
4185	310620005670448	URBANO	310620005154	Madre Gertrudes	31062000567
4186	310620005670449	URBANO	310620005150	Cabana do Pai Tom	31062000567
4187	310620005670450	URBANO	310620005149	Jardin¾polis	31062000567
4188	310620005670451	URBANO	310620005150	Cabana do Pai Tom	31062000567
4189	310620005670452	URBANO	310620005149	Jardin¾polis	31062000567
4190	310620005670453	URBANO	310620005147	Vila Nova Gameleira	31062000567
4191	310620005670454	URBANO	310620005146	Nova Gameleira	31062000567
4192	310620005670455	URBANO	310620005149	Jardin¾polis	31062000567
4193	310620005670456	URBANO	310620005166	Gameleira	31062000567
4194	310620005670457	URBANO	310620005146	Nova Gameleira	31062000567
4195	310620005670458	URBANO	310620005135	Nova SuÝþa	31062000567
4196	310620005670459	URBANO	310620005166	Gameleira	31062000567
4197	310620005670460	URBANO	310620005128	Calafate	31062000567
4198	310620005670461	URBANO	310620005135	Nova SuÝþa	31062000567
4199	310620005670462	URBANO	310620005133	BarÒo Homem de M	31062000567
4200	310620005670463	URBANO	310620005167	BarÒo Homem de M	31062000567
4201	310620005670464	URBANO	310620005177	SÒo Jorge 3¬ SeþÒc	31062000567
4202	310620005670465	URBANO	310620005178	Vila Antena	31062000567
4203	310620005670466	URBANO	310620005156	Vila Madre Gertrud	31062000567
4204	310620005670467	URBANO	310620005153	Custodinha	31062000567
4205	310620005670468	URBANO	310620005156	Vila Madre Gertrud	31062000567
4206	310620005670469	URBANO	310620005159	Camargos	31062000567
4207	310620005670470	URBANO	310620005159	Camargos	31062000567
4219	310620005680013	URBANO	310620005262	Castelo	31062000568
4220	310620005680014	URBANO	310620005278	Paquetß	31062000568
4221	310620005680015	URBANO	310620005264	Conjunto Lagoa	31062000568
4222	310620005680016	URBANO	310620005278	Paquetß	31062000568
4223	310620005680017	URBANO	310620005237	Vila Paquetß	31062000568
4224	310620005680018	URBANO	310620005277	Ouro Preto	31062000568
4225	310620005680019	URBANO	310620005277	Ouro Preto	31062000568
4226	310620005680020	URBANO	310620005277	Ouro Preto	31062000568
4227	310620005680021	URBANO	310620005277	Ouro Preto	31062000568
4228	310620005680022	URBANO	310620005277	Ouro Preto	31062000568
4229	310620005680023	URBANO	310620005283	SÒo Luiz	31062000568
4230	310620005680024	URBANO	310620005283	SÒo Luiz	31062000568
4231	310620005680025	URBANO	310620005282	SÒo JosÚ	31062000568
4232	310620005680026	URBANO	310620005214	Engenho Nogueira	31062000568
4233	310620005680027	URBANO	310620005277	Ouro Preto	31062000568
4234	310620005680028	URBANO	310620005276	Novo Ouro Preto	31062000568
4235	310620005680029	URBANO	310620005277	Ouro Preto	31062000568
4236	310620005680030	URBANO	310620005277	Ouro Preto	31062000568
4237	310620005680031	URBANO	310620005262	Castelo	31062000568
4238	310620005680032	URBANO	310620005262	Castelo	31062000568
4239	310620005680033	URBANO	310620005262	Castelo	31062000568
4240	310620005680034	URBANO	310620005280	Santa Terezinha	31062000568
4241	310620005680035	URBANO	310620005280	Santa Terezinha	31062000568
4242	310620005680036	URBANO	310620005280	Santa Terezinha	31062000568
4243	310620005680037	URBANO	310620005280	Santa Terezinha	31062000568

4244	310620005680038	URBANO	310620005280	Santa Terezinha	31062000568
4245	310620005680039	URBANO	310620005280	Santa Terezinha	31062000568
4246	310620005680040	URBANO	310620005258	Bandeirantes	31062000568
4247	310620005680041	URBANO	310620005258	Bandeirantes	31062000568
4248	310620005680042	URBANO	310620005258	Bandeirantes	31062000568
4249	310620005680043	URBANO	310620005283	SÒo Luiz	31062000568
4250	310620005680044	URBANO	310620005283	SÒo Luiz	31062000568
4251	310620005680045	URBANO	310620005289	Vila Aeroporto Jara	31062000568
4252	310620005680046	URBANO	310620005257	Aeroporto	31062000568
4253	310620005680047	URBANO	310620005257	Aeroporto	31062000568
4254	310620005680048	URBANO	310620005202	Vila Rica	31062000568
4255	310620005680049	URBANO	310620005271	Jaraguß	31062000568
4256	310620005680050	URBANO	310620005273	Liberdade	31062000568
4257	310620005680051	URBANO	310620005273	Liberdade	31062000568
4258	310620005680052	URBANO	310620005273	Liberdade	31062000568
4259	310620005680053	URBANO	310620005273	Liberdade	31062000568
4260	310620005680054	URBANO	310620005273	Liberdade	31062000568
4261	310620005680055	URBANO	310620005269	Indaiß	31062000568
4262	310620005680056	URBANO	310620005269	Indaiß	31062000568
4263	310620005680057	URBANO	310620005279	Santa Rosa	31062000568
4264	310620005680058	URBANO	310620005279	Santa Rosa	31062000568
4265	310620005680059	URBANO	310620005281	SÒo Francisco	31062000568
4266	310620005680060	URBANO	310620005281	SÒo Francisco	31062000568
4267	310620005680061	URBANO	310620005281	SÒo Francisco	31062000568
4268	310620005680062	URBANO	310620005277	Ouro Preto	31062000568
4269	310620005680063	URBANO	310620005214	Engenho Nogueira	31062000568
4270	310620005680064	URBANO	310620005274	Manacas	31062000568
4271	310620005680065	URBANO	310620005249	Vila Jardim SÒo Jos	31062000568
4272	310620005680066	URBANO	310620005274	Manacas	31062000568
4273	310620005680067	URBANO	310620005274	Manacas	31062000568
4274	310620005680068	URBANO	310620005274	Manacas	31062000568
4275	310620005680069	URBANO	310620005262	Castelo	31062000568
4276	310620005680070	URBANO	310620005262	Castelo	31062000568
4277	310620005680071	URBANO	310620005241	Serrano	31062000568
4278	310620005680072	URBANO	310620005241	Serrano	31062000568
4279	310620005680073	URBANO	310620005241	Serrano	31062000568
4280	310620005680074	URBANO	310620005241	Serrano	31062000568
4281	310620005680075	URBANO	310620005241	Serrano	31062000568
4282	310620005680076	URBANO	310620005280	Santa Terezinha	31062000568
4283	310620005680077	URBANO	310620005280	Santa Terezinha	31062000568
4284	310620005680078	URBANO	310620005280	Santa Terezinha	31062000568
4285	310620005680079	URBANO	310620005280	Santa Terezinha	31062000568
4286	310620005680080	URBANO	310620005280	Santa Terezinha	31062000568
4287	310620005680081	URBANO	310620005280	Santa Terezinha	31062000568
4288	310620005680082	URBANO	310620005280	Santa Terezinha	31062000568
4289	310620005680083	URBANO	310620005258	Bandeirantes	31062000568
4290	310620005680084	URBANO	310620005280	Santa Terezinha	31062000568
4291	310620005680085	URBANO	310620005280	Santa Terezinha	31062000568
4292	310620005680086	URBANO	310620005270	Itatiaia	31062000568
4293	310620005680087	URBANO	310620005280	Santa Terezinha	31062000568

4294	310620005680088	URBANO	310620005241	Serrano	31062000568
4295	310620005680089	URBANO	310620005287	Urca	31062000568
4296	310620005680090	URBANO	310620005287	Urca	31062000568
4297	310620005680091	URBANO	310620005270	Itatiaia	31062000568
4298	310620005680092	URBANO	310620005263	Confisco	31062000568
4299	310620005680093	URBANO	310620005263	Confisco	31062000568
4300	310620005680094	URBANO	310620005260	Bra·nas	31062000568
4301	310620005680095	URBANO	310620005258	Bandeirantes	31062000568
4302	310620005680096	URBANO	310620005285	Trevo	31062000568
4303	310620005680097	URBANO	310620005285	Trevo	31062000568
4304	310620005680098	URBANO	310620005285	Trevo	31062000568
4305	310620005680099	URBANO	310620005268	Garþas	31062000568
4306	310620005680100	URBANO	310620005268	Garþas	31062000568
4307	310620005680101	URBANO	310620005272	Jardim AtlÔntico	31062000568
4308	310620005680102	URBANO	310620005260	Bra·nas	31062000568
4309	310620005680103	URBANO	310620005260	Bra·nas	31062000568
4310	310620005680104	URBANO	310620005258	Bandeirantes	31062000568
4311	310620005680105	URBANO	310620005257	Aeroporto	31062000568
4312	310620005680106	URBANO	310620005288	Vila Aeroporto	31062000568
4313	310620005680107	URBANO	310620005271	Jaraguß	31062000568
4314	310620005680108	URBANO	310620005271	Jaraguß	31062000568
4315	310620005680109	URBANO	310620005279	Santa Rosa	31062000568
4316	310620005680110	URBANO	310620005267	Dona Clara	31062000568
4317	310620005680111	URBANO	310620005267	Dona Clara	31062000568
4318	310620005680112	URBANO	310620005279	Santa Rosa	31062000568
4319	310620005680113	URBANO	310620005279	Santa Rosa	31062000568
4320	310620005680114	URBANO	310620005279	Santa Rosa	31062000568
4321	310620005680115	URBANO	310620005267	Dona Clara	31062000568
4322	310620005680116	URBANO	310620005267	Dona Clara	31062000568
4323	310620005680117	URBANO	310620005084	Vila Suzana Primeira	31062000568
4324	310620005680118	URBANO	310620005284	Suzana	31062000568
4325	310620005680119	URBANO	310620005284	Suzana	31062000568
4326	310620005680120	URBANO	310620005284	Suzana	31062000568
4327	310620005680121	URBANO	310620005267	Dona Clara	31062000568
4328	310620005680122	URBANO	310620005286	Universitßrio	31062000568
4329	310620005680123	URBANO	310620005286	Universitßrio	31062000568
4330	310620005680124	URBANO	310620005279	Santa Rosa	31062000568
4331	310620005680125	URBANO	310620005286	Universitßrio	31062000568
4332	310620005680126	URBANO	310620005281	SÒo Francisco	31062000568
4333	310620005680127	URBANO	310620005171	Vila Santa Rosa	31062000568
4334	310620005680128	URBANO	310620005281	SÒo Francisco	31062000568
4335	310620005680129	URBANO	310620005292	Vila Real 2¬ SeþÒo	31062000568
4336	310620005680130	URBANO	310620005291	Vila Real 1¬ SeþÒo	31062000568
4337	310620005680131	URBANO	310620005281	SÒo Francisco	31062000568
4338	310620005680132	URBANO	310620005281	SÒo Francisco	31062000568
4339	310620005680133	URBANO	310620005281	SÒo Francisco	31062000568
4340	310620005680134	URBANO	310620005263	Confisco	31062000568
4341	310620005680135	URBANO	310620005283	SÒo Luiz	31062000568
4342	310620005680136	URBANO	310620005214	Engenho Nogueira	31062000568
4343	310620005680137	URBANO	310620005277	Ouro Preto	31062000568

4344	310620005680138	URBANO	310620005277	Ouro Preto	31062000568
4345	310620005680139	URBANO	310620005278	Paquetß	31062000568
4346	310620005680140	URBANO	310620005277	Ouro Preto	31062000568
4347	310620005680141	URBANO	310620005277	Ouro Preto	31062000568
4348	310620005680142	URBANO	310620005278	Paquetß	31062000568
4349	310620005680143	URBANO	310620005247	Vila Jardim Alvorada	31062000568
4350	310620005680144	URBANO	310620005262	Castelo	31062000568
4351	310620005680145	URBANO	310620005262	Castelo	31062000568
4352	310620005680146	URBANO	310620005262	Castelo	31062000568
4353	310620005680147	URBANO	310620005262	Castelo	31062000568
4354	310620005680148	URBANO	310620005262	Castelo	31062000568
4355	310620005680149	URBANO	310620005262	Castelo	31062000568
4356	310620005680150	URBANO	310620005262	Castelo	31062000568
4357	310620005680151	URBANO	310620005262	Castelo	31062000568
4358	310620005680152	URBANO	310620005262	Castelo	31062000568
4359	310620005680153	URBANO	310620005262	Castelo	31062000568
4360	310620005680154	URBANO	310620005262	Castelo	31062000568
4361	310620005680155	URBANO	310620005262	Castelo	31062000568
4362	310620005680156	URBANO	310620005262	Castelo	31062000568
4363	310620005680157	URBANO	310620005262	Castelo	31062000568
4364	310620005680158	URBANO	310620005262	Castelo	31062000568
4365	310620005680159	URBANO	310620005278	Paquetß	31062000568
4366	310620005680160	URBANO	310620005278	Paquetß	31062000568
4367	310620005680161	URBANO	310620005278	Paquetß	31062000568
4368	310620005680162	URBANO	310620005278	Paquetß	31062000568
4369	310620005680163	URBANO	310620005277	Ouro Preto	31062000568
4370	310620005680164	URBANO	310620005277	Ouro Preto	31062000568
4371	310620005680165	URBANO	310620005277	Ouro Preto	31062000568
4372	310620005680166	URBANO	310620005278	Paquetß	31062000568
4373	310620005680167	URBANO	310620005277	Ouro Preto	31062000568
4374	310620005680168	URBANO	310620005277	Ouro Preto	31062000568
4375	310620005680169	URBANO	310620005277	Ouro Preto	31062000568
4376	310620005680170	URBANO	310620005277	Ouro Preto	31062000568
4377	310620005680171	URBANO	310620005277	Ouro Preto	31062000568
4378	310620005680172	URBANO	310620005283	SÒo Luiz	31062000568
4379	310620005680173	URBANO	310620005277	Ouro Preto	31062000568
4380	310620005680174	URBANO	310620005282	SÒo JosÚ	31062000568
4381	310620005680175	URBANO	310620005262	Castelo	31062000568
4382	310620005680176	URBANO	310620005262	Castelo	31062000568
4383	310620005680177	URBANO	310620005262	Castelo	31062000568
4384	310620005680178	URBANO	310620005262	Castelo	31062000568
4385	310620005680179	URBANO	310620005262	Castelo	31062000568
4386	310620005680180	URBANO	310620005280	Santa Terezinha	31062000568
4387	310620005680181	URBANO	310620005241	Serrano	31062000568
4388	310620005680182	URBANO	310620005280	Santa Terezinha	31062000568
4389	310620005680183	URBANO	310620005264	Conjunto Lagoa	31062000568
4390	310620005680184	URBANO	310620005277	Ouro Preto	31062000568
4391	310620005680185	URBANO	310620005258	Bandeirantes	31062000568
4392	310620005680186	URBANO	310620005277	Ouro Preto	31062000568
4393	310620005680187	URBANO	310620005283	SÒo Luiz	31062000568

4394	310620005680188	URBANO	310620005273	Liberdade	31062000568
4395	310620005680189	URBANO	310620005271	Jaraguß	31062000568
4396	310620005680190	URBANO	310620005271	Jaraguß	31062000568
4397	310620005680191	URBANO	310620005279	Santa Rosa	31062000568
4398	310620005680192	URBANO	310620005273	Liberdade	31062000568
4399	310620005680193	URBANO	310620005273	Liberdade	31062000568
4400	310620005680194	URBANO	310620005053	Vila Santo Ant¶nio	31062000568
4401	310620005680195	URBANO	310620005269	Indaiß	31062000568
4402	310620005680196	URBANO	310620005269	Indaiß	31062000568
4403	310620005680197	URBANO	310620005269	Indaiß	31062000568
4404	310620005680198	URBANO	310620005281	SÒo Francisco	31062000568
4405	310620005680199	URBANO	310620005279	Santa Rosa	31062000568
4406	310620005680200	URBANO	310620005286	Universitßrio	31062000568
4407	310620005680201	URBANO	310620005290	Vila Engenho Nogue	31062000568
4408	310620005680202	URBANO	310620005214	Engenho Nogueira	31062000568
4409	310620005680203	URBANO	310620005290	Vila Engenho Nogue	31062000568
4410	310620005680204	URBANO	310620005274	Manacas	31062000568
4411	310620005680205	URBANO	310620005274	Manacas	31062000568
4412	310620005680206	URBANO	310620005274	Manacas	31062000568
4413	310620005680207	URBANO	310620005274	Manacas	31062000568
4414	310620005680208	URBANO	310620005274	Manacas	31062000568
4415	310620005680209	URBANO	310620005274	Manacas	31062000568
4416	310620005680210	URBANO	310620005274	Manacas	31062000568
4417	310620005680211	URBANO	310620005274	Manacas	31062000568
4418	310620005680212	URBANO	310620005274	Manacas	31062000568
4419	310620005680213	URBANO	310620005262	Castelo	31062000568
4420	310620005680214	URBANO	310620005262	Castelo	31062000568
4421	310620005680215	URBANO	310620005262	Castelo	31062000568
4422	310620005680216	URBANO	310620005262	Castelo	31062000568
4423	310620005680217	URBANO	310620005262	Castelo	31062000568
4424	310620005680218	URBANO	310620005262	Castelo	31062000568
4425	310620005680219	URBANO	310620005262	Castelo	31062000568
4426	310620005680220	URBANO	310620005262	Castelo	31062000568
4427	310620005680221	URBANO	310620005241	Serrano	31062000568
4428	310620005680222	URBANO	310620005280	Santa Terezinha	31062000568
4429	310620005680223	URBANO	310620005280	Santa Terezinha	31062000568
4430	310620005680224	URBANO	310620005287	Urca	31062000568
4431	310620005680225	URBANO	310620005280	Santa Terezinha	31062000568
4432	310620005680226	URBANO	310620005287	Urca	31062000568
4433	310620005680227	URBANO	310620005287	Urca	31062000568
4434	310620005680228	URBANO	310620005287	Urca	31062000568
4435	310620005680229	URBANO	310620005258	Bandeirantes	31062000568
4436	310620005680230	URBANO	310620005270	Itatiaia	31062000568
4437	310620005680231	URBANO	310620005260	Bra·nas	31062000568
4438	310620005680232	URBANO	310620005033	Xangri-lß	31062000568
4439	310620005680233	URBANO	310620005190	CÚu Azul	31062000568
4440	310620005680234	URBANO	310620005265	Conjunto SÒo Franc	31062000568
4441	310620005680235	URBANO	310620005260	Bra·nas	31062000568
4442	310620005680236	URBANO	310620005190	CÚu Azul	31062000568
4443	310620005680237	URBANO	310620005285	Trevo	31062000568

4444	310620005680238	URBANO	310620005260	Bra·nas	31062000568
4445	310620005680239	URBANO	310620005266	Copacabana	31062000568
4446	310620005680240	URBANO	310620005285	Trevo	31062000568
4447	310620005680241	URBANO	310620005283	SÒo Luiz	31062000568
4448	310620005680242	URBANO	310620005267	Dona Clara	31062000568
4449	310620005680243	URBANO	310620005271	Jaraguß	31062000568
4450	310620005680244	URBANO	310620005288	Vila Aeroporto	31062000568
4451	310620005680245	URBANO	310620005271	Jaraguß	31062000568
4452	310620005680246	URBANO	310620005267	Dona Clara	31062000568
4453	310620005680247	URBANO	310620005279	Santa Rosa	31062000568
4454	310620005680248	URBANO	310620005267	Dona Clara	31062000568
4455	310620005680249	URBANO	310620005267	Dona Clara	31062000568
4456	310620005680250	URBANO	310620005267	Dona Clara	31062000568
4457	310620005680251	URBANO	310620005267	Dona Clara	31062000568
4458	310620005680252	URBANO	310620005267	Dona Clara	31062000568
4459	310620005680253	URBANO	310620005267	Dona Clara	31062000568
4460	310620005680254	URBANO	310620005267	Dona Clara	31062000568
4461	310620005680255	URBANO	310620005284	Suzana	31062000568
4462	310620005680256	URBANO	310620005284	Suzana	31062000568
4463	310620005680257	URBANO	310620005085	Vila Suzana Segunda	31062000568
4464	310620005680258	URBANO	310620005267	Dona Clara	31062000568
4465	310620005680259	URBANO	310620005284	Suzana	31062000568
4466	310620005680260	URBANO	310620005279	Santa Rosa	31062000568
4467	310620005680261	URBANO	310620005281	SÒo Francisco	31062000568
4468	310620005680262	URBANO	310620005171	Vila Santa Rosa	31062000568
4469	310620005680263	URBANO	310620005259	Vila SÒo Francisco	31062000568
4470	310620005680264	URBANO	310620005287	Urca	31062000568
4471	310620005680265	URBANO	310620005190	CÚu Azul	31062000568
4472	310620005680266	URBANO	310620005249	Vila Jardim SÒo Jos	31062000568
4473	310620005680267	URBANO	310620005249	Vila Jardim SÒo Jos	31062000568
4474	310620005680268	URBANO	310620005275	Nova Pampulha	31062000568
4475	310620005680269	URBANO	310620005293	Lagoa da Pampulha	31062000568
4476	310620005690001	URBANO	310620005190	CÚu Azul	31062000569
4477	310620005690002	URBANO	310620005190	CÚu Azul	31062000569
4478	310620005690003	URBANO	310620005190	CÚu Azul	31062000569
4479	310620005690004	URBANO	310620005266	Copacabana	31062000569
4480	310620025610001	URBANO	310620025347	Diamante	31062002561
4481	310620025610002	URBANO	310620025353	Miramar	31062002561
4482	310620025610003	URBANO	310620025346	Brasil Industrial	31062002561
4483	310620025610004	URBANO	310620025346	Brasil Industrial	31062002561
4484	310620025610005	URBANO	310620025346	Brasil Industrial	31062002561
4485	310620025610006	URBANO	310620025313	Cardoso	31062002561
4486	310620025610007	URBANO	310620025313	Cardoso	31062002561
4487	310620025610008	URBANO	310620025313	Cardoso	31062002561
4488	310620025610009	URBANO	310620025344	Novo Santa Cecilia	31062002561
4489	310620025610010	URBANO	310620025345	Vila Pinho	31062002561
4490	310620025610011	URBANO	310620025345	Vila Pinho	31062002561
4491	310620025610012	URBANO	310620025345	Vila Pinho	31062002561
4492	310620025610013	URBANO	310620025345	Vila Pinho	31062002561
4493	310620025610014	URBANO	310620025345	Vila Pinho	31062002561

4494	310620025610015	URBANO	310620025345	Vila Pinho	31062002561
4495	310620025610016	URBANO	310620025348	Olaria	31062002561
4496	310620025610017	URBANO	310620025348	Olaria	31062002561
4497	310620025610018	URBANO	310620025348	Olaria	31062002561
4509	310620025610030	URBANO	310620025346	Brasil Industrial	31062002561
4510	310620025610031	URBANO	310620025313	Cardoso	31062002561
4511	310620025610032	URBANO	310620025313	Cardoso	31062002561
4512	310620025610033	URBANO	310620025313	Cardoso	31062002561
4513	310620025610034	URBANO	310620025313	Cardoso	31062002561
4514	310620025610035	URBANO	310620025313	Cardoso	31062002561
4515	310620025610036	URBANO	310620025313	Cardoso	31062002561
4516	310620025610037	URBANO	310620025326	Vila Ecol¾gica	31062002561
4517	310620025610038	URBANO	310620025337	Petropolis	31062002561
4518	310620025610039	URBANO	310620025339	Santa Rita	31062002561
4519	310620025610040	URBANO	310620025340	Ernesto Nascimento	31062002561
4520	310620025610041	URBANO	310620025340	Ernesto Nascimento	31062002561
4521	310620025610042	URBANO	310620025339	Santa Rita	31062002561
4522	310620025610043	URBANO	310620025350	Santa Cecilia	31062002561
4523	310620025610044	URBANO	310620025351	Vila Formosa	31062002561
4524	310620025610045	URBANO	310620025352	Castanheira	31062002561
4525	310620025610046	URBANO	310620025348	Olaria	31062002561
4526	310620025610047	URBANO	310620025348	Olaria	31062002561
4527	310620025610048	URBANO	310620025348	Olaria	31062002561
4528	310620025610049	URBANO	310620025347	Diamante	31062002561
4529	310620025610050	URBANO	310620025347	Diamante	31062002561
4530	310620025610051	URBANO	310620025355	Teixeira Dias	31062002561
4531	310620025610052	URBANO	310620025355	Teixeira Dias	31062002561
4532	310620025610053	URBANO	310620025354	Santa Helena	31062002561
4533	310620025610054	URBANO	310620025354	Santa Helena	31062002561
4534	310620025610055	URBANO	310620025353	Miramar	31062002561
4535	310620025610056	URBANO	310620025353	Miramar	31062002561
4536	310620025610057	URBANO	310620025311	Flavio Marques Lisb	31062002561
4537	310620025610058	URBANO	310620025313	Cardoso	31062002561
4538	310620025610059	URBANO	310620025312	Flavio De Oliveira	31062002561
4539	310620025610060	URBANO	310620025312	Flavio De Oliveira	31062002561
4540	310620025610061	URBANO	310620025312	Flavio De Oliveira	31062002561
4541	310620025610062	URBANO	310620025313	Cardoso	31062002561
4542	310620025610063	URBANO	310620025323	Pongelupe	31062002561
4543	310620025610064	URBANO	310620025323	Pongelupe	31062002561
4544	310620025610065	URBANO	310620025323	Pongelupe	31062002561
4545	310620025610066	URBANO	310620025324	Solar do Barreiro	31062002561
4546	310620025610067	URBANO	310620025324	Solar do Barreiro	31062002561
4547	310620025610068	URBANO	310620025330	Vila Independencia	31062002561
4548	310620025610069	URBANO	310620025333	IndependÛncia	31062002561
4549	310620025610070	URBANO	310620025333	IndependÛncia	31062002561
4550	310620025610071	URBANO	310620025330	Vila Independencia	31062002561
4551	310620025610072	URBANO	310620025337	Petropolis	31062002561
4552	310620025610073	URBANO	310620025335	Vila Mangueiras	31062002561
4553	310620025610074	URBANO	310620025336	Mangueiras	31062002561
4554	310620025610075	URBANO	310620025336	Mangueiras	31062002561

4555	310620025610076	URBANO	310620025336	Mangueiras	31062002561
4556	310620025610077	URBANO	310620025336	Mangueiras	31062002561
4557	310620025610078	URBANO	310620025341	Vale do Jatoba	31062002561
4558	310620025610079	URBANO	310620025341	Vale do Jatoba	31062002561
4559	310620025610080	URBANO	310620025341	Vale do Jatoba	31062002561
4560	310620025610081	URBANO	310620025341	Vale do Jatoba	31062002561
4561	310620025610082	URBANO	310620025341	Vale do Jatoba	31062002561
4562	310620025610083	URBANO	310620025341	Vale do Jatoba	31062002561
4563	310620025610084	URBANO	310620025341	Vale do Jatoba	31062002561
4564	310620025610085	URBANO	310620025350	Santa Cecilia	31062002561
4565	310620025610086	URBANO	310620025350	Santa Cecilia	31062002561
4566	310620025610087	URBANO	310620025352	Castanheira	31062002561
4567	310620025610088	URBANO	310620025352	Castanheira	31062002561
4568	310620025610089	URBANO	310620025350	Santa Cecilia	31062002561
4569	310620025610090	URBANO	310620025349	CDI Jatoba	31062002561
4570	310620025610091	URBANO	310620025343	Conjunto Jatoba	31062002561
4571	310620025610092	URBANO	310620025343	Conjunto Jatoba	31062002561
4572	310620025610093	URBANO	310620025343	Conjunto Jatoba	31062002561
4573	310620025610094	URBANO	310620025349	CDI Jatoba	31062002561
4574	310620025610095	URBANO	310620025300	Marilandia	31062002561
4575	310620025610096	URBANO	310620025363	Tirol	31062002561
4576	310620025610097	URBANO	310620025347	Diamante	31062002561
4577	310620025610098	URBANO	310620025358	Ademar Maldonadc	31062002561
4578	310620025610099	URBANO	310620025358	Ademar Maldonadc	31062002561
4579	310620025610100	URBANO	310620025358	Ademar Maldonadc	31062002561
4580	310620025610101	URBANO	310620025355	Teixeira Dias	31062002561
4581	310620025610102	URBANO	310620025356	Barreiro	31062002561
4582	310620025610103	URBANO	310620025355	Teixeira Dias	31062002561
4583	310620025610104	URBANO	310620025354	Santa Helena	31062002561
4584	310620025610105	URBANO	310620025354	Santa Helena	31062002561
4585	310620025610106	URBANO	310620025354	Santa Helena	31062002561
4586	310620025610107	URBANO	310620025354	Santa Helena	31062002561
4587	310620025610108	URBANO	310620025356	Barreiro	31062002561
4588	310620025610109	URBANO	310620025356	Barreiro	31062002561
4589	310620025610110	URBANO	310620025314	Araguaia	31062002561
4590	310620025610111	URBANO	310620025314	Araguaia	31062002561
4591	310620025610112	URBANO	310620025311	Flavio Marques Lisb	31062002561
4592	310620025610113	URBANO	310620025311	Flavio Marques Lisb	31062002561
4593	310620025610114	URBANO	310620025311	Flavio Marques Lisb	31062002561
4594	310620025610115	URBANO	310620025305	Serra do Curral	31062002561
4595	310620025610116	URBANO	310620025306	Bonsucesso	31062002561
4596	310620025610117	URBANO	310620025304	SÒo JoÒo	31062002561
4597	310620025610118	URBANO	310620025304	SÒo JoÒo	31062002561
4598	310620025610119	URBANO	310620025189	Pilar	31062002561
4599	310620025610120	URBANO	310620025305	Serra do Curral	31062002561
4600	310620025610121	URBANO	310620025305	Serra do Curral	31062002561
4601	310620025610122	URBANO	310620025333	IndependÛncia	31062002561
4602	310620025610123	URBANO	310620025334	MineirÒo	31062002561
4603	310620025610124	URBANO	310620025333	IndependÛncia	31062002561
4604	310620025610125	URBANO	310620025334	MineirÒo	31062002561

4605	310620025610126	URBANO	310620025334	MineirÒo	31062002561
4606	310620025610127	URBANO	310620025333	IndependÛncia	31062002561
4607	310620025610128	URBANO	310620025333	IndependÛncia	31062002561
4608	310620025610129	URBANO	310620025333	IndependÛncia	31062002561
4609	310620025610130	URBANO	310620025333	IndependÛncia	31062002561
4610	310620025610131	URBANO	310620025333	IndependÛncia	31062002561
4611	310620025610132	URBANO	310620025333	IndependÛncia	31062002561
4612	310620025610133	URBANO	310620025492	Itaipu	31062002561
4613	310620025610134	URBANO	310620025492	Itaipu	31062002561
4614	310620025610135	URBANO	310620025363	Tirol	31062002561
4615	310620025610136	URBANO	310620025363	Tirol	31062002561
4616	310620025610137	URBANO	310620025363	Tirol	31062002561
4617	310620025610138	URBANO	310620025363	Tirol	31062002561
4618	310620025610139	URBANO	310620025363	Tirol	31062002561
4619	310620025610140	URBANO	310620025363	Tirol	31062002561
4620	310620025610141	URBANO	310620025363	Tirol	31062002561
4621	310620025610142	URBANO	310620025347	Diamante	31062002561
4622	310620025610143	URBANO	310620025361	JoÒo Paulo II	31062002561
4623	310620025610144	URBANO	310620025356	Barreiro	31062002561
4624	310620025610145	URBANO	310620025356	Barreiro	31062002561
4625	310620025610146	URBANO	310620025356	Barreiro	31062002561
4626	310620025610147	URBANO	310620025356	Barreiro	31062002561
4627	310620025610148	URBANO	310620025356	Barreiro	31062002561
4628	310620025610149	URBANO	310620025356	Barreiro	31062002561
4629	310620025610150	URBANO	310620025356	Barreiro	31062002561
4630	310620025610151	URBANO	310620025315	Milionario	31062002561
4631	310620025610152	URBANO	310620025315	Milionario	31062002561
4632	310620025610153	URBANO	310620025314	Araguaia	31062002561
4633	310620025610154	URBANO	310620025314	Araguaia	31062002561
4634	310620025610155	URBANO	310620025314	Araguaia	31062002561
4635	310620025610156	URBANO	310620025311	Flavio Marques Lisb	31062002561
4636	310620025610157	URBANO	310620025299	Jatobß	31062002561
4637	310620025610158	URBANO	310620025299	Jatobß	31062002561
4638	310620025610159	URBANO	310620025298	Vila Piratininga	31062002561
4639	310620025610160	URBANO	310620025492	Itaipu	31062002561
4640	310620025610161	URBANO	310620025492	Itaipu	31062002561
4641	310620025610162	URBANO	310620025492	Itaipu	31062002561
4642	310620025610163	URBANO	310620025492	Itaipu	31062002561
4643	310620025610164	URBANO	310620025492	Itaipu	31062002561
4644	310620025610165	URBANO	310620025492	Itaipu	31062002561
4645	310620025610166	URBANO	310620025294	T·nel de IbiritÚ	31062002561
4646	310620025610167	URBANO	310620025363	Tirol	31062002561
4647	310620025610168	URBANO	310620025363	Tirol	31062002561
4648	310620025610169	URBANO	310620025361	JoÒo Paulo II	31062002561
4649	310620025610170	URBANO	310620025361	JoÒo Paulo II	31062002561
4650	310620025610171	URBANO	310620025360	Vila Atila de Paiva	31062002561
4651	310620025610172	URBANO	310620025360	Vila Atila de Paiva	31062002561
4652	310620025610173	URBANO	310620025357	Santa Margarida	31062002561
4653	310620025610183	URBANO	310620025319	Novo das Industrias	31062002561
4654	310620025610184	URBANO	310620025319	Novo das Industrias	31062002561

4655	310620025610185	URBANO	310620025319	Novo das Industrias	31062002561
4656	310620025610186	URBANO	310620025321	Alta TensÒo 2¬ Seþ	31062002561
4657	310620025610187	URBANO	310620025319	Novo das Industrias	31062002561
4658	310620025610188	URBANO	310620025319	Novo das Industrias	31062002561
4659	310620025610189	URBANO	310620025315	Milionario	31062002561
4660	310620025610190	URBANO	310620025315	Milionario	31062002561
4661	310620025610191	URBANO	310620025315	Milionario	31062002561
4662	310620025610192	URBANO	310620025315	Milionario	31062002561
4663	310620025610193	URBANO	310620025315	Milionario	31062002561
4664	310620025610194	URBANO	310620025316	Vila Nova dos Milio	31062002561
4665	310620025610195	URBANO	310620025315	Milionario	31062002561
4666	310620025610196	URBANO	310620025315	Milionario	31062002561
4667	310620025610197	URBANO	310620025315	Milionario	31062002561
4668	310620025610198	URBANO	310620025314	Araguaia	31062002561
4669	310620025610199	URBANO	310620025314	Araguaia	31062002561
4670	310620025610200	URBANO	310620025311	Flavio Marques Lisb	31062002561
4671	310620025610201	URBANO	310620025310	Vila Cemig	31062002561
4672	310620025610202	URBANO	310620025310	Vila Cemig	31062002561
4673	310620025610203	URBANO	310620025310	Vila Cemig	31062002561
4674	310620025610204	URBANO	310620025310	Vila Cemig	31062002561
4675	310620025610205	URBANO	310620025311	Flavio Marques Lisb	31062002561
4676	310620025610206	URBANO	310620025308	Alto das Antenas	31062002561
4677	310620025610207	URBANO	310620025189	Pilar	31062002561
4678	310620025610208	URBANO	310620025189	Pilar	31062002561
4679	310620025610209	URBANO	310620025299	Jatobß	31062002561
4680	310620025610210	URBANO	310620025299	Jatobß	31062002561
4681	310620025610211	URBANO	310620025364	LindÚia	31062002561
4682	310620025610212	URBANO	310620025364	LindÚia	31062002561
4683	310620025610213	URBANO	310620025492	Itaipu	31062002561
4684	310620025610214	URBANO	310620025492	Itaipu	31062002561
4685	310620025610215	URBANO	310620025364	LindÚia	31062002561
4686	310620025610216	URBANO	310620025364	LindÚia	31062002561
4687	310620025610217	URBANO	310620025322	das Industrias I	31062002561
4688	310620025610218	URBANO	310620025322	das Industrias I	31062002561
4689	310620025610219	URBANO	310620025322	das Industrias I	31062002561
4690	310620025610220	URBANO	310620025322	das Industrias I	31062002561
4691	310620025610221	URBANO	310620025319	Novo das Industrias	31062002561
4692	310620025610222	URBANO	310620025319	Novo das Industrias	31062002561
4693	310620025610223	URBANO	310620025319	Novo das Industrias	31062002561
4694	310620025610224	URBANO	310620025315	Milionario	31062002561
4695	310620025610225	URBANO	310620025315	Milionario	31062002561
4696	310620025610226	URBANO	310620025315	Milionario	31062002561
4697	310620025610227	URBANO	310620025314	Araguaia	31062002561
4698	310620025610228	URBANO	310620025314	Araguaia	31062002561
4699	310620025610229	URBANO	310620025314	Araguaia	31062002561
4700	310620025610230	URBANO	310620025314	Araguaia	31062002561
4701	310620025610231	URBANO	310620025311	Flavio Marques Lisb	31062002561
4702	310620025610232	URBANO	310620025311	Flavio Marques Lisb	31062002561
4703	310620025610233	URBANO	310620025309	Esperanþa	31062002561
4704	310620025610234	URBANO	310620025309	Esperanþa	31062002561

4705	310620025610235	URBANO	310620025364	LindÚia	31062002561
4706	310620025610236	URBANO	310620025364	LindÚia	31062002561
4707	310620025610237	URBANO	310620025364	LindÚia	31062002561
4708	310620025610238	URBANO	310620025364	LindÚia	31062002561
4709	310620025610239	URBANO	310620025364	LindÚia	31062002561
4710	310620025610240	URBANO	310620025364	LindÚia	31062002561
4711	310620025610241	URBANO	310620025364	LindÚia	31062002561
4712	310620025610242	URBANO	310620025322	das Industrias I	31062002561
4713	310620025610243	URBANO	310620025322	das Industrias I	31062002561
4714	310620025610244	URBANO	310620025322	das Industrias I	31062002561
4715	310620025610245	URBANO	310620025322	das Industrias I	31062002561
4716	310620025610246	URBANO	310620025322	das Industrias I	31062002561
4717	310620025610247	URBANO	310620025319	Novo das Industrias	31062002561
4718	310620025610248	URBANO	310620025306	Bonsucesso	31062002561
4719	310620025610249	URBANO	310620025306	Bonsucesso	31062002561
4720	310620025610250	URBANO	310620025314	Araguaia	31062002561
4721	310620025610251	URBANO	310620025306	Bonsucesso	31062002561
4722	310620025610252	URBANO	310620025314	Araguaia	31062002561
4723	310620025610253	URBANO	310620025307	Bernadete	31062002561
4724	310620025610254	URBANO	310620025187	Olhos D'┴gua	31062002561
4725	310620025610255	URBANO	310620025187	Olhos D'┴gua	31062002561
4726	310620025610256	URBANO	310620025364	LindÚia	31062002561
4727	310620025610257	URBANO	310620025364	LindÚia	31062002561
4728	310620025610258	URBANO	310620025364	LindÚia	31062002561
4729	310620025610259	URBANO	310620025364	LindÚia	31062002561
4730	310620025610260	URBANO	310620025364	LindÚia	31062002561
4731	310620025610261	URBANO	310620025364	LindÚia	31062002561
4732	310620025610262	URBANO	310620025364	LindÚia	31062002561
4733	310620025610263	URBANO	310620025364	LindÚia	31062002561
4734	310620025610264	URBANO	310620025364	LindÚia	31062002561
4735	310620025610265	URBANO	310620025364	LindÚia	31062002561
4736	310620025610266	URBANO	310620025364	LindÚia	31062002561
4737	310620025610267	URBANO	310620025364	LindÚia	31062002561
4738	310620025610268	URBANO	310620025364	LindÚia	31062002561
4739	310620025610269	URBANO	310620025364	LindÚia	31062002561
4740	310620025610270	URBANO	310620025364	LindÚia	31062002561
4741	310620025610271	URBANO	310620025353	Miramar	31062002561
4742	310620025610272	URBANO	310620025346	Brasil Industrial	31062002561
4743	310620025610273	URBANO	310620025344	Novo Santa Cecilia	31062002561
4744	310620025610274	URBANO	310620025347	Diamante	31062002561
4745	310620025610275	URBANO	310620025347	Diamante	31062002561
4746	310620025610276	URBANO	310620025347	Diamante	31062002561
4747	310620025610277	URBANO	310620025346	Brasil Industrial	31062002561
4748	310620025610278	URBANO	310620025346	Brasil Industrial	31062002561
4749	310620025610279	URBANO	310620025313	Cardoso	31062002561
4750	310620025610280	URBANO	310620025324	Solar do Barreiro	31062002561
4751	310620025610281	URBANO	310620025345	Vila Pinho	31062002561
4752	310620025610282	URBANO	310620025345	Vila Pinho	31062002561
4753	310620025610283	URBANO	310620025335	Vila Mangueiras	31062002561
4754	310620025610284	URBANO	310620025345	Vila Pinho	31062002561

4755	310620025610285	URBANO	310620025352	Castanheira	31062002561
4756	310620025610286	URBANO	310620025348	Olaria	31062002561
4757	310620025610287	URBANO	310620025348	Olaria	31062002561
4758	310620025610288	URBANO	310620025348	Olaria	31062002561
4759	310620025610289	URBANO	310620025330	Vila Independencia	31062002561
4760	310620025610290	URBANO	310620025347	Diamante	31062002561
4761	310620025610291	URBANO	310620025355	Teixeira Dias	31062002561
4762	310620025610292	URBANO	310620025353	Miramar	31062002561
4763	310620025610293	URBANO	310620025328	Aguas Claras	31062002561
4764	310620025610294	URBANO	310620025346	Brasil Industrial	31062002561
4765	310620025610295	URBANO	310620025328	Aguas Claras	31062002561
4766	310620025610296	URBANO	310620025337	Petropolis	31062002561
4767	310620025610297	URBANO	310620025340	Ernesto Nasciment	31062002561
4768	310620025610298	URBANO	310620025317	Vila Copasa	31062002561
4769	310620025610299	URBANO	310620025352	Castanheira	31062002561
4770	310620025610300	URBANO	310620025351	Vila Formosa	31062002561
4771	310620025610301	URBANO	310620025355	Teixeira Dias	31062002561
4772	310620025610302	URBANO	310620025354	Santa Helena	31062002561
4773	310620025610303	URBANO	310620025353	Miramar	31062002561
4774	310620025610304	URBANO	310620025313	Cardoso	31062002561
4775	310620025610305	URBANO	310620025341	Vale do Jatoba	31062002561
4776	310620025610306	URBANO	310620025352	Castanheira	31062002561
4777	310620025610307	URBANO	310620025352	Castanheira	31062002561
4778	310620025610308	URBANO	310620025355	Teixeira Dias	31062002561
4779	310620025610309	URBANO	310620025314	Araguaia	31062002561
4780	310620025610310	URBANO	310620025306	Bonsucesso	31062002561
4781	310620025610311	URBANO	310620025301	Vila Batik	31062002561
4782	310620025610312	URBANO	310620025341	Vale do Jatoba	31062002561
4783	310620025610313	URBANO	310620025343	Conjunto Jatoba	31062002561
4784	310620025610314	URBANO	310620025341	Vale do Jatoba	31062002561
4785	310620025610315	URBANO	310620025363	Tirol	31062002561
4786	310620025610316	URBANO	310620025300	Marilandia	31062002561
4787	310620025610317	URBANO	310620025297	Marieta 3¬ SeþÒo	31062002561
4788	310620025610318	URBANO	310620025296	Marieta 2¬ SeþÒo	31062002561
4789	310620025610319	URBANO	310620025295	Marieta 1¬ SeþÒo	31062002561
4790	310620025610320	URBANO	310620025363	Tirol	31062002561
4791	310620025610321	URBANO	310620025295	Marieta 1¬ SeþÒo	31062002561
4792	310620025610322	URBANO	310620025311	Flavio Marques Lisb	31062002561
4793	310620025610323	URBANO	310620025189	Pilar	31062002561
4794	310620025610324	URBANO	310620025189	Pilar	31062002561
4806	310620025610336	URBANO	310620025328	Aguas Claras	31062002561
4807	310620025610337	URBANO	310620025337	Petropolis	31062002561
4808	310620025610338	URBANO	310620025338	Vila Petropolis	31062002561
4809	310620025610339	URBANO	310620025329	Vitoria da Conquist	31062002561
4810	310620025610340	URBANO	310620025337	Petropolis	31062002561
4811	310620025610341	URBANO	310620025327	Distrito Industrial d	31062002561
4812	310620025610342	URBANO	310620025327	Distrito Industrial d	31062002561
4813	310620025610343	URBANO	310620025352	Castanheira	31062002561
4814	310620025610344	URBANO	310620025348	Olaria	31062002561
4815	310620025610345	URBANO	310620025358	Ademar Maldonad	31062002561

4816	310620025610346	URBANO	310620025356	Barreiro	31062002561
4817	310620025610347	URBANO	310620025347	Diamante	31062002561
4818	310620025610348	URBANO	310620025355	Teixeira Dias	31062002561
4819	310620025610349	URBANO	310620025311	Flavio Marques Lisb	31062002561
4820	310620025610350	URBANO	310620025312	Flavio De Oliveira	31062002561
4821	310620025610351	URBANO	310620025325	Corumbiara	31062002561
4822	310620025610352	URBANO	310620025324	Solar do Barreiro	31062002561
4823	310620025610353	URBANO	310620025324	Solar do Barreiro	31062002561
4824	310620025610354	URBANO	310620025324	Solar do Barreiro	31062002561
4825	310620025610355	URBANO	310620025333	IndependÛncia	31062002561
4826	310620025610356	URBANO	310620025333	IndependÛncia	31062002561
4827	310620025610357	URBANO	310620025332	Vila Independencia	31062002561
4828	310620025610358	URBANO	310620025336	Mangueiras	31062002561
4829	310620025610359	URBANO	310620025334	MineirÒo	31062002561
4830	310620025610360	URBANO	310620025336	Mangueiras	31062002561
4831	310620060640030	URBANO	310620060397	Tres Marias	31062006064
4832	310620060640031	URBANO	310620060396	Boa Esperanþa	31062006064
4833	310620060640032	URBANO	310620060400	Belmonte	31062006064
4834	310620060640033	URBANO	310620060407	Ribeiro de Abreu	31062006064
4835	310620060640034	URBANO	310620060407	Ribeiro de Abreu	31062006064
4836	310620060640035	URBANO	310620060406	Beira Linha	31062006064
4837	310620060640036	URBANO	310620060406	Beira Linha	31062006064
4838	310620060640037	URBANO	310620060405	Paulo VI	31062006064
4839	310620060640038	URBANO	310620060405	Paulo VI	31062006064
4840	310620025610361	URBANO	310620025341	Vale do Jatoba	31062002561
4841	310620025610362	URBANO	310620025352	Castanheira	31062002561
4842	310620025610363	URBANO	310620025341	Vale do Jatoba	31062002561
4843	310620025610364	URBANO	310620025356	Barreiro	31062002561
4844	310620025610365	URBANO	310620025356	Barreiro	31062002561
4845	310620025610366	URBANO	310620025356	Barreiro	31062002561
4846	310620025610367	URBANO	310620025354	Santa Helena	31062002561
4847	310620025610368	URBANO	310620025355	Teixeira Dias	31062002561
4848	310620025610369	URBANO	310620025355	Teixeira Dias	31062002561
4849	310620025610370	URBANO	310620025354	Santa Helena	31062002561
4850	310620025610371	URBANO	310620025314	Araguaia	31062002561
4851	310620025610372	URBANO	310620025314	Araguaia	31062002561
4852	310620025610373	URBANO	310620025315	Milionario	31062002561
4853	310620025610374	URBANO	310620025315	Milionario	31062002561
4854	310620025610375	URBANO	310620025362	Vila Tirol	31062002561
4855	310620025610376	URBANO	310620025359	Atila de Paiva	31062002561
4856	310620025610377	URBANO	310620025320	Alta TensÒo 1¬ Seþ	31062002561
4857	310620025610378	URBANO	310620025320	Alta TensÒo 1¬ Seþ	31062002561
4858	310620025610379	URBANO	310620025322	das Industrias I	31062002561
4859	310620025610380	URBANO	310620025321	Alta TensÒo 2¬ Seþ	31062002561
4860	310620025610381	URBANO	310620025321	Alta TensÒo 2¬ Seþ	31062002561
4861	310620025610382	URBANO	310620025315	Milionario	31062002561
4862	310620025610383	URBANO	310620025314	Araguaia	31062002561
4863	310620025610384	URBANO	310620025299	Jatobß	31062002561
4864	310620025610385	URBANO	310620025364	LindÚia	31062002561
4865	310620025610386	URBANO	310620025364	LindÚia	31062002561

4866	310620025610387	URBANO	310620025364	LindÚia	31062002561
4867	310620025610388	URBANO	310620025364	LindÚia	31062002561
4868	310620025610389	URBANO	310620025364	LindÚia	31062002561
4869	310620025610390	URBANO	310620025364	LindÚia	31062002561
4870	310620025610391	URBANO	310620025294	T·nel de IbiritÚ	31062002561
4871	310620025610392	URBANO	310620025321	Alta TensÒo 2¬ Seþ	31062002561
4872	310620025610393	URBANO	310620025319	Novo das Industrias	31062002561
4873	310620025610394	URBANO	310620025314	Araguaia	31062002561
4874	310620025610395	URBANO	310620025306	Bonsucesso	31062002561
4875	310620025610396	URBANO	310620025311	Flavio Marques Lisb	31062002561
4876	310620025610397	URBANO	310620025306	Bonsucesso	31062002561
4877	310620025610398	URBANO	310620025310	Vila Cemig	31062002561
4878	310620025610399	URBANO	310620025363	Tirol	31062002561
4879	310620025610400	URBANO	310620025306	Bonsucesso	31062002561
4880	310620025610401	URBANO	310620025314	Araguaia	31062002561
4881	310620025610402	URBANO	310620025302	Vila Olhos D'┴gua	31062002561
4882	310620025610403	URBANO	310620025189	Pilar	31062002561
4883	310620025610404	URBANO	310620025364	LindÚia	31062002561
4884	310620025610405	URBANO	310620025364	LindÚia	31062002561
4885	310620025610406	URBANO	310620025342	Jardim do Vale	31062002561
4886	310620025610407	URBANO	310620025339	Santa Rita	31062002561
4887	310620025610408	URBANO	310620025335	Vila Mangueiras	31062002561
4888	310620025610409	URBANO	310620025492	Itaipu	31062002561
4889	310620025610410	URBANO	310620025298	Vila Piratininga	31062002561
4890	310620025610411	URBANO	310620025294	T·nel de IbiritÚ	31062002561
4891	310620025610412	URBANO	310620025357	Santa Margarida	31062002561
4892	310620025610413	URBANO	310620025357	Santa Margarida	31062002561
4893	310620025610414	URBANO	310620025315	Milionario	31062002561
4894	310620025610415	URBANO	310620025315	Milionario	31062002561
4895	310620025610416	URBANO	310620025306	Bonsucesso	31062002561
4896	310620025610417	URBANO	310620025310	Vila Cemig	31062002561
4897	310620025610418	URBANO	310620025321	Alta TensÒo 2¬ Seþ	31062002561
4898	310620025610419	URBANO	310620025319	Novo das Industrias	31062002561
4899	310620025610420	URBANO	310620025318	Conjunto Bonsucess	31062002561
4900	310620025610421	URBANO	310620025306	Bonsucesso	31062002561
4901	310620025610422	URBANO	310620025307	Bernadete	31062002561
4902	310620025610423	URBANO	310620025347	Diamante	31062002561
4903	310620025610424	URBANO	310620025327	Distrito Industrial d	31062002561
4904	310620025610425	URBANO	310620025330	Vila Independencia	31062002561
4905	310620025610426	URBANO	310620025355	Teixeira Dias	31062002561
4906	310620025610427	URBANO	310620025355	Teixeira Dias	31062002561
4907	310620025610428	URBANO	310620025342	Jardim do Vale	31062002561
4908	310620025610429	URBANO	310620025350	Santa Cecilia	31062002561
4909	310620025610430	URBANO	310620025345	Vila Pinho	31062002561
4910	310620025610431	URBANO	310620025345	Vila Pinho	31062002561
4911	310620025670001	URBANO	310620025158	Vila Madre Gertrud	31062002567
4912	310620025670002	URBANO	310620025157	Vila Madre Gertrud	31062002567
4913	310620025670003	URBANO	310620025154	Madre Gertrudes	31062002567
4914	310620025670004	URBANO	310620025154	Madre Gertrudes	31062002567
4915	310620025670005	URBANO	310620025154	Madre Gertrudes	31062002567

4916	310620025670006	URBANO	310620025159	Camargos	31062002567
4917	310620060640001	URBANO	310620060402	Vista do Sol	31062006064
4918	310620060640002	URBANO	310620060402	Vista do Sol	31062006064
4919	310620060640003	URBANO	310620060402	Vista do Sol	31062006064
4920	310620060640004	URBANO	310620060401	Nazare	31062006064
4921	310620060640005	URBANO	310620060401	Nazare	31062006064
4922	310620060640006	URBANO	310620060401	Nazare	31062006064
4923	310620060640007	URBANO	310620060404	Acaiaca	31062006064
4924	310620060640008	URBANO	310620060405	Paulo VI	31062006064
4925	310620060640009	URBANO	310620060402	Vista do Sol	31062006064
4926	310620060640010	URBANO	310620060401	Nazare	31062006064
4927	310620060640011	URBANO	310620060401	Nazare	31062006064
4928	310620060640012	URBANO	310620060401	Nazare	31062006064
4929	310620060640013	URBANO	310620060401	Nazare	31062006064
4930	310620060640014	URBANO	310620060400	Belmonte	31062006064
4931	310620060640015	URBANO	310620060400	Belmonte	31062006064
4932	310620060640016	URBANO	310620060407	Ribeiro de Abreu	31062006064
4933	310620060640017	URBANO	310620060405	Paulo VI	31062006064
4934	310620060640018	URBANO	310620060405	Paulo VI	31062006064
4935	310620060640019	URBANO	310620060387	Jardim Vitoria	31062006064
4936	310620060640020	URBANO	310620060388	Vila Maria	31062006064
4937	310620060640021	URBANO	310620060388	Vila Maria	31062006064
4938	310620060640022	URBANO	310620060388	Vila Maria	31062006064
4939	310620060640023	URBANO	310620060388	Vila Maria	31062006064
4940	310620060640024	URBANO	310620060387	Jardim Vitoria	31062006064
4941	310620060640025	URBANO	310620060387	Jardim Vitoria	31062006064
4942	310620060640026	URBANO	310620060385	Pousada Santo Antc	31062006064
4943	310620060640027	URBANO	310620060380	Vila da Luz	31062006064
4944	310620060640028	URBANO	310620060376	Goiania	31062006064
4945	310620060640029	URBANO	310620060401	Nazare	31062006064
4946	310620060640039	URBANO	310620060406	Beira Linha	31062006064
4947	310620060640040	URBANO	310620060405	Paulo VI	31062006064
4948	310620060640041	URBANO	310620060405	Paulo VI	31062006064
4949	310620060640042	URBANO	310620060387	Jardim Vitoria	31062006064
4950	310620060640043	URBANO	310620060387	Jardim Vitoria	31062006064
4951	310620060640044	URBANO	310620060388	Vila Maria	31062006064
4952	310620060640045	URBANO	310620060395	Dom Silverio	31062006064
4953	310620060640046	URBANO	310620060395	Dom Silverio	31062006064
4954	310620060640047	URBANO	310620060395	Dom Silverio	31062006064
4955	310620060640048	URBANO	310620060390	SÒo Gabriel	31062006064
4956	310620060640049	URBANO	310620060390	SÒo Gabriel	31062006064
4957	310620060640050	URBANO	310620060398	Ouro Minas	31062006064
4958	310620060640051	URBANO	310620060398	Ouro Minas	31062006064
4959	310620060640052	URBANO	310620060407	Ribeiro de Abreu	31062006064
4960	310620060640053	URBANO	310620060407	Ribeiro de Abreu	31062006064
4961	310620060640054	URBANO	310620060407	Ribeiro de Abreu	31062006064
4962	310620060640055	URBANO	310620060407	Ribeiro de Abreu	31062006064
4963	310620060640056	URBANO	310620060407	Ribeiro de Abreu	31062006064
4964	310620060640057	URBANO	310620060387	Jardim Vitoria	31062006064
4965	310620060640058	URBANO	310620060376	Goiania	31062006064

4966	310620060640059	URBANO	310620060376	Goiania	31062006064
4967	310620060640060	URBANO	310620060376	Goiania	31062006064
4968	310620060640061	URBANO	310620060376	Goiania	31062006064
4969	310620060640062	URBANO	310620060376	Goiania	31062006064
4970	310620060640063	URBANO	310620060395	Dom Silverio	31062006064
4971	310620060640064	URBANO	310620060390	SÒo Gabriel	31062006064
4972	310620060640065	URBANO	310620060390	SÒo Gabriel	31062006064
4973	310620060640066	URBANO	310620060390	SÒo Gabriel	31062006064
4974	310620060640067	URBANO	310620060390	SÒo Gabriel	31062006064
4975	310620060640068	URBANO	310620060394	Vila SÒo Gabriel	31062006064
4976	310620060640069	URBANO	310620060407	Ribeiro de Abreu	31062006064
4977	310620060640070	URBANO	310620060407	Ribeiro de Abreu	31062006064
4978	310620060640071	URBANO	310620060407	Ribeiro de Abreu	31062006064
4979	310620060640072	URBANO	310620060407	Ribeiro de Abreu	31062006064
4980	310620060640073	URBANO	310620060412	Conjunto Paulo VI	31062006064
4981	310620060640074	URBANO	310620060412	Conjunto Paulo VI	31062006064
4982	310620060640075	URBANO	310620060412	Conjunto Paulo VI	31062006064
4983	310620060640076	URBANO	310620060387	Jardim Vitoria	31062006064
4984	310620060640077	URBANO	310620060387	Jardim Vitoria	31062006064
4985	310620060640078	URBANO	310620060376	Goiania	31062006064
4986	310620060640079	URBANO	310620060376	Goiania	31062006064
4987	310620060640080	URBANO	310620060376	Goiania	31062006064
4988	310620060640081	URBANO	310620060376	Goiania	31062006064
4989	310620060640082	URBANO	310620060381	Maria Goretti	31062006064
4990	310620060640083	URBANO	310620060381	Maria Goretti	31062006064
4991	310620060640084	URBANO	310620060381	Maria Goretti	31062006064
4992	310620060640085	URBANO	310620060381	Maria Goretti	31062006064
5004	310620060640097	URBANO	310620060381	Maria Goretti	31062006064
5005	310620060640098	URBANO	310620060381	Maria Goretti	31062006064
5006	310620060640099	URBANO	310620060383	Eymard	31062006064
5007	310620060640100	URBANO	310620060383	Eymard	31062006064
5008	310620060640101	URBANO	310620060383	Eymard	31062006064
5009	310620060640102	URBANO	310620060390	SÒo Gabriel	31062006064
5010	310620060640103	URBANO	310620060390	SÒo Gabriel	31062006064
5011	310620060640104	URBANO	310620060390	SÒo Gabriel	31062006064
5012	310620060640105	URBANO	310620060390	SÒo Gabriel	31062006064
5013	310620060640106	URBANO	310620060407	Ribeiro de Abreu	31062006064
5014	310620060640107	URBANO	310620060407	Ribeiro de Abreu	31062006064
5015	310620060640108	URBANO	310620060407	Ribeiro de Abreu	31062006064
5016	310620060640109	URBANO	310620060407	Ribeiro de Abreu	31062006064
5017	310620060640110	URBANO	310620060411	CapitÒo Eduardo	31062006064
5018	310620060640111	URBANO	310620060376	Goiania	31062006064
5019	310620060640112	URBANO	310620060376	Goiania	31062006064
5020	310620060640113	URBANO	310620060376	Goiania	31062006064
5021	310620060640114	URBANO	310620060375	Ipe	31062006064
5022	310620060640115	URBANO	310620060374	SÒo Marcos	31062006064
5023	310620060640116	URBANO	310620060375	Ipe	31062006064
5024	310620060640117	URBANO	310620060382	Piraja	31062006064
5025	310620060640118	URBANO	310620060383	Eymard	31062006064
5026	310620060640119	URBANO	310620060390	SÒo Gabriel	31062006064

5027	310620060640120	URBANO	310620060390	SÒo Gabriel	31062006064
5028	310620060640121	URBANO	310620060413	Conjunto CapitÒo E	31062006064
5029	310620060640122	URBANO	310620060413	Conjunto CapitÒo E	31062006064
5030	310620060640123	URBANO	310620060414	Beija Flor	31062006064
5031	310620060640124	URBANO	310620060375	Ipe	31062006064
5032	310620060640125	URBANO	310620060374	SÒo Marcos	31062006064
5033	310620060640126	URBANO	310620060374	SÒo Marcos	31062006064
5034	310620060640127	URBANO	310620060374	SÒo Marcos	31062006064
5035	310620060640128	URBANO	310620060374	SÒo Marcos	31062006064
5036	310620060640129	URBANO	310620060382	Piraja	31062006064
5037	310620060640130	URBANO	310620060383	Eymard	31062006064
5038	310620060640131	URBANO	310620060404	Acaiaca	31062006064
5039	310620060640132	URBANO	310620060401	Nazare	31062006064
5040	310620060640133	URBANO	310620060401	Nazare	31062006064
5041	310620060640134	URBANO	310620060403	Grotinha	31062006064
5042	310620060640135	URBANO	310620060402	Vista do Sol	31062006064
5043	310620060640136	URBANO	310620060401	Nazare	31062006064
5044	310620060640137	URBANO	310620060402	Vista do Sol	31062006064
5045	310620060640138	URBANO	310620060401	Nazare	31062006064
5046	310620060640139	URBANO	310620060404	Acaiaca	31062006064
5047	310620060640140	URBANO	310620060406	Beira Linha	31062006064
5048	310620060640141	URBANO	310620060401	Nazare	31062006064
5049	310620060640142	URBANO	310620060404	Acaiaca	31062006064
5050	310620060640143	URBANO	310620060401	Nazare	31062006064
5051	310620060640144	URBANO	310620060401	Nazare	31062006064
5052	310620060640145	URBANO	310620060401	Nazare	31062006064
5053	310620060640146	URBANO	310620060395	Dom Silverio	31062006064
5054	310620060640147	URBANO	310620060400	Belmonte	31062006064
5055	310620060640148	URBANO	310620060387	Jardim Vitoria	31062006064
5056	310620060640149	URBANO	310620060388	Vila Maria	31062006064
5057	310620060640150	URBANO	310620060387	Jardim Vitoria	31062006064
5058	310620060640151	URBANO	310620060386	Bela Vitoria	31062006064
5059	310620060640152	URBANO	310620060385	Pousada Santo Antc	31062006064
5060	310620060640153	URBANO	310620060384	Vitoria	31062006064
5061	310620060640154	URBANO	310620060386	Bela Vitoria	31062006064
5062	310620060640155	URBANO	310620060376	Goiania	31062006064
5063	310620060640156	URBANO	310620060395	Dom Silverio	31062006064
5064	310620060640157	URBANO	310620060395	Dom Silverio	31062006064
5065	310620060640158	URBANO	310620060401	Nazare	31062006064
5066	310620060640159	URBANO	310620060395	Dom Silverio	31062006064
5067	310620060640160	URBANO	310620060398	Ouro Minas	31062006064
5068	310620060640161	URBANO	310620060398	Ouro Minas	31062006064
5069	310620060640162	URBANO	310620060408	Antonio Ribeiro de /	31062006064
5070	310620060640163	URBANO	310620060407	Ribeiro de Abreu	31062006064
5071	310620060640164	URBANO	310620060405	Paulo VI	31062006064
5072	310620060640165	URBANO	310620060405	Paulo VI	31062006064
5073	310620060640166	URBANO	310620060405	Paulo VI	31062006064
5074	310620060640167	URBANO	310620060405	Paulo VI	31062006064
5075	310620060640168	URBANO	310620060389	Mirtes	31062006064
5076	310620060640169	URBANO	310620060388	Vila Maria	31062006064

5077	310620060640170	URBANO	310620060390	SÒo Gabriel	31062006064
5078	310620060640171	URBANO	310620060390	SÒo Gabriel	31062006064
5079	310620060640172	URBANO	310620060390	SÒo Gabriel	31062006064
5080	310620060640173	URBANO	310620060408	Antonio Ribeiro de	31062006064
5081	310620060640174	URBANO	310620060407	Ribeiro de Abreu	31062006064
5082	310620060640175	URBANO	310620060379	Guanabara	31062006064
5083	310620060640176	URBANO	310620060376	Goiania	31062006064
5084	310620060640177	URBANO	310620060376	Goiania	31062006064
5085	310620060640178	URBANO	310620060376	Goiania	31062006064
5086	310620060640179	URBANO	310620060390	SÒo Gabriel	31062006064
5087	310620060640180	URBANO	310620060399	Vila Ouro Minas	31062006064
5088	310620060640181	URBANO	310620060408	Antonio Ribeiro de	31062006064
5089	310620060640182	URBANO	310620060407	Ribeiro de Abreu	31062006064
5090	310620060640183	URBANO	310620060407	Ribeiro de Abreu	31062006064
5091	310620060640184	URBANO	310620060406	Beira Linha	31062006064
5092	310620060640185	URBANO	310620060412	Conjunto Paulo VI	31062006064
5093	310620060640186	URBANO	310620060412	Conjunto Paulo VI	31062006064
5094	310620060640187	URBANO	310620060387	Jardim Vitoria	31062006064
5095	310620060640188	URBANO	310620060384	Vitoria	31062006064
5096	310620060640189	URBANO	310620060376	Goiania	31062006064
5097	310620060640190	URBANO	310620060376	Goiania	31062006064
5098	310620060640191	URBANO	310620060376	Goiania	31062006064
5099	310620060640192	URBANO	310620060376	Goiania	31062006064
5100	310620060640193	URBANO	310620060381	Maria Goretti	31062006064
5101	310620060640194	URBANO	310620060383	Eymard	31062006064
5102	310620060640195	URBANO	310620060390	SÒo Gabriel	31062006064
5103	310620060640196	URBANO	310620060393	Vila Esplanada	31062006064
5104	310620060640206	URBANO	310620060374	SÒo Marcos	31062006064
5105	310620060640207	URBANO	310620060374	SÒo Marcos	31062006064
5106	310620060640208	URBANO	310620060383	Eymard	31062006064
5107	310620060640209	URBANO	310620060382	Piraja	31062006064
5108	310620060640210	URBANO	310620060392	Vila SÒo Dimas	31062006064
5109	310620060640211	URBANO	310620060391	Vila SÒo Gabriel Jac	31062006064
5110	310620060640212	URBANO	310620060411	CapitÒo Eduardo	31062006064
5111	310620060640213	URBANO	310620060413	Conjunto CapitÒo E	31062006064
5112	310620060640214	URBANO	310620060374	SÒo Marcos	31062006064
5113	310620060640215	URBANO	310620060374	SÒo Marcos	31062006064
5114	310620060640216	URBANO	310620060374	SÒo Marcos	31062006064
5115	310620060640217	URBANO	310620060082	FernÒo Dias	31062006064
5116	310620060640218	URBANO	310620060382	Piraja	31062006064
5117	310620060640219	URBANO	310620060076	Vila SÒo Paulo	31062006064
5118	310620060640220	URBANO	310620060074	SÒo Paulo	31062006064
5119	310620060640221	URBANO	310620060384	Vitoria	31062006064
5120	310620060640222	URBANO	310620060384	Vitoria	31062006064
5121	310620060640223	URBANO	310620060384	Vitoria	31062006064
5122	310620060640224	URBANO	310620060384	Vitoria	31062006064
5123	310620060640225	URBANO	310620060384	Vitoria	31062006064
5124	310620060640226	URBANO	310620060384	Vitoria	31062006064
5125	310620060660001	URBANO	310620060432	Floramar	31062006066
5126	310620060660002	URBANO	310620060438	Jardim Felicidade	31062006066

5127	310620060660003	URBANO	310620060460	Tupi A	31062006066
5128	310620060660004	URBANO	310620060460	Tupi A	31062006066
5129	310620060660005	URBANO	310620060432	Floramar	31062006066
5130	310620060660006	URBANO	310620060432	Floramar	31062006066
5131	310620060660007	URBANO	310620060432	Floramar	31062006066
5132	310620060660008	URBANO	310620060439	Jardim Guanabara	31062006066
5133	310620060660009	URBANO	310620060439	Jardim Guanabara	31062006066
5134	310620060660010	URBANO	310620060439	Jardim Guanabara	31062006066
5135	310620060660011	URBANO	310620060439	Jardim Guanabara	31062006066
5136	310620060660012	URBANO	310620060439	Jardim Guanabara	31062006066
5137	310620060660013	URBANO	310620060438	Jardim Felicidade	31062006066
5138	310620060660014	URBANO	310620060438	Jardim Felicidade	31062006066
5139	310620060660015	URBANO	310620060438	Jardim Felicidade	31062006066
5140	310620060660016	URBANO	310620060438	Jardim Felicidade	31062006066
5141	310620060660017	URBANO	310620060438	Jardim Felicidade	31062006066
5142	310620060660018	URBANO	310620060438	Jardim Felicidade	31062006066
5143	310620060660019	URBANO	310620060460	Tupi A	31062006066
5144	310620060660020	URBANO	310620060460	Tupi A	31062006066
5145	310620060660021	URBANO	310620060460	Tupi A	31062006066
5146	310620060660022	URBANO	310620060432	Floramar	31062006066
5147	310620060660023	URBANO	310620060432	Floramar	31062006066
5148	310620060660024	URBANO	310620060432	Floramar	31062006066
5149	310620060660025	URBANO	310620060432	Floramar	31062006066
5150	310620060660026	URBANO	310620060432	Floramar	31062006066
5151	310620060660027	URBANO	310620060432	Floramar	31062006066
5152	310620060660028	URBANO	310620060439	Jardim Guanabara	31062006066
5153	310620060660029	URBANO	310620060439	Jardim Guanabara	31062006066
5154	310620060660030	URBANO	310620060439	Jardim Guanabara	31062006066
5155	310620060660031	URBANO	310620060439	Jardim Guanabara	31062006066
5156	310620060660032	URBANO	310620060466	Xodo-Marize	31062006066
5157	310620060660033	URBANO	310620060439	Jardim Guanabara	31062006066
5158	310620060660034	URBANO	310620060439	Jardim Guanabara	31062006066
5159	310620060660035	URBANO	310620060438	Jardim Felicidade	31062006066
5160	310620060660036	URBANO	310620060459	Solimoes	31062006066
5161	310620060660037	URBANO	310620060438	Jardim Felicidade	31062006066
5162	310620060660038	URBANO	310620060438	Jardim Felicidade	31062006066
5163	310620060660039	URBANO	310620060438	Jardim Felicidade	31062006066
5164	310620060660040	URBANO	310620060438	Jardim Felicidade	31062006066
5165	310620060660041	URBANO	310620060434	Granja Werneck	31062006066
5166	310620060660042	URBANO	310620060460	Tupi A	31062006066
5167	310620060660043	URBANO	310620060460	Tupi A	31062006066
5168	310620060660044	URBANO	310620060460	Tupi A	31062006066
5169	310620060660045	URBANO	310620060460	Tupi A	31062006066
5170	310620060660046	URBANO	310620060435	Guarani	31062006066
5171	310620060660047	URBANO	310620060435	Guarani	31062006066
5172	310620060660048	URBANO	310620060435	Guarani	31062006066
5173	310620060660049	URBANO	310620060435	Guarani	31062006066
5174	310620060660050	URBANO	310620060435	Guarani	31062006066
5175	310620060660051	URBANO	310620060435	Guarani	31062006066
5176	310620060660052	URBANO	310620060436	Heliopolis	31062006066

5177	310620060660053	URBANO	310620060436	Heliopolis	31062006066
5178	310620060660054	URBANO	310620060436	Heliopolis	31062006066
5179	310620060660055	URBANO	310620060436	Heliopolis	31062006066
5180	310620060660056	URBANO	310620060436	Heliopolis	31062006066
5181	310620060660057	URBANO	310620060450	Planalto	31062006066
5182	310620060660058	URBANO	310620060450	Planalto	31062006066
5183	310620060660059	URBANO	310620060450	Planalto	31062006066
5184	310620060660060	URBANO	310620060462	Vila Cloris	31062006066
5185	310620060660061	URBANO	310620060439	Jardim Guanabara	31062006066
5186	310620060660062	URBANO	310620060440	Juliana	31062006066
5187	310620060660063	URBANO	310620060444	Mariquinhas	31062006066
5188	310620060660064	URBANO	310620060442	Madri	31062006066
5189	310620060660065	URBANO	310620060434	Granja Werneck	31062006066
5190	310620060660066	URBANO	310620060461	Tupi B	31062006066
5586	310620060690021	URBANO	310620060489	Rio Branco	31062006069
5587	310620060690022	URBANO	310620060489	Rio Branco	31062006069
5588	310620060690023	URBANO	310620060489	Rio Branco	31062006069
5589	310620060690024	URBANO	310620060489	Rio Branco	31062006069
5590	310620060690025	URBANO	310620060489	Rio Branco	31062006069
5591	310620060690026	URBANO	310620060488	Piratininga	31062006069
5592	310620060690027	URBANO	310620060488	Piratininga	31062006069
5593	310620060690028	URBANO	310620060488	Piratininga	31062006069
5594	310620060690029	URBANO	310620060488	Piratininga	31062006069
5595	310620060690030	URBANO	310620060488	Piratininga	31062006069
5596	310620060690031	URBANO	310620060482	Mantiqueira	31062006069
5895	310620060690338	URBANO	310620060470	Cenaculo	31062006069
5896	310620060690339	URBANO	310620060469	Candelaria	31062006069
5897	310620060690340	URBANO	310620060418	Santa Monica	31062006069
5898	310620060690341	URBANO	310620060418	Santa Monica	31062006069
5899	310620060690342	URBANO	310620060477	Jardim Leblon	31062006069
5900	310620060690343	URBANO	310620060468	Apolonia	31062006069
5901	310620060690344	URBANO	310620060488	Piratininga	31062006069
5902	310620060690345	URBANO	310620060488	Piratininga	31062006069
5903	310620060690346	URBANO	310620060478	Lagoa	31062006069
5904	310620060690347	URBANO	310620060478	Lagoa	31062006069
5905	310620060690348	URBANO	310620060482	Mantiqueira	31062006069
5191	310620060660067	URBANO	310620060441	Lajedo	31062006066
5192	310620060660068	URBANO	310620060446	Mirante	31062006066
5193	310620060660069	URBANO	310620060461	Tupi B	31062006066
5194	310620060660070	URBANO	310620060461	Tupi B	31062006066
5195	310620060660071	URBANO	310620060461	Tupi B	31062006066
5196	310620060660072	URBANO	310620060441	Lajedo	31062006066
5197	310620060660073	URBANO	310620060441	Lajedo	31062006066
5198	310620060660074	URBANO	310620060441	Lajedo	31062006066
5199	310620060660075	URBANO	310620060448	Novo AarÒo Reis	31062006066
5200	310620060660076	URBANO	310620060448	Novo AarÒo Reis	31062006066
5201	310620060660077	URBANO	310620060448	Novo AarÒo Reis	31062006066
5202	310620060660078	URBANO	310620060449	Novo Tupi	31062006066
5203	310620060660079	URBANO	310620060461	Tupi B	31062006066
5204	310620060660080	URBANO	310620060461	Tupi B	31062006066

5205	310620060660081	URBANO	310620060461	Tupi B	31062006066
5206	310620060660082	URBANO	310620060461	Tupi B	31062006066
5207	310620060660083	URBANO	310620060461	Tupi B	31062006066
5208	310620060660084	URBANO	310620060448	Novo AarÒo Reis	31062006066
5209	310620060660085	URBANO	310620060448	Novo AarÒo Reis	31062006066
5210	310620060660086	URBANO	310620060461	Tupi B	31062006066
5211	310620060660087	URBANO	310620060435	Guarani	31062006066
5212	310620060660088	URBANO	310620060435	Guarani	31062006066
5213	310620060660089	URBANO	310620060435	Guarani	31062006066
5214	310620060660090	URBANO	310620060456	SÒo Gonþalo	31062006066
5215	310620060660091	URBANO	310620060452	Providencia	31062006066
5216	310620060660092	URBANO	310620060422	AarÒo Reis	31062006066
5217	310620060660093	URBANO	310620060422	AarÒo Reis	31062006066
5218	310620060660094	URBANO	310620060456	SÒo Gonþalo	31062006066
5219	310620060660095	URBANO	310620060456	SÒo Gonþalo	31062006066
5220	310620060660096	URBANO	310620060452	Providencia	31062006066
5221	310620060660097	URBANO	310620060452	Providencia	31062006066
5222	310620060660098	URBANO	310620060452	Providencia	31062006066
5223	310620060660099	URBANO	310620060445	Minaslandia	31062006066
5224	310620060660100	URBANO	310620060445	Minaslandia	31062006066
5225	310620060660101	URBANO	310620060452	Providencia	31062006066
5226	310620060660102	URBANO	310620060452	Providencia	31062006066
5227	310620060660103	URBANO	310620060452	Providencia	31062006066
5228	310620060660104	URBANO	310620060452	Providencia	31062006066
5229	310620060660105	URBANO	310620060463	Vila Minaslandia	31062006066
5230	310620060660106	URBANO	310620060451	Primeiro de Maio	31062006066
5231	310620060660107	URBANO	310620060451	Primeiro de Maio	31062006066
5232	310620060660108	URBANO	310620060465	Vila Primeiro de Ma	31062006066
5233	310620060660109	URBANO	310620060465	Vila Primeiro de Ma	31062006066
5234	310620060660110	URBANO	310620060465	Vila Primeiro de Ma	31062006066
5235	310620060660111	URBANO	310620060465	Vila Primeiro de Ma	31062006066
5236	310620060660112	URBANO	310620060451	Primeiro de Maio	31062006066
5237	310620060660113	URBANO	310620060445	Minaslandia	31062006066
5238	310620060660114	URBANO	310620060445	Minaslandia	31062006066
5239	310620060660115	URBANO	310620060445	Minaslandia	31062006066
5240	310620060660116	URBANO	310620060445	Minaslandia	31062006066
5241	310620060660117	URBANO	310620060454	SÒo Bernardo	31062006066
5242	310620060660118	URBANO	310620060454	SÒo Bernardo	31062006066
5243	310620060660119	URBANO	310620060454	SÒo Bernardo	31062006066
5244	310620060660120	URBANO	310620060454	SÒo Bernardo	31062006066
5245	310620060660121	URBANO	310620060454	SÒo Bernardo	31062006066
5246	310620060660122	URBANO	310620060454	SÒo Bernardo	31062006066
5247	310620060660123	URBANO	310620060454	SÒo Bernardo	31062006066
5248	310620060660124	URBANO	310620060454	SÒo Bernardo	31062006066
5249	310620060660125	URBANO	310620060454	SÒo Bernardo	31062006066
5250	310620060660126	URBANO	310620060436	Heliopolis	31062006066
5251	310620060660127	URBANO	310620060450	Planalto	31062006066
5252	310620060660128	URBANO	310620060450	Planalto	31062006066
5253	310620060660129	URBANO	310620060450	Planalto	31062006066
5254	310620060660130	URBANO	310620060454	SÒo Bernardo	31062006066

5255	310620060660131	URBANO	310620060419	SÒo Tomaz	31062006066
5256	310620060660132	URBANO	310620060419	SÒo Tomaz	31062006066
5257	310620060660133	URBANO	310620060419	SÒo Tomaz	31062006066
5258	310620060660134	URBANO	310620060419	SÒo Tomaz	31062006066
5259	310620060660135	URBANO	310620060419	SÒo Tomaz	31062006066
5260	310620060660136	URBANO	310620060419	SÒo Tomaz	31062006066
5261	310620060660137	URBANO	310620060419	SÒo Tomaz	31062006066
5262	310620060660138	URBANO	310620060419	SÒo Tomaz	31062006066
5263	310620060660139	URBANO	310620060450	Planalto	31062006066
5264	310620060660140	URBANO	310620060450	Planalto	31062006066
5265	310620060660141	URBANO	310620060450	Planalto	31062006066
5266	310620060660142	URBANO	310620060450	Planalto	31062006066
5267	310620060660143	URBANO	310620060450	Planalto	31062006066
5268	310620060660144	URBANO	310620060450	Planalto	31062006066
5269	310620060660145	URBANO	310620060450	Planalto	31062006066
5270	310620060660146	URBANO	310620060427	Campo Alegre	31062006066
5271	310620060660147	URBANO	310620060450	Planalto	31062006066
5272	310620060660148	URBANO	310620060427	Campo Alegre	31062006066
5273	310620060660149	URBANO	310620060450	Planalto	31062006066
5274	310620060660150	URBANO	310620060450	Planalto	31062006066
5275	310620060660151	URBANO	310620060450	Planalto	31062006066
5276	310620060660152	URBANO	310620060450	Planalto	31062006066
5277	310620060660153	URBANO	310620060462	Vila Cloris	31062006066
5278	310620060660154	URBANO	310620060462	Vila Cloris	31062006066
5279	310620060660155	URBANO	310620060462	Vila Cloris	31062006066
5280	310620060660156	URBANO	310620060462	Vila Cloris	31062006066
5281	310620060660157	URBANO	310620060462	Vila Cloris	31062006066
5282	310620060660158	URBANO	310620060450	Planalto	31062006066
5283	310620060660159	URBANO	310620060462	Vila Cloris	31062006066
5284	310620060660160	URBANO	310620060462	Vila Cloris	31062006066
5285	310620060660161	URBANO	310620060440	Juliana	31062006066
5286	310620060660162	URBANO	310620060444	Mariquinhas	31062006066
5287	310620060660163	URBANO	310620060440	Juliana	31062006066
5288	310620060660164	URBANO	310620060431	Etelvina Carneiro	31062006066
5289	310620060660165	URBANO	310620060431	Etelvina Carneiro	31062006066
5290	310620060660166	URBANO	310620060437	Jaqueline	31062006066
5291	310620060660167	URBANO	310620060437	Jaqueline	31062006066
5292	310620060660168	URBANO	310620060433	Frei Leopoldo	31062006066
5293	310620060660169	URBANO	310620060437	Jaqueline	31062006066
5294	310620060660170	URBANO	310620060437	Jaqueline	31062006066
5295	310620060660171	URBANO	310620060464	Vila Nova	31062006066
5296	310620060660172	URBANO	310620060428	Canaa	31062006066
5297	310620060660173	URBANO	310620060428	Canaa	31062006066
5298	310620060660174	URBANO	310620060437	Jaqueline	31062006066
5299	310620060660175	URBANO	310620060437	Jaqueline	31062006066
5300	310620060660176	URBANO	310620060437	Jaqueline	31062006066
5301	310620060660177	URBANO	310620060437	Jaqueline	31062006066
5302	310620060660178	URBANO	310620060437	Jaqueline	31062006066
5303	310620060660179	URBANO	310620060437	Jaqueline	31062006066
5304	310620060660180	URBANO	310620060437	Jaqueline	31062006066

5305	310620060660181	URBANO	310620060437	Jaqueline	31062006066
5306	310620060660182	URBANO	310620060437	Jaqueline	31062006066
5307	310620060660183	URBANO	310620060437	Jaqueline	31062006066
5308	310620060660184	URBANO	310620060467	Zilah Sposito	31062006066
5309	310620060660185	URBANO	310620060467	Zilah Sposito	31062006066
5310	310620060660186	URBANO	310620060434	Granja Werneck	31062006066
5311	310620060660187	URBANO	310620060407	Ribeiro de Abreu	31062006066
5312	310620060660188	URBANO	310620060434	Granja Werneck	31062006066
5313	310620060660189	URBANO	310620060447	Monte Azul	31062006066
5314	310620060660190	URBANO	310620060447	Monte Azul	31062006066
5315	310620060660191	URBANO	310620060447	Monte Azul	31062006066
5316	310620060660192	URBANO	310620060443	Maria Tereza	31062006066
5317	310620060660193	URBANO	310620060449	Novo Tupi	31062006066
5318	310620060660194	URBANO	310620060440	Juliana	31062006066
5319	310620060660195	URBANO	310620060458	Satelite	31062006066
5320	310620060660196	URBANO	310620060462	Vila Cloris	31062006066
5321	310620060660197	URBANO	310620060462	Vila Cloris	31062006066
5322	310620060660198	URBANO	310620060427	Campo Alegre	31062006066
5323	310620060660199	URBANO	310620060427	Campo Alegre	31062006066
5324	310620060660200	URBANO	310620060424	Biquinhas	31062006066
5325	310620060660201	URBANO	310620060432	Floramar	31062006066
5326	310620060660202	URBANO	310620060439	Jardim Guanabara	31062006066
5327	310620060660203	URBANO	310620060439	Jardim Guanabara	31062006066
5328	310620060660204	URBANO	310620060424	Biquinhas	31062006066
5329	310620060660205	URBANO	310620060462	Vila Cloris	31062006066
5330	310620060660206	URBANO	310620060429	Conjunto Floramar	31062006066
5331	310620060660207	URBANO	310620060433	Frei Leopoldo	31062006066
5332	310620060660208	URBANO	310620060437	Jaqueline	31062006066
5333	310620060660209	URBANO	310620060442	Madri	31062006066
5334	310620060660210	URBANO	310620060440	Juliana	31062006066
5335	310620060660211	URBANO	310620060424	Biquinhas	31062006066
5336	310620060660212	URBANO	310620060431	Etelvina Carneiro	31062006066
5337	310620060660213	URBANO	310620060440	Juliana	31062006066
5338	310620060660214	URBANO	310620060444	Mariquinhas	31062006066
5339	310620060660215	URBANO	310620060440	Juliana	31062006066
5340	310620060660216	URBANO	310620060419	SÒo Tomaz	31062006066
5341	310620060660217	URBANO	310620060459	Solimoes	31062006066
5342	310620060660218	URBANO	310620060438	Jardim Felicidade	31062006066
5343	310620060660219	URBANO	310620060460	Tupi A	31062006066
5344	310620060660220	URBANO	310620060437	Jaqueline	31062006066
5345	310620060660221	URBANO	310620060460	Tupi A	31062006066
5346	310620060660312	URBANO	310620060438	Jardim Felicidade	31062006066
5347	310620060660313	URBANO	310620060423	Bacurau	31062006066
5348	310620060660314	URBANO	310620060460	Tupi A	31062006066
5349	310620060660315	URBANO	310620060460	Tupi A	31062006066
5350	310620060660316	URBANO	310620060407	Ribeiro de Abreu	31062006066
5351	310620060660317	URBANO	310620060435	Guarani	31062006066
5352	310620060660318	URBANO	310620060445	Minaslandia	31062006066
5353	310620060660319	URBANO	310620060436	Heliopolis	31062006066
5354	310620060660320	URBANO	310620060452	Providencia	31062006066

5355	310620060690123	URBANO	310620060365	Serra Verde	31062006069
5356	310620060690124	URBANO	310620060471	Conjunto Minas Cai	31062006069
5357	310620060690125	URBANO	310620060471	Conjunto Minas Cai	31062006069
5358	310620060690126	URBANO	310620060471	Conjunto Minas Cai	31062006069
5359	310620060690127	URBANO	310620060470	Cenaculo	31062006069
5360	310620060690128	URBANO	310620060484	Minas Caixa	31062006069
5361	310620060690129	URBANO	310620060484	Minas Caixa	31062006069
5362	310620060690130	URBANO	310620060484	Minas Caixa	31062006069
5407	310620060660222	URBANO	310620060431	Etelvina Carneiro	31062006066
5408	310620060660223	URBANO	310620060431	Etelvina Carneiro	31062006066
5409	310620060660224	URBANO	310620060424	Biquinhas	31062006066
5410	310620060660225	URBANO	310620060424	Biquinhas	31062006066
5411	310620060660226	URBANO	310620060454	SÒo Bernardo	31062006066
5412	310620060660227	URBANO	310620060450	Planalto	31062006066
5413	310620060660228	URBANO	310620060454	SÒo Bernardo	31062006066
5414	310620060660229	URBANO	310620060288	Vila Aeroporto	31062006066
5415	310620060660230	URBANO	310620060422	AarÒo Reis	31062006066
5416	310620060660231	URBANO	310620060456	SÒo Gonþalo	31062006066
5417	310620060660232	URBANO	310620060430	Conjunto Providenc	31062006066
5418	310620060660233	URBANO	310620060452	Providencia	31062006066
5419	310620060660234	URBANO	310620060452	Providencia	31062006066
5420	310620060660235	URBANO	310620060451	Primeiro de Maio	31062006066
5421	310620060660236	URBANO	310620060451	Primeiro de Maio	31062006066
5422	310620060660237	URBANO	310620060452	Providencia	31062006066
5423	310620060660238	URBANO	310620060426	Boa UniÒo 2¬ SeþÒ	31062006066
5424	310620060660239	URBANO	310620060425	Boa UniÒo 1¬ SeþÒ	31062006066
5425	310620060660240	URBANO	310620060422	AarÒo Reis	31062006066
5426	310620060660241	URBANO	310620060460	Tupi A	31062006066
5427	310620060660242	URBANO	310620060461	Tupi B	31062006066
5428	310620060660243	URBANO	310620060449	Novo Tupi	31062006066
5429	310620060660244	URBANO	310620060461	Tupi B	31062006066
5430	310620060660245	URBANO	310620060461	Tupi B	31062006066
5431	310620060660246	URBANO	310620060461	Tupi B	31062006066
5432	310620060660247	URBANO	310620060441	Lajedo	31062006066
5433	310620060660248	URBANO	310620060449	Novo Tupi	31062006066
5434	310620060660249	URBANO	310620060447	Monte Azul	31062006066
5435	310620060660250	URBANO	310620060449	Novo Tupi	31062006066
5436	310620060660251	URBANO	310620060465	Vila Primeiro de Ma	31062006066
5437	310620060660252	URBANO	310620060460	Tupi A	31062006066
5438	310620060660253	URBANO	310620060437	Jaqueline	31062006066
5439	310620060660254	URBANO	310620060432	Floramar	31062006066
5440	310620060660255	URBANO	310620060439	Jardim Guanabara	31062006066
5441	310620060660256	URBANO	310620060450	Planalto	31062006066
5442	310620060660257	URBANO	310620060428	Canaa	31062006066
5443	310620060660258	URBANO	310620060438	Jardim Felicidade	31062006066
5444	310620060660259	URBANO	310620060460	Tupi A	31062006066
5445	310620060660260	URBANO	310620060460	Tupi A	31062006066
5446	310620060660261	URBANO	310620060432	Floramar	31062006066
5447	310620060660262	URBANO	310620060432	Floramar	31062006066
5448	310620060660263	URBANO	310620060432	Floramar	31062006066

5449	310620060660264	URBANO	310620060438	Jardim Felicidade	31062006066
5450	310620060660265	URBANO	310620060450	Planalto	31062006066
5451	310620060660266	URBANO	310620060450	Planalto	31062006066
5452	310620060660267	URBANO	310620060440	Juliana	31062006066
5453	310620060660268	URBANO	310620060440	Juliana	31062006066
5454	310620060660269	URBANO	310620060440	Juliana	31062006066
5455	310620060660270	URBANO	310620060440	Juliana	31062006066
5456	310620060660271	URBANO	310620060461	Tupi B	31062006066
5457	310620060660272	URBANO	310620060461	Tupi B	31062006066
5458	310620060660273	URBANO	310620060448	Novo AarÒo Reis	31062006066
5459	310620060660274	URBANO	310620060448	Novo AarÒo Reis	31062006066
5460	310620060660275	URBANO	310620060448	Novo AarÒo Reis	31062006066
5461	310620060660276	URBANO	310620060461	Tupi B	31062006066
5462	310620060660277	URBANO	310620060439	Jardim Guanabara	31062006066
5463	310620060660278	URBANO	310620060452	Providencia	31062006066
5464	310620060660279	URBANO	310620060463	Vila Minaslandia	31062006066
5465	310620060660280	URBANO	310620060435	Guarani	31062006066
5466	310620060660281	URBANO	310620060436	Heliopolis	31062006066
5467	310620060660282	URBANO	310620060436	Heliopolis	31062006066
5468	310620060660283	URBANO	310620060450	Planalto	31062006066
5469	310620060660284	URBANO	310620060452	Providencia	31062006066
5470	310620060660285	URBANO	310620060452	Providencia	31062006066
5471	310620060660286	URBANO	310620060437	Jaqueline	31062006066
5472	310620060660287	URBANO	310620060445	Minaslandia	31062006066
5473	310620060660288	URBANO	310620060445	Minaslandia	31062006066
5474	310620060660289	URBANO	310620060454	SÒo Bernardo	31062006066
5475	310620060660290	URBANO	310620060419	SÒo Tomaz	31062006066
5476	310620060660291	URBANO	310620060419	SÒo Tomaz	31062006066
5477	310620060660292	URBANO	310620060419	SÒo Tomaz	31062006066
5478	310620060660293	URBANO	310620060450	Planalto	31062006066
5479	310620060660294	URBANO	310620060437	Jaqueline	31062006066
5480	310620060660295	URBANO	310620060450	Planalto	31062006066
5481	310620060660296	URBANO	310620060450	Planalto	31062006066
5482	310620060660297	URBANO	310620060455	SÒo DamiÒo	31062006066
5483	310620060660298	URBANO	310620060467	Zilah Sposito	31062006066
5484	310620060660299	URBANO	310620060434	Granja Werneck	31062006066
5485	310620060660300	URBANO	310620060434	Granja Werneck	31062006066
5486	310620060660301	URBANO	310620060434	Granja Werneck	31062006066
5487	310620060660302	URBANO	310620060434	Granja Werneck	31062006066
5488	310620060660303	URBANO	310620060407	Ribeiro de Abreu	31062006066
5489	310620060660304	URBANO	310620060461	Tupi B	31062006066
5490	310620060660305	URBANO	310620060461	Tupi B	31062006066
5491	310620060660306	URBANO	310620060440	Juliana	31062006066
5492	310620060660307	URBANO	310620060466	Xodo-Marize	31062006066
5493	310620060660308	URBANO	310620060440	Juliana	31062006066
5494	310620060660309	URBANO	310620060438	Jardim Felicidade	31062006066
5495	310620060660310	URBANO	310620060439	Jardim Guanabara	31062006066
5496	310620060660311	URBANO	310620060466	Xodo-Marize	31062006066
5497	310620060660321	URBANO	310620060435	Guarani	31062006066
5498	310620060660322	URBANO	310620060422	AarÒo Reis	31062006066

5499	310620060660323	URBANO	310620060466	Xodo-Marize	31062006066
5500	310620060660324	URBANO	310620060411	CapitÒo Eduardo	31062006066
5501	310620060680001	URBANO	310620060417	Santa Branca	31062006068
5502	310620060680002	URBANO	310620060416	Santa Amelia	31062006068
5503	310620060680003	URBANO	310620060416	Santa Amelia	31062006068
5504	310620060680004	URBANO	310620060416	Santa Amelia	31062006068
5505	310620060680005	URBANO	310620060416	Santa Amelia	31062006068
5506	310620060680006	URBANO	310620060416	Santa Amelia	31062006068
5507	310620060680007	URBANO	310620060417	Santa Branca	31062006068
5508	310620060680008	URBANO	310620060417	Santa Branca	31062006068
5509	310620060680009	URBANO	310620060417	Santa Branca	31062006068
5510	310620060680010	URBANO	310620060415	Itapoa	31062006068
5511	310620060680011	URBANO	310620060415	Itapoa	31062006068
5512	310620060680012	URBANO	310620060416	Santa Amelia	31062006068
5513	310620060680013	URBANO	310620060272	Jardim Atlantico	31062006068
5514	310620060680014	URBANO	310620060266	Copacabana	31062006068
5515	310620060680015	URBANO	310620060416	Santa Amelia	31062006068
5516	310620060680016	URBANO	310620060416	Santa Amelia	31062006068
5517	310620060680017	URBANO	310620060416	Santa Amelia	31062006068
5518	310620060680018	URBANO	310620060416	Santa Amelia	31062006068
5519	310620060680019	URBANO	310620060416	Santa Amelia	31062006068
5520	310620060680020	URBANO	310620060416	Santa Amelia	31062006068
5521	310620060680021	URBANO	310620060416	Santa Amelia	31062006068
5522	310620060680022	URBANO	310620060418	Santa Monica	31062006068
5523	310620060680023	URBANO	310620060418	Santa Monica	31062006068
5524	310620060680024	URBANO	310620060418	Santa Monica	31062006068
5525	310620060680025	URBANO	310620060418	Santa Monica	31062006068
5526	310620060680026	URBANO	310620060415	Itapoa	31062006068
5527	310620060680027	URBANO	310620060415	Itapoa	31062006068
5528	310620060680028	URBANO	310620060415	Itapoa	31062006068
5529	310620060680029	URBANO	310620060415	Itapoa	31062006068
5530	310620060680030	URBANO	310620060415	Itapoa	31062006068
5531	310620060680031	URBANO	310620060415	Itapoa	31062006068
5532	310620060680032	URBANO	310620060415	Itapoa	31062006068
5533	310620060680033	URBANO	310620060415	Itapoa	31062006068
5534	310620060680034	URBANO	310620060419	SÒo Tomaz	31062006068
5535	310620060680035	URBANO	310620060416	Santa Amelia	31062006068
5536	310620060680036	URBANO	310620060416	Santa Amelia	31062006068
5537	310620060680037	URBANO	310620060416	Santa Amelia	31062006068
5538	310620060680038	URBANO	310620060417	Santa Branca	31062006068
5539	310620060680039	URBANO	310620060416	Santa Amelia	31062006068
5540	310620060680040	URBANO	310620060417	Santa Branca	31062006068
5541	310620060680041	URBANO	310620060417	Santa Branca	31062006068
5542	310620060680042	URBANO	310620060417	Santa Branca	31062006068
5543	310620060680043	URBANO	310620060417	Santa Branca	31062006068
5544	310620060680044	URBANO	310620060415	Itapoa	31062006068
5545	310620060680045	URBANO	310620060415	Itapoa	31062006068
5546	310620060680046	URBANO	310620060272	Jardim Atlantico	31062006068
5547	310620060680047	URBANO	310620060416	Santa Amelia	31062006068
5548	310620060680048	URBANO	310620060266	Copacabana	31062006068

5549	310620060680049	URBANO	310620060266	Copacabana	31062006068
5550	310620060680050	URBANO	310620060416	Santa Amelia	31062006068
5551	310620060680051	URBANO	310620060416	Santa Amelia	31062006068
5552	310620060680052	URBANO	310620060416	Santa Amelia	31062006068
5553	310620060680053	URBANO	310620060416	Santa Amelia	31062006068
5554	310620060680054	URBANO	310620060420	Unidas	31062006068
5555	310620060680055	URBANO	310620060421	Universo	31062006068
5556	310620060680056	URBANO	310620060418	Santa Monica	31062006068
5557	310620060680057	URBANO	310620060417	Santa Branca	31062006068
5558	310620060680058	URBANO	310620060418	Santa Monica	31062006068
5559	310620060680059	URBANO	310620060417	Santa Branca	31062006068
5560	310620060680060	URBANO	310620060417	Santa Branca	31062006068
5561	310620060680061	URBANO	310620060417	Santa Branca	31062006068
5562	310620060680062	URBANO	310620060415	Itapoa	31062006068
5563	310620060680063	URBANO	310620060415	Itapoa	31062006068
5564	310620060680064	URBANO	310620060272	Jardim Atlantico	31062006068
5565	310620060680065	URBANO	310620060419	SÒo Tomaz	31062006068
5566	310620060690001	URBANO	310620060481	Leticia	31062006069
5567	310620060690002	URBANO	310620060481	Leticia	31062006069
5568	310620060690003	URBANO	310620060481	Leticia	31062006069
5569	310620060690004	URBANO	310620060481	Leticia	31062006069
5570	310620060690005	URBANO	310620060481	Leticia	31062006069
5571	310620060690006	URBANO	310620060481	Leticia	31062006069
5572	310620060690007	URBANO	310620060481	Leticia	31062006069
5573	310620060690008	URBANO	310620060489	Rio Branco	31062006069
5574	310620060690009	URBANO	310620060489	Rio Branco	31062006069
5575	310620060690010	URBANO	310620060488	Piratininga	31062006069
5576	310620060690011	URBANO	310620060488	Piratininga	31062006069
5577	310620060690012	URBANO	310620060488	Piratininga	31062006069
5578	310620060690013	URBANO	310620060488	Piratininga	31062006069
5579	310620060690014	URBANO	310620060481	Leticia	31062006069
5580	310620060690015	URBANO	310620060481	Leticia	31062006069
5581	310620060690016	URBANO	310620060469	Candelaria	31062006069
5582	310620060690017	URBANO	310620060469	Candelaria	31062006069
5583	310620060690018	URBANO	310620060469	Candelaria	31062006069
5584	310620060690019	URBANO	310620060489	Rio Branco	31062006069
5585	310620060690020	URBANO	310620060489	Rio Branco	31062006069
5597	310620060690032	URBANO	310620060481	Leticia	31062006069
5598	310620060690033	URBANO	310620060469	Candelaria	31062006069
5599	310620060690034	URBANO	310620060489	Rio Branco	31062006069
5600	310620060690035	URBANO	310620060489	Rio Branco	31062006069
5601	310620060690036	URBANO	310620060489	Rio Branco	31062006069
5602	310620060690037	URBANO	310620060489	Rio Branco	31062006069
5603	310620060690038	URBANO	310620060488	Piratininga	31062006069
5604	310620060690039	URBANO	310620060488	Piratininga	31062006069
5605	310620060690040	URBANO	310620060488	Piratininga	31062006069
5606	310620060690041	URBANO	310620060488	Piratininga	31062006069
5607	310620060690042	URBANO	310620060488	Piratininga	31062006069
5608	310620060690043	URBANO	310620060488	Piratininga	31062006069
5609	310620060690044	URBANO	310620060479	Lagoinha Leblon	31062006069

5610	310620060690045	URBANO	310620060479	Lagoinha Leblon	31062006069
5611	310620060690046	URBANO	310620060479	Lagoinha Leblon	31062006069
5612	310620060690047	URBANO	310620060483	Maria Helena	31062006069
5613	310620060690048	URBANO	310620060482	Mantiqueira	31062006069
5614	310620060690049	URBANO	310620060482	Mantiqueira	31062006069
5615	310620060690050	URBANO	310620060482	Mantiqueira	31062006069
5616	310620060690051	URBANO	310620060482	Mantiqueira	31062006069
5617	310620060690052	URBANO	310620060482	Mantiqueira	31062006069
5618	310620060690053	URBANO	310620060482	Mantiqueira	31062006069
5619	310620060690054	URBANO	310620060476	Jardim dos Comerci	31062006069
5620	310620060690055	URBANO	310620060476	Jardim dos Comerci	31062006069
5621	310620060690056	URBANO	310620060476	Jardim dos Comerci	31062006069
5622	310620060690057	URBANO	310620060474	Europa	31062006069
5623	310620060690058	URBANO	310620060474	Europa	31062006069
5624	310620060690059	URBANO	310620060474	Europa	31062006069
5625	310620060690060	URBANO	310620060470	Cenaculo	31062006069
5626	310620060690061	URBANO	310620060469	Candelaria	31062006069
5627	310620060690062	URBANO	310620060469	Candelaria	31062006069
5628	310620060690063	URBANO	310620060469	Candelaria	31062006069
5629	310620060690064	URBANO	310620060485	SÒo JoÒo Batista	31062006069
5630	310620060690065	URBANO	310620060485	SÒo JoÒo Batista	31062006069
5631	310620060690066	URBANO	310620060418	Santa Monica	31062006069
5632	310620060690067	URBANO	310620060418	Santa Monica	31062006069
5633	310620060690068	URBANO	310620060418	Santa Monica	31062006069
5634	310620060690069	URBANO	310620060418	Santa Monica	31062006069
5635	310620060690070	URBANO	310620060418	Santa Monica	31062006069
5636	310620060690071	URBANO	310620060418	Santa Monica	31062006069
5637	310620060690072	URBANO	310620060418	Santa Monica	31062006069
5638	310620060690073	URBANO	310620060489	Rio Branco	31062006069
5639	310620060690074	URBANO	310620060418	Santa Monica	31062006069
5640	310620060690075	URBANO	310620060418	Santa Monica	31062006069
5641	310620060690076	URBANO	310620060477	Jardim Leblon	31062006069
5642	310620060690077	URBANO	310620060366	Varzea da Palma	31062006069
5643	310620060690078	URBANO	310620060477	Jardim Leblon	31062006069
5644	310620060690079	URBANO	310620060477	Jardim Leblon	31062006069
5645	310620060690080	URBANO	310620060477	Jardim Leblon	31062006069
5646	310620060690081	URBANO	310620060468	Apolonia	31062006069
5647	310620060690082	URBANO	310620060468	Apolonia	31062006069
5648	310620060690083	URBANO	310620060477	Jardim Leblon	31062006069
5649	310620060690084	URBANO	310620060477	Jardim Leblon	31062006069
5650	310620060690085	URBANO	310620060468	Apolonia	31062006069
5651	310620060690086	URBANO	310620060477	Jardim Leblon	31062006069
5652	310620060690087	URBANO	310620060477	Jardim Leblon	31062006069
5653	310620060690088	URBANO	310620060477	Jardim Leblon	31062006069
5654	310620060690089	URBANO	310620060190	CÚu Azul	31062006069
5655	310620060690090	URBANO	310620060490	Vila Santa Monica 1	31062006069
5656	310620060690091	URBANO	310620060488	Piratininga	31062006069
5657	310620060690092	URBANO	310620060190	CÚu Azul	31062006069
5658	310620060690093	URBANO	310620060488	Piratininga	31062006069
5659	310620060690094	URBANO	310620060190	CÚu Azul	31062006069

5660	310620060690095	URBANO	310620060478	Lagoa	31062006069
5661	310620060690096	URBANO	310620060478	Lagoa	31062006069
5662	310620060690097	URBANO	310620060478	Lagoa	31062006069
5663	310620060690098	URBANO	310620060479	Lagoinha Leblon	31062006069
5664	310620060690099	URBANO	310620060478	Lagoa	31062006069
5665	310620060690100	URBANO	310620060478	Lagoa	31062006069
5666	310620060690101	URBANO	310620060479	Lagoinha Leblon	31062006069
5667	310620060690102	URBANO	310620060479	Lagoinha Leblon	31062006069
5668	310620060690103	URBANO	310620060483	Maria Helena	31062006069
5669	310620060690104	URBANO	310620060483	Maria Helena	31062006069
5670	310620060690105	URBANO	310620060483	Maria Helena	31062006069
5671	310620060690106	URBANO	310620060483	Maria Helena	31062006069
5672	310620060690107	URBANO	310620060483	Maria Helena	31062006069
5673	310620060690108	URBANO	310620060483	Maria Helena	31062006069
5674	310620060690109	URBANO	310620060483	Maria Helena	31062006069
5675	310620060690110	URBANO	310620060482	Mantiqueira	31062006069
5676	310620060690111	URBANO	310620060482	Mantiqueira	31062006069
5677	310620060690112	URBANO	310620060482	Mantiqueira	31062006069
5678	310620060690113	URBANO	310620060482	Mantiqueira	31062006069
5679	310620060690114	URBANO	310620060476	Jardim dos Comerci	31062006069
5680	310620060690115	URBANO	310620060476	Jardim dos Comerci	31062006069
5681	310620060690116	URBANO	310620060476	Jardim dos Comerci	31062006069
5682	310620060690117	URBANO	310620060476	Jardim dos Comerci	31062006069
5683	310620060690118	URBANO	310620060474	Europa	31062006069
5684	310620060690119	URBANO	310620060474	Europa	31062006069
5685	310620060690120	URBANO	310620060474	Europa	31062006069
5686	310620060690121	URBANO	310620060474	Europa	31062006069
5687	310620060690122	URBANO	310620060474	Europa	31062006069
5688	310620060690131	URBANO	310620060367	Venda Nova	31062006069
5689	310620060690132	URBANO	310620060469	Candelaria	31062006069
5690	310620060690133	URBANO	310620060469	Candelaria	31062006069
5691	310620060690134	URBANO	310620060485	SÒo JoÒo Batista	31062006069
5692	310620060690135	URBANO	310620060367	Venda Nova	31062006069
5693	310620060690136	URBANO	310620060485	SÒo JoÒo Batista	31062006069
5694	310620060690137	URBANO	310620060372	Vila SÒo JoÒo Batist	31062006069
5695	310620060690138	URBANO	310620060372	Vila SÒo JoÒo Batist	31062006069
5696	310620060690139	URBANO	310620060372	Vila SÒo JoÒo Batist	31062006069
5697	310620060690140	URBANO	310620060485	SÒo JoÒo Batista	31062006069
5698	310620060690141	URBANO	310620060485	SÒo JoÒo Batista	31062006069
5699	310620060690142	URBANO	310620060493	Nossa Senhora Apa	31062006069
5700	310620060690143	URBANO	310620060493	Nossa Senhora Apa	31062006069
5701	310620060690144	URBANO	310620060493	Nossa Senhora Apa	31062006069
5702	310620060690145	URBANO	310620060418	Santa Monica	31062006069
5703	310620060690146	URBANO	310620060485	SÒo JoÒo Batista	31062006069
5704	310620060690147	URBANO	310620060485	SÒo JoÒo Batista	31062006069
5705	310620060690148	URBANO	310620060418	Santa Monica	31062006069
5706	310620060690149	URBANO	310620060418	Santa Monica	31062006069
5707	310620060690150	URBANO	310620060418	Santa Monica	31062006069
5708	310620060690151	URBANO	310620060418	Santa Monica	31062006069
5709	310620060690152	URBANO	310620060418	Santa Monica	31062006069

5710	310620060690153	URBANO	310620060418	Santa Monica	31062006069
5711	310620060690154	URBANO	310620060418	Santa Monica	31062006069
5712	310620060690155	URBANO	310620060418	Santa Monica	31062006069
5713	310620060690156	URBANO	310620060418	Santa Monica	31062006069
5714	310620060690157	URBANO	310620060418	Santa Monica	31062006069
5715	310620060690158	URBANO	310620060418	Santa Monica	31062006069
5716	310620060690159	URBANO	310620060266	Copacabana	31062006069
5717	310620060690160	URBANO	310620060266	Copacabana	31062006069
5718	310620060690161	URBANO	310620060266	Copacabana	31062006069
5719	310620060690162	URBANO	310620060266	Copacabana	31062006069
5720	310620060690163	URBANO	310620060266	Copacabana	31062006069
5721	310620060690164	URBANO	310620060266	Copacabana	31062006069
5722	310620060690165	URBANO	310620060266	Copacabana	31062006069
5723	310620060690166	URBANO	310620060468	Apolonia	31062006069
5724	310620060690167	URBANO	310620060468	Apolonia	31062006069
5725	310620060690168	URBANO	310620060477	Jardim Leblon	31062006069
5726	310620060690169	URBANO	310620060468	Apolonia	31062006069
5727	310620060690170	URBANO	310620060190	CÚu Azul	31062006069
5728	310620060690171	URBANO	310620060190	CÚu Azul	31062006069
5729	310620060690172	URBANO	310620060190	CÚu Azul	31062006069
5730	310620060690173	URBANO	310620060370	Vila dos Anjos	31062006069
5731	310620060690174	URBANO	310620060190	CÚu Azul	31062006069
5732	310620060690175	URBANO	310620060190	CÚu Azul	31062006069
5733	310620060690176	URBANO	310620060190	CÚu Azul	31062006069
5734	310620060690177	URBANO	310620060190	CÚu Azul	31062006069
5735	310620060690178	URBANO	310620060190	CÚu Azul	31062006069
5736	310620060690179	URBANO	310620060190	CÚu Azul	31062006069
5737	310620060690180	URBANO	310620060190	CÚu Azul	31062006069
5738	310620060690181	URBANO	310620060190	CÚu Azul	31062006069
5739	310620060690182	URBANO	310620060190	CÚu Azul	31062006069
5740	310620060690183	URBANO	310620060190	CÚu Azul	31062006069
5741	310620060690184	URBANO	310620060190	CÚu Azul	31062006069
5742	310620060690185	URBANO	310620060190	CÚu Azul	31062006069
5743	310620060690186	URBANO	310620060190	CÚu Azul	31062006069
5744	310620060690187	URBANO	310620060190	CÚu Azul	31062006069
5745	310620060690188	URBANO	310620060190	CÚu Azul	31062006069
5746	310620060690189	URBANO	310620060478	Lagoa	31062006069
5747	310620060690190	URBANO	310620060478	Lagoa	31062006069
5748	310620060690191	URBANO	310620060478	Lagoa	31062006069
5749	310620060690192	URBANO	310620060482	Mantiqueira	31062006069
5750	310620060690193	URBANO	310620060482	Mantiqueira	31062006069
5751	310620060690194	URBANO	310620060482	Mantiqueira	31062006069
5752	310620060690195	URBANO	310620060482	Mantiqueira	31062006069
5753	310620060690196	URBANO	310620060482	Mantiqueira	31062006069
5754	310620060690197	URBANO	310620060482	Mantiqueira	31062006069
5755	310620060690198	URBANO	310620060482	Mantiqueira	31062006069
5756	310620060690199	URBANO	310620060482	Mantiqueira	31062006069
5757	310620060690200	URBANO	310620060482	Mantiqueira	31062006069
5758	310620060690201	URBANO	310620060476	Jardim dos Comerci	31062006069
5759	310620060690202	URBANO	310620060476	Jardim dos Comerci	31062006069

5760	310620060690203	URBANO	310620060482	Mantiqueira	31062006069
5761	310620060690204	URBANO	310620060476	Jardim dos Comerci	31062006069
5762	310620060690205	URBANO	310620060476	Jardim dos Comerci	31062006069
5763	310620060690206	URBANO	310620060476	Jardim dos Comerci	31062006069
5764	310620060690207	URBANO	310620060476	Jardim dos Comerci	31062006069
5765	310620060690208	URBANO	310620060476	Jardim dos Comerci	31062006069
5766	310620060690209	URBANO	310620060476	Jardim dos Comerci	31062006069
5767	310620060690210	URBANO	310620060476	Jardim dos Comerci	31062006069
5768	310620060690211	URBANO	310620060476	Jardim dos Comerci	31062006069
5769	310620060690212	URBANO	310620060476	Jardim dos Comerci	31062006069
5770	310620060690213	URBANO	310620060476	Jardim dos Comerci	31062006069
5771	310620060690214	URBANO	310620060476	Jardim dos Comerci	31062006069
5772	310620060690215	URBANO	310620060476	Jardim dos Comerci	31062006069
5773	310620060690216	URBANO	310620060474	Europa	31062006069
5774	310620060690217	URBANO	310620060365	Serra Verde	31062006069
5775	310620060690218	URBANO	310620060474	Europa	31062006069
5776	310620060690219	URBANO	310620060365	Serra Verde	31062006069
5777	310620060690220	URBANO	310620060365	Serra Verde	31062006069
5778	310620060690221	URBANO	310620060365	Serra Verde	31062006069
5779	310620060690222	URBANO	310620060365	Serra Verde	31062006069
5780	310620060690223	URBANO	310620060365	Serra Verde	31062006069
5781	310620060690224	URBANO	310620060365	Serra Verde	31062006069
5782	310620060690225	URBANO	310620060365	Serra Verde	31062006069
5783	310620060690226	URBANO	310620060365	Serra Verde	31062006069
5784	310620060690227	URBANO	310620060365	Serra Verde	31062006069
5785	310620060690228	URBANO	310620060471	Conjunto Minas Cai:	31062006069
5786	310620060690229	URBANO	310620060365	Serra Verde	31062006069
5787	310620060690230	URBANO	310620060484	Minas Caixa	31062006069
5788	310620060690231	URBANO	310620060484	Minas Caixa	31062006069
5789	310620060690232	URBANO	310620060484	Minas Caixa	31062006069
5790	310620060690233	URBANO	310620060484	Minas Caixa	31062006069
5791	310620060690234	URBANO	310620060484	Minas Caixa	31062006069
5792	310620060690235	URBANO	310620060484	Minas Caixa	31062006069
5793	310620060690236	URBANO	310620060484	Minas Caixa	31062006069
5794	310620060690237	URBANO	310620060487	Parque SÒo Pedro	31062006069
5795	310620060690238	URBANO	310620060487	Parque SÒo Pedro	31062006069
5796	310620060690239	URBANO	310620060367	Venda Nova	31062006069
5797	310620060690240	URBANO	310620060367	Venda Nova	31062006069
5798	310620060690241	URBANO	310620060485	SÒo JoÒo Batista	31062006069
5799	310620060690242	URBANO	310620060485	SÒo JoÒo Batista	31062006069
5800	310620060690243	URBANO	310620060485	SÒo JoÒo Batista	31062006069
5801	310620060690244	URBANO	310620060485	SÒo JoÒo Batista	31062006069
5802	310620060690245	URBANO	310620060485	SÒo JoÒo Batista	31062006069
5803	310620060690246	URBANO	310620060485	SÒo JoÒo Batista	31062006069
5804	310620060690247	URBANO	310620060485	SÒo JoÒo Batista	31062006069
5805	310620060690248	URBANO	310620060485	SÒo JoÒo Batista	31062006069
5806	310620060690249	URBANO	310620060266	Copacabana	31062006069
5807	310620060690250	URBANO	310620060266	Copacabana	31062006069
5808	310620060690251	URBANO	310620060266	Copacabana	31062006069
5809	310620060690252	URBANO	310620060266	Copacabana	31062006069

5810	310620060690253	URBANO	310620060266	Copacabana	31062006069
5811	310620060690254	URBANO	310620060489	Rio Branco	31062006069
5812	310620060690255	URBANO	310620060488	Piratininga	31062006069
5813	310620060690256	URBANO	310620060489	Rio Branco	31062006069
5814	310620060690257	URBANO	310620060488	Piratininga	31062006069
5815	310620060690258	URBANO	310620060488	Piratininga	31062006069
5816	310620060690259	URBANO	310620060483	Maria Helena	31062006069
5817	310620060690260	URBANO	310620060369	Vila Copacabana	31062006069
5818	310620060690261	URBANO	310620060453	Vila Sesc	31062006069
5819	310620060690262	URBANO	310620060474	Europa	31062006069
5820	310620060690263	URBANO	310620060367	Venda Nova	31062006069
5821	310620060690264	URBANO	310620060480	Laranjeiras	31062006069
5822	310620060690265	URBANO	310620060485	SÒo JoÒo Batista	31062006069
5823	310620060690266	URBANO	310620060471	Conjunto Minas Cai	31062006069
5824	310620060690267	URBANO	310620060474	Europa	31062006069
5825	310620060690268	URBANO	310620060484	Minas Caixa	31062006069
5826	310620060690269	URBANO	310620060367	Venda Nova	31062006069
5827	310620060690270	URBANO	310620060485	SÒo JoÒo Batista	31062006069
5828	310620060690271	URBANO	310620060487	Parque SÒo Pedro	31062006069
5829	310620060690272	URBANO	310620060487	Parque SÒo Pedro	31062006069
5830	310620060690273	URBANO	310620060484	Minas Caixa	31062006069
5831	310620060690274	URBANO	310620060468	Apolonia	31062006069
5832	310620060690275	URBANO	310620060418	Santa Monica	31062006069
5833	310620060690276	URBANO	310620060468	Apolonia	31062006069
5834	310620060690277	URBANO	310620060488	Piratininga	31062006069
5835	310620060690278	URBANO	310620060418	Santa Monica	31062006069
5836	310620060690279	URBANO	310620060478	Lagoa	31062006069
5837	310620060690280	URBANO	310620060190	CÚu Azul	31062006069
5838	310620060690281	URBANO	310620060479	Lagoinha Leblon	31062006069
5839	310620060690282	URBANO	310620060478	Lagoa	31062006069
5840	310620060690283	URBANO	310620060477	Jardim Leblon	31062006069
5841	310620060690284	URBANO	310620060421	Universo	31062006069
5842	310620060690285	URBANO	310620060368	Vila Canto do Sabiß	31062006069
5843	310620060690286	URBANO	310620060471	Conjunto Minas Cai	31062006069
5844	310620060690287	URBANO	310620060367	Venda Nova	31062006069
5845	310620060690288	URBANO	310620060487	Parque SÒo Pedro	31062006069
5846	310620060690289	URBANO	310620060482	Mantiqueira	31062006069
5847	310620060690290	URBANO	310620060476	Jardim dos Comerci	31062006069
5848	310620060690291	URBANO	310620060482	Mantiqueira	31062006069
5849	310620060690292	URBANO	310620060476	Jardim dos Comerci	31062006069
5850	310620060690293	URBANO	310620060472	Conjunto Serra Verc	31062006069
5851	310620060690294	URBANO	310620060474	Europa	31062006069
5852	310620060690295	URBANO	310620060468	Apolonia	31062006069
5853	310620060690296	URBANO	310620060468	Apolonia	31062006069
5854	310620060690297	URBANO	310620060478	Lagoa	31062006069
5855	310620060690298	URBANO	310620060473	Vila Mantiqueira	31062006069
5856	310620060690299	URBANO	310620060482	Mantiqueira	31062006069
5857	310620060690300	URBANO	310620060473	Vila Mantiqueira	31062006069
5858	310620060690301	URBANO	310620060457	Vila Jardim Leblon	31062006069
5859	310620060690302	URBANO	310620060482	Mantiqueira	31062006069

5860	310620060690303	URBANO	310620060478	Lagoa	31062006069
5861	310620060690304	URBANO	310620060475	Flamengo	31062006069
5862	310620060690305	URBANO	310620060373	Vila Piratininga Ven	31062006069
5863	310620060690306	URBANO	310620060491	Vila Santa Monica 2	31062006069
5864	310620060690307	URBANO	310620060490	Vila Santa Monica 1	31062006069
5865	310620060690308	URBANO	310620060482	Mantiqueira	31062006069
5866	310620060690309	URBANO	310620060488	Piratininga	31062006069
5867	310620060690310	URBANO	310620060490	Vila Santa Monica 1	31062006069
5868	310620060690311	URBANO	310620060477	Jardim Leblon	31062006069
5869	310620060690312	URBANO	310620060477	Jardim Leblon	31062006069
5870	310620060690313	URBANO	310620060477	Jardim Leblon	31062006069
5871	310620060690314	URBANO	310620060477	Jardim Leblon	31062006069
5872	310620060690315	URBANO	310620060418	Santa Monica	31062006069
5873	310620060690316	URBANO	310620060485	SÒo JoÒo Batista	31062006069
5874	310620060690317	URBANO	310620060421	Universo	31062006069
5875	310620060690318	URBANO	310620060421	Universo	31062006069
5876	310620060690319	URBANO	310620060421	Universo	31062006069
5877	310620060690320	URBANO	310620060421	Universo	31062006069
5878	310620060690321	URBANO	310620060421	Universo	31062006069
5879	310620060690322	URBANO	310620060365	Serra Verde	31062006069
5880	310620060690323	URBANO	310620060477	Jardim Leblon	31062006069
5881	310620060690324	URBANO	310620060485	SÒo JoÒo Batista	31062006069
5882	310620060690325	URBANO	310620060190	CÚu Azul	31062006069
5883	310620060690326	URBANO	310620060481	Leticia	31062006069
5884	310620060690327	URBANO	310620060469	Candelaria	31062006069
5885	310620060690328	URBANO	310620060488	Piratininga	31062006069
5886	310620060690329	URBANO	310620060488	Piratininga	31062006069
5887	310620060690330	URBANO	310620060488	Piratininga	31062006069
5888	310620060690331	URBANO	310620060488	Piratininga	31062006069
5889	310620060690332	URBANO	310620060488	Piratininga	31062006069
5890	310620060690333	URBANO	310620060479	Lagoinha Leblon	31062006069
5891	310620060690334	URBANO	310620060479	Lagoinha Leblon	31062006069
5892	310620060690335	URBANO	310620060483	Maria Helena	31062006069
5893	310620060690336	URBANO	310620060482	Mantiqueira	31062006069
5894	310620060690337	URBANO	310620060482	Mantiqueira	31062006069
5906	310620060690349	URBANO	310620060485	SÒo JoÒo Batista	31062006069
5907	310620060690350	URBANO	310620060485	SÒo JoÒo Batista	31062006069
5908	310620060690351	URBANO	310620060418	Santa Monica	31062006069
5909	310620060690352	URBANO	310620060468	Apolonia	31062006069
5910	310620060690353	URBANO	310620060190	CÚu Azul	31062006069
5911	310620060690354	URBANO	310620060190	CÚu Azul	31062006069
5912	310620060690355	URBANO	310620060190	CÚu Azul	31062006069
5913	310620060690356	URBANO	310620060476	Jardim dos Comerci	31062006069
5914	310620060690357	URBANO	310620060476	Jardim dos Comerci	31062006069
5915	310620060690358	URBANO	310620060476	Jardim dos Comerci	31062006069
5916	310620060690359	URBANO	310620060365	Serra Verde	31062006069
5917	310620060690360	URBANO	310620060484	Minas Caixa	31062006069
5918	310620060690361	URBANO	310620060484	Minas Caixa	31062006069
5919	310620060690362	URBANO	310620060485	SÒo JoÒo Batista	31062006069
5920	310620060690363	URBANO	310620060485	SÒo JoÒo Batista	31062006069

5921	310620060690364	URBANO	310620060266	Copacabana	31062006069
5922	310620060690365	URBANO	310620060474	Europa	31062006069
5923	310620060690366	URBANO	310620060365	Serra Verde	31062006069
5924	310620060690367	URBANO	310620060484	Minas Caixa	31062006069
5925	310620060690368	URBANO	310620060365	Serra Verde	31062006069
5926	310620060690369	URBANO	310620060365	Serra Verde	31062006069
5927	310620060690370	URBANO	310620060474	Europa	31062006069
5928	310620060690371	URBANO	310620060420	Unidas	31062006069
5929	310620060690372	URBANO	310620060486	Nova America	31062006069
5930	310620060690373	URBANO	310620060474	Europa	31062006069
5931	310620060690374	URBANO	310620060474	Europa	31062006069
5932	310620060690375	URBANO	310620060481	Leticia	31062006069
5933	310620060690376	URBANO	310620060489	Rio Branco	31062006069
5934	310620060690377	URBANO	310620060367	Venda Nova	31062006069
5935	310620060690378	URBANO	310620060493	Nossa Senhora Apar	31062006069
5936	310620060690379	URBANO	310620060477	Jardim Leblon	31062006069
5937	310620060690380	URBANO	310620060489	Rio Branco	31062006069
5938	310620060690381	URBANO	310620060477	Jardim Leblon	31062006069
5939	310620060690382	URBANO	310620060468	Apolonia	31062006069
5940	310620060690383	URBANO	310620060477	Jardim Leblon	31062006069
5941	310620060690384	URBANO	310620060468	Apolonia	31062006069
5942	310620060690385	URBANO	310620060266	Copacabana	31062006069
5943	310620060690386	URBANO	310620060421	Universo	31062006069
5944	310620060690387	URBANO	310620060190	CÚu Azul	31062006069
5945	310620060690388	URBANO	310620060482	Mantiqueira	31062006069
5946	310620060690389	URBANO	310620060266	Copacabana	31062006069
5947	310620060690390	URBANO	310620060266	Copacabana	31062006069
5948	310620060690391	URBANO	310620060485	SÒo JoÒo Batista	31062006069
5949	310620060690392	URBANO	310620060477	Jardim Leblon	31062006069
5950	310620060690393	URBANO	310620060477	Jardim Leblon	31062006069
5951	310620060690394	URBANO	310620060477	Jardim Leblon	31062006069
5952	310620060690395	URBANO	310620060371	Vila SatÚlite	31062006069
5953	310620060690396	URBANO	310620060266	Copacabana	31062006069
5954	310620060690397	URBANO	310620060477	Jardim Leblon	31062006069
5955	310620060690398	URBANO	310620060474	Europa	31062006069
5956	310620060690399	URBANO	310620060477	Jardim Leblon	31062006069
5957	310620060690400	URBANO	310620060476	Jardim dos Comerci	31062006069

NM_SUBDIST	CD_GEOCODD	NM_DISTRIT	CD_GEOCOD	COD_DISTRI	COD_SUBDIS
CENTRO-SUL	310620005	BELO HORIZONT	3106200	310620005	31062000562
CENTRO-SUL	310620005	BELO HORIZONT	3106200	310620005	31062000562
CENTRO-SUL	310620005	BELO HORIZONT	3106200	310620005	31062000562
CENTRO-SUL	310620005	BELO HORIZONT	3106200	310620005	31062000562
CENTRO-SUL	310620005	BELO HORIZONT	3106200	310620005	31062000562
CENTRO-SUL	310620005	BELO HORIZONT	3106200	310620005	31062000562
CENTRO-SUL	310620005	BELO HORIZONT	3106200	310620005	31062000562
CENTRO-SUL	310620005	BELO HORIZONT	3106200	310620005	31062000562
CENTRO-SUL	310620005	BELO HORIZONT	3106200	310620005	31062000562
CENTRO-SUL	310620005	BELO HORIZONT	3106200	310620005	31062000562
CENTRO-SUL	310620005	BELO HORIZONT	3106200	310620005	31062000562
CENTRO-SUL	310620005	BELO HORIZONT	3106200	310620005	31062000562
CENTRO-SUL	310620005	BELO HORIZONT	3106200	310620005	31062000562
CENTRO-SUL	310620005	BELO HORIZONT	3106200	310620005	31062000562
CENTRO-SUL	310620005	BELO HORIZONT	3106200	310620005	31062000562
CENTRO-SUL	310620005	BELO HORIZONT	3106200	310620005	31062000562
CENTRO-SUL	310620005	BELO HORIZONT	3106200	310620005	31062000562
CENTRO-SUL	310620005	BELO HORIZONT	3106200	310620005	31062000562
CENTRO-SUL	310620005	BELO HORIZONT	3106200	310620005	31062000562
CENTRO-SUL	310620005	BELO HORIZONT	3106200	310620005	31062000562
CENTRO-SUL	310620005	BELO HORIZONT	3106200	310620005	31062000562
CENTRO-SUL	310620005	BELO HORIZONT	3106200	310620005	31062000562
CENTRO-SUL	310620005	BELO HORIZONT	3106200	310620005	31062000562
CENTRO-SUL	310620005	BELO HORIZONT	3106200	310620005	31062000562
CENTRO-SUL	310620005	BELO HORIZONT	3106200	310620005	31062000562
CENTRO-SUL	310620005	BELO HORIZONT	3106200	310620005	31062000562
CENTRO-SUL	310620005	BELO HORIZONT	3106200	310620005	31062000562
LESTE	310620005	BELO HORIZONT	3106200	310620005	31062000563
LESTE	310620005	BELO HORIZONT	3106200	310620005	31062000563
LESTE	310620005	BELO HORIZONT	3106200	310620005	31062000563
LESTE	310620005	BELO HORIZONT	3106200	310620005	31062000563
LESTE	310620005	BELO HORIZONT	3106200	310620005	31062000563
LESTE	310620005	BELO HORIZONT	3106200	310620005	31062000563
LESTE	310620005	BELO HORIZONT	3106200	310620005	31062000563
LESTE	310620005	BELO HORIZONT	3106200	310620005	31062000563
LESTE	310620005	BELO HORIZONT	3106200	310620005	31062000563
LESTE	310620005	BELO HORIZONT	3106200	310620005	31062000563
LESTE	310620005	BELO HORIZONT	3106200	310620005	31062000563
LESTE	310620005	BELO HORIZONT	3106200	310620005	31062000563
LESTE	310620005	BELO HORIZONT	3106200	310620005	31062000563
LESTE	310620005	BELO HORIZONT	3106200	310620005	31062000563
LESTE	310620005	BELO HORIZONT	3106200	310620005	31062000563
LESTE	310620005	BELO HORIZONT	3106200	310620005	31062000563
LESTE	310620005	BELO HORIZONT	3106200	310620005	31062000563
LESTE	310620005	BELO HORIZONT	3106200	310620005	31062000563
NORDESTE	310620005	BELO HORIZONT	3106200	310620005	31062000564
NORDESTE	310620005	BELO HORIZONT	3106200	310620005	31062000564
NORDESTE	310620005	BELO HORIZONT	3106200	310620005	31062000564
NORDESTE	310620005	BELO HORIZONT	3106200	310620005	31062000564

NORDESTE	310620005	BELO HORIZONT	3106200	310620005	31062000564
NORDESTE	310620005	BELO HORIZONT	3106200	310620005	31062000564
NORDESTE	310620005	BELO HORIZONT	3106200	310620005	31062000564
NORDESTE	310620005	BELO HORIZONT	3106200	310620005	31062000564
NOROESTE	310620005	BELO HORIZONT	3106200	310620005	31062000565
NOROESTE	310620005	BELO HORIZONT	3106200	310620005	31062000565
NOROESTE	310620005	BELO HORIZONT	3106200	310620005	31062000565
NOROESTE	310620005	BELO HORIZONT	3106200	310620005	31062000565
NOROESTE	310620005	BELO HORIZONT	3106200	310620005	31062000565
NOROESTE	310620005	BELO HORIZONT	3106200	310620005	31062000565
NOROESTE	310620005	BELO HORIZONT	3106200	310620005	31062000565
NOROESTE	310620005	BELO HORIZONT	3106200	310620005	31062000565
NOROESTE	310620005	BELO HORIZONT	3106200	310620005	31062000565
NOROESTE	310620005	BELO HORIZONT	3106200	310620005	31062000565
NOROESTE	310620005	BELO HORIZONT	3106200	310620005	31062000565
NOROESTE	310620005	BELO HORIZONT	3106200	310620005	31062000565
NOROESTE	310620005	BELO HORIZONT	3106200	310620005	31062000565
NOROESTE	310620005	BELO HORIZONT	3106200	310620005	31062000565
NOROESTE	310620005	BELO HORIZONT	3106200	310620005	31062000565
NOROESTE	310620005	BELO HORIZONT	3106200	310620005	31062000565
CENTRO-SUL	310620005	BELO HORIZONT	3106200	310620005	31062000562
CENTRO-SUL	310620005	BELO HORIZONT	3106200	310620005	31062000562
CENTRO-SUL	310620005	BELO HORIZONT	3106200	310620005	31062000562
CENTRO-SUL	310620005	BELO HORIZONT	3106200	310620005	31062000562
CENTRO-SUL	310620005	BELO HORIZONT	3106200	310620005	31062000562
CENTRO-SUL	310620005	BELO HORIZONT	3106200	310620005	31062000562
CENTRO-SUL	310620005	BELO HORIZONT	3106200	310620005	31062000562
CENTRO-SUL	310620005	BELO HORIZONT	3106200	310620005	31062000562
CENTRO-SUL	310620005	BELO HORIZONT	3106200	310620005	31062000562
CENTRO-SUL	310620005	BELO HORIZONT	3106200	310620005	31062000562
CENTRO-SUL	310620005	BELO HORIZONT	3106200	310620005	31062000562
CENTRO-SUL	310620005	BELO HORIZONT	3106200	310620005	31062000562
CENTRO-SUL	310620005	BELO HORIZONT	3106200	310620005	31062000562
CENTRO-SUL	310620005	BELO HORIZONT	3106200	310620005	31062000562
CENTRO-SUL	310620005	BELO HORIZONT	3106200	310620005	31062000562
CENTRO-SUL	310620005	BELO HORIZONT	3106200	310620005	31062000562
CENTRO-SUL	310620005	BELO HORIZONT	3106200	310620005	31062000562
CENTRO-SUL	310620005	BELO HORIZONT	3106200	310620005	31062000562
CENTRO-SUL	310620005	BELO HORIZONT	3106200	310620005	31062000562
CENTRO-SUL	310620005	BELO HORIZONT	3106200	310620005	31062000562
CENTRO-SUL	310620005	BELO HORIZONT	3106200	310620005	31062000562
CENTRO-SUL	310620005	BELO HORIZONT	3106200	310620005	31062000562
CENTRO-SUL	310620005	BELO HORIZONT	3106200	310620005	31062000562
CENTRO-SUL	310620005	BELO HORIZONT	3106200	310620005	31062000562
CENTRO-SUL	310620005	BELO HORIZONT	3106200	310620005	31062000562
CENTRO-SUL	310620005	BELO HORIZONT	3106200	310620005	31062000562
CENTRO-SUL	310620005	BELO HORIZONT	3106200	310620005	31062000562
CENTRO-SUL	310620005	BELO HORIZONT	3106200	310620005	31062000562
CENTRO-SUL	310620005	BELO HORIZONT	3106200	310620005	31062000562
CENTRO-SUL	310620005	BELO HORIZONT	3106200	310620005	31062000562

CENTRO-SUL	310620005	BELO HORIZONT	3106200	310620005	31062000562
CENTRO-SUL	310620005	BELO HORIZONT	3106200	310620005	31062000562
CENTRO-SUL	310620005	BELO HORIZONT	3106200	310620005	31062000562
CENTRO-SUL	310620005	BELO HORIZONT	3106200	310620005	31062000562
CENTRO-SUL	310620005	BELO HORIZONT	3106200	310620005	31062000562
CENTRO-SUL	310620005	BELO HORIZONT	3106200	310620005	31062000562
CENTRO-SUL	310620005	BELO HORIZONT	3106200	310620005	31062000562
CENTRO-SUL	310620005	BELO HORIZONT	3106200	310620005	31062000562
CENTRO-SUL	310620005	BELO HORIZONT	3106200	310620005	31062000562
CENTRO-SUL	310620005	BELO HORIZONT	3106200	310620005	31062000562
CENTRO-SUL	310620005	BELO HORIZONT	3106200	310620005	31062000562
CENTRO-SUL	310620005	BELO HORIZONT	3106200	310620005	31062000562
CENTRO-SUL	310620005	BELO HORIZONT	3106200	310620005	31062000562
CENTRO-SUL	310620005	BELO HORIZONT	3106200	310620005	31062000562
CENTRO-SUL	310620005	BELO HORIZONT	3106200	310620005	31062000562
CENTRO-SUL	310620005	BELO HORIZONT	3106200	310620005	31062000562
CENTRO-SUL	310620005	BELO HORIZONT	3106200	310620005	31062000562
CENTRO-SUL	310620005	BELO HORIZONT	3106200	310620005	31062000562
CENTRO-SUL	310620005	BELO HORIZONT	3106200	310620005	31062000562
CENTRO-SUL	310620005	BELO HORIZONT	3106200	310620005	31062000562
CENTRO-SUL	310620005	BELO HORIZONT	3106200	310620005	31062000562
CENTRO-SUL	310620005	BELO HORIZONT	3106200	310620005	31062000562
CENTRO-SUL	310620005	BELO HORIZONT	3106200	310620005	31062000562
CENTRO-SUL	310620005	BELO HORIZONT	3106200	310620005	31062000562
CENTRO-SUL	310620005	BELO HORIZONT	3106200	310620005	31062000562
CENTRO-SUL	310620005	BELO HORIZONT	3106200	310620005	31062000562
CENTRO-SUL	310620005	BELO HORIZONT	3106200	310620005	31062000562
CENTRO-SUL	310620005	BELO HORIZONT	3106200	310620005	31062000562
CENTRO-SUL	310620005	BELO HORIZONT	3106200	310620005	31062000562
CENTRO-SUL	310620005	BELO HORIZONT	3106200	310620005	31062000562
CENTRO-SUL	310620005	BELO HORIZONT	3106200	310620005	31062000562
CENTRO-SUL	310620005	BELO HORIZONT	3106200	310620005	31062000562
CENTRO-SUL	310620005	BELO HORIZONT	3106200	310620005	31062000562
CENTRO-SUL	310620005	BELO HORIZONT	3106200	310620005	31062000562
CENTRO-SUL	310620005	BELO HORIZONT	3106200	310620005	31062000562
CENTRO-SUL	310620005	BELO HORIZONT	3106200	310620005	31062000562
CENTRO-SUL	310620005	BELO HORIZONT	3106200	310620005	31062000562
CENTRO-SUL	310620005	BELO HORIZONT	3106200	310620005	31062000562
CENTRO-SUL	310620005	BELO HORIZONT	3106200	310620005	31062000562
CENTRO-SUL	310620005	BELO HORIZONT	3106200	310620005	31062000562
CENTRO-SUL	310620005	BELO HORIZONT	3106200	310620005	31062000562
CENTRO-SUL	310620005	BELO HORIZONT	3106200	310620005	31062000562
CENTRO-SUL	310620005	BELO HORIZONT	3106200	310620005	31062000562
CENTRO-SUL	310620005	BELO HORIZONT	3106200	310620005	31062000562
CENTRO-SUL	310620005	BELO HORIZONT	3106200	310620005	31062000562
CENTRO-SUL	310620005	BELO HORIZONT	3106200	310620005	31062000562
CENTRO-SUL	310620005	BELO HORIZONT	3106200	310620005	31062000562
CENTRO-SUL	310620005	BELO HORIZONT	3106200	310620005	31062000562
CENTRO-SUL	310620005	BELO HORIZONT	3106200	310620005	31062000562
CENTRO-SUL	310620005	BELO HORIZONT	3106200	310620005	31062000562

CENTRO-SUL	310620005	BELO HORIZONT	3106200	310620005	31062000562
CENTRO-SUL	310620005	BELO HORIZONT	3106200	310620005	31062000562
CENTRO-SUL	310620005	BELO HORIZONT	3106200	310620005	31062000562
CENTRO-SUL	310620005	BELO HORIZONT	3106200	310620005	31062000562
CENTRO-SUL	310620005	BELO HORIZONT	3106200	310620005	31062000562
CENTRO-SUL	310620005	BELO HORIZONT	3106200	310620005	31062000562
CENTRO-SUL	310620005	BELO HORIZONT	3106200	310620005	31062000562
CENTRO-SUL	310620005	BELO HORIZONT	3106200	310620005	31062000562
CENTRO-SUL	310620005	BELO HORIZONT	3106200	310620005	31062000562
CENTRO-SUL	310620005	BELO HORIZONT	3106200	310620005	31062000562
CENTRO-SUL	310620005	BELO HORIZONT	3106200	310620005	31062000562
CENTRO-SUL	310620005	BELO HORIZONT	3106200	310620005	31062000562
CENTRO-SUL	310620005	BELO HORIZONT	3106200	310620005	31062000562
CENTRO-SUL	310620005	BELO HORIZONT	3106200	310620005	31062000562
CENTRO-SUL	310620005	BELO HORIZONT	3106200	310620005	31062000562
CENTRO-SUL	310620005	BELO HORIZONT	3106200	310620005	31062000562
CENTRO-SUL	310620005	BELO HORIZONT	3106200	310620005	31062000562
CENTRO-SUL	310620005	BELO HORIZONT	3106200	310620005	31062000562
CENTRO-SUL	310620005	BELO HORIZONT	3106200	310620005	31062000562
CENTRO-SUL	310620005	BELO HORIZONT	3106200	310620005	31062000562
CENTRO-SUL	310620005	BELO HORIZONT	3106200	310620005	31062000562
CENTRO-SUL	310620005	BELO HORIZONT	3106200	310620005	31062000562
CENTRO-SUL	310620005	BELO HORIZONT	3106200	310620005	31062000562
CENTRO-SUL	310620005	BELO HORIZONT	3106200	310620005	31062000562
CENTRO-SUL	310620005	BELO HORIZONT	3106200	310620005	31062000562
CENTRO-SUL	310620005	BELO HORIZONT	3106200	310620005	31062000562
CENTRO-SUL	310620005	BELO HORIZONT	3106200	310620005	31062000562
CENTRO-SUL	310620005	BELO HORIZONT	3106200	310620005	31062000562
CENTRO-SUL	310620005	BELO HORIZONT	3106200	310620005	31062000562
CENTRO-SUL	310620005	BELO HORIZONT	3106200	310620005	31062000562
CENTRO-SUL	310620005	BELO HORIZONT	3106200	310620005	31062000562
CENTRO-SUL	310620005	BELO HORIZONT	3106200	310620005	31062000562
CENTRO-SUL	310620005	BELO HORIZONT	3106200	310620005	31062000562
CENTRO-SUL	310620005	BELO HORIZONT	3106200	310620005	31062000562
CENTRO-SUL	310620005	BELO HORIZONT	3106200	310620005	31062000562
CENTRO-SUL	310620005	BELO HORIZONT	3106200	310620005	31062000562
CENTRO-SUL	310620005	BELO HORIZONT	3106200	310620005	31062000562
CENTRO-SUL	310620005	BELO HORIZONT	3106200	310620005	31062000562
CENTRO-SUL	310620005	BELO HORIZONT	3106200	310620005	31062000562
CENTRO-SUL	310620005	BELO HORIZONT	3106200	310620005	31062000562
CENTRO-SUL	310620005	BELO HORIZONT	3106200	310620005	31062000562
CENTRO-SUL	310620005	BELO HORIZONT	3106200	310620005	31062000562
CENTRO-SUL	310620005	BELO HORIZONT	3106200	310620005	31062000562
CENTRO-SUL	310620005	BELO HORIZONT	3106200	310620005	31062000562
CENTRO-SUL	310620005	BELO HORIZONT	3106200	310620005	31062000562
CENTRO-SUL	310620005	BELO HORIZONT	3106200	310620005	31062000562
CENTRO-SUL	310620005	BELO HORIZONT	3106200	310620005	31062000562
CENTRO-SUL	310620005	BELO HORIZONT	3106200	310620005	31062000562
CENTRO-SUL	310620005	BELO HORIZONT	3106200	310620005	31062000562
CENTRO-SUL	310620005	BELO HORIZONT	3106200	310620005	31062000562

CENTRO-SUL	310620005	BELO HORIZONT	3106200	310620005	31062000562
CENTRO-SUL	310620005	BELO HORIZONT	3106200	310620005	31062000562
CENTRO-SUL	310620005	BELO HORIZONT	3106200	310620005	31062000562
CENTRO-SUL	310620005	BELO HORIZONT	3106200	310620005	31062000562
CENTRO-SUL	310620005	BELO HORIZONT	3106200	310620005	31062000562
CENTRO-SUL	310620005	BELO HORIZONT	3106200	310620005	31062000562
CENTRO-SUL	310620005	BELO HORIZONT	3106200	310620005	31062000562
CENTRO-SUL	310620005	BELO HORIZONT	3106200	310620005	31062000562
CENTRO-SUL	310620005	BELO HORIZONT	3106200	310620005	31062000562
CENTRO-SUL	310620005	BELO HORIZONT	3106200	310620005	31062000562
CENTRO-SUL	310620005	BELO HORIZONT	3106200	310620005	31062000562
CENTRO-SUL	310620005	BELO HORIZONT	3106200	310620005	31062000562
CENTRO-SUL	310620005	BELO HORIZONT	3106200	310620005	31062000562
CENTRO-SUL	310620005	BELO HORIZONT	3106200	310620005	31062000562
CENTRO-SUL	310620005	BELO HORIZONT	3106200	310620005	31062000562
CENTRO-SUL	310620005	BELO HORIZONT	3106200	310620005	31062000562
CENTRO-SUL	310620005	BELO HORIZONT	3106200	310620005	31062000562
CENTRO-SUL	310620005	BELO HORIZONT	3106200	310620005	31062000562
CENTRO-SUL	310620005	BELO HORIZONT	3106200	310620005	31062000562
CENTRO-SUL	310620005	BELO HORIZONT	3106200	310620005	31062000562
CENTRO-SUL	310620005	BELO HORIZONT	3106200	310620005	31062000562
CENTRO-SUL	310620005	BELO HORIZONT	3106200	310620005	31062000562
CENTRO-SUL	310620005	BELO HORIZONT	3106200	310620005	31062000562
CENTRO-SUL	310620005	BELO HORIZONT	3106200	310620005	31062000562
CENTRO-SUL	310620005	BELO HORIZONT	3106200	310620005	31062000562
CENTRO-SUL	310620005	BELO HORIZONT	3106200	310620005	31062000562
CENTRO-SUL	310620005	BELO HORIZONT	3106200	310620005	31062000562
CENTRO-SUL	310620005	BELO HORIZONT	3106200	310620005	31062000562
CENTRO-SUL	310620005	BELO HORIZONT	3106200	310620005	31062000562
CENTRO-SUL	310620005	BELO HORIZONT	3106200	310620005	31062000562
CENTRO-SUL	310620005	BELO HORIZONT	3106200	310620005	31062000562
CENTRO-SUL	310620005	BELO HORIZONT	3106200	310620005	31062000562
CENTRO-SUL	310620005	BELO HORIZONT	3106200	310620005	31062000562
CENTRO-SUL	310620005	BELO HORIZONT	3106200	310620005	31062000562
CENTRO-SUL	310620005	BELO HORIZONT	3106200	310620005	31062000562
CENTRO-SUL	310620005	BELO HORIZONT	3106200	310620005	31062000562
CENTRO-SUL	310620005	BELO HORIZONT	3106200	310620005	31062000562
CENTRO-SUL	310620005	BELO HORIZONT	3106200	310620005	31062000562
CENTRO-SUL	310620005	BELO HORIZONT	3106200	310620005	31062000562
CENTRO-SUL	310620005	BELO HORIZONT	3106200	310620005	31062000562
CENTRO-SUL	310620005	BELO HORIZONT	3106200	310620005	31062000562
CENTRO-SUL	310620005	BELO HORIZONT	3106200	310620005	31062000562
CENTRO-SUL	310620005	BELO HORIZONT	3106200	310620005	31062000562
CENTRO-SUL	310620005	BELO HORIZONT	3106200	310620005	31062000562
CENTRO-SUL	310620005	BELO HORIZONT	3106200	310620005	31062000562
CENTRO-SUL	310620005	BELO HORIZONT	3106200	310620005	31062000562
CENTRO-SUL	310620005	BELO HORIZONT	3106200	310620005	31062000562
CENTRO-SUL	310620005	BELO HORIZONT	3106200	310620005	31062000562
CENTRO-SUL	310620005	BELO HORIZONT	3106200	310620005	31062000562
CENTRO-SUL	310620005	BELO HORIZONT	3106200	310620005	31062000562

CENTRO-SUL	310620005	BELO HORIZONT	3106200	310620005	31062000562
CENTRO-SUL	310620005	BELO HORIZONT	3106200	310620005	31062000562
CENTRO-SUL	310620005	BELO HORIZONT	3106200	310620005	31062000562
CENTRO-SUL	310620005	BELO HORIZONT	3106200	310620005	31062000562
CENTRO-SUL	310620005	BELO HORIZONT	3106200	310620005	31062000562
CENTRO-SUL	310620005	BELO HORIZONT	3106200	310620005	31062000562
CENTRO-SUL	310620005	BELO HORIZONT	3106200	310620005	31062000562
CENTRO-SUL	310620005	BELO HORIZONT	3106200	310620005	31062000562
CENTRO-SUL	310620005	BELO HORIZONT	3106200	310620005	31062000562
CENTRO-SUL	310620005	BELO HORIZONT	3106200	310620005	31062000562
CENTRO-SUL	310620005	BELO HORIZONT	3106200	310620005	31062000562
CENTRO-SUL	310620005	BELO HORIZONT	3106200	310620005	31062000562
CENTRO-SUL	310620005	BELO HORIZONT	3106200	310620005	31062000562
CENTRO-SUL	310620005	BELO HORIZONT	3106200	310620005	31062000562
CENTRO-SUL	310620005	BELO HORIZONT	3106200	310620005	31062000562
CENTRO-SUL	310620005	BELO HORIZONT	3106200	310620005	31062000562
CENTRO-SUL	310620005	BELO HORIZONT	3106200	310620005	31062000562
CENTRO-SUL	310620005	BELO HORIZONT	3106200	310620005	31062000562
CENTRO-SUL	310620005	BELO HORIZONT	3106200	310620005	31062000562
CENTRO-SUL	310620005	BELO HORIZONT	3106200	310620005	31062000562
CENTRO-SUL	310620005	BELO HORIZONT	3106200	310620005	31062000562
CENTRO-SUL	310620005	BELO HORIZONT	3106200	310620005	31062000562
CENTRO-SUL	310620005	BELO HORIZONT	3106200	310620005	31062000562
CENTRO-SUL	310620005	BELO HORIZONT	3106200	310620005	31062000562
CENTRO-SUL	310620005	BELO HORIZONT	3106200	310620005	31062000562
CENTRO-SUL	310620005	BELO HORIZONT	3106200	310620005	31062000562
CENTRO-SUL	310620005	BELO HORIZONT	3106200	310620005	31062000562
CENTRO-SUL	310620005	BELO HORIZONT	3106200	310620005	31062000562
CENTRO-SUL	310620005	BELO HORIZONT	3106200	310620005	31062000562
CENTRO-SUL	310620005	BELO HORIZONT	3106200	310620005	31062000562
CENTRO-SUL	310620005	BELO HORIZONT	3106200	310620005	31062000562
CENTRO-SUL	310620005	BELO HORIZONT	3106200	310620005	31062000562
CENTRO-SUL	310620005	BELO HORIZONT	3106200	310620005	31062000562
CENTRO-SUL	310620005	BELO HORIZONT	3106200	310620005	31062000562
CENTRO-SUL	310620005	BELO HORIZONT	3106200	310620005	31062000562
CENTRO-SUL	310620005	BELO HORIZONT	3106200	310620005	31062000562
CENTRO-SUL	310620005	BELO HORIZONT	3106200	310620005	31062000562
CENTRO-SUL	310620005	BELO HORIZONT	3106200	310620005	31062000562
CENTRO-SUL	310620005	BELO HORIZONT	3106200	310620005	31062000562
CENTRO-SUL	310620005	BELO HORIZONT	3106200	310620005	31062000562
CENTRO-SUL	310620005	BELO HORIZONT	3106200	310620005	31062000562
CENTRO-SUL	310620005	BELO HORIZONT	3106200	310620005	31062000562
CENTRO-SUL	310620005	BELO HORIZONT	3106200	310620005	31062000562
CENTRO-SUL	310620005	BELO HORIZONT	3106200	310620005	31062000562
CENTRO-SUL	310620005	BELO HORIZONT	3106200	310620005	31062000562
CENTRO-SUL	310620005	BELO HORIZONT	3106200	310620005	31062000562
CENTRO-SUL	310620005	BELO HORIZONT	3106200	310620005	31062000562
CENTRO-SUL	310620005	BELO HORIZONT	3106200	310620005	31062000562
CENTRO-SUL	310620005	BELO HORIZONT	3106200	310620005	31062000562
CENTRO-SUL	310620005	BELO HORIZONT	3106200	310620005	31062000562

CENTRO-SUL	310620005	BELO HORIZONT	3106200	310620005	31062000562
CENTRO-SUL	310620005	BELO HORIZONT	3106200	310620005	31062000562
CENTRO-SUL	310620005	BELO HORIZONT	3106200	310620005	31062000562
CENTRO-SUL	310620005	BELO HORIZONT	3106200	310620005	31062000562
CENTRO-SUL	310620005	BELO HORIZONT	3106200	310620005	31062000562
CENTRO-SUL	310620005	BELO HORIZONT	3106200	310620005	31062000562
CENTRO-SUL	310620005	BELO HORIZONT	3106200	310620005	31062000562
CENTRO-SUL	310620005	BELO HORIZONT	3106200	310620005	31062000562
CENTRO-SUL	310620005	BELO HORIZONT	3106200	310620005	31062000562
CENTRO-SUL	310620005	BELO HORIZONT	3106200	310620005	31062000562
CENTRO-SUL	310620005	BELO HORIZONT	3106200	0	0
CENTRO-SUL	310620005	BELO HORIZONT	3106200	310620005	31062000562
CENTRO-SUL	310620005	BELO HORIZONT	3106200	310620005	31062000562
CENTRO-SUL	310620005	BELO HORIZONT	3106200	310620005	31062000562
CENTRO-SUL	310620005	BELO HORIZONT	3106200	310620005	31062000562
CENTRO-SUL	310620005	BELO HORIZONT	3106200	310620005	31062000562
CENTRO-SUL	310620005	BELO HORIZONT	3106200	310620005	31062000562
CENTRO-SUL	310620005	BELO HORIZONT	3106200	310620005	31062000562
CENTRO-SUL	310620005	BELO HORIZONT	3106200	310620005	31062000562
CENTRO-SUL	310620005	BELO HORIZONT	3106200	310620005	31062000562
CENTRO-SUL	310620005	BELO HORIZONT	3106200	310620005	31062000562
CENTRO-SUL	310620005	BELO HORIZONT	3106200	310620005	31062000562
CENTRO-SUL	310620005	BELO HORIZONT	3106200	310620005	31062000562
CENTRO-SUL	310620005	BELO HORIZONT	3106200	310620005	31062000562
CENTRO-SUL	310620005	BELO HORIZONT	3106200	310620005	31062000562
CENTRO-SUL	310620005	BELO HORIZONT	3106200	310620005	31062000562
CENTRO-SUL	310620005	BELO HORIZONT	3106200	310620005	31062000562
CENTRO-SUL	310620005	BELO HORIZONT	3106200	310620005	31062000562
CENTRO-SUL	310620005	BELO HORIZONT	3106200	310620005	31062000562
CENTRO-SUL	310620005	BELO HORIZONT	3106200	310620005	31062000562
CENTRO-SUL	310620005	BELO HORIZONT	3106200	310620005	31062000562
CENTRO-SUL	310620005	BELO HORIZONT	3106200	310620005	31062000562
CENTRO-SUL	310620005	BELO HORIZONT	3106200	310620005	31062000562
CENTRO-SUL	310620005	BELO HORIZONT	3106200	310620005	31062000562
CENTRO-SUL	310620005	BELO HORIZONT	3106200	310620005	31062000562
CENTRO-SUL	310620005	BELO HORIZONT	3106200	310620005	31062000562
CENTRO-SUL	310620005	BELO HORIZONT	3106200	310620005	31062000562
CENTRO-SUL	310620005	BELO HORIZONT	3106200	310620005	31062000562
CENTRO-SUL	310620005	BELO HORIZONT	3106200	310620005	31062000562
CENTRO-SUL	310620005	BELO HORIZONT	3106200	310620005	31062000562
CENTRO-SUL	310620005	BELO HORIZONT	3106200	310620005	31062000562
CENTRO-SUL	310620005	BELO HORIZONT	3106200	310620005	31062000562
CENTRO-SUL	310620005	BELO HORIZONT	3106200	310620005	31062000562
CENTRO-SUL	310620005	BELO HORIZONT	3106200	310620005	31062000562
CENTRO-SUL	310620005	BELO HORIZONT	3106200	310620005	31062000562
CENTRO-SUL	310620005	BELO HORIZONT	3106200	310620005	31062000562
CENTRO-SUL	310620005	BELO HORIZONT	3106200	310620005	31062000562
CENTRO-SUL	310620005	BELO HORIZONT	3106200	310620005	31062000562
CENTRO-SUL	310620005	BELO HORIZONT	3106200	310620005	31062000562
CENTRO-SUL	310620005	BELO HORIZONT	3106200	310620005	31062000562

CENTRO-SUL	310620005	BELO HORIZONT	3106200	310620005	31062000562
CENTRO-SUL	310620005	BELO HORIZONT	3106200	310620005	31062000562
CENTRO-SUL	310620005	BELO HORIZONT	3106200	310620005	31062000562
CENTRO-SUL	310620005	BELO HORIZONT	3106200	310620005	31062000562
CENTRO-SUL	310620005	BELO HORIZONT	3106200	310620005	31062000562
CENTRO-SUL	310620005	BELO HORIZONT	3106200	310620005	31062000562
CENTRO-SUL	310620005	BELO HORIZONT	3106200	310620005	31062000562
CENTRO-SUL	310620005	BELO HORIZONT	3106200	310620005	31062000562
CENTRO-SUL	310620005	BELO HORIZONT	3106200	310620005	31062000562
CENTRO-SUL	310620005	BELO HORIZONT	3106200	310620005	31062000562
CENTRO-SUL	310620005	BELO HORIZONT	3106200	310620005	31062000562
CENTRO-SUL	310620005	BELO HORIZONT	3106200	310620005	31062000562
CENTRO-SUL	310620005	BELO HORIZONT	3106200	310620005	31062000562
CENTRO-SUL	310620005	BELO HORIZONT	3106200	310620005	31062000562
CENTRO-SUL	310620005	BELO HORIZONT	3106200	310620005	31062000562
CENTRO-SUL	310620005	BELO HORIZONT	3106200	310620005	31062000562
CENTRO-SUL	310620005	BELO HORIZONT	3106200	310620005	31062000562
CENTRO-SUL	310620005	BELO HORIZONT	3106200	310620005	31062000562
CENTRO-SUL	310620005	BELO HORIZONT	3106200	310620005	31062000562
CENTRO-SUL	310620005	BELO HORIZONT	3106200	310620005	31062000562
CENTRO-SUL	310620005	BELO HORIZONT	3106200	310620005	31062000562
CENTRO-SUL	310620005	BELO HORIZONT	3106200	310620005	31062000562
CENTRO-SUL	310620005	BELO HORIZONT	3106200	310620005	31062000562
CENTRO-SUL	310620005	BELO HORIZONT	3106200	310620005	31062000562
CENTRO-SUL	310620005	BELO HORIZONT	3106200	310620005	31062000562
CENTRO-SUL	310620005	BELO HORIZONT	3106200	310620005	31062000562
CENTRO-SUL	310620005	BELO HORIZONT	3106200	310620005	31062000562
CENTRO-SUL	310620005	BELO HORIZONT	3106200	310620005	31062000562
CENTRO-SUL	310620005	BELO HORIZONT	3106200	310620005	31062000562
CENTRO-SUL	310620005	BELO HORIZONT	3106200	310620005	31062000562
CENTRO-SUL	310620005	BELO HORIZONT	3106200	310620005	31062000562
CENTRO-SUL	310620005	BELO HORIZONT	3106200	310620005	31062000562
CENTRO-SUL	310620005	BELO HORIZONT	3106200	310620005	31062000562
CENTRO-SUL	310620005	BELO HORIZONT	3106200	310620005	31062000562
CENTRO-SUL	310620005	BELO HORIZONT	3106200	310620005	31062000562
CENTRO-SUL	310620005	BELO HORIZONT	3106200	310620005	31062000562
CENTRO-SUL	310620005	BELO HORIZONT	3106200	310620005	31062000562
CENTRO-SUL	310620005	BELO HORIZONT	3106200	310620005	31062000562
CENTRO-SUL	310620005	BELO HORIZONT	3106200	310620005	31062000562
CENTRO-SUL	310620005	BELO HORIZONT	3106200	310620005	31062000562
CENTRO-SUL	310620005	BELO HORIZONT	3106200	310620005	31062000562
CENTRO-SUL	310620005	BELO HORIZONT	3106200	310620005	31062000562
CENTRO-SUL	310620005	BELO HORIZONT	3106200	310620005	31062000562
CENTRO-SUL	310620005	BELO HORIZONT	3106200	310620005	31062000562
CENTRO-SUL	310620005	BELO HORIZONT	3106200	310620005	31062000562
CENTRO-SUL	310620005	BELO HORIZONT	3106200	310620005	31062000562
CENTRO-SUL	310620005	BELO HORIZONT	3106200	310620005	31062000562
CENTRO-SUL	310620005	BELO HORIZONT	3106200	310620005	31062000562
CENTRO-SUL	310620005	BELO HORIZONT	3106200	310620005	31062000562
CENTRO-SUL	310620005	BELO HORIZONT	3106200	310620005	31062000562

CENTRO-SUL	310620005	BELO HORIZONT	3106200	310620005	31062000562
CENTRO-SUL	310620005	BELO HORIZONT	3106200	310620005	31062000562
CENTRO-SUL	310620005	BELO HORIZONT	3106200	310620005	31062000562
CENTRO-SUL	310620005	BELO HORIZONT	3106200	310620005	31062000562
CENTRO-SUL	310620005	BELO HORIZONT	3106200	310620005	31062000562
CENTRO-SUL	310620005	BELO HORIZONT	3106200	310620005	31062000562
CENTRO-SUL	310620005	BELO HORIZONT	3106200	0	0
CENTRO-SUL	310620005	BELO HORIZONT	3106200	310620005	31062000562
CENTRO-SUL	310620005	BELO HORIZONT	3106200	310620005	31062000562
CENTRO-SUL	310620005	BELO HORIZONT	3106200	310620005	31062000562
CENTRO-SUL	310620005	BELO HORIZONT	3106200	310620005	31062000562
CENTRO-SUL	310620005	BELO HORIZONT	3106200	310620005	31062000562
CENTRO-SUL	310620005	BELO HORIZONT	3106200	310620005	31062000562
CENTRO-SUL	310620005	BELO HORIZONT	3106200	310620005	31062000562
CENTRO-SUL	310620005	BELO HORIZONT	3106200	310620005	31062000562
CENTRO-SUL	310620005	BELO HORIZONT	3106200	310620005	31062000562
CENTRO-SUL	310620005	BELO HORIZONT	3106200	310620005	31062000562
CENTRO-SUL	310620005	BELO HORIZONT	3106200	310620005	31062000562
CENTRO-SUL	310620005	BELO HORIZONT	3106200	310620005	31062000562
CENTRO-SUL	310620005	BELO HORIZONT	3106200	310620005	31062000562
CENTRO-SUL	310620005	BELO HORIZONT	3106200	310620005	31062000562
CENTRO-SUL	310620005	BELO HORIZONT	3106200	310620005	31062000562
CENTRO-SUL	310620005	BELO HORIZONT	3106200	310620005	31062000562
CENTRO-SUL	310620005	BELO HORIZONT	3106200	310620005	31062000562
CENTRO-SUL	310620005	BELO HORIZONT	3106200	310620005	31062000562
CENTRO-SUL	310620005	BELO HORIZONT	3106200	310620005	31062000562
CENTRO-SUL	310620005	BELO HORIZONT	3106200	310620005	31062000562
CENTRO-SUL	310620005	BELO HORIZONT	3106200	310620005	31062000562
CENTRO-SUL	310620005	BELO HORIZONT	3106200	310620005	31062000562
CENTRO-SUL	310620005	BELO HORIZONT	3106200	310620005	31062000562
CENTRO-SUL	310620005	BELO HORIZONT	3106200	310620005	31062000562
CENTRO-SUL	310620005	BELO HORIZONT	3106200	310620005	31062000562
CENTRO-SUL	310620005	BELO HORIZONT	3106200	310620005	31062000562
CENTRO-SUL	310620005	BELO HORIZONT	3106200	310620005	31062000562
CENTRO-SUL	310620005	BELO HORIZONT	3106200	310620005	31062000562
CENTRO-SUL	310620005	BELO HORIZONT	3106200	310620005	31062000562
CENTRO-SUL	310620005	BELO HORIZONT	3106200	310620005	31062000562
CENTRO-SUL	310620005	BELO HORIZONT	3106200	310620005	31062000562
CENTRO-SUL	310620005	BELO HORIZONT	3106200	310620005	31062000562
CENTRO-SUL	310620005	BELO HORIZONT	3106200	310620005	31062000562
CENTRO-SUL	310620005	BELO HORIZONT	3106200	310620005	31062000562
CENTRO-SUL	310620005	BELO HORIZONT	3106200	310620005	31062000562
CENTRO-SUL	310620005	BELO HORIZONT	3106200	310620005	31062000562
CENTRO-SUL	310620005	BELO HORIZONT	3106200	310620005	31062000562
CENTRO-SUL	310620005	BELO HORIZONT	3106200	310620005	31062000562
CENTRO-SUL	310620005	BELO HORIZONT	3106200	310620005	31062000562
CENTRO-SUL	310620005	BELO HORIZONT	3106200	310620005	31062000562
CENTRO-SUL	310620005	BELO HORIZONT	3106200	310620005	31062000562
CENTRO-SUL	310620005	BELO HORIZONT	3106200	310620005	31062000562
CENTRO-SUL	310620005	BELO HORIZONT	3106200	310620005	31062000562

CENTRO-SUL	310620005	BELO HORIZONT	3106200	310620005	31062000562
CENTRO-SUL	310620005	BELO HORIZONT	3106200	310620005	31062000562
CENTRO-SUL	310620005	BELO HORIZONT	3106200	310620005	31062000562
CENTRO-SUL	310620005	BELO HORIZONT	3106200	310620005	31062000562
CENTRO-SUL	310620005	BELO HORIZONT	3106200	310620005	31062000562
CENTRO-SUL	310620005	BELO HORIZONT	3106200	310620005	31062000562
CENTRO-SUL	310620005	BELO HORIZONT	3106200	310620005	31062000562
CENTRO-SUL	310620005	BELO HORIZONT	3106200	310620005	31062000562
CENTRO-SUL	310620005	BELO HORIZONT	3106200	310620005	31062000562
CENTRO-SUL	310620005	BELO HORIZONT	3106200	310620005	31062000562
CENTRO-SUL	310620005	BELO HORIZONT	3106200	310620005	31062000562
CENTRO-SUL	310620005	BELO HORIZONT	3106200	310620005	31062000562
CENTRO-SUL	310620005	BELO HORIZONT	3106200	310620005	31062000562
CENTRO-SUL	310620005	BELO HORIZONT	3106200	310620005	31062000562
CENTRO-SUL	310620005	BELO HORIZONT	3106200	310620005	31062000562
CENTRO-SUL	310620005	BELO HORIZONT	3106200	310620005	31062000562
CENTRO-SUL	310620005	BELO HORIZONT	3106200	310620005	31062000562
CENTRO-SUL	310620005	BELO HORIZONT	3106200	310620005	31062000562
CENTRO-SUL	310620005	BELO HORIZONT	3106200	310620005	31062000562
CENTRO-SUL	310620005	BELO HORIZONT	3106200	310620005	31062000562
CENTRO-SUL	310620005	BELO HORIZONT	3106200	310620005	31062000562
CENTRO-SUL	310620005	BELO HORIZONT	3106200	310620005	31062000562
CENTRO-SUL	310620005	BELO HORIZONT	3106200	310620005	31062000562
CENTRO-SUL	310620005	BELO HORIZONT	3106200	310620005	31062000562
CENTRO-SUL	310620005	BELO HORIZONT	3106200	310620005	31062000562
CENTRO-SUL	310620005	BELO HORIZONT	3106200	310620005	31062000562
CENTRO-SUL	310620005	BELO HORIZONT	3106200	310620005	31062000562
CENTRO-SUL	310620005	BELO HORIZONT	3106200	310620005	31062000562
CENTRO-SUL	310620005	BELO HORIZONT	3106200	310620005	31062000562
CENTRO-SUL	310620005	BELO HORIZONT	3106200	310620005	31062000562
CENTRO-SUL	310620005	BELO HORIZONT	3106200	310620005	31062000562
CENTRO-SUL	310620005	BELO HORIZONT	3106200	310620005	31062000562
CENTRO-SUL	310620005	BELO HORIZONT	3106200	310620005	31062000562
CENTRO-SUL	310620005	BELO HORIZONT	3106200	310620005	31062000562
CENTRO-SUL	310620005	BELO HORIZONT	3106200	310620005	31062000562
CENTRO-SUL	310620005	BELO HORIZONT	3106200	310620005	31062000562
CENTRO-SUL	310620005	BELO HORIZONT	3106200	0	0
CENTRO-SUL	310620005	BELO HORIZONT	3106200	310620005	31062000562
CENTRO-SUL	310620005	BELO HORIZONT	3106200	310620005	31062000562
CENTRO-SUL	310620005	BELO HORIZONT	3106200	310620005	31062000562
CENTRO-SUL	310620005	BELO HORIZONT	3106200	310620005	31062000562
CENTRO-SUL	310620005	BELO HORIZONT	3106200	310620005	31062000562
CENTRO-SUL	310620005	BELO HORIZONT	3106200	310620005	31062000562
CENTRO-SUL	310620005	BELO HORIZONT	3106200	310620005	31062000562
CENTRO-SUL	310620005	BELO HORIZONT	3106200	310620005	31062000562
CENTRO-SUL	310620005	BELO HORIZONT	3106200	310620005	31062000562
CENTRO-SUL	310620005	BELO HORIZONT	3106200	310620005	31062000562
CENTRO-SUL	310620005	BELO HORIZONT	3106200	310620005	31062000562
CENTRO-SUL	310620005	BELO HORIZONT	3106200	0	0
CENTRO-SUL	310620005	BELO HORIZONT	3106200	310620005	31062000562

CENTRO-SUL	310620005	BELO HORIZONT	3106200	310620005	31062000562
CENTRO-SUL	310620005	BELO HORIZONT	3106200	310620005	31062000562
CENTRO-SUL	310620005	BELO HORIZONT	3106200	310620005	31062000562
CENTRO-SUL	310620005	BELO HORIZONT	3106200	0	0
CENTRO-SUL	310620005	BELO HORIZONT	3106200	310620005	31062000562
CENTRO-SUL	310620005	BELO HORIZONT	3106200	310620005	31062000562
CENTRO-SUL	310620005	BELO HORIZONT	3106200	310620005	31062000562
CENTRO-SUL	310620005	BELO HORIZONT	3106200	310620005	31062000562
CENTRO-SUL	310620005	BELO HORIZONT	3106200	310620005	31062000562
CENTRO-SUL	310620005	BELO HORIZONT	3106200	310620005	31062000562
CENTRO-SUL	310620005	BELO HORIZONT	3106200	310620005	31062000562
CENTRO-SUL	310620005	BELO HORIZONT	3106200	310620005	31062000562
CENTRO-SUL	310620005	BELO HORIZONT	3106200	0	0
CENTRO-SUL	310620005	BELO HORIZONT	3106200	310620005	31062000562
CENTRO-SUL	310620005	BELO HORIZONT	3106200	310620005	31062000562
CENTRO-SUL	310620005	BELO HORIZONT	3106200	310620005	31062000562
CENTRO-SUL	310620005	BELO HORIZONT	3106200	310620005	31062000562
CENTRO-SUL	310620005	BELO HORIZONT	3106200	310620005	31062000562
CENTRO-SUL	310620005	BELO HORIZONT	3106200	310620005	31062000562
CENTRO-SUL	310620005	BELO HORIZONT	3106200	310620005	31062000562
CENTRO-SUL	310620005	BELO HORIZONT	3106200	310620005	31062000562
CENTRO-SUL	310620005	BELO HORIZONT	3106200	310620005	31062000562
CENTRO-SUL	310620005	BELO HORIZONT	3106200	310620005	31062000562
CENTRO-SUL	310620005	BELO HORIZONT	3106200	310620005	31062000562
CENTRO-SUL	310620005	BELO HORIZONT	3106200	310620005	31062000562
CENTRO-SUL	310620005	BELO HORIZONT	3106200	310620005	31062000562
CENTRO-SUL	310620005	BELO HORIZONT	3106200	310620005	31062000562
CENTRO-SUL	310620005	BELO HORIZONT	3106200	310620005	31062000562
CENTRO-SUL	310620005	BELO HORIZONT	3106200	310620005	31062000562
CENTRO-SUL	310620005	BELO HORIZONT	3106200	310620005	31062000562
CENTRO-SUL	310620005	BELO HORIZONT	3106200	310620005	31062000562
CENTRO-SUL	310620005	BELO HORIZONT	3106200	310620005	31062000562
CENTRO-SUL	310620005	BELO HORIZONT	3106200	310620005	31062000562
CENTRO-SUL	310620005	BELO HORIZONT	3106200	310620005	31062000562
CENTRO-SUL	310620005	BELO HORIZONT	3106200	310620005	31062000562
CENTRO-SUL	310620005	BELO HORIZONT	3106200	310620005	31062000562
CENTRO-SUL	310620005	BELO HORIZONT	3106200	310620005	31062000562
CENTRO-SUL	310620005	BELO HORIZONT	3106200	310620005	31062000562
CENTRO-SUL	310620005	BELO HORIZONT	3106200	310620005	31062000562
CENTRO-SUL	310620005	BELO HORIZONT	3106200	310620005	31062000562
CENTRO-SUL	310620005	BELO HORIZONT	3106200	310620005	31062000562
LESTE	310620005	BELO HORIZONT	3106200	310620005	31062000563
LESTE	310620005	BELO HORIZONT	3106200	310620005	31062000563
LESTE	310620005	BELO HORIZONT	3106200	310620005	31062000563
LESTE	310620005	BELO HORIZONT	3106200	310620005	31062000563
LESTE	310620005	BELO HORIZONT	3106200	310620005	31062000563
LESTE	310620005	BELO HORIZONT	3106200	310620005	31062000563
LESTE	310620005	BELO HORIZONT	3106200	310620005	31062000563
LESTE	310620005	BELO HORIZONT	3106200	310620005	31062000563
LESTE	310620005	BELO HORIZONT	3106200	310620005	31062000563

LESTE	310620005	BELO HORIZONT	3106200	310620005	31062000563
LESTE	310620005	BELO HORIZONT	3106200	310620005	31062000563
LESTE	310620005	BELO HORIZONT	3106200	310620005	31062000563
LESTE	310620005	BELO HORIZONT	3106200	310620005	31062000563
LESTE	310620005	BELO HORIZONT	3106200	310620005	31062000563
LESTE	310620005	BELO HORIZONT	3106200	310620005	31062000563
LESTE	310620005	BELO HORIZONT	3106200	310620005	31062000563
LESTE	310620005	BELO HORIZONT	3106200	310620005	31062000563
LESTE	310620005	BELO HORIZONT	3106200	310620005	31062000563
LESTE	310620005	BELO HORIZONT	3106200	310620005	31062000563
LESTE	310620005	BELO HORIZONT	3106200	310620005	31062000563
LESTE	310620005	BELO HORIZONT	3106200	310620005	31062000563
LESTE	310620005	BELO HORIZONT	3106200	310620005	31062000563
LESTE	310620005	BELO HORIZONT	3106200	310620005	31062000563
LESTE	310620005	BELO HORIZONT	3106200	310620005	31062000563
LESTE	310620005	BELO HORIZONT	3106200	310620005	31062000563
LESTE	310620005	BELO HORIZONT	3106200	310620005	31062000563
LESTE	310620005	BELO HORIZONT	3106200	310620005	31062000563
LESTE	310620005	BELO HORIZONT	3106200	310620005	31062000563
LESTE	310620005	BELO HORIZONT	3106200	310620005	31062000563
LESTE	310620005	BELO HORIZONT	3106200	310620005	31062000563
LESTE	310620005	BELO HORIZONT	3106200	310620005	31062000563
LESTE	310620005	BELO HORIZONT	3106200	310620005	31062000563
LESTE	310620005	BELO HORIZONT	3106200	310620005	31062000563
LESTE	310620005	BELO HORIZONT	3106200	310620005	31062000563
LESTE	310620005	BELO HORIZONT	3106200	310620005	31062000563
LESTE	310620005	BELO HORIZONT	3106200	310620005	31062000563
LESTE	310620005	BELO HORIZONT	3106200	310620005	31062000563
LESTE	310620005	BELO HORIZONT	3106200	310620005	31062000563
LESTE	310620005	BELO HORIZONT	3106200	310620005	31062000563
LESTE	310620005	BELO HORIZONT	3106200	310620005	31062000563
LESTE	310620005	BELO HORIZONT	3106200	310620005	31062000563
LESTE	310620005	BELO HORIZONT	3106200	310620005	31062000563
LESTE	310620005	BELO HORIZONT	3106200	310620005	31062000563
LESTE	310620005	BELO HORIZONT	3106200	310620005	31062000563
LESTE	310620005	BELO HORIZONT	3106200	310620005	31062000563
LESTE	310620005	BELO HORIZONT	3106200	310620005	31062000563
LESTE	310620005	BELO HORIZONT	3106200	310620005	31062000563
LESTE	310620005	BELO HORIZONT	3106200	310620005	31062000563
LESTE	310620005	BELO HORIZONT	3106200	310620005	31062000563
LESTE	310620005	BELO HORIZONT	3106200	310620005	31062000563
LESTE	310620005	BELO HORIZONT	3106200	310620005	31062000563
LESTE	310620005	BELO HORIZONT	3106200	310620005	31062000563
LESTE	310620005	BELO HORIZONT	3106200	310620005	31062000563
LESTE	310620005	BELO HORIZONT	3106200	310620005	31062000563
LESTE	310620005	BELO HORIZONT	3106200	310620005	31062000563
LESTE	310620005	BELO HORIZONT	3106200	310620005	31062000563
LESTE	310620005	BELO HORIZONT	3106200	310620005	31062000563
LESTE	310620005	BELO HORIZONT	3106200	310620005	31062000563
LESTE	310620005	BELO HORIZONT	3106200	310620005	31062000563

LESTE	310620005	BELO HORIZONT	3106200	310620005	31062000563
LESTE	310620005	BELO HORIZONT	3106200	310620005	31062000563
LESTE	310620005	BELO HORIZONT	3106200	310620005	31062000563
LESTE	310620005	BELO HORIZONT	3106200	310620005	31062000563
LESTE	310620005	BELO HORIZONT	3106200	310620005	31062000563
LESTE	310620005	BELO HORIZONT	3106200	310620005	31062000563
LESTE	310620005	BELO HORIZONT	3106200	310620005	31062000563
LESTE	310620005	BELO HORIZONT	3106200	310620005	31062000563
LESTE	310620005	BELO HORIZONT	3106200	310620005	31062000563
LESTE	310620005	BELO HORIZONT	3106200	310620005	31062000563
LESTE	310620005	BELO HORIZONT	3106200	310620005	31062000563
LESTE	310620005	BELO HORIZONT	3106200	310620005	31062000563
LESTE	310620005	BELO HORIZONT	3106200	310620005	31062000563
LESTE	310620005	BELO HORIZONT	3106200	310620005	31062000563
LESTE	310620005	BELO HORIZONT	3106200	310620005	31062000563
LESTE	310620005	BELO HORIZONT	3106200	310620005	31062000563
LESTE	310620005	BELO HORIZONT	3106200	310620005	31062000563
LESTE	310620005	BELO HORIZONT	3106200	310620005	31062000563
LESTE	310620005	BELO HORIZONT	3106200	310620005	31062000563
LESTE	310620005	BELO HORIZONT	3106200	310620005	31062000563
LESTE	310620005	BELO HORIZONT	3106200	310620005	31062000563
LESTE	310620005	BELO HORIZONT	3106200	310620005	31062000563
LESTE	310620005	BELO HORIZONT	3106200	310620005	31062000563
LESTE	310620005	BELO HORIZONT	3106200	310620005	31062000563
LESTE	310620005	BELO HORIZONT	3106200	310620005	31062000563
LESTE	310620005	BELO HORIZONT	3106200	310620005	31062000563
LESTE	310620005	BELO HORIZONT	3106200	310620005	31062000563
LESTE	310620005	BELO HORIZONT	3106200	310620005	31062000563
LESTE	310620005	BELO HORIZONT	3106200	310620005	31062000563
LESTE	310620005	BELO HORIZONT	3106200	310620005	31062000563
LESTE	310620005	BELO HORIZONT	3106200	310620005	31062000563
LESTE	310620005	BELO HORIZONT	3106200	310620005	31062000563
LESTE	310620005	BELO HORIZONT	3106200	310620005	31062000563
LESTE	310620005	BELO HORIZONT	3106200	310620005	31062000563
LESTE	310620005	BELO HORIZONT	3106200	310620005	31062000563
LESTE	310620005	BELO HORIZONT	3106200	310620005	31062000563
LESTE	310620005	BELO HORIZONT	3106200	310620005	31062000563
LESTE	310620005	BELO HORIZONT	3106200	310620005	31062000563
LESTE	310620005	BELO HORIZONT	3106200	310620005	31062000563
LESTE	310620005	BELO HORIZONT	3106200	310620005	31062000563
LESTE	310620005	BELO HORIZONT	3106200	310620005	31062000563
LESTE	310620005	BELO HORIZONT	3106200	310620005	31062000563
LESTE	310620005	BELO HORIZONT	3106200	310620005	31062000563
LESTE	310620005	BELO HORIZONT	3106200	310620005	31062000563
LESTE	310620005	BELO HORIZONT	3106200	310620005	31062000563
LESTE	310620005	BELO HORIZONT	3106200	310620005	31062000563
LESTE	310620005	BELO HORIZONT	3106200	310620005	31062000563
LESTE	310620005	BELO HORIZONT	3106200	310620005	31062000563
LESTE	310620005	BELO HORIZONT	3106200	310620005	31062000563
LESTE	310620005	BELO HORIZONT	3106200	310620005	31062000563

LESTE	310620005	BELO HORIZONT	3106200	310620005	31062000563
LESTE	310620005	BELO HORIZONT	3106200	310620005	31062000563
LESTE	310620005	BELO HORIZONT	3106200	310620005	31062000563
LESTE	310620005	BELO HORIZONT	3106200	310620005	31062000563
LESTE	310620005	BELO HORIZONT	3106200	310620005	31062000563
LESTE	310620005	BELO HORIZONT	3106200	310620005	31062000563
LESTE	310620005	BELO HORIZONT	3106200	310620005	31062000563
LESTE	310620005	BELO HORIZONT	3106200	310620005	31062000563
LESTE	310620005	BELO HORIZONT	3106200	310620005	31062000563
LESTE	310620005	BELO HORIZONT	3106200	310620005	31062000563
LESTE	310620005	BELO HORIZONT	3106200	310620005	31062000563
LESTE	310620005	BELO HORIZONT	3106200	310620005	31062000563
LESTE	310620005	BELO HORIZONT	3106200	310620005	31062000563
LESTE	310620005	BELO HORIZONT	3106200	310620005	31062000563
LESTE	310620005	BELO HORIZONT	3106200	310620005	31062000563
LESTE	310620005	BELO HORIZONT	3106200	310620005	31062000563
LESTE	310620005	BELO HORIZONT	3106200	310620005	31062000563
LESTE	310620005	BELO HORIZONT	3106200	310620005	31062000563
LESTE	310620005	BELO HORIZONT	3106200	310620005	31062000563
LESTE	310620005	BELO HORIZONT	3106200	310620005	31062000563
LESTE	310620005	BELO HORIZONT	3106200	310620005	31062000563
LESTE	310620005	BELO HORIZONT	3106200	310620005	31062000563
LESTE	310620005	BELO HORIZONT	3106200	310620005	31062000563
LESTE	310620005	BELO HORIZONT	3106200	310620005	31062000563
LESTE	310620005	BELO HORIZONT	3106200	310620005	31062000563
LESTE	310620005	BELO HORIZONT	3106200	310620005	31062000563
LESTE	310620005	BELO HORIZONT	3106200	310620005	31062000563
LESTE	310620005	BELO HORIZONT	3106200	310620005	31062000563
LESTE	310620005	BELO HORIZONT	3106200	310620005	31062000563
LESTE	310620005	BELO HORIZONT	3106200	310620005	31062000563
LESTE	310620005	BELO HORIZONT	3106200	310620005	31062000563
LESTE	310620005	BELO HORIZONT	3106200	310620005	31062000563
LESTE	310620005	BELO HORIZONT	3106200	310620005	31062000563
LESTE	310620005	BELO HORIZONT	3106200	310620005	31062000563
LESTE	310620005	BELO HORIZONT	3106200	310620005	31062000563
LESTE	310620005	BELO HORIZONT	3106200	310620005	31062000563
LESTE	310620005	BELO HORIZONT	3106200	310620005	31062000563
LESTE	310620005	BELO HORIZONT	3106200	310620005	31062000563
LESTE	310620005	BELO HORIZONT	3106200	310620005	31062000563
LESTE	310620005	BELO HORIZONT	3106200	310620005	31062000563
LESTE	310620005	BELO HORIZONT	3106200	310620005	31062000563
LESTE	310620005	BELO HORIZONT	3106200	310620005	31062000563
LESTE	310620005	BELO HORIZONT	3106200	310620005	31062000563
LESTE	310620005	BELO HORIZONT	3106200	310620005	31062000563
LESTE	310620005	BELO HORIZONT	3106200	310620005	31062000563
LESTE	310620005	BELO HORIZONT	3106200	310620005	31062000563
LESTE	310620005	BELO HORIZONT	3106200	310620005	31062000563
LESTE	310620005	BELO HORIZONT	3106200	310620005	31062000563
LESTE	310620005	BELO HORIZONT	3106200	310620005	31062000563
LESTE	310620005	BELO HORIZONT	3106200	310620005	31062000563

LESTE	310620005	BELO HORIZONT	3106200	310620005	31062000563
LESTE	310620005	BELO HORIZONT	3106200	310620005	31062000563
LESTE	310620005	BELO HORIZONT	3106200	310620005	31062000563
LESTE	310620005	BELO HORIZONT	3106200	310620005	31062000563
LESTE	310620005	BELO HORIZONT	3106200	310620005	31062000563
LESTE	310620005	BELO HORIZONT	3106200	310620005	31062000563
LESTE	310620005	BELO HORIZONT	3106200	310620005	31062000563
LESTE	310620005	BELO HORIZONT	3106200	310620005	31062000563
LESTE	310620005	BELO HORIZONT	3106200	310620005	31062000563
LESTE	310620005	BELO HORIZONT	3106200	310620005	31062000563
LESTE	310620005	BELO HORIZONT	3106200	310620005	31062000563
LESTE	310620005	BELO HORIZONT	3106200	310620005	31062000563
LESTE	310620005	BELO HORIZONT	3106200	310620005	31062000563
LESTE	310620005	BELO HORIZONT	3106200	310620005	31062000563
LESTE	310620005	BELO HORIZONT	3106200	310620005	31062000563
LESTE	310620005	BELO HORIZONT	3106200	310620005	31062000563
LESTE	310620005	BELO HORIZONT	3106200	310620005	31062000563
LESTE	310620005	BELO HORIZONT	3106200	310620005	31062000563
LESTE	310620005	BELO HORIZONT	3106200	310620005	31062000563
LESTE	310620005	BELO HORIZONT	3106200	310620005	31062000563
LESTE	310620005	BELO HORIZONT	3106200	310620005	31062000563
LESTE	310620005	BELO HORIZONT	3106200	310620005	31062000563
LESTE	310620005	BELO HORIZONT	3106200	310620005	31062000563
LESTE	310620005	BELO HORIZONT	3106200	310620005	31062000563
LESTE	310620005	BELO HORIZONT	3106200	310620005	31062000563
LESTE	310620005	BELO HORIZONT	3106200	310620005	31062000563
LESTE	310620005	BELO HORIZONT	3106200	310620005	31062000563
LESTE	310620005	BELO HORIZONT	3106200	310620005	31062000563
LESTE	310620005	BELO HORIZONT	3106200	310620005	31062000563
LESTE	310620005	BELO HORIZONT	3106200	310620005	31062000563
LESTE	310620005	BELO HORIZONT	3106200	310620005	31062000563
LESTE	310620005	BELO HORIZONT	3106200	310620005	31062000563
LESTE	310620005	BELO HORIZONT	3106200	310620005	31062000563
LESTE	310620005	BELO HORIZONT	3106200	310620005	31062000563
LESTE	310620005	BELO HORIZONT	3106200	310620005	31062000563
LESTE	310620005	BELO HORIZONT	3106200	310620005	31062000563
LESTE	310620005	BELO HORIZONT	3106200	310620005	31062000563
LESTE	310620005	BELO HORIZONT	3106200	310620005	31062000563
LESTE	310620005	BELO HORIZONT	3106200	310620005	31062000563
LESTE	310620005	BELO HORIZONT	3106200	310620005	31062000563
LESTE	310620005	BELO HORIZONT	3106200	310620005	31062000563
LESTE	310620005	BELO HORIZONT	3106200	310620005	31062000563
LESTE	310620005	BELO HORIZONT	3106200	310620005	31062000563
LESTE	310620005	BELO HORIZONT	3106200	310620005	31062000563
LESTE	310620005	BELO HORIZONT	3106200	310620005	31062000563
LESTE	310620005	BELO HORIZONT	3106200	310620005	31062000563
LESTE	310620005	BELO HORIZONT	3106200	310620005	31062000563
LESTE	310620005	BELO HORIZONT	3106200	310620005	31062000563
LESTE	310620005	BELO HORIZONT	3106200	310620005	31062000563
LESTE	310620005	BELO HORIZONT	3106200	310620005	31062000563

LESTE	310620005	BELO HORIZONT	3106200	310620005	31062000563
LESTE	310620005	BELO HORIZONT	3106200	310620005	31062000563
LESTE	310620005	BELO HORIZONT	3106200	310620005	31062000563
LESTE	310620005	BELO HORIZONT	3106200	310620005	31062000563
LESTE	310620005	BELO HORIZONT	3106200	310620005	31062000563
LESTE	310620005	BELO HORIZONT	3106200	310620005	31062000563
LESTE	310620005	BELO HORIZONT	3106200	310620005	31062000563
LESTE	310620005	BELO HORIZONT	3106200	310620005	31062000563
LESTE	310620005	BELO HORIZONT	3106200	310620005	31062000563
LESTE	310620005	BELO HORIZONT	3106200	310620005	31062000563
LESTE	310620005	BELO HORIZONT	3106200	310620005	31062000563
LESTE	310620005	BELO HORIZONT	3106200	310620005	31062000563
LESTE	310620005	BELO HORIZONT	3106200	310620005	31062000563
LESTE	310620005	BELO HORIZONT	3106200	310620005	31062000563
LESTE	310620005	BELO HORIZONT	3106200	310620005	31062000563
LESTE	310620005	BELO HORIZONT	3106200	310620005	31062000563
LESTE	310620005	BELO HORIZONT	3106200	310620005	31062000563
LESTE	310620005	BELO HORIZONT	3106200	310620005	31062000563
LESTE	310620005	BELO HORIZONT	3106200	310620005	31062000563
LESTE	310620005	BELO HORIZONT	3106200	310620005	31062000563
LESTE	310620005	BELO HORIZONT	3106200	310620005	31062000563
LESTE	310620005	BELO HORIZONT	3106200	310620005	31062000563
LESTE	310620005	BELO HORIZONT	3106200	310620005	31062000563
LESTE	310620005	BELO HORIZONT	3106200	310620005	31062000563
LESTE	310620005	BELO HORIZONT	3106200	310620005	31062000563
LESTE	310620005	BELO HORIZONT	3106200	310620005	31062000563
LESTE	310620005	BELO HORIZONT	3106200	310620005	31062000563
LESTE	310620005	BELO HORIZONT	3106200	310620005	31062000563
LESTE	310620005	BELO HORIZONT	3106200	310620005	31062000563
LESTE	310620005	BELO HORIZONT	3106200	310620005	31062000563
LESTE	310620005	BELO HORIZONT	3106200	310620005	31062000563
LESTE	310620005	BELO HORIZONT	3106200	310620005	31062000563
LESTE	310620005	BELO HORIZONT	3106200	310620005	31062000563
LESTE	310620005	BELO HORIZONT	3106200	310620005	31062000563
LESTE	310620005	BELO HORIZONT	3106200	310620005	31062000563
LESTE	310620005	BELO HORIZONT	3106200	310620005	31062000563
LESTE	310620005	BELO HORIZONT	3106200	310620005	31062000563
LESTE	310620005	BELO HORIZONT	3106200	310620005	31062000563
LESTE	310620005	BELO HORIZONT	3106200	310620005	31062000563
LESTE	310620005	BELO HORIZONT	3106200	310620005	31062000563
LESTE	310620005	BELO HORIZONT	3106200	310620005	31062000563
LESTE	310620005	BELO HORIZONT	3106200	310620005	31062000563
LESTE	310620005	BELO HORIZONT	3106200	310620005	31062000563
LESTE	310620005	BELO HORIZONT	3106200	310620005	31062000563
LESTE	310620005	BELO HORIZONT	3106200	310620005	31062000563
LESTE	310620005	BELO HORIZONT	3106200	310620005	31062000563
LESTE	310620005	BELO HORIZONT	3106200	310620005	31062000563
LESTE	310620005	BELO HORIZONT	3106200	310620005	31062000563
LESTE	310620005	BELO HORIZONT	3106200	310620005	31062000563
LESTE	310620005	BELO HORIZONT	3106200	310620005	31062000563

LESTE	310620005	BELO HORIZONT	3106200	310620005	31062000563
LESTE	310620005	BELO HORIZONT	3106200	310620005	31062000563
LESTE	310620005	BELO HORIZONT	3106200	310620005	31062000563
LESTE	310620005	BELO HORIZONT	3106200	310620005	31062000563
LESTE	310620005	BELO HORIZONT	3106200	310620005	31062000563
LESTE	310620005	BELO HORIZONT	3106200	310620005	31062000563
LESTE	310620005	BELO HORIZONT	3106200	310620005	31062000563
LESTE	310620005	BELO HORIZONT	3106200	310620005	31062000563
LESTE	310620005	BELO HORIZONT	3106200	310620005	31062000563
LESTE	310620005	BELO HORIZONT	3106200	310620005	31062000563
LESTE	310620005	BELO HORIZONT	3106200	310620005	31062000563
LESTE	310620005	BELO HORIZONT	3106200	310620005	31062000563
LESTE	310620005	BELO HORIZONT	3106200	310620005	31062000563
LESTE	310620005	BELO HORIZONT	3106200	310620005	31062000563
LESTE	310620005	BELO HORIZONT	3106200	310620005	31062000563
LESTE	310620005	BELO HORIZONT	3106200	310620005	31062000563
LESTE	310620005	BELO HORIZONT	3106200	310620005	31062000563
LESTE	310620005	BELO HORIZONT	3106200	310620005	31062000563
LESTE	310620005	BELO HORIZONT	3106200	310620005	31062000563
LESTE	310620005	BELO HORIZONT	3106200	310620005	31062000563
LESTE	310620005	BELO HORIZONT	3106200	310620005	31062000563
LESTE	310620005	BELO HORIZONT	3106200	310620005	31062000563
LESTE	310620005	BELO HORIZONT	3106200	310620005	31062000563
LESTE	310620005	BELO HORIZONT	3106200	310620005	31062000563
LESTE	310620005	BELO HORIZONT	3106200	310620005	31062000563
LESTE	310620005	BELO HORIZONT	3106200	310620005	31062000563
LESTE	310620005	BELO HORIZONT	3106200	310620005	31062000563
LESTE	310620005	BELO HORIZONT	3106200	310620005	31062000563
LESTE	310620005	BELO HORIZONT	3106200	310620005	31062000563
LESTE	310620005	BELO HORIZONT	3106200	310620005	31062000563
LESTE	310620005	BELO HORIZONT	3106200	310620005	31062000563
LESTE	310620005	BELO HORIZONT	3106200	310620005	31062000563
LESTE	310620005	BELO HORIZONT	3106200	310620005	31062000563
LESTE	310620005	BELO HORIZONT	3106200	310620005	31062000563
LESTE	310620005	BELO HORIZONT	3106200	310620005	31062000563
LESTE	310620005	BELO HORIZONT	3106200	0	0
LESTE	310620005	BELO HORIZONT	3106200	310620005	31062000563
LESTE	310620005	BELO HORIZONT	3106200	310620005	31062000563
LESTE	310620005	BELO HORIZONT	3106200	310620005	31062000563
LESTE	310620005	BELO HORIZONT	3106200	310620005	31062000563
LESTE	310620005	BELO HORIZONT	3106200	310620005	31062000563
LESTE	310620005	BELO HORIZONT	3106200	0	0
LESTE	310620005	BELO HORIZONT	3106200	310620005	31062000563
LESTE	310620005	BELO HORIZONT	3106200	310620005	31062000563
LESTE	310620005	BELO HORIZONT	3106200	310620005	31062000563
LESTE	310620005	BELO HORIZONT	3106200	310620005	31062000563
LESTE	310620005	BELO HORIZONT	3106200	310620005	31062000563
LESTE	310620005	BELO HORIZONT	3106200	310620005	31062000563
LESTE	310620005	BELO HORIZONT	3106200	310620005	31062000563
LESTE	310620005	BELO HORIZONT	3106200	310620005	31062000563

LESTE	310620005	BELO HORIZONT	3106200	310620005	31062000563
LESTE	310620005	BELO HORIZONT	3106200	310620005	31062000563
LESTE	310620005	BELO HORIZONT	3106200	310620005	31062000563
LESTE	310620005	BELO HORIZONT	3106200	310620005	31062000563
LESTE	310620005	BELO HORIZONT	3106200	310620005	31062000563
LESTE	310620005	BELO HORIZONT	3106200	310620005	31062000563
LESTE	310620005	BELO HORIZONT	3106200	310620005	31062000563
LESTE	310620005	BELO HORIZONT	3106200	310620005	31062000563
LESTE	310620005	BELO HORIZONT	3106200	310620005	31062000563
LESTE	310620005	BELO HORIZONT	3106200	310620005	31062000563
LESTE	310620005	BELO HORIZONT	3106200	310620005	31062000563
LESTE	310620005	BELO HORIZONT	3106200	310620005	31062000563
LESTE	310620005	BELO HORIZONT	3106200	310620005	31062000563
LESTE	310620005	BELO HORIZONT	3106200	310620005	31062000563
LESTE	310620005	BELO HORIZONT	3106200	310620005	31062000563
LESTE	310620005	BELO HORIZONT	3106200	310620005	31062000563
LESTE	310620005	BELO HORIZONT	3106200	310620005	31062000563
LESTE	310620005	BELO HORIZONT	3106200	310620005	31062000563
LESTE	310620005	BELO HORIZONT	3106200	310620005	31062000563
LESTE	310620005	BELO HORIZONT	3106200	310620005	31062000563
LESTE	310620005	BELO HORIZONT	3106200	310620005	31062000563
LESTE	310620005	BELO HORIZONT	3106200	310620005	31062000563
LESTE	310620005	BELO HORIZONT	3106200	310620005	31062000563
LESTE	310620005	BELO HORIZONT	3106200	310620005	31062000563
LESTE	310620005	BELO HORIZONT	3106200	310620005	31062000563
LESTE	310620005	BELO HORIZONT	3106200	310620005	31062000563
LESTE	310620005	BELO HORIZONT	3106200	310620005	31062000563
LESTE	310620005	BELO HORIZONT	3106200	310620005	31062000563
LESTE	310620005	BELO HORIZONT	3106200	310620005	31062000563
LESTE	310620005	BELO HORIZONT	3106200	310620005	31062000563
LESTE	310620005	BELO HORIZONT	3106200	310620005	31062000563
LESTE	310620005	BELO HORIZONT	3106200	310620005	31062000563
LESTE	310620005	BELO HORIZONT	3106200	310620005	31062000563
LESTE	310620005	BELO HORIZONT	3106200	310620005	31062000563
LESTE	310620005	BELO HORIZONT	3106200	310620005	31062000563
LESTE	310620005	BELO HORIZONT	3106200	310620005	31062000563
LESTE	310620005	BELO HORIZONT	3106200	310620005	31062000563
LESTE	310620005	BELO HORIZONT	3106200	310620005	31062000563
LESTE	310620005	BELO HORIZONT	3106200	310620005	31062000563
LESTE	310620005	BELO HORIZONT	3106200	310620005	31062000563
LESTE	310620005	BELO HORIZONT	3106200	310620005	31062000563
LESTE	310620005	BELO HORIZONT	3106200	310620005	31062000563
LESTE	310620005	BELO HORIZONT	3106200	310620005	31062000563
LESTE	310620005	BELO HORIZONT	3106200	310620005	31062000563
LESTE	310620005	BELO HORIZONT	3106200	310620005	31062000563
LESTE	310620005	BELO HORIZONT	3106200	310620005	31062000563
LESTE	310620005	BELO HORIZONT	3106200	310620005	31062000563
LESTE	310620005	BELO HORIZONT	3106200	310620005	31062000563
LESTE	310620005	BELO HORIZONT	3106200	310620005	31062000563
LESTE	310620005	BELO HORIZONT	3106200	310620005	31062000563

LESTE	310620005	BELO HORIZONT	3106200	310620005	31062000563
LESTE	310620005	BELO HORIZONT	3106200	310620005	31062000563
LESTE	310620005	BELO HORIZONT	3106200	310620005	31062000563
LESTE	310620005	BELO HORIZONT	3106200	310620005	31062000563
LESTE	310620005	BELO HORIZONT	3106200	310620005	31062000563
LESTE	310620005	BELO HORIZONT	3106200	310620005	31062000563
LESTE	310620005	BELO HORIZONT	3106200	310620005	31062000563
LESTE	310620005	BELO HORIZONT	3106200	310620005	31062000563
LESTE	310620005	BELO HORIZONT	3106200	310620005	31062000563
LESTE	310620005	BELO HORIZONT	3106200	310620005	31062000563
LESTE	310620005	BELO HORIZONT	3106200	310620005	31062000563
LESTE	310620005	BELO HORIZONT	3106200	310620005	31062000563
LESTE	310620005	BELO HORIZONT	3106200	310620005	31062000563
LESTE	310620005	BELO HORIZONT	3106200	310620005	31062000563
LESTE	310620005	BELO HORIZONT	3106200	310620005	31062000563
LESTE	310620005	BELO HORIZONT	3106200	310620005	31062000563
LESTE	310620005	BELO HORIZONT	3106200	310620005	31062000563
LESTE	310620005	BELO HORIZONT	3106200	310620005	31062000563
LESTE	310620005	BELO HORIZONT	3106200	310620005	31062000563
LESTE	310620005	BELO HORIZONT	3106200	310620005	31062000563
LESTE	310620005	BELO HORIZONT	3106200	310620005	31062000563
NOROESTE	310620005	BELO HORIZONT	3106200	310620005	31062000565
NOROESTE	310620005	BELO HORIZONT	3106200	310620005	31062000565
NOROESTE	310620005	BELO HORIZONT	3106200	310620005	31062000565
NOROESTE	310620005	BELO HORIZONT	3106200	310620005	31062000565
NOROESTE	310620005	BELO HORIZONT	3106200	310620005	31062000565
NOROESTE	310620005	BELO HORIZONT	3106200	310620005	31062000565
NOROESTE	310620005	BELO HORIZONT	3106200	310620005	31062000565
NOROESTE	310620005	BELO HORIZONT	3106200	310620005	31062000565
NOROESTE	310620005	BELO HORIZONT	3106200	310620005	31062000565
NOROESTE	310620005	BELO HORIZONT	3106200	310620005	31062000565
NOROESTE	310620005	BELO HORIZONT	3106200	310620005	31062000565
NOROESTE	310620005	BELO HORIZONT	3106200	310620005	31062000565
NOROESTE	310620005	BELO HORIZONT	3106200	310620005	31062000565
NOROESTE	310620005	BELO HORIZONT	3106200	310620005	31062000565
NOROESTE	310620005	BELO HORIZONT	3106200	310620005	31062000565
NOROESTE	310620005	BELO HORIZONT	3106200	310620005	31062000565
NOROESTE	310620005	BELO HORIZONT	3106200	310620005	31062000565
NOROESTE	310620005	BELO HORIZONT	3106200	310620005	31062000565
NOROESTE	310620005	BELO HORIZONT	3106200	310620005	31062000565
NOROESTE	310620005	BELO HORIZONT	3106200	310620005	31062000565
NOROESTE	310620005	BELO HORIZONT	3106200	310620005	31062000565
NOROESTE	310620005	BELO HORIZONT	3106200	310620005	31062000565
NOROESTE	310620005	BELO HORIZONT	3106200	310620005	31062000565
NOROESTE	310620005	BELO HORIZONT	3106200	310620005	31062000565
NOROESTE	310620005	BELO HORIZONT	3106200	310620005	31062000565
NOROESTE	310620005	BELO HORIZONT	3106200	310620005	31062000565
NOROESTE	310620005	BELO HORIZONT	3106200	310620005	31062000565
NOROESTE	310620005	BELO HORIZONT	3106200	310620005	31062000565
NOROESTE	310620005	BELO HORIZONT	3106200	310620005	31062000565

NOROESTE	310620005	BELO HORIZONT	3106200	310620005	31062000565
OESTE	310620005	BELO HORIZONT	3106200	310620005	31062000567
PAMPULHA	310620005	BELO HORIZONT	3106200	0	0
PAMPULHA	310620005	BELO HORIZONT	3106200	310620005	31062000568
PAMPULHA	310620005	BELO HORIZONT	3106200	310620005	31062000568
PAMPULHA	310620005	BELO HORIZONT	3106200	310620005	31062000568
PAMPULHA	310620005	BELO HORIZONT	3106200	310620005	31062000568
PAMPULHA	310620005	BELO HORIZONT	3106200	310620005	31062000568
PAMPULHA	310620005	BELO HORIZONT	3106200	310620005	31062000568
PAMPULHA	310620005	BELO HORIZONT	3106200	310620005	31062000568
PAMPULHA	310620005	BELO HORIZONT	3106200	310620005	31062000568
PAMPULHA	310620005	BELO HORIZONT	3106200	310620005	31062000568
BARREIRO	310620025	BARREIRO	3106200	310620025	31062002561
BARREIRO	310620025	BARREIRO	3106200	310620025	31062002561
BARREIRO	310620025	BARREIRO	3106200	310620025	31062002561
BARREIRO	310620025	BARREIRO	3106200	310620025	31062002561
BARREIRO	310620025	BARREIRO	3106200	310620025	31062002561
BARREIRO	310620025	BARREIRO	3106200	310620025	31062002561
BARREIRO	310620025	BARREIRO	3106200	310620025	31062002561
BARREIRO	310620025	BARREIRO	3106200	310620025	31062002561
BARREIRO	310620025	BARREIRO	3106200	310620025	31062002561
BARREIRO	310620025	BARREIRO	3106200	310620025	31062002561
BARREIRO	310620025	BARREIRO	3106200	310620025	31062002561
BARREIRO	310620025	BARREIRO	3106200	310620025	31062002561
BARREIRO	310620025	BARREIRO	3106200	310620025	31062002561
BARREIRO	310620025	BARREIRO	3106200	310620025	31062002561
BARREIRO	310620025	BARREIRO	3106200	310620025	31062002561
BARREIRO	310620025	BARREIRO	3106200	310620025	31062002561
BARREIRO	310620025	BARREIRO	3106200	310620025	31062002561
BARREIRO	310620025	BARREIRO	3106200	310620025	31062002561
BARREIRO	310620025	BARREIRO	3106200	310620025	31062002561
BARREIRO	310620025	BARREIRO	3106200	310620025	31062002561
BARREIRO	310620025	BARREIRO	3106200	310620025	31062002561
BARREIRO	310620025	BARREIRO	3106200	310620025	31062002561
NORDESTE	310620060	VENDA NOVA	3106200	310620060	31062006064
NORDESTE	310620060	VENDA NOVA	3106200	310620060	31062006064
NORDESTE	310620060	VENDA NOVA	3106200	310620060	31062006064
NORDESTE	310620060	VENDA NOVA	3106200	310620060	31062006064
NORDESTE	310620060	VENDA NOVA	3106200	310620060	31062006064
NORDESTE	310620060	VENDA NOVA	3106200	310620060	31062006064
NORDESTE	310620060	VENDA NOVA	3106200	310620060	31062006064
NORDESTE	310620060	VENDA NOVA	3106200	310620060	31062006064
NORDESTE	310620060	VENDA NOVA	3106200	310620060	31062006064
NORDESTE	310620060	VENDA NOVA	3106200	310620060	31062006064
NORDESTE	310620060	VENDA NOVA	3106200	310620060	31062006064
LESTE	310620005	BELO HORIZONT	3106200	310620005	31062000563
LESTE	310620005	BELO HORIZONT	3106200	310620005	31062000563
LESTE	310620005	BELO HORIZONT	3106200	310620005	31062000563
LESTE	310620005	BELO HORIZONT	3106200	310620005	31062000563
LESTE	310620005	BELO HORIZONT	3106200	0	0

LESTE	310620005	BELO HORIZONT	3106200	310620005	31062000563
LESTE	310620005	BELO HORIZONT	3106200	310620005	31062000563
LESTE	310620005	BELO HORIZONT	3106200	310620005	31062000563
LESTE	310620005	BELO HORIZONT	3106200	310620005	31062000563
LESTE	310620005	BELO HORIZONT	3106200	310620005	31062000563
LESTE	310620005	BELO HORIZONT	3106200	310620005	31062000563
LESTE	310620005	BELO HORIZONT	3106200	310620005	31062000563
NORDESTE	310620005	BELO HORIZONT	3106200	310620005	31062000564
NORDESTE	310620005	BELO HORIZONT	3106200	310620005	31062000564
NORDESTE	310620005	BELO HORIZONT	3106200	310620005	31062000564
NORDESTE	310620005	BELO HORIZONT	3106200	310620005	31062000564
NORDESTE	310620005	BELO HORIZONT	3106200	310620005	31062000564
NORDESTE	310620005	BELO HORIZONT	3106200	310620005	31062000564
NORDESTE	310620005	BELO HORIZONT	3106200	310620005	31062000564
NORDESTE	310620005	BELO HORIZONT	3106200	310620005	31062000564
NORDESTE	310620005	BELO HORIZONT	3106200	310620005	31062000564
NORDESTE	310620005	BELO HORIZONT	3106200	310620005	31062000564
NORDESTE	310620005	BELO HORIZONT	3106200	310620005	31062000564
NORDESTE	310620005	BELO HORIZONT	3106200	310620005	31062000564
NORDESTE	310620005	BELO HORIZONT	3106200	310620005	31062000564
NORDESTE	310620005	BELO HORIZONT	3106200	310620005	31062000564
NORDESTE	310620005	BELO HORIZONT	3106200	310620005	31062000564
NORDESTE	310620005	BELO HORIZONT	3106200	310620005	31062000564
NORDESTE	310620005	BELO HORIZONT	3106200	310620005	31062000564
NORDESTE	310620005	BELO HORIZONT	3106200	310620005	31062000564
NORDESTE	310620005	BELO HORIZONT	3106200	310620005	31062000564
NORDESTE	310620005	BELO HORIZONT	3106200	310620005	31062000564
NORDESTE	310620005	BELO HORIZONT	3106200	310620005	31062000564
NORDESTE	310620005	BELO HORIZONT	3106200	310620005	31062000564
NORDESTE	310620005	BELO HORIZONT	3106200	310620005	31062000564
NORDESTE	310620005	BELO HORIZONT	3106200	310620005	31062000564
NORDESTE	310620005	BELO HORIZONT	3106200	310620005	31062000564
NORDESTE	310620005	BELO HORIZONT	3106200	310620005	31062000564
NORDESTE	310620005	BELO HORIZONT	3106200	310620005	31062000564
NORDESTE	310620005	BELO HORIZONT	3106200	310620005	31062000564
NORDESTE	310620005	BELO HORIZONT	3106200	310620005	31062000564
NORDESTE	310620005	BELO HORIZONT	3106200	310620005	31062000564
NORDESTE	310620005	BELO HORIZONT	3106200	310620005	31062000564
NORDESTE	310620005	BELO HORIZONT	3106200	310620005	31062000564
NORDESTE	310620005	BELO HORIZONT	3106200	310620005	31062000564
NORDESTE	310620005	BELO HORIZONT	3106200	310620005	31062000564
NORDESTE	310620005	BELO HORIZONT	3106200	310620005	31062000564
NORDESTE	310620005	BELO HORIZONT	3106200	310620005	31062000564
NORDESTE	310620005	BELO HORIZONT	3106200	310620005	31062000564
NORDESTE	310620005	BELO HORIZONT	3106200	310620005	31062000564
NORDESTE	310620005	BELO HORIZONT	3106200	310620005	31062000564
NORDESTE	310620005	BELO HORIZONT	3106200	310620005	31062000564
NORDESTE	310620005	BELO HORIZONT	3106200	310620005	31062000564
NORDESTE	310620005	BELO HORIZONT	3106200	310620005	31062000564
NORDESTE	310620005	BELO HORIZONT	3106200	310620005	31062000564

NORDESTE	310620005	BELO HORIZONT	3106200	310620005	31062000564
NORDESTE	310620005	BELO HORIZONT	3106200	310620005	31062000564
NORDESTE	310620005	BELO HORIZONT	3106200	310620005	31062000564
NORDESTE	310620005	BELO HORIZONT	3106200	310620005	31062000564
NORDESTE	310620005	BELO HORIZONT	3106200	310620005	31062000564
NORDESTE	310620005	BELO HORIZONT	3106200	310620005	31062000564
NORDESTE	310620005	BELO HORIZONT	3106200	310620005	31062000564
NORDESTE	310620005	BELO HORIZONT	3106200	310620005	31062000564
NORDESTE	310620005	BELO HORIZONT	3106200	310620005	31062000564
NORDESTE	310620005	BELO HORIZONT	3106200	310620005	31062000564
NORDESTE	310620005	BELO HORIZONT	3106200	310620005	31062000564
NORDESTE	310620005	BELO HORIZONT	3106200	310620005	31062000564
NORDESTE	310620005	BELO HORIZONT	3106200	310620005	31062000564
NORDESTE	310620005	BELO HORIZONT	3106200	310620005	31062000564
NORDESTE	310620005	BELO HORIZONT	3106200	310620005	31062000564
NORDESTE	310620005	BELO HORIZONT	3106200	310620005	31062000564
NORDESTE	310620005	BELO HORIZONT	3106200	310620005	31062000564
NORDESTE	310620005	BELO HORIZONT	3106200	310620005	31062000564
NORDESTE	310620005	BELO HORIZONT	3106200	310620005	31062000564
NORDESTE	310620005	BELO HORIZONT	3106200	310620005	31062000564
NORDESTE	310620005	BELO HORIZONT	3106200	310620005	31062000564
NORDESTE	310620005	BELO HORIZONT	3106200	310620005	31062000564
NORDESTE	310620005	BELO HORIZONT	3106200	310620005	31062000564
NORDESTE	310620005	BELO HORIZONT	3106200	310620005	31062000564
NORDESTE	310620005	BELO HORIZONT	3106200	310620005	31062000564
NORDESTE	310620005	BELO HORIZONT	3106200	310620005	31062000564
NORDESTE	310620005	BELO HORIZONT	3106200	310620005	31062000564
NORDESTE	310620005	BELO HORIZONT	3106200	310620005	31062000564
NORDESTE	310620005	BELO HORIZONT	3106200	310620005	31062000564
NORDESTE	310620005	BELO HORIZONT	3106200	310620005	31062000564
NORDESTE	310620005	BELO HORIZONT	3106200	310620005	31062000564
NORDESTE	310620005	BELO HORIZONT	3106200	310620005	31062000564
NORDESTE	310620005	BELO HORIZONT	3106200	310620005	31062000564
NORDESTE	310620005	BELO HORIZONT	3106200	310620005	31062000564
NORDESTE	310620005	BELO HORIZONT	3106200	310620005	31062000564
NORDESTE	310620005	BELO HORIZONT	3106200	310620005	31062000564
NORDESTE	310620005	BELO HORIZONT	3106200	310620005	31062000564
NORDESTE	310620005	BELO HORIZONT	3106200	310620005	31062000564
NORDESTE	310620005	BELO HORIZONT	3106200	310620005	31062000564
NORDESTE	310620005	BELO HORIZONT	3106200	310620005	31062000564
NORDESTE	310620005	BELO HORIZONT	3106200	310620005	31062000564
NORDESTE	310620005	BELO HORIZONT	3106200	310620005	31062000564
NORDESTE	310620005	BELO HORIZONT	3106200	310620005	31062000564
NORDESTE	310620005	BELO HORIZONT	3106200	310620005	31062000564
NORDESTE	310620005	BELO HORIZONT	3106200	310620005	31062000564
NORDESTE	310620005	BELO HORIZONT	3106200	310620005	31062000564
NORDESTE	310620005	BELO HORIZONT	3106200	310620005	31062000564
NORDESTE	310620005	BELO HORIZONT	3106200	310620005	31062000564
NORDESTE	310620005	BELO HORIZONT	3106200	310620005	31062000564
NORDESTE	310620005	BELO HORIZONT	3106200	310620005	31062000564

NORDESTE	310620005	BELO HORIZONT	3106200	310620005	31062000564
NORDESTE	310620005	BELO HORIZONT	3106200	310620005	31062000564
NORDESTE	310620005	BELO HORIZONT	3106200	310620005	31062000564
NORDESTE	310620005	BELO HORIZONT	3106200	310620005	31062000564
NORDESTE	310620005	BELO HORIZONT	3106200	310620005	31062000564
NORDESTE	310620005	BELO HORIZONT	3106200	310620005	31062000564
NORDESTE	310620005	BELO HORIZONT	3106200	310620005	31062000564
NORDESTE	310620005	BELO HORIZONT	3106200	310620005	31062000564
NORDESTE	310620005	BELO HORIZONT	3106200	310620005	31062000564
NORDESTE	310620005	BELO HORIZONT	3106200	310620005	31062000564
NORDESTE	310620005	BELO HORIZONT	3106200	310620005	31062000564
NORDESTE	310620005	BELO HORIZONT	3106200	310620005	31062000564
NORDESTE	310620005	BELO HORIZONT	3106200	310620005	31062000564
NORDESTE	310620005	BELO HORIZONT	3106200	310620005	31062000564
NORDESTE	310620005	BELO HORIZONT	3106200	310620005	31062000564
NORDESTE	310620005	BELO HORIZONT	3106200	310620005	31062000564
NORDESTE	310620005	BELO HORIZONT	3106200	310620005	31062000564
NORDESTE	310620005	BELO HORIZONT	3106200	310620005	31062000564
NORDESTE	310620005	BELO HORIZONT	3106200	310620005	31062000564
NORDESTE	310620005	BELO HORIZONT	3106200	310620005	31062000564
NORDESTE	310620005	BELO HORIZONT	3106200	310620005	31062000564
NORDESTE	310620005	BELO HORIZONT	3106200	310620005	31062000564
NORDESTE	310620005	BELO HORIZONT	3106200	310620005	31062000564
NORDESTE	310620005	BELO HORIZONT	3106200	310620005	31062000564
NORDESTE	310620005	BELO HORIZONT	3106200	310620005	31062000564
NORDESTE	310620005	BELO HORIZONT	3106200	310620005	31062000564
NORDESTE	310620005	BELO HORIZONT	3106200	310620005	31062000564
NORDESTE	310620005	BELO HORIZONT	3106200	310620005	31062000564
NORDESTE	310620005	BELO HORIZONT	3106200	310620005	31062000564
NORDESTE	310620005	BELO HORIZONT	3106200	310620005	31062000564
NORDESTE	310620005	BELO HORIZONT	3106200	310620005	31062000564
NORDESTE	310620005	BELO HORIZONT	3106200	310620005	31062000564
NORDESTE	310620005	BELO HORIZONT	3106200	310620005	31062000564
NORDESTE	310620005	BELO HORIZONT	3106200	310620005	31062000564
NORDESTE	310620005	BELO HORIZONT	3106200	310620005	31062000564
NORDESTE	310620005	BELO HORIZONT	3106200	310620005	31062000564
NORDESTE	310620005	BELO HORIZONT	3106200	310620005	31062000564
NORDESTE	310620005	BELO HORIZONT	3106200	310620005	31062000564
NORDESTE	310620005	BELO HORIZONT	3106200	310620005	31062000564
NORDESTE	310620005	BELO HORIZONT	3106200	310620005	31062000564
NORDESTE	310620005	BELO HORIZONT	3106200	0	0
NORDESTE	310620005	BELO HORIZONT	3106200	310620005	31062000564
NORDESTE	310620005	BELO HORIZONT	3106200	0	0
NORDESTE	310620005	BELO HORIZONT	3106200	310620005	31062000564
NORDESTE	310620005	BELO HORIZONT	3106200	310620005	31062000564
NORDESTE	310620005	BELO HORIZONT	3106200	310620005	31062000564
NORDESTE	310620005	BELO HORIZONT	3106200	310620005	31062000564
NORDESTE	310620005	BELO HORIZONT	3106200	310620005	31062000564
NORDESTE	310620005	BELO HORIZONT	3106200	310620005	31062000564
NORDESTE	310620005	BELO HORIZONT	3106200	310620005	31062000564

NORDESTE	310620005	BELO HORIZONT	3106200	310620005	31062000564
NORDESTE	310620005	BELO HORIZONT	3106200	310620005	31062000564
NORDESTE	310620005	BELO HORIZONT	3106200	310620005	31062000564
NORDESTE	310620005	BELO HORIZONT	3106200	310620005	31062000564
NORDESTE	310620005	BELO HORIZONT	3106200	310620005	31062000564
NORDESTE	310620005	BELO HORIZONT	3106200	310620005	31062000564
NORDESTE	310620005	BELO HORIZONT	3106200	310620005	31062000564
NORDESTE	310620005	BELO HORIZONT	3106200	310620005	31062000564
NORDESTE	310620005	BELO HORIZONT	3106200	310620005	31062000564
NORDESTE	310620005	BELO HORIZONT	3106200	310620005	31062000564
NORDESTE	310620005	BELO HORIZONT	3106200	310620005	31062000564
NORDESTE	310620005	BELO HORIZONT	3106200	310620005	31062000564
NORDESTE	310620005	BELO HORIZONT	3106200	310620005	31062000564
NORDESTE	310620005	BELO HORIZONT	3106200	310620005	31062000564
NORDESTE	310620005	BELO HORIZONT	3106200	310620005	31062000564
NORDESTE	310620005	BELO HORIZONT	3106200	310620005	31062000564
NORDESTE	310620005	BELO HORIZONT	3106200	310620005	31062000564
NORDESTE	310620005	BELO HORIZONT	3106200	310620005	31062000564
NORDESTE	310620005	BELO HORIZONT	3106200	310620005	31062000564
NORDESTE	310620005	BELO HORIZONT	3106200	310620005	31062000564
NORDESTE	310620005	BELO HORIZONT	3106200	310620005	31062000564
NORDESTE	310620005	BELO HORIZONT	3106200	310620005	31062000564
NORDESTE	310620005	BELO HORIZONT	3106200	310620005	31062000564
NORDESTE	310620005	BELO HORIZONT	3106200	310620005	31062000564
NORDESTE	310620005	BELO HORIZONT	3106200	310620005	31062000564
NORDESTE	310620005	BELO HORIZONT	3106200	310620005	31062000564
NORDESTE	310620005	BELO HORIZONT	3106200	310620005	31062000564
NORDESTE	310620005	BELO HORIZONT	3106200	310620005	31062000564
NORDESTE	310620005	BELO HORIZONT	3106200	310620005	31062000564
NORDESTE	310620005	BELO HORIZONT	3106200	310620005	31062000564
NORDESTE	310620005	BELO HORIZONT	3106200	310620005	31062000564
NORDESTE	310620005	BELO HORIZONT	3106200	310620005	31062000564
NORDESTE	310620005	BELO HORIZONT	3106200	310620005	31062000564
NORDESTE	310620005	BELO HORIZONT	3106200	310620005	31062000564
NORDESTE	310620005	BELO HORIZONT	3106200	310620005	31062000564
NORDESTE	310620005	BELO HORIZONT	3106200	310620005	31062000564
NORDESTE	310620005	BELO HORIZONT	3106200	310620005	31062000564
NORDESTE	310620005	BELO HORIZONT	3106200	310620005	31062000564
NORDESTE	310620005	BELO HORIZONT	3106200	310620005	31062000564
NORDESTE	310620005	BELO HORIZONT	3106200	310620005	31062000564
NORDESTE	310620005	BELO HORIZONT	3106200	310620005	31062000564
NORDESTE	310620005	BELO HORIZONT	3106200	310620005	31062000564
NORDESTE	310620005	BELO HORIZONT	3106200	310620005	31062000564
NORDESTE	310620005	BELO HORIZONT	3106200	310620005	31062000564
NORDESTE	310620005	BELO HORIZONT	3106200	310620005	31062000564
NORDESTE	310620005	BELO HORIZONT	3106200	310620005	31062000564
NORDESTE	310620005	BELO HORIZONT	3106200	310620005	31062000564
NORDESTE	310620005	BELO HORIZONT	3106200	310620005	31062000564
NORDESTE	310620005	BELO HORIZONT	3106200	310620005	31062000564
NORDESTE	310620005	BELO HORIZONT	3106200	310620005	31062000564

NORDESTE	310620005	BELO HORIZONT	3106200	310620005	31062000564
NORDESTE	310620005	BELO HORIZONT	3106200	310620005	31062000564
NORDESTE	310620005	BELO HORIZONT	3106200	310620005	31062000564
NORDESTE	310620005	BELO HORIZONT	3106200	310620005	31062000564
NORDESTE	310620005	BELO HORIZONT	3106200	310620005	31062000564
NORDESTE	310620005	BELO HORIZONT	3106200	310620005	31062000564
NORDESTE	310620005	BELO HORIZONT	3106200	310620005	31062000564
NORDESTE	310620005	BELO HORIZONT	3106200	310620005	31062000564
NORDESTE	310620005	BELO HORIZONT	3106200	310620005	31062000564
NORDESTE	310620005	BELO HORIZONT	3106200	310620005	31062000564
NORDESTE	310620005	BELO HORIZONT	3106200	310620005	31062000564
NORDESTE	310620005	BELO HORIZONT	3106200	0	0
NORDESTE	310620005	BELO HORIZONT	3106200	310620005	31062000564
NORDESTE	310620005	BELO HORIZONT	3106200	310620005	31062000564
NORDESTE	310620005	BELO HORIZONT	3106200	310620005	31062000564
NORDESTE	310620005	BELO HORIZONT	3106200	310620005	31062000564
NORDESTE	310620005	BELO HORIZONT	3106200	310620005	31062000564
NORDESTE	310620005	BELO HORIZONT	3106200	310620005	31062000564
NORDESTE	310620005	BELO HORIZONT	3106200	310620005	31062000564
NORDESTE	310620005	BELO HORIZONT	3106200	310620005	31062000564
NORDESTE	310620005	BELO HORIZONT	3106200	310620005	31062000564
NORDESTE	310620005	BELO HORIZONT	3106200	310620005	31062000564
NORDESTE	310620005	BELO HORIZONT	3106200	310620005	31062000564
NORDESTE	310620005	BELO HORIZONT	3106200	0	0
NORDESTE	310620005	BELO HORIZONT	3106200	310620005	31062000564
NORDESTE	310620005	BELO HORIZONT	3106200	310620005	31062000564
NORDESTE	310620005	BELO HORIZONT	3106200	310620005	31062000564
NORDESTE	310620005	BELO HORIZONT	3106200	310620005	31062000564
NORDESTE	310620005	BELO HORIZONT	3106200	310620005	31062000564
NORDESTE	310620005	BELO HORIZONT	3106200	310620005	31062000564
NOROESTE	310620005	BELO HORIZONT	3106200	310620005	31062000565
NOROESTE	310620005	BELO HORIZONT	3106200	310620005	31062000565
NOROESTE	310620005	BELO HORIZONT	3106200	310620005	31062000565
NOROESTE	310620005	BELO HORIZONT	3106200	310620005	31062000565
NOROESTE	310620005	BELO HORIZONT	3106200	310620005	31062000565
NOROESTE	310620005	BELO HORIZONT	3106200	310620005	31062000565
NOROESTE	310620005	BELO HORIZONT	3106200	310620005	31062000565
NOROESTE	310620005	BELO HORIZONT	3106200	310620005	31062000565
NOROESTE	310620005	BELO HORIZONT	3106200	310620005	31062000565
NOROESTE	310620005	BELO HORIZONT	3106200	310620005	31062000565
NOROESTE	310620005	BELO HORIZONT	3106200	310620005	31062000565
NOROESTE	310620005	BELO HORIZONT	3106200	310620005	31062000565
NOROESTE	310620005	BELO HORIZONT	3106200	310620005	31062000565
NOROESTE	310620005	BELO HORIZONT	3106200	310620005	31062000565
NOROESTE	310620005	BELO HORIZONT	3106200	310620005	31062000565
NOROESTE	310620005	BELO HORIZONT	3106200	310620005	31062000565
NOROESTE	310620005	BELO HORIZONT	3106200	310620005	31062000565
NOROESTE	310620005	BELO HORIZONT	3106200	310620005	31062000565
NOROESTE	310620005	BELO HORIZONT	3106200	310620005	31062000565

NOROESTE	310620005	BELO HORIZONT	3106200	310620005	31062000565
NOROESTE	310620005	BELO HORIZONT	3106200	310620005	31062000565
NOROESTE	310620005	BELO HORIZONT	3106200	310620005	31062000565
NOROESTE	310620005	BELO HORIZONT	3106200	310620005	31062000565
NOROESTE	310620005	BELO HORIZONT	3106200	310620005	31062000565
NOROESTE	310620005	BELO HORIZONT	3106200	310620005	31062000565
NOROESTE	310620005	BELO HORIZONT	3106200	310620005	31062000565
NOROESTE	310620005	BELO HORIZONT	3106200	310620005	31062000565
NOROESTE	310620005	BELO HORIZONT	3106200	310620005	31062000565
NOROESTE	310620005	BELO HORIZONT	3106200	310620005	31062000565
NOROESTE	310620005	BELO HORIZONT	3106200	310620005	31062000565
NOROESTE	310620005	BELO HORIZONT	3106200	310620005	31062000565
NOROESTE	310620005	BELO HORIZONT	3106200	310620005	31062000565
NOROESTE	310620005	BELO HORIZONT	3106200	310620005	31062000565
NOROESTE	310620005	BELO HORIZONT	3106200	310620005	31062000565
NOROESTE	310620005	BELO HORIZONT	3106200	310620005	31062000565
NOROESTE	310620005	BELO HORIZONT	3106200	310620005	31062000565
NOROESTE	310620005	BELO HORIZONT	3106200	310620005	31062000565
NOROESTE	310620005	BELO HORIZONT	3106200	310620005	31062000565
NOROESTE	310620005	BELO HORIZONT	3106200	310620005	31062000565
NOROESTE	310620005	BELO HORIZONT	3106200	310620005	31062000565
NOROESTE	310620005	BELO HORIZONT	3106200	310620005	31062000565
NOROESTE	310620005	BELO HORIZONT	3106200	310620005	31062000565
NOROESTE	310620005	BELO HORIZONT	3106200	310620005	31062000565
NOROESTE	310620005	BELO HORIZONT	3106200	310620005	31062000565
NOROESTE	310620005	BELO HORIZONT	3106200	310620005	31062000565
NOROESTE	310620005	BELO HORIZONT	3106200	310620005	31062000565
NOROESTE	310620005	BELO HORIZONT	3106200	310620005	31062000565
NOROESTE	310620005	BELO HORIZONT	3106200	310620005	31062000565
NOROESTE	310620005	BELO HORIZONT	3106200	310620005	31062000565
NOROESTE	310620005	BELO HORIZONT	3106200	310620005	31062000565
NOROESTE	310620005	BELO HORIZONT	3106200	310620005	31062000565
NOROESTE	310620005	BELO HORIZONT	3106200	310620005	31062000565
NOROESTE	310620005	BELO HORIZONT	3106200	310620005	31062000565
NOROESTE	310620005	BELO HORIZONT	3106200	310620005	31062000565
NOROESTE	310620005	BELO HORIZONT	3106200	310620005	31062000565
NOROESTE	310620005	BELO HORIZONT	3106200	310620005	31062000565
NOROESTE	310620005	BELO HORIZONT	3106200	310620005	31062000565
NOROESTE	310620005	BELO HORIZONT	3106200	310620005	31062000565
NOROESTE	310620005	BELO HORIZONT	3106200	310620005	31062000565
NOROESTE	310620005	BELO HORIZONT	3106200	310620005	31062000565
NOROESTE	310620005	BELO HORIZONT	3106200	310620005	31062000565
NOROESTE	310620005	BELO HORIZONT	3106200	310620005	31062000565
NOROESTE	310620005	BELO HORIZONT	3106200	310620005	31062000565
NOROESTE	310620005	BELO HORIZONT	3106200	310620005	31062000565
NOROESTE	310620005	BELO HORIZONT	3106200	310620005	31062000565
NOROESTE	310620005	BELO HORIZONT	3106200	310620005	31062000565
NOROESTE	310620005	BELO HORIZONT	3106200	310620005	31062000565
NOROESTE	310620005	BELO HORIZONT	3106200	310620005	31062000565
NOROESTE	310620005	BELO HORIZONT	3106200	310620005	31062000565

NOROESTE	310620005	BELO HORIZONT	3106200	310620005	31062000565
NOROESTE	310620005	BELO HORIZONT	3106200	310620005	31062000565
NOROESTE	310620005	BELO HORIZONT	3106200	310620005	31062000565
NOROESTE	310620005	BELO HORIZONT	3106200	310620005	31062000565
NOROESTE	310620005	BELO HORIZONT	3106200	310620005	31062000565
NOROESTE	310620005	BELO HORIZONT	3106200	310620005	31062000565
NOROESTE	310620005	BELO HORIZONT	3106200	310620005	31062000565
NOROESTE	310620005	BELO HORIZONT	3106200	310620005	31062000565
NOROESTE	310620005	BELO HORIZONT	3106200	310620005	31062000565
NOROESTE	310620005	BELO HORIZONT	3106200	310620005	31062000565
NOROESTE	310620005	BELO HORIZONT	3106200	310620005	31062000565
NOROESTE	310620005	BELO HORIZONT	3106200	310620005	31062000565
NOROESTE	310620005	BELO HORIZONT	3106200	310620005	31062000565
NOROESTE	310620005	BELO HORIZONT	3106200	310620005	31062000565
NOROESTE	310620005	BELO HORIZONT	3106200	310620005	31062000565
NOROESTE	310620005	BELO HORIZONT	3106200	310620005	31062000565
NOROESTE	310620005	BELO HORIZONT	3106200	310620005	31062000565
NOROESTE	310620005	BELO HORIZONT	3106200	310620005	31062000565
NOROESTE	310620005	BELO HORIZONT	3106200	310620005	31062000565
NOROESTE	310620005	BELO HORIZONT	3106200	310620005	31062000565
NOROESTE	310620005	BELO HORIZONT	3106200	310620005	31062000565
NOROESTE	310620005	BELO HORIZONT	3106200	310620005	31062000565
NOROESTE	310620005	BELO HORIZONT	3106200	310620005	31062000565
NOROESTE	310620005	BELO HORIZONT	3106200	310620005	31062000565
NOROESTE	310620005	BELO HORIZONT	3106200	310620005	31062000565
NOROESTE	310620005	BELO HORIZONT	3106200	310620005	31062000565
NOROESTE	310620005	BELO HORIZONT	3106200	310620005	31062000565
NOROESTE	310620005	BELO HORIZONT	3106200	310620005	31062000565
NOROESTE	310620005	BELO HORIZONT	3106200	310620005	31062000565
NOROESTE	310620005	BELO HORIZONT	3106200	310620005	31062000565
NOROESTE	310620005	BELO HORIZONT	3106200	310620005	31062000565
NOROESTE	310620005	BELO HORIZONT	3106200	310620005	31062000565
NOROESTE	310620005	BELO HORIZONT	3106200	310620005	31062000565
NOROESTE	310620005	BELO HORIZONT	3106200	310620005	31062000565
NOROESTE	310620005	BELO HORIZONT	3106200	310620005	31062000565
NOROESTE	310620005	BELO HORIZONT	3106200	310620005	31062000565
NOROESTE	310620005	BELO HORIZONT	3106200	310620005	31062000565
NOROESTE	310620005	BELO HORIZONT	3106200	310620005	31062000565
NOROESTE	310620005	BELO HORIZONT	3106200	310620005	31062000565
NOROESTE	310620005	BELO HORIZONT	3106200	310620005	31062000565
NOROESTE	310620005	BELO HORIZONT	3106200	310620005	31062000565
NOROESTE	310620005	BELO HORIZONT	3106200	310620005	31062000565
NOROESTE	310620005	BELO HORIZONT	3106200	310620005	31062000565
NOROESTE	310620005	BELO HORIZONT	3106200	310620005	31062000565
NOROESTE	310620005	BELO HORIZONT	3106200	310620005	31062000565
NOROESTE	310620005	BELO HORIZONT	3106200	310620005	31062000565
NOROESTE	310620005	BELO HORIZONT	3106200	310620005	31062000565
NOROESTE	310620005	BELO HORIZONT	3106200	310620005	31062000565
NOROESTE	310620005	BELO HORIZONT	3106200	310620005	31062000565
NOROESTE	310620005	BELO HORIZONT	3106200	310620005	31062000565

NOROESTE	310620005	BELO HORIZONT	3106200	310620005	31062000565
NOROESTE	310620005	BELO HORIZONT	3106200	310620005	31062000565
NOROESTE	310620005	BELO HORIZONT	3106200	310620005	31062000565
NOROESTE	310620005	BELO HORIZONT	3106200	310620005	31062000565
NOROESTE	310620005	BELO HORIZONT	3106200	310620005	31062000565
NOROESTE	310620005	BELO HORIZONT	3106200	310620005	31062000565
NOROESTE	310620005	BELO HORIZONT	3106200	310620005	31062000565
NOROESTE	310620005	BELO HORIZONT	3106200	310620005	31062000565
NOROESTE	310620005	BELO HORIZONT	3106200	310620005	31062000565
NOROESTE	310620005	BELO HORIZONT	3106200	310620005	31062000565
NOROESTE	310620005	BELO HORIZONT	3106200	310620005	31062000565
NOROESTE	310620005	BELO HORIZONT	3106200	310620005	31062000565
OESTE	310620005	BELO HORIZONT	3106200	310620005	31062000567
OESTE	310620005	BELO HORIZONT	3106200	310620005	31062000567
OESTE	310620005	BELO HORIZONT	3106200	310620005	31062000567
OESTE	310620005	BELO HORIZONT	3106200	310620005	31062000567
OESTE	310620005	BELO HORIZONT	3106200	310620005	31062000567
OESTE	310620005	BELO HORIZONT	3106200	310620005	31062000567
OESTE	310620005	BELO HORIZONT	3106200	310620005	31062000567
OESTE	310620005	BELO HORIZONT	3106200	310620005	31062000567
OESTE	310620005	BELO HORIZONT	3106200	310620005	31062000567
OESTE	310620005	BELO HORIZONT	3106200	310620005	31062000567
OESTE	310620005	BELO HORIZONT	3106200	310620005	31062000567
OESTE	310620005	BELO HORIZONT	3106200	310620005	31062000567
OESTE	310620005	BELO HORIZONT	3106200	310620005	31062000567
OESTE	310620005	BELO HORIZONT	3106200	310620005	31062000567
OESTE	310620005	BELO HORIZONT	3106200	310620005	31062000567
OESTE	310620005	BELO HORIZONT	3106200	310620005	31062000567
BARREIRO	310620025	BARREIRO	3106200	310620025	31062002561
BARREIRO	310620025	BARREIRO	3106200	310620025	31062002561
BARREIRO	310620025	BARREIRO	3106200	310620025	31062002561
BARREIRO	310620025	BARREIRO	3106200	310620025	31062002561
BARREIRO	310620025	BARREIRO	3106200	310620025	31062002561
BARREIRO	310620025	BARREIRO	3106200	310620025	31062002561
BARREIRO	310620025	BARREIRO	3106200	310620025	31062002561
BARREIRO	310620025	BARREIRO	3106200	310620025	31062002561
BARREIRO	310620025	BARREIRO	3106200	310620025	31062002561
NORDESTE	310620060	VENDA NOVA	3106200	310620060	31062006064
NORDESTE	310620060	VENDA NOVA	3106200	310620060	31062006064
NORDESTE	310620060	VENDA NOVA	3106200	310620060	31062006064
NORDESTE	310620060	VENDA NOVA	3106200	310620060	31062006064
NORDESTE	310620060	VENDA NOVA	3106200	310620060	31062006064
NORDESTE	310620060	VENDA NOVA	3106200	310620060	31062006064
NORDESTE	310620060	VENDA NOVA	3106200	310620060	31062006064
NORDESTE	310620060	VENDA NOVA	3106200	310620060	31062006064
NORDESTE	310620060	VENDA NOVA	3106200	310620060	31062006064
NOROESTE	310620005	BELO HORIZONT	3106200	310620005	31062000565
NOROESTE	310620005	BELO HORIZONT	3106200	310620005	31062000565
NOROESTE	310620005	BELO HORIZONT	3106200	310620005	31062000565
NOROESTE	310620005	BELO HORIZONT	3106200	310620005	31062000565

NOROESTE	310620005	BELO HORIZONT	3106200	310620005	31062000565
NOROESTE	310620005	BELO HORIZONT	3106200	0	0
NOROESTE	310620005	BELO HORIZONT	3106200	310620005	31062000565
NOROESTE	310620005	BELO HORIZONT	3106200	310620005	31062000565
NOROESTE	310620005	BELO HORIZONT	3106200	310620005	31062000565
NOROESTE	310620005	BELO HORIZONT	3106200	310620005	31062000565
NOROESTE	310620005	BELO HORIZONT	3106200	310620005	31062000565
NOROESTE	310620005	BELO HORIZONT	3106200	310620005	31062000565
NOROESTE	310620005	BELO HORIZONT	3106200	310620005	31062000565
NOROESTE	310620005	BELO HORIZONT	3106200	310620005	31062000565
NOROESTE	310620005	BELO HORIZONT	3106200	310620005	31062000565
NOROESTE	310620005	BELO HORIZONT	3106200	310620005	31062000565
NOROESTE	310620005	BELO HORIZONT	3106200	310620005	31062000565
NOROESTE	310620005	BELO HORIZONT	3106200	310620005	31062000565
NOROESTE	310620005	BELO HORIZONT	3106200	310620005	31062000565
NOROESTE	310620005	BELO HORIZONT	3106200	310620005	31062000565
NOROESTE	310620005	BELO HORIZONT	3106200	310620005	31062000565
NOROESTE	310620005	BELO HORIZONT	3106200	310620005	31062000565
NOROESTE	310620005	BELO HORIZONT	3106200	310620005	31062000565
NOROESTE	310620005	BELO HORIZONT	3106200	310620005	31062000565
NOROESTE	310620005	BELO HORIZONT	3106200	310620005	31062000565
NOROESTE	310620005	BELO HORIZONT	3106200	310620005	31062000565
NOROESTE	310620005	BELO HORIZONT	3106200	310620005	31062000565
NOROESTE	310620005	BELO HORIZONT	3106200	310620005	31062000565
NOROESTE	310620005	BELO HORIZONT	3106200	310620005	31062000565
NOROESTE	310620005	BELO HORIZONT	3106200	310620005	31062000565
NOROESTE	310620005	BELO HORIZONT	3106200	310620005	31062000565
NOROESTE	310620005	BELO HORIZONT	3106200	310620005	31062000565
NOROESTE	310620005	BELO HORIZONT	3106200	310620005	31062000565
NOROESTE	310620005	BELO HORIZONT	3106200	310620005	31062000565
NOROESTE	310620005	BELO HORIZONT	3106200	310620005	31062000565
NOROESTE	310620005	BELO HORIZONT	3106200	310620005	31062000565
NOROESTE	310620005	BELO HORIZONT	3106200	310620005	31062000565
NOROESTE	310620005	BELO HORIZONT	3106200	310620005	31062000565
NOROESTE	310620005	BELO HORIZONT	3106200	310620005	31062000565
NOROESTE	310620005	BELO HORIZONT	3106200	310620005	31062000565
NOROESTE	310620005	BELO HORIZONT	3106200	310620005	31062000565
NOROESTE	310620005	BELO HORIZONT	3106200	310620005	31062000565
NOROESTE	310620005	BELO HORIZONT	3106200	310620005	31062000565
NOROESTE	310620005	BELO HORIZONT	3106200	310620005	31062000565
NOROESTE	310620005	BELO HORIZONT	3106200	310620005	31062000565
NOROESTE	310620005	BELO HORIZONT	3106200	310620005	31062000565
NOROESTE	310620005	BELO HORIZONT	3106200	310620005	31062000565
NOROESTE	310620005	BELO HORIZONT	3106200	310620005	31062000565
NOROESTE	310620005	BELO HORIZONT	3106200	310620005	31062000565
NOROESTE	310620005	BELO HORIZONT	3106200	310620005	31062000565
NOROESTE	310620005	BELO HORIZONT	3106200	310620005	31062000565
NOROESTE	310620005	BELO HORIZONT	3106200	310620005	31062000565
NOROESTE	310620005	BELO HORIZONT	3106200	310620005	31062000565
NOROESTE	310620005	BELO HORIZONT	3106200	310620005	31062000565

NOROESTE	310620005	BELO HORIZONT	3106200	310620005	31062000565
NOROESTE	310620005	BELO HORIZONT	3106200	310620005	31062000565
NOROESTE	310620005	BELO HORIZONT	3106200	310620005	31062000565
NOROESTE	310620005	BELO HORIZONT	3106200	310620005	31062000565
NOROESTE	310620005	BELO HORIZONT	3106200	310620005	31062000565
NOROESTE	310620005	BELO HORIZONT	3106200	310620005	31062000565
NOROESTE	310620005	BELO HORIZONT	3106200	310620005	31062000565
NOROESTE	310620005	BELO HORIZONT	3106200	310620005	31062000565
NOROESTE	310620005	BELO HORIZONT	3106200	310620005	31062000565
NOROESTE	310620005	BELO HORIZONT	3106200	310620005	31062000565
NOROESTE	310620005	BELO HORIZONT	3106200	310620005	31062000565
NOROESTE	310620005	BELO HORIZONT	3106200	310620005	31062000565
NOROESTE	310620005	BELO HORIZONT	3106200	310620005	31062000565
NOROESTE	310620005	BELO HORIZONT	3106200	310620005	31062000565
NOROESTE	310620005	BELO HORIZONT	3106200	310620005	31062000565
NOROESTE	310620005	BELO HORIZONT	3106200	310620005	31062000565
NOROESTE	310620005	BELO HORIZONT	3106200	310620005	31062000565
NOROESTE	310620005	BELO HORIZONT	3106200	310620005	31062000565
NOROESTE	310620005	BELO HORIZONT	3106200	310620005	31062000565
NOROESTE	310620005	BELO HORIZONT	3106200	310620005	31062000565
NOROESTE	310620005	BELO HORIZONT	3106200	310620005	31062000565
NOROESTE	310620005	BELO HORIZONT	3106200	310620005	31062000565
NOROESTE	310620005	BELO HORIZONT	3106200	310620005	31062000565
NOROESTE	310620005	BELO HORIZONT	3106200	310620005	31062000565
NOROESTE	310620005	BELO HORIZONT	3106200	310620005	31062000565
NOROESTE	310620005	BELO HORIZONT	3106200	310620005	31062000565
NOROESTE	310620005	BELO HORIZONT	3106200	310620005	31062000565
NOROESTE	310620005	BELO HORIZONT	3106200	310620005	31062000565
NOROESTE	310620005	BELO HORIZONT	3106200	310620005	31062000565
NOROESTE	310620005	BELO HORIZONT	3106200	310620005	31062000565
NOROESTE	310620005	BELO HORIZONT	3106200	310620005	31062000565
NOROESTE	310620005	BELO HORIZONT	3106200	310620005	31062000565
NOROESTE	310620005	BELO HORIZONT	3106200	310620005	31062000565
NOROESTE	310620005	BELO HORIZONT	3106200	310620005	31062000565
NOROESTE	310620005	BELO HORIZONT	3106200	310620005	31062000565
NOROESTE	310620005	BELO HORIZONT	3106200	310620005	31062000565
NOROESTE	310620005	BELO HORIZONT	3106200	310620005	31062000565
NOROESTE	310620005	BELO HORIZONT	3106200	310620005	31062000565
NOROESTE	310620005	BELO HORIZONT	3106200	310620005	31062000565
NOROESTE	310620005	BELO HORIZONT	3106200	310620005	31062000565
NOROESTE	310620005	BELO HORIZONT	3106200	310620005	31062000565
NOROESTE	310620005	BELO HORIZONT	3106200	310620005	31062000565
NOROESTE	310620005	BELO HORIZONT	3106200	310620005	31062000565
NOROESTE	310620005	BELO HORIZONT	3106200	310620005	31062000565
NOROESTE	310620005	BELO HORIZONT	3106200	310620005	31062000565
NOROESTE	310620005	BELO HORIZONT	3106200	310620005	31062000565
NOROESTE	310620005	BELO HORIZONT	3106200	310620005	31062000565
NOROESTE	310620005	BELO HORIZONT	3106200	310620005	31062000565
NOROESTE	310620005	BELO HORIZONT	3106200	310620005	31062000565
NOROESTE	310620005	BELO HORIZONT	3106200	310620005	31062000565

NOROESTE	310620005	BELO HORIZONT	3106200	310620005	31062000565
NOROESTE	310620005	BELO HORIZONT	3106200	310620005	31062000565
NOROESTE	310620005	BELO HORIZONT	3106200	310620005	31062000565
NOROESTE	310620005	BELO HORIZONT	3106200	310620005	31062000565
NOROESTE	310620005	BELO HORIZONT	3106200	310620005	31062000565
NOROESTE	310620005	BELO HORIZONT	3106200	310620005	31062000565
NOROESTE	310620005	BELO HORIZONT	3106200	310620005	31062000565
NOROESTE	310620005	BELO HORIZONT	3106200	310620005	31062000565
NOROESTE	310620005	BELO HORIZONT	3106200	310620005	31062000565
NOROESTE	310620005	BELO HORIZONT	3106200	310620005	31062000565
NOROESTE	310620005	BELO HORIZONT	3106200	310620005	31062000565
NOROESTE	310620005	BELO HORIZONT	3106200	310620005	31062000565
NOROESTE	310620005	BELO HORIZONT	3106200	310620005	31062000565
NOROESTE	310620005	BELO HORIZONT	3106200	310620005	31062000565
NOROESTE	310620005	BELO HORIZONT	3106200	310620005	31062000565
NOROESTE	310620005	BELO HORIZONT	3106200	310620005	31062000565
NOROESTE	310620005	BELO HORIZONT	3106200	310620005	31062000565
NOROESTE	310620005	BELO HORIZONT	3106200	310620005	31062000565
NOROESTE	310620005	BELO HORIZONT	3106200	310620005	31062000565
NOROESTE	310620005	BELO HORIZONT	3106200	310620005	31062000565
NOROESTE	310620005	BELO HORIZONT	3106200	310620005	31062000565
NOROESTE	310620005	BELO HORIZONT	3106200	310620005	31062000565
NOROESTE	310620005	BELO HORIZONT	3106200	310620005	31062000565
NOROESTE	310620005	BELO HORIZONT	3106200	310620005	31062000565
NOROESTE	310620005	BELO HORIZONT	3106200	310620005	31062000565
NOROESTE	310620005	BELO HORIZONT	3106200	310620005	31062000565
NOROESTE	310620005	BELO HORIZONT	3106200	310620005	31062000565
NOROESTE	310620005	BELO HORIZONT	3106200	310620005	31062000565
NOROESTE	310620005	BELO HORIZONT	3106200	310620005	31062000565
NOROESTE	310620005	BELO HORIZONT	3106200	310620005	31062000565
NOROESTE	310620005	BELO HORIZONT	3106200	310620005	31062000565
NOROESTE	310620005	BELO HORIZONT	3106200	0	0
NOROESTE	310620005	BELO HORIZONT	3106200	310620005	31062000565
NOROESTE	310620005	BELO HORIZONT	3106200	310620005	31062000565
NOROESTE	310620005	BELO HORIZONT	3106200	310620005	31062000565
NOROESTE	310620005	BELO HORIZONT	3106200	310620005	31062000565
NOROESTE	310620005	BELO HORIZONT	3106200	310620005	31062000565
NOROESTE	310620005	BELO HORIZONT	3106200	310620005	31062000565
NOROESTE	310620005	BELO HORIZONT	3106200	310620005	31062000565
NOROESTE	310620005	BELO HORIZONT	3106200	310620005	31062000565
NOROESTE	310620005	BELO HORIZONT	3106200	310620005	31062000565
NOROESTE	310620005	BELO HORIZONT	3106200	310620005	31062000565
NOROESTE	310620005	BELO HORIZONT	3106200	310620005	31062000565
NOROESTE	310620005	BELO HORIZONT	3106200	310620005	31062000565
NOROESTE	310620005	BELO HORIZONT	3106200	310620005	31062000565
NOROESTE	310620005	BELO HORIZONT	3106200	310620005	31062000565
NOROESTE	310620005	BELO HORIZONT	3106200	310620005	31062000565
NOROESTE	310620005	BELO HORIZONT	3106200	310620005	31062000565
NOROESTE	310620005	BELO HORIZONT	3106200	310620005	31062000565
NOROESTE	310620005	BELO HORIZONT	3106200	310620005	31062000565

NOROESTE	310620005	BELO HORIZONT	3106200	310620005	31062000565
NOROESTE	310620005	BELO HORIZONT	3106200	310620005	31062000565
NOROESTE	310620005	BELO HORIZONT	3106200	310620005	31062000565
NOROESTE	310620005	BELO HORIZONT	3106200	310620005	31062000565
NOROESTE	310620005	BELO HORIZONT	3106200	310620005	31062000565
NOROESTE	310620005	BELO HORIZONT	3106200	310620005	31062000565
NOROESTE	310620005	BELO HORIZONT	3106200	310620005	31062000565
NOROESTE	310620005	BELO HORIZONT	3106200	310620005	31062000565
NOROESTE	310620005	BELO HORIZONT	3106200	310620005	31062000565
NOROESTE	310620005	BELO HORIZONT	3106200	310620005	31062000565
NOROESTE	310620005	BELO HORIZONT	3106200	310620005	31062000565
NOROESTE	310620005	BELO HORIZONT	3106200	310620005	31062000565
NOROESTE	310620005	BELO HORIZONT	3106200	310620005	31062000565
NOROESTE	310620005	BELO HORIZONT	3106200	310620005	31062000565
NOROESTE	310620005	BELO HORIZONT	3106200	310620005	31062000565
NOROESTE	310620005	BELO HORIZONT	3106200	310620005	31062000565
NOROESTE	310620005	BELO HORIZONT	3106200	310620005	31062000565
NOROESTE	310620005	BELO HORIZONT	3106200	310620005	31062000565
NOROESTE	310620005	BELO HORIZONT	3106200	310620005	31062000565
NOROESTE	310620005	BELO HORIZONT	3106200	310620005	31062000565
NOROESTE	310620005	BELO HORIZONT	3106200	310620005	31062000565
NOROESTE	310620005	BELO HORIZONT	3106200	310620005	31062000565
NOROESTE	310620005	BELO HORIZONT	3106200	310620005	31062000565
NOROESTE	310620005	BELO HORIZONT	3106200	310620005	31062000565
NOROESTE	310620005	BELO HORIZONT	3106200	310620005	31062000565
NOROESTE	310620005	BELO HORIZONT	3106200	310620005	31062000565
NOROESTE	310620005	BELO HORIZONT	3106200	310620005	31062000565
NOROESTE	310620005	BELO HORIZONT	3106200	310620005	31062000565
NOROESTE	310620005	BELO HORIZONT	3106200	310620005	31062000565
NOROESTE	310620005	BELO HORIZONT	3106200	310620005	31062000565
NOROESTE	310620005	BELO HORIZONT	3106200	310620005	31062000565
NOROESTE	310620005	BELO HORIZONT	3106200	310620005	31062000565
NOROESTE	310620005	BELO HORIZONT	3106200	310620005	31062000565
NOROESTE	310620005	BELO HORIZONT	3106200	310620005	31062000565
NOROESTE	310620005	BELO HORIZONT	3106200	310620005	31062000565
NOROESTE	310620005	BELO HORIZONT	3106200	310620005	31062000565
NOROESTE	310620005	BELO HORIZONT	3106200	310620005	31062000565
NOROESTE	310620005	BELO HORIZONT	3106200	310620005	31062000565
NOROESTE	310620005	BELO HORIZONT	3106200	310620005	31062000565
NOROESTE	310620005	BELO HORIZONT	3106200	310620005	31062000565
NOROESTE	310620005	BELO HORIZONT	3106200	310620005	31062000565
NOROESTE	310620005	BELO HORIZONT	3106200	310620005	31062000565
NOROESTE	310620005	BELO HORIZONT	3106200	310620005	31062000565
NOROESTE	310620005	BELO HORIZONT	3106200	310620005	31062000565
NOROESTE	310620005	BELO HORIZONT	3106200	310620005	31062000565
NOROESTE	310620005	BELO HORIZONT	3106200	310620005	31062000565
NOROESTE	310620005	BELO HORIZONT	3106200	310620005	31062000565
NOROESTE	310620005	BELO HORIZONT	3106200	310620005	31062000565
NOROESTE	310620005	BELO HORIZONT	3106200	310620005	31062000565
NOROESTE	310620005	BELO HORIZONT	3106200	310620005	31062000565

NOROESTE	310620005	BELO HORIZONT	3106200	310620005	31062000565
NOROESTE	310620005	BELO HORIZONT	3106200	310620005	31062000565
NOROESTE	310620005	BELO HORIZONT	3106200	310620005	31062000565
NOROESTE	310620005	BELO HORIZONT	3106200	310620005	31062000565
NOROESTE	310620005	BELO HORIZONT	3106200	310620005	31062000565
NOROESTE	310620005	BELO HORIZONT	3106200	310620005	31062000565
NOROESTE	310620005	BELO HORIZONT	3106200	310620005	31062000565
NOROESTE	310620005	BELO HORIZONT	3106200	310620005	31062000565
NOROESTE	310620005	BELO HORIZONT	3106200	310620005	31062000565
NOROESTE	310620005	BELO HORIZONT	3106200	310620005	31062000565
NOROESTE	310620005	BELO HORIZONT	3106200	310620005	31062000565
NOROESTE	310620005	BELO HORIZONT	3106200	310620005	31062000565
NOROESTE	310620005	BELO HORIZONT	3106200	310620005	31062000565
NOROESTE	310620005	BELO HORIZONT	3106200	310620005	31062000565
NOROESTE	310620005	BELO HORIZONT	3106200	310620005	31062000565
NOROESTE	310620005	BELO HORIZONT	3106200	310620005	31062000565
NOROESTE	310620005	BELO HORIZONT	3106200	310620005	31062000565
NOROESTE	310620005	BELO HORIZONT	3106200	310620005	31062000565
NOROESTE	310620005	BELO HORIZONT	3106200	310620005	31062000565
NOROESTE	310620005	BELO HORIZONT	3106200	310620005	31062000565
NOROESTE	310620005	BELO HORIZONT	3106200	310620005	31062000565
NOROESTE	310620005	BELO HORIZONT	3106200	310620005	31062000565
NOROESTE	310620005	BELO HORIZONT	3106200	310620005	31062000565
NOROESTE	310620005	BELO HORIZONT	3106200	310620005	31062000565
NOROESTE	310620005	BELO HORIZONT	3106200	310620005	31062000565
NOROESTE	310620005	BELO HORIZONT	3106200	310620005	31062000565
NOROESTE	310620005	BELO HORIZONT	3106200	310620005	31062000565
NOROESTE	310620005	BELO HORIZONT	3106200	310620005	31062000565
NOROESTE	310620005	BELO HORIZONT	3106200	310620005	31062000565
NOROESTE	310620005	BELO HORIZONT	3106200	310620005	31062000565
NOROESTE	310620005	BELO HORIZONT	3106200	310620005	31062000565
NOROESTE	310620005	BELO HORIZONT	3106200	310620005	31062000565
NOROESTE	310620005	BELO HORIZONT	3106200	310620005	31062000565
NOROESTE	310620005	BELO HORIZONT	3106200	310620005	31062000565
NOROESTE	310620005	BELO HORIZONT	3106200	310620005	31062000565
NOROESTE	310620005	BELO HORIZONT	3106200	310620005	31062000565
NOROESTE	310620005	BELO HORIZONT	3106200	310620005	31062000565
NOROESTE	310620005	BELO HORIZONT	3106200	310620005	31062000565
NOROESTE	310620005	BELO HORIZONT	3106200	310620005	31062000565
NOROESTE	310620005	BELO HORIZONT	3106200	310620005	31062000565
NOROESTE	310620005	BELO HORIZONT	3106200	310620005	31062000565
NOROESTE	310620005	BELO HORIZONT	3106200	310620005	31062000565
NOROESTE	310620005	BELO HORIZONT	3106200	310620005	31062000565
NOROESTE	310620005	BELO HORIZONT	3106200	310620005	31062000565
NOROESTE	310620005	BELO HORIZONT	3106200	310620005	31062000565
NOROESTE	310620005	BELO HORIZONT	3106200	310620005	31062000565
NOROESTE	310620005	BELO HORIZONT	3106200	310620005	31062000565
NOROESTE	310620005	BELO HORIZONT	3106200	310620005	31062000565
NOROESTE	310620005	BELO HORIZONT	3106200	310620005	31062000565
NOROESTE	310620005	BELO HORIZONT	3106200	310620005	31062000565

NOROESTE	310620005	BELO HORIZONT	3106200	310620005	31062000565
NOROESTE	310620005	BELO HORIZONT	3106200	310620005	31062000565
NOROESTE	310620005	BELO HORIZONT	3106200	0	0
NOROESTE	310620005	BELO HORIZONT	3106200	310620005	31062000565
NOROESTE	310620005	BELO HORIZONT	3106200	310620005	31062000565
NOROESTE	310620005	BELO HORIZONT	3106200	310620005	31062000565
NOROESTE	310620005	BELO HORIZONT	3106200	310620005	31062000565
NOROESTE	310620005	BELO HORIZONT	3106200	310620005	31062000565
NOROESTE	310620005	BELO HORIZONT	3106200	310620005	31062000565
NOROESTE	310620005	BELO HORIZONT	3106200	310620005	31062000565
NOROESTE	310620005	BELO HORIZONT	3106200	310620005	31062000565
NOROESTE	310620005	BELO HORIZONT	3106200	310620005	31062000565
NOROESTE	310620005	BELO HORIZONT	3106200	310620005	31062000565
NOROESTE	310620005	BELO HORIZONT	3106200	310620005	31062000565
NOROESTE	310620005	BELO HORIZONT	3106200	310620005	31062000565
NOROESTE	310620005	BELO HORIZONT	3106200	310620005	31062000565
NOROESTE	310620005	BELO HORIZONT	3106200	310620005	31062000565
NOROESTE	310620005	BELO HORIZONT	3106200	310620005	31062000565
NOROESTE	310620005	BELO HORIZONT	3106200	310620005	31062000565
NOROESTE	310620005	BELO HORIZONT	3106200	310620005	31062000565
NOROESTE	310620005	BELO HORIZONT	3106200	310620005	31062000565
NOROESTE	310620005	BELO HORIZONT	3106200	310620005	31062000565
NOROESTE	310620005	BELO HORIZONT	3106200	310620005	31062000565
NOROESTE	310620005	BELO HORIZONT	3106200	310620005	31062000565
NOROESTE	310620005	BELO HORIZONT	3106200	310620005	31062000565
NOROESTE	310620005	BELO HORIZONT	3106200	310620005	31062000565
NOROESTE	310620005	BELO HORIZONT	3106200	310620005	31062000565
NOROESTE	310620005	BELO HORIZONT	3106200	310620005	31062000565
NOROESTE	310620005	BELO HORIZONT	3106200	310620005	31062000565
NOROESTE	310620005	BELO HORIZONT	3106200	310620005	31062000565
NOROESTE	310620005	BELO HORIZONT	3106200	310620005	31062000565
NOROESTE	310620005	BELO HORIZONT	3106200	310620005	31062000565
NOROESTE	310620005	BELO HORIZONT	3106200	310620005	31062000565
NOROESTE	310620005	BELO HORIZONT	3106200	310620005	31062000565
NOROESTE	310620005	BELO HORIZONT	3106200	310620005	31062000565
NOROESTE	310620005	BELO HORIZONT	3106200	310620005	31062000565
NOROESTE	310620005	BELO HORIZONT	3106200	310620005	31062000565
NOROESTE	310620005	BELO HORIZONT	3106200	310620005	31062000565
NOROESTE	310620005	BELO HORIZONT	3106200	310620005	31062000565
NOROESTE	310620005	BELO HORIZONT	3106200	310620005	31062000565
NOROESTE	310620005	BELO HORIZONT	3106200	310620005	31062000565
NOROESTE	310620005	BELO HORIZONT	3106200	310620005	31062000565
NOROESTE	310620005	BELO HORIZONT	3106200	310620005	31062000565
NOROESTE	310620005	BELO HORIZONT	3106200	310620005	31062000565
NOROESTE	310620005	BELO HORIZONT	3106200	310620005	31062000565
NOROESTE	310620005	BELO HORIZONT	3106200	310620005	31062000565
NOROESTE	310620005	BELO HORIZONT	3106200	310620005	31062000565
NOROESTE	310620005	BELO HORIZONT	3106200	310620005	31062000565
NOROESTE	310620005	BELO HORIZONT	3106200	310620005	31062000565
NOROESTE	310620005	BELO HORIZONT	3106200	310620005	31062000565

NOROESTE	310620005	BELO HORIZONT	3106200	310620005	31062000565
NOROESTE	310620005	BELO HORIZONT	3106200	310620005	31062000565
NOROESTE	310620005	BELO HORIZONT	3106200	310620005	31062000565
NOROESTE	310620005	BELO HORIZONT	3106200	310620005	31062000565
NOROESTE	310620005	BELO HORIZONT	3106200	310620005	31062000565
NOROESTE	310620005	BELO HORIZONT	3106200	310620005	31062000565
NOROESTE	310620005	BELO HORIZONT	3106200	310620005	31062000565
NOROESTE	310620005	BELO HORIZONT	3106200	310620005	31062000565
NOROESTE	310620005	BELO HORIZONT	3106200	310620005	31062000565
NOROESTE	310620005	BELO HORIZONT	3106200	310620005	31062000565
NOROESTE	310620005	BELO HORIZONT	3106200	310620005	31062000565
NOROESTE	310620005	BELO HORIZONT	3106200	310620005	31062000565
NOROESTE	310620005	BELO HORIZONT	3106200	310620005	31062000565
NOROESTE	310620005	BELO HORIZONT	3106200	310620005	31062000565
NOROESTE	310620005	BELO HORIZONT	3106200	310620005	31062000565
NOROESTE	310620005	BELO HORIZONT	3106200	310620005	31062000565
NOROESTE	310620005	BELO HORIZONT	3106200	310620005	31062000565
NOROESTE	310620005	BELO HORIZONT	3106200	310620005	31062000565
NOROESTE	310620005	BELO HORIZONT	3106200	310620005	31062000565
NOROESTE	310620005	BELO HORIZONT	3106200	310620005	31062000565
NOROESTE	310620005	BELO HORIZONT	3106200	310620005	31062000565
NOROESTE	310620005	BELO HORIZONT	3106200	310620005	31062000565
NOROESTE	310620005	BELO HORIZONT	3106200	310620005	31062000565
NOROESTE	310620005	BELO HORIZONT	3106200	310620005	31062000565
NOROESTE	310620005	BELO HORIZONT	3106200	310620005	31062000565
NOROESTE	310620005	BELO HORIZONT	3106200	310620005	31062000565
NOROESTE	310620005	BELO HORIZONT	3106200	310620005	31062000565
NOROESTE	310620005	BELO HORIZONT	3106200	310620005	31062000565
NOROESTE	310620005	BELO HORIZONT	3106200	310620005	31062000565
NOROESTE	310620005	BELO HORIZONT	3106200	0	0
NOROESTE	310620005	BELO HORIZONT	3106200	310620005	31062000565
NOROESTE	310620005	BELO HORIZONT	3106200	310620005	31062000565
NOROESTE	310620005	BELO HORIZONT	3106200	0	0
NOROESTE	310620005	BELO HORIZONT	3106200	310620005	31062000565
NOROESTE	310620005	BELO HORIZONT	3106200	310620005	31062000565
NOROESTE	310620005	BELO HORIZONT	3106200	310620005	31062000565
NOROESTE	310620005	BELO HORIZONT	3106200	310620005	31062000565
NOROESTE	310620005	BELO HORIZONT	3106200	310620005	31062000565
NOROESTE	310620005	BELO HORIZONT	3106200	310620005	31062000565
NOROESTE	310620005	BELO HORIZONT	3106200	310620005	31062000565
NOROESTE	310620005	BELO HORIZONT	3106200	310620005	31062000565
NOROESTE	310620005	BELO HORIZONT	3106200	310620005	31062000565
NOROESTE	310620005	BELO HORIZONT	3106200	310620005	31062000565
NOROESTE	310620005	BELO HORIZONT	3106200	310620005	31062000565
NOROESTE	310620005	BELO HORIZONT	3106200	310620005	31062000565
NOROESTE	310620005	BELO HORIZONT	3106200	310620005	31062000565
NOROESTE	310620005	BELO HORIZONT	3106200	310620005	31062000565
NOROESTE	310620005	BELO HORIZONT	3106200	310620005	31062000565
NOROESTE	310620005	BELO HORIZONT	3106200	310620005	31062000565
NOROESTE	310620005	BELO HORIZONT	3106200	310620005	31062000565

NOROESTE	310620005	BELO HORIZONT	3106200	310620005	31062000565
NOROESTE	310620005	BELO HORIZONT	3106200	310620005	31062000565
NOROESTE	310620005	BELO HORIZONT	3106200	310620005	31062000565
NOROESTE	310620005	BELO HORIZONT	3106200	310620005	31062000565
NOROESTE	310620005	BELO HORIZONT	3106200	310620005	31062000565
NOROESTE	310620005	BELO HORIZONT	3106200	310620005	31062000565
NOROESTE	310620005	BELO HORIZONT	3106200	310620005	31062000565
NOROESTE	310620005	BELO HORIZONT	3106200	310620005	31062000565
NOROESTE	310620005	BELO HORIZONT	3106200	310620005	31062000565
NOROESTE	310620005	BELO HORIZONT	3106200	310620005	31062000565
NOROESTE	310620005	BELO HORIZONT	3106200	310620005	31062000565
NOROESTE	310620005	BELO HORIZONT	3106200	310620005	31062000565
NOROESTE	310620005	BELO HORIZONT	3106200	310620005	31062000565
NOROESTE	310620005	BELO HORIZONT	3106200	310620005	31062000565
NOROESTE	310620005	BELO HORIZONT	3106200	310620005	31062000565
NOROESTE	310620005	BELO HORIZONT	3106200	310620005	31062000565
NOROESTE	310620005	BELO HORIZONT	3106200	310620005	31062000565
NOROESTE	310620005	BELO HORIZONT	3106200	310620005	31062000565
NOROESTE	310620005	BELO HORIZONT	3106200	310620005	31062000565
NOROESTE	310620005	BELO HORIZONT	3106200	310620005	31062000565
NOROESTE	310620005	BELO HORIZONT	3106200	310620005	31062000565
NOROESTE	310620005	BELO HORIZONT	3106200	310620005	31062000565
NOROESTE	310620005	BELO HORIZONT	3106200	310620005	31062000565
NOROESTE	310620005	BELO HORIZONT	3106200	310620005	31062000565
NOROESTE	310620005	BELO HORIZONT	3106200	310620005	31062000565
NOROESTE	310620005	BELO HORIZONT	3106200	310620005	31062000565
NOROESTE	310620005	BELO HORIZONT	3106200	310620005	31062000565
NOROESTE	310620005	BELO HORIZONT	3106200	310620005	31062000565
NOROESTE	310620005	BELO HORIZONT	3106200	310620005	31062000565
NOROESTE	310620005	BELO HORIZONT	3106200	310620005	31062000565
NOROESTE	310620005	BELO HORIZONT	3106200	310620005	31062000565
NOROESTE	310620005	BELO HORIZONT	3106200	310620005	31062000565
NOROESTE	310620005	BELO HORIZONT	3106200	0	0
NOROESTE	310620005	BELO HORIZONT	3106200	310620005	31062000565
NOROESTE	310620005	BELO HORIZONT	3106200	310620005	31062000565
NOROESTE	310620005	BELO HORIZONT	3106200	310620005	31062000565
NOROESTE	310620005	BELO HORIZONT	3106200	310620005	31062000565
NOROESTE	310620005	BELO HORIZONT	3106200	310620005	31062000565
NOROESTE	310620005	BELO HORIZONT	3106200	310620005	31062000565
NOROESTE	310620005	BELO HORIZONT	3106200	310620005	31062000565
NOROESTE	310620005	BELO HORIZONT	3106200	310620005	31062000565
NOROESTE	310620005	BELO HORIZONT	3106200	310620005	31062000565
NOROESTE	310620005	BELO HORIZONT	3106200	310620005	31062000565
NOROESTE	310620005	BELO HORIZONT	3106200	310620005	31062000565
NOROESTE	310620005	BELO HORIZONT	3106200	310620005	31062000565
NOROESTE	310620005	BELO HORIZONT	3106200	310620005	31062000565
OESTE	310620005	BELO HORIZONT	3106200	310620005	31062000567
OESTE	310620005	BELO HORIZONT	3106200	310620005	31062000567
OESTE	310620005	BELO HORIZONT	3106200	310620005	31062000567
OESTE	310620005	BELO HORIZONT	3106200	310620005	31062000567

OESTE	310620005	BELO HORIZONT	3106200	310620005	31062000567
OESTE	310620005	BELO HORIZONT	3106200	310620005	31062000567
OESTE	310620005	BELO HORIZONT	3106200	310620005	31062000567
OESTE	310620005	BELO HORIZONT	3106200	310620005	31062000567
OESTE	310620005	BELO HORIZONT	3106200	310620005	31062000567
OESTE	310620005	BELO HORIZONT	3106200	310620005	31062000567
OESTE	310620005	BELO HORIZONT	3106200	310620005	31062000567
OESTE	310620005	BELO HORIZONT	3106200	310620005	31062000567
OESTE	310620005	BELO HORIZONT	3106200	310620005	31062000567
OESTE	310620005	BELO HORIZONT	3106200	310620005	31062000567
OESTE	310620005	BELO HORIZONT	3106200	310620005	31062000567
OESTE	310620005	BELO HORIZONT	3106200	310620005	31062000567
OESTE	310620005	BELO HORIZONT	3106200	310620005	31062000567
OESTE	310620005	BELO HORIZONT	3106200	310620005	31062000567
OESTE	310620005	BELO HORIZONT	3106200	310620005	31062000567
OESTE	310620005	BELO HORIZONT	3106200	310620005	31062000567
OESTE	310620005	BELO HORIZONT	3106200	310620005	31062000567
OESTE	310620005	BELO HORIZONT	3106200	310620005	31062000567
OESTE	310620005	BELO HORIZONT	3106200	310620005	31062000567
OESTE	310620005	BELO HORIZONT	3106200	310620005	31062000567
OESTE	310620005	BELO HORIZONT	3106200	310620005	31062000567
OESTE	310620005	BELO HORIZONT	3106200	310620005	31062000567
OESTE	310620005	BELO HORIZONT	3106200	310620005	31062000567
OESTE	310620005	BELO HORIZONT	3106200	310620005	31062000567
OESTE	310620005	BELO HORIZONT	3106200	310620005	31062000567
OESTE	310620005	BELO HORIZONT	3106200	310620005	31062000567
OESTE	310620005	BELO HORIZONT	3106200	310620005	31062000567
OESTE	310620005	BELO HORIZONT	3106200	310620005	31062000567
OESTE	310620005	BELO HORIZONT	3106200	310620005	31062000567
OESTE	310620005	BELO HORIZONT	3106200	310620005	31062000567
OESTE	310620005	BELO HORIZONT	3106200	310620005	31062000567
OESTE	310620005	BELO HORIZONT	3106200	310620005	31062000567
OESTE	310620005	BELO HORIZONT	3106200	310620005	31062000567
OESTE	310620005	BELO HORIZONT	3106200	310620005	31062000567
OESTE	310620005	BELO HORIZONT	3106200	310620005	31062000567
OESTE	310620005	BELO HORIZONT	3106200	310620005	31062000567
OESTE	310620005	BELO HORIZONT	3106200	310620005	31062000567
OESTE	310620005	BELO HORIZONT	3106200	310620005	31062000567
OESTE	310620005	BELO HORIZONT	3106200	310620005	31062000567
OESTE	310620005	BELO HORIZONT	3106200	310620005	31062000567
OESTE	310620005	BELO HORIZONT	3106200	310620005	31062000567
OESTE	310620005	BELO HORIZONT	3106200	310620005	31062000567
OESTE	310620005	BELO HORIZONT	3106200	310620005	31062000567
OESTE	310620005	BELO HORIZONT	3106200	310620005	31062000567
OESTE	310620005	BELO HORIZONT	3106200	310620005	31062000567
OESTE	310620005	BELO HORIZONT	3106200	310620005	31062000567
OESTE	310620005	BELO HORIZONT	3106200	310620005	31062000567
OESTE	310620005	BELO HORIZONT	3106200	310620005	31062000567
OESTE	310620005	BELO HORIZONT	3106200	310620005	31062000567
OESTE	310620005	BELO HORIZONT	3106200	310620005	31062000567

OESTE	310620005	BELO HORIZONT	3106200	310620005	31062000567
OESTE	310620005	BELO HORIZONT	3106200	310620005	31062000567
OESTE	310620005	BELO HORIZONT	3106200	310620005	31062000567
OESTE	310620005	BELO HORIZONT	3106200	310620005	31062000567
OESTE	310620005	BELO HORIZONT	3106200	310620005	31062000567
OESTE	310620005	BELO HORIZONT	3106200	310620005	31062000567
OESTE	310620005	BELO HORIZONT	3106200	310620005	31062000567
OESTE	310620005	BELO HORIZONT	3106200	310620005	31062000567
OESTE	310620005	BELO HORIZONT	3106200	310620005	31062000567
OESTE	310620005	BELO HORIZONT	3106200	310620005	31062000567
OESTE	310620005	BELO HORIZONT	3106200	310620005	31062000567
OESTE	310620005	BELO HORIZONT	3106200	310620005	31062000567
OESTE	310620005	BELO HORIZONT	3106200	310620005	31062000567
OESTE	310620005	BELO HORIZONT	3106200	310620005	31062000567
OESTE	310620005	BELO HORIZONT	3106200	310620005	31062000567
OESTE	310620005	BELO HORIZONT	3106200	310620005	31062000567
OESTE	310620005	BELO HORIZONT	3106200	310620005	31062000567
OESTE	310620005	BELO HORIZONT	3106200	310620005	31062000567
OESTE	310620005	BELO HORIZONT	3106200	310620005	31062000567
OESTE	310620005	BELO HORIZONT	3106200	310620005	31062000567
OESTE	310620005	BELO HORIZONT	3106200	310620005	31062000567
OESTE	310620005	BELO HORIZONT	3106200	310620005	31062000567
OESTE	310620005	BELO HORIZONT	3106200	310620005	31062000567
OESTE	310620005	BELO HORIZONT	3106200	310620005	31062000567
OESTE	310620005	BELO HORIZONT	3106200	310620005	31062000567
OESTE	310620005	BELO HORIZONT	3106200	310620005	31062000567
OESTE	310620005	BELO HORIZONT	3106200	310620005	31062000567
OESTE	310620005	BELO HORIZONT	3106200	310620005	31062000567
OESTE	310620005	BELO HORIZONT	3106200	310620005	31062000567
OESTE	310620005	BELO HORIZONT	3106200	310620005	31062000567
OESTE	310620005	BELO HORIZONT	3106200	310620005	31062000567
OESTE	310620005	BELO HORIZONT	3106200	310620005	31062000567
OESTE	310620005	BELO HORIZONT	3106200	310620005	31062000567
OESTE	310620005	BELO HORIZONT	3106200	310620005	31062000567
OESTE	310620005	BELO HORIZONT	3106200	310620005	31062000567
OESTE	310620005	BELO HORIZONT	3106200	310620005	31062000567
OESTE	310620005	BELO HORIZONT	3106200	310620005	31062000567
OESTE	310620005	BELO HORIZONT	3106200	310620005	31062000567
OESTE	310620005	BELO HORIZONT	3106200	310620005	31062000567
OESTE	310620005	BELO HORIZONT	3106200	310620005	31062000567
OESTE	310620005	BELO HORIZONT	3106200	310620005	31062000567
OESTE	310620005	BELO HORIZONT	3106200	310620005	31062000567
OESTE	310620005	BELO HORIZONT	3106200	310620005	31062000567
OESTE	310620005	BELO HORIZONT	3106200	310620005	31062000567
OESTE	310620005	BELO HORIZONT	3106200	310620005	31062000567
OESTE	310620005	BELO HORIZONT	3106200	310620005	31062000567
OESTE	310620005	BELO HORIZONT	3106200	310620005	31062000567
OESTE	310620005	BELO HORIZONT	3106200	310620005	31062000567
OESTE	310620005	BELO HORIZONT	3106200	310620005	31062000567
OESTE	310620005	BELO HORIZONT	3106200	310620005	31062000567

OESTE	310620005	BELO HORIZONT	3106200	310620005	31062000567
OESTE	310620005	BELO HORIZONT	3106200	310620005	31062000567
OESTE	310620005	BELO HORIZONT	3106200	310620005	31062000567
OESTE	310620005	BELO HORIZONT	3106200	310620005	31062000567
OESTE	310620005	BELO HORIZONT	3106200	310620005	31062000567
OESTE	310620005	BELO HORIZONT	3106200	310620005	31062000567
OESTE	310620005	BELO HORIZONT	3106200	310620005	31062000567
OESTE	310620005	BELO HORIZONT	3106200	310620005	31062000567
OESTE	310620005	BELO HORIZONT	3106200	310620005	31062000567
OESTE	310620005	BELO HORIZONT	3106200	310620005	31062000567
OESTE	310620005	BELO HORIZONT	3106200	310620005	31062000567
OESTE	310620005	BELO HORIZONT	3106200	310620005	31062000567
OESTE	310620005	BELO HORIZONT	3106200	310620005	31062000567
OESTE	310620005	BELO HORIZONT	3106200	310620005	31062000567
OESTE	310620005	BELO HORIZONT	3106200	310620005	31062000567
OESTE	310620005	BELO HORIZONT	3106200	310620005	31062000567
OESTE	310620005	BELO HORIZONT	3106200	310620005	31062000567
OESTE	310620005	BELO HORIZONT	3106200	310620005	31062000567
OESTE	310620005	BELO HORIZONT	3106200	310620005	31062000567
OESTE	310620005	BELO HORIZONT	3106200	310620005	31062000567
OESTE	310620005	BELO HORIZONT	3106200	310620005	31062000567
OESTE	310620005	BELO HORIZONT	3106200	310620005	31062000567
OESTE	310620005	BELO HORIZONT	3106200	310620005	31062000567
OESTE	310620005	BELO HORIZONT	3106200	310620005	31062000567
OESTE	310620005	BELO HORIZONT	3106200	310620005	31062000567
OESTE	310620005	BELO HORIZONT	3106200	310620005	31062000567
OESTE	310620005	BELO HORIZONT	3106200	310620005	31062000567
OESTE	310620005	BELO HORIZONT	3106200	310620005	31062000567
OESTE	310620005	BELO HORIZONT	3106200	310620005	31062000567
OESTE	310620005	BELO HORIZONT	3106200	310620005	31062000567
OESTE	310620005	BELO HORIZONT	3106200	310620005	31062000567
OESTE	310620005	BELO HORIZONT	3106200	310620005	31062000567
OESTE	310620005	BELO HORIZONT	3106200	310620005	31062000567
OESTE	310620005	BELO HORIZONT	3106200	310620005	31062000567
OESTE	310620005	BELO HORIZONT	3106200	310620005	31062000567
OESTE	310620005	BELO HORIZONT	3106200	310620005	31062000567
OESTE	310620005	BELO HORIZONT	3106200	310620005	31062000567
OESTE	310620005	BELO HORIZONT	3106200	310620005	31062000567
OESTE	310620005	BELO HORIZONT	3106200	310620005	31062000567
OESTE	310620005	BELO HORIZONT	3106200	310620005	31062000567
OESTE	310620005	BELO HORIZONT	3106200	310620005	31062000567
OESTE	310620005	BELO HORIZONT	3106200	310620005	31062000567
OESTE	310620005	BELO HORIZONT	3106200	310620005	31062000567
OESTE	310620005	BELO HORIZONT	3106200	310620005	31062000567
OESTE	310620005	BELO HORIZONT	3106200	310620005	31062000567
OESTE	310620005	BELO HORIZONT	3106200	310620005	31062000567
OESTE	310620005	BELO HORIZONT	3106200	310620005	31062000567
OESTE	310620005	BELO HORIZONT	3106200	310620005	31062000567
OESTE	310620005	BELO HORIZONT	3106200	310620005	31062000567
OESTE	310620005	BELO HORIZONT	3106200	310620005	31062000567

OESTE	310620005	BELO HORIZONT	3106200	310620005	31062000567
OESTE	310620005	BELO HORIZONT	3106200	310620005	31062000567
OESTE	310620005	BELO HORIZONT	3106200	310620005	31062000567
OESTE	310620005	BELO HORIZONT	3106200	310620005	31062000567
OESTE	310620005	BELO HORIZONT	3106200	310620005	31062000567
OESTE	310620005	BELO HORIZONT	3106200	310620005	31062000567
OESTE	310620005	BELO HORIZONT	3106200	310620005	31062000567
OESTE	310620005	BELO HORIZONT	3106200	310620005	31062000567
OESTE	310620005	BELO HORIZONT	3106200	310620005	31062000567
OESTE	310620005	BELO HORIZONT	3106200	310620005	31062000567
OESTE	310620005	BELO HORIZONT	3106200	310620005	31062000567
OESTE	310620005	BELO HORIZONT	3106200	310620005	31062000567
OESTE	310620005	BELO HORIZONT	3106200	310620005	31062000567
OESTE	310620005	BELO HORIZONT	3106200	310620005	31062000567
OESTE	310620005	BELO HORIZONT	3106200	310620005	31062000567
OESTE	310620005	BELO HORIZONT	3106200	310620005	31062000567
OESTE	310620005	BELO HORIZONT	3106200	310620005	31062000567
OESTE	310620005	BELO HORIZONT	3106200	310620005	31062000567
OESTE	310620005	BELO HORIZONT	3106200	310620005	31062000567
OESTE	310620005	BELO HORIZONT	3106200	310620005	31062000567
OESTE	310620005	BELO HORIZONT	3106200	310620005	31062000567
OESTE	310620005	BELO HORIZONT	3106200	310620005	31062000567
OESTE	310620005	BELO HORIZONT	3106200	310620005	31062000567
OESTE	310620005	BELO HORIZONT	3106200	310620005	31062000567
OESTE	310620005	BELO HORIZONT	3106200	310620005	31062000567
OESTE	310620005	BELO HORIZONT	3106200	310620005	31062000567
OESTE	310620005	BELO HORIZONT	3106200	310620005	31062000567
OESTE	310620005	BELO HORIZONT	3106200	310620005	31062000567
OESTE	310620005	BELO HORIZONT	3106200	310620005	31062000567
OESTE	310620005	BELO HORIZONT	3106200	310620005	31062000567
OESTE	310620005	BELO HORIZONT	3106200	310620005	31062000567
OESTE	310620005	BELO HORIZONT	3106200	310620005	31062000567
OESTE	310620005	BELO HORIZONT	3106200	310620005	31062000567
OESTE	310620005	BELO HORIZONT	3106200	310620005	31062000567
OESTE	310620005	BELO HORIZONT	3106200	310620005	31062000567
OESTE	310620005	BELO HORIZONT	3106200	310620005	31062000567
OESTE	310620005	BELO HORIZONT	3106200	310620005	31062000567
OESTE	310620005	BELO HORIZONT	3106200	310620005	31062000567
OESTE	310620005	BELO HORIZONT	3106200	310620005	31062000567
OESTE	310620005	BELO HORIZONT	3106200	310620005	31062000567
OESTE	310620005	BELO HORIZONT	3106200	310620005	31062000567
OESTE	310620005	BELO HORIZONT	3106200	310620005	31062000567
OESTE	310620005	BELO HORIZONT	3106200	310620005	31062000567
OESTE	310620005	BELO HORIZONT	3106200	310620005	31062000567
OESTE	310620005	BELO HORIZONT	3106200	310620005	31062000567
OESTE	310620005	BELO HORIZONT	3106200	310620005	31062000567
OESTE	310620005	BELO HORIZONT	3106200	310620005	31062000567
OESTE	310620005	BELO HORIZONT	3106200	310620005	31062000567
OESTE	310620005	BELO HORIZONT	3106200	310620005	31062000567
OESTE	310620005	BELO HORIZONT	3106200	310620005	31062000567

OESTE	310620005	BELO HORIZONT	3106200	310620005	31062000567
OESTE	310620005	BELO HORIZONT	3106200	310620005	31062000567
OESTE	310620005	BELO HORIZONT	3106200	310620005	31062000567
OESTE	310620005	BELO HORIZONT	3106200	310620005	31062000567
OESTE	310620005	BELO HORIZONT	3106200	310620005	31062000567
OESTE	310620005	BELO HORIZONT	3106200	310620005	31062000567
OESTE	310620005	BELO HORIZONT	3106200	310620005	31062000567
OESTE	310620005	BELO HORIZONT	3106200	310620005	31062000567
OESTE	310620005	BELO HORIZONT	3106200	310620005	31062000567
OESTE	310620005	BELO HORIZONT	3106200	310620005	31062000567
OESTE	310620005	BELO HORIZONT	3106200	310620005	31062000567
OESTE	310620005	BELO HORIZONT	3106200	310620005	31062000567
OESTE	310620005	BELO HORIZONT	3106200	310620005	31062000567
OESTE	310620005	BELO HORIZONT	3106200	310620005	31062000567
OESTE	310620005	BELO HORIZONT	3106200	310620005	31062000567
OESTE	310620005	BELO HORIZONT	3106200	310620005	31062000567
OESTE	310620005	BELO HORIZONT	3106200	310620005	31062000567
OESTE	310620005	BELO HORIZONT	3106200	310620005	31062000567
OESTE	310620005	BELO HORIZONT	3106200	310620005	31062000567
OESTE	310620005	BELO HORIZONT	3106200	310620005	31062000567
OESTE	310620005	BELO HORIZONT	3106200	310620005	31062000567
OESTE	310620005	BELO HORIZONT	3106200	310620005	31062000567
OESTE	310620005	BELO HORIZONT	3106200	310620005	31062000567
OESTE	310620005	BELO HORIZONT	3106200	310620005	31062000567
OESTE	310620005	BELO HORIZONT	3106200	310620005	31062000567
OESTE	310620005	BELO HORIZONT	3106200	310620005	31062000567
OESTE	310620005	BELO HORIZONT	3106200	310620005	31062000567
OESTE	310620005	BELO HORIZONT	3106200	310620005	31062000567
OESTE	310620005	BELO HORIZONT	3106200	310620005	31062000567
OESTE	310620005	BELO HORIZONT	3106200	310620005	31062000567
OESTE	310620005	BELO HORIZONT	3106200	310620005	31062000567
OESTE	310620005	BELO HORIZONT	3106200	310620005	31062000567
OESTE	310620005	BELO HORIZONT	3106200	310620005	31062000567
OESTE	310620005	BELO HORIZONT	3106200	310620005	31062000567
OESTE	310620005	BELO HORIZONT	3106200	310620005	31062000567
OESTE	310620005	BELO HORIZONT	3106200	310620005	31062000567
OESTE	310620005	BELO HORIZONT	3106200	310620005	31062000567
OESTE	310620005	BELO HORIZONT	3106200	310620005	31062000567
OESTE	310620005	BELO HORIZONT	3106200	310620005	31062000567
OESTE	310620005	BELO HORIZONT	3106200	310620005	31062000567
OESTE	310620005	BELO HORIZONT	3106200	310620005	31062000567
OESTE	310620005	BELO HORIZONT	3106200	310620005	31062000567
OESTE	310620005	BELO HORIZONT	3106200	310620005	31062000567
OESTE	310620005	BELO HORIZONT	3106200	310620005	31062000567
OESTE	310620005	BELO HORIZONT	3106200	310620005	31062000567
OESTE	310620005	BELO HORIZONT	3106200	310620005	31062000567
OESTE	310620005	BELO HORIZONT	3106200	310620005	31062000567
OESTE	310620005	BELO HORIZONT	3106200	310620005	31062000567
OESTE	310620005	BELO HORIZONT	3106200	310620005	31062000567
OESTE	310620005	BELO HORIZONT	3106200	310620005	31062000567

OESTE	310620005	BELO HORIZONT	3106200	310620005	31062000567
OESTE	310620005	BELO HORIZONT	3106200	310620005	31062000567
OESTE	310620005	BELO HORIZONT	3106200	310620005	31062000567
OESTE	310620005	BELO HORIZONT	3106200	310620005	31062000567
OESTE	310620005	BELO HORIZONT	3106200	310620005	31062000567
OESTE	310620005	BELO HORIZONT	3106200	310620005	31062000567
OESTE	310620005	BELO HORIZONT	3106200	310620005	31062000567
OESTE	310620005	BELO HORIZONT	3106200	310620005	31062000567
OESTE	310620005	BELO HORIZONT	3106200	310620005	31062000567
OESTE	310620005	BELO HORIZONT	3106200	310620005	31062000567
OESTE	310620005	BELO HORIZONT	3106200	310620005	31062000567
OESTE	310620005	BELO HORIZONT	3106200	310620005	31062000567
OESTE	310620005	BELO HORIZONT	3106200	310620005	31062000567
OESTE	310620005	BELO HORIZONT	3106200	310620005	31062000567
OESTE	310620005	BELO HORIZONT	3106200	310620005	31062000567
OESTE	310620005	BELO HORIZONT	3106200	310620005	31062000567
OESTE	310620005	BELO HORIZONT	3106200	310620005	31062000567
OESTE	310620005	BELO HORIZONT	3106200	310620005	31062000567
OESTE	310620005	BELO HORIZONT	3106200	310620005	31062000567
OESTE	310620005	BELO HORIZONT	3106200	310620005	31062000567
OESTE	310620005	BELO HORIZONT	3106200	310620005	31062000567
OESTE	310620005	BELO HORIZONT	3106200	310620005	31062000567
OESTE	310620005	BELO HORIZONT	3106200	310620005	31062000567
OESTE	310620005	BELO HORIZONT	3106200	310620005	31062000567
OESTE	310620005	BELO HORIZONT	3106200	310620005	31062000567
OESTE	310620005	BELO HORIZONT	3106200	310620005	31062000567
OESTE	310620005	BELO HORIZONT	3106200	310620005	31062000567
OESTE	310620005	BELO HORIZONT	3106200	310620005	31062000567
OESTE	310620005	BELO HORIZONT	3106200	310620005	31062000567
OESTE	310620005	BELO HORIZONT	3106200	310620005	31062000567
OESTE	310620005	BELO HORIZONT	3106200	310620005	31062000567
OESTE	310620005	BELO HORIZONT	3106200	310620005	31062000567
OESTE	310620005	BELO HORIZONT	3106200	310620005	31062000567
OESTE	310620005	BELO HORIZONT	3106200	310620005	31062000567
OESTE	310620005	BELO HORIZONT	3106200	310620005	31062000567
OESTE	310620005	BELO HORIZONT	3106200	310620005	31062000567
OESTE	310620005	BELO HORIZONT	3106200	310620005	31062000567
OESTE	310620005	BELO HORIZONT	3106200	310620005	31062000567
OESTE	310620005	BELO HORIZONT	3106200	310620005	31062000567
OESTE	310620005	BELO HORIZONT	3106200	310620005	31062000567
OESTE	310620005	BELO HORIZONT	3106200	310620005	31062000567
OESTE	310620005	BELO HORIZONT	3106200	310620005	31062000567
OESTE	310620005	BELO HORIZONT	3106200	310620005	31062000567
OESTE	310620005	BELO HORIZONT	3106200	310620005	31062000567
OESTE	310620005	BELO HORIZONT	3106200	310620005	31062000567
OESTE	310620005	BELO HORIZONT	3106200	310620005	31062000567
OESTE	310620005	BELO HORIZONT	3106200	310620005	31062000567
OESTE	310620005	BELO HORIZONT	3106200	310620005	31062000567
OESTE	310620005	BELO HORIZONT	3106200	310620005	31062000567
OESTE	310620005	BELO HORIZONT	3106200	310620005	31062000567

OESTE	310620005	BELO HORIZONT	3106200	310620005	31062000567
OESTE	310620005	BELO HORIZONT	3106200	310620005	31062000567
OESTE	310620005	BELO HORIZONT	3106200	310620005	31062000567
OESTE	310620005	BELO HORIZONT	3106200	310620005	31062000567
OESTE	310620005	BELO HORIZONT	3106200	310620005	31062000567
OESTE	310620005	BELO HORIZONT	3106200	310620005	31062000567
OESTE	310620005	BELO HORIZONT	3106200	310620005	31062000567
OESTE	310620005	BELO HORIZONT	3106200	310620005	31062000567
OESTE	310620005	BELO HORIZONT	3106200	310620005	31062000567
OESTE	310620005	BELO HORIZONT	3106200	310620005	31062000567
OESTE	310620005	BELO HORIZONT	3106200	310620005	31062000567
OESTE	310620005	BELO HORIZONT	3106200	310620005	31062000567
OESTE	310620005	BELO HORIZONT	3106200	310620005	31062000567
OESTE	310620005	BELO HORIZONT	3106200	310620005	31062000567
OESTE	310620005	BELO HORIZONT	3106200	310620005	31062000567
OESTE	310620005	BELO HORIZONT	3106200	310620005	31062000567
OESTE	310620005	BELO HORIZONT	3106200	310620005	31062000567
OESTE	310620005	BELO HORIZONT	3106200	310620005	31062000567
OESTE	310620005	BELO HORIZONT	3106200	310620005	31062000567
OESTE	310620005	BELO HORIZONT	3106200	310620005	31062000567
OESTE	310620005	BELO HORIZONT	3106200	310620005	31062000567
OESTE	310620005	BELO HORIZONT	3106200	310620005	31062000567
OESTE	310620005	BELO HORIZONT	3106200	310620005	31062000567
OESTE	310620005	BELO HORIZONT	3106200	310620005	31062000567
OESTE	310620005	BELO HORIZONT	3106200	310620005	31062000567
OESTE	310620005	BELO HORIZONT	3106200	310620005	31062000567
OESTE	310620005	BELO HORIZONT	3106200	310620005	31062000567
OESTE	310620005	BELO HORIZONT	3106200	310620005	31062000567
OESTE	310620005	BELO HORIZONT	3106200	310620005	31062000567
OESTE	310620005	BELO HORIZONT	3106200	310620005	31062000567
OESTE	310620005	BELO HORIZONT	3106200	310620005	31062000567
OESTE	310620005	BELO HORIZONT	3106200	310620005	31062000567
OESTE	310620005	BELO HORIZONT	3106200	310620005	31062000567
OESTE	310620005	BELO HORIZONT	3106200	310620005	31062000567
OESTE	310620005	BELO HORIZONT	3106200	310620005	31062000567
OESTE	310620005	BELO HORIZONT	3106200	310620005	31062000567
OESTE	310620005	BELO HORIZONT	3106200	310620005	31062000567
OESTE	310620005	BELO HORIZONT	3106200	310620005	31062000567
OESTE	310620005	BELO HORIZONT	3106200	310620005	31062000567
OESTE	310620005	BELO HORIZONT	3106200	310620005	31062000567
OESTE	310620005	BELO HORIZONT	3106200	310620005	31062000567
OESTE	310620005	BELO HORIZONT	3106200	310620005	31062000567
OESTE	310620005	BELO HORIZONT	3106200	310620005	31062000567
OESTE	310620005	BELO HORIZONT	3106200	310620005	31062000567
OESTE	310620005	BELO HORIZONT	3106200	310620005	31062000567
OESTE	310620005	BELO HORIZONT	3106200	310620005	31062000567
OESTE	310620005	BELO HORIZONT	3106200	310620005	31062000567
OESTE	310620005	BELO HORIZONT	3106200	310620005	31062000567
OESTE	310620005	BELO HORIZONT	3106200	310620005	31062000567
OESTE	310620005	BELO HORIZONT	3106200	310620005	31062000567

OESTE	310620005	BELO HORIZONT	3106200	310620005	31062000567
OESTE	310620005	BELO HORIZONT	3106200	310620005	31062000567
OESTE	310620005	BELO HORIZONT	3106200	310620005	31062000567
OESTE	310620005	BELO HORIZONT	3106200	310620005	31062000567
OESTE	310620005	BELO HORIZONT	3106200	310620005	31062000567
OESTE	310620005	BELO HORIZONT	3106200	310620005	31062000567
OESTE	310620005	BELO HORIZONT	3106200	310620005	31062000567
OESTE	310620005	BELO HORIZONT	3106200	310620005	31062000567
OESTE	310620005	BELO HORIZONT	3106200	310620005	31062000567
OESTE	310620005	BELO HORIZONT	3106200	310620005	31062000567
OESTE	310620005	BELO HORIZONT	3106200	310620005	31062000567
OESTE	310620005	BELO HORIZONT	3106200	310620005	31062000567
OESTE	310620005	BELO HORIZONT	3106200	310620005	31062000567
OESTE	310620005	BELO HORIZONT	3106200	310620005	31062000567
OESTE	310620005	BELO HORIZONT	3106200	310620005	31062000567
PAMPULHA	310620005	BELO HORIZONT	3106200	310620005	31062000568
PAMPULHA	310620005	BELO HORIZONT	3106200	310620005	31062000568
OESTE	310620005	BELO HORIZONT	3106200	310620005	31062000567
OESTE	310620005	BELO HORIZONT	3106200	310620005	31062000567
OESTE	310620005	BELO HORIZONT	3106200	310620005	31062000567
OESTE	310620005	BELO HORIZONT	3106200	310620005	31062000567
OESTE	310620005	BELO HORIZONT	3106200	310620005	31062000567
OESTE	310620005	BELO HORIZONT	3106200	310620005	31062000567
OESTE	310620005	BELO HORIZONT	3106200	310620005	31062000567
OESTE	310620005	BELO HORIZONT	3106200	310620005	31062000567
OESTE	310620005	BELO HORIZONT	3106200	310620005	31062000567
OESTE	310620005	BELO HORIZONT	3106200	310620005	31062000567
OESTE	310620005	BELO HORIZONT	3106200	310620005	31062000567
OESTE	310620005	BELO HORIZONT	3106200	310620005	31062000567
OESTE	310620005	BELO HORIZONT	3106200	310620005	31062000567
OESTE	310620005	BELO HORIZONT	3106200	310620005	31062000567
OESTE	310620005	BELO HORIZONT	3106200	310620005	31062000567
OESTE	310620005	BELO HORIZONT	3106200	310620005	31062000567
OESTE	310620005	BELO HORIZONT	3106200	310620005	31062000567
OESTE	310620005	BELO HORIZONT	3106200	310620005	31062000567
OESTE	310620005	BELO HORIZONT	3106200	310620005	31062000567
OESTE	310620005	BELO HORIZONT	3106200	310620005	31062000567
OESTE	310620005	BELO HORIZONT	3106200	310620005	31062000567
OESTE	310620005	BELO HORIZONT	3106200	310620005	31062000567
OESTE	310620005	BELO HORIZONT	3106200	310620005	31062000567
OESTE	310620005	BELO HORIZONT	3106200	310620005	31062000567
OESTE	310620005	BELO HORIZONT	3106200	310620005	31062000567
OESTE	310620005	BELO HORIZONT	3106200	310620005	31062000567
OESTE	310620005	BELO HORIZONT	3106200	310620005	31062000567
OESTE	310620005	BELO HORIZONT	3106200	310620005	31062000567
OESTE	310620005	BELO HORIZONT	3106200	310620005	31062000567
OESTE	310620005	BELO HORIZONT	3106200	310620005	31062000567
OESTE	310620005	BELO HORIZONT	3106200	310620005	31062000567
OESTE	310620005	BELO HORIZONT	3106200	310620005	31062000567
OESTE	310620005	BELO HORIZONT	3106200	310620005	31062000567

OESTE	310620005	BELO HORIZONT	3106200	310620005	31062000567
OESTE	310620005	BELO HORIZONT	3106200	310620005	31062000567
OESTE	310620005	BELO HORIZONT	3106200	310620005	31062000567
OESTE	310620005	BELO HORIZONT	3106200	310620005	31062000567
OESTE	310620005	BELO HORIZONT	3106200	310620005	31062000567
OESTE	310620005	BELO HORIZONT	3106200	310620005	31062000567
OESTE	310620005	BELO HORIZONT	3106200	310620005	31062000567
OESTE	310620005	BELO HORIZONT	3106200	310620005	31062000567
OESTE	310620005	BELO HORIZONT	3106200	310620005	31062000567
OESTE	310620005	BELO HORIZONT	3106200	310620005	31062000567
OESTE	310620005	BELO HORIZONT	3106200	310620005	31062000567
OESTE	310620005	BELO HORIZONT	3106200	310620005	31062000567
OESTE	310620005	BELO HORIZONT	3106200	310620005	31062000567
OESTE	310620005	BELO HORIZONT	3106200	310620005	31062000567
OESTE	310620005	BELO HORIZONT	3106200	310620005	31062000567
OESTE	310620005	BELO HORIZONT	3106200	310620005	31062000567
OESTE	310620005	BELO HORIZONT	3106200	310620005	31062000567
OESTE	310620005	BELO HORIZONT	3106200	0	0
OESTE	310620005	BELO HORIZONT	3106200	310620005	31062000567
OESTE	310620005	BELO HORIZONT	3106200	310620005	31062000567
OESTE	310620005	BELO HORIZONT	3106200	310620005	31062000567
OESTE	310620005	BELO HORIZONT	3106200	310620005	31062000567
OESTE	310620005	BELO HORIZONT	3106200	310620005	31062000567
OESTE	310620005	BELO HORIZONT	3106200	310620005	31062000567
OESTE	310620005	BELO HORIZONT	3106200	310620005	31062000567
OESTE	310620005	BELO HORIZONT	3106200	310620005	31062000567
OESTE	310620005	BELO HORIZONT	3106200	310620005	31062000567
OESTE	310620005	BELO HORIZONT	3106200	310620005	31062000567
OESTE	310620005	BELO HORIZONT	3106200	310620005	31062000567
OESTE	310620005	BELO HORIZONT	3106200	310620005	31062000567
OESTE	310620005	BELO HORIZONT	3106200	310620005	31062000567
OESTE	310620005	BELO HORIZONT	3106200	310620005	31062000567
OESTE	310620005	BELO HORIZONT	3106200	310620005	31062000567
OESTE	310620005	BELO HORIZONT	3106200	310620005	31062000567
OESTE	310620005	BELO HORIZONT	3106200	310620005	31062000567
OESTE	310620005	BELO HORIZONT	3106200	310620005	31062000567
OESTE	310620005	BELO HORIZONT	3106200	310620005	31062000567
OESTE	310620005	BELO HORIZONT	3106200	310620005	31062000567
OESTE	310620005	BELO HORIZONT	3106200	310620005	31062000567
OESTE	310620005	BELO HORIZONT	3106200	310620005	31062000567
OESTE	310620005	BELO HORIZONT	3106200	310620005	31062000567
OESTE	310620005	BELO HORIZONT	3106200	310620005	31062000567
OESTE	310620005	BELO HORIZONT	3106200	310620005	31062000567
OESTE	310620005	BELO HORIZONT	3106200	310620005	31062000567
OESTE	310620005	BELO HORIZONT	3106200	310620005	31062000567
OESTE	310620005	BELO HORIZONT	3106200	310620005	31062000567
OESTE	310620005	BELO HORIZONT	3106200	310620005	31062000567
OESTE	310620005	BELO HORIZONT	3106200	310620005	31062000567
OESTE	310620005	BELO HORIZONT	3106200	310620005	31062000567
OESTE	310620005	BELO HORIZONT	3106200	310620005	31062000567

OESTE	310620005	BELO HORIZONT	3106200	310620005	31062000567
OESTE	310620005	BELO HORIZONT	3106200	310620005	31062000567
OESTE	310620005	BELO HORIZONT	3106200	310620005	31062000567
OESTE	310620005	BELO HORIZONT	3106200	310620005	31062000567
OESTE	310620005	BELO HORIZONT	3106200	310620005	31062000567
OESTE	310620005	BELO HORIZONT	3106200	310620005	31062000567
OESTE	310620005	BELO HORIZONT	3106200	310620005	31062000567
OESTE	310620005	BELO HORIZONT	3106200	310620005	31062000567
OESTE	310620005	BELO HORIZONT	3106200	310620005	31062000567
OESTE	310620005	BELO HORIZONT	3106200	310620005	31062000567
OESTE	310620005	BELO HORIZONT	3106200	310620005	31062000567
OESTE	310620005	BELO HORIZONT	3106200	310620005	31062000567
OESTE	310620005	BELO HORIZONT	3106200	310620005	31062000567
OESTE	310620005	BELO HORIZONT	3106200	310620005	31062000567
OESTE	310620005	BELO HORIZONT	3106200	310620005	31062000567
OESTE	310620005	BELO HORIZONT	3106200	310620005	31062000567
OESTE	310620005	BELO HORIZONT	3106200	310620005	31062000567
OESTE	310620005	BELO HORIZONT	3106200	310620005	31062000567
OESTE	310620005	BELO HORIZONT	3106200	310620005	31062000567
OESTE	310620005	BELO HORIZONT	3106200	310620005	31062000567
OESTE	310620005	BELO HORIZONT	3106200	310620005	31062000567
OESTE	310620005	BELO HORIZONT	3106200	310620005	31062000567
OESTE	310620005	BELO HORIZONT	3106200	310620005	31062000567
OESTE	310620005	BELO HORIZONT	3106200	310620005	31062000567
OESTE	310620005	BELO HORIZONT	3106200	0	0
PAMPULHA	310620005	BELO HORIZONT	3106200	310620005	31062000568
PAMPULHA	310620005	BELO HORIZONT	3106200	310620005	31062000568
PAMPULHA	310620005	BELO HORIZONT	3106200	310620005	31062000568
PAMPULHA	310620005	BELO HORIZONT	3106200	310620005	31062000568
PAMPULHA	310620005	BELO HORIZONT	3106200	310620005	31062000568
PAMPULHA	310620005	BELO HORIZONT	3106200	310620005	31062000568
PAMPULHA	310620005	BELO HORIZONT	3106200	310620005	31062000568
PAMPULHA	310620005	BELO HORIZONT	3106200	310620005	31062000568
PAMPULHA	310620005	BELO HORIZONT	3106200	310620005	31062000568
PAMPULHA	310620005	BELO HORIZONT	3106200	310620005	31062000568
PAMPULHA	310620005	BELO HORIZONT	3106200	310620005	31062000568
PAMPULHA	310620005	BELO HORIZONT	3106200	310620005	31062000568
PAMPULHA	310620005	BELO HORIZONT	3106200	310620005	31062000568
PAMPULHA	310620005	BELO HORIZONT	3106200	0	0
PAMPULHA	310620005	BELO HORIZONT	3106200	310620005	31062000568
PAMPULHA	310620005	BELO HORIZONT	3106200	310620005	31062000568
PAMPULHA	310620005	BELO HORIZONT	3106200	310620005	31062000568
PAMPULHA	310620005	BELO HORIZONT	3106200	310620005	31062000568
PAMPULHA	310620005	BELO HORIZONT	3106200	310620005	31062000568
PAMPULHA	310620005	BELO HORIZONT	3106200	0	0
PAMPULHA	310620005	BELO HORIZONT	3106200	310620005	31062000568
PAMPULHA	310620005	BELO HORIZONT	3106200	310620005	31062000568
PAMPULHA	310620005	BELO HORIZONT	3106200	310620005	31062000568
PAMPULHA	310620005	BELO HORIZONT	3106200	310620005	31062000568
PAMPULHA	310620005	BELO HORIZONT	3106200	310620005	31062000568

PAMPULHA	310620005	BELO HORIZONT	3106200	310620005	31062000568
PAMPULHA	310620005	BELO HORIZONT	3106200	310620005	31062000568
PAMPULHA	310620005	BELO HORIZONT	3106200	310620005	31062000568
PAMPULHA	310620005	BELO HORIZONT	3106200	310620005	31062000568
PAMPULHA	310620005	BELO HORIZONT	3106200	310620005	31062000568
PAMPULHA	310620005	BELO HORIZONT	3106200	310620005	31062000568
PAMPULHA	310620005	BELO HORIZONT	3106200	310620005	31062000568
PAMPULHA	310620005	BELO HORIZONT	3106200	310620005	31062000568
PAMPULHA	310620005	BELO HORIZONT	3106200	310620005	31062000568
PAMPULHA	310620005	BELO HORIZONT	3106200	310620005	31062000568
PAMPULHA	310620005	BELO HORIZONT	3106200	310620005	31062000568
PAMPULHA	310620005	BELO HORIZONT	3106200	310620005	31062000568
PAMPULHA	310620005	BELO HORIZONT	3106200	310620005	31062000568
PAMPULHA	310620005	BELO HORIZONT	3106200	310620005	31062000568
PAMPULHA	310620005	BELO HORIZONT	3106200	310620005	31062000568
PAMPULHA	310620005	BELO HORIZONT	3106200	310620005	31062000568
PAMPULHA	310620005	BELO HORIZONT	3106200	310620005	31062000568
PAMPULHA	310620005	BELO HORIZONT	3106200	310620005	31062000568
PAMPULHA	310620005	BELO HORIZONT	3106200	310620005	31062000568
PAMPULHA	310620005	BELO HORIZONT	3106200	310620005	31062000568
PAMPULHA	310620005	BELO HORIZONT	3106200	310620005	31062000568
PAMPULHA	310620005	BELO HORIZONT	3106200	310620005	31062000568
PAMPULHA	310620005	BELO HORIZONT	3106200	310620005	31062000568
PAMPULHA	310620005	BELO HORIZONT	3106200	310620005	31062000568
PAMPULHA	310620005	BELO HORIZONT	3106200	310620005	31062000568
PAMPULHA	310620005	BELO HORIZONT	3106200	310620005	31062000568
PAMPULHA	310620005	BELO HORIZONT	3106200	310620005	31062000568
PAMPULHA	310620005	BELO HORIZONT	3106200	0	0
PAMPULHA	310620005	BELO HORIZONT	3106200	310620005	31062000568
PAMPULHA	310620005	BELO HORIZONT	3106200	310620005	31062000568
PAMPULHA	310620005	BELO HORIZONT	3106200	310620005	31062000568
PAMPULHA	310620005	BELO HORIZONT	3106200	310620005	31062000568
PAMPULHA	310620005	BELO HORIZONT	3106200	310620005	31062000568
PAMPULHA	310620005	BELO HORIZONT	3106200	310620005	31062000568
PAMPULHA	310620005	BELO HORIZONT	3106200	310620005	31062000568
PAMPULHA	310620005	BELO HORIZONT	3106200	310620005	31062000568
PAMPULHA	310620005	BELO HORIZONT	3106200	310620005	31062000568
PAMPULHA	310620005	BELO HORIZONT	3106200	310620005	31062000568
PAMPULHA	310620005	BELO HORIZONT	3106200	310620005	31062000568
PAMPULHA	310620005	BELO HORIZONT	3106200	310620005	31062000568
PAMPULHA	310620005	BELO HORIZONT	3106200	310620005	31062000568
PAMPULHA	310620005	BELO HORIZONT	3106200	310620005	31062000568
PAMPULHA	310620005	BELO HORIZONT	3106200	310620005	31062000568
PAMPULHA	310620005	BELO HORIZONT	3106200	310620005	31062000568
PAMPULHA	310620005	BELO HORIZONT	3106200	310620005	31062000568
PAMPULHA	310620005	BELO HORIZONT	3106200	310620005	31062000568
PAMPULHA	310620005	BELO HORIZONT	3106200	310620005	31062000568
PAMPULHA	310620005	BELO HORIZONT	3106200	310620005	31062000568
PAMPULHA	310620005	BELO HORIZONT	3106200	310620005	31062000568
PAMPULHA	310620005	BELO HORIZONT	3106200	310620005	31062000568

PAMPULHA	310620005	BELO HORIZONT	3106200	310620005	31062000568
PAMPULHA	310620005	BELO HORIZONT	3106200	310620005	31062000568
PAMPULHA	310620005	BELO HORIZONT	3106200	310620005	31062000568
PAMPULHA	310620005	BELO HORIZONT	3106200	310620005	31062000568
PAMPULHA	310620005	BELO HORIZONT	3106200	310620005	31062000568
PAMPULHA	310620005	BELO HORIZONT	3106200	310620005	31062000568
PAMPULHA	310620005	BELO HORIZONT	3106200	310620005	31062000568
PAMPULHA	310620005	BELO HORIZONT	3106200	310620005	31062000568
PAMPULHA	310620005	BELO HORIZONT	3106200	310620005	31062000568
PAMPULHA	310620005	BELO HORIZONT	3106200	310620005	31062000568
PAMPULHA	310620005	BELO HORIZONT	3106200	310620005	31062000568
PAMPULHA	310620005	BELO HORIZONT	3106200	310620005	31062000568
PAMPULHA	310620005	BELO HORIZONT	3106200	310620005	31062000568
PAMPULHA	310620005	BELO HORIZONT	3106200	310620005	31062000568
PAMPULHA	310620005	BELO HORIZONT	3106200	310620005	31062000568
PAMPULHA	310620005	BELO HORIZONT	3106200	310620005	31062000568
PAMPULHA	310620005	BELO HORIZONT	3106200	310620005	31062000568
PAMPULHA	310620005	BELO HORIZONT	3106200	310620005	31062000568
PAMPULHA	310620005	BELO HORIZONT	3106200	310620005	31062000568
PAMPULHA	310620005	BELO HORIZONT	3106200	310620005	31062000568
PAMPULHA	310620005	BELO HORIZONT	3106200	310620005	31062000568
PAMPULHA	310620005	BELO HORIZONT	3106200	310620005	31062000568
PAMPULHA	310620005	BELO HORIZONT	3106200	310620005	31062000568
PAMPULHA	310620005	BELO HORIZONT	3106200	310620005	31062000568
PAMPULHA	310620005	BELO HORIZONT	3106200	310620005	31062000568
PAMPULHA	310620005	BELO HORIZONT	3106200	310620005	31062000568
PAMPULHA	310620005	BELO HORIZONT	3106200	310620005	31062000568
PAMPULHA	310620005	BELO HORIZONT	3106200	310620005	31062000568
PAMPULHA	310620005	BELO HORIZONT	3106200	310620005	31062000568
PAMPULHA	310620005	BELO HORIZONT	3106200	310620005	31062000568
PAMPULHA	310620005	BELO HORIZONT	3106200	310620005	31062000568
PAMPULHA	310620005	BELO HORIZONT	3106200	310620005	31062000568
PAMPULHA	310620005	BELO HORIZONT	3106200	0	0
PAMPULHA	310620005	BELO HORIZONT	3106200	310620005	31062000568
PAMPULHA	310620005	BELO HORIZONT	3106200	310620005	31062000568
PAMPULHA	310620005	BELO HORIZONT	3106200	310620005	31062000568
PAMPULHA	310620005	BELO HORIZONT	3106200	310620005	31062000568
PAMPULHA	310620005	BELO HORIZONT	3106200	310620005	31062000568
PAMPULHA	310620005	BELO HORIZONT	3106200	310620005	31062000568
PAMPULHA	310620005	BELO HORIZONT	3106200	310620005	31062000568
PAMPULHA	310620005	BELO HORIZONT	3106200	310620005	31062000568
PAMPULHA	310620005	BELO HORIZONT	3106200	310620005	31062000568
PAMPULHA	310620005	BELO HORIZONT	3106200	310620005	31062000568
PAMPULHA	310620005	BELO HORIZONT	3106200	310620005	31062000568
PAMPULHA	310620005	BELO HORIZONT	3106200	310620005	31062000568
PAMPULHA	310620005	BELO HORIZONT	3106200	310620005	31062000568
PAMPULHA	310620005	BELO HORIZONT	3106200	310620005	31062000568
PAMPULHA	310620005	BELO HORIZONT	3106200	310620005	31062000568
PAMPULHA	310620005	BELO HORIZONT	3106200	310620005	31062000568
PAMPULHA	310620005	BELO HORIZONT	3106200	310620005	31062000568

PAMPULHA	310620005	BELO HORIZONT	3106200	310620005	31062000568
PAMPULHA	310620005	BELO HORIZONT	3106200	310620005	31062000568
PAMPULHA	310620005	BELO HORIZONT	3106200	310620005	31062000568
PAMPULHA	310620005	BELO HORIZONT	3106200	310620005	31062000568
PAMPULHA	310620005	BELO HORIZONT	3106200	310620005	31062000568
PAMPULHA	310620005	BELO HORIZONT	3106200	310620005	31062000568
PAMPULHA	310620005	BELO HORIZONT	3106200	310620005	31062000568
PAMPULHA	310620005	BELO HORIZONT	3106200	310620005	31062000568
PAMPULHA	310620005	BELO HORIZONT	3106200	310620005	31062000568
PAMPULHA	310620005	BELO HORIZONT	3106200	310620005	31062000568
PAMPULHA	310620005	BELO HORIZONT	3106200	310620005	31062000568
PAMPULHA	310620005	BELO HORIZONT	3106200	310620005	31062000568
PAMPULHA	310620005	BELO HORIZONT	3106200	310620005	31062000568
PAMPULHA	310620005	BELO HORIZONT	3106200	310620005	31062000568
PAMPULHA	310620005	BELO HORIZONT	3106200	310620005	31062000568
PAMPULHA	310620005	BELO HORIZONT	3106200	310620005	31062000568
PAMPULHA	310620005	BELO HORIZONT	3106200	310620005	31062000568
PAMPULHA	310620005	BELO HORIZONT	3106200	310620005	31062000568
PAMPULHA	310620005	BELO HORIZONT	3106200	310620005	31062000568
PAMPULHA	310620005	BELO HORIZONT	3106200	310620005	31062000568
PAMPULHA	310620005	BELO HORIZONT	3106200	310620005	31062000568
PAMPULHA	310620005	BELO HORIZONT	3106200	310620005	31062000568
PAMPULHA	310620005	BELO HORIZONT	3106200	310620005	31062000568
PAMPULHA	310620005	BELO HORIZONT	3106200	310620005	31062000568
PAMPULHA	310620005	BELO HORIZONT	3106200	310620005	31062000568
PAMPULHA	310620005	BELO HORIZONT	3106200	310620005	31062000568
PAMPULHA	310620005	BELO HORIZONT	3106200	310620005	31062000568
PAMPULHA	310620005	BELO HORIZONT	3106200	310620005	31062000568
PAMPULHA	310620005	BELO HORIZONT	3106200	310620005	31062000568
PAMPULHA	310620005	BELO HORIZONT	3106200	310620005	31062000568
PAMPULHA	310620005	BELO HORIZONT	3106200	310620005	31062000568
PAMPULHA	310620005	BELO HORIZONT	3106200	310620005	31062000568
PAMPULHA	310620005	BELO HORIZONT	3106200	310620005	31062000568
PAMPULHA	310620005	BELO HORIZONT	3106200	310620005	31062000568
PAMPULHA	310620005	BELO HORIZONT	3106200	310620005	31062000568
PAMPULHA	310620005	BELO HORIZONT	3106200	310620005	31062000568
PAMPULHA	310620005	BELO HORIZONT	3106200	310620005	31062000568
PAMPULHA	310620005	BELO HORIZONT	3106200	310620005	31062000568
PAMPULHA	310620005	BELO HORIZONT	3106200	310620005	31062000568
PAMPULHA	310620005	BELO HORIZONT	3106200	310620005	31062000568
PAMPULHA	310620005	BELO HORIZONT	3106200	310620005	31062000568
PAMPULHA	310620005	BELO HORIZONT	3106200	310620005	31062000568
PAMPULHA	310620005	BELO HORIZONT	3106200	310620005	31062000568
PAMPULHA	310620005	BELO HORIZONT	3106200	310620005	31062000568
PAMPULHA	310620005	BELO HORIZONT	3106200	310620005	31062000568
PAMPULHA	310620005	BELO HORIZONT	3106200	310620005	31062000568
PAMPULHA	310620005	BELO HORIZONT	3106200	310620005	31062000568
PAMPULHA	310620005	BELO HORIZONT	3106200	310620005	31062000568
PAMPULHA	310620005	BELO HORIZONT	3106200	310620005	31062000568
PAMPULHA	310620005	BELO HORIZONT	3106200	310620005	31062000568

PAMPULHA	310620005	BELO HORIZONT	3106200	310620005	31062000568
PAMPULHA	310620005	BELO HORIZONT	3106200	310620005	31062000568
PAMPULHA	310620005	BELO HORIZONT	3106200	310620005	31062000568
PAMPULHA	310620005	BELO HORIZONT	3106200	310620005	31062000568
PAMPULHA	310620005	BELO HORIZONT	3106200	310620005	31062000568
PAMPULHA	310620005	BELO HORIZONT	3106200	310620005	31062000568
PAMPULHA	310620005	BELO HORIZONT	3106200	310620005	31062000568
PAMPULHA	310620005	BELO HORIZONT	3106200	310620005	31062000568
PAMPULHA	310620005	BELO HORIZONT	3106200	310620005	31062000568
PAMPULHA	310620005	BELO HORIZONT	3106200	310620005	31062000568
PAMPULHA	310620005	BELO HORIZONT	3106200	310620005	31062000568
PAMPULHA	310620005	BELO HORIZONT	3106200	310620005	31062000568
PAMPULHA	310620005	BELO HORIZONT	3106200	310620005	31062000568
PAMPULHA	310620005	BELO HORIZONT	3106200	310620005	31062000568
PAMPULHA	310620005	BELO HORIZONT	3106200	310620005	31062000568
PAMPULHA	310620005	BELO HORIZONT	3106200	310620005	31062000568
PAMPULHA	310620005	BELO HORIZONT	3106200	310620005	31062000568
PAMPULHA	310620005	BELO HORIZONT	3106200	310620005	31062000568
PAMPULHA	310620005	BELO HORIZONT	3106200	310620005	31062000568
PAMPULHA	310620005	BELO HORIZONT	3106200	310620005	31062000568
PAMPULHA	310620005	BELO HORIZONT	3106200	310620005	31062000568
PAMPULHA	310620005	BELO HORIZONT	3106200	310620005	31062000568
PAMPULHA	310620005	BELO HORIZONT	3106200	310620005	31062000568
PAMPULHA	310620005	BELO HORIZONT	3106200	310620005	31062000568
PAMPULHA	310620005	BELO HORIZONT	3106200	310620005	31062000568
PAMPULHA	310620005	BELO HORIZONT	3106200	310620005	31062000568
PAMPULHA	310620005	BELO HORIZONT	3106200	310620005	31062000568
PAMPULHA	310620005	BELO HORIZONT	3106200	310620005	31062000568
PAMPULHA	310620005	BELO HORIZONT	3106200	310620005	31062000568
PAMPULHA	310620005	BELO HORIZONT	3106200	310620005	31062000568
PAMPULHA	310620005	BELO HORIZONT	3106200	310620005	31062000568
PAMPULHA	310620005	BELO HORIZONT	3106200	310620005	31062000568
PAMPULHA	310620005	BELO HORIZONT	3106200	310620005	31062000568
PAMPULHA	310620005	BELO HORIZONT	3106200	310620005	31062000568
PAMPULHA	310620005	BELO HORIZONT	3106200	310620005	31062000568
PAMPULHA	310620005	BELO HORIZONT	3106200	310620005	31062000568
PAMPULHA	310620005	BELO HORIZONT	3106200	310620005	31062000568
PAMPULHA	310620005	BELO HORIZONT	3106200	310620005	31062000568
PAMPULHA	310620005	BELO HORIZONT	3106200	310620005	31062000568
PAMPULHA	310620005	BELO HORIZONT	3106200	310620005	31062000568
PAMPULHA	310620005	BELO HORIZONT	3106200	310620005	31062000568
PAMPULHA	310620005	BELO HORIZONT	3106200	310620005	31062000568
PAMPULHA	310620005	BELO HORIZONT	3106200	310620005	31062000568
PAMPULHA	310620005	BELO HORIZONT	3106200	310620005	31062000568
PAMPULHA	310620005	BELO HORIZONT	3106200	310620005	31062000568
PAMPULHA	310620005	BELO HORIZONT	3106200	310620005	31062000568
PAMPULHA	310620005	BELO HORIZONT	3106200	310620005	31062000568
PAMPULHA	310620005	BELO HORIZONT	3106200	0	0
PAMPULHA	310620005	BELO HORIZONT	3106200	310620005	31062000568
PAMPULHA	310620005	BELO HORIZONT	3106200	310620005	31062000568

PAMPULHA	310620005	BELO HORIZONT	3106200	310620005	31062000568
PAMPULHA	310620005	BELO HORIZONT	3106200	310620005	31062000568
PAMPULHA	310620005	BELO HORIZONT	3106200	310620005	31062000568
PAMPULHA	310620005	BELO HORIZONT	3106200	310620005	31062000568
PAMPULHA	310620005	BELO HORIZONT	3106200	310620005	31062000568
PAMPULHA	310620005	BELO HORIZONT	3106200	310620005	31062000568
PAMPULHA	310620005	BELO HORIZONT	3106200	310620005	31062000568
PAMPULHA	310620005	BELO HORIZONT	3106200	310620005	31062000568
PAMPULHA	310620005	BELO HORIZONT	3106200	310620005	31062000568
PAMPULHA	310620005	BELO HORIZONT	3106200	310620005	31062000568
PAMPULHA	310620005	BELO HORIZONT	3106200	310620005	31062000568
PAMPULHA	310620005	BELO HORIZONT	3106200	310620005	31062000568
PAMPULHA	310620005	BELO HORIZONT	3106200	310620005	31062000568
PAMPULHA	310620005	BELO HORIZONT	3106200	310620005	31062000568
PAMPULHA	310620005	BELO HORIZONT	3106200	310620005	31062000568
PAMPULHA	310620005	BELO HORIZONT	3106200	310620005	31062000568
PAMPULHA	310620005	BELO HORIZONT	3106200	310620005	31062000568
PAMPULHA	310620005	BELO HORIZONT	3106200	310620005	31062000568
PAMPULHA	310620005	BELO HORIZONT	3106200	310620005	31062000568
PAMPULHA	310620005	BELO HORIZONT	3106200	310620005	31062000568
PAMPULHA	310620005	BELO HORIZONT	3106200	310620005	31062000568
PAMPULHA	310620005	BELO HORIZONT	3106200	310620005	31062000568
PAMPULHA	310620005	BELO HORIZONT	3106200	310620005	31062000568
PAMPULHA	310620005	BELO HORIZONT	3106200	310620005	31062000568
PAMPULHA	310620005	BELO HORIZONT	3106200	310620005	31062000568
PAMPULHA	310620005	BELO HORIZONT	3106200	310620005	31062000568
PAMPULHA	310620005	BELO HORIZONT	3106200	310620005	31062000568
PAMPULHA	310620005	BELO HORIZONT	3106200	310620005	31062000568
PAMPULHA	310620005	BELO HORIZONT	3106200	0	0
PAMPULHA	310620005	BELO HORIZONT	3106200	0	0
PAMPULHA	310620005	BELO HORIZONT	3106200	310620005	31062000568
PAMPULHA	310620005	BELO HORIZONT	3106200	0	0
VENDA NOVA	310620005	BELO HORIZONT	3106200	310620005	31062000569
VENDA NOVA	310620005	BELO HORIZONT	3106200	310620005	31062000569
VENDA NOVA	310620005	BELO HORIZONT	3106200	310620005	31062000569
VENDA NOVA	310620005	BELO HORIZONT	3106200	310620005	31062000569
BARREIRO	310620025	BARREIRO	3106200	310620025	31062002561
BARREIRO	310620025	BARREIRO	3106200	310620025	31062002561
BARREIRO	310620025	BARREIRO	3106200	310620025	31062002561
BARREIRO	310620025	BARREIRO	3106200	310620025	31062002561
BARREIRO	310620025	BARREIRO	3106200	310620025	31062002561
BARREIRO	310620025	BARREIRO	3106200	310620025	31062002561
BARREIRO	310620025	BARREIRO	3106200	310620025	31062002561
BARREIRO	310620025	BARREIRO	3106200	310620025	31062002561
BARREIRO	310620025	BARREIRO	3106200	310620025	31062002561
BARREIRO	310620025	BARREIRO	3106200	310620025	31062002561
BARREIRO	310620025	BARREIRO	3106200	310620025	31062002561
BARREIRO	310620025	BARREIRO	3106200	310620025	31062002561
BARREIRO	310620025	BARREIRO	3106200	310620025	31062002561
BARREIRO	310620025	BARREIRO	3106200	310620025	31062002561

BARREIRO	310620025	BARREIRO	3106200	310620025	31062002561
BARREIRO	310620025	BARREIRO	3106200	310620025	31062002561
BARREIRO	310620025	BARREIRO	3106200	310620025	31062002561
BARREIRO	310620025	BARREIRO	3106200	310620025	31062002561
BARREIRO	310620025	BARREIRO	3106200	310620025	31062002561
BARREIRO	310620025	BARREIRO	3106200	310620025	31062002561
BARREIRO	310620025	BARREIRO	3106200	310620025	31062002561
BARREIRO	310620025	BARREIRO	3106200	310620025	31062002561
BARREIRO	310620025	BARREIRO	3106200	310620025	31062002561
BARREIRO	310620025	BARREIRO	3106200	310620025	31062002561
BARREIRO	310620025	BARREIRO	3106200	310620025	31062002561
BARREIRO	310620025	BARREIRO	3106200	310620025	31062002561
BARREIRO	310620025	BARREIRO	3106200	310620025	31062002561
BARREIRO	310620025	BARREIRO	3106200	310620025	31062002561
BARREIRO	310620025	BARREIRO	3106200	310620025	31062002561
BARREIRO	310620025	BARREIRO	3106200	310620025	31062002561
BARREIRO	310620025	BARREIRO	3106200	310620025	31062002561
BARREIRO	310620025	BARREIRO	3106200	310620025	31062002561
BARREIRO	310620025	BARREIRO	3106200	310620025	31062002561
BARREIRO	310620025	BARREIRO	3106200	310620025	31062002561
BARREIRO	310620025	BARREIRO	3106200	310620025	31062002561
BARREIRO	310620025	BARREIRO	3106200	310620025	31062002561
BARREIRO	310620025	BARREIRO	3106200	310620025	31062002561
BARREIRO	310620025	BARREIRO	3106200	310620025	31062002561
BARREIRO	310620025	BARREIRO	3106200	310620025	31062002561
BARREIRO	310620025	BARREIRO	3106200	310620025	31062002561
BARREIRO	310620025	BARREIRO	3106200	310620025	31062002561
BARREIRO	310620025	BARREIRO	3106200	310620025	31062002561
BARREIRO	310620025	BARREIRO	3106200	310620025	31062002561
BARREIRO	310620025	BARREIRO	3106200	310620025	31062002561
BARREIRO	310620025	BARREIRO	3106200	310620025	31062002561
BARREIRO	310620025	BARREIRO	3106200	0	0
BARREIRO	310620025	BARREIRO	3106200	310620025	31062002561
BARREIRO	310620025	BARREIRO	3106200	310620025	31062002561
BARREIRO	310620025	BARREIRO	3106200	310620025	31062002561
BARREIRO	310620025	BARREIRO	3106200	310620025	31062002561
BARREIRO	310620025	BARREIRO	3106200	310620025	31062002561
BARREIRO	310620025	BARREIRO	3106200	310620025	31062002561
BARREIRO	310620025	BARREIRO	3106200	310620025	31062002561
BARREIRO	310620025	BARREIRO	3106200	310620025	31062002561
BARREIRO	310620025	BARREIRO	3106200	310620025	31062002561
BARREIRO	310620025	BARREIRO	3106200	310620025	31062002561
BARREIRO	310620025	BARREIRO	3106200	310620025	31062002561
BARREIRO	310620025	BARREIRO	3106200	310620025	31062002561
BARREIRO	310620025	BARREIRO	3106200	310620025	31062002561
BARREIRO	310620025	BARREIRO	3106200	310620025	31062002561
BARREIRO	310620025	BARREIRO	3106200	310620025	31062002561
BARREIRO	310620025	BARREIRO	3106200	310620025	31062002561
BARREIRO	310620025	BARREIRO	3106200	310620025	31062002561

BARREIRO	310620025	BARREIRO	3106200	310620025	31062002561
BARREIRO	310620025	BARREIRO	3106200	310620025	31062002561
BARREIRO	310620025	BARREIRO	3106200	310620025	31062002561
BARREIRO	310620025	BARREIRO	3106200	310620025	31062002561
BARREIRO	310620025	BARREIRO	3106200	310620025	31062002561
BARREIRO	310620025	BARREIRO	3106200	310620025	31062002561
BARREIRO	310620025	BARREIRO	3106200	310620025	31062002561
BARREIRO	310620025	BARREIRO	3106200	310620025	31062002561
BARREIRO	310620025	BARREIRO	3106200	310620025	31062002561
BARREIRO	310620025	BARREIRO	3106200	310620025	31062002561
BARREIRO	310620025	BARREIRO	3106200	310620025	31062002561
BARREIRO	310620025	BARREIRO	3106200	310620025	31062002561
BARREIRO	310620025	BARREIRO	3106200	310620025	31062002561
BARREIRO	310620025	BARREIRO	3106200	310620025	31062002561
BARREIRO	310620025	BARREIRO	3106200	310620025	31062002561
BARREIRO	310620025	BARREIRO	3106200	310620025	31062002561
BARREIRO	310620025	BARREIRO	3106200	310620025	31062002561
BARREIRO	310620025	BARREIRO	3106200	310620025	31062002561
BARREIRO	310620025	BARREIRO	3106200	310620025	31062002561
BARREIRO	310620025	BARREIRO	3106200	310620025	31062002561
BARREIRO	310620025	BARREIRO	3106200	310620025	31062002561
BARREIRO	310620025	BARREIRO	3106200	310620025	31062002561
BARREIRO	310620025	BARREIRO	3106200	310620025	31062002561
BARREIRO	310620025	BARREIRO	3106200	310620025	31062002561
BARREIRO	310620025	BARREIRO	3106200	310620025	31062002561
BARREIRO	310620025	BARREIRO	3106200	310620025	31062002561
BARREIRO	310620025	BARREIRO	3106200	310620025	31062002561
BARREIRO	310620025	BARREIRO	3106200	310620025	31062002561
BARREIRO	310620025	BARREIRO	3106200	310620025	31062002561
BARREIRO	310620025	BARREIRO	3106200	310620025	31062002561
BARREIRO	310620025	BARREIRO	3106200	310620025	31062002561
BARREIRO	310620025	BARREIRO	3106200	310620025	31062002561
BARREIRO	310620025	BARREIRO	3106200	310620025	31062002561
BARREIRO	310620025	BARREIRO	3106200	310620025	31062002561
BARREIRO	310620025	BARREIRO	3106200	310620025	31062002561
BARREIRO	310620025	BARREIRO	3106200	310620025	31062002561
BARREIRO	310620025	BARREIRO	3106200	310620025	31062002561
BARREIRO	310620025	BARREIRO	3106200	310620025	31062002561
BARREIRO	310620025	BARREIRO	3106200	310620025	31062002561
BARREIRO	310620025	BARREIRO	3106200	310620025	31062002561
BARREIRO	310620025	BARREIRO	3106200	310620025	31062002561
BARREIRO	310620025	BARREIRO	3106200	310620025	31062002561
BARREIRO	310620025	BARREIRO	3106200	310620025	31062002561
BARREIRO	310620025	BARREIRO	3106200	310620025	31062002561
BARREIRO	310620025	BARREIRO	3106200	310620025	31062002561
BARREIRO	310620025	BARREIRO	3106200	310620025	31062002561
BARREIRO	310620025	BARREIRO	3106200	310620025	31062002561
BARREIRO	310620025	BARREIRO	3106200	310620025	31062002561
BARREIRO	310620025	BARREIRO	3106200	310620025	31062002561
BARREIRO	310620025	BARREIRO	3106200	310620025	31062002561

BARREIRO	310620025	BARREIRO	3106200	310620025	31062002561
BARREIRO	310620025	BARREIRO	3106200	310620025	31062002561
BARREIRO	310620025	BARREIRO	3106200	310620025	31062002561
BARREIRO	310620025	BARREIRO	3106200	310620025	31062002561
BARREIRO	310620025	BARREIRO	3106200	310620025	31062002561
BARREIRO	310620025	BARREIRO	3106200	310620025	31062002561
BARREIRO	310620025	BARREIRO	3106200	310620025	31062002561
BARREIRO	310620025	BARREIRO	3106200	310620025	31062002561
BARREIRO	310620025	BARREIRO	3106200	310620025	31062002561
BARREIRO	310620025	BARREIRO	3106200	310620025	31062002561
BARREIRO	310620025	BARREIRO	3106200	310620025	31062002561
BARREIRO	310620025	BARREIRO	3106200	310620025	31062002561
BARREIRO	310620025	BARREIRO	3106200	310620025	31062002561
BARREIRO	310620025	BARREIRO	3106200	310620025	31062002561
BARREIRO	310620025	BARREIRO	3106200	310620025	31062002561
BARREIRO	310620025	BARREIRO	3106200	310620025	31062002561
BARREIRO	310620025	BARREIRO	3106200	310620025	31062002561
BARREIRO	310620025	BARREIRO	3106200	0	0
BARREIRO	310620025	BARREIRO	3106200	310620025	31062002561
BARREIRO	310620025	BARREIRO	3106200	310620025	31062002561
BARREIRO	310620025	BARREIRO	3106200	310620025	31062002561
BARREIRO	310620025	BARREIRO	3106200	310620025	31062002561
BARREIRO	310620025	BARREIRO	3106200	310620025	31062002561
BARREIRO	310620025	BARREIRO	3106200	310620025	31062002561
BARREIRO	310620025	BARREIRO	3106200	310620025	31062002561
BARREIRO	310620025	BARREIRO	3106200	310620025	31062002561
BARREIRO	310620025	BARREIRO	3106200	310620025	31062002561
BARREIRO	310620025	BARREIRO	3106200	310620025	31062002561
BARREIRO	310620025	BARREIRO	3106200	310620025	31062002561
BARREIRO	310620025	BARREIRO	3106200	310620025	31062002561
BARREIRO	310620025	BARREIRO	3106200	310620025	31062002561
BARREIRO	310620025	BARREIRO	3106200	310620025	31062002561
BARREIRO	310620025	BARREIRO	3106200	310620025	31062002561
BARREIRO	310620025	BARREIRO	3106200	310620025	31062002561
BARREIRO	310620025	BARREIRO	3106200	310620025	31062002561
BARREIRO	310620025	BARREIRO	3106200	310620025	31062002561
BARREIRO	310620025	BARREIRO	3106200	310620025	31062002561
BARREIRO	310620025	BARREIRO	3106200	310620025	31062002561
BARREIRO	310620025	BARREIRO	3106200	310620025	31062002561
BARREIRO	310620025	BARREIRO	3106200	310620025	31062002561
BARREIRO	310620025	BARREIRO	3106200	310620025	31062002561
BARREIRO	310620025	BARREIRO	3106200	310620025	31062002561
BARREIRO	310620025	BARREIRO	3106200	310620025	31062002561
BARREIRO	310620025	BARREIRO	3106200	310620025	31062002561
BARREIRO	310620025	BARREIRO	3106200	310620025	31062002561
BARREIRO	310620025	BARREIRO	3106200	310620025	31062002561
BARREIRO	310620025	BARREIRO	3106200	310620025	31062002561
BARREIRO	310620025	BARREIRO	3106200	310620025	31062002561
BARREIRO	310620025	BARREIRO	3106200	310620025	31062002561
BARREIRO	310620025	BARREIRO	3106200	310620025	31062002561

BARREIRO	310620025	BARREIRO	3106200	310620025	31062002561
BARREIRO	310620025	BARREIRO	3106200	310620025	31062002561
BARREIRO	310620025	BARREIRO	3106200	310620025	31062002561
BARREIRO	310620025	BARREIRO	3106200	0	0
BARREIRO	310620025	BARREIRO	3106200	310620025	31062002561
BARREIRO	310620025	BARREIRO	3106200	310620025	31062002561
BARREIRO	310620025	BARREIRO	3106200	310620025	31062002561
BARREIRO	310620025	BARREIRO	3106200	310620025	31062002561
BARREIRO	310620025	BARREIRO	3106200	310620025	31062002561
BARREIRO	310620025	BARREIRO	3106200	310620025	31062002561
BARREIRO	310620025	BARREIRO	3106200	310620025	31062002561
BARREIRO	310620025	BARREIRO	3106200	310620025	31062002561
BARREIRO	310620025	BARREIRO	3106200	310620025	31062002561
BARREIRO	310620025	BARREIRO	3106200	310620025	31062002561
BARREIRO	310620025	BARREIRO	3106200	310620025	31062002561
BARREIRO	310620025	BARREIRO	3106200	310620025	31062002561
BARREIRO	310620025	BARREIRO	3106200	310620025	31062002561
BARREIRO	310620025	BARREIRO	3106200	310620025	31062002561
BARREIRO	310620025	BARREIRO	3106200	310620025	31062002561
BARREIRO	310620025	BARREIRO	3106200	310620025	31062002561
BARREIRO	310620025	BARREIRO	3106200	310620025	31062002561
BARREIRO	310620025	BARREIRO	3106200	310620025	31062002561
BARREIRO	310620025	BARREIRO	3106200	310620025	31062002561
BARREIRO	310620025	BARREIRO	3106200	310620025	31062002561
BARREIRO	310620025	BARREIRO	3106200	310620025	31062002561
BARREIRO	310620025	BARREIRO	3106200	310620025	31062002561
BARREIRO	310620025	BARREIRO	3106200	310620025	31062002561
BARREIRO	310620025	BARREIRO	3106200	310620025	31062002561
BARREIRO	310620025	BARREIRO	3106200	310620025	31062002561
BARREIRO	310620025	BARREIRO	3106200	310620025	31062002561
BARREIRO	310620025	BARREIRO	3106200	310620025	31062002561
BARREIRO	310620025	BARREIRO	3106200	310620025	31062002561
BARREIRO	310620025	BARREIRO	3106200	310620025	31062002561
BARREIRO	310620025	BARREIRO	3106200	310620025	31062002561
BARREIRO	310620025	BARREIRO	3106200	310620025	31062002561
BARREIRO	310620025	BARREIRO	3106200	310620025	31062002561
BARREIRO	310620025	BARREIRO	3106200	310620025	31062002561
BARREIRO	310620025	BARREIRO	3106200	310620025	31062002561
BARREIRO	310620025	BARREIRO	3106200	310620025	31062002561
BARREIRO	310620025	BARREIRO	3106200	310620025	31062002561
BARREIRO	310620025	BARREIRO	3106200	310620025	31062002561
BARREIRO	310620025	BARREIRO	3106200	310620025	31062002561
BARREIRO	310620025	BARREIRO	3106200	310620025	31062002561
BARREIRO	310620025	BARREIRO	3106200	310620025	31062002561
BARREIRO	310620025	BARREIRO	3106200	310620025	31062002561
BARREIRO	310620025	BARREIRO	3106200	310620025	31062002561
BARREIRO	310620025	BARREIRO	3106200	310620025	31062002561
BARREIRO	310620025	BARREIRO	3106200	310620025	31062002561
BARREIRO	310620025	BARREIRO	3106200	310620025	31062002561
BARREIRO	310620025	BARREIRO	3106200	310620025	31062002561

BARREIRO	310620025	BARREIRO	3106200	310620025	31062002561
BARREIRO	310620025	BARREIRO	3106200	310620025	31062002561
BARREIRO	310620025	BARREIRO	3106200	310620025	31062002561
BARREIRO	310620025	BARREIRO	3106200	310620025	31062002561
BARREIRO	310620025	BARREIRO	3106200	310620025	31062002561
BARREIRO	310620025	BARREIRO	3106200	310620025	31062002561
BARREIRO	310620025	BARREIRO	3106200	310620025	31062002561
BARREIRO	310620025	BARREIRO	3106200	310620025	31062002561
BARREIRO	310620025	BARREIRO	3106200	310620025	31062002561
BARREIRO	310620025	BARREIRO	3106200	310620025	31062002561
BARREIRO	310620025	BARREIRO	3106200	310620025	31062002561
BARREIRO	310620025	BARREIRO	3106200	310620025	31062002561
BARREIRO	310620025	BARREIRO	3106200	310620025	31062002561
BARREIRO	310620025	BARREIRO	3106200	310620025	31062002561
BARREIRO	310620025	BARREIRO	3106200	310620025	31062002561
BARREIRO	310620025	BARREIRO	3106200	310620025	31062002561
BARREIRO	310620025	BARREIRO	3106200	310620025	31062002561
BARREIRO	310620025	BARREIRO	3106200	310620025	31062002561
BARREIRO	310620025	BARREIRO	3106200	310620025	31062002561
BARREIRO	310620025	BARREIRO	3106200	310620025	31062002561
BARREIRO	310620025	BARREIRO	3106200	310620025	31062002561
BARREIRO	310620025	BARREIRO	3106200	310620025	31062002561
BARREIRO	310620025	BARREIRO	3106200	310620025	31062002561
BARREIRO	310620025	BARREIRO	3106200	310620025	31062002561
BARREIRO	310620025	BARREIRO	3106200	310620025	31062002561
BARREIRO	310620025	BARREIRO	3106200	310620025	31062002561
BARREIRO	310620025	BARREIRO	3106200	310620025	31062002561
BARREIRO	310620025	BARREIRO	3106200	310620025	31062002561
BARREIRO	310620025	BARREIRO	3106200	310620025	31062002561
BARREIRO	310620025	BARREIRO	3106200	310620025	31062002561
BARREIRO	310620025	BARREIRO	3106200	310620025	31062002561
BARREIRO	310620025	BARREIRO	3106200	310620025	31062002561
BARREIRO	310620025	BARREIRO	3106200	310620025	31062002561
BARREIRO	310620025	BARREIRO	3106200	310620025	31062002561
BARREIRO	310620025	BARREIRO	3106200	310620025	31062002561
BARREIRO	310620025	BARREIRO	3106200	310620025	31062002561
BARREIRO	310620025	BARREIRO	3106200	310620025	31062002561
BARREIRO	310620025	BARREIRO	3106200	310620025	31062002561
BARREIRO	310620025	BARREIRO	3106200	310620025	31062002561
BARREIRO	310620025	BARREIRO	3106200	310620025	31062002561
BARREIRO	310620025	BARREIRO	3106200	310620025	31062002561
BARREIRO	310620025	BARREIRO	3106200	0	0
BARREIRO	310620025	BARREIRO	3106200	310620025	31062002561
BARREIRO	310620025	BARREIRO	3106200	310620025	31062002561
BARREIRO	310620025	BARREIRO	3106200	310620025	31062002561
BARREIRO	310620025	BARREIRO	3106200	310620025	31062002561
BARREIRO	310620025	BARREIRO	3106200	310620025	31062002561
BARREIRO	310620025	BARREIRO	3106200	310620025	31062002561
BARREIRO	310620025	BARREIRO	3106200	310620025	31062002561
BARREIRO	310620025	BARREIRO	3106200	310620025	31062002561

BARREIRO	310620025	BARREIRO	3106200	310620025	31062002561
BARREIRO	310620025	BARREIRO	3106200	310620025	31062002561
BARREIRO	310620025	BARREIRO	3106200	310620025	31062002561
BARREIRO	310620025	BARREIRO	3106200	310620025	31062002561
BARREIRO	310620025	BARREIRO	3106200	310620025	31062002561
BARREIRO	310620025	BARREIRO	3106200	310620025	31062002561
BARREIRO	310620025	BARREIRO	3106200	310620025	31062002561
BARREIRO	310620025	BARREIRO	3106200	310620025	31062002561
BARREIRO	310620025	BARREIRO	3106200	310620025	31062002561
BARREIRO	310620025	BARREIRO	3106200	310620025	31062002561
BARREIRO	310620025	BARREIRO	3106200	310620025	31062002561
BARREIRO	310620025	BARREIRO	3106200	310620025	31062002561
BARREIRO	310620025	BARREIRO	3106200	310620025	31062002561
BARREIRO	310620025	BARREIRO	3106200	310620025	31062002561
BARREIRO	310620025	BARREIRO	3106200	310620025	31062002561
BARREIRO	310620025	BARREIRO	3106200	310620025	31062002561
BARREIRO	310620025	BARREIRO	3106200	310620025	31062002561
BARREIRO	310620025	BARREIRO	3106200	310620025	31062002561
BARREIRO	310620025	BARREIRO	3106200	310620025	31062002561
BARREIRO	310620025	BARREIRO	3106200	310620025	31062002561
BARREIRO	310620025	BARREIRO	3106200	310620025	31062002561
BARREIRO	310620025	BARREIRO	3106200	310620025	31062002561
BARREIRO	310620025	BARREIRO	3106200	310620025	31062002561
BARREIRO	310620025	BARREIRO	3106200	310620025	31062002561
BARREIRO	310620025	BARREIRO	3106200	310620025	31062002561
BARREIRO	310620025	BARREIRO	3106200	310620025	31062002561
BARREIRO	310620025	BARREIRO	3106200	310620025	31062002561
BARREIRO	310620025	BARREIRO	3106200	310620025	31062002561
BARREIRO	310620025	BARREIRO	3106200	310620025	31062002561
BARREIRO	310620025	BARREIRO	3106200	310620025	31062002561
BARREIRO	310620025	BARREIRO	3106200	310620025	31062002561
BARREIRO	310620025	BARREIRO	3106200	310620025	31062002561
BARREIRO	310620025	BARREIRO	3106200	310620025	31062002561
BARREIRO	310620025	BARREIRO	3106200	310620025	31062002561
BARREIRO	310620025	BARREIRO	3106200	310620025	31062002561
BARREIRO	310620025	BARREIRO	3106200	310620025	31062002561
BARREIRO	310620025	BARREIRO	3106200	310620025	31062002561
BARREIRO	310620025	BARREIRO	3106200	310620025	31062002561
BARREIRO	310620025	BARREIRO	3106200	310620025	31062002561
BARREIRO	310620025	BARREIRO	3106200	310620025	31062002561
BARREIRO	310620025	BARREIRO	3106200	310620025	31062002561
BARREIRO	310620025	BARREIRO	3106200	310620025	31062002561
BARREIRO	310620025	BARREIRO	3106200	310620025	31062002561
BARREIRO	310620025	BARREIRO	3106200	310620025	31062002561
BARREIRO	310620025	BARREIRO	3106200	310620025	31062002561
BARREIRO	310620025	BARREIRO	3106200	0	0
BARREIRO	310620025	BARREIRO	3106200	310620025	31062002561
BARREIRO	310620025	BARREIRO	3106200	310620025	31062002561
BARREIRO	310620025	BARREIRO	3106200	310620025	31062002561
BARREIRO	310620025	BARREIRO	3106200	310620025	31062002561

BARREIRO	310620025	BARREIRO	3106200	310620025	31062002561
BARREIRO	310620025	BARREIRO	3106200	310620025	31062002561
BARREIRO	310620025	BARREIRO	3106200	310620025	31062002561
BARREIRO	310620025	BARREIRO	3106200	310620025	31062002561
BARREIRO	310620025	BARREIRO	3106200	310620025	31062002561
BARREIRO	310620025	BARREIRO	3106200	310620025	31062002561
BARREIRO	310620025	BARREIRO	3106200	310620025	31062002561
BARREIRO	310620025	BARREIRO	3106200	310620025	31062002561
BARREIRO	310620025	BARREIRO	3106200	310620025	31062002561
BARREIRO	310620025	BARREIRO	3106200	310620025	31062002561
BARREIRO	310620025	BARREIRO	3106200	310620025	31062002561
BARREIRO	310620025	BARREIRO	3106200	310620025	31062002561
BARREIRO	310620025	BARREIRO	3106200	310620025	31062002561
BARREIRO	310620025	BARREIRO	3106200	310620025	31062002561
BARREIRO	310620025	BARREIRO	3106200	310620025	31062002561
NORDESTE	310620060	VENDA NOVA	3106200	310620060	31062006064
NORDESTE	310620060	VENDA NOVA	3106200	310620060	31062006064
NORDESTE	310620060	VENDA NOVA	3106200	310620060	31062006064
NORDESTE	310620060	VENDA NOVA	3106200	310620060	31062006064
NORDESTE	310620060	VENDA NOVA	3106200	310620060	31062006064
NORDESTE	310620060	VENDA NOVA	3106200	310620060	31062006064
NORDESTE	310620060	VENDA NOVA	3106200	310620060	31062006064
NORDESTE	310620060	VENDA NOVA	3106200	310620060	31062006064
NORDESTE	310620060	VENDA NOVA	3106200	310620060	31062006064
BARREIRO	310620025	BARREIRO	3106200	310620025	31062002561
BARREIRO	310620025	BARREIRO	3106200	310620025	31062002561
BARREIRO	310620025	BARREIRO	3106200	310620025	31062002561
BARREIRO	310620025	BARREIRO	3106200	310620025	31062002561
BARREIRO	310620025	BARREIRO	3106200	310620025	31062002561
BARREIRO	310620025	BARREIRO	3106200	310620025	31062002561
BARREIRO	310620025	BARREIRO	3106200	310620025	31062002561
BARREIRO	310620025	BARREIRO	3106200	310620025	31062002561
BARREIRO	310620025	BARREIRO	3106200	310620025	31062002561
BARREIRO	310620025	BARREIRO	3106200	310620025	31062002561
BARREIRO	310620025	BARREIRO	3106200	310620025	31062002561
BARREIRO	310620025	BARREIRO	3106200	310620025	31062002561
BARREIRO	310620025	BARREIRO	3106200	310620025	31062002561
BARREIRO	310620025	BARREIRO	3106200	310620025	31062002561
BARREIRO	310620025	BARREIRO	3106200	310620025	31062002561
BARREIRO	310620025	BARREIRO	3106200	310620025	31062002561
BARREIRO	310620025	BARREIRO	3106200	310620025	31062002561
BARREIRO	310620025	BARREIRO	3106200	310620025	31062002561
BARREIRO	310620025	BARREIRO	3106200	310620025	31062002561
BARREIRO	310620025	BARREIRO	3106200	310620025	31062002561
BARREIRO	310620025	BARREIRO	3106200	310620025	31062002561
BARREIRO	310620025	BARREIRO	3106200	310620025	31062002561
BARREIRO	310620025	BARREIRO	3106200	310620025	31062002561
BARREIRO	310620025	BARREIRO	3106200	310620025	31062002561
BARREIRO	310620025	BARREIRO	3106200	310620025	31062002561
BARREIRO	310620025	BARREIRO	3106200	310620025	31062002561

BARREIRO	310620025	BARREIRO	3106200	310620025	31062002561
BARREIRO	310620025	BARREIRO	3106200	310620025	31062002561
BARREIRO	310620025	BARREIRO	3106200	310620025	31062002561
BARREIRO	310620025	BARREIRO	3106200	310620025	31062002561
BARREIRO	310620025	BARREIRO	3106200	310620025	31062002561
BARREIRO	310620025	BARREIRO	3106200	310620025	31062002561
BARREIRO	310620025	BARREIRO	3106200	310620025	31062002561
BARREIRO	310620025	BARREIRO	3106200	310620025	31062002561
BARREIRO	310620025	BARREIRO	3106200	310620025	31062002561
BARREIRO	310620025	BARREIRO	3106200	0	0
BARREIRO	310620025	BARREIRO	3106200	310620025	31062002561
BARREIRO	310620025	BARREIRO	3106200	310620025	31062002561
BARREIRO	310620025	BARREIRO	3106200	310620025	31062002561
BARREIRO	310620025	BARREIRO	3106200	310620025	31062002561
BARREIRO	310620025	BARREIRO	3106200	310620025	31062002561
BARREIRO	310620025	BARREIRO	3106200	310620025	31062002561
BARREIRO	310620025	BARREIRO	3106200	310620025	31062002561
BARREIRO	310620025	BARREIRO	3106200	310620025	31062002561
BARREIRO	310620025	BARREIRO	3106200	310620025	31062002561
BARREIRO	310620025	BARREIRO	3106200	310620025	31062002561
BARREIRO	310620025	BARREIRO	3106200	310620025	31062002561
BARREIRO	310620025	BARREIRO	3106200	310620025	31062002561
BARREIRO	310620025	BARREIRO	3106200	310620025	31062002561
BARREIRO	310620025	BARREIRO	3106200	310620025	31062002561
BARREIRO	310620025	BARREIRO	3106200	310620025	31062002561
BARREIRO	310620025	BARREIRO	3106200	310620025	31062002561
BARREIRO	310620025	BARREIRO	3106200	310620025	31062002561
BARREIRO	310620025	BARREIRO	3106200	310620025	31062002561
BARREIRO	310620025	BARREIRO	3106200	310620025	31062002561
BARREIRO	310620025	BARREIRO	3106200	310620025	31062002561
BARREIRO	310620025	BARREIRO	3106200	310620025	31062002561
BARREIRO	310620025	BARREIRO	3106200	310620025	31062002561
BARREIRO	310620025	BARREIRO	3106200	310620025	31062002561
BARREIRO	310620025	BARREIRO	3106200	310620025	31062002561
BARREIRO	310620025	BARREIRO	3106200	310620025	31062002561
BARREIRO	310620025	BARREIRO	3106200	310620025	31062002561
BARREIRO	310620025	BARREIRO	3106200	310620025	31062002561
BARREIRO	310620025	BARREIRO	3106200	0	0
BARREIRO	310620025	BARREIRO	3106200	310620025	31062002561
BARREIRO	310620025	BARREIRO	3106200	310620025	31062002561
BARREIRO	310620025	BARREIRO	3106200	310620025	31062002561
BARREIRO	310620025	BARREIRO	3106200	310620025	31062002561
BARREIRO	310620025	BARREIRO	3106200	310620025	31062002561
BARREIRO	310620025	BARREIRO	3106200	310620025	31062002561
BARREIRO	310620025	BARREIRO	3106200	310620025	31062002561
OESTE	310620025	BARREIRO	3106200	310620025	31062002567
OESTE	310620025	BARREIRO	3106200	310620025	31062002567
OESTE	310620025	BARREIRO	3106200	310620025	31062002567
OESTE	310620025	BARREIRO	3106200	310620025	31062002567
OESTE	310620025	BARREIRO	3106200	310620025	31062002567

OESTE	310620025	BARREIRO	3106200	310620025	31062002567
NORDESTE	310620060	VENDA NOVA	3106200	310620060	31062006064
NORDESTE	310620060	VENDA NOVA	3106200	310620060	31062006064
NORDESTE	310620060	VENDA NOVA	3106200	310620060	31062006064
NORDESTE	310620060	VENDA NOVA	3106200	310620060	31062006064
NORDESTE	310620060	VENDA NOVA	3106200	310620060	31062006064
NORDESTE	310620060	VENDA NOVA	3106200	310620060	31062006064
NORDESTE	310620060	VENDA NOVA	3106200	310620060	31062006064
NORDESTE	310620060	VENDA NOVA	3106200	310620060	31062006064
NORDESTE	310620060	VENDA NOVA	3106200	310620060	31062006064
NORDESTE	310620060	VENDA NOVA	3106200	310620060	31062006064
NORDESTE	310620060	VENDA NOVA	3106200	310620060	31062006064
NORDESTE	310620060	VENDA NOVA	3106200	310620060	31062006064
NORDESTE	310620060	VENDA NOVA	3106200	310620060	31062006064
NORDESTE	310620060	VENDA NOVA	3106200	310620060	31062006064
NORDESTE	310620060	VENDA NOVA	3106200	310620060	31062006064
NORDESTE	310620060	VENDA NOVA	3106200	310620060	31062006064
NORDESTE	310620060	VENDA NOVA	3106200	310620060	31062006064
NORDESTE	310620060	VENDA NOVA	3106200	310620060	31062006064
NORDESTE	310620060	VENDA NOVA	3106200	310620060	31062006064
NORDESTE	310620060	VENDA NOVA	3106200	310620060	31062006064
NORDESTE	310620060	VENDA NOVA	3106200	310620060	31062006064
NORDESTE	310620060	VENDA NOVA	3106200	310620060	31062006064
NORDESTE	310620060	VENDA NOVA	3106200	310620060	31062006064
NORDESTE	310620060	VENDA NOVA	3106200	310620060	31062006064
NORDESTE	310620060	VENDA NOVA	3106200	310620060	31062006064
NORDESTE	310620060	VENDA NOVA	3106200	310620060	31062006064
NORDESTE	310620060	VENDA NOVA	3106200	310620060	31062006064
NORDESTE	310620060	VENDA NOVA	3106200	310620060	31062006064
NORDESTE	310620060	VENDA NOVA	3106200	310620060	31062006064
NORDESTE	310620060	VENDA NOVA	3106200	310620060	31062006064
NORDESTE	310620060	VENDA NOVA	3106200	310620060	31062006064
NORDESTE	310620060	VENDA NOVA	3106200	310620060	31062006064
NORDESTE	310620060	VENDA NOVA	3106200	310620060	31062006064
NORDESTE	310620060	VENDA NOVA	3106200	310620060	31062006064
NORDESTE	310620060	VENDA NOVA	3106200	310620060	31062006064
NORDESTE	310620060	VENDA NOVA	3106200	310620060	31062006064
NORDESTE	310620060	VENDA NOVA	3106200	310620060	31062006064
NORDESTE	310620060	VENDA NOVA	3106200	310620060	31062006064
NORDESTE	310620060	VENDA NOVA	3106200	310620060	31062006064
NORDESTE	310620060	VENDA NOVA	3106200	310620060	31062006064
NORDESTE	310620060	VENDA NOVA	3106200	310620060	31062006064
NORDESTE	310620060	VENDA NOVA	3106200	310620060	31062006064
NORDESTE	310620060	VENDA NOVA	3106200	310620060	31062006064
NORDESTE	310620060	VENDA NOVA	3106200	310620060	31062006064
NORDESTE	310620060	VENDA NOVA	3106200	310620060	31062006064
NORDESTE	310620060	VENDA NOVA	3106200	310620060	31062006064
NORDESTE	310620060	VENDA NOVA	3106200	310620060	31062006064
NORDESTE	310620060	VENDA NOVA	3106200	310620060	31062006064
NORDESTE	310620060	VENDA NOVA	3106200	310620060	31062006064

NORDESTE	310620060	VENDA NOVA	3106200	310620060	31062006064
NORDESTE	310620060	VENDA NOVA	3106200	310620060	31062006064
NORDESTE	310620060	VENDA NOVA	3106200	310620060	31062006064
NORDESTE	310620060	VENDA NOVA	3106200	310620060	31062006064
NORDESTE	310620060	VENDA NOVA	3106200	310620060	31062006064
NORDESTE	310620060	VENDA NOVA	3106200	310620060	31062006064
NORDESTE	310620060	VENDA NOVA	3106200	310620060	31062006064
NORDESTE	310620060	VENDA NOVA	3106200	310620060	31062006064
NORDESTE	310620060	VENDA NOVA	3106200	310620060	31062006064
NORDESTE	310620060	VENDA NOVA	3106200	310620060	31062006064
NORDESTE	310620060	VENDA NOVA	3106200	310620060	31062006064
NORDESTE	310620060	VENDA NOVA	3106200	310620060	31062006064
NORDESTE	310620060	VENDA NOVA	3106200	310620060	31062006064
NORDESTE	310620060	VENDA NOVA	3106200	310620060	31062006064
NORDESTE	310620060	VENDA NOVA	3106200	310620060	31062006064
NORDESTE	310620060	VENDA NOVA	3106200	310620060	31062006064
NORDESTE	310620060	VENDA NOVA	3106200	310620060	31062006064
NORDESTE	310620060	VENDA NOVA	3106200	310620060	31062006064
NORDESTE	310620060	VENDA NOVA	3106200	310620060	31062006064
NORDESTE	310620060	VENDA NOVA	3106200	310620060	31062006064
NORDESTE	310620060	VENDA NOVA	3106200	310620060	31062006064
NORDESTE	310620060	VENDA NOVA	3106200	310620060	31062006064
NORDESTE	310620060	VENDA NOVA	3106200	310620060	31062006064
NORDESTE	310620060	VENDA NOVA	3106200	310620060	31062006064
NORDESTE	310620060	VENDA NOVA	3106200	310620060	31062006064
NORDESTE	310620060	VENDA NOVA	3106200	310620060	31062006064
NORDESTE	310620060	VENDA NOVA	3106200	310620060	31062006064
NORDESTE	310620060	VENDA NOVA	3106200	310620060	31062006064
NORDESTE	310620060	VENDA NOVA	3106200	310620060	31062006064
NORDESTE	310620060	VENDA NOVA	3106200	310620060	31062006064
NORDESTE	310620060	VENDA NOVA	3106200	310620060	31062006064
NORDESTE	310620060	VENDA NOVA	3106200	310620060	31062006064
NORDESTE	310620060	VENDA NOVA	3106200	310620060	31062006064
NORDESTE	310620060	VENDA NOVA	3106200	310620060	31062006064
NORDESTE	310620060	VENDA NOVA	3106200	310620060	31062006064
NORDESTE	310620060	VENDA NOVA	3106200	310620060	31062006064
NORDESTE	310620060	VENDA NOVA	3106200	310620060	31062006064
NORDESTE	310620060	VENDA NOVA	3106200	310620060	31062006064
NORDESTE	310620060	VENDA NOVA	3106200	310620060	31062006064
NORDESTE	310620060	VENDA NOVA	3106200	310620060	31062006064
NORDESTE	310620060	VENDA NOVA	3106200	310620060	31062006064
NORDESTE	310620060	VENDA NOVA	3106200	310620060	31062006064
NORDESTE	310620060	VENDA NOVA	3106200	310620060	31062006064
NORDESTE	310620060	VENDA NOVA	3106200	310620060	31062006064
NORDESTE	310620060	VENDA NOVA	3106200	310620060	31062006064
NORDESTE	310620060	VENDA NOVA	3106200	310620060	31062006064
NORDESTE	310620060	VENDA NOVA	3106200	310620060	31062006064
NORDESTE	310620060	VENDA NOVA	3106200	310620060	31062006064
NORDESTE	310620060	VENDA NOVA	3106200	310620060	31062006064
NORDESTE	310620060	VENDA NOVA	3106200	310620060	31062006064

NORDESTE	310620060	VENDA NOVA	3106200	310620060	31062006064
NORDESTE	310620060	VENDA NOVA	3106200	310620060	31062006064
NORDESTE	310620060	VENDA NOVA	3106200	310620060	31062006064
NORDESTE	310620060	VENDA NOVA	3106200	310620060	31062006064
NORDESTE	310620060	VENDA NOVA	3106200	310620060	31062006064
NORDESTE	310620060	VENDA NOVA	3106200	310620060	31062006064
NORDESTE	310620060	VENDA NOVA	3106200	310620060	31062006064
NORDESTE	310620060	VENDA NOVA	3106200	310620060	31062006064
NORDESTE	310620060	VENDA NOVA	3106200	310620060	31062006064
NORDESTE	310620060	VENDA NOVA	3106200	310620060	31062006064
NORDESTE	310620060	VENDA NOVA	3106200	310620060	31062006064
NORDESTE	310620060	VENDA NOVA	3106200	310620060	31062006064
NORDESTE	310620060	VENDA NOVA	3106200	310620060	31062006064
NORDESTE	310620060	VENDA NOVA	3106200	310620060	31062006064
NORDESTE	310620060	VENDA NOVA	3106200	310620060	31062006064
NORDESTE	310620060	VENDA NOVA	3106200	310620060	31062006064
NORDESTE	310620060	VENDA NOVA	3106200	310620060	31062006064
NORDESTE	310620060	VENDA NOVA	3106200	310620060	31062006064
NORDESTE	310620060	VENDA NOVA	3106200	310620060	31062006064
NORDESTE	310620060	VENDA NOVA	3106200	310620060	31062006064
NORDESTE	310620060	VENDA NOVA	3106200	310620060	31062006064
NORDESTE	310620060	VENDA NOVA	3106200	310620060	31062006064
NORDESTE	310620060	VENDA NOVA	3106200	310620060	31062006064
NORDESTE	310620060	VENDA NOVA	3106200	310620060	31062006064
NORDESTE	310620060	VENDA NOVA	3106200	310620060	31062006064
NORDESTE	310620060	VENDA NOVA	3106200	310620060	31062006064
NORDESTE	310620060	VENDA NOVA	3106200	310620060	31062006064
NORDESTE	310620060	VENDA NOVA	3106200	310620060	31062006064
NORDESTE	310620060	VENDA NOVA	3106200	310620060	31062006064
NORDESTE	310620060	VENDA NOVA	3106200	310620060	31062006064
NORDESTE	310620060	VENDA NOVA	3106200	310620060	31062006064
NORDESTE	310620060	VENDA NOVA	3106200	310620060	31062006064
NORDESTE	310620060	VENDA NOVA	3106200	310620060	31062006064
NORDESTE	310620060	VENDA NOVA	3106200	310620060	31062006064
NORDESTE	310620060	VENDA NOVA	3106200	310620060	31062006064
NORDESTE	310620060	VENDA NOVA	3106200	310620060	31062006064
NORDESTE	310620060	VENDA NOVA	3106200	310620060	31062006064
NORDESTE	310620060	VENDA NOVA	3106200	310620060	31062006064
NORDESTE	310620060	VENDA NOVA	3106200	310620060	31062006064
NORDESTE	310620060	VENDA NOVA	3106200	310620060	31062006064
NORDESTE	310620060	VENDA NOVA	3106200	310620060	31062006064
NORDESTE	310620060	VENDA NOVA	3106200	310620060	31062006064
NORDESTE	310620060	VENDA NOVA	3106200	310620060	31062006064
NORDESTE	310620060	VENDA NOVA	3106200	310620060	31062006064
NORDESTE	310620060	VENDA NOVA	3106200	310620060	31062006064
NORDESTE	310620060	VENDA NOVA	3106200	310620060	31062006064
NORDESTE	310620060	VENDA NOVA	3106200	310620060	31062006064
NORDESTE	310620060	VENDA NOVA	3106200	310620060	31062006064
NORDESTE	310620060	VENDA NOVA	3106200	310620060	31062006064
NORDESTE	310620060	VENDA NOVA	3106200	310620060	31062006064

NORDESTE	310620060	VENDA NOVA	3106200	310620060	31062006064
NORDESTE	310620060	VENDA NOVA	3106200	310620060	31062006064
NORDESTE	310620060	VENDA NOVA	3106200	310620060	31062006064
NORDESTE	310620060	VENDA NOVA	3106200	310620060	31062006064
NORDESTE	310620060	VENDA NOVA	3106200	310620060	31062006064
NORDESTE	310620060	VENDA NOVA	3106200	0	0
NORDESTE	310620060	VENDA NOVA	3106200	310620060	31062006064
NORDESTE	310620060	VENDA NOVA	3106200	310620060	31062006064
NORDESTE	310620060	VENDA NOVA	3106200	310620060	31062006064
NORDESTE	310620060	VENDA NOVA	3106200	310620060	31062006064
NORDESTE	310620060	VENDA NOVA	3106200	310620060	31062006064
NORDESTE	310620060	VENDA NOVA	3106200	310620060	31062006064
NORDESTE	310620060	VENDA NOVA	3106200	310620060	31062006064
NORDESTE	310620060	VENDA NOVA	3106200	310620060	31062006064
NORDESTE	310620060	VENDA NOVA	3106200	310620060	31062006064
NORDESTE	310620060	VENDA NOVA	3106200	310620060	31062006064
NORDESTE	310620060	VENDA NOVA	3106200	310620060	31062006064
NORDESTE	310620060	VENDA NOVA	3106200	310620060	31062006064
NORDESTE	310620060	VENDA NOVA	3106200	310620060	31062006064
NORDESTE	310620060	VENDA NOVA	3106200	310620060	31062006064
NORDESTE	310620060	VENDA NOVA	3106200	310620060	31062006064
NORDESTE	310620060	VENDA NOVA	3106200	310620060	31062006064
NORDESTE	310620060	VENDA NOVA	3106200	310620060	31062006064
NORDESTE	310620060	VENDA NOVA	3106200	310620060	31062006064
NORDESTE	310620060	VENDA NOVA	3106200	310620060	31062006064
NORDESTE	310620060	VENDA NOVA	3106200	310620060	31062006064
NORDESTE	310620060	VENDA NOVA	3106200	310620060	31062006064
NORDESTE	310620060	VENDA NOVA	3106200	310620060	31062006064
NORDESTE	310620060	VENDA NOVA	3106200	310620060	31062006064
NORDESTE	310620060	VENDA NOVA	3106200	310620060	31062006064
NORDESTE	310620060	VENDA NOVA	3106200	310620060	31062006064
NORDESTE	310620060	VENDA NOVA	3106200	310620060	31062006064
NORDESTE	310620060	VENDA NOVA	3106200	310620060	31062006064
NORDESTE	310620060	VENDA NOVA	3106200	310620060	31062006064
NORDESTE	310620060	VENDA NOVA	3106200	310620060	31062006064
NORDESTE	310620060	VENDA NOVA	3106200	310620060	31062006064
NORDESTE	310620060	VENDA NOVA	3106200	310620060	31062006064
NORDESTE	310620060	VENDA NOVA	3106200	310620060	31062006064
NORDESTE	310620060	VENDA NOVA	3106200	310620060	31062006064
NORDESTE	310620060	VENDA NOVA	3106200	310620060	31062006064
NORDESTE	310620060	VENDA NOVA	3106200	310620060	31062006064
NORDESTE	310620060	VENDA NOVA	3106200	310620060	31062006064
NORDESTE	310620060	VENDA NOVA	3106200	310620060	31062006064
NORDESTE	310620060	VENDA NOVA	3106200	310620060	31062006064
NORDESTE	310620060	VENDA NOVA	3106200	310620060	31062006064
NORDESTE	310620060	VENDA NOVA	3106200	310620060	31062006064
NORDESTE	310620060	VENDA NOVA	3106200	310620060	31062006064
NORDESTE	310620060	VENDA NOVA	3106200	310620060	31062006064
NORTE	310620060	VENDA NOVA	3106200	310620060	31062006066
NORTE	310620060	VENDA NOVA	3106200	310620060	31062006066

NORTE	310620060	VENDA NOVA	3106200	310620060	31062006066
NORTE	310620060	VENDA NOVA	3106200	310620060	31062006066
NORTE	310620060	VENDA NOVA	3106200	310620060	31062006066
NORTE	310620060	VENDA NOVA	3106200	310620060	31062006066
NORTE	310620060	VENDA NOVA	3106200	310620060	31062006066
NORTE	310620060	VENDA NOVA	3106200	310620060	31062006066
NORTE	310620060	VENDA NOVA	3106200	310620060	31062006066
NORTE	310620060	VENDA NOVA	3106200	310620060	31062006066
NORTE	310620060	VENDA NOVA	3106200	310620060	31062006066
NORTE	310620060	VENDA NOVA	3106200	310620060	31062006066
NORTE	310620060	VENDA NOVA	3106200	310620060	31062006066
NORTE	310620060	VENDA NOVA	3106200	310620060	31062006066
NORTE	310620060	VENDA NOVA	3106200	310620060	31062006066
NORTE	310620060	VENDA NOVA	3106200	310620060	31062006066
NORTE	310620060	VENDA NOVA	3106200	310620060	31062006066
NORTE	310620060	VENDA NOVA	3106200	310620060	31062006066
NORTE	310620060	VENDA NOVA	3106200	310620060	31062006066
NORTE	310620060	VENDA NOVA	3106200	310620060	31062006066
NORTE	310620060	VENDA NOVA	3106200	310620060	31062006066
NORTE	310620060	VENDA NOVA	3106200	310620060	31062006066
NORTE	310620060	VENDA NOVA	3106200	310620060	31062006066
NORTE	310620060	VENDA NOVA	3106200	310620060	31062006066
NORTE	310620060	VENDA NOVA	3106200	310620060	31062006066
NORTE	310620060	VENDA NOVA	3106200	310620060	31062006066
NORTE	310620060	VENDA NOVA	3106200	310620060	31062006066
NORTE	310620060	VENDA NOVA	3106200	310620060	31062006066
NORTE	310620060	VENDA NOVA	3106200	310620060	31062006066
NORTE	310620060	VENDA NOVA	3106200	310620060	31062006066
NORTE	310620060	VENDA NOVA	3106200	310620060	31062006066
NORTE	310620060	VENDA NOVA	3106200	310620060	31062006066
NORTE	310620060	VENDA NOVA	3106200	310620060	31062006066
NORTE	310620060	VENDA NOVA	3106200	310620060	31062006066
NORTE	310620060	VENDA NOVA	3106200	310620060	31062006066
NORTE	310620060	VENDA NOVA	3106200	310620060	31062006066
NORTE	310620060	VENDA NOVA	3106200	310620060	31062006066
NORTE	310620060	VENDA NOVA	3106200	310620060	31062006066
NORTE	310620060	VENDA NOVA	3106200	310620060	31062006066
NORTE	310620060	VENDA NOVA	3106200	310620060	31062006066
NORTE	310620060	VENDA NOVA	3106200	310620060	31062006066
NORTE	310620060	VENDA NOVA	3106200	310620060	31062006066
NORTE	310620060	VENDA NOVA	3106200	310620060	31062006066
NORTE	310620060	VENDA NOVA	3106200	310620060	31062006066
NORTE	310620060	VENDA NOVA	3106200	310620060	31062006066
NORTE	310620060	VENDA NOVA	3106200	310620060	31062006066
NORTE	310620060	VENDA NOVA	3106200	310620060	31062006066
NORTE	310620060	VENDA NOVA	3106200	310620060	31062006066
NORTE	310620060	VENDA NOVA	3106200	310620060	31062006066
NORTE	310620060	VENDA NOVA	3106200	310620060	31062006066
NORTE	310620060	VENDA NOVA	3106200	310620060	31062006066
NORTE	310620060	VENDA NOVA	3106200	310620060	31062006066

NORTE	310620060	VENDA NOVA	3106200	310620060	31062006066
NORTE	310620060	VENDA NOVA	3106200	310620060	31062006066
NORTE	310620060	VENDA NOVA	3106200	310620060	31062006066
NORTE	310620060	VENDA NOVA	3106200	310620060	31062006066
NORTE	310620060	VENDA NOVA	3106200	310620060	31062006066
NORTE	310620060	VENDA NOVA	3106200	310620060	31062006066
NORTE	310620060	VENDA NOVA	3106200	310620060	31062006066
NORTE	310620060	VENDA NOVA	3106200	310620060	31062006066
NORTE	310620060	VENDA NOVA	3106200	310620060	31062006066
NORTE	310620060	VENDA NOVA	3106200	310620060	31062006066
NORTE	310620060	VENDA NOVA	3106200	310620060	31062006066
NORTE	310620060	VENDA NOVA	3106200	310620060	31062006066
NORTE	310620060	VENDA NOVA	3106200	310620060	31062006066
NORTE	310620060	VENDA NOVA	3106200	310620060	31062006066
VENDA NOVA	310620060	VENDA NOVA	3106200	310620060	31062006069
VENDA NOVA	310620060	VENDA NOVA	3106200	310620060	31062006069
VENDA NOVA	310620060	VENDA NOVA	3106200	310620060	31062006069
VENDA NOVA	310620060	VENDA NOVA	3106200	310620060	31062006069
VENDA NOVA	310620060	VENDA NOVA	3106200	310620060	31062006069
VENDA NOVA	310620060	VENDA NOVA	3106200	310620060	31062006069
VENDA NOVA	310620060	VENDA NOVA	3106200	310620060	31062006069
VENDA NOVA	310620060	VENDA NOVA	3106200	310620060	31062006069
VENDA NOVA	310620060	VENDA NOVA	3106200	310620060	31062006069
VENDA NOVA	310620060	VENDA NOVA	3106200	310620060	31062006069
VENDA NOVA	310620060	VENDA NOVA	3106200	310620060	31062006069
VENDA NOVA	310620060	VENDA NOVA	3106200	310620060	31062006069
VENDA NOVA	310620060	VENDA NOVA	3106200	310620060	31062006069
VENDA NOVA	310620060	VENDA NOVA	3106200	310620060	31062006069
VENDA NOVA	310620060	VENDA NOVA	3106200	310620060	31062006069
VENDA NOVA	310620060	VENDA NOVA	3106200	310620060	31062006069
VENDA NOVA	310620060	VENDA NOVA	3106200	310620060	31062006069
VENDA NOVA	310620060	VENDA NOVA	3106200	310620060	31062006069
VENDA NOVA	310620060	VENDA NOVA	3106200	310620060	31062006069
VENDA NOVA	310620060	VENDA NOVA	3106200	310620060	31062006069
VENDA NOVA	310620060	VENDA NOVA	3106200	310620060	31062006069
VENDA NOVA	310620060	VENDA NOVA	3106200	310620060	31062006069
NORTE	310620060	VENDA NOVA	3106200	310620060	31062006066
NORTE	310620060	VENDA NOVA	3106200	310620060	31062006066
NORTE	310620060	VENDA NOVA	3106200	310620060	31062006066
NORTE	310620060	VENDA NOVA	3106200	310620060	31062006066
NORTE	310620060	VENDA NOVA	3106200	310620060	31062006066
NORTE	310620060	VENDA NOVA	3106200	310620060	31062006066
NORTE	310620060	VENDA NOVA	3106200	310620060	31062006066
NORTE	310620060	VENDA NOVA	3106200	310620060	31062006066
NORTE	310620060	VENDA NOVA	3106200	310620060	31062006066
NORTE	310620060	VENDA NOVA	3106200	310620060	31062006066
NORTE	310620060	VENDA NOVA	3106200	310620060	31062006066
NORTE	310620060	VENDA NOVA	3106200	310620060	31062006066
NORTE	310620060	VENDA NOVA	3106200	310620060	31062006066
NORTE	310620060	VENDA NOVA	3106200	310620060	31062006066

NORTE	310620060	VENDA NOVA	3106200	310620060	31062006066
NORTE	310620060	VENDA NOVA	3106200	310620060	31062006066
NORTE	310620060	VENDA NOVA	3106200	310620060	31062006066
NORTE	310620060	VENDA NOVA	3106200	310620060	31062006066
NORTE	310620060	VENDA NOVA	3106200	310620060	31062006066
NORTE	310620060	VENDA NOVA	3106200	310620060	31062006066
NORTE	310620060	VENDA NOVA	3106200	310620060	31062006066
NORTE	310620060	VENDA NOVA	3106200	310620060	31062006066
NORTE	310620060	VENDA NOVA	3106200	0	0
NORTE	310620060	VENDA NOVA	3106200	310620060	31062006066
NORTE	310620060	VENDA NOVA	3106200	310620060	31062006066
NORTE	310620060	VENDA NOVA	3106200	310620060	31062006066
NORTE	310620060	VENDA NOVA	3106200	310620060	31062006066
NORTE	310620060	VENDA NOVA	3106200	310620060	31062006066
NORTE	310620060	VENDA NOVA	3106200	310620060	31062006066
NORTE	310620060	VENDA NOVA	3106200	310620060	31062006066
NORTE	310620060	VENDA NOVA	3106200	310620060	31062006066
NORTE	310620060	VENDA NOVA	3106200	310620060	31062006066
NORTE	310620060	VENDA NOVA	3106200	310620060	31062006066
NORTE	310620060	VENDA NOVA	3106200	310620060	31062006066
NORTE	310620060	VENDA NOVA	3106200	310620060	31062006066
NORTE	310620060	VENDA NOVA	3106200	310620060	31062006066
NORTE	310620060	VENDA NOVA	3106200	310620060	31062006066
NORTE	310620060	VENDA NOVA	3106200	310620060	31062006066
NORTE	310620060	VENDA NOVA	3106200	310620060	31062006066
NORTE	310620060	VENDA NOVA	3106200	310620060	31062006066
NORTE	310620060	VENDA NOVA	3106200	310620060	31062006066
NORTE	310620060	VENDA NOVA	3106200	310620060	31062006066
NORTE	310620060	VENDA NOVA	3106200	310620060	31062006066
NORTE	310620060	VENDA NOVA	3106200	310620060	31062006066
NORTE	310620060	VENDA NOVA	3106200	310620060	31062006066
NORTE	310620060	VENDA NOVA	3106200	310620060	31062006066
NORTE	310620060	VENDA NOVA	3106200	310620060	31062006066
NORTE	310620060	VENDA NOVA	3106200	310620060	31062006066
NORTE	310620060	VENDA NOVA	3106200	310620060	31062006066
NORTE	310620060	VENDA NOVA	3106200	310620060	31062006066
NORTE	310620060	VENDA NOVA	3106200	310620060	31062006066
NORTE	310620060	VENDA NOVA	3106200	310620060	31062006066
NORTE	310620060	VENDA NOVA	3106200	310620060	31062006066
NORTE	310620060	VENDA NOVA	3106200	310620060	31062006066
NORTE	310620060	VENDA NOVA	3106200	310620060	31062006066
NORTE	310620060	VENDA NOVA	3106200	310620060	31062006066
NORTE	310620060	VENDA NOVA	3106200	310620060	31062006066
NORTE	310620060	VENDA NOVA	3106200	310620060	31062006066
NORTE	310620060	VENDA NOVA	3106200	310620060	31062006066
NORTE	310620060	VENDA NOVA	3106200	310620060	31062006066
NORTE	310620060	VENDA NOVA	3106200	310620060	31062006066
NORTE	310620060	VENDA NOVA	3106200	310620060	31062006066
NORTE	310620060	VENDA NOVA	3106200	310620060	31062006066
NORTE	310620060	VENDA NOVA	3106200	310620060	31062006066

NORTE	310620060	VENDA NOVA	3106200	310620060	31062006066
NORTE	310620060	VENDA NOVA	3106200	310620060	31062006066
NORTE	310620060	VENDA NOVA	3106200	310620060	31062006066
NORTE	310620060	VENDA NOVA	3106200	310620060	31062006066
NORTE	310620060	VENDA NOVA	3106200	310620060	31062006066
NORTE	310620060	VENDA NOVA	3106200	310620060	31062006066
NORTE	310620060	VENDA NOVA	3106200	310620060	31062006066
NORTE	310620060	VENDA NOVA	3106200	310620060	31062006066
NORTE	310620060	VENDA NOVA	3106200	310620060	31062006066
NORTE	310620060	VENDA NOVA	3106200	310620060	31062006066
NORTE	310620060	VENDA NOVA	3106200	310620060	31062006066
NORTE	310620060	VENDA NOVA	3106200	310620060	31062006066
NORTE	310620060	VENDA NOVA	3106200	310620060	31062006066
NORTE	310620060	VENDA NOVA	3106200	310620060	31062006066
NORTE	310620060	VENDA NOVA	3106200	310620060	31062006066
NORTE	310620060	VENDA NOVA	3106200	310620060	31062006066
NORTE	310620060	VENDA NOVA	3106200	310620060	31062006066
NORTE	310620060	VENDA NOVA	3106200	310620060	31062006066
NORTE	310620060	VENDA NOVA	3106200	310620060	31062006066
NORTE	310620060	VENDA NOVA	3106200	310620060	31062006066
NORTE	310620060	VENDA NOVA	3106200	310620060	31062006066
NORTE	310620060	VENDA NOVA	3106200	310620060	31062006066
NORTE	310620060	VENDA NOVA	3106200	310620060	31062006066
NORTE	310620060	VENDA NOVA	3106200	310620060	31062006066
NORTE	310620060	VENDA NOVA	3106200	310620060	31062006066
NORTE	310620060	VENDA NOVA	3106200	310620060	31062006066
NORTE	310620060	VENDA NOVA	3106200	310620060	31062006066
NORTE	310620060	VENDA NOVA	3106200	310620060	31062006066
NORTE	310620060	VENDA NOVA	3106200	310620060	31062006066
NORTE	310620060	VENDA NOVA	3106200	310620060	31062006066
NORTE	310620060	VENDA NOVA	3106200	310620060	31062006066
NORTE	310620060	VENDA NOVA	3106200	310620060	31062006066
NORTE	310620060	VENDA NOVA	3106200	310620060	31062006066
NORTE	310620060	VENDA NOVA	3106200	310620060	31062006066
NORTE	310620060	VENDA NOVA	3106200	310620060	31062006066
NORTE	310620060	VENDA NOVA	3106200	310620060	31062006066
NORTE	310620060	VENDA NOVA	3106200	310620060	31062006066
NORTE	310620060	VENDA NOVA	3106200	310620060	31062006066
NORTE	310620060	VENDA NOVA	3106200	310620060	31062006066
NORTE	310620060	VENDA NOVA	3106200	310620060	31062006066
NORTE	310620060	VENDA NOVA	3106200	310620060	31062006066
NORTE	310620060	VENDA NOVA	3106200	310620060	31062006066
NORTE	310620060	VENDA NOVA	3106200	310620060	31062006066
NORTE	310620060	VENDA NOVA	3106200	310620060	31062006066
NORTE	310620060	VENDA NOVA	3106200	310620060	31062006066
NORTE	310620060	VENDA NOVA	3106200	310620060	31062006066
NORTE	310620060	VENDA NOVA	3106200	310620060	31062006066
NORTE	310620060	VENDA NOVA	3106200	310620060	31062006066
NORTE	310620060	VENDA NOVA	3106200	310620060	31062006066
NORTE	310620060	VENDA NOVA	3106200	310620060	31062006066

NORTE	310620060	VENDA NOVA	3106200	310620060	31062006066
NORTE	310620060	VENDA NOVA	3106200	310620060	31062006066
NORTE	310620060	VENDA NOVA	3106200	310620060	31062006066
NORTE	310620060	VENDA NOVA	3106200	310620060	31062006066
NORTE	310620060	VENDA NOVA	3106200	310620060	31062006066
NORTE	310620060	VENDA NOVA	3106200	310620060	31062006066
NORTE	310620060	VENDA NOVA	3106200	310620060	31062006066
NORTE	310620060	VENDA NOVA	3106200	310620060	31062006066
NORTE	310620060	VENDA NOVA	3106200	310620060	31062006066
NORTE	310620060	VENDA NOVA	3106200	310620060	31062006066
NORTE	310620060	VENDA NOVA	3106200	310620060	31062006066
NORTE	310620060	VENDA NOVA	3106200	310620060	31062006066
NORTE	310620060	VENDA NOVA	3106200	310620060	31062006066
NORTE	310620060	VENDA NOVA	3106200	310620060	31062006066
NORTE	310620060	VENDA NOVA	3106200	310620060	31062006066
NORTE	310620060	VENDA NOVA	3106200	310620060	31062006066
NORTE	310620060	VENDA NOVA	3106200	310620060	31062006066
NORTE	310620060	VENDA NOVA	3106200	310620060	31062006066
NORTE	310620060	VENDA NOVA	3106200	310620060	31062006066
NORTE	310620060	VENDA NOVA	3106200	310620060	31062006066
NORTE	310620060	VENDA NOVA	3106200	310620060	31062006066
NORTE	310620060	VENDA NOVA	3106200	310620060	31062006066
NORTE	310620060	VENDA NOVA	3106200	310620060	31062006066
NORTE	310620060	VENDA NOVA	3106200	310620060	31062006066
NORTE	310620060	VENDA NOVA	3106200	310620060	31062006066
NORTE	310620060	VENDA NOVA	3106200	310620060	31062006066
NORTE	310620060	VENDA NOVA	3106200	310620060	31062006066
NORTE	310620060	VENDA NOVA	3106200	310620060	31062006066
NORTE	310620060	VENDA NOVA	3106200	310620060	31062006066
NORTE	310620060	VENDA NOVA	3106200	310620060	31062006066
NORTE	310620060	VENDA NOVA	3106200	310620060	31062006066
NORTE	310620060	VENDA NOVA	3106200	310620060	31062006066
NORTE	310620060	VENDA NOVA	3106200	310620060	31062006066
NORTE	310620060	VENDA NOVA	3106200	310620060	31062006066
NORTE	310620060	VENDA NOVA	3106200	310620060	31062006066
NORTE	310620060	VENDA NOVA	3106200	310620060	31062006066
NORTE	310620060	VENDA NOVA	3106200	310620060	31062006066
NORTE	310620060	VENDA NOVA	3106200	310620060	31062006066
NORTE	310620060	VENDA NOVA	3106200	310620060	31062006066
NORTE	310620060	VENDA NOVA	3106200	310620060	31062006066
NORTE	310620060	VENDA NOVA	3106200	310620060	31062006066
NORTE	310620060	VENDA NOVA	3106200	310620060	31062006066
NORTE	310620060	VENDA NOVA	3106200	310620060	31062006066
NORTE	310620060	VENDA NOVA	3106200	310620060	31062006066
NORTE	310620060	VENDA NOVA	3106200	310620060	31062006066
NORTE	310620060	VENDA NOVA	3106200	310620060	31062006066
NORTE	310620060	VENDA NOVA	3106200	310620060	31062006066
NORTE	310620060	VENDA NOVA	3106200	310620060	31062006066
NORTE	310620060	VENDA NOVA	3106200	310620060	31062006066
NORTE	310620060	VENDA NOVA	3106200	310620060	31062006066

VENDA NOVA	310620060	VENDA NOVA	3106200	310620060	31062006069
VENDA NOVA	310620060	VENDA NOVA	3106200	310620060	31062006069
VENDA NOVA	310620060	VENDA NOVA	3106200	310620060	31062006069
VENDA NOVA	310620060	VENDA NOVA	3106200	310620060	31062006069
VENDA NOVA	310620060	VENDA NOVA	3106200	310620060	31062006069
VENDA NOVA	310620060	VENDA NOVA	3106200	310620060	31062006069
VENDA NOVA	310620060	VENDA NOVA	3106200	310620060	31062006069
VENDA NOVA	310620060	VENDA NOVA	3106200	310620060	31062006069
NORTE	310620060	VENDA NOVA	3106200	310620060	31062006066
NORTE	310620060	VENDA NOVA	3106200	310620060	31062006066
NORTE	310620060	VENDA NOVA	3106200	310620060	31062006066
NORTE	310620060	VENDA NOVA	3106200	310620060	31062006066
NORTE	310620060	VENDA NOVA	3106200	310620060	31062006066
NORTE	310620060	VENDA NOVA	3106200	310620060	31062006066
NORTE	310620060	VENDA NOVA	3106200	310620060	31062006066
NORTE	310620060	VENDA NOVA	3106200	310620060	31062006066
NORTE	310620060	VENDA NOVA	3106200	310620060	31062006066
NORTE	310620060	VENDA NOVA	3106200	310620060	31062006066
NORTE	310620060	VENDA NOVA	3106200	310620060	31062006066
NORTE	310620060	VENDA NOVA	3106200	310620060	31062006066
NORTE	310620060	VENDA NOVA	3106200	310620060	31062006066
NORTE	310620060	VENDA NOVA	3106200	310620060	31062006066
NORTE	310620060	VENDA NOVA	3106200	310620060	31062006066
NORTE	310620060	VENDA NOVA	3106200	310620060	31062006066
NORTE	310620060	VENDA NOVA	3106200	310620060	31062006066
NORTE	310620060	VENDA NOVA	3106200	310620060	31062006066
NORTE	310620060	VENDA NOVA	3106200	310620060	31062006066
NORTE	310620060	VENDA NOVA	3106200	310620060	31062006066
NORTE	310620060	VENDA NOVA	3106200	310620060	31062006066
NORTE	310620060	VENDA NOVA	3106200	310620060	31062006066
NORTE	310620060	VENDA NOVA	3106200	310620060	31062006066
NORTE	310620060	VENDA NOVA	3106200	310620060	31062006066
NORTE	310620060	VENDA NOVA	3106200	310620060	31062006066
NORTE	310620060	VENDA NOVA	3106200	310620060	31062006066
NORTE	310620060	VENDA NOVA	3106200	310620060	31062006066
NORTE	310620060	VENDA NOVA	3106200	310620060	31062006066
NORTE	310620060	VENDA NOVA	3106200	310620060	31062006066
NORTE	310620060	VENDA NOVA	3106200	310620060	31062006066
NORTE	310620060	VENDA NOVA	3106200	310620060	31062006066
NORTE	310620060	VENDA NOVA	3106200	310620060	31062006066
NORTE	310620060	VENDA NOVA	3106200	310620060	31062006066
NORTE	310620060	VENDA NOVA	3106200	310620060	31062006066
NORTE	310620060	VENDA NOVA	3106200	310620060	31062006066
NORTE	310620060	VENDA NOVA	3106200	310620060	31062006066
NORTE	310620060	VENDA NOVA	3106200	310620060	31062006066
NORTE	310620060	VENDA NOVA	3106200	310620060	31062006066
NORTE	310620060	VENDA NOVA	3106200	310620060	31062006066
NORTE	310620060	VENDA NOVA	3106200	310620060	31062006066
NORTE	310620060	VENDA NOVA	3106200	310620060	31062006066
NORTE	310620060	VENDA NOVA	3106200	310620060	31062006066

NORTE	310620060	VENDA NOVA	3106200	310620060	31062006066
NORTE	310620060	VENDA NOVA	3106200	310620060	31062006066
NORTE	310620060	VENDA NOVA	3106200	310620060	31062006066
NORTE	310620060	VENDA NOVA	3106200	310620060	31062006066
NORTE	310620060	VENDA NOVA	3106200	310620060	31062006066
NORTE	310620060	VENDA NOVA	3106200	310620060	31062006066
NORTE	310620060	VENDA NOVA	3106200	310620060	31062006066
NORTE	310620060	VENDA NOVA	3106200	310620060	31062006066
NORTE	310620060	VENDA NOVA	3106200	310620060	31062006066
NORTE	310620060	VENDA NOVA	3106200	310620060	31062006066
NORTE	310620060	VENDA NOVA	3106200	310620060	31062006066
NORTE	310620060	VENDA NOVA	3106200	310620060	31062006066
NORTE	310620060	VENDA NOVA	3106200	310620060	31062006066
NORTE	310620060	VENDA NOVA	3106200	310620060	31062006066
NORTE	310620060	VENDA NOVA	3106200	310620060	31062006066
NORTE	310620060	VENDA NOVA	3106200	310620060	31062006066
NORTE	310620060	VENDA NOVA	3106200	310620060	31062006066
NORTE	310620060	VENDA NOVA	3106200	310620060	31062006066
NORTE	310620060	VENDA NOVA	3106200	310620060	31062006066
NORTE	310620060	VENDA NOVA	3106200	310620060	31062006066
NORTE	310620060	VENDA NOVA	3106200	310620060	31062006066
NORTE	310620060	VENDA NOVA	3106200	310620060	31062006066
NORTE	310620060	VENDA NOVA	3106200	310620060	31062006066
NORTE	310620060	VENDA NOVA	3106200	310620060	31062006066
NORTE	310620060	VENDA NOVA	3106200	310620060	31062006066
NORTE	310620060	VENDA NOVA	3106200	310620060	31062006066
NORTE	310620060	VENDA NOVA	3106200	310620060	31062006066
NORTE	310620060	VENDA NOVA	3106200	310620060	31062006066
NORTE	310620060	VENDA NOVA	3106200	310620060	31062006066
NORTE	310620060	VENDA NOVA	3106200	310620060	31062006066
NORTE	310620060	VENDA NOVA	3106200	310620060	31062006066
NORTE	310620060	VENDA NOVA	3106200	310620060	31062006066
NORTE	310620060	VENDA NOVA	3106200	310620060	31062006066
NORTE	310620060	VENDA NOVA	3106200	310620060	31062006066
NORTE	310620060	VENDA NOVA	3106200	310620060	31062006066
NORTE	310620060	VENDA NOVA	3106200	310620060	31062006066
NORTE	310620060	VENDA NOVA	3106200	310620060	31062006066
NORTE	310620060	VENDA NOVA	3106200	310620060	31062006066
NORTE	310620060	VENDA NOVA	3106200	310620060	31062006066
NORTE	310620060	VENDA NOVA	3106200	310620060	31062006066
NORTE	310620060	VENDA NOVA	3106200	310620060	31062006066
NORTE	310620060	VENDA NOVA	3106200	310620060	31062006066
NORTE	310620060	VENDA NOVA	3106200	310620060	31062006066
NORTE	310620060	VENDA NOVA	3106200	310620060	31062006066
NORTE	310620060	VENDA NOVA	3106200	310620060	31062006066
NORTE	310620060	VENDA NOVA	3106200	310620060	31062006066
NORTE	310620060	VENDA NOVA	3106200	310620060	31062006066
NORTE	310620060	VENDA NOVA	3106200	310620060	31062006066
NORTE	310620060	VENDA NOVA	3106200	310620060	31062006066
NORTE	310620060	VENDA NOVA	3106200	310620060	31062006066

NORTE	310620060	VENDA NOVA	3106200	310620060	31062006066
NORTE	310620060	VENDA NOVA	3106200	0	0
PAMPULHA	310620060	VENDA NOVA	3106200	310620060	31062006068
PAMPULHA	310620060	VENDA NOVA	3106200	310620060	31062006068
PAMPULHA	310620060	VENDA NOVA	3106200	310620060	31062006068
PAMPULHA	310620060	VENDA NOVA	3106200	310620060	31062006068
PAMPULHA	310620060	VENDA NOVA	3106200	310620060	31062006068
PAMPULHA	310620060	VENDA NOVA	3106200	310620060	31062006068
PAMPULHA	310620060	VENDA NOVA	3106200	310620060	31062006068
PAMPULHA	310620060	VENDA NOVA	3106200	310620060	31062006068
PAMPULHA	310620060	VENDA NOVA	3106200	310620060	31062006068
PAMPULHA	310620060	VENDA NOVA	3106200	310620060	31062006068
PAMPULHA	310620060	VENDA NOVA	3106200	310620060	31062006068
PAMPULHA	310620060	VENDA NOVA	3106200	310620060	31062006068
PAMPULHA	310620060	VENDA NOVA	3106200	310620060	31062006068
PAMPULHA	310620060	VENDA NOVA	3106200	310620060	31062006068
PAMPULHA	310620060	VENDA NOVA	3106200	310620060	31062006068
PAMPULHA	310620060	VENDA NOVA	3106200	310620060	31062006068
PAMPULHA	310620060	VENDA NOVA	3106200	310620060	31062006068
PAMPULHA	310620060	VENDA NOVA	3106200	310620060	31062006068
PAMPULHA	310620060	VENDA NOVA	3106200	310620060	31062006068
PAMPULHA	310620060	VENDA NOVA	3106200	310620060	31062006068
PAMPULHA	310620060	VENDA NOVA	3106200	310620060	31062006068
PAMPULHA	310620060	VENDA NOVA	3106200	310620060	31062006068
PAMPULHA	310620060	VENDA NOVA	3106200	310620060	31062006068
PAMPULHA	310620060	VENDA NOVA	3106200	310620060	31062006068
PAMPULHA	310620060	VENDA NOVA	3106200	310620060	31062006068
PAMPULHA	310620060	VENDA NOVA	3106200	310620060	31062006068
PAMPULHA	310620060	VENDA NOVA	3106200	310620060	31062006068
PAMPULHA	310620060	VENDA NOVA	3106200	310620060	31062006068
PAMPULHA	310620060	VENDA NOVA	3106200	310620060	31062006068
PAMPULHA	310620060	VENDA NOVA	3106200	310620060	31062006068
PAMPULHA	310620060	VENDA NOVA	3106200	310620060	31062006068
PAMPULHA	310620060	VENDA NOVA	3106200	310620060	31062006068
PAMPULHA	310620060	VENDA NOVA	3106200	310620060	31062006068
PAMPULHA	310620060	VENDA NOVA	3106200	310620060	31062006068
PAMPULHA	310620060	VENDA NOVA	3106200	310620060	31062006068
PAMPULHA	310620060	VENDA NOVA	3106200	310620060	31062006068
PAMPULHA	310620060	VENDA NOVA	3106200	310620060	31062006068
PAMPULHA	310620060	VENDA NOVA	3106200	310620060	31062006068
PAMPULHA	310620060	VENDA NOVA	3106200	310620060	31062006068
PAMPULHA	310620060	VENDA NOVA	3106200	310620060	31062006068
PAMPULHA	310620060	VENDA NOVA	3106200	310620060	31062006068
PAMPULHA	310620060	VENDA NOVA	3106200	310620060	31062006068
PAMPULHA	310620060	VENDA NOVA	3106200	310620060	31062006068
PAMPULHA	310620060	VENDA NOVA	3106200	310620060	31062006068
PAMPULHA	310620060	VENDA NOVA	3106200	310620060	31062006068
PAMPULHA	310620060	VENDA NOVA	3106200	310620060	31062006068
PAMPULHA	310620060	VENDA NOVA	3106200	310620060	31062006068

PAMPULHA	310620060	VENDA NOVA	3106200	310620060	31062006068
PAMPULHA	310620060	VENDA NOVA	3106200	310620060	31062006068
PAMPULHA	310620060	VENDA NOVA	3106200	310620060	31062006068
PAMPULHA	310620060	VENDA NOVA	3106200	310620060	31062006068
PAMPULHA	310620060	VENDA NOVA	3106200	310620060	31062006068
PAMPULHA	310620060	VENDA NOVA	3106200	310620060	31062006068
PAMPULHA	310620060	VENDA NOVA	3106200	310620060	31062006068
PAMPULHA	310620060	VENDA NOVA	3106200	310620060	31062006068
PAMPULHA	310620060	VENDA NOVA	3106200	310620060	31062006068
PAMPULHA	310620060	VENDA NOVA	3106200	310620060	31062006068
PAMPULHA	310620060	VENDA NOVA	3106200	310620060	31062006068
PAMPULHA	310620060	VENDA NOVA	3106200	310620060	31062006068
PAMPULHA	310620060	VENDA NOVA	3106200	310620060	31062006068
PAMPULHA	310620060	VENDA NOVA	3106200	310620060	31062006068
PAMPULHA	310620060	VENDA NOVA	3106200	310620060	31062006068
PAMPULHA	310620060	VENDA NOVA	3106200	310620060	31062006068
PAMPULHA	310620060	VENDA NOVA	3106200	310620060	31062006068
VENDA NOVA	310620060	VENDA NOVA	3106200	310620060	31062006069
VENDA NOVA	310620060	VENDA NOVA	3106200	310620060	31062006069
VENDA NOVA	310620060	VENDA NOVA	3106200	310620060	31062006069
VENDA NOVA	310620060	VENDA NOVA	3106200	310620060	31062006069
VENDA NOVA	310620060	VENDA NOVA	3106200	310620060	31062006069
VENDA NOVA	310620060	VENDA NOVA	3106200	310620060	31062006069
VENDA NOVA	310620060	VENDA NOVA	3106200	310620060	31062006069
VENDA NOVA	310620060	VENDA NOVA	3106200	310620060	31062006069
VENDA NOVA	310620060	VENDA NOVA	3106200	310620060	31062006069
VENDA NOVA	310620060	VENDA NOVA	3106200	310620060	31062006069
VENDA NOVA	310620060	VENDA NOVA	3106200	310620060	31062006069
VENDA NOVA	310620060	VENDA NOVA	3106200	310620060	31062006069
VENDA NOVA	310620060	VENDA NOVA	3106200	310620060	31062006069
VENDA NOVA	310620060	VENDA NOVA	3106200	310620060	31062006069
VENDA NOVA	310620060	VENDA NOVA	3106200	310620060	31062006069
VENDA NOVA	310620060	VENDA NOVA	3106200	310620060	31062006069
VENDA NOVA	310620060	VENDA NOVA	3106200	310620060	31062006069
VENDA NOVA	310620060	VENDA NOVA	3106200	310620060	31062006069
VENDA NOVA	310620060	VENDA NOVA	3106200	310620060	31062006069
VENDA NOVA	310620060	VENDA NOVA	3106200	310620060	31062006069
VENDA NOVA	310620060	VENDA NOVA	3106200	310620060	31062006069
VENDA NOVA	310620060	VENDA NOVA	3106200	310620060	31062006069
VENDA NOVA	310620060	VENDA NOVA	3106200	310620060	31062006069
VENDA NOVA	310620060	VENDA NOVA	3106200	310620060	31062006069
VENDA NOVA	310620060	VENDA NOVA	3106200	310620060	31062006069
VENDA NOVA	310620060	VENDA NOVA	3106200	310620060	31062006069
VENDA NOVA	310620060	VENDA NOVA	3106200	310620060	31062006069
VENDA NOVA	310620060	VENDA NOVA	3106200	310620060	31062006069
VENDA NOVA	310620060	VENDA NOVA	3106200	310620060	31062006069
VENDA NOVA	310620060	VENDA NOVA	3106200	310620060	31062006069
VENDA NOVA	310620060	VENDA NOVA	3106200	310620060	31062006069
VENDA NOVA	310620060	VENDA NOVA	3106200	310620060	31062006069
VENDA NOVA	310620060	VENDA NOVA	3106200	310620060	31062006069

VENDA NOVA	310620060	VENDA NOVA	3106200	310620060	31062006069
VENDA NOVA	310620060	VENDA NOVA	3106200	310620060	31062006069
VENDA NOVA	310620060	VENDA NOVA	3106200	310620060	31062006069
VENDA NOVA	310620060	VENDA NOVA	3106200	310620060	31062006069
VENDA NOVA	310620060	VENDA NOVA	3106200	310620060	31062006069
VENDA NOVA	310620060	VENDA NOVA	3106200	310620060	31062006069
VENDA NOVA	310620060	VENDA NOVA	3106200	310620060	31062006069
VENDA NOVA	310620060	VENDA NOVA	3106200	310620060	31062006069
VENDA NOVA	310620060	VENDA NOVA	3106200	310620060	31062006069
VENDA NOVA	310620060	VENDA NOVA	3106200	310620060	31062006069
VENDA NOVA	310620060	VENDA NOVA	3106200	310620060	31062006069
VENDA NOVA	310620060	VENDA NOVA	3106200	310620060	31062006069
VENDA NOVA	310620060	VENDA NOVA	3106200	310620060	31062006069
VENDA NOVA	310620060	VENDA NOVA	3106200	310620060	31062006069
VENDA NOVA	310620060	VENDA NOVA	3106200	310620060	31062006069
VENDA NOVA	310620060	VENDA NOVA	3106200	310620060	31062006069
VENDA NOVA	310620060	VENDA NOVA	3106200	310620060	31062006069
VENDA NOVA	310620060	VENDA NOVA	3106200	310620060	31062006069
VENDA NOVA	310620060	VENDA NOVA	3106200	310620060	31062006069
VENDA NOVA	310620060	VENDA NOVA	3106200	310620060	31062006069
VENDA NOVA	310620060	VENDA NOVA	3106200	310620060	31062006069
VENDA NOVA	310620060	VENDA NOVA	3106200	310620060	31062006069
VENDA NOVA	310620060	VENDA NOVA	3106200	310620060	31062006069
VENDA NOVA	310620060	VENDA NOVA	3106200	310620060	31062006069
VENDA NOVA	310620060	VENDA NOVA	3106200	310620060	31062006069
VENDA NOVA	310620060	VENDA NOVA	3106200	310620060	31062006069
VENDA NOVA	310620060	VENDA NOVA	3106200	310620060	31062006069
VENDA NOVA	310620060	VENDA NOVA	3106200	310620060	31062006069
VENDA NOVA	310620060	VENDA NOVA	3106200	310620060	31062006069
VENDA NOVA	310620060	VENDA NOVA	3106200	310620060	31062006069
VENDA NOVA	310620060	VENDA NOVA	3106200	310620060	31062006069
VENDA NOVA	310620060	VENDA NOVA	3106200	310620060	31062006069
VENDA NOVA	310620060	VENDA NOVA	3106200	310620060	31062006069
VENDA NOVA	310620060	VENDA NOVA	3106200	310620060	31062006069
VENDA NOVA	310620060	VENDA NOVA	3106200	310620060	31062006069
VENDA NOVA	310620060	VENDA NOVA	3106200	310620060	31062006069
VENDA NOVA	310620060	VENDA NOVA	3106200	310620060	31062006069
VENDA NOVA	310620060	VENDA NOVA	3106200	310620060	31062006069
VENDA NOVA	310620060	VENDA NOVA	3106200	310620060	31062006069
VENDA NOVA	310620060	VENDA NOVA	3106200	310620060	31062006069
VENDA NOVA	310620060	VENDA NOVA	3106200	310620060	31062006069
VENDA NOVA	310620060	VENDA NOVA	3106200	310620060	31062006069
VENDA NOVA	310620060	VENDA NOVA	3106200	310620060	31062006069
VENDA NOVA	310620060	VENDA NOVA	3106200	310620060	31062006069
VENDA NOVA	310620060	VENDA NOVA	3106200	310620060	31062006069
VENDA NOVA	310620060	VENDA NOVA	3106200	310620060	31062006069
VENDA NOVA	310620060	VENDA NOVA	3106200	310620060	31062006069
VENDA NOVA	310620060	VENDA NOVA	3106200	310620060	31062006069
VENDA NOVA	310620060	VENDA NOVA	3106200	310620060	31062006069
VENDA NOVA	310620060	VENDA NOVA	3106200	310620060	31062006069

VENDA NOVA	310620060	VENDA NOVA	3106200	310620060	31062006069
VENDA NOVA	310620060	VENDA NOVA	3106200	310620060	31062006069
VENDA NOVA	310620060	VENDA NOVA	3106200	310620060	31062006069
VENDA NOVA	310620060	VENDA NOVA	3106200	310620060	31062006069
VENDA NOVA	310620060	VENDA NOVA	3106200	310620060	31062006069
VENDA NOVA	310620060	VENDA NOVA	3106200	310620060	31062006069
VENDA NOVA	310620060	VENDA NOVA	3106200	310620060	31062006069
VENDA NOVA	310620060	VENDA NOVA	3106200	310620060	31062006069
VENDA NOVA	310620060	VENDA NOVA	3106200	310620060	31062006069
VENDA NOVA	310620060	VENDA NOVA	3106200	310620060	31062006069
VENDA NOVA	310620060	VENDA NOVA	3106200	310620060	31062006069
VENDA NOVA	310620060	VENDA NOVA	3106200	310620060	31062006069
VENDA NOVA	310620060	VENDA NOVA	3106200	310620060	31062006069
VENDA NOVA	310620060	VENDA NOVA	3106200	310620060	31062006069
VENDA NOVA	310620060	VENDA NOVA	3106200	310620060	31062006069
VENDA NOVA	310620060	VENDA NOVA	3106200	310620060	31062006069
VENDA NOVA	310620060	VENDA NOVA	3106200	310620060	31062006069
VENDA NOVA	310620060	VENDA NOVA	3106200	310620060	31062006069
VENDA NOVA	310620060	VENDA NOVA	3106200	310620060	31062006069
VENDA NOVA	310620060	VENDA NOVA	3106200	310620060	31062006069
VENDA NOVA	310620060	VENDA NOVA	3106200	310620060	31062006069
VENDA NOVA	310620060	VENDA NOVA	3106200	310620060	31062006069
VENDA NOVA	310620060	VENDA NOVA	3106200	310620060	31062006069
VENDA NOVA	310620060	VENDA NOVA	3106200	310620060	31062006069
VENDA NOVA	310620060	VENDA NOVA	3106200	310620060	31062006069
VENDA NOVA	310620060	VENDA NOVA	3106200	310620060	31062006069
VENDA NOVA	310620060	VENDA NOVA	3106200	310620060	31062006069
VENDA NOVA	310620060	VENDA NOVA	3106200	310620060	31062006069
VENDA NOVA	310620060	VENDA NOVA	3106200	310620060	31062006069
VENDA NOVA	310620060	VENDA NOVA	3106200	310620060	31062006069
VENDA NOVA	310620060	VENDA NOVA	3106200	310620060	31062006069
VENDA NOVA	310620060	VENDA NOVA	3106200	310620060	31062006069
VENDA NOVA	310620060	VENDA NOVA	3106200	310620060	31062006069
VENDA NOVA	310620060	VENDA NOVA	3106200	310620060	31062006069
VENDA NOVA	310620060	VENDA NOVA	3106200	310620060	31062006069
VENDA NOVA	310620060	VENDA NOVA	3106200	310620060	31062006069
VENDA NOVA	310620060	VENDA NOVA	3106200	310620060	31062006069
VENDA NOVA	310620060	VENDA NOVA	3106200	310620060	31062006069
VENDA NOVA	310620060	VENDA NOVA	3106200	310620060	31062006069
VENDA NOVA	310620060	VENDA NOVA	3106200	310620060	31062006069
VENDA NOVA	310620060	VENDA NOVA	3106200	310620060	31062006069
VENDA NOVA	310620060	VENDA NOVA	3106200	310620060	31062006069
VENDA NOVA	310620060	VENDA NOVA	3106200	310620060	31062006069
VENDA NOVA	310620060	VENDA NOVA	3106200	310620060	31062006069
VENDA NOVA	310620060	VENDA NOVA	3106200	310620060	31062006069
VENDA NOVA	310620060	VENDA NOVA	3106200	310620060	31062006069
VENDA NOVA	310620060	VENDA NOVA	3106200	310620060	31062006069
VENDA NOVA	310620060	VENDA NOVA	3106200	310620060	31062006069
VENDA NOVA	310620060	VENDA NOVA	3106200	310620060	31062006069
VENDA NOVA	310620060	VENDA NOVA	3106200	310620060	31062006069

VENDA NOVA	310620060	VENDA NOVA	3106200	310620060	31062006069
VENDA NOVA	310620060	VENDA NOVA	3106200	310620060	31062006069
VENDA NOVA	310620060	VENDA NOVA	3106200	310620060	31062006069
VENDA NOVA	310620060	VENDA NOVA	3106200	310620060	31062006069
VENDA NOVA	310620060	VENDA NOVA	3106200	310620060	31062006069
VENDA NOVA	310620060	VENDA NOVA	3106200	310620060	31062006069
VENDA NOVA	310620060	VENDA NOVA	3106200	310620060	31062006069
VENDA NOVA	310620060	VENDA NOVA	3106200	310620060	31062006069
VENDA NOVA	310620060	VENDA NOVA	3106200	310620060	31062006069
VENDA NOVA	310620060	VENDA NOVA	3106200	310620060	31062006069
VENDA NOVA	310620060	VENDA NOVA	3106200	310620060	31062006069
VENDA NOVA	310620060	VENDA NOVA	3106200	310620060	31062006069
VENDA NOVA	310620060	VENDA NOVA	3106200	310620060	31062006069
VENDA NOVA	310620060	VENDA NOVA	3106200	310620060	31062006069
VENDA NOVA	310620060	VENDA NOVA	3106200	310620060	31062006069
VENDA NOVA	310620060	VENDA NOVA	3106200	310620060	31062006069
VENDA NOVA	310620060	VENDA NOVA	3106200	310620060	31062006069
VENDA NOVA	310620060	VENDA NOVA	3106200	310620060	31062006069
VENDA NOVA	310620060	VENDA NOVA	3106200	310620060	31062006069
VENDA NOVA	310620060	VENDA NOVA	3106200	310620060	31062006069
VENDA NOVA	310620060	VENDA NOVA	3106200	310620060	31062006069
VENDA NOVA	310620060	VENDA NOVA	3106200	310620060	31062006069
VENDA NOVA	310620060	VENDA NOVA	3106200	310620060	31062006069
VENDA NOVA	310620060	VENDA NOVA	3106200	310620060	31062006069
VENDA NOVA	310620060	VENDA NOVA	3106200	310620060	31062006069
VENDA NOVA	310620060	VENDA NOVA	3106200	310620060	31062006069
VENDA NOVA	310620060	VENDA NOVA	3106200	310620060	31062006069
VENDA NOVA	310620060	VENDA NOVA	3106200	310620060	31062006069
VENDA NOVA	310620060	VENDA NOVA	3106200	310620060	31062006069
VENDA NOVA	310620060	VENDA NOVA	3106200	310620060	31062006069
VENDA NOVA	310620060	VENDA NOVA	3106200	310620060	31062006069
VENDA NOVA	310620060	VENDA NOVA	3106200	310620060	31062006069
VENDA NOVA	310620060	VENDA NOVA	3106200	310620060	31062006069
VENDA NOVA	310620060	VENDA NOVA	3106200	310620060	31062006069
VENDA NOVA	310620060	VENDA NOVA	3106200	310620060	31062006069
VENDA NOVA	310620060	VENDA NOVA	3106200	310620060	31062006069
VENDA NOVA	310620060	VENDA NOVA	3106200	310620060	31062006069
VENDA NOVA	310620060	VENDA NOVA	3106200	310620060	31062006069
VENDA NOVA	310620060	VENDA NOVA	3106200	310620060	31062006069
VENDA NOVA	310620060	VENDA NOVA	3106200	310620060	31062006069
VENDA NOVA	310620060	VENDA NOVA	3106200	310620060	31062006069
VENDA NOVA	310620060	VENDA NOVA	3106200	310620060	31062006069
VENDA NOVA	310620060	VENDA NOVA	3106200	310620060	31062006069
VENDA NOVA	310620060	VENDA NOVA	3106200	310620060	31062006069
VENDA NOVA	310620060	VENDA NOVA	3106200	310620060	31062006069
VENDA NOVA	310620060	VENDA NOVA	3106200	310620060	31062006069
VENDA NOVA	310620060	VENDA NOVA	3106200	310620060	31062006069
VENDA NOVA	310620060	VENDA NOVA	3106200	310620060	31062006069
VENDA NOVA	310620060	VENDA NOVA	3106200	310620060	31062006069
VENDA NOVA	310620060	VENDA NOVA	3106200	310620060	31062006069

VENDA NOVA	310620060	VENDA NOVA	3106200	310620060	31062006069
VENDA NOVA	310620060	VENDA NOVA	3106200	310620060	31062006069
VENDA NOVA	310620060	VENDA NOVA	3106200	310620060	31062006069
VENDA NOVA	310620060	VENDA NOVA	3106200	310620060	31062006069
VENDA NOVA	310620060	VENDA NOVA	3106200	310620060	31062006069
VENDA NOVA	310620060	VENDA NOVA	3106200	310620060	31062006069
VENDA NOVA	310620060	VENDA NOVA	3106200	310620060	31062006069
VENDA NOVA	310620060	VENDA NOVA	3106200	310620060	31062006069
VENDA NOVA	310620060	VENDA NOVA	3106200	310620060	31062006069
VENDA NOVA	310620060	VENDA NOVA	3106200	310620060	31062006069
VENDA NOVA	310620060	VENDA NOVA	3106200	310620060	31062006069
VENDA NOVA	310620060	VENDA NOVA	3106200	310620060	31062006069
VENDA NOVA	310620060	VENDA NOVA	3106200	310620060	31062006069
VENDA NOVA	310620060	VENDA NOVA	3106200	310620060	31062006069
VENDA NOVA	310620060	VENDA NOVA	3106200	310620060	31062006069
VENDA NOVA	310620060	VENDA NOVA	3106200	310620060	31062006069
VENDA NOVA	310620060	VENDA NOVA	3106200	310620060	31062006069
VENDA NOVA	310620060	VENDA NOVA	3106200	310620060	31062006069
VENDA NOVA	310620060	VENDA NOVA	3106200	310620060	31062006069
VENDA NOVA	310620060	VENDA NOVA	3106200	310620060	31062006069
VENDA NOVA	310620060	VENDA NOVA	3106200	310620060	31062006069
VENDA NOVA	310620060	VENDA NOVA	3106200	310620060	31062006069
VENDA NOVA	310620060	VENDA NOVA	3106200	310620060	31062006069
VENDA NOVA	310620060	VENDA NOVA	3106200	310620060	31062006069
VENDA NOVA	310620060	VENDA NOVA	3106200	310620060	31062006069
VENDA NOVA	310620060	VENDA NOVA	3106200	310620060	31062006069
VENDA NOVA	310620060	VENDA NOVA	3106200	310620060	31062006069
VENDA NOVA	310620060	VENDA NOVA	3106200	310620060	31062006069
VENDA NOVA	310620060	VENDA NOVA	3106200	310620060	31062006069
VENDA NOVA	310620060	VENDA NOVA	3106200	310620060	31062006069
VENDA NOVA	310620060	VENDA NOVA	3106200	310620060	31062006069
VENDA NOVA	310620060	VENDA NOVA	3106200	310620060	31062006069
VENDA NOVA	310620060	VENDA NOVA	3106200	310620060	31062006069
VENDA NOVA	310620060	VENDA NOVA	3106200	310620060	31062006069
VENDA NOVA	310620060	VENDA NOVA	3106200	310620060	31062006069
VENDA NOVA	310620060	VENDA NOVA	3106200	310620060	31062006069
VENDA NOVA	310620060	VENDA NOVA	3106200	310620060	31062006069
VENDA NOVA	310620060	VENDA NOVA	3106200	310620060	31062006069
VENDA NOVA	310620060	VENDA NOVA	3106200	310620060	31062006069
VENDA NOVA	310620060	VENDA NOVA	3106200	310620060	31062006069
VENDA NOVA	310620060	VENDA NOVA	3106200	310620060	31062006069
VENDA NOVA	310620060	VENDA NOVA	3106200	0	0
VENDA NOVA	310620060	VENDA NOVA	3106200	310620060	31062006069
VENDA NOVA	310620060	VENDA NOVA	3106200	310620060	31062006069
VENDA NOVA	310620060	VENDA NOVA	3106200	310620060	31062006069
VENDA NOVA	310620060	VENDA NOVA	3106200	310620060	31062006069
VENDA NOVA	310620060	VENDA NOVA	3106200	310620060	31062006069
VENDA NOVA	310620060	VENDA NOVA	3106200	310620060	31062006069
VENDA NOVA	310620060	VENDA NOVA	3106200	310620060	31062006069
VENDA NOVA	310620060	VENDA NOVA	3106200	310620060	31062006069

VENDA NOVA	310620060	VENDA NOVA	3106200	310620060	31062006069
VENDA NOVA	310620060	VENDA NOVA	3106200	310620060	31062006069
VENDA NOVA	310620060	VENDA NOVA	3106200	310620060	31062006069
VENDA NOVA	310620060	VENDA NOVA	3106200	310620060	31062006069
VENDA NOVA	310620060	VENDA NOVA	3106200	310620060	31062006069
VENDA NOVA	310620060	VENDA NOVA	3106200	310620060	31062006069
VENDA NOVA	310620060	VENDA NOVA	3106200	310620060	31062006069
VENDA NOVA	310620060	VENDA NOVA	3106200	310620060	31062006069
VENDA NOVA	310620060	VENDA NOVA	3106200	310620060	31062006069
VENDA NOVA	310620060	VENDA NOVA	3106200	310620060	31062006069
VENDA NOVA	310620060	VENDA NOVA	3106200	310620060	31062006069
VENDA NOVA	310620060	VENDA NOVA	3106200	310620060	31062006069
VENDA NOVA	310620060	VENDA NOVA	3106200	310620060	31062006069
VENDA NOVA	310620060	VENDA NOVA	3106200	310620060	31062006069
VENDA NOVA	310620060	VENDA NOVA	3106200	310620060	31062006069
VENDA NOVA	310620060	VENDA NOVA	3106200	310620060	31062006069
VENDA NOVA	310620060	VENDA NOVA	3106200	310620060	31062006069
VENDA NOVA	310620060	VENDA NOVA	3106200	310620060	31062006069
VENDA NOVA	310620060	VENDA NOVA	3106200	310620060	31062006069
VENDA NOVA	310620060	VENDA NOVA	3106200	310620060	31062006069
VENDA NOVA	310620060	VENDA NOVA	3106200	310620060	31062006069
VENDA NOVA	310620060	VENDA NOVA	3106200	310620060	31062006069
VENDA NOVA	310620060	VENDA NOVA	3106200	310620060	31062006069
VENDA NOVA	310620060	VENDA NOVA	3106200	310620060	31062006069
VENDA NOVA	310620060	VENDA NOVA	3106200	310620060	31062006069
VENDA NOVA	310620060	VENDA NOVA	3106200	310620060	31062006069
VENDA NOVA	310620060	VENDA NOVA	3106200	310620060	31062006069
VENDA NOVA	310620060	VENDA NOVA	3106200	310620060	31062006069
VENDA NOVA	310620060	VENDA NOVA	3106200	310620060	31062006069
VENDA NOVA	310620060	VENDA NOVA	3106200	310620060	31062006069
VENDA NOVA	310620060	VENDA NOVA	3106200	310620060	31062006069
VENDA NOVA	310620060	VENDA NOVA	3106200	310620060	31062006069
VENDA NOVA	310620060	VENDA NOVA	3106200	310620060	31062006069
VENDA NOVA	310620060	VENDA NOVA	3106200	310620060	31062006069
VENDA NOVA	310620060	VENDA NOVA	3106200	310620060	31062006069
VENDA NOVA	310620060	VENDA NOVA	3106200	310620060	31062006069
VENDA NOVA	310620060	VENDA NOVA	3106200	310620060	31062006069
VENDA NOVA	310620060	VENDA NOVA	3106200	310620060	31062006069
VENDA NOVA	310620060	VENDA NOVA	3106200	310620060	31062006069
VENDA NOVA	310620060	VENDA NOVA	3106200	310620060	31062006069
VENDA NOVA	310620060	VENDA NOVA	3106200	310620060	31062006069
VENDA NOVA	310620060	VENDA NOVA	3106200	310620060	31062006069
VENDA NOVA	310620060	VENDA NOVA	3106200	310620060	31062006069
VENDA NOVA	310620060	VENDA NOVA	3106200	310620060	31062006069
VENDA NOVA	310620060	VENDA NOVA	3106200	310620060	31062006069
VENDA NOVA	310620060	VENDA NOVA	3106200	310620060	31062006069
VENDA NOVA	310620060	VENDA NOVA	3106200	310620060	31062006069
VENDA NOVA	310620060	VENDA NOVA	3106200	310620060	31062006069
VENDA NOVA	310620060	VENDA NOVA	3106200	310620060	31062006069
VENDA NOVA	310620060	VENDA NOVA	3106200	310620060	31062006069

VENDA NOVA	310620060	VENDA NOVA	3106200	310620060	31062006069
VENDA NOVA	310620060	VENDA NOVA	3106200	310620060	31062006069
VENDA NOVA	310620060	VENDA NOVA	3106200	310620060	31062006069
VENDA NOVA	310620060	VENDA NOVA	3106200	310620060	31062006069
VENDA NOVA	310620060	VENDA NOVA	3106200	310620060	31062006069
VENDA NOVA	310620060	VENDA NOVA	3106200	310620060	31062006069
VENDA NOVA	310620060	VENDA NOVA	3106200	310620060	31062006069
VENDA NOVA	310620060	VENDA NOVA	3106200	310620060	31062006069
VENDA NOVA	310620060	VENDA NOVA	3106200	310620060	31062006069
VENDA NOVA	310620060	VENDA NOVA	3106200	310620060	31062006069
VENDA NOVA	310620060	VENDA NOVA	3106200	310620060	31062006069
VENDA NOVA	310620060	VENDA NOVA	3106200	310620060	31062006069
VENDA NOVA	310620060	VENDA NOVA	3106200	310620060	31062006069
VENDA NOVA	310620060	VENDA NOVA	3106200	310620060	31062006069
VENDA NOVA	310620060	VENDA NOVA	3106200	310620060	31062006069
VENDA NOVA	310620060	VENDA NOVA	3106200	310620060	31062006069
VENDA NOVA	310620060	VENDA NOVA	3106200	310620060	31062006069
VENDA NOVA	310620060	VENDA NOVA	3106200	310620060	31062006069
VENDA NOVA	310620060	VENDA NOVA	3106200	310620060	31062006069
VENDA NOVA	310620060	VENDA NOVA	3106200	310620060	31062006069
VENDA NOVA	310620060	VENDA NOVA	3106200	310620060	31062006069
VENDA NOVA	310620060	VENDA NOVA	3106200	310620060	31062006069
VENDA NOVA	310620060	VENDA NOVA	3106200	310620060	31062006069
VENDA NOVA	310620060	VENDA NOVA	3106200	310620060	31062006069
VENDA NOVA	310620060	VENDA NOVA	3106200	310620060	31062006069
VENDA NOVA	310620060	VENDA NOVA	3106200	310620060	31062006069
VENDA NOVA	310620060	VENDA NOVA	3106200	310620060	31062006069
VENDA NOVA	310620060	VENDA NOVA	3106200	310620060	31062006069
VENDA NOVA	310620060	VENDA NOVA	3106200	310620060	31062006069
VENDA NOVA	310620060	VENDA NOVA	3106200	310620060	31062006069
VENDA NOVA	310620060	VENDA NOVA	3106200	310620060	31062006069
VENDA NOVA	310620060	VENDA NOVA	3106200	310620060	31062006069
VENDA NOVA	310620060	VENDA NOVA	3106200	310620060	31062006069
VENDA NOVA	310620060	VENDA NOVA	3106200	310620060	31062006069
VENDA NOVA	310620060	VENDA NOVA	3106200	310620060	31062006069
VENDA NOVA	310620060	VENDA NOVA	3106200	310620060	31062006069
VENDA NOVA	310620060	VENDA NOVA	3106200	310620060	31062006069
VENDA NOVA	310620060	VENDA NOVA	3106200	310620060	31062006069
VENDA NOVA	310620060	VENDA NOVA	3106200	310620060	31062006069
VENDA NOVA	310620060	VENDA NOVA	3106200	310620060	31062006069
VENDA NOVA	310620060	VENDA NOVA	3106200	310620060	31062006069
VENDA NOVA	310620060	VENDA NOVA	3106200	310620060	31062006069
VENDA NOVA	310620060	VENDA NOVA	3106200	310620060	31062006069
VENDA NOVA	310620060	VENDA NOVA	3106200	310620060	31062006069
VENDA NOVA	310620060	VENDA NOVA	3106200	310620060	31062006069
VENDA NOVA	310620060	VENDA NOVA	3106200	310620060	31062006069
VENDA NOVA	310620060	VENDA NOVA	3106200	310620060	31062006069
VENDA NOVA	310620060	VENDA NOVA	3106200	310620060	31062006069
VENDA NOVA	310620060	VENDA NOVA	3106200	310620060	31062006069
VENDA NOVA	310620060	VENDA NOVA	3106200	310620060	31062006069

VENDA NOVA	310620060	VENDA NOVA	3106200	310620060	31062006069
VENDA NOVA	310620060	VENDA NOVA	3106200	310620060	31062006069
VENDA NOVA	310620060	VENDA NOVA	3106200	310620060	31062006069
VENDA NOVA	310620060	VENDA NOVA	3106200	310620060	31062006069
VENDA NOVA	310620060	VENDA NOVA	3106200	310620060	31062006069
VENDA NOVA	310620060	VENDA NOVA	3106200	310620060	31062006069
VENDA NOVA	310620060	VENDA NOVA	3106200	310620060	31062006069
VENDA NOVA	310620060	VENDA NOVA	3106200	310620060	31062006069
VENDA NOVA	310620060	VENDA NOVA	3106200	310620060	31062006069
VENDA NOVA	310620060	VENDA NOVA	3106200	310620060	31062006069
VENDA NOVA	310620060	VENDA NOVA	3106200	310620060	31062006069
VENDA NOVA	310620060	VENDA NOVA	3106200	310620060	31062006069
VENDA NOVA	310620060	VENDA NOVA	3106200	310620060	31062006069
VENDA NOVA	310620060	VENDA NOVA	3106200	310620060	31062006069
VENDA NOVA	310620060	VENDA NOVA	3106200	310620060	31062006069
VENDA NOVA	310620060	VENDA NOVA	3106200	310620060	31062006069
VENDA NOVA	310620060	VENDA NOVA	3106200	310620060	31062006069
VENDA NOVA	310620060	VENDA NOVA	3106200	310620060	31062006069
VENDA NOVA	310620060	VENDA NOVA	3106200	310620060	31062006069
VENDA NOVA	310620060	VENDA NOVA	3106200	310620060	31062006069
VENDA NOVA	310620060	VENDA NOVA	3106200	310620060	31062006069
VENDA NOVA	310620060	VENDA NOVA	3106200	310620060	31062006069
VENDA NOVA	310620060	VENDA NOVA	3106200	310620060	31062006069
VENDA NOVA	310620060	VENDA NOVA	3106200	310620060	31062006069
VENDA NOVA	310620060	VENDA NOVA	3106200	310620060	31062006069
VENDA NOVA	310620060	VENDA NOVA	3106200	310620060	31062006069
VENDA NOVA	310620060	VENDA NOVA	3106200	310620060	31062006069
VENDA NOVA	310620060	VENDA NOVA	3106200	310620060	31062006069
VENDA NOVA	310620060	VENDA NOVA	3106200	310620060	31062006069
VENDA NOVA	310620060	VENDA NOVA	3106200	310620060	31062006069
VENDA NOVA	310620060	VENDA NOVA	3106200	310620060	31062006069
VENDA NOVA	310620060	VENDA NOVA	3106200	310620060	31062006069
VENDA NOVA	310620060	VENDA NOVA	3106200	310620060	31062006069
VENDA NOVA	310620060	VENDA NOVA	3106200	310620060	31062006069
VENDA NOVA	310620060	VENDA NOVA	3106200	310620060	31062006069
VENDA NOVA	310620060	VENDA NOVA	3106200	310620060	31062006069

SITUACAO_BASICO	DOMPPER	POPURESI	MEDNUMMO	VARENDIN	Situacao_1
1	296	625	2,11	3350,19	1
1	180	444	2,47	2233,41	1
1	252	662	2,63	4728,53	1
1	133	357	2,68	6013,56	1
1	289	680	2,35	3911,70	1
1	317	1055	3,33	906,74	1
1	156	489	3,13	755,32	1
1	150	411	2,74	13063,88	1
1	199	704	3,54	8011,87	1
1	198	605	3,06	8051,08	1
1	242	681	2,81	2916,34	1
1	265	641	2,42	3056,91	1
1	188	487	2,59	4047,15	1
1	276	725	2,63	5668,78	1
1	237	624	2,63	5508,63	1
1	283	796	2,81	7866,05	1
1	151	437	2,89	7117,48	1
1	188	638	3,39	10638,81	1
1	133	434	3,26	8377,14	1
1	191	520	2,72	7863,68	1
1	294	900	3,06	7669,99	1
1	203	428	2,11	3692,99	1
1	152	401	2,64	5092,13	1
1	211	649	3,08	8999,92	1
1	176	496	2,82	7530,20	1
1	207	632	3,05	51720,43	1
1	337	996	2,96	4351,36	1
1	319	1019	3,19	2330,96	1
1	275	856	3,11	2799,84	1
1	319	970	3,04	2473,67	1
1	0	0	0,00	0,00	1
1	320	1002	3,13	2080,77	1
1	219	723	3,30	1778,18	1
1	196	621	3,17	2987,14	1
1	242	748	3,09	1579,27	1
1	73	206	2,82	1766,16	1
1	167	523	3,13	3985,03	1
1	300	914	3,05	2494,93	1
1	281	916	3,26	1813,36	1
1	187	597	3,19	1584,91	1
1	344	1134	3,30	1195,01	1
1	309	974	3,15	1496,96	1
1	229	784	3,42	1584,22	1
1	257	865	3,37	1057,48	1
1	289	1057	3,66	680,21	1
1	221	717	3,24	2156,99	1
1	186	568	3,05	1944,76	1
1	302	874	2,89	4659,56	1
1	239	654	2,74	3072,84	1

1	208	521	2,50	3392,43	1
1	252	725	2,88	4824,51	1
1	319	835	2,62	4904,61	1
1	97	292	3,01	4557,13	1
1	220	650	2,95	3493,08	1
1	137	411	3,00	2606,68	1
1	176	504	2,86	2735,78	1
1	268	732	2,73	1937,96	1
1	289	803	2,78	1656,97	1
1	188	507	2,70	1990,98	1
1	193	555	2,88	1682,40	1
1	236	577	2,44	1745,47	1
1	210	670	3,19	3309,60	1
1	330	1018	3,08	4724,72	1
1	315	1009	3,20	3218,91	1
1	173	502	2,90	2599,83	1
1	153	433	2,83	1915,00	1
1	107	397	3,71	2315,51	1
1	212	551	2,60	2177,86	1
1	340	943	2,77	2495,28	1
1	212	511	2,41	6980,83	1
1	189	587	3,11	11174,20	1
1	295	823	2,79	8025,51	1
1	226	642	2,84	5359,17	1
1	295	801	2,72	7274,49	1
1	301	869	2,89	8731,33	1
1	363	1025	2,82	8038,02	1
1	212	550	2,59	6885,03	1
1	277	776	2,80	7203,21	1
1	165	420	2,55	5529,41	1
1	299	731	2,44	7073,85	1
1	194	479	2,47	9237,22	1
1	190	571	3,01	11240,55	1
1	342	733	2,14	6140,09	1
1	80	156	1,95	6264,11	1
1	340	845	2,49	6943,37	1
1	259	673	2,60	6168,53	1
1	222	649	2,92	8841,66	1
1	251	607	2,42	7351,51	1
1	266	731	2,75	4000,86	1
1	270	665	2,46	3571,24	1
1	329	982	2,98	3811,40	1
1	164	449	2,74	3942,90	1
1	156	411	2,63	5169,01	1
1	247	650	2,63	3153,96	1
1	279	730	2,62	5495,93	1
1	286	801	2,80	7204,94	1
1	237	625	2,64	5027,48	1
1	259	688	2,66	4951,93	1
1	252	736	2,92	6511,40	1

1	144	380	2,64	8712,51	1
1	266	824	3,10	10059,28	1
1	294	803	2,73	6449,19	1
1	237	643	2,71	5057,99	1
1	298	761	2,55	5489,40	1
1	253	711	2,81	5546,96	1
1	307	780	2,54	4669,00	1
1	313	903	2,88	6707,04	1
1	222	658	2,96	8091,40	1
1	152	369	2,43	4757,57	1
1	207	491	2,37	4565,31	1
1	177	402	2,27	6560,58	1
1	223	539	2,42	4448,16	1
1	155	349	2,25	4473,34	1
1	247	591	2,39	7599,97	1
1	363	841	2,32	8395,23	1
1	165	395	2,39	7812,69	1
1	108	232	2,15	7462,30	1
1	166	353	2,13	5711,40	1
1	180	426	2,37	6350,01	1
1	0	0	0,00	0,00	1
1	97	218	2,25	5740,90	1
1	185	472	2,55	4389,73	1
1	159	370	2,33	2190,75	1
1	0	0	0,00	0,00	1
1	277	578	2,09	4587,19	1
1	218	461	2,11	3602,31	1
1	332	943	2,84	6976,13	1
1	212	562	2,65	4075,15	1
1	182	474	2,60	4738,37	1
1	263	732	2,78	2794,09	1
1	199	605	3,04	6944,11	1
1	181	522	2,88	4892,04	1
1	398	969	2,43	5335,43	1
1	190	497	2,62	3113,92	1
1	108	284	2,63	5968,52	1
1	145	349	2,41	5329,22	1
1	278	663	2,38	4387,78	1
1	257	756	2,94	7790,30	1
1	227	545	2,40	6179,75	1
1	104	225	2,16	6142,12	1
1	224	468	2,09	5589,87	1
1	217	444	2,05	7347,13	1
1	189	457	2,42	11158,50	1
1	220	458	2,08	9350,43	1
1	180	466	2,59	6364,37	1
1	301	683	2,27	7572,73	1
1	337	718	2,13	5032,38	1
1	115	393	3,42	1028,29	1
1	209	800	3,83	731,65	1

1	179	645	3,60	741,84	1
1	236	832	3,53	689,63	1
1	28	127	4,54	579,57	1
1	234	751	3,21	715,12	1
1	151	487	3,23	771,70	1
1	189	684	3,62	642,68	1
1	180	555	3,08	11837,06	1
1	48	158	3,29	855,75	1
1	153	562	3,67	849,08	1
1	240	898	3,74	692,16	1
1	138	468	3,39	639,50	1
1	213	750	3,52	597,15	1
1	242	905	3,74	590,67	1
1	182	698	3,84	703,68	1
1	170	633	3,72	615,03	1
1	250	850	3,40	551,94	1
1	137	523	3,82	677,19	1
1	81	382	4,72	569,93	1
1	119	397	3,34	678,50	1
1	103	422	4,10	516,43	1
1	173	584	3,38	752,92	1
1	269	981	3,65	544,26	1
1	93	311	3,34	639,95	1
1	76	278	3,66	574,91	1
1	142	523	3,68	638,13	1
1	184	698	3,79	875,43	1
1	106	400	3,77	642,44	1
1	152	582	3,83	644,51	1
1	303	1044	3,45	863,14	1
1	264	871	3,30	764,45	1
1	165	575	3,48	1191,27	1
1	192	689	3,59	610,81	1
1	80	271	3,39	742,55	1
1	113	413	3,65	941,74	1
1	167	657	3,93	695,63	1
1	112	346	3,09	833,59	1
1	224	708	3,16	743,87	1
1	98	322	3,29	777,19	1
1	280	936	3,34	732,96	1
1	190	658	3,46	820,17	1
1	116	410	3,53	747,41	1
1	272	978	3,60	677,74	1
1	284	967	3,40	832,10	1
1	311	1173	3,77	590,98	1
1	94	332	3,53	13008,09	1
1	230	816	3,55	9810,47	1
1	154	422	2,74	7470,32	1
1	229	653	2,85	8234,50	1
1	310	598	1,93	4455,30	1
1	239	614	2,57	6799,80	1

1	253	608	2,40	4369,84	1
1	246	725	2,95	6953,53	1
1	268	782	2,92	8621,32	1
1	113	301	2,66	3409,12	1
1	314	736	2,34	4333,77	1
1	281	747	2,66	5582,92	1
1	418	1162	2,78	8339,99	1
1	92	201	2,18	4989,35	1
1	166	444	2,67	9927,90	1
1	193	547	2,83	8361,17	1
1	156	415	2,66	5428,20	1
1	276	783	2,84	7376,83	1
1	227	584	2,57	4745,91	1
1	154	400	2,60	4640,36	1
1	184	512	2,78	6524,97	1
1	180	381	2,12	10553,72	1
1	320	859	2,68	10052,68	1
1	20	47	2,35	7055,00	1
1	246	519	2,11	4337,44	1
1	64	147	2,30	3701,63	1
1	202	469	2,32	6352,68	1
1	160	284	1,78	2589,51	1
1	190	445	2,34	3282,22	1
1	19	78	4,11	10604,95	1
1	146	320	2,19	1933,71	1
1	124	244	1,97	2059,32	1
1	240	441	1,84	2072,15	1
1	263	544	2,07	2983,73	1
1	187	384	2,05	2720,85	1
1	249	649	2,61	2736,48	1
1	272	681	2,50	3120,63	1
1	269	733	2,72	3256,28	1
1	233	575	2,47	3591,78	1
1	189	451	2,39	2957,15	1
1	239	700	2,93	2492,92	1
1	215	556	2,59	2207,44	1
1	44	130	2,95	2686,36	1
1	241	563	2,34	3922,57	1
1	232	560	2,41	3786,42	1
1	182	392	2,15	3062,56	1
1	212	533	2,51	3457,50	1
1	195	555	2,85	7941,79	1
1	252	726	2,88	11429,88	1
1	179	488	2,73	6691,35	1
1	241	700	2,90	6840,46	1
1	283	783	2,77	6888,24	1
1	178	509	2,86	6393,77	1
1	216	576	2,67	4873,38	1
1	285	778	2,73	4772,74	1
1	271	682	2,52	5525,38	1

1	235	659	2,80	4264,85	1
1	257	696	2,71	8326,25	1
1	245	645	2,63	7019,52	1
1	229	629	2,75	8768,59	1
1	326	808	2,48	8537,55	1
1	306	845	2,76	12146,60	1
1	190	597	3,14	12727,79	1
1	199	464	2,33	2861,48	1
1	225	443	1,97	3453,66	1
1	345	666	1,93	4691,94	1
1	133	274	2,06	4979,17	1
1	242	551	2,28	3597,38	1
1	265	599	2,26	3721,04	1
1	153	319	2,08	2597,83	1
1	174	442	2,54	4053,72	1
1	231	581	2,52	4674,12	1
1	126	308	2,44	3292,78	1
1	215	534	2,48	2480,89	1
1	185	409	2,21	1768,64	1
1	203	494	2,43	2872,31	1
1	371	653	1,76	2518,37	1
1	252	617	2,45	7899,36	1
1	256	734	2,87	8855,14	1
1	279	740	2,65	6420,83	1
1	271	712	2,63	6444,92	1
1	262	770	2,94	7441,42	1
1	284	724	2,55	6981,41	1
1	270	788	2,92	6326,61	1
1	216	582	2,69	6880,56	1
1	233	639	2,74	7093,45	1
1	287	776	2,70	5138,68	1
1	221	601	2,72	5571,57	1
1	202	566	2,80	4795,43	1
1	246	654	2,66	4022,28	1
1	313	773	2,47	5389,09	1
1	296	756	2,55	6161,45	1
1	364	922	2,53	5526,62	1
1	243	583	2,40	6651,89	1
1	184	441	2,40	8106,30	1
1	283	804	2,84	10046,57	1
1	226	717	3,17	13595,81	1
1	182	530	2,91	11608,79	1
1	215	557	2,59	8896,51	1
1	278	683	2,46	6483,52	1
1	205	401	1,96	4949,12	1
1	247	632	2,56	8057,81	1
1	128	299	2,34	5882,34	1
1	215	577	2,68	2855,07	1
1	154	274	1,78	2299,41	1
1	251	498	1,98	2256,36	1

1	280	638	2,28	2193,23	1
1	234	505	2,16	2514,91	1
1	191	391	2,05	2100,41	1
1	247	845	3,42	13940,72	1
1	193	602	3,12	9451,04	1
1	272	735	2,70	8283,39	1
1	242	664	2,74	5995,42	1
1	385	1141	2,96	9186,59	1
1	203	626	3,08	8867,95	1
1	228	511	2,24	6697,37	1
0	0	0	0,00	0,00	0
1	387	1061	2,74	7106,11	1
1	288	704	2,44	5321,66	1
1	282	630	2,23	5279,12	1
1	331	859	2,60	6203,95	1
1	104	254	2,44	5015,91	1
1	202	496	2,46	5452,19	1
1	311	770	2,48	6377,60	1
1	231	607	2,63	5315,15	1
1	234	626	2,68	5943,51	1
1	279	777	2,78	8264,36	1
1	240	668	2,78	10386,41	1
1	349	993	2,85	10363,49	1
1	242	692	2,86	12538,26	1
1	369	841	2,28	8986,18	1
1	147	415	2,82	9971,73	1
1	110	294	2,67	8907,15	1
1	152	331	2,18	5018,31	1
1	223	368	1,65	2713,77	1
1	228	407	1,79	2729,75	1
1	169	256	1,51	1901,54	1
1	174	257	1,48	1930,76	1
1	140	277	1,98	2964,16	1
1	229	454	1,98	2510,39	1
1	276	457	1,66	1952,85	1
1	224	499	2,23	3170,18	1
1	243	556	2,29	2452,21	1
1	251	760	3,03	8807,65	1
1	254	935	3,68	618,11	1
1	136	494	3,63	759,83	1
1	278	1054	3,79	705,69	1
1	86	319	3,71	707,45	1
1	186	701	3,77	1452,60	1
1	150	531	3,54	653,61	1
1	238	896	3,76	827,49	1
1	188	666	3,54	583,56	1
1	97	363	3,74	1005,67	1
1	330	1127	3,42	1486,95	1
1	240	845	3,52	1398,40	1
1	418	1336	3,20	686,21	1

1	266	980	3,68	747,26	1
1	303	1029	3,40	1464,97	1
1	213	823	3,86	1159,50	1
1	237	801	3,38	1624,41	1
1	282	986	3,50	927,03	1
1	53	183	3,45	776,77	1
1	311	1138	3,66	817,34	1
1	118	353	2,99	6998,11	1
1	204	542	2,66	6923,92	1
1	310	788	2,54	5371,05	1
1	155	405	2,61	5565,94	1
1	325	886	2,73	6453,38	1
1	230	583	2,53	6546,57	1
1	316	866	2,74	6638,04	1
1	336	890	2,65	5552,38	1
1	205	470	2,29	7064,10	1
1	324	883	2,73	5946,68	1
1	296	565	1,91	5663,71	1
1	223	515	2,31	4407,93	1
1	199	465	2,34	5214,42	1
1	193	482	2,50	7411,84	1
1	208	613	2,95	8147,16	1
1	117	289	2,47	6924,62	1
1	243	697	2,87	5920,73	1
1	210	536	2,55	5766,81	1
1	207	377	1,82	3157,55	1
1	146	304	2,08	2756,60	1
1	160	395	2,47	2874,07	1
1	135	298	2,21	3611,94	1
1	290	560	1,93	2111,09	1
1	187	566	3,03	1850,12	1
1	0	0	0,00	0,00	1
1	286	982	3,43	7146,50	1
1	247	751	3,04	6787,43	1
1	168	503	2,99	5671,44	1
1	286	836	2,92	8017,09	1
1	92	306	3,33	17244,57	1
1	290	889	3,07	5253,92	1
1	258	743	2,88	8953,64	1
1	119	336	2,82	4760,15	1
1	299	876	2,93	7984,76	1
1	217	555	2,56	4791,47	1
1	207	582	2,81	7245,77	1
1	188	490	2,61	6346,66	1
1	92	233	2,53	3846,96	1
1	189	497	2,63	6287,09	1
1	212	525	2,48	4264,92	1
1	205	540	2,63	4221,75	1
1	217	492	2,27	4346,35	1
1	170	485	2,85	7333,95	1

1	191	546	2,86	8667,23	1
1	193	551	2,85	5880,14	1
1	269	834	3,10	9682,29	1
1	121	382	3,16	5927,42	1
1	0	0	0,00	0,00	1
1	239	627	2,62	3997,46	1
0	0	0	0,00	0,00	0
1	293	990	3,38	10516,62	1
1	172	559	3,25	18096,40	1
1	356	1160	3,26	20342,57	1
1	341	844	2,48	10638,66	1
1	329	795	2,42	10830,36	1
1	242	646	2,67	5474,59	1
1	197	609	3,09	6556,50	1
1	151	577	3,82	9767,38	1
1	304	1087	3,58	8290,59	1
1	1	1	1,00	1020,00	1
1	278	768	2,76	6728,88	1
1	257	688	2,68	5666,97	1
1	396	1071	2,70	9086,06	1
1	357	1126	3,15	8320,08	1
1	230	594	2,58	6655,78	1
1	122	365	2,99	9569,32	1
1	132	362	2,74	4900,27	1
1	211	585	2,77	5895,62	1
1	303	772	2,55	5548,22	1
1	237	719	3,03	8176,92	1
1	323	1111	3,44	769,92	1
1	109	296	2,72	6001,55	1
1	152	358	2,36	7170,99	1
1	147	342	2,33	6917,25	1
1	230	658	2,86	10011,83	1
1	247	573	2,32	6457,06	1
1	147	388	2,64	3543,40	1
1	182	523	2,87	7789,18	1
1	182	367	2,02	5372,70	1
1	197	433	2,20	5320,40	1
1	163	442	2,71	4073,84	1
1	171	440	2,57	7005,33	1
1	177	560	3,16	7165,22	1
1	226	578	2,56	7886,43	1
1	206	539	2,62	6909,47	1
1	137	355	2,59	3475,76	1
1	164	466	2,84	7944,46	1
1	175	383	2,19	7309,31	1
1	126	366	2,90	6553,48	1
1	124	293	2,36	5913,87	1
1	169	328	1,94	2255,85	1
1	191	577	3,02	7490,13	1
1	224	610	2,72	10039,91	1

1	202	431	2,13	6612,14	1
1	136	321	2,36	3856,69	1
1	261	729	2,79	5860,82	1
1	314	870	2,77	11558,19	1
1	211	568	2,69	13990,53	1
1	177	533	3,01	16389,27	1
1	198	452	2,28	7095,24	1
1	68	205	3,01	7251,62	1
1	139	339	2,44	5652,99	1
1	103	257	2,50	5209,93	1
1	221	598	2,71	9948,05	1
1	294	649	2,21	9359,69	1
1	83	160	1,93	4847,11	1
1	241	693	2,88	3545,41	1
1	242	710	2,93	5015,54	1
1	150	453	3,02	15009,54	1
1	312	928	2,97	18589,79	1
1	124	270	2,18	4559,98	1
1	141	401	2,84	8048,14	1
1	133	360	2,71	5346,99	1
1	40	104	2,60	6083,65	1
1	333	924	2,77	8528,03	1
1	122	329	2,70	5111,32	1
1	285	636	2,23	4877,32	1
1	64	206	3,22	5222,34	1
1	176	469	2,66	6048,77	1
1	61	172	2,82	6024,85	1
1	128	416	3,25	9193,83	1
1	227	648	2,85	8380,51	1
1	216	480	2,22	5113,54	1
1	11	39	3,55	4212,55	1
1	31	81	2,61	6326,16	1
1	61	143	2,34	9053,11	1
1	38	95	2,50	7865,39	1
1	159	504	3,17	11998,18	1
1	152	426	2,80	8734,34	1
0	0	0	0,00	0,00	0
1	71	157	2,21	3676,20	1
1	104	301	2,89	4177,35	1
1	51	160	3,14	5447,47	1
1	105	318	3,03	4167,20	1
1	207	509	2,46	7448,00	1
1	68	160	2,35	3906,47	1
1	46	156	3,39	10514,13	1
1	43	51	1,19	5852,09	1
1	143	394	2,76	7061,38	1
1	46	148	3,22	5352,17	1
1	81	303	3,74	12561,93	1
0	0	0	0,00	0,00	0
1	44	141	3,20	8134,09	1

1	131	486	3,71	718,09	1
1	66	151	2,29	2573,18	1
1	29	111	3,83	9596,59	1
0	0	0	0,00	0,00	0
1	89	323	3,63	554,72	1
1	150	373	2,49	4531,18	1
1	36	119	3,31	870,92	1
1	54	199	3,69	585,57	1
1	195	524	2,69	3934,69	1
1	73	251	3,44	708,73	1
1	116	453	3,91	739,04	1
1	212	715	3,37	14396,56	1
0	0	0	0,00	0,00	0
1	166	583	3,51	630,61	1
1	185	747	4,04	613,11	1
1	80	289	3,61	12077,03	1
1	87	323	3,71	8419,54	1
1	130	256	1,97	4234,85	1
1	61	212	3,48	484,77	1
1	102	350	3,43	787,37	1
1	114	405	3,55	836,06	1
1	352	1127	3,20	1009,33	1
1	104	271	2,61	5166,22	1
1	228	498	2,18	3733,84	1
1	14	34	2,43	2757,86	1
1	157	402	2,56	5656,89	1
1	59	192	3,25	8045,93	1
1	39	90	2,31	4194,87	1
1	142	418	2,94	2513,20	1
1	37	139	3,76	900,54	1
1	22	72	3,27	1011,82	1
1	46	153	3,33	1021,91	1
1	18	60	3,33	1946,67	1
1	8	29	3,63	9861,75	1
1	195	541	2,77	6355,66	1
1	284	735	2,59	6965,69	1
1	141	435	3,09	7215,12	1
1	147	414	2,82	5408,63	1
1	189	448	2,37	4933,28	1
1	67	223	3,33	13198,51	1
1	184	559	3,04	11869,51	1
1	257	786	3,06	2618,89	1
1	287	853	2,97	3156,01	1
1	192	673	3,51	1032,93	1
1	306	869	2,84	2322,57	1
1	156	451	2,89	2851,18	1
1	255	778	3,05	2431,90	1
1	200	609	3,05	2577,66	1
1	183	627	3,43	962,22	1
1	134	464	3,46	1546,21	1

1	238	732	3,08	1706,99	1
1	30	89	2,97	1886,37	1
1	0	0	0,00	0,00	1
1	196	635	3,24	1327,90	1
1	254	881	3,47	1310,48	1
1	195	685	3,51	1384,93	1
1	311	1036	3,33	1250,76	1
1	304	963	3,17	1244,12	1
1	272	932	3,43	959,21	1
1	250	809	3,24	1394,91	1
1	191	612	3,20	1796,94	1
1	265	839	3,17	1495,62	1
1	196	683	3,48	1339,59	1
1	200	638	3,19	837,50	1
1	236	760	3,22	1741,32	1
1	213	629	2,95	1980,60	1
1	250	732	2,93	3933,06	1
1	249	652	2,62	3130,80	1
1	179	480	2,68	3067,02	1
1	318	951	2,99	2268,66	1
1	206	624	3,03	3344,23	1
1	183	526	2,87	2440,39	1
1	261	695	2,66	2345,98	1
1	350	954	2,73	2498,56	1
1	269	850	3,16	1392,87	1
1	184	619	3,36	1512,47	1
1	163	529	3,25	1451,19	1
1	174	451	2,59	1845,95	1
1	206	646	3,14	2708,48	1
1	296	853	2,88	1618,98	1
1	235	692	2,94	1621,41	1
1	156	467	2,99	1366,46	1
1	189	630	3,33	1244,33	1
1	173	523	3,02	1536,20	1
1	230	745	3,24	1573,85	1
1	207	738	3,57	930,32	1
1	283	943	3,33	959,02	1
1	197	668	3,39	664,32	1
1	292	963	3,30	1976,57	1
1	168	515	3,07	1401,49	1
1	151	537	3,56	1570,66	1
1	258	851	3,30	1408,76	1
1	147	498	3,39	1029,19	1
1	166	515	3,10	1603,64	1
1	276	818	2,96	3265,07	1
1	151	456	3,02	2556,17	1
1	306	815	2,66	3176,23	1
1	185	388	2,10	3595,51	1
1	289	747	2,58	3380,17	1
1	260	656	2,52	2330,60	1

1	206	436	2,12	2634,85	1
1	304	859	2,83	3357,09	1
1	279	774	2,77	2890,40	1
1	244	693	2,84	1980,46	1
1	322	909	2,82	2599,61	1
1	304	837	2,75	2744,18	1
1	173	513	2,97	3169,61	1
1	127	377	2,97	3569,61	1
1	267	934	3,50	1614,70	1
1	70	217	3,10	2020,00	1
1	253	830	3,28	2004,59	1
1	245	795	3,24	2397,84	1
1	415	1014	2,44	2160,98	1
1	304	959	3,15	1599,52	1
1	283	889	3,14	1621,00	1
1	240	737	3,07	1953,43	1
1	336	1108	3,30	1251,36	1
1	150	464	3,09	2270,63	1
1	186	583	3,13	1286,67	1
1	256	955	3,73	535,63	1
1	148	516	3,49	1201,49	1
1	236	824	3,49	949,43	1
1	240	782	3,26	935,09	1
1	315	1101	3,50	956,19	1
1	305	1033	3,39	970,94	1
1	195	571	2,93	916,18	1
1	242	742	3,07	1737,85	1
1	148	495	3,34	1671,12	1
1	218	682	3,13	2532,92	1
1	154	503	3,27	1983,38	1
1	127	446	3,51	841,79	1
1	184	578	3,14	2333,75	1
1	287	728	2,54	3031,69	1
1	180	484	2,69	2758,72	1
1	250	662	2,65	3248,07	1
1	252	744	2,95	3353,44	1
1	277	811	2,93	3122,19	1
1	313	822	2,63	4180,78	1
1	210	530	2,52	3568,08	1
1	300	787	2,62	3031,57	1
1	244	603	2,47	2610,45	1
1	231	605	2,62	2834,98	1
1	283	843	2,98	2445,56	1
1	306	866	2,83	4657,26	1
1	165	474	2,87	4244,36	1
1	281	836	2,98	2035,67	1
1	127	375	2,95	2284,50	1
1	183	589	3,22	1181,75	1
1	236	834	3,53	890,22	1
1	189	701	3,71	571,27	1

1	186	642	3,45	1096,54	1
1	252	862	3,42	692,87	1
1	225	837	3,72	769,94	1
1	318	996	3,13	804,01	1
1	260	790	3,04	1519,56	1
1	186	587	3,16	1227,12	1
1	243	739	3,04	1736,48	1
1	264	873	3,31	1979,52	1
1	181	578	3,19	2006,62	1
1	246	600	2,44	2069,61	1
1	187	472	2,52	3696,00	1
1	409	1149	2,81	3101,47	1
1	231	760	3,29	823,55	1
1	257	711	2,77	2925,05	1
1	214	569	2,66	2712,89	1
1	295	830	2,81	3276,65	1
1	270	752	2,79	2625,71	1
1	278	745	2,68	2838,64	1
1	192	556	2,90	4778,53	1
1	225	594	2,64	3041,86	1
1	312	930	2,98	2443,14	1
1	181	489	2,70	3318,43	1
1	364	1066	2,93	2538,89	1
1	205	606	2,96	2917,63	1
1	356	1095	3,08	2017,20	1
1	255	822	3,22	1529,18	1
1	183	558	3,05	1616,80	1
1	267	931	3,49	2135,60	1
1	315	1023	3,25	1368,06	1
1	369	1254	3,40	1350,37	1
1	278	917	3,30	1275,32	1
1	271	872	3,22	1061,73	1
1	195	653	3,35	1047,23	1
1	255	788	3,09	1207,14	1
1	113	399	3,53	810,94	1
1	158	559	3,54	780,91	1
1	300	1085	3,62	745,75	1
1	220	783	3,56	928,11	1
1	253	845	3,34	1134,34	1
1	236	833	3,53	894,83	1
1	158	568	3,59	624,67	1
1	131	486	3,71	705,96	1
1	249	838	3,37	669,02	1
1	168	578	3,44	897,84	1
1	253	892	3,53	811,76	1
1	116	380	3,28	804,42	1
1	261	859	3,29	1770,17	1
1	222	705	3,18	1089,40	1
1	245	726	2,96	2491,39	1
1	188	576	3,06	2604,69	1

1	145	451	3,11	1815,58	1
1	225	618	2,75	2191,12	1
1	267	662	2,48	2036,47	1
1	256	734	2,87	1641,22	1
1	157	439	2,80	2266,18	1
1	367	1025	2,79	2427,55	1
1	241	601	2,49	4304,90	1
1	299	999	3,34	868,62	1
1	185	485	2,62	2284,43	1
1	149	505	3,39	630,99	1
1	256	743	2,90	3630,70	1
1	209	512	2,45	3866,03	1
1	131	326	2,49	2657,66	1
1	81	246	3,04	3374,22	1
1	152	432	2,84	3099,62	1
1	264	768	2,91	3368,57	1
1	277	775	2,80	2987,54	1
1	262	706	2,69	3172,62	1
1	160	395	2,47	2498,63	1
1	173	494	2,86	2946,40	1
1	390	1074	2,75	3923,84	1
1	299	878	2,94	3771,27	1
1	249	772	3,10	4827,12	1
1	248	735	2,96	2998,88	1
1	198	560	2,83	3750,70	1
1	135	436	3,23	1312,89	1
1	243	810	3,33	1433,00	1
1	297	911	3,07	1898,11	1
1	327	1055	3,23	984,58	1
1	151	526	3,48	3128,14	1
1	4	10	2,50	791,50	1
1	412	1155	2,80	602,76	1
1	311	1047	3,37	740,60	1
1	312	1049	3,36	1412,63	1
1	174	542	3,11	2080,55	1
1	277	851	3,07	1116,82	1
1	289	830	2,87	2974,73	1
1	211	413	1,96	2097,35	1
1	244	579	2,37	2756,44	1
1	245	639	2,61	2795,00	1
1	275	759	2,76	3238,93	1
1	284	839	2,95	3590,27	1
1	182	455	2,50	2377,09	1
1	176	445	2,53	3127,77	1
1	185	552	2,98	3108,24	1
1	144	424	2,94	2784,89	1
1	196	531	2,71	2543,34	1
1	180	554	3,08	2436,56	1
1	318	874	2,75	2828,74	1
1	173	540	3,12	2449,32	1

1	414	1238	2,99	2258,71	1
1	199	645	3,24	1007,82	1
1	166	522	3,14	867,93	1
1	352	1241	3,53	1630,35	1
1	129	411	3,19	887,16	1
1	178	509	2,86	4596,26	1
1	171	557	3,26	1755,65	1
1	322	890	2,76	3130,03	1
1	316	847	2,68	3226,38	1
1	223	562	2,52	1863,44	1
1	245	690	2,82	2791,79	1
1	343	1088	3,17	607,70	1
1	227	672	2,96	4501,06	1
1	266	646	2,43	2745,92	1
1	217	568	2,62	3537,83	1
1	201	516	2,57	3734,96	1
1	142	411	2,89	4045,00	1
1	341	875	2,57	3439,90	1
1	197	506	2,57	2465,75	1
1	247	696	2,82	3220,63	1
1	230	665	2,89	3549,32	1
1	202	616	3,05	4830,01	1
1	225	640	2,84	3438,17	1
1	234	641	2,74	3221,61	1
1	264	813	3,08	1256,06	1
1	505	2051	4,06	606,39	1
1	269	968	3,60	633,02	1
1	289	1098	3,80	811,76	1
1	36	111	3,08	5284,53	1
1	283	1044	3,69	639,41	1
1	226	922	4,08	585,46	1
1	165	744	4,51	550,75	1
1	313	1118	3,57	666,49	1
1	203	731	3,60	609,22	1
1	308	1120	3,64	437,25	1
1	310	1106	3,57	528,65	1
1	194	686	3,54	515,82	1
1	166	595	3,58	606,16	1
1	197	785	3,98	606,22	1
1	241	849	3,52	597,96	1
1	280	972	3,47	585,43	1
1	207	845	4,08	335,67	1
1	134	545	4,07	567,49	1
1	128	481	3,76	503,38	1
1	162	518	3,20	1493,89	1
1	65	188	2,89	1775,54	1
1	13	37	2,85	2586,15	1
1	113	327	2,89	1851,49	1
1	199	489	2,46	2439,42	1
1	176	478	2,72	3004,67	1

1	52	113	2,17	2127,23	1
1	80	213	2,66	3502,68	1
1	161	480	2,98	3272,33	1
1	132	403	3,05	3612,39	1
1	164	431	2,63	2945,98	1
1	156	484	3,10	2617,39	1
1	156	417	2,67	2695,74	1
1	167	497	2,98	2850,85	1
1	205	558	2,72	1687,13	1
1	137	406	2,96	2048,07	1
1	103	213	2,07	4010,20	1
1	211	581	2,75	2768,58	1
1	146	381	2,61	2976,10	1
1	195	488	2,50	2896,09	1
1	171	483	2,82	3422,61	1
1	217	623	2,87	1930,18	1
1	141	413	2,93	1401,66	1
1	118	306	2,59	2576,99	1
1	50	136	2,72	2272,60	1
1	39	121	3,10	2992,77	1
1	117	295	2,52	2614,13	1
1	257	708	2,75	2588,25	1
1	49	129	2,63	1600,00	1
1	143	311	2,17	3121,85	1
1	220	636	2,89	2252,50	1
1	165	510	3,09	2661,70	1
1	29	89	3,07	3675,52	1
1	151	503	3,33	846,46	1
1	19	59	3,11	1224,68	1
1	80	244	3,05	1058,45	1
1	67	179	2,67	1334,96	1
1	49	143	2,92	1124,00	1
1	148	447	3,02	2948,75	1
1	6	16	2,67	755,00	1
1	151	420	2,78	3613,94	1
0	0	0	0,00	0,00	0
1	107	387	3,62	694,86	1
1	57	236	4,14	763,72	1
1	68	261	3,84	719,25	1
1	99	379	3,83	1444,97	1
1	111	375	3,38	1883,20	1
0	0	0	0,00	0,00	0
1	31	122	3,94	871,13	1
1	202	673	3,33	984,32	1
1	60	195	3,25	1014,02	1
1	168	512	3,05	993,63	1
1	194	602	3,10	1585,87	1
1	176	599	3,40	1140,42	1
1	119	361	3,03	1297,98	1
1	197	527	2,68	2228,81	1

1	212	712	3,36	540,49	1
1	129	473	3,67	714,21	1
1	6	14	2,33	706,67	1
1	13	48	3,69	1038,46	1
1	33	112	3,39	1751,21	1
1	58	192	3,31	1686,60	1
1	193	637	3,30	1141,34	1
1	151	419	2,77	1373,94	1
1	163	534	3,28	1178,49	1
1	107	370	3,46	504,94	1
1	4	13	3,25	927,50	1
1	104	366	3,52	1254,30	1
1	62	222	3,58	869,68	1
1	185	572	3,09	1368,87	1
1	11	40	3,64	1249,09	1
1	242	746	3,08	3502,08	1
1	150	492	3,28	1275,79	1
1	49	150	3,06	1087,45	1
1	126	573	4,55	739,51	1
1	172	599	3,48	915,20	1
1	115	371	3,23	831,17	1
1	240	815	3,40	687,87	1
1	92	268	2,91	1072,35	1
1	58	197	3,40	1569,09	1
1	110	414	3,76	623,27	1
1	17	43	2,53	873,06	1
1	450	1083	2,41	3051,80	1
1	89	283	3,18	1332,73	1
1	90	254	2,82	2127,04	1
1	121	385	3,18	1343,59	1
1	53	182	3,43	760,42	1
1	19	52	2,74	1396,53	1
1	163	612	3,75	517,64	1
1	160	599	3,74	1171,48	1
1	109	359	3,29	1272,45	1
1	221	729	3,30	1198,61	1
1	181	630	3,48	631,90	1
1	124	461	3,72	668,69	1
1	46	158	3,43	1045,43	1
1	185	529	2,86	2587,15	1
1	170	526	3,09	2104,72	1
1	189	698	3,69	763,42	1
1	62	186	3,00	923,71	1
1	137	488	3,56	790,94	1
1	53	172	3,25	2313,49	1
1	29	109	3,76	926,03	1
1	244	821	3,36	1290,16	1
1	112	247	2,21	2555,14	1
1	40	126	3,15	1854,18	1
1	101	184	1,82	2861,90	1

1	324	750	2,31	4325,02	1
1	241	721	2,99	3400,17	1
1	233	676	2,90	3105,91	1
1	167	524	3,14	1097,44	1
1	16	62	3,88	6690,63	1
1	3	7	2,33	806,67	1
1	249	896	3,60	486,33	1
1	47	161	3,43	1630,85	1
1	173	551	3,18	2278,23	1
1	180	561	3,12	810,54	1
1	215	673	3,13	846,40	1
1	172	606	3,52	749,84	1
1	139	396	2,85	3671,94	1
1	111	431	3,88	638,38	1
1	60	231	3,85	532,23	1
1	81	315	3,89	570,78	1
1	19	65	3,42	678,68	1
1	28	110	3,93	1231,07	1
1	109	463	4,25	488,81	1
1	197	787	3,99	619,63	1
1	85	340	4,00	652,22	1
1	236	677	2,87	1481,36	1
1	219	634	2,89	897,89	1
1	277	1011	3,65	1368,10	1
1	160	518	3,24	1005,17	1
1	49	155	3,16	1301,12	1
1	233	741	3,18	2800,56	1
1	206	602	2,92	1988,62	1
1	226	680	3,01	1269,96	1
1	294	879	2,99	1292,94	1
1	230	692	3,01	722,10	1
1	131	407	3,11	2449,95	1
1	241	743	3,08	3223,68	1
1	116	306	2,64	2755,39	1
1	237	704	2,97	3683,57	1
1	232	695	3,00	3674,72	1
1	172	602	3,50	560,99	1
1	221	590	2,67	3408,44	1
1	207	627	3,03	3891,81	1
1	3	14	4,67	2983,33	1
1	183	472	2,58	3578,41	1
1	177	582	3,29	1196,21	1
1	207	563	2,72	2032,59	1
1	293	914	3,12	2122,04	1
1	214	640	2,99	1965,95	1
1	306	965	3,15	1096,62	1
1	290	991	3,42	1140,95	1
1	283	926	3,27	1424,92	1
1	203	667	3,29	914,06	1
1	214	632	2,95	1027,21	1

1	194	610	3,14	1312,18	1
1	154	529	3,44	663,64	1
0	0	0	0,00	0,00	0
1	83	192	2,31	3028,31	1
1	268	789	2,94	4891,34	1
1	276	840	3,04	5387,99	1
1	195	564	2,89	3455,11	1
1	211	610	2,89	3362,72	1
1	210	722	3,44	1175,34	1
1	95	209	2,20	1691,42	1
1	100	331	3,31	1799,00	1
1	173	562	3,25	2166,99	1
1	272	877	3,22	1072,97	1
1	335	1100	3,28	1380,41	1
1	8	32	4,00	1792,75	1
1	334	1070	3,20	1107,46	1
1	219	641	2,93	1317,25	1
1	268	783	2,92	1381,93	1
1	21	84	4,00	855,48	1
1	277	882	3,18	1485,76	1
1	299	948	3,17	1099,17	1
1	165	494	2,99	1128,82	1
1	236	792	3,36	1392,54	1
1	227	803	3,54	1128,15	1
1	168	566	3,37	936,79	1
1	145	453	3,12	900,39	1
1	50	177	3,54	774,74	1
1	158	613	3,88	661,83	1
1	193	623	3,23	785,70	1
1	304	991	3,26	671,66	1
1	240	910	3,79	652,90	1
1	210	691	3,29	1067,02	1
1	307	1041	3,39	1289,36	1
1	165	595	3,61	574,84	1
1	235	758	3,23	852,88	1
1	187	622	3,33	1139,11	1
1	163	540	3,31	1724,96	1
1	159	549	3,45	712,74	1
1	174	593	3,41	595,61	1
1	118	454	3,85	442,99	1
1	174	574	3,30	1164,40	1
1	355	1249	3,52	581,14	1
1	276	901	3,26	1019,08	1
1	304	1046	3,44	873,03	1
1	284	901	3,17	937,52	1
1	121	530	4,38	558,79	1
1	125	476	3,81	542,06	1
1	109	317	2,91	4530,55	1
1	45	173	3,84	564,44	1
0	0	0	0,00	0,00	0

1	294	1076	3,66	578,31	1
1	106	390	3,68	587,87	1
1	254	910	3,58	652,78	1
1	149	574	3,85	628,89	1
1	83	263	3,17	581,60	1
1	120	442	3,68	647,77	1
1	175	617	3,53	703,17	1
1	268	783	2,92	2054,88	1
1	270	785	2,91	2608,72	1
1	156	470	3,01	2610,83	1
1	119	329	2,76	1751,68	1
1	336	903	2,69	2238,24	1
1	274	830	3,03	2949,96	1
1	193	515	2,67	6326,11	1
1	214	614	2,87	1442,01	1
1	192	599	3,12	2370,30	1
1	183	529	2,89	2509,22	1
1	309	882	2,85	2423,77	1
1	322	910	2,83	2084,70	1
1	280	806	2,88	1080,73	1
1	296	886	2,99	3538,27	1
1	214	661	3,09	2356,07	1
1	188	527	2,80	2001,82	1
1	167	510	3,05	2788,47	1
1	202	629	3,11	2180,74	1
1	285	936	3,28	1323,63	1
1	292	921	3,15	1036,36	1
1	305	926	3,04	1592,77	1
1	343	971	2,83	2166,32	1
1	327	990	3,03	1414,22	1
1	207	570	2,75	5034,04	1
1	320	877	2,74	4410,88	1
1	31	98	3,16	1410,65	1
1	190	567	2,98	2587,82	1
1	243	702	2,89	2547,03	1
1	125	374	2,99	1904,85	1
1	317	1027	3,24	1335,46	1
1	355	1092	3,08	1337,37	1
1	263	846	3,22	840,38	1
1	381	1082	2,84	2411,10	1
1	201	484	2,41	2357,98	1
1	196	451	2,30	2558,58	1
1	252	822	3,26	5856,57	1
1	283	865	3,06	4670,89	1
1	283	807	2,85	4656,05	1
1	169	530	3,14	4237,38	1
1	297	853	2,87	6236,27	1
1	139	451	3,24	5751,71	1
1	264	783	2,97	3430,41	1
1	206	606	2,94	2099,54	1

1	290	896	3,09	2013,34	1
1	305	987	3,24	2402,78	1
1	271	830	3,06	2258,97	1
1	316	912	2,89	1225,86	1
1	176	540	3,07	1931,94	1
1	268	808	3,01	1492,72	1
1	163	534	3,28	860,39	1
1	307	988	3,22	1147,38	1
1	254	825	3,25	1078,36	1
1	203	601	2,96	983,29	1
1	266	833	3,13	1451,45	1
1	254	857	3,37	1572,01	1
1	299	788	2,64	2651,48	1
1	296	799	2,70	4072,98	1
1	297	951	3,20	7373,41	1
1	243	794	3,27	8051,60	1
1	301	732	2,43	2158,80	1
1	266	703	2,64	3932,68	1
1	223	633	2,84	4046,81	1
1	297	872	2,94	4083,99	1
1	202	604	2,99	5437,46	1
1	223	687	3,08	4015,54	1
1	318	1002	3,15	6019,46	1
1	341	970	2,84	3238,26	1
1	289	824	2,85	3840,81	1
1	174	461	2,65	2800,82	1
1	274	878	3,20	1652,77	1
1	209	614	2,94	1059,74	1
1	98	321	3,28	1098,67	1
1	177	585	3,31	1433,97	1
1	288	835	2,90	1776,84	1
1	180	698	3,88	830,38	1
1	315	1032	3,28	1257,74	1
1	289	945	3,27	1399,43	1
1	394	1213	3,08	5141,95	1
1	539	1581	2,93	3775,51	1
1	334	1055	3,16	1333,71	1
1	295	972	3,29	1357,19	1
1	266	882	3,32	1772,11	1
1	320	1065	3,33	4610,02	1
1	307	957	3,12	2699,12	1
1	322	1013	3,15	5082,04	1
1	319	1013	3,18	5839,98	1
1	277	840	3,03	6105,99	1
1	153	453	2,96	6641,17	1
1	285	857	3,01	2692,31	1
1	149	467	3,13	1960,28	1
1	262	770	2,94	1740,29	1
1	240	752	3,13	1896,24	1
1	255	775	3,04	1149,65	1

1	266	878	3,30	1232,95	1
1	229	716	3,13	1397,50	1
1	243	800	3,29	634,79	1
1	251	861	3,43	627,72	1
1	161	514	3,19	1805,83	1
1	157	492	3,13	1842,83	1
1	293	894	3,05	1301,70	1
1	154	501	3,25	1517,15	1
1	281	827	2,94	1170,81	1
1	219	718	3,28	1343,44	1
1	210	708	3,37	1697,39	1
1	187	642	3,43	1381,09	1
1	39	133	3,41	1309,72	1
1	262	869	3,32	2171,35	1
1	301	1033	3,43	1786,14	1
1	278	862	3,10	1093,05	1
1	326	876	2,69	2918,56	1
1	342	935	2,73	1156,21	1
1	276	941	3,41	847,92	1
1	312	936	3,00	1287,86	1
1	167	561	3,36	1455,78	1
1	295	963	3,26	1727,37	1
1	232	719	3,10	2391,44	1
1	293	859	2,93	3992,72	1
1	127	332	2,61	1427,91	1
1	211	643	3,05	2782,09	1
1	294	610	2,07	2253,13	1
1	51	164	3,22	1593,33	1
1	99	356	3,60	592,58	1
1	287	941	3,28	878,02	1
1	287	895	3,12	2062,18	1
1	298	996	3,34	1568,86	1
1	261	657	2,52	2865,80	1
1	201	574	2,86	2039,73	1
1	266	772	2,90	1825,78	1
1	157	439	2,80	1570,06	1
1	295	850	2,88	2827,94	1
1	75	223	2,97	1888,53	1
1	111	315	2,84	1861,01	1
1	201	590	2,94	3002,81	1
0	0	0	0,00	0,00	0
1	215	791	3,68	595,67	1
0	0	0	0,00	0,00	0
1	241	743	3,08	1526,29	1
1	175	563	3,22	1608,46	1
1	306	933	3,05	1471,27	1
1	322	903	2,80	3882,21	1
1	262	790	3,02	2341,04	1
1	532	1437	2,70	2757,96	1
1	45	129	2,87	3089,11	1

1	185	591	3,19	2058,82	1
1	310	1045	3,37	1529,00	1
1	44	157	3,57	1241,14	1
1	286	854	2,99	1374,97	1
1	283	689	2,43	2266,69	1
1	263	757	2,88	2873,04	1
1	187	483	2,58	2265,48	1
1	309	806	2,61	2196,68	1
1	132	407	3,08	1204,00	1
1	210	698	3,32	1036,99	1
1	247	819	3,32	1098,04	1
1	230	709	3,08	1780,19	1
1	245	713	2,91	3574,85	1
1	191	494	2,59	5389,88	1
1	130	382	2,94	2651,94	1
1	68	184	2,71	4066,59	1
1	231	695	3,01	2504,86	1
1	84	234	2,79	2095,38	1
1	60	176	2,93	1678,73	1
1	169	472	2,79	2098,14	1
1	208	530	2,55	3597,93	1
1	144	443	3,08	4272,10	1
1	95	270	2,84	3831,58	1
1	72	178	2,47	2713,08	1
1	157	491	3,13	4823,07	1
1	179	534	2,98	4853,58	1
1	200	701	3,51	595,09	1
1	162	475	2,93	1496,98	1
1	27	79	2,93	701,85	1
1	75	219	2,92	1813,20	1
1	62	186	3,00	728,61	1
1	286	766	2,68	2371,77	1
1	156	546	3,50	1752,95	1
1	0	0	0,00	0,00	1
1	130	319	2,45	2688,40	1
1	145	363	2,50	1986,74	1
1	59	142	2,41	3924,29	1
1	98	273	2,79	2401,88	1
1	291	798	2,74	5754,32	1
1	34	109	3,21	1456,47	1
1	124	305	2,46	1802,84	1
1	38	121	3,18	2054,47	1
1	35	108	3,09	5674,57	1
1	153	437	2,86	3151,33	1
1	93	280	3,01	2883,41	1
1	156	513	3,29	697,06	1
1	210	601	2,86	3461,34	1
1	79	254	3,22	4517,09	1
1	95	285	3,00	5086,87	1
1	67	202	3,01	3567,79	1

1	190	605	3,18	5120,90	1
1	178	538	3,02	1937,87	1
1	189	489	2,59	1317,49	1
1	48	150	3,13	1529,38	1
1	40	142	3,55	956,50	1
1	47	142	3,02	1888,13	1
1	58	163	2,81	2326,33	1
1	159	462	2,91	1168,69	1
1	76	226	2,97	1770,97	1
1	15	45	3,00	3515,33	1
1	12	39	3,25	1358,33	1
0	0	0	0,00	0,00	0
1	144	433	3,01	1500,42	1
1	180	547	3,04	2666,56	1
1	10	32	3,20	2808,50	1
1	236	635	2,69	1982,62	1
1	87	252	2,90	4169,33	1
1	141	462	3,28	606,49	1
1	108	279	2,58	3219,57	1
1	142	465	3,27	3765,01	1
1	237	777	3,28	977,19	1
1	152	520	3,42	981,02	1
1	180	568	3,16	4521,18	1
0	0	0	0,00	0,00	0
1	12	40	3,33	2776,67	1
1	89	316	3,55	2190,44	1
1	266	971	3,65	674,79	1
1	217	728	3,35	854,61	1
1	47	147	3,13	985,49	1
1	25	73	2,92	2571,04	1
1	180	496	2,76	2028,37	1
1	245	640	2,61	1779,50	1
1	295	772	2,62	2177,09	1
1	259	808	3,12	2436,72	1
1	226	673	2,98	3328,15	1
1	267	758	2,84	2562,70	1
1	199	509	2,56	3260,13	1
1	247	726	2,94	2989,47	1
1	310	864	2,79	2740,15	1
1	0	0	0,00	0,00	1
1	337	936	2,78	2361,54	1
1	246	692	2,81	3417,22	1
1	212	666	3,14	4596,61	1
1	154	511	3,32	1742,50	1
1	210	666	3,17	1863,28	1
1	249	800	3,21	1764,13	1
1	207	624	3,01	2301,06	1
1	140	448	3,20	798,89	1
1	229	756	3,30	675,47	1
1	384	1148	2,99	1402,46	1

1	1	2	2,00	2400,00	1
1	144	348	2,42	2042,75	1
1	163	478	2,93	1921,94	1
1	193	556	2,88	1909,12	1
1	279	837	3,00	1375,49	1
1	326	1052	3,23	964,28	1
1	240	734	3,06	988,97	1
1	290	931	3,21	1677,76	1
1	188	590	3,14	1856,77	1
1	261	801	3,07	1337,47	1
1	192	597	3,11	1253,01	1
1	293	872	2,98	802,10	1
1	333	1065	3,20	1029,76	1
1	281	887	3,16	1023,33	1
1	215	664	3,09	902,92	1
1	312	1049	3,36	1084,22	1
1	179	562	3,14	1345,90	1
1	168	490	2,92	1297,01	1
1	249	745	2,99	1468,84	1
1	258	765	2,97	1259,94	1
1	181	605	3,34	1657,22	1
1	224	563	2,51	2941,21	1
1	212	632	2,98	3577,04	1
1	252	847	3,36	1302,63	1
1	239	699	2,92	3564,13	1
1	108	345	3,19	3060,97	1
1	271	802	2,96	3172,31	1
1	179	509	2,84	3210,72	1
1	295	897	3,04	2864,93	1
1	258	751	2,91	2825,31	1
1	207	617	2,98	2376,37	1
1	153	494	3,23	2621,84	1
1	283	853	3,01	3091,90	1
1	277	821	2,96	2926,31	1
1	145	475	3,28	6747,17	1
1	186	559	3,01	2236,79	1
1	279	823	2,95	1755,29	1
1	172	496	2,88	1793,65	1
1	278	854	3,07	1845,91	1
1	168	488	2,90	2042,56	1
1	131	374	2,85	2240,89	1
1	176	529	3,01	2324,01	1
1	201	506	2,52	1431,72	1
1	292	866	2,97	2343,72	1
1	75	184	2,45	2003,08	1
1	170	508	2,99	3416,48	1
1	213	642	3,01	4177,69	1
1	261	770	2,95	4381,07	1
1	320	940	2,94	4324,43	1
1	294	803	2,73	3748,42	1

1	215	612	2,85	3012,57	1
1	208	527	2,53	3504,17	1
1	231	557	2,41	2737,61	1
1	147	495	3,37	1869,94	1
1	197	585	2,97	2185,05	1
1	191	600	3,14	1629,98	1
1	95	320	3,37	632,16	1
1	78	246	3,15	1708,81	1
1	274	783	2,86	1612,89	1
1	300	959	3,20	2002,42	1
1	303	906	2,99	1644,21	1
1	232	768	3,31	1266,74	1
1	220	711	3,23	1586,43	1
1	339	1074	3,17	1594,96	1
1	260	871	3,35	3344,83	1
1	315	968	3,07	1440,74	1
1	351	1047	2,98	1361,66	1
1	319	1088	3,41	745,65	1
1	327	1041	3,18	1222,38	1
1	141	436	3,09	1293,72	1
1	263	801	3,05	1313,12	1
1	252	919	3,65	693,33	1
1	293	993	3,39	593,28	1
1	236	820	3,47	1127,33	1
1	242	806	3,33	1353,61	1
1	389	1239	3,19	1358,11	1
1	240	801	3,34	1160,49	1
1	145	448	3,09	1140,31	1
1	144	470	3,26	1495,71	1
1	175	589	3,37	1278,26	1
1	191	648	3,39	1098,25	1
1	260	871	3,35	1327,45	1
1	349	1054	3,02	1559,42	1
1	264	787	2,98	1502,83	1
1	139	478	3,44	896,24	1
1	133	468	3,52	778,89	1
1	251	766	3,05	1838,73	1
1	297	836	2,81	1929,00	1
1	295	842	2,85	1956,45	1
1	225	669	2,97	1987,21	1
1	317	925	2,92	2290,35	1
1	262	677	2,58	2795,10	1
1	313	811	2,59	2202,91	1
1	303	700	2,31	2099,15	1
1	261	719	2,75	2299,79	1
1	153	445	2,91	1780,13	1
1	264	847	3,21	2683,21	1
1	210	630	3,00	2730,06	1
1	170	531	3,12	2571,20	1
1	245	726	2,96	3717,36	1

1	201	554	2,76	3396,14	1
1	213	613	2,88	2184,88	1
1	198	560	2,83	2783,75	1
1	315	794	2,52	3405,78	1
1	38	102	2,68	1134,50	1
1	7	12	1,71	1671,43	1
1	25	89	3,56	983,60	1
1	79	218	2,76	1309,10	1
1	20	64	3,20	1573,75	1
1	29	97	3,34	1055,86	1
1	2	6	3,00	800,00	1
1	40	145	3,63	776,45	1
1	3	13	4,33	608,00	1
1	66	205	3,11	840,83	1
1	182	602	3,31	482,36	1
1	15	41	2,73	1229,33	1
1	20	69	3,45	842,25	1
1	2	4	2,00	655,00	1
1	73	270	3,70	780,90	1
1	185	518	2,80	1752,53	1
1	112	402	3,59	677,40	1
1	73	208	2,85	2344,92	1
1	192	507	2,64	3246,32	1
1	63	205	3,25	1066,60	1
1	84	260	3,10	904,94	1
1	80	287	3,59	571,99	1
1	77	208	2,70	2681,40	1
1	142	410	2,89	3573,15	1
1	258	815	3,16	1121,09	1
1	166	495	2,98	1314,43	1
1	31	99	3,19	2774,71	1
1	25	75	3,00	5748,80	1
1	96	305	3,18	762,85	1
1	157	496	3,16	1416,19	1
1	307	949	3,09	758,07	1
1	240	786	3,28	1229,87	1
1	235	725	3,09	1130,49	1
1	66	198	3,00	668,48	1
1	157	538	3,43	1175,98	1
1	64	214	3,34	785,33	1
1	138	458	3,32	698,32	1
1	50	158	3,16	1061,76	1
1	0	0	0,00	0,00	1
1	88	340	3,86	539,01	1
1	138	468	3,39	990,12	1
1	163	571	3,50	845,17	1
1	331	1053	3,18	2673,91	1
1	220	677	3,08	2573,55	1
1	325	959	2,95	2435,60	1
1	123	372	3,02	1348,07	1

1	325	947	2,91	1353,11	1
0	0	0	0,00	0,00	0
1	249	801	3,22	884,79	1
1	205	646	3,15	1053,31	1
1	301	949	3,15	1291,47	1
1	325	1027	3,16	1259,16	1
1	284	856	3,01	1117,31	1
1	300	973	3,24	1256,05	1
1	295	935	3,17	1005,83	1
1	248	792	3,19	1337,06	1
1	142	459	3,23	1264,30	1
1	277	858	3,10	1271,73	1
1	127	378	2,98	1874,58	1
1	253	806	3,19	1113,27	1
1	317	942	2,97	1858,88	1
1	340	1010	2,97	1974,43	1
1	269	738	2,74	1240,29	1
1	100	300	3,00	1807,20	1
1	197	583	2,96	3148,35	1
1	250	661	2,64	2211,34	1
1	201	552	2,75	2041,22	1
1	260	767	2,95	2112,14	1
1	242	717	2,96	2019,09	1
1	100	294	2,94	2197,86	1
1	228	623	2,73	3206,56	1
1	263	737	2,80	3380,54	1
1	129	403	3,12	2716,74	1
1	11	27	2,45	1004,45	1
1	177	587	3,32	901,45	1
1	302	856	2,83	2231,31	1
1	228	673	2,95	2047,37	1
1	225	581	2,58	1973,97	1
1	34	120	3,53	1472,94	1
1	137	442	3,23	2127,79	1
1	260	810	3,12	1536,05	1
1	441	1268	2,88	1583,63	1
1	29	101	3,48	926,59	1
1	239	668	2,79	1237,56	1
1	201	599	2,98	1216,35	1
1	165	498	3,02	1766,47	1
1	128	494	3,86	1901,23	1
1	266	736	2,77	1346,22	1
1	441	1295	2,94	2567,26	1
1	263	860	3,27	1287,86	1
1	218	666	3,06	1223,82	1
1	338	974	2,88	2251,35	1
1	240	686	2,86	1701,87	1
1	198	599	3,03	1137,81	1
1	293	788	2,69	1568,39	1
1	193	509	2,64	2573,21	1

1	350	905	2,59	1930,49	1
1	288	974	3,38	1250,02	1
1	369	1239	3,36	1042,85	1
1	279	845	3,03	1227,79	1
1	311	1053	3,39	1820,98	1
1	311	994	3,20	1485,11	1
1	237	765	3,23	2000,16	1
1	253	838	3,31	1563,48	1
1	309	925	2,99	2313,20	1
1	340	1154	3,39	1146,46	1
1	310	915	2,95	1515,82	1
1	292	822	2,82	1293,66	1
1	109	416	3,82	600,71	1
1	201	737	3,67	652,83	1
1	145	487	3,36	626,34	1
1	294	992	3,37	655,69	1
1	135	465	3,44	673,46	1
1	239	736	3,08	1323,89	1
1	213	650	3,05	1766,08	1
1	138	437	3,17	1142,28	1
1	229	773	3,38	1007,98	1
1	258	788	3,05	920,65	1
1	226	767	3,39	1082,52	1
1	210	662	3,15	791,84	1
1	62	219	3,53	570,58	1
1	152	514	3,38	616,63	1
1	156	552	3,54	946,23	1
1	125	458	3,66	671,82	1
1	269	858	3,19	838,76	1
1	359	1129	3,14	927,13	1
1	318	1016	3,19	1030,71	1
1	437	1449	3,32	1216,12	1
1	260	839	3,23	1152,54	1
1	396	1241	3,13	1145,65	1
1	261	887	3,40	1089,30	1
1	161	506	3,14	1060,01	1
1	231	708	3,06	878,34	1
1	257	768	2,99	995,80	1
1	271	857	3,16	1360,35	1
1	429	1377	3,21	1440,71	1
1	206	632	3,07	1127,18	1
1	255	790	3,10	1798,84	1
1	287	924	3,22	804,70	1
1	273	896	3,28	1321,42	1
1	153	472	3,08	1510,49	1
1	406	1414	3,48	1412,19	1
1	362	1225	3,38	1332,67	1
1	413	1458	3,53	1136,89	1
1	317	1019	3,21	1463,00	1
1	225	758	3,37	1233,80	1

1	181	591	3,27	1139,43	1
1	289	927	3,21	1205,23	1
1	229	788	3,44	1124,73	1
1	214	678	3,17	1208,33	1
1	265	773	2,92	1088,21	1
1	254	775	3,05	1247,59	1
1	283	877	3,10	1457,45	1
1	328	1083	3,30	1025,45	1
1	306	949	3,10	1364,22	1
1	294	888	3,02	1260,84	1
1	270	905	3,35	1153,56	1
1	334	1013	3,03	1213,77	1
1	187	620	3,32	1938,87	1
1	304	844	2,78	2040,20	1
1	271	905	3,34	2634,64	1
1	301	1060	3,52	2808,66	1
1	291	891	3,06	2508,60	1
1	245	802	3,27	2190,84	1
1	166	528	3,18	2380,51	1
1	184	600	3,26	2296,39	1
1	282	960	3,40	3425,77	1
1	230	768	3,34	2769,74	1
1	209	663	3,17	2375,89	1
1	303	1017	3,36	2802,46	1
1	157	440	2,80	1951,57	1
1	199	636	3,20	1618,63	1
1	147	494	3,36	2354,53	1
1	101	297	2,94	1040,41	1
1	158	526	3,33	1115,61	1
1	185	643	3,48	888,81	1
1	27	76	2,81	683,70	1
0	0	0	0,00	0,00	0
1	34	115	3,38	5635,59	1
1	51	215	4,22	714,57	1
1	86	262	3,05	689,09	1
1	7	24	3,43	491,43	1
1	75	284	3,79	780,08	1
1	305	974	3,19	1144,10	1
1	296	902	3,05	1028,75	1
1	283	913	3,23	885,71	1
1	256	822	3,21	1071,91	1
1	218	752	3,45	1067,82	1
1	252	802	3,18	841,44	1
1	205	675	3,29	1017,91	1
1	119	419	3,52	881,13	1
1	328	1084	3,30	650,02	1
1	83	278	3,35	932,59	1
1	90	305	3,39	722,16	1
1	147	513	3,49	836,35	1
1	138	448	3,25	714,06	1

1	256	822	3,21	1127,90	1
1	185	624	3,37	1273,24	1
1	66	234	3,55	1017,79	1
1	370	1182	3,19	3285,68	1
1	134	455	3,40	1270,71	1
1	334	1101	3,30	1726,99	1
1	222	662	2,98	1098,64	1
1	156	455	2,92	1127,91	1
1	165	525	3,18	1030,21	1
1	266	856	3,22	921,62	1
1	262	847	3,23	1612,15	1
1	202	740	3,66	468,55	1
1	204	657	3,22	452,11	1
1	185	687	3,71	651,26	1
1	269	903	3,36	698,43	1
1	280	863	3,08	791,11	1
1	283	814	2,88	1936,07	1
1	247	542	2,19	1696,93	1
1	331	916	2,77	1994,99	1
1	270	844	3,13	1323,87	1
1	228	622	2,73	1615,28	1
1	240	735	3,06	1930,96	1
1	252	790	3,13	2171,42	1
1	190	661	3,48	616,37	1
1	106	350	3,30	642,58	1
1	234	737	3,15	642,47	1
1	298	1186	3,98	608,63	1
1	236	809	3,43	1463,61	1
1	178	538	3,02	1590,85	1
1	291	876	3,01	1550,24	1
1	214	690	3,22	1202,00	1
1	240	662	2,76	1377,13	1
1	229	701	3,06	1025,02	1
1	263	792	3,01	1604,92	1
1	187	549	2,94	1385,78	1
1	202	599	2,97	1565,86	1
1	103	293	2,84	1445,27	1
1	250	741	2,96	1307,86	1
1	219	672	3,07	1353,33	1
1	62	204	3,29	994,92	1
1	54	156	2,89	1347,91	1
1	252	804	3,19	1013,58	1
1	271	819	3,02	1016,25	1
1	270	839	3,11	1071,01	1
1	208	627	3,01	970,14	1
1	258	834	3,23	351,82	1
1	17	49	2,88	1171,18	1
1	317	1088	3,43	697,61	1
1	253	892	3,53	657,97	1
1	109	357	3,28	640,65	1

1	276	703	2,55	1385,48	1
1	276	640	2,32	1555,46	1
1	297	746	2,51	1592,93	1
1	167	489	2,93	1570,53	1
1	115	483	4,20	705,50	1
1	209	750	3,59	870,14	1
1	232	783	3,38	571,81	1
1	175	585	3,34	770,73	1
1	237	817	3,45	516,89	1
1	172	496	2,88	1362,65	1
1	131	438	3,34	803,13	1
1	157	450	2,87	1782,47	1
1	255	759	2,98	1832,06	1
1	297	909	3,06	1668,67	1
1	270	719	2,66	1918,01	1
1	130	387	2,98	2248,32	1
1	142	400	2,82	1936,65	1
1	217	508	2,34	1873,55	1
1	192	578	3,01	1386,79	1
1	249	643	2,58	1553,52	1
1	72	232	3,22	3628,93	1
1	207	529	2,56	1472,39	1
1	238	557	2,34	2268,48	1
1	167	445	2,66	2076,66	1
1	276	749	2,71	1607,64	1
1	152	449	2,95	2416,17	1
1	243	615	2,53	2031,51	1
1	212	542	2,56	2382,63	1
1	148	396	2,68	1249,48	1
1	230	679	2,95	1718,20	1
1	233	655	2,81	1996,67	1
1	242	678	2,80	1748,09	1
1	264	733	2,78	2637,39	1
1	242	695	2,87	2427,71	1
1	215	623	2,90	1700,75	1
1	293	1346	4,59	666,45	1
1	215	594	2,76	2733,58	1
1	250	669	2,68	2839,19	1
1	284	744	2,62	2702,26	1
1	179	516	2,88	3223,56	1
1	11	27	2,45	1730,64	1
1	26	91	3,50	4227,88	1
1	14	40	2,86	2991,43	1
1	132	392	2,97	4343,57	1
1	184	564	3,07	3435,67	1
1	229	641	2,80	1654,01	1
1	179	535	2,99	2662,73	1
1	193	533	2,76	2195,53	1
1	236	597	2,53	2256,59	1
1	97	260	2,68	2468,67	1

1	51	133	2,61	1957,96	1
1	99	290	2,93	4880,77	1
0	0	0	0,00	0,00	0
1	92	266	2,89	2289,93	1
1	25	89	3,56	1164,96	1
1	98	287	2,93	1588,14	1
1	106	303	2,86	2040,81	1
1	45	144	3,20	748,00	1
1	9	32	3,56	795,56	1
1	17	48	2,82	1052,94	1
1	42	145	3,45	842,17	1
1	137	440	3,21	972,07	1
1	221	562	2,54	2639,10	1
1	16	38	2,38	3801,06	1
1	126	358	2,84	2278,51	1
1	165	474	2,87	4967,15	1
1	41	142	3,46	1023,66	1
1	157	496	3,16	1735,03	1
1	85	271	3,19	2714,15	1
1	44	136	3,09	1173,25	1
1	24	72	3,00	1701,58	1
1	26	96	3,69	2226,15	1
1	49	158	3,22	1737,73	1
1	64	183	2,86	947,25	1
1	186	560	3,01	2785,63	1
1	62	170	2,74	2782,10	1
1	81	278	3,43	1598,77	1
1	51	142	2,78	1532,55	1
1	97	315	3,25	711,80	1
1	22	78	3,55	465,82	1
1	122	399	3,27	1804,28	1
1	219	694	3,17	1456,25	1
1	48	158	3,29	1315,21	1
1	23	76	3,30	1475,00	1
1	55	192	3,49	1216,42	1
1	25	94	3,76	730,00	1
1	19	62	3,26	1152,11	1
1	28	93	3,32	1764,29	1
1	153	492	3,22	1304,57	1
1	67	203	3,03	1211,15	1
1	47	148	3,15	639,53	1
1	231	666	2,88	2986,01	1
1	201	645	3,21	2482,64	1
1	143	356	2,49	2348,87	1
1	144	482	3,35	911,56	1
1	134	426	3,18	1005,31	1
1	20	66	3,30	862,50	1
1	19	61	3,21	1351,58	1
1	145	450	3,10	1200,12	1
1	192	610	3,18	1468,11	1

1	25	73	2,92	1891,24	1
1	36	91	2,53	2364,03	1
1	145	427	2,94	1691,04	1
1	36	125	3,47	2109,44	1
1	192	517	2,69	1884,81	1
1	145	479	3,30	3575,71	1
1	80	212	2,65	4090,88	1
1	18	67	3,72	1900,61	1
1	78	241	3,09	1046,33	1
1	5	12	2,40	1600,00	1
1	256	816	3,19	1181,30	1
1	174	550	3,16	1809,35	1
1	86	235	2,73	1133,86	1
1	167	476	2,85	2459,38	1
1	143	469	3,28	1495,55	1
1	80	232	2,90	1743,88	1
1	238	755	3,17	1053,91	1
1	72	252	3,50	783,40	1
1	151	477	3,16	684,30	1
1	13	45	3,46	1173,85	1
1	302	809	2,68	1337,72	1
1	202	567	2,81	1426,33	1
1	2	10	5,00	655,00	1
1	220	748	3,40	2322,90	1
1	310	834	2,69	2374,74	1
1	209	590	2,82	2488,04	1
1	17	56	3,29	992,12	1
1	189	705	3,73	1522,10	1
1	71	247	3,48	3573,48	1
0	0	0	0,00	0,00	0
1	85	298	3,51	911,81	1
1	50	151	3,02	940,30	1
0	0	0	0,00	0,00	0
1	53	191	3,60	792,00	1
1	252	656	2,60	1431,34	1
1	330	1084	3,28	827,94	1
1	9	27	3,00	739,56	1
1	90	286	3,18	936,96	1
1	83	296	3,57	1109,28	1
1	51	147	2,88	1080,20	1
1	46	140	3,04	1041,50	1
1	66	228	3,45	764,41	1
1	22	77	3,50	612,82	1
1	34	108	3,18	730,68	1
1	35	104	2,97	1006,71	1
1	110	339	3,08	1319,45	1
1	91	259	2,85	1035,49	1
1	82	255	3,11	957,85	1
1	35	107	3,06	1156,80	1
1	47	157	3,34	1399,57	1

1	5	15	3,00	950,00	1
1	53	175	3,30	1228,02	1
1	150	352	2,35	2544,31	1
1	30	111	3,70	3060,83	1
1	36	107	2,97	1035,28	1
1	90	288	3,20	1276,97	1
1	102	325	3,19	1951,55	1
1	115	362	3,15	1532,11	1
1	118	389	3,30	930,35	1
1	202	667	3,30	1181,37	1
1	23	96	4,17	664,35	1
1	12	43	3,58	1169,17	1
1	50	168	3,36	1157,24	1
1	26	72	2,77	821,92	1
1	89	326	3,66	691,48	1
1	64	227	3,55	970,94	1
1	19	58	3,05	1182,11	1
1	66	211	3,20	820,91	1
1	48	182	3,79	629,60	1
1	14	43	3,07	1034,29	1
1	103	356	3,46	997,50	1
1	56	203	3,63	816,25	1
1	4	12	3,00	968,75	1
1	45	145	3,22	4158,51	1
1	141	406	2,88	1324,49	1
1	49	168	3,43	997,67	1
1	107	307	2,87	1064,16	1
1	110	356	3,24	908,27	1
1	60	199	3,32	1406,02	1
1	8	15	1,88	1883,75	1
1	66	226	3,42	1187,39	1
1	40	119	2,98	1224,50	1
0	0	0	0,00	0,00	0
1	50	131	2,62	1451,14	1
1	17	58	3,41	646,76	1
1	33	107	3,24	643,24	1
1	12	33	2,75	1428,42	1
1	95	204	2,15	1939,22	1
1	51	156	3,06	2756,31	1
1	152	428	2,82	3113,01	1
1	177	523	2,95	3647,88	1
1	155	417	2,69	3091,50	1
1	144	343	2,38	3392,47	1
1	15	56	3,73	773,33	1
1	25	78	3,12	1116,80	1
1	306	962	3,14	2301,32	1
1	229	729	3,18	1559,88	1
1	76	226	2,97	959,88	1
1	362	1221	3,37	1030,97	1
1	298	935	3,14	2293,67	1

1	155	483	3,12	1848,97	1
1	174	588	3,38	2381,36	1
1	204	617	3,02	1055,24	1
1	183	578	3,16	767,33	1
1	316	926	2,93	1242,75	1
1	259	769	2,97	1443,90	1
1	262	945	3,61	1026,21	1
1	241	774	3,21	1361,49	1
1	285	906	3,18	1235,66	1
1	122	404	3,31	1451,82	1
1	420	1252	2,98	3250,75	1
1	222	706	3,18	1773,94	1
1	385	1187	3,08	2011,94	1
1	228	733	3,21	1224,21	1
1	297	1032	3,47	1446,79	1
1	70	216	3,09	1011,43	1
1	252	772	3,06	2474,58	1
1	382	1372	3,59	990,04	1
1	362	1180	3,26	1363,41	1
1	350	1025	2,93	2547,49	1
1	267	917	3,43	1728,28	1
1	283	931	3,29	1892,40	1
1	236	788	3,34	2081,54	1
1	310	1073	3,46	3754,91	1
1	364	1278	3,51	731,77	1
1	415	1291	3,11	946,44	1
1	230	708	3,08	868,43	1
1	304	904	2,97	925,46	1
1	261	819	3,14	833,79	1
1	306	929	3,04	1089,39	1
1	240	783	3,26	1213,86	1
1	210	794	3,78	1007,16	1
1	127	449	3,54	1094,28	1
1	247	763	3,09	1260,52	1
1	313	881	2,81	2394,18	1
1	196	616	3,14	1985,39	1
1	231	630	2,73	2129,57	1
1	109	392	3,60	728,99	1
1	176	614	3,49	597,88	1
1	44	201	4,57	718,48	1
1	178	646	3,63	629,39	1
1	139	562	4,04	874,24	1
1	84	353	4,20	638,18	1
1	333	1231	3,70	801,73	1
1	94	332	3,53	840,57	1
1	310	1170	3,77	833,78	1
1	178	616	3,46	1399,47	1
1	361	1061	2,94	1876,88	1
1	146	385	2,64	3927,60	1
1	191	525	2,75	4656,27	1

1	257	554	2,16	2397,75	1
1	228	513	2,25	3315,72	1
1	179	545	3,04	2383,05	1
1	331	969	2,93	2454,59	1
1	247	740	3,00	2124,57	1
1	209	608	2,91	2328,26	1
1	189	534	2,83	2044,06	1
1	494	1443	2,92	1161,76	1
1	213	719	3,38	1335,24	1
1	210	676	3,22	1512,36	1
1	336	1025	3,05	1626,88	1
1	273	946	3,47	2054,50	1
1	209	748	3,58	1607,05	1
1	207	728	3,52	976,82	1
1	184	565	3,07	584,28	1
1	338	1038	3,07	789,75	1
1	350	1119	3,20	732,05	1
1	220	641	2,91	1011,41	1
1	312	1022	3,28	748,21	1
1	317	966	3,05	564,48	1
1	156	473	3,03	1020,17	1
1	299	844	2,82	832,33	1
1	331	1196	3,61	672,21	1
1	247	834	3,38	2298,53	1
1	95	318	3,35	3139,65	1
1	170	428	2,52	2223,94	1
1	243	760	3,13	2022,73	1
1	117	385	3,29	8898,24	1
1	209	678	3,24	1684,00	1
1	278	794	2,86	2074,15	1
1	257	901	3,51	1624,54	1
1	292	880	3,01	2937,95	1
1	102	316	3,10	2522,79	1
1	195	529	2,71	3308,03	1
1	315	969	3,08	1734,95	1
1	197	630	3,20	1815,31	1
1	184	581	3,16	1396,95	1
1	118	371	3,14	845,55	1
1	184	654	3,55	4809,03	1
1	340	840	2,47	3794,82	1
1	264	777	2,94	2863,20	1
1	371	1028	2,77	4138,05	1
1	199	613	3,08	7384,51	1
1	230	633	2,75	3051,16	1
1	138	358	2,59	4939,12	1
1	218	656	3,01	6883,49	1
1	301	901	2,99	6315,10	1
1	206	586	2,84	5032,09	1
1	362	973	2,69	5808,47	1
1	182	467	2,57	4995,34	1

1	287	906	3,16	7363,72	1
1	357	1183	3,31	1427,53	1
1	217	731	3,37	1240,70	1
1	309	971	3,14	1387,20	1
1	247	781	3,16	1510,47	1
1	206	657	3,19	1371,73	1
1	230	719	3,13	1545,87	1
1	174	545	3,13	1600,80	1
1	379	1188	3,13	1501,17	1
1	216	688	3,19	1144,68	1
1	169	497	2,94	1239,24	1
1	304	1030	3,39	551,52	1
1	323	984	3,05	1206,66	1
1	319	1190	3,73	690,19	1
1	142	505	3,56	872,08	1
1	200	683	3,42	835,16	1
1	169	589	3,49	573,95	1
1	103	357	3,47	893,81	1
1	13	44	3,38	760,77	1
1	124	457	3,69	568,05	1
1	98	317	3,23	744,31	1
1	76	277	3,64	664,01	1
1	224	766	3,42	767,14	1
1	233	742	3,18	773,12	1
1	77	211	2,74	632,06	1
1	233	728	3,12	674,30	1
1	155	507	3,27	905,91	1
1	146	523	3,58	909,17	1
1	128	441	3,45	612,59	1
1	142	451	3,18	721,36	1
1	275	887	3,23	759,51	1
1	64	168	2,63	677,41	1
1	102	317	3,11	872,20	1
1	106	358	3,38	626,97	1
1	161	541	3,36	996,75	1
1	165	528	3,20	888,53	1
1	139	456	3,28	833,82	1
1	233	817	3,51	1086,49	1
1	102	330	3,24	750,60	1
1	320	998	3,12	1546,00	1
1	132	403	3,05	1819,58	1
1	187	477	2,55	1604,51	1
1	353	999	2,83	1803,33	1
1	150	420	2,80	941,22	1
1	58	193	3,33	1827,22	1
1	268	745	2,78	2510,32	1
1	311	936	3,01	3008,51	1
1	264	671	2,54	1986,52	1
1	184	686	3,73	1206,08	1
1	254	712	2,80	2973,22	1

1	237	602	2,54	2401,74	1
1	199	653	3,28	646,04	1
1	432	1254	2,90	3032,99	1
1	211	582	2,76	3374,96	1
1	213	583	2,74	1629,44	1
1	295	704	2,39	2623,11	1
1	120	341	2,84	3311,23	1
1	174	530	3,05	3194,26	1
1	139	469	3,37	1577,41	1
1	156	466	2,99	3947,85	1
1	226	832	3,68	575,79	1
1	240	746	3,11	676,65	1
1	240	784	3,27	1410,14	1
1	133	480	3,61	616,39	1
1	240	769	3,20	507,94	1
1	111	426	3,84	649,86	1
1	198	644	3,25	979,26	1
1	156	534	3,42	726,32	1
1	80	248	3,10	629,26	1
1	80	286	3,58	847,13	1
1	646	1788	2,77	5076,25	1
1	573	1616	2,82	6367,56	1
1	470	1198	2,55	4389,04	1
1	309	867	2,81	4813,04	1
1	415	1040	2,51	5769,60	1
1	337	976	2,90	5868,90	1
1	540	1474	2,73	5885,56	1
1	235	641	2,73	5152,07	1
1	304	830	2,73	5264,28	1
1	283	867	3,06	1217,93	1
1	79	188	2,38	4058,48	1
1	280	867	3,10	942,40	1
1	125	389	3,11	1352,77	1
1	315	968	3,07	973,09	1
1	452	1417	3,13	783,40	1
1	240	666	2,78	1116,03	1
1	206	528	2,56	1140,52	1
1	3	8	2,67	2053,33	1
1	303	935	3,09	754,93	1
1	168	519	3,09	853,00	1
1	329	1060	3,22	782,98	1
1	129	410	3,18	1278,05	1
1	200	571	2,86	2027,59	1
1	173	520	3,01	1973,24	1
1	234	743	3,18	747,78	1
1	127	352	2,77	1234,50	1
1	272	810	2,98	2475,16	1
1	246	716	2,91	2852,12	1
1	297	858	2,89	3184,56	1
1	214	648	3,03	2905,78	1

1	301	872	2,90	2001,84	1
1	288	726	2,52	3385,38	1
1	348	1012	2,91	2979,30	1
1	237	666	2,81	2716,50	1
1	282	818	2,90	2550,15	1
1	298	746	2,50	1861,60	1
1	419	1110	2,65	3018,66	1
1	173	485	2,80	2008,76	1
1	14	52	3,71	1034,29	1
1	59	267	4,53	526,20	1
1	184	709	3,85	649,32	1
1	427	1529	3,58	880,04	1
1	93	318	3,42	1327,55	1
1	278	903	3,25	1276,87	1
1	30	106	3,53	992,67	1
1	154	516	3,35	603,03	1
1	92	284	3,09	654,41	1
1	262	859	3,28	703,40	1
1	42	139	3,31	847,26	1
1	231	664	2,87	2865,45	1
1	267	784	2,94	3575,90	1
1	219	670	3,06	2920,34	1
1	284	834	2,94	1461,23	1
1	347	1013	2,92	2666,48	1
1	293	875	2,99	2764,34	1
1	305	857	2,81	3156,90	1
1	367	1039	2,83	2805,09	1
1	70	200	2,86	2031,93	1
1	259	762	2,94	2941,77	1
1	211	653	3,09	3113,18	1
1	331	940	2,84	3385,05	1
1	345	911	2,64	3195,94	1
1	316	866	2,74	1770,47	1
1	155	593	3,83	709,00	1
1	108	385	3,56	836,89	1
1	81	279	3,44	698,91	1
1	95	369	3,88	513,84	1
1	103	423	4,11	566,41	1
1	225	610	2,71	3416,09	1
1	101	290	2,87	2460,87	1
1	257	721	2,81	4116,76	1
1	338	957	2,83	3207,17	1
1	357	1014	2,84	4767,73	1
1	302	897	2,97	4064,93	1
1	367	985	2,68	3057,82	1
1	275	743	2,70	2883,97	1
1	254	689	2,71	3057,62	1
1	357	974	2,73	4382,30	1
1	244	663	2,72	3870,94	1
1	266	706	2,65	3921,12	1

1	256	752	2,94	4746,50	1
1	259	733	2,83	4245,02	1
1	254	716	2,82	4936,95	1
1	325	934	2,87	5186,19	1
1	303	911	3,01	5997,50	1
1	81	350	4,32	796,12	1
1	178	663	3,72	760,80	1
1	123	442	3,59	949,20	1
1	148	488	3,30	749,53	1
1	212	793	3,74	626,92	1
1	100	305	3,05	525,16	1
1	171	592	3,46	557,70	1
1	237	592	2,50	3355,76	1
1	225	637	2,83	2808,95	1
1	361	964	2,67	3691,68	1
1	96	276	2,88	2321,13	1
1	254	677	2,67	3700,83	1
1	300	874	2,91	4160,19	1
1	157	446	2,84	4194,01	1
1	226	649	2,87	3924,89	1
1	278	818	2,94	6631,24	1
1	358	931	2,60	1791,83	1
1	180	431	2,39	3945,98	1
1	348	977	2,81	4628,29	1
1	215	607	2,82	3378,70	1
1	262	723	2,76	4832,15	1
1	276	740	2,68	4064,83	1
1	193	543	2,81	3477,74	1
1	290	771	2,66	3915,38	1
1	313	948	3,03	5429,61	1
1	240	683	2,85	5171,69	1
1	204	558	2,74	3817,32	1
1	284	852	3,00	6171,45	1
1	259	759	2,93	6664,39	1
1	90	218	2,42	5583,90	1
1	33	119	3,61	625,94	1
1	149	448	3,01	7266,98	1
1	389	1263	3,25	6900,90	1
1	136	470	3,46	450,95	1
1	126	481	3,82	566,97	1
1	156	388	2,49	3067,94	1
1	157	396	2,52	3542,01	1
1	265	732	2,76	3483,25	1
1	213	588	2,76	3628,51	1
1	334	941	2,82	4620,23	1
1	310	906	2,92	4655,74	1
1	251	700	2,79	3552,39	1
1	226	690	3,05	3976,55	1
1	209	599	2,87	4468,03	1
1	195	538	2,76	4128,18	1

1	243	654	2,69	4745,37	1
1	233	620	2,66	4332,09	1
1	251	683	2,72	4076,77	1
1	284	764	2,69	4544,20	1
1	280	784	2,80	4313,20	1
1	410	1306	3,19	8260,97	1
1	335	892	2,66	5376,12	1
1	336	967	2,88	5462,85	1
1	356	1056	2,97	6141,81	1
1	253	752	2,97	6474,10	1
1	268	730	2,72	5538,22	1
1	355	1065	3,00	10981,38	1
1	281	856	3,05	2813,42	1
1	217	593	2,73	1458,36	1
1	43	123	2,86	2314,00	1
1	157	407	2,59	1137,47	1
1	55	186	3,38	785,96	1
1	102	324	3,18	789,22	1
1	161	502	3,12	969,01	1
1	83	260	3,13	1812,05	1
1	88	323	3,67	702,77	1
1	24	72	3,00	2169,83	1
1	65	205	3,15	995,63	1
1	92	373	4,05	619,89	1
1	100	239	2,39	1705,00	1
1	22	94	4,27	857,41	1
1	4	17	4,25	825,00	1
1	291	824	2,83	1978,12	1
1	87	347	3,99	586,79	1
1	385	1260	3,27	720,81	1
1	323	1007	3,12	1305,10	1
1	80	270	3,38	1562,45	1
1	161	518	3,22	1496,38	1
1	49	162	3,31	751,96	1
1	144	451	3,13	1583,33	1
1	66	212	3,21	693,33	1
1	27	94	3,48	847,22	1
1	219	699	3,19	977,51	1
1	270	842	3,12	1486,56	1
1	155	579	3,74	747,01	1
1	21	80	3,81	703,33	1
1	12	44	3,67	774,42	1
1	21	85	4,05	818,57	1
1	138	352	2,55	3059,27	1
1	150	479	3,19	1269,45	1
1	57	206	3,61	1129,63	1
1	91	297	3,26	1488,75	1
1	170	573	3,37	1219,88	1
1	210	735	3,50	1539,24	1
1	461	1280	2,78	1841,20	1

1	324	825	2,55	2687,42	1
1	25	92	3,68	4563,60	1
1	14	39	2,79	1052,14	1
1	95	243	2,56	3486,28	1
1	108	338	3,13	3570,56	1
1	102	296	2,90	6362,20	1
1	10	29	2,90	7830,00	1
1	146	532	3,64	514,22	1
1	23	95	4,13	739,13	1
1	56	158	2,82	4358,98	1
1	83	271	3,27	809,27	1
1	235	692	2,94	1025,74	1
1	35	119	3,40	4166,83	1
1	27	95	3,52	1404,07	1
1	136	430	3,16	917,05	1
1	205	658	3,21	6008,76	1
1	163	521	3,20	7124,96	1
1	49	147	3,00	631,63	1
1	150	426	2,84	1774,01	1
1	238	774	3,25	2033,05	1
1	45	147	3,27	1583,82	1
1	10	39	3,90	807,00	1
1	70	236	3,37	637,40	1
1	35	95	2,71	2417,37	1
1	3	10	3,33	903,33	1
1	33	118	3,58	807,73	1
1	139	400	2,88	1737,12	1
1	15	63	4,20	767,20	1
1	12	36	3,00	547,50	1
1	123	398	3,24	1106,50	1
1	264	919	3,48	657,70	1
1	77	244	3,17	914,08	1
1	5	16	3,20	2370,00	1
1	239	609	2,55	4700,20	1
1	246	551	2,24	3947,31	1
1	274	776	2,83	8862,96	1
1	338	856	2,53	5170,90	1
1	147	434	2,95	5618,07	1
1	362	770	2,13	5564,91	1
1	204	572	2,80	5467,25	1
1	223	617	2,77	6286,25	1
1	236	630	2,67	5681,90	1
1	272	740	2,72	5309,93	1
1	176	500	2,84	6854,39	1
1	417	1160	2,78	6284,43	1
1	218	618	2,83	4041,68	1
1	200	643	3,22	2926,36	1
1	64	207	3,23	1124,77	1
1	94	298	3,17	1195,05	1
1	152	498	3,28	1573,26	1

1	200	704	3,52	596,11	1
1	3	15	5,00	1056,67	1
1	41	140	3,41	1087,80	1
1	54	182	3,37	674,02	1
1	119	373	3,13	880,06	1
1	44	168	3,82	730,48	1
1	47	176	3,74	605,53	1
1	12	37	3,08	834,33	1
1	129	413	3,20	680,19	1
1	29	96	3,31	786,83	1
1	164	568	3,46	656,09	1
1	22	67	3,05	1315,45	1
1	82	274	3,34	620,37	1
1	174	580	3,33	1148,06	1
1	80	245	3,06	1669,55	1
1	34	113	3,32	1919,71	1
1	229	819	3,58	1107,32	1
0	0	0	0,00	0,00	0
1	23	79	3,43	568,70	1
1	78	232	2,97	1679,68	1
1	73	206	2,82	1926,58	1
1	63	175	2,78	2149,68	1
1	95	253	2,66	2915,39	1
1	90	263	2,92	3034,53	1
1	49	156	3,18	2122,04	1
1	108	334	3,09	2009,44	1
1	196	569	2,90	2023,88	1
1	235	659	2,80	2379,31	1
1	58	189	3,26	599,74	1
1	61	247	4,05	626,02	1
1	173	467	2,70	3207,04	1
1	33	108	3,27	1153,03	1
1	9	39	4,33	1637,78	1
1	4	13	3,25	1430,00	1
1	11	40	3,64	649,55	1
1	216	806	3,73	612,15	1
1	31	110	3,55	1042,58	1
1	239	902	3,77	635,14	1
1	15	65	4,33	890,00	1
1	93	369	3,97	554,75	1
1	535	1345	2,51	5146,35	1
1	94	342	3,64	10704,95	1
1	24	88	3,67	6721,25	1
1	1	2	2,00	580,00	1
1	67	195	2,91	1700,87	1
1	279	861	3,09	834,51	1
1	191	674	3,53	1122,07	1
1	145	474	3,27	865,99	1
1	57	183	3,21	624,56	1
1	71	263	3,70	818,38	1

1	74	244	3,30	738,62	1
1	29	86	2,97	1004,14	1
1	300	910	3,03	1763,36	1
1	255	784	3,07	777,50	1
1	14	40	2,86	821,93	1
1	65	220	3,38	773,11	1
1	11	37	3,36	1966,36	1
1	21	76	3,62	678,76	1
1	84	289	3,44	853,65	1
1	64	192	3,00	1215,63	1
1	32	107	3,34	1957,50	1
1	3	12	4,00	686,67	1
1	93	255	2,74	2333,38	1
1	2	13	6,50	900,00	1
1	75	222	2,96	3438,73	1
1	92	214	2,33	2063,18	1
1	45	207	4,60	492,67	1
1	22	74	3,36	510,45	1
1	63	199	3,16	477,98	1
1	77	280	3,64	704,47	1
1	146	541	3,71	523,80	1
1	96	319	3,32	607,84	1
1	33	125	3,79	436,82	1
1	7	25	3,57	1008,57	1
0	0	0	0,00	0,00	0
1	96	237	2,47	1274,04	1
1	9	28	3,11	6033,33	1
1	68	236	3,47	1875,93	1
1	225	657	2,92	1685,70	1
1	193	703	3,64	688,18	1
1	285	759	2,66	3092,86	1
1	172	499	2,90	3640,97	1
1	275	865	3,15	5221,12	1
1	312	984	3,15	3048,48	1
1	275	795	2,89	2396,29	1
1	134	492	3,67	8244,15	1
1	270	967	3,58	9478,09	1
1	137	425	3,10	4670,93	1
0	0	0	0,00	0,00	0
1	243	752	3,09	1780,16	1
1	132	502	3,80	604,18	1
1	126	452	3,59	696,66	1
1	397	1408	3,55	903,39	1
1	273	784	2,87	4143,44	1
0	0	0	0,00	0,00	0
1	232	677	2,92	3287,93	1
1	227	671	2,96	1370,07	1
1	239	843	3,53	1068,27	1
1	323	1101	3,41	1288,73	1
1	244	790	3,24	1441,08	1

1	198	622	3,14	927,95	1
1	241	782	3,24	1347,15	1
1	33	118	3,58	868,85	1
1	393	1395	3,55	6624,16	1
1	314	920	2,93	5247,25	1
1	233	806	3,46	5603,73	1
1	230	725	3,15	4152,24	1
1	166	618	3,72	704,59	1
1	206	590	2,86	2817,64	1
1	241	671	2,78	2406,83	1
1	65	199	3,06	3846,31	1
1	220	703	3,20	3876,76	1
1	216	664	3,07	4061,99	1
1	215	661	3,07	2573,60	1
1	443	1250	2,82	2442,02	1
1	185	514	2,78	2743,70	1
1	21	55	2,62	3659,52	1
1	222	720	3,24	3231,26	1
1	296	867	2,93	3007,61	1
1	168	526	3,13	2593,65	1
1	175	559	3,19	1698,62	1
1	236	575	2,44	1760,21	1
1	222	629	2,83	1572,81	1
1	298	836	2,81	1976,01	1
1	158	553	3,50	1138,92	1
1	149	519	3,48	935,87	1
1	188	674	3,59	1292,57	1
0	0	0	0,00	0,00	0
1	114	338	2,96	2453,18	1
1	271	748	2,76	1998,65	1
1	197	538	2,73	2897,55	1
1	238	674	2,83	3274,55	1
1	148	431	2,91	3527,04	1
1	211	675	3,20	1875,49	1
1	253	786	3,11	1264,68	1
1	283	872	3,08	1445,35	1
1	239	678	2,84	2447,60	1
1	208	571	2,75	1775,70	1
1	277	879	3,17	1288,12	1
1	282	920	3,26	1152,17	1
1	244	797	3,27	1229,63	1
1	224	745	3,33	989,65	1
1	252	824	3,27	991,27	1
1	272	896	3,29	1819,60	1
1	309	909	2,94	1890,14	1
1	51	173	3,39	1717,65	1
1	244	800	3,28	993,17	1
1	273	765	2,80	1531,07	1
1	168	513	3,05	613,57	1
1	209	618	2,96	1328,80	1

1	128	396	3,09	1300,32	1
1	224	690	3,08	576,32	1
1	383	1330	3,47	862,32	1
1	312	1032	3,31	990,49	1
1	490	1622	3,31	1035,31	1
1	391	1467	3,75	646,84	1
1	112	391	3,49	1133,43	1
1	241	830	3,44	4092,02	1
1	231	767	3,32	1363,61	1
1	534	1969	3,69	600,56	1
1	188	665	3,54	2867,55	1
1	171	605	3,54	4472,71	1
1	84	325	3,87	6305,42	1
1	203	658	3,24	6905,72	1
1	184	589	3,20	2183,15	1
1	120	423	3,53	3099,08	1
1	297	1003	3,38	9496,75	1
1	19	63	3,32	8056,95	1
1	217	749	3,45	681,05	1
1	168	565	3,36	4297,56	1
1	178	595	3,34	4928,89	1
1	209	672	3,22	2616,34	1
1	181	610	3,37	3946,82	1
1	132	436	3,30	5006,02	1
1	270	830	3,07	3649,51	1
1	269	854	3,17	2951,90	1
1	222	681	3,07	3849,36	1
1	210	655	3,12	3874,98	1
1	221	738	3,34	4090,85	1
1	182	646	3,55	1041,96	1
1	167	532	3,19	795,48	1
1	252	801	3,18	951,87	1
0	0	0	0,00	0,00	0
1	306	941	3,08	2746,58	1
1	56	209	3,73	1056,61	1
1	257	797	3,10	1453,31	1
1	212	702	3,31	2379,60	1
1	288	928	3,22	1188,61	1
1	109	264	2,42	1550,28	1
1	318	1133	3,56	572,99	1
1	74	233	3,15	847,32	1
1	193	683	3,54	426,19	1
1	29	109	3,76	524,00	1
1	76	290	3,82	1130,39	1
1	181	537	2,97	1209,18	1
1	269	853	3,17	1347,62	1
1	336	1194	3,55	848,00	1
1	5	13	2,60	1753,00	1
1	92	286	3,11	5724,36	1
1	170	525	3,09	4091,38	1

1	210	643	3,06	3533,61	1
1	19	64	3,37	1284,05	1
1	250	758	3,03	2448,14	1
1	138	509	3,69	694,04	1
1	235	864	3,68	9691,15	1
1	79	270	3,42	653,86	1
1	211	515	2,44	5218,05	1
1	201	573	2,85	4182,84	1
1	143	436	3,05	4743,52	1
1	186	536	2,88	4964,77	1
1	214	686	3,21	5098,95	1
1	168	493	2,93	4829,70	1
1	224	621	2,77	3906,71	1
1	177	534	3,02	4510,93	1
1	154	462	3,00	3815,25	1
1	204	614	3,01	4167,26	1
1	171	474	2,77	4299,37	1
1	133	406	3,05	4658,32	1
1	230	723	3,14	4814,93	1
1	206	629	3,05	4673,73	1
1	204	602	2,95	4953,49	1
1	276	655	2,37	2235,79	1
1	231	674	2,92	2939,49	1
1	250	724	2,90	4024,99	1
1	233	711	3,05	3994,57	1
1	145	344	2,37	1876,28	1
1	129	284	2,20	3332,94	1
1	117	280	2,39	4402,31	1
1	351	1102	3,14	2420,19	1
1	98	272	2,78	2997,47	1
1	2	10	5,00	3600,00	1
1	367	1097	2,99	3054,05	1
1	0	0	0,00	0,00	1
1	154	462	3,00	4357,94	1
1	44	98	2,23	3139,09	1
1	119	396	3,33	4056,63	1
1	198	576	2,91	4750,68	1
1	129	353	2,74	4521,37	1
1	229	592	2,59	3595,74	1
1	31	92	2,97	4209,84	1
1	174	447	2,57	2733,09	1
1	250	645	2,58	2868,73	1
1	133	417	3,14	1818,23	1
1	170	548	3,22	1957,04	1
1	80	270	3,38	1674,88	1
1	107	426	3,98	1527,99	1
1	25	70	2,80	2787,64	1
1	342	809	2,37	2713,13	1
1	32	103	3,22	3623,13	1
1	266	828	3,11	5524,09	1

1	101	306	3,03	2223,39	1
1	214	616	2,88	3041,71	1
1	217	617	2,84	2462,74	1
1	67	204	3,04	2650,60	1
1	216	662	3,06	5795,08	1
1	112	365	3,26	3869,38	1
1	111	335	3,02	622,87	1
1	279	721	2,58	2089,30	1
1	211	577	2,73	2065,01	1
1	158	403	2,55	2428,76	1
1	2	3	1,50	630,00	1
1	214	644	3,01	2599,39	1
1	19	61	3,21	1110,74	1
1	42	160	3,81	708,52	1
1	83	324	3,90	893,84	1
1	27	103	3,81	952,96	1
1	215	665	3,09	1809,60	1
1	82	273	3,33	1787,78	1
1	237	901	3,80	478,70	1
1	402	1538	3,83	644,43	1
1	113	351	3,11	577,09	1
1	181	672	3,71	333,29	1
1	256	654	2,55	3076,24	1
1	166	441	2,66	3681,24	1
1	171	455	2,66	4259,60	1
1	150	389	2,59	3060,63	1
1	198	514	2,60	2876,51	1
1	175	435	2,49	3110,89	1
1	152	345	2,27	3937,82	1
1	182	525	2,88	4840,56	1
1	255	690	2,71	3453,22	1
1	165	419	2,54	3542,40	1
1	162	489	3,02	4174,63	1
1	187	615	3,29	3118,30	1
1	182	585	3,21	930,31	1
1	261	824	3,16	1910,69	1
1	186	584	3,14	940,07	1
1	223	712	3,19	1105,23	1
1	182	576	3,16	579,25	1
1	231	829	3,59	751,98	1
1	56	178	3,18	942,50	1
1	6	20	3,33	11633,33	1
1	69	227	3,29	1336,09	1
1	159	533	3,35	3656,39	1
1	2	8	4,00	720,00	1
1	19	61	3,21	1301,58	1
1	79	300	3,80	708,57	1
0	0	0	0,00	0,00	0
1	120	392	3,27	2455,85	1
1	187	676	3,61	2230,81	1

1	2	8	4,00	2500,00	1
1	52	184	3,54	2609,08	1
1	46	172	3,74	3134,74	1
1	10	36	3,60	5437,00	1
1	290	852	2,94	1995,23	1
1	68	249	3,66	3712,31	1
1	230	773	3,36	691,96	1
1	248	706	2,85	3487,52	1
1	110	367	3,34	3415,64	1
1	182	585	3,21	4394,58	1
1	216	730	3,38	2603,72	1
1	145	446	3,08	3703,44	1
1	219	662	3,02	4065,06	1
1	30	98	3,27	3149,67	1
1	240	703	2,93	3800,22	1
1	180	474	2,63	3033,76	1
1	198	560	2,83	2707,25	1
1	15	50	3,33	816,00	1
1	162	565	3,49	823,46	1
1	14	60	4,29	627,43	1
1	197	567	2,88	2880,41	1
1	13	38	2,92	1393,08	1
1	160	464	2,90	3228,94	1
1	30	105	3,50	777,30	1
1	83	258	3,11	453,73	1
1	23	69	3,00	879,26	1
1	132	494	3,74	942,11	1
1	112	409	3,65	2552,53	1
0	0	0	0,00	0,00	0
0	0	0	0,00	0,00	0
1	103	350	3,40	1796,69	1
0	0	0	0,00	0,00	0
1	175	576	3,29	1471,75	1
1	320	1100	3,44	1522,82	1
1	354	1154	3,26	1354,79	1
1	179	571	3,19	2302,86	1
1	495	1782	3,60	1003,70	1
1	251	815	3,25	1025,78	1
1	232	764	3,29	1113,31	1
1	285	883	3,10	1110,46	1
1	282	897	3,18	1034,27	1
1	288	868	3,01	918,43	1
1	185	620	3,35	949,56	1
1	250	820	3,28	1296,95	1
1	256	847	3,31	1040,45	1
1	305	1060	3,48	752,78	1
1	320	1219	3,81	659,15	1
1	354	1287	3,64	551,04	1
1	356	1257	3,53	697,73	1
1	359	1428	3,98	864,65	1

1	113	386	3,42	645,81	1
1	204	689	3,38	659,03	1
1	240	768	3,20	716,24	1
1	147	469	3,19	1426,75	1
1	235	719	3,06	1580,53	1
1	214	660	3,08	1220,35	1
1	195	640	3,28	1310,78	1
1	312	1020	3,27	1496,23	1
1	271	941	3,47	1082,87	1
1	282	860	3,05	1561,08	1
1	320	1065	3,33	1322,49	1
1	464	1704	3,67	585,86	1
1	254	826	3,25	1024,42	1
1	294	1084	3,69	727,05	1
1	259	948	3,66	1407,05	1
1	306	1074	3,51	1403,15	1
1	364	1271	3,49	738,23	1
1	36	115	3,19	871,03	1
1	249	932	3,74	587,93	1
1	190	753	3,96	627,36	1
1	88	304	3,45	1101,43	1
1	183	616	3,37	607,32	1
1	349	1125	3,22	517,35	1
1	270	887	3,29	1754,39	1
1	253	828	3,27	1109,77	1
1	186	665	3,58	1125,22	1
1	183	597	3,26	924,43	1
1	140	470	3,36	1120,27	1
1	250	777	3,11	1543,21	1
1	174	559	3,21	1428,73	1
1	233	741	3,18	1265,80	1
0	0	0	0,00	0,00	0
1	280	885	3,16	1267,77	1
1	288	956	3,32	1354,53	1
1	189	645	3,41	1412,47	1
1	283	848	3,00	1255,76	1
1	284	896	3,15	1415,96	1
1	257	830	3,23	1683,35	1
1	225	727	3,23	1613,16	1
1	321	1123	3,50	1052,83	1
1	568	1707	3,01	875,73	1
1	216	808	3,74	1112,54	1
1	206	678	3,29	743,64	1
1	255	796	3,12	747,80	1
1	71	242	3,41	803,21	1
1	249	812	3,26	746,10	1
1	162	536	3,31	1103,88	1
1	154	575	3,73	332,15	1
1	137	433	3,16	946,62	1
1	61	191	3,13	907,93	1

1	311	998	3,21	1147,16	1
1	332	1116	3,36	1273,64	1
1	206	696	3,38	705,53	1
1	219	761	3,47	1272,40	1
1	237	797	3,36	1117,64	1
1	306	1035	3,38	1178,56	1
1	361	1219	3,38	693,37	1
1	240	750	3,13	759,78	1
1	299	970	3,24	1120,18	1
1	269	928	3,45	820,68	1
1	228	697	3,06	726,34	1
1	279	921	3,30	799,88	1
1	237	810	3,42	693,29	1
1	283	914	3,23	748,80	1
1	1	2	2,00	0,00	1
1	256	839	3,28	697,84	1
1	232	809	3,49	717,36	1
1	278	953	3,43	715,58	1
1	52	169	3,25	1283,33	1
1	275	820	2,98	939,96	1
1	151	464	3,07	1378,96	1
1	401	1212	3,02	1964,72	1
1	122	413	3,39	1546,10	1
1	304	1031	3,39	1705,59	1
1	96	335	3,49	1825,35	1
1	146	478	3,27	1814,47	1
1	199	628	3,16	4439,16	1
1	295	1016	3,44	1701,82	1
1	328	1065	3,25	1656,05	1
1	268	824	3,07	1500,47	1
1	181	576	3,18	1149,98	1
1	262	787	3,00	2038,08	1
1	143	482	3,37	1628,57	1
1	233	732	3,14	1449,50	1
1	283	863	3,05	1182,05	1
1	447	1403	3,14	1256,31	1
1	330	1104	3,35	920,13	1
1	313	998	3,19	1183,29	1
1	332	1062	3,20	1200,91	1
1	56	210	3,75	980,55	1
1	530	1888	3,56	804,79	1
1	202	769	3,81	617,41	1
1	250	917	3,67	813,07	1
1	285	955	3,35	1066,02	1
1	5	17	3,40	646,60	1
1	34	117	3,44	1020,79	1
1	158	540	3,42	692,78	1
1	163	582	3,57	852,87	1
1	179	607	3,39	694,91	1
1	410	1311	3,20	942,63	1

1	195	660	3,38	543,79	1
1	332	1140	3,43	533,48	1
1	267	875	3,28	998,53	1
1	300	1035	3,45	915,41	1
1	285	931	3,27	526,65	1
1	271	828	3,06	1087,58	1
1	328	1012	3,09	637,07	1
1	84	263	3,13	817,62	1
1	269	842	3,13	906,08	1
1	186	599	3,22	1742,74	1
1	212	675	3,18	1004,49	1
1	223	722	3,24	1820,09	1
1	257	811	3,16	905,00	1
1	281	947	3,37	1749,99	1
1	376	1212	3,22	2072,32	1
1	314	1060	3,38	1741,65	1
1	417	1327	3,18	2129,29	1
0	0	0	0,00	0,00	0
1	247	725	2,94	2426,95	1
1	249	797	3,20	2012,51	1
1	201	620	3,08	1974,50	1
1	168	543	3,23	2468,93	1
1	197	598	3,04	3723,67	1
1	265	802	3,03	3686,54	1
1	263	844	3,21	1677,39	1
1	143	439	3,07	978,19	1
1	175	556	3,18	1184,21	1
1	346	1108	3,20	1241,86	1
1	395	1234	3,12	1373,83	1
1	299	924	3,09	1297,38	1
1	203	639	3,15	871,70	1
1	272	847	3,11	934,34	1
1	245	857	3,50	865,00	1
1	308	1096	3,56	681,83	1
1	320	1092	3,41	622,69	1
1	55	185	3,36	892,15	1
1	255	805	3,16	939,64	1
1	309	1120	3,62	713,12	1
1	337	1115	3,31	1018,31	1
1	255	849	3,33	1176,04	1
1	318	1077	3,39	1281,62	1
1	110	328	2,98	1116,60	1
1	391	1297	3,32	1559,34	1
1	326	909	2,79	1323,24	1
1	313	883	2,82	1377,69	1
1	284	979	3,45	520,38	1
1	172	603	3,51	616,97	1
1	255	740	2,90	763,25	1
1	144	488	3,39	1093,02	1
1	120	364	3,03	768,22	1

1	57	197	3,46	679,18	1
1	312	1058	3,39	782,61	1
1	233	728	3,12	1115,09	1
0	0	0	0,00	0,00	0
1	217	720	3,32	1314,56	1
1	185	590	3,19	1430,17	1
1	206	691	3,35	1254,29	1
1	284	824	2,90	1228,40	1
1	196	616	3,14	1249,89	1
1	167	587	3,51	561,10	1
1	280	870	3,11	1694,21	1
1	281	921	3,28	1569,97	1
1	223	735	3,30	1571,64	1
1	181	608	3,36	846,23	1
1	282	864	3,06	993,55	1
1	147	511	3,48	630,56	1
1	199	661	3,32	640,93	1
1	122	436	3,57	367,88	1
1	332	1117	3,36	615,17	1
1	343	1210	3,53	618,73	1
1	42	153	3,64	916,45	1
1	104	384	3,69	406,36	1
1	79	242	3,06	788,33	1
1	164	540	3,29	1007,16	1
1	271	871	3,21	899,94	1
1	185	619	3,35	779,82	1
1	267	847	3,17	1116,86	1
1	227	752	3,31	963,47	1
1	75	287	3,83	721,05	1
1	329	1085	3,30	1062,73	1
1	167	535	3,20	532,07	1
1	236	748	3,17	985,14	1
1	171	513	3,00	840,07	1
1	114	327	2,87	1222,71	1
1	282	901	3,20	1220,20	1
1	357	1049	2,94	1046,60	1
1	285	951	3,34	1125,58	1
1	210	660	3,14	933,72	1
1	216	754	3,49	1318,79	1
1	334	1161	3,48	980,81	1
1	258	889	3,45	1003,50	1
1	131	412	3,15	1544,33	1
1	273	898	3,29	1191,46	1
1	259	802	3,10	1099,28	1
1	306	1016	3,32	749,02	1
1	221	732	3,31	1188,01	1
1	269	744	2,77	1105,03	1
1	337	1049	3,11	1212,23	1
1	280	1076	3,84	655,43	1
1	279	1070	3,84	299,89	1

1	287	903	3,15	959,57	1
1	354	1021	2,88	992,08	1
1	156	522	3,35	781,60	1
1	134	449	3,35	1014,54	1
1	221	712	3,22	990,52	1
1	283	962	3,40	921,06	1
1	179	533	2,98	1003,48	1
1	226	665	2,94	907,32	1
1	133	428	3,22	981,63	1
1	150	428	2,85	1212,54	1
1	202	599	2,97	1084,66	1
1	212	686	3,24	887,07	1
1	2	13	6,50	860,00	1
1	29	107	3,69	822,24	1
1	308	1080	3,51	980,95	1
1	135	444	3,29	799,85	1
1	243	805	3,31	1176,35	1
1	257	842	3,28	1057,74	1
1	186	649	3,49	801,28	1
1	204	665	3,26	1107,36	1
1	140	506	3,61	857,51	1
1	186	604	3,25	826,16	1
1	215	671	3,12	1115,75	1
1	193	601	3,11	878,84	1
1	223	714	3,20	1008,87	1
1	172	571	3,32	650,55	1
1	248	768	3,10	1040,21	1
1	244	806	3,30	1173,23	1
1	75	250	3,33	935,19	1
1	174	610	3,51	651,50	1
1	283	896	3,17	778,35	1
1	333	1050	3,15	837,08	1
1	351	1131	3,22	1021,37	1
1	331	1059	3,20	1073,10	1
1	301	988	3,28	897,33	1
1	223	686	3,08	638,71	1
1	106	376	3,55	1901,60	1
1	189	640	3,39	1163,63	1
1	132	467	3,54	1102,23	1
1	306	1020	3,33	1369,99	1
1	79	261	3,30	3263,57	1
0	0	0	0,00	0,00	0
1	213	671	3,15	1360,11	1
1	148	458	3,09	1031,20	1
1	97	332	3,42	1306,80	1
1	293	713	2,43	890,19	1
1	92	380	4,13	612,93	1
1	96	367	3,82	471,88	1
1	56	195	3,48	473,93	1
1	91	336	3,69	491,56	1

1	287	1001	3,49	938,13	1
1	99	322	3,25	636,65	1
1	215	701	3,26	796,47	1
1	33	129	3,91	1289,09	1
1	300	1029	3,43	700,53	1
1	228	651	2,86	1315,68	1
1	58	194	3,34	1430,60	1
1	328	1049	3,20	1336,48	1
1	244	873	3,58	565,98	1
1	213	711	3,34	1035,73	1
1	161	533	3,31	671,80	1
1	54	194	3,59	1618,33	1
1	32	120	3,75	937,34	1
1	91	321	3,53	669,18	1
1	155	544	3,51	727,96	1
1	155	511	3,30	712,32	1
1	191	672	3,52	2049,06	1
1	120	377	3,14	1480,25	1
1	205	749	3,65	1524,07	1
1	298	928	3,11	1559,57	1
1	174	583	3,35	1114,10	1
1	145	520	3,59	524,25	1
1	101	320	3,17	671,73	1
1	266	868	3,26	2603,12	1
1	209	637	3,05	1327,07	1
1	267	827	3,10	1287,74	1
1	45	192	4,27	420,38	1
1	59	181	3,07	1148,93	1
1	300	1084	3,61	743,47	1
1	117	391	3,34	993,38	1
1	399	1335	3,35	1552,18	1
1	18	44	2,44	1126,72	1
1	29	96	3,31	621,03	1
1	54	181	3,35	679,52	1
1	108	392	3,63	764,84	1
1	415	1361	3,28	1229,52	1
1	251	835	3,33	775,22	1
1	165	600	3,64	1361,89	1
1	35	125	3,57	788,29	1
1	89	314	3,53	894,90	1
1	173	617	3,57	577,98	1
1	151	532	3,52	994,79	1
1	96	298	3,10	615,42	1
1	177	589	3,33	685,29	1
1	15	55	3,67	1243,67	1
0	0	0	0,00	0,00	0
1	176	644	3,66	483,90	1
1	211	694	3,29	737,15	1
1	200	653	3,27	641,25	1
1	10	24	2,40	1800,00	1

1	103	338	3,28	1824,58	1
1	119	380	3,19	1516,06	1
1	76	236	3,11	930,38	1
1	319	999	3,13	976,84	1
1	153	423	2,76	1285,56	1
1	259	952	3,68	668,29	1
1	64	163	2,55	701,56	1
1	250	639	2,56	1143,08	1
1	133	355	2,67	583,91	1
1	71	245	3,45	712,39	1
1	26	81	3,12	965,38	1
1	106	367	3,46	666,83	1
1	54	181	3,35	760,85	1
1	200	663	3,32	930,91	1
1	174	547	3,14	674,29	1
1	159	587	3,69	451,26	1
1	150	546	3,64	694,24	1
1	225	623	2,77	1199,66	1
1	351	1197	3,41	795,70	1
1	148	538	3,64	868,28	1
1	272	1001	3,68	630,03	1
1	171	582	3,40	635,92	1
1	225	774	3,44	993,49	1
1	292	995	3,41	920,22	1
1	151	485	3,21	997,32	1
1	21	87	4,14	495,95	1
1	57	179	3,14	892,30	1
1	147	483	3,29	1454,32	1
1	271	882	3,25	4124,75	1
1	172	537	3,12	1564,39	1
1	53	168	3,17	1122,87	1
1	57	159	2,79	1096,58	1
1	40	124	3,10	1303,63	1
1	70	228	3,26	987,54	1
1	153	476	3,11	851,54	1
1	168	586	3,49	936,65	1
1	141	443	3,14	1485,96	1
1	245	797	3,25	1149,09	1
1	62	208	3,35	643,45	1
1	362	1207	3,33	1486,59	1
1	66	215	3,26	738,41	1
1	57	182	3,19	563,46	1
1	66	206	3,12	1204,56	1
1	45	154	3,42	793,38	1
1	101	391	3,87	545,54	1
1	31	100	3,23	864,90	1
1	77	244	3,17	818,64	1
1	72	250	3,47	903,57	1
1	168	505	3,01	743,45	1
1	190	599	3,15	991,77	1

1	185	630	3,41	844,65	1
1	192	630	3,28	551,61	1
1	133	435	3,27	1002,86	1
1	89	273	3,07	742,63	1
1	13	52	4,00	1442,31	1
1	59	219	3,71	733,41	1
1	44	151	3,43	655,00	1
1	200	712	3,56	899,08	1
1	107	393	3,67	1073,54	1
0	0	0	0,00	0,00	0
1	76	269	3,54	1211,51	1
1	189	654	3,46	650,14	1
1	185	561	3,03	1142,76	1
1	144	503	3,49	1191,55	1
1	65	233	3,58	964,38	1
1	234	892	3,81	742,29	1
1	18	63	3,50	872,78	1
1	242	757	3,13	518,51	1
1	285	868	3,05	1039,15	1
1	223	855	3,83	783,07	1
1	122	435	3,57	740,52	1
1	339	1133	3,34	724,63	1
1	127	410	3,23	1104,31	1
1	167	597	3,57	626,56	1
1	219	765	3,49	1021,20	1
1	45	131	2,91	912,22	1
1	40	125	3,13	1006,05	1
1	218	620	2,84	1330,54	1
1	221	771	3,49	1159,24	1
1	47	166	3,53	1067,26	1
1	179	598	3,34	601,42	1
1	54	188	3,48	553,89	1
1	93	308	3,31	1096,14	1
1	115	417	3,63	509,14	1
1	93	322	3,46	934,35	1
1	255	866	3,40	627,36	1
1	262	915	3,49	1891,53	1
0	0	0	0,00	0,00	0
1	144	502	3,49	685,54	1
1	16	71	4,44	1376,88	1
1	34	114	3,35	1425,65	1
1	73	262	3,59	672,12	1
1	74	262	3,54	1013,34	1
1	63	299	4,75	631,43	1
1	54	201	3,72	507,50	1
1	65	201	3,09	669,15	1
1	129	417	3,23	643,40	1
1	8	30	3,75	848,13	1
1	39	123	3,15	920,26	1
1	54	187	3,46	759,96	1

1	5	19	3,80	862,00	1
1	381	1279	3,36	830,87	1
1	307	1105	3,60	761,78	1
1	242	815	3,37	898,11	1
1	110	404	3,67	987,78	1
1	213	662	3,11	896,84	1
1	172	583	3,39	665,38	1
1	301	1003	3,33	1132,16	1
1	71	232	3,27	878,23	1
1	332	1116	3,36	960,57	1
1	223	721	3,23	910,79	1
1	186	587	3,16	497,61	1
1	232	715	3,08	585,17	1
1	262	862	3,29	975,16	1
1	1	4	4,00	500,00	1
1	339	1145	3,38	820,03	1
1	120	391	3,26	780,28	1
1	216	697	3,23	902,89	1
1	232	794	3,42	952,62	1
1	227	715	3,15	885,02	1
1	219	739	3,37	674,52	1
1	436	1532	3,51	675,50	1
1	263	909	3,46	613,64	1
1	278	938	3,37	574,98	1
1	117	439	3,75	633,65	1
1	164	538	3,28	629,71	1
1	352	1086	3,09	1291,52	1
1	429	1460	3,40	526,67	1
1	231	754	3,26	831,76	1
1	97	322	3,32	592,07	1
1	263	960	3,65	675,56	1
1	321	1134	3,53	837,35	1
1	212	733	3,46	598,18	1
1	107	387	3,62	730,85	1
1	154	563	3,66	839,77	1
1	214	787	3,68	641,07	1
1	304	1017	3,35	918,82	1
1	354	1012	2,86	1143,35	1
1	123	399	3,24	818,89	1
1	360	1140	3,17	1009,79	1
1	325	1077	3,31	995,45	1
1	383	1268	3,31	1386,21	1
1	387	1354	3,50	792,06	1
1	336	1062	3,16	874,58	1
1	140	477	3,41	808,56	1
1	263	886	3,37	863,37	1
1	291	943	3,24	928,65	1
1	263	863	3,28	793,34	1
1	343	1110	3,24	667,30	1
1	255	799	3,13	1112,96	1

1	257	866	3,37	879,35	1
1	370	1208	3,26	915,75	1
1	250	764	3,06	1034,34	1
1	250	817	3,27	883,15	1
1	310	1030	3,32	681,36	1
1	258	850	3,29	848,96	1
1	225	685	3,04	859,15	1
1	293	927	3,16	1423,12	1
1	167	567	3,40	1082,49	1
1	131	420	3,21	588,82	1
1	363	1199	3,30	947,12	1
1	172	598	3,48	882,81	1
1	219	723	3,30	838,81	1
1	169	609	3,60	900,08	1
1	219	768	3,51	670,34	1
1	205	760	3,71	612,27	1
1	179	641	3,58	488,18	1
1	208	663	3,19	676,42	1
1	426	1538	3,61	873,56	1
1	209	645	3,09	907,82	1
1	232	742	3,20	880,25	1
1	222	715	3,22	905,88	1
1	210	700	3,33	964,95	1
1	117	394	3,37	859,91	1
1	235	778	3,31	1042,35	1
1	286	918	3,21	1007,20	1
1	294	947	3,22	1156,47	1
1	280	939	3,35	1069,53	1
1	162	507	3,13	866,35	1
1	257	895	3,48	1316,56	1
1	284	944	3,32	817,27	1
1	209	697	3,33	817,42	1
1	334	1130	3,38	1281,04	1
1	254	802	3,16	1442,98	1
1	227	702	3,09	1982,18	1
1	291	925	3,18	1893,53	1
1	211	640	3,03	1137,45	1
1	286	855	2,99	1279,60	1
1	193	597	3,09	1275,67	1
1	263	954	3,63	1707,08	1
1	333	1191	3,58	705,08	1
1	199	644	3,24	732,42	1
1	222	754	3,40	849,98	1
1	191	689	3,61	957,40	1
1	207	680	3,29	840,16	1
1	175	551	3,15	1052,89	1
1	100	317	3,17	1441,90	1
1	315	1028	3,26	1156,10	1
1	193	655	3,39	1190,93	1
1	233	731	3,14	1393,48	1

1	64	218	3,41	954,56	1
1	307	1072	3,49	660,13	1
1	254	884	3,48	681,83	1
1	349	1258	3,60	909,12	1
1	332	1072	3,23	1538,71	1
1	164	572	3,49	950,27	1
1	219	722	3,30	1164,37	1
1	282	890	3,16	1159,06	1
1	329	1120	3,40	1114,27	1
1	256	845	3,30	2179,89	1
1	117	380	3,25	891,49	1
1	14	49	3,50	1137,14	1
1	55	159	2,89	544,65	1
1	81	274	3,38	529,38	1
1	124	447	3,60	300,55	1
1	161	532	3,30	837,59	1
1	26	97	3,73	1115,19	1
1	88	269	3,06	857,98	1
1	193	622	3,22	949,98	1
1	110	359	3,26	1105,49	1
1	203	726	3,58	639,57	1
1	27	77	2,85	1117,89	1
1	331	1141	3,45	1326,90	1
1	237	739	3,12	1015,57	1
1	173	566	3,27	811,72	1
1	207	676	3,27	765,78	1
1	64	210	3,28	794,14	1
1	158	464	2,94	879,32	1
1	184	607	3,30	936,96	1
1	10	34	3,40	805,00	1
1	123	403	3,28	627,55	1
1	64	223	3,48	1500,63	1
1	160	543	3,39	993,00	1
1	143	479	3,35	1395,00	1
1	138	441	3,20	741,40	1
1	475	1490	3,14	1025,16	1
1	65	192	2,95	877,25	1
1	66	227	3,44	462,82	1
1	27	106	3,93	823,63	1
1	9	28	3,11	823,33	1
1	280	932	3,33	1069,66	1
1	182	587	3,23	717,08	1
1	77	245	3,18	750,83	1
1	180	653	3,63	795,58	1
1	45	144	3,20	888,56	1
1	46	162	3,52	787,28	1
1	81	258	3,19	789,44	1
1	86	279	3,24	644,88	1
1	83	346	4,17	657,36	1
1	97	365	3,76	521,71	1

1	252	659	2,62	1209,49	1
1	56	161	2,88	540,46	1
1	248	759	3,06	485,55	1
1	24	89	3,71	628,63	1
1	40	129	3,23	1163,93	1
0	0	0	0,00	0,00	0
1	28	101	3,61	1556,79	1
1	180	573	3,18	832,31	1
1	160	489	3,06	898,29	1
1	155	538	3,47	1434,63	1
1	159	588	3,70	773,10	1
1	43	150	3,49	760,42	1
1	195	667	3,42	1093,17	1
1	153	521	3,41	963,67	1
1	241	863	3,58	823,61	1
1	76	282	3,71	627,38	1
1	203	760	3,74	504,35	1
1	719	2570	3,57	1012,61	1
1	17	68	4,00	731,76	1
1	194	551	2,84	890,71	1
1	115	384	3,34	1011,78	1
1	249	797	3,20	668,51	1
1	148	436	2,95	1133,98	1
1	194	639	3,29	1117,84	1
1	67	234	3,49	1002,88	1
1	212	691	3,26	1183,76	1
1	286	990	3,46	1274,22	1
1	202	632	3,13	782,21	1
1	136	445	3,27	1044,74	1
1	46	142	3,09	1502,39	1
1	169	557	3,30	1051,10	1
1	55	194	3,53	500,84	1
1	199	699	3,51	677,84	1
1	1	3	3,00	3500,00	1
1	219	825	3,77	684,00	1
1	55	204	3,71	855,69	1
1	236	767	3,25	968,03	1
1	129	450	3,49	906,09	1
1	136	480	3,53	675,33	1
1	186	643	3,46	1154,77	1
1	194	650	3,35	887,44	1
1	51	159	3,12	984,18	1
1	131	436	3,33	1685,27	1
1	146	513	3,51	1426,55	1
1	294	787	2,68	1155,42	1
1	321	809	2,52	1114,21	1
1	1	5	5,00	1800,00	1
1	94	315	3,35	599,83	1
1	149	449	3,01	1159,46	1
1	319	1133	3,55	689,53	1

1	247	797	3,23	958,85	1
1	228	733	3,21	1136,85	1
1	277	917	3,31	1240,72	1
1	221	706	3,19	1144,89	1
1	163	539	3,31	1066,12	1
1	190	640	3,37	1035,55	1
1	250	851	3,40	957,68	1
1	247	754	3,05	1296,74	1
1	85	273	3,21	988,58	1
1	68	246	3,62	1080,50	1
1	276	1038	3,76	655,95	1
1	385	1369	3,56	731,11	1
1	362	1299	3,59	695,23	1
1	230	819	3,56	644,43	1
1	260	980	3,77	647,27	1
1	211	795	3,77	613,09	1
1	304	1002	3,30	898,55	1
1	189	639	3,38	1072,71	1
1	184	611	3,32	1030,61	1
1	216	690	3,19	954,82	1
1	352	1136	3,23	1538,52	1
1	261	867	3,32	1447,33	1
1	247	673	2,72	1396,79	1
1	222	722	3,25	1339,23	1
1	222	686	3,09	1870,00	1
1	320	963	3,01	2239,90	1
1	263	898	3,41	1064,88	1
1	367	1208	3,29	1251,86	1
1	441	1449	3,29	1102,09	1
1	244	833	3,41	592,74	1
1	169	573	3,39	990,13	1
1	238	831	3,49	1169,94	1
1	406	1622	4,00	696,84	1
1	186	654	3,52	1096,01	1
1	295	1084	3,67	654,99	1
1	304	1178	3,88	542,92	1
1	279	1005	3,60	663,06	1
1	166	589	3,55	672,30	1
2	15	55	3,67	405,00	2
1	385	1272	3,30	1025,89	1
1	274	914	3,34	1275,05	1
1	299	907	3,03	1136,78	1
1	304	986	3,24	1115,78	1
1	337	1082	3,21	1600,00	1
1	291	892	3,07	1216,81	1
1	284	908	3,20	1096,31	1
1	278	927	3,33	1602,24	1
1	243	810	3,33	1124,90	1
1	139	459	3,30	1375,24	1
1	320	978	3,06	2202,30	1

1	596	1807	3,03	2755,19	1
1	205	597	2,91	2335,82	1
1	303	843	2,78	2655,82	1
1	70	240	3,43	1312,71	1
1	120	394	3,28	1526,28	1
1	258	804	3,12	1966,94	1
1	245	649	2,65	1502,99	1
1	149	420	2,82	1363,19	1
1	217	701	3,23	1078,94	1
1	127	396	3,12	718,07	1
1	309	1086	3,51	646,54	1
1	218	781	3,58	711,13	1
1	68	233	3,43	788,90	1
1	279	987	3,54	528,84	1
1	218	717	3,29	1015,51	1
1	239	795	3,33	1129,31	1
1	334	1135	3,40	1113,71	1
1	105	368	3,50	1057,46	1
1	295	916	3,11	1212,13	1
1	340	1153	3,39	810,70	1
1	186	614	3,30	829,34	1
1	193	631	3,27	972,32	1
1	223	749	3,36	1089,07	1
1	318	1083	3,41	1500,12	1
1	395	1191	3,02	1056,51	1
1	320	1033	3,23	1251,42	1
1	189	626	3,31	2200,20	1
1	261	753	2,89	1971,63	1
1	201	642	3,19	1175,55	1
1	178	546	3,07	1197,13	1
1	181	645	3,56	528,62	1
1	338	954	2,82	1024,16	1
1	203	634	3,12	923,24	1
1	101	331	3,28	1101,14	1
1	238	899	3,78	459,25	1
1	191	650	3,40	867,73	1
1	325	1136	3,50	768,14	1
1	165	559	3,39	442,18	1
1	290	989	3,41	1014,37	1
1	166	544	3,28	1008,67	1
1	215	705	3,28	1214,35	1
1	112	411	3,67	1030,94	1
1	419	1383	3,30	997,85	1
1	325	1218	3,75	883,97	1
1	262	975	3,72	658,16	1
1	252	923	3,66	525,02	1
1	242	919	3,80	633,69	1
1	319	1130	3,54	493,93	1
1	191	651	3,41	989,66	1
1	310	1007	3,25	1005,98	1

1	337	1030	3,06	1041,00	1
1	356	1174	3,30	1171,73	1
1	311	1046	3,36	1143,46	1
1	348	1166	3,35	439,09	1
1	463	1518	3,28	450,37	1
1	277	870	3,14	952,30	1
1	274	785	2,86	2084,83	1
1	395	1206	3,05	2109,61	1
0	0	0	0,00	0,00	0
1	177	606	3,42	793,97	1
1	194	618	3,19	859,73	1
1	340	1134	3,34	1244,06	1
1	294	983	3,34	1222,02	1
1	217	731	3,37	980,89	1
1	264	887	3,36	1195,55	1
1	236	785	3,33	1065,79	1
1	267	828	3,10	1169,40	1
1	149	483	3,24	922,02	1
1	341	1024	3,00	1218,15	1
1	370	1202	3,25	928,71	1
1	330	1078	3,27	860,91	1
1	282	915	3,24	933,70	1
1	308	1052	3,42	684,35	1
1	111	369	3,32	898,52	1
1	243	774	3,19	660,33	1
1	220	797	3,62	761,04	1
1	327	1013	3,10	943,05	1
1	233	752	3,23	641,82	1
1	215	710	3,30	604,58	1
1	342	1127	3,30	733,94	1
1	236	824	3,49	400,89	1
1	49	155	3,16	1148,41	1
1	175	587	3,35	950,69	1
1	216	670	3,10	1122,13	1
1	237	782	3,30	1996,35	1
1	256	863	3,37	808,67	1
1	202	675	3,34	885,17	1
1	292	972	3,33	1347,29	1
1	276	885	3,21	852,22	1
1	326	1153	3,54	1021,15	1
1	288	980	3,40	1042,28	1
1	200	655	3,28	876,52	1
1	32	84	2,63	692,91	1
1	332	1043	3,14	902,57	1
1	143	506	3,54	1086,79	1
1	199	519	2,61	2303,27	1
1	40	120	3,00	2191,45	1
1	215	706	3,28	1292,58	1
1	205	634	3,09	911,20	1
1	159	494	3,11	1227,83	1

1	233	803	3,45	1065,60	1
1	153	461	3,01	754,77	1
1	101	305	3,02	792,55	1
1	106	376	3,55	677,72	1
1	270	934	3,46	677,54	1
1	255	838	3,29	578,08	1
1	128	382	2,98	924,61	1
1	120	398	3,32	1161,74	1
1	372	1165	3,13	1849,92	1
1	307	993	3,23	1637,63	1
1	255	715	2,80	2227,66	1
1	146	437	2,99	2300,01	1
1	207	672	3,25	1892,91	1
1	199	629	3,16	3022,15	1
1	212	638	3,01	2475,13	1
1	309	1206	3,90	1278,53	1
1	130	424	3,26	4890,23	1
1	195	648	3,32	891,26	1
1	288	918	3,19	2838,91	1
1	329	1044	3,17	3499,95	1
1	213	668	3,14	2582,21	1
1	125	382	3,06	2244,50	1
1	244	831	3,41	1469,80	1
1	272	847	3,11	1345,90	1
1	213	617	2,90	2259,31	1
1	250	767	3,07	1542,84	1
1	226	718	3,18	2508,92	1
1	145	428	2,95	2168,54	1
1	266	835	3,14	1977,82	1
1	449	1311	2,92	1840,31	1
1	430	1436	3,34	648,22	1
1	155	584	3,77	579,66	1
1	185	583	3,15	836,93	1
1	240	827	3,45	1376,72	1
1	294	1009	3,43	1416,53	1
1	257	876	3,41	1141,52	1
1	244	841	3,45	737,13	1
1	419	1490	3,56	1016,91	1
1	263	915	3,48	889,26	1
1	250	774	3,10	1662,25	1
1	171	591	3,46	632,81	1
1	324	1091	3,37	1284,39	1
1	380	1310	3,45	1796,13	1
1	312	1000	3,21	1204,88	1
1	170	532	3,13	586,38	1
1	258	823	3,19	846,72	1
1	290	971	3,35	1001,41	1
1	311	968	3,11	1004,82	1
1	362	1223	3,38	791,31	1
1	400	1344	3,36	874,90	1

1	235	836	3,56	1021,02	1
1	346	1132	3,27	829,86	1
1	247	851	3,45	1007,72	1
1	259	986	3,81	638,01	1
1	340	1306	3,84	495,41	1
2	43	146	3,40	806,56	2
1	173	575	3,32	1064,51	1
2	1	2	2,00	510,00	2
1	322	1069	3,32	731,48	1
1	282	925	3,28	791,58	1
1	312	1042	3,34	387,09	1
1	111	373	3,36	526,15	1
1	145	553	3,81	298,41	1
1	249	820	3,29	1052,32	1
1	155	481	3,10	1242,09	1
1	107	362	3,38	1815,35	1
1	71	250	3,52	2427,27	1
1	81	299	3,69	1521,11	1
1	177	621	3,51	1315,30	1
1	29	122	4,21	798,28	1
1	105	358	3,41	2127,29	1
1	80	196	2,45	1375,51	1
1	336	984	2,93	1460,44	1
1	96	341	3,55	830,33	1
1	54	183	3,39	1000,28	1
1	228	916	4,02	666,45	1
1	134	496	3,70	1070,61	1
1	57	185	3,25	1066,84	1
1	44	158	3,59	903,75	1
1	134	437	3,26	855,66	1
1	137	546	3,99	435,45	1
1	75	249	3,32	1620,72	1
1	74	251	3,39	909,04	1
1	137	477	3,48	648,02	1
1	67	233	3,48	1158,09	1
1	145	458	3,16	693,80	1
1	249	865	3,47	798,48	1
1	160	604	3,78	782,82	1
1	134	433	3,23	969,12	1
1	255	851	3,34	567,09	1
1	139	438	3,15	907,30	1
1	18	66	3,67	770,00	1
1	27	104	3,85	964,30	1
1	36	122	3,39	1323,22	1
1	30	88	2,93	737,00	1
1	3	10	3,33	340,00	1
1	31	90	2,90	1070,84	1
1	35	126	3,60	1021,26	1
1	127	382	3,01	2305,57	1
1	32	84	2,63	807,50	1

1	256	821	3,21	777,80	1
1	409	1432	3,50	638,21	1
1	333	1217	3,65	664,28	1
1	221	813	3,68	550,03	1
1	196	620	3,16	634,16	1
1	346	1121	3,24	931,08	1
1	323	1056	3,27	1014,29	1
1	138	455	3,30	1078,92	1
1	34	109	3,21	1184,29	1
1	40	137	3,43	1295,95	1
1	133	469	3,53	586,28	1
1	57	193	3,39	730,89	1
1	195	662	3,39	1068,00	1
1	43	131	3,05	1816,21	1
1	56	175	3,13	1540,75	1
1	157	570	3,63	802,85	1
1	124	437	3,52	1147,27	1
1	120	368	3,07	1250,29	1
1	77	252	3,27	706,66	1
1	52	171	3,29	881,73	1
1	79	230	2,91	1064,44	1
1	53	179	3,38	952,72	1
1	85	277	3,26	648,68	1
1	63	216	3,43	909,92	1
1	46	177	3,85	405,22	1
1	91	309	3,40	675,25	1
1	78	265	3,40	751,88	1
1	75	226	3,01	797,49	1
1	94	306	3,26	865,00	1
1	93	289	3,11	920,09	1
1	33	117	3,55	1271,82	1
1	246	906	3,68	951,59	1
1	63	195	3,10	1093,62	1
1	204	712	3,49	972,24	1
1	222	817	3,68	677,50	1
1	129	458	3,55	837,02	1
1	80	259	3,24	630,11	1
1	171	612	3,58	556,37	1
1	135	450	3,33	730,50	1
1	164	568	3,46	853,45	1
1	41	136	3,32	1520,73	1
1	106	343	3,24	1610,86	1
1	59	212	3,59	2391,44	1
1	76	213	2,80	1223,62	1
1	252	889	3,53	823,75	1
1	195	589	3,02	970,88	1
1	148	449	3,03	740,45	1
1	135	415	3,07	1123,87	1
1	205	613	2,99	1156,24	1
1	249	713	2,86	1459,66	1

1	232	883	3,81	600,81	1
1	292	825	2,83	1383,69	1
1	208	626	3,01	1289,85	1
1	49	129	2,63	1002,65	1
1	181	460	2,54	1017,73	1
1	172	435	2,53	1088,27	1
1	201	573	2,85	1319,53	1
1	464	1538	3,31	1020,87	1
1	183	601	3,28	727,67	1
1	257	945	3,68	667,64	1
1	212	777	3,67	534,31	1
1	315	1063	3,37	642,04	1
1	191	576	3,02	951,57	1
1	184	621	3,38	635,42	1
1	27	78	2,89	818,52	1
1	21	82	3,90	454,76	1
1	199	554	2,78	1508,85	1
1	188	622	3,31	1281,73	1
1	274	671	2,45	1040,44	1
1	166	515	3,10	2115,36	1
1	140	420	3,00	1062,85	1
1	197	632	3,21	1080,08	1
1	244	763	3,13	857,32	1
1	210	642	3,06	1138,65	1
1	175	554	3,17	969,46	1
1	246	804	3,27	904,42	1
1	200	595	2,98	928,82	1
1	336	1106	3,29	590,15	1
1	77	243	3,16	860,78	1
1	184	504	2,74	2509,96	1
1	238	717	3,01	1259,90	1
1	228	689	3,02	2653,13	1
1	230	659	2,87	1951,77	1
1	96	320	3,33	788,51	1
1	80	343	4,29	480,99	1
1	203	746	3,67	576,34	1
1	76	273	3,59	584,12	1
1	86	273	3,17	508,60	1
1	79	259	3,28	557,72	1
1	272	933	3,43	906,38	1
1	26	102	3,92	906,92	1
1	53	177	3,34	912,94	1
1	21	80	3,81	948,57	1
1	6	21	3,50	621,67	1
1	11	44	4,00	928,18	1
1	26	118	4,54	687,92	1
1	21	75	3,57	1714,29	1
1	36	114	3,17	1003,36	1
1	0	0	0,00	0,00	1
1	45	133	2,96	797,53	1

1	168	529	3,15	751,74	1
0	0	0	0,00	0,00	0
1	108	383	3,55	2869,02	1
1	191	613	3,21	3840,27	1
1	343	1119	3,26	3449,01	1
1	252	780	3,10	2872,07	1
1	310	979	3,16	2937,06	1
1	303	997	3,29	2345,79	1
1	192	608	3,17	2360,89	1
1	172	540	3,14	2522,20	1
1	287	892	3,11	2814,29	1
1	204	630	3,09	4492,87	1
1	286	876	3,06	2614,72	1
1	265	829	3,13	2254,46	1
1	137	427	3,12	3017,52	1
1	149	496	3,33	2104,74	1
1	304	958	3,15	2481,55	1
1	212	669	3,16	2405,59	1
1	282	851	3,02	2716,65	1
1	199	582	2,92	2357,93	1
1	347	1110	3,20	2642,63	1
1	194	658	3,39	1864,85	1
1	246	829	3,37	2781,56	1
1	207	606	2,93	2197,86	1
1	193	616	3,19	1604,56	1
1	93	311	3,34	1128,34	1
1	232	608	2,62	2078,11	1
1	253	817	3,23	3921,79	1
1	246	775	3,15	3286,35	1
1	133	443	3,33	2263,76	1
1	225	658	2,92	2921,03	1
1	269	874	3,25	3165,16	1
1	253	816	3,23	3192,18	1
1	265	848	3,20	3765,80	1
1	283	838	2,96	1984,30	1
1	64	256	4,00	432,58	1
1	308	919	2,98	3421,68	1
1	125	404	3,23	2743,10	1
1	236	686	2,91	2521,09	1
1	2	9	4,50	2350,00	1
1	191	674	3,53	4617,24	1
1	237	596	2,51	2583,78	1
1	86	284	3,30	2493,90	1
1	113	339	3,00	1807,88	1
1	183	559	3,05	2505,13	1
1	171	497	2,91	3197,37	1
1	150	438	2,92	2565,77	1
1	42	134	3,19	2637,14	1
1	315	901	2,86	4592,23	1
1	61	171	2,80	1900,13	1

1	97	245	2,53	769,71	1
1	306	919	3,00	3079,05	1
1	320	1152	3,60	4857,65	1
1	277	778	2,81	2712,18	1
1	258	776	3,01	3606,73	1
1	66	248	3,76	720,26	1
1	94	323	3,44	665,28	1
1	150	408	2,72	2976,25	1
1	24	78	3,25	2765,25	1
1	168	556	3,31	1384,03	1
1	132	451	3,42	1993,48	1
1	203	517	2,55	2026,04	1
1	33	99	3,00	2583,48	1
1	142	355	2,50	1792,08	1
1	244	608	2,49	1964,50	1
1	69	220	3,19	694,09	1
1	78	256	3,28	638,63	1
1	255	770	3,02	1156,16	1
1	145	486	3,35	1047,71	1
1	235	757	3,22	514,01	1
1	319	1042	3,27	1146,08	1
1	236	817	3,46	1311,75	1
1	245	756	3,09	1118,27	1
1	80	270	3,38	1120,90	1
1	163	546	3,35	1982,87	1
1	285	903	3,17	989,35	1
1	292	928	3,18	936,76	1
1	343	1069	3,12	827,43	1
1	197	681	3,46	927,28	1
1	322	958	2,98	857,74	1
1	180	611	3,39	1274,49	1
1	352	1096	3,11	986,14	1
1	127	435	3,43	1505,12	1
1	235	777	3,31	1022,25	1
1	87	325	3,74	1618,48	1
1	241	835	3,46	1044,64	1
1	220	766	3,48	1310,81	1
1	94	340	3,62	903,72	1
1	300	1016	3,39	1681,76	1
1	275	938	3,41	1290,79	1
1	396	1261	3,18	2135,92	1
1	229	770	3,36	1651,55	1
1	130	441	3,39	1666,17	1
1	211	688	3,26	1302,27	1
1	302	1072	3,55	898,62	1
1	182	598	3,29	890,20	1
1	240	811	3,38	854,13	1
1	202	608	3,01	1120,63	1
1	279	961	3,44	1008,45	1
1	97	326	3,36	668,67	1

1	213	672	3,15	934,69	1
1	206	671	3,26	942,65	1
1	193	656	3,40	1478,83	1
1	187	652	3,49	902,80	1
1	219	712	3,25	942,08	1
1	348	1122	3,22	986,65	1
1	183	637	3,48	877,71	1
1	328	1034	3,15	854,27	1
1	163	525	3,22	1149,31	1
1	219	717	3,27	943,92	1
1	338	1123	3,32	1229,23	1
1	84	278	3,31	921,19	1
1	258	853	3,31	1052,47	1
1	281	906	3,22	1148,37	1
1	181	636	3,51	1165,69	1
1	196	619	3,16	1323,40	1
1	228	699	3,07	1219,96	1
1	256	801	3,13	1121,04	1
1	222	692	3,12	1656,96	1
1	253	860	3,40	1558,45	1
1	486	1507	3,10	2248,73	1
1	315	1007	3,20	2435,23	1
1	302	984	3,26	1869,00	1
1	194	585	3,02	964,26	1
1	184	497	2,70	1166,92	1
1	292	956	3,27	1261,35	1
1	329	1086	3,30	1940,49	1
1	213	640	3,00	1988,17	1
1	389	1138	2,93	1676,57	1
1	318	1051	3,31	966,65	1
1	349	1127	3,23	1237,66	1
1	27	91	3,37	699,33	1
1	40	125	3,13	812,30	1
1	253	800	3,16	1174,40	1
1	198	575	2,90	1332,26	1
1	190	611	3,22	969,07	1
1	215	750	3,49	579,83	1
1	254	886	3,49	642,79	1
1	77	250	3,25	1065,44	1
1	148	455	3,07	1045,45	1
1	178	614	3,45	722,58	1
1	299	964	3,22	1012,16	1
1	42	138	3,29	768,60	1
1	242	802	3,31	1030,46	1
1	72	214	2,97	970,33	1
1	391	1306	3,34	518,05	1
1	240	779	3,25	1031,42	1
1	295	1025	3,47	1265,74	1
1	260	855	3,29	1026,93	1
1	327	1092	3,34	1310,66	1

1	106	390	3,68	581,49	1
1	253	904	3,57	799,81	1
1	174	576	3,31	547,36	1
1	203	670	3,30	751,85	1
1	155	502	3,24	379,30	1
1	199	654	3,29	398,29	1
1	314	1012	3,22	677,12	1
1	290	881	3,04	1047,67	1
1	290	955	3,29	1279,50	1
1	264	858	3,25	900,69	1
1	353	1160	3,29	1087,74	1
1	383	1193	3,11	1101,79	1
1	246	793	3,22	854,24	1
1	222	727	3,27	932,05	1
1	47	172	3,66	1125,19	1
1	208	683	3,28	664,45	1
1	190	625	3,29	440,29	1
1	259	883	3,41	1097,74	1
1	32	103	3,22	659,53	1
1	204	743	3,64	957,29	1
1	204	765	3,75	847,08	1
1	200	738	3,69	858,63	1
1	224	718	3,21	976,02	1
1	242	607	2,51	1216,65	1
1	355	1016	2,86	1070,12	1
1	337	1098	3,26	1043,96	1
1	186	602	3,24	1179,60	1
1	336	1136	3,38	884,16	1
1	102	355	3,48	1248,96	1
1	191	637	3,34	1238,09	1
1	167	537	3,22	1059,50	1
1	262	716	2,73	2146,97	1
1	217	468	2,16	985,39	1
1	302	921	3,05	1012,36	1
1	190	641	3,37	458,06	1
1	170	643	3,78	671,95	1
1	342	1199	3,51	640,21	1
1	331	1022	3,09	1474,18	1
1	234	728	3,11	2127,45	1
1	185	594	3,21	636,29	1
1	79	266	3,37	594,10	1
1	194	669	3,45	442,63	1
1	252	761	3,02	1855,89	1
1	268	885	3,30	1055,31	1
1	251	605	2,41	1846,81	1
1	191	599	3,14	1630,39	1
1	381	1232	3,23	1462,92	1
1	367	1111	3,03	1232,80	1
1	413	1301	3,15	1731,99	1
1	349	1153	3,30	2003,04	1

1	311	986	3,17	1867,23	1
1	177	530	2,99	1896,24	1
1	280	976	3,49	1735,83	1
1	106	374	3,53	1332,44	1
1	101	338	3,35	445,61	1
1	48	167	3,48	1047,75	1
1	187	667	3,57	1295,71	1
1	184	606	3,29	1335,63	1
1	367	1219	3,32	1459,64	1
1	222	683	3,08	1182,43	1
1	262	855	3,26	950,64	1
1	423	1323	3,13	1582,01	1
1	269	784	2,91	1969,83	1
1	131	449	3,43	669,42	1
1	217	716	3,30	538,82	1
1	212	708	3,34	999,78	1
1	261	898	3,44	544,34	1
1	272	902	3,32	1181,93	1
1	283	944	3,34	1420,37	1
1	342	1113	3,25	1380,37	1
1	211	808	3,83	431,75	1
1	207	706	3,41	989,77	1
1	250	816	3,26	1172,12	1
1	211	698	3,31	1106,75	1
1	236	759	3,22	1231,38	1
1	352	1220	3,47	1108,54	1
1	195	636	3,26	1091,68	1
1	283	934	3,30	1017,40	1
1	206	651	3,16	845,03	1
1	318	1065	3,35	890,22	1
1	250	818	3,27	614,25	1
1	293	960	3,28	864,09	1
1	404	1315	3,25	1081,15	1
1	232	757	3,26	755,20	1
1	106	324	3,06	1025,79	1
1	89	338	3,80	433,61	1
1	196	661	3,37	981,67	1
1	168	555	3,30	971,01	1
1	363	1259	3,47	725,90	1
1	336	1125	3,35	554,42	1
1	244	786	3,22	964,48	1
1	233	825	3,54	904,88	1
1	251	824	3,28	845,16	1
1	257	856	3,33	964,93	1
1	274	984	3,59	695,27	1
1	298	1053	3,53	887,60	1
1	247	828	3,35	849,24	1
1	267	946	3,54	754,10	1
1	276	949	3,44	800,98	1
1	177	586	3,31	574,47	1

1	168	559	3,33	617,15	1
1	335	1164	3,47	485,04	1
1	363	1240	3,42	537,39	1
1	178	632	3,55	1004,84	1
1	285	1229	4,31	762,18	1
1	150	504	3,36	756,67	1
1	260	857	3,30	886,24	1
1	234	784	3,35	780,25	1
1	136	446	3,28	862,45	1
1	287	945	3,29	675,18	1
1	247	851	3,45	801,77	1
1	423	1509	3,57	781,41	1
1	196	718	3,66	849,29	1
1	199	708	3,56	991,45	1
1	154	542	3,52	475,64	1
1	170	561	3,30	648,92	1
1	354	1179	3,33	877,16	1
1	305	965	3,16	900,84	1
1	337	1068	3,17	1283,51	1
1	131	415	3,17	1029,87	1
1	211	676	3,20	681,10	1
1	212	683	3,22	1020,50	1
1	331	1016	3,07	1352,52	1
1	381	1190	3,12	883,18	1
1	330	1108	3,36	819,65	1
1	182	629	3,46	528,27	1
1	201	613	3,05	1111,15	1
1	6	20	3,33	1668,00	1
1	200	627	3,14	755,20	1
1	270	1006	3,73	1994,46	1
1	314	935	2,98	861,23	1
1	137	457	3,34	939,66	1
1	160	493	3,08	1302,34	1
1	277	881	3,18	1325,63	1
1	190	665	3,50	1141,37	1
1	247	827	3,35	1196,56	1
1	36	127	3,53	1236,39	1
1	251	777	3,10	806,06	1
1	355	1008	2,84	2363,61	1
1	245	793	3,24	2359,43	1
1	195	614	3,15	1738,63	1
0	0	0	0,00	0,00	0
1	283	776	2,74	1260,20	1
1	262	775	2,96	1924,65	1
1	227	627	2,76	1897,52	1
1	174	547	3,14	1225,80	1
1	178	606	3,40	1283,78	1
1	212	576	2,72	2002,07	1
1	299	972	3,25	2267,64	1
1	236	797	3,38	1396,77	1

1	238	788	3,31	2234,43	1
1	160	508	3,18	1067,53	1
1	86	264	3,07	1218,79	1
1	197	520	2,64	1595,63	1
1	88	290	3,30	1299,93	1
1	65	215	3,31	995,78	1
1	189	679	3,59	1581,18	1
1	391	1348	3,45	650,15	1
1	101	343	3,40	720,32	1
1	124	384	3,10	1072,39	1
1	8	27	3,38	995,63	1
1	46	163	3,54	898,20	1
1	281	891	3,17	1469,90	1
1	73	274	3,75	564,96	1
1	58	182	3,14	614,76	1
1	279	897	3,22	987,12	1
1	222	687	3,09	1200,14	1
1	98	338	3,45	979,71	1
1	32	108	3,38	942,66	1
1	58	211	3,64	1204,66	1
1	136	455	3,35	694,21	1
1	28	97	3,46	657,04	1
1	62	189	3,05	1517,42	1
1	23	85	3,70	609,04	1
1	188	636	3,38	704,08	1
1	267	866	3,24	1683,62	1
1	208	707	3,40	821,39	1
1	204	642	3,15	1168,83	1
1	139	487	3,50	919,19	1
1	244	814	3,34	575,51	1
1	145	491	3,39	878,94	1
1	187	613	3,28	549,03	1
1	54	173	3,20	785,06	1
1	17	56	3,29	886,47	1
1	37	121	3,27	1005,59	1
1	153	491	3,21	1443,95	1
1	107	360	3,36	737,42	1
1	271	877	3,24	1338,81	1
1	137	505	3,69	595,59	1
1	89	343	3,85	604,90	1
1	66	240	3,64	627,77	1
1	81	250	3,09	1036,05	1
1	92	335	3,64	590,01	1
1	116	408	3,52	581,16	1
1	99	329	3,32	953,83	1
1	33	124	3,76	800,00	1
1	25	87	3,48	894,80	1
1	175	587	3,35	757,30	1
1	65	225	3,46	616,29	1
1	50	142	2,84	1078,88	1

1	135	422	3,13	644,07	1
1	86	328	3,81	690,57	1
1	96	366	3,81	592,65	1
1	93	343	3,69	628,09	1
1	22	75	3,41	796,32	1
1	183	610	3,33	983,36	1
1	43	148	3,44	819,02	1
1	114	408	3,58	662,35	1
1	68	236	3,47	722,66	1
1	175	625	3,57	846,61	1
1	52	156	3,00	589,92	1
1	43	159	3,70	892,35	1
1	65	202	3,11	1172,00	1
1	43	178	4,14	1657,21	1
1	157	507	3,23	692,44	1
1	132	463	3,51	577,13	1
1	77	268	3,48	558,03	1
1	89	300	3,37	557,51	1
1	72	271	3,76	520,97	1
1	350	944	2,70	1065,01	1
1	233	682	2,93	998,74	1
1	37	114	3,08	1556,49	1
1	28	95	3,39	1197,11	1
1	257	704	2,74	1125,24	1
1	359	914	2,55	1388,23	1
1	190	640	3,37	789,34	1
1	261	781	2,99	981,98	1
1	306	937	3,06	1190,17	1
1	235	783	3,33	881,09	1
1	183	609	3,33	932,47	1
1	225	748	3,32	831,82	1
1	150	506	3,37	1429,37	1
1	152	488	3,21	1307,42	1
1	136	456	3,35	956,76	1
1	125	416	3,33	1406,59	1
1	239	763	3,19	3100,19	1
1	353	925	2,62	1643,82	1
1	190	598	3,15	985,88	1
1	159	528	3,32	648,90	1
1	234	774	3,31	915,49	1
1	210	692	3,30	945,18	1
1	176	558	3,17	962,85	1
1	279	926	3,32	979,53	1
1	286	945	3,30	777,28	1
1	167	546	3,27	835,19	1
1	254	743	2,93	964,81	1
1	225	732	3,25	1641,27	1
1	216	710	3,29	858,73	1
1	75	324	4,32	608,43	1
1	167	500	2,99	2343,49	1

1	308	998	3,24	1758,93	1
1	169	539	3,19	777,35	1
1	226	804	3,56	728,57	1
1	231	740	3,20	1013,33	1
1	243	837	3,44	972,86	1
1	88	350	3,98	546,23	1
1	46	155	3,37	977,67	1
1	31	119	3,84	668,61	1
1	28	99	3,54	552,57	1
1	18	62	3,44	1137,33	1
1	27	100	3,70	1098,15	1
1	18	66	3,67	826,11	1
1	17	56	3,29	1042,35	1
1	23	67	2,91	1087,26	1
1	25	74	2,96	610,80	1
1	45	155	3,44	945,49	1
1	50	154	3,08	877,74	1
1	22	70	3,18	1384,55	1
1	8	31	3,88	658,75	1
1	86	229	2,66	1500,21	1
1	33	125	3,79	592,12	1
1	43	135	3,14	885,79	1
1	29	88	3,03	910,14	1
1	11	37	3,36	926,82	1
1	24	84	3,50	818,17	1
1	87	282	3,24	856,44	1
1	72	221	3,07	1334,86	1
1	20	62	3,10	666,50	1
1	11	43	3,91	858,73	1
1	29	85	2,93	1076,90	1
1	105	358	3,41	638,83	1
1	33	128	3,88	894,58	1
1	32	107	3,34	958,13	1
1	9	36	4,00	1052,22	1
1	78	269	3,45	901,77	1
1	30	84	2,80	622,00	1
1	29	90	3,10	597,76	1

DOMCO	DOMPAECO	DOMPER	DOMTCAS	DOMVILCON	DOMTAP
0,00	296	100,00	0,34	0,00	99,66
0,00	180	100,00	0,00	0,00	100,00
6,35	268	100,00	5,22	0,00	88,81
0,00	133	100,00	29,32	0,00	70,68
0,35	290	100,00	31,03	0,34	68,28
0,00	317	100,00	100,00	0,00	0,00
0,00	156	100,00	98,72	0,64	0,64
0,00	150	100,00	2,00	0,67	97,33
0,00	199	100,00	94,47	3,52	2,01
0,00	198	100,00	16,67	0,00	83,33
0,00	242	100,00	19,42	0,00	80,58
0,00	265	100,00	19,62	0,00	80,38
5,32	198	100,00	16,67	0,00	78,28
0,00	276	100,00	22,10	0,00	77,90
0,00	237	100,00	22,36	0,00	77,64
8,13	306	100,00	4,25	0,33	87,91
1,99	154	100,00	3,25	0,00	94,81
0,00	188	100,00	93,09	0,53	6,38
0,00	133	100,00	91,73	0,00	8,27
0,00	191	100,00	3,66	0,00	96,34
9,86	323	100,00	9,91	52,63	28,48
0,00	203	100,00	0,00	0,00	100,00
0,00	152	100,00	0,00	0,00	100,00
0,47	212	100,00	3,30	0,00	96,23
0,00	176	100,00	0,57	0,00	99,43
0,00	207	100,00	21,26	0,00	78,74
0,00	337	100,00	2,08	0,00	97,92
0,00	319	100,00	79,00	0,63	20,38
0,36	276	100,00	79,71	0,36	15,94
0,00	319	100,00	89,97	0,00	10,03
0,00	4	0,00	0,00	0,00	0,00
0,31	321	100,00	92,52	0,31	6,85
0,00	219	100,00	94,52	0,46	5,02
0,51	197	100,00	74,11	0,51	22,84
0,41	243	100,00	97,53	0,00	2,06
0,00	73	100,00	89,04	0,00	10,96
3,59	173	100,00	28,90	0,00	67,63
0,00	300	100,00	81,00	0,00	19,00
0,00	281	100,00	85,05	0,00	14,95
0,00	187	100,00	84,49	0,00	11,76
0,00	344	100,00	96,51	0,00	1,74
0,32	310	100,00	96,45	0,00	3,23
0,00	229	100,00	96,51	0,00	2,62
0,00	257	100,00	96,11	0,00	3,89
0,00	289	100,00	98,27	0,00	1,73
0,00	221	100,00	69,68	0,00	30,32
0,00	186	100,00	71,51	0,00	28,49
0,00	302	100,00	6,29	0,00	93,71
0,00	239	100,00	3,35	0,00	96,65

0,00	208	100,00	0,48	0,96	98,56
0,00	252	100,00	4,37	0,00	95,63
0,00	319	100,00	2,51	0,00	97,49
0,00	97	100,00	3,09	0,00	96,91
0,00	220	100,00	21,82	0,00	78,18
0,00	137	100,00	57,66	0,00	40,88
0,00	176	100,00	42,61	7,39	50,00
0,00	268	100,00	82,84	5,22	11,94
0,00	289	100,00	62,98	0,00	37,02
0,00	188	100,00	43,09	0,00	56,91
0,00	193	100,00	38,86	1,55	59,59
0,00	236	100,00	5,08	2,54	91,10
0,00	215	100,00	56,28	3,72	36,28
0,00	331	100,00	49,85	0,00	49,55
0,00	315	100,00	78,41	0,32	21,27
0,00	173	100,00	17,34	0,00	82,08
0,00	153	100,00	67,32	1,31	31,37
0,00	107	100,00	92,52	0,00	3,74
0,00	213	100,00	38,03	0,00	61,50
0,00	357	100,00	33,05	0,28	61,90
0,00	212	100,00	4,72	0,00	95,28
0,00	189	100,00	1,06	0,00	98,94
0,00	295	100,00	1,02	1,02	97,97
0,00	231	100,00	32,47	0,00	65,37
2,38	309	100,00	6,80	0,00	88,67
0,30	323	100,00	7,12	0,00	86,07
0,00	363	100,00	3,86	0,00	96,14
0,00	212	100,00	4,25	0,47	95,28
0,00	277	100,00	10,83	0,00	89,17
0,00	165	100,00	0,61	3,03	96,36
0,47	301	100,00	7,64	0,00	90,37
5,00	194	100,00	3,09	0,00	96,91
0,00	190	100,00	0,53	0,00	99,47
0,00	342	100,00	1,46	2,05	96,20
0,00	80	100,00	3,75	0,00	96,25
2,21	340	100,00	1,76	0,00	98,24
4,75	260	100,00	1,15	2,69	95,77
7,31	222	100,00	3,60	0,00	96,40
0,00	251	100,00	3,19	1,20	95,62
0,00	332	100,00	28,92	0,30	50,90
0,00	271	100,00	15,13	0,00	84,50
0,00	347	100,00	35,16	0,00	59,65
0,67	164	100,00	17,68	3,66	78,66
0,00	156	100,00	17,31	0,00	81,41
0,00	247	100,00	17,00	0,81	82,19
0,00	279	100,00	16,85	0,00	83,15
0,00	286	100,00	3,85	0,00	96,15
0,00	237	100,00	14,77	0,42	84,81
0,39	259	100,00	15,06	0,77	84,17
0,00	252	100,00	6,75	0,00	93,25

0,00	144	100,00	2,08	0,69	97,22
24,81	266	100,00	3,76	0,00	95,86
0,37	294	100,00	1,36	0,00	98,64
5,47	237	100,00	0,84	1,27	97,89
0,00	298	100,00	7,38	0,00	92,62
0,00	253	100,00	5,53	0,00	94,47
0,00	307	100,00	4,89	0,00	95,11
0,00	313	100,00	12,46	0,00	87,54
0,00	222	100,00	7,21	0,00	92,79
0,00	152	100,00	5,92	0,00	94,08
0,00	207	100,00	6,76	0,00	93,24
0,00	177	100,00	2,82	0,56	96,61
0,00	223	100,00	3,14	0,00	96,86
0,00	155	100,00	1,29	9,68	89,03
0,00	271	100,00	1,85	0,00	89,30
0,00	364	100,00	2,20	0,55	96,98
0,00	165	100,00	2,42	1,21	96,36
0,00	109	100,00	9,17	0,00	89,91
0,00	166	100,00	7,23	0,00	92,77
0,00	180	100,00	4,44	0,00	95,56
100,00	37	0,00	0,00	0,00	0,00
0,00	98	100,00	4,08	0,00	94,90
0,00	185	100,00	5,95	0,00	94,05
0,00	166	100,00	21,69	12,05	62,05
100,00	38	0,00	0,00	0,00	0,00
0,00	279	100,00	3,23	0,36	95,70
9,72	219	100,00	26,94	0,00	72,60
0,28	339	100,00	16,22	2,65	79,06
0,00	212	100,00	9,43	0,47	90,09
0,93	182	100,00	9,34	0,00	90,66
0,00	264	100,00	17,42	1,89	80,30
0,00	199	100,00	5,03	0,50	94,47
0,00	181	100,00	1,10	0,55	98,34
1,03	398	100,00	3,52	0,00	96,48
0,00	200	100,00	12,00	0,50	82,50
4,40	108	100,00	23,15	0,93	75,93
2,07	148	100,00	4,05	7,43	86,49
0,72	279	100,00	9,32	1,08	89,25
0,46	258	100,00	6,20	0,78	92,25
2,11	227	100,00	1,32	0,88	97,80
0,00	104	100,00	3,85	0,96	95,19
0,00	224	100,00	1,34	0,00	98,66
0,38	217	100,00	1,84	0,00	98,16
0,00	189	100,00	6,35	0,00	93,65
0,00	220	100,00	0,91	1,36	97,73
0,00	180	100,00	1,11	0,00	98,89
5,26	409	100,00	1,22	0,00	72,37
0,00	350	100,00	0,86	0,29	95,14
2,07	115	100,00	97,39	0,00	2,61
0,36	209	100,00	99,52	0,00	0,48

0,39	179	100,00	98,88	0,00	1,12
0,00	236	100,00	100,00	0,00	0,00
0,00	28	100,00	7,14	0,00	92,86
0,00	234	100,00	98,29	0,00	1,71
0,00	154	100,00	97,40	0,00	0,65
0,00	189	100,00	91,01	6,88	1,59
0,00	180	100,00	1,11	0,56	98,33
0,00	48	100,00	6,25	91,67	2,08
35,88	153	100,00	98,04	0,00	1,96
3,86	240	100,00	96,67	0,00	3,33
0,00	138	100,00	12,32	0,72	86,23
0,00	213	100,00	74,18	0,00	25,82
0,00	242	100,00	98,76	0,00	0,83
0,00	182	100,00	52,20	0,00	47,80
0,00	170	100,00	78,82	0,00	21,18
0,00	250	100,00	100,00	0,00	0,00
1,99	137	100,00	94,16	0,00	0,73
0,00	81	100,00	100,00	0,00	0,00
0,00	119	100,00	99,16	0,84	0,00
0,00	103	100,00	92,23	0,00	7,77
0,00	174	100,00	97,13	0,00	2,30
0,00	269	100,00	98,88	0,00	1,12
0,00	93	100,00	97,85	0,00	2,15
0,00	76	100,00	97,37	0,00	2,63
0,00	142	100,00	98,59	0,00	1,41
0,00	184	100,00	96,20	0,00	3,80
0,00	106	100,00	1,89	96,23	1,89
0,00	152	100,00	97,37	0,00	2,63
0,00	303	100,00	96,04	0,00	3,96
0,00	264	100,00	95,83	0,38	3,79
0,00	165	100,00	86,67	0,00	13,33
0,00	192	100,00	100,00	0,00	0,00
0,58	81	100,00	97,53	0,00	1,23
0,00	113	100,00	47,79	0,00	52,21
0,00	167	100,00	100,00	0,00	0,00
0,00	113	100,00	99,12	0,00	0,00
0,00	224	100,00	95,54	0,00	4,46
0,00	98	100,00	98,98	0,00	1,02
0,00	280	100,00	3,93	95,71	0,00
0,00	195	100,00	95,90	0,00	1,54
0,00	116	100,00	99,14	0,86	0,00
0,00	272	100,00	100,00	0,00	0,00
0,00	284	100,00	98,59	0,00	1,06
0,00	311	100,00	3,54	96,14	0,32
1,25	94	100,00	100,00	0,00	0,00
0,00	230	100,00	94,78	0,00	5,22
0,00	154	100,00	6,49	1,30	92,21
0,89	235	100,00	5,53	0,00	91,91
0,00	310	100,00	1,61	0,00	98,39
0,00	239	100,00	20,50	0,00	79,50

0,00	253	100,00	16,21	0,40	83,40
2,63	246	100,00	5,69	0,41	93,90
0,00	268	100,00	3,73	0,00	96,27
0,00	113	100,00	20,35	0,00	79,65
0,00	314	100,00	13,38	3,82	82,48
0,00	281	100,00	11,39	0,36	88,26
0,00	418	100,00	8,37	0,00	91,63
0,00	92	100,00	5,43	0,00	94,57
0,00	166	100,00	23,49	0,00	76,51
0,00	193	100,00	3,63	0,00	96,37
0,00	163	100,00	9,20	0,00	86,50
0,00	276	100,00	25,36	0,00	74,64
0,00	227	100,00	9,69	0,00	90,31
0,00	154	100,00	5,19	0,65	94,16
0,00	184	100,00	6,52	0,00	93,48
0,00	180	100,00	4,44	0,00	95,56
0,00	320	100,00	2,50	0,00	97,50
0,00	20	100,00	0,00	0,00	100,00
0,00	246	100,00	7,72	0,00	91,87
0,00	64	100,00	7,81	0,00	92,19
3,96	210	100,00	7,14	0,00	89,05
0,00	160	100,00	0,00	0,63	99,38
0,00	190	100,00	3,16	0,53	96,32
0,00	19	100,00	10,53	10,53	78,95
0,00	146	100,00	8,22	0,00	91,78
0,00	124	100,00	5,65	0,00	92,74
0,00	240	100,00	1,67	0,00	98,33
0,00	263	100,00	1,90	0,00	98,10
0,00	187	100,00	6,42	0,53	92,51
0,00	249	100,00	0,80	0,40	98,80
0,00	272	100,00	4,41	0,00	95,59
0,00	269	100,00	4,83	0,00	95,17
0,00	233	100,00	3,43	0,43	96,14
2,65	194	100,00	1,03	0,00	95,88
0,00	239	100,00	1,26	0,00	98,74
0,00	215	100,00	0,00	0,00	100,00
13,64	50	100,00	4,00	0,00	80,00
0,00	241	100,00	3,32	0,00	96,68
8,62	252	100,00	23,02	1,59	66,27
0,00	182	100,00	1,65	0,55	97,80
4,72	222	100,00	24,77	0,45	70,27
0,00	195	100,00	0,51	0,00	99,49
0,00	252	100,00	0,40	0,00	99,60
0,00	179	100,00	0,00	0,00	100,00
0,00	241	100,00	3,73	67,22	29,05
0,00	283	100,00	4,59	0,71	94,70
0,00	178	100,00	5,06	1,12	93,82
0,00	216	100,00	5,09	0,00	94,91
1,75	290	100,00	9,31	0,00	88,97
0,00	271	100,00	7,75	0,00	91,88

0,00	235	100,00	12,77	0,00	87,23
0,00	257	100,00	3,89	0,00	96,11
0,00	245	100,00	1,63	0,00	98,37
0,00	229	100,00	2,62	0,87	96,51
0,00	326	100,00	1,23	0,00	98,77
0,00	306	100,00	1,31	1,63	97,06
0,00	190	100,00	0,53	0,00	99,47
3,52	206	100,00	0,00	0,00	96,60
0,00	225	100,00	0,00	0,00	100,00
0,00	345	100,00	0,00	0,00	100,00
3,01	137	100,00	6,57	7,30	83,21
0,00	242	100,00	0,00	0,00	100,00
0,00	265	100,00	0,38	0,75	98,87
0,00	153	100,00	0,00	0,65	99,35
0,00	174	100,00	0,00	0,00	100,00
0,00	231	100,00	0,00	0,00	100,00
0,00	126	100,00	0,00	0,00	100,00
0,47	216	100,00	0,93	0,00	98,61
0,00	185	100,00	0,00	0,54	98,92
0,00	203	100,00	0,00	0,00	100,00
0,00	371	100,00	0,00	1,08	98,92
0,00	252	100,00	1,98	0,79	97,22
0,00	256	100,00	8,59	1,95	89,45
0,00	279	100,00	3,94	0,72	95,34
2,58	278	100,00	3,96	0,36	93,17
6,49	279	100,00	4,30	0,00	89,61
0,00	284	100,00	2,46	0,00	97,54
0,00	270	100,00	2,96	0,00	97,04
3,70	224	100,00	3,13	0,45	92,86
0,43	234	100,00	2,56	0,00	97,01
0,00	287	100,00	2,44	0,00	97,56
0,00	221	100,00	3,62	0,90	95,02
0,00	202	100,00	15,35	0,00	83,66
0,00	246	100,00	9,35	0,41	90,24
0,00	313	100,00	17,57	0,96	81,47
6,42	315	100,00	9,52	0,32	84,13
0,00	364	100,00	7,97	0,00	92,03
0,00	243	100,00	7,00	0,41	92,59
0,00	184	100,00	1,63	1,09	97,28
0,00	283	100,00	1,41	0,35	98,23
0,00	226	100,00	2,21	0,44	97,35
0,00	182	100,00	0,00	0,00	100,00
0,00	215	100,00	0,47	0,47	98,60
0,00	278	100,00	1,08	0,00	98,92
3,90	213	100,00	3,29	0,94	92,02
0,00	247	100,00	0,81	0,40	98,79
0,00	128	100,00	0,78	0,00	99,22
0,00	215	100,00	1,40	0,00	98,60
16,88	180	100,00	0,00	0,00	85,00
0,00	251	100,00	0,40	0,00	99,60

0,00	280	100,00	0,00	0,71	99,29
0,00	234	100,00	0,85	0,43	98,72
0,00	191	100,00	0,00	0,00	100,00
0,00	247	100,00	97,57	0,00	2,43
0,00	193	100,00	1,55	0,00	98,45
0,00	272	100,00	2,94	0,00	96,69
0,00	242	100,00	0,00	0,00	100,00
0,00	385	100,00	2,08	0,26	97,66
0,00	203	100,00	7,88	0,00	92,12
0,00	228	100,00	0,44	0,44	98,68
0,00	0	0,00	0,00	0,00	0,00
0,52	389	100,00	2,57	1,03	95,89
0,00	288	100,00	1,04	0,35	98,61
0,00	282	100,00	1,77	0,00	98,23
0,00	331	100,00	9,97	0,00	90,03
0,00	104	100,00	23,08	0,00	76,92
0,00	202	100,00	8,42	0,00	91,58
0,00	311	100,00	12,54	1,29	86,17
0,00	231	100,00	21,65	0,43	77,92
0,00	234	100,00	14,53	2,14	83,33
0,00	279	100,00	3,94	0,00	96,06
0,00	240	100,00	1,67	0,42	97,92
0,00	349	100,00	0,86	1,15	97,99
0,00	242	100,00	1,24	0,00	98,76
0,00	369	100,00	1,63	0,00	98,37
0,00	147	100,00	2,04	0,00	97,96
0,00	110	100,00	4,55	0,00	95,45
0,00	152	100,00	1,32	0,00	98,68
0,00	223	100,00	0,00	0,90	99,10
0,00	228	100,00	0,00	0,00	100,00
0,00	169	100,00	0,00	0,59	99,41
0,00	174	100,00	0,00	0,57	98,85
7,14	150	100,00	2,67	0,00	90,67
0,00	229	100,00	0,00	0,44	99,56
0,00	276	100,00	0,00	0,36	99,64
0,00	224	100,00	0,45	0,45	99,11
0,00	243	100,00	2,88	1,23	95,88
0,00	251	100,00	2,79	0,00	97,21
0,00	254	100,00	98,43	0,00	1,57
0,74	137	100,00	96,35	0,00	2,92
1,08	281	100,00	96,80	0,00	2,14
0,00	86	100,00	95,35	0,00	4,65
0,54	187	100,00	88,24	0,00	10,70
0,00	150	100,00	98,00	0,00	2,00
0,00	238	100,00	97,06	0,00	2,94
0,00	188	100,00	100,00	0,00	0,00
0,00	97	100,00	94,85	0,00	2,06
0,00	330	100,00	83,33	0,30	16,06
0,00	240	100,00	90,83	0,00	9,17
0,24	419	100,00	94,75	0,00	1,67

0,00	266	100,00	96,62	0,75	2,63
0,00	303	100,00	90,76	0,00	9,24
0,47	214	100,00	46,73	44,86	7,94
0,00	237	100,00	27,43	52,74	19,83
0,00	282	100,00	95,04	0,35	4,61
0,00	53	100,00	3,77	96,23	0,00
0,00	311	100,00	98,07	0,00	1,93
0,00	118	100,00	5,08	0,00	94,92
0,00	204	100,00	3,43	0,00	96,57
0,00	310	100,00	0,65	0,00	99,35
0,00	155	100,00	0,00	0,00	100,00
0,00	325	100,00	0,31	0,00	99,69
0,00	230	100,00	3,48	0,00	96,52
0,00	316	100,00	0,95	0,00	99,05
0,00	336	100,00	12,50	0,60	86,90
0,00	205	100,00	1,95	1,46	96,59
0,00	324	100,00	10,19	0,62	89,20
0,00	296	100,00	0,34	0,34	99,32
0,45	224	100,00	12,95	1,34	85,27
0,00	199	100,00	12,06	0,00	87,94
0,00	193	100,00	0,00	0,00	100,00
0,00	208	100,00	0,00	0,48	99,04
0,00	117	100,00	0,85	0,00	99,15
1,23	246	100,00	3,66	0,00	95,12
0,48	211	100,00	10,43	0,95	88,15
0,00	207	100,00	2,90	0,00	96,14
0,00	146	100,00	1,37	0,00	98,63
0,00	160	100,00	7,50	0,00	86,88
0,74	136	100,00	16,18	0,00	78,68
0,00	290	100,00	1,03	0,34	98,62
2,14	191	100,00	2,62	0,00	94,76
0,00	1	0,00	0,00	0,00	0,00
1,40	290	100,00	57,24	0,00	41,38
0,00	247	100,00	32,39	0,00	67,61
0,60	169	100,00	20,12	0,00	79,29
0,00	286	100,00	35,66	0,00	64,34
0,00	92	100,00	97,83	0,00	2,17
0,00	290	100,00	30,69	0,69	68,62
0,00	258	100,00	0,00	0,00	100,00
0,00	119	100,00	14,29	0,00	85,71
0,00	299	100,00	1,00	0,00	99,00
0,00	217	100,00	6,45	0,92	92,63
1,45	210	100,00	43,81	0,48	54,29
0,00	188	100,00	18,62	1,06	80,32
0,00	92	100,00	0,00	0,00	100,00
0,00	189	100,00	1,59	0,00	98,41
0,00	212	100,00	4,72	0,47	94,81
0,00	205	100,00	3,90	4,39	91,71
0,00	217	100,00	7,83	0,00	92,17
34,71	229	100,00	44,98	0,44	28,82

0,00	191	100,00	1,05	0,00	98,95
0,00	193	100,00	0,52	0,00	99,48
0,00	269	100,00	10,41	0,00	89,59
0,00	121	100,00	15,70	0,00	84,30
0,00	29	0,00	0,00	0,00	0,00
0,00	239	100,00	14,64	0,00	84,94
0,00	0	0,00	0,00	0,00	0,00
0,00	293	100,00	96,93	0,34	2,73
0,00	172	100,00	22,09	0,00	77,91
0,00	356	100,00	0,00	0,28	99,72
0,00	341	100,00	0,29	0,00	99,71
0,00	329	100,00	0,00	0,00	100,00
0,00	242	100,00	16,12	0,00	83,88
0,00	197	100,00	26,90	0,00	73,10
0,00	151	100,00	88,08	0,00	11,92
0,00	304	100,00	94,41	0,00	5,59
0,00	1	100,00	0,00	0,00	0,00
0,00	278	100,00	0,36	0,00	99,28
0,00	257	100,00	1,17	0,78	98,05
0,00	396	100,00	1,01	0,00	98,99
0,00	357	100,00	16,81	0,00	83,19
0,00	230	100,00	4,35	0,00	95,65
0,00	122	100,00	4,10	0,00	95,90
0,00	132	100,00	0,00	0,00	100,00
0,00	211	100,00	6,16	0,47	93,36
0,00	303	100,00	1,32	0,33	98,35
0,00	237	100,00	15,61	0,42	83,97
0,00	323	100,00	99,07	0,00	0,93
0,00	109	100,00	3,67	0,00	96,33
0,00	152	100,00	1,97	0,00	98,03
0,00	147	100,00	1,36	2,04	96,60
0,00	230	100,00	0,87	0,00	99,13
0,00	247	100,00	3,24	0,00	96,76
0,00	147	100,00	19,05	0,00	80,95
0,00	182	100,00	1,10	0,00	98,90
0,00	182	100,00	7,69	0,00	92,31
0,00	197	100,00	8,12	0,00	91,88
0,00	163	100,00	30,67	0,00	69,33
4,09	178	100,00	6,74	0,56	88,76
0,00	177	100,00	31,07	0,00	68,36
0,44	227	100,00	8,37	0,00	91,19
0,49	207	100,00	2,42	0,00	97,10
0,00	137	100,00	32,12	0,00	67,88
0,00	164	100,00	9,76	0,00	90,24
0,00	175	100,00	3,43	0,00	96,57
0,00	126	100,00	6,35	0,00	93,65
0,00	124	100,00	0,00	0,00	100,00
0,00	169	100,00	0,00	0,59	99,41
0,00	191	100,00	6,28	0,00	93,72
0,00	224	100,00	0,45	0,00	99,55

0,00	202	100,00	2,48	0,00	97,52
8,09	147	100,00	12,24	0,00	80,27
0,00	261	100,00	4,60	0,00	95,40
0,00	314	100,00	0,64	0,00	99,04
0,00	211	100,00	0,47	0,95	98,58
0,00	177	100,00	0,56	1,13	98,31
0,00	198	100,00	1,01	0,00	98,99
0,00	68	100,00	0,00	0,00	100,00
0,00	139	100,00	13,67	0,00	86,33
11,65	115	100,00	20,00	0,00	69,57
0,00	221	100,00	3,17	0,00	96,83
0,00	294	100,00	0,34	0,34	99,32
0,00	83	100,00	0,00	0,00	100,00
0,00	241	100,00	4,56	0,41	95,02
0,00	242	100,00	0,83	0,00	99,17
0,00	150	100,00	0,00	0,00	100,00
0,00	312	100,00	0,00	0,00	100,00
0,00	124	100,00	0,81	0,00	99,19
0,00	141	100,00	2,13	0,00	97,87
0,00	133	100,00	2,26	0,00	97,74
0,00	40	100,00	10,00	0,00	90,00
0,00	333	100,00	3,00	0,00	97,00
0,00	122	100,00	3,28	0,00	96,72
0,00	285	100,00	0,00	3,51	96,49
0,00	64	100,00	17,19	0,00	82,81
0,00	176	100,00	12,50	1,70	85,80
0,00	61	100,00	0,00	1,64	98,36
0,00	128	100,00	59,38	0,00	40,63
0,00	227	100,00	3,96	0,44	95,59
0,00	216	100,00	1,39	0,46	98,15
0,00	11	100,00	0,00	0,00	100,00
0,00	31	100,00	3,23	0,00	96,77
0,00	61	100,00	0,00	3,28	96,72
0,00	38	100,00	0,00	0,00	100,00
0,00	159	100,00	0,00	0,00	100,00
0,00	152	100,00	1,32	0,00	98,68
0,00	0	0,00	0,00	0,00	0,00
0,00	71	100,00	8,45	0,00	91,55
0,00	104	100,00	5,77	0,00	94,23
0,00	51	100,00	9,80	0,00	90,20
0,00	105	100,00	0,95	0,00	99,05
0,00	207	100,00	8,70	0,00	91,30
0,00	68	100,00	5,88	0,00	94,12
0,00	46	100,00	97,83	0,00	2,17
0,00	43	100,00	2,33	0,00	97,67
0,00	143	100,00	7,69	0,00	92,31
0,00	46	100,00	19,57	0,00	80,43
0,00	81	100,00	93,83	0,00	6,17
0,00	0	0,00	0,00	0,00	0,00
0,00	44	100,00	2,27	0,00	97,73

0,00	131	100,00	90,84	0,00	9,16
0,00	66	100,00	0,00	0,00	100,00
0,00	29	100,00	96,55	0,00	3,45
0,00	0	0,00	0,00	0,00	0,00
0,00	89	100,00	100,00	0,00	0,00
0,00	150	100,00	2,67	0,67	96,67
0,00	36	100,00	100,00	0,00	0,00
0,00	54	100,00	98,15	0,00	1,85
0,00	195	100,00	36,92	16,41	46,67
0,00	73	100,00	98,63	0,00	1,37
0,00	116	100,00	97,41	0,00	1,72
0,00	212	100,00	81,13	10,38	8,49
0,00	0	0,00	0,00	0,00	0,00
0,60	167	100,00	98,20	0,00	0,00
0,00	185	100,00	83,78	1,62	14,59
1,25	81	100,00	91,36	0,00	7,41
0,00	87	100,00	89,66	0,00	10,34
0,00	130	100,00	0,77	0,00	99,23
0,00	61	100,00	98,36	0,00	0,00
0,00	102	100,00	94,12	0,00	0,98
0,00	114	100,00	97,37	0,00	2,63
0,00	352	100,00	44,89	0,00	52,84
0,00	104	100,00	0,96	0,00	99,04
0,00	228	100,00	0,44	0,44	99,12
0,00	14	100,00	0,00	0,00	0,00
0,00	157	100,00	0,00	0,00	100,00
0,00	59	100,00	86,44	0,00	13,56
0,00	39	100,00	5,13	0,00	94,87
0,00	142	100,00	9,15	1,41	89,44
0,00	37	100,00	8,11	86,49	5,41
0,00	22	100,00	90,91	4,55	4,55
0,00	46	100,00	91,30	0,00	8,70
0,00	18	100,00	88,89	0,00	11,11
0,00	8	100,00	0,00	0,00	0,00
0,00	195	100,00	5,13	0,00	94,87
0,00	284	100,00	2,46	0,00	97,54
0,00	141	100,00	5,67	0,00	94,33
0,00	147	100,00	0,00	0,00	100,00
0,00	189	100,00	0,53	0,00	99,47
0,00	67	100,00	2,99	0,00	97,01
0,00	184	100,00	3,26	0,00	96,74
0,00	257	100,00	78,21	0,00	21,79
0,00	287	100,00	79,79	0,35	19,86
0,00	192	100,00	94,79	0,00	5,21
0,00	306	100,00	64,38	0,33	35,29
10,26	172	100,00	87,21	0,00	3,49
0,39	256	100,00	85,16	0,00	14,45
0,00	200	100,00	67,00	0,00	33,00
0,00	183	100,00	98,91	0,00	1,09
0,00	134	100,00	97,01	0,00	2,99

0,00	238	100,00	77,73	0,00	22,27
0,00	30	100,00	56,67	0,00	16,67
0,00	90	0,00	0,00	0,00	0,00
0,00	196	100,00	100,00	0,00	0,00
2,76	261	100,00	95,40	0,00	1,92
0,00	195	100,00	97,44	0,00	2,56
0,00	311	100,00	98,07	0,00	1,93
1,97	310	100,00	95,81	0,97	1,29
0,00	272	100,00	85,66	0,37	0,74
11,60	279	100,00	81,72	2,87	5,02
0,52	192	100,00	97,40	1,04	1,04
0,38	266	100,00	92,86	0,00	4,51
0,00	196	100,00	90,82	1,53	2,55
0,00	200	100,00	96,00	0,00	4,00
0,00	236	100,00	87,29	0,00	12,71
0,00	213	100,00	72,30	0,00	27,70
0,40	251	100,00	48,61	0,80	50,20
0,00	249	100,00	59,04	0,00	40,96
0,00	179	100,00	46,93	0,00	52,51
0,00	318	100,00	58,81	2,20	33,33
0,00	206	100,00	43,20	0,00	56,80
0,00	183	100,00	39,34	0,00	45,90
0,00	261	100,00	68,20	0,00	31,80
0,00	350	100,00	41,14	0,00	58,86
0,00	269	100,00	76,95	0,74	19,70
0,00	184	100,00	92,93	0,00	5,43
0,00	163	100,00	94,48	0,00	5,52
0,00	174	100,00	0,57	0,00	99,43
0,00	206	100,00	73,79	0,00	25,24
2,36	303	100,00	83,50	0,00	14,19
0,00	235	100,00	93,19	2,55	4,26
0,00	156	100,00	95,51	0,00	4,49
0,00	189	100,00	86,77	0,00	8,99
10,40	191	100,00	88,48	0,00	1,05
0,00	230	100,00	58,26	0,43	40,00
0,00	207	100,00	95,65	2,42	1,93
0,00	283	100,00	98,59	0,00	1,41
0,00	197	100,00	100,00	0,00	0,00
0,00	292	100,00	78,77	0,00	21,23
0,00	168	100,00	97,62	0,00	2,38
0,00	151	100,00	96,03	0,00	3,97
8,14	279	100,00	88,89	0,00	3,58
0,00	147	100,00	62,59	0,00	37,41
0,00	166	100,00	98,19	0,00	1,81
0,00	276	100,00	88,04	0,36	11,59
0,00	151	100,00	60,26	0,00	33,77
0,00	306	100,00	54,90	1,63	42,81
0,00	185	100,00	18,92	0,00	81,08
1,04	292	100,00	32,53	0,00	66,44
0,00	260	100,00	0,38	0,00	99,62

0,00	206	100,00	22,33	0,00	77,67
0,00	304	100,00	55,26	0,00	44,74
0,00	279	100,00	69,53	0,00	30,47
0,00	244	100,00	79,51	0,00	20,49
0,31	323	100,00	28,17	0,00	71,52
0,00	304	100,00	0,00	0,33	99,67
0,00	173	100,00	48,55	0,00	51,45
0,00	127	100,00	39,37	0,79	59,84
0,37	268	100,00	97,39	0,00	2,24
35,71	95	100,00	44,21	0,00	29,47
0,40	254	100,00	62,99	0,00	35,43
0,00	245	100,00	90,20	0,00	9,80
0,00	415	100,00	46,02	0,24	53,73
0,00	304	100,00	89,14	0,00	10,53
0,00	283	100,00	90,81	0,00	9,19
0,00	240	100,00	68,33	4,58	22,92
0,00	336	100,00	79,76	10,42	9,82
0,00	150	100,00	72,00	0,67	26,67
0,00	186	100,00	93,55	0,00	5,38
0,00	256	100,00	98,44	0,00	1,56
0,00	148	100,00	71,62	0,00	6,08
0,00	236	100,00	94,07	4,24	1,69
0,00	240	100,00	99,17	0,00	0,83
4,76	330	100,00	91,82	1,52	2,12
0,00	305	100,00	48,20	0,00	6,56
0,00	195	100,00	94,87	0,00	5,13
0,00	242	100,00	95,45	0,00	4,13
0,00	148	100,00	90,54	0,00	9,46
0,00	218	100,00	88,53	0,46	11,01
0,00	154	100,00	85,71	0,00	14,29
0,00	127	100,00	6,30	88,19	3,15
0,00	184	100,00	77,17	0,00	22,83
0,00	287	100,00	37,63	0,00	61,67
0,00	180	100,00	57,22	0,00	40,00
0,00	250	100,00	60,40	0,40	39,20
1,98	257	100,00	53,70	0,00	26,07
2,53	284	100,00	58,10	0,00	39,44
0,32	314	100,00	50,96	6,05	42,68
7,62	226	100,00	24,34	0,00	68,14
0,00	300	100,00	11,00	0,00	89,00
0,00	244	100,00	4,51	0,41	95,08
0,00	231	100,00	13,85	0,00	86,15
0,00	283	100,00	74,56	0,00	25,44
0,00	306	100,00	58,82	0,00	39,54
0,00	165	100,00	73,33	0,00	26,67
0,00	281	100,00	45,20	0,00	30,96
0,00	127	100,00	84,25	0,00	15,75
0,00	183	100,00	95,63	0,00	4,37
0,00	236	100,00	50,00	24,15	0,42
0,00	189	100,00	98,94	0,00	0,00

0,00	186	100,00	94,09	0,00	5,91
0,00	252	100,00	30,16	69,44	0,40
0,00	225	100,00	97,78	0,00	2,22
0,00	318	100,00	19,18	80,82	0,00
0,00	260	100,00	80,00	10,00	8,08
0,00	186	100,00	95,16	0,54	1,08
0,00	243	100,00	95,06	0,00	4,94
0,00	264	100,00	87,50	0,00	12,50
0,00	181	100,00	96,13	0,00	3,87
0,00	246	100,00	15,04	0,00	84,96
0,00	187	100,00	7,49	1,07	91,44
10,51	452	100,00	22,57	0,66	67,26
0,00	231	100,00	3,46	76,62	19,91
1,56	261	100,00	55,56	0,00	42,91
0,00	214	100,00	41,59	0,00	58,41
0,00	295	100,00	52,88	0,00	47,12
0,00	270	100,00	54,07	0,00	45,93
0,00	278	100,00	39,21	0,00	60,79
0,00	192	100,00	52,60	0,52	46,88
0,44	226	100,00	35,84	0,00	63,72
0,00	312	100,00	56,73	0,00	42,95
0,00	181	100,00	20,99	1,10	77,35
0,00	364	100,00	86,81	0,00	12,91
0,00	205	100,00	62,93	0,00	37,07
0,00	356	100,00	73,03	0,56	26,40
0,00	255	100,00	89,02	1,18	9,80
0,00	183	100,00	95,63	0,00	4,37
0,00	267	100,00	94,38	0,75	4,87
2,54	323	100,00	95,36	0,31	1,86
0,00	369	100,00	95,39	0,00	4,61
0,00	278	100,00	98,56	0,36	0,72
0,00	271	100,00	98,89	0,00	1,11
0,00	195	100,00	98,97	0,00	1,03
5,49	269	100,00	91,82	0,00	2,97
0,00	113	100,00	100,00	0,00	0,00
0,00	158	100,00	99,37	0,00	0,63
0,00	300	100,00	96,67	0,00	3,00
0,45	221	100,00	95,48	0,00	4,07
0,00	253	100,00	89,72	6,72	3,16
0,00	236	100,00	98,31	0,00	1,69
0,00	158	100,00	100,00	0,00	0,00
0,00	131	100,00	100,00	0,00	0,00
0,00	249	100,00	47,39	0,00	0,00
0,00	168	100,00	97,02	2,38	0,60
0,00	253	100,00	100,00	0,00	0,00
0,00	116	100,00	99,14	0,00	0,86
1,92	266	100,00	93,23	0,00	4,89
0,00	222	100,00	81,53	0,00	1,80
0,00	245	100,00	47,35	0,00	52,65
14,36	215	100,00	67,44	0,00	20,00

0,00	145	100,00	66,21	0,69	23,45
0,00	225	100,00	41,33	0,44	58,22
1,87	272	100,00	1,84	0,00	95,96
0,00	256	100,00	79,30	0,00	20,70
0,00	157	100,00	62,42	0,00	37,58
0,00	367	100,00	63,22	0,54	35,97
4,56	252	100,00	12,70	0,00	82,94
1,34	303	100,00	93,07	0,00	5,61
4,32	193	100,00	48,70	0,52	46,63
0,00	149	100,00	98,66	0,67	0,67
1,56	260	100,00	50,00	0,00	48,46
0,00	209	100,00	23,92	0,00	75,60
16,79	153	100,00	37,91	0,00	47,71
0,00	81	100,00	92,59	0,00	7,41
0,00	152	100,00	56,58	0,66	42,11
0,00	264	100,00	35,23	0,38	64,39
0,00	277	100,00	43,68	6,86	49,46
0,00	262	100,00	39,69	0,00	60,31
0,00	160	100,00	30,00	1,88	68,13
0,00	173	100,00	16,76	0,00	83,24
0,00	390	100,00	21,03	0,51	78,46
0,00	299	100,00	18,73	1,34	79,93
0,00	249	100,00	0,80	0,00	99,20
0,00	248	100,00	61,29	0,00	36,69
0,00	198	100,00	45,45	0,00	54,55
0,00	135	100,00	99,26	0,00	0,00
0,00	243	100,00	94,24	0,00	4,53
0,34	298	100,00	95,64	0,00	4,03
0,31	328	100,00	99,09	0,30	0,30
0,00	151	100,00	96,69	0,66	1,32
0,00	4	100,00	0,00	0,00	0,00
0,00	412	100,00	31,55	68,20	0,00
0,00	311	100,00	98,71	0,64	0,32
0,00	312	100,00	97,12	0,96	1,92
0,00	174	100,00	82,18	0,00	17,82
0,00	277	100,00	98,19	0,00	1,44
2,42	296	100,00	42,91	0,68	54,05
0,47	212	100,00	15,57	0,00	83,02
6,56	260	100,00	12,69	0,00	80,38
4,08	255	100,00	46,67	0,00	49,41
0,00	275	100,00	45,45	0,00	53,82
0,00	284	100,00	41,90	0,35	57,39
0,00	182	100,00	36,81	1,65	60,99
0,00	176	100,00	51,14	3,41	44,89
0,00	185	100,00	44,86	0,00	55,14
0,00	144	100,00	50,00	0,00	50,00
0,00	196	100,00	25,00	0,00	75,00
0,00	180	100,00	44,44	1,11	54,44
0,00	318	100,00	11,64	0,63	87,74
0,00	173	100,00	79,77	0,00	20,23

0,00	414	100,00	85,75	0,24	14,01
0,00	199	100,00	62,31	18,59	0,00
0,00	166	100,00	95,78	0,00	4,22
0,00	352	100,00	94,89	0,00	5,11
0,00	129	100,00	76,74	0,00	12,40
0,00	178	100,00	24,16	0,00	70,79
0,00	171	100,00	91,23	0,00	8,77
0,00	322	100,00	20,19	2,48	76,40
0,00	316	100,00	9,81	0,00	90,19
0,00	223	100,00	3,59	0,00	95,07
0,00	245	100,00	68,16	0,00	27,76
0,00	343	100,00	1,46	98,54	0,00
0,00	227	100,00	16,30	1,32	82,38
2,63	273	100,00	4,76	0,00	92,67
10,60	240	100,00	20,83	0,00	69,58
0,00	201	100,00	27,86	0,00	72,14
0,00	142	100,00	10,56	0,00	89,44
1,17	345	100,00	17,39	0,00	81,16
0,00	197	100,00	4,57	0,00	95,43
0,00	247	100,00	36,03	0,00	56,68
0,00	230	100,00	45,65	0,00	54,35
0,00	202	100,00	13,37	0,00	86,63
11,56	251	100,00	48,21	0,40	41,04
0,43	235	100,00	62,13	0,43	37,02
0,00	264	100,00	86,36	0,76	9,09
0,00	505	100,00	51,49	0,20	48,32
0,00	269	100,00	92,94	0,00	2,60
0,00	289	100,00	99,65	0,00	0,35
0,00	36	100,00	72,22	27,78	0,00
0,00	283	100,00	100,00	0,00	0,00
0,00	226	100,00	26,99	0,00	1,33
0,00	165	100,00	98,79	0,00	0,61
0,32	314	100,00	97,45	0,00	0,00
0,49	204	100,00	41,67	0,00	0,00
0,32	309	100,00	98,38	0,00	0,00
0,00	310	100,00	3,55	0,00	0,00
0,00	194	100,00	97,42	0,00	0,00
0,00	166	100,00	67,47	0,60	1,20
0,00	197	100,00	100,00	0,00	0,00
0,00	241	100,00	78,42	0,00	0,41
0,00	280	100,00	7,14	0,36	0,71
0,00	207	100,00	100,00	0,00	0,00
0,00	134	100,00	44,78	0,00	0,00
0,00	128	100,00	2,34	0,00	0,78
0,00	162	100,00	67,90	0,62	31,48
0,00	65	100,00	21,54	0,00	76,92
0,00	13	100,00	100,00	0,00	0,00
0,00	113	100,00	57,52	0,00	42,48
22,61	244	100,00	20,08	0,00	61,48
2,27	180	100,00	8,33	0,56	88,33

0,00	52	100,00	26,92	0,00	73,08
0,00	80	100,00	35,00	0,00	65,00
0,00	161	100,00	42,24	0,00	57,76
0,00	132	100,00	37,88	0,76	61,36
0,00	164	100,00	35,98	0,00	64,02
0,00	156	100,00	62,82	0,00	37,18
0,00	156	100,00	27,56	0,00	72,44
0,00	167	100,00	25,15	0,00	70,66
0,00	205	100,00	48,78	0,98	50,24
0,00	137	100,00	54,01	0,00	45,99
0,00	103	100,00	9,71	0,00	71,84
0,47	212	100,00	43,87	0,00	55,66
0,00	146	100,00	45,21	0,68	54,11
0,00	195	100,00	43,59	0,00	56,41
0,00	171	100,00	53,80	0,00	46,20
0,00	217	100,00	44,70	0,00	54,84
0,00	141	100,00	73,05	0,00	26,95
0,00	118	100,00	20,34	0,00	79,66
0,00	50	100,00	62,00	0,00	38,00
0,00	39	100,00	46,15	0,00	53,85
0,00	117	100,00	65,81	0,00	34,19
0,00	257	100,00	20,23	0,00	79,77
0,00	49	100,00	42,86	0,00	57,14
0,00	143	100,00	12,59	5,59	81,82
0,00	220	100,00	30,91	0,00	69,09
0,00	165	100,00	42,42	0,00	57,58
0,00	29	100,00	6,90	0,00	93,10
0,00	151	100,00	98,68	0,00	0,00
0,00	19	100,00	100,00	0,00	0,00
0,00	80	100,00	61,25	0,00	38,75
13,43	76	100,00	76,32	0,00	11,84
0,00	49	100,00	89,80	0,00	10,20
0,00	148	100,00	62,16	4,05	33,78
0,00	6	100,00	100,00	0,00	0,00
7,95	163	100,00	48,47	0,00	44,17
0,00	0	0,00	0,00	0,00	0,00
1,87	109	100,00	27,52	9,17	0,00
0,00	57	100,00	100,00	0,00	0,00
0,00	68	100,00	91,18	5,88	0,00
0,00	99	100,00	96,97	0,00	2,02
0,00	111	100,00	96,40	0,00	3,60
0,00	0	0,00	0,00	0,00	0,00
0,00	31	100,00	100,00	0,00	0,00
0,00	202	100,00	98,02	0,00	1,98
0,00	60	100,00	58,33	41,67	0,00
0,00	168	100,00	94,05	0,00	5,95
0,00	194	100,00	94,85	0,00	4,64
0,00	176	100,00	98,30	0,00	1,70
0,00	119	100,00	78,15	0,00	21,85
0,00	197	100,00	58,88	0,00	41,12

0,00	212	100,00	2,36	96,23	1,42
3,88	134	100,00	0,00	96,27	0,00
283,33	23	100,00	26,09	0,00	0,00
0,00	13	100,00	76,92	0,00	15,38
0,00	33	100,00	100,00	0,00	0,00
86,21	108	100,00	52,78	0,00	0,93
4,15	201	100,00	95,02	0,00	0,50
0,00	151	100,00	3,31	0,00	96,69
0,00	163	100,00	93,25	0,00	6,75
0,00	107	100,00	14,02	85,98	0,00
0,00	4	100,00	0,00	0,00	0,00
0,96	105	100,00	98,10	0,95	0,00
0,00	62	100,00	98,39	0,00	0,00
0,00	185	100,00	83,78	0,00	11,35
0,00	11	100,00	100,00	0,00	0,00
0,00	242	100,00	52,07	1,24	39,26
0,00	150	100,00	95,33	0,00	2,67
0,00	49	100,00	0,00	0,00	0,00
0,00	126	100,00	98,41	0,79	0,00
0,00	172	100,00	80,81	0,00	0,58
0,00	115	100,00	98,26	0,00	1,74
0,00	240	100,00	94,17	4,17	0,83
0,00	92	100,00	100,00	0,00	0,00
0,00	58	100,00	100,00	0,00	0,00
0,00	110	100,00	3,64	96,36	0,00
0,00	17	100,00	100,00	0,00	0,00
0,00	450	100,00	1,11	0,22	98,67
0,00	89	100,00	91,01	0,00	8,99
0,00	90	100,00	34,44	0,00	65,56
0,00	121	100,00	100,00	0,00	0,00
0,00	53	100,00	32,08	67,92	0,00
0,00	19	100,00	63,16	0,00	36,84
0,61	164	100,00	84,15	15,24	0,00
0,00	160	100,00	99,38	0,00	0,63
0,00	109	100,00	100,00	0,00	0,00
0,00	221	100,00	95,48	0,00	4,52
0,00	181	100,00	100,00	0,00	0,00
0,00	124	100,00	87,10	12,90	0,00
0,00	46	100,00	100,00	0,00	0,00
1,08	187	100,00	59,36	0,00	39,57
2,94	175	100,00	70,86	6,86	19,43
0,00	189	100,00	96,30	0,53	3,17
0,00	62	100,00	100,00	0,00	0,00
0,00	137	100,00	83,94	0,00	16,06
0,00	53	100,00	88,68	0,00	11,32
0,00	29	100,00	96,55	0,00	3,45
0,41	245	100,00	98,37	0,00	1,22
0,00	112	100,00	50,89	0,00	48,21
355,00	182	100,00	12,64	0,00	9,34
0,00	101	100,00	9,90	0,99	89,11

0,00	324	100,00	4,94	2,78	92,28
0,00	241	100,00	49,79	0,00	50,21
0,00	233	100,00	41,20	0,43	55,79
0,00	167	100,00	97,60	0,00	2,40
0,00	16	100,00	100,00	0,00	0,00
0,00	3	100,00	0,00	0,00	0,00
0,00	249	100,00	82,73	0,00	17,27
0,00	47	100,00	89,36	0,00	10,64
0,00	173	100,00	88,44	0,00	11,56
0,00	180	100,00	95,56	3,33	1,11
0,00	215	100,00	99,07	0,47	0,47
0,00	172	100,00	98,26	1,74	0,00
0,00	139	100,00	63,31	0,00	36,69
0,00	111	100,00	0,90	0,00	0,90
0,00	60	100,00	10,00	0,00	0,00
7,41	87	100,00	93,10	0,00	0,00
0,00	19	100,00	100,00	0,00	0,00
0,00	28	100,00	100,00	0,00	0,00
0,00	109	100,00	100,00	0,00	0,00
0,00	197	100,00	48,22	1,02	0,00
0,00	85	100,00	9,41	0,00	1,18
0,00	236	100,00	92,80	0,00	7,20
0,46	220	100,00	95,45	0,45	2,73
0,00	277	100,00	92,42	0,00	7,58
0,63	161	100,00	98,14	0,00	1,24
0,00	49	100,00	95,92	0,00	4,08
0,00	233	100,00	4,29	0,00	95,71
0,00	206	100,00	33,98	0,00	66,02
0,00	226	100,00	22,12	0,00	77,88
0,00	294	100,00	62,59	7,82	12,24
0,00	230	100,00	87,83	0,00	0,87
0,00	131	100,00	81,68	3,82	14,50
3,32	249	100,00	28,11	0,80	67,87
0,00	116	100,00	58,62	0,00	41,38
0,00	237	100,00	46,84	3,38	49,79
0,00	232	100,00	23,71	0,00	76,29
0,00	172	100,00	99,42	0,00	0,58
0,00	221	100,00	9,05	0,00	90,95
0,00	207	100,00	13,04	0,48	86,47
0,00	3	100,00	0,00	0,00	0,00
0,00	183	100,00	9,84	0,55	89,62
0,00	177	100,00	95,48	0,00	4,52
0,00	207	100,00	2,90	2,42	94,69
0,00	293	100,00	43,34	0,68	55,97
0,00	214	100,00	39,72	0,00	58,88
0,00	306	100,00	93,79	0,00	6,21
0,00	290	100,00	97,93	0,34	1,72
0,00	283	100,00	97,53	0,00	2,47
0,00	203	100,00	4,43	93,60	1,97
0,00	214	100,00	95,79	0,00	4,21

0,00	194	100,00	70,62	0,00	1,03
0,00	154	100,00	100,00	0,00	0,00
0,00	0	0,00	0,00	0,00	0,00
0,00	83	100,00	14,46	0,00	85,54
0,00	268	100,00	38,43	2,24	59,33
5,80	292	100,00	35,27	0,00	59,25
0,00	195	100,00	37,95	0,00	59,49
0,00	211	100,00	42,65	0,00	57,35
0,00	210	100,00	99,05	0,00	0,95
0,00	95	100,00	9,47	0,00	90,53
0,00	100	100,00	87,00	0,00	13,00
0,00	173	100,00	83,24	0,00	16,76
0,00	272	100,00	94,12	0,00	5,88
0,00	335	100,00	95,52	0,00	4,48
787,50	71	100,00	8,45	0,00	2,82
0,00	334	100,00	33,23	0,30	66,47
0,00	219	100,00	10,05	0,00	89,95
0,00	268	100,00	1,87	0,00	98,13
0,00	21	100,00	90,48	0,00	9,52
0,36	278	100,00	80,94	0,00	15,47
0,00	299	100,00	91,30	0,33	7,69
0,00	165	100,00	90,91	0,61	8,48
0,00	236	100,00	82,20	0,42	9,32
0,00	227	100,00	100,00	0,00	0,00
0,00	168	100,00	100,00	0,00	0,00
0,00	145	100,00	100,00	0,00	0,00
0,00	50	100,00	100,00	0,00	0,00
0,00	158	100,00	100,00	0,00	0,00
0,00	193	100,00	89,64	0,00	3,63
0,00	304	100,00	100,00	0,00	0,00
0,00	240	100,00	99,58	0,00	0,42
7,62	226	100,00	92,04	0,00	0,88
0,00	307	100,00	98,70	0,00	0,65
0,00	165	100,00	100,00	0,00	0,00
0,00	235	100,00	86,38	0,00	13,62
0,00	187	100,00	90,91	0,00	9,09
0,00	163	100,00	98,16	0,00	1,84
0,00	159	100,00	99,37	0,63	0,00
0,57	175	100,00	98,29	1,14	0,00
0,00	118	100,00	100,00	0,00	0,00
0,00	174	100,00	94,83	0,00	5,17
0,00	355	100,00	80,56	19,15	0,28
0,00	276	100,00	98,91	0,36	0,36
0,33	305	100,00	99,02	0,00	0,66
0,35	285	100,00	99,30	0,35	0,00
0,00	121	100,00	93,39	6,61	0,00
0,00	125	100,00	28,00	0,00	1,60
0,00	109	100,00	23,85	0,00	76,15
0,00	45	100,00	97,78	0,00	2,22
0,00	0	0,00	0,00	0,00	0,00

0,00	294	100,00	98,98	0,00	0,00
14,15	121	100,00	38,02	0,00	49,59
0,00	254	100,00	100,00	0,00	0,00
0,00	149	100,00	100,00	0,00	0,00
0,00	83	100,00	25,30	74,70	0,00
0,83	121	100,00	89,26	0,83	0,00
0,00	175	100,00	100,00	0,00	0,00
0,00	268	100,00	53,36	0,00	46,64
0,37	271	100,00	42,07	21,77	35,79
0,00	156	100,00	62,82	0,00	37,18
0,00	119	100,00	94,96	0,00	5,04
0,60	338	100,00	48,52	0,59	50,30
0,73	276	100,00	48,55	0,00	50,72
0,00	193	100,00	2,07	0,00	97,93
0,00	214	100,00	61,21	0,93	32,24
0,00	192	100,00	82,29	0,00	17,71
0,00	183	100,00	79,78	0,00	19,67
0,00	309	100,00	57,28	5,18	21,04
4,04	335	100,00	75,22	0,00	20,60
0,00	280	100,00	92,50	0,00	2,86
0,00	296	100,00	53,04	0,68	45,95
0,00	214	100,00	80,84	0,93	18,22
0,00	188	100,00	67,02	0,00	32,98
1,20	169	100,00	89,35	0,00	8,88
0,00	202	100,00	87,13	0,00	12,87
0,00	285	100,00	96,49	0,00	2,46
0,00	292	100,00	99,66	0,34	0,00
0,33	306	100,00	97,06	0,00	2,61
0,29	344	100,00	60,76	0,00	38,95
0,00	327	100,00	82,87	0,31	16,82
0,00	207	100,00	31,40	0,00	68,60
0,00	320	100,00	3,75	0,31	95,31
0,00	31	100,00	100,00	0,00	0,00
0,00	190	100,00	80,53	0,00	18,42
0,00	243	100,00	88,48	0,00	11,52
0,00	125	100,00	92,80	0,00	7,20
0,00	317	100,00	98,74	0,00	1,26
0,00	355	100,00	90,14	0,00	3,94
0,00	263	100,00	90,49	0,38	2,28
0,00	381	100,00	42,78	7,09	50,13
0,00	201	100,00	1,49	1,00	96,52
0,00	196	100,00	0,51	0,00	99,49
0,00	252	100,00	34,52	0,00	65,48
0,00	283	100,00	18,73	0,00	81,27
0,00	283	100,00	7,42	0,00	92,58
0,00	169	100,00	15,98	0,59	83,43
0,00	297	100,00	0,34	0,00	99,66
0,00	139	100,00	8,63	0,00	91,37
0,00	264	100,00	43,18	0,00	56,82
0,00	206	100,00	64,56	0,00	35,44

0,00	290	100,00	66,55	0,00	33,45
0,00	305	100,00	78,03	0,33	21,64
0,00	271	100,00	77,49	0,37	21,77
0,32	317	100,00	72,24	0,00	25,24
0,57	177	100,00	84,75	1,13	13,56
0,00	268	100,00	67,91	0,00	12,69
0,00	163	100,00	96,93	0,00	3,07
0,00	307	100,00	85,99	0,00	6,51
0,00	254	100,00	96,06	0,00	3,94
0,00	203	100,00	100,00	0,00	0,00
0,00	266	100,00	96,24	0,00	3,76
0,00	254	100,00	91,73	0,00	8,27
0,00	299	100,00	2,34	0,33	97,32
0,00	296	100,00	4,05	0,00	95,95
0,00	297	100,00	5,05	0,34	94,61
0,00	243	100,00	3,70	0,00	96,30
0,00	301	100,00	2,33	0,00	97,67
0,00	266	100,00	6,77	0,75	92,48
0,00	223	100,00	4,04	0,00	95,96
0,00	297	100,00	14,81	2,02	83,16
0,00	202	100,00	18,32	0,00	81,68
0,45	224	100,00	8,93	0,00	90,63
0,00	318	100,00	1,57	0,31	98,11
0,00	341	100,00	40,18	0,59	59,24
0,00	289	100,00	12,80	0,35	82,70
0,00	174	100,00	38,51	1,72	59,77
3,65	284	100,00	83,10	0,00	11,62
0,00	209	100,00	92,34	0,00	7,66
0,00	98	100,00	84,69	0,00	15,31
0,00	177	100,00	98,87	0,00	1,13
0,00	288	100,00	84,38	0,00	14,58
0,00	180	100,00	97,78	0,00	2,22
0,00	315	100,00	90,48	0,32	9,21
0,00	289	100,00	91,00	0,35	8,65
0,00	394	100,00	22,34	1,02	76,65
0,00	539	100,00	20,04	0,74	79,22
3,59	346	100,00	89,60	0,00	6,94
3,39	305	100,00	53,77	0,00	31,15
0,00	266	100,00	47,37	0,00	47,37
0,00	320	100,00	19,69	0,63	79,69
0,00	307	100,00	69,06	1,30	29,64
3,11	332	100,00	26,81	3,92	66,27
0,00	319	100,00	17,24	0,00	82,76
0,00	277	100,00	9,75	0,00	90,25
0,00	153	100,00	57,52	0,00	42,48
0,00	285	100,00	62,11	0,35	37,19
0,00	149	100,00	55,03	0,00	44,97
10,69	290	100,00	78,62	1,03	10,69
0,00	240	100,00	81,67	0,42	17,92
0,00	255	100,00	92,94	1,57	5,10

0,00	266	100,00	95,49	0,00	4,51
0,00	229	100,00	96,07	0,00	3,93
0,00	243	100,00	98,77	0,00	1,23
0,00	251	100,00	42,63	39,84	0,40
8,07	174	100,00	53,45	18,97	16,09
0,00	157	100,00	76,43	0,00	23,57
0,34	294	100,00	89,80	0,34	9,52
0,00	154	100,00	94,16	0,65	5,19
1,07	284	100,00	86,62	0,00	7,39
0,46	220	100,00	96,36	0,00	0,45
0,95	212	100,00	75,94	5,19	9,91
0,53	188	100,00	81,38	1,60	10,11
207,69	120	100,00	30,83	0,00	1,67
0,00	262	100,00	71,37	5,73	17,56
0,00	301	100,00	83,39	5,65	10,63
4,32	290	100,00	94,48	0,00	1,38
0,00	326	100,00	13,50	22,39	64,11
0,29	343	100,00	75,22	0,00	4,96
0,00	276	100,00	82,97	0,36	7,97
0,00	312	100,00	99,04	0,00	0,96
0,60	168	100,00	86,31	0,00	13,10
0,00	295	100,00	80,68	0,00	19,32
0,00	232	100,00	81,47	0,00	18,53
1,37	297	100,00	37,04	0,34	61,28
0,00	127	100,00	53,54	0,79	45,67
0,00	211	100,00	39,81	1,42	58,77
0,00	294	100,00	1,02	0,34	98,64
0,00	51	100,00	98,04	0,00	1,96
0,00	99	100,00	100,00	0,00	0,00
0,70	289	100,00	90,66	0,00	8,65
0,00	287	100,00	49,13	0,35	50,52
0,00	298	100,00	86,91	0,00	13,09
0,38	262	100,00	22,14	3,05	74,43
9,95	221	100,00	61,99	0,00	28,96
0,00	266	100,00	83,46	0,00	16,54
0,00	157	100,00	84,71	0,00	15,29
0,34	296	100,00	19,26	0,68	79,73
0,00	75	100,00	93,33	0,00	6,67
0,00	111	100,00	54,05	0,90	36,94
0,00	201	100,00	50,25	0,50	49,25
0,00	0	0,00	0,00	0,00	0,00
0,00	215	100,00	66,98	0,47	3,26
0,00	0	0,00	0,00	0,00	0,00
0,00	241	100,00	95,44	0,00	4,56
1,71	178	100,00	98,31	0,00	0,00
0,00	306	100,00	93,79	0,00	6,21
0,00	322	100,00	13,35	1,24	85,09
2,67	269	100,00	48,70	0,00	48,70
0,19	533	100,00	24,77	1,13	73,92
0,00	45	100,00	86,67	0,00	13,33

0,00	185	100,00	78,38	1,08	14,59
0,00	310	100,00	94,19	0,00	5,81
0,00	44	100,00	93,18	0,00	6,82
0,00	286	100,00	51,75	5,24	12,59
0,00	283	100,00	38,87	0,00	55,48
0,00	263	100,00	50,57	0,38	49,05
12,30	210	100,00	25,71	14,76	48,57
0,65	311	100,00	13,50	1,61	77,17
0,00	132	100,00	87,12	9,09	3,79
0,00	210	100,00	99,52	0,00	0,48
0,00	247	100,00	97,98	0,00	2,02
0,00	230	100,00	96,96	0,00	3,04
0,00	245	100,00	3,27	0,00	96,73
0,00	191	100,00	2,62	0,00	97,38
0,00	130	100,00	43,85	0,77	34,62
0,00	68	100,00	45,59	0,00	50,00
0,00	231	100,00	54,98	0,43	43,72
0,00	84	100,00	57,14	0,00	42,86
0,00	60	100,00	65,00	0,00	35,00
0,59	170	100,00	56,47	0,00	42,35
0,00	208	100,00	14,90	0,00	85,10
0,00	144	100,00	32,64	47,22	20,14
0,00	95	100,00	41,05	0,00	58,95
0,00	72	100,00	19,44	0,00	80,56
0,00	157	100,00	19,75	0,00	80,25
0,00	179	100,00	1,12	0,00	98,32
0,00	200	100,00	99,50	0,00	0,50
0,00	162	100,00	90,12	1,23	7,41
0,00	27	100,00	96,30	0,00	3,70
0,00	75	100,00	96,00	0,00	4,00
1,61	63	100,00	20,63	68,25	0,00
0,70	288	100,00	35,76	14,58	48,61
5,77	165	100,00	60,61	0,61	33,33
0,00	97	0,00	0,00	0,00	0,00
0,00	130	100,00	0,77	0,00	99,23
0,00	145	100,00	26,90	0,00	68,97
0,00	59	100,00	5,08	3,39	91,53
0,00	98	100,00	56,12	0,00	43,88
0,69	293	100,00	13,31	0,00	85,67
0,00	34	100,00	70,59	0,00	29,41
0,00	124	100,00	50,00	0,81	49,19
0,00	38	100,00	89,47	0,00	10,53
0,00	35	100,00	97,14	0,00	2,86
0,00	153	100,00	9,15	0,00	90,85
0,00	93	100,00	63,44	1,08	35,48
0,00	156	100,00	100,00	0,00	0,00
0,00	210	100,00	0,95	0,48	98,57
0,00	79	100,00	13,92	0,00	86,08
0,00	95	100,00	44,21	0,00	55,79
0,00	67	100,00	35,82	0,00	64,18

0,00	190	100,00	10,00	0,00	90,00
0,00	178	100,00	70,22	0,00	29,78
0,00	189	100,00	86,24	0,00	4,23
0,00	48	100,00	68,75	4,17	27,08
0,00	40	100,00	40,00	55,00	5,00
0,00	47	100,00	95,74	0,00	4,26
0,00	58	100,00	96,55	0,00	3,45
0,00	159	100,00	88,05	0,00	11,95
0,00	76	100,00	59,21	6,58	34,21
0,00	15	100,00	73,33	6,67	20,00
0,00	12	100,00	100,00	0,00	0,00
0,00	0	0,00	0,00	0,00	0,00
0,69	145	100,00	91,72	0,00	7,59
0,00	180	100,00	66,67	0,00	33,33
0,00	10	100,00	100,00	0,00	0,00
0,00	236	100,00	51,27	0,42	48,31
0,00	87	100,00	6,90	14,94	73,56
0,00	141	100,00	9,93	1,42	0,00
0,00	108	100,00	2,78	21,30	75,93
0,00	142	100,00	30,99	0,00	69,01
0,00	237	100,00	100,00	0,00	0,00
0,00	152	100,00	100,00	0,00	0,00
0,00	180	100,00	22,22	0,56	77,22
0,00	0	0,00	0,00	0,00	0,00
0,00	12	100,00	83,33	0,00	16,67
0,00	89	100,00	82,02	0,00	17,98
0,00	266	100,00	100,00	0,00	0,00
0,00	217	100,00	97,24	0,00	2,30
0,00	47	100,00	65,96	0,00	34,04
0,00	25	100,00	80,00	4,00	16,00
0,00	180	100,00	44,44	0,00	54,44
2,86	252	100,00	69,84	0,00	20,63
0,34	296	100,00	23,65	0,34	75,68
0,00	259	100,00	45,17	6,95	46,33
0,00	226	100,00	28,76	0,44	62,83
0,00	267	100,00	60,67	0,37	33,71
0,00	199	100,00	31,16	0,00	68,34
1,62	251	100,00	25,90	0,00	72,51
2,26	317	100,00	38,17	0,00	59,62
0,00	57	0,00	0,00	0,00	0,00
0,00	337	100,00	27,89	2,97	69,14
0,41	247	100,00	47,77	0,00	51,01
0,00	212	100,00	35,38	0,00	64,62
2,60	158	100,00	94,30	0,00	2,53
0,00	210	100,00	99,52	0,00	0,48
0,00	249	100,00	100,00	0,00	0,00
3,86	215	100,00	96,28	0,00	0,00
0,71	141	100,00	97,16	0,00	1,42
0,00	229	100,00	95,63	3,93	0,44
0,00	384	100,00	74,22	13,02	9,11

1500,00	16	100,00	0,00	0,00	0,00
0,69	145	100,00	54,48	0,69	44,14
0,61	164	100,00	52,44	0,61	42,07
6,74	206	100,00	61,17	0,49	28,64
0,36	280	100,00	88,21	0,00	10,36
0,31	327	100,00	95,72	0,00	0,92
0,00	240	100,00	88,75	1,25	10,00
0,00	290	100,00	97,59	0,00	2,41
0,00	188	100,00	85,11	0,00	14,89
0,00	261	100,00	76,25	1,15	8,05
0,00	192	100,00	46,35	0,00	5,21
0,00	293	100,00	95,22	2,39	0,68
0,00	333	100,00	99,10	0,00	0,90
0,00	281	100,00	94,66	0,00	4,63
0,00	215	100,00	48,84	0,47	5,12
0,00	312	100,00	88,46	4,49	7,05
1,12	181	100,00	61,33	0,00	12,71
0,00	168	100,00	91,67	0,00	8,33
0,00	249	100,00	74,30	9,64	16,06
3,10	266	100,00	64,29	1,13	31,58
0,00	181	100,00	44,20	11,05	43,65
0,00	224	100,00	4,91	0,00	95,09
0,00	212	100,00	7,55	0,47	91,98
0,00	252	100,00	93,25	0,00	6,75
0,84	241	100,00	63,07	0,83	35,27
0,00	108	100,00	45,37	0,00	54,63
0,00	271	100,00	39,85	0,74	59,04
0,00	179	100,00	41,90	0,00	58,10
0,00	295	100,00	62,03	0,00	36,27
0,00	258	100,00	53,49	8,14	35,27
0,00	207	100,00	74,88	0,00	24,15
39,87	214	100,00	38,32	4,21	27,10
0,00	283	100,00	49,47	0,71	49,82
0,36	278	100,00	53,96	0,36	45,32
0,69	146	100,00	4,11	0,00	95,21
0,00	186	100,00	79,03	0,00	20,97
0,00	279	100,00	75,63	1,43	22,94
9,30	188	100,00	69,68	0,00	15,96
0,36	279	100,00	96,77	0,00	2,87
5,36	177	100,00	70,06	0,56	24,29
0,00	131	100,00	15,27	0,76	83,97
0,00	176	100,00	63,07	0,00	36,93
2,99	207	100,00	38,65	19,32	39,13
0,00	292	100,00	53,42	0,00	46,58
0,00	75	100,00	34,67	1,33	64,00
0,00	170	100,00	55,88	0,00	44,12
5,63	225	100,00	17,33	0,44	76,00
1,53	265	100,00	19,25	0,38	78,49
0,00	320	100,00	2,19	0,00	97,81
0,68	296	100,00	14,53	0,00	84,12

0,00	215	100,00	25,12	0,00	71,63
0,00	208	100,00	6,25	1,44	91,83
5,63	244	100,00	22,54	9,84	62,30
0,00	147	100,00	100,00	0,00	0,00
0,00	197	100,00	61,42	0,00	38,58
0,00	191	100,00	78,01	2,62	19,37
0,00	95	100,00	96,84	0,00	3,16
0,00	78	100,00	84,62	0,00	12,82
0,00	274	100,00	72,99	0,00	7,30
13,00	339	100,00	68,14	0,00	20,35
0,00	303	100,00	77,89	0,33	21,78
0,00	232	100,00	96,98	0,00	3,02
0,00	220	100,00	98,64	1,36	0,00
7,08	363	100,00	92,84	0,00	0,55
0,00	260	100,00	84,62	0,77	13,85
0,00	315	100,00	99,37	0,32	0,32
0,00	351	100,00	99,15	0,00	0,28
0,00	319	100,00	98,43	0,00	1,25
0,00	327	100,00	85,02	0,00	3,98
0,00	141	100,00	89,36	0,00	2,13
0,00	263	100,00	100,00	0,00	0,00
0,00	252	100,00	99,60	0,00	0,40
0,34	294	100,00	97,62	0,00	2,04
0,00	236	100,00	96,61	0,00	3,39
0,00	242	100,00	100,00	0,00	0,00
0,00	389	100,00	97,94	0,00	1,80
0,00	240	100,00	100,00	0,00	0,00
0,00	145	100,00	99,31	0,00	0,69
5,56	152	100,00	94,08	0,00	0,66
0,00	175	100,00	84,00	0,00	3,43
0,00	191	100,00	96,34	0,00	2,09
0,00	260	100,00	90,77	0,00	9,23
0,00	349	100,00	85,10	0,00	14,90
3,03	272	100,00	83,82	4,41	7,35
0,72	140	100,00	90,00	0,71	7,86
0,00	133	100,00	31,58	0,00	6,02
0,00	251	100,00	34,66	0,00	63,75
0,00	297	100,00	41,41	0,34	58,25
0,00	295	100,00	33,90	1,69	64,07
0,00	225	100,00	48,89	8,00	43,11
0,00	317	100,00	53,63	0,95	44,79
0,00	262	100,00	12,21	0,38	87,40
0,00	313	100,00	2,56	0,96	96,49
0,00	303	100,00	1,32	0,33	98,35
0,00	261	100,00	38,31	0,38	61,30
0,00	153	100,00	56,86	0,00	43,14
0,00	264	100,00	61,74	0,76	37,50
0,00	210	100,00	44,76	0,95	54,29
0,00	170	100,00	58,82	0,00	39,41
0,00	245	100,00	45,71	1,63	51,84

0,00	201	100,00	35,82	3,48	60,70
0,00	213	100,00	64,32	5,63	30,05
0,00	198	100,00	33,33	2,02	64,65
2,22	322	100,00	28,88	0,00	68,94
0,00	38	100,00	92,11	0,00	2,63
0,00	7	100,00	85,71	0,00	14,29
0,00	25	100,00	48,00	52,00	0,00
0,00	79	100,00	67,09	0,00	13,92
0,00	20	100,00	100,00	0,00	0,00
0,00	29	100,00	0,00	0,00	0,00
0,00	2	100,00	0,00	0,00	0,00
0,00	40	100,00	97,50	0,00	2,50
0,00	3	100,00	0,00	0,00	0,00
0,00	66	100,00	40,91	0,00	18,18
0,00	182	100,00	100,00	0,00	0,00
0,00	15	100,00	93,33	0,00	6,67
0,00	20	100,00	90,00	0,00	0,00
0,00	2	100,00	0,00	0,00	0,00
0,00	73	100,00	100,00	0,00	0,00
0,00	185	100,00	49,19	0,54	50,27
0,00	112	100,00	99,11	0,89	0,00
0,00	73	100,00	76,71	0,00	23,29
0,00	192	100,00	15,63	0,00	84,38
0,00	63	100,00	82,54	15,87	1,59
0,00	84	100,00	92,86	7,14	0,00
0,00	80	100,00	88,75	1,25	0,00
1,30	78	100,00	20,51	0,00	78,21
0,00	142	100,00	13,38	2,82	83,10
0,00	258	100,00	100,00	0,00	0,00
0,00	166	100,00	99,40	0,00	0,60
0,00	31	100,00	41,94	0,00	58,06
0,00	25	100,00	24,00	0,00	76,00
0,00	96	100,00	95,83	0,00	4,17
0,00	157	100,00	100,00	0,00	0,00
0,00	307	100,00	100,00	0,00	0,00
0,00	240	100,00	99,58	0,00	0,00
0,00	235	100,00	100,00	0,00	0,00
0,00	66	100,00	100,00	0,00	0,00
0,00	157	100,00	100,00	0,00	0,00
0,00	64	100,00	100,00	0,00	0,00
0,00	138	100,00	97,10	0,00	2,90
0,00	50	100,00	100,00	0,00	0,00
0,00	62	0,00	0,00	0,00	0,00
0,00	88	100,00	100,00	0,00	0,00
0,00	138	100,00	100,00	0,00	0,00
0,00	163	100,00	100,00	0,00	0,00
2,42	339	100,00	54,87	0,00	39,82
0,00	220	100,00	82,27	1,82	14,09
0,00	325	100,00	77,85	0,00	22,15
0,00	123	100,00	90,24	0,00	9,76

0,00	325	100,00	64,62	0,00	29,23
0,00	0	0,00	0,00	0,00	0,00
0,00	249	100,00	92,77	0,00	7,23
0,00	205	100,00	99,02	0,00	0,98
0,00	301	100,00	88,04	0,66	10,96
0,31	326	100,00	89,57	0,61	3,68
0,00	284	100,00	94,72	0,35	4,93
3,00	309	100,00	94,82	0,00	2,27
0,00	295	100,00	96,27	3,39	0,34
0,00	248	100,00	95,97	0,40	3,63
0,00	142	100,00	100,00	0,00	0,00
0,00	277	100,00	79,42	0,00	11,19
0,00	127	100,00	51,18	15,75	25,98
0,40	254	100,00	98,43	0,00	0,39
0,00	317	100,00	70,35	0,63	29,02
0,29	341	100,00	77,71	0,00	21,99
0,00	269	100,00	55,02	0,37	44,24
0,00	100	100,00	93,00	0,00	6,00
10,66	218	100,00	50,92	0,46	38,99
2,40	256	100,00	48,05	0,78	48,83
0,00	201	100,00	58,71	0,00	41,29
0,00	260	100,00	68,08	0,38	31,15
0,00	242	100,00	79,34	0,83	19,42
0,00	100	100,00	93,00	4,00	3,00
0,00	228	100,00	3,95	0,00	96,05
0,00	263	100,00	8,37	0,00	90,87
0,00	129	100,00	28,68	0,00	71,32
0,00	11	100,00	100,00	0,00	0,00
0,00	177	100,00	88,14	0,00	11,86
0,00	302	100,00	11,92	0,00	88,08
1,75	232	100,00	58,62	6,90	32,76
0,00	225	100,00	12,44	5,78	81,78
0,00	34	100,00	100,00	0,00	0,00
0,00	137	100,00	91,24	0,00	8,76
0,00	260	100,00	75,00	0,00	25,00
0,23	442	100,00	38,01	0,45	61,31
0,00	29	100,00	96,55	0,00	3,45
0,00	239	100,00	94,56	0,00	5,44
0,00	201	100,00	80,60	0,00	19,40
0,00	165	100,00	64,85	0,00	34,55
2,34	131	100,00	97,71	0,00	0,00
0,00	266	100,00	32,71	1,88	65,04
0,68	444	100,00	21,85	0,45	77,03
0,00	263	100,00	93,92	0,00	6,08
0,00	218	100,00	97,71	0,00	2,29
0,00	338	100,00	25,44	0,59	73,96
0,00	240	100,00	34,17	0,00	65,83
4,04	206	100,00	96,12	0,00	0,00
0,00	293	100,00	19,80	2,73	77,47
0,00	193	100,00	1,04	9,33	89,64

0,29	351	100,00	16,24	9,97	73,50
0,00	288	100,00	90,63	0,35	9,03
0,00	369	100,00	91,60	0,00	8,40
0,00	279	100,00	5,02	0,72	94,27
0,00	311	100,00	98,07	0,00	1,93
0,32	312	100,00	95,19	0,00	4,49
0,00	237	100,00	67,51	0,42	32,07
0,00	253	100,00	97,23	0,00	2,77
0,00	309	100,00	14,24	0,00	85,76
0,00	340	100,00	95,29	0,29	2,35
0,00	310	100,00	3,23	1,29	92,90
0,00	292	100,00	6,16	0,00	93,84
0,00	109	100,00	100,00	0,00	0,00
0,00	201	100,00	90,05	6,97	2,99
0,69	146	100,00	4,79	93,84	0,68
0,00	294	100,00	4,42	65,65	29,93
0,00	135	100,00	85,19	0,74	14,07
0,00	239	100,00	97,49	0,00	2,51
1,88	217	100,00	33,64	0,00	64,52
0,00	138	100,00	98,55	0,00	1,45
0,00	229	100,00	99,56	0,00	0,44
0,00	258	100,00	98,84	0,00	0,78
0,00	226	100,00	100,00	0,00	0,00
0,00	210	100,00	99,52	0,00	0,00
0,00	62	100,00	100,00	0,00	0,00
1,32	154	100,00	14,29	84,42	0,00
0,64	157	100,00	95,54	0,00	0,64
0,00	125	100,00	26,40	0,00	0,80
1,49	273	100,00	97,80	0,37	0,37
0,00	359	100,00	100,00	0,00	0,00
0,00	318	100,00	98,74	0,94	0,31
0,00	437	100,00	97,94	0,00	2,06
0,00	260	100,00	99,23	0,00	0,77
0,00	396	100,00	96,21	0,51	2,78
0,00	261	100,00	93,10	2,30	4,60
0,00	161	100,00	96,89	1,86	1,24
0,00	231	100,00	95,67	1,30	3,03
0,00	257	100,00	98,83	0,00	1,17
0,00	271	100,00	74,54	0,37	6,27
0,00	429	100,00	95,10	0,00	2,80
0,00	206	100,00	96,12	0,00	3,88
0,39	256	100,00	49,22	0,39	50,00
0,00	287	100,00	87,80	0,70	1,39
0,00	273	100,00	98,17	0,00	1,83
0,00	153	100,00	98,04	0,00	1,96
0,25	407	100,00	93,86	0,00	5,65
0,00	362	100,00	98,07	0,00	1,93
4,36	431	100,00	93,27	0,00	2,55
0,00	317	100,00	76,97	0,00	16,09
0,00	225	100,00	78,67	0,00	4,44

0,00	181	100,00	98,34	0,00	1,66
0,00	289	100,00	97,92	0,00	2,08
0,00	229	100,00	97,82	0,00	2,18
0,47	215	100,00	90,70	0,00	6,05
0,00	265	100,00	56,60	0,00	43,40
0,00	254	100,00	96,06	0,00	3,94
0,00	283	100,00	81,98	0,00	18,02
0,00	328	100,00	93,90	0,00	6,10
0,33	307	100,00	95,11	0,00	4,23
0,34	295	100,00	96,95	0,00	2,37
0,00	270	100,00	95,19	0,00	2,59
0,00	334	100,00	90,42	2,10	7,49
0,00	187	100,00	98,93	0,00	0,00
0,00	304	100,00	17,76	2,30	79,93
0,00	271	100,00	100,00	0,00	0,00
0,00	301	100,00	98,01	0,00	1,99
0,00	291	100,00	55,67	0,00	44,33
0,00	245	100,00	97,55	0,00	2,45
0,00	166	100,00	64,46	0,00	27,71
0,00	184	100,00	85,33	0,00	14,67
0,00	282	100,00	92,20	0,00	3,90
0,00	230	100,00	95,22	0,00	4,78
0,00	209	100,00	26,79	0,00	73,21
0,00	303	100,00	100,00	0,00	0,00
0,64	158	100,00	87,34	0,00	12,03
0,00	199	100,00	96,48	0,00	2,51
0,00	147	100,00	98,64	0,68	0,00
0,00	101	100,00	98,02	0,99	0,99
0,00	158	100,00	97,47	0,00	2,53
0,00	185	100,00	98,92	1,08	0,00
0,00	27	100,00	92,59	0,00	7,41
0,00	0	0,00	0,00	0,00	0,00
0,00	34	100,00	100,00	0,00	0,00
0,00	51	100,00	98,04	0,00	1,96
0,00	86	100,00	100,00	0,00	0,00
0,00	7	100,00	100,00	0,00	0,00
1,33	76	100,00	89,47	0,00	9,21
0,00	305	100,00	97,38	0,00	2,62
0,00	296	100,00	93,92	0,00	2,36
0,35	284	100,00	91,90	0,00	0,35
0,39	257	100,00	98,83	0,00	0,39
0,00	218	100,00	96,79	0,00	0,00
0,00	252	100,00	96,43	0,00	3,57
0,00	205	100,00	96,59	0,49	2,93
0,00	119	100,00	100,00	0,00	0,00
0,00	328	100,00	99,70	0,00	0,30
0,00	83	100,00	100,00	0,00	0,00
0,00	90	100,00	100,00	0,00	0,00
0,00	147	100,00	100,00	0,00	0,00
0,00	138	100,00	99,28	0,00	0,00

0,00	256	100,00	100,00	0,00	0,00
0,00	185	100,00	90,81	0,00	8,11
0,00	66	100,00	0,00	0,00	0,00
0,00	370	100,00	75,68	0,00	24,32
0,00	134	100,00	87,31	0,00	12,69
0,00	334	100,00	92,81	0,30	6,89
0,00	222	100,00	87,39	0,00	12,61
0,00	156	100,00	71,79	26,28	1,92
0,00	165	100,00	89,70	7,27	3,03
0,00	266	100,00	99,25	0,38	0,38
0,00	262	100,00	65,27	33,59	1,15
0,00	202	100,00	99,50	0,50	0,00
0,00	204	100,00	100,00	0,00	0,00
0,00	185	100,00	3,78	96,22	0,00
0,00	269	100,00	7,06	92,57	0,37
8,21	303	100,00	61,72	19,14	2,31
0,00	283	100,00	73,14	1,06	25,80
0,00	247	100,00	3,64	0,00	96,36
0,00	331	100,00	1,81	0,30	97,89
0,00	270	100,00	89,26	2,96	7,78
0,00	228	100,00	10,09	1,32	88,16
0,00	240	100,00	45,42	1,25	53,33
0,00	252	100,00	82,54	0,00	17,06
0,53	191	100,00	61,26	36,13	0,00
0,00	106	100,00	100,00	0,00	0,00
0,00	234	100,00	100,00	0,00	0,00
0,00	298	100,00	13,42	85,91	0,67
0,00	236	100,00	92,37	4,24	3,39
0,00	178	100,00	96,07	0,00	3,93
0,00	291	100,00	86,25	0,34	13,40
0,93	216	100,00	59,26	37,04	2,78
0,00	240	100,00	62,50	0,00	37,50
11,35	255	100,00	84,31	0,00	1,18
0,00	263	100,00	98,86	0,00	1,14
0,00	187	100,00	90,37	0,00	9,63
0,00	202	100,00	79,21	0,00	20,79
0,00	103	100,00	77,67	0,97	11,65
0,00	250	100,00	99,60	0,00	0,40
0,00	219	100,00	80,37	0,46	19,18
0,00	62	100,00	98,39	0,00	1,61
0,00	54	100,00	98,15	0,00	1,85
0,00	252	100,00	70,63	0,40	28,97
0,00	271	100,00	91,14	0,00	8,86
1,48	274	100,00	68,25	22,26	8,03
3,85	216	100,00	31,02	0,00	4,63
0,00	258	100,00	0,39	81,01	18,60
0,00	17	100,00	82,35	0,00	17,65
0,00	317	100,00	90,85	0,00	9,15
0,00	253	100,00	98,02	0,00	0,40
0,00	109	100,00	88,07	0,00	0,00

0,00	276	100,00	6,16	0,00	92,75
0,00	276	100,00	3,99	0,00	96,01
2,36	304	100,00	13,16	0,00	84,54
1,20	169	100,00	56,80	0,00	40,83
0,00	115	100,00	93,04	0,00	6,09
0,00	209	100,00	89,95	1,44	8,61
0,00	232	100,00	79,31	0,43	16,81
0,00	175	100,00	92,57	0,00	7,43
0,00	237	100,00	58,23	18,14	15,19
0,00	172	100,00	71,51	2,33	25,00
0,00	131	100,00	87,79	1,53	10,69
1,27	159	100,00	67,92	0,00	20,13
0,00	255	100,00	78,43	0,39	21,18
0,00	297	100,00	84,51	0,00	15,49
0,00	270	100,00	94,44	0,00	5,56
1,54	132	100,00	75,76	2,27	20,45
0,00	142	100,00	71,13	0,00	28,87
16,13	252	100,00	24,21	0,40	61,51
0,00	192	100,00	74,48	0,00	19,27
1,20	252	100,00	38,49	26,19	32,94
2,78	74	100,00	89,19	0,00	8,11
1,93	211	100,00	35,07	1,90	54,98
0,00	238	100,00	3,78	0,00	96,22
0,00	167	100,00	5,39	0,00	92,81
13,77	314	100,00	38,54	0,32	39,17
6,58	162	100,00	38,27	0,00	55,56
0,00	243	100,00	39,09	3,70	48,97
1,42	215	100,00	56,74	0,47	40,93
0,68	149	100,00	89,93	4,03	5,37
0,43	231	100,00	66,23	5,19	27,71
0,00	233	100,00	89,70	0,86	9,44
0,83	244	100,00	66,80	1,64	30,74
0,76	266	100,00	61,28	1,13	36,09
0,83	244	100,00	60,25	0,00	38,93
0,00	215	100,00	81,86	0,00	18,14
0,00	293	100,00	12,29	0,00	0,34
8,37	233	100,00	47,64	12,02	32,62
0,00	250	100,00	12,80	1,20	86,00
0,00	284	100,00	14,44	0,00	85,56
0,00	179	100,00	34,08	0,56	65,36
0,00	11	100,00	100,00	0,00	0,00
0,00	26	100,00	80,77	0,00	7,69
0,00	14	100,00	42,86	0,00	57,14
0,00	132	100,00	40,15	0,00	59,85
0,00	184	100,00	53,80	0,00	46,20
0,87	231	100,00	38,96	0,00	60,17
1,12	181	100,00	6,63	0,00	91,71
0,00	193	100,00	12,95	0,52	86,53
0,00	236	100,00	35,17	0,85	60,17
0,00	97	100,00	25,77	0,00	61,86

0,00	51	100,00	3,92	0,00	96,08
0,00	99	100,00	31,31	0,00	68,69
0,00	0	0,00	0,00	0,00	0,00
67,39	154	100,00	40,91	0,00	18,83
0,00	25	100,00	96,00	0,00	4,00
1,02	99	100,00	81,82	1,01	16,16
0,00	106	100,00	34,91	0,00	65,09
0,00	45	100,00	97,78	0,00	2,22
0,00	9	100,00	100,00	0,00	0,00
0,00	17	100,00	17,65	0,00	5,88
0,00	42	100,00	95,24	0,00	4,76
0,00	137	100,00	94,89	0,00	5,11
0,00	221	100,00	4,07	0,00	95,93
0,00	16	100,00	37,50	0,00	62,50
0,00	126	100,00	69,05	0,00	30,95
0,00	165	100,00	24,85	0,00	75,15
0,00	41	100,00	92,68	0,00	7,32
0,00	157	100,00	80,89	1,91	13,38
0,00	85	100,00	65,88	0,00	34,12
0,00	44	100,00	63,64	0,00	0,00
0,00	24	100,00	79,17	0,00	0,00
0,00	26	100,00	92,31	0,00	3,85
0,00	49	100,00	77,55	0,00	22,45
0,00	64	100,00	92,19	0,00	1,56
0,00	186	100,00	61,29	0,54	38,17
0,00	62	100,00	12,90	0,00	87,10
0,00	81	100,00	50,62	0,00	49,38
0,00	51	100,00	90,20	0,00	9,80
1,03	98	100,00	98,98	0,00	0,00
0,00	22	100,00	100,00	0,00	0,00
0,00	122	100,00	28,69	0,82	70,49
0,91	221	100,00	91,86	0,00	4,98
0,00	48	100,00	93,75	0,00	6,25
0,00	23	100,00	91,30	0,00	8,70
0,00	55	100,00	98,18	0,00	1,82
0,00	25	100,00	100,00	0,00	0,00
0,00	19	100,00	100,00	0,00	0,00
3,57	29	100,00	96,55	0,00	0,00
0,00	153	100,00	94,12	3,27	2,61
0,00	67	100,00	97,01	0,00	2,99
0,00	47	100,00	100,00	0,00	0,00
0,00	231	100,00	3,90	0,43	94,37
0,00	201	100,00	67,16	6,97	22,89
0,00	143	100,00	51,05	0,00	48,95
0,00	144	100,00	99,31	0,00	0,69
0,00	134	100,00	100,00	0,00	0,00
0,00	20	100,00	100,00	0,00	0,00
0,00	19	100,00	100,00	0,00	0,00
0,69	146	100,00	93,84	0,00	2,05
0,00	192	100,00	90,63	0,00	8,85

120,00	55	100,00	38,18	1,82	5,45
0,00	36	100,00	97,22	0,00	2,78
0,69	146	100,00	80,82	0,68	17,81
0,00	36	100,00	94,44	0,00	5,56
9,38	210	100,00	68,57	0,00	22,86
0,00	145	100,00	34,48	0,00	65,52
0,00	80	100,00	5,00	0,00	95,00
0,00	18	100,00	38,89	0,00	61,11
0,00	78	100,00	1,28	97,44	1,28
0,00	5	100,00	20,00	0,00	80,00
0,39	257	100,00	81,32	0,00	13,23
0,00	174	100,00	63,79	0,00	36,21
1,16	87	100,00	81,61	0,00	17,24
0,00	167	100,00	83,23	0,60	16,17
0,00	143	100,00	90,91	0,00	9,09
31,25	105	100,00	51,43	0,00	24,76
0,00	238	100,00	97,06	0,00	2,94
0,00	72	100,00	86,11	0,00	13,89
0,00	151	100,00	100,00	0,00	0,00
0,00	13	100,00	100,00	0,00	0,00
0,00	302	100,00	9,60	0,33	88,74
0,00	202	100,00	2,48	2,48	94,06
0,00	2	100,00	0,00	0,00	0,00
2,27	225	100,00	87,11	0,89	9,78
0,00	310	100,00	29,68	0,00	70,32
0,00	209	100,00	41,15	0,48	58,37
0,00	17	100,00	82,35	0,00	17,65
0,00	189	100,00	96,83	0,00	3,17
4,23	74	100,00	85,14	0,00	10,81
0,00	0	0,00	0,00	0,00	0,00
0,00	85	100,00	90,59	0,00	9,41
0,00	50	100,00	26,00	0,00	0,00
0,00	0	0,00	0,00	0,00	0,00
0,00	53	100,00	96,23	0,00	3,77
0,00	252	100,00	1,19	0,00	98,81
0,00	330	100,00	0,91	0,00	99,09
0,00	9	100,00	100,00	0,00	0,00
0,00	90	100,00	91,11	1,11	2,22
0,00	83	100,00	98,80	0,00	1,20
0,00	51	100,00	100,00	0,00	0,00
0,00	46	100,00	100,00	0,00	0,00
0,00	66	100,00	12,12	86,36	1,52
4,55	23	100,00	95,65	0,00	0,00
0,00	34	100,00	100,00	0,00	0,00
0,00	35	100,00	94,29	0,00	5,71
0,91	111	100,00	90,99	0,00	8,11
0,00	91	100,00	96,70	0,00	3,30
0,00	82	100,00	97,56	2,44	0,00
0,00	35	100,00	82,86	0,00	8,57
0,00	47	100,00	89,36	0,00	10,64

0,00	5	100,00	100,00	0,00	0,00
0,00	53	100,00	50,94	0,00	1,89
0,00	150	100,00	6,00	5,33	88,67
0,00	30	100,00	96,67	0,00	3,33
0,00	36	100,00	94,44	0,00	5,56
0,00	90	100,00	98,89	0,00	1,11
0,00	102	100,00	93,14	0,00	6,86
0,00	115	100,00	79,13	6,96	13,91
0,00	118	100,00	93,22	4,24	2,54
0,00	202	100,00	97,03	0,00	2,97
0,00	23	100,00	95,65	0,00	4,35
0,00	12	100,00	25,00	58,33	16,67
0,00	50	100,00	50,00	0,00	10,00
0,00	26	100,00	50,00	0,00	3,85
0,00	89	100,00	95,51	0,00	3,37
0,00	64	100,00	100,00	0,00	0,00
0,00	19	100,00	100,00	0,00	0,00
0,00	66	100,00	92,42	0,00	7,58
0,00	48	100,00	95,83	0,00	4,17
0,00	14	100,00	64,29	0,00	0,00
0,00	103	100,00	91,26	0,00	8,74
0,00	56	100,00	96,43	0,00	3,57
0,00	4	100,00	0,00	0,00	0,00
0,00	45	100,00	71,11	0,00	28,89
0,00	141	100,00	90,07	0,00	9,93
0,00	49	100,00	95,92	0,00	4,08
0,00	107	100,00	95,33	0,00	4,67
0,00	110	100,00	96,36	0,00	3,64
0,00	60	100,00	95,00	0,00	3,33
0,00	8	100,00	50,00	0,00	50,00
0,00	66	100,00	100,00	0,00	0,00
0,00	40	100,00	97,50	0,00	2,50
0,00	0	0,00	0,00	0,00	0,00
0,00	50	100,00	78,00	0,00	22,00
0,00	17	100,00	100,00	0,00	0,00
0,00	33	100,00	100,00	0,00	0,00
0,00	12	100,00	50,00	0,00	50,00
0,00	95	100,00	2,11	0,00	97,89
0,00	51	100,00	31,37	7,84	58,82
0,00	152	100,00	53,29	0,00	46,71
0,56	178	100,00	34,83	5,62	58,43
12,26	174	100,00	28,74	0,00	60,34
0,00	144	100,00	6,25	1,39	92,36
0,00	15	100,00	100,00	0,00	0,00
0,00	25	100,00	100,00	0,00	0,00
0,00	306	100,00	60,78	0,00	36,27
0,00	229	100,00	38,43	0,87	58,52
0,00	76	100,00	100,00	0,00	0,00
2,49	371	100,00	96,50	0,00	0,27
1,34	302	100,00	73,51	0,66	24,50

7,74	167	100,00	0,00	0,00	0,00
13,79	198	100,00	82,83	0,00	5,05
0,00	204	100,00	96,08	0,00	3,92
0,00	183	100,00	61,20	0,00	3,83
0,00	316	100,00	97,47	0,00	2,53
0,00	259	100,00	98,84	0,39	0,77
0,00	262	100,00	96,56	0,76	2,67
0,00	241	100,00	94,61	0,00	5,39
0,00	285	100,00	94,39	0,00	5,61
0,00	122	100,00	93,44	0,00	6,56
0,00	420	100,00	41,19	0,48	56,90
0,00	222	100,00	38,74	2,70	54,50
0,26	386	100,00	57,51	0,26	41,97
0,00	228	100,00	96,05	0,44	3,51
0,00	297	100,00	89,90	0,34	9,76
0,00	70	100,00	64,29	0,00	35,71
0,00	252	100,00	64,68	6,75	28,57
0,26	383	100,00	96,87	0,26	2,61
0,00	362	100,00	95,58	0,00	4,42
0,29	351	100,00	12,82	3,99	78,06
0,00	267	100,00	86,14	0,00	8,61
9,89	311	100,00	71,06	0,64	18,65
0,00	236	100,00	93,22	0,00	6,78
0,00	310	100,00	83,23	0,00	9,03
0,00	364	100,00	95,33	0,00	4,40
0,48	417	100,00	98,80	0,24	0,48
0,00	230	100,00	97,39	0,00	1,30
0,33	305	100,00	93,44	0,33	5,90
0,00	261	100,00	99,62	0,00	0,38
0,00	306	100,00	91,50	0,00	3,27
0,00	240	100,00	95,00	0,00	3,75
0,00	210	100,00	95,71	0,00	4,29
0,00	127	100,00	92,91	0,00	7,09
13,77	281	100,00	73,31	0,36	14,23
0,00	313	100,00	51,76	0,00	48,24
0,00	196	100,00	74,49	0,00	25,51
0,00	231	100,00	64,07	0,00	35,93
0,00	109	100,00	96,33	0,00	2,75
0,00	176	100,00	98,30	0,00	1,70
0,00	44	100,00	100,00	0,00	0,00
0,00	178	100,00	0,00	99,44	0,56
0,00	139	100,00	97,12	0,72	2,16
0,00	84	100,00	97,62	0,00	2,38
0,00	333	100,00	98,20	0,00	1,80
0,00	94	100,00	3,19	89,36	3,19
0,00	310	100,00	73,23	23,87	2,90
0,00	178	100,00	93,26	0,00	6,74
0,00	361	100,00	57,89	0,28	41,83
0,00	146	100,00	4,11	0,00	95,89
0,52	192	100,00	2,08	0,00	97,40

0,00	257	100,00	0,39	0,00	99,61
0,00	228	100,00	0,88	1,75	97,37
0,00	179	100,00	62,57	6,15	31,28
0,00	331	100,00	2,11	0,30	97,58
0,00	247	100,00	3,24	0,00	96,76
0,00	209	100,00	2,39	0,00	97,61
0,00	189	100,00	1,59	0,00	98,41
0,00	494	100,00	74,09	0,61	22,06
0,00	213	100,00	98,59	0,00	0,94
0,00	210	100,00	64,29	0,48	4,76
0,30	337	100,00	26,41	0,30	64,09
1,47	277	100,00	98,19	0,00	0,00
0,96	211	100,00	91,94	0,00	4,74
0,00	207	100,00	94,20	0,00	5,80
0,00	184	100,00	92,93	1,09	4,89
0,00	338	100,00	97,93	0,00	1,48
0,00	350	100,00	99,14	0,00	0,86
0,00	220	100,00	97,27	0,00	2,73
0,00	312	100,00	99,36	0,00	0,64
0,00	317	100,00	32,49	0,00	7,26
0,00	156	100,00	95,51	0,64	3,85
0,00	299	100,00	97,99	0,33	1,67
0,00	331	100,00	12,69	86,40	0,60
6,07	262	100,00	87,40	0,38	6,49
6,32	101	100,00	76,24	0,00	17,82
24,12	211	100,00	18,01	0,00	61,61
0,00	243	100,00	76,13	0,00	23,87
0,00	117	100,00	61,54	0,00	38,46
0,00	209	100,00	65,07	0,00	29,67
0,00	278	100,00	41,01	0,00	58,99
0,39	258	100,00	86,43	0,00	13,18
0,00	292	100,00	32,88	0,00	67,12
0,00	102	100,00	40,20	0,00	59,80
0,00	195	100,00	22,05	2,05	75,90
0,00	315	100,00	28,25	19,05	50,48
0,00	197	100,00	38,07	25,38	30,46
3,26	190	100,00	55,26	12,63	5,26
0,00	118	100,00	94,07	0,00	5,93
0,00	184	100,00	78,26	9,78	11,96
0,00	340	100,00	3,24	0,88	95,88
0,00	264	100,00	1,14	0,00	98,86
0,00	371	100,00	13,75	0,54	85,71
0,00	199	100,00	3,52	0,50	95,98
0,00	230	100,00	26,52	1,30	72,17
0,72	139	100,00	0,00	0,72	98,56
0,00	218	100,00	0,00	0,46	99,54
0,00	301	100,00	0,33	0,00	99,67
0,00	206	100,00	0,97	0,00	99,03
0,00	362	100,00	1,66	0,00	97,79
0,00	182	100,00	8,79	0,00	91,21

0,35	288	100,00	4,51	24,65	70,49
0,00	357	100,00	61,90	0,28	11,48
0,00	217	100,00	90,78	0,46	0,46
0,00	309	100,00	68,28	0,00	17,80
0,00	247	100,00	76,92	0,00	12,96
0,00	206	100,00	92,72	0,00	7,28
0,43	231	100,00	92,21	0,00	7,36
0,00	174	100,00	68,39	2,30	12,07
0,00	379	100,00	64,38	0,00	35,62
0,00	216	100,00	99,07	0,00	0,93
0,00	169	100,00	96,45	0,00	3,55
0,00	304	100,00	97,04	0,66	2,30
0,00	323	100,00	97,21	0,00	2,79
0,00	319	100,00	98,75	0,00	1,25
0,00	142	100,00	98,59	0,00	1,41
0,00	200	100,00	98,00	0,00	2,00
0,00	169	100,00	0,59	99,41	0,00
0,00	103	100,00	96,12	0,00	3,88
0,00	13	100,00	100,00	0,00	0,00
0,00	124	100,00	0,81	96,77	2,42
0,00	98	100,00	97,96	0,00	2,04
0,00	76	100,00	92,11	0,00	1,32
0,00	224	100,00	19,20	78,57	2,23
0,00	233	100,00	99,57	0,00	0,43
0,00	77	100,00	96,10	0,00	3,90
0,00	233	100,00	99,57	0,43	0,00
0,00	155	100,00	4,52	95,48	0,00
0,00	146	100,00	99,32	0,68	0,00
0,00	128	100,00	0,78	99,22	0,00
0,00	142	100,00	2,82	96,48	0,00
0,00	275	100,00	2,18	97,82	0,00
0,00	64	100,00	100,00	0,00	0,00
0,00	102	100,00	98,04	0,00	1,96
0,00	106	100,00	100,00	0,00	0,00
0,00	161	100,00	96,89	0,00	3,11
0,00	165	100,00	92,12	4,85	0,00
0,00	139	100,00	100,00	0,00	0,00
0,00	233	100,00	96,14	0,00	3,86
0,00	102	100,00	97,06	0,00	2,94
0,00	320	100,00	91,56	0,00	3,75
0,00	132	100,00	98,48	0,00	1,52
0,00	187	100,00	10,16	0,53	89,30
0,00	353	100,00	26,91	2,27	70,82
0,00	150	100,00	4,00	1,33	94,67
25,86	73	100,00	71,23	0,00	8,22
0,00	268	100,00	0,37	1,49	98,13
0,00	311	100,00	0,00	0,00	100,00
0,00	264	100,00	0,00	1,89	98,11
0,54	185	100,00	95,68	0,00	3,78
0,00	254	100,00	34,65	1,57	63,78

0,42	238	100,00	21,01	0,00	78,57
0,00	199	100,00	98,49	0,50	0,00
0,00	432	100,00	25,69	0,23	74,07
0,00	211	100,00	26,54	0,47	72,99
0,00	213	100,00	41,78	0,00	58,22
0,00	295	100,00	11,19	0,68	88,14
0,00	120	100,00	38,33	0,00	61,67
0,00	174	100,00	19,54	0,00	80,46
0,00	139	100,00	56,12	2,16	41,73
2,56	160	100,00	20,63	0,00	76,88
0,00	226	100,00	34,51	32,74	26,55
0,00	240	100,00	97,92	1,67	0,00
0,00	240	100,00	65,83	10,00	23,75
0,00	133	100,00	5,26	94,74	0,00
0,00	240	100,00	97,50	0,42	2,08
0,00	111	100,00	5,41	92,79	1,80
0,00	198	100,00	48,99	0,00	51,01
0,00	156	100,00	26,28	0,00	73,72
0,00	80	100,00	2,50	0,00	97,50
123,75	179	100,00	0,00	0,00	0,00
0,00	646	100,00	9,75	2,48	87,62
0,35	575	100,00	10,78	0,17	88,70
0,00	470	100,00	9,79	0,21	90,00
0,00	309	100,00	4,21	0,00	95,79
0,00	415	100,00	9,16	0,00	90,84
0,00	337	100,00	9,50	0,30	89,61
0,19	541	100,00	1,85	1,11	96,86
1,28	238	100,00	0,84	0,42	97,48
0,33	305	100,00	8,20	0,00	91,48
0,00	283	100,00	99,29	0,35	0,35
0,00	79	100,00	7,59	29,11	63,29
0,00	280	100,00	68,21	0,36	31,43
8,00	135	100,00	92,59	0,00	0,00
1,59	320	100,00	58,75	1,25	38,44
0,00	452	100,00	99,34	0,66	0,00
0,00	240	100,00	8,75	0,83	90,42
0,00	206	100,00	58,25	0,00	39,32
0,00	3	100,00	0,00	0,00	0,00
0,33	304	100,00	98,03	0,66	0,99
0,00	168	100,00	97,62	0,00	2,38
0,00	329	100,00	70,21	29,79	0,00
0,00	129	100,00	96,90	0,00	3,10
1,00	202	100,00	95,05	0,00	3,96
0,00	173	100,00	46,82	0,00	53,18
0,00	234	100,00	96,15	0,00	3,42
0,79	128	100,00	73,44	0,00	21,88
0,00	272	100,00	33,82	5,15	59,19
0,00	246	100,00	69,92	0,41	29,67
5,72	314	100,00	35,99	0,00	58,60
0,00	214	100,00	43,46	0,00	56,54

0,66	303	100,00	42,57	0,00	56,44
5,56	304	100,00	14,80	0,00	79,93
0,00	348	100,00	11,78	0,00	88,22
0,00	237	100,00	3,80	0,00	96,20
0,00	282	100,00	41,13	0,00	58,87
0,00	298	100,00	12,75	0,00	87,25
0,00	419	100,00	42,00	1,43	56,56
0,00	173	100,00	24,86	0,00	56,07
0,00	14	100,00	57,14	0,00	42,86
0,00	59	100,00	96,61	1,69	1,69
0,00	184	100,00	100,00	0,00	0,00
0,47	429	100,00	98,60	0,00	0,93
0,00	93	100,00	100,00	0,00	0,00
12,23	312	100,00	54,17	0,00	34,62
0,00	30	100,00	100,00	0,00	0,00
0,00	154	100,00	100,00	0,00	0,00
0,00	92	100,00	100,00	0,00	0,00
0,76	264	100,00	99,24	0,00	0,00
0,00	42	100,00	16,67	9,52	0,00
2,16	236	100,00	22,88	8,47	66,53
2,25	273	100,00	30,77	0,00	64,84
0,00	219	100,00	48,40	0,00	51,60
2,82	292	100,00	45,21	0,68	51,37
0,00	347	100,00	46,11	0,00	53,89
0,00	293	100,00	26,96	0,00	71,67
0,00	305	100,00	11,80	0,00	88,20
0,00	367	100,00	26,16	0,27	73,57
0,00	70	100,00	34,29	0,00	65,71
0,00	259	100,00	49,03	0,00	50,19
0,00	211	100,00	54,98	0,00	45,02
0,00	331	100,00	17,52	0,00	82,48
0,00	345	100,00	5,22	0,00	94,78
0,00	316	100,00	46,20	0,32	53,48
0,00	155	100,00	69,68	30,32	0,00
0,00	108	100,00	72,22	23,15	4,63
1,23	82	100,00	6,10	92,68	0,00
0,00	95	100,00	100,00	0,00	0,00
0,00	103	100,00	100,00	0,00	0,00
0,00	225	100,00	32,89	0,44	66,67
11,88	113	100,00	19,47	0,00	69,91
0,78	259	100,00	23,94	0,00	75,29
0,00	338	100,00	18,05	0,59	81,07
2,24	365	100,00	12,33	1,64	83,84
0,00	302	100,00	12,91	0,00	87,09
0,00	367	100,00	26,43	0,00	73,57
0,36	276	100,00	26,45	2,90	70,29
0,00	254	100,00	31,50	0,39	68,11
0,84	360	100,00	20,28	0,28	78,33
0,00	244	100,00	20,90	0,41	78,69
0,00	266	100,00	12,78	0,00	87,22

0,00	256	100,00	10,16	0,00	89,84
0,77	261	100,00	16,48	0,00	82,76
7,87	274	100,00	13,14	0,00	79,56
0,00	325	100,00	26,77	0,31	72,92
0,00	303	100,00	20,46	6,93	72,61
0,00	81	100,00	100,00	0,00	0,00
0,00	178	100,00	8,43	90,45	0,00
0,81	124	100,00	87,10	10,48	1,61
0,00	148	100,00	91,89	0,00	0,00
0,00	212	100,00	3,77	96,23	0,00
0,00	100	100,00	95,00	0,00	2,00
0,00	171	100,00	100,00	0,00	0,00
0,00	237	100,00	24,05	0,00	75,95
0,00	225	100,00	53,33	0,00	46,67
3,32	373	100,00	36,19	0,27	60,05
12,50	108	100,00	21,30	0,00	67,59
1,57	258	100,00	28,68	0,39	69,38
0,33	301	100,00	36,54	0,00	63,12
0,00	157	100,00	19,11	0,00	80,89
0,00	226	100,00	11,50	0,00	88,50
9,35	304	100,00	6,91	0,00	84,54
0,56	360	100,00	1,67	0,00	95,56
0,00	180	100,00	31,67	0,00	68,33
0,86	351	100,00	46,44	0,28	52,14
0,00	215	100,00	46,51	0,47	53,02
0,00	262	100,00	37,40	0,38	62,21
3,26	285	100,00	24,21	0,00	72,28
0,00	193	100,00	9,33	0,00	90,67
0,00	290	100,00	25,17	2,41	72,41
0,00	313	100,00	5,11	1,60	93,29
0,00	240	100,00	4,58	0,00	91,67
0,00	204	100,00	3,43	0,00	95,59
0,00	284	100,00	1,76	0,00	97,18
0,00	259	100,00	4,63	0,00	94,59
0,00	90	100,00	6,67	0,00	93,33
0,00	33	100,00	100,00	0,00	0,00
0,00	149	100,00	11,41	0,00	87,92
0,00	389	100,00	7,20	0,51	92,29
0,00	136	100,00	100,00	0,00	0,00
0,00	126	100,00	80,16	19,05	0,00
0,64	157	100,00	38,22	0,00	59,24
3,18	162	100,00	24,07	1,23	71,60
0,38	266	100,00	33,46	0,00	66,17
0,00	213	100,00	22,54	0,00	72,77
2,69	343	100,00	36,73	0,00	60,35
0,32	311	100,00	27,01	0,64	72,03
0,00	251	100,00	24,30	1,59	74,10
0,88	228	100,00	30,26	0,00	68,86
5,74	221	100,00	12,67	0,00	81,90
0,00	195	100,00	7,69	0,00	92,31

0,00	243	100,00	10,29	0,00	89,71
2,58	239	100,00	8,79	0,00	88,28
0,00	251	100,00	8,76	0,00	91,24
0,00	284	100,00	3,87	0,00	96,13
0,00	280	100,00	14,64	0,00	85,36
0,00	410	100,00	5,12	0,00	94,88
0,00	335	100,00	7,16	0,00	92,84
0,00	336	100,00	4,76	0,00	95,24
0,00	356	100,00	11,52	0,00	88,48
0,00	253	100,00	9,49	0,00	90,51
0,37	269	100,00	5,95	0,00	93,68
0,00	355	100,00	3,66	0,00	96,34
0,00	281	100,00	2,85	0,00	97,15
0,00	217	100,00	25,81	0,46	73,73
0,00	43	100,00	100,00	0,00	0,00
0,00	157	100,00	32,48	0,64	66,88
0,00	55	100,00	100,00	0,00	0,00
0,00	102	100,00	100,00	0,00	0,00
0,00	161	100,00	98,14	0,00	1,86
1,20	84	100,00	97,62	0,00	1,19
0,00	88	100,00	100,00	0,00	0,00
0,00	24	100,00	79,17	20,83	0,00
0,00	65	100,00	100,00	0,00	0,00
0,00	92	100,00	100,00	0,00	0,00
0,00	100	100,00	12,00	0,00	87,00
0,00	22	100,00	100,00	0,00	0,00
0,00	4	100,00	0,00	0,00	0,00
0,00	291	100,00	72,51	0,34	27,15
0,00	87	100,00	100,00	0,00	0,00
0,00	385	100,00	92,47	0,00	7,01
0,00	323	100,00	67,18	2,48	26,63
0,00	80	100,00	100,00	0,00	0,00
0,00	161	100,00	96,27	0,00	0,00
0,00	49	100,00	97,96	0,00	2,04
0,00	144	100,00	68,75	0,69	30,56
0,00	66	100,00	100,00	0,00	0,00
0,00	27	100,00	100,00	0,00	0,00
0,00	219	100,00	96,80	0,00	2,74
0,00	270	100,00	80,37	0,00	18,15
0,00	155	100,00	100,00	0,00	0,00
0,00	21	100,00	100,00	0,00	0,00
0,00	12	100,00	100,00	0,00	0,00
0,00	21	100,00	100,00	0,00	0,00
0,00	138	100,00	1,45	0,00	98,55
0,00	150	100,00	92,00	0,00	8,00
0,00	57	100,00	98,25	0,00	1,75
0,00	91	100,00	79,12	2,20	5,49
0,00	170	100,00	97,65	0,00	2,35
0,00	210	100,00	97,14	0,00	2,86
0,00	461	100,00	3,25	0,43	96,31

0,00	324	100,00	31,48	0,31	66,67
0,00	25	100,00	24,00	0,00	76,00
0,00	14	100,00	100,00	0,00	0,00
0,00	95	100,00	27,37	0,00	72,63
0,00	108	100,00	46,30	0,93	51,85
0,98	103	100,00	7,77	0,00	91,26
0,00	10	100,00	10,00	0,00	90,00
0,00	146	100,00	100,00	0,00	0,00
0,00	23	100,00	100,00	0,00	0,00
0,00	56	100,00	10,71	0,00	89,29
0,00	83	100,00	100,00	0,00	0,00
0,00	235	100,00	51,49	0,00	48,51
2,86	36	100,00	97,22	0,00	0,00
0,00	27	100,00	100,00	0,00	0,00
0,00	136	100,00	71,32	0,00	5,15
7,80	221	100,00	34,39	2,26	56,11
0,00	163	100,00	55,83	0,00	44,17
0,00	49	100,00	100,00	0,00	0,00
0,00	150	100,00	37,33	0,00	62,67
0,00	238	100,00	67,65	0,00	32,35
0,00	45	100,00	100,00	0,00	0,00
0,00	10	100,00	100,00	0,00	0,00
0,00	70	100,00	94,29	5,71	0,00
0,00	35	100,00	42,86	0,00	57,14
0,00	3	100,00	0,00	0,00	0,00
0,00	33	100,00	93,94	6,06	0,00
0,00	139	100,00	34,53	4,32	51,08
0,00	15	100,00	100,00	0,00	0,00
0,00	12	100,00	100,00	0,00	0,00
0,00	123	100,00	100,00	0,00	0,00
0,00	264	100,00	98,11	1,14	0,76
1,30	78	100,00	96,15	0,00	2,56
0,00	5	100,00	20,00	0,00	80,00
0,00	239	100,00	5,86	0,00	94,14
0,00	246	100,00	3,66	0,00	96,34
1,82	279	100,00	5,73	1,08	91,40
0,00	338	100,00	7,69	0,00	92,31
0,68	148	100,00	1,35	0,00	97,97
0,00	362	100,00	2,21	0,55	97,24
0,00	204	100,00	2,45	0,00	97,55
2,24	228	100,00	2,63	0,44	92,54
0,00	236	100,00	0,00	0,00	96,19
0,00	272	100,00	0,37	0,37	95,59
0,00	176	100,00	0,57	0,00	95,45
0,00	417	100,00	11,51	0,00	88,49
0,00	218	100,00	8,26	1,38	90,37
2,00	204	100,00	50,00	19,12	27,94
0,00	64	100,00	84,38	0,00	0,00
0,00	94	100,00	96,81	0,00	1,06
0,00	152	100,00	84,21	0,66	15,13

0,00	200	100,00	100,00	0,00	0,00
0,00	3	100,00	0,00	0,00	0,00
0,00	41	100,00	100,00	0,00	0,00
0,00	54	100,00	0,00	0,00	0,00
0,00	119	100,00	100,00	0,00	0,00
0,00	44	100,00	100,00	0,00	0,00
0,00	47	100,00	2,13	97,87	0,00
0,00	12	100,00	91,67	8,33	0,00
0,00	129	100,00	98,45	0,00	1,55
0,00	29	100,00	100,00	0,00	0,00
0,00	164	100,00	100,00	0,00	0,00
0,00	22	100,00	100,00	0,00	0,00
0,00	82	100,00	100,00	0,00	0,00
0,00	174	100,00	95,98	0,00	2,30
0,00	80	100,00	86,25	5,00	8,75
0,00	34	100,00	88,24	0,00	11,76
0,00	229	100,00	72,49	0,00	18,34
0,00	0	0,00	0,00	0,00	0,00
0,00	23	100,00	91,30	0,00	8,70
0,00	78	100,00	41,03	0,00	58,97
0,00	73	100,00	38,36	0,00	61,64
0,00	63	100,00	88,89	0,00	11,11
0,00	95	100,00	8,42	0,00	91,58
0,00	90	100,00	54,44	1,11	44,44
24,49	61	100,00	59,02	3,28	18,03
0,00	108	100,00	14,81	0,93	84,26
0,00	196	100,00	28,57	1,53	69,90
0,00	235	100,00	30,21	0,43	69,36
0,00	58	100,00	13,79	86,21	0,00
0,00	61	100,00	75,41	24,59	0,00
0,00	173	100,00	27,75	1,16	71,10
0,00	33	100,00	100,00	0,00	0,00
0,00	9	100,00	0,00	0,00	0,00
0,00	4	100,00	0,00	0,00	0,00
0,00	11	100,00	100,00	0,00	0,00
0,00	216	100,00	100,00	0,00	0,00
0,00	31	100,00	0,00	0,00	0,00
0,42	240	100,00	99,58	0,00	0,00
0,00	15	100,00	100,00	0,00	0,00
0,00	93	100,00	34,41	1,08	64,52
0,00	535	100,00	3,36	2,06	94,58
0,00	94	100,00	88,30	0,00	11,70
0,00	24	100,00	0,00	0,00	0,00
0,00	1	100,00	0,00	0,00	0,00
0,00	67	100,00	62,69	0,00	37,31
0,00	279	100,00	95,34	0,00	1,79
0,00	191	100,00	97,38	1,57	0,00
0,00	145	100,00	99,31	0,69	0,00
0,00	57	100,00	100,00	0,00	0,00
0,00	71	100,00	52,11	38,03	9,86

0,00	74	100,00	100,00	0,00	0,00
0,00	29	100,00	100,00	0,00	0,00
0,00	300	100,00	20,33	5,00	74,33
0,00	255	100,00	100,00	0,00	0,00
0,00	14	100,00	71,43	0,00	28,57
0,00	65	100,00	100,00	0,00	0,00
0,00	11	100,00	100,00	0,00	0,00
0,00	21	100,00	100,00	0,00	0,00
0,00	84	100,00	96,43	0,00	3,57
0,00	64	100,00	98,44	0,00	1,56
0,00	32	100,00	0,00	0,00	0,00
0,00	3	100,00	0,00	0,00	0,00
0,00	93	100,00	26,88	9,68	63,44
0,00	2	100,00	0,00	0,00	0,00
0,00	75	100,00	44,00	0,00	56,00
0,00	92	100,00	9,78	0,00	90,22
0,00	45	100,00	100,00	0,00	0,00
0,00	22	100,00	100,00	0,00	0,00
0,00	63	100,00	90,48	0,00	9,52
0,00	77	100,00	100,00	0,00	0,00
0,00	146	100,00	73,29	26,71	0,00
0,00	96	100,00	82,29	0,00	0,00
0,00	33	100,00	100,00	0,00	0,00
0,00	7	100,00	100,00	0,00	0,00
0,00	0	0,00	0,00	0,00	0,00
3,13	99	100,00	5,05	0,00	91,92
11,11	10	100,00	60,00	0,00	30,00
0,00	68	100,00	100,00	0,00	0,00
0,00	225	100,00	19,11	11,56	69,33
0,00	193	100,00	98,96	1,04	0,00
4,56	298	100,00	24,83	0,00	70,81
0,00	172	100,00	38,37	0,00	61,63
0,00	275	100,00	36,36	14,18	49,45
0,00	312	100,00	46,79	0,00	53,21
2,18	281	100,00	63,70	0,71	33,45
8,21	145	100,00	91,72	0,69	0,00
3,70	280	100,00	88,21	0,00	8,21
0,00	137	100,00	43,80	0,00	56,20
0,00	0	0,00	0,00	0,00	0,00
0,00	243	100,00	73,66	0,00	26,34
0,00	132	100,00	100,00	0,00	0,00
0,00	126	100,00	98,41	0,00	0,00
0,50	399	100,00	93,73	0,00	0,00
0,00	273	100,00	2,56	0,37	95,60
0,00	0	0,00	0,00	0,00	0,00
0,00	232	100,00	11,64	0,00	87,07
0,00	227	100,00	29,52	0,00	66,96
0,00	239	100,00	100,00	0,00	0,00
0,31	324	100,00	97,22	0,00	1,54
0,00	244	100,00	77,46	0,00	21,72

0,00	198	100,00	88,38	0,00	8,59
0,41	242	100,00	88,02	0,00	11,57
0,00	33	100,00	100,00	0,00	0,00
30,79	514	100,00	76,07	0,00	0,00
9,24	343	100,00	54,23	0,00	37,32
39,91	326	100,00	50,31	0,00	21,17
0,00	230	100,00	13,48	19,13	67,39
0,00	166	100,00	100,00	0,00	0,00
0,49	207	100,00	7,73	0,00	90,82
0,00	241	100,00	36,51	0,41	61,83
1,54	66	100,00	96,97	0,00	1,52
0,00	220	100,00	54,55	0,00	45,45
0,00	216	100,00	38,89	0,93	60,19
0,00	215	100,00	32,09	46,51	21,40
0,00	443	100,00	1,13	6,32	92,55
0,00	185	100,00	51,89	4,86	43,24
0,00	21	100,00	23,81	0,00	76,19
0,00	222	100,00	68,92	1,35	29,73
5,41	312	100,00	43,59	11,54	39,74
0,00	168	100,00	64,29	2,38	29,17
0,00	175	100,00	65,14	26,86	8,00
0,00	236	100,00	5,93	0,85	93,22
0,00	222	100,00	37,39	15,32	47,30
0,00	298	100,00	43,96	0,00	55,70
0,00	158	100,00	91,14	0,00	8,86
0,00	149	100,00	97,32	0,00	2,01
0,00	188	100,00	89,36	0,53	10,11
0,00	0	0,00	0,00	0,00	0,00
0,00	114	100,00	10,53	0,00	89,47
0,00	271	100,00	12,55	1,48	85,98
0,00	197	100,00	4,06	0,00	95,94
0,00	238	100,00	11,34	0,42	87,82
0,00	148	100,00	5,41	0,00	92,57
0,00	211	100,00	76,30	0,00	23,70
0,00	253	100,00	69,96	0,00	30,04
0,00	283	100,00	31,10	0,35	67,84
0,00	239	100,00	22,59	3,77	73,64
0,00	208	100,00	0,48	0,00	99,52
0,36	278	100,00	70,86	17,99	10,79
0,00	282	100,00	53,55	41,13	5,32
0,41	245	100,00	99,59	0,00	0,00
0,00	224	100,00	99,11	0,00	0,89
0,00	252	100,00	99,60	0,40	0,00
0,00	272	100,00	5,15	0,00	94,12
0,00	309	100,00	0,32	0,97	98,71
0,00	51	100,00	74,51	0,00	25,49
0,00	244	100,00	100,00	0,00	0,00
0,00	273	100,00	0,00	0,00	100,00
0,00	168	100,00	98,81	0,00	1,19
0,00	209	100,00	49,28	33,49	15,79

0,00	128	100,00	32,03	0,00	67,97
0,00	224	100,00	96,88	0,00	2,68
0,00	383	100,00	85,64	10,44	2,35
0,64	314	100,00	98,09	0,00	1,27
0,00	490	100,00	71,22	0,00	28,78
0,00	391	100,00	95,91	1,28	0,00
0,00	112	100,00	0,00	0,00	0,00
2,90	248	100,00	91,53	0,81	4,03
3,90	240	100,00	59,17	0,00	36,67
0,19	535	100,00	97,38	0,00	2,43
19,68	225	100,00	80,44	0,00	3,11
21,05	207	100,00	82,61	0,00	0,00
0,00	84	100,00	97,62	0,00	0,00
0,00	203	100,00	89,16	0,99	6,40
14,67	211	100,00	86,26	0,00	0,95
21,67	146	100,00	82,19	0,00	0,00
0,00	297	100,00	96,63	0,34	1,68
0,00	19	100,00	100,00	0,00	0,00
0,00	217	100,00	17,51	69,12	13,36
0,00	168	100,00	100,00	0,00	0,00
4,49	186	100,00	59,68	0,00	36,02
0,00	209	100,00	61,72	11,00	27,27
0,00	181	100,00	36,46	12,71	50,83
0,00	132	100,00	30,30	6,06	63,64
0,00	270	100,00	44,81	0,00	55,19
0,00	269	100,00	40,89	15,99	40,15
0,45	223	100,00	38,57	0,90	60,09
0,00	210	100,00	32,86	0,48	66,67
0,00	221	100,00	41,18	0,00	58,82
0,00	182	100,00	96,15	0,00	2,75
0,00	167	100,00	69,46	26,35	3,59
0,00	252	100,00	98,81	0,00	1,19
0,00	0	0,00	0,00	0,00	0,00
0,00	306	100,00	39,22	0,00	60,78
0,00	56	100,00	100,00	0,00	0,00
0,00	257	100,00	93,77	0,78	5,45
0,47	213	100,00	50,70	12,21	36,62
0,00	288	100,00	98,26	0,00	1,74
10,09	120	100,00	7,50	0,00	83,33
0,00	318	100,00	2,83	96,86	0,31
0,00	74	100,00	89,19	2,70	8,11
0,00	193	100,00	9,33	90,67	0,00
0,00	29	100,00	6,90	93,10	0,00
0,00	76	100,00	76,32	14,47	9,21
1,10	183	100,00	87,98	0,00	10,93
18,96	320	100,00	57,19	0,00	26,88
0,00	336	100,00	100,00	0,00	0,00
0,00	5	100,00	100,00	0,00	0,00
0,00	92	100,00	30,43	1,09	68,48
0,00	170	100,00	43,53	0,00	56,47

0,00	210	100,00	22,86	0,00	77,14
210,53	59	100,00	32,20	0,00	0,00
0,00	250	100,00	64,80	0,80	32,80
0,00	138	100,00	100,00	0,00	0,00
0,00	235	100,00	28,09	71,91	0,00
0,00	79	100,00	100,00	0,00	0,00
0,47	212	100,00	12,26	0,00	84,43
1,49	204	100,00	11,27	0,00	87,25
0,00	143	100,00	53,85	0,70	45,45
0,00	186	100,00	17,74	0,00	82,26
0,00	214	100,00	51,87	0,00	48,13
0,00	168	100,00	41,07	0,00	58,93
0,00	224	100,00	16,07	31,70	52,23
3,39	183	100,00	44,26	2,19	50,27
0,00	154	100,00	50,65	7,79	41,56
0,00	204	100,00	35,29	3,43	61,27
0,00	171	100,00	30,41	4,68	64,91
0,00	133	100,00	20,30	3,76	75,94
0,00	230	100,00	46,96	2,61	50,43
0,00	206	100,00	31,07	0,97	67,96
7,35	219	100,00	41,55	2,74	48,86
0,00	276	100,00	9,06	3,26	87,68
0,43	232	100,00	21,98	3,02	74,14
0,40	251	100,00	38,65	7,57	53,39
0,00	233	100,00	36,48	1,29	62,23
0,00	145	100,00	31,03	0,00	68,97
0,00	129	100,00	3,10	0,00	96,90
7,69	126	100,00	7,14	0,00	85,71
0,00	351	100,00	76,35	2,85	20,80
0,00	98	100,00	48,98	3,06	47,96
0,00	2	100,00	0,00	0,00	0,00
0,27	368	100,00	42,66	0,54	56,52
0,00	196	0,00	0,00	0,00	0,00
0,00	154	100,00	30,52	1,30	68,18
0,00	44	100,00	18,18	2,27	79,55
0,00	119	100,00	64,71	29,41	5,88
0,00	198	100,00	27,27	0,00	72,73
0,00	129	100,00	3,10	0,00	96,90
0,00	229	100,00	7,42	0,44	92,14
0,00	31	100,00	32,26	0,00	67,74
0,00	174	100,00	4,60	0,00	95,40
0,00	250	100,00	9,60	0,00	90,40
0,00	133	100,00	36,09	0,00	63,91
0,00	170	100,00	63,53	0,59	35,88
0,00	80	100,00	96,25	0,00	3,75
0,00	107	100,00	100,00	0,00	0,00
0,00	25	100,00	20,00	0,00	80,00
0,00	342	100,00	14,62	0,29	85,09
793,75	286	100,00	5,24	0,00	5,94
2,26	272	100,00	41,91	5,88	50,00

0,00	101	100,00	81,19	0,00	18,81
0,00	214	100,00	55,14	5,61	39,25
0,00	217	100,00	74,19	0,00	25,81
0,00	67	100,00	68,66	1,49	29,85
0,00	216	100,00	42,59	3,24	54,17
0,00	112	100,00	54,46	0,00	45,54
0,00	111	100,00	100,00	0,00	0,00
0,00	279	100,00	2,51	0,00	97,49
0,00	211	100,00	21,33	1,90	76,30
7,59	170	100,00	6,47	0,59	85,88
0,00	2	100,00	0,00	0,00	0,00
0,00	214	100,00	75,23	4,21	20,56
0,00	19	100,00	100,00	0,00	0,00
0,00	42	100,00	97,62	0,00	0,00
0,00	83	100,00	100,00	0,00	0,00
0,00	27	100,00	100,00	0,00	0,00
0,00	215	100,00	3,26	0,93	95,81
0,00	82	100,00	13,41	1,22	85,37
0,00	237	100,00	2,11	0,42	97,05
0,00	402	100,00	1,74	1,24	97,01
0,00	113	100,00	7,08	0,88	92,04
0,00	181	100,00	0,55	0,55	98,90
0,00	256	100,00	3,91	0,00	96,09
0,00	166	100,00	6,63	1,20	92,17
0,00	171	100,00	2,34	0,00	97,66
0,00	150	100,00	5,33	0,00	94,67
0,00	198	100,00	12,63	0,00	87,37
0,00	175	100,00	6,86	0,00	93,14
0,00	152	100,00	3,95	0,00	96,05
0,00	182	100,00	13,19	0,55	86,26
0,00	255	100,00	7,06	0,39	92,55
0,00	165	100,00	9,09	0,00	90,91
0,00	162	100,00	17,90	0,00	81,48
0,00	187	100,00	59,89	0,00	39,57
0,00	182	100,00	97,25	0,00	2,75
2,30	267	100,00	74,16	6,74	16,85
0,00	186	100,00	100,00	0,00	0,00
0,00	223	100,00	95,07	0,00	4,48
0,00	182	100,00	2,20	0,55	97,25
0,43	232	100,00	98,28	0,00	1,29
0,00	56	100,00	100,00	0,00	0,00
16,67	7	100,00	57,14	28,57	0,00
0,00	69	100,00	100,00	0,00	0,00
21,38	193	100,00	61,14	17,10	4,15
0,00	2	100,00	0,00	0,00	0,00
5,26	20	100,00	90,00	5,00	0,00
0,00	79	100,00	100,00	0,00	0,00
0,00	0	0,00	0,00	0,00	0,00
0,00	120	100,00	99,17	0,83	0,00
2,67	192	100,00	92,71	4,69	0,00

0,00	2	100,00	0,00	0,00	0,00
0,00	52	100,00	86,54	0,00	13,46
0,00	46	100,00	100,00	0,00	0,00
0,00	10	100,00	50,00	0,00	50,00
0,00	290	100,00	43,79	1,03	54,48
0,00	68	100,00	66,18	14,71	19,12
0,00	230	100,00	7,39	92,17	0,43
0,00	248	100,00	43,55	0,40	55,65
0,00	110	100,00	30,00	0,00	70,00
0,00	182	100,00	59,34	9,89	30,77
0,00	216	100,00	38,43	1,39	60,19
0,00	145	100,00	40,00	0,69	59,31
0,00	219	100,00	34,70	0,00	65,30
0,00	30	100,00	13,33	0,00	86,67
2,08	245	100,00	15,92	11,43	70,61
0,00	180	100,00	23,89	1,67	74,44
0,00	198	100,00	23,23	10,61	66,16
0,00	15	100,00	100,00	0,00	0,00
0,00	162	100,00	8,02	91,98	0,00
0,00	14	100,00	92,86	7,14	0,00
0,00	197	100,00	22,34	41,12	36,55
0,00	13	100,00	100,00	0,00	0,00
0,00	160	100,00	14,38	0,63	85,00
0,00	30	100,00	0,00	0,00	0,00
0,00	83	100,00	7,23	91,57	1,20
0,00	23	100,00	100,00	0,00	0,00
0,00	132	100,00	98,48	0,00	1,52
8,93	122	100,00	91,80	0,00	0,00
0,00	0	0,00	0,00	0,00	0,00
0,00	0	0,00	0,00	0,00	0,00
0,00	103	100,00	99,03	0,00	0,97
0,00	0	0,00	0,00	0,00	0,00
0,00	175	100,00	90,29	1,71	6,29
0,00	320	100,00	93,13	0,00	6,56
0,28	355	100,00	96,90	0,00	1,41
0,00	179	100,00	65,36	0,00	34,08
0,20	496	100,00	94,96	4,03	0,81
0,40	252	100,00	96,43	0,00	3,17
0,00	232	100,00	99,57	0,00	0,00
0,00	285	100,00	98,60	1,05	0,35
0,00	282	100,00	85,46	0,00	14,54
0,35	289	100,00	94,46	0,00	5,19
0,00	185	100,00	100,00	0,00	0,00
0,00	250	100,00	96,40	0,00	3,60
0,00	256	100,00	100,00	0,00	0,00
0,33	306	100,00	99,67	0,00	0,00
0,00	320	100,00	99,69	0,00	0,00
0,00	354	100,00	100,00	0,00	0,00
0,56	358	100,00	97,49	0,00	0,28
0,00	359	100,00	100,00	0,00	0,00

0,00	113	100,00	99,12	0,00	0,88
0,00	204	100,00	97,06	0,00	2,94
0,00	240	100,00	60,83	8,33	0,83
0,00	147	100,00	100,00	0,00	0,00
0,00	235	100,00	97,45	0,00	2,55
0,00	214	100,00	49,53	0,00	14,49
0,00	195	100,00	88,21	0,00	11,79
0,00	312	100,00	87,50	0,32	12,18
0,00	271	100,00	87,08	0,00	12,92
4,96	296	100,00	40,54	0,34	54,39
0,00	320	100,00	99,38	0,00	0,63
0,00	464	100,00	99,57	0,43	0,00
0,00	254	100,00	98,82	0,00	0,79
0,00	294	100,00	97,62	2,38	0,00
0,00	259	100,00	81,08	0,00	18,92
0,00	306	100,00	75,16	0,00	22,55
0,00	364	100,00	100,00	0,00	0,00
2,78	37	100,00	97,30	0,00	0,00
0,40	250	100,00	99,60	0,00	0,00
0,00	190	100,00	100,00	0,00	0,00
0,00	88	100,00	100,00	0,00	0,00
0,00	183	100,00	99,45	0,00	0,00
0,00	349	100,00	98,85	0,00	0,57
0,00	270	100,00	93,70	2,59	3,70
0,40	254	100,00	93,70	0,79	4,72
0,00	186	100,00	89,78	0,00	10,22
0,00	183	100,00	100,00	0,00	0,00
0,00	140	100,00	95,71	0,00	1,43
0,00	250	100,00	94,00	0,40	5,60
0,00	174	100,00	99,43	0,00	0,57
0,00	233	100,00	100,00	0,00	0,00
0,00	0	0,00	0,00	0,00	0,00
11,07	311	100,00	76,85	0,00	12,54
0,00	288	100,00	2,78	0,69	96,53
0,00	189	100,00	2,12	0,53	97,35
0,00	283	100,00	2,47	0,71	96,82
0,00	284	100,00	87,32	1,76	10,92
0,00	257	100,00	55,64	0,78	43,58
0,00	225	100,00	0,89	0,00	99,11
0,00	321	100,00	99,07	0,00	0,93
0,00	568	100,00	54,05	0,00	45,95
0,00	216	100,00	94,44	0,00	5,56
0,49	207	100,00	91,30	1,45	0,00
0,00	255	100,00	100,00	0,00	0,00
0,00	71	100,00	100,00	0,00	0,00
0,00	249	100,00	99,60	0,00	0,40
0,00	162	100,00	99,38	0,00	0,62
0,00	154	100,00	4,55	91,56	0,65
0,00	137	100,00	97,81	0,00	2,19
0,00	61	100,00	100,00	0,00	0,00

0,00	311	100,00	99,68	0,00	0,32
0,30	333	100,00	94,59	0,30	2,40
0,00	206	100,00	100,00	0,00	0,00
0,00	219	100,00	100,00	0,00	0,00
0,00	237	100,00	100,00	0,00	0,00
0,00	306	100,00	100,00	0,00	0,00
0,00	361	100,00	98,61	0,55	0,00
0,00	240	100,00	100,00	0,00	0,00
0,00	299	100,00	100,00	0,00	0,00
0,00	269	100,00	99,63	0,37	0,00
0,00	228	100,00	100,00	0,00	0,00
0,00	279	100,00	98,57	0,36	1,08
0,00	237	100,00	100,00	0,00	0,00
0,00	283	100,00	95,41	0,00	4,59
3300,00	34	100,00	0,00	0,00	0,00
0,39	257	100,00	95,72	0,00	3,89
0,00	232	100,00	100,00	0,00	0,00
0,00	278	100,00	98,92	0,00	0,36
0,00	52	100,00	0,00	0,00	0,00
0,36	276	100,00	94,93	0,36	2,54
0,00	151	100,00	54,97	0,00	45,03
0,00	401	100,00	30,67	0,00	67,58
0,00	122	100,00	98,36	0,00	1,64
0,00	304	100,00	98,36	0,00	1,64
0,00	96	100,00	96,88	0,00	3,13
0,00	146	100,00	78,08	0,68	21,23
0,50	200	100,00	67,50	0,00	32,00
0,00	295	100,00	47,12	0,00	51,53
0,00	328	100,00	95,73	0,00	3,96
0,00	268	100,00	95,52	0,00	4,48
0,00	181	100,00	97,79	0,00	2,21
0,00	262	100,00	11,45	0,00	88,55
0,00	143	100,00	86,01	0,00	13,99
8,58	253	100,00	77,87	0,00	14,23
0,00	283	100,00	71,38	0,00	28,62
0,45	449	100,00	77,95	0,22	21,38
0,61	332	100,00	54,22	0,00	6,93
0,00	313	100,00	99,36	0,00	0,64
0,00	332	100,00	78,01	0,30	21,69
0,00	56	100,00	100,00	0,00	0,00
0,00	530	100,00	99,43	0,38	0,00
0,00	202	100,00	100,00	0,00	0,00
0,00	250	100,00	99,60	0,00	0,40
0,00	285	100,00	98,95	0,00	0,00
0,00	5	100,00	100,00	0,00	0,00
0,00	34	100,00	100,00	0,00	0,00
0,00	158	100,00	100,00	0,00	0,00
0,00	163	100,00	99,39	0,00	0,61
0,00	179	100,00	100,00	0,00	0,00
0,00	410	100,00	98,05	0,00	1,95

0,00	195	100,00	100,00	0,00	0,00
0,00	332	100,00	100,00	0,00	0,00
0,37	268	100,00	98,51	0,00	0,75
0,00	300	100,00	100,00	0,00	0,00
0,00	285	100,00	100,00	0,00	0,00
0,37	272	100,00	99,26	0,00	0,37
0,00	328	100,00	98,17	1,52	0,30
0,00	84	100,00	90,48	0,00	0,00
0,00	269	100,00	94,42	0,00	5,58
0,00	186	100,00	98,39	0,00	1,61
0,00	212	100,00	94,34	0,00	5,66
0,00	223	100,00	96,86	0,00	3,14
0,00	257	100,00	97,67	0,00	2,33
0,00	281	100,00	99,29	0,00	0,71
0,00	376	100,00	63,03	0,00	35,37
0,00	314	100,00	64,01	0,32	35,67
0,00	417	100,00	83,45	0,24	16,31
0,00	0	0,00	0,00	0,00	0,00
0,00	247	100,00	52,23	0,00	47,77
0,00	249	100,00	15,66	2,81	81,53
0,00	201	100,00	72,14	1,00	26,87
0,00	168	100,00	59,52	0,00	40,48
0,51	198	100,00	29,29	0,51	69,19
0,00	265	100,00	58,87	0,00	41,13
9,89	289	100,00	78,55	0,35	12,11
0,00	143	100,00	98,60	0,00	1,40
0,00	175	100,00	85,14	0,00	14,86
0,00	346	100,00	97,40	0,29	2,31
0,00	395	100,00	68,61	0,51	30,89
0,00	299	100,00	75,59	0,00	24,41
0,00	203	100,00	95,07	0,49	3,94
0,00	272	100,00	97,06	0,37	2,57
0,00	245	100,00	92,65	0,82	6,53
0,00	308	100,00	99,03	0,00	0,97
0,00	320	100,00	99,38	0,00	0,63
0,00	55	100,00	100,00	0,00	0,00
0,00	255	100,00	98,04	0,00	1,96
0,00	309	100,00	14,56	0,65	84,79
0,00	337	100,00	99,41	0,00	0,00
0,00	255	100,00	97,25	0,00	2,75
0,00	318	100,00	98,74	1,26	0,00
0,00	110	100,00	66,36	0,91	32,73
0,00	391	100,00	77,24	0,00	22,76
0,00	326	100,00	3,68	0,00	96,32
0,00	313	100,00	7,03	0,00	92,97
0,00	284	100,00	9,51	0,00	0,35
0,00	172	100,00	100,00	0,00	0,00
1,96	260	100,00	96,92	0,38	0,77
0,00	144	100,00	99,31	0,69	0,00
0,00	120	100,00	66,67	6,67	13,33

0,00	57	100,00	100,00	0,00	0,00
0,00	312	100,00	100,00	0,00	0,00
0,00	233	100,00	97,85	0,00	2,15
0,00	0	0,00	0,00	0,00	0,00
0,00	217	100,00	92,63	0,00	6,45
0,00	185	100,00	89,19	0,00	9,73
0,00	206	100,00	98,06	0,00	1,46
0,00	284	100,00	67,61	0,00	32,39
0,00	196	100,00	77,55	0,00	15,82
0,00	167	100,00	23,35	76,05	0,60
0,36	281	100,00	82,92	0,00	9,96
0,00	281	100,00	8,54	0,00	89,68
3,59	231	100,00	89,61	0,43	6,49
0,00	181	100,00	88,40	0,00	11,60
0,00	282	100,00	76,60	0,00	23,40
0,00	147	100,00	97,96	0,00	2,04
0,00	199	100,00	62,81	34,17	3,02
0,82	123	100,00	96,75	0,00	0,81
0,00	332	100,00	100,00	0,00	0,00
0,00	343	100,00	17,20	82,80	0,00
0,00	42	100,00	100,00	0,00	0,00
0,00	104	100,00	100,00	0,00	0,00
0,00	79	100,00	100,00	0,00	0,00
0,00	164	100,00	100,00	0,00	0,00
0,37	272	100,00	95,22	0,74	1,84
0,54	186	100,00	98,39	0,00	1,08
2,25	273	100,00	87,55	0,37	9,89
0,00	227	100,00	100,00	0,00	0,00
0,00	75	100,00	100,00	0,00	0,00
0,00	329	100,00	99,70	0,00	0,30
0,00	167	100,00	100,00	0,00	0,00
0,00	236	100,00	100,00	0,00	0,00
0,00	171	100,00	46,20	0,00	6,43
0,00	114	100,00	60,53	0,88	14,91
0,00	282	100,00	70,92	0,00	4,26
12,89	403	100,00	85,61	0,00	2,98
0,00	285	100,00	100,00	0,00	0,00
0,00	210	100,00	100,00	0,00	0,00
0,00	216	100,00	100,00	0,00	0,00
0,00	334	100,00	99,40	0,00	0,00
0,00	258	100,00	99,22	0,00	0,78
0,00	131	100,00	96,18	0,00	3,82
0,00	273	100,00	82,42	1,47	16,12
0,39	260	100,00	91,92	0,38	7,31
0,00	306	100,00	98,04	0,00	1,96
0,00	221	100,00	87,78	0,00	12,22
0,00	269	100,00	36,80	33,83	29,37
0,00	337	100,00	70,92	0,00	29,08
0,00	280	100,00	100,00	0,00	0,00
0,00	279	100,00	6,45	93,19	0,00

0,00	287	100,00	94,08	0,00	5,92
0,28	355	100,00	51,55	0,00	1,41
0,00	156	100,00	100,00	0,00	0,00
0,00	134	100,00	86,57	0,00	2,99
0,00	221	100,00	97,74	0,00	2,26
0,00	283	100,00	98,23	0,00	1,77
0,00	179	100,00	100,00	0,00	0,00
7,96	244	100,00	87,70	0,00	4,92
6,02	141	100,00	92,91	0,00	0,71
0,00	150	100,00	100,00	0,00	0,00
22,77	248	100,00	75,81	0,00	4,44
0,00	212	100,00	100,00	0,00	0,00
0,00	2	100,00	0,00	0,00	0,00
0,00	29	100,00	100,00	0,00	0,00
0,00	308	100,00	99,03	0,00	0,00
0,00	135	100,00	100,00	0,00	0,00
0,00	243	100,00	99,59	0,00	0,00
0,78	259	100,00	99,23	0,00	0,00
0,54	187	100,00	99,47	0,00	0,00
0,00	204	100,00	99,02	0,98	0,00
0,71	141	100,00	97,16	0,71	0,00
0,00	186	100,00	97,31	0,00	2,69
0,00	215	100,00	51,63	0,00	0,00
0,00	193	100,00	51,30	0,00	0,00
0,00	223	100,00	76,23	0,00	0,00
0,00	172	100,00	98,84	1,16	0,00
0,00	248	100,00	54,03	0,00	0,40
0,00	244	100,00	96,72	2,46	0,82
0,00	75	100,00	94,67	0,00	4,00
0,00	174	100,00	97,70	0,00	2,30
0,00	283	100,00	99,29	0,00	0,71
0,00	333	100,00	99,10	0,00	0,90
0,00	351	100,00	96,30	1,42	1,71
0,00	331	100,00	99,70	0,00	0,30
11,30	335	100,00	89,25	0,00	0,60
0,00	223	100,00	96,86	0,00	3,14
0,00	106	100,00	96,23	0,00	3,77
0,00	189	100,00	100,00	0,00	0,00
0,00	132	100,00	98,48	0,00	1,52
0,00	306	100,00	31,70	1,63	66,67
0,00	79	100,00	72,15	1,27	26,58
0,00	0	0,00	0,00	0,00	0,00
0,00	213	100,00	99,06	0,00	0,94
0,00	148	100,00	100,00	0,00	0,00
0,00	97	100,00	93,81	0,00	6,19
0,00	293	100,00	2,39	96,59	1,02
0,00	92	100,00	14,13	81,52	1,09
0,00	96	100,00	100,00	0,00	0,00
0,00	56	100,00	100,00	0,00	0,00
0,00	91	100,00	100,00	0,00	0,00

0,00	287	100,00	98,95	0,00	1,05
0,00	99	100,00	59,60	9,09	0,00
0,00	215	100,00	100,00	0,00	0,00
0,00	33	100,00	87,88	0,00	0,00
0,00	300	100,00	99,33	0,33	0,00
0,00	228	100,00	1,75	0,44	97,81
0,00	58	100,00	79,31	0,00	20,69
0,00	328	100,00	97,87	0,91	1,22
0,00	244	100,00	2,05	1,23	96,72
0,47	214	100,00	93,46	0,47	5,61
0,00	161	100,00	1,86	0,00	98,14
12,96	61	100,00	81,97	0,00	6,56
0,00	32	100,00	84,38	0,00	15,63
0,00	91	100,00	4,40	93,41	2,20
0,00	155	100,00	98,71	0,00	1,29
0,00	155	100,00	100,00	0,00	0,00
0,00	191	100,00	97,91	0,00	2,09
0,00	120	100,00	75,83	0,00	24,17
0,00	205	100,00	91,71	0,00	8,29
0,00	298	100,00	14,43	25,50	60,07
0,00	174	100,00	98,28	0,57	1,15
0,00	145	100,00	12,41	0,00	87,59
0,00	101	100,00	49,50	17,82	32,67
1,13	269	100,00	60,97	0,00	37,92
0,00	209	100,00	70,81	0,00	26,79
0,00	267	100,00	49,06	0,37	48,69
0,00	45	100,00	31,11	0,00	0,00
0,00	59	100,00	100,00	0,00	0,00
11,00	333	100,00	89,49	0,60	0,00
0,00	117	100,00	98,29	0,85	0,85
0,00	399	100,00	96,49	0,00	3,51
0,00	18	100,00	100,00	0,00	0,00
0,00	29	100,00	6,90	93,10	0,00
0,00	54	100,00	14,81	83,33	1,85
0,00	108	100,00	98,15	0,00	1,85
0,00	415	100,00	92,29	0,00	5,78
0,00	251	100,00	99,20	0,40	0,40
0,00	165	100,00	97,58	0,00	2,42
0,00	35	100,00	82,86	17,14	0,00
0,00	89	100,00	100,00	0,00	0,00
0,00	173	100,00	9,83	0,00	90,17
0,00	151	100,00	100,00	0,00	0,00
0,00	96	100,00	10,42	89,58	0,00
0,00	177	100,00	100,00	0,00	0,00
0,00	15	100,00	100,00	0,00	0,00
0,00	0	0,00	0,00	0,00	0,00
0,00	176	100,00	99,43	0,57	0,00
0,00	211	100,00	100,00	0,00	0,00
0,00	200	100,00	65,00	2,00	3,50
0,00	10	100,00	100,00	0,00	0,00

0,00	103	100,00	53,40	8,74	37,86
0,00	119	100,00	92,44	0,00	7,56
0,00	76	100,00	92,11	0,00	7,89
0,00	319	100,00	96,55	0,00	3,45
0,00	153	100,00	1,31	0,65	98,04
0,00	259	100,00	99,61	0,39	0,00
0,00	64	100,00	4,69	0,00	95,31
0,00	250	100,00	8,80	0,40	90,80
0,00	133	100,00	16,54	0,00	83,46
0,00	71	100,00	98,59	1,41	0,00
0,00	26	100,00	100,00	0,00	0,00
0,94	107	100,00	99,07	0,00	0,00
0,00	54	100,00	94,44	0,00	5,56
0,00	200	100,00	100,00	0,00	0,00
0,00	174	100,00	99,43	0,00	0,00
0,00	159	100,00	0,00	98,74	1,26
0,00	150	100,00	24,00	75,33	0,67
0,00	225	100,00	45,33	0,44	54,22
0,00	351	100,00	100,00	0,00	0,00
0,00	148	100,00	100,00	0,00	0,00
0,00	272	100,00	77,94	22,06	0,00
0,00	171	100,00	92,98	0,00	0,00
0,00	225	100,00	99,56	0,44	0,00
0,00	292	100,00	100,00	0,00	0,00
0,00	151	100,00	100,00	0,00	0,00
0,00	21	100,00	100,00	0,00	0,00
0,00	57	100,00	100,00	0,00	0,00
0,00	147	100,00	87,07	0,00	12,24
0,00	271	100,00	83,76	0,00	16,24
4,07	179	100,00	76,54	0,00	19,55
0,00	53	100,00	98,11	0,00	1,89
0,00	57	100,00	96,49	0,00	3,51
0,00	40	100,00	100,00	0,00	0,00
0,00	70	100,00	94,29	0,00	5,71
0,00	153	100,00	81,70	0,65	17,65
0,00	168	100,00	99,40	0,00	0,60
0,00	141	100,00	85,11	0,00	14,89
0,00	245	100,00	96,73	0,00	3,27
0,00	62	100,00	75,81	0,00	1,61
0,00	362	100,00	99,72	0,28	0,00
0,00	66	100,00	100,00	0,00	0,00
0,00	57	100,00	100,00	0,00	0,00
0,00	66	100,00	96,97	0,00	3,03
0,00	45	100,00	100,00	0,00	0,00
0,00	101	100,00	100,00	0,00	0,00
0,00	31	100,00	41,94	9,68	48,39
0,00	77	100,00	87,01	1,30	11,69
0,00	72	100,00	100,00	0,00	0,00
0,00	168	100,00	100,00	0,00	0,00
0,00	190	100,00	100,00	0,00	0,00

7,03	198	100,00	78,28	0,00	10,61
0,52	193	100,00	99,48	0,00	0,00
0,00	133	100,00	93,98	0,00	6,02
0,00	89	100,00	100,00	0,00	0,00
0,00	13	100,00	100,00	0,00	0,00
0,00	59	100,00	100,00	0,00	0,00
0,00	44	100,00	0,00	0,00	0,00
0,00	200	100,00	67,00	0,00	33,00
0,00	107	100,00	99,07	0,93	0,00
0,00	0	0,00	0,00	0,00	0,00
84,21	140	100,00	54,29	0,00	0,00
0,00	189	100,00	36,51	63,49	0,00
0,00	185	100,00	91,35	0,00	8,11
0,00	144	100,00	99,31	0,69	0,00
0,00	65	100,00	100,00	0,00	0,00
0,00	234	100,00	100,00	0,00	0,00
0,00	18	100,00	83,33	16,67	0,00
0,00	242	100,00	100,00	0,00	0,00
0,00	285	100,00	100,00	0,00	0,00
0,00	223	100,00	99,55	0,45	0,00
0,00	122	100,00	100,00	0,00	0,00
0,00	339	100,00	100,00	0,00	0,00
0,00	127	100,00	100,00	0,00	0,00
0,00	167	100,00	100,00	0,00	0,00
0,00	219	100,00	100,00	0,00	0,00
0,00	45	100,00	88,89	0,00	11,11
0,00	40	100,00	60,00	0,00	0,00
3,67	226	100,00	60,62	0,00	35,84
0,00	221	100,00	86,43	0,45	13,12
0,00	47	100,00	100,00	0,00	0,00
0,00	179	100,00	100,00	0,00	0,00
0,00	54	100,00	11,11	0,00	0,00
0,00	93	100,00	98,92	0,00	1,08
0,00	115	100,00	100,00	0,00	0,00
0,00	93	100,00	98,92	1,08	0,00
0,00	255	100,00	100,00	0,00	0,00
0,00	262	100,00	94,27	0,00	5,73
0,00	0	0,00	0,00	0,00	0,00
0,00	144	100,00	56,25	43,75	0,00
0,00	16	100,00	100,00	0,00	0,00
0,00	34	100,00	5,88	0,00	94,12
0,00	73	100,00	100,00	0,00	0,00
0,00	74	100,00	95,95	0,00	4,05
0,00	63	100,00	0,00	100,00	0,00
0,00	54	100,00	1,85	98,15	0,00
0,00	65	100,00	100,00	0,00	0,00
3,10	133	100,00	96,99	0,00	0,00
0,00	8	100,00	0,00	0,00	0,00
0,00	39	100,00	100,00	0,00	0,00
0,00	54	100,00	100,00	0,00	0,00

220,00	16	100,00	0,00	0,00	0,00
0,00	381	100,00	100,00	0,00	0,00
0,00	307	100,00	99,02	0,00	0,98
0,41	243	100,00	99,18	0,41	0,00
0,91	111	100,00	92,79	2,70	3,60
0,47	214	100,00	97,20	0,47	1,87
0,58	173	100,00	99,42	0,00	0,00
3,65	312	100,00	91,35	0,00	5,13
0,00	71	100,00	100,00	0,00	0,00
0,00	332	100,00	100,00	0,00	0,00
0,45	224	100,00	99,55	0,00	0,00
0,54	187	100,00	99,47	0,00	0,00
0,00	232	100,00	98,28	0,00	1,29
0,38	263	100,00	92,40	1,90	5,32
0,00	1	100,00	0,00	0,00	0,00
0,00	339	100,00	96,46	0,59	2,95
0,00	120	100,00	96,67	0,83	2,50
0,00	216	100,00	97,69	2,31	0,00
0,00	232	100,00	98,71	0,00	1,29
0,00	227	100,00	97,80	0,00	2,20
0,00	219	100,00	100,00	0,00	0,00
0,00	436	100,00	76,15	23,85	0,00
0,00	263	100,00	99,24	0,00	0,76
0,36	279	100,00	96,06	0,36	3,23
0,00	117	100,00	100,00	0,00	0,00
0,00	164	100,00	100,00	0,00	0,00
0,00	352	100,00	64,49	0,28	35,23
4,20	447	100,00	0,45	95,53	0,00
0,43	232	100,00	98,28	0,00	1,29
0,00	97	100,00	96,91	3,09	0,00
0,00	263	100,00	100,00	0,00	0,00
0,00	321	100,00	100,00	0,00	0,00
0,00	212	100,00	99,53	0,00	0,00
0,00	107	100,00	99,07	0,00	0,00
0,00	154	100,00	99,35	0,00	0,00
0,47	215	100,00	99,53	0,00	0,00
0,00	304	100,00	100,00	0,00	0,00
0,28	355	100,00	38,03	3,10	58,59
20,33	148	100,00	83,11	0,00	0,00
0,00	360	100,00	99,72	0,00	0,28
0,00	325	100,00	99,38	0,00	0,62
0,26	384	100,00	91,67	0,00	8,07
0,26	388	100,00	98,45	0,00	0,77
0,00	336	100,00	94,35	5,36	0,00
0,00	140	100,00	60,00	40,00	0,00
0,00	263	100,00	100,00	0,00	0,00
0,00	291	100,00	100,00	0,00	0,00
0,00	263	100,00	98,48	0,00	0,38
0,00	343	100,00	99,13	0,87	0,00
0,00	255	100,00	94,12	0,00	5,88

0,00	257	100,00	67,32	9,73	8,95
0,00	370	100,00	97,84	0,00	1,89
0,00	250	100,00	98,40	0,00	1,60
0,00	250	100,00	98,80	0,00	1,20
0,00	310	100,00	97,74	0,00	1,94
0,00	258	100,00	98,84	0,78	0,00
0,00	225	100,00	99,56	0,00	0,44
0,00	293	100,00	90,78	0,34	8,87
0,00	167	100,00	100,00	0,00	0,00
0,00	131	100,00	99,24	0,00	0,00
0,00	363	100,00	99,45	0,28	0,28
0,00	172	100,00	100,00	0,00	0,00
0,00	219	100,00	100,00	0,00	0,00
0,00	169	100,00	100,00	0,00	0,00
0,46	220	100,00	92,73	0,00	0,00
0,49	206	100,00	99,03	0,00	0,49
0,56	180	100,00	99,44	0,00	0,00
0,00	208	100,00	99,52	0,48	0,00
0,23	427	100,00	98,83	0,94	0,00
0,00	209	100,00	88,52	1,44	5,74
0,00	232	100,00	99,14	0,86	0,00
0,00	222	100,00	87,39	4,50	4,50
0,00	210	100,00	97,62	0,00	2,38
0,00	117	100,00	99,15	0,85	0,00
0,00	235	100,00	100,00	0,00	0,00
0,00	286	100,00	100,00	0,00	0,00
0,00	294	100,00	97,62	0,00	1,02
0,00	280	100,00	97,50	0,00	2,50
0,00	162	100,00	99,38	0,00	0,62
0,00	257	100,00	100,00	0,00	0,00
0,00	284	100,00	87,32	11,62	1,06
0,00	209	100,00	97,13	0,48	0,96
0,00	334	100,00	99,10	0,00	0,90
0,00	254	100,00	98,82	0,39	0,00
0,00	227	100,00	40,09	0,88	59,03
0,34	292	100,00	94,86	0,00	3,42
0,00	211	100,00	0,95	0,00	98,58
0,00	286	100,00	38,11	0,35	61,54
0,00	193	100,00	24,35	0,00	75,65
0,00	263	100,00	100,00	0,00	0,00
0,00	333	100,00	100,00	0,00	0,00
0,00	199	100,00	98,99	0,00	1,01
0,00	222	100,00	93,24	0,00	6,76
0,00	191	100,00	89,01	0,00	10,99
0,00	207	100,00	100,00	0,00	0,00
0,00	175	100,00	97,71	0,00	2,29
0,00	100	100,00	100,00	0,00	0,00
0,00	315	100,00	91,75	0,32	7,62
0,00	193	100,00	99,48	0,00	0,00
0,00	233	100,00	99,57	0,00	0,00

0,00	64	100,00	100,00	0,00	0,00
0,00	307	100,00	100,00	0,00	0,00
0,00	254	100,00	100,00	0,00	0,00
0,00	349	100,00	97,71	0,29	2,01
0,00	332	100,00	96,69	0,60	2,71
0,00	164	100,00	99,39	0,00	0,61
0,00	219	100,00	99,54	0,46	0,00
0,00	282	100,00	95,39	0,35	3,19
0,00	329	100,00	96,96	0,30	2,13
0,00	256	100,00	58,20	0,00	41,80
0,00	117	100,00	89,74	0,00	9,40
0,00	14	100,00	100,00	0,00	0,00
0,00	55	100,00	100,00	0,00	0,00
0,00	81	100,00	100,00	0,00	0,00
0,00	124	100,00	99,19	0,00	0,81
0,00	161	100,00	98,76	0,00	1,24
0,00	26	100,00	100,00	0,00	0,00
1,14	89	100,00	98,88	0,00	0,00
0,00	193	100,00	95,85	0,00	2,07
0,00	110	100,00	100,00	0,00	0,00
0,00	203	100,00	9,85	89,16	0,99
0,00	27	100,00	100,00	0,00	0,00
0,00	331	100,00	100,00	0,00	0,00
0,00	237	100,00	94,51	1,69	3,38
0,00	173	100,00	99,42	0,00	0,00
0,00	207	100,00	97,58	0,00	0,97
0,00	64	100,00	100,00	0,00	0,00
0,00	158	100,00	0,00	0,00	0,00
0,00	184	100,00	100,00	0,00	0,00
0,00	10	100,00	100,00	0,00	0,00
0,00	123	100,00	100,00	0,00	0,00
0,00	64	100,00	100,00	0,00	0,00
0,00	160	100,00	100,00	0,00	0,00
0,00	143	100,00	97,90	0,00	2,10
0,00	138	100,00	94,93	3,62	1,45
0,00	475	100,00	72,21	0,00	27,37
0,00	65	100,00	98,46	0,00	1,54
0,00	66	100,00	96,97	0,00	3,03
0,00	27	100,00	100,00	0,00	0,00
0,00	9	100,00	100,00	0,00	0,00
0,00	280	100,00	95,36	0,00	4,64
0,00	182	100,00	80,22	0,00	19,78
0,00	77	100,00	62,34	37,66	0,00
0,00	180	100,00	90,56	9,44	0,00
0,00	45	100,00	100,00	0,00	0,00
2,17	47	100,00	97,87	0,00	0,00
0,00	81	100,00	91,36	8,64	0,00
0,00	86	100,00	100,00	0,00	0,00
0,00	83	100,00	100,00	0,00	0,00
0,00	97	100,00	100,00	0,00	0,00

0,00	252	100,00	26,19	1,19	72,62
0,00	56	100,00	100,00	0,00	0,00
0,40	249	100,00	99,60	0,00	0,00
0,00	24	100,00	100,00	0,00	0,00
0,00	40	100,00	0,00	0,00	0,00
0,00	0	0,00	0,00	0,00	0,00
0,00	28	100,00	100,00	0,00	0,00
0,00	180	100,00	100,00	0,00	0,00
0,00	160	100,00	100,00	0,00	0,00
0,00	155	100,00	97,42	0,00	2,58
0,00	159	100,00	98,74	0,00	1,26
0,00	43	100,00	88,37	0,00	0,00
0,00	195	100,00	99,49	0,51	0,00
0,00	153	100,00	99,35	0,00	0,65
0,00	241	100,00	99,59	0,00	0,41
1,32	77	100,00	98,70	0,00	0,00
0,00	203	100,00	68,97	31,03	0,00
0,00	719	100,00	99,30	0,00	0,70
0,00	17	100,00	0,00	0,00	0,00
0,00	194	100,00	97,94	0,00	2,06
0,00	115	100,00	96,52	1,74	0,00
0,00	249	100,00	97,19	0,00	2,81
0,00	148	100,00	95,95	1,35	1,35
0,00	194	100,00	98,45	0,00	1,55
0,00	67	100,00	100,00	0,00	0,00
0,00	212	100,00	99,06	0,00	0,94
0,00	286	100,00	87,41	2,45	10,14
0,00	202	100,00	100,00	0,00	0,00
0,00	136	100,00	100,00	0,00	0,00
0,00	46	100,00	100,00	0,00	0,00
0,00	169	100,00	94,67	0,00	5,33
0,00	55	100,00	100,00	0,00	0,00
0,00	199	100,00	40,20	0,00	0,00
0,00	1	100,00	0,00	0,00	0,00
0,00	219	100,00	100,00	0,00	0,00
0,00	55	100,00	100,00	0,00	0,00
0,00	236	100,00	99,58	0,00	0,42
0,78	130	100,00	96,92	0,77	1,54
0,00	136	100,00	2,21	0,00	96,32
0,00	186	100,00	96,77	0,00	3,23
0,00	194	100,00	98,45	0,52	0,00
0,00	51	100,00	100,00	0,00	0,00
17,56	154	100,00	85,06	0,00	0,00
0,00	146	100,00	100,00	0,00	0,00
0,00	294	100,00	3,40	0,00	95,24
0,00	321	100,00	0,31	0,00	99,69
0,00	1	100,00	0,00	0,00	0,00
0,00	94	100,00	19,15	0,00	80,85
101,34	300	100,00	44,67	0,00	5,00
0,00	319	100,00	94,67	0,00	0,00

0,00	247	100,00	100,00	0,00	0,00
0,00	228	100,00	98,25	0,00	0,00
0,00	277	100,00	98,92	0,00	1,08
0,00	221	100,00	99,10	0,90	0,00
0,00	163	100,00	96,32	0,00	3,68
0,00	190	100,00	87,89	0,00	3,68
0,00	250	100,00	99,60	0,00	0,40
0,00	247	100,00	60,32	0,00	39,68
0,00	85	100,00	91,76	1,18	1,18
0,00	68	100,00	100,00	0,00	0,00
0,00	276	100,00	100,00	0,00	0,00
2,34	394	100,00	97,72	0,00	0,00
0,00	362	100,00	98,07	0,00	0,00
0,00	230	100,00	94,78	0,00	0,00
0,00	260	100,00	100,00	0,00	0,00
0,00	211	100,00	100,00	0,00	0,00
0,00	304	100,00	100,00	0,00	0,00
0,00	189	100,00	100,00	0,00	0,00
0,00	184	100,00	98,37	1,09	0,54
0,00	216	100,00	91,20	0,00	8,80
0,00	352	100,00	96,59	0,00	3,41
0,00	261	100,00	75,10	0,00	24,90
0,00	247	100,00	3,64	0,40	95,95
0,00	222	100,00	95,95	0,45	1,80
0,45	223	100,00	85,20	0,90	13,45
0,31	321	100,00	41,74	0,00	50,78
0,00	263	100,00	97,72	0,76	1,52
0,00	367	100,00	97,00	0,00	3,00
0,00	441	100,00	78,46	0,00	21,54
0,00	244	100,00	94,67	0,00	0,41
0,00	169	100,00	100,00	0,00	0,00
2,94	245	100,00	91,84	0,00	0,82
0,25	407	100,00	99,51	0,25	0,00
0,00	186	100,00	100,00	0,00	0,00
0,00	295	100,00	100,00	0,00	0,00
0,00	304	100,00	99,67	0,33	0,00
0,72	281	100,00	85,41	0,00	0,36
0,60	167	100,00	99,40	0,00	0,00
0,00	15	100,00	0,00	0,00	0,00
2,60	395	100,00	96,96	0,00	0,51
0,00	274	100,00	99,27	0,00	0,73
0,00	299	100,00	94,31	0,00	5,69
0,00	304	100,00	97,37	0,00	2,63
0,00	337	100,00	97,92	0,00	2,08
0,00	291	100,00	86,25	4,12	9,62
0,35	285	100,00	92,98	0,00	2,11
0,36	279	100,00	91,04	0,00	8,60
0,41	244	100,00	96,31	0,41	2,87
0,00	139	100,00	99,28	0,72	0,00
0,00	320	100,00	64,69	0,31	35,00

2,68	612	100,00	38,56	0,33	58,33
0,00	205	100,00	3,90	0,00	96,10
0,00	303	100,00	29,04	0,33	70,63
0,00	70	100,00	81,43	0,00	18,57
0,00	120	100,00	92,50	0,83	5,00
13,18	292	100,00	67,81	0,00	20,55
0,00	245	100,00	19,18	0,00	80,82
0,00	149	100,00	57,05	0,00	42,95
0,00	217	100,00	58,53	38,25	3,23
3,94	132	100,00	95,45	0,00	0,76
0,00	309	100,00	96,12	2,27	0,97
0,00	218	100,00	97,71	0,00	1,83
0,00	68	100,00	100,00	0,00	0,00
0,00	279	100,00	98,57	0,00	1,43
0,00	218	100,00	96,79	0,00	3,21
0,00	239	100,00	70,71	1,67	6,28
0,00	334	100,00	94,61	0,00	5,09
0,00	105	100,00	98,10	0,00	1,90
0,34	296	100,00	98,65	0,00	1,01
0,29	341	100,00	83,58	0,00	1,17
0,00	186	100,00	99,46	0,54	0,00
0,00	193	100,00	100,00	0,00	0,00
0,45	224	100,00	96,88	0,00	2,68
0,00	318	100,00	100,00	0,00	0,00
0,25	396	100,00	85,35	0,00	14,14
0,00	320	100,00	98,44	0,00	1,56
0,00	189	100,00	93,12	0,00	6,88
0,00	261	100,00	39,08	2,30	58,62
0,00	201	100,00	79,60	0,00	19,90
0,00	178	100,00	43,82	3,37	52,81
0,00	181	100,00	2,76	88,40	8,84
0,00	338	100,00	33,73	0,00	66,27
0,00	203	100,00	57,64	1,48	40,89
0,99	102	100,00	92,16	0,00	5,88
0,00	238	100,00	1,26	98,74	0,00
0,00	191	100,00	98,43	0,00	1,57
0,00	325	100,00	99,69	0,00	0,31
1,21	167	100,00	68,86	29,94	0,00
0,00	290	100,00	97,93	0,69	1,38
0,00	166	100,00	73,49	0,00	5,42
0,47	216	100,00	94,44	0,00	2,31
0,00	112	100,00	97,32	0,89	1,79
0,00	419	100,00	99,52	0,00	0,48
0,00	325	100,00	86,15	13,85	0,00
0,00	262	100,00	100,00	0,00	0,00
0,00	252	100,00	100,00	0,00	0,00
0,00	242	100,00	99,59	0,41	0,00
0,00	319	100,00	98,75	0,00	1,25
0,00	191	100,00	99,48	0,00	0,52
0,00	310	100,00	97,74	0,00	2,26

0,00	337	100,00	99,11	0,00	0,89
0,00	356	100,00	89,33	0,00	1,69
0,00	311	100,00	87,14	0,00	2,25
0,00	348	100,00	97,13	0,00	0,57
0,00	463	100,00	97,84	0,00	0,22
0,36	278	100,00	81,65	0,36	4,68
0,00	274	100,00	1,09	0,36	98,54
0,00	395	100,00	0,00	0,76	99,24
0,00	0	0,00	0,00	0,00	0,00
0,00	177	100,00	98,31	0,00	1,69
0,00	194	100,00	98,97	0,00	1,03
2,94	350	100,00	84,00	0,00	12,57
0,00	294	100,00	92,86	0,68	5,78
0,00	217	100,00	99,54	0,00	0,46
2,27	270	100,00	97,41	0,00	0,37
0,00	236	100,00	98,31	0,00	1,69
0,37	268	100,00	97,39	0,00	0,37
0,00	149	100,00	100,00	0,00	0,00
6,45	363	100,00	93,39	0,00	0,55
0,27	371	100,00	99,73	0,00	0,00
0,00	330	100,00	94,24	0,30	1,52
0,00	282	100,00	97,87	0,00	2,13
0,00	308	100,00	95,13	0,97	3,57
0,00	111	100,00	100,00	0,00	0,00
0,00	243	100,00	97,94	0,00	2,06
0,00	220	100,00	98,64	0,00	1,36
0,00	327	100,00	99,69	0,00	0,31
0,00	233	100,00	93,56	5,15	1,29
0,00	215	100,00	7,91	88,84	3,26
0,00	342	100,00	98,83	0,58	0,00
0,00	236	100,00	17,37	82,63	0,00
0,00	49	100,00	100,00	0,00	0,00
0,00	175	100,00	100,00	0,00	0,00
5,09	227	100,00	93,39	0,00	1,76
0,00	237	100,00	67,93	0,00	32,07
0,39	257	100,00	97,28	1,95	0,39
0,00	202	100,00	99,50	0,00	0,50
0,34	293	100,00	90,44	0,00	9,22
0,72	278	100,00	97,48	1,08	0,00
0,00	326	100,00	99,39	0,00	0,61
0,00	288	100,00	91,32	0,00	8,68
0,00	200	100,00	99,50	0,00	0,00
0,00	32	100,00	100,00	0,00	0,00
0,00	332	100,00	97,29	0,00	2,71
0,00	143	100,00	98,60	0,00	1,40
0,00	199	100,00	1,51	0,00	98,49
0,00	40	100,00	85,00	0,00	10,00
2,79	221	100,00	95,02	0,00	2,26
4,39	214	100,00	90,65	5,14	0,00
0,63	160	100,00	99,38	0,00	0,00

0,00	233	100,00	97,42	0,86	1,29
0,65	154	100,00	94,81	3,25	0,00
0,00	101	100,00	99,01	0,99	0,00
0,00	106	100,00	97,17	0,00	2,83
0,00	270	100,00	91,11	2,22	6,67
0,00	255	100,00	99,22	0,78	0,00
0,00	128	100,00	97,66	2,34	0,00
0,00	120	100,00	92,50	0,00	4,17
0,00	372	100,00	94,09	0,00	5,91
3,26	317	100,00	74,13	0,00	19,87
0,00	255	100,00	70,59	0,00	29,41
0,00	146	100,00	76,03	0,00	23,97
1,93	211	100,00	93,84	0,00	4,27
0,00	199	100,00	82,41	0,50	17,09
8,49	230	100,00	68,70	0,43	22,17
0,65	311	100,00	95,18	0,00	4,18
0,00	130	100,00	60,77	0,00	39,23
0,00	195	100,00	94,36	3,59	0,51
9,03	314	100,00	81,85	0,00	9,87
0,00	329	100,00	83,89	0,30	15,81
8,92	232	100,00	73,71	0,43	17,24
8,80	136	100,00	78,68	0,00	13,24
0,00	244	100,00	54,10	0,00	45,90
0,00	272	100,00	65,07	0,00	34,93
0,00	213	100,00	49,77	10,80	38,03
0,00	250	100,00	60,00	9,60	30,40
0,88	228	100,00	87,72	0,44	10,96
0,00	145	100,00	69,66	24,83	5,52
5,26	280	100,00	52,14	0,71	41,07
0,00	449	100,00	34,74	0,22	65,03
0,00	430	100,00	100,00	0,00	0,00
0,00	155	100,00	98,71	1,29	0,00
0,00	185	100,00	99,46	0,00	0,54
0,00	240	100,00	94,58	0,00	4,58
0,00	294	100,00	97,96	0,00	1,70
0,00	257	100,00	96,89	1,17	1,56
0,41	245	100,00	88,57	0,00	11,02
0,00	419	100,00	96,66	0,00	3,34
0,00	263	100,00	98,86	0,00	1,14
0,00	250	100,00	3,20	0,40	96,40
0,00	171	100,00	97,66	0,00	2,34
0,00	324	100,00	99,07	0,00	0,93
0,00	380	100,00	98,95	0,00	1,05
0,00	312	100,00	68,27	0,64	31,09
0,00	170	100,00	100,00	0,00	0,00
0,00	258	100,00	98,84	0,00	1,16
5,17	305	100,00	93,77	0,00	0,98
0,00	311	100,00	67,85	0,00	32,15
0,00	362	100,00	100,00	0,00	0,00
0,00	400	100,00	98,00	1,00	1,00

0,00	235	100,00	95,32	0,00	4,68
0,29	347	100,00	99,14	0,00	0,58
0,40	248	100,00	99,60	0,00	0,00
0,39	260	100,00	99,62	0,00	0,00
0,00	340	100,00	91,76	0,29	7,94
165,12	114	100,00	37,72	0,00	0,00
0,00	173	100,00	97,11	0,00	1,73
0,00	1	100,00	0,00	0,00	0,00
0,00	322	100,00	99,69	0,00	0,31
0,00	282	100,00	100,00	0,00	0,00
0,00	312	100,00	100,00	0,00	0,00
0,00	111	100,00	100,00	0,00	0,00
0,00	145	100,00	100,00	0,00	0,00
0,40	250	100,00	99,60	0,00	0,00
0,65	156	100,00	88,46	7,05	3,85
0,00	107	100,00	69,16	0,00	30,84
0,00	71	100,00	84,51	0,00	15,49
0,00	81	100,00	100,00	0,00	0,00
0,00	177	100,00	99,44	0,00	0,00
0,00	29	100,00	96,55	3,45	0,00
0,00	105	100,00	86,67	0,00	13,33
0,00	80	100,00	28,75	0,00	71,25
0,00	336	100,00	25,60	0,60	73,81
0,00	96	100,00	62,50	37,50	0,00
0,00	54	100,00	100,00	0,00	0,00
0,00	228	100,00	100,00	0,00	0,00
6,72	143	100,00	93,71	0,00	0,00
0,00	57	100,00	100,00	0,00	0,00
0,00	44	100,00	100,00	0,00	0,00
0,00	134	100,00	99,25	0,00	0,00
0,00	137	100,00	100,00	0,00	0,00
0,00	75	100,00	100,00	0,00	0,00
0,00	74	100,00	100,00	0,00	0,00
0,00	137	100,00	78,10	0,00	0,00
0,00	67	100,00	97,01	0,00	2,99
0,00	145	100,00	98,62	0,00	1,38
0,00	249	100,00	29,72	0,00	70,28
0,00	160	100,00	98,75	0,00	1,25
0,00	134	100,00	97,01	1,49	1,49
0,00	255	100,00	5,10	0,00	94,90
0,00	139	100,00	91,37	0,00	2,16
0,00	18	100,00	100,00	0,00	0,00
0,00	27	100,00	92,59	0,00	3,70
0,00	36	100,00	86,11	0,00	13,89
0,00	30	100,00	90,00	0,00	0,00
0,00	3	100,00	0,00	0,00	0,00
3,23	32	100,00	96,88	0,00	0,00
14,29	40	100,00	87,50	0,00	0,00
0,00	127	100,00	0,00	0,00	100,00
0,00	32	100,00	84,38	0,00	9,38

0,00	256	100,00	100,00	0,00	0,00
0,00	409	100,00	99,27	0,73	0,00
0,00	333	100,00	98,50	0,00	1,50
0,00	221	100,00	98,19	0,00	1,36
0,51	197	100,00	97,46	0,00	2,03
0,00	346	100,00	100,00	0,00	0,00
0,00	323	100,00	97,52	0,62	1,86
0,00	138	100,00	99,28	0,00	0,72
0,00	34	100,00	100,00	0,00	0,00
0,00	40	100,00	100,00	0,00	0,00
0,00	133	100,00	99,25	0,00	0,00
0,00	57	100,00	98,25	1,75	0,00
0,00	195	100,00	98,46	0,00	1,54
0,00	43	100,00	100,00	0,00	0,00
0,00	56	100,00	100,00	0,00	0,00
0,00	157	100,00	45,86	54,14	0,00
0,00	124	100,00	100,00	0,00	0,00
0,00	120	100,00	97,50	0,00	2,50
11,69	86	100,00	89,53	0,00	0,00
0,00	52	100,00	100,00	0,00	0,00
12,66	89	100,00	88,76	0,00	0,00
0,00	53	100,00	100,00	0,00	0,00
0,00	85	100,00	100,00	0,00	0,00
0,00	63	100,00	100,00	0,00	0,00
0,00	46	100,00	100,00	0,00	0,00
0,00	91	100,00	100,00	0,00	0,00
0,00	78	100,00	100,00	0,00	0,00
0,00	75	100,00	90,67	0,00	9,33
0,00	94	100,00	100,00	0,00	0,00
0,00	93	100,00	100,00	0,00	0,00
0,00	33	100,00	84,85	0,00	15,15
0,00	246	100,00	100,00	0,00	0,00
0,00	63	100,00	98,41	0,00	1,59
0,49	205	100,00	99,51	0,00	0,00
0,00	222	100,00	99,10	0,00	0,90
0,00	129	100,00	98,45	0,00	1,55
0,00	80	100,00	100,00	0,00	0,00
0,00	171	100,00	100,00	0,00	0,00
0,00	135	100,00	97,04	2,96	0,00
0,61	165	100,00	99,39	0,00	0,00
4,88	43	100,00	79,07	0,00	16,28
0,94	107	100,00	90,65	0,00	8,41
0,00	59	100,00	76,27	13,56	3,39
0,00	76	100,00	18,42	0,00	81,58
0,00	252	100,00	99,60	0,40	0,00
0,00	195	100,00	100,00	0,00	0,00
0,68	149	100,00	97,99	1,34	0,00
0,00	135	100,00	100,00	0,00	0,00
0,00	205	100,00	90,24	0,98	2,93
0,00	249	100,00	32,53	4,02	63,45

0,00	232	100,00	99,14	0,86	0,00
0,00	292	100,00	58,90	4,45	36,64
2,40	213	100,00	92,02	1,88	0,94
0,00	49	100,00	30,61	4,08	51,02
0,00	181	100,00	0,55	1,10	98,34
0,00	172	100,00	0,00	10,47	89,53
0,00	201	100,00	31,84	1,00	54,73
0,22	465	100,00	97,85	0,00	1,94
0,00	183	100,00	98,36	0,00	1,64
0,39	258	100,00	99,61	0,00	0,00
0,00	212	100,00	98,58	0,00	1,42
0,00	315	100,00	93,02	0,00	6,98
0,00	191	100,00	97,91	0,00	1,05
8,70	200	100,00	90,50	0,00	1,50
0,00	27	100,00	100,00	0,00	0,00
0,00	21	100,00	66,67	0,00	0,00
0,00	199	100,00	37,19	0,00	61,81
0,00	188	100,00	84,57	4,26	11,17
0,00	274	100,00	3,28	0,00	96,72
48,19	246	100,00	53,25	4,88	8,54
0,00	140	100,00	93,57	0,00	6,43
0,00	197	100,00	97,46	0,00	2,54
0,00	244	100,00	100,00	0,00	0,00
0,00	210	100,00	52,38	0,00	47,62
0,00	175	100,00	94,29	1,71	4,00
0,00	246	100,00	100,00	0,00	0,00
0,00	200	100,00	96,50	1,50	0,00
0,30	337	100,00	99,41	0,30	0,00
0,00	77	100,00	100,00	0,00	0,00
2,17	188	100,00	6,38	0,00	91,49
0,00	238	100,00	0,00	0,00	100,00
2,63	234	100,00	59,83	0,00	37,61
0,00	230	100,00	2,17	0,43	97,39
0,00	96	100,00	100,00	0,00	0,00
0,00	80	100,00	5,00	95,00	0,00
0,00	203	100,00	99,51	0,00	0,49
0,00	76	100,00	94,74	0,00	5,26
3,49	89	100,00	96,63	0,00	0,00
0,00	79	100,00	100,00	0,00	0,00
0,00	272	100,00	83,46	0,00	13,24
0,00	26	100,00	96,15	0,00	3,85
0,00	53	100,00	100,00	0,00	0,00
0,00	21	100,00	100,00	0,00	0,00
0,00	6	100,00	100,00	0,00	0,00
0,00	11	100,00	100,00	0,00	0,00
0,00	26	100,00	100,00	0,00	0,00
0,00	21	100,00	100,00	0,00	0,00
0,00	36	100,00	77,78	0,00	13,89
0,00	1	0,00	0,00	0,00	0,00
0,00	45	100,00	97,78	0,00	2,22

1,19	170	100,00	97,65	0,00	0,59
0,00	0	0,00	0,00	0,00	0,00
0,00	108	100,00	85,19	0,00	14,81
0,00	191	100,00	58,12	7,33	34,55
0,00	343	100,00	62,97	4,66	32,36
0,00	252	100,00	53,57	0,00	46,43
0,00	310	100,00	48,06	12,26	39,68
0,00	303	100,00	80,53	0,00	19,47
0,00	192	100,00	90,63	2,60	6,77
0,00	172	100,00	50,58	20,35	27,91
0,00	287	100,00	68,29	6,27	25,44
0,00	204	100,00	47,55	4,90	47,55
0,00	286	100,00	74,13	2,80	23,08
0,00	265	100,00	55,85	0,38	43,77
0,00	137	100,00	29,20	24,09	46,72
0,00	149	100,00	71,14	0,00	28,86
0,00	304	100,00	70,72	0,00	29,28
0,00	212	100,00	60,85	0,00	39,15
0,00	282	100,00	60,64	0,35	38,65
0,00	199	100,00	14,07	0,00	85,93
0,00	347	100,00	76,95	0,00	23,05
0,00	194	100,00	100,00	0,00	0,00
0,00	246	100,00	77,24	5,28	17,48
0,00	207	100,00	5,31	0,00	94,69
0,00	193	100,00	100,00	0,00	0,00
0,00	93	100,00	98,92	0,00	1,08
0,00	232	100,00	16,38	2,16	81,03
0,00	253	100,00	54,55	7,51	37,55
0,00	246	100,00	76,83	0,81	22,36
0,00	133	100,00	100,00	0,00	0,00
0,44	226	100,00	63,72	7,96	26,99
0,00	269	100,00	65,80	12,64	21,56
0,00	253	100,00	71,54	0,00	28,46
0,00	265	100,00	76,23	0,00	23,77
0,35	284	100,00	40,49	23,94	35,21
1,56	65	100,00	21,54	76,92	0,00
0,00	308	100,00	44,16	6,82	49,03
0,00	125	100,00	77,60	0,00	22,40
0,00	236	100,00	26,69	0,42	72,88
0,00	2	100,00	0,00	0,00	0,00
1,57	194	100,00	68,04	0,52	29,90
0,00	237	100,00	0,42	0,00	99,58
0,00	86	100,00	56,98	2,33	40,70
0,00	113	100,00	18,58	0,00	81,42
0,00	183	100,00	68,31	0,00	31,69
0,00	171	100,00	43,27	13,45	43,27
0,00	150	100,00	10,67	81,33	8,00
26,19	53	100,00	62,26	1,89	15,09
0,00	315	100,00	25,40	13,02	61,59
19,67	73	100,00	28,77	13,70	41,10

1,03	98	100,00	58,16	0,00	40,82
0,00	306	100,00	64,71	1,96	33,33
0,00	320	100,00	40,31	11,56	48,13
0,72	279	100,00	53,76	4,30	40,50
0,00	258	100,00	48,06	0,00	51,94
0,00	66	100,00	100,00	0,00	0,00
0,00	94	100,00	100,00	0,00	0,00
0,00	150	100,00	32,67	24,67	42,67
0,00	24	100,00	0,00	0,00	0,00
5,95	178	100,00	92,13	0,56	1,69
0,00	132	100,00	96,97	0,76	2,27
0,00	203	100,00	26,11	2,46	64,04
0,00	33	100,00	54,55	0,00	45,45
0,00	142	100,00	4,23	0,00	95,77
0,00	244	100,00	5,74	0,00	94,26
0,00	69	100,00	13,04	86,96	0,00
0,00	78	100,00	100,00	0,00	0,00
0,00	255	100,00	100,00	0,00	0,00
0,00	145	100,00	99,31	0,00	0,69
0,00	235	100,00	99,57	0,00	0,43
0,00	319	100,00	97,81	0,31	1,88
0,00	236	100,00	96,61	0,00	3,39
0,00	245	100,00	97,55	0,00	2,45
0,00	80	100,00	92,50	0,00	7,50
0,00	163	100,00	40,49	0,00	59,51
0,00	285	100,00	92,28	0,00	7,72
0,00	292	100,00	91,10	0,00	8,56
0,29	344	100,00	87,79	0,29	7,27
0,00	197	100,00	100,00	0,00	0,00
0,31	323	100,00	95,05	2,48	2,17
0,00	180	100,00	100,00	0,00	0,00
0,00	352	100,00	95,45	0,00	4,26
0,00	127	100,00	91,34	0,00	0,00
0,00	235	100,00	100,00	0,00	0,00
0,00	87	100,00	100,00	0,00	0,00
0,00	241	100,00	99,59	0,00	0,41
0,00	220	100,00	100,00	0,00	0,00
0,00	94	100,00	100,00	0,00	0,00
4,33	313	100,00	93,93	0,00	1,60
0,00	275	100,00	90,18	0,00	4,00
0,00	396	100,00	84,85	1,01	14,14
0,00	229	100,00	98,69	0,00	0,87
0,00	130	100,00	100,00	0,00	0,00
5,21	222	100,00	91,44	0,00	3,15
0,00	302	100,00	95,36	0,00	4,64
0,00	182	100,00	85,71	0,00	14,29
0,00	240	100,00	99,17	0,00	0,83
0,00	202	100,00	67,82	28,22	1,98
0,00	279	100,00	91,76	0,00	2,15
0,00	97	100,00	100,00	0,00	0,00

0,00	213	100,00	99,06	0,00	0,94
0,00	206	100,00	96,60	0,00	3,40
0,00	193	100,00	95,34	0,00	4,66
0,00	187	100,00	94,65	0,00	0,00
0,00	219	100,00	88,58	0,00	0,00
0,00	348	100,00	68,68	2,01	16,95
0,00	183	100,00	65,03	0,00	1,09
0,00	328	100,00	100,00	0,00	0,00
0,00	163	100,00	98,16	0,00	0,61
0,00	219	100,00	99,54	0,00	0,46
0,00	338	100,00	93,49	0,30	1,18
14,29	96	100,00	82,29	0,00	0,00
0,00	258	100,00	99,22	0,00	0,78
0,00	281	100,00	96,44	0,00	3,56
0,00	181	100,00	96,13	0,00	3,87
13,27	222	100,00	44,14	0,90	43,24
0,00	228	100,00	59,65	0,00	39,91
0,00	256	100,00	58,59	0,00	41,41
10,36	245	100,00	62,45	0,82	27,35
0,00	253	100,00	81,03	0,79	18,18
3,29	502	100,00	62,35	0,20	34,26
0,32	316	100,00	68,35	0,32	31,01
0,00	302	100,00	58,28	12,58	29,14
0,00	194	100,00	98,45	0,00	1,55
0,00	184	100,00	0,00	1,63	98,37
0,00	292	100,00	88,36	2,05	7,53
0,00	329	100,00	87,54	0,30	12,16
1,88	217	100,00	36,41	0,46	61,29
0,00	389	100,00	37,28	0,00	62,72
0,00	318	100,00	38,05	0,31	61,64
0,86	352	100,00	52,84	0,85	45,45
0,00	27	100,00	100,00	0,00	0,00
0,00	40	100,00	62,50	35,00	2,50
11,46	282	100,00	86,52	0,00	3,19
0,00	198	100,00	46,46	1,01	52,53
0,00	190	100,00	94,74	2,63	2,63
0,47	216	100,00	0,46	99,07	0,00
0,00	254	100,00	2,36	97,64	0,00
1,30	78	100,00	98,72	0,00	0,00
0,00	148	100,00	97,97	0,00	2,03
0,00	178	100,00	5,62	94,38	0,00
0,00	299	100,00	92,64	0,00	7,36
0,00	42	100,00	97,62	0,00	2,38
0,00	242	100,00	82,23	0,41	17,36
0,00	72	100,00	95,83	4,17	0,00
0,00	391	100,00	6,65	93,09	0,26
0,00	240	100,00	33,75	2,92	63,33
0,00	295	100,00	98,31	0,00	1,69
0,00	260	100,00	98,85	0,38	0,77
0,00	327	100,00	92,05	0,00	7,95

42,45	151	100,00	52,32	0,00	17,88
0,00	253	100,00	100,00	0,00	0,00
0,00	174	100,00	99,43	0,00	0,00
0,00	203	100,00	99,51	0,00	0,49
0,00	155	100,00	100,00	0,00	0,00
0,00	199	100,00	100,00	0,00	0,00
0,00	314	100,00	99,04	0,00	0,96
0,00	290	100,00	94,83	0,34	4,83
0,34	291	100,00	93,13	0,00	6,19
0,00	264	100,00	98,86	0,00	1,14
0,00	353	100,00	98,87	0,00	1,13
0,26	384	100,00	91,67	0,00	8,07
0,00	246	100,00	97,15	0,41	2,44
0,00	222	100,00	99,10	0,45	0,45
0,00	47	100,00	97,87	2,13	0,00
0,00	208	100,00	98,08	1,44	0,48
0,00	190	100,00	98,42	1,58	0,00
0,00	259	100,00	99,23	0,77	0,00
0,00	32	100,00	100,00	0,00	0,00
0,00	204	100,00	98,04	0,49	1,47
0,00	204	100,00	99,51	0,49	0,00
0,00	200	100,00	99,50	0,00	0,50
0,00	224	100,00	99,11	0,00	0,00
0,00	242	100,00	1,65	1,24	97,11
0,00	355	100,00	5,35	2,25	92,11
0,30	338	100,00	98,82	0,00	0,89
0,00	186	100,00	99,46	0,54	0,00
0,00	336	100,00	100,00	0,00	0,00
0,00	102	100,00	98,04	0,00	1,96
0,00	191	100,00	77,49	0,00	4,71
0,00	167	100,00	89,82	0,00	9,58
0,00	262	100,00	92,37	0,00	7,63
0,00	217	100,00	94,01	0,00	5,99
0,00	302	100,00	100,00	0,00	0,00
0,00	190	100,00	0,53	11,05	0,00
0,00	170	100,00	2,94	97,06	0,00
0,29	343	100,00	59,48	40,23	0,00
0,00	331	100,00	79,76	0,30	19,94
0,43	235	100,00	68,09	0,00	30,21
0,00	185	100,00	0,00	100,00	0,00
0,00	79	100,00	0,00	100,00	0,00
0,00	194	100,00	23,20	43,81	0,00
4,37	263	100,00	26,62	0,00	69,20
0,00	268	100,00	94,03	0,00	5,97
0,00	251	100,00	1,20	0,00	98,80
0,00	191	100,00	78,53	0,00	21,47
0,00	381	100,00	74,28	1,05	24,67
4,90	385	100,00	91,95	0,00	3,38
0,00	413	100,00	70,46	0,24	29,30
0,00	349	100,00	66,19	0,29	33,52

0,96	314	100,00	83,76	0,00	15,29
0,00	177	100,00	33,90	0,00	66,10
3,93	291	100,00	78,35	0,69	17,18
0,00	106	100,00	30,19	1,89	67,92
0,00	101	100,00	11,88	0,00	88,12
0,00	48	100,00	2,08	0,00	97,92
8,56	203	100,00	89,66	0,49	1,97
1,63	187	100,00	90,91	0,53	6,95
0,00	367	100,00	92,92	0,00	6,81
0,00	222	100,00	66,22	31,53	2,25
0,76	264	100,00	93,94	0,00	5,30
0,00	423	100,00	60,28	10,17	16,55
3,35	278	100,00	71,58	3,96	21,22
0,00	131	100,00	96,95	3,05	0,00
0,00	217	100,00	11,52	88,48	0,00
0,47	213	100,00	62,44	0,47	35,68
0,00	261	100,00	1,53	98,08	0,38
0,00	272	100,00	77,57	0,00	9,19
0,00	283	100,00	86,57	1,06	12,01
0,00	342	100,00	82,46	0,29	17,25
0,00	211	100,00	66,35	33,65	0,00
0,00	207	100,00	100,00	0,00	0,00
0,00	250	100,00	89,20	2,40	1,20
0,00	211	100,00	99,53	0,00	0,00
0,00	236	100,00	99,58	0,00	0,42
0,00	352	100,00	99,72	0,28	0,00
0,00	195	100,00	98,97	0,00	1,03
0,00	283	100,00	99,65	0,35	0,00
0,00	206	100,00	76,70	0,00	23,30
4,40	332	100,00	85,84	0,30	9,64
0,00	250	100,00	97,60	0,00	2,40
0,34	294	100,00	99,66	0,00	0,00
0,74	407	100,00	99,02	0,00	0,25
0,86	234	100,00	98,29	0,00	0,00
0,00	106	100,00	99,06	0,00	0,94
0,00	89	100,00	100,00	0,00	0,00
0,00	196	100,00	92,86	0,00	0,00
0,00	168	100,00	85,12	0,00	0,00
0,28	364	100,00	96,43	3,30	0,00
0,30	337	100,00	98,81	0,00	0,89
0,00	244	100,00	97,54	0,00	1,23
0,00	233	100,00	99,57	0,43	0,00
0,00	251	100,00	96,41	0,00	1,59
0,39	258	100,00	99,61	0,00	0,00
0,00	274	100,00	96,72	0,00	0,00
0,34	299	100,00	94,98	1,00	0,00
0,00	247	100,00	100,00	0,00	0,00
0,37	268	100,00	57,84	0,00	6,72
0,00	276	100,00	98,55	0,36	0,00
0,00	177	100,00	98,87	1,13	0,00

0,00	168	100,00	100,00	0,00	0,00
0,00	335	100,00	94,33	5,67	0,00
0,00	363	100,00	98,90	0,83	0,00
0,00	178	100,00	100,00	0,00	0,00
1,05	288	100,00	78,13	0,35	0,00
0,00	150	100,00	100,00	0,00	0,00
0,00	260	100,00	100,00	0,00	0,00
0,00	234	100,00	100,00	0,00	0,00
0,00	136	100,00	100,00	0,00	0,00
0,00	287	100,00	100,00	0,00	0,00
0,00	247	100,00	100,00	0,00	0,00
0,00	423	100,00	97,40	0,24	0,00
0,00	196	100,00	99,49	0,00	0,51
0,00	199	100,00	100,00	0,00	0,00
0,00	154	100,00	100,00	0,00	0,00
0,00	170	100,00	100,00	0,00	0,00
0,00	354	100,00	69,49	0,28	30,23
0,00	305	100,00	1,97	0,00	98,03
0,00	337	100,00	1,78	0,59	97,63
0,00	131	100,00	0,76	3,82	95,42
0,00	211	100,00	12,32	1,90	85,78
0,00	212	100,00	2,36	0,00	97,64
0,00	331	100,00	8,16	0,00	91,54
0,00	381	100,00	96,85	0,00	1,05
0,00	330	100,00	99,70	0,00	0,00
0,00	182	100,00	100,00	0,00	0,00
0,00	201	100,00	53,23	0,50	46,27
0,00	6	100,00	100,00	0,00	0,00
0,00	200	100,00	100,00	0,00	0,00
0,00	270	100,00	100,00	0,00	0,00
0,32	315	100,00	95,24	0,00	4,13
0,00	137	100,00	100,00	0,00	0,00
0,00	160	100,00	81,25	0,63	18,13
0,72	279	100,00	96,42	0,00	0,36
0,00	190	100,00	100,00	0,00	0,00
0,40	248	100,00	79,44	0,00	0,81
50,00	54	100,00	51,85	0,00	0,00
0,00	251	100,00	88,05	0,40	11,55
0,28	356	100,00	43,26	1,40	55,06
0,00	245	100,00	0,00	0,82	99,18
1,03	197	100,00	81,73	0,00	17,26
0,00	0	0,00	0,00	0,00	0,00
0,00	283	100,00	82,69	2,47	14,84
0,00	262	100,00	0,76	0,00	99,24
0,00	227	100,00	23,35	0,00	76,65
0,00	174	100,00	78,74	1,15	20,11
0,00	178	100,00	73,03	0,56	25,84
0,00	212	100,00	30,66	0,00	69,34
0,33	300	100,00	91,67	0,00	8,00
0,00	236	100,00	96,61	0,00	3,39

0,00	238	100,00	72,69	8,82	15,13
0,00	160	100,00	85,00	0,00	5,63
0,00	86	100,00	76,74	0,00	9,30
0,00	197	100,00	1,02	0,51	97,97
0,00	88	100,00	94,32	0,00	2,27
0,00	65	100,00	100,00	0,00	0,00
0,00	189	100,00	95,24	0,00	4,76
0,00	391	100,00	35,55	64,45	0,00
0,00	101	100,00	100,00	0,00	0,00
0,00	124	100,00	98,39	0,00	1,61
0,00	8	100,00	100,00	0,00	0,00
0,00	46	100,00	100,00	0,00	0,00
0,00	281	100,00	100,00	0,00	0,00
0,00	73	100,00	100,00	0,00	0,00
0,00	58	100,00	98,28	0,00	0,00
0,00	279	100,00	99,28	0,36	0,36
16,67	259	100,00	41,31	0,00	44,40
0,00	98	100,00	97,96	0,00	0,00
31,25	42	100,00	73,81	2,38	0,00
0,00	58	100,00	100,00	0,00	0,00
0,00	136	100,00	100,00	0,00	0,00
0,00	28	100,00	100,00	0,00	0,00
0,00	62	100,00	35,48	1,61	62,90
0,00	23	100,00	47,83	30,43	0,00
0,00	188	100,00	1,06	1,06	97,87
0,00	267	100,00	94,01	0,37	3,00
0,48	209	100,00	88,52	0,00	10,53
0,00	204	100,00	92,65	0,00	7,35
0,00	139	100,00	100,00	0,00	0,00
0,00	244	100,00	100,00	0,00	0,00
0,00	145	100,00	98,62	0,00	1,38
0,00	187	100,00	6,42	93,05	0,53
0,00	54	100,00	100,00	0,00	0,00
0,00	17	100,00	100,00	0,00	0,00
0,00	37	100,00	100,00	0,00	0,00
0,00	153	100,00	99,35	0,00	0,65
0,00	107	100,00	98,13	0,00	1,87
0,00	271	100,00	95,57	0,37	3,69
0,73	138	100,00	90,58	8,70	0,00
0,00	89	100,00	88,76	0,00	0,00
0,00	66	100,00	92,42	0,00	7,58
0,00	81	100,00	65,43	0,00	34,57
0,00	92	100,00	100,00	0,00	0,00
0,00	116	100,00	98,28	0,86	0,86
0,00	99	100,00	100,00	0,00	0,00
0,00	33	100,00	100,00	0,00	0,00
0,00	25	100,00	96,00	0,00	4,00
0,00	175	100,00	97,71	0,00	2,29
0,00	65	100,00	98,46	0,00	1,54
0,00	50	100,00	100,00	0,00	0,00

0,00	135	100,00	99,26	0,00	0,74
0,00	86	100,00	97,67	0,00	2,33
0,00	96	100,00	94,79	1,04	4,17
0,00	93	100,00	95,70	0,00	1,08
0,00	22	100,00	100,00	0,00	0,00
1,09	185	100,00	61,62	0,00	2,16
0,00	43	100,00	83,72	4,65	11,63
0,00	114	100,00	7,89	89,47	2,63
0,00	68	100,00	95,59	0,00	4,41
0,00	175	100,00	99,43	0,00	0,57
0,00	52	100,00	17,31	19,23	63,46
0,00	43	100,00	93,02	0,00	6,98
1,54	66	100,00	89,39	0,00	1,52
0,00	43	100,00	93,02	0,00	6,98
0,00	157	100,00	10,19	87,26	2,55
0,00	132	100,00	99,24	0,00	0,00
0,00	77	100,00	9,09	90,91	0,00
0,00	89	100,00	12,36	86,52	1,12
0,00	72	100,00	18,06	81,94	0,00
0,00	350	100,00	1,43	0,29	98,29
0,00	233	100,00	62,23	16,31	21,46
0,00	37	100,00	86,49	13,51	0,00
0,00	28	100,00	96,43	0,00	3,57
0,00	257	100,00	12,84	0,00	84,05
0,00	359	100,00	2,23	0,00	97,77
0,00	190	100,00	87,89	0,00	0,53
0,00	261	100,00	25,29	5,75	68,97
0,00	306	100,00	100,00	0,00	0,00
0,00	235	100,00	97,45	2,55	0,00
0,00	183	100,00	96,72	0,00	2,73
0,00	225	100,00	99,56	0,00	0,44
0,00	150	100,00	100,00	0,00	0,00
0,00	152	100,00	76,97	0,66	22,37
0,00	136	100,00	100,00	0,00	0,00
0,00	125	100,00	97,60	0,80	0,00
0,00	239	100,00	89,12	0,84	10,04
0,57	355	100,00	13,80	0,85	84,79
0,00	190	100,00	88,42	0,00	6,32
0,00	159	100,00	98,74	1,26	0,00
0,00	234	100,00	89,74	0,00	1,28
0,00	210	100,00	100,00	0,00	0,00
0,00	176	100,00	97,73	0,00	2,27
0,00	279	100,00	97,85	0,00	2,15
0,00	286	100,00	98,95	0,00	0,00
0,00	167	100,00	100,00	0,00	0,00
0,00	254	100,00	7,48	0,00	92,52
0,00	225	100,00	100,00	0,00	0,00
0,46	217	100,00	93,09	1,84	4,61
0,00	75	100,00	93,33	0,00	6,67
0,00	167	100,00	52,69	0,00	47,31

0,00	308	100,00	63,96	1,30	34,74
0,00	169	100,00	98,82	1,18	0,00
0,44	227	100,00	99,12	0,44	0,00
0,00	231	100,00	98,70	0,87	0,43
0,00	243	100,00	100,00	0,00	0,00
56,82	138	100,00	63,77	0,00	0,00
0,00	46	100,00	95,65	4,35	0,00
0,00	31	100,00	0,00	100,00	0,00
0,00	28	100,00	28,57	71,43	0,00
0,00	18	100,00	100,00	0,00	0,00
0,00	27	100,00	100,00	0,00	0,00
0,00	18	100,00	100,00	0,00	0,00
0,00	17	100,00	100,00	0,00	0,00
0,00	23	100,00	82,61	0,00	17,39
0,00	25	100,00	4,00	96,00	0,00
0,00	45	100,00	100,00	0,00	0,00
0,00	50	100,00	100,00	0,00	0,00
0,00	22	100,00	100,00	0,00	0,00
0,00	8	100,00	100,00	0,00	0,00
0,00	86	100,00	38,37	0,00	61,63
0,00	33	100,00	100,00	0,00	0,00
2,33	44	100,00	88,64	0,00	9,09
0,00	29	100,00	20,69	79,31	0,00
0,00	11	100,00	100,00	0,00	0,00
0,00	24	100,00	95,83	4,17	0,00
0,00	87	100,00	81,61	16,09	1,15
0,00	72	100,00	44,44	1,39	54,17
0,00	20	100,00	100,00	0,00	0,00
0,00	11	100,00	100,00	0,00	0,00
0,00	29	100,00	100,00	0,00	0,00
0,00	105	100,00	94,29	0,95	2,86
0,00	33	100,00	100,00	0,00	0,00
0,00	32	100,00	75,00	25,00	0,00
122,22	20	100,00	45,00	0,00	0,00
0,00	78	100,00	100,00	0,00	0,00
0,00	30	100,00	100,00	0,00	0,00
0,00	29	100,00	100,00	0,00	0,00

DOMPRQUI	DOMPRAQ	DOMALUG	DOMCEDID	DOMCEDOU	DOMOUTCO	DOMAGGER
60,14	3,72	31,08	0,00	5,07	0,00	100,00
59,44	0,00	36,67	0,00	3,89	0,00	100,00
57,84	2,99	28,97	0,00	4,76	1,59	100,00
80,45	1,50	16,54	0,00	1,50	0,00	100,00
54,83	3,79	36,68	0,69	3,46	0,35	100,00
66,56	1,26	11,04	0,00	13,56	7,57	99,68
89,10	0,00	5,77	0,00	5,13	0,00	100,00
85,33	1,33	13,33	0,00	0,00	0,00	100,00
88,94	7,04	3,02	0,50	0,50	0,00	100,00
85,86	4,04	9,60	0,51	0,00	0,00	100,00
65,29	12,81	21,07	0,00	0,83	0,00	100,00
79,25	6,04	12,83	0,00	1,51	0,38	100,00
71,21	0,51	21,28	0,53	2,66	0,00	100,00
63,04	8,70	20,65	0,00	7,25	0,36	99,64
76,37	0,42	18,99	0,00	3,38	0,84	100,00
71,90	3,27	15,90	0,35	2,47	0,00	100,00
78,57	0,65	15,89	0,00	3,31	0,00	100,00
95,21	0,00	3,72	0,53	0,53	0,00	100,00
96,24	0,00	3,76	0,00	0,00	0,00	100,00
79,58	2,09	16,23	0,00	2,09	0,00	100,00
78,02	1,55	11,56	0,34	0,68	0,00	100,00
48,77	2,46	46,80	0,00	0,49	1,48	100,00
75,66	0,66	22,37	0,00	1,32	0,00	100,00
74,06	1,89	18,48	0,00	4,27	0,95	100,00
71,59	5,68	17,61	0,00	5,11	0,00	100,00
83,09	2,90	12,08	0,48	0,48	0,97	100,00
83,98	0,30	15,43	0,00	0,00	0,30	100,00
62,38	4,39	23,82	0,00	8,15	1,25	100,00
60,14	0,72	29,45	0,36	8,73	0,36	100,00
65,20	1,57	22,57	0,00	8,78	1,88	100,00
0,00	0,00	0,00	0,00	0,00	0,00	0,00
65,73	1,25	26,56	0,00	5,63	0,63	100,00
69,41	2,28	20,09	1,37	6,85	0,00	100,00
61,93	4,57	28,06	0,00	4,59	0,51	100,00
63,37	1,65	26,03	0,00	3,31	5,37	100,00
54,79	1,37	36,99	0,00	6,85	0,00	100,00
57,23	10,98	23,35	1,20	4,19	0,60	100,00
68,67	1,00	28,00	0,00	2,00	0,33	100,00
55,52	3,91	31,32	0,00	8,54	0,71	100,00
57,22	5,88	25,13	0,00	11,76	0,00	100,00
60,47	0,00	28,49	0,00	11,05	0,00	100,00
52,58	0,32	30,42	0,65	13,27	2,59	100,00
63,32	0,00	23,58	0,87	12,23	0,00	100,00
68,87	0,78	27,24	0,00	3,11	0,00	100,00
89,27	0,69	9,69	0,00	0,35	0,00	100,00
59,73	12,22	23,98	0,00	3,17	0,90	100,00
61,83	6,99	21,51	0,54	9,14	0,00	100,00
70,86	6,62	21,52	0,33	0,66	0,00	100,00
63,18	4,60	26,36	0,00	5,86	0,00	100,00

60,58	4,33	29,33	0,00	5,77	0,00	100,00
68,65	7,14	21,03	0,40	2,38	0,40	100,00
53,92	27,27	17,55	0,00	1,25	0,00	100,00
75,26	6,19	16,49	0,00	2,06	0,00	100,00
62,27	10,00	22,73	0,00	5,00	0,00	100,00
69,34	2,92	23,36	0,00	4,38	0,00	100,00
59,66	5,68	24,43	0,57	9,09	0,57	100,00
66,04	1,49	27,61	1,49	2,61	0,75	99,25
59,52	3,46	30,45	0,00	4,50	2,08	100,00
63,83	6,91	21,81	0,00	6,38	1,06	100,00
63,21	10,36	25,39	0,00	1,04	0,00	100,00
58,47	10,59	28,39	1,27	1,27	0,00	99,58
63,26	6,05	25,71	0,00	3,33	0,00	100,00
76,44	4,83	13,94	0,00	4,55	0,00	100,00
67,30	5,08	13,02	0,63	12,38	1,59	100,00
64,16	9,83	21,97	0,00	4,05	0,00	100,00
79,08	1,31	17,65	0,00	1,96	0,00	100,00
80,37	2,80	14,02	0,00	2,80	0,00	100,00
59,62	8,92	26,42	0,00	4,72	0,00	100,00
53,22	9,80	28,82	0,00	3,24	1,76	100,00
82,55	1,42	13,21	0,00	2,83	0,00	100,00
83,07	1,59	14,81	0,00	0,53	0,00	100,00
74,24	3,39	18,64	0,34	2,03	1,36	99,66
75,32	7,79	12,83	0,44	1,77	0,00	100,00
68,28	3,56	20,68	1,02	3,05	0,00	99,66
75,23	0,93	16,61	0,66	0,33	0,66	100,00
79,89	4,13	12,67	0,28	3,03	0,00	100,00
61,79	3,77	30,19	0,00	3,30	0,94	100,00
80,51	4,33	13,00	0,00	1,81	0,36	100,00
67,27	1,21	29,09	0,61	1,82	0,00	100,00
69,44	3,99	21,74	0,33	3,68	0,33	100,00
61,34	5,67	30,93	0,00	1,55	0,52	100,00
90,53	0,00	6,84	0,53	2,11	0,00	100,00
60,23	3,80	33,92	0,00	1,75	0,29	100,00
52,50	5,00	41,25	0,00	1,25	0,00	100,00
82,35	0,00	17,35	0,00	0,29	0,00	100,00
75,38	4,62	18,53	0,39	0,39	0,39	100,00
86,49	5,41	6,31	0,45	1,35	0,00	100,00
66,93	5,98	17,53	0,80	7,57	1,20	100,00
57,53	3,92	15,41	0,00	6,77	1,13	100,00
61,62	8,86	21,85	0,00	7,41	0,00	99,63
69,45	7,20	15,20	0,00	3,34	0,61	100,00
75,00	4,27	15,24	0,61	3,66	1,22	100,00
85,26	2,56	10,26	0,64	1,28	0,00	99,36
70,85	2,02	24,29	0,00	2,83	0,00	100,00
69,53	11,47	15,77	0,00	3,23	0,00	100,00
67,83	6,29	21,33	0,35	4,20	0,00	98,60
71,31	0,42	23,63	0,42	3,38	0,84	100,00
72,97	9,65	14,67	0,00	2,70	0,00	100,00
78,97	6,75	12,70	0,40	1,19	0,00	100,00

72,92	2,78	22,92	0,00	1,39	0,00	100,00
81,95	1,50	15,04	0,38	0,75	0,38	100,00
72,45	5,78	20,75	0,00	1,02	0,00	100,00
74,26	2,53	21,52	0,00	1,69	0,00	100,00
66,78	4,03	24,50	0,00	4,70	0,00	93,29
68,38	3,56	25,30	0,00	2,77	0,00	100,00
61,89	6,19	24,76	0,00	6,84	0,33	100,00
74,44	4,47	16,93	0,96	2,88	0,32	100,00
80,63	7,21	9,91	0,00	2,25	0,00	100,00
73,68	5,26	19,08	0,00	1,97	0,00	100,00
71,01	2,42	23,67	0,00	2,90	0,00	100,00
64,97	0,56	33,33	0,56	0,56	0,00	100,00
64,13	7,17	25,56	0,90	2,24	0,00	100,00
59,35	1,94	38,71	0,00	0,00	0,00	100,00
60,15	1,85	24,70	0,00	7,29	0,00	100,00
62,91	6,04	28,10	0,00	1,93	0,83	100,00
58,18	6,06	32,12	0,61	3,03	0,00	100,00
55,96	0,92	38,89	0,00	3,70	0,00	100,00
69,28	2,41	27,11	0,60	0,60	0,00	100,00
72,22	0,00	26,67	0,00	1,11	0,00	100,00
0,00	0,00	0,00	0,00	0,00	0,00	0,00
52,04	3,06	21,65	0,00	1,03	21,65	100,00
69,73	4,32	24,32	0,54	1,08	0,00	100,00
36,75	2,41	54,72	0,63	3,77	0,00	100,00
0,00	0,00	0,00	0,00	0,00	0,00	0,00
59,86	2,51	35,02	0,00	2,17	0,00	100,00
44,29	1,83	50,92	0,46	2,29	0,00	100,00
82,30	3,24	12,35	0,00	0,30	0,00	100,00
71,23	6,60	20,28	0,47	1,42	0,00	100,00
74,18	6,59	18,13	0,00	1,10	0,00	100,00
62,12	13,64	19,77	0,38	3,80	0,00	100,00
70,85	7,54	16,58	0,50	4,02	0,50	100,00
67,40	6,63	25,41	0,00	0,00	0,55	100,00
75,63	4,77	18,09	0,00	1,51	0,00	94,97
64,50	6,00	23,68	0,53	1,58	0,00	99,47
84,26	2,78	12,96	0,00	0,00	0,00	100,00
65,54	7,43	23,45	0,00	1,38	0,69	100,00
67,03	6,09	21,94	0,36	4,32	0,00	100,00
78,29	4,65	15,18	0,00	0,39	1,17	100,00
64,32	1,76	27,75	0,00	6,17	0,00	100,00
79,81	0,00	18,27	0,00	1,92	0,00	100,00
56,25	4,91	34,38	0,00	4,46	0,00	100,00
62,67	4,61	28,11	0,46	3,69	0,46	100,00
78,31	4,76	16,40	0,00	0,53	0,00	100,00
58,18	5,91	32,27	2,27	1,36	0,00	100,00
68,33	7,22	20,56	0,00	3,89	0,00	100,00
50,37	2,44	27,91	0,33	0,00	0,00	100,00
54,00	4,29	32,64	0,30	6,53	0,00	100,00
86,09	0,00	13,04	0,00	0,87	0,00	100,00
88,04	1,44	7,66	0,00	1,91	0,96	100,00

85,47	0,56	10,06	0,00	3,91	0,00	100,00
91,10	0,00	8,47	0,00	0,42	0,00	100,00
100,00	0,00	0,00	0,00	0,00	0,00	100,00
87,61	0,00	9,83	0,00	2,56	0,00	100,00
84,42	0,00	8,61	0,00	5,30	0,00	100,00
86,24	1,59	8,47	0,00	3,70	0,00	99,47
75,56	8,89	13,89	0,00	1,67	0,00	100,00
89,58	0,00	8,33	0,00	2,08	0,00	100,00
86,93	0,00	9,15	0,00	3,92	0,00	100,00
89,58	1,25	6,25	0,00	2,92	0,00	100,00
94,93	0,72	4,35	0,00	0,00	0,00	100,00
92,49	0,00	5,63	0,00	1,88	0,00	98,59
84,30	2,07	6,20	0,00	7,44	0,00	98,35
11,54	0,00	1,65	0,55	86,26	0,00	100,00
89,41	0,59	7,65	0,00	2,35	0,00	99,41
86,00	0,40	8,00	0,00	5,60	0,00	100,00
80,29	0,73	13,87	0,00	5,11	0,00	100,00
85,19	0,00	13,58	0,00	1,23	0,00	100,00
84,03	1,68	10,08	0,00	4,20	0,00	100,00
84,47	0,00	13,59	0,00	1,94	0,00	100,00
79,89	0,00	18,50	0,00	1,16	0,00	98,84
82,16	0,37	10,78	0,00	5,95	0,74	100,00
89,25	0,00	4,30	0,00	6,45	0,00	100,00
89,47	0,00	5,26	0,00	5,26	0,00	100,00
92,96	0,70	4,93	0,00	1,41	0,00	100,00
91,30	0,00	4,35	0,00	4,35	0,00	99,46
91,51	0,00	8,49	0,00	0,00	0,00	100,00
84,87	0,00	12,50	0,00	2,63	0,00	99,34
80,20	0,00	10,23	0,00	9,57	0,00	100,00
78,03	0,38	16,29	0,38	4,92	0,00	100,00
85,45	0,00	11,52	0,00	3,03	0,00	100,00
88,02	0,00	7,29	0,00	4,69	0,00	100,00
87,65	0,00	11,25	0,00	0,00	0,00	100,00
95,58	0,88	0,88	0,88	1,77	0,00	99,12
90,42	0,00	8,98	0,00	0,60	0,00	100,00
85,84	0,00	10,71	0,00	2,68	0,00	100,00
71,43	0,45	16,96	0,45	8,48	2,23	100,00
84,69	0,00	11,22	0,00	4,08	0,00	100,00
84,29	0,00	14,29	0,00	1,43	0,00	100,00
76,92	0,00	18,42	0,53	2,11	0,00	100,00
90,52	0,00	6,90	0,00	2,59	0,00	100,00
80,15	1,84	13,24	0,00	4,78	0,00	99,63
80,99	0,35	13,38	0,00	5,28	0,00	100,00
85,21	0,00	11,90	0,64	2,25	0,00	97,43
90,43	1,06	4,26	0,00	2,13	2,13	98,94
89,57	5,22	4,35	0,00	0,87	0,00	100,00
77,27	7,79	14,29	0,00	0,65	0,00	100,00
77,45	5,53	12,23	0,00	2,62	0,00	100,00
52,90	3,87	41,29	0,32	1,61	0,00	100,00
78,24	6,69	12,55	0,00	2,09	0,42	100,00

71,94	3,16	24,11	0,00	0,79	0,00	99,60
78,46	4,07	16,26	0,00	0,41	0,81	100,00
81,34	5,97	11,94	0,37	0,37	0,00	100,00
76,99	4,42	16,81	0,00	1,77	0,00	100,00
71,34	0,32	26,75	0,00	1,59	0,00	100,00
83,27	1,07	14,59	0,00	1,07	0,00	100,00
75,84	5,26	14,83	0,00	4,07	0,00	100,00
48,91	16,30	33,70	0,00	1,09	0,00	100,00
81,93	4,22	12,05	0,00	1,81	0,00	100,00
76,17	5,70	13,99	0,52	3,63	0,00	100,00
65,03	10,43	19,87	0,00	1,28	0,00	100,00
70,29	5,80	19,93	0,00	3,99	0,00	100,00
74,01	3,08	19,82	0,00	2,64	0,44	100,00
65,58	0,65	27,27	0,00	6,49	0,00	100,00
73,91	2,72	17,39	0,54	5,43	0,00	100,00
71,11	2,22	22,78	0,56	3,33	0,00	100,00
76,25	1,88	19,38	0,00	2,19	0,31	100,00
75,00	0,00	25,00	0,00	0,00	0,00	100,00
58,54	0,41	38,62	0,00	2,44	0,00	99,59
76,56	4,69	18,75	0,00	0,00	0,00	100,00
58,10	7,62	28,22	1,98	1,49	0,00	100,00
31,25	3,13	62,50	0,00	1,88	1,25	100,00
68,95	0,53	30,00	0,00	0,53	0,00	100,00
10,53	0,00	89,47	0,00	0,00	0,00	100,00
39,04	6,85	53,42	0,00	0,68	0,00	100,00
37,90	2,42	54,84	1,61	3,23	0,00	100,00
24,17	1,67	71,25	0,00	2,92	0,00	100,00
45,63	4,18	47,53	0,00	1,90	0,76	100,00
48,13	4,28	42,25	0,53	4,81	0,00	100,00
70,28	1,20	27,31	0,40	0,40	0,40	100,00
63,60	2,57	30,51	0,74	2,57	0,00	100,00
65,43	3,35	26,39	0,00	4,83	0,00	100,00
63,52	2,15	25,75	0,00	8,58	0,00	100,00
61,86	3,09	29,63	0,00	3,70	0,00	100,00
60,25	2,09	32,22	0,84	4,18	0,42	100,00
46,51	6,05	42,79	0,93	3,26	0,47	99,53
46,00	8,00	27,27	4,55	6,82	0,00	100,00
60,58	4,98	29,05	0,00	5,39	0,00	100,00
59,13	3,97	28,45	0,00	3,02	0,00	99,57
59,34	6,59	29,12	0,00	4,95	0,00	100,00
72,52	1,80	20,75	0,47	0,94	0,00	100,00
87,18	0,00	10,77	0,51	1,54	0,00	100,00
81,75	3,97	14,29	0,00	0,00	0,00	100,00
68,16	6,15	23,46	0,00	2,23	0,00	99,44
80,08	0,83	17,01	0,00	2,07	0,00	100,00
77,74	2,12	16,25	0,00	3,89	0,00	100,00
75,84	5,62	14,04	0,56	3,37	0,56	100,00
72,22	2,78	20,83	0,00	3,70	0,46	100,00
72,41	4,14	21,05	0,00	0,70	0,35	100,00
64,21	5,90	27,68	0,00	2,21	0,00	100,00

67,23	7,66	21,70	0,00	3,40	0,00	100,00
71,98	2,33	21,40	0,39	3,89	0,00	100,00
77,14	1,63	16,33	0,00	4,90	0,00	100,00
85,59	2,18	10,92	0,00	0,44	0,87	100,00
76,07	3,37	18,71	0,61	1,23	0,00	100,00
81,05	2,94	12,75	0,65	1,96	0,65	100,00
83,16	2,63	12,63	0,00	1,58	0,00	100,00
57,77	1,46	38,19	0,50	0,00	0,00	100,00
51,11	0,89	42,67	0,00	5,33	0,00	100,00
49,86	0,29	44,64	0,00	5,22	0,00	100,00
65,69	2,19	30,08	0,00	0,00	0,00	100,00
55,79	2,07	39,67	0,41	1,65	0,41	100,00
69,06	1,51	21,13	0,00	7,92	0,38	100,00
40,52	0,65	55,56	0,00	3,27	0,00	100,00
67,24	5,17	24,14	0,00	3,45	0,00	100,00
67,53	3,03	25,97	0,00	3,03	0,43	100,00
63,49	5,56	28,57	0,00	2,38	0,00	100,00
46,76	4,17	42,33	0,93	5,58	0,00	100,00
35,14	1,08	59,46	0,54	3,24	0,54	100,00
67,49	3,94	26,11	0,49	1,97	0,00	100,00
32,88	10,24	51,48	0,54	4,31	0,54	100,00
69,84	7,54	19,84	0,00	1,98	0,79	100,00
91,41	0,39	7,42	0,00	0,78	0,00	100,00
78,85	5,73	12,90	0,00	2,51	0,00	100,00
69,42	7,55	18,08	0,00	2,95	0,00	100,00
71,68	5,02	16,41	0,00	1,91	0,00	99,24
77,11	0,00	19,01	0,00	3,87	0,00	100,00
67,41	8,15	22,96	0,00	1,48	0,00	100,00
69,64	5,80	20,37	0,46	0,46	0,46	100,00
76,07	2,56	15,02	0,00	6,01	0,00	100,00
66,20	9,41	19,86	0,35	3,83	0,35	100,00
68,78	4,98	18,10	0,90	6,33	0,90	100,00
59,41	7,43	29,70	0,00	3,47	0,00	100,00
70,73	8,13	16,67	0,41	4,07	0,00	100,00
70,29	9,27	17,25	0,00	3,19	0,00	100,00
64,13	6,03	23,99	0,34	1,01	0,00	100,00
66,21	2,20	22,53	0,27	8,79	0,00	100,00
67,90	3,70	21,40	0,00	6,58	0,41	100,00
62,50	5,43	29,35	0,54	2,17	0,00	100,00
89,75	2,47	7,42	0,00	0,35	0,00	100,00
86,73	0,44	10,18	0,88	1,77	0,00	100,00
92,31	0,55	6,04	0,00	1,10	0,00	100,00
74,88	2,33	21,40	0,00	1,40	0,00	99,07
68,35	6,12	20,86	0,36	3,60	0,72	100,00
52,11	9,39	34,63	0,98	0,49	0,00	100,00
69,23	8,10	21,46	0,00	1,21	0,00	100,00
47,66	0,78	50,78	0,00	0,78	0,00	100,00
48,84	6,05	34,88	0,93	8,84	0,47	100,00
38,33	2,22	48,05	0,00	4,55	0,00	100,00
43,43	5,58	49,80	0,00	1,20	0,00	100,00

54,29	9,29	33,21	0,36	2,86	0,00	100,00
47,44	10,68	38,89	0,00	2,14	0,85	100,00
41,88	2,62	52,88	0,00	2,09	0,52	100,00
90,69	3,24	4,86	0,00	1,21	0,00	100,00
84,46	1,55	12,44	0,00	1,55	0,00	100,00
70,96	7,35	19,12	0,74	1,84	0,00	100,00
74,38	5,37	14,88	0,00	5,37	0,00	100,00
67,01	19,74	11,43	0,00	1,82	0,00	100,00
76,85	7,39	12,32	0,00	3,45	0,00	100,00
79,82	3,95	14,04	0,88	1,32	0,00	100,00
0,00	0,00	0,00	0,00	0,00	0,00	0,00
68,12	8,23	20,16	0,00	3,10	0,00	100,00
69,44	6,25	20,83	0,00	3,47	0,00	100,00
65,25	4,26	29,43	0,00	1,06	0,00	100,00
71,60	11,78	12,99	0,00	3,63	0,00	100,00
73,08	1,92	22,12	0,00	2,88	0,00	100,00
58,91	5,94	27,23	0,00	7,92	0,00	100,00
66,24	6,11	27,01	0,00	0,64	0,00	100,00
75,76	4,33	17,75	0,43	1,73	0,00	100,00
76,92	4,70	16,24	0,00	2,14	0,00	100,00
74,19	3,23	21,86	0,00	0,36	0,36	100,00
86,25	0,42	12,08	0,42	0,83	0,00	100,00
77,08	2,58	18,62	0,00	1,72	0,00	100,00
77,27	2,48	19,83	0,41	0,00	0,00	100,00
68,29	5,69	24,93	0,27	0,81	0,00	100,00
75,51	3,40	19,73	0,00	0,68	0,68	100,00
84,55	5,45	8,18	0,00	1,82	0,00	100,00
48,68	9,87	34,87	0,00	6,58	0,00	100,00
38,57	4,48	52,02	0,00	4,93	0,00	98,21
50,00	10,53	36,40	1,32	1,32	0,44	100,00
39,64	4,73	52,66	0,00	2,96	0,00	100,00
39,08	8,05	50,57	0,00	1,72	0,57	100,00
47,33	1,33	46,43	0,00	0,71	0,71	100,00
51,97	4,37	41,05	0,00	1,31	1,31	95,63
31,88	1,45	65,22	0,00	1,45	0,00	100,00
58,93	12,50	26,34	0,00	2,23	0,00	100,00
44,03	9,05	42,39	0,41	0,41	3,70	100,00
71,31	12,35	13,94	0,00	2,39	0,00	100,00
79,13	1,97	18,11	0,00	0,79	0,00	100,00
87,59	1,46	10,29	0,00	0,00	0,00	100,00
86,48	0,36	11,15	0,00	1,08	0,00	100,00
89,53	0,00	9,30	0,00	1,16	0,00	100,00
78,07	0,00	16,67	0,00	4,30	0,54	99,46
75,33	0,00	20,67	0,67	3,33	0,00	100,00
83,61	0,00	14,71	0,00	1,68	0,00	99,58
80,32	0,00	18,62	0,00	1,06	0,00	99,47
90,72	0,00	7,22	0,00	2,06	0,00	100,00
83,94	2,12	11,52	0,00	2,42	0,00	99,39
84,17	0,00	9,17	0,42	6,25	0,00	100,00
79,47	0,00	15,55	0,24	4,55	0,00	100,00

85,34	0,00	13,16	0,00	1,50	0,00	100,00
81,19	0,33	16,50	0,00	0,33	1,65	100,00
81,31	0,47	16,43	0,00	1,41	0,00	100,00
75,11	3,80	17,72	0,42	2,11	0,84	100,00
81,91	1,06	13,83	0,00	3,19	0,00	100,00
92,45	0,00	5,66	0,00	1,89	0,00	100,00
89,39	0,32	9,32	0,00	0,96	0,00	99,36
89,83	0,00	10,17	0,00	0,00	0,00	100,00
67,16	14,22	15,69	0,00	2,94	0,00	100,00
65,48	15,16	17,74	0,32	1,29	0,00	100,00
66,45	14,19	18,71	0,00	0,65	0,00	100,00
72,62	11,08	15,08	0,00	1,23	0,00	100,00
66,52	9,57	21,30	0,00	2,61	0,00	100,00
68,04	8,23	21,52	0,00	2,22	0,00	100,00
76,79	3,87	16,07	0,00	2,38	0,89	100,00
67,80	7,32	21,95	0,00	2,93	0,00	99,51
64,20	8,33	19,14	0,00	8,33	0,00	100,00
54,73	4,39	37,50	1,01	2,36	0,00	100,00
69,64	6,25	23,32	0,00	0,45	0,00	100,00
65,33	4,52	28,14	0,00	2,01	0,00	100,00
69,95	6,22	22,28	0,52	1,04	0,00	100,00
76,44	8,17	14,90	0,00	0,48	0,00	100,00
74,36	11,11	11,11	0,00	2,56	0,85	100,00
75,20	6,91	16,05	0,00	0,82	0,00	100,00
54,98	7,11	32,86	0,00	3,33	1,43	100,00
55,56	3,86	36,71	0,00	0,97	2,90	100,00
48,63	4,79	40,41	0,00	6,16	0,00	60,27
66,25	0,00	26,88	2,50	4,38	0,00	100,00
41,18	2,94	45,93	2,22	7,41	0,00	100,00
53,79	3,79	38,28	0,00	4,14	0,00	100,00
44,50	3,66	48,13	0,53	2,14	0,00	100,00
0,00	0,00	0,00	0,00	0,00	0,00	0,00
81,38	12,41	4,55	0,35	0,00	0,00	99,65
82,59	7,29	7,69	0,00	1,62	0,81	100,00
84,62	0,59	12,50	0,00	1,19	0,60	100,00
86,71	2,45	10,14	0,35	0,35	0,00	100,00
93,48	3,26	2,17	0,00	1,09	0,00	100,00
85,86	2,76	10,69	0,00	0,69	0,00	100,00
67,83	5,81	20,93	0,39	4,65	0,39	100,00
85,71	1,68	12,61	0,00	0,00	0,00	100,00
80,27	5,69	14,05	0,00	0,00	0,00	100,00
79,26	0,92	18,89	0,00	0,92	0,00	100,00
76,19	7,14	12,08	0,48	2,90	0,00	99,52
67,02	13,83	15,43	0,53	3,19	0,00	100,00
78,26	4,35	15,22	0,00	1,09	1,09	100,00
67,20	6,35	22,22	0,00	3,70	0,53	100,00
71,23	0,94	25,00	0,00	2,36	0,47	100,00
74,15	3,90	21,46	0,00	0,49	0,00	100,00
57,14	3,69	35,48	0,00	3,69	0,00	100,00
63,76	0,87	10,59	0,59	1,76	0,00	100,00

82,20	1,57	13,09	0,00	3,14	0,00	100,00
70,47	6,74	15,54	0,00	7,25	0,00	100,00
84,76	3,72	7,81	0,00	3,35	0,37	97,77
38,84	1,65	9,09	28,10	12,40	9,92	100,00
0,00	0,00	0,00	0,00	0,00	0,00	0,00
48,95	6,28	38,08	1,26	5,44	0,00	100,00
0,00	0,00	0,00	0,00	0,00	0,00	0,00
93,17	0,00	4,10	0,00	2,73	0,00	100,00
89,53	3,49	6,40	0,00	0,58	0,00	100,00
84,55	4,49	9,55	0,00	1,40	0,00	100,00
68,62	5,57	24,34	0,00	0,88	0,59	100,00
53,50	13,07	32,22	0,30	0,91	0,00	100,00
80,99	4,13	12,40	0,00	2,48	0,00	100,00
84,77	3,05	11,68	0,00	0,51	0,00	99,49
94,04	1,32	3,31	0,00	1,32	0,00	100,00
94,08	0,33	4,28	0,00	1,32	0,00	100,00
0,00	0,00	0,00	0,00	0,00	0,00	0,00
78,78	3,60	15,47	0,00	2,16	0,00	100,00
58,75	13,62	23,74	0,00	3,89	0,00	100,00
66,41	10,61	22,47	0,51	0,00	0,00	100,00
82,07	4,48	12,32	0,00	0,84	0,28	100,00
72,17	8,26	18,26	0,43	0,43	0,43	100,00
83,61	5,74	7,38	2,46	0,00	0,82	100,00
78,03	9,09	11,36	0,00	1,52	0,00	100,00
67,77	7,11	20,38	0,47	4,27	0,00	100,00
69,31	8,25	19,47	0,00	2,97	0,00	100,00
79,75	5,91	12,66	0,00	1,27	0,42	100,00
70,59	1,86	17,03	0,00	10,53	0,00	100,00
83,49	0,92	15,60	0,00	0,00	0,00	95,41
65,13	7,24	24,34	0,66	2,63	0,00	100,00
68,03	0,68	27,89	0,00	3,40	0,00	100,00
80,00	9,13	9,13	0,00	1,30	0,43	100,00
64,78	1,21	31,58	0,81	1,62	0,00	100,00
63,95	10,20	23,13	0,00	2,72	0,00	100,00
73,63	4,40	21,43	0,00	0,55	0,00	100,00
60,99	7,69	31,32	0,00	0,00	0,00	100,00
58,38	4,06	26,90	0,00	10,66	0,00	100,00
57,06	5,52	36,81	0,00	0,61	0,00	100,00
77,53	0,56	18,13	0,00	0,58	0,00	100,00
90,40	0,00	8,47	0,00	1,13	0,00	100,00
69,16	7,93	20,35	0,00	2,21	0,00	100,00
77,29	3,86	17,48	0,00	0,97	0,00	100,00
71,53	2,19	22,63	0,00	3,65	0,00	100,00
85,37	4,27	9,76	0,00	0,61	0,00	100,00
80,57	0,00	17,71	0,00	1,71	0,00	100,00
65,87	11,90	19,84	0,00	2,38	0,00	100,00
67,74	5,65	20,97	0,81	4,84	0,00	100,00
38,46	1,78	52,07	0,00	5,92	1,78	100,00
76,44	9,42	12,57	0,00	1,57	0,00	100,00
81,70	0,00	17,41	0,45	0,45	0,00	100,00

61,88	5,45	30,69	0,50	1,49	0,00	100,00
57,14	2,04	36,03	0,00	0,00	0,00	100,00
72,80	5,75	19,54	0,00	1,92	0,00	100,00
86,62	2,55	9,24	0,00	1,27	0,32	100,00
71,09	8,53	13,27	0,47	6,64	0,00	100,00
84,75	0,56	12,43	0,00	2,26	0,00	100,00
74,24	3,54	20,71	0,51	1,01	0,00	100,00
70,59	13,24	14,71	0,00	1,47	0,00	100,00
56,83	7,91	22,30	0,00	12,95	0,00	100,00
60,87	6,96	20,39	0,97	2,91	0,00	100,00
74,21	7,69	15,84	0,45	1,81	0,00	99,55
74,15	1,02	23,81	0,00	1,02	0,00	100,00
50,60	2,41	44,58	0,00	2,41	0,00	100,00
73,44	0,41	24,07	0,00	2,07	0,00	100,00
69,83	11,57	16,12	0,00	0,41	2,07	100,00
68,00	18,00	13,33	0,00	0,67	0,00	97,33
66,03	12,82	19,55	0,32	0,96	0,32	97,76
78,23	1,61	19,35	0,00	0,00	0,81	100,00
78,72	3,55	12,06	0,00	5,67	0,00	100,00
84,21	2,26	11,28	0,00	2,26	0,00	100,00
75,00	0,00	25,00	0,00	0,00	0,00	100,00
69,37	5,71	20,42	0,00	4,50	0,00	100,00
68,85	4,92	16,39	0,00	9,84	0,00	100,00
66,32	5,26	25,61	0,35	2,46	0,00	100,00
87,50	3,13	7,81	0,00	1,56	0,00	100,00
78,98	7,39	10,23	0,00	2,84	0,57	100,00
80,33	3,28	16,39	0,00	0,00	0,00	100,00
87,50	4,69	6,25	0,00	1,56	0,00	100,00
85,46	0,44	13,22	0,00	0,44	0,44	100,00
50,93	2,78	40,74	0,46	5,09	0,00	100,00
63,64	0,00	36,36	0,00	0,00	0,00	100,00
83,87	3,23	12,90	0,00	0,00	0,00	100,00
73,77	13,11	9,84	1,64	1,64	0,00	88,52
92,11	2,63	5,26	0,00	0,00	0,00	100,00
90,57	2,52	6,92	0,00	0,00	0,00	100,00
69,08	10,53	19,08	0,00	1,32	0,00	100,00
0,00	0,00	0,00	0,00	0,00	0,00	0,00
66,20	5,63	19,72	0,00	8,45	0,00	100,00
70,19	4,81	18,27	0,00	6,73	0,00	100,00
80,39	0,00	19,61	0,00	0,00	0,00	100,00
68,57	5,71	22,86	0,00	2,86	0,00	100,00
70,53	7,25	14,98	0,00	7,25	0,00	100,00
72,06	4,41	20,59	0,00	2,94	0,00	100,00
95,65	0,00	4,35	0,00	0,00	0,00	100,00
32,56	4,65	60,47	0,00	2,33	0,00	100,00
90,21	0,00	9,09	0,00	0,70	0,00	100,00
86,96	2,17	10,87	0,00	0,00	0,00	100,00
83,95	0,00	9,88	3,70	0,00	2,47	100,00
0,00	0,00	0,00	0,00	0,00	0,00	0,00
79,55	0,00	20,45	0,00	0,00	0,00	100,00

83,21	0,00	12,21	0,00	4,58	0,00	100,00
62,12	0,00	33,33	1,52	3,03	0,00	100,00
100,00	0,00	0,00	0,00	0,00	0,00	100,00
0,00	0,00	0,00	0,00	0,00	0,00	0,00
92,13	0,00	5,62	0,00	2,25	0,00	100,00
64,67	6,67	24,67	0,00	3,33	0,67	100,00
75,00	0,00	13,89	0,00	11,11	0,00	100,00
90,74	0,00	7,41	0,00	1,85	0,00	98,15
67,18	4,62	21,54	0,00	6,15	0,51	100,00
5,48	2,74	9,59	0,00	1,37	80,82	98,63
85,34	0,00	9,48	0,00	2,59	2,59	100,00
92,92	3,30	3,30	0,00	0,47	0,00	98,58
0,00	0,00	0,00	0,00	0,00	0,00	0,00
82,63	0,00	12,05	0,00	4,82	0,00	100,00
85,95	1,08	9,73	0,00	3,24	0,00	99,46
95,06	0,00	3,75	0,00	0,00	0,00	98,75
89,66	3,45	3,45	1,15	2,30	0,00	100,00
51,54	0,77	40,77	0,00	3,08	3,85	100,00
77,05	0,00	22,95	0,00	0,00	0,00	96,72
73,53	0,00	22,55	0,98	2,94	0,00	100,00
81,58	0,00	16,67	0,00	1,75	0,00	100,00
77,27	0,28	19,60	0,00	2,56	0,28	100,00
67,31	4,81	12,50	0,00	15,38	0,00	100,00
60,96	3,51	34,21	0,00	1,32	0,00	100,00
0,00	0,00	0,00	0,00	0,00	0,00	0,00
77,07	3,18	13,38	0,00	4,46	1,91	100,00
89,83	0,00	8,47	1,69	0,00	0,00	100,00
66,67	5,13	20,51	0,00	5,13	2,56	100,00
63,38	0,70	31,69	0,00	3,52	0,70	100,00
94,59	0,00	5,41	0,00	0,00	0,00	100,00
81,82	0,00	13,64	0,00	4,55	0,00	100,00
69,57	0,00	6,52	0,00	15,22	8,70	100,00
83,33	0,00	0,00	0,00	16,67	0,00	100,00
0,00	0,00	0,00	0,00	0,00	0,00	0,00
77,95	8,21	11,79	0,00	2,05	0,00	100,00
77,46	2,46	18,31	0,00	1,76	0,00	100,00
77,30	4,26	16,31	0,00	2,13	0,00	100,00
68,71	0,00	25,17	1,36	4,76	0,00	100,00
63,49	2,65	25,40	0,00	8,47	0,00	99,47
86,57	0,00	11,94	0,00	1,49	0,00	100,00
87,50	5,43	5,98	0,00	1,09	0,00	100,00
69,65	0,78	22,96	0,00	5,45	1,17	100,00
65,51	3,14	20,91	0,35	10,10	0,00	100,00
84,38	0,52	7,81	0,00	7,29	0,00	100,00
51,31	3,92	30,39	0,33	14,05	0,00	100,00
61,05	4,65	17,95	0,64	8,97	0,00	100,00
60,16	0,78	30,20	0,00	7,45	1,18	100,00
58,50	4,50	23,50	0,50	9,50	3,50	100,00
73,77	0,00	15,30	0,00	7,65	3,28	100,00
59,70	3,73	18,66	0,00	14,18	3,73	100,00

65,13	4,62	26,89	0,00	3,36	0,00	100,00
83,33	0,00	6,67	6,67	3,33	0,00	100,00
0,00	0,00	0,00	0,00	0,00	0,00	0,00
82,14	1,02	12,76	2,04	1,53	0,51	100,00
68,58	0,00	18,50	0,00	11,02	0,00	100,00
73,33	0,00	20,51	0,00	6,15	0,00	100,00
67,20	2,57	18,01	0,00	6,75	5,47	100,00
33,55	2,26	18,75	0,66	44,08	0,00	100,00
81,62	0,37	10,29	0,00	6,99	0,74	100,00
60,22	0,36	22,40	0,00	9,20	0,80	100,00
77,08	0,00	15,71	0,00	6,81	0,00	100,00
74,44	0,00	24,91	0,00	0,38	0,00	99,62
80,61	0,51	14,80	0,00	3,57	0,51	100,00
76,50	0,50	22,50	0,50	0,00	0,00	99,50
61,86	2,54	21,61	0,85	11,86	1,27	100,00
66,67	6,10	22,54	0,00	4,69	0,00	100,00
72,51	7,17	19,60	0,00	0,40	0,00	100,00
52,61	10,04	26,51	0,00	10,84	0,00	100,00
45,81	5,03	34,64	0,00	13,97	0,56	100,00
46,86	2,52	43,08	0,63	6,92	0,00	100,00
64,56	8,25	22,82	0,00	3,88	0,49	100,00
64,48	3,83	19,67	1,64	10,38	0,00	100,00
72,03	6,90	20,69	0,00	0,38	0,00	100,00
60,57	11,71	16,00	0,00	11,71	0,00	100,00
78,07	0,37	18,59	0,37	2,60	0,00	100,00
65,76	1,63	14,13	0,54	15,76	2,17	99,46
55,83	0,61	7,36	1,23	34,97	0,00	100,00
48,85	18,39	26,44	0,00	6,32	0,00	100,00
81,07	0,97	15,53	0,00	2,43	0,00	100,00
46,86	3,96	33,78	0,34	13,18	0,68	100,00
65,96	0,85	16,60	0,00	16,60	0,00	100,00
66,03	0,64	32,69	0,00	0,64	0,00	99,36
53,97	0,53	16,40	0,53	28,57	0,00	100,00
41,36	1,57	23,12	0,00	29,48	0,00	100,00
44,35	28,70	23,48	0,43	3,04	0,00	100,00
78,74	0,00	16,91	0,97	3,38	0,00	100,00
68,90	1,06	18,37	0,00	11,66	0,00	99,65
76,65	0,00	17,26	0,00	6,09	0,00	100,00
80,48	2,05	14,73	0,00	2,74	0,00	99,66
52,98	0,60	36,31	0,00	10,12	0,00	100,00
77,48	1,32	16,56	0,00	4,64	0,00	100,00
62,37	1,43	22,48	0,00	8,53	0,00	99,22
68,71	2,04	17,01	0,68	10,88	0,68	100,00
61,45	1,81	18,07	0,00	17,47	1,20	100,00
68,48	1,81	21,74	0,00	5,80	2,17	100,00
63,58	2,65	30,46	0,66	2,65	0,00	100,00
64,38	4,58	23,53	0,65	6,21	0,65	100,00
60,00	14,59	17,84	0,00	7,57	0,00	100,00
69,52	2,40	26,30	0,00	1,04	0,00	100,00
68,85	1,15	30,00	0,00	0,00	0,00	100,00

53,40	13,11	27,18	0,00	6,31	0,00	100,00
66,45	5,92	21,38	0,00	5,92	0,33	100,00
56,27	5,02	35,13	0,00	3,23	0,36	100,00
72,95	0,41	20,90	0,00	5,74	0,00	100,00
52,94	17,34	21,12	0,00	8,39	0,00	100,00
54,28	23,36	19,08	0,00	2,96	0,33	100,00
63,58	10,40	16,76	0,00	9,25	0,00	100,00
54,33	7,09	27,56	0,00	11,02	0,00	100,00
68,66	0,00	7,87	0,00	23,22	0,00	100,00
44,21	2,11	14,29	12,86	10,00	0,00	100,00
65,75	7,09	11,46	0,40	15,02	0,00	99,60
68,57	3,27	22,86	0,00	4,90	0,41	100,00
81,93	4,10	10,60	0,00	3,37	0,00	100,00
73,03	2,96	24,01	0,00	0,00	0,00	100,00
56,89	1,41	28,98	0,35	12,37	0,00	99,65
64,58	3,33	23,33	0,00	8,75	0,00	100,00
68,75	1,79	16,07	0,00	12,80	0,60	100,00
61,33	1,33	33,33	0,00	4,00	0,00	100,00
36,02	1,61	27,42	0,00	34,41	0,54	100,00
91,02	0,00	6,25	0,00	2,73	0,00	100,00
79,73	0,00	11,49	0,00	8,78	0,00	100,00
70,34	0,00	14,41	0,00	10,59	4,66	100,00
75,00	0,00	18,33	0,00	6,67	0,00	100,00
57,27	0,30	24,76	0,00	14,60	0,32	100,00
45,25	0,66	9,51	0,00	44,59	0,00	100,00
63,59	0,51	34,36	0,00	1,54	0,00	100,00
72,31	0,00	22,73	0,00	4,96	0,00	100,00
68,92	0,00	20,27	0,00	10,81	0,00	100,00
70,64	0,46	25,69	0,00	2,29	0,92	100,00
60,39	0,65	22,73	0,00	12,99	3,25	100,00
90,55	0,00	7,09	0,79	1,57	0,00	100,00
78,26	3,26	17,39	0,00	1,09	0,00	100,00
60,63	8,36	27,53	0,35	2,79	0,35	100,00
55,00	5,00	32,22	1,67	5,56	0,56	100,00
56,00	3,20	30,40	0,00	7,20	3,20	100,00
66,54	3,11	24,21	0,40	4,37	0,00	100,00
56,69	5,28	26,71	0,36	9,39	0,00	100,00
57,96	8,60	27,48	0,32	5,43	0,00	100,00
45,13	12,39	34,76	0,00	2,86	0,48	100,00
61,33	12,33	23,33	0,67	2,33	0,00	99,67
74,18	0,00	23,77	0,00	2,05	0,00	100,00
58,44	19,91	21,65	0,00	0,00	0,00	100,00
61,84	4,24	21,91	0,35	9,19	2,47	99,65
61,11	5,23	20,26	2,29	11,11	0,00	100,00
73,94	3,03	19,39	1,21	2,42	0,00	100,00
45,55	5,69	27,05	0,00	19,93	1,78	100,00
59,06	2,36	33,07	0,00	5,51	0,00	100,00
46,45	1,64	25,14	0,00	26,78	0,00	100,00
66,10	0,85	19,92	0,42	11,86	0,85	98,73
90,48	0,00	8,99	0,00	0,53	0,00	99,47

67,74	1,61	16,13	0,00	14,52	0,00	100,00
83,73	0,40	10,32	0,00	4,76	0,79	99,60
80,00	0,44	9,78	0,44	9,33	0,00	100,00
73,58	0,31	18,87	0,00	6,92	0,31	99,37
66,54	1,54	24,62	0,38	6,54	0,38	100,00
64,52	0,00	21,51	1,08	12,90	0,00	100,00
67,08	0,41	25,10	0,00	7,41	0,00	100,00
70,83	2,27	21,21	0,00	5,68	0,00	100,00
76,80	1,66	18,78	0,55	2,21	0,00	100,00
67,07	12,60	19,51	0,41	0,41	0,00	100,00
68,45	8,02	20,86	0,00	2,14	0,53	100,00
55,53	4,42	32,52	0,73	0,49	0,00	100,00
71,86	0,00	9,96	0,43	17,75	0,00	100,00
61,30	1,92	28,40	0,00	7,39	0,00	100,00
76,17	3,27	17,76	0,47	2,34	0,00	100,00
71,19	6,10	18,64	0,34	3,05	0,68	100,00
63,70	7,41	25,19	0,37	2,96	0,37	100,00
54,32	11,15	29,86	0,00	4,32	0,36	100,00
74,48	0,00	23,44	0,52	1,56	0,00	100,00
54,42	10,62	27,56	0,00	7,11	0,00	100,00
62,18	7,37	23,40	0,00	6,41	0,64	100,00
64,09	16,57	18,23	0,00	1,10	0,00	100,00
64,29	1,37	21,43	0,27	12,64	0,00	100,00
62,44	10,73	19,02	0,98	6,83	0,00	100,00
59,55	9,27	24,16	0,00	7,02	0,00	100,00
67,45	3,53	24,71	0,00	4,31	0,00	100,00
57,38	2,19	28,96	0,00	11,48	0,00	98,91
68,16	1,87	20,22	0,37	9,36	0,00	100,00
56,97	0,31	24,44	0,00	16,83	0,00	100,00
66,94	0,27	30,89	0,00	1,90	0,00	100,00
65,83	0,72	28,06	0,00	1,44	3,96	100,00
53,14	0,74	30,26	0,00	15,87	0,00	100,00
70,26	2,05	19,49	0,00	8,21	0,00	100,00
43,12	0,37	27,84	0,00	25,49	0,78	100,00
75,22	2,65	8,85	0,00	13,27	0,00	100,00
94,94	0,00	4,43	0,00	0,63	0,00	96,20
85,00	0,67	9,33	0,00	5,00	0,00	99,67
87,33	0,45	10,45	0,00	1,36	0,00	100,00
64,03	0,40	15,81	0,00	19,76	0,00	100,00
73,73	0,42	16,53	1,27	8,05	0,00	100,00
79,11	0,63	13,92	0,00	6,33	0,00	99,37
67,18	0,00	21,37	0,00	11,45	0,00	98,47
58,23	1,61	18,47	0,40	21,29	0,00	100,00
75,60	0,00	14,88	0,00	9,52	0,00	100,00
69,96	0,00	13,04	0,00	14,62	2,37	99,21
91,38	0,86	6,90	0,00	0,86	0,00	100,00
69,17	0,00	25,67	0,00	3,83	0,00	99,62
62,61	0,00	34,23	0,00	3,15	0,00	100,00
55,51	13,88	23,67	0,00	6,94	0,00	100,00
69,30	0,47	15,43	0,00	4,79	0,00	100,00

52,41	3,45	23,45	0,00	20,69	0,00	100,00
63,56	8,44	24,44	0,00	3,56	0,00	100,00
56,25	15,44	23,60	0,00	1,87	1,50	100,00
58,59	1,95	32,42	0,00	3,91	3,13	100,00
60,51	6,37	32,48	0,00	0,00	0,64	100,00
59,13	5,99	28,34	0,27	5,99	0,27	100,00
64,68	1,19	27,80	0,00	2,90	0,41	100,00
62,38	0,99	28,43	0,33	7,02	0,00	100,00
65,28	4,15	23,78	0,00	3,78	0,00	100,00
77,85	0,00	20,81	0,00	1,34	0,00	100,00
64,62	5,77	25,00	0,39	3,13	0,00	100,00
57,42	8,61	31,10	0,00	2,87	0,00	100,00
54,90	5,23	23,66	0,00	6,11	0,00	100,00
72,84	2,47	22,22	0,00	2,47	0,00	100,00
49,34	0,66	44,74	0,66	4,61	0,00	99,34
66,29	10,23	20,45	0,00	2,27	0,76	99,24
59,93	7,94	26,71	0,36	3,97	1,08	100,00
58,40	11,07	26,34	0,76	3,44	0,00	100,00
76,88	2,50	19,38	0,00	0,63	0,63	100,00
52,02	19,65	21,97	0,58	5,78	0,00	100,00
63,08	13,59	18,72	0,00	4,62	0,00	100,00
67,22	12,71	16,05	0,67	3,34	0,00	100,00
69,48	13,25	12,45	0,00	4,82	0,00	100,00
72,58	11,29	12,50	0,00	2,02	1,61	100,00
62,63	11,11	17,68	0,00	8,59	0,00	100,00
80,00	2,22	15,56	0,00	2,22	0,00	100,00
64,20	1,23	30,04	0,00	4,53	0,00	100,00
58,72	1,01	26,94	0,00	13,13	0,00	100,00
68,60	0,61	22,02	0,31	8,26	0,00	100,00
87,42	0,00	9,27	0,00	2,65	0,66	100,00
0,00	0,00	0,00	0,00	0,00	0,00	0,00
83,50	0,49	14,08	0,00	1,70	0,24	98,54
77,49	2,57	12,22	0,00	7,72	0,00	97,11
73,72	0,64	23,40	0,00	2,24	0,00	100,00
72,41	5,75	17,82	0,00	4,02	0,00	100,00
59,57	0,00	37,91	0,00	2,53	0,00	100,00
53,38	7,77	33,56	0,00	3,46	0,35	100,00
41,51	5,19	50,24	0,47	2,37	0,00	100,00
45,38	6,15	38,93	0,41	1,64	4,10	100,00
61,18	5,49	26,12	0,00	4,49	0,00	100,00
56,73	9,45	32,73	0,00	0,73	0,36	100,00
62,68	10,92	23,24	0,00	2,11	1,06	99,65
50,55	7,69	34,62	1,65	4,95	0,55	100,00
54,55	3,41	38,07	0,00	3,41	0,57	100,00
70,27	9,73	16,22	0,00	3,78	0,00	100,00
72,92	4,86	20,14	0,00	1,39	0,69	100,00
73,98	7,14	16,33	0,00	0,51	2,04	100,00
76,11	5,00	15,56	0,00	2,22	1,11	100,00
65,41	9,75	18,87	0,00	5,03	0,94	100,00
60,12	7,51	22,54	0,00	9,83	0,00	100,00

78,26	0,48	18,36	0,24	1,45	1,21	100,00
62,81	0,50	17,59	0,50	18,09	0,50	100,00
69,28	0,00	24,70	0,00	6,02	0,00	100,00
69,03	0,00	18,47	0,00	12,50	0,00	100,00
64,34	0,78	29,46	0,00	5,43	0,00	100,00
68,54	11,24	19,66	0,00	0,00	0,56	100,00
63,74	2,92	21,64	0,00	7,60	4,09	100,00
69,88	13,98	12,42	0,31	3,42	0,00	100,00
65,19	13,92	18,04	0,00	2,85	0,00	100,00
60,54	9,42	23,32	0,00	6,73	0,00	100,00
68,98	2,45	21,63	0,41	5,31	1,22	100,00
23,62	0,00	0,29	0,29	75,80	0,00	98,54
68,28	2,20	23,79	0,44	1,76	3,52	100,00
38,10	14,65	42,48	0,00	3,38	0,00	100,00
59,58	1,25	28,11	0,00	4,61	0,00	100,00
56,72	1,99	34,83	0,00	6,47	0,00	100,00
71,83	10,56	16,90	0,00	0,70	0,00	100,00
61,45	7,83	24,93	0,29	4,40	0,29	100,00
51,78	8,63	36,55	0,00	3,05	0,00	100,00
68,02	4,45	17,81	2,83	5,26	1,62	100,00
56,52	8,70	27,39	0,87	5,65	0,87	100,00
65,35	8,42	23,76	0,50	1,49	0,50	99,50
57,77	3,19	25,78	0,00	6,22	0,00	100,00
60,85	6,38	27,35	0,00	5,13	0,00	100,00
71,59	0,00	21,59	0,00	6,44	0,38	100,00
25,15	1,98	1,98	0,20	70,30	0,40	99,80
88,10	0,00	10,41	0,00	1,49	0,00	100,00
77,85	0,69	17,65	0,00	3,11	0,69	99,65
27,78	0,00	0,00	58,33	13,89	0,00	36,11
81,63	3,18	8,83	0,00	5,65	0,71	100,00
84,07	0,44	4,42	0,44	10,62	0,00	100,00
81,82	0,61	16,36	0,00	1,21	0,00	100,00
86,31	0,00	8,95	0,00	4,15	0,32	100,00
86,27	0,49	6,90	0,00	5,91	0,00	98,52
82,85	0,65	13,96	0,00	2,27	0,00	100,00
83,55	0,00	10,00	0,00	6,45	0,00	100,00
76,80	0,00	17,53	0,52	4,64	0,52	100,00
76,51	0,00	13,25	0,00	10,24	0,00	100,00
84,77	1,52	8,63	0,00	5,08	0,00	98,48
78,42	1,24	14,94	0,00	5,39	0,00	100,00
80,00	0,71	10,71	0,00	8,57	0,00	99,64
88,89	0,48	10,14	0,00	0,48	0,00	100,00
85,82	0,00	10,45	0,00	3,73	0,00	100,00
86,72	0,78	10,94	0,00	1,56	0,00	100,00
61,73	9,88	24,07	0,00	3,70	0,62	100,00
60,00	1,54	35,38	0,00	3,08	0,00	100,00
84,62	0,00	7,69	0,00	7,69	0,00	100,00
69,03	3,54	24,78	0,00	2,65	0,00	100,00
34,84	15,98	34,67	0,50	2,01	0,50	100,00
53,89	8,89	32,39	0,00	3,41	0,00	99,43

46,15	7,69	42,31	0,00	3,85	0,00	100,00
57,50	12,50	26,25	0,00	3,75	0,00	100,00
73,91	5,59	16,15	0,00	4,35	0,00	100,00
65,91	5,30	23,48	2,27	3,03	0,00	100,00
71,95	0,00	26,83	0,00	0,61	0,61	100,00
66,03	7,05	25,64	0,00	1,28	0,00	100,00
58,97	16,03	23,08	0,00	1,28	0,64	100,00
56,89	12,57	28,74	0,00	1,80	0,00	100,00
79,51	0,00	19,51	0,98	0,00	0,00	100,00
78,83	5,11	12,41	0,00	2,92	0,73	100,00
40,78	12,62	40,78	0,00	4,85	0,97	100,00
51,42	14,15	25,12	0,00	9,00	0,00	100,00
54,79	9,59	29,45	0,00	4,11	2,05	100,00
55,90	6,67	23,08	0,00	14,36	0,00	100,00
69,01	7,60	18,13	0,00	5,26	0,00	100,00
58,53	13,82	25,81	0,00	1,84	0,00	100,00
70,21	0,71	23,40	0,71	4,96	0,00	100,00
63,56	1,69	31,36	0,00	3,39	0,00	100,00
78,00	2,00	20,00	0,00	0,00	0,00	100,00
61,54	2,56	30,77	2,56	2,56	0,00	100,00
56,41	0,85	36,75	0,85	5,13	0,00	100,00
54,86	17,12	24,12	0,39	3,50	0,00	99,61
69,39	0,00	28,57	0,00	0,00	2,04	100,00
51,75	22,38	22,38	0,70	2,10	0,70	100,00
55,00	12,73	27,73	0,45	3,64	0,45	99,55
60,61	14,55	16,97	0,00	6,67	1,21	100,00
72,41	13,79	10,34	0,00	3,45	0,00	100,00
57,62	0,00	9,93	0,00	32,45	0,00	100,00
42,11	0,00	42,11	0,00	15,79	0,00	100,00
82,50	0,00	17,50	0,00	0,00	0,00	100,00
47,37	1,32	38,81	4,48	1,49	0,00	100,00
75,51	0,00	22,45	0,00	2,04	0,00	100,00
60,14	5,41	33,11	0,00	0,68	0,68	100,00
33,33	0,00	33,33	0,00	33,33	0,00	100,00
55,83	5,52	23,84	0,66	9,27	0,00	100,00
0,00	0,00	0,00	0,00	0,00	0,00	0,00
76,15	0,92	14,02	0,00	7,48	0,00	100,00
78,95	0,00	3,51	0,00	12,28	5,26	100,00
45,59	1,47	20,59	0,00	30,88	1,47	100,00
75,76	1,01	20,20	0,00	3,03	0,00	100,00
56,76	11,71	15,32	0,00	15,32	0,90	100,00
0,00	0,00	0,00	0,00	0,00	0,00	0,00
93,55	0,00	0,00	3,23	3,23	0,00	100,00
72,28	0,00	19,80	0,00	7,92	0,00	100,00
65,00	0,00	28,33	0,00	6,67	0,00	100,00
64,88	0,60	23,81	0,00	10,71	0,00	100,00
56,19	0,00	31,96	1,03	10,82	0,00	100,00
68,18	0,57	26,14	0,00	4,55	0,57	100,00
70,59	2,52	22,69	0,00	4,20	0,00	100,00
61,93	5,08	31,47	0,00	0,00	1,52	100,00

96,70	0,00	2,83	0,00	0,47	0,00	99,53
94,78	0,75	0,00	0,00	0,78	0,00	100,00
8,70	0,00	0,00	0,00	66,67	0,00	100,00
100,00	0,00	0,00	0,00	0,00	0,00	100,00
75,76	0,00	12,12	0,00	12,12	0,00	100,00
31,48	0,00	32,76	1,72	6,90	0,00	100,00
78,61	1,00	9,84	0,00	6,74	0,52	100,00
66,23	11,92	18,54	0,00	3,31	0,00	100,00
75,46	0,61	16,56	0,00	7,36	0,00	99,39
82,24	0,93	3,74	0,00	11,21	1,87	100,00
0,00	0,00	0,00	0,00	0,00	0,00	0,00
59,05	0,00	39,42	0,00	0,96	0,00	99,04
85,48	6,45	8,06	0,00	0,00	0,00	100,00
58,92	0,54	24,86	0,00	8,11	7,57	99,46
90,91	9,09	0,00	0,00	0,00	0,00	100,00
71,49	2,48	23,55	0,41	1,65	0,41	100,00
46,67	0,67	19,33	0,00	32,67	0,67	100,00
0,00	0,00	0,00	0,00	0,00	0,00	0,00
91,27	0,00	4,76	0,00	3,97	0,00	100,00
81,98	0,00	11,63	0,00	6,40	0,00	100,00
86,09	0,87	12,17	0,00	0,87	0,00	100,00
75,42	1,67	17,50	0,42	5,00	0,00	100,00
42,39	0,00	26,09	0,00	31,52	0,00	100,00
50,00	0,00	34,48	0,00	15,52	0,00	100,00
90,00	0,91	9,09	0,00	0,00	0,00	99,09
88,24	0,00	11,76	0,00	0,00	0,00	100,00
43,11	34,00	19,78	1,11	2,00	0,00	100,00
64,04	0,00	10,11	0,00	25,84	0,00	100,00
82,22	2,22	15,56	0,00	0,00	0,00	100,00
57,85	0,00	36,36	0,00	1,65	4,13	100,00
79,25	0,00	3,77	0,00	15,09	1,89	100,00
36,84	0,00	47,37	0,00	15,79	0,00	100,00
78,66	0,00	5,52	0,00	15,34	0,00	100,00
81,25	0,00	18,13	0,00	0,63	0,00	100,00
63,30	0,92	24,77	0,00	11,01	0,00	100,00
61,99	0,90	15,38	0,45	21,27	0,00	100,00
90,61	0,00	6,08	0,00	3,31	0,00	100,00
58,87	1,61	20,97	1,61	16,13	0,81	99,19
73,91	0,00	23,91	0,00	2,17	0,00	100,00
75,40	1,60	17,84	0,54	3,78	0,00	100,00
68,57	5,14	15,88	0,00	7,06	1,18	100,00
65,08	0,53	20,11	0,53	13,76	0,00	100,00
70,97	0,00	17,74	0,00	11,29	0,00	100,00
78,10	2,19	10,95	0,00	8,76	0,00	100,00
83,02	0,00	13,21	0,00	3,77	0,00	100,00
82,76	0,00	17,24	0,00	0,00	0,00	100,00
62,45	0,82	20,90	0,82	14,75	0,00	100,00
35,71	7,14	51,79	0,00	5,36	0,00	100,00
17,03	1,10	12,50	2,50	2,50	0,00	100,00
58,42	0,00	34,65	0,00	5,94	0,99	100,00

69,14	3,70	25,00	0,00	2,16	0,00	100,00
61,00	19,50	15,77	0,00	3,73	0,00	100,00
57,94	14,59	19,31	0,00	8,15	0,00	99,14
65,27	1,20	29,34	0,00	4,19	0,00	100,00
100,00	0,00	0,00	0,00	0,00	0,00	100,00
0,00	0,00	0,00	0,00	0,00	0,00	0,00
87,15	0,40	8,43	0,00	4,02	0,00	100,00
74,47	2,13	21,28	0,00	2,13	0,00	100,00
64,16	3,47	21,39	0,00	10,98	0,00	100,00
50,00	0,56	21,11	1,11	27,22	0,00	100,00
48,37	0,00	18,60	0,93	32,09	0,00	100,00
55,81	1,74	18,60	0,58	22,09	1,16	100,00
79,86	2,88	14,39	0,00	2,16	0,72	100,00
72,97	0,00	18,92	0,00	8,11	0,00	100,00
90,00	0,00	6,67	0,00	3,33	0,00	100,00
81,61	0,00	6,17	0,00	6,17	0,00	98,77
57,89	0,00	42,11	0,00	0,00	0,00	100,00
96,43	0,00	0,00	0,00	3,57	0,00	100,00
51,38	4,59	3,67	0,00	40,37	0,00	99,08
77,66	0,51	6,09	0,00	15,23	0,51	99,49
72,94	4,71	3,53	0,00	18,82	0,00	100,00
66,53	2,12	24,58	0,00	6,78	0,00	100,00
73,64	0,45	17,35	0,00	3,65	4,57	100,00
76,17	1,44	16,61	0,00	5,42	0,36	100,00
74,53	3,11	20,00	0,00	1,88	0,00	100,00
75,51	2,04	22,45	0,00	0,00	0,00	100,00
72,96	12,45	13,30	0,00	0,43	0,86	97,85
60,19	18,45	17,48	0,00	2,91	0,97	99,51
83,63	3,54	8,85	0,88	2,65	0,44	100,00
50,68	0,34	39,12	0,00	9,86	0,00	100,00
73,04	0,43	20,87	0,00	3,04	2,61	100,00
68,70	0,76	22,14	0,76	7,63	0,00	100,00
62,65	6,83	20,75	0,00	6,22	1,24	99,59
63,79	3,45	29,31	0,00	2,59	0,86	100,00
74,26	3,80	17,72	0,00	4,22	0,00	100,00
72,41	7,33	17,67	0,00	2,59	0,00	100,00
83,14	0,58	12,79	0,00	3,49	0,00	99,42
49,32	12,67	36,20	0,00	1,81	0,00	100,00
61,84	7,73	27,05	0,97	2,42	0,00	100,00
0,00	0,00	0,00	0,00	0,00	0,00	0,00
61,75	6,56	27,32	0,55	3,28	0,55	100,00
58,19	0,56	35,03	0,00	6,21	0,00	98,31
39,61	42,03	16,43	0,00	1,93	0,00	100,00
79,52	6,48	12,97	0,00	1,02	0,00	100,00
77,57	1,87	18,69	0,47	0,93	0,47	100,00
77,78	1,63	19,61	0,65	0,33	0,00	100,00
57,93	2,07	18,97	0,34	19,31	1,38	100,00
76,33	0,00	18,73	0,00	4,95	0,00	100,00
66,50	1,48	10,84	0,49	20,20	0,49	97,04
62,62	0,00	29,44	1,87	5,14	0,93	100,00

48,97	0,00	25,77	1,55	23,71	0,00	100,00
64,29	1,30	19,48	1,30	12,34	1,30	100,00
0,00	0,00	0,00	0,00	0,00	0,00	0,00
43,37	13,25	40,96	0,00	2,41	0,00	100,00
63,43	11,57	22,76	0,00	2,24	0,00	100,00
63,36	9,93	20,65	1,09	0,72	0,00	100,00
61,54	13,85	23,59	0,00	1,03	0,00	100,00
52,61	17,54	24,64	0,00	4,74	0,47	100,00
69,05	0,48	27,14	0,48	2,38	0,48	100,00
25,26	41,05	30,53	1,05	2,11	0,00	100,00
76,00	0,00	18,00	1,00	5,00	0,00	100,00
48,55	13,87	17,92	1,73	13,29	4,62	100,00
76,47	0,00	21,69	0,00	1,47	0,37	100,00
63,58	0,60	28,96	1,19	5,67	0,00	99,70
5,63	0,00	50,00	0,00	0,00	0,00	100,00
70,36	9,58	17,37	0,00	2,69	0,00	100,00
10,50	82,65	5,02	0,00	0,00	1,83	100,00
79,48	0,00	19,03	0,00	1,49	0,00	100,00
76,19	0,00	9,52	0,00	14,29	0,00	100,00
62,23	1,08	28,88	0,00	7,58	0,00	100,00
60,20	2,68	25,08	0,00	11,37	0,67	100,00
56,36	1,21	27,88	0,00	14,55	0,00	100,00
63,56	1,27	31,36	0,00	3,81	0,00	100,00
79,74	1,76	5,73	0,00	12,78	0,00	98,68
75,60	0,00	15,48	0,00	8,93	0,00	100,00
66,90	1,38	22,76	0,00	8,97	0,00	100,00
86,00	0,00	10,00	0,00	4,00	0,00	100,00
85,44	1,27	4,43	0,00	8,86	0,00	98,73
53,37	1,04	34,20	0,00	10,88	0,52	100,00
79,28	0,33	8,88	0,00	10,86	0,66	100,00
98,33	0,00	1,67	0,00	0,00	0,00	99,58
59,73	0,44	26,67	1,43	6,67	0,48	99,52
77,85	1,30	16,29	0,00	4,56	0,00	100,00
93,33	0,00	3,64	0,00	3,03	0,00	100,00
65,53	11,06	17,87	0,43	5,11	0,00	99,57
53,48	3,21	32,62	0,53	9,63	0,53	100,00
83,44	1,23	13,50	0,00	1,84	0,00	99,39
89,94	2,52	4,40	0,00	3,14	0,00	100,00
92,00	0,00	6,90	0,00	0,57	0,00	100,00
93,22	0,00	4,24	0,00	2,54	0,00	100,00
66,67	0,00	20,69	0,00	12,64	0,00	100,00
70,70	0,56	23,38	0,00	5,35	0,00	100,00
55,07	0,00	20,65	0,00	5,43	18,84	100,00
61,64	0,33	24,34	0,33	13,16	0,00	100,00
50,88	0,00	15,14	0,00	33,80	0,00	100,00
86,78	3,31	7,44	0,00	2,48	0,00	100,00
80,80	0,00	13,60	0,00	5,60	0,00	100,00
72,48	11,01	16,51	0,00	0,00	0,00	100,00
71,11	2,22	24,44	0,00	2,22	0,00	97,78
0,00	0,00	0,00	0,00	0,00	0,00	0,00

65,65	2,38	7,48	0,34	10,54	13,61	100,00
76,86	0,00	11,32	0,94	0,00	0,00	100,00
68,90	1,57	23,23	0,00	5,51	0,79	100,00
81,88	0,67	16,11	0,00	1,34	0,00	100,00
84,34	0,00	12,05	0,00	3,61	0,00	100,00
78,51	0,83	19,17	0,00	0,83	0,00	100,00
82,86	0,00	8,57	0,00	8,57	0,00	100,00
50,75	7,84	30,60	0,75	10,07	0,00	100,00
59,78	5,90	27,41	0,00	5,93	0,74	100,00
65,38	3,21	19,87	0,00	10,90	0,64	100,00
58,82	1,68	33,61	2,52	3,36	0,00	100,00
51,48	9,17	31,85	0,30	6,85	0,00	100,00
60,51	6,88	24,09	0,00	6,57	1,46	100,00
53,37	27,46	17,10	0,00	2,07	0,00	100,00
70,09	0,47	27,57	0,00	1,87	0,00	100,00
68,23	0,52	23,44	0,00	6,25	1,56	100,00
73,77	2,73	22,40	0,00	1,09	0,00	100,00
66,67	2,59	21,68	0,00	9,06	0,00	100,00
70,15	2,69	21,43	0,00	2,80	0,00	100,00
56,43	0,00	42,50	0,00	1,07	0,00	99,64
72,64	8,45	15,20	0,34	2,70	0,68	100,00
60,75	3,27	27,10	0,00	8,41	0,47	100,00
69,68	6,38	18,09	0,00	4,26	1,60	99,47
62,13	3,55	20,96	0,60	11,38	0,60	100,00
61,88	1,49	24,75	0,00	11,88	0,00	100,00
73,33	0,00	23,86	0,00	2,81	0,00	100,00
73,63	0,00	23,63	0,00	2,74	0,00	100,00
63,40	1,63	25,25	0,00	9,51	0,00	99,67
43,31	7,27	40,23	0,00	8,75	0,29	100,00
54,43	7,65	29,97	0,00	7,95	0,00	100,00
63,77	1,93	32,37	0,00	1,93	0,00	100,00
63,75	2,50	31,25	0,31	1,88	0,31	100,00
61,29	0,00	25,81	3,23	9,68	0,00	100,00
71,05	0,00	20,00	0,00	7,89	1,05	100,00
57,20	2,06	32,51	0,00	8,23	0,00	100,00
72,00	2,40	21,60	0,80	3,20	0,00	100,00
68,14	0,63	25,55	0,00	5,68	0,00	100,00
49,86	0,85	27,04	0,85	21,41	0,00	100,00
76,05	0,76	17,87	0,00	5,32	0,00	100,00
50,39	22,57	16,54	0,26	9,97	0,26	100,00
69,15	13,43	16,92	0,00	0,50	0,00	100,00
62,24	14,80	19,90	0,00	3,06	0,00	100,00
80,95	1,19	14,29	0,40	3,17	0,00	100,00
76,68	4,95	16,25	0,00	2,12	0,00	100,00
71,02	7,42	18,37	0,71	2,47	0,00	99,65
68,05	4,14	27,81	0,00	0,00	0,00	100,00
69,36	11,45	17,51	0,00	1,35	0,34	100,00
69,06	11,51	12,95	0,72	5,04	0,72	100,00
65,15	10,61	20,45	0,38	3,03	0,38	100,00
66,02	3,88	24,27	0,00	5,83	0,00	100,00

59,66	6,55	26,55	0,00	7,24	0,00	100,00
64,92	4,59	23,28	0,33	5,90	0,98	100,00
62,73	2,58	27,68	0,37	6,27	0,37	100,00
59,62	0,00	35,13	0,00	3,48	1,58	100,00
50,85	2,82	34,66	0,57	10,80	0,00	100,00
64,55	0,37	34,70	0,00	0,37	0,00	100,00
77,30	0,00	8,59	0,00	13,50	0,61	100,00
75,57	0,33	24,10	0,00	0,00	0,00	99,67
68,50	1,18	23,62	0,39	5,91	0,39	100,00
75,86	0,99	19,70	0,00	3,45	0,00	100,00
76,32	1,13	19,17	0,00	3,38	0,00	100,00
72,05	0,79	19,69	0,00	7,48	0,00	100,00
43,81	28,43	24,75	0,00	3,01	0,00	100,00
64,86	13,18	17,57	0,34	4,05	0,00	100,00
70,03	10,44	16,84	0,00	2,69	0,00	100,00
73,66	9,47	13,99	0,00	2,88	0,00	100,00
65,45	9,30	19,93	0,00	4,65	0,66	87,04
67,67	7,52	22,18	0,38	2,26	0,00	100,00
63,23	12,11	22,42	0,00	1,79	0,45	100,00
77,44	4,38	15,15	0,34	2,02	0,67	99,66
70,30	6,44	21,29	0,00	1,49	0,50	100,00
70,54	7,14	20,18	0,00	1,35	0,45	100,00
71,38	11,95	14,15	0,00	2,52	0,00	100,00
59,53	15,25	19,94	0,29	4,99	0,00	100,00
56,75	13,84	25,26	0,00	3,81	0,35	100,00
51,15	10,92	27,01	0,00	9,20	1,72	100,00
44,01	1,06	33,94	0,00	19,34	0,00	100,00
53,59	1,44	29,19	0,00	15,31	0,48	100,00
47,96	4,08	23,47	0,00	23,47	1,02	100,00
58,19	0,56	19,77	0,00	21,47	0,00	100,00
57,99	1,39	34,72	0,35	4,86	0,69	100,00
80,00	1,67	12,78	0,00	5,00	0,56	100,00
67,94	0,00	25,40	0,32	5,71	0,63	100,00
68,86	2,42	22,49	0,00	6,23	0,00	99,65
72,59	14,47	10,66	0,51	1,52	0,25	99,75
83,67	6,12	8,72	0,74	0,74	0,00	100,00
52,02	2,02	23,65	3,29	17,07	0,00	100,00
69,18	0,00	27,80	0,00	0,68	0,00	100,00
75,19	0,75	21,80	0,00	2,26	0,00	100,00
74,38	8,13	15,63	0,00	1,88	0,00	100,00
75,24	3,58	17,26	0,00	3,91	0,00	100,00
69,88	10,84	15,22	0,00	1,24	0,31	99,69
82,13	5,02	11,60	0,00	1,25	0,00	100,00
74,01	7,58	16,61	1,08	0,72	0,00	100,00
60,13	4,58	24,84	0,65	8,50	1,31	100,00
74,39	1,75	20,70	0,00	3,16	0,00	100,00
67,11	8,05	14,77	0,00	9,40	0,67	100,00
50,00	0,69	27,10	0,00	16,41	0,38	100,00
70,42	2,50	24,17	0,00	2,92	0,00	100,00
72,16	0,00	25,10	0,39	1,96	0,39	100,00

67,29	0,00	30,08	0,00	2,63	0,00	100,00
65,94	0,00	31,00	0,44	2,18	0,44	100,00
89,71	0,41	9,05	0,00	0,82	0,00	100,00
85,26	0,40	9,96	0,00	4,38	0,00	100,00
44,25	4,60	42,24	0,00	4,97	0,00	100,00
61,15	1,91	28,03	0,00	8,28	0,64	100,00
51,70	1,36	32,76	0,68	10,92	2,39	100,00
65,58	0,00	27,27	0,00	6,49	0,65	100,00
42,25	0,00	40,21	0,71	13,88	2,49	100,00
59,09	0,45	30,59	0,91	8,22	0,46	100,00
54,25	1,42	42,38	0,00	1,43	0,00	99,52
58,51	1,06	27,27	0,53	11,76	0,53	100,00
19,17	1,67	7,69	0,00	25,64	2,56	100,00
59,16	6,49	28,24	0,38	5,73	0,00	100,00
74,75	3,99	19,60	0,33	1,00	0,33	99,67
60,69	0,69	25,18	0,00	10,79	0,00	100,00
47,55	31,60	18,40	0,00	2,15	0,31	100,00
47,81	0,58	40,06	0,88	10,23	0,29	99,71
69,20	0,72	29,71	0,00	0,36	0,00	100,00
71,15	0,32	18,27	0,64	7,69	1,92	99,68
61,90	1,19	29,34	1,20	4,19	1,80	100,00
61,69	3,05	29,15	0,34	4,75	1,02	100,00
59,05	6,47	28,02	0,00	4,74	1,72	100,00
57,24	13,13	23,55	0,34	4,44	0,34	99,66
52,76	0,00	40,94	0,79	5,51	0,00	100,00
48,34	31,28	17,06	0,95	2,37	0,00	100,00
71,09	10,88	16,33	0,00	1,36	0,34	100,00
60,78	0,00	29,41	3,92	3,92	1,96	100,00
68,69	1,01	1,01	0,00	2,02	27,27	98,99
71,97	0,35	21,60	0,00	5,57	0,00	98,26
68,29	1,05	26,48	0,00	4,18	0,00	100,00
66,44	1,68	29,87	0,34	1,68	0,00	100,00
36,26	17,18	40,61	0,00	5,75	0,00	100,00
54,75	3,17	33,33	1,00	1,99	0,00	100,00
60,90	0,38	37,22	0,00	1,50	0,00	99,25
67,52	0,00	27,39	0,00	5,10	0,00	100,00
60,14	7,09	26,78	0,00	5,76	0,00	100,00
45,33	0,00	52,00	2,67	0,00	0,00	100,00
51,35	4,50	37,84	0,00	5,41	0,90	100,00
56,22	20,40	18,41	0,00	4,98	0,00	99,50
0,00	0,00	0,00	0,00	0,00	0,00	0,00
85,58	0,00	13,02	0,00	1,40	0,00	100,00
0,00	0,00	0,00	0,00	0,00	0,00	0,00
81,74	0,83	17,43	0,00	0,00	0,00	100,00
56,74	0,00	17,14	0,00	25,14	0,00	100,00
61,44	1,31	16,67	0,00	20,26	0,33	100,00
52,80	32,61	12,73	0,00	1,86	0,00	100,00
82,53	4,83	9,54	0,00	0,00	0,76	100,00
71,86	9,19	17,11	0,00	1,69	0,00	100,00
62,22	4,44	31,11	0,00	2,22	0,00	100,00

69,19	1,62	27,57	0,00	1,62	0,00	100,00
73,23	0,32	17,74	1,29	7,42	0,00	100,00
72,73	2,27	22,73	0,00	2,27	0,00	97,73
53,85	1,40	23,78	3,50	17,13	0,35	100,00
48,41	6,36	43,11	0,00	2,12	0,00	100,00
50,57	5,70	33,46	0,38	9,51	0,38	100,00
28,57	4,29	45,45	0,00	0,53	17,11	100,00
55,31	9,65	32,04	0,32	1,62	0,65	100,00
61,36	3,03	27,27	0,00	6,82	1,52	100,00
70,48	0,48	17,14	0,00	11,90	0,00	100,00
79,35	0,00	19,03	0,00	1,62	0,00	100,00
69,13	1,30	17,83	0,00	11,30	0,43	100,00
77,14	4,90	13,88	0,00	4,08	0,00	99,59
64,92	15,71	15,71	0,00	3,66	0,00	100,00
60,77	3,85	26,92	0,77	6,15	1,54	100,00
57,35	1,47	32,35	1,47	7,35	0,00	100,00
65,37	6,06	20,35	0,00	7,79	0,43	100,00
38,10	8,33	30,95	0,00	22,62	0,00	100,00
60,00	1,67	20,00	0,00	13,33	5,00	100,00
67,65	7,06	20,71	0,00	4,14	0,00	100,00
53,37	23,08	20,67	0,00	2,88	0,00	100,00
29,86	2,78	12,50	47,22	4,17	3,47	100,00
53,68	9,47	29,47	0,00	7,37	0,00	100,00
58,33	15,28	20,83	0,00	2,78	2,78	100,00
69,43	12,74	15,92	0,00	1,91	0,00	100,00
65,92	10,06	21,79	0,00	2,23	0,00	100,00
92,50	0,50	3,50	3,50	0,00	0,00	100,00
61,73	0,62	27,16	0,00	10,49	0,00	100,00
77,78	3,70	14,81	0,00	3,70	0,00	100,00
48,00	0,00	30,67	1,33	20,00	0,00	100,00
63,49	1,59	27,42	0,00	6,45	0,00	98,39
41,32	20,83	32,17	0,70	4,55	0,00	99,30
38,79	12,73	16,67	0,00	28,85	0,00	100,00
0,00	0,00	0,00	0,00	0,00	0,00	0,00
42,31	24,62	28,46	0,77	3,85	0,00	100,00
62,07	6,90	30,34	0,00	0,69	0,00	100,00
27,12	45,76	27,12	0,00	0,00	0,00	100,00
69,39	9,18	20,41	0,00	1,02	0,00	100,00
66,21	17,06	11,68	1,37	2,75	0,34	100,00
52,94	5,88	32,35	0,00	5,88	2,94	100,00
45,16	8,06	43,55	0,00	3,23	0,00	100,00
84,21	0,00	10,53	0,00	0,00	5,26	100,00
80,00	0,00	20,00	0,00	0,00	0,00	100,00
58,17	10,46	25,49	0,00	5,88	0,00	100,00
64,52	6,45	26,88	0,00	2,15	0,00	100,00
90,38	0,00	5,13	0,00	4,49	0,00	100,00
78,57	0,48	20,95	0,00	0,00	0,00	100,00
86,08	1,27	11,39	0,00	1,27	0,00	100,00
63,16	6,32	20,00	0,00	10,53	0,00	100,00
76,12	0,00	20,90	0,00	2,99	0,00	100,00

82,63	6,84	10,00	0,00	0,53	0,00	100,00
42,13	5,06	30,34	0,00	21,35	1,12	100,00
61,38	0,00	35,98	0,53	1,59	0,53	100,00
70,83	14,58	12,50	0,00	2,08	0,00	100,00
75,00	0,00	10,00	0,00	5,00	10,00	100,00
78,72	0,00	12,77	0,00	8,51	0,00	100,00
56,90	0,00	29,31	0,00	13,79	0,00	100,00
62,89	5,66	25,16	0,00	6,29	0,00	98,74
59,21	6,58	23,68	0,00	10,53	0,00	98,68
40,00	13,33	40,00	0,00	6,67	0,00	100,00
41,67	0,00	50,00	0,00	8,33	0,00	100,00
0,00	0,00	0,00	0,00	0,00	0,00	0,00
55,17	1,38	31,94	0,69	10,42	0,00	100,00
71,11	8,33	19,44	0,56	0,56	0,00	100,00
70,00	0,00	10,00	0,00	20,00	0,00	100,00
48,73	16,53	22,88	0,00	11,44	0,42	100,00
58,62	24,14	9,20	3,45	3,45	1,15	98,85
70,92	0,00	10,64	0,00	12,77	5,67	97,16
37,04	44,44	16,67	0,00	1,85	0,00	100,00
72,54	2,82	22,54	0,00	2,11	0,00	100,00
59,49	0,84	24,05	0,00	14,77	0,84	100,00
61,18	0,00	14,47	0,00	24,34	0,00	100,00
68,89	17,78	11,67	0,00	1,67	0,00	100,00
0,00	0,00	0,00	0,00	0,00	0,00	0,00
75,00	0,00	25,00	0,00	0,00	0,00	100,00
64,04	1,12	34,83	0,00	0,00	0,00	100,00
89,47	0,38	8,27	0,00	1,88	0,00	100,00
88,94	0,46	9,68	0,00	0,92	0,00	100,00
36,17	0,00	57,45	0,00	6,38	0,00	100,00
76,00	8,00	12,00	0,00	4,00	0,00	100,00
54,44	8,33	30,56	0,00	6,67	0,00	100,00
42,06	6,35	42,45	0,41	2,04	5,31	99,59
55,07	12,16	24,75	0,68	6,78	0,34	99,66
54,44	10,04	29,73	0,00	4,63	1,16	100,00
57,96	10,18	29,65	0,44	1,77	0,00	100,00
50,94	3,75	39,70	0,00	5,62	0,00	100,00
57,79	8,54	31,16	0,00	2,51	0,00	99,50
58,57	7,57	29,15	0,00	3,24	0,40	100,00
49,53	8,83	30,97	0,32	8,71	0,32	100,00
0,00	0,00	0,00	0,00	0,00	0,00	0,00
55,79	9,79	27,60	0,30	6,23	0,30	100,00
73,68	0,40	21,14	0,00	4,47	0,00	100,00
63,21	9,43	25,47	0,00	1,42	0,47	100,00
82,28	0,00	9,74	0,65	5,19	0,00	100,00
72,38	3,33	13,33	0,00	9,52	1,43	100,00
74,30	4,82	17,27	0,00	1,61	2,01	99,60
73,49	3,72	14,01	0,97	4,83	0,00	100,00
86,52	0,00	12,14	0,00	0,71	0,00	100,00
91,70	0,00	6,99	0,00	1,31	0,00	100,00
69,01	3,39	25,26	0,00	2,34	0,00	100,00

0,00	0,00	0,00	0,00	0,00	0,00	0,00
42,07	0,69	43,06	0,00	4,86	9,03	99,31
45,73	1,22	43,56	0,00	9,20	0,00	100,00
52,91	2,43	36,27	0,00	4,66	0,00	100,00
59,64	0,71	23,30	0,36	15,77	0,00	100,00
73,09	0,92	21,78	0,31	3,37	0,31	100,00
59,17	0,42	26,25	8,75	5,42	0,00	100,00
66,55	2,07	23,10	0,34	7,93	0,00	100,00
72,34	5,85	18,62	0,00	3,19	0,00	100,00
63,22	0,38	28,74	0,77	6,90	0,00	100,00
59,38	0,52	26,04	1,04	13,02	0,00	100,00
62,12	0,00	27,99	0,00	8,87	1,02	100,00
57,96	0,60	32,73	0,00	8,71	0,00	100,00
58,36	0,71	19,22	0,36	21,35	0,00	100,00
62,33	0,00	17,67	0,47	19,53	0,00	100,00
49,68	2,56	28,85	0,00	17,63	1,28	100,00
69,06	0,00	24,02	0,56	4,47	1,12	99,44
49,40	0,00	38,10	1,19	5,95	5,36	100,00
56,63	3,61	28,92	0,00	7,63	3,21	100,00
57,89	9,77	18,22	0,00	12,02	0,00	99,61
57,46	12,71	24,86	0,00	4,97	0,00	100,00
45,98	19,20	31,25	0,00	3,57	0,00	100,00
66,98	9,91	20,75	0,00	2,36	0,00	100,00
66,27	2,78	18,65	0,00	11,51	0,79	100,00
70,54	3,73	21,76	0,00	3,35	0,00	100,00
65,74	5,56	23,15	0,00	5,56	0,00	100,00
73,06	3,32	19,93	0,37	3,32	0,00	100,00
55,31	7,82	30,17	0,56	6,15	0,00	100,00
68,14	6,78	17,29	0,00	7,12	0,68	100,00
63,95	3,10	25,58	2,33	5,04	0,00	100,00
57,97	2,42	30,92	0,00	8,70	0,00	100,00
50,00	4,67	18,95	0,00	3,92	0,65	100,00
69,61	4,24	23,67	0,35	1,41	0,71	100,00
64,03	3,60	21,30	0,72	10,11	0,00	100,00
73,29	13,01	12,41	0,00	0,69	0,00	100,00
72,04	2,69	16,13	0,00	9,14	0,00	100,00
67,38	3,23	25,09	0,00	3,58	0,72	100,00
46,81	1,60	30,81	0,00	15,70	0,58	100,00
73,48	2,51	16,19	0,00	4,68	2,88	100,00
37,29	1,69	54,17	0,60	2,38	1,79	100,00
65,65	5,34	25,95	0,00	3,05	0,00	100,00
74,43	5,68	17,05	0,00	1,70	1,14	100,00
45,41	2,42	43,78	0,00	6,97	0,00	100,00
61,99	5,14	25,00	0,00	7,53	0,34	100,00
44,00	9,33	36,00	0,00	5,33	5,33	98,67
61,18	5,88	25,88	0,59	6,47	0,00	100,00
68,00	6,67	19,72	0,00	1,41	0,00	100,00
72,45	0,75	22,22	0,00	3,45	0,00	100,00
63,75	5,94	29,06	0,00	1,25	0,00	100,00
70,61	1,35	25,85	0,00	1,70	0,00	100,00

75,35	6,51	14,88	0,00	3,26	0,00	100,00
57,69	7,69	29,33	0,48	4,33	0,48	100,00
55,74	4,51	35,06	0,43	0,87	0,00	99,57
78,91	1,36	12,93	0,00	6,12	0,68	100,00
58,88	8,63	21,32	0,00	10,66	0,51	100,00
63,35	3,14	28,80	0,00	4,71	0,00	99,48
87,37	0,00	12,63	0,00	0,00	0,00	100,00
43,59	0,00	34,62	1,28	19,23	1,28	100,00
45,62	2,55	38,32	0,00	10,95	2,55	100,00
53,98	2,65	24,00	0,33	11,33	0,33	100,00
55,78	6,27	29,04	0,00	8,25	0,66	100,00
75,00	0,43	24,14	0,00	0,43	0,00	100,00
81,82	0,45	11,82	0,00	5,91	0,00	100,00
69,97	1,10	21,24	0,00	2,06	0,59	100,00
85,38	4,62	7,69	0,00	1,92	0,38	100,00
60,00	0,63	27,94	0,32	11,11	0,00	100,00
67,52	0,57	27,92	0,00	3,99	0,00	100,00
76,49	0,00	17,24	0,00	6,27	0,00	100,00
73,39	0,61	22,32	0,00	3,36	0,31	100,00
59,57	4,26	29,79	0,00	6,38	0,00	100,00
57,41	3,04	32,70	0,38	6,46	0,00	100,00
79,37	0,79	13,10	0,00	6,35	0,40	100,00
88,78	2,04	6,83	0,34	1,71	0,00	99,66
65,68	0,42	22,03	0,42	11,44	0,00	100,00
74,38	0,00	18,18	0,41	7,02	0,00	100,00
47,81	3,86	22,37	0,00	25,45	0,51	99,74
74,58	0,42	20,42	0,00	3,75	0,83	100,00
59,31	0,69	17,24	0,00	22,76	0,00	100,00
73,68	0,00	19,44	0,00	2,78	0,00	100,00
84,57	1,14	9,14	0,00	4,00	1,14	98,86
72,25	0,00	25,13	0,00	2,62	0,00	100,00
78,08	0,77	16,54	0,38	3,85	0,38	100,00
58,45	7,16	23,50	0,57	10,32	0,00	100,00
55,88	1,10	37,88	0,00	3,41	0,00	100,00
77,86	1,43	12,23	0,00	7,91	0,00	100,00
86,47	0,00	5,26	0,00	8,27	0,00	99,25
65,74	10,76	14,34	0,00	8,76	0,40	100,00
55,89	15,49	20,54	1,35	6,73	0,00	100,00
57,63	10,85	27,46	1,02	3,05	0,00	100,00
48,89	5,33	43,56	0,44	1,78	0,00	100,00
64,35	5,68	26,50	0,32	3,15	0,00	100,00
53,05	11,83	27,86	0,38	6,87	0,00	100,00
73,48	4,47	19,49	0,00	2,24	0,32	100,00
56,44	9,24	26,73	0,33	7,26	0,00	100,00
61,69	9,58	27,20	0,38	1,15	0,00	100,00
66,67	1,96	28,76	0,00	2,61	0,00	100,00
67,42	3,41	27,27	0,00	1,89	0,00	100,00
53,33	7,62	32,38	0,00	6,19	0,48	100,00
68,24	0,00	26,47	0,00	4,12	1,18	100,00
60,00	8,98	22,45	0,00	6,94	1,63	100,00

68,66	5,47	22,39	0,00	2,99	0,50	100,00
54,46	5,63	30,05	0,00	6,57	3,29	100,00
61,11	6,06	24,75	0,51	7,07	0,51	99,49
59,32	6,52	29,84	0,32	2,22	0,32	100,00
57,89	2,63	31,58	0,00	7,89	0,00	100,00
57,14	0,00	28,57	0,00	14,29	0,00	100,00
88,00	0,00	4,00	4,00	4,00	0,00	100,00
48,10	1,27	41,77	0,00	8,86	0,00	100,00
60,00	0,00	35,00	5,00	0,00	0,00	100,00
0,00	0,00	0,00	0,00	0,00	0,00	0,00
0,00	0,00	0,00	0,00	0,00	0,00	0,00
80,00	0,00	7,50	0,00	12,50	0,00	100,00
0,00	0,00	0,00	0,00	0,00	0,00	0,00
53,03	0,00	34,85	0,00	12,12	0,00	98,48
79,67	0,00	17,58	0,00	2,75	0,00	100,00
66,67	0,00	26,67	0,00	6,67	0,00	100,00
85,00	0,00	0,00	0,00	0,00	15,00	95,00
0,00	0,00	0,00	0,00	0,00	0,00	0,00
86,30	1,37	9,59	1,37	1,37	0,00	97,26
31,35	36,76	28,65	0,00	3,24	0,00	100,00
73,21	0,89	10,71	2,68	12,50	0,00	99,11
50,68	1,37	45,21	0,00	2,74	0,00	100,00
57,81	4,69	27,08	0,00	10,42	0,00	100,00
85,71	1,59	12,70	0,00	0,00	0,00	100,00
85,71	0,00	13,10	0,00	1,19	0,00	100,00
75,00	1,25	23,75	0,00	0,00	0,00	98,75
60,26	5,13	28,57	0,00	5,19	0,00	100,00
71,13	14,08	14,79	0,00	0,00	0,00	100,00
57,36	0,00	32,56	0,00	9,30	0,78	100,00
77,71	0,00	20,48	0,60	1,20	0,00	100,00
61,29	3,23	19,35	3,23	6,45	6,45	100,00
92,00	0,00	4,00	0,00	4,00	0,00	100,00
59,38	0,00	35,42	2,08	3,13	0,00	100,00
69,43	0,00	21,02	1,27	8,28	0,00	100,00
54,40	0,00	28,34	1,30	15,96	0,00	100,00
62,08	1,67	27,92	0,00	8,33	0,00	99,58
73,62	0,00	20,43	0,00	5,53	0,43	100,00
69,70	0,00	25,76	1,52	3,03	0,00	100,00
60,51	0,00	19,75	1,27	18,47	0,00	99,36
68,75	0,00	18,75	0,00	12,50	0,00	100,00
70,29	0,72	17,39	0,00	11,59	0,00	100,00
52,00	4,00	18,00	0,00	26,00	0,00	100,00
0,00	0,00	0,00	0,00	0,00	0,00	0,00
88,64	0,00	11,36	0,00	0,00	0,00	100,00
69,57	0,72	13,04	0,00	16,67	0,00	100,00
63,80	3,07	17,79	0,00	15,34	0,00	100,00
63,42	10,03	22,96	0,60	1,21	0,00	100,00
68,18	4,55	23,64	0,00	3,18	0,45	100,00
72,00	1,23	20,62	0,00	6,15	0,00	100,00
68,29	1,63	29,27	0,00	0,81	0,00	100,00

57,54	10,15	29,54	0,31	1,23	1,23	99,69
0,00	0,00	0,00	0,00	0,00	0,00	0,00
55,82	0,80	32,93	0,00	10,44	0,00	100,00
62,93	0,49	33,17	0,00	3,41	0,00	100,00
70,43	1,99	23,92	0,33	2,66	0,66	99,34
46,01	0,31	33,54	0,31	19,08	0,62	99,69
63,73	0,35	33,45	0,00	1,76	0,70	100,00
77,35	0,32	19,33	0,00	0,33	0,33	99,67
71,53	0,68	22,03	0,34	5,42	0,00	100,00
69,76	0,40	21,77	0,00	7,26	0,81	100,00
71,83	1,41	24,65	0,70	1,41	0,00	99,30
44,40	0,72	46,93	0,36	2,89	4,69	100,00
61,42	6,30	27,56	0,00	4,72	0,00	100,00
66,14	0,00	21,34	0,40	11,46	0,40	100,00
67,19	3,79	16,09	0,00	11,67	1,26	99,68
66,57	1,17	26,18	0,00	5,88	0,00	100,00
64,31	1,86	30,86	0,00	2,97	0,00	100,00
58,00	2,00	32,00	0,00	8,00	0,00	100,00
60,09	2,29	26,40	0,00	4,57	0,00	100,00
47,66	6,64	41,20	0,40	2,00	0,80	100,00
76,62	0,50	20,40	0,00	2,49	0,00	100,00
56,92	4,23	28,08	0,38	10,00	0,38	100,00
60,74	2,07	33,88	0,83	2,48	0,00	100,00
55,00	0,00	33,00	0,00	7,00	5,00	100,00
63,16	8,77	26,32	0,44	1,32	0,00	100,00
57,79	8,75	30,80	0,38	2,28	0,00	100,00
69,77	5,43	23,26	0,00	1,55	0,00	100,00
81,82	9,09	0,00	0,00	9,09	0,00	100,00
70,62	1,13	20,34	0,00	7,91	0,00	100,00
73,18	3,31	22,19	0,00	0,99	0,33	100,00
65,09	6,90	22,81	0,88	3,07	0,00	100,00
46,22	25,33	26,22	0,00	2,22	0,00	100,00
61,76	5,88	17,65	0,00	14,71	0,00	100,00
56,93	1,46	26,28	0,00	14,60	0,73	100,00
70,38	1,92	18,85	0,38	8,46	0,00	99,62
54,30	24,89	16,55	0,23	3,40	0,45	100,00
65,52	0,00	27,59	3,45	0,00	3,45	100,00
55,23	0,42	34,31	0,42	9,62	0,00	100,00
39,30	10,45	35,82	6,47	7,46	0,50	99,50
50,91	23,03	20,00	0,00	6,06	0,00	99,39
79,39	4,58	7,03	0,00	7,03	0,00	100,00
90,98	0,00	8,27	0,00	0,75	0,00	100,00
45,27	34,91	17,23	0,23	1,36	0,45	100,00
68,06	2,28	27,38	0,00	2,28	0,00	100,00
63,76	1,83	30,28	1,38	2,29	0,46	100,00
51,48	23,96	23,08	0,00	1,48	0,00	100,00
60,83	16,25	22,92	0,00	0,00	0,00	100,00
69,42	0,00	21,21	0,00	6,57	0,00	99,49
52,22	21,84	24,23	0,00	1,71	0,00	100,00
27,46	54,40	17,62	0,52	0,00	0,00	100,00

31,05	44,44	23,71	0,00	0,57	0,00	100,00
65,97	1,74	23,96	0,35	2,78	5,21	98,96
68,56	2,17	19,78	0,54	8,94	0,00	100,00
21,15	49,10	11,11	2,15	16,49	0,00	99,64
75,24	3,86	16,40	0,96	3,54	0,00	100,00
67,31	0,00	25,40	0,00	7,07	0,00	100,00
66,67	11,39	15,61	0,00	5,91	0,42	99,58
64,43	0,79	22,53	0,00	12,25	0,00	100,00
66,34	14,24	14,24	0,00	3,24	1,94	99,68
69,41	0,29	22,06	0,00	2,94	5,29	97,94
62,26	17,42	16,13	0,00	4,19	0,00	100,00
56,16	25,68	17,12	0,00	1,03	0,00	100,00
84,40	0,00	5,50	0,00	10,09	0,00	100,00
82,09	1,00	12,44	0,00	4,48	0,00	100,00
74,66	1,37	17,24	0,00	6,21	0,00	100,00
82,99	1,02	10,88	0,00	5,10	0,00	100,00
73,33	2,96	16,30	0,00	7,41	0,00	100,00
57,74	0,42	26,36	0,00	15,06	0,42	99,58
36,41	32,26	23,94	0,94	5,16	0,00	99,06
52,17	0,72	29,71	0,00	16,67	0,72	100,00
65,50	1,31	16,16	0,00	16,59	0,44	100,00
72,87	0,78	18,22	0,00	8,14	0,00	100,00
76,11	0,88	15,04	0,00	7,96	0,00	100,00
65,71	0,95	26,67	0,48	6,19	0,00	99,52
91,94	0,00	6,45	0,00	1,61	0,00	100,00
81,82	0,65	4,61	0,00	11,84	0,00	100,00
66,88	0,00	21,15	0,64	10,90	0,00	100,00
91,20	0,00	1,60	0,00	7,20	0,00	100,00
75,82	0,00	16,73	0,00	6,32	0,00	98,51
60,17	0,28	21,45	0,00	18,11	0,00	100,00
50,94	1,26	21,70	0,31	25,79	0,00	97,17
50,34	2,29	21,05	0,00	25,40	0,92	99,54
68,46	0,00	26,54	0,00	4,62	0,38	99,62
66,67	0,51	23,48	0,25	9,09	0,00	100,00
61,30	0,38	32,95	0,00	5,36	0,00	99,62
71,43	0,62	21,12	0,00	6,83	0,00	100,00
52,38	0,43	29,00	0,87	17,32	0,00	100,00
64,98	0,00	30,35	0,00	4,67	0,00	100,00
53,51	0,74	29,15	0,74	15,87	0,00	99,63
61,54	0,00	28,44	0,00	10,02	0,00	99,77
74,27	0,00	24,27	0,97	0,49	0,00	99,51
50,78	23,05	22,35	0,78	2,75	0,00	100,00
67,25	0,00	22,65	0,00	9,76	0,35	100,00
69,96	1,83	23,44	0,73	3,66	0,37	100,00
73,86	0,00	22,22	0,00	3,92	0,00	100,00
57,99	1,23	31,53	0,00	9,11	0,00	99,51
67,96	0,28	23,76	0,00	8,01	0,00	99,72
70,53	0,46	20,82	0,00	5,08	0,00	99,76
69,09	2,52	23,66	0,00	4,73	0,00	99,68
55,56	0,44	29,78	0,44	13,78	0,00	100,00

64,09	0,55	29,83	0,00	5,52	0,00	99,45
54,33	2,77	28,37	0,35	14,19	0,00	100,00
55,90	0,44	21,40	0,00	22,27	0,00	99,13
62,79	5,58	21,03	0,47	9,81	0,00	100,00
34,34	42,64	16,23	0,00	6,42	0,38	100,00
51,97	0,79	36,61	0,00	10,63	0,00	99,61
66,08	6,71	25,80	0,35	1,06	0,00	100,00
63,72	2,74	24,39	0,00	9,15	0,00	100,00
71,01	0,33	27,45	0,65	0,33	0,00	99,02
63,39	0,68	22,79	1,70	11,22	0,00	97,96
67,04	4,07	25,56	0,00	3,33	0,00	100,00
55,69	2,10	23,35	0,00	18,86	0,00	99,70
67,91	3,74	14,97	2,67	10,70	0,00	100,00
30,92	46,05	20,39	0,00	1,97	0,66	99,67
81,92	7,38	10,70	0,00	0,00	0,00	100,00
81,73	9,30	7,31	0,00	0,66	1,00	100,00
69,07	11,00	16,49	1,37	2,06	0,00	100,00
81,63	4,08	10,61	0,82	2,86	0,00	100,00
87,95	2,41	9,04	0,00	0,60	0,00	100,00
75,54	10,87	13,59	0,00	0,00	0,00	100,00
89,72	3,90	6,03	0,00	0,35	0,00	100,00
85,22	6,09	6,96	0,00	1,74	0,00	100,00
51,67	32,06	14,35	0,00	1,44	0,48	100,00
77,23	8,91	9,90	0,00	3,63	0,33	100,00
65,19	1,27	25,48	0,64	7,01	0,00	100,00
67,84	1,01	21,61	0,00	9,05	0,50	100,00
72,11	1,36	20,41	0,00	6,12	0,00	100,00
66,34	0,00	26,73	0,00	6,93	0,00	100,00
67,72	0,63	19,62	1,27	10,76	0,00	100,00
69,19	0,54	20,54	0,00	9,73	0,00	100,00
66,67	0,00	25,93	0,00	3,70	3,70	100,00
0,00	0,00	0,00	0,00	0,00	0,00	0,00
97,06	2,94	0,00	0,00	0,00	0,00	100,00
82,35	1,96	9,80	0,00	5,88	0,00	100,00
77,91	0,00	9,30	0,00	12,79	0,00	100,00
100,00	0,00	0,00	0,00	0,00	0,00	100,00
80,26	1,32	16,00	0,00	1,33	0,00	100,00
62,95	1,64	21,64	0,33	13,44	0,00	99,67
66,55	1,69	27,03	1,01	3,72	0,00	100,00
54,23	0,35	30,04	0,71	14,49	0,00	100,00
54,09	1,56	21,09	0,00	23,05	0,00	99,61
55,05	0,92	27,06	0,92	16,06	0,00	100,00
55,56	0,40	28,97	1,19	13,49	0,40	99,60
69,76	0,00	19,02	0,98	10,24	0,00	99,02
70,59	0,84	15,13	0,00	13,45	0,00	99,16
64,63	1,83	17,99	0,00	13,41	2,13	100,00
65,06	0,00	16,87	0,00	18,07	0,00	100,00
82,22	0,00	16,67	0,00	1,11	0,00	100,00
64,63	1,36	11,56	0,00	21,77	0,68	100,00
81,16	3,62	10,87	0,00	4,35	0,00	100,00

69,14	0,78	26,56	0,00	3,52	0,00	100,00
62,16	0,00	29,73	0,54	7,03	0,54	99,46
0,00	0,00	0,00	0,00	0,00	0,00	0,00
73,24	3,24	14,32	0,81	7,30	1,08	99,19
56,72	2,24	32,84	0,00	8,21	0,00	100,00
76,35	2,99	20,06	0,30	0,30	0,00	99,70
65,32	2,70	30,18	0,00	1,80	0,00	100,00
71,79	0,00	28,21	0,00	0,00	0,00	100,00
74,55	0,00	24,85	0,00	0,61	0,00	100,00
79,32	0,38	19,17	0,38	0,75	0,00	99,62
68,32	0,38	29,01	0,38	1,91	0,00	99,62
97,52	0,00	2,48	0,00	0,00	0,00	100,00
96,57	0,00	3,43	0,00	0,00	0,00	100,00
93,51	0,00	5,95	0,00	0,54	0,00	100,00
78,07	0,00	15,24	0,00	6,69	0,00	100,00
46,86	0,33	38,57	0,36	10,00	0,00	97,50
55,83	3,53	35,69	0,00	3,89	1,06	84,81
55,06	23,89	16,19	0,00	4,86	0,00	100,00
61,93	18,73	14,20	0,30	3,32	1,51	100,00
71,11	1,85	21,11	0,00	5,19	0,74	99,63
60,53	11,40	26,75	0,44	0,44	0,44	100,00
66,25	5,83	23,75	0,00	3,75	0,42	100,00
69,05	7,94	21,43	0,40	1,19	0,00	100,00
79,58	0,00	16,32	0,00	3,68	0,00	100,00
80,19	0,94	9,43	0,00	8,49	0,94	100,00
80,77	0,85	8,55	0,00	9,83	0,00	100,00
84,90	1,01	7,38	0,00	5,03	1,68	99,33
68,22	0,85	18,64	0,00	10,17	2,12	99,15
58,43	1,12	24,16	0,56	13,48	2,25	98,31
57,39	0,69	29,21	0,00	12,71	0,00	99,66
72,22	0,93	23,83	0,00	1,40	0,93	100,00
62,50	3,33	29,17	0,00	3,75	1,25	99,58
67,84	0,00	20,96	0,00	3,49	0,00	100,00
54,75	0,76	35,74	0,38	7,98	0,38	100,00
72,73	0,00	24,60	0,00	2,67	0,00	100,00
40,59	8,42	35,15	0,00	12,87	2,97	100,00
69,90	0,97	19,42	0,00	9,71	0,00	100,00
66,00	0,00	20,00	0,40	13,60	0,00	99,60
51,14	5,02	28,77	0,00	15,07	0,00	100,00
74,19	0,00	20,97	0,00	4,84	0,00	100,00
57,41	3,70	18,52	0,00	20,37	0,00	100,00
55,95	1,98	38,89	0,00	3,17	0,00	100,00
60,89	0,37	34,32	0,37	3,32	0,74	100,00
47,81	1,82	40,74	0,74	8,15	0,00	100,00
42,13	0,46	45,19	0,00	10,58	0,00	100,00
90,70	1,16	5,04	0,00	3,10	0,00	100,00
64,71	17,65	17,65	0,00	0,00	0,00	100,00
81,07	0,95	8,20	0,00	9,15	0,63	99,37
79,05	0,79	7,11	0,00	12,25	0,79	99,60
75,23	0,92	21,10	0,00	2,75	0,00	100,00

68,84	8,70	18,48	0,00	3,99	0,00	100,00
71,01	1,81	25,36	1,09	0,72	0,00	99,64
55,92	5,92	27,95	0,00	8,42	0,34	100,00
49,70	0,59	44,31	1,20	1,20	2,40	100,00
87,83	1,74	10,43	0,00	0,00	0,00	100,00
80,86	1,44	14,35	0,00	3,35	0,00	100,00
56,47	1,29	18,53	0,43	11,21	12,07	100,00
93,71	0,00	4,00	0,00	2,29	0,00	100,00
83,12	0,84	10,55	0,00	5,49	0,00	100,00
44,77	1,16	48,84	0,00	5,23	0,00	100,00
77,86	0,00	11,45	0,00	10,69	0,00	100,00
44,65	2,52	39,49	0,00	12,74	0,00	100,00
54,12	4,71	40,39	0,00	0,78	0,00	100,00
45,79	6,40	38,72	0,00	9,09	0,00	99,66
52,96	1,48	32,96	0,37	12,22	0,00	100,00
59,85	6,82	24,62	0,00	6,15	1,54	100,00
40,14	1,41	45,07	0,00	13,38	0,00	100,00
47,22	6,35	35,02	0,92	1,84	0,00	100,00
42,71	3,65	40,10	0,00	10,94	2,60	100,00
39,68	2,78	51,81	0,40	4,02	0,80	99,60
33,78	0,00	58,33	0,00	5,56	1,39	100,00
47,39	0,00	49,76	0,48	0,48	0,97	100,00
58,40	13,87	22,69	0,00	5,04	0,00	100,00
67,07	2,40	23,95	0,00	5,99	0,60	100,00
44,59	4,46	28,26	0,72	12,32	2,90	100,00
48,77	16,67	21,71	3,29	3,29	1,97	100,00
63,79	4,12	22,22	0,82	9,05	0,00	100,00
57,21	0,47	35,85	0,47	4,25	0,94	99,53
59,06	0,67	33,78	0,68	5,41	0,00	100,00
54,11	3,03	38,70	0,43	3,48	0,00	100,00
61,80	0,43	34,33	0,00	3,43	0,00	100,00
65,98	3,28	26,45	0,83	2,89	0,00	100,00
64,29	6,02	26,14	0,00	2,65	0,38	100,00
62,30	6,97	26,03	0,00	2,89	1,24	100,00
54,42	1,40	40,47	0,00	1,40	2,33	100,00
54,95	1,02	10,58	2,73	24,91	5,80	100,00
56,22	1,72	35,81	0,00	0,93	0,47	100,00
52,80	12,80	28,40	0,00	6,00	0,00	100,00
61,27	17,25	16,20	0,00	5,28	0,00	100,00
64,25	7,82	25,70	0,00	2,23	0,00	100,00
45,45	0,00	54,55	0,00	0,00	0,00	100,00
50,00	0,00	7,69	0,00	19,23	23,08	100,00
57,14	7,14	28,57	0,00	7,14	0,00	100,00
59,85	11,36	22,73	0,00	6,06	0,00	100,00
66,30	7,61	22,28	0,00	3,26	0,54	100,00
53,68	15,15	26,20	0,00	2,18	2,18	100,00
46,96	24,31	26,82	0,00	1,12	0,00	100,00
62,69	0,52	32,64	0,00	4,15	0,00	100,00
54,24	10,17	27,54	0,42	6,36	1,27	100,00
57,73	13,40	25,77	0,00	3,09	0,00	100,00

54,90	11,76	27,45	0,00	5,88	0,00	3,92
71,72	7,07	18,18	0,00	3,03	0,00	100,00
0,00	0,00	0,00	0,00	0,00	0,00	0,00
44,16	1,30	23,91	0,00	0,00	0,00	100,00
60,00	0,00	16,00	0,00	24,00	0,00	100,00
54,55	2,02	28,57	0,00	6,12	8,16	100,00
27,36	37,74	33,02	1,89	0,00	0,00	99,06
75,56	0,00	20,00	0,00	4,44	0,00	100,00
66,67	0,00	11,11	0,00	22,22	0,00	100,00
23,53	5,88	29,41	0,00	41,18	0,00	100,00
71,43	2,38	9,52	0,00	16,67	0,00	100,00
53,28	1,46	25,55	0,00	13,14	6,57	100,00
49,32	23,98	22,62	0,00	3,62	0,45	100,00
50,00	18,75	18,75	0,00	12,50	0,00	100,00
65,87	4,76	19,84	0,00	9,52	0,00	100,00
67,27	13,94	15,15	0,00	3,64	0,00	100,00
56,10	2,44	26,83	2,44	9,76	2,44	97,56
78,34	6,37	12,74	0,00	1,91	0,64	100,00
68,24	1,18	27,06	1,18	2,35	0,00	100,00
50,00	2,27	13,64	4,55	29,55	0,00	100,00
66,67	8,33	20,83	0,00	4,17	0,00	100,00
88,46	0,00	11,54	0,00	0,00	0,00	100,00
59,18	8,16	30,61	0,00	2,04	0,00	97,96
64,06	0,00	18,75	0,00	15,63	1,56	98,44
62,37	9,68	19,89	1,08	6,99	0,00	100,00
62,90	17,74	14,52	0,00	4,84	0,00	100,00
62,96	3,70	12,35	0,00	20,99	0,00	100,00
58,82	3,92	33,33	0,00	3,92	0,00	100,00
80,61	1,02	2,06	0,00	11,34	4,12	100,00
59,09	0,00	9,09	0,00	4,55	27,27	100,00
66,39	9,84	17,21	2,46	3,28	0,82	100,00
64,71	1,81	32,42	0,00	0,46	0,00	100,00
72,92	2,08	16,67	0,00	8,33	0,00	100,00
60,87	0,00	21,74	0,00	17,39	0,00	100,00
58,18	1,82	23,64	0,00	16,36	0,00	100,00
88,00	0,00	12,00	0,00	0,00	0,00	100,00
78,95	0,00	21,05	0,00	0,00	0,00	100,00
82,76	0,00	14,29	0,00	0,00	0,00	100,00
63,40	1,31	30,72	0,00	4,58	0,00	100,00
65,67	0,00	29,85	0,00	4,48	0,00	100,00
78,72	0,00	19,15	0,00	2,13	0,00	100,00
66,67	14,29	18,18	0,00	0,87	0,00	100,00
70,65	1,99	23,38	1,00	2,49	0,50	100,00
69,93	3,50	21,68	0,00	4,90	0,00	100,00
70,83	0,00	25,00	0,69	3,47	0,00	99,31
71,64	0,00	26,12	0,75	1,49	0,00	100,00
90,00	0,00	5,00	0,00	5,00	0,00	95,00
100,00	0,00	0,00	0,00	0,00	0,00	100,00
59,59	0,68	26,90	0,69	11,72	0,00	100,00
64,58	2,08	26,56	0,52	5,73	0,52	100,00

23,64	0,00	24,00	0,00	12,00	12,00	100,00
41,67	2,78	38,89	0,00	16,67	0,00	100,00
53,42	1,37	42,07	0,00	2,07	0,69	99,31
50,00	8,33	38,89	0,00	2,78	0,00	100,00
43,81	2,38	41,15	0,52	7,81	0,00	100,00
68,28	6,21	19,31	0,69	4,83	0,69	100,00
57,50	17,50	21,25	0,00	3,75	0,00	100,00
83,33	11,11	5,56	0,00	0,00	0,00	100,00
1,28	0,00	8,97	3,85	85,90	0,00	100,00
40,00	20,00	40,00	0,00	0,00	0,00	100,00
82,10	1,95	8,20	0,00	7,03	0,39	100,00
40,80	9,20	24,14	0,00	1,72	24,14	100,00
31,03	3,45	45,35	3,49	16,28	0,00	100,00
64,07	4,19	20,96	0,00	10,78	0,00	100,00
67,13	6,99	19,58	0,00	6,29	0,00	100,00
49,52	7,62	20,00	0,00	5,00	0,00	100,00
67,65	4,62	17,65	0,00	8,40	1,68	100,00
61,11	4,17	22,22	1,39	11,11	0,00	100,00
78,81	0,00	11,26	0,66	9,27	0,00	98,68
53,85	0,00	0,00	0,00	46,15	0,00	100,00
10,93	87,09	1,32	0,00	0,66	0,00	100,00
9,90	81,68	7,92	0,00	0,50	0,00	100,00
0,00	0,00	0,00	0,00	0,00	0,00	0,00
72,89	3,11	17,27	0,00	5,00	0,00	100,00
67,74	6,13	23,87	0,00	1,94	0,32	100,00
71,29	7,18	21,53	0,00	0,00	0,00	100,00
64,71	5,88	29,41	0,00	0,00	0,00	100,00
65,61	0,53	17,99	1,06	12,17	2,65	100,00
63,51	1,35	26,76	1,41	4,23	0,00	100,00
0,00	0,00	0,00	0,00	0,00	0,00	0,00
65,88	1,18	25,88	0,00	7,06	0,00	100,00
32,00	0,00	34,00	0,00	34,00	0,00	100,00
0,00	0,00	0,00	0,00	0,00	0,00	0,00
73,58	1,89	22,64	0,00	1,89	0,00	100,00
27,38	53,97	15,87	0,00	1,98	0,79	100,00
68,18	23,64	7,58	0,30	0,30	0,00	100,00
66,67	0,00	0,00	0,00	33,33	0,00	100,00
63,33	1,11	10,00	0,00	25,56	0,00	100,00
68,67	0,00	9,64	0,00	19,28	2,41	100,00
56,86	3,92	23,53	0,00	15,69	0,00	100,00
60,87	0,00	6,52	0,00	32,61	0,00	100,00
90,91	0,00	1,52	0,00	7,58	0,00	100,00
73,91	0,00	22,73	0,00	0,00	0,00	100,00
64,71	0,00	8,82	0,00	23,53	2,94	100,00
65,71	0,00	22,86	0,00	11,43	0,00	100,00
66,67	6,31	22,73	1,82	1,82	0,00	100,00
61,54	1,10	21,98	2,20	13,19	0,00	100,00
63,41	0,00	24,39	0,00	12,20	0,00	100,00
57,14	0,00	37,14	0,00	5,71	0,00	100,00
61,70	0,00	19,15	0,00	19,15	0,00	100,00

100,00	0,00	0,00	0,00	0,00	0,00	100,00
30,19	0,00	41,51	1,89	26,42	0,00	100,00
16,67	66,67	16,00	0,00	0,67	0,00	100,00
63,33	16,67	13,33	0,00	3,33	3,33	100,00
80,56	0,00	19,44	0,00	0,00	0,00	100,00
75,56	1,11	20,00	0,00	3,33	0,00	100,00
53,92	4,90	27,45	0,00	13,73	0,00	100,00
71,30	0,87	22,61	0,00	5,22	0,00	100,00
50,85	0,00	16,95	0,00	32,20	0,00	100,00
75,74	1,98	17,33	0,00	1,49	3,47	100,00
78,26	0,00	17,39	0,00	4,35	0,00	100,00
83,33	0,00	0,00	0,00	16,67	0,00	100,00
50,00	2,00	18,00	0,00	30,00	0,00	100,00
61,54	0,00	11,54	0,00	26,92	0,00	100,00
92,13	0,00	6,74	0,00	1,12	0,00	100,00
42,19	0,00	10,94	0,00	46,88	0,00	100,00
84,21	0,00	10,53	0,00	5,26	0,00	100,00
83,33	0,00	16,67	0,00	0,00	0,00	100,00
83,33	2,08	12,50	0,00	2,08	0,00	100,00
57,14	0,00	0,00	7,14	35,71	0,00	100,00
64,08	1,94	24,27	0,00	8,74	0,97	99,03
89,29	0,00	10,71	0,00	0,00	0,00	100,00
0,00	0,00	0,00	0,00	0,00	0,00	0,00
80,00	6,67	11,11	0,00	2,22	0,00	100,00
36,88	1,42	39,72	0,00	21,28	0,71	100,00
69,39	2,04	24,49	0,00	0,00	4,08	100,00
54,21	0,00	43,93	0,00	1,87	0,00	100,00
60,91	0,00	36,36	0,00	2,73	0,00	100,00
55,00	0,00	15,00	0,00	15,00	15,00	100,00
37,50	0,00	50,00	0,00	12,50	0,00	100,00
78,79	0,00	16,67	1,52	3,03	0,00	100,00
42,50	0,00	37,50	0,00	20,00	0,00	100,00
0,00	0,00	0,00	0,00	0,00	0,00	0,00
58,00	0,00	30,00	0,00	10,00	2,00	98,00
47,06	0,00	52,94	0,00	0,00	0,00	100,00
36,36	0,00	63,64	0,00	0,00	0,00	100,00
58,33	0,00	41,67	0,00	0,00	0,00	100,00
60,00	12,63	21,05	0,00	6,32	0,00	100,00
52,94	15,69	27,45	0,00	1,96	1,96	100,00
49,34	0,00	32,24	0,00	8,55	9,87	100,00
64,04	5,62	25,99	0,56	2,26	1,13	100,00
48,85	14,37	27,10	0,00	1,94	0,00	100,00
54,86	25,00	19,44	0,00	0,69	0,00	100,00
46,67	0,00	20,00	0,00	33,33	0,00	100,00
40,00	0,00	24,00	0,00	36,00	0,00	100,00
64,38	13,40	19,28	0,65	2,29	0,00	100,00
60,26	9,17	20,52	1,31	8,73	0,00	100,00
88,16	0,00	9,21	0,00	2,63	0,00	100,00
61,19	0,81	21,82	0,00	14,64	0,00	100,00
54,30	9,60	21,81	0,67	12,08	0,67	100,00

0,00	0,00	0,00	0,00	0,00	0,00	0,00
58,59	2,02	19,54	0,00	8,62	2,87	100,00
61,76	0,00	25,00	1,47	11,76	0,00	99,51
72,13	0,55	17,49	0,00	9,84	0,00	100,00
50,63	1,58	34,49	0,32	12,97	0,00	100,00
55,21	0,00	30,50	0,00	14,29	0,00	99,61
68,32	1,91	15,65	0,00	11,45	2,67	99,62
60,17	2,49	17,84	0,00	19,50	0,00	100,00
67,02	0,70	29,82	0,00	1,40	1,05	100,00
71,31	1,64	25,41	0,00	1,64	0,00	100,00
62,38	20,48	13,10	0,24	3,57	0,24	100,00
63,51	11,26	20,27	0,90	0,90	3,15	100,00
58,29	16,58	19,74	0,78	4,42	0,00	100,00
62,28	13,16	15,35	0,00	8,77	0,44	100,00
54,88	2,02	25,93	3,03	14,14	0,00	100,00
44,29	34,29	20,00	1,43	0,00	0,00	100,00
54,76	17,86	21,43	0,00	5,95	0,00	100,00
70,76	1,04	19,90	0,26	7,59	0,26	100,00
62,71	0,00	29,56	1,93	5,80	0,00	100,00
34,19	41,88	18,57	0,00	4,29	0,86	100,00
56,55	3,00	21,35	0,00	0,75	18,35	100,00
69,77	0,32	19,08	0,00	3,89	0,00	100,00
73,73	0,42	14,83	0,00	8,05	2,97	100,00
65,16	2,58	22,90	0,00	2,58	6,77	100,00
84,89	0,55	12,91	0,00	1,65	0,00	98,90
70,98	0,00	23,61	0,00	5,06	0,00	99,76
73,04	0,43	22,61	0,00	3,91	0,00	100,00
68,85	0,33	26,64	0,00	3,95	0,00	100,00
74,33	1,15	15,71	0,38	8,43	0,00	100,00
54,90	0,65	28,10	1,63	14,38	0,33	99,67
64,58	0,42	24,58	0,00	9,58	0,83	99,17
77,14	1,43	6,67	0,00	11,43	3,33	100,00
72,44	2,36	19,69	0,00	3,15	2,36	100,00
59,79	1,42	24,29	0,00	5,67	0,40	99,60
61,02	15,02	18,53	0,00	5,43	0,00	100,00
61,73	4,59	27,04	0,00	6,12	0,51	100,00
46,32	27,71	18,18	0,00	6,93	0,87	99,57
69,72	0,00	12,84	0,92	15,60	0,92	100,00
87,50	0,57	6,25	0,00	5,68	0,00	100,00
97,73	0,00	0,00	0,00	0,00	2,27	97,73
91,01	0,56	7,87	0,00	0,56	0,00	100,00
78,42	0,72	12,23	0,00	5,76	2,88	100,00
94,05	0,00	4,76	0,00	1,19	0,00	100,00
83,78	0,00	6,01	0,30	9,91	0,00	99,70
82,98	3,19	11,70	0,00	2,13	0,00	100,00
75,48	1,61	16,77	0,00	5,48	0,65	100,00
55,06	1,69	24,72	0,56	15,73	2,25	100,00
74,24	3,05	19,94	0,00	2,77	0,00	100,00
52,74	23,29	20,55	0,00	2,05	1,37	100,00
61,46	23,96	11,52	0,52	1,57	0,52	100,00

36,96	28,40	31,91	0,00	2,72	0,00	100,00
43,42	21,93	31,14	0,44	3,07	0,00	100,00
55,87	10,61	28,49	0,00	5,03	0,00	100,00
64,35	3,93	25,38	0,00	6,34	0,00	99,40
45,34	24,29	23,08	0,00	5,26	2,02	100,00
53,11	22,49	19,14	0,00	4,31	0,96	100,00
41,80	33,86	17,99	0,53	4,23	1,59	100,00
63,36	16,19	18,83	0,81	0,81	0,00	100,00
70,89	0,94	26,76	0,00	1,41	0,00	100,00
71,43	0,48	20,95	0,00	0,95	6,19	99,52
58,46	9,79	22,32	0,30	8,93	0,00	100,00
85,20	0,00	9,89	0,00	3,30	0,37	100,00
78,67	0,95	16,27	0,48	2,87	0,00	100,00
60,39	1,45	13,53	0,00	23,67	0,97	100,00
83,15	0,54	15,76	0,00	0,54	0,00	98,91
80,77	0,59	14,50	0,59	3,25	0,30	99,41
62,57	0,57	17,43	0,00	19,43	0,00	100,00
56,36	0,91	33,18	0,00	9,55	0,00	100,00
60,26	2,56	22,44	0,32	14,42	0,00	99,68
71,92	0,00	15,14	0,00	12,93	0,00	100,00
57,05	0,64	40,38	0,00	1,92	0,00	100,00
52,51	0,67	37,79	0,33	8,70	0,00	99,67
94,56	0,60	4,23	0,00	0,60	0,00	100,00
65,65	0,38	24,70	0,00	5,26	0,00	99,60
61,39	9,90	24,21	0,00	0,00	0,00	100,00
33,18	30,33	19,41	0,00	1,76	0,00	100,00
66,67	4,94	23,05	0,00	4,94	0,41	100,00
57,26	9,40	26,50	2,56	2,56	1,71	100,00
65,55	2,87	23,92	0,00	7,66	0,00	100,00
67,27	3,96	26,26	0,00	2,52	0,00	100,00
64,34	2,71	19,84	0,00	12,45	0,39	100,00
69,18	13,01	17,81	0,00	0,00	0,00	100,00
65,69	8,82	21,57	0,00	3,92	0,00	100,00
66,67	11,28	22,05	0,00	0,00	0,00	100,00
55,24	9,84	28,57	0,00	4,13	2,22	100,00
65,48	8,12	23,86	0,00	2,54	0,00	100,00
71,05	0,00	21,74	0,00	4,89	0,00	100,00
64,41	0,00	34,75	0,00	0,85	0,00	100,00
79,89	0,54	16,30	0,00	3,26	0,00	100,00
41,47	20,59	32,35	0,00	4,71	0,88	100,00
53,41	21,21	17,05	0,00	7,20	1,14	100,00
52,56	22,10	22,64	0,54	2,16	0,00	99,73
59,30	15,58	23,12	0,50	1,01	0,50	99,50
56,09	11,74	27,39	0,43	4,35	0,00	100,00
59,71	7,19	30,43	0,00	2,17	0,00	99,28
63,76	15,14	20,64	0,00	0,46	0,00	100,00
49,83	32,23	17,61	0,00	0,00	0,33	100,00
73,79	11,65	14,08	0,00	0,49	0,00	100,00
64,92	19,06	14,92	0,00	1,10	0,00	99,72
56,59	22,53	17,58	0,55	2,75	0,00	98,90

60,07	20,14	18,47	0,00	1,05	0,00	100,00
58,26	0,00	33,61	0,00	8,12	0,00	100,00
62,67	0,00	28,11	0,46	8,76	0,00	100,00
63,75	0,97	31,72	0,00	3,56	0,00	100,00
61,54	0,00	34,41	0,00	4,05	0,00	100,00
66,50	5,83	25,73	0,49	0,97	0,49	100,00
56,28	2,60	30,87	0,87	9,13	0,00	99,57
56,32	1,15	25,86	1,15	11,49	4,02	100,00
56,99	16,36	16,36	0,53	9,23	0,53	100,00
71,76	0,46	21,76	0,46	5,56	0,00	99,07
60,36	0,00	35,50	0,00	4,14	0,00	98,82
82,24	0,66	14,14	0,00	2,96	0,00	100,00
60,99	0,00	33,44	0,93	4,64	0,00	100,00
88,71	0,63	9,72	0,00	0,94	0,00	100,00
87,32	0,70	4,23	0,00	7,75	0,00	100,00
77,00	2,00	7,00	0,00	14,00	0,00	100,00
95,86	0,00	4,14	0,00	0,00	0,00	100,00
78,64	3,88	9,71	0,97	6,80	0,00	100,00
46,15	0,00	53,85	0,00	0,00	0,00	100,00
94,35	0,00	5,65	0,00	0,00	0,00	100,00
73,47	4,08	10,20	1,02	11,22	0,00	100,00
90,79	0,00	5,26	0,00	3,95	0,00	100,00
86,61	1,79	9,38	0,00	2,23	0,00	100,00
69,96	0,00	17,17	0,00	12,88	0,00	99,57
68,83	2,60	23,38	0,00	5,19	0,00	100,00
74,25	5,58	12,45	0,00	6,44	1,29	100,00
86,45	0,00	8,39	0,65	4,52	0,00	100,00
69,18	1,37	19,18	0,00	10,27	0,00	100,00
96,09	0,00	2,34	0,00	1,56	0,00	100,00
92,96	0,00	3,52	0,00	3,52	0,00	100,00
90,18	0,00	7,64	0,00	2,18	0,00	100,00
73,44	0,00	21,88	0,00	4,69	0,00	100,00
79,41	1,96	14,71	0,00	3,92	0,00	100,00
84,91	0,00	10,38	0,00	4,72	0,00	100,00
85,71	0,62	6,21	0,00	7,45	0,00	100,00
68,48	0,61	12,12	0,00	18,79	0,00	100,00
74,10	1,44	11,51	0,00	11,51	1,44	100,00
84,12	0,86	6,87	0,00	8,15	0,00	100,00
75,49	0,00	18,63	0,00	5,88	0,00	100,00
59,69	1,56	28,75	0,00	10,00	0,00	99,06
71,97	0,00	22,73	0,00	4,55	0,76	100,00
35,29	43,32	18,72	0,00	2,67	0,00	100,00
54,11	19,83	18,41	0,57	7,08	0,00	99,72
85,33	2,00	8,67	0,67	3,33	0,00	100,00
57,53	0,00	24,14	0,00	3,45	0,00	100,00
61,57	6,34	24,63	0,00	7,09	0,37	100,00
64,31	14,47	16,08	0,00	4,82	0,32	100,00
56,44	9,85	29,17	0,00	3,79	0,76	100,00
82,16	1,08	6,52	0,00	5,98	3,80	99,46
63,78	7,87	26,38	0,00	1,57	0,39	100,00

58,82	7,98	28,27	0,00	4,64	0,00	100,00
76,88	0,00	16,58	0,00	6,03	0,50	98,49
58,33	14,35	18,98	0,23	8,10	0,00	100,00
63,98	7,58	24,64	0,95	2,84	0,00	100,00
72,30	1,41	21,60	0,00	4,69	0,00	100,00
62,03	16,61	19,66	0,00	1,02	0,68	100,00
66,67	5,83	25,83	0,00	1,67	0,00	100,00
69,54	11,49	16,67	0,00	2,30	0,00	100,00
66,19	6,47	14,39	0,00	10,07	2,88	100,00
66,25	12,50	17,31	0,00	1,92	0,00	100,00
89,82	0,44	5,31	0,00	3,98	0,44	99,56
64,58	0,42	32,50	0,00	2,50	0,00	100,00
82,92	7,92	6,67	0,00	2,50	0,00	98,33
92,48	0,00	5,26	0,00	2,26	0,00	100,00
87,92	0,42	8,75	0,00	2,92	0,00	99,58
88,29	0,90	9,91	0,00	0,90	0,00	98,20
28,79	46,97	10,10	0,00	14,14	0,00	97,98
88,46	3,21	7,05	0,00	1,28	0,00	100,00
96,25	1,25	1,25	0,00	0,00	1,25	100,00
0,00	0,00	0,00	0,00	0,00	0,00	0,00
56,50	25,08	15,33	0,77	2,17	0,15	100,00
56,87	17,91	21,47	0,70	2,44	0,35	100,00
55,74	12,34	28,72	0,43	2,13	0,64	100,00
54,05	18,77	25,57	0,65	0,97	0,00	100,00
65,30	14,22	18,80	0,00	1,69	0,00	100,00
59,05	18,99	19,58	0,30	1,78	0,30	100,00
65,43	11,28	21,11	0,37	1,67	0,00	100,00
73,95	6,72	17,87	0,00	0,43	0,00	100,00
59,02	15,08	23,36	0,00	2,30	0,00	100,00
54,06	0,71	32,16	0,35	12,72	0,00	100,00
32,91	36,71	29,11	0,00	1,27	0,00	100,00
77,14	0,00	21,07	0,36	1,43	0,00	100,00
60,00	0,00	17,60	11,20	6,40	0,00	100,00
71,25	0,31	26,67	0,00	0,63	0,00	100,00
78,76	0,00	19,03	0,00	1,99	0,22	100,00
59,58	17,08	16,25	3,75	2,92	0,42	100,00
36,89	36,89	20,87	0,00	4,85	0,49	88,35
0,00	0,00	0,00	0,00	0,00	0,00	0,00
72,37	0,33	20,13	0,33	6,60	0,00	100,00
73,81	0,00	18,45	0,00	5,95	1,79	100,00
66,87	1,22	17,63	0,30	13,98	0,00	100,00
52,71	1,55	43,41	0,00	1,55	0,78	100,00
61,88	1,98	34,50	0,00	0,50	0,50	100,00
78,61	2,89	18,50	0,00	0,00	0,00	100,00
83,76	0,85	9,40	0,00	5,98	0,00	100,00
31,25	1,56	54,33	3,94	7,09	1,57	100,00
57,72	6,99	28,31	0,00	6,99	0,00	99,63
68,29	4,07	21,14	0,00	6,50	0,00	100,00
62,10	8,60	20,54	0,00	4,71	0,00	99,66
66,82	4,67	23,36	0,00	5,14	0,00	100,00

63,70	9,24	24,58	0,00	1,33	0,66	99,67
59,87	13,16	18,06	0,69	3,82	0,35	100,00
68,68	10,92	16,09	0,00	4,31	0,00	100,00
68,78	13,50	14,35	0,00	2,95	0,42	100,00
74,82	4,61	15,96	0,35	4,26	0,00	99,65
53,02	22,82	18,46	0,67	4,36	0,67	100,00
60,14	10,02	26,25	0,24	3,10	0,24	100,00
55,49	19,65	21,97	0,00	1,73	1,16	100,00
42,86	0,00	57,14	0,00	0,00	0,00	100,00
86,44	0,00	11,86	0,00	1,69	0,00	100,00
91,30	0,54	7,07	0,00	1,09	0,00	100,00
85,55	0,23	11,71	0,00	2,11	0,00	100,00
87,10	3,23	7,53	0,00	2,15	0,00	100,00
60,90	8,97	14,39	0,36	6,12	0,72	100,00
13,33	0,00	6,67	80,00	0,00	0,00	100,00
74,03	1,30	7,79	0,00	16,88	0,00	98,05
93,48	0,00	6,52	0,00	0,00	0,00	100,00
94,32	0,38	3,44	0,00	1,15	0,00	100,00
45,24	0,00	42,86	0,00	11,90	0,00	100,00
61,02	10,17	25,54	0,00	1,73	0,00	100,00
58,61	9,52	24,34	0,00	5,99	0,00	100,00
61,19	1,83	34,25	0,00	2,74	0,00	100,00
69,86	3,08	22,89	0,70	0,70	0,70	100,00
59,08	4,61	35,45	0,58	0,29	0,00	100,00
41,30	7,51	48,12	0,00	3,07	0,00	100,00
48,52	8,85	41,31	0,33	0,98	0,00	100,00
67,85	5,99	21,80	0,27	3,27	0,82	100,00
61,43	8,57	14,29	2,86	11,43	1,43	100,00
66,80	5,02	22,39	0,00	4,63	1,16	100,00
76,78	2,37	18,96	0,00	1,90	0,00	100,00
64,95	8,16	20,85	0,30	5,74	0,00	100,00
37,68	37,68	23,19	0,58	0,87	0,00	100,00
73,73	2,85	18,04	0,00	5,38	0,00	100,00
90,32	0,00	7,74	0,00	1,29	0,65	100,00
83,33	0,00	13,89	0,00	2,78	0,00	100,00
89,02	0,00	9,88	0,00	0,00	0,00	100,00
94,74	0,00	5,26	0,00	0,00	0,00	100,00
87,38	2,91	8,74	0,97	0,00	0,00	99,03
63,11	2,67	33,33	0,44	0,44	0,00	100,00
55,75	5,31	28,71	0,00	2,97	0,00	100,00
67,18	0,39	28,02	0,78	3,11	0,00	100,00
67,46	4,73	23,67	0,00	3,85	0,30	100,00
74,25	1,10	18,77	0,00	4,20	0,00	100,00
66,56	5,63	23,84	1,99	1,32	0,66	100,00
67,57	6,54	21,80	0,00	3,54	0,54	100,00
65,58	4,71	24,36	0,73	4,36	0,00	100,00
64,57	7,48	24,41	0,00	3,54	0,00	100,00
69,44	8,33	19,33	0,00	2,24	0,00	99,72
61,07	10,66	26,23	0,41	1,64	0,00	100,00
63,16	17,29	16,92	0,00	2,63	0,00	100,00

67,97	9,77	18,36	0,00	3,91	0,00	100,00
59,39	11,49	23,55	0,77	4,25	0,00	100,00
54,74	12,77	16,14	1,18	9,45	0,39	100,00
72,62	5,23	18,77	0,00	3,38	0,00	100,00
77,56	5,94	13,53	0,66	2,31	0,00	100,00
86,42	1,23	4,94	0,00	7,41	0,00	100,00
86,52	0,00	11,24	0,00	2,25	0,00	100,00
82,26	0,00	15,45	0,81	0,81	0,00	100,00
82,43	0,00	15,54	0,00	2,03	0,00	100,00
93,40	0,00	5,66	0,00	0,94	0,00	100,00
89,00	0,00	10,00	0,00	1,00	0,00	98,00
87,72	0,00	12,28	0,00	0,00	0,00	100,00
53,59	13,92	24,47	0,00	8,02	0,00	100,00
52,44	1,78	43,56	0,00	2,22	0,00	100,00
61,66	8,85	18,84	0,00	8,31	0,00	100,00
64,81	2,78	15,63	0,00	8,33	0,00	100,00
69,77	5,43	22,44	0,00	1,18	0,00	100,00
70,10	6,31	19,67	0,00	3,33	0,33	100,00
68,15	7,01	22,29	0,00	1,91	0,64	100,00
66,81	7,08	23,01	1,33	1,77	0,00	100,00
67,11	7,24	15,83	0,00	2,16	0,72	100,00
57,22	11,11	25,42	0,56	5,31	0,00	100,00
71,11	0,56	26,11	0,00	2,22	0,00	100,00
63,82	3,42	27,30	0,57	4,31	0,00	100,00
79,53	0,00	19,07	0,00	1,40	0,00	99,53
74,05	4,20	19,47	0,00	2,29	0,00	100,00
70,53	4,56	19,20	0,36	2,90	0,00	100,00
68,91	6,22	22,80	0,00	2,07	0,00	100,00
75,17	2,41	17,59	0,00	4,83	0,00	100,00
99,68	0,00	0,00	0,00	0,32	0,00	100,00
74,58	8,75	16,25	0,00	0,42	0,00	100,00
82,84	0,98	13,73	0,00	2,45	0,00	100,00
83,10	0,70	15,49	0,00	0,70	0,00	100,00
71,43	11,58	14,29	0,39	2,32	0,00	100,00
84,44	0,00	12,22	2,22	1,11	0,00	100,00
81,82	0,00	15,15	0,00	3,03	0,00	100,00
83,22	7,38	8,05	0,67	0,67	0,00	100,00
87,92	1,29	8,23	1,03	1,54	0,00	100,00
94,12	0,00	5,88	0,00	0,00	0,00	100,00
88,10	1,59	7,94	0,00	2,38	0,00	100,00
61,15	5,10	18,59	0,64	12,18	1,92	100,00
52,47	8,02	34,39	0,64	2,55	0,00	100,00
71,43	1,50	24,15	0,00	2,64	0,00	100,00
61,97	6,10	27,23	0,00	4,69	0,00	100,00
61,22	4,37	23,95	0,00	8,08	0,60	100,00
76,85	5,47	15,81	0,00	1,61	0,00	100,00
65,34	1,59	29,08	0,00	3,98	0,00	100,00
57,46	4,39	36,28	0,44	0,88	0,00	100,00
64,71	5,88	20,57	0,96	3,83	0,00	100,00
69,23	4,62	22,56	0,00	3,59	0,00	100,00

73,25	0,41	19,75	0,00	6,58	0,00	99,59
66,95	5,86	23,61	0,00	1,29	0,43	100,00
73,71	4,38	20,72	0,00	0,80	0,40	100,00
78,52	2,46	15,85	0,00	2,82	0,35	100,00
72,50	4,64	19,29	0,00	3,57	0,00	100,00
88,29	3,41	7,07	0,24	0,98	0,00	100,00
74,33	7,46	15,82	0,00	2,09	0,30	100,00
73,21	5,95	17,56	0,00	2,98	0,30	100,00
75,28	3,65	18,26	0,00	2,81	0,00	97,19
75,89	7,11	13,04	0,00	3,95	0,00	100,00
55,39	3,35	37,69	0,75	2,61	0,00	100,00
79,72	5,63	13,52	0,00	0,85	0,28	100,00
30,60	30,60	11,39	21,00	6,05	0,36	100,00
53,00	13,36	30,41	0,00	3,23	0,00	98,62
67,44	0,00	27,91	0,00	4,65	0,00	100,00
49,04	34,39	13,38	0,00	2,55	0,64	99,36
83,64	0,00	16,36	0,00	0,00	0,00	100,00
60,78	0,00	21,57	0,00	17,65	0,00	100,00
60,25	0,62	32,30	0,00	6,83	0,00	100,00
69,05	1,19	26,51	0,00	2,41	0,00	100,00
86,36	0,00	9,09	0,00	4,55	0,00	100,00
79,17	0,00	16,67	0,00	4,17	0,00	100,00
72,31	0,00	20,00	0,00	7,69	0,00	100,00
71,74	1,09	14,13	0,00	7,61	5,43	98,91
42,00	42,00	16,00	0,00	0,00	0,00	100,00
45,45	18,18	18,18	0,00	13,64	4,55	100,00
0,00	0,00	0,00	0,00	0,00	0,00	0,00
51,20	10,65	32,65	0,00	5,50	0,00	99,66
87,36	1,15	9,20	0,00	2,30	0,00	100,00
58,96	0,52	19,74	0,52	20,26	0,00	100,00
57,28	11,46	25,39	0,00	5,57	0,31	99,69
78,75	0,00	17,50	1,25	2,50	0,00	98,75
80,75	0,00	19,25	0,00	0,00	0,00	100,00
57,14	2,04	36,73	0,00	4,08	0,00	100,00
65,28	4,86	26,39	0,00	3,47	0,00	100,00
43,94	0,00	46,97	0,00	9,09	0,00	100,00
44,44	0,00	44,44	0,00	11,11	0,00	100,00
74,89	0,00	22,83	0,00	2,28	0,00	100,00
59,63	8,15	22,59	0,00	8,89	0,74	100,00
74,19	0,65	23,23	0,00	1,94	0,00	100,00
85,71	0,00	14,29	0,00	0,00	0,00	100,00
58,33	0,00	25,00	0,00	16,67	0,00	100,00
80,95	0,00	9,52	0,00	9,52	0,00	100,00
55,80	17,39	26,81	0,00	0,00	0,00	100,00
66,00	3,33	26,67	2,00	2,00	0,00	98,67
57,89	0,00	40,35	1,75	0,00	0,00	100,00
86,81	0,00	12,09	0,00	1,10	0,00	100,00
65,88	1,76	28,24	0,00	4,12	0,00	100,00
69,52	1,43	25,24	0,00	3,81	0,00	99,52
33,41	43,60	18,44	0,00	4,12	0,43	100,00

72,84	7,10	15,12	0,31	4,63	0,00	100,00
52,00	20,00	20,00	4,00	4,00	0,00	100,00
71,43	7,14	21,43	0,00	0,00	0,00	100,00
63,16	1,05	28,42	2,11	5,26	0,00	100,00
78,70	2,78	14,81	0,00	3,70	0,00	100,00
77,67	7,77	9,80	0,00	3,92	0,00	100,00
90,00	10,00	0,00	0,00	0,00	0,00	100,00
88,36	0,00	10,27	0,00	1,37	0,00	99,32
95,65	0,00	4,35	0,00	0,00	0,00	100,00
55,36	3,57	25,00	0,00	16,07	0,00	100,00
78,31	2,41	13,25	0,00	4,82	1,20	100,00
35,32	45,11	15,74	0,00	3,83	0,00	100,00
75,00	0,00	5,71	0,00	14,29	2,86	100,00
74,07	3,70	7,41	0,00	14,81	0,00	96,30
67,65	0,00	15,44	0,00	15,44	1,47	98,53
66,52	5,43	16,59	0,98	4,88	0,00	100,00
78,53	5,52	14,11	0,00	1,23	0,61	95,09
67,35	0,00	30,61	0,00	2,04	0,00	97,96
54,00	15,33	25,33	0,00	5,33	0,00	100,00
50,84	5,04	23,95	0,00	19,75	0,42	100,00
73,33	2,22	15,56	0,00	6,67	2,22	100,00
100,00	0,00	0,00	0,00	0,00	0,00	100,00
64,29	8,57	21,43	0,00	5,71	0,00	100,00
80,00	2,86	17,14	0,00	0,00	0,00	100,00
0,00	0,00	0,00	0,00	0,00	0,00	0,00
93,94	0,00	0,00	0,00	6,06	0,00	100,00
67,63	7,91	21,58	0,00	2,88	0,00	100,00
93,33	0,00	6,67	0,00	0,00	0,00	100,00
50,00	0,00	41,67	0,00	8,33	0,00	100,00
50,41	0,00	31,71	0,00	17,89	0,00	97,56
93,18	0,00	5,30	0,00	1,52	0,00	100,00
42,31	0,00	44,16	0,00	12,99	0,00	100,00
60,00	0,00	40,00	0,00	0,00	0,00	100,00
47,70	24,69	22,59	0,00	5,02	0,00	100,00
64,23	2,85	29,67	0,81	1,22	1,22	100,00
51,25	26,52	20,07	0,00	0,36	0,36	100,00
42,90	27,51	27,81	0,00	1,78	0,00	100,00
66,22	16,89	14,29	0,00	2,04	0,00	100,00
44,75	14,36	37,02	0,00	3,87	0,00	99,72
62,75	14,22	22,06	0,49	0,49	0,00	99,51
56,14	22,37	19,73	0,00	0,00	0,00	100,00
51,27	22,46	25,00	0,00	0,42	0,85	100,00
76,47	6,62	15,81	0,00	1,10	0,00	100,00
63,07	15,91	19,89	0,00	1,14	0,00	100,00
57,31	18,47	20,38	0,24	3,36	0,24	100,00
63,76	26,61	7,80	0,00	1,83	0,00	100,00
61,76	10,78	15,50	0,00	9,50	1,00	100,00
54,69	0,00	34,38	1,56	9,38	0,00	100,00
68,09	5,32	23,40	0,00	3,19	0,00	98,94
69,74	3,95	19,74	0,66	5,92	0,00	100,00

85,50	1,00	10,00	0,00	3,50	0,00	99,50
0,00	0,00	0,00	0,00	0,00	0,00	0,00
70,73	0,00	19,51	0,00	9,76	0,00	100,00
0,00	0,00	0,00	0,00	0,00	0,00	0,00
77,31	0,84	9,24	0,00	12,61	0,00	100,00
93,18	0,00	4,55	0,00	2,27	0,00	100,00
91,49	0,00	8,51	0,00	0,00	0,00	100,00
58,33	0,00	41,67	0,00	0,00	0,00	100,00
78,29	0,00	13,95	0,78	6,98	0,00	100,00
31,03	0,00	65,52	0,00	3,45	0,00	100,00
68,29	3,66	9,15	0,61	18,29	0,00	100,00
77,27	0,00	22,73	0,00	0,00	0,00	100,00
95,12	0,00	2,44	0,00	2,44	0,00	100,00
77,01	2,87	11,49	0,57	8,05	0,00	100,00
73,75	0,00	13,75	0,00	12,50	0,00	100,00
55,88	0,00	41,18	0,00	2,94	0,00	100,00
71,62	1,75	26,64	0,00	0,00	0,00	100,00
0,00	0,00	0,00	0,00	0,00	0,00	0,00
69,57	4,35	26,09	0,00	0,00	0,00	100,00
58,97	16,67	17,95	0,00	6,41	0,00	100,00
60,27	4,11	26,03	0,00	9,59	0,00	100,00
36,51	3,17	39,68	0,00	20,63	0,00	100,00
55,79	9,47	28,42	0,00	6,32	0,00	100,00
77,78	3,33	18,89	0,00	0,00	0,00	100,00
54,10	1,64	26,53	0,00	4,08	0,00	100,00
90,74	0,93	7,41	0,00	0,93	0,00	100,00
78,06	4,59	16,33	0,00	1,02	0,00	100,00
59,15	13,19	23,40	0,43	3,40	0,43	100,00
89,66	0,00	5,17	0,00	5,17	0,00	100,00
91,80	0,00	8,20	0,00	0,00	0,00	100,00
54,91	19,65	19,08	0,00	6,36	0,00	100,00
54,55	0,00	45,45	0,00	0,00	0,00	87,88
0,00	0,00	0,00	0,00	0,00	0,00	0,00
0,00	0,00	0,00	0,00	0,00	0,00	0,00
81,82	9,09	9,09	0,00	0,00	0,00	100,00
81,94	0,00	15,74	0,00	2,31	0,00	100,00
0,00	0,00	0,00	0,00	0,00	0,00	0,00
91,25	0,00	6,28	0,00	2,09	0,00	99,58
93,33	0,00	0,00	6,67	0,00	0,00	100,00
93,55	0,00	4,30	0,00	1,08	1,08	100,00
60,75	11,21	25,42	0,19	2,43	0,00	100,00
95,74	0,00	4,26	0,00	0,00	0,00	100,00
0,00	0,00	0,00	0,00	0,00	0,00	0,00
0,00	0,00	0,00	0,00	0,00	0,00	0,00
62,69	25,37	8,96	0,00	2,99	0,00	100,00
73,84	0,00	24,01	0,00	2,15	0,00	100,00
63,35	16,75	16,23	0,00	3,66	0,00	100,00
74,48	0,00	22,76	0,69	2,07	0,00	100,00
94,74	0,00	5,26	0,00	0,00	0,00	100,00
56,34	0,00	35,21	1,41	7,04	0,00	100,00

85,14	0,00	12,16	0,00	2,70	0,00	100,00
93,10	0,00	6,90	0,00	0,00	0,00	100,00
63,00	23,67	12,67	0,00	0,67	0,00	100,00
76,08	0,00	14,51	0,39	9,02	0,00	100,00
42,86	14,29	21,43	7,14	14,29	0,00	100,00
73,85	0,00	15,38	0,00	10,77	0,00	100,00
100,00	0,00	0,00	0,00	0,00	0,00	100,00
80,95	4,76	9,52	0,00	4,76	0,00	100,00
72,62	0,00	22,62	1,19	3,57	0,00	100,00
48,44	0,00	50,00	1,56	0,00	0,00	98,44
0,00	0,00	0,00	0,00	0,00	0,00	0,00
0,00	0,00	0,00	0,00	0,00	0,00	0,00
62,37	2,15	26,88	0,00	7,53	1,08	100,00
0,00	0,00	0,00	0,00	0,00	0,00	0,00
62,67	4,00	18,67	1,33	13,33	0,00	100,00
57,61	14,13	26,09	0,00	2,17	0,00	100,00
97,78	0,00	2,22	0,00	0,00	0,00	100,00
100,00	0,00	0,00	0,00	0,00	0,00	100,00
85,71	0,00	14,29	0,00	0,00	0,00	100,00
74,03	0,00	22,08	0,00	3,90	0,00	100,00
95,21	0,00	2,74	0,00	2,05	0,00	100,00
78,13	0,00	17,71	0,00	4,17	0,00	100,00
100,00	0,00	0,00	0,00	0,00	0,00	100,00
57,14	0,00	28,57	0,00	14,29	0,00	100,00
0,00	0,00	0,00	0,00	0,00	0,00	0,00
34,34	38,38	21,88	0,00	3,13	0,00	100,00
40,00	50,00	0,00	0,00	0,00	0,00	100,00
67,65	13,24	10,29	0,00	7,35	1,47	100,00
21,33	64,44	10,67	0,44	3,11	0,00	98,22
84,46	2,07	8,29	0,52	4,66	0,00	100,00
35,57	29,53	24,56	0,35	7,02	0,00	100,00
58,14	16,28	20,93	1,16	3,49	0,00	100,00
62,18	12,00	24,73	0,00	1,09	0,00	100,00
62,18	10,26	20,51	0,32	6,73	0,00	100,00
66,19	11,39	15,27	0,36	1,82	3,27	100,00
81,38	3,45	2,24	3,73	2,24	0,00	99,25
86,07	1,43	5,19	1,11	2,22	0,74	98,15
87,59	2,92	6,57	0,00	2,92	0,00	100,00
0,00	0,00	0,00	0,00	0,00	0,00	0,00
83,13	6,17	9,47	0,00	1,23	0,00	100,00
82,58	2,27	6,06	0,00	9,09	0,00	99,24
72,22	0,79	23,81	0,00	3,17	0,00	99,21
74,44	1,25	15,87	0,00	8,06	0,00	100,00
48,35	26,01	23,08	0,00	2,20	0,37	100,00
0,00	0,00	0,00	0,00	0,00	0,00	0,00
56,47	26,29	15,52	0,43	1,29	0,00	100,00
28,19	56,39	10,57	0,44	4,41	0,00	100,00
66,11	0,00	18,83	0,00	15,06	0,00	100,00
61,42	5,56	21,98	0,62	9,60	0,62	100,00
58,61	5,74	31,15	0,41	4,10	0,00	99,18

65,66	1,01	30,81	0,00	2,53	0,00	100,00
50,00	3,31	26,56	0,00	19,92	0,00	99,59
69,70	0,00	24,24	0,00	6,06	0,00	100,00
70,62	0,58	3,82	0,25	2,54	0,25	100,00
59,48	9,33	20,70	0,64	3,18	0,32	100,00
59,20	0,00	14,59	0,86	1,72	0,00	100,00
38,26	20,00	20,87	20,00	0,87	0,00	100,00
87,95	0,00	7,23	0,00	4,82	0,00	99,40
55,07	9,66	27,18	0,97	5,83	0,97	100,00
37,34	12,45	27,39	12,86	8,71	1,24	100,00
77,27	0,00	13,85	0,00	7,69	0,00	100,00
63,64	10,00	21,36	0,45	4,55	0,00	100,00
68,06	13,89	10,65	0,00	7,41	0,00	100,00
60,47	12,09	21,86	0,00	5,12	0,47	99,07
36,34	22,35	39,95	0,00	1,13	0,23	100,00
52,43	10,81	35,14	0,00	0,00	1,62	100,00
61,90	28,57	4,76	0,00	4,76	0,00	100,00
71,62	6,76	17,12	0,00	4,05	0,45	100,00
57,37	14,42	20,95	0,34	2,70	0,34	100,00
57,14	4,17	33,33	0,60	4,17	0,60	100,00
48,00	1,71	34,29	0,57	10,86	4,57	98,86
33,90	27,54	30,93	0,00	7,63	0,00	100,00
53,15	17,57	26,13	1,35	1,80	0,00	99,55
46,31	23,15	22,48	4,36	3,69	0,00	100,00
61,39	3,80	22,15	1,27	11,39	0,00	99,37
59,73	2,68	30,20	0,00	7,38	0,00	99,33
69,68	6,38	12,77	0,00	5,85	5,32	100,00
0,00	0,00	0,00	0,00	0,00	0,00	0,00
41,23	43,86	14,91	0,00	0,00	0,00	100,00
39,48	44,28	12,55	0,00	3,32	0,37	100,00
47,21	32,99	17,26	0,00	2,54	0,00	100,00
52,52	27,31	18,49	0,00	1,68	0,00	99,58
56,76	22,30	20,27	0,00	0,00	0,68	100,00
55,45	9,95	27,01	0,00	7,58	0,00	100,00
62,85	17,39	16,21	0,00	3,56	0,00	100,00
51,24	23,32	20,49	0,35	4,59	0,00	99,65
56,49	22,18	18,41	0,00	2,51	0,42	100,00
39,90	40,87	18,27	0,00	0,48	0,48	100,00
53,60	1,80	30,69	0,00	13,72	0,00	100,00
52,84	1,42	26,60	0,00	19,15	0,00	99,65
66,53	1,63	21,72	0,00	9,84	0,00	100,00
71,88	2,23	16,52	0,00	9,38	0,00	99,55
67,06	5,16	16,27	0,00	11,11	0,40	99,60
63,24	18,38	14,71	0,00	3,68	0,00	100,00
70,55	15,86	13,27	0,00	0,00	0,32	100,00
54,90	21,57	19,61	1,96	1,96	0,00	100,00
58,20	0,82	29,10	0,41	11,48	0,00	100,00
63,00	23,81	13,19	0,00	0,00	0,00	100,00
52,98	0,60	31,55	1,19	13,69	0,00	100,00
54,55	5,26	30,62	0,00	9,57	0,00	98,56

46,09	17,19	33,59	0,00	3,13	0,00	100,00
58,04	0,00	16,52	0,00	25,45	0,00	100,00
56,14	2,09	21,67	0,52	19,58	0,00	100,00
62,10	1,27	28,53	0,32	7,37	0,00	99,36
36,12	32,24	8,57	0,00	22,86	0,20	100,00
26,09	0,77	4,09	0,26	66,50	2,30	100,00
0,00	0,00	0,00	0,00	0,00	0,00	0,00
77,82	3,23	8,30	5,39	2,90	0,00	98,34
60,00	10,83	16,88	3,90	5,63	0,00	99,57
64,49	0,56	6,55	3,18	5,06	20,04	78,09
70,22	2,22	6,38	2,13	3,19	1,60	99,47
67,15	1,93	9,36	3,51	3,51	0,00	98,83
90,48	0,00	1,19	8,33	0,00	0,00	100,00
83,25	2,46	7,39	4,93	1,97	0,00	99,01
68,25	0,95	9,78	4,35	6,52	0,00	100,00
64,38	2,05	3,33	14,17	1,67	0,00	99,17
87,21	3,03	5,39	2,02	2,02	0,34	99,66
10,53	0,00	5,26	84,21	0,00	0,00	100,00
74,19	10,14	13,82	0,00	1,84	0,00	99,08
85,12	0,60	13,69	0,00	0,60	0,00	100,00
62,90	12,37	15,17	1,12	5,06	0,00	100,00
73,21	2,39	20,57	0,00	3,83	0,00	100,00
68,51	13,81	13,81	0,00	3,87	0,00	100,00
76,52	6,82	15,91	0,00	0,76	0,00	100,00
56,30	13,33	25,56	0,00	4,07	0,74	100,00
58,36	15,61	17,84	0,37	7,81	0,00	100,00
57,40	22,42	17,12	0,00	2,70	0,00	100,00
52,38	20,95	22,86	0,00	3,81	0,00	100,00
69,23	12,22	17,19	0,00	1,36	0,00	100,00
81,32	0,55	10,99	0,55	5,49	1,10	100,00
65,27	1,20	23,35	0,00	10,18	0,00	100,00
50,79	0,79	34,13	1,19	11,51	1,59	99,60
0,00	0,00	0,00	0,00	0,00	0,00	0,00
62,42	19,93	16,01	0,33	1,31	0,00	99,67
62,50	0,00	23,21	0,00	12,50	1,79	100,00
64,98	0,78	27,63	0,00	6,61	0,00	100,00
61,97	7,51	25,00	0,00	5,19	0,00	100,00
67,36	0,35	23,96	0,35	7,29	0,69	100,00
32,50	25,00	35,78	0,00	0,92	0,00	100,00
83,65	0,94	7,86	0,31	7,23	0,00	100,00
60,81	0,00	39,19	0,00	0,00	0,00	100,00
84,46	0,00	15,54	0,00	0,00	0,00	100,00
100,00	0,00	0,00	0,00	0,00	0,00	93,10
53,95	0,00	40,79	1,32	2,63	1,32	100,00
48,09	1,09	44,75	1,10	2,21	2,21	99,45
38,75	24,69	20,82	0,74	2,23	0,74	99,63
90,77	0,00	7,44	0,00	1,79	0,00	100,00
60,00	0,00	0,00	40,00	0,00	0,00	100,00
71,74	9,78	17,39	0,00	1,09	0,00	100,00
60,00	20,59	16,47	0,59	2,35	0,00	100,00

67,62	12,38	16,19	0,95	2,38	0,48	100,00
20,34	0,00	36,84	0,00	0,00	0,00	84,21
65,60	16,40	14,40	0,40	3,20	0,00	99,60
63,77	0,00	21,74	0,00	13,77	0,72	97,83
89,79	5,11	4,26	0,00	0,85	0,00	100,00
73,42	3,80	21,52	0,00	1,27	0,00	100,00
44,34	36,79	16,11	0,00	1,90	0,47	100,00
44,12	39,71	13,43	0,00	1,49	0,00	100,00
66,43	23,78	9,09	0,00	0,70	0,00	100,00
67,74	20,97	8,60	0,54	2,15	0,00	100,00
73,36	18,22	7,94	0,00	0,47	0,00	100,00
85,12	7,74	6,55	0,00	0,60	0,00	100,00
49,11	32,59	16,52	0,00	1,79	0,00	100,00
56,28	24,04	15,25	0,56	0,56	0,56	99,44
62,99	17,53	13,64	1,30	4,55	0,00	99,35
68,14	18,63	11,27	0,49	0,98	0,49	100,00
60,82	26,32	11,11	0,00	1,75	0,00	100,00
69,92	17,29	12,78	0,00	0,00	0,00	100,00
71,74	18,70	9,13	0,00	0,43	0,00	100,00
84,95	7,77	6,80	0,49	0,00	0,00	100,00
53,42	15,07	25,00	0,49	0,98	0,00	100,00
21,01	57,61	17,75	0,00	3,62	0,00	100,00
44,83	25,43	27,71	0,87	0,43	0,43	99,57
52,59	21,91	20,00	0,40	4,80	0,00	100,00
49,79	24,46	23,18	0,43	2,15	0,00	100,00
11,03	68,97	17,24	0,69	2,07	0,00	100,00
30,23	34,11	31,01	0,00	4,65	0,00	100,00
30,95	37,30	24,79	0,00	1,71	0,00	100,00
70,09	2,85	21,08	0,28	5,70	0,00	99,72
55,10	21,43	19,39	1,02	3,06	0,00	100,00
0,00	0,00	0,00	0,00	0,00	0,00	0,00
67,12	16,03	15,26	0,00	1,36	0,00	99,73
0,00	0,00	0,00	0,00	0,00	0,00	0,00
77,27	3,90	14,94	0,00	2,60	1,30	100,00
36,36	29,55	31,82	0,00	2,27	0,00	100,00
65,55	15,97	13,45	0,00	5,04	0,00	100,00
60,10	5,56	32,83	0,00	1,52	0,00	100,00
55,04	31,78	12,40	0,00	0,00	0,78	100,00
41,05	40,61	17,90	0,00	0,44	0,00	100,00
61,29	19,35	19,35	0,00	0,00	0,00	100,00
42,53	35,06	17,24	0,00	4,60	0,57	100,00
40,80	36,00	22,40	0,00	0,80	0,00	100,00
42,11	39,10	10,53	0,00	7,52	0,75	100,00
66,47	13,53	18,24	0,59	1,18	0,00	100,00
66,25	0,00	23,75	0,00	10,00	0,00	100,00
60,75	13,08	22,43	0,00	3,74	0,00	100,00
48,00	32,00	16,00	0,00	4,00	0,00	100,00
41,81	30,70	24,85	0,29	2,34	0,00	100,00
8,39	0,35	12,50	0,00	9,38	0,00	100,00
67,28	11,40	19,17	0,38	0,00	0,00	100,00

69,31	6,93	20,79	0,00	2,97	0,00	100,00
66,82	10,75	21,03	0,47	0,93	0,00	100,00
63,13	12,44	22,58	0,00	0,92	0,92	100,00
68,66	4,48	20,90	0,00	5,97	0,00	100,00
66,20	11,57	15,74	0,46	5,56	0,46	100,00
84,82	2,68	9,82	0,89	1,79	0,00	100,00
99,10	0,00	0,90	0,00	0,00	0,00	99,10
34,05	20,79	43,37	0,00	1,79	0,00	100,00
53,55	14,22	22,75	0,00	7,11	2,37	99,05
30,59	32,94	29,11	0,63	1,90	0,00	100,00
0,00	0,00	0,00	0,00	0,00	0,00	0,00
71,50	3,74	16,82	0,00	7,48	0,47	100,00
73,68	0,00	10,53	0,00	15,79	0,00	100,00
73,81	2,38	14,29	0,00	7,14	2,38	100,00
78,31	2,41	14,46	0,00	4,82	0,00	100,00
77,78	11,11	11,11	0,00	0,00	0,00	100,00
66,05	24,19	9,77	0,00	0,00	0,00	100,00
86,59	12,20	1,22	0,00	0,00	0,00	98,78
99,58	0,00	0,00	0,00	0,42	0,00	100,00
97,76	0,00	2,24	0,00	0,00	0,00	100,00
97,35	0,00	2,65	0,00	0,00	0,00	100,00
99,45	0,00	0,55	0,00	0,00	0,00	100,00
39,06	44,53	15,63	0,00	0,78	0,00	100,00
46,39	45,18	8,43	0,00	0,00	0,00	100,00
39,18	43,27	16,96	0,00	0,58	0,00	100,00
44,67	34,67	18,00	0,00	2,67	0,00	100,00
53,54	23,23	22,73	0,00	0,51	0,00	100,00
42,86	38,29	15,43	0,00	3,43	0,00	100,00
41,45	34,87	19,08	0,66	3,95	0,00	100,00
58,79	24,73	14,84	0,00	1,65	0,00	98,35
46,27	35,29	17,65	0,39	0,39	0,00	98,82
49,09	27,88	18,18	0,00	4,24	0,61	100,00
58,64	19,75	20,99	0,00	0,62	0,00	100,00
70,59	14,97	11,76	1,07	1,60	0,00	100,00
65,38	6,59	17,03	0,00	10,44	0,55	100,00
57,30	10,49	21,46	0,00	9,20	0,00	100,00
70,43	0,00	18,82	1,61	9,14	0,00	100,00
59,19	1,35	26,46	0,45	12,56	0,00	100,00
4,40	95,60	0,00	0,00	0,00	0,00	100,00
58,62	0,43	26,84	0,43	13,42	0,00	100,00
58,93	0,00	21,43	1,79	17,86	0,00	100,00
42,86	28,57	0,00	16,67	0,00	0,00	100,00
84,06	1,45	11,59	0,00	2,90	0,00	100,00
49,74	3,11	17,61	8,18	9,43	0,63	82,39
0,00	0,00	0,00	0,00	0,00	0,00	0,00
65,00	0,00	31,58	0,00	0,00	0,00	100,00
56,96	2,53	13,92	0,00	26,58	0,00	100,00
0,00	0,00	0,00	0,00	0,00	0,00	0,00
72,50	3,33	15,83	2,50	5,83	0,00	99,17
76,04	9,90	8,02	2,14	1,60	0,00	98,40

0,00	0,00	0,00	0,00	0,00	0,00	0,00
59,62	3,85	23,08	9,62	3,85	0,00	98,08
86,96	0,00	10,87	0,00	2,17	0,00	97,83
40,00	30,00	10,00	10,00	10,00	0,00	100,00
51,03	14,14	20,69	0,34	11,03	2,76	100,00
80,88	7,35	11,76	0,00	0,00	0,00	100,00
90,00	0,00	3,91	0,00	3,04	3,04	100,00
59,27	15,73	19,76	0,00	5,24	0,00	100,00
66,36	12,73	19,09	0,00	1,82	0,00	100,00
75,27	4,95	16,48	0,00	2,75	0,55	100,00
71,30	7,87	19,91	0,46	0,46	0,00	100,00
62,76	11,72	22,07	0,00	3,45	0,00	100,00
61,19	15,53	21,00	0,00	1,83	0,46	100,00
63,33	13,33	10,00	0,00	13,33	0,00	100,00
51,84	26,12	17,50	0,42	2,50	0,00	100,00
36,67	33,89	23,89	0,00	4,44	1,11	100,00
48,48	24,24	24,24	1,52	1,52	0,00	100,00
80,00	0,00	13,33	0,00	6,67	0,00	100,00
66,05	0,00	32,10	0,00	1,85	0,00	100,00
78,57	0,00	7,14	0,00	7,14	7,14	100,00
51,78	18,78	26,40	0,00	3,05	0,00	100,00
61,54	0,00	23,08	0,00	0,00	15,38	100,00
58,13	28,75	11,25	0,00	1,88	0,00	100,00
0,00	0,00	0,00	0,00	0,00	0,00	0,00
90,36	0,00	6,02	0,00	1,20	2,41	100,00
65,22	0,00	8,70	0,00	8,70	17,39	100,00
74,24	0,76	14,39	0,00	10,61	0,00	100,00
77,05	1,64	10,71	0,89	2,68	0,00	100,00
0,00	0,00	0,00	0,00	0,00	0,00	0,00
0,00	0,00	0,00	0,00	0,00	0,00	0,00
77,67	6,80	12,62	0,00	1,94	0,97	100,00
0,00	0,00	0,00	0,00	0,00	0,00	0,00
53,71	1,14	40,57	0,57	4,00	0,00	100,00
67,81	0,31	28,75	0,31	2,81	0,00	100,00
58,31	0,00	33,62	0,56	7,06	0,28	99,44
82,12	1,68	15,08	0,00	1,12	0,00	100,00
42,54	47,98	6,87	0,81	1,62	0,00	98,38
67,46	0,00	21,12	0,40	10,76	0,00	100,00
68,10	0,00	26,29	0,00	5,60	0,00	100,00
58,25	0,35	26,67	0,00	14,74	0,00	100,00
54,26	1,77	38,30	0,00	5,67	0,00	99,29
69,55	1,38	22,57	0,00	5,90	0,35	100,00
68,11	0,54	22,16	0,00	9,19	0,00	99,46
63,60	0,80	27,20	0,00	8,40	0,00	100,00
78,91	0,39	18,36	0,00	2,34	0,00	100,00
80,39	0,00	6,56	0,00	12,79	0,00	100,00
96,56	0,00	2,19	0,00	1,25	0,00	100,00
88,42	0,28	4,80	0,00	3,95	2,54	100,00
81,56	0,00	8,99	0,00	8,99	0,00	100,00
94,71	0,00	4,46	0,00	0,84	0,00	100,00

93,81	0,88	5,31	0,00	0,00	0,00	100,00
69,12	0,00	24,51	0,00	6,37	0,00	100,00
61,67	0,83	26,67	0,42	10,42	0,00	100,00
73,47	1,36	19,73	0,00	5,44	0,00	100,00
60,85	2,55	34,89	0,00	1,70	0,00	100,00
55,61	1,87	29,91	0,00	12,62	0,00	100,00
53,33	2,05	38,97	0,51	5,13	0,00	100,00
66,99	2,56	27,56	0,00	2,88	0,00	100,00
70,48	0,37	28,04	0,00	1,11	0,00	99,26
51,01	18,24	23,05	0,00	3,90	0,35	99,29
55,94	0,00	31,88	0,00	12,19	0,00	99,06
94,83	0,43	2,37	0,22	2,16	0,00	98,71
68,90	0,39	21,65	0,39	7,87	0,79	100,00
76,87	0,68	7,82	0,34	13,27	1,02	100,00
72,97	13,51	10,04	0,00	3,09	0,39	100,00
74,51	9,15	8,82	0,00	5,56	1,96	100,00
89,84	0,27	8,24	0,00	1,65	0,00	100,00
81,08	0,00	11,11	0,00	5,56	0,00	100,00
89,60	0,40	8,84	0,00	0,80	0,00	100,00
79,47	0,00	15,26	0,00	4,74	0,53	99,47
89,77	0,00	10,23	0,00	0,00	0,00	97,73
51,37	0,55	26,78	2,19	19,13	0,00	100,00
66,76	0,57	24,64	0,86	7,16	0,00	100,00
71,11	0,00	25,19	0,00	3,33	0,37	100,00
59,45	2,76	34,78	0,00	2,37	0,40	100,00
86,02	3,76	5,38	0,00	4,84	0,00	100,00
87,43	0,00	9,84	0,00	1,64	1,09	100,00
64,29	0,00	28,57	0,71	5,71	0,71	99,29
62,00	0,00	24,80	0,00	13,20	0,00	100,00
63,79	0,00	34,48	0,00	1,72	0,00	100,00
62,66	0,43	25,32	0,00	11,59	0,00	100,00
0,00	0,00	0,00	0,00	0,00	0,00	0,00
59,16	6,11	16,79	0,00	10,71	0,00	100,00
75,00	17,01	6,94	0,00	1,04	0,00	100,00
60,32	22,75	14,81	0,00	2,12	0,00	100,00
66,78	13,78	17,31	0,00	1,41	0,71	100,00
61,62	4,58	23,94	0,35	6,34	3,17	100,00
60,70	12,84	25,68	0,00	0,78	0,00	100,00
57,33	24,89	16,44	0,00	0,89	0,44	100,00
40,81	46,73	9,66	0,00	2,80	0,00	100,00
38,73	45,95	9,86	0,00	5,46	0,00	99,65
73,61	1,39	19,44	0,46	5,09	0,00	99,54
86,47	0,48	6,31	0,00	6,31	0,00	100,00
59,22	0,00	18,82	0,78	21,18	0,00	98,82
35,21	0,00	35,21	1,41	28,17	0,00	98,59
77,11	1,61	10,84	0,40	10,04	0,00	100,00
60,49	1,23	19,14	0,00	19,14	0,00	100,00
90,26	0,00	7,14	0,00	2,60	0,00	100,00
76,64	1,46	13,87	0,00	8,03	0,00	100,00
70,49	0,00	18,03	0,00	11,48	0,00	100,00

63,67	0,32	22,83	0,00	12,86	0,32	100,00
75,68	2,40	20,78	0,00	0,90	0,00	99,70
78,64	0,00	13,59	0,49	7,28	0,00	100,00
73,52	4,11	10,96	0,00	11,42	0,00	100,00
76,37	2,11	10,97	0,00	9,28	1,27	100,00
80,07	4,90	9,80	0,00	5,23	0,00	100,00
70,91	5,54	10,53	0,00	12,74	0,28	100,00
56,25	0,42	18,33	2,08	22,92	0,00	100,00
43,14	2,01	26,42	0,33	27,42	0,67	100,00
63,94	0,37	23,05	0,00	12,27	0,37	100,00
67,54	0,00	24,12	0,00	7,89	0,44	100,00
70,97	0,00	10,39	0,00	18,64	0,00	100,00
89,87	0,00	5,91	0,00	4,22	0,00	100,00
72,44	0,71	19,43	0,00	6,71	0,71	99,65
0,00	0,00	0,00	0,00	0,00	0,00	0,00
73,15	1,17	14,06	0,39	10,55	0,39	100,00
78,45	0,43	14,66	0,43	6,03	0,00	100,00
82,37	0,00	15,83	0,00	1,80	0,00	100,00
0,00	0,00	0,00	0,00	0,00	0,00	0,00
65,58	0,36	31,64	0,00	2,18	0,00	100,00
57,62	11,26	22,52	0,00	8,61	0,00	100,00
36,66	32,17	27,93	0,00	3,24	0,00	100,00
70,49	3,28	18,85	0,00	7,38	0,00	100,00
77,96	5,59	9,21	0,99	6,25	0,00	100,00
75,00	7,29	10,42	0,00	7,29	0,00	100,00
77,40	2,05	13,70	0,00	6,85	0,00	99,32
65,50	5,00	26,13	0,00	3,02	0,00	100,00
75,93	11,53	10,17	0,00	2,03	0,34	100,00
62,80	0,30	28,66	0,30	6,40	1,52	100,00
65,67	1,12	23,88	0,00	6,72	2,61	100,00
60,22	0,00	28,73	0,55	10,50	0,00	100,00
63,36	13,74	21,37	0,38	0,76	0,38	100,00
55,24	2,80	27,27	1,40	9,09	4,20	100,00
57,31	1,19	29,18	0,00	7,30	0,00	100,00
62,54	12,72	19,08	0,35	4,95	0,35	99,65
54,57	9,80	24,16	0,22	10,96	0,00	99,55
56,63	1,20	28,18	0,30	13,33	0,00	100,00
75,72	0,00	18,21	0,00	5,75	0,32	99,68
54,52	15,96	17,77	0,00	11,45	0,30	100,00
75,00	1,79	12,50	0,00	10,71	0,00	98,21
84,72	3,02	10,00	0,38	1,89	0,00	99,62
81,68	2,97	9,90	0,00	5,45	0,00	99,01
90,80	0,00	6,80	0,00	2,40	0,00	100,00
70,18	0,70	22,81	0,35	5,26	0,70	100,00
40,00	0,00	40,00	0,00	20,00	0,00	100,00
82,35	0,00	14,71	0,00	2,94	0,00	100,00
70,25	0,00	15,19	0,63	13,92	0,00	99,37
79,75	1,23	13,50	0,00	4,91	0,61	100,00
72,63	0,00	16,20	0,00	11,17	0,00	100,00
64,39	0,00	25,37	0,00	10,24	0,00	100,00

75,38	0,00	19,49	0,00	5,13	0,00	100,00
72,89	0,90	18,98	0,00	7,23	0,00	100,00
70,52	0,00	27,34	0,00	1,12	0,75	100,00
74,33	0,00	18,67	0,00	7,00	0,00	100,00
73,68	0,35	17,19	1,05	7,72	0,00	99,65
66,18	0,00	31,73	0,00	1,85	0,00	100,00
59,45	0,61	21,65	0,00	16,46	1,83	100,00
67,86	0,00	32,14	0,00	0,00	0,00	100,00
58,74	0,00	27,51	0,37	12,27	1,12	99,63
78,49	0,00	21,51	0,00	0,00	0,00	100,00
53,77	0,94	33,02	0,00	12,26	0,00	100,00
62,78	0,00	37,22	0,00	0,00	0,00	100,00
70,04	0,00	29,96	0,00	0,00	0,00	100,00
65,84	6,41	22,06	0,71	4,98	0,00	100,00
69,41	7,18	21,28	0,00	2,13	0,00	100,00
53,18	26,11	14,33	0,00	6,37	0,00	100,00
62,35	5,04	26,14	0,00	6,47	0,00	100,00
0,00	0,00	0,00	0,00	0,00	0,00	0,00
53,44	6,48	31,17	0,81	8,10	0,00	100,00
71,08	13,65	12,05	0,00	3,21	0,00	99,20
62,69	0,50	23,38	0,00	12,94	0,50	100,00
63,10	3,57	25,60	0,60	7,14	0,00	100,00
64,14	1,01	28,93	0,51	5,08	0,00	100,00
66,04	1,13	24,53	1,13	6,79	0,38	100,00
61,59	2,08	23,19	0,38	6,46	0,00	100,00
61,54	0,70	30,07	0,00	7,69	0,00	100,00
73,71	1,71	22,29	0,00	2,29	0,00	98,86
60,69	0,29	32,08	0,00	6,94	0,00	100,00
57,97	3,29	34,43	0,25	4,05	0,00	99,75
44,82	11,37	24,75	0,67	18,06	0,33	99,67
57,14	2,96	21,67	0,00	18,23	0,00	99,51
55,88	1,84	34,19	0,74	7,35	0,00	100,00
58,37	0,00	31,02	0,00	10,61	0,00	99,18
86,69	0,97	8,44	1,62	1,95	0,32	99,68
68,75	0,63	25,94	0,00	3,75	0,94	100,00
81,82	0,00	12,73	0,00	5,45	0,00	100,00
61,57	2,75	20,00	0,00	15,29	0,39	98,82
14,56	82,20	1,62	0,00	1,62	0,00	99,35
76,85	0,89	18,10	0,00	4,15	0,00	99,70
58,82	0,78	30,20	0,00	10,20	0,00	99,61
79,56	7,86	7,23	0,63	4,72	0,00	100,00
75,45	0,91	19,09	0,91	3,64	0,00	100,00
71,10	1,53	24,55	0,51	2,30	0,00	98,72
59,82	18,10	19,63	0,31	2,15	0,00	100,00
67,09	12,14	16,93	0,00	3,83	0,00	100,00
87,32	0,35	7,04	0,00	5,28	0,00	99,65
95,93	0,00	3,49	0,00	0,58	0,00	100,00
45,00	0,38	34,12	0,39	19,22	0,00	100,00
70,14	0,00	29,86	0,00	0,00	0,00	100,00
64,17	1,67	24,17	0,00	10,00	0,00	100,00

66,67	0,00	15,79	0,00	17,54	0,00	100,00
75,64	0,96	15,06	0,00	8,01	0,32	100,00
51,50	0,86	25,32	1,29	21,03	0,00	100,00
0,00	0,00	0,00	0,00	0,00	0,00	0,00
75,58	1,84	15,67	0,46	6,45	0,00	100,00
50,81	3,78	25,95	1,08	18,38	0,00	100,00
69,42	0,00	24,27	0,00	6,31	0,00	100,00
61,97	13,03	19,01	0,00	5,99	0,00	99,65
57,14	5,61	26,53	0,00	10,71	0,00	99,49
71,86	1,20	10,78	0,60	14,97	0,60	100,00
66,19	4,63	23,21	0,00	5,71	0,00	100,00
70,46	16,37	10,68	0,71	1,42	0,36	100,00
59,74	2,16	22,42	0,45	13,00	0,00	100,00
70,72	0,55	24,31	0,00	4,42	0,00	100,00
63,48	2,48	32,62	0,00	1,06	0,35	100,00
70,75	0,00	21,09	0,00	8,16	0,00	98,64
82,91	1,51	9,55	0,50	5,53	0,00	97,99
82,93	7,32	9,02	0,00	0,00	0,00	100,00
83,43	0,00	8,43	1,20	6,93	0,00	99,10
82,80	1,17	10,20	0,00	5,83	0,00	99,71
61,90	0,00	23,81	4,76	9,52	0,00	100,00
90,38	0,00	8,65	0,00	0,96	0,00	100,00
77,22	0,00	18,99	0,00	3,80	0,00	94,94
63,41	0,00	34,15	0,00	1,83	0,61	100,00
45,59	1,84	25,83	1,11	25,46	0,00	100,00
41,94	0,54	26,49	0,00	30,81	0,00	100,00
62,27	0,00	30,71	0,37	5,24	0,00	100,00
61,23	0,44	24,67	0,00	13,66	0,00	100,00
92,00	0,00	6,67	0,00	1,33	0,00	100,00
65,35	0,30	17,02	0,00	17,33	0,00	100,00
58,68	0,00	40,12	0,60	0,60	0,00	100,00
66,53	0,00	28,39	0,00	5,08	0,00	100,00
34,50	0,58	54,97	0,58	5,85	3,51	100,00
47,37	0,00	43,86	0,00	8,77	0,00	100,00
45,39	0,00	40,43	0,71	13,48	0,00	100,00
36,72	0,00	31,93	1,96	24,65	0,00	100,00
64,91	1,05	27,02	1,05	5,96	0,00	100,00
66,19	0,95	30,48	0,48	1,90	0,00	100,00
65,28	1,85	17,59	0,46	14,81	0,00	100,00
74,25	0,60	17,66	0,00	6,59	0,90	100,00
75,19	0,00	20,16	0,00	4,65	0,00	100,00
54,20	0,76	29,77	0,76	14,50	0,00	100,00
66,30	1,83	10,99	0,00	8,79	12,09	100,00
50,77	1,54	26,64	0,39	20,46	0,00	100,00
78,43	0,00	16,67	0,00	4,25	0,65	99,02
50,23	1,36	38,46	0,00	9,50	0,45	100,00
40,52	44,24	6,69	1,49	4,83	2,23	98,88
59,35	12,76	20,18	0,59	6,82	0,30	100,00
90,00	0,00	5,36	0,00	4,64	0,00	100,00
97,13	0,00	2,15	0,00	0,36	0,36	100,00

41,81	0,00	46,34	1,39	10,45	0,00	100,00
49,01	0,28	28,25	0,00	22,32	0,00	100,00
47,44	0,00	31,41	0,00	21,15	0,00	100,00
69,40	0,75	20,90	0,00	8,96	0,00	100,00
67,42	0,45	18,55	1,36	12,22	0,00	100,00
55,12	0,00	29,68	0,00	15,19	0,00	98,59
67,60	0,56	30,73	0,00	1,12	0,00	98,88
29,51	0,41	55,75	0,00	11,95	0,00	100,00
43,26	0,00	39,85	1,50	12,03	0,75	100,00
30,00	0,00	63,33	0,67	6,00	0,00	100,00
23,79	0,40	54,46	1,49	14,36	0,00	100,00
36,79	0,00	61,79	0,00	1,42	0,00	100,00
0,00	0,00	0,00	0,00	0,00	0,00	0,00
55,17	0,00	3,45	6,90	34,48	0,00	44,83
64,94	1,95	18,51	0,65	13,96	0,00	100,00
84,44	0,00	11,85	0,00	3,70	0,00	99,26
67,49	1,23	25,93	0,00	5,35	0,00	99,59
61,00	0,77	29,18	0,00	8,56	0,00	100,00
88,24	1,60	2,69	0,00	6,99	0,00	98,92
71,57	0,00	25,49	0,49	2,45	0,00	99,51
78,01	1,42	4,29	0,00	15,71	0,00	97,86
52,69	0,00	32,80	1,08	13,44	0,00	100,00
54,88	0,47	19,07	0,00	25,58	0,00	100,00
58,03	0,52	30,57	0,52	10,36	0,00	100,00
71,30	0,00	21,97	0,00	6,73	0,00	100,00
81,40	0,00	14,53	0,00	4,07	0,00	100,00
47,98	0,00	32,66	0,00	19,35	0,00	99,60
75,00	0,41	21,72	0,00	2,87	0,00	100,00
62,67	2,67	22,67	0,00	12,00	0,00	98,67
51,72	1,72	23,56	0,00	22,41	0,57	100,00
63,96	0,00	22,26	0,35	13,43	0,00	100,00
61,86	0,00	24,02	0,00	14,11	0,00	100,00
51,85	1,14	30,48	0,00	16,24	0,28	99,72
59,21	0,00	23,87	0,00	16,62	0,30	100,00
47,46	0,00	28,24	0,00	18,60	0,33	100,00
80,27	0,00	17,04	0,00	2,69	0,00	100,00
68,87	0,00	19,81	0,00	11,32	0,00	100,00
74,07	0,00	17,46	0,00	7,41	1,06	100,00
70,45	0,00	13,64	0,00	15,91	0,00	100,00
38,24	22,88	4,58	5,56	28,43	0,33	100,00
68,35	16,46	15,19	0,00	0,00	0,00	100,00
0,00	0,00	0,00	0,00	0,00	0,00	0,00
58,22	0,00	32,39	0,00	9,39	0,00	100,00
66,89	2,03	15,54	0,00	15,54	0,00	100,00
74,23	1,03	23,71	0,00	1,03	0,00	100,00
2,73	95,22	0,34	0,00	0,68	1,02	97,61
95,65	1,09	0,00	0,00	3,26	0,00	100,00
97,92	0,00	1,04	0,00	1,04	0,00	100,00
76,79	0,00	7,14	0,00	16,07	0,00	100,00
90,11	1,10	8,79	0,00	0,00	0,00	100,00

85,37	0,35	10,45	0,35	3,48	0,00	100,00
59,60	0,00	23,23	0,00	17,17	0,00	100,00
80,93	0,00	17,21	0,00	1,40	0,47	100,00
63,64	3,03	30,30	0,00	3,03	0,00	100,00
79,00	1,00	8,33	0,33	11,00	0,33	99,67
6,14	84,21	7,46	0,00	1,75	0,44	99,56
44,83	0,00	51,72	0,00	3,45	0,00	100,00
60,67	2,44	35,98	0,00	0,91	0,00	100,00
4,10	0,82	0,82	0,41	93,85	0,00	100,00
60,75	1,40	23,47	0,00	14,08	0,00	100,00
3,11	0,00	0,62	0,00	96,27	0,00	100,00
75,41	3,28	11,11	0,00	0,00	0,00	100,00
87,50	3,13	9,38	0,00	0,00	0,00	100,00
84,62	0,00	10,99	0,00	4,40	0,00	100,00
38,06	0,65	11,61	0,00	48,39	1,29	98,71
81,94	0,00	18,06	0,00	0,00	0,00	98,71
80,10	7,33	8,38	0,00	3,66	0,52	100,00
55,00	0,00	38,33	0,00	6,67	0,00	99,17
79,02	0,00	14,63	0,49	5,85	0,00	100,00
23,15	65,77	9,73	0,00	1,01	0,34	100,00
83,91	1,72	6,90	0,00	7,47	0,00	100,00
94,48	4,14	0,00	0,00	1,38	0,00	100,00
89,11	1,98	7,92	0,00	0,99	0,00	100,00
64,31	5,95	24,44	0,38	3,38	0,75	100,00
62,20	12,44	20,10	0,00	5,26	0,00	100,00
38,58	50,19	7,12	0,00	4,12	0,00	100,00
84,44	2,22	4,44	0,00	2,22	6,67	100,00
57,63	0,00	33,90	0,00	8,47	0,00	100,00
78,98	0,00	6,67	0,33	5,33	0,00	100,00
84,62	0,85	10,26	0,00	4,27	0,00	100,00
80,45	1,00	15,54	0,25	2,76	0,00	100,00
72,22	0,00	27,78	0,00	0,00	0,00	100,00
93,10	0,00	6,90	0,00	0,00	0,00	100,00
87,04	0,00	7,41	0,00	5,56	0,00	100,00
84,26	0,00	14,81	0,00	0,93	0,00	100,00
72,05	1,45	20,24	0,48	5,78	0,00	99,76
84,46	0,40	5,98	0,00	9,16	0,00	99,60
63,64	0,61	23,64	0,00	12,12	0,00	100,00
74,29	2,86	17,14	0,00	5,71	0,00	100,00
84,27	0,00	14,61	0,00	1,12	0,00	100,00
9,25	0,00	0,00	0,00	87,86	2,89	100,00
60,26	0,66	17,22	0,66	21,19	0,00	100,00
88,54	0,00	10,42	0,00	1,04	0,00	100,00
88,70	0,00	9,60	0,00	1,13	0,56	99,44
80,00	6,67	13,33	0,00	0,00	0,00	100,00
0,00	0,00	0,00	0,00	0,00	0,00	0,00
3,98	0,00	0,00	1,14	2,84	92,05	98,86
67,30	0,00	19,91	0,00	12,80	0,00	99,53
55,50	0,00	25,00	1,50	17,50	0,50	100,00
90,00	0,00	0,00	0,00	0,00	10,00	100,00

64,08	6,80	24,27	0,00	4,85	0,00	100,00
72,27	0,00	26,89	0,00	0,00	0,84	100,00
68,42	2,63	21,05	0,00	1,32	6,58	100,00
74,29	2,51	12,85	0,00	10,34	0,00	98,12
72,55	5,23	22,22	0,00	0,00	0,00	100,00
89,58	0,00	6,95	0,00	3,47	0,00	100,00
98,44	1,56	0,00	0,00	0,00	0,00	100,00
10,80	81,20	7,60	0,00	0,40	0,00	97,20
67,67	27,07	4,51	0,00	0,00	0,75	100,00
74,65	0,00	8,45	0,00	16,90	0,00	100,00
73,08	0,00	7,69	0,00	19,23	0,00	100,00
78,50	0,00	11,32	0,94	8,49	0,00	100,00
70,37	0,00	20,37	0,00	9,26	0,00	100,00
73,00	0,00	22,50	0,50	4,00	0,00	100,00
68,39	0,00	24,71	0,00	6,32	0,57	98,85
86,16	1,26	8,18	0,00	4,40	0,00	99,37
81,33	2,00	6,67	0,00	10,00	0,00	100,00
27,56	44,89	18,22	0,44	8,89	0,00	99,11
71,51	0,85	12,25	0,00	15,38	0,00	100,00
75,00	0,68	11,49	0,00	12,84	0,00	100,00
78,31	3,68	2,94	0,00	14,34	0,74	99,63
94,74	1,75	1,75	0,00	1,75	0,00	99,42
70,67	1,78	16,89	0,00	10,67	0,00	99,56
73,29	1,03	16,10	0,00	9,59	0,00	99,32
68,87	4,64	13,91	0,00	12,58	0,00	100,00
100,00	0,00	0,00	0,00	0,00	0,00	100,00
56,14	0,00	24,56	0,00	19,30	0,00	100,00
64,63	4,08	27,89	0,00	2,04	1,36	99,32
73,06	4,80	18,45	0,37	3,32	0,00	100,00
59,78	1,68	26,74	0,00	9,30	0,00	100,00
58,49	0,00	32,08	3,77	5,66	0,00	100,00
64,91	0,00	33,33	0,00	1,75	0,00	100,00
62,50	0,00	37,50	0,00	0,00	0,00	100,00
60,00	0,00	21,43	0,00	18,57	0,00	100,00
64,05	0,65	26,14	0,00	9,15	0,00	100,00
78,57	1,79	14,29	0,00	4,76	0,60	99,40
59,57	2,13	26,95	0,00	11,35	0,00	100,00
61,63	1,22	32,24	0,00	4,90	0,00	99,59
72,58	1,61	12,90	0,00	12,90	0,00	100,00
73,48	1,66	14,64	0,28	9,94	0,00	100,00
71,21	0,00	6,06	0,00	22,73	0,00	100,00
85,96	0,00	0,00	0,00	14,04	0,00	100,00
48,48	0,00	24,24	1,52	25,76	0,00	100,00
80,00	2,22	13,33	0,00	2,22	2,22	100,00
91,09	0,00	2,97	0,00	5,94	0,00	100,00
51,61	0,00	38,71	3,23	6,45	0,00	100,00
63,64	0,00	33,77	0,00	2,60	0,00	100,00
69,44	0,00	26,39	0,00	4,17	0,00	100,00
72,62	0,00	23,81	0,00	3,57	0,00	100,00
57,37	0,00	25,26	1,05	16,32	0,00	95,26

55,05	0,00	28,65	0,00	12,43	0,00	100,00
45,60	0,00	27,08	0,00	27,08	0,00	100,00
73,68	0,00	23,31	0,00	3,01	0,00	99,25
80,90	0,00	14,61	0,00	4,49	0,00	100,00
92,31	0,00	7,69	0,00	0,00	0,00	100,00
72,88	0,00	15,25	0,00	11,86	0,00	100,00
0,00	0,00	0,00	0,00	0,00	0,00	0,00
60,00	6,50	15,50	0,00	18,00	0,00	100,00
65,42	0,00	10,28	0,00	24,30	0,00	99,07
0,00	0,00	0,00	0,00	0,00	0,00	0,00
44,29	1,43	13,16	1,32	1,32	0,00	98,68
81,48	0,00	8,47	0,00	10,05	0,00	98,41
58,38	0,00	40,00	0,00	1,62	0,00	100,00
84,03	0,00	8,33	0,00	7,64	0,00	100,00
75,38	3,08	10,77	0,00	10,77	0,00	100,00
85,90	0,43	10,26	0,00	3,42	0,00	99,57
77,78	0,00	16,67	0,00	5,56	0,00	100,00
59,92	0,00	35,54	0,00	4,55	0,00	97,52
60,70	0,00	30,88	0,00	8,42	0,00	100,00
94,62	0,00	4,04	0,00	1,35	0,00	100,00
94,26	0,00	4,92	0,00	0,82	0,00	100,00
93,81	0,00	5,90	0,00	0,29	0,00	100,00
74,80	0,00	18,90	0,00	6,30	0,00	100,00
87,43	1,80	6,59	0,60	2,40	1,20	100,00
85,84	0,00	11,42	0,00	2,74	0,00	100,00
48,89	0,00	46,67	2,22	2,22	0,00	100,00
42,50	0,00	45,00	0,00	2,50	10,00	100,00
52,21	17,26	19,72	0,92	6,88	0,46	100,00
52,49	24,43	16,74	0,45	4,52	1,36	98,19
85,11	0,00	12,77	0,00	2,13	0,00	100,00
84,36	0,56	12,85	0,00	1,68	0,56	98,32
75,93	0,00	1,85	1,85	20,37	0,00	100,00
60,22	0,00	19,35	1,08	19,35	0,00	100,00
77,39	0,00	14,78	0,87	6,96	0,00	100,00
66,67	1,08	15,05	1,08	16,13	0,00	96,77
88,63	0,78	6,67	0,39	3,53	0,00	94,51
69,08	2,67	24,43	0,38	3,44	0,00	99,62
0,00	0,00	0,00	0,00	0,00	0,00	0,00
88,89	0,00	4,17	0,00	6,94	0,00	100,00
87,50	6,25	6,25	0,00	0,00	0,00	100,00
55,88	17,65	26,47	0,00	0,00	0,00	100,00
82,19	0,00	12,33	0,00	5,48	0,00	100,00
67,57	0,00	29,73	0,00	2,70	0,00	98,65
98,41	0,00	0,00	0,00	1,59	0,00	100,00
98,15	0,00	1,85	0,00	0,00	0,00	100,00
84,62	0,00	10,77	0,00	0,00	4,62	100,00
73,68	0,75	16,28	0,00	6,98	0,00	100,00
0,00	0,00	0,00	0,00	0,00	0,00	0,00
84,62	0,00	15,38	0,00	0,00	0,00	100,00
79,63	0,00	18,52	1,85	0,00	0,00	100,00

0,00	0,00	0,00	0,00	0,00	0,00	0,00
65,62	1,05	25,98	0,00	7,35	0,00	98,95
75,57	0,33	19,87	0,00	3,91	0,33	100,00
66,67	0,82	26,45	0,00	5,37	0,41	100,00
56,76	6,31	14,55	0,91	20,91	0,00	100,00
53,27	0,47	24,41	1,88	19,72	0,00	99,06
73,99	0,00	19,77	0,00	5,23	0,58	100,00
67,31	1,92	21,59	0,00	6,64	0,00	99,67
64,79	1,41	21,13	1,41	11,27	0,00	100,00
78,31	0,60	18,98	0,00	2,11	0,00	100,00
72,32	3,57	21,52	0,45	1,35	0,45	99,55
85,56	0,53	12,37	0,00	1,08	0,00	100,00
54,31	0,43	36,21	0,00	8,62	0,43	100,00
59,32	2,28	29,77	0,00	8,40	0,00	100,00
0,00	0,00	0,00	0,00	0,00	0,00	0,00
66,37	0,88	23,89	0,00	8,85	0,00	99,71
70,00	1,67	20,83	0,00	7,50	0,00	100,00
73,15	1,85	14,35	0,00	10,65	0,00	99,54
70,69	0,00	16,81	0,43	11,64	0,43	99,14
72,25	0,00	23,35	0,44	1,76	2,20	96,48
74,43	0,00	17,35	0,46	7,76	0,00	100,00
83,26	0,00	13,07	0,00	3,44	0,23	99,54
69,20	1,90	10,65	0,38	16,35	1,52	99,62
92,83	1,43	4,68	0,00	0,72	0,00	100,00
56,41	0,85	26,50	0,00	16,24	0,00	100,00
58,54	0,61	20,73	0,00	20,12	0,00	100,00
47,73	35,23	10,51	0,00	6,53	0,00	100,00
90,60	0,00	3,50	0,00	1,86	0,23	99,77
72,84	0,43	21,21	0,87	4,33	0,00	100,00
60,82	0,00	28,87	0,00	9,28	1,03	100,00
77,19	0,76	15,97	0,38	4,56	1,14	100,00
67,91	0,31	20,25	0,00	11,53	0,00	99,69
83,96	0,00	12,74	0,00	3,30	0,00	100,00
70,09	0,00	16,82	7,48	5,61	0,00	99,07
72,73	0,00	16,23	0,00	11,04	0,00	100,00
82,33	0,00	10,28	0,00	7,01	0,00	100,00
46,38	1,32	18,09	4,61	29,28	0,33	100,00
29,86	60,28	5,93	0,00	3,39	0,28	100,00
54,05	2,03	22,76	0,00	8,94	0,81	100,00
66,94	0,00	25,28	0,00	7,78	0,00	100,00
70,77	0,00	23,08	0,31	5,85	0,00	99,69
65,10	0,78	29,77	0,00	3,66	0,52	100,00
78,87	0,52	16,80	0,00	3,62	0,00	100,00
74,40	1,79	15,48	0,00	8,33	0,00	100,00
69,29	0,71	7,86	0,71	17,86	3,57	99,29
58,17	1,14	25,48	0,38	14,83	0,00	100,00
71,82	0,00	13,75	0,34	13,06	1,03	99,31
73,00	3,04	15,59	0,76	7,60	0,00	100,00
67,06	0,87	15,16	0,29	16,03	0,58	100,00
72,16	0,78	22,35	0,39	3,92	0,39	100,00

54,47	0,78	22,18	5,45	10,51	6,61	100,00
64,59	0,81	23,78	0,27	10,54	0,00	100,00
72,00	0,40	25,60	0,00	2,00	0,00	100,00
72,00	0,40	21,20	6,40	0,00	0,00	100,00
70,32	0,32	19,35	0,00	10,00	0,00	100,00
56,98	0,00	25,58	3,49	13,95	0,00	100,00
57,78	0,00	22,22	4,00	16,00	0,00	100,00
67,92	5,46	20,48	0,00	5,46	0,68	100,00
80,24	0,00	19,16	0,60	0,00	0,00	100,00
88,55	0,76	3,05	0,00	7,63	0,00	98,47
46,83	0,00	21,21	0,55	31,13	0,28	100,00
65,12	0,58	23,26	0,00	11,05	0,00	98,26
67,12	1,37	15,98	0,46	15,07	0,00	99,54
66,86	0,59	17,75	0,00	14,79	0,00	100,00
77,27	0,91	7,31	0,00	14,16	0,00	100,00
79,13	1,46	8,29	0,00	10,73	0,00	100,00
78,33	0,00	6,70	0,00	14,53	0,00	96,65
81,25	0,48	5,77	8,17	3,85	0,48	1,44
44,73	26,23	10,33	0,70	17,84	0,00	99,77
52,15	0,00	39,71	0,00	8,13	0,00	100,00
61,64	0,00	31,47	0,00	6,90	0,00	100,00
64,41	0,90	26,58	0,45	7,66	0,00	100,00
73,33	0,48	17,62	0,00	6,67	1,90	100,00
74,36	0,00	13,68	0,00	11,97	0,00	100,00
60,85	0,43	22,13	0,43	16,17	0,00	100,00
69,93	0,35	28,32	0,35	1,05	0,00	100,00
78,91	0,00	19,05	0,00	0,00	2,04	99,66
67,50	1,43	21,07	0,71	9,29	0,00	100,00
65,43	0,62	14,81	0,00	19,14	0,00	100,00
52,14	0,00	19,84	0,39	27,63	0,00	100,00
75,00	0,35	24,65	0,00	0,00	0,00	100,00
84,21	0,00	12,44	0,48	2,39	0,48	100,00
55,99	0,30	22,75	0,60	20,36	0,00	100,00
79,13	0,79	13,78	0,00	5,91	0,39	100,00
72,25	2,20	18,94	0,00	6,61	0,00	100,00
65,75	6,85	19,59	0,00	7,56	0,00	100,00
55,45	24,17	17,54	0,00	2,84	0,00	100,00
63,64	19,23	14,34	0,00	2,80	0,00	99,65
64,77	21,24	9,84	0,52	3,63	0,00	100,00
74,90	6,84	8,37	0,00	9,89	0,00	100,00
83,18	1,80	6,61	0,30	5,11	3,00	99,10
78,89	0,00	18,09	0,00	3,02	0,00	100,00
73,87	0,45	23,87	0,00	1,80	0,00	97,75
80,63	1,05	18,32	0,00	0,00	0,00	100,00
57,49	0,97	20,77	0,00	20,77	0,00	100,00
70,29	1,71	17,14	0,00	10,86	0,00	100,00
70,00	1,00	10,00	0,00	19,00	0,00	100,00
72,70	0,95	17,14	0,95	8,25	0,00	100,00
58,55	4,66	25,39	0,52	9,33	1,55	100,00
67,38	6,44	19,31	0,00	6,44	0,43	99,14

65,63	0,00	34,38	0,00	0,00	0,00	100,00
78,18	0,98	12,05	0,00	8,47	0,33	100,00
31,89	1,57	4,33	0,00	61,42	0,79	100,00
82,23	0,57	13,18	0,29	3,44	0,29	98,85
69,28	2,11	15,36	0,30	11,75	1,20	100,00
55,49	1,83	25,00	0,61	13,41	3,66	100,00
69,41	0,91	12,79	0,00	16,89	0,00	99,54
62,77	0,35	22,70	0,35	13,12	0,71	100,00
75,99	1,52	19,15	0,30	2,43	0,61	100,00
57,81	23,44	13,67	0,00	5,08	0,00	100,00
65,81	0,00	25,64	0,00	8,55	0,00	100,00
92,86	0,00	0,00	0,00	7,14	0,00	100,00
72,73	0,00	18,18	0,00	9,09	0,00	100,00
88,89	0,00	7,41	0,00	3,70	0,00	100,00
79,84	1,61	13,71	0,00	4,84	0,00	98,39
70,19	0,00	22,36	0,00	7,45	0,00	99,38
88,46	0,00	11,54	0,00	0,00	0,00	100,00
79,78	1,12	11,36	0,00	6,82	0,00	100,00
67,36	1,04	19,17	0,00	12,44	0,00	100,00
67,27	1,82	24,55	0,00	4,55	1,82	99,09
81,28	0,49	10,34	0,00	7,88	0,00	100,00
62,96	0,00	33,33	0,00	3,70	0,00	100,00
76,13	0,30	14,50	0,30	8,46	0,30	99,70
67,51	7,17	16,88	1,27	6,33	0,84	100,00
80,92	0,58	15,03	0,58	2,89	0,00	100,00
72,95	0,00	25,12	1,45	0,48	0,00	100,00
82,81	0,00	14,06	0,00	3,13	0,00	100,00
0,00	0,00	0,00	0,00	0,00	0,00	0,00
66,85	0,00	19,02	0,00	14,13	0,00	100,00
80,00	0,00	20,00	0,00	0,00	0,00	100,00
71,54	0,81	16,26	0,00	11,38	0,00	100,00
71,88	4,69	14,06	0,00	9,38	0,00	100,00
79,38	0,00	13,13	0,00	6,88	0,63	99,38
79,02	13,29	7,69	0,00	0,00	0,00	100,00
65,22	0,72	22,46	0,00	11,59	0,00	99,28
40,42	30,95	14,53	2,32	10,74	1,05	99,79
52,31	3,08	29,23	0,00	15,38	0,00	100,00
75,76	1,52	18,18	0,00	4,55	0,00	100,00
88,89	0,00	11,11	0,00	0,00	0,00	100,00
77,78	0,00	0,00	0,00	22,22	0,00	100,00
64,29	0,71	31,07	0,00	3,93	0,00	100,00
59,34	0,55	39,01	0,00	1,10	0,00	98,90
62,34	2,60	10,39	0,00	20,78	3,90	100,00
63,89	0,56	15,56	0,00	20,00	0,00	100,00
44,44	0,00	42,22	0,00	13,33	0,00	100,00
91,49	0,00	4,35	0,00	2,17	0,00	100,00
71,60	4,94	13,58	0,00	8,64	1,23	96,30
86,05	0,00	13,95	0,00	0,00	0,00	100,00
86,75	3,61	2,41	1,20	6,02	0,00	100,00
78,35	0,00	15,46	0,00	6,19	0,00	100,00

23,41	69,44	5,56	0,79	0,79	0,00	100,00
76,79	0,00	23,21	0,00	0,00	0,00	100,00
59,84	0,40	33,06	0,00	6,45	0,00	100,00
70,83	0,00	25,00	0,00	4,17	0,00	100,00
0,00	0,00	0,00	0,00	0,00	0,00	0,00
0,00	0,00	0,00	0,00	0,00	0,00	0,00
78,57	0,00	7,14	3,57	10,71	0,00	100,00
80,56	0,00	16,11	2,22	1,11	0,00	100,00
72,50	0,00	20,00	0,00	7,50	0,00	100,00
74,84	1,29	23,23	0,00	0,65	0,00	100,00
81,13	4,40	6,29	0,63	7,55	0,00	100,00
58,14	0,00	6,98	0,00	11,63	23,26	100,00
72,82	3,08	7,18	0,00	16,41	0,51	100,00
62,09	1,96	11,11	0,00	24,84	0,00	100,00
64,73	4,98	7,88	0,00	17,01	5,39	99,59
85,71	1,30	3,95	0,00	7,89	0,00	100,00
38,42	0,49	0,49	0,00	2,46	58,13	99,51
24,90	66,48	6,95	0,14	1,53	0,00	99,86
0,00	0,00	0,00	0,00	0,00	0,00	0,00
65,46	0,00	24,23	1,03	9,28	0,00	100,00
45,22	1,74	38,26	0,00	14,78	0,00	100,00
75,10	0,00	24,10	0,00	0,80	0,00	100,00
58,11	0,00	36,49	0,00	5,41	0,00	100,00
62,89	0,00	17,01	0,52	19,59	0,00	100,00
55,22	0,00	2,99	1,49	40,30	0,00	100,00
56,13	1,42	33,49	1,89	7,08	0,00	99,53
81,47	5,59	11,19	0,35	1,40	0,00	100,00
65,84	0,50	16,34	0,50	16,83	0,00	100,00
51,47	0,00	20,59	0,00	25,00	2,94	99,26
58,70	0,00	30,43	0,00	10,87	0,00	100,00
49,70	0,59	38,46	0,00	11,24	0,00	100,00
87,27	0,00	12,73	0,00	0,00	0,00	100,00
87,44	0,00	8,04	0,00	4,52	0,00	100,00
0,00	0,00	0,00	0,00	0,00	0,00	0,00
49,32	1,83	6,85	0,00	12,79	29,22	99,54
56,36	1,82	14,55	0,00	27,27	0,00	100,00
57,20	2,97	22,03	0,00	17,80	0,00	99,15
69,23	0,77	24,03	0,00	5,43	0,00	100,00
2,21	95,59	2,21	0,00	0,00	0,00	100,00
60,75	0,00	26,34	0,54	12,37	0,00	100,00
81,44	1,03	12,89	0,00	4,64	0,00	100,00
70,59	0,00	21,57	0,00	7,84	0,00	100,00
69,48	5,19	12,21	0,00	0,00	0,00	100,00
83,56	6,85	8,22	0,00	1,37	0,00	100,00
4,08	93,88	1,02	0,00	1,02	0,00	100,00
4,36	95,33	0,00	0,00	0,31	0,00	100,00
0,00	0,00	0,00	0,00	0,00	0,00	0,00
36,17	63,83	0,00	0,00	0,00	0,00	100,00
32,00	0,00	24,83	1,34	8,72	0,67	100,00
81,19	0,94	8,15	0,00	9,40	0,31	100,00

70,45	0,00	19,43	0,00	10,12	0,00	100,00
65,35	0,00	19,74	0,00	14,91	0,00	100,00
75,09	0,36	22,02	0,00	2,53	0,00	100,00
84,62	0,45	12,67	2,26	0,00	0,00	100,00
81,60	1,23	13,50	0,00	3,68	0,00	100,00
54,21	2,11	24,74	0,53	18,42	0,00	100,00
72,40	2,00	21,60	0,00	3,60	0,40	100,00
59,11	16,19	20,24	0,00	4,45	0,00	100,00
72,94	2,35	18,82	0,00	5,88	0,00	100,00
75,00	0,00	17,65	0,00	7,35	0,00	98,53
89,49	0,00	5,80	0,00	4,71	0,00	100,00
83,50	0,00	10,13	0,00	4,42	0,00	100,00
70,99	0,55	5,80	0,00	22,65	0,00	99,72
68,70	1,74	10,00	0,00	18,26	1,30	99,57
91,54	1,15	4,62	0,00	2,69	0,00	100,00
96,68	0,00	1,90	0,00	1,42	0,00	100,00
76,32	0,66	17,43	0,66	4,61	0,33	99,67
71,96	0,00	22,75	0,00	5,29	0,00	99,47
57,61	0,54	22,83	0,00	19,02	0,00	100,00
46,30	0,00	29,63	0,00	24,07	0,00	99,54
65,91	0,28	22,73	0,28	10,80	0,00	100,00
73,56	2,30	15,71	1,15	7,28	0,00	100,00
59,92	11,74	25,10	0,00	2,83	0,40	100,00
67,12	2,25	21,17	0,00	9,46	0,00	97,75
69,96	1,35	24,77	0,00	3,15	0,45	100,00
48,91	21,18	25,00	0,00	3,44	1,25	100,00
73,76	1,52	21,29	0,00	3,04	0,38	100,00
70,57	3,00	23,16	0,27	2,45	0,54	100,00
48,53	20,41	23,13	0,00	7,94	0,00	100,00
54,10	0,82	19,26	2,05	22,54	1,23	100,00
66,27	1,18	12,43	0,00	20,12	0,00	100,00
62,45	1,22	21,43	0,84	11,76	0,42	100,00
75,43	0,25	5,91	0,00	16,26	1,97	99,01
74,19	0,00	22,58	1,08	2,15	0,00	100,00
44,07	0,00	5,76	0,34	49,15	0,68	99,66
72,37	0,00	1,64	0,00	25,99	0,00	100,00
72,95	1,07	10,75	0,00	14,70	0,00	100,00
91,02	0,60	4,82	0,00	3,01	0,00	100,00
0,00	0,00	0,00	0,00	0,00	0,00	0,00
72,66	0,76	20,00	0,00	4,42	0,26	99,74
57,66	0,73	21,90	0,00	19,71	0,00	98,54
38,80	0,00	27,09	3,34	30,77	0,00	99,67
64,80	0,66	26,97	0,00	7,57	0,00	99,67
68,84	0,59	23,15	0,30	7,12	0,00	100,00
52,92	2,06	29,21	2,06	13,75	0,00	100,00
67,37	1,05	20,77	0,35	10,21	0,00	99,30
74,55	0,00	22,66	0,00	2,52	0,00	100,00
76,64	0,00	20,58	0,41	2,06	0,00	99,59
66,19	0,00	25,18	0,00	8,63	0,00	100,00
71,25	7,81	19,38	0,63	0,94	0,00	100,00

71,08	7,52	16,44	0,34	2,52	0,00	100,00
33,66	44,39	21,46	0,00	0,00	0,49	99,51
38,28	44,55	14,85	0,00	2,31	0,00	100,00
64,29	17,14	8,57	4,29	4,29	1,43	100,00
65,83	9,17	24,17	0,00	0,00	0,83	98,33
60,27	7,19	19,77	0,00	3,88	0,00	99,61
30,20	49,80	18,78	0,00	0,82	0,41	100,00
46,31	30,87	20,13	0,00	2,01	0,67	100,00
58,53	2,76	31,80	0,00	6,91	0,00	100,00
83,33	0,00	13,39	0,00	0,00	0,00	100,00
74,43	0,97	10,68	0,32	13,59	0,00	99,68
79,82	4,13	12,84	0,00	3,21	0,00	100,00
79,41	1,47	16,18	1,47	1,47	0,00	94,12
81,00	1,08	12,19	0,36	5,38	0,00	100,00
59,17	2,75	14,68	0,92	22,48	0,00	100,00
56,07	1,26	26,78	0,00	15,90	0,00	100,00
62,87	1,50	27,25	0,30	8,08	0,00	100,00
69,52	0,95	25,71	0,00	3,81	0,00	100,00
59,80	1,01	24,41	0,00	14,58	0,00	99,32
73,02	1,47	19,41	0,29	4,71	0,88	99,41
67,20	0,54	22,04	0,54	9,68	0,00	100,00
60,62	0,52	30,05	0,00	8,81	0,00	100,00
68,75	1,34	28,25	0,45	0,45	0,45	100,00
70,44	5,35	14,47	0,63	8,81	0,31	99,37
56,57	9,60	26,58	0,51	6,58	0,00	99,49
75,31	0,00	19,69	0,00	5,00	0,00	100,00
62,96	5,82	21,16	0,00	10,05	0,00	100,00
49,81	33,72	15,33	0,00	0,77	0,38	100,00
80,60	1,99	16,92	0,00	0,50	0,00	100,00
34,83	50,56	12,36	0,56	1,69	0,00	100,00
73,48	0,00	21,55	0,00	4,97	0,00	99,45
33,14	55,03	10,06	0,00	1,78	0,00	100,00
36,45	42,86	14,29	0,49	5,91	0,00	99,01
73,53	1,96	19,80	0,99	2,97	0,00	100,00
96,22	0,42	2,52	0,00	0,84	0,00	99,58
55,50	0,00	18,32	0,00	26,18	0,00	100,00
70,15	0,92	19,69	0,31	8,92	0,00	100,00
88,62	0,60	7,27	0,00	2,42	0,00	99,39
72,07	0,00	22,41	0,00	5,52	0,00	100,00
63,86	0,60	24,70	0,00	10,84	0,00	100,00
52,31	0,46	32,56	0,47	13,95	0,00	96,28
75,00	0,00	19,64	0,00	5,36	0,00	100,00
73,27	0,00	18,38	0,24	8,11	0,00	99,52
73,23	0,00	23,08	0,00	3,69	0,00	99,08
77,86	0,00	15,65	0,00	6,49	0,00	100,00
80,16	0,40	9,52	0,00	9,52	0,40	96,43
83,47	0,00	10,33	0,00	6,20	0,00	100,00
87,46	0,31	7,52	1,88	2,51	0,31	99,37
66,49	0,00	19,37	0,00	14,14	0,00	99,48
58,71	1,94	27,10	0,00	12,26	0,00	100,00

59,94	0,30	25,82	0,00	11,57	2,37	100,00
61,52	0,84	16,85	0,28	19,94	0,56	99,44
63,67	2,25	26,05	0,00	7,72	0,32	99,36
76,44	0,29	22,13	0,00	1,15	0,00	99,14
86,18	0,00	10,80	0,00	3,02	0,00	100,00
55,40	0,00	34,66	0,00	9,75	0,00	99,28
47,81	35,04	12,77	0,00	2,92	1,46	100,00
45,06	35,44	15,95	0,00	2,78	0,76	100,00
0,00	0,00	0,00	0,00	0,00	0,00	0,00
87,01	0,56	8,47	0,00	3,95	0,00	100,00
62,89	0,00	34,02	0,52	2,58	0,00	100,00
60,86	2,57	19,41	0,29	14,12	0,88	100,00
62,93	1,02	25,51	0,00	10,20	0,34	98,98
70,97	0,46	16,59	0,00	11,52	0,46	100,00
61,11	2,22	19,70	0,38	14,39	0,76	100,00
67,37	2,54	17,37	0,85	11,86	0,00	99,58
65,30	1,49	23,60	0,37	8,99	0,00	100,00
69,80	1,34	18,79	1,34	7,38	1,34	100,00
61,71	0,55	26,39	0,00	7,33	0,00	100,00
63,34	0,27	26,49	0,00	9,73	0,00	99,73
63,94	0,61	17,27	0,00	17,58	0,61	100,00
60,99	1,42	24,82	1,06	11,70	0,00	100,00
61,04	2,27	21,75	0,32	14,61	0,00	100,00
63,06	0,00	23,42	0,00	13,51	0,00	99,10
63,37	0,00	13,58	0,00	23,05	0,00	100,00
82,27	0,00	10,00	0,00	7,73	0,00	100,00
62,39	0,00	10,40	0,00	27,22	0,00	100,00
78,54	2,58	9,44	0,86	8,58	0,00	100,00
83,26	0,47	10,23	0,00	6,05	0,00	100,00
83,92	0,29	9,36	0,00	6,43	0,00	100,00
98,31	0,00	0,42	0,00	1,27	0,00	100,00
59,18	0,00	24,49	0,00	16,33	0,00	100,00
48,00	0,00	38,86	0,00	13,14	0,00	100,00
47,58	0,00	33,80	0,00	16,20	0,00	100,00
66,67	9,28	12,24	0,42	10,97	0,42	100,00
71,98	0,39	21,48	0,00	4,30	1,56	100,00
68,32	0,50	18,81	0,50	11,88	0,00	100,00
60,07	3,41	29,79	1,03	5,48	0,00	100,00
48,92	1,08	25,00	1,09	22,46	1,09	99,64
80,06	1,23	13,50	0,00	4,60	0,61	100,00
61,46	2,78	17,71	0,69	16,67	0,69	100,00
78,00	1,50	16,50	0,00	3,50	0,50	100,00
96,88	0,00	3,13	0,00	0,00	0,00	100,00
71,99	0,90	25,60	0,00	1,51	0,00	100,00
81,82	2,10	12,59	0,00	3,50	0,00	100,00
41,71	28,14	24,12	0,00	5,53	0,50	100,00
65,00	5,00	17,50	0,00	12,50	0,00	100,00
70,14	0,00	20,00	0,47	6,05	1,40	100,00
64,95	0,47	22,44	0,00	9,27	0,00	100,00
66,88	1,88	21,38	0,00	9,43	0,00	100,00

77,68	0,86	18,45	0,43	2,58	0,00	99,57
51,30	0,00	41,18	0,00	7,19	0,00	100,00
73,27	0,99	24,75	0,00	0,99	0,00	100,00
81,13	0,00	16,98	0,00	1,89	0,00	100,00
75,19	6,67	15,93	0,00	2,22	0,00	100,00
88,63	0,00	9,41	0,00	1,96	0,00	100,00
61,72	1,56	22,66	0,00	14,06	0,00	99,22
57,50	2,50	24,17	0,83	15,00	0,00	100,00
65,86	2,69	19,09	0,00	12,37	0,00	100,00
53,94	9,15	26,71	0,00	8,14	0,00	100,00
65,88	3,14	25,10	0,00	5,88	0,00	100,00
69,86	4,11	23,29	0,68	2,05	0,00	100,00
70,14	3,79	22,22	0,00	2,42	0,00	100,00
77,39	5,53	16,58	0,00	0,50	0,00	100,00
60,00	16,52	14,15	0,00	2,83	0,00	100,00
81,35	6,11	10,68	0,00	1,29	0,00	100,00
79,23	6,92	13,08	0,00	0,77	0,00	100,00
71,28	5,13	12,31	0,00	11,28	0,00	100,00
57,32	8,28	22,22	1,04	5,21	0,00	100,00
60,79	7,90	23,71	0,00	7,60	0,00	100,00
66,38	10,34	13,62	0,00	2,35	0,47	100,00
63,97	4,41	20,80	1,60	1,60	1,60	100,00
48,36	6,15	4,10	0,00	40,98	0,41	100,00
60,66	18,75	15,81	0,37	4,41	0,00	100,00
54,46	23,47	21,13	0,00	0,94	0,00	100,00
56,40	15,60	23,20	0,00	4,80	0,00	100,00
70,61	6,58	16,37	0,00	5,75	0,00	100,00
53,79	18,62	26,90	0,00	0,69	0,00	100,00
63,57	7,14	21,80	0,00	3,76	0,00	100,00
37,64	32,96	22,72	0,89	5,79	0,00	100,00
89,77	0,23	8,60	0,00	1,40	0,00	99,07
22,58	0,00	6,45	0,00	35,48	35,48	100,00
60,00	0,00	24,32	0,00	15,68	0,00	100,00
65,00	4,58	19,58	0,00	10,42	0,42	100,00
77,89	1,02	17,35	0,34	3,40	0,00	99,66
47,08	38,91	7,78	2,33	3,50	0,39	98,83
84,49	0,82	11,89	0,41	2,05	0,00	100,00
65,63	0,00	27,45	0,00	6,92	0,00	99,76
76,05	4,18	14,07	0,38	3,42	1,90	100,00
32,00	48,80	15,60	0,00	2,00	1,60	99,60
86,55	1,75	7,60	0,00	3,51	0,58	100,00
77,16	5,25	14,20	0,31	3,09	0,00	99,38
58,68	13,42	17,37	0,26	7,63	2,63	99,74
53,21	26,92	13,78	0,32	4,17	1,60	100,00
77,06	0,00	18,82	0,00	4,12	0,00	100,00
61,63	0,39	13,95	0,00	24,03	0,00	100,00
56,39	1,97	25,52	0,00	13,10	0,00	100,00
72,99	4,18	17,68	0,00	5,14	0,00	99,68
58,29	0,00	22,38	0,28	19,06	0,00	100,00
74,00	0,00	22,75	0,00	3,25	0,00	100,00

72,77	2,13	17,45	0,00	7,23	0,43	100,00
68,30	1,44	26,01	0,00	4,05	0,00	100,00
47,98	38,31	9,72	0,40	3,24	0,00	100,00
78,46	0,38	12,74	0,00	7,34	0,77	100,00
41,76	0,88	10,59	0,00	46,47	0,29	100,00
34,21	0,00	2,33	6,98	0,00	0,00	86,05
77,46	0,58	17,34	0,00	4,62	0,00	99,42
0,00	0,00	0,00	0,00	0,00	0,00	0,00
81,06	0,00	11,80	0,31	6,83	0,00	100,00
82,62	0,00	9,57	0,00	7,80	0,00	99,29
81,09	0,00	11,54	0,00	7,37	0,00	99,04
89,19	0,90	9,91	0,00	0,00	0,00	99,10
93,10	0,00	5,52	0,00	1,38	0,00	100,00
85,20	0,00	13,25	0,00	0,80	0,40	99,60
72,44	0,64	16,13	1,29	9,03	0,00	100,00
76,64	5,61	17,76	0,00	0,00	0,00	100,00
63,38	11,27	25,35	0,00	0,00	0,00	100,00
67,90	6,17	11,11	2,47	11,11	1,23	100,00
70,06	4,52	9,60	0,00	14,69	1,13	99,44
68,97	0,00	20,69	0,00	10,34	0,00	100,00
70,48	3,81	22,86	0,00	2,86	0,00	100,00
33,75	46,25	16,25	0,00	3,75	0,00	100,00
51,79	34,82	8,93	0,00	4,17	0,30	100,00
77,08	0,00	11,46	0,00	10,42	1,04	98,96
66,67	0,00	20,37	0,00	11,11	1,85	100,00
96,05	0,00	2,63	0,00	1,32	0,00	100,00
60,14	4,20	17,16	0,75	13,43	0,00	100,00
56,14	7,02	29,82	0,00	7,02	0,00	100,00
59,09	0,00	18,18	0,00	22,73	0,00	100,00
73,88	9,70	5,22	0,75	10,45	0,00	99,25
89,78	0,00	10,22	0,00	0,00	0,00	100,00
85,33	0,00	10,67	0,00	4,00	0,00	100,00
48,65	0,00	12,16	0,00	39,19	0,00	100,00
89,05	0,00	7,30	0,00	3,65	0,00	100,00
77,61	0,00	20,90	0,00	1,49	0,00	100,00
82,76	0,00	15,86	0,00	1,38	0,00	99,31
73,90	14,06	8,84	0,00	3,21	0,00	100,00
91,88	1,25	5,00	0,00	1,88	0,00	99,38
53,73	0,00	23,13	0,00	23,13	0,00	100,00
99,22	0,78	0,00	0,00	0,00	0,00	100,00
60,43	0,72	20,86	0,72	17,27	0,00	100,00
88,89	0,00	5,56	0,00	5,56	0,00	100,00
85,19	3,70	0,00	0,00	0,00	11,11	100,00
83,33	0,00	13,89	0,00	2,78	0,00	100,00
63,33	0,00	30,00	0,00	6,67	0,00	100,00
0,00	0,00	0,00	0,00	0,00	0,00	0,00
46,88	0,00	48,39	0,00	3,23	0,00	100,00
57,50	0,00	22,86	0,00	11,43	0,00	100,00
57,48	7,87	33,07	0,00	1,57	0,00	100,00
62,50	0,00	28,13	0,00	9,38	0,00	100,00

60,94	0,00	27,73	0,00	11,33	0,00	100,00
80,68	0,00	16,38	0,00	2,93	0,00	97,56
74,17	2,10	5,71	0,00	18,02	0,00	100,00
86,88	0,00	7,69	0,00	5,43	0,00	100,00
57,36	0,51	25,51	0,00	12,76	3,57	98,98
65,32	0,00	21,39	0,00	12,43	0,87	100,00
56,35	1,24	24,15	0,31	15,48	2,48	100,00
70,29	0,72	18,12	0,00	10,14	0,72	100,00
76,47	0,00	14,71	0,00	8,82	0,00	97,06
75,00	0,00	22,50	0,00	2,50	0,00	100,00
81,95	0,00	16,54	0,00	1,50	0,00	100,00
89,47	0,00	8,77	0,00	0,00	1,75	100,00
73,33	0,51	18,97	1,03	6,15	0,00	100,00
65,12	0,00	18,60	0,00	16,28	0,00	100,00
50,00	0,00	26,79	0,00	19,64	3,57	100,00
67,52	0,00	5,73	0,00	17,83	8,92	99,36
87,10	0,00	12,90	0,00	0,00	0,00	100,00
59,17	1,67	22,50	0,00	16,67	0,00	100,00
72,09	0,00	15,58	0,00	3,90	0,00	100,00
65,38	0,00	34,62	0,00	0,00	0,00	100,00
48,31	0,00	16,46	0,00	29,11	0,00	100,00
66,04	0,00	18,87	0,00	13,21	1,89	100,00
80,00	0,00	10,59	0,00	9,41	0,00	100,00
63,49	0,00	14,29	0,00	22,22	0,00	100,00
71,74	0,00	23,91	0,00	4,35	0,00	100,00
80,22	0,00	19,78	0,00	0,00	0,00	100,00
80,77	0,00	15,38	0,00	3,85	0,00	100,00
65,33	0,00	32,00	1,33	1,33	0,00	100,00
72,34	1,06	22,34	0,00	4,26	0,00	100,00
78,49	0,00	17,20	0,00	4,30	0,00	100,00
84,85	0,00	9,09	0,00	6,06	0,00	96,97
84,96	0,41	11,38	0,00	1,63	1,63	32,93
66,67	1,59	26,98	0,00	4,76	0,00	100,00
77,56	0,00	12,25	0,00	9,80	0,00	100,00
90,99	0,00	7,21	0,00	1,80	0,00	97,75
69,77	0,00	15,50	0,00	13,95	0,78	100,00
72,50	0,00	20,00	0,00	7,50	0,00	100,00
85,96	0,00	8,19	0,00	5,85	0,00	100,00
65,19	0,00	18,52	0,00	16,30	0,00	100,00
79,39	0,61	8,54	0,61	10,37	0,00	100,00
79,07	0,00	17,07	0,00	0,00	0,00	100,00
65,42	6,54	23,58	0,00	2,83	0,94	99,06
61,02	13,56	13,56	3,39	8,47	0,00	100,00
40,79	38,16	17,11	2,63	0,00	1,32	100,00
81,75	0,40	9,52	0,00	7,94	0,40	100,00
42,56	0,00	15,90	1,03	40,51	0,00	100,00
68,46	0,67	27,03	0,00	3,38	0,00	100,00
65,93	0,74	22,96	0,00	10,37	0,00	100,00
57,07	0,00	25,85	0,00	17,07	0,00	100,00
33,33	55,02	9,64	0,00	0,80	1,20	100,00

92,24	0,00	6,90	0,00	0,86	0,00	100,00
45,55	22,60	26,37	1,03	4,45	0,00	99,66
65,26	1,41	25,96	0,00	5,77	0,00	100,00
34,69	46,94	14,29	0,00	4,08	0,00	100,00
0,55	97,24	0,00	0,55	0,55	1,10	100,00
9,30	88,95	0,58	0,00	1,16	0,00	100,00
35,32	36,82	23,88	0,50	3,48	0,00	100,00
70,97	1,08	19,18	0,22	8,41	0,00	99,78
56,83	0,00	23,50	2,19	16,94	0,55	100,00
86,43	0,00	8,56	0,00	4,67	0,00	100,00
94,81	0,47	3,77	0,00	0,94	0,00	99,06
63,49	0,63	24,44	0,00	11,11	0,32	99,37
54,45	0,00	38,74	0,00	6,81	0,00	100,00
70,00	0,00	20,65	0,00	3,26	0,00	100,00
88,89	0,00	7,41	3,70	0,00	0,00	96,30
85,71	0,00	9,52	0,00	4,76	0,00	100,00
51,76	19,10	24,62	1,01	3,52	0,00	100,00
54,26	12,77	22,87	0,53	8,51	1,06	100,00
2,19	95,99	1,82	0,00	0,00	0,00	100,00
44,31	6,50	20,48	0,00	4,22	0,00	100,00
70,71	0,00	21,43	0,00	5,71	2,14	100,00
70,56	0,51	19,80	0,00	9,14	0,00	100,00
65,98	0,41	26,64	0,00	6,97	0,00	100,00
40,00	33,81	18,57	0,48	7,14	0,00	100,00
49,71	1,71	35,43	2,86	9,71	0,57	99,43
66,26	0,00	23,98	0,00	9,35	0,41	99,59
69,00	0,00	14,50	0,00	16,50	0,00	100,00
84,57	0,59	11,01	0,60	2,98	0,00	100,00
61,04	0,00	37,66	0,00	1,30	0,00	100,00
26,60	48,40	17,39	2,72	3,26	0,00	100,00
14,29	73,95	11,34	0,00	0,00	0,42	100,00
60,26	16,24	18,86	0,00	2,63	0,00	100,00
39,57	38,26	18,26	0,00	1,74	2,17	100,00
70,83	1,04	21,88	0,00	5,21	1,04	100,00
38,75	3,75	1,25	0,00	56,25	0,00	100,00
31,53	0,49	9,85	0,00	2,46	55,67	66,50
90,79	2,63	5,26	0,00	1,32	0,00	98,68
96,63	0,00	0,00	0,00	0,00	0,00	100,00
100,00	0,00	0,00	0,00	0,00	0,00	89,87
76,10	0,74	17,65	0,00	5,15	0,37	99,63
84,62	0,00	15,38	0,00	0,00	0,00	100,00
79,25	0,00	16,98	0,00	3,77	0,00	98,11
57,14	0,00	23,81	0,00	19,05	0,00	100,00
50,00	0,00	0,00	33,33	16,67	0,00	100,00
90,91	0,00	9,09	0,00	0,00	0,00	100,00
84,62	0,00	11,54	0,00	3,85	0,00	100,00
90,48	4,76	0,00	0,00	4,76	0,00	95,24
66,67	0,00	25,00	2,78	5,56	0,00	100,00
0,00	0,00	0,00	0,00	0,00	0,00	0,00
75,56	0,00	17,78	0,00	6,67	0,00	100,00

53,53	2,35	29,17	0,60	13,69	0,00	98,81
0,00	0,00	0,00	0,00	0,00	0,00	0,00
67,59	17,59	12,96	0,93	0,00	0,93	100,00
55,50	25,65	17,28	0,52	1,05	0,00	100,00
66,47	15,16	14,87	0,00	3,50	0,00	100,00
58,73	21,43	17,06	0,00	2,78	0,00	100,00
65,48	14,84	17,74	0,32	1,61	0,00	100,00
77,89	14,19	7,59	0,00	0,33	0,00	100,00
66,15	12,50	19,27	1,56	0,00	0,52	100,00
60,47	21,51	17,44	0,00	0,58	0,00	100,00
78,75	1,05	19,16	0,00	1,05	0,00	100,00
75,00	3,92	16,67	0,98	3,43	0,00	99,51
74,13	5,94	18,88	0,00	1,05	0,00	100,00
54,34	28,30	15,47	1,13	0,38	0,38	100,00
51,82	25,55	20,44	0,73	1,46	0,00	100,00
63,09	18,12	16,11	0,00	2,68	0,00	100,00
51,64	34,87	11,84	0,00	1,64	0,00	100,00
54,25	23,58	17,92	0,00	0,00	4,25	100,00
61,70	18,79	16,67	0,35	2,48	0,00	100,00
45,23	33,17	16,08	0,50	3,02	2,01	100,00
62,54	22,19	13,83	0,58	0,86	0,00	100,00
73,71	8,25	10,31	0,00	7,73	0,00	100,00
71,54	13,82	11,38	0,00	3,25	0,00	100,00
36,23	39,61	18,36	0,00	5,31	0,48	94,69
66,32	5,70	22,80	0,00	5,18	0,00	100,00
73,12	2,15	20,43	0,00	4,30	0,00	100,00
46,98	20,26	25,86	0,43	5,60	0,86	100,00
74,31	10,28	11,86	0,00	3,56	0,00	100,00
82,11	3,66	11,38	0,81	2,03	0,00	100,00
84,96	3,01	7,52	0,00	4,51	0,00	100,00
73,89	4,42	20,00	0,00	1,33	0,00	100,00
66,17	12,64	15,24	0,37	5,58	0,00	100,00
80,24	4,74	14,23	0,00	0,79	0,00	99,60
72,83	6,04	19,62	0,00	1,51	0,00	100,00
47,18	29,93	18,37	0,00	4,24	0,00	100,00
67,69	0,00	18,75	0,00	12,50	0,00	100,00
54,55	20,13	22,40	0,00	2,60	0,32	100,00
78,40	10,40	8,00	0,00	3,20	0,00	100,00
55,08	24,15	18,64	0,42	1,69	0,00	100,00
0,00	0,00	0,00	0,00	0,00	0,00	0,00
61,86	20,10	15,71	0,00	1,05	0,00	100,00
40,93	43,04	15,19	0,00	0,42	0,42	100,00
76,74	8,14	11,63	0,00	3,49	0,00	100,00
61,95	22,12	13,27	0,00	2,65	0,00	100,00
61,75	16,39	18,58	0,55	2,73	0,00	100,00
63,74	7,60	25,15	0,00	3,51	0,00	100,00
44,67	28,67	22,67	1,33	2,00	0,67	100,00
58,49	7,55	16,67	0,00	0,00	0,00	100,00
53,97	21,90	22,54	0,00	1,59	0,00	100,00
49,32	21,92	11,48	0,00	3,28	0,00	100,00

91,84	1,02	3,09	0,00	3,09	0,00	100,00
49,02	25,16	23,53	0,00	2,29	0,00	100,00
71,25	20,63	6,56	0,31	1,25	0,00	100,00
42,65	34,05	17,33	0,00	2,89	2,53	100,00
82,17	3,49	13,18	0,00	1,16	0,00	100,00
80,30	0,00	13,64	0,00	4,55	1,52	98,48
75,53	3,19	11,70	0,00	8,51	1,06	100,00
40,00	44,00	15,33	0,00	0,00	0,67	99,33
0,00	0,00	0,00	0,00	0,00	0,00	0,00
55,06	0,56	29,17	0,00	11,90	0,00	100,00
73,48	2,27	18,94	0,00	5,30	0,00	100,00
33,99	34,48	28,57	0,00	2,46	0,49	100,00
75,76	3,03	21,21	0,00	0,00	0,00	100,00
40,14	36,62	22,54	0,00	0,70	0,00	100,00
40,57	28,69	27,87	0,00	2,87	0,00	100,00
75,36	2,90	15,94	0,00	5,80	0,00	100,00
80,77	0,00	15,38	0,00	3,85	0,00	100,00
57,25	19,22	16,86	0,39	6,27	0,00	100,00
58,62	0,00	27,59	0,00	13,79	0,00	99,31
67,23	0,00	27,66	1,28	3,83	0,00	100,00
64,89	1,88	26,96	0,63	5,64	0,00	99,69
66,10	0,42	26,69	0,00	6,78	0,00	100,00
64,90	0,00	33,47	0,00	1,63	0,00	100,00
58,75	1,25	35,00	0,00	5,00	0,00	98,75
41,10	42,33	15,95	0,00	0,61	0,00	100,00
62,81	3,51	29,12	1,05	3,51	0,00	100,00
64,04	0,00	27,40	0,00	8,56	0,00	100,00
55,23	1,16	30,61	1,17	11,66	0,00	100,00
59,90	1,02	22,34	0,51	13,20	3,05	99,49
52,94	5,57	30,43	0,00	10,87	0,00	100,00
57,22	1,67	30,00	0,00	11,11	0,00	100,00
60,51	1,14	34,66	0,00	3,41	0,28	98,86
57,48	0,00	22,05	0,00	20,47	0,00	100,00
67,23	0,85	24,68	0,85	6,38	0,00	98,72
64,37	0,00	22,99	0,00	12,64	0,00	100,00
89,21	3,32	7,47	0,00	0,00	0,00	100,00
63,64	18,18	12,73	0,00	4,55	0,91	100,00
62,77	1,06	18,09	0,00	18,09	0,00	100,00
59,11	1,28	29,67	0,33	6,00	1,00	99,33
77,82	0,00	13,09	0,36	8,73	0,00	100,00
63,64	13,89	20,71	0,00	1,01	0,76	99,75
79,91	4,80	13,97	0,00	1,31	0,00	99,13
63,08	24,62	6,15	0,77	4,62	0,77	100,00
57,21	4,50	27,49	0,95	6,16	0,47	99,53
55,30	18,54	12,91	0,99	11,92	0,33	100,00
70,33	1,10	15,93	0,55	11,54	0,55	100,00
59,58	1,25	28,75	0,00	7,92	2,50	100,00
56,93	23,27	14,85	0,00	4,95	0,00	100,00
77,78	3,58	17,20	0,72	0,72	0,00	99,64
57,73	2,06	29,90	0,00	9,28	1,03	97,94

80,28	0,00	17,37	0,00	1,88	0,47	100,00
75,24	0,00	24,76	0,00	0,00	0,00	100,00
56,48	0,00	36,79	0,00	6,74	0,00	96,89
55,08	0,53	14,97	1,60	25,67	2,14	100,00
67,58	0,91	21,92	0,00	9,59	0,00	100,00
58,05	13,79	20,69	0,00	7,47	0,00	100,00
60,66	0,55	24,59	0,00	14,21	0,00	100,00
57,32	0,91	28,35	0,00	11,89	1,52	100,00
58,90	0,00	29,45	0,00	11,66	0,00	99,39
66,67	0,91	19,63	0,00	12,79	0,00	100,00
58,28	3,25	24,56	0,00	13,61	0,30	99,70
36,46	0,00	41,67	0,00	16,67	0,00	100,00
65,89	0,00	24,42	0,39	9,30	0,00	100,00
72,60	1,78	20,64	0,36	4,63	0,00	100,00
72,38	2,76	13,81	0,00	9,39	1,66	100,00
40,09	23,87	22,45	0,00	5,10	0,00	99,49
42,98	31,14	12,28	0,00	13,60	0,00	100,00
50,78	21,88	16,41	0,39	10,16	0,39	100,00
55,51	14,69	19,37	0,00	3,15	0,00	100,00
64,82	4,74	24,51	0,00	5,93	0,00	100,00
53,59	20,72	20,16	0,00	3,09	0,00	100,00
56,65	18,04	22,22	0,63	2,22	0,00	100,00
68,21	9,27	20,20	0,00	1,66	0,66	99,34
51,55	0,00	22,68	0,00	25,77	0,00	100,00
46,74	30,43	21,74	0,00	1,09	0,00	100,00
78,08	0,34	18,49	0,00	2,40	0,68	99,32
60,18	11,85	21,88	0,00	4,26	1,82	100,00
55,30	16,59	23,00	0,00	3,76	0,00	100,00
41,90	31,36	7,46	0,00	19,28	0,00	100,00
67,30	17,92	12,26	0,00	2,20	0,31	100,00
53,41	20,17	18,05	0,00	4,87	2,87	99,71
77,78	3,70	18,52	0,00	0,00	0,00	100,00
82,50	0,00	12,50	0,00	5,00	0,00	100,00
47,16	1,42	31,23	0,79	5,93	7,91	100,00
50,51	13,64	27,27	0,00	8,08	0,51	100,00
58,95	2,11	31,05	0,00	6,84	1,05	98,95
67,13	6,02	14,42	1,40	10,23	0,47	100,00
71,65	3,15	16,93	0,00	8,27	0,00	99,61
70,51	0,00	12,99	0,00	14,29	1,30	100,00
66,22	0,68	23,65	0,00	9,46	0,00	97,97
79,78	5,06	10,11	0,00	4,49	0,56	100,00
55,52	17,06	21,40	0,67	5,02	0,33	100,00
69,05	0,00	21,43	0,00	9,52	0,00	100,00
52,89	2,89	34,71	0,00	8,68	0,83	100,00
70,83	4,17	23,61	0,00	1,39	0,00	100,00
86,19	0,77	7,42	0,00	5,63	0,00	99,74
29,58	61,67	5,83	0,00	2,50	0,42	100,00
75,25	0,00	24,07	0,00	0,68	0,00	100,00
63,46	0,77	22,31	0,00	13,46	0,00	100,00
50,46	2,14	32,72	0,31	13,15	1,22	99,39

25,83	18,54	2,83	0,00	33,96	0,00	100,00
72,73	0,40	10,67	0,00	16,21	0,00	100,00
70,69	0,00	21,84	1,72	5,75	0,00	100,00
75,86	0,99	21,67	0,00	1,48	0,00	100,00
65,81	0,65	13,55	0,00	20,00	0,00	100,00
51,76	1,01	32,16	0,00	14,57	0,50	100,00
52,23	0,96	30,89	0,64	15,29	0,00	99,68
52,41	0,34	41,72	0,00	5,17	0,34	100,00
59,11	2,06	31,38	0,00	7,24	0,00	100,00
66,67	0,76	26,52	0,76	5,30	0,00	100,00
69,97	0,00	21,81	0,00	8,22	0,00	100,00
60,94	9,11	24,54	0,00	5,22	0,00	99,48
58,94	0,41	27,24	0,00	13,41	0,00	100,00
48,20	0,90	20,72	0,00	30,18	0,00	99,55
82,98	0,00	10,64	6,38	0,00	0,00	100,00
68,27	1,44	18,27	0,00	12,02	0,00	100,00
84,21	0,00	15,26	0,00	0,53	0,00	99,47
67,95	0,00	19,69	0,00	12,36	0,00	100,00
71,88	0,00	12,50	0,00	15,63	0,00	100,00
73,53	0,00	19,61	0,00	6,86	0,00	100,00
73,04	0,00	14,22	0,00	12,75	0,00	100,00
75,50	0,50	18,50	0,00	5,50	0,00	97,00
54,91	0,00	30,36	0,45	14,29	0,00	95,98
41,74	37,19	19,83	0,41	0,83	0,00	100,00
45,35	32,68	21,13	0,00	0,85	0,00	100,00
54,44	0,00	24,93	0,00	20,47	0,00	100,00
79,57	1,08	13,98	0,00	5,38	0,00	98,92
83,63	0,00	13,10	0,00	2,98	0,30	99,40
81,37	0,00	16,67	0,98	0,98	0,00	100,00
54,45	1,57	20,42	0,00	23,04	0,52	100,00
73,05	0,00	17,96	0,60	8,38	0,00	100,00
80,15	4,20	15,27	0,00	0,38	0,00	100,00
67,28	0,46	30,41	0,00	1,84	0,00	99,54
62,25	3,64	23,18	0,00	10,93	0,00	99,67
87,89	0,00	5,26	0,00	6,84	0,00	100,00
76,47	0,00	16,47	0,00	7,06	0,00	100,00
82,22	0,29	14,04	0,00	3,22	0,00	100,00
71,60	2,42	22,05	0,60	3,32	0,00	99,70
54,89	17,87	23,08	0,43	3,42	0,00	100,00
87,03	0,00	12,43	0,00	0,00	0,54	97,30
92,41	0,00	7,59	0,00	0,00	0,00	100,00
75,77	1,03	7,22	0,00	15,98	0,00	100,00
47,15	27,76	18,25	0,40	3,17	0,00	100,00
73,51	2,24	22,39	0,00	1,87	0,00	98,88
63,35	19,92	16,33	0,00	0,40	0,00	100,00
62,30	7,33	19,37	0,00	10,99	0,00	100,00
56,17	18,11	18,90	0,00	6,56	0,26	99,74
62,86	0,78	22,89	0,54	9,81	0,00	100,00
51,57	22,03	18,16	0,24	7,26	0,73	100,00
67,34	13,18	14,33	3,15	2,01	0,00	100,00

71,34	12,10	14,79	0,00	0,96	0,00	100,00
57,63	23,73	18,64	0,00	0,00	0,00	100,00
71,48	15,81	6,79	0,00	2,50	0,00	99,64
69,81	21,70	6,60	0,00	1,89	0,00	97,17
96,04	0,00	0,99	0,00	1,98	0,99	100,00
52,08	18,75	18,75	0,00	10,42	0,00	100,00
69,95	0,49	22,99	0,00	0,53	0,00	100,00
64,71	0,53	31,52	0,00	2,17	0,00	100,00
54,77	8,17	29,43	0,00	7,08	0,54	94,82
48,65	16,22	29,28	0,45	5,41	0,00	99,10
54,55	15,91	20,99	7,25	0,76	0,00	99,62
53,90	22,70	20,80	0,24	2,36	0,00	99,29
50,72	29,14	15,61	0,00	1,86	0,00	100,00
80,92	3,05	9,16	0,00	6,87	0,00	100,00
78,80	0,46	13,82	0,00	6,45	0,46	100,00
49,77	4,23	16,04	0,00	29,72	0,00	100,00
91,19	0,00	8,05	0,00	0,77	0,00	100,00
56,25	0,74	37,13	0,00	5,88	0,00	100,00
67,84	2,83	22,97	0,71	5,30	0,35	100,00
53,80	2,92	35,96	0,00	7,31	0,00	99,71
89,57	0,47	1,90	0,47	7,11	0,47	100,00
72,95	0,48	16,91	3,86	5,80	0,00	99,52
61,60	0,00	32,80	0,40	4,80	0,40	100,00
66,82	0,95	24,64	0,00	6,64	0,95	99,53
68,64	0,00	25,42	0,00	5,93	0,00	100,00
73,30	0,28	21,88	0,00	4,55	0,00	100,00
80,51	0,51	15,90	2,05	0,00	1,03	100,00
69,61	0,00	22,97	0,35	7,07	0,00	100,00
67,96	16,02	14,08	0,00	1,94	0,00	100,00
55,12	14,46	19,81	0,00	7,23	0,31	100,00
78,40	0,40	20,80	0,00	0,40	0,00	100,00
78,91	0,00	17,06	0,00	3,41	0,34	100,00
66,58	1,72	24,26	0,00	6,93	0,00	99,75
63,68	0,00	16,81	0,00	18,53	0,43	99,14
54,72	1,89	24,53	0,00	15,09	3,77	100,00
74,16	0,00	22,47	0,00	3,37	0,00	98,88
72,96	0,00	23,47	0,51	3,06	0,00	100,00
56,55	0,60	30,36	1,19	11,31	0,00	100,00
71,70	0,82	21,49	0,00	5,23	0,55	100,00
71,81	0,00	19,94	0,00	8,04	0,00	100,00
62,70	1,23	25,82	0,00	9,43	0,82	100,00
66,95	0,43	21,46	0,00	11,16	0,00	100,00
66,53	0,00	20,72	0,40	11,95	0,40	100,00
79,84	0,00	12,45	0,00	7,00	0,39	98,44
64,96	1,09	10,22	0,36	22,26	1,09	100,00
77,59	0,33	15,10	0,34	6,04	0,34	96,64
75,71	0,40	20,24	0,00	2,43	1,21	99,19
65,67	0,00	23,22	0,37	10,49	0,00	98,13
73,55	3,26	18,48	0,72	3,62	0,36	99,64
83,62	0,00	10,73	0,00	3,95	1,69	100,00

61,90	1,79	19,64	1,19	15,48	0,00	100,00
80,00	0,60	9,55	0,60	8,66	0,60	99,70
66,39	0,00	21,21	0,00	11,02	1,38	99,72
80,34	2,25	15,17	0,00	1,69	0,56	100,00
77,43	2,43	18,95	0,00	0,35	0,00	99,30
74,67	0,00	15,33	8,67	1,33	0,00	100,00
60,77	0,00	28,08	0,00	11,15	0,00	99,23
64,96	0,00	12,39	0,00	22,65	0,00	100,00
86,76	0,00	7,35	0,00	5,88	0,00	100,00
70,38	0,00	18,82	0,00	10,80	0,00	97,56
78,14	0,00	14,98	6,88	0,00	0,00	97,17
81,32	0,24	10,64	5,91	1,89	0,00	100,00
72,45	0,51	15,82	0,51	10,71	0,00	99,49
79,90	6,53	11,56	0,00	2,01	0,00	100,00
73,38	0,00	14,94	0,00	11,69	0,00	100,00
58,24	0,00	13,53	0,00	27,65	0,59	99,41
64,41	1,98	16,67	0,00	16,95	0,00	99,44
69,18	14,43	15,41	0,00	0,98	0,00	100,00
66,47	14,54	15,43	0,00	3,56	0,00	100,00
68,70	18,32	9,16	0,00	3,82	0,00	100,00
87,20	1,90	10,43	0,00	0,47	0,00	100,00
78,77	4,25	14,15	0,94	1,89	0,00	100,00
52,27	23,26	21,15	0,30	2,72	0,30	100,00
49,34	0,26	31,50	0,00	18,90	0,00	100,00
60,30	0,30	28,48	0,00	10,91	0,00	100,00
82,97	0,55	9,34	0,00	6,59	0,55	100,00
56,22	38,81	4,48	0,00	0,50	0,00	95,02
66,67	0,00	33,33	0,00	0,00	0,00	100,00
74,50	3,00	19,50	0,00	3,00	0,00	100,00
80,37	4,44	11,48	0,37	2,59	0,74	100,00
55,24	5,40	26,11	0,64	12,42	0,00	100,00
83,21	0,73	11,68	0,00	4,38	0,00	99,27
81,25	0,00	18,75	0,00	0,00	0,00	100,00
66,31	0,00	24,55	0,00	5,78	2,89	97,83
81,58	1,05	17,37	0,00	0,00	0,00	100,00
66,53	1,21	17,41	0,00	12,96	1,62	100,00
44,44	0,00	8,33	0,00	5,56	19,44	100,00
58,17	0,00	41,04	0,40	0,40	0,00	100,00
45,51	36,24	15,49	0,28	1,97	0,28	99,72
50,61	29,39	17,55	0,00	2,45	0,00	100,00
58,88	10,66	25,13	0,51	2,05	2,05	99,49
0,00	0,00	0,00	0,00	0,00	0,00	0,00
41,70	42,76	15,19	0,00	0,35	0,00	100,00
33,59	45,42	19,08	0,00	1,15	0,76	100,00
50,66	21,15	26,43	0,00	1,76	0,00	100,00
65,52	13,79	14,94	0,57	5,17	0,00	99,43
65,73	3,37	26,97	0,00	3,37	0,56	100,00
48,58	26,42	23,11	0,00	1,89	0,00	100,00
50,00	28,00	16,72	0,33	4,68	0,00	100,00
63,98	10,59	19,49	0,42	5,51	0,00	100,00

58,40	27,73	10,92	0,00	1,68	1,26	100,00
57,50	1,25	28,13	0,63	12,50	0,00	98,13
56,98	3,49	29,07	0,00	10,47	0,00	100,00
37,06	41,12	20,81	0,00	0,00	1,02	100,00
79,55	2,27	15,91	0,00	2,27	0,00	100,00
69,23	1,54	6,15	0,00	23,08	0,00	100,00
73,02	1,06	22,75	0,53	2,65	0,00	100,00
83,38	0,00	8,95	0,00	7,67	0,00	99,74
85,15	0,00	9,90	0,00	4,95	0,00	100,00
64,52	0,00	24,19	2,42	8,06	0,81	99,19
62,50	0,00	37,50	0,00	0,00	0,00	100,00
86,96	0,00	13,04	0,00	0,00	0,00	100,00
75,80	4,27	16,73	0,00	3,20	0,00	99,64
80,82	0,00	5,48	0,00	13,70	0,00	100,00
81,03	0,00	5,17	0,00	13,79	0,00	100,00
79,57	0,00	20,43	0,00	0,00	0,00	100,00
44,02	18,53	24,77	0,00	2,25	0,00	100,00
77,55	1,02	12,24	0,00	9,18	0,00	100,00
54,76	0,00	18,75	0,00	9,38	0,00	100,00
50,00	0,00	25,86	0,00	24,14	0,00	98,28
77,21	2,94	19,12	0,00	0,74	0,00	100,00
75,00	0,00	10,71	0,00	14,29	0,00	100,00
51,61	29,03	16,13	0,00	3,23	0,00	100,00
69,57	0,00	26,09	0,00	0,00	4,35	100,00
1,60	97,34	0,00	0,00	0,53	0,53	100,00
65,54	5,99	21,72	0,00	6,74	0,00	100,00
64,11	0,48	17,31	0,48	17,31	0,00	100,00
54,90	6,86	15,69	0,98	21,57	0,00	99,51
65,47	0,00	34,53	0,00	0,00	0,00	100,00
69,67	0,00	15,98	0,82	13,52	0,00	99,18
64,83	6,21	25,52	0,00	2,76	0,69	100,00
89,30	0,53	8,56	0,00	0,53	1,07	96,79
75,93	0,00	22,22	0,00	1,85	0,00	100,00
70,59	0,00	0,00	0,00	23,53	5,88	100,00
78,38	0,00	16,22	0,00	5,41	0,00	100,00
48,37	0,65	32,68	0,00	17,65	0,65	100,00
74,77	1,87	18,69	0,00	3,74	0,93	94,39
70,85	1,11	18,45	0,00	9,23	0,37	100,00
79,71	1,45	8,76	0,00	9,49	0,00	98,54
64,04	2,25	20,22	1,12	12,36	0,00	100,00
77,27	4,55	6,06	1,52	10,61	0,00	100,00
67,90	23,46	7,41	0,00	0,00	1,23	100,00
71,74	1,09	13,04	0,00	10,87	3,26	100,00
72,41	0,00	18,97	0,00	8,62	0,00	100,00
59,60	1,01	33,33	0,00	6,06	0,00	98,99
87,88	3,03	3,03	0,00	0,00	6,06	100,00
80,00	0,00	16,00	0,00	4,00	0,00	100,00
92,57	0,57	4,00	0,00	2,86	0,00	100,00
80,00	1,54	12,31	0,00	6,15	0,00	93,85
36,00	0,00	50,00	2,00	12,00	0,00	100,00

58,52	0,74	14,81	1,48	24,44	0,00	100,00
79,07	2,33	11,63	0,00	3,49	3,49	100,00
81,25	4,17	7,29	0,00	7,29	0,00	100,00
83,87	1,08	12,90	0,00	2,15	0,00	98,92
68,18	4,55	27,27	0,00	0,00	0,00	100,00
58,92	1,08	26,23	0,00	13,11	0,00	100,00
51,16	11,63	25,58	0,00	11,63	0,00	100,00
72,81	2,63	16,67	0,00	7,89	0,00	98,25
72,06	0,00	26,47	0,00	1,47	0,00	100,00
82,29	0,57	11,43	0,00	5,71	0,00	100,00
71,15	13,46	9,62	1,92	3,85	0,00	100,00
72,09	2,33	20,93	0,00	0,00	4,65	100,00
66,67	0,00	29,23	0,00	3,08	0,00	100,00
93,02	2,33	4,65	0,00	0,00	0,00	100,00
85,99	2,55	8,28	0,00	3,18	0,00	100,00
83,33	3,79	8,33	0,00	4,55	0,00	100,00
88,31	1,30	9,09	0,00	0,00	1,30	98,70
91,01	1,12	7,87	0,00	0,00	0,00	100,00
95,83	0,00	2,78	0,00	1,39	0,00	98,61
8,86	82,57	6,29	0,00	2,29	0,00	100,00
39,91	36,05	21,03	1,29	1,29	0,43	100,00
78,38	5,41	16,22	0,00	0,00	0,00	100,00
60,71	0,00	28,57	0,00	10,71	0,00	100,00
47,08	30,35	18,68	0,39	3,11	0,39	100,00
21,17	54,60	18,38	0,00	5,29	0,56	100,00
72,63	0,00	23,16	0,00	2,63	1,58	100,00
22,61	70,88	6,51	0,00	0,00	0,00	100,00
59,80	13,73	19,61	0,33	5,88	0,65	99,67
59,57	0,43	22,13	0,43	17,45	0,00	100,00
72,13	1,09	14,21	0,00	12,57	0,00	100,00
84,00	0,00	15,11	0,00	0,89	0,00	100,00
62,67	0,00	28,00	0,67	8,67	0,00	99,33
57,24	0,00	39,47	1,97	1,32	0,00	100,00
80,88	0,00	16,91	1,47	0,74	0,00	100,00
59,20	0,80	27,20	0,00	12,80	0,00	100,00
68,20	11,72	17,99	0,00	1,67	0,42	100,00
29,30	54,65	14,73	0,28	0,28	0,28	100,00
57,89	2,11	16,32	0,00	23,68	0,00	100,00
74,84	3,77	13,21	2,52	5,66	0,00	100,00
64,10	0,00	23,50	0,00	12,39	0,00	99,15
80,00	0,00	17,62	0,00	0,95	1,43	100,00
70,45	2,27	24,43	0,00	2,84	0,00	98,86
66,31	2,15	24,37	0,72	6,09	0,36	99,64
63,29	0,00	20,98	0,00	15,73	0,00	100,00
83,23	0,60	8,98	0,00	7,19	0,00	100,00
81,50	0,79	14,96	0,00	2,36	0,39	100,00
60,44	3,11	24,44	0,00	11,56	0,44	100,00
65,90	1,38	13,89	0,00	18,52	0,00	100,00
74,67	0,00	17,33	4,00	4,00	0,00	100,00
51,50	24,55	22,75	0,00	1,20	0,00	100,00

46,75	31,49	17,53	0,00	3,57	0,65	100,00
59,76	0,00	10,65	1,18	28,40	0,00	100,00
85,46	0,44	9,29	0,00	3,54	0,88	100,00
83,98	0,00	16,02	0,00	0,00	0,00	100,00
49,79	0,82	30,04	0,41	18,93	0,00	100,00
61,59	0,00	3,41	0,00	0,00	0,00	96,59
69,57	0,00	28,26	0,00	2,17	0,00	100,00
90,32	0,00	3,23	0,00	6,45	0,00	100,00
92,86	0,00	3,57	0,00	3,57	0,00	92,86
94,44	0,00	0,00	0,00	5,56	0,00	100,00
77,78	0,00	7,41	0,00	14,81	0,00	100,00
88,89	0,00	11,11	0,00	0,00	0,00	100,00
82,35	5,88	0,00	0,00	11,76	0,00	100,00
78,26	0,00	13,04	8,70	0,00	0,00	100,00
88,00	0,00	12,00	0,00	0,00	0,00	100,00
66,67	0,00	13,33	0,00	20,00	0,00	93,33
80,00	0,00	18,00	0,00	2,00	0,00	98,00
77,27	0,00	22,73	0,00	0,00	0,00	100,00
87,50	12,50	0,00	0,00	0,00	0,00	100,00
25,58	65,12	8,14	0,00	1,16	0,00	100,00
72,73	0,00	27,27	0,00	0,00	0,00	100,00
47,73	9,09	34,88	0,00	4,65	2,33	100,00
68,97	0,00	27,59	0,00	3,45	0,00	100,00
72,73	0,00	0,00	0,00	27,27	0,00	100,00
70,83	0,00	29,17	0,00	0,00	0,00	100,00
45,98	0,00	49,43	0,00	4,60	0,00	100,00
56,94	23,61	15,28	0,00	4,17	0,00	98,61
80,00	0,00	20,00	0,00	0,00	0,00	100,00
100,00	0,00	0,00	0,00	0,00	0,00	100,00
82,76	0,00	13,79	3,45	0,00	0,00	100,00
69,52	0,95	26,67	0,00	1,90	0,95	100,00
27,27	0,00	6,06	0,00	12,12	54,55	100,00
62,50	34,38	3,13	0,00	0,00	0,00	100,00
45,00	0,00	0,00	0,00	0,00	0,00	100,00
83,33	1,28	7,69	0,00	7,69	0,00	100,00
63,33	3,33	23,33	3,33	6,67	0,00	100,00
72,41	0,00	3,45	0,00	20,69	3,45	89,66

DOMAGPOC	DOMAGCHU	DOMAGOUT	DOMBHEXS	DOMBHGER
0,00	0,00	0,00	100,00	100,00
0,00	0,00	0,00	100,00	100,00
0,00	0,00	0,00	100,00	100,00
0,00	0,00	0,00	100,00	100,00
0,00	0,00	0,00	100,00	100,00
0,00	0,00	0,32	99,68	99,68
0,00	0,00	0,00	100,00	100,00
0,00	0,00	0,00	100,00	100,00
0,00	0,00	0,00	100,00	93,97
0,00	0,00	0,00	100,00	100,00
0,00	0,00	0,00	100,00	100,00
0,00	0,00	0,00	100,00	100,00
0,00	0,00	0,00	100,00	100,00
0,36	0,00	0,00	100,00	100,00
0,00	0,00	0,00	100,00	100,00
0,00	0,00	0,00	100,00	100,00
0,00	0,00	0,00	100,00	100,00
0,00	0,00	0,00	100,00	98,94
0,00	0,00	0,00	100,00	100,00
0,00	0,00	0,00	100,00	100,00
0,00	0,00	0,00	100,00	100,00
0,00	0,00	0,00	99,51	99,51
0,00	0,00	0,00	100,00	100,00
0,00	0,00	0,00	100,00	100,00
0,00	0,00	0,00	100,00	100,00
0,00	0,00	0,00	100,00	100,00
0,00	0,00	0,00	100,00	100,00
0,00	0,00	0,00	100,00	100,00
0,00	0,00	0,00	100,00	99,27
0,00	0,00	0,00	100,00	99,06
0,00	0,00	0,00	0,00	0,00
0,00	0,00	0,00	100,00	100,00
0,00	0,00	0,00	100,00	100,00
0,00	0,00	0,00	100,00	100,00
0,00	0,00	0,00	100,00	99,59
0,00	0,00	0,00	100,00	98,63
0,00	0,00	0,00	100,00	100,00
0,00	0,00	0,00	100,00	100,00
0,00	0,00	0,00	100,00	99,64
0,00	0,00	0,00	100,00	99,47
0,00	0,00	0,00	100,00	99,42
0,00	0,00	0,00	100,00	98,71
0,00	0,00	0,00	100,00	99,56
0,00	0,00	0,00	100,00	99,61
0,00	0,00	0,00	99,31	97,23
0,00	0,00	0,00	100,00	100,00
0,00	0,00	0,00	100,00	100,00
0,00	0,00	0,00	100,00	100,00
0,00	0,00	0,00	100,00	100,00

0,00	0,00	0,00	100,00	100,00
0,00	0,00	0,00	100,00	100,00
0,00	0,00	0,00	100,00	100,00
0,00	0,00	0,00	100,00	100,00
0,00	0,00	0,00	100,00	98,18
0,00	0,00	0,00	100,00	99,27
0,00	0,00	0,00	100,00	97,73
0,37	0,00	0,37	100,00	99,25
0,00	0,00	0,00	100,00	100,00
0,00	0,00	0,00	100,00	100,00
0,00	0,00	0,00	100,00	99,48
0,42	0,00	0,00	100,00	100,00
0,00	0,00	0,00	100,00	99,52
0,00	0,00	0,00	100,00	100,00
0,00	0,00	0,00	99,68	98,41
0,00	0,00	0,00	100,00	100,00
0,00	0,00	0,00	100,00	100,00
0,00	0,00	0,00	100,00	100,00
0,00	0,00	0,00	100,00	100,00
0,00	0,00	0,00	100,00	100,00
0,00	0,00	0,00	100,00	100,00
0,00	0,00	0,00	100,00	100,00
0,34	0,00	0,00	100,00	100,00
0,00	0,00	0,00	100,00	100,00
0,34	0,00	0,00	100,00	100,00
0,00	0,00	0,00	100,00	100,00
0,00	0,00	0,00	100,00	100,00
0,00	0,00	0,00	100,00	100,00
0,00	0,00	0,00	100,00	100,00
0,00	0,00	0,00	100,00	100,00
0,00	0,00	0,00	100,00	100,00
0,00	0,00	0,00	100,00	100,00
0,00	0,00	0,00	100,00	100,00
0,00	0,00	0,00	100,00	100,00
0,00	0,00	0,00	100,00	100,00
0,00	0,00	0,00	100,00	100,00
0,00	0,00	0,00	100,00	100,00
0,00	0,00	0,00	100,00	100,00
0,00	0,00	0,00	100,00	100,00
0,00	0,00	0,00	100,00	100,00
0,37	0,00	0,00	100,00	100,00
0,00	0,00	0,00	100,00	99,70
0,00	0,00	0,00	100,00	100,00
0,64	0,00	0,00	100,00	100,00
0,00	0,00	0,00	100,00	100,00
0,00	0,00	0,00	100,00	100,00
1,40	0,00	0,00	100,00	98,95
0,00	0,00	0,00	100,00	99,58
0,00	0,00	0,00	100,00	100,00
0,00	0,00	0,00	100,00	100,00

0,00	0,00	0,00	100,00	100,00
0,00	0,00	0,00	100,00	100,00
0,00	0,00	0,00	100,00	99,66
0,00	0,00	0,00	100,00	100,00
6,71	0,00	0,00	100,00	100,00
0,00	0,00	0,00	100,00	100,00
0,00	0,00	0,00	100,00	100,00
0,00	0,00	0,00	100,00	100,00
0,00	0,00	0,00	100,00	100,00
0,00	0,00	0,00	100,00	100,00
0,00	0,00	0,00	100,00	100,00
0,00	0,00	0,00	100,00	100,00
0,00	0,00	0,00	100,00	100,00
0,00	0,00	0,00	100,00	100,00
0,00	0,00	0,00	100,00	100,00
0,00	0,00	0,00	100,00	100,00
0,00	0,00	0,00	100,00	100,00
0,00	0,00	0,00	100,00	100,00
0,00	0,00	0,00	100,00	100,00
0,00	0,00	0,00	100,00	100,00
0,00	0,00	0,00	0,00	0,00
0,00	0,00	0,00	100,00	100,00
0,00	0,00	0,00	100,00	100,00
0,00	0,00	0,00	90,57	90,57
0,00	0,00	0,00	0,00	0,00
0,00	0,00	0,00	100,00	100,00
0,00	0,00	0,00	100,00	100,00
0,00	0,00	0,00	100,00	100,00
0,00	0,00	0,00	100,00	100,00
0,00	0,00	0,00	100,00	100,00
0,00	0,00	0,00	100,00	100,00
0,00	0,00	0,00	100,00	100,00
0,00	0,00	0,00	100,00	100,00
5,03	0,00	0,00	100,00	100,00
0,53	0,00	0,00	100,00	100,00
0,00	0,00	0,00	100,00	100,00
0,00	0,00	0,00	100,00	100,00
0,00	0,00	0,00	100,00	100,00
0,00	0,00	0,00	100,00	99,61
0,00	0,00	0,00	100,00	100,00
0,00	0,00	0,00	100,00	100,00
0,00	0,00	0,00	100,00	99,55
0,00	0,00	0,00	100,00	100,00
0,00	0,00	0,00	100,00	100,00
0,00	0,00	0,00	100,00	100,00
0,00	0,00	0,00	100,00	100,00
0,00	0,00	0,00	100,00	100,00
0,00	0,00	0,00	100,00	100,00
0,00	0,00	0,00	100,00	99,13
0,00	0,00	0,00	99,52	99,52

0,00	0,00	0,00	100,00	100,00
0,00	0,00	0,00	100,00	100,00
0,00	0,00	0,00	100,00	100,00
0,00	0,00	0,00	100,00	100,00
0,00	0,00	0,00	100,00	100,00
0,00	0,00	0,53	99,47	97,88
0,00	0,00	0,00	100,00	100,00
0,00	0,00	0,00	100,00	97,92
0,00	0,00	0,00	98,69	98,69
0,00	0,00	0,00	100,00	92,92
0,00	0,00	0,00	100,00	100,00
0,00	0,00	1,41	100,00	100,00
0,00	0,00	1,65	99,59	99,17
0,00	0,00	0,00	100,00	99,45
0,59	0,00	0,00	98,82	98,24
0,00	0,00	0,00	100,00	100,00
0,00	0,00	0,00	100,00	99,27
0,00	0,00	0,00	100,00	100,00
0,00	0,00	0,00	99,16	68,07
0,00	0,00	0,00	100,00	100,00
0,00	0,00	1,16	99,42	99,42
0,00	0,00	0,00	100,00	100,00
0,00	0,00	0,00	100,00	100,00
0,00	0,00	0,00	100,00	93,42
0,00	0,00	0,00	100,00	100,00
0,00	0,00	0,54	98,91	98,91
0,00	0,00	0,00	100,00	100,00
0,00	0,00	0,66	100,00	98,68
0,00	0,00	0,00	99,34	99,01
0,00	0,00	0,00	100,00	99,24
0,00	0,00	0,00	100,00	99,39
0,00	0,00	0,00	100,00	99,48
0,00	0,00	0,00	100,00	90,00
0,00	0,00	0,88	100,00	100,00
0,00	0,00	0,00	100,00	95,81
0,00	0,00	0,00	100,00	100,00
0,00	0,00	0,00	100,00	100,00
0,00	0,00	0,00	100,00	100,00
0,00	0,00	0,00	100,00	100,00
0,00	0,00	0,00	100,00	100,00
0,00	0,00	0,00	100,00	100,00
0,00	0,00	0,37	99,63	93,01
0,00	0,00	0,00	100,00	91,20
0,32	0,00	2,25	100,00	9,32
0,00	1,06	0,00	100,00	100,00
0,00	0,00	0,00	100,00	100,00
0,00	0,00	0,00	100,00	100,00
0,00	0,00	0,00	100,00	100,00
0,00	0,00	0,00	100,00	100,00
0,00	0,00	0,00	100,00	100,00

0,40	0,00	0,00	100,00	100,00
0,00	0,00	0,00	100,00	100,00
0,00	0,00	0,00	100,00	100,00
0,00	0,00	0,00	100,00	100,00
0,00	0,00	0,00	100,00	100,00
0,00	0,00	0,00	100,00	99,64
0,00	0,00	0,00	100,00	100,00
0,00	0,00	0,00	100,00	100,00
0,00	0,00	0,00	100,00	100,00
0,00	0,00	0,00	100,00	100,00
0,00	0,00	0,00	100,00	100,00
0,00	0,00	0,00	100,00	99,64
0,00	0,00	0,00	100,00	100,00
0,00	0,00	0,00	100,00	100,00
0,00	0,00	0,00	100,00	100,00
0,00	0,00	0,00	100,00	100,00
0,00	0,00	0,00	100,00	100,00
0,00	0,00	0,00	100,00	100,00
0,00	0,41	0,00	100,00	100,00
0,00	0,00	0,00	100,00	100,00
0,00	0,00	0,00	100,00	100,00
0,00	0,00	0,00	100,00	100,00
0,00	0,00	0,00	100,00	100,00
0,00	0,00	0,00	100,00	100,00
0,00	0,00	0,00	100,00	100,00
0,00	0,00	0,00	100,00	100,00
0,00	0,00	0,00	100,00	100,00
0,00	0,00	0,00	100,00	100,00
0,00	0,00	0,00	100,00	100,00
0,00	0,00	0,00	100,00	100,00
0,00	0,00	0,00	100,00	99,63
0,00	0,00	0,00	100,00	100,00
0,00	0,00	0,00	100,00	100,00
0,00	0,00	0,00	100,00	100,00
0,00	0,00	0,00	100,00	100,00
0,47	0,00	0,00	100,00	100,00
0,00	0,00	0,00	100,00	97,73
0,00	0,00	0,00	100,00	100,00
0,00	0,43	0,00	99,57	99,57
0,00	0,00	0,00	100,00	99,45
0,00	0,00	0,00	100,00	100,00
0,00	0,00	0,00	100,00	100,00
0,00	0,00	0,00	100,00	100,00
0,56	0,00	0,00	100,00	100,00
0,00	0,00	0,00	100,00	100,00
0,00	0,00	0,00	100,00	100,00
0,00	0,00	0,00	100,00	100,00
0,00	0,00	0,00	100,00	100,00
0,00	0,00	0,00	100,00	100,00
0,00	0,00	0,00	100,00	100,00

0,00	0,00	0,00	100,00	100,00
0,00	0,00	0,00	100,00	100,00
0,00	0,00	0,00	100,00	100,00
0,00	0,00	0,00	100,00	100,00
0,00	0,00	0,00	100,00	100,00
0,00	0,00	0,00	100,00	100,00
0,00	0,00	0,00	100,00	100,00
0,00	0,00	0,00	100,00	100,00
0,00	0,00	0,00	100,00	100,00
0,00	0,00	0,00	100,00	100,00
0,00	0,00	0,00	100,00	100,00
0,00	0,00	0,00	100,00	99,17
0,00	0,00	0,00	100,00	100,00
0,00	0,00	0,00	100,00	100,00
0,00	0,00	0,00	100,00	100,00
0,00	0,00	0,00	100,00	100,00
0,00	0,00	0,00	100,00	100,00
0,00	0,00	0,00	100,00	100,00
0,00	0,00	0,00	100,00	99,46
0,00	0,00	0,00	100,00	100,00
0,00	0,00	0,00	99,73	99,73
0,00	0,00	0,00	100,00	99,60
0,00	0,00	0,00	100,00	100,00
0,00	0,00	0,00	100,00	100,00
0,00	0,00	0,00	100,00	100,00
0,76	0,00	0,00	100,00	100,00
0,00	0,00	0,00	100,00	100,00
0,00	0,00	0,00	100,00	100,00
0,00	0,00	0,00	100,00	100,00
0,00	0,00	0,00	100,00	100,00
0,00	0,00	0,00	100,00	100,00
0,00	0,00	0,00	100,00	100,00
0,00	0,00	0,00	100,00	100,00
0,00	0,00	0,00	100,00	99,19
0,00	0,00	0,00	100,00	99,68
0,00	0,00	0,00	100,00	100,00
0,00	0,00	0,00	100,00	100,00
0,00	0,00	0,00	100,00	100,00
0,00	0,00	0,00	100,00	100,00
0,00	0,00	0,00	100,00	100,00
0,00	0,00	0,00	100,00	100,00
0,00	0,00	0,00	100,00	100,00
0,93	0,00	0,00	100,00	100,00
0,00	0,00	0,00	100,00	100,00
0,00	0,00	0,00	100,00	100,00
0,00	0,00	0,00	100,00	100,00
0,00	0,00	0,00	100,00	100,00
0,00	0,00	0,00	100,00	100,00
0,00	0,00	0,00	100,00	100,00
0,00	0,00	0,00	100,00	100,00

0,00	0,00	0,00	100,00	100,00
0,00	0,00	0,00	100,00	100,00
0,00	0,00	0,00	100,00	100,00
0,00	0,00	0,00	100,00	99,60
0,00	0,00	0,00	100,00	100,00
0,00	0,00	0,00	100,00	100,00
0,00	0,00	0,00	100,00	100,00
0,00	0,00	0,00	100,00	100,00
0,00	0,00	0,00	100,00	99,51
0,00	0,00	0,00	100,00	100,00
0,00	0,00	0,00	0,00	0,00
0,00	0,00	0,00	100,00	99,74
0,00	0,00	0,00	100,00	100,00
0,00	0,00	0,00	100,00	100,00
0,00	0,00	0,00	100,00	99,70
0,00	0,00	0,00	100,00	100,00
0,00	0,00	0,00	100,00	100,00
0,00	0,00	0,00	100,00	99,36
0,00	0,00	0,00	100,00	100,00
0,00	0,00	0,00	100,00	100,00
0,00	0,00	0,00	100,00	100,00
0,00	0,00	0,00	100,00	100,00
0,00	0,00	0,00	100,00	100,00
0,00	0,00	0,00	100,00	100,00
0,00	0,00	0,00	100,00	100,00
0,00	0,00	0,00	100,00	100,00
0,00	0,00	0,00	100,00	100,00
0,00	0,00	0,00	100,00	100,00
1,35	0,00	0,45	100,00	100,00
0,00	0,00	0,00	100,00	100,00
0,00	0,00	0,00	100,00	100,00
0,00	0,00	0,00	100,00	100,00
0,00	0,00	0,00	100,00	100,00
3,93	0,00	0,44	100,00	99,13
0,00	0,00	0,00	100,00	100,00
0,00	0,00	0,00	100,00	100,00
0,00	0,00	0,00	100,00	100,00
0,00	0,00	0,00	100,00	100,00
0,00	0,00	0,00	99,21	99,21
0,00	0,00	0,00	100,00	100,00
0,00	0,00	0,00	100,00	49,64
0,00	0,00	0,00	100,00	100,00
0,00	0,00	0,54	100,00	92,47
0,00	0,00	0,00	100,00	95,33
0,42	0,00	0,00	100,00	98,32
0,00	0,00	0,53	100,00	99,47
0,00	0,00	0,00	100,00	98,97
0,30	0,00	0,30	100,00	97,58
0,00	0,00	0,00	100,00	100,00
0,00	0,00	0,00	99,76	99,76

0,00	0,00	0,00	100,00	100,00
0,00	0,00	0,00	99,67	99,01
0,00	0,00	0,00	100,00	100,00
0,00	0,00	0,00	100,00	100,00
0,00	0,00	0,00	100,00	81,21
0,00	0,00	0,00	100,00	96,23
0,00	0,00	0,64	100,00	94,53
0,00	0,00	0,00	100,00	100,00
0,00	0,00	0,00	100,00	100,00
0,00	0,00	0,00	100,00	100,00
0,00	0,00	0,00	100,00	100,00
0,00	0,00	0,00	100,00	100,00
0,00	0,00	0,00	100,00	100,00
0,00	0,00	0,00	100,00	99,68
0,00	0,00	0,00	100,00	100,00
0,49	0,00	0,00	100,00	100,00
0,00	0,00	0,00	100,00	100,00
0,00	0,00	0,00	100,00	100,00
0,00	0,00	0,00	100,00	100,00
0,00	0,00	0,00	100,00	100,00
0,00	0,00	0,00	100,00	100,00
0,00	0,00	0,00	100,00	100,00
0,00	0,00	0,00	100,00	100,00
0,00	0,00	0,00	100,00	100,00
0,00	0,00	0,00	100,00	100,00
0,00	0,00	0,00	100,00	99,52
38,36	0,00	1,37	100,00	100,00
0,00	0,00	0,00	100,00	100,00
0,00	0,00	0,00	100,00	100,00
0,00	0,00	0,00	100,00	100,00
0,00	0,00	0,00	100,00	100,00
0,00	0,00	0,00	0,00	0,00
0,35	0,00	0,00	100,00	99,30
0,00	0,00	0,00	100,00	100,00
0,00	0,00	0,00	100,00	100,00
0,00	0,00	0,00	100,00	100,00
0,00	0,00	0,00	100,00	100,00
0,00	0,00	0,00	100,00	100,00
0,00	0,00	0,00	100,00	100,00
0,00	0,00	0,00	100,00	100,00
0,00	0,00	0,00	100,00	100,00
0,00	0,00	0,00	100,00	100,00
0,48	0,00	0,00	100,00	100,00
0,00	0,00	0,00	100,00	99,47
0,00	0,00	0,00	100,00	100,00
0,00	0,00	0,00	100,00	99,47
0,00	0,00	0,00	100,00	100,00
0,00	0,00	0,00	100,00	100,00
0,00	0,00	0,00	100,00	100,00
0,00	0,00	0,00	100,00	100,00

0,00	0,00	0,00	100,00	100,00
0,00	0,00	0,00	100,00	100,00
2,23	0,00	0,00	100,00	100,00
0,00	0,00	0,00	100,00	100,00
0,00	0,00	0,00	0,00	0,00
0,00	0,00	0,00	100,00	100,00
0,00	0,00	0,00	0,00	0,00
0,00	0,00	0,00	100,00	100,00
0,00	0,00	0,00	100,00	100,00
0,00	0,00	0,00	100,00	100,00
0,00	0,00	0,00	100,00	100,00
0,00	0,00	0,00	100,00	100,00
0,00	0,00	0,00	100,00	99,59
0,51	0,00	0,00	100,00	100,00
0,00	0,00	0,00	100,00	100,00
0,00	0,00	0,00	100,00	100,00
0,00	0,00	0,00	0,00	0,00
0,00	0,00	0,00	100,00	100,00
0,00	0,00	0,00	100,00	100,00
0,00	0,00	0,00	100,00	100,00
0,00	0,00	0,00	100,00	100,00
0,00	0,00	0,00	100,00	99,57
0,00	0,00	0,00	100,00	100,00
0,00	0,00	0,00	100,00	100,00
0,00	0,00	0,00	100,00	100,00
0,00	0,00	0,00	100,00	100,00
0,00	0,00	0,00	100,00	100,00
0,00	0,00	0,00	99,69	99,69
3,67	0,00	0,92	100,00	100,00
0,00	0,00	0,00	100,00	100,00
0,00	0,00	0,00	100,00	100,00
0,00	0,00	0,00	100,00	100,00
0,00	0,00	0,00	100,00	100,00
0,00	0,00	0,00	100,00	100,00
0,00	0,00	0,00	100,00	100,00
0,00	0,00	0,00	100,00	100,00
0,00	0,00	0,00	100,00	100,00
0,00	0,00	0,00	100,00	100,00
0,00	0,00	0,00	100,00	98,83
0,00	0,00	0,00	100,00	97,18
0,00	0,00	0,00	100,00	100,00
0,00	0,00	0,00	100,00	100,00
0,00	0,00	0,00	100,00	100,00
0,00	0,00	0,00	100,00	100,00
0,00	0,00	0,00	100,00	100,00
0,00	0,00	0,00	100,00	100,00
0,00	0,00	0,00	100,00	100,00
0,00	0,00	0,00	99,41	99,41
0,00	0,00	0,00	100,00	100,00
0,00	0,00	0,00	100,00	100,00

0,00	0,00	0,00	100,00	100,00
0,00	0,00	0,00	100,00	100,00
0,00	0,00	0,00	100,00	99,62
0,00	0,00	0,00	100,00	100,00
0,00	0,00	0,00	100,00	100,00
0,00	0,00	0,00	100,00	100,00
0,00	0,00	0,00	100,00	100,00
0,00	0,00	0,00	100,00	100,00
0,00	0,00	0,00	100,00	100,00
0,00	0,00	0,00	100,00	100,00
0,45	0,00	0,00	100,00	100,00
0,00	0,00	0,00	100,00	100,00
0,00	0,00	0,00	100,00	100,00
0,00	0,00	0,00	100,00	97,10
0,00	0,00	0,00	100,00	99,59
2,67	0,00	0,00	100,00	100,00
2,24	0,00	0,00	100,00	99,68
0,00	0,00	0,00	100,00	100,00
0,00	0,00	0,00	100,00	100,00
0,00	0,00	0,00	100,00	100,00
0,00	0,00	0,00	100,00	100,00
0,00	0,00	0,00	100,00	100,00
0,00	0,00	0,00	100,00	100,00
0,00	0,00	0,00	100,00	100,00
0,00	0,00	0,00	100,00	100,00
0,00	0,00	0,00	100,00	99,43
0,00	0,00	0,00	100,00	100,00
0,00	0,00	0,00	100,00	100,00
0,00	0,00	0,00	99,56	99,56
0,00	0,00	0,00	100,00	100,00
0,00	0,00	0,00	100,00	100,00
0,00	0,00	0,00	100,00	100,00
11,48	0,00	0,00	100,00	100,00
0,00	0,00	0,00	100,00	100,00
0,00	0,00	0,00	100,00	100,00
0,00	0,00	0,00	100,00	100,00
0,00	0,00	0,00	0,00	0,00
0,00	0,00	0,00	100,00	100,00
0,00	0,00	0,00	100,00	100,00
0,00	0,00	0,00	100,00	100,00
0,00	0,00	0,00	100,00	100,00
0,00	0,00	0,00	100,00	100,00
0,00	0,00	0,00	100,00	100,00
0,00	0,00	0,00	100,00	100,00
0,00	0,00	0,00	100,00	100,00
0,00	0,00	0,00	100,00	100,00
0,00	0,00	0,00	100,00	100,00
0,00	0,00	0,00	100,00	100,00
0,00	0,00	0,00	0,00	0,00
0,00	0,00	0,00	100,00	100,00

0,00	0,00	0,00	100,00	100,00
0,00	0,00	0,00	100,00	100,00
0,00	0,00	0,00	100,00	100,00
0,00	0,00	0,00	0,00	0,00
0,00	0,00	0,00	100,00	100,00
0,00	0,00	0,00	100,00	100,00
0,00	0,00	0,00	97,22	63,89
1,85	0,00	0,00	98,15	98,15
0,00	0,00	0,00	100,00	100,00
0,00	0,00	1,37	98,63	98,63
0,00	0,00	0,00	100,00	94,83
1,42	0,00	0,00	100,00	100,00
0,00	0,00	0,00	0,00	0,00
0,00	0,00	0,00	100,00	100,00
0,00	0,00	0,54	99,46	99,46
1,25	0,00	0,00	100,00	100,00
0,00	0,00	0,00	100,00	96,55
0,00	0,00	0,00	100,00	100,00
0,00	0,00	3,28	96,72	36,07
0,00	0,00	0,00	100,00	100,00
0,00	0,00	0,00	100,00	97,37
0,00	0,00	0,00	100,00	99,15
0,00	0,00	0,00	100,00	100,00
0,00	0,00	0,00	100,00	100,00
0,00	0,00	0,00	0,00	0,00
0,00	0,00	0,00	100,00	100,00
0,00	0,00	0,00	100,00	100,00
0,00	0,00	0,00	100,00	100,00
0,00	0,00	0,00	100,00	100,00
0,00	0,00	0,00	100,00	100,00
0,00	0,00	0,00	100,00	100,00
0,00	0,00	0,00	100,00	100,00
0,00	0,00	0,00	100,00	94,44
0,00	0,00	0,00	0,00	0,00
0,00	0,00	0,00	100,00	100,00
0,00	0,00	0,00	100,00	100,00
0,00	0,00	0,00	100,00	100,00
0,00	0,00	0,00	100,00	100,00
0,53	0,00	0,00	100,00	100,00
0,00	0,00	0,00	100,00	100,00
0,00	0,00	0,00	100,00	99,46
0,00	0,00	0,00	100,00	100,00
0,00	0,00	0,00	100,00	100,00
0,00	0,00	0,00	100,00	100,00
0,00	0,00	0,00	100,00	100,00
0,00	0,00	0,00	100,00	100,00
0,00	0,00	0,00	100,00	100,00
0,00	0,00	0,00	100,00	99,50
0,00	0,00	0,00	100,00	100,00
0,00	0,00	0,00	100,00	97,76

0,00	0,00	0,00	100,00	100,00
0,00	0,00	0,00	100,00	100,00
0,00	0,00	0,00	0,00	0,00
0,00	0,00	0,00	100,00	96,43
0,00	0,00	0,00	100,00	100,00
0,00	0,00	0,00	100,00	98,97
0,00	0,00	0,00	100,00	98,71
0,00	0,00	0,00	100,00	98,03
0,00	0,00	0,00	100,00	98,16
0,00	0,00	0,00	100,00	100,00
0,00	0,00	0,00	100,00	100,00
0,38	0,00	0,00	100,00	100,00
0,00	0,00	0,00	100,00	100,00
0,00	0,00	0,50	100,00	99,50
0,00	0,00	0,00	100,00	99,15
0,00	0,00	0,00	100,00	100,00
0,00	0,00	0,00	100,00	100,00
0,00	0,00	0,00	100,00	100,00
0,00	0,00	0,00	100,00	100,00
0,00	0,00	0,00	100,00	99,06
0,00	0,00	0,00	100,00	100,00
0,00	0,00	0,00	100,00	100,00
0,00	0,00	0,00	100,00	100,00
0,00	0,00	0,00	99,71	99,14
0,00	0,00	0,00	100,00	100,00
0,00	0,00	0,54	100,00	100,00
0,00	0,00	0,00	100,00	100,00
0,00	0,00	0,00	100,00	100,00
0,00	0,00	0,00	100,00	100,00
0,00	0,00	0,00	99,32	99,32
0,00	0,00	0,00	100,00	99,57
0,64	0,00	0,00	100,00	100,00
0,00	0,00	0,00	100,00	100,00
0,00	0,00	0,00	100,00	100,00
0,00	0,00	0,00	100,00	100,00
0,00	0,00	0,00	100,00	94,20
0,35	0,00	0,00	99,29	98,59
0,00	0,00	0,00	100,00	100,00
0,00	0,00	0,34	100,00	98,63
0,00	0,00	0,00	100,00	100,00
0,00	0,00	0,00	100,00	100,00
0,39	0,00	0,39	100,00	99,61
0,00	0,00	0,00	100,00	97,28
0,00	0,00	0,00	100,00	96,39
0,00	0,00	0,00	100,00	99,64
0,00	0,00	0,00	99,34	99,34
0,00	0,00	0,00	100,00	100,00
0,00	0,00	0,00	100,00	100,00
0,00	0,00	0,00	100,00	100,00
0,00	0,00	0,00	100,00	100,00

0,00	0,00	0,00	100,00	100,00
0,00	0,00	0,00	100,00	100,00
0,00	0,00	0,00	100,00	100,00
0,00	0,00	0,00	100,00	100,00
0,00	0,00	0,00	100,00	100,00
0,00	0,00	0,00	100,00	100,00
0,00	0,00	0,00	100,00	100,00
0,00	0,00	0,00	100,00	100,00
0,00	0,00	0,00	100,00	100,00
0,00	0,00	0,00	100,00	82,86
0,00	0,00	0,40	98,02	97,23
0,00	0,00	0,00	100,00	100,00
0,00	0,00	0,00	100,00	100,00
0,00	0,00	0,00	100,00	100,00
0,00	0,00	0,35	100,00	100,00
0,00	0,00	0,00	100,00	100,00
0,00	0,00	0,00	99,70	99,11
0,00	0,00	0,00	100,00	98,67
0,00	0,00	0,00	99,46	97,31
0,00	0,00	0,00	100,00	99,22
0,00	0,00	0,00	99,32	97,97
0,00	0,00	0,00	100,00	100,00
0,00	0,00	0,00	100,00	100,00
0,00	0,00	0,00	100,00	100,00
0,00	0,00	0,00	100,00	100,00
0,00	0,00	0,00	100,00	100,00
0,00	0,00	0,00	100,00	100,00
0,00	0,00	0,00	100,00	85,81
0,00	0,00	0,00	100,00	100,00
0,00	0,00	0,00	100,00	100,00
0,00	0,00	0,00	100,00	100,00
0,00	0,00	0,00	99,46	99,46
0,00	0,00	0,00	100,00	99,30
0,00	0,00	0,00	100,00	100,00
0,00	0,00	0,00	100,00	100,00
0,00	0,00	0,00	100,00	100,00
0,00	0,00	0,00	98,92	98,56
0,00	0,00	0,00	100,00	100,00
0,00	0,00	0,00	100,00	100,00
0,00	0,00	0,33	100,00	100,00
0,00	0,00	0,00	100,00	100,00
0,00	0,00	0,00	100,00	100,00
0,35	0,00	0,00	100,00	99,65
0,00	0,00	0,00	100,00	89,22
0,00	0,00	0,00	100,00	100,00
0,00	0,00	0,00	100,00	100,00
0,00	0,00	0,00	100,00	100,00
0,00	0,00	0,00	100,00	100,00
0,42	0,00	0,85	100,00	95,76
0,00	0,00	0,53	98,94	97,35

0,00	0,00	0,00	99,46	98,92
0,40	0,00	0,00	99,60	98,02
0,00	0,00	0,00	100,00	100,00
0,00	0,00	0,63	99,37	99,37
0,00	0,00	0,00	100,00	92,31
0,00	0,00	0,00	100,00	91,94
0,00	0,00	0,00	100,00	100,00
0,00	0,00	0,00	100,00	100,00
0,00	0,00	0,00	100,00	100,00
0,00	0,00	0,00	100,00	100,00
0,00	0,00	0,00	100,00	100,00
0,00	0,00	0,00	100,00	99,76
0,00	0,00	0,00	100,00	96,54
0,00	0,00	0,00	100,00	98,44
0,00	0,00	0,00	100,00	100,00
0,00	0,00	0,00	100,00	100,00
0,00	0,00	0,00	100,00	100,00
0,00	0,00	0,00	100,00	100,00
0,00	0,00	0,00	100,00	100,00
0,00	0,00	0,00	100,00	100,00
0,00	0,00	0,00	99,68	99,68
0,00	0,00	0,00	100,00	100,00
0,00	0,00	0,00	100,00	100,00
0,00	0,00	0,00	100,00	100,00
0,00	0,00	0,00	100,00	100,00
0,00	0,00	0,00	100,00	99,61
0,00	0,00	1,09	100,00	100,00
0,00	0,00	0,00	100,00	100,00
0,00	0,00	0,00	100,00	96,51
0,00	0,00	0,00	100,00	100,00
0,00	0,00	0,00	100,00	97,12
0,00	0,00	0,00	100,00	99,63
0,00	0,00	0,00	100,00	100,00
0,00	0,00	0,00	100,00	95,29
0,00	0,00	0,00	99,12	80,53
1,90	0,00	1,90	98,73	69,62
0,00	0,00	0,33	100,00	87,33
0,00	0,00	0,00	100,00	100,00
0,00	0,00	0,00	100,00	82,61
0,00	0,00	0,00	100,00	41,53
0,00	0,00	0,63	99,37	98,10
0,00	0,00	1,53	98,47	97,71
0,00	0,00	0,00	100,00	10,44
0,00	0,00	0,00	100,00	100,00
0,79	0,00	0,00	100,00	91,30
0,00	0,00	0,00	99,14	99,14
0,00	0,00	0,38	100,00	96,55
0,00	0,00	0,00	99,55	99,55
0,00	0,00	0,00	100,00	100,00
0,00	0,00	0,00	100,00	100,00

0,00	0,00	0,00	100,00	100,00
0,00	0,00	0,00	100,00	100,00
0,00	0,00	0,00	100,00	100,00
0,00	0,00	0,00	100,00	100,00
0,00	0,00	0,00	100,00	100,00
0,00	0,00	0,00	100,00	100,00
0,00	0,00	0,00	100,00	100,00
0,00	0,00	0,00	99,67	99,33
0,00	0,00	0,00	100,00	100,00
0,00	0,00	0,00	100,00	100,00
0,00	0,00	0,00	100,00	99,22
0,00	0,00	0,00	100,00	100,00
0,00	0,00	0,00	100,00	100,00
0,00	0,00	0,00	100,00	100,00
0,66	0,00	0,00	100,00	100,00
0,38	0,00	0,38	100,00	100,00
0,00	0,00	0,00	100,00	100,00
0,00	0,00	0,00	100,00	99,62
0,00	0,00	0,00	100,00	100,00
0,00	0,00	0,00	100,00	100,00
0,00	0,00	0,00	100,00	100,00
0,00	0,00	0,00	100,00	99,33
0,00	0,00	0,00	100,00	99,60
0,00	0,00	0,00	100,00	100,00
0,00	0,00	0,00	100,00	100,00
0,00	0,00	0,00	100,00	100,00
0,00	0,00	0,00	100,00	99,18
0,00	0,00	0,00	100,00	99,66
0,00	0,00	0,00	100,00	94,80
0,00	0,00	0,00	100,00	99,34
0,00	0,00	0,00	0,00	0,00
1,21	0,00	0,24	99,76	67,48
0,32	0,00	2,57	99,04	96,46
0,00	0,00	0,00	99,68	99,68
0,00	0,00	0,00	100,00	100,00
0,00	0,00	0,00	99,64	98,56
0,00	0,00	0,00	100,00	100,00
0,00	0,00	0,00	100,00	100,00
0,00	0,00	0,00	99,59	98,77
0,00	0,00	0,00	100,00	100,00
0,00	0,00	0,00	100,00	100,00
0,00	0,00	0,35	100,00	100,00
0,00	0,00	0,00	100,00	100,00
0,00	0,00	0,00	100,00	100,00
0,00	0,00	0,00	100,00	100,00
0,00	0,00	0,00	100,00	100,00
0,00	0,00	0,00	100,00	100,00
0,00	0,00	0,00	100,00	100,00
0,00	0,00	0,00	100,00	99,69
0,00	0,00	0,00	100,00	100,00

0,00	0,00	0,00	100,00	100,00
0,00	0,00	0,00	100,00	98,49
0,00	0,00	0,00	100,00	100,00
0,00	0,00	0,00	100,00	100,00
0,00	0,00	0,00	100,00	99,22
0,00	0,00	0,00	100,00	83,15
0,00	0,00	0,00	100,00	95,91
0,00	0,00	0,00	100,00	99,69
0,00	0,00	0,00	100,00	100,00
0,00	0,00	0,00	100,00	100,00
0,00	0,00	0,00	100,00	99,59
0,00	0,00	1,46	99,42	95,04
0,00	0,00	0,00	100,00	100,00
0,00	0,00	0,00	100,00	100,00
0,00	0,00	0,00	100,00	100,00
0,00	0,00	0,00	100,00	100,00
0,00	0,00	0,00	100,00	100,00
0,00	0,00	0,00	100,00	100,00
0,00	0,00	0,00	100,00	100,00
0,00	0,00	0,00	100,00	100,00
0,00	0,00	0,00	100,00	100,00
0,00	0,00	0,50	100,00	100,00
0,00	0,00	0,00	100,00	100,00
0,00	0,00	0,00	100,00	100,00
0,00	0,00	0,00	100,00	99,24
0,20	0,00	0,00	100,00	97,23
0,00	0,00	0,00	100,00	97,40
0,35	0,00	0,00	99,65	83,39
50,00	0,00	13,89	100,00	2,78
0,00	0,00	0,00	100,00	75,27
0,00	0,00	0,00	100,00	99,12
0,00	0,00	0,00	100,00	98,79
0,00	0,00	0,00	100,00	92,65
0,00	0,00	1,48	98,52	90,15
0,00	0,00	0,00	99,68	15,91
0,00	0,00	0,00	98,06	85,48
0,00	0,00	0,00	100,00	100,00
0,00	0,00	0,00	100,00	100,00
0,00	0,00	1,52	100,00	31,98
0,00	0,00	0,00	100,00	100,00
0,00	0,00	0,36	100,00	100,00
0,00	0,00	0,00	99,52	96,62
0,00	0,00	0,00	100,00	75,37
0,00	0,00	0,00	98,44	97,66
0,00	0,00	0,00	100,00	100,00
0,00	0,00	0,00	100,00	100,00
0,00	0,00	0,00	100,00	100,00
0,00	0,00	0,00	100,00	100,00
0,00	0,00	0,00	100,00	99,50
0,57	0,00	0,00	100,00	100,00

0,00	0,00	0,00	100,00	100,00
0,00	0,00	0,00	100,00	100,00
0,00	0,00	0,00	100,00	100,00
0,00	0,00	0,00	100,00	100,00
0,00	0,00	0,00	100,00	100,00
0,00	0,00	0,00	100,00	100,00
0,00	0,00	0,00	100,00	100,00
0,00	0,00	0,00	100,00	100,00
0,00	0,00	0,00	100,00	100,00
0,00	0,00	0,00	100,00	99,27
0,00	0,00	0,00	100,00	100,00
0,00	0,00	0,00	100,00	99,53
0,00	0,00	0,00	100,00	100,00
0,00	0,00	0,00	100,00	100,00
0,00	0,00	0,00	100,00	100,00
0,00	0,00	0,00	100,00	100,00
0,00	0,00	0,00	99,29	99,29
0,00	0,00	0,00	100,00	100,00
0,00	0,00	0,00	100,00	100,00
0,00	0,00	0,00	100,00	100,00
0,00	0,00	0,00	100,00	100,00
0,39	0,00	0,00	100,00	100,00
0,00	0,00	0,00	100,00	100,00
0,00	0,00	0,00	100,00	100,00
0,45	0,00	0,00	100,00	99,55
0,00	0,00	0,00	100,00	100,00
0,00	0,00	0,00	100,00	100,00
0,00	0,00	0,00	100,00	100,00
0,00	0,00	0,00	100,00	100,00
0,00	0,00	0,00	100,00	100,00
0,00	0,00	0,00	100,00	100,00
0,00	0,00	0,00	100,00	100,00
0,00	0,00	0,00	100,00	100,00
0,00	0,00	0,00	100,00	100,00
0,00	0,00	0,00	100,00	100,00
0,00	0,00	0,00	0,00	0,00
0,00	0,00	0,00	100,00	72,90
0,00	0,00	0,00	100,00	94,74
0,00	0,00	0,00	100,00	100,00
0,00	0,00	0,00	100,00	63,64
0,00	0,00	0,00	100,00	100,00
0,00	0,00	0,00	0,00	0,00
0,00	0,00	0,00	100,00	100,00
0,00	0,00	0,00	100,00	100,00
0,00	0,00	0,00	100,00	100,00
0,00	0,00	0,00	100,00	100,00
0,00	0,00	0,00	100,00	99,48
0,00	0,00	0,00	100,00	100,00
0,00	0,00	0,00	100,00	100,00
0,00	0,00	0,00	100,00	100,00

0,00	0,00	0,47	100,00	36,32
0,00	0,00	0,00	100,00	94,57
0,00	0,00	0,00	100,00	100,00
0,00	0,00	0,00	100,00	100,00
0,00	0,00	0,00	100,00	100,00
0,00	0,00	0,00	100,00	94,83
0,00	0,00	0,00	100,00	100,00
0,00	0,00	0,00	100,00	100,00
0,61	0,00	0,00	100,00	100,00
0,00	0,00	0,00	100,00	65,42
0,00	0,00	0,00	0,00	0,00
0,00	0,00	0,96	100,00	100,00
0,00	0,00	0,00	100,00	100,00
0,54	0,00	0,00	100,00	100,00
0,00	0,00	0,00	100,00	100,00
0,00	0,00	0,00	100,00	100,00
0,00	0,00	0,00	100,00	100,00
0,00	0,00	0,00	0,00	0,00
0,00	0,00	0,00	100,00	46,03
0,00	0,00	0,00	100,00	97,67
0,00	0,00	0,00	100,00	100,00
0,00	0,00	0,00	100,00	100,00
0,00	0,00	0,00	100,00	98,91
0,00	0,00	0,00	100,00	100,00
0,91	0,00	0,00	100,00	7,27
0,00	0,00	0,00	100,00	100,00
0,00	0,00	0,00	100,00	100,00
0,00	0,00	0,00	100,00	100,00
0,00	0,00	0,00	100,00	100,00
0,00	0,00	0,00	100,00	100,00
0,00	0,00	0,00	100,00	50,94
0,00	0,00	0,00	100,00	100,00
0,00	0,00	0,00	100,00	98,77
0,00	0,00	0,00	100,00	100,00
0,00	0,00	0,00	99,08	96,33
0,00	0,00	0,00	99,55	99,55
0,00	0,00	0,00	100,00	100,00
0,81	0,00	0,00	100,00	100,00
0,00	0,00	0,00	100,00	84,78
0,00	0,00	0,00	100,00	99,46
0,00	0,00	0,00	100,00	100,00
0,00	0,00	0,00	100,00	100,00
0,00	0,00	0,00	100,00	100,00
0,00	0,00	0,00	100,00	100,00
0,00	0,00	0,00	100,00	100,00
0,00	0,00	0,00	100,00	100,00
0,00	0,00	0,00	100,00	99,59
0,00	0,00	0,00	100,00	100,00
0,00	0,00	0,00	100,00	97,50
0,00	0,00	0,00	100,00	100,00

0,00	0,00	0,00	100,00	100,00
0,00	0,00	0,00	100,00	100,00
0,86	0,00	0,00	100,00	99,57
0,00	0,00	0,00	100,00	100,00
0,00	0,00	0,00	100,00	12,50
0,00	0,00	0,00	0,00	0,00
0,00	0,00	0,00	99,60	99,20
0,00	0,00	0,00	100,00	100,00
0,00	0,00	0,00	100,00	100,00
0,00	0,00	0,00	100,00	100,00
0,00	0,00	0,00	100,00	100,00
0,00	0,00	0,00	100,00	88,37
0,00	0,00	0,00	100,00	100,00
0,00	0,00	0,00	99,10	91,89
0,00	0,00	0,00	98,33	98,33
0,00	0,00	1,23	100,00	2,47
0,00	0,00	0,00	100,00	10,53
0,00	0,00	0,00	100,00	100,00
0,00	0,00	0,92	99,08	92,66
0,00	0,00	0,51	99,49	79,70
0,00	0,00	0,00	100,00	95,29
0,00	0,00	0,00	100,00	100,00
0,00	0,00	0,00	100,00	100,00
0,00	0,00	0,00	100,00	99,28
0,00	0,00	0,00	100,00	98,13
0,00	0,00	0,00	100,00	100,00
2,15	0,00	0,00	100,00	100,00
0,49	0,00	0,00	100,00	99,51
0,00	0,00	0,00	100,00	100,00
0,00	0,00	0,00	100,00	98,30
0,00	0,00	0,00	100,00	98,70
0,00	0,00	0,00	100,00	100,00
0,41	0,00	0,00	100,00	100,00
0,00	0,00	0,00	100,00	100,00
0,00	0,00	0,00	100,00	100,00
0,00	0,00	0,00	100,00	99,57
0,58	0,00	0,00	100,00	98,26
0,00	0,00	0,00	100,00	99,55
0,00	0,00	0,00	100,00	100,00
0,00	0,00	0,00	0,00	0,00
0,00	0,00	0,00	100,00	98,91
1,69	0,00	0,00	100,00	100,00
0,00	0,00	0,00	100,00	100,00
0,00	0,00	0,00	100,00	100,00
0,00	0,00	0,00	100,00	100,00
0,00	0,00	0,00	100,00	97,39
0,00	0,00	0,00	100,00	99,31
0,00	0,00	0,00	100,00	100,00
1,97	0,00	0,99	100,00	85,71
0,00	0,00	0,00	100,00	88,79

0,00	0,00	0,00	100,00	100,00
0,00	0,00	0,00	100,00	90,91
0,00	0,00	0,00	0,00	0,00
0,00	0,00	0,00	100,00	98,80
0,00	0,00	0,00	100,00	99,63
0,00	0,00	0,00	100,00	99,64
0,00	0,00	0,00	100,00	89,74
0,00	0,00	0,00	100,00	89,57
0,00	0,00	0,00	100,00	99,05
0,00	0,00	0,00	100,00	98,95
0,00	0,00	0,00	100,00	99,00
0,00	0,00	0,00	100,00	94,80
0,00	0,00	0,00	100,00	100,00
0,30	0,00	0,00	100,00	100,00
0,00	0,00	0,00	100,00	100,00
0,00	0,00	0,00	100,00	100,00
0,00	0,00	0,00	100,00	100,00
0,00	0,00	0,00	100,00	100,00
0,00	0,00	0,00	100,00	90,48
0,00	0,00	0,00	100,00	99,64
0,00	0,00	0,00	99,67	98,66
0,00	0,00	0,00	100,00	100,00
0,00	0,00	0,00	100,00	99,58
0,44	0,00	0,88	99,56	32,16
0,00	0,00	0,00	100,00	98,21
0,00	0,00	0,00	100,00	97,93
0,00	0,00	0,00	100,00	82,00
0,00	0,00	1,27	98,73	83,54
0,00	0,00	0,00	100,00	100,00
0,00	0,00	0,00	100,00	95,07
0,00	0,00	0,42	100,00	100,00
0,48	0,00	0,00	100,00	88,10
0,00	0,00	0,00	99,67	96,42
0,00	0,00	0,00	99,39	99,39
0,43	0,00	0,00	100,00	99,57
0,00	0,00	0,00	100,00	98,93
0,00	0,00	0,61	100,00	98,77
0,00	0,00	0,00	100,00	100,00
0,00	0,00	0,00	100,00	100,00
0,00	0,00	0,00	98,31	98,31
0,00	0,00	0,00	100,00	98,85
0,00	0,00	0,00	99,72	99,72
0,00	0,00	0,00	100,00	100,00
0,00	0,00	0,00	100,00	99,01
0,00	0,00	0,00	100,00	99,65
0,00	0,00	0,00	100,00	48,76
0,00	0,00	0,00	100,00	56,80
0,00	0,00	0,00	100,00	100,00
0,00	0,00	2,22	100,00	82,22
0,00	0,00	0,00	0,00	0,00

0,00	0,00	0,00	100,00	100,00
0,00	0,00	0,00	100,00	100,00
0,00	0,00	0,00	99,21	99,21
0,00	0,00	0,00	100,00	28,19
0,00	0,00	0,00	100,00	100,00
0,00	0,00	0,00	100,00	98,33
0,00	0,00	0,00	98,86	98,86
0,00	0,00	0,00	100,00	100,00
0,00	0,00	0,00	99,63	99,63
0,00	0,00	0,00	100,00	100,00
0,00	0,00	0,00	100,00	100,00
0,00	0,00	0,00	100,00	99,70
0,00	0,00	0,00	99,64	99,64
0,00	0,00	0,00	100,00	100,00
0,00	0,00	0,00	100,00	100,00
0,00	0,00	0,00	100,00	100,00
0,00	0,00	0,00	100,00	100,00
0,00	0,00	0,00	100,00	100,00
0,00	0,00	0,00	100,00	100,00
0,36	0,00	0,00	99,64	99,64
0,00	0,00	0,00	100,00	100,00
0,00	0,00	0,00	100,00	100,00
0,53	0,00	0,00	100,00	100,00
0,00	0,00	0,00	100,00	100,00
0,00	0,00	0,00	100,00	100,00
0,00	0,00	0,00	100,00	96,14
0,00	0,00	0,00	100,00	98,63
0,33	0,00	0,00	100,00	100,00
0,00	0,00	0,00	99,71	99,71
0,00	0,00	0,00	100,00	100,00
0,00	0,00	0,00	100,00	100,00
0,00	0,00	0,00	99,69	99,69
0,00	0,00	0,00	100,00	100,00
0,00	0,00	0,00	100,00	100,00
0,00	0,00	0,00	100,00	100,00
0,00	0,00	0,00	98,40	97,60
0,00	0,00	0,00	99,68	99,68
0,00	0,00	0,00	99,44	99,44
0,00	0,00	0,00	100,00	99,24
0,00	0,00	0,00	100,00	100,00
0,00	0,00	0,00	100,00	100,00
0,00	0,00	0,00	100,00	100,00
0,00	0,00	0,00	100,00	100,00
0,00	0,00	0,00	100,00	100,00
0,00	0,00	0,35	100,00	100,00
0,00	0,00	0,00	100,00	100,00
0,00	0,00	0,00	100,00	100,00
0,00	0,00	0,00	100,00	100,00
0,00	0,00	0,00	100,00	100,00
0,00	0,00	0,00	100,00	100,00

0,00	0,00	0,00	100,00	99,66
0,00	0,00	0,00	100,00	99,34
0,00	0,00	0,00	99,63	97,79
0,00	0,00	0,00	100,00	100,00
0,00	0,00	0,00	100,00	100,00
0,00	0,00	0,00	100,00	100,00
0,00	0,00	0,00	99,39	99,39
0,33	0,00	0,00	99,67	99,67
0,00	0,00	0,00	100,00	98,82
0,00	0,00	0,00	100,00	100,00
0,00	0,00	0,00	100,00	99,25
0,00	0,00	0,00	100,00	97,64
0,00	0,00	0,00	100,00	100,00
0,00	0,00	0,00	100,00	100,00
0,00	0,00	0,00	100,00	100,00
0,00	0,00	0,00	100,00	99,59
12,96	0,00	0,00	100,00	100,00
0,00	0,00	0,00	100,00	99,25
0,00	0,00	0,00	100,00	99,10
0,00	0,00	0,34	100,00	96,97
0,00	0,00	0,00	100,00	99,01
0,00	0,00	0,00	100,00	97,76
0,00	0,00	0,00	100,00	100,00
0,00	0,00	0,00	100,00	100,00
0,00	0,00	0,00	100,00	100,00
0,00	0,00	0,00	100,00	100,00
0,00	0,00	0,00	99,27	99,27
0,00	0,00	0,00	100,00	100,00
0,00	0,00	0,00	100,00	100,00
0,00	0,00	0,00	100,00	98,87
0,00	0,00	0,00	100,00	100,00
0,00	0,00	0,00	100,00	100,00
0,00	0,00	0,00	100,00	100,00
0,35	0,00	0,00	100,00	99,31
0,25	0,00	0,00	100,00	100,00
0,00	0,00	0,00	100,00	99,63
0,00	0,00	0,00	100,00	100,00
0,00	0,00	0,00	100,00	100,00
0,00	0,00	0,00	100,00	100,00
0,00	0,00	0,00	100,00	100,00
0,00	0,00	0,00	100,00	100,00
0,31	0,00	0,00	100,00	100,00
0,00	0,00	0,00	100,00	100,00
0,00	0,00	0,00	100,00	100,00
0,00	0,00	0,00	100,00	100,00
0,00	0,00	0,00	100,00	100,00
0,00	0,00	0,00	100,00	100,00
0,00	0,00	0,00	100,00	99,62
0,00	0,00	0,00	100,00	99,58
0,00	0,00	0,00	100,00	100,00

0,00	0,00	0,00	100,00	100,00
0,00	0,00	0,00	100,00	99,13
0,00	0,00	0,00	100,00	99,59
0,00	0,00	0,00	100,00	100,00
0,00	0,00	0,00	100,00	100,00
0,00	0,00	0,00	100,00	99,36
0,00	0,00	0,00	99,32	95,90
0,00	0,00	0,00	100,00	98,70
0,00	0,00	0,00	100,00	98,58
0,00	0,00	0,00	99,54	98,17
0,48	0,00	0,00	100,00	98,57
0,00	0,00	0,00	100,00	100,00
0,00	0,00	0,00	100,00	94,87
0,00	0,00	0,00	100,00	100,00
0,33	0,00	0,00	100,00	99,67
0,00	0,00	0,00	100,00	100,00
0,00	0,00	0,00	100,00	100,00
0,00	0,00	0,29	100,00	95,32
0,00	0,00	0,00	100,00	92,75
0,00	0,00	0,32	100,00	97,76
0,00	0,00	0,00	100,00	99,40
0,00	0,00	0,00	100,00	100,00
0,00	0,00	0,00	100,00	98,71
0,00	0,00	0,34	100,00	100,00
0,00	0,00	0,00	100,00	100,00
0,00	0,00	0,00	100,00	99,53
0,00	0,00	0,00	100,00	100,00
0,00	0,00	0,00	100,00	96,08
0,00	0,00	1,01	100,00	98,99
1,05	0,00	0,70	98,95	90,59
0,00	0,00	0,00	100,00	100,00
0,00	0,00	0,00	100,00	99,66
0,00	0,00	0,00	100,00	100,00
0,00	0,00	0,00	100,00	100,00
0,75	0,00	0,00	100,00	99,62
0,00	0,00	0,00	100,00	100,00
0,00	0,00	0,00	100,00	98,64
0,00	0,00	0,00	100,00	98,67
0,00	0,00	0,00	100,00	100,00
0,50	0,00	0,00	100,00	99,00
0,00	0,00	0,00	0,00	0,00
0,00	0,00	0,00	100,00	99,07
0,00	0,00	0,00	0,00	0,00
0,00	0,00	0,00	100,00	99,59
0,00	0,00	0,00	100,00	100,00
0,00	0,00	0,00	100,00	100,00
0,00	0,00	0,00	100,00	99,07
0,00	0,00	0,00	100,00	100,00
0,00	0,00	0,00	100,00	100,00
0,00	0,00	0,00	100,00	100,00

0,00	0,00	0,00	100,00	100,00
0,00	0,00	0,00	100,00	99,35
0,00	0,00	2,27	100,00	95,45
0,00	0,00	0,00	100,00	100,00
0,00	0,00	0,00	100,00	99,65
0,00	0,00	0,00	100,00	100,00
0,00	0,00	0,00	99,47	99,47
0,00	0,00	0,00	100,00	100,00
0,00	0,00	0,00	100,00	96,97
0,00	0,00	0,00	100,00	100,00
0,00	0,00	0,00	100,00	100,00
0,00	0,00	0,00	100,00	100,00
0,41	0,00	0,00	100,00	100,00
0,00	0,00	0,00	100,00	100,00
0,00	0,00	0,00	100,00	100,00
0,00	0,00	0,00	100,00	100,00
0,00	0,00	0,00	100,00	100,00
0,00	0,00	0,00	100,00	100,00
0,00	0,00	0,00	100,00	100,00
0,00	0,00	0,00	100,00	100,00
0,00	0,00	0,00	100,00	100,00
0,00	0,00	0,00	100,00	100,00
0,00	0,00	0,00	100,00	100,00
0,00	0,00	0,00	100,00	100,00
0,00	0,00	0,00	100,00	100,00
0,00	0,00	0,00	100,00	100,00
0,00	0,00	0,00	100,00	76,50
0,00	0,00	0,00	98,77	98,77
0,00	0,00	0,00	100,00	100,00
0,00	0,00	0,00	100,00	100,00
0,00	0,00	1,61	100,00	100,00
0,70	0,00	0,00	100,00	99,65
0,00	0,00	0,00	100,00	100,00
0,00	0,00	0,00	0,00	0,00
0,00	0,00	0,00	100,00	100,00
0,00	0,00	0,00	100,00	100,00
0,00	0,00	0,00	100,00	100,00
0,00	0,00	0,00	100,00	100,00
0,00	0,00	0,00	100,00	100,00
0,00	0,00	0,00	100,00	100,00
0,00	0,00	0,00	100,00	100,00
0,00	0,00	0,00	100,00	100,00
0,00	0,00	0,00	100,00	100,00
0,00	0,00	0,00	100,00	98,69
0,00	0,00	0,00	100,00	100,00
0,00	0,00	0,00	100,00	99,36
0,00	0,00	0,00	100,00	100,00
0,00	0,00	0,00	100,00	100,00
0,00	0,00	0,00	100,00	100,00
0,00	0,00	0,00	100,00	100,00

0,00	0,00	0,00	100,00	100,00
0,00	0,00	0,00	100,00	100,00
0,00	0,00	0,00	100,00	100,00
0,00	0,00	0,00	97,92	97,92
0,00	0,00	0,00	100,00	100,00
0,00	0,00	0,00	100,00	97,87
0,00	0,00	0,00	100,00	100,00
1,26	0,00	0,00	100,00	100,00
1,32	0,00	0,00	100,00	100,00
0,00	0,00	0,00	100,00	100,00
0,00	0,00	0,00	100,00	100,00
0,00	0,00	0,00	0,00	0,00
0,00	0,00	0,00	100,00	100,00
0,00	0,00	0,00	100,00	100,00
0,00	0,00	0,00	100,00	90,00
0,00	0,00	0,00	100,00	100,00
1,15	0,00	0,00	100,00	98,85
0,00	0,00	2,84	100,00	58,87
0,00	0,00	0,00	100,00	100,00
0,00	0,00	0,00	100,00	100,00
0,00	0,00	0,00	100,00	99,58
0,00	0,00	0,00	100,00	100,00
0,00	0,00	0,00	100,00	100,00
0,00	0,00	0,00	0,00	0,00
0,00	0,00	0,00	100,00	100,00
0,00	0,00	0,00	100,00	98,88
0,00	0,00	0,00	100,00	99,62
0,00	0,00	0,00	100,00	100,00
0,00	0,00	0,00	100,00	100,00
0,00	0,00	0,00	100,00	100,00
0,00	0,00	0,00	100,00	100,00
0,00	0,00	0,41	99,59	99,59
0,34	0,00	0,00	100,00	100,00
0,00	0,00	0,00	98,46	98,46
0,00	0,00	0,00	100,00	100,00
0,00	0,00	0,00	100,00	99,25
0,00	0,00	0,50	100,00	100,00
0,00	0,00	0,00	100,00	100,00
0,00	0,00	0,00	100,00	100,00
0,00	0,00	0,00	0,00	0,00
0,00	0,00	0,00	100,00	99,41
0,00	0,00	0,00	100,00	100,00
0,00	0,00	0,00	100,00	100,00
0,00	0,00	0,00	100,00	100,00
0,00	0,00	0,00	100,00	100,00
0,00	0,00	0,40	100,00	98,80
0,00	0,00	0,00	100,00	99,03
0,00	0,00	0,00	100,00	100,00
0,00	0,00	0,00	100,00	100,00
0,00	0,00	0,00	100,00	99,22

0,00	0,00	0,00	0,00	0,00
0,00	0,00	0,69	100,00	97,22
0,00	0,00	0,00	100,00	100,00
0,00	0,00	0,00	100,00	98,45
0,00	0,00	0,00	99,64	98,57
0,00	0,00	0,00	100,00	99,08
0,00	0,00	0,00	100,00	99,58
0,00	0,00	0,00	100,00	100,00
0,00	0,00	0,00	100,00	100,00
0,00	0,00	0,00	99,62	99,62
0,00	0,00	0,00	100,00	100,00
0,00	0,00	0,00	100,00	100,00
0,00	0,00	0,00	100,00	95,50
0,00	0,00	0,00	100,00	99,29
0,00	0,00	0,00	100,00	72,56
0,00	0,00	0,00	99,36	91,03
0,00	0,00	0,56	100,00	98,88
0,00	0,00	0,00	100,00	100,00
0,00	0,00	0,00	100,00	98,39
0,39	0,00	0,00	100,00	98,84
0,00	0,00	0,00	100,00	100,00
0,00	0,00	0,00	100,00	100,00
0,00	0,00	0,00	100,00	100,00
0,00	0,00	0,00	100,00	97,62
0,00	0,00	0,00	100,00	100,00
0,00	0,00	0,00	100,00	100,00
0,00	0,00	0,00	100,00	100,00
0,00	0,00	0,00	100,00	97,77
0,00	0,00	0,00	100,00	99,32
0,00	0,00	0,00	100,00	98,84
0,00	0,00	0,00	100,00	98,07
0,00	0,00	0,00	100,00	100,00
0,00	0,00	0,00	100,00	100,00
0,00	0,00	0,00	100,00	100,00
0,00	0,00	0,00	100,00	100,00
0,00	0,00	0,00	100,00	100,00
0,00	0,00	0,00	100,00	100,00
0,00	0,00	0,00	100,00	99,42
0,00	0,00	0,00	100,00	100,00
0,00	0,00	0,00	100,00	100,00
0,00	0,00	0,00	100,00	100,00
0,00	0,00	0,00	100,00	100,00
0,00	0,00	0,00	100,00	100,00
0,00	0,00	0,00	100,00	100,00
0,00	0,00	1,33	98,67	98,67
0,00	0,00	0,00	100,00	99,41
0,00	0,00	0,00	100,00	100,00
0,00	0,00	0,00	100,00	100,00
0,00	0,00	0,00	100,00	100,00
0,00	0,00	0,00	100,00	99,66

0,00	0,00	0,00	99,53	99,53
0,00	0,00	0,00	100,00	100,00
0,00	0,00	0,43	100,00	99,57
0,00	0,00	0,00	100,00	99,32
0,00	0,00	0,00	100,00	100,00
0,00	0,52	0,00	100,00	98,95
0,00	0,00	0,00	100,00	100,00
0,00	0,00	0,00	100,00	98,72
0,00	0,00	0,00	100,00	100,00
0,00	0,00	0,00	100,00	99,67
0,00	0,00	0,00	100,00	100,00
0,00	0,00	0,00	100,00	99,57
0,00	0,00	0,00	99,55	99,55
0,00	0,00	0,00	100,00	100,00
0,00	0,00	0,00	100,00	99,62
0,00	0,00	0,00	99,68	99,68
0,00	0,00	0,00	100,00	98,86
0,00	0,00	0,00	100,00	100,00
0,00	0,00	0,00	100,00	99,39
0,00	0,00	0,00	100,00	100,00
0,00	0,00	0,00	100,00	97,34
0,00	0,00	0,00	99,60	99,60
0,00	0,00	0,34	99,32	99,32
0,00	0,00	0,00	100,00	100,00
0,00	0,00	0,00	100,00	95,04
0,26	0,00	0,00	100,00	97,94
0,00	0,00	0,00	99,58	98,75
0,00	0,00	0,00	100,00	98,62
0,00	0,00	0,00	100,00	100,00
0,00	0,00	1,14	100,00	98,86
0,00	0,00	0,00	100,00	98,95
0,00	0,00	0,00	100,00	98,85
0,00	0,00	0,00	100,00	100,00
0,00	0,00	0,00	100,00	100,00
0,00	0,00	0,00	99,28	13,67
0,00	0,00	0,75	99,25	13,53
0,00	0,00	0,00	100,00	87,65
0,00	0,00	0,00	100,00	99,66
0,00	0,00	0,00	100,00	97,97
0,00	0,00	0,00	100,00	100,00
0,00	0,00	0,00	100,00	99,68
0,00	0,00	0,00	100,00	100,00
0,00	0,00	0,00	100,00	100,00
0,00	0,00	0,00	100,00	100,00
0,00	0,00	0,00	100,00	100,00
0,00	0,00	0,00	100,00	100,00
0,00	0,00	0,00	100,00	99,62
0,00	0,00	0,00	100,00	99,52
0,00	0,00	0,00	100,00	100,00
0,00	0,00	0,00	100,00	100,00

0,00	0,00	0,00	100,00	100,00
0,00	0,00	0,00	100,00	99,53
0,51	0,00	0,00	100,00	100,00
0,00	0,00	0,00	100,00	100,00
0,00	0,00	0,00	100,00	100,00
0,00	0,00	0,00	100,00	100,00
0,00	0,00	0,00	100,00	100,00
0,00	0,00	0,00	100,00	100,00
0,00	0,00	0,00	100,00	100,00
0,00	0,00	0,00	0,00	0,00
0,00	0,00	0,00	0,00	0,00
0,00	0,00	0,00	100,00	100,00
0,00	0,00	0,00	0,00	0,00
1,52	0,00	0,00	100,00	100,00
0,00	0,00	0,00	100,00	95,60
0,00	0,00	0,00	100,00	100,00
0,00	0,00	5,00	100,00	45,00
0,00	0,00	0,00	0,00	0,00
2,74	0,00	0,00	100,00	95,89
0,00	0,00	0,00	100,00	97,84
0,00	0,00	0,89	100,00	84,82
0,00	0,00	0,00	100,00	100,00
0,00	0,00	0,00	100,00	100,00
0,00	0,00	0,00	100,00	100,00
0,00	0,00	0,00	100,00	100,00
0,00	0,00	1,25	100,00	96,25
0,00	0,00	0,00	100,00	100,00
0,00	0,00	0,00	100,00	100,00
0,00	0,00	0,00	100,00	99,61
0,00	0,00	0,00	100,00	100,00
0,00	0,00	0,00	100,00	100,00
0,00	0,00	0,00	100,00	100,00
0,00	0,00	0,00	100,00	97,92
0,00	0,00	0,00	100,00	99,36
0,00	0,00	0,00	100,00	99,67
0,00	0,00	0,42	99,58	99,58
0,00	0,00	0,00	100,00	100,00
0,00	0,00	0,00	100,00	100,00
0,64	0,00	0,00	100,00	100,00
0,00	0,00	0,00	100,00	100,00
0,00	0,00	0,00	99,28	99,28
0,00	0,00	0,00	100,00	100,00
0,00	0,00	0,00	0,00	0,00
0,00	0,00	0,00	100,00	100,00
0,00	0,00	0,00	100,00	98,55
0,00	0,00	0,00	100,00	98,77
0,00	0,00	0,00	100,00	99,70
0,00	0,00	0,00	100,00	100,00
0,00	0,00	0,00	100,00	100,00
0,00	0,00	0,00	100,00	100,00

0,00	0,00	0,31	99,69	95,08
0,00	0,00	0,00	0,00	0,00
0,00	0,00	0,00	100,00	100,00
0,00	0,00	0,00	100,00	99,51
0,00	0,00	0,66	99,67	92,03
0,00	0,00	0,31	100,00	98,15
0,00	0,00	0,00	100,00	100,00
0,33	0,00	0,00	99,67	99,67
0,00	0,00	0,00	100,00	98,98
0,00	0,00	0,00	100,00	100,00
0,70	0,00	0,00	100,00	100,00
0,00	0,00	0,00	100,00	94,58
0,00	0,00	0,00	95,28	94,49
0,00	0,00	0,00	100,00	92,49
0,00	0,00	0,32	100,00	89,91
0,00	0,00	0,00	100,00	100,00
0,00	0,00	0,00	100,00	100,00
0,00	0,00	0,00	100,00	100,00
0,00	0,00	0,00	100,00	100,00
0,00	0,00	0,00	100,00	99,60
0,00	0,00	0,00	100,00	100,00
0,00	0,00	0,00	100,00	100,00
0,00	0,00	0,00	100,00	97,52
0,00	0,00	0,00	100,00	100,00
0,00	0,00	0,00	100,00	100,00
0,00	0,00	0,00	100,00	100,00
0,00	0,00	0,00	100,00	98,45
0,00	0,00	0,00	100,00	81,82
0,00	0,00	0,00	99,44	97,18
0,00	0,00	0,00	100,00	100,00
0,00	0,00	0,00	100,00	98,25
0,00	0,00	0,00	100,00	100,00
0,00	0,00	0,00	100,00	100,00
0,00	0,00	0,00	100,00	100,00
0,38	0,00	0,00	100,00	94,62
0,00	0,00	0,00	100,00	99,32
0,00	0,00	0,00	100,00	100,00
0,00	0,00	0,00	100,00	100,00
0,00	0,00	0,50	99,50	99,50
0,00	0,00	0,61	100,00	98,18
0,00	0,00	0,00	100,00	98,44
0,00	0,00	0,00	100,00	100,00
0,00	0,00	0,00	100,00	99,55
0,00	0,00	0,00	100,00	100,00
0,00	0,00	0,00	99,54	99,08
0,00	0,00	0,00	100,00	100,00
0,00	0,00	0,00	100,00	99,17
0,51	0,00	0,00	100,00	99,49
0,00	0,00	0,00	100,00	99,66
0,00	0,00	0,00	100,00	99,48

0,00	0,00	0,00	100,00	99,71
1,04	0,00	0,00	100,00	98,96
0,00	0,00	0,00	99,46	96,75
0,00	0,00	0,36	100,00	100,00
0,00	0,00	0,00	100,00	100,00
0,00	0,00	0,00	100,00	100,00
0,42	0,00	0,00	100,00	99,16
0,00	0,00	0,00	100,00	79,05
0,32	0,00	0,00	100,00	100,00
1,76	0,00	0,29	100,00	92,35
0,00	0,00	0,00	100,00	99,68
0,00	0,00	0,00	100,00	100,00
0,00	0,00	0,00	99,08	92,66
0,00	0,00	0,00	99,50	43,78
0,00	0,00	0,00	99,31	90,34
0,00	0,00	0,00	100,00	96,60
0,00	0,00	0,00	100,00	100,00
0,00	0,00	0,42	100,00	100,00
0,94	0,00	0,00	100,00	88,73
0,00	0,00	0,00	100,00	65,22
0,00	0,00	0,00	100,00	99,13
0,00	0,00	0,00	99,61	98,84
0,00	0,00	0,00	100,00	99,56
0,48	0,00	0,00	100,00	88,57
0,00	0,00	0,00	100,00	96,77
0,00	0,00	0,00	100,00	100,00
0,00	0,00	0,00	100,00	97,44
0,00	0,00	0,00	100,00	100,00
1,49	0,00	0,00	99,63	99,26
0,00	0,00	0,00	100,00	96,38
1,89	0,94	0,00	100,00	99,06
0,00	0,00	0,46	99,77	90,16
0,00	0,00	0,38	100,00	98,85
0,00	0,00	0,00	100,00	100,00
0,00	0,38	0,00	100,00	99,62
0,00	0,00	0,00	100,00	99,38
0,00	0,00	0,00	100,00	100,00
0,00	0,00	0,00	100,00	99,22
0,00	0,00	0,37	100,00	100,00
0,23	0,00	0,00	100,00	96,04
0,49	0,00	0,00	100,00	75,24
0,00	0,00	0,00	100,00	98,82
0,00	0,00	0,00	100,00	99,65
0,00	0,00	0,00	100,00	100,00
0,00	0,00	0,00	100,00	100,00
0,00	0,00	0,49	100,00	99,75
0,28	0,00	0,00	100,00	98,90
0,24	0,00	0,00	100,00	99,52
0,32	0,00	0,00	100,00	99,05
0,00	0,00	0,00	100,00	99,56

0,55	0,00	0,00	100,00	100,00
0,00	0,00	0,00	100,00	100,00
0,87	0,00	0,00	100,00	97,38
0,00	0,00	0,00	100,00	99,53
0,00	0,00	0,00	100,00	99,62
0,39	0,00	0,00	100,00	99,21
0,00	0,00	0,00	100,00	99,29
0,00	0,00	0,00	100,00	99,70
0,98	0,00	0,00	100,00	99,67
2,04	0,00	0,00	100,00	99,32
0,00	0,00	0,00	100,00	99,63
0,00	0,00	0,30	100,00	99,70
0,00	0,00	0,00	100,00	100,00
0,00	0,00	0,33	100,00	99,34
0,00	0,00	0,00	100,00	100,00
0,00	0,00	0,00	100,00	99,67
0,00	0,00	0,00	100,00	99,66
0,00	0,00	0,00	100,00	100,00
0,00	0,00	0,00	100,00	100,00
0,00	0,00	0,00	100,00	100,00
0,00	0,00	0,00	100,00	100,00
0,00	0,00	0,00	100,00	100,00
0,00	0,00	0,00	100,00	100,00
0,00	0,00	0,00	100,00	100,00
0,00	0,00	0,00	100,00	99,36
0,00	0,00	0,00	100,00	100,00
0,00	0,00	0,00	100,00	100,00
0,00	0,00	0,00	100,00	100,00
0,00	0,00	0,00	100,00	99,37
0,00	0,00	0,00	100,00	81,62
0,00	0,00	0,00	100,00	92,59
0,00	0,00	0,00	0,00	0,00
0,00	0,00	0,00	100,00	97,06
0,00	0,00	0,00	98,04	98,04
0,00	0,00	0,00	98,84	1,16
0,00	0,00	0,00	100,00	85,71
0,00	0,00	0,00	100,00	21,33
0,00	0,00	0,33	99,67	95,41
0,00	0,00	0,00	99,32	98,99
0,00	0,00	0,00	99,65	97,88
0,00	0,00	0,39	100,00	97,66
0,00	0,00	0,00	100,00	98,62
0,00	0,40	0,00	100,00	99,21
0,00	0,98	0,00	100,00	99,51
0,00	0,00	0,84	100,00	97,48
0,00	0,00	0,00	99,70	96,95
0,00	0,00	0,00	100,00	100,00
0,00	0,00	0,00	100,00	100,00
0,00	0,00	0,00	99,32	82,31
0,00	0,00	0,00	100,00	91,30

0,00	0,00	0,00	100,00	100,00
0,00	0,00	0,54	99,46	96,22
0,00	0,00	0,00	0,00	0,00
0,27	0,00	0,54	100,00	91,35
0,00	0,00	0,00	100,00	99,25
0,00	0,00	0,30	99,70	99,40
0,00	0,00	0,00	100,00	89,19
0,00	0,00	0,00	100,00	98,72
0,00	0,00	0,00	100,00	100,00
0,38	0,00	0,00	100,00	96,24
0,00	0,00	0,38	100,00	98,47
0,00	0,00	0,00	100,00	100,00
0,00	0,00	0,00	100,00	100,00
0,00	0,00	0,00	100,00	100,00
0,00	0,00	0,00	98,88	98,88
2,14	0,00	0,36	100,00	82,86
14,84	0,00	0,35	100,00	98,94
0,00	0,00	0,00	100,00	100,00
0,00	0,00	0,00	100,00	100,00
0,00	0,00	0,37	100,00	99,26
0,00	0,00	0,00	100,00	98,68
0,00	0,00	0,00	100,00	99,17
0,00	0,00	0,00	100,00	99,60
0,00	0,00	0,00	100,00	97,89
0,00	0,00	0,00	99,06	99,06
0,00	0,00	0,00	100,00	98,72
0,00	0,00	0,67	100,00	96,31
0,85	0,00	0,00	100,00	89,83
0,00	1,69	0,00	100,00	100,00
0,00	0,00	0,34	99,31	99,31
0,00	0,00	0,00	100,00	98,13
0,00	0,00	0,42	100,00	99,58
0,00	0,00	0,00	100,00	99,13
0,00	0,00	0,00	100,00	100,00
0,00	0,00	0,00	100,00	98,93
0,00	0,00	0,00	100,00	98,02
0,00	0,00	0,00	100,00	100,00
0,00	0,00	0,40	99,60	99,20
0,00	0,00	0,00	100,00	100,00
0,00	0,00	0,00	100,00	80,65
0,00	0,00	0,00	100,00	100,00
0,00	0,00	0,00	100,00	100,00
0,00	0,00	0,00	99,63	99,63
0,00	0,00	0,00	100,00	98,15
0,00	0,00	0,00	100,00	99,52
0,00	0,00	0,00	99,61	99,61
0,00	0,00	0,00	100,00	100,00
0,32	0,00	0,32	99,05	98,11
0,00	0,00	0,40	100,00	99,60
0,00	0,00	0,00	100,00	99,08

0,00	0,00	0,00	100,00	100,00
0,36	0,00	0,00	100,00	100,00
0,00	0,00	0,00	100,00	100,00
0,00	0,00	0,00	100,00	100,00
0,00	0,00	0,00	100,00	100,00
0,00	0,00	0,00	100,00	99,04
0,00	0,00	0,00	100,00	98,28
0,00	0,00	0,00	98,86	82,29
0,00	0,00	0,00	99,58	99,58
0,00	0,00	0,00	100,00	100,00
0,00	0,00	0,00	100,00	97,71
0,00	0,00	0,00	100,00	100,00
0,00	0,00	0,00	100,00	100,00
0,00	0,00	0,34	99,66	98,99
0,00	0,00	0,00	100,00	99,63
0,00	0,00	0,00	100,00	100,00
0,00	0,00	0,00	100,00	100,00
0,00	0,00	0,00	100,00	99,54
0,00	0,00	0,00	100,00	100,00
0,00	0,00	0,40	99,60	99,60
0,00	0,00	0,00	100,00	100,00
0,00	0,00	0,00	100,00	100,00
0,00	0,00	0,00	100,00	100,00
0,00	0,00	0,00	100,00	100,00
0,00	0,00	0,00	100,00	99,64
0,00	0,00	0,00	100,00	92,76
0,00	0,00	0,00	100,00	99,59
0,47	0,00	0,00	100,00	99,53
0,00	0,00	0,00	100,00	99,32
0,00	0,00	0,00	100,00	100,00
0,00	0,00	0,00	100,00	100,00
0,00	0,00	0,00	100,00	99,59
0,00	0,00	0,00	100,00	100,00
0,00	0,00	0,00	100,00	100,00
0,00	0,00	0,00	100,00	100,00
0,00	0,00	0,00	100,00	100,00
0,00	0,00	0,00	100,00	100,00
0,00	0,00	0,00	100,00	100,00
0,00	0,00	0,00	100,00	100,00
0,00	0,00	0,00	100,00	100,00
0,00	0,00	0,00	100,00	100,00
0,00	0,00	0,00	100,00	100,00
0,00	0,00	0,00	100,00	100,00
0,00	0,00	0,00	100,00	100,00
0,00	0,00	0,00	100,00	100,00
0,00	0,00	0,00	100,00	99,56
0,00	0,00	0,00	100,00	100,00
0,00	0,00	0,00	100,00	100,00
0,00	0,00	0,00	100,00	100,00
0,00	0,00	0,00	100,00	100,00

96,08	0,00	0,00	100,00	100,00
0,00	0,00	0,00	100,00	100,00
0,00	0,00	0,00	0,00	0,00
0,00	0,00	0,00	100,00	100,00
0,00	0,00	0,00	100,00	100,00
0,00	0,00	0,00	100,00	76,53
0,94	0,00	0,00	100,00	100,00
0,00	0,00	0,00	100,00	100,00
0,00	0,00	0,00	100,00	100,00
0,00	0,00	0,00	100,00	100,00
0,00	0,00	0,00	100,00	50,00
0,00	0,00	0,00	100,00	100,00
0,00	0,00	0,00	100,00	100,00
0,00	0,00	0,00	100,00	100,00
0,00	0,00	0,00	100,00	100,00
0,00	0,00	0,00	100,00	100,00
0,00	0,00	2,44	100,00	100,00
0,00	0,00	0,00	100,00	100,00
0,00	0,00	0,00	100,00	100,00
0,00	0,00	0,00	100,00	100,00
0,00	0,00	0,00	100,00	100,00
0,00	0,00	0,00	100,00	100,00
2,04	0,00	0,00	100,00	100,00
0,00	0,00	1,56	100,00	98,44
0,00	0,00	0,00	100,00	100,00
0,00	0,00	0,00	100,00	98,39
0,00	0,00	0,00	100,00	98,77
0,00	0,00	0,00	100,00	94,12
0,00	0,00	0,00	100,00	100,00
0,00	0,00	0,00	100,00	100,00
0,00	0,00	0,00	100,00	100,00
0,00	0,00	0,00	100,00	99,54
0,00	0,00	0,00	100,00	100,00
0,00	0,00	0,00	100,00	100,00
0,00	0,00	0,00	100,00	100,00
0,00	0,00	0,00	100,00	100,00
0,00	0,00	0,00	100,00	100,00
0,00	0,00	0,00	100,00	100,00
0,00	0,00	0,00	100,00	100,00
0,00	0,00	0,00	100,00	100,00
0,00	0,00	0,00	100,00	36,17
0,00	0,00	0,00	100,00	100,00
0,00	0,00	0,00	100,00	98,51
0,00	0,00	0,00	100,00	100,00
0,00	0,00	0,69	100,00	64,58
0,00	0,00	0,00	100,00	100,00
0,00	0,00	5,00	100,00	100,00
0,00	0,00	0,00	100,00	100,00
0,00	0,00	0,00	100,00	100,00
0,00	0,00	0,00	100,00	97,92

0,00	0,00	0,00	100,00	100,00
0,00	0,00	0,00	100,00	100,00
0,69	0,00	0,00	100,00	99,31
0,00	0,00	0,00	100,00	100,00
0,00	0,00	0,00	100,00	100,00
0,00	0,00	0,00	100,00	100,00
0,00	0,00	0,00	100,00	100,00
0,00	0,00	0,00	100,00	100,00
0,00	0,00	0,00	100,00	100,00
0,00	0,00	0,00	100,00	100,00
0,00	0,00	0,00	100,00	98,05
0,00	0,00	0,00	100,00	91,95
0,00	0,00	0,00	98,84	98,84
0,00	0,00	0,00	100,00	100,00
0,00	0,00	0,00	100,00	100,00
0,00	0,00	0,00	100,00	100,00
0,00	0,00	0,00	100,00	99,58
0,00	0,00	0,00	100,00	100,00
1,32	0,00	0,00	100,00	72,19
0,00	0,00	0,00	100,00	100,00
0,00	0,00	0,00	100,00	99,67
0,00	0,00	0,00	100,00	100,00
0,00	0,00	0,00	0,00	0,00
0,00	0,00	0,00	100,00	99,09
0,00	0,00	0,00	100,00	100,00
0,00	0,00	0,00	100,00	100,00
0,00	0,00	0,00	100,00	82,35
0,00	0,00	0,00	100,00	96,30
0,00	0,00	0,00	100,00	100,00
0,00	0,00	0,00	0,00	0,00
0,00	0,00	0,00	100,00	95,29
0,00	0,00	0,00	100,00	100,00
0,00	0,00	0,00	0,00	0,00
0,00	0,00	0,00	100,00	100,00
0,00	0,00	0,00	100,00	100,00
0,00	0,00	0,00	100,00	100,00
0,00	0,00	0,00	100,00	100,00
0,00	0,00	0,00	100,00	98,89
0,00	0,00	0,00	98,80	96,39
0,00	0,00	0,00	100,00	96,08
0,00	0,00	0,00	100,00	100,00
0,00	0,00	0,00	100,00	98,48
0,00	0,00	0,00	100,00	95,45
0,00	0,00	0,00	100,00	97,06
0,00	0,00	0,00	100,00	100,00
0,00	0,00	0,00	100,00	100,00
0,00	0,00	0,00	100,00	98,90
0,00	0,00	0,00	100,00	100,00
0,00	0,00	0,00	100,00	100,00
0,00	0,00	0,00	100,00	100,00

0,00	0,00	0,00	100,00	100,00
0,00	0,00	0,00	100,00	100,00
0,00	0,00	0,00	100,00	100,00
0,00	0,00	0,00	100,00	100,00
0,00	0,00	0,00	100,00	100,00
0,00	0,00	0,00	100,00	100,00
0,00	0,00	0,00	100,00	100,00
0,00	0,00	0,00	100,00	99,13
0,00	0,00	0,00	100,00	98,31
0,00	0,00	0,00	99,50	93,07
0,00	0,00	0,00	100,00	100,00
0,00	0,00	0,00	100,00	100,00
0,00	0,00	0,00	100,00	100,00
0,00	0,00	0,00	100,00	100,00
0,00	0,00	0,00	98,88	92,13
0,00	0,00	0,00	100,00	95,31
0,00	0,00	0,00	100,00	100,00
0,00	0,00	0,00	100,00	100,00
0,00	0,00	0,00	100,00	100,00
0,00	0,00	0,00	100,00	100,00
0,00	0,00	0,97	100,00	87,38
0,00	0,00	0,00	100,00	87,50
0,00	0,00	0,00	0,00	0,00
0,00	0,00	0,00	100,00	100,00
0,00	0,00	0,00	100,00	100,00
0,00	0,00	0,00	100,00	100,00
0,00	0,00	0,00	100,00	45,79
0,00	0,00	0,00	100,00	60,00
0,00	0,00	0,00	100,00	100,00
0,00	0,00	0,00	100,00	100,00
0,00	0,00	0,00	100,00	100,00
0,00	0,00	0,00	100,00	95,00
0,00	0,00	0,00	0,00	0,00
0,00	0,00	2,00	100,00	100,00
0,00	0,00	0,00	100,00	58,82
0,00	0,00	0,00	100,00	93,94
0,00	0,00	0,00	100,00	100,00
0,00	0,00	0,00	100,00	100,00
0,00	0,00	0,00	100,00	100,00
0,00	0,00	0,00	100,00	98,03
0,00	0,00	0,00	100,00	98,87
0,00	0,00	0,00	100,00	100,00
0,00	0,00	0,00	100,00	100,00
0,00	0,00	0,00	100,00	100,00
0,00	0,00	0,00	100,00	100,00
0,00	0,00	0,00	100,00	100,00
0,00	0,00	0,00	100,00	94,32
0,00	0,00	0,00	100,00	86,84
0,00	0,00	0,00	100,00	100,00
0,00	0,00	0,00	100,00	100,00

0,00	0,00	0,00	0,00	0,00
0,00	0,00	0,00	100,00	100,00
0,00	0,00	0,49	99,51	92,65
0,00	0,00	0,00	100,00	96,17
0,00	0,00	0,00	100,00	100,00
0,39	0,00	0,00	99,61	99,61
0,00	0,00	0,38	99,62	97,33
0,00	0,00	0,00	99,59	99,17
0,00	0,00	0,00	100,00	100,00
0,00	0,00	0,00	100,00	100,00
0,00	0,00	0,00	100,00	100,00
0,00	0,00	0,00	100,00	82,43
0,00	0,00	0,00	100,00	97,14
0,00	0,00	0,00	100,00	97,37
0,00	0,00	0,00	100,00	99,66
0,00	0,00	0,00	100,00	17,14
0,00	0,00	0,00	100,00	100,00
0,00	0,00	0,00	100,00	86,13
0,00	0,00	0,00	100,00	85,64
0,00	0,00	0,00	100,00	98,29
0,00	0,00	0,00	100,00	100,00
0,00	0,00	0,00	100,00	99,65
0,00	0,00	0,00	100,00	100,00
0,00	0,00	0,00	100,00	100,00
0,82	0,00	0,27	100,00	99,73
0,00	0,00	0,24	100,00	100,00
0,00	0,00	0,00	100,00	100,00
0,00	0,00	0,00	100,00	100,00
0,00	0,00	0,00	100,00	98,85
0,00	0,00	0,33	100,00	98,37
0,00	0,00	0,83	100,00	92,50
0,00	0,00	0,00	100,00	91,90
0,00	0,00	0,00	100,00	99,21
0,40	0,00	0,00	100,00	100,00
0,00	0,00	0,00	100,00	100,00
0,00	0,00	0,00	100,00	99,49
0,43	0,00	0,00	100,00	99,13
0,00	0,00	0,00	100,00	100,00
0,00	0,00	0,00	100,00	100,00
0,00	0,00	2,27	97,73	97,73
0,00	0,00	0,00	100,00	100,00
0,00	0,00	0,00	100,00	100,00
0,00	0,00	0,00	100,00	100,00
0,00	0,00	0,30	100,00	100,00
0,00	0,00	0,00	100,00	100,00
0,00	0,00	0,00	100,00	98,71
0,00	0,00	0,00	99,44	99,44
0,00	0,00	0,00	100,00	97,51
0,00	0,00	0,00	100,00	100,00
0,00	0,00	0,00	100,00	99,48

0,00	0,00	0,00	100,00	100,00
0,00	0,00	0,00	100,00	100,00
0,00	0,00	0,00	100,00	93,30
0,00	0,00	0,60	100,00	99,40
0,00	0,00	0,00	100,00	99,19
0,00	0,00	0,00	100,00	100,00
0,00	0,00	0,00	100,00	100,00
0,00	0,00	0,00	100,00	99,80
0,00	0,00	0,00	100,00	97,65
0,00	0,00	0,48	100,00	99,52
0,00	0,00	0,00	99,70	95,24
0,00	0,00	0,00	100,00	100,00
0,00	0,00	0,00	100,00	100,00
0,00	0,00	0,00	100,00	94,69
0,00	0,00	1,09	100,00	84,24
0,00	0,00	0,59	100,00	95,27
0,00	0,00	0,00	100,00	95,71
0,00	0,00	0,00	100,00	99,55
0,32	0,00	0,00	100,00	99,36
0,00	0,00	0,00	100,00	100,00
0,00	0,00	0,00	100,00	100,00
0,00	0,00	0,33	100,00	100,00
0,00	0,00	0,00	100,00	99,70
0,40	0,00	0,00	100,00	98,79
0,00	0,00	0,00	100,00	95,79
0,00	0,00	0,00	100,00	100,00
0,00	0,00	0,00	100,00	100,00
0,00	0,00	0,00	100,00	100,00
0,00	0,00	0,00	100,00	100,00
0,00	0,00	0,00	100,00	100,00
0,00	0,00	0,00	100,00	98,83
0,00	0,00	0,00	100,00	100,00
0,00	0,00	0,00	100,00	100,00
0,00	0,00	0,00	100,00	98,46
0,00	0,00	0,00	100,00	99,37
0,00	0,00	0,00	100,00	99,49
0,00	0,00	0,00	100,00	99,46
0,00	0,00	0,00	100,00	100,00
0,00	0,00	0,00	100,00	91,30
0,00	0,00	0,00	100,00	97,06
0,00	0,00	0,00	100,00	100,00
0,27	0,00	0,00	100,00	98,92
0,00	0,00	0,50	100,00	99,50
0,00	0,00	0,00	100,00	100,00
0,72	0,00	0,00	100,00	100,00
0,00	0,00	0,00	100,00	100,00
0,00	0,00	0,00	100,00	96,68
0,00	0,00	0,00	100,00	100,00
0,28	0,00	0,00	100,00	100,00
1,10	0,00	0,00	100,00	98,90

0,00	0,00	0,00	100,00	99,30
0,00	0,00	0,00	100,00	77,59
0,00	0,00	0,00	100,00	76,96
0,00	0,00	0,00	100,00	99,68
0,00	0,00	0,00	100,00	100,00
0,00	0,00	0,00	100,00	99,51
0,00	0,00	0,43	100,00	90,00
0,00	0,00	0,00	100,00	97,13
0,00	0,00	0,00	100,00	97,89
0,93	0,00	0,00	100,00	99,07
1,18	0,00	0,00	100,00	100,00
0,00	0,00	0,00	100,00	100,00
0,00	0,00	0,00	100,00	100,00
0,00	0,00	0,00	99,69	98,12
0,00	0,00	0,00	100,00	98,59
0,00	0,00	0,00	100,00	100,00
0,00	0,00	0,00	100,00	100,00
0,00	0,00	0,00	100,00	100,00
0,00	0,00	0,00	100,00	100,00
0,00	0,00	0,00	100,00	100,00
0,00	0,00	0,00	100,00	100,00
0,00	0,00	0,00	100,00	100,00
0,00	0,00	0,00	100,00	100,00
0,00	0,00	0,43	99,14	99,14
0,00	0,00	0,00	100,00	100,00
0,00	0,00	0,00	100,00	96,57
0,00	0,00	0,00	99,35	99,35
0,00	0,00	0,00	100,00	98,63
0,00	0,00	0,00	100,00	100,00
0,00	0,00	0,00	100,00	99,30
0,00	0,00	0,00	100,00	99,64
0,00	0,00	0,00	100,00	100,00
0,00	0,00	0,00	99,02	97,06
0,00	0,00	0,00	100,00	97,17
0,00	0,00	0,00	100,00	94,41
0,00	0,00	0,00	100,00	98,18
0,00	0,00	0,00	100,00	100,00
0,00	0,00	0,00	100,00	100,00
0,00	0,00	0,00	98,04	97,06
0,63	0,31	0,00	100,00	97,19
0,00	0,00	0,00	99,24	98,48
0,00	0,00	0,00	100,00	99,47
0,00	0,28	0,00	100,00	99,72
0,00	0,00	0,00	100,00	100,00
0,00	0,00	0,00	100,00	98,28
0,00	0,00	0,00	100,00	100,00
0,00	0,00	0,00	100,00	100,00
0,00	0,00	0,00	100,00	100,00
0,00	0,54	0,00	99,46	85,87
0,00	0,00	0,00	100,00	99,61

0,00	0,00	0,00	100,00	96,62
0,50	0,00	1,01	100,00	100,00
0,00	0,00	0,00	100,00	99,54
0,00	0,00	0,00	100,00	100,00
0,00	0,00	0,00	100,00	100,00
0,00	0,00	0,00	100,00	99,32
0,00	0,00	0,00	100,00	99,17
0,00	0,00	0,00	100,00	100,00
0,00	0,00	0,00	100,00	100,00
0,00	0,00	0,00	100,00	98,72
0,44	0,00	0,00	100,00	100,00
0,00	0,00	0,00	100,00	93,33
0,00	0,00	1,67	99,17	97,92
0,00	0,00	0,00	100,00	100,00
0,00	0,00	0,42	100,00	100,00
0,00	0,00	1,80	100,00	90,99
2,02	0,00	0,00	100,00	89,39
0,00	0,00	0,00	100,00	100,00
0,00	0,00	0,00	100,00	100,00
0,00	0,00	0,00	0,00	0,00
0,00	0,00	0,00	100,00	98,76
0,00	0,00	0,00	100,00	99,83
0,00	0,00	0,00	100,00	100,00
0,00	0,00	0,00	100,00	100,00
0,00	0,00	0,00	100,00	100,00
0,00	0,00	0,00	100,00	99,11
0,00	0,00	0,00	100,00	99,81
0,00	0,00	0,00	100,00	100,00
0,00	0,00	0,00	100,00	100,00
0,00	0,00	0,00	100,00	95,76
0,00	0,00	0,00	100,00	100,00
0,00	0,00	0,00	100,00	99,29
0,00	0,00	0,00	100,00	100,00
0,00	0,00	0,00	100,00	100,00
0,00	0,00	0,00	100,00	100,00
0,00	0,00	0,00	100,00	100,00
6,31	0,00	5,34	100,00	99,51
0,00	0,00	0,00	0,00	0,00
0,00	0,00	0,00	100,00	99,67
0,00	0,00	0,00	100,00	98,21
0,00	0,00	0,00	99,70	89,36
0,00	0,00	0,00	100,00	100,00
0,00	0,00	0,00	100,00	100,00
0,00	0,00	0,00	100,00	100,00
0,00	0,00	0,00	100,00	81,20
0,00	0,00	0,00	100,00	90,55
0,37	0,00	0,00	100,00	99,26
0,00	0,00	0,00	100,00	100,00
0,34	0,00	0,00	100,00	99,66
0,00	0,00	0,00	100,00	100,00

0,33	0,00	0,00	100,00	99,67
0,00	0,00	0,00	100,00	99,65
0,00	0,00	0,00	100,00	99,14
0,00	0,00	0,00	100,00	100,00
0,00	0,00	0,35	100,00	100,00
0,00	0,00	0,00	100,00	100,00
0,00	0,00	0,00	100,00	99,76
0,00	0,00	0,00	100,00	100,00
0,00	0,00	0,00	100,00	100,00
0,00	0,00	0,00	100,00	100,00
0,00	0,00	0,00	99,46	99,46
0,00	0,00	0,00	100,00	97,89
0,00	0,00	0,00	100,00	100,00
0,00	0,00	0,00	100,00	99,64
0,00	0,00	0,00	100,00	100,00
0,65	0,00	1,30	100,00	69,48
0,00	0,00	0,00	100,00	34,78
0,00	0,00	0,00	100,00	100,00
0,00	0,00	0,00	100,00	90,48
0,00	0,00	0,00	100,00	100,00
0,00	0,00	0,00	100,00	100,00
0,00	0,00	0,00	100,00	100,00
0,00	0,00	0,00	100,00	100,00
0,00	0,00	0,00	100,00	88,47
0,00	0,00	0,00	100,00	100,00
0,00	0,00	0,00	100,00	100,00
0,00	0,00	0,00	100,00	99,73
0,00	0,00	0,00	100,00	100,00
0,00	0,00	0,00	100,00	100,00
0,00	0,00	0,00	100,00	100,00
0,00	0,00	0,00	100,00	100,00
0,00	0,00	0,00	100,00	100,00
0,00	0,00	0,00	100,00	100,00
0,00	0,00	0,00	100,00	100,00
0,00	0,00	0,00	99,07	99,07
0,00	0,00	0,00	100,00	100,00
0,00	0,00	0,00	100,00	100,00
0,97	0,00	0,00	100,00	100,00
0,00	0,00	0,00	100,00	100,00
0,00	0,00	0,00	100,00	100,00
0,00	0,00	0,00	100,00	100,00
0,00	0,00	0,00	100,00	99,70
0,00	0,00	0,00	100,00	99,44
0,00	0,00	0,00	100,00	100,00
0,00	0,00	0,00	100,00	100,00
0,00	0,00	0,00	100,00	99,27
0,00	0,00	0,00	100,00	99,61
0,28	0,00	0,00	100,00	99,72
0,00	0,00	0,00	100,00	99,59
0,00	0,00	0,00	100,00	100,00

0,00	0,00	0,00	100,00	100,00
0,00	0,00	0,00	100,00	99,61
0,00	0,00	0,00	100,00	100,00
0,00	0,00	0,00	100,00	99,69
0,00	0,00	0,00	100,00	100,00
0,00	0,00	0,00	100,00	98,77
0,00	0,00	0,00	99,44	99,44
0,00	0,00	0,00	100,00	100,00
0,00	0,00	0,00	100,00	100,00
0,00	0,00	0,00	100,00	100,00
0,00	0,00	2,00	96,00	95,00
0,00	0,00	0,00	100,00	100,00
0,00	0,00	0,00	100,00	99,58
0,00	0,00	0,00	100,00	100,00
0,00	0,00	0,00	100,00	99,17
0,00	0,00	0,00	100,00	100,00
0,00	0,00	0,00	100,00	100,00
0,00	0,00	0,00	100,00	100,00
0,00	0,00	0,00	100,00	100,00
0,00	0,00	0,00	100,00	100,00
0,00	0,00	0,00	100,00	100,00
0,00	0,00	0,00	100,00	100,00
0,00	0,00	0,00	100,00	100,00
0,00	0,00	0,00	100,00	100,00
0,47	0,00	0,00	100,00	97,21
0,00	0,00	0,00	100,00	98,09
0,00	0,00	0,00	100,00	100,00
0,00	0,00	0,00	100,00	100,00
0,00	0,00	0,00	100,00	99,66
0,00	0,00	0,00	100,00	100,00
0,00	0,00	0,00	100,00	100,00
0,00	0,00	0,00	100,00	100,00
0,00	0,00	0,00	100,00	100,00
0,00	0,00	0,00	100,00	100,00
0,00	0,00	0,00	100,00	98,89
0,00	0,00	0,00	100,00	100,00
0,00	0,00	0,00	100,00	100,00
0,00	0,00	0,00	100,00	96,40
0,00	0,00	0,00	100,00	100,00
0,00	0,00	0,00	99,21	97,62
0,00	0,00	0,00	100,00	100,00
0,00	0,00	0,00	100,00	98,73
0,00	0,00	0,00	100,00	100,00
0,00	0,00	0,00	100,00	100,00
0,00	0,00	0,00	100,00	100,00
0,00	0,00	0,00	100,00	99,35
0,00	0,00	0,00	100,00	100,00
0,00	0,00	0,00	100,00	100,00
0,00	0,00	0,00	100,00	100,00
0,00	0,00	0,00	100,00	100,00

0,00	0,00	0,41	99,18	99,18
0,00	0,00	0,00	100,00	100,00
0,00	0,00	0,00	100,00	100,00
0,00	0,00	0,00	100,00	100,00
0,00	0,00	0,00	100,00	100,00
0,00	0,00	0,00	100,00	100,00
0,00	0,00	0,00	100,00	100,00
0,00	0,00	0,00	100,00	100,00
2,81	0,00	0,00	100,00	99,72
0,00	0,00	0,00	100,00	100,00
0,00	0,00	0,00	100,00	100,00
0,00	0,00	0,00	100,00	100,00
0,00	0,00	0,00	100,00	100,00
0,00	0,00	1,38	100,00	96,77
0,00	0,00	0,00	100,00	100,00
0,00	0,00	0,64	100,00	100,00
0,00	0,00	0,00	100,00	100,00
0,00	0,00	0,00	100,00	99,02
0,00	0,00	0,00	100,00	100,00
0,00	0,00	0,00	100,00	97,59
0,00	0,00	0,00	100,00	65,91
0,00	0,00	0,00	100,00	100,00
0,00	0,00	0,00	100,00	15,38
0,00	0,00	1,09	100,00	65,22
0,00	0,00	0,00	100,00	99,00
0,00	0,00	0,00	100,00	95,45
0,00	0,00	0,00	0,00	0,00
0,34	0,00	0,00	100,00	100,00
0,00	0,00	0,00	100,00	81,61
0,00	0,00	0,00	100,00	88,05
0,00	0,00	0,31	100,00	84,21
0,00	0,00	1,25	100,00	96,25
0,00	0,00	0,00	100,00	100,00
0,00	0,00	0,00	100,00	100,00
0,00	0,00	0,00	100,00	98,61
0,00	0,00	0,00	100,00	100,00
0,00	0,00	0,00	100,00	59,26
0,00	0,00	0,00	100,00	100,00
0,00	0,00	0,00	100,00	100,00
0,00	0,00	0,00	100,00	98,06
0,00	0,00	0,00	100,00	100,00
0,00	0,00	0,00	100,00	100,00
0,00	0,00	0,00	100,00	100,00
0,00	0,00	0,00	100,00	100,00
0,00	0,00	1,33	100,00	68,00
0,00	0,00	0,00	100,00	100,00
0,00	0,00	0,00	100,00	100,00
0,00	0,00	0,00	100,00	100,00
0,48	0,00	0,00	100,00	99,05
0,00	0,00	0,00	100,00	100,00

0,00	0,00	0,00	100,00	99,07
0,00	0,00	0,00	100,00	96,00
0,00	0,00	0,00	100,00	100,00
0,00	0,00	0,00	100,00	100,00
0,00	0,00	0,00	100,00	100,00
0,00	0,00	0,00	100,00	100,00
0,00	0,00	0,00	100,00	100,00
0,00	0,00	0,68	98,63	98,63
0,00	0,00	0,00	100,00	100,00
0,00	0,00	0,00	100,00	100,00
0,00	0,00	0,00	100,00	68,67
0,00	0,00	0,00	100,00	92,34
0,00	0,00	0,00	100,00	80,00
3,70	0,00	0,00	100,00	62,96
0,74	0,00	0,74	100,00	100,00
0,00	0,00	0,00	100,00	96,59
4,91	0,00	0,00	100,00	100,00
2,04	0,00	0,00	100,00	100,00
0,00	0,00	0,00	100,00	98,67
0,00	0,00	0,00	100,00	99,58
0,00	0,00	0,00	100,00	100,00
0,00	0,00	0,00	100,00	100,00
0,00	0,00	0,00	100,00	100,00
0,00	0,00	0,00	100,00	100,00
0,00	0,00	0,00	0,00	0,00
0,00	0,00	0,00	100,00	96,97
0,00	0,00	0,00	100,00	94,96
0,00	0,00	0,00	100,00	100,00
0,00	0,00	0,00	100,00	100,00
2,44	0,00	0,00	100,00	100,00
0,00	0,00	0,00	100,00	82,20
0,00	0,00	0,00	100,00	100,00
0,00	0,00	0,00	100,00	100,00
0,00	0,00	0,00	100,00	99,58
0,00	0,00	0,00	100,00	100,00
0,00	0,00	0,00	100,00	100,00
0,00	0,00	0,00	100,00	99,11
0,00	0,00	0,00	100,00	99,32
0,28	0,00	0,00	100,00	98,62
0,00	0,00	0,49	100,00	97,55
0,00	0,00	0,00	100,00	99,55
0,00	0,00	0,00	100,00	99,58
0,00	0,00	0,00	100,00	100,00
0,00	0,00	0,00	100,00	100,00
0,00	0,00	0,00	100,00	100,00
0,00	0,00	0,00	100,00	100,00
0,00	0,00	0,00	100,00	86,00
0,00	0,00	0,00	100,00	95,31
0,00	0,00	1,06	100,00	90,43
0,00	0,00	0,00	100,00	100,00

0,50	0,00	0,00	100,00	98,50
0,00	0,00	0,00	0,00	0,00
0,00	0,00	0,00	100,00	100,00
0,00	0,00	0,00	0,00	0,00
0,00	0,00	0,00	100,00	100,00
0,00	0,00	0,00	100,00	100,00
0,00	0,00	0,00	100,00	100,00
0,00	0,00	0,00	100,00	100,00
0,00	0,00	0,00	100,00	99,22
0,00	0,00	0,00	100,00	100,00
0,00	0,00	0,00	100,00	96,95
0,00	0,00	0,00	100,00	100,00
0,00	0,00	0,00	100,00	100,00
0,00	0,00	0,00	100,00	100,00
0,00	0,00	0,00	100,00	100,00
0,00	0,00	0,00	100,00	100,00
0,00	0,00	0,00	100,00	99,56
0,00	0,00	0,00	0,00	0,00
0,00	0,00	0,00	100,00	100,00
0,00	0,00	0,00	100,00	100,00
0,00	0,00	0,00	100,00	100,00
0,00	0,00	0,00	100,00	100,00
0,00	0,00	0,00	100,00	100,00
0,00	0,00	0,00	100,00	96,67
0,00	0,00	0,00	100,00	97,96
0,00	0,00	0,00	100,00	99,07
0,00	0,00	0,00	100,00	100,00
0,00	0,00	0,00	100,00	100,00
0,00	0,00	0,00	100,00	100,00
0,00	0,00	0,00	100,00	100,00
0,00	0,00	0,00	100,00	98,27
12,12	0,00	0,00	100,00	100,00
0,00	0,00	0,00	0,00	0,00
0,00	0,00	0,00	0,00	0,00
0,00	0,00	0,00	100,00	100,00
0,00	0,00	0,00	100,00	98,15
0,00	0,00	0,00	0,00	0,00
0,42	0,00	0,00	100,00	95,82
0,00	0,00	0,00	100,00	40,00
0,00	0,00	0,00	100,00	100,00
0,00	0,00	0,00	100,00	99,81
0,00	0,00	0,00	100,00	82,98
0,00	0,00	0,00	0,00	0,00
0,00	0,00	0,00	0,00	0,00
0,00	0,00	0,00	100,00	100,00
0,00	0,00	0,00	100,00	98,57
0,00	0,00	0,00	100,00	99,48
0,00	0,00	0,00	100,00	99,31
0,00	0,00	0,00	100,00	100,00
0,00	0,00	0,00	100,00	87,32

0,00	0,00	0,00	100,00	100,00
0,00	0,00	0,00	100,00	100,00
0,00	0,00	0,00	100,00	100,00
0,00	0,00	0,00	99,61	99,22
0,00	0,00	0,00	100,00	100,00
0,00	0,00	0,00	100,00	100,00
0,00	0,00	0,00	100,00	100,00
0,00	0,00	0,00	100,00	100,00
0,00	0,00	0,00	100,00	100,00
1,56	0,00	0,00	100,00	90,63
0,00	0,00	0,00	0,00	0,00
0,00	0,00	0,00	0,00	0,00
0,00	0,00	0,00	100,00	100,00
0,00	0,00	0,00	0,00	0,00
0,00	0,00	0,00	100,00	100,00
0,00	0,00	0,00	100,00	100,00
0,00	0,00	0,00	100,00	95,56
0,00	0,00	0,00	100,00	100,00
0,00	0,00	0,00	100,00	100,00
0,00	0,00	0,00	100,00	100,00
0,00	0,00	0,00	100,00	100,00
0,00	0,00	0,00	100,00	95,83
0,00	0,00	0,00	96,97	96,97
0,00	0,00	0,00	100,00	100,00
0,00	0,00	0,00	0,00	0,00
0,00	0,00	0,00	100,00	100,00
0,00	0,00	0,00	100,00	100,00
0,00	0,00	0,00	100,00	98,53
1,78	0,00	0,00	100,00	99,56
0,00	0,00	0,00	100,00	100,00
0,00	0,00	0,00	100,00	99,65
0,00	0,00	0,00	100,00	100,00
0,00	0,00	0,00	100,00	100,00
0,00	0,00	0,00	100,00	97,44
0,00	0,00	0,00	100,00	100,00
0,75	0,00	0,00	100,00	96,27
1,85	0,00	0,00	100,00	98,15
0,00	0,00	0,00	100,00	100,00
0,00	0,00	0,00	0,00	0,00
0,00	0,00	0,00	100,00	97,94
0,76	0,00	0,00	100,00	97,73
0,00	0,00	0,79	98,41	94,44
0,00	0,00	0,00	99,75	93,95
0,00	0,00	0,00	100,00	100,00
0,00	0,00	0,00	0,00	0,00
0,00	0,00	0,00	100,00	100,00
0,00	0,00	0,00	100,00	93,83
0,00	0,00	0,00	100,00	100,00
0,00	0,00	0,00	100,00	99,69
0,00	0,00	0,82	100,00	98,77

0,00	0,00	0,00	100,00	100,00
0,00	0,00	0,41	100,00	100,00
0,00	0,00	0,00	100,00	100,00
0,00	0,00	0,00	100,00	64,12
0,00	0,00	0,00	100,00	91,40
0,00	0,00	0,00	100,00	100,00
0,00	0,00	0,00	100,00	99,57
0,60	0,00	0,00	100,00	100,00
0,00	0,00	0,00	100,00	100,00
0,00	0,00	0,00	100,00	98,34
0,00	0,00	0,00	100,00	100,00
0,00	0,00	0,00	100,00	100,00
0,00	0,00	0,00	100,00	98,61
0,47	0,00	0,47	100,00	97,67
0,00	0,00	0,00	100,00	98,19
0,00	0,00	0,00	100,00	96,22
0,00	0,00	0,00	100,00	100,00
0,00	0,00	0,00	100,00	93,69
0,00	0,00	0,00	100,00	99,66
0,00	0,00	0,00	100,00	98,21
0,57	0,00	0,57	100,00	92,00
0,00	0,00	0,00	100,00	100,00
0,45	0,00	0,00	100,00	98,65
0,00	0,00	0,00	100,00	93,96
0,63	0,00	0,00	100,00	97,47
0,67	0,00	0,00	100,00	97,99
0,00	0,00	0,00	100,00	92,55
0,00	0,00	0,00	0,00	0,00
0,00	0,00	0,00	100,00	100,00
0,00	0,00	0,00	100,00	100,00
0,00	0,00	0,00	100,00	100,00
0,42	0,00	0,00	100,00	98,74
0,00	0,00	0,00	100,00	98,65
0,00	0,00	0,00	100,00	100,00
0,00	0,00	0,00	100,00	100,00
0,35	0,00	0,00	100,00	100,00
0,00	0,00	0,00	100,00	100,00
0,00	0,00	0,00	100,00	98,56
0,00	0,00	0,00	100,00	99,28
0,35	0,00	0,00	100,00	100,00
0,00	0,00	0,00	100,00	95,90
0,45	0,00	0,00	100,00	95,09
0,40	0,00	0,00	100,00	100,00
0,00	0,00	0,00	100,00	99,63
0,00	0,00	0,00	100,00	100,00
0,00	0,00	0,00	100,00	100,00
0,00	0,00	0,00	100,00	100,00
0,00	0,00	0,00	100,00	100,00
0,00	0,00	0,00	99,40	99,40
1,44	0,00	0,00	100,00	100,00

0,00	0,00	0,00	100,00	100,00
0,00	0,00	0,00	100,00	100,00
0,00	0,00	0,00	99,48	97,91
0,32	0,32	0,00	100,00	76,92
0,00	0,00	0,00	100,00	100,00
0,00	0,00	0,00	100,00	100,00
0,00	0,00	0,00	0,00	0,00
1,66	0,00	0,00	100,00	85,89
0,00	0,00	0,43	99,57	55,84
0,56	0,00	21,35	99,81	38,58
0,00	0,00	0,53	100,00	67,02
0,58	0,00	0,58	100,00	57,89
0,00	0,00	0,00	100,00	48,81
0,99	0,00	0,00	100,00	74,38
0,00	0,00	0,00	100,00	3,80
0,83	0,00	0,00	98,33	9,17
0,34	0,00	0,00	100,00	97,64
0,00	0,00	0,00	100,00	100,00
0,00	0,00	0,92	99,54	82,49
0,00	0,00	0,00	100,00	98,21
0,00	0,00	0,00	100,00	100,00
0,00	0,00	0,00	100,00	100,00
0,00	0,00	0,00	100,00	100,00
0,00	0,00	0,00	100,00	100,00
0,00	0,00	0,00	100,00	100,00
0,00	0,00	0,00	99,63	99,26
0,00	0,00	0,00	100,00	100,00
0,00	0,00	0,00	100,00	100,00
0,00	0,00	0,00	100,00	99,55
0,00	0,00	0,00	100,00	88,46
0,00	0,00	0,00	100,00	100,00
0,40	0,00	0,00	100,00	87,30
0,00	0,00	0,00	0,00	0,00
0,00	0,00	0,33	100,00	98,37
0,00	0,00	0,00	100,00	100,00
0,00	0,00	0,00	100,00	100,00
0,00	0,00	0,00	100,00	96,23
0,00	0,00	0,00	100,00	100,00
0,00	0,00	0,00	100,00	100,00
0,00	0,00	0,00	100,00	97,80
0,00	0,00	0,00	100,00	94,59
0,00	0,00	0,00	100,00	99,48
0,00	0,00	6,90	100,00	96,55
0,00	0,00	0,00	100,00	98,68
0,55	0,00	0,00	100,00	88,95
0,37	0,00	0,00	100,00	85,87
0,00	0,00	0,00	99,70	99,70
0,00	0,00	0,00	100,00	100,00
0,00	0,00	0,00	100,00	95,65
0,00	0,00	0,00	100,00	100,00

0,00	0,00	0,00	100,00	99,52
0,00	0,00	15,79	94,74	10,53
0,00	0,00	0,40	100,00	92,80
2,17	0,00	0,00	99,28	92,75
0,00	0,00	0,00	100,00	99,57
0,00	0,00	0,00	98,73	72,15
0,00	0,00	0,00	100,00	99,05
0,00	0,00	0,00	100,00	100,00
0,00	0,00	0,00	100,00	97,20
0,00	0,00	0,00	100,00	100,00
0,00	0,00	0,00	100,00	100,00
0,00	0,00	0,00	100,00	100,00
0,00	0,00	0,00	100,00	100,00
0,56	0,00	0,00	100,00	99,44
0,00	0,00	0,65	99,35	97,40
0,00	0,00	0,00	100,00	100,00
0,00	0,00	0,00	100,00	98,83
0,00	0,00	0,00	100,00	99,25
0,00	0,00	0,00	100,00	98,70
0,00	0,00	0,00	100,00	99,51
0,00	0,00	0,00	100,00	98,04
0,00	0,00	0,00	100,00	96,38
0,43	0,00	0,00	100,00	96,54
0,00	0,00	0,00	100,00	99,20
0,00	0,00	0,00	100,00	100,00
0,00	0,00	0,00	100,00	100,00
0,00	0,00	0,00	100,00	100,00
0,00	0,00	0,00	100,00	100,00
0,28	0,00	0,00	100,00	99,72
0,00	0,00	0,00	100,00	100,00
0,00	0,00	0,00	0,00	0,00
0,27	0,00	0,00	100,00	99,18
0,00	0,00	0,00	0,00	0,00
0,00	0,00	0,00	100,00	100,00
0,00	0,00	0,00	100,00	100,00
0,00	0,00	0,00	100,00	97,48
0,00	0,00	0,00	100,00	100,00
0,00	0,00	0,00	100,00	100,00
0,00	0,00	0,00	100,00	99,56
0,00	0,00	0,00	100,00	93,55
0,00	0,00	0,00	100,00	98,85
0,00	0,00	0,00	100,00	100,00
0,00	0,00	0,00	100,00	96,24
0,00	0,00	0,00	100,00	98,82
0,00	0,00	0,00	100,00	100,00
0,00	0,00	0,00	100,00	100,00
0,00	0,00	0,00	100,00	100,00
0,00	0,00	0,00	100,00	98,83
0,00	0,00	0,00	100,00	96,88
0,00	0,00	0,00	100,00	98,87

0,00	0,00	0,00	100,00	100,00
0,00	0,00	0,00	100,00	100,00
0,00	0,00	0,00	100,00	100,00
0,00	0,00	0,00	100,00	94,03
0,00	0,00	0,00	100,00	97,69
0,00	0,00	0,00	100,00	98,21
0,00	0,00	0,90	96,40	96,40
0,00	0,00	0,00	100,00	100,00
0,95	0,00	0,00	100,00	85,78
0,00	0,00	0,00	100,00	100,00
0,00	0,00	0,00	0,00	0,00
0,00	0,00	0,00	100,00	99,07
0,00	0,00	0,00	100,00	94,74
0,00	0,00	0,00	100,00	71,43
0,00	0,00	0,00	98,80	96,39
0,00	0,00	0,00	100,00	92,59
0,00	0,00	0,00	100,00	100,00
1,22	0,00	0,00	100,00	100,00
0,00	0,00	0,00	100,00	100,00
0,00	0,00	0,00	100,00	100,00
0,00	0,00	0,00	100,00	100,00
0,00	0,00	0,00	100,00	100,00
0,00	0,00	0,00	100,00	100,00
0,00	0,00	0,00	100,00	99,40
0,00	0,00	0,00	100,00	100,00
0,00	0,00	0,00	100,00	100,00
0,00	0,00	0,00	100,00	100,00
0,00	0,00	0,00	100,00	100,00
0,00	0,00	0,00	100,00	99,34
0,00	0,00	1,65	100,00	97,80
0,00	0,00	1,18	100,00	98,82
0,00	0,00	0,00	100,00	100,00
0,00	0,00	0,00	100,00	99,38
0,00	0,00	0,00	99,47	99,47
0,00	0,00	0,00	100,00	100,00
0,00	0,00	0,00	100,00	99,62
0,00	0,00	0,00	99,46	98,92
0,00	0,00	0,00	100,00	100,00
0,00	0,00	0,00	100,00	100,00
0,00	0,00	0,00	100,00	99,57
0,00	0,00	0,00	100,00	100,00
0,00	0,00	0,00	100,00	33,33
0,00	0,00	0,00	100,00	98,55
15,72	0,00	1,89	99,37	7,55
0,00	0,00	0,00	0,00	0,00
0,00	0,00	0,00	100,00	63,16
0,00	0,00	0,00	100,00	91,14
0,00	0,00	0,00	0,00	0,00
0,00	0,00	0,83	99,17	80,83
1,07	0,00	0,53	100,00	15,51

0,00	0,00	0,00	0,00	0,00
1,92	0,00	0,00	100,00	71,15
2,17	0,00	0,00	100,00	10,87
0,00	0,00	0,00	100,00	100,00
0,00	0,00	0,00	100,00	97,24
0,00	0,00	0,00	100,00	100,00
0,00	0,00	0,00	100,00	86,09
0,00	0,00	0,00	100,00	100,00
0,00	0,00	0,00	100,00	100,00
0,00	0,00	0,00	100,00	98,90
0,00	0,00	0,00	100,00	96,76
0,00	0,00	0,00	100,00	100,00
0,00	0,00	0,00	100,00	100,00
0,00	0,00	0,00	100,00	96,67
0,00	0,00	0,00	100,00	98,75
0,00	0,00	0,00	100,00	98,33
0,00	0,00	0,00	100,00	98,48
0,00	0,00	0,00	100,00	93,33
0,00	0,00	0,00	100,00	99,38
0,00	0,00	0,00	100,00	92,86
0,00	0,00	0,00	100,00	100,00
0,00	0,00	0,00	100,00	100,00
0,00	0,00	0,00	100,00	99,38
0,00	0,00	0,00	0,00	0,00
0,00	0,00	0,00	100,00	100,00
0,00	0,00	0,00	100,00	100,00
0,00	0,00	0,00	100,00	96,97
0,00	0,00	0,00	100,00	83,93
0,00	0,00	0,00	0,00	0,00
0,00	0,00	0,00	0,00	0,00
0,00	0,00	0,00	100,00	99,03
0,00	0,00	0,00	0,00	0,00
0,00	0,00	0,00	100,00	100,00
0,00	0,00	0,00	99,69	89,06
0,56	0,00	0,00	100,00	81,07
0,00	0,00	0,00	100,00	97,21
0,20	0,00	1,41	99,60	92,32
0,00	0,00	0,00	100,00	99,60
0,00	0,00	0,00	100,00	98,71
0,00	0,00	0,00	100,00	99,65
0,00	0,00	0,71	99,29	99,29
0,00	0,00	0,00	100,00	98,96
0,54	0,00	0,00	100,00	99,46
0,00	0,00	0,00	100,00	99,60
0,00	0,00	0,00	100,00	100,00
0,00	0,00	0,00	100,00	100,00
0,00	0,00	0,00	100,00	100,00
0,00	0,00	0,00	100,00	100,00
0,00	0,00	0,00	100,00	99,44
0,00	0,00	0,00	100,00	100,00

0,00	0,00	0,00	100,00	100,00
0,00	0,00	0,00	100,00	100,00
0,00	0,00	0,00	100,00	67,50
0,00	0,00	0,00	100,00	100,00
0,00	0,00	0,00	100,00	100,00
0,00	0,00	0,00	100,00	100,00
0,00	0,00	0,00	100,00	100,00
0,00	0,00	0,00	100,00	100,00
0,00	0,00	0,74	100,00	92,62
0,71	0,00	0,00	100,00	99,65
0,31	0,31	0,31	100,00	99,69
0,00	0,00	1,29	99,35	59,05
0,00	0,00	0,00	100,00	98,43
0,00	0,00	0,00	100,00	98,64
0,00	0,00	0,00	100,00	100,00
0,00	0,00	0,00	100,00	100,00
0,00	0,00	0,00	100,00	99,45
0,00	0,00	0,00	100,00	94,44
0,00	0,00	0,00	100,00	100,00
0,53	0,00	0,00	100,00	97,89
0,00	1,14	1,14	100,00	19,32
0,00	0,00	0,00	100,00	90,71
0,00	0,00	0,00	100,00	99,43
0,00	0,00	0,00	100,00	98,89
0,00	0,00	0,00	100,00	98,81
0,00	0,00	0,00	100,00	100,00
0,00	0,00	0,00	100,00	100,00
0,00	0,71	0,00	100,00	97,86
0,00	0,00	0,00	100,00	100,00
0,00	0,00	0,00	100,00	100,00
0,00	0,00	0,00	100,00	88,84
0,00	0,00	0,00	0,00	0,00
0,00	0,00	0,00	100,00	98,21
0,00	0,00	0,00	100,00	99,65
0,00	0,00	0,00	100,00	100,00
0,00	0,00	0,00	100,00	98,94
0,00	0,00	0,00	100,00	98,94
0,00	0,00	0,00	100,00	100,00
0,00	0,00	0,00	100,00	100,00
0,00	0,00	0,00	100,00	100,00
0,35	0,00	0,00	100,00	99,82
0,00	0,00	0,46	100,00	78,24
0,00	0,00	0,00	100,00	98,06
1,18	0,00	0,00	100,00	95,29
1,41	0,00	0,00	100,00	98,59
0,00	0,00	0,00	99,60	99,60
0,00	0,00	0,00	100,00	100,00
0,00	0,00	0,00	100,00	94,81
0,00	0,00	0,00	100,00	100,00
0,00	0,00	0,00	100,00	100,00

0,00	0,00	0,00	100,00	99,68
0,30	0,00	0,00	100,00	99,70
0,00	0,00	0,00	100,00	100,00
0,00	0,00	0,00	100,00	100,00
0,00	0,00	0,00	100,00	98,31
0,00	0,00	0,00	100,00	100,00
0,00	0,00	0,00	100,00	98,06
0,00	0,00	0,00	100,00	90,42
0,00	0,00	0,00	100,00	99,33
0,00	0,00	0,00	100,00	99,63
0,00	0,00	0,00	100,00	99,56
0,00	0,00	0,00	100,00	99,64
0,00	0,00	0,00	100,00	100,00
0,35	0,00	0,00	100,00	95,76
0,00	0,00	0,00	0,00	0,00
0,00	0,00	0,00	100,00	99,22
0,00	0,00	0,00	100,00	100,00
0,00	0,00	0,00	100,00	98,92
0,00	0,00	0,00	0,00	0,00
0,00	0,00	0,00	100,00	99,27
0,00	0,00	0,00	100,00	100,00
0,00	0,00	0,00	100,00	99,50
0,00	0,00	0,00	100,00	100,00
0,00	0,00	0,00	100,00	100,00
0,00	0,00	0,00	100,00	100,00
0,68	0,00	0,00	100,00	100,00
0,00	0,00	0,00	100,00	100,00
0,00	0,00	0,00	100,00	100,00
0,00	0,00	0,00	100,00	100,00
0,00	0,00	0,00	100,00	98,88
0,00	0,00	0,00	100,00	98,90
0,00	0,00	0,00	100,00	98,85
0,00	0,00	0,00	100,00	100,00
0,00	0,00	0,00	100,00	100,00
0,00	0,00	0,35	100,00	95,05
0,45	0,00	0,00	100,00	77,63
0,00	0,00	0,00	99,70	99,39
0,32	0,00	0,00	100,00	99,68
0,00	0,00	0,00	100,00	98,49
1,79	0,00	0,00	100,00	98,21
0,00	0,00	0,38	99,81	31,70
0,99	0,00	0,00	100,00	86,14
0,00	0,00	0,00	100,00	98,00
0,00	0,00	0,00	100,00	95,09
0,00	0,00	0,00	100,00	100,00
0,00	0,00	0,00	100,00	11,76
0,63	0,00	0,00	100,00	74,68
0,00	0,00	0,00	100,00	86,50
0,00	0,00	0,00	100,00	84,92
0,00	0,00	0,00	100,00	99,51

0,00	0,00	0,00	100,00	100,00
0,00	0,00	0,00	100,00	100,00
0,00	0,00	0,00	100,00	100,00
0,00	0,00	0,00	100,00	99,00
0,35	0,00	0,00	100,00	86,67
0,00	0,00	0,00	100,00	94,46
0,00	0,00	0,00	100,00	97,26
0,00	0,00	0,00	100,00	100,00
0,00	0,00	0,37	99,26	99,26
0,00	0,00	0,00	100,00	100,00
0,00	0,00	0,00	100,00	100,00
0,00	0,00	0,00	100,00	100,00
0,00	0,00	0,00	100,00	100,00
0,00	0,00	0,00	100,00	100,00
0,00	0,00	0,00	100,00	100,00
0,00	0,00	0,00	100,00	99,36
0,00	0,00	0,00	100,00	99,52
0,00	0,00	0,00	0,00	0,00
0,00	0,00	0,00	100,00	100,00
0,40	0,00	0,40	100,00	100,00
0,00	0,00	0,00	100,00	100,00
0,00	0,00	0,00	100,00	100,00
0,00	0,00	0,00	100,00	100,00
0,00	0,00	0,00	100,00	100,00
0,00	0,00	0,00	100,00	100,00
0,00	0,00	0,00	100,00	90,91
0,00	0,00	1,14	100,00	100,00
0,00	0,00	0,00	100,00	99,42
0,25	0,00	0,00	100,00	99,75
0,00	0,00	0,33	100,00	98,66
0,00	0,00	0,49	100,00	100,00
0,00	0,00	0,00	100,00	99,63
0,41	0,00	0,41	100,00	95,51
0,32	0,00	0,00	99,68	99,35
0,00	0,00	0,00	100,00	100,00
0,00	0,00	0,00	100,00	87,27
1,18	0,00	0,00	100,00	97,25
0,00	0,65	0,00	100,00	99,35
0,30	0,00	0,00	100,00	98,81
0,39	0,00	0,00	100,00	99,61
0,00	0,00	0,00	100,00	99,69
0,00	0,00	0,00	100,00	100,00
0,51	0,00	0,77	100,00	65,22
0,00	0,00	0,00	100,00	98,16
0,00	0,00	0,00	100,00	98,40
0,00	0,00	0,35	100,00	97,54
0,00	0,00	0,00	100,00	100,00
0,00	0,00	0,00	99,61	99,22
0,00	0,00	0,00	100,00	99,31
0,00	0,00	0,00	100,00	99,17

0,00	0,00	0,00	100,00	100,00
0,00	0,00	0,00	100,00	59,29
0,00	0,00	0,00	100,00	98,28
0,00	0,00	0,00	0,00	0,00
0,00	0,00	0,00	100,00	99,08
0,00	0,00	0,00	100,00	100,00
0,00	0,00	0,00	100,00	98,06
0,35	0,00	0,00	100,00	99,30
0,51	0,00	0,00	100,00	99,49
0,00	0,00	0,00	100,00	99,40
0,00	0,00	0,00	100,00	100,00
0,00	0,00	0,00	100,00	100,00
0,00	0,00	0,00	100,00	99,55
0,00	0,00	0,00	100,00	100,00
0,00	0,00	0,00	100,00	100,00
0,00	0,00	1,36	100,00	95,92
1,51	0,00	0,50	100,00	91,46
0,00	0,00	0,00	100,00	98,36
0,00	0,00	0,90	100,00	94,28
0,29	0,00	0,00	100,00	93,00
0,00	0,00	0,00	100,00	100,00
0,00	0,00	0,00	100,00	14,42
0,00	0,00	5,06	100,00	54,43
0,00	0,00	0,00	100,00	73,78
0,00	0,00	0,00	100,00	100,00
0,00	0,00	0,00	100,00	99,46
0,00	0,00	0,00	100,00	100,00
0,00	0,00	0,00	100,00	100,00
0,00	0,00	0,00	100,00	100,00
0,00	0,00	0,00	100,00	99,70
0,00	0,00	0,00	99,40	99,40
0,00	0,00	0,00	100,00	100,00
0,00	0,00	0,00	100,00	100,00
0,00	0,00	0,00	100,00	100,00
0,00	0,00	0,00	100,00	99,29
0,00	0,00	0,00	100,00	91,88
0,00	0,00	0,00	100,00	99,65
0,00	0,00	0,00	100,00	100,00
0,00	0,00	0,00	100,00	98,61
0,00	0,00	0,00	100,00	62,87
0,00	0,00	0,00	100,00	95,74
0,00	0,00	0,00	100,00	99,24
0,00	0,00	0,00	100,00	100,00
0,00	0,00	0,00	99,61	97,30
0,98	0,00	0,00	100,00	96,73
0,00	0,00	0,00	100,00	100,00
0,74	0,00	0,37	100,00	95,54
0,00	0,00	0,00	100,00	99,11
0,00	0,00	0,00	100,00	96,07
0,00	0,00	0,00	100,00	81,00

0,00	0,00	0,00	100,00	99,65
0,00	0,00	0,00	100,00	100,00
0,00	0,00	0,00	100,00	99,36
0,00	0,00	0,00	100,00	98,51
0,00	0,00	0,00	100,00	100,00
1,06	0,35	0,00	100,00	100,00
0,56	0,00	0,56	100,00	100,00
0,00	0,00	0,00	100,00	100,00
0,00	0,00	0,00	100,00	100,00
0,00	0,00	0,00	99,33	99,33
0,00	0,00	0,00	99,50	99,50
0,00	0,00	0,00	100,00	100,00
0,00	0,00	0,00	0,00	0,00
48,28	3,45	3,45	100,00	0,00
0,00	0,00	0,00	100,00	94,81
0,74	0,00	0,00	98,52	91,85
0,00	0,00	0,41	100,00	86,83
0,00	0,00	0,00	100,00	97,28
0,00	0,00	1,08	100,00	61,29
0,00	0,00	0,49	100,00	68,14
0,00	0,00	2,14	100,00	32,86
0,00	0,00	0,00	100,00	100,00
0,00	0,00	0,00	99,07	99,07
0,00	0,00	0,00	100,00	100,00
0,00	0,00	0,00	100,00	99,10
0,00	0,00	0,00	100,00	100,00
0,40	0,00	0,00	100,00	100,00
0,00	0,00	0,00	100,00	100,00
1,33	0,00	0,00	100,00	98,67
0,00	0,00	0,00	100,00	100,00
0,00	0,00	0,00	100,00	100,00
0,00	0,00	0,00	100,00	99,70
0,00	0,28	0,00	100,00	99,15
0,00	0,00	0,00	100,00	100,00
0,00	0,00	0,00	99,67	99,67
0,00	0,00	0,00	100,00	100,00
0,00	0,00	0,00	100,00	100,00
0,00	0,00	0,00	100,00	100,00
0,00	0,00	0,00	100,00	58,33
0,00	0,00	0,00	100,00	99,35
0,00	0,00	0,00	100,00	100,00
0,00	0,00	0,00	0,00	0,00
0,00	0,00	0,00	100,00	100,00
0,00	0,00	0,00	100,00	100,00
0,00	0,00	0,00	100,00	100,00
1,37	0,00	1,02	100,00	95,56
0,00	0,00	0,00	100,00	0,00
0,00	0,00	0,00	100,00	12,50
0,00	0,00	0,00	100,00	100,00
0,00	0,00	0,00	100,00	23,08

0,00	0,00	0,00	100,00	94,08
0,00	0,00	0,00	100,00	62,63
0,00	0,00	0,00	100,00	93,95
0,00	0,00	0,00	100,00	96,97
0,33	0,00	0,00	100,00	97,33
0,00	0,00	0,44	100,00	99,12
0,00	0,00	0,00	100,00	100,00
0,00	0,00	0,00	100,00	100,00
0,00	0,00	0,00	100,00	100,00
0,00	0,00	0,00	100,00	100,00
0,00	0,00	0,00	100,00	100,00
0,00	0,00	0,00	100,00	100,00
0,00	0,00	0,00	100,00	100,00
0,00	0,00	0,00	100,00	100,00
1,29	0,00	0,00	100,00	97,42
0,65	0,00	0,65	99,35	98,06
0,00	0,00	0,00	100,00	100,00
0,00	0,00	0,83	100,00	100,00
0,00	0,00	0,00	100,00	100,00
0,00	0,00	0,00	100,00	98,66
0,00	0,00	0,00	100,00	100,00
0,00	0,00	0,00	100,00	100,00
0,00	0,00	0,00	100,00	100,00
0,00	0,00	0,00	100,00	99,62
0,00	0,00	0,00	100,00	81,34
0,00	0,00	0,00	100,00	90,64
0,00	0,00	0,00	100,00	55,56
0,00	0,00	0,00	100,00	100,00
0,00	0,00	0,00	100,00	100,00
0,00	0,00	0,00	100,00	100,00
0,00	0,00	0,00	99,75	99,25
0,00	0,00	0,00	100,00	100,00
0,00	0,00	0,00	100,00	100,00
0,00	0,00	0,00	100,00	100,00
0,00	0,00	0,00	100,00	79,63
0,24	0,00	0,00	100,00	93,98
0,40	0,00	0,00	100,00	19,92
0,00	0,00	0,00	100,00	100,00
0,00	0,00	0,00	100,00	94,29
0,00	0,00	0,00	100,00	100,00
0,00	0,00	0,00	100,00	98,84
0,00	0,00	0,00	100,00	99,34
0,00	0,00	0,00	100,00	97,92
0,00	0,00	0,56	100,00	100,00
0,00	0,00	0,00	100,00	93,33
0,00	0,00	0,00	0,00	0,00
0,57	0,00	0,57	86,93	48,86
0,00	0,00	0,47	100,00	89,57
0,00	0,00	0,00	100,00	33,50
0,00	0,00	0,00	100,00	100,00

0,00	0,00	0,00	100,00	99,03
0,00	0,00	0,00	100,00	99,16
0,00	0,00	0,00	100,00	100,00
0,94	0,63	0,31	99,69	96,24
0,00	0,00	0,00	100,00	100,00
0,00	0,00	0,00	99,23	89,19
0,00	0,00	0,00	100,00	98,44
0,00	0,00	2,80	100,00	95,60
0,00	0,00	0,00	100,00	100,00
0,00	0,00	0,00	100,00	100,00
0,00	0,00	0,00	100,00	7,69
0,00	0,00	0,00	100,00	15,09
0,00	0,00	0,00	100,00	38,89
0,00	0,00	0,00	100,00	99,00
0,57	0,00	0,57	100,00	83,91
0,00	0,00	0,63	100,00	94,97
0,00	0,00	0,00	99,33	98,67
0,89	0,00	0,00	100,00	99,56
0,00	0,00	0,00	100,00	69,80
0,00	0,00	0,00	100,00	91,22
0,00	0,00	0,37	100,00	97,79
0,00	0,00	0,58	100,00	61,40
0,00	0,00	0,44	99,56	98,22
0,68	0,00	0,00	100,00	100,00
0,00	0,00	0,00	100,00	100,00
0,00	0,00	0,00	100,00	100,00
0,00	0,00	0,00	100,00	100,00
0,00	0,00	0,68	100,00	100,00
0,00	0,00	0,00	100,00	99,26
0,00	0,00	0,00	100,00	100,00
0,00	0,00	0,00	100,00	100,00
0,00	0,00	0,00	100,00	100,00
0,00	0,00	0,00	100,00	100,00
0,00	0,00	0,00	100,00	100,00
0,00	0,00	0,00	100,00	100,00
0,00	0,60	0,00	100,00	87,50
0,00	0,00	0,00	100,00	99,29
0,00	0,00	0,41	100,00	99,59
0,00	0,00	0,00	100,00	100,00
0,00	0,00	0,00	99,72	99,72
0,00	0,00	0,00	100,00	56,06
0,00	0,00	0,00	100,00	96,49
0,00	0,00	0,00	100,00	93,94
0,00	0,00	0,00	100,00	100,00
0,00	0,00	0,00	100,00	100,00
0,00	0,00	0,00	100,00	100,00
0,00	0,00	0,00	100,00	98,70
0,00	0,00	0,00	100,00	100,00
0,00	0,00	0,00	100,00	100,00
3,68	0,53	0,53	100,00	99,47

0,00	0,00	0,00	100,00	100,00
0,00	0,00	0,00	100,00	100,00
0,75	0,00	0,00	100,00	100,00
0,00	0,00	0,00	100,00	100,00
0,00	0,00	0,00	100,00	100,00
0,00	0,00	0,00	100,00	100,00
0,00	0,00	0,00	0,00	0,00
0,00	0,00	0,00	100,00	100,00
0,93	0,00	0,00	100,00	94,39
0,00	0,00	0,00	0,00	0,00
1,32	0,00	0,00	100,00	86,84
1,59	0,00	0,00	99,47	95,77
0,00	0,00	0,00	100,00	98,92
0,00	0,00	0,00	100,00	65,28
0,00	0,00	0,00	100,00	23,08
0,00	0,00	0,43	100,00	65,38
0,00	0,00	0,00	100,00	83,33
0,00	2,07	0,41	100,00	100,00
0,00	0,00	0,00	100,00	99,30
0,00	0,00	0,00	100,00	99,55
0,00	0,00	0,00	100,00	100,00
0,00	0,00	0,00	100,00	100,00
0,00	0,00	0,00	100,00	98,43
0,00	0,00	0,00	99,40	79,64
0,00	0,00	0,00	100,00	99,54
0,00	0,00	0,00	100,00	97,78
0,00	0,00	0,00	100,00	97,50
0,00	0,00	0,00	100,00	96,33
0,90	0,45	0,45	100,00	99,10
0,00	0,00	0,00	100,00	21,28
0,00	0,00	1,68	100,00	96,65
0,00	0,00	0,00	100,00	14,81
0,00	0,00	0,00	100,00	54,84
0,00	0,00	0,00	100,00	63,48
0,00	0,00	3,23	100,00	31,18
0,00	0,00	5,49	98,82	85,49
0,00	0,00	0,38	100,00	96,18
0,00	0,00	0,00	0,00	0,00
0,00	0,00	0,00	100,00	55,56
0,00	0,00	0,00	100,00	100,00
0,00	0,00	0,00	100,00	100,00
0,00	0,00	0,00	100,00	82,19
1,35	0,00	0,00	100,00	100,00
0,00	0,00	0,00	100,00	0,00
0,00	0,00	0,00	100,00	0,00
0,00	0,00	0,00	100,00	98,46
0,00	0,00	0,00	100,00	7,75
0,00	0,00	0,00	0,00	0,00
0,00	0,00	0,00	100,00	100,00
0,00	0,00	0,00	100,00	100,00

0,00	0,00	0,00	0,00	0,00
0,26	0,00	0,79	100,00	51,71
0,00	0,00	0,00	100,00	91,21
0,00	0,00	0,00	100,00	99,17
0,00	0,00	0,00	100,00	97,27
0,94	0,00	0,00	100,00	99,53
0,00	0,00	0,00	100,00	100,00
0,00	0,00	0,33	100,00	99,34
0,00	0,00	0,00	100,00	81,69
0,00	0,00	0,00	100,00	97,89
0,00	0,00	0,45	99,55	98,21
0,00	0,00	0,00	100,00	100,00
0,00	0,00	0,00	100,00	100,00
0,00	0,00	0,00	99,62	93,89
0,00	0,00	0,00	0,00	0,00
0,29	0,00	0,00	99,71	99,12
0,00	0,00	0,00	100,00	96,67
0,46	0,00	0,00	100,00	77,31
0,86	0,00	0,00	100,00	96,12
0,00	0,00	3,52	100,00	96,92
0,00	0,00	0,00	100,00	100,00
0,00	0,00	0,46	100,00	99,08
0,38	0,00	0,00	98,86	98,86
0,00	0,00	0,00	100,00	100,00
0,00	0,00	0,00	100,00	86,32
0,00	0,00	0,00	100,00	70,12
0,00	0,00	0,00	100,00	96,31
0,00	0,00	0,23	99,53	51,05
0,00	0,00	0,00	100,00	100,00
0,00	0,00	0,00	100,00	100,00
0,00	0,00	0,00	100,00	89,73
0,31	0,00	0,00	100,00	79,75
0,00	0,00	0,00	100,00	99,06
0,00	0,00	0,93	99,07	58,88
0,00	0,00	0,00	99,35	98,05
0,00	0,00	0,00	100,00	98,60
0,00	0,00	0,00	100,00	99,01
0,00	0,00	0,00	100,00	94,63
0,00	0,00	0,00	100,00	88,62
0,00	0,00	0,00	100,00	99,72
0,00	0,31	0,00	100,00	99,08
0,00	0,00	0,00	100,00	100,00
0,00	0,00	0,00	100,00	97,16
0,00	0,00	0,00	99,70	96,43
0,00	0,00	0,71	100,00	55,00
0,00	0,00	0,00	100,00	98,86
0,00	0,00	0,69	100,00	97,25
0,00	0,00	0,00	100,00	73,38
0,00	0,00	0,00	100,00	95,04
0,00	0,00	0,00	100,00	98,82

0,00	0,00	0,00	100,00	98,83
0,00	0,00	0,00	100,00	99,73
0,00	0,00	0,00	100,00	99,20
0,00	0,00	0,00	100,00	99,60
0,00	0,00	0,00	100,00	98,06
0,00	0,00	0,00	100,00	100,00
0,00	0,00	0,00	100,00	100,00
0,00	0,00	0,00	100,00	99,66
0,00	0,00	0,00	100,00	100,00
0,00	0,00	1,53	98,47	4,58
0,00	0,00	0,00	100,00	91,18
1,74	0,00	0,00	100,00	94,77
0,00	0,00	0,46	100,00	90,87
0,00	0,00	0,00	99,41	77,51
0,00	0,00	0,00	99,54	98,63
0,00	0,00	0,00	100,00	100,00
0,00	0,00	3,35	97,21	85,47
1,44	0,00	97,12	100,00	0,48
0,00	0,00	0,23	100,00	98,83
0,00	0,00	0,00	100,00	100,00
0,00	0,00	0,00	100,00	100,00
0,00	0,00	0,00	100,00	100,00
0,00	0,00	0,00	100,00	99,52
0,00	0,00	0,00	100,00	100,00
0,00	0,00	0,00	100,00	97,45
0,00	0,00	0,00	100,00	99,65
0,34	0,00	0,00	100,00	100,00
0,00	0,00	0,00	100,00	99,29
0,00	0,00	0,00	100,00	100,00
0,00	0,00	0,00	100,00	100,00
0,00	0,00	0,00	100,00	99,65
0,00	0,00	0,00	100,00	99,04
0,00	0,00	0,00	100,00	100,00
0,00	0,00	0,00	100,00	100,00
0,00	0,00	0,00	100,00	98,68
0,00	0,00	0,00	100,00	98,63
0,00	0,00	0,00	100,00	99,53
0,00	0,00	0,35	100,00	98,25
0,00	0,00	0,00	99,48	95,85
0,00	0,00	0,00	100,00	98,48
0,90	0,00	0,00	99,10	10,51
0,00	0,00	0,00	100,00	98,99
2,25	0,00	0,00	100,00	99,10
0,00	0,00	0,00	100,00	100,00
0,00	0,00	0,00	100,00	98,07
0,00	0,00	0,00	100,00	99,43
0,00	0,00	0,00	100,00	100,00
0,00	0,00	0,00	100,00	99,68
0,00	0,00	0,00	100,00	97,93
0,00	0,00	0,86	100,00	97,42

0,00	0,00	0,00	100,00	100,00
0,00	0,00	0,00	100,00	71,66
0,00	0,00	0,00	99,61	99,61
0,00	0,00	1,15	100,00	71,35
0,00	0,00	0,00	100,00	100,00
0,00	0,00	0,00	99,39	95,12
0,46	0,00	0,00	100,00	97,26
0,00	0,00	0,00	100,00	99,29
0,00	0,00	0,00	100,00	99,09
0,00	0,00	0,00	100,00	100,00
0,00	0,00	0,00	100,00	100,00
0,00	0,00	0,00	100,00	100,00
0,00	0,00	0,00	100,00	94,55
0,00	0,00	0,00	100,00	98,77
0,00	0,00	1,61	99,19	99,19
0,00	0,00	0,62	100,00	93,17
0,00	0,00	0,00	100,00	76,92
0,00	0,00	0,00	100,00	100,00
0,00	0,00	0,00	100,00	98,45
0,91	0,00	0,00	100,00	98,18
0,00	0,00	0,00	99,51	83,74
0,00	0,00	0,00	100,00	100,00
0,00	0,00	0,30	100,00	97,58
0,00	0,00	0,00	100,00	98,73
0,00	0,00	0,00	100,00	100,00
0,00	0,00	0,00	100,00	100,00
0,00	0,00	0,00	100,00	79,69
0,00	0,00	0,00	0,00	0,00
0,00	0,00	0,00	100,00	100,00
0,00	0,00	0,00	100,00	100,00
0,00	0,00	0,00	100,00	97,56
0,00	0,00	0,00	100,00	67,19
0,00	0,00	0,63	100,00	67,50
0,00	0,00	0,00	100,00	100,00
0,00	0,00	0,72	100,00	94,20
0,00	0,00	0,21	100,00	94,95
0,00	0,00	0,00	100,00	100,00
0,00	0,00	0,00	100,00	45,45
0,00	0,00	0,00	100,00	100,00
0,00	0,00	0,00	100,00	100,00
0,00	0,00	0,00	100,00	99,64
0,00	0,00	1,10	100,00	96,15
0,00	0,00	0,00	100,00	44,16
0,00	0,00	0,00	98,89	98,33
0,00	0,00	0,00	100,00	66,67
0,00	0,00	0,00	100,00	82,61
0,00	0,00	3,70	98,77	66,67
0,00	0,00	0,00	98,84	98,84
0,00	0,00	0,00	100,00	100,00
0,00	0,00	0,00	100,00	100,00

0,00	0,00	0,00	100,00	99,60
0,00	0,00	0,00	100,00	100,00
0,00	0,00	0,00	100,00	100,00
0,00	0,00	0,00	100,00	16,67
0,00	0,00	0,00	0,00	0,00
0,00	0,00	0,00	0,00	0,00
0,00	0,00	0,00	100,00	100,00
0,00	0,00	0,00	100,00	100,00
0,00	0,00	0,00	100,00	99,38
0,00	0,00	0,00	100,00	99,35
0,00	0,00	0,00	99,37	74,84
0,00	0,00	0,00	100,00	6,98
0,00	0,00	0,00	98,97	93,85
0,00	0,00	0,00	100,00	92,16
0,41	0,00	0,00	100,00	62,24
0,00	0,00	0,00	100,00	100,00
0,00	0,00	0,49	99,01	51,23
0,00	0,00	0,14	100,00	98,75
0,00	0,00	0,00	0,00	0,00
0,00	0,00	0,00	100,00	99,48
0,00	0,00	0,00	100,00	100,00
0,00	0,00	0,00	100,00	100,00
0,00	0,00	0,00	100,00	100,00
0,00	0,00	0,00	99,48	99,48
0,00	0,00	0,00	100,00	100,00
0,47	0,00	0,00	100,00	99,53
0,00	0,00	0,00	99,65	96,85
0,00	0,00	0,00	99,50	98,51
0,00	0,00	0,74	100,00	94,85
0,00	0,00	0,00	100,00	100,00
0,00	0,00	0,00	100,00	99,41
0,00	0,00	0,00	100,00	100,00
0,00	0,00	0,00	100,00	100,00
0,00	0,00	0,00	0,00	0,00
0,46	0,00	0,00	98,63	96,80
0,00	0,00	0,00	100,00	100,00
0,00	0,00	0,85	100,00	98,31
0,00	0,00	0,00	100,00	100,00
0,00	0,00	0,00	100,00	100,00
0,00	0,00	0,00	100,00	100,00
0,00	0,00	0,00	100,00	100,00
0,00	0,00	0,00	100,00	84,31
0,00	0,00	0,00	100,00	100,00
0,00	0,00	0,00	100,00	100,00
0,00	0,00	0,00	100,00	100,00
0,00	0,00	0,00	100,00	100,00
0,00	0,00	0,00	0,00	0,00
0,00	0,00	0,00	100,00	100,00
0,00	0,00	0,00	100,00	100,00
0,00	0,00	0,00	100,00	99,37

0,00	0,00	0,00	100,00	93,12
0,00	0,00	0,00	100,00	88,60
0,00	0,00	0,00	100,00	99,64
0,00	0,00	0,00	100,00	100,00
0,00	0,00	0,00	100,00	99,39
0,00	0,00	0,00	100,00	98,95
0,00	0,00	0,00	100,00	100,00
0,00	0,00	0,00	100,00	97,17
0,00	0,00	0,00	100,00	100,00
0,00	0,00	1,47	100,00	100,00
0,00	0,00	0,00	100,00	93,84
0,00	0,00	0,00	99,74	45,19
0,00	0,00	0,28	99,72	99,17
0,00	0,00	0,43	100,00	43,91
0,00	0,00	0,00	100,00	99,23
0,00	0,00	0,00	100,00	98,58
0,00	0,00	0,33	100,00	79,93
0,00	0,00	0,53	100,00	100,00
0,00	0,00	0,00	100,00	100,00
0,00	0,00	0,46	99,54	99,54
0,00	0,00	0,00	100,00	100,00
0,00	0,00	0,00	100,00	93,87
0,00	0,00	0,00	100,00	100,00
2,25	0,00	0,00	100,00	96,40
0,00	0,00	0,00	100,00	98,20
0,00	0,00	0,00	100,00	100,00
0,00	0,00	0,00	99,62	99,62
0,00	0,00	0,00	99,73	98,37
0,00	0,00	0,00	100,00	85,03
0,00	0,00	0,00	99,59	62,70
0,00	0,00	0,00	100,00	84,02
0,00	0,00	0,00	100,00	99,58
0,74	0,00	0,25	98,28	93,10
0,00	0,00	0,00	100,00	79,03
0,00	0,00	0,34	99,66	98,64
0,00	0,00	0,00	100,00	93,09
0,00	0,00	0,00	98,92	98,21
0,00	0,00	0,00	100,00	98,19
0,00	0,00	0,00	0,00	0,00
0,00	0,00	0,26	99,74	90,65
0,00	1,46	0,00	100,00	100,00
0,00	0,00	0,33	100,00	100,00
0,33	0,00	0,00	100,00	100,00
0,00	0,00	0,00	100,00	100,00
0,00	0,00	0,00	100,00	100,00
0,70	0,00	0,00	100,00	99,30
0,00	0,00	0,00	100,00	100,00
0,41	0,00	0,00	100,00	100,00
0,00	0,00	0,00	100,00	100,00
0,00	0,00	0,00	100,00	100,00

0,00	0,00	0,00	100,00	100,00
0,00	0,00	0,49	100,00	99,51
0,00	0,00	0,00	100,00	100,00
0,00	0,00	0,00	100,00	88,57
1,67	0,00	0,00	100,00	77,50
0,39	0,00	0,00	100,00	93,80
0,00	0,00	0,00	100,00	94,29
0,00	0,00	0,00	100,00	100,00
0,00	0,00	0,00	100,00	63,59
0,00	0,00	0,00	100,00	100,00
0,00	0,00	0,32	99,68	89,00
0,00	0,00	0,00	100,00	96,33
5,88	0,00	0,00	98,53	73,53
0,00	0,00	0,00	100,00	24,37
0,00	0,00	0,00	100,00	100,00
0,00	0,00	0,00	100,00	98,33
0,00	0,00	0,00	100,00	99,70
0,00	0,00	0,00	100,00	99,05
0,34	0,00	0,34	100,00	100,00
0,59	0,00	0,00	100,00	100,00
0,00	0,00	0,00	100,00	100,00
0,00	0,00	0,00	100,00	100,00
0,00	0,00	0,00	100,00	99,55
0,31	0,31	0,00	100,00	99,37
0,25	0,00	0,25	100,00	99,75
0,00	0,00	0,00	100,00	97,19
0,00	0,00	0,00	100,00	100,00
0,00	0,00	0,00	100,00	100,00
0,00	0,00	0,00	100,00	100,00
0,00	0,00	0,00	100,00	100,00
0,55	0,00	0,00	100,00	99,45
0,00	0,00	0,00	100,00	100,00
0,00	0,00	0,99	100,00	99,51
0,00	0,00	0,00	100,00	32,67
0,42	0,00	0,00	99,58	98,74
0,00	0,00	0,00	100,00	62,30
0,00	0,00	0,00	99,69	89,85
0,61	0,00	0,00	100,00	85,45
0,00	0,00	0,00	100,00	86,90
0,00	0,00	0,00	100,00	99,40
2,79	0,93	0,00	100,00	99,53
0,00	0,00	0,00	100,00	99,11
0,00	0,00	0,48	100,00	81,38
0,00	0,00	0,92	100,00	35,69
0,00	0,00	0,00	99,62	56,11
0,00	0,00	3,57	100,00	66,27
0,00	0,00	0,00	100,00	100,00
0,63	0,00	0,00	99,69	95,30
0,52	0,00	0,00	100,00	89,53
0,00	0,00	0,00	100,00	99,35

0,00	0,00	0,00	99,70	99,11
0,56	0,00	0,00	100,00	85,67
0,64	0,00	0,00	100,00	97,11
0,57	0,00	0,29	100,00	94,83
0,00	0,00	0,00	99,78	85,31
0,72	0,00	0,00	100,00	99,28
0,00	0,00	0,00	100,00	100,00
0,00	0,00	0,00	100,00	100,00
0,00	0,00	0,00	0,00	0,00
0,00	0,00	0,00	100,00	99,44
0,00	0,00	0,00	100,00	99,48
0,00	0,00	0,00	100,00	100,00
1,02	0,00	0,00	100,00	100,00
0,00	0,00	0,00	100,00	99,54
0,00	0,00	0,00	100,00	100,00
0,00	0,42	0,00	100,00	96,19
0,00	0,00	0,00	99,63	98,50
0,00	0,00	0,00	100,00	100,00
0,00	0,00	0,00	100,00	99,71
0,27	0,00	0,00	100,00	99,46
0,00	0,00	0,00	100,00	99,70
0,00	0,00	0,00	100,00	100,00
0,00	0,00	0,00	100,00	100,00
0,00	0,00	0,90	100,00	71,17
0,00	0,00	0,00	100,00	100,00
0,00	0,00	0,00	99,55	99,55
0,00	0,00	0,00	100,00	100,00
0,00	0,00	0,00	99,57	97,42
0,00	0,00	0,00	100,00	99,07
0,00	0,00	0,00	100,00	99,71
0,00	0,00	0,00	100,00	94,92
0,00	0,00	0,00	100,00	100,00
0,00	0,00	0,00	100,00	100,00
0,00	0,00	0,00	100,00	100,00
0,00	0,00	0,00	100,00	100,00
0,00	0,00	0,00	100,00	99,22
0,00	0,00	0,00	100,00	94,06
0,00	0,00	0,00	100,00	98,63
0,00	0,00	0,36	100,00	99,64
0,00	0,00	0,00	100,00	98,47
0,00	0,00	0,00	100,00	98,61
0,00	0,00	0,00	100,00	100,00
0,00	0,00	0,00	100,00	100,00
0,00	0,00	0,00	100,00	100,00
0,00	0,00	0,00	100,00	100,00
0,00	0,00	0,00	100,00	100,00
0,00	0,00	0,00	100,00	52,50
0,00	0,00	0,00	100,00	86,05
0,00	0,00	0,00	100,00	92,68
0,00	0,00	0,00	100,00	98,11

0,00	0,00	0,43	100,00	99,14
0,00	0,00	0,00	98,69	98,69
0,00	0,00	0,00	100,00	84,16
0,00	0,00	0,00	100,00	34,91
0,00	0,00	0,00	99,63	98,52
0,00	0,00	0,00	99,61	55,29
0,00	0,00	0,78	99,22	99,22
0,00	0,00	0,00	100,00	100,00
0,00	0,00	0,00	100,00	100,00
0,00	0,00	0,00	100,00	99,35
0,00	0,00	0,00	100,00	98,04
0,00	0,00	0,00	100,00	100,00
0,00	0,00	0,00	100,00	98,07
0,00	0,00	0,00	100,00	100,00
0,00	0,00	0,00	100,00	100,00
0,00	0,00	0,00	100,00	99,68
0,00	0,00	0,00	100,00	100,00
0,00	0,00	0,00	100,00	98,97
0,00	0,00	0,00	100,00	98,61
0,00	0,00	0,00	100,00	99,70
0,00	0,00	0,00	100,00	100,00
0,00	0,00	0,00	100,00	100,00
0,00	0,00	0,00	100,00	100,00
0,00	0,00	0,00	100,00	99,63
0,00	0,00	0,00	100,00	99,06
0,00	0,00	0,00	99,60	99,20
0,00	0,00	0,00	100,00	100,00
0,00	0,00	0,00	100,00	97,24
0,00	0,00	0,00	100,00	99,62
0,00	0,00	0,00	100,00	95,10
0,00	0,93	0,00	100,00	92,56
0,00	0,00	0,00	100,00	64,52
0,00	0,00	0,00	99,46	97,84
0,00	0,00	0,00	100,00	100,00
0,00	0,34	0,00	100,00	98,30
1,17	0,00	0,00	99,61	77,43
0,00	0,00	0,00	100,00	93,03
0,00	0,00	0,24	100,00	95,23
0,00	0,00	0,00	100,00	99,62
0,00	0,00	0,40	100,00	100,00
0,00	0,00	0,00	99,42	99,42
0,00	0,00	0,62	99,38	93,52
0,26	0,00	0,00	100,00	97,11
0,00	0,00	0,00	100,00	93,91
0,00	0,00	0,00	100,00	99,41
0,00	0,00	0,00	100,00	100,00
0,00	0,00	0,00	100,00	99,31
0,00	0,00	0,32	100,00	93,89
0,00	0,00	0,00	100,00	99,72
0,00	0,00	0,00	100,00	99,50

0,00	0,00	0,00	100,00	39,57
0,00	0,00	0,00	100,00	99,13
0,00	0,00	0,00	100,00	93,52
0,00	0,00	0,00	100,00	100,00
0,00	0,00	0,00	100,00	95,00
13,95	0,00	0,00	100,00	62,79
0,58	0,00	0,00	99,42	73,41
0,00	0,00	0,00	0,00	0,00
0,00	0,00	0,00	100,00	90,68
0,35	0,00	0,35	100,00	85,82
0,00	0,96	0,00	100,00	98,08
0,90	0,00	0,00	100,00	5,41
0,00	0,00	0,00	100,00	99,31
0,00	0,00	0,40	100,00	98,39
0,00	0,00	0,00	100,00	77,42
0,00	0,00	0,00	100,00	100,00
0,00	0,00	0,00	100,00	98,59
0,00	0,00	0,00	100,00	100,00
0,00	0,00	0,56	100,00	98,31
0,00	0,00	0,00	100,00	96,55
0,00	0,00	0,00	100,00	100,00
0,00	0,00	0,00	100,00	100,00
0,00	0,00	0,00	100,00	99,11
0,00	0,00	1,04	100,00	94,79
0,00	0,00	0,00	100,00	83,33
0,00	0,00	0,00	100,00	100,00
0,00	0,00	0,00	100,00	96,27
0,00	0,00	0,00	100,00	100,00
0,00	0,00	0,00	100,00	95,45
0,00	0,00	0,75	99,25	90,30
0,00	0,00	0,00	100,00	5,11
0,00	0,00	0,00	100,00	94,67
0,00	0,00	0,00	100,00	95,95
0,00	0,00	0,00	100,00	86,13
0,00	0,00	0,00	100,00	71,64
0,00	0,00	0,69	100,00	84,83
0,00	0,00	0,00	100,00	76,31
0,00	0,00	0,63	100,00	90,63
0,00	0,00	0,00	100,00	95,52
0,00	0,00	0,00	100,00	100,00
0,00	0,00	0,00	100,00	97,12
0,00	0,00	0,00	100,00	100,00
0,00	0,00	0,00	100,00	100,00
0,00	0,00	0,00	100,00	100,00
0,00	0,00	0,00	100,00	43,33
0,00	0,00	0,00	0,00	0,00
0,00	0,00	0,00	100,00	100,00
0,00	0,00	0,00	100,00	100,00
0,00	0,00	0,00	100,00	100,00
0,00	0,00	0,00	100,00	100,00

0,00	0,00	0,00	100,00	100,00
0,24	0,00	2,20	100,00	94,13
0,00	0,00	0,00	100,00	96,10
0,00	0,00	0,00	100,00	100,00
0,00	0,00	1,02	100,00	71,94
0,00	0,00	0,00	100,00	99,13
0,00	0,00	0,00	100,00	99,38
0,00	0,00	0,00	100,00	100,00
0,00	0,00	2,94	100,00	85,29
0,00	0,00	0,00	100,00	100,00
0,00	0,00	0,00	100,00	0,75
0,00	0,00	0,00	100,00	0,00
0,00	0,00	0,00	99,49	99,49
0,00	0,00	0,00	100,00	100,00
0,00	0,00	0,00	100,00	100,00
0,00	0,00	0,64	99,36	99,36
0,00	0,00	0,00	100,00	100,00
0,00	0,00	0,00	100,00	100,00
0,00	0,00	0,00	100,00	100,00
0,00	0,00	0,00	100,00	100,00
0,00	0,00	0,00	100,00	100,00
0,00	0,00	0,00	100,00	100,00
0,00	0,00	0,00	100,00	100,00
0,00	0,00	0,00	100,00	100,00
0,00	0,00	0,00	100,00	100,00
0,00	0,00	0,00	100,00	100,00
0,00	0,00	0,00	100,00	100,00
0,00	0,00	0,00	100,00	100,00
0,00	0,00	0,00	100,00	100,00
0,00	0,00	0,00	100,00	82,80
3,03	0,00	0,00	100,00	100,00
1,63	0,00	65,45	100,00	17,48
0,00	0,00	0,00	100,00	100,00
0,00	0,00	0,00	100,00	76,96
0,00	0,00	2,25	100,00	96,85
0,00	0,00	0,00	100,00	87,60
0,00	0,00	0,00	100,00	100,00
0,00	0,00	0,00	100,00	100,00
0,00	0,00	0,00	100,00	65,93
0,00	0,00	0,00	100,00	100,00
0,00	0,00	0,00	100,00	100,00
0,00	0,00	0,94	99,06	98,11
0,00	0,00	0,00	100,00	100,00
0,00	0,00	0,00	100,00	97,37
0,00	0,00	0,00	100,00	63,49
0,00	0,00	0,00	100,00	93,85
0,00	0,00	0,00	100,00	100,00
0,00	0,00	0,00	100,00	100,00
0,00	0,00	0,00	100,00	99,51
0,00	0,00	0,00	100,00	100,00

0,00	0,00	0,00	100,00	82,76
0,34	0,00	0,00	100,00	90,75
0,00	0,00	0,00	100,00	99,52
0,00	0,00	0,00	100,00	100,00
0,00	0,00	0,00	100,00	98,90
0,00	0,00	0,00	100,00	96,51
0,00	0,00	0,00	100,00	84,58
0,00	0,00	0,22	99,78	83,19
0,00	0,00	0,00	100,00	100,00
0,00	0,00	0,00	100,00	81,32
0,00	0,00	0,94	100,00	88,68
0,00	0,00	0,63	99,68	90,48
0,00	0,00	0,00	100,00	78,53
0,00	0,00	0,00	100,00	73,37
3,70	0,00	0,00	100,00	96,30
0,00	0,00	0,00	100,00	100,00
0,00	0,00	0,00	100,00	100,00
0,00	0,00	0,00	100,00	100,00
0,00	0,00	0,00	100,00	99,64
0,00	0,00	0,00	100,00	99,40
0,00	0,00	0,00	100,00	100,00
0,00	0,00	0,00	100,00	100,00
0,00	0,00	0,00	100,00	99,59
0,00	0,00	0,00	100,00	100,00
0,00	0,00	0,57	100,00	96,57
0,41	0,00	0,00	100,00	99,19
0,00	0,00	0,00	99,50	97,50
0,00	0,00	0,00	98,81	4,17
0,00	0,00	0,00	100,00	98,70
0,00	0,00	0,00	100,00	100,00
0,00	0,00	0,00	100,00	100,00
0,00	0,00	0,00	100,00	100,00
0,00	0,00	0,00	100,00	100,00
0,00	0,00	0,00	100,00	97,92
0,00	0,00	0,00	100,00	100,00
0,99	0,00	32,51	100,00	4,43
0,00	0,00	1,32	98,68	10,53
0,00	0,00	0,00	100,00	1,16
0,00	0,00	10,13	100,00	0,00
0,00	0,00	0,37	100,00	72,79
0,00	0,00	0,00	100,00	100,00
0,00	0,00	1,89	100,00	96,23
0,00	0,00	0,00	100,00	9,52
0,00	0,00	0,00	100,00	16,67
0,00	0,00	0,00	100,00	90,91
0,00	0,00	0,00	100,00	100,00
0,00	0,00	4,76	100,00	100,00
0,00	0,00	0,00	100,00	75,00
0,00	0,00	0,00	0,00	0,00
0,00	0,00	0,00	100,00	97,78

1,19	0,00	0,00	100,00	70,24
0,00	0,00	0,00	0,00	0,00
0,00	0,00	0,00	100,00	100,00
0,00	0,00	0,00	100,00	100,00
0,00	0,00	0,00	100,00	99,42
0,00	0,00	0,00	100,00	99,60
0,00	0,00	0,00	100,00	100,00
0,00	0,00	0,00	100,00	99,01
0,00	0,00	0,00	100,00	100,00
0,00	0,00	0,00	100,00	100,00
0,00	0,00	0,00	100,00	100,00
0,49	0,00	0,00	100,00	100,00
0,00	0,00	0,00	100,00	98,60
0,00	0,00	0,00	100,00	99,62
0,00	0,00	0,00	100,00	97,81
0,00	0,00	0,00	100,00	100,00
0,00	0,00	0,00	100,00	98,68
0,00	0,00	0,00	100,00	79,25
0,00	0,00	0,00	100,00	95,04
0,00	0,00	0,00	100,00	90,95
0,00	0,00	0,00	100,00	94,81
0,00	0,00	0,00	100,00	99,48
0,00	0,00	0,00	100,00	100,00
5,31	0,00	0,00	100,00	98,55
0,00	0,00	0,00	100,00	98,96
0,00	0,00	0,00	100,00	97,85
0,00	0,00	0,00	100,00	98,71
0,00	0,00	0,00	100,00	99,21
0,00	0,00	0,00	100,00	99,59
0,00	0,00	0,00	100,00	99,25
0,00	0,00	0,00	100,00	97,78
0,00	0,00	0,00	100,00	98,88
0,00	0,00	0,40	100,00	100,00
0,00	0,00	0,00	100,00	99,62
0,00	0,00	0,00	100,00	100,00
0,00	0,00	0,00	100,00	96,88
0,00	0,00	0,00	100,00	100,00
0,00	0,00	0,00	100,00	100,00
0,00	0,00	0,00	100,00	100,00
0,00	0,00	0,00	0,00	0,00
0,00	0,00	0,00	100,00	100,00
0,00	0,00	0,00	100,00	99,58
0,00	0,00	0,00	100,00	100,00
0,00	0,00	0,00	100,00	100,00
0,00	0,00	0,00	100,00	100,00
0,00	0,00	0,00	100,00	99,42
0,00	0,00	0,00	100,00	99,33
0,00	0,00	0,00	100,00	83,33
0,00	0,00	0,00	100,00	99,68
0,00	0,00	0,00	100,00	96,72

0,00	0,00	0,00	100,00	100,00
0,00	0,00	0,00	100,00	99,35
0,00	0,00	0,00	100,00	100,00
0,00	0,00	0,00	100,00	96,03
0,00	0,00	0,00	100,00	98,06
1,52	0,00	0,00	100,00	4,55
0,00	0,00	0,00	98,94	32,98
0,00	0,00	0,67	100,00	99,33
0,00	0,00	0,00	0,00	0,00
0,00	0,00	0,00	100,00	97,62
0,00	0,00	0,00	100,00	100,00
0,00	0,00	0,00	100,00	99,51
0,00	0,00	0,00	100,00	100,00
0,00	0,00	0,00	100,00	99,30
0,00	0,00	0,00	100,00	99,18
0,00	0,00	0,00	100,00	100,00
0,00	0,00	0,00	100,00	96,15
0,00	0,00	0,00	100,00	100,00
0,69	0,00	0,00	100,00	99,31
0,00	0,00	0,00	100,00	100,00
0,31	0,00	0,00	100,00	100,00
0,00	0,00	0,00	100,00	98,73
0,00	0,00	0,00	100,00	100,00
0,00	0,00	1,25	100,00	100,00
0,00	0,00	0,00	100,00	100,00
0,00	0,00	0,00	100,00	100,00
0,00	0,00	0,00	99,66	99,66
0,00	0,00	0,00	100,00	100,00
0,51	0,00	0,00	100,00	100,00
0,00	0,00	0,00	100,00	99,69
0,00	0,00	0,00	100,00	100,00
0,00	0,00	1,14	100,00	99,72
0,00	0,00	0,00	100,00	96,06
1,28	0,00	0,00	100,00	99,57
0,00	0,00	0,00	100,00	100,00
0,00	0,00	0,00	100,00	100,00
0,00	0,00	0,00	100,00	100,00
0,00	0,00	0,00	100,00	97,87
0,00	0,00	0,67	100,00	99,67
0,00	0,00	0,00	100,00	100,00
0,00	0,00	0,25	100,00	99,49
0,87	0,00	0,00	100,00	88,21
0,00	0,00	0,00	100,00	98,46
0,47	0,00	0,00	100,00	97,63
0,00	0,00	0,00	100,00	86,42
0,00	0,00	0,00	100,00	100,00
0,00	0,00	0,00	100,00	98,75
0,00	0,00	0,00	100,00	17,33
0,00	0,00	0,36	100,00	40,14
0,00	0,00	2,06	100,00	69,07

0,00	0,00	0,00	100,00	100,00
0,00	0,00	0,00	100,00	100,00
3,11	0,00	0,00	100,00	100,00
0,00	0,00	0,00	100,00	25,67
0,00	0,00	0,00	100,00	100,00
0,00	0,00	0,00	99,71	99,71
0,00	0,00	0,00	100,00	99,45
0,00	0,00	0,00	100,00	100,00
0,61	0,00	0,00	100,00	83,44
0,00	0,00	0,00	100,00	98,63
0,00	0,00	0,30	100,00	99,11
0,00	0,00	0,00	100,00	100,00
0,00	0,00	0,00	100,00	99,61
0,00	0,00	0,00	100,00	100,00
0,00	0,00	0,00	100,00	100,00
0,51	0,00	0,00	100,00	99,49
0,00	0,00	0,00	100,00	100,00
0,00	0,00	0,00	100,00	100,00
0,00	0,00	0,00	100,00	100,00
0,00	0,00	0,00	100,00	100,00
0,00	0,00	0,00	100,00	100,00
0,00	0,00	0,00	100,00	100,00
0,00	0,00	0,66	99,67	98,68
0,00	0,00	0,00	100,00	100,00
0,00	0,00	0,00	100,00	99,46
0,68	0,00	0,00	100,00	99,32
0,00	0,00	0,00	100,00	100,00
0,00	0,00	0,00	100,00	100,00
0,00	0,00	0,00	100,00	100,00
0,00	0,00	0,00	100,00	100,00
0,00	0,00	0,29	100,00	85,67
0,00	0,00	0,00	100,00	100,00
0,00	0,00	0,00	100,00	97,50
0,00	0,00	0,00	100,00	100,00
0,00	0,00	0,00	100,00	98,99
1,05	0,00	0,00	100,00	92,11
0,00	0,00	0,00	99,53	99,53
0,39	0,00	0,00	100,00	63,78
0,00	0,00	0,00	98,70	98,70
2,03	0,00	0,00	100,00	94,59
0,00	0,00	0,00	98,31	96,63
0,00	0,00	0,00	100,00	100,00
0,00	0,00	0,00	100,00	100,00
0,00	0,00	0,00	100,00	100,00
0,00	0,00	0,00	100,00	100,00
0,26	0,00	0,00	99,49	93,35
0,00	0,00	0,00	100,00	95,00
0,00	0,00	0,00	100,00	100,00
0,00	0,00	0,00	100,00	100,00
0,61	0,00	0,00	100,00	82,26

0,00	0,00	0,00	100,00	99,06
0,00	0,00	0,00	100,00	100,00
0,00	0,00	0,00	100,00	100,00
0,00	0,00	0,00	100,00	99,01
0,00	0,00	0,00	100,00	100,00
0,00	0,00	0,00	100,00	100,00
0,00	0,00	0,32	100,00	99,68
0,00	0,00	0,00	100,00	100,00
0,00	0,00	0,00	100,00	100,00
0,00	0,00	0,00	100,00	100,00
0,00	0,00	0,00	100,00	100,00
0,00	0,00	0,52	99,74	97,13
0,00	0,00	0,00	100,00	99,19
0,00	0,00	0,45	100,00	97,75
0,00	0,00	0,00	100,00	100,00
0,00	0,00	0,00	100,00	99,04
0,53	0,00	0,00	100,00	98,42
0,00	0,00	0,00	99,61	96,14
0,00	0,00	0,00	100,00	100,00
0,00	0,00	0,00	100,00	94,12
0,00	0,00	0,00	100,00	51,96
3,00	0,00	0,00	100,00	69,00
4,02	0,00	0,00	100,00	78,57
0,00	0,00	0,00	100,00	98,35
0,00	0,00	0,00	100,00	98,31
0,00	0,00	0,00	100,00	100,00
1,08	0,00	0,00	100,00	98,92
0,30	0,00	0,30	99,40	98,81
0,00	0,00	0,00	100,00	93,14
0,00	0,00	0,00	100,00	100,00
0,00	0,00	0,00	100,00	100,00
0,00	0,00	0,00	100,00	100,00
0,46	0,00	0,00	100,00	100,00
0,33	0,00	0,00	100,00	96,69
0,00	0,00	0,00	100,00	100,00
0,00	0,00	0,00	99,41	97,65
0,00	0,00	0,00	100,00	62,57
0,30	0,00	0,00	100,00	85,80
0,00	0,00	0,00	100,00	98,29
0,54	0,00	2,16	100,00	11,89
0,00	0,00	0,00	100,00	41,77
0,00	0,00	0,00	99,48	97,42
0,00	0,00	0,00	100,00	100,00
0,75	0,00	0,37	100,00	100,00
0,00	0,00	0,00	100,00	99,60
0,00	0,00	0,00	100,00	100,00
0,00	0,00	0,26	100,00	99,48
0,00	0,00	0,00	100,00	100,00
0,00	0,00	0,00	100,00	87,41
0,00	0,00	0,00	100,00	96,28

0,00	0,00	0,00	100,00	100,00
0,00	0,00	0,00	100,00	100,00
0,36	0,00	0,00	100,00	94,64
0,94	0,00	1,89	100,00	33,96
0,00	0,00	0,00	100,00	99,01
0,00	0,00	0,00	100,00	93,75
0,00	0,00	0,00	100,00	95,72
0,00	0,00	0,00	100,00	99,46
5,18	0,00	0,00	100,00	99,18
0,90	0,00	0,00	100,00	88,29
0,38	0,00	0,00	100,00	96,95
0,24	0,47	0,00	99,76	99,76
0,00	0,00	0,00	100,00	100,00
0,00	0,00	0,00	98,47	97,71
0,00	0,00	0,00	100,00	100,00
0,00	0,00	0,00	100,00	95,75
0,00	0,00	0,00	99,62	99,62
0,00	0,00	0,00	100,00	98,53
0,00	0,00	0,00	100,00	92,58
0,29	0,00	0,00	100,00	98,25
0,00	0,00	0,00	100,00	86,73
0,48	0,00	0,00	100,00	100,00
0,00	0,00	0,00	100,00	100,00
0,47	0,00	0,00	100,00	100,00
0,00	0,00	0,00	100,00	98,73
0,00	0,00	0,00	100,00	99,43
0,00	0,00	0,00	100,00	99,49
0,00	0,00	0,00	99,65	93,99
0,00	0,00	0,00	100,00	100,00
0,00	0,00	0,00	99,69	88,99
0,00	0,00	0,00	99,20	98,80
0,00	0,00	0,00	100,00	92,83
0,00	0,00	0,25	100,00	98,02
0,86	0,00	0,00	100,00	83,62
0,00	0,00	0,00	100,00	97,17
0,00	1,12	0,00	100,00	92,13
0,00	0,00	0,00	100,00	99,49
0,00	0,00	0,00	100,00	100,00
0,00	0,00	0,00	99,72	99,72
0,00	0,00	0,00	99,70	99,70
0,00	0,00	0,00	100,00	100,00
0,00	0,00	0,00	99,57	99,14
0,00	0,00	0,00	100,00	99,60
1,56	0,00	0,00	100,00	99,61
0,00	0,00	0,00	100,00	73,36
3,36	0,00	0,00	100,00	97,32
0,81	0,00	0,00	100,00	61,13
1,87	0,00	0,00	99,63	95,88
0,36	0,00	0,00	100,00	80,07
0,00	0,00	0,00	100,00	53,67

0,00	0,00	0,00	100,00	89,88
0,00	0,00	0,30	99,70	73,13
0,00	0,00	0,28	100,00	78,24
0,00	0,00	0,00	100,00	82,02
0,70	0,00	0,00	100,00	97,89
0,00	0,00	0,00	100,00	95,33
0,77	0,00	0,00	99,62	69,62
0,00	0,00	0,00	99,57	96,58
0,00	0,00	0,00	100,00	81,62
1,05	0,00	1,39	100,00	63,76
2,43	0,00	0,40	100,00	42,11
0,00	0,00	0,00	100,00	93,62
0,00	0,00	0,51	100,00	92,86
0,00	0,00	0,00	99,50	99,50
0,00	0,00	0,00	100,00	98,05
0,00	0,00	0,59	97,65	96,47
0,56	0,00	0,00	100,00	100,00
0,00	0,00	0,00	100,00	100,00
0,00	0,00	0,00	100,00	99,70
0,00	0,00	0,00	100,00	96,18
0,00	0,00	0,00	100,00	98,58
0,00	0,00	0,00	100,00	99,53
0,00	0,00	0,00	100,00	96,07
0,00	0,00	0,00	100,00	100,00
0,00	0,00	0,00	100,00	99,09
0,00	0,00	0,00	100,00	100,00
4,98	0,00	0,00	100,00	84,58
0,00	0,00	0,00	100,00	66,67
0,00	0,00	0,00	100,00	100,00
0,00	0,00	0,00	100,00	100,00
0,00	0,00	0,00	99,68	99,36
0,00	0,00	0,73	100,00	99,27
0,00	0,00	0,00	100,00	100,00
2,17	0,00	0,00	100,00	100,00
0,00	0,00	0,00	100,00	97,37
0,00	0,00	0,00	100,00	100,00
0,00	0,00	0,00	100,00	86,11
0,00	0,00	0,00	100,00	100,00
0,28	0,00	0,00	100,00	99,44
0,00	0,00	0,00	100,00	100,00
0,51	0,00	0,00	100,00	97,44
0,00	0,00	0,00	0,00	0,00
0,00	0,00	0,00	100,00	100,00
0,00	0,00	0,00	100,00	100,00
0,00	0,00	0,00	100,00	100,00
0,00	0,57	0,00	100,00	100,00
0,00	0,00	0,00	100,00	83,71
0,00	0,00	0,00	100,00	98,11
0,00	0,00	0,00	100,00	86,29
0,00	0,00	0,00	100,00	96,19

0,00	0,00	0,00	100,00	99,16
0,00	1,88	0,00	99,38	99,38
0,00	0,00	0,00	100,00	100,00
0,00	0,00	0,00	100,00	100,00
0,00	0,00	0,00	100,00	86,36
0,00	0,00	0,00	100,00	100,00
0,00	0,00	0,00	100,00	100,00
0,26	0,00	0,00	100,00	35,55
0,00	0,00	0,00	100,00	31,68
0,00	0,00	0,81	100,00	99,19
0,00	0,00	0,00	100,00	100,00
0,00	0,00	0,00	100,00	100,00
0,00	0,36	0,00	100,00	99,64
0,00	0,00	0,00	100,00	100,00
0,00	0,00	0,00	100,00	100,00
0,00	0,00	0,00	100,00	99,64
0,00	0,00	0,00	100,00	99,10
0,00	0,00	0,00	100,00	100,00
0,00	0,00	0,00	100,00	100,00
0,00	0,00	1,72	100,00	96,55
0,00	0,00	0,00	100,00	100,00
0,00	0,00	0,00	100,00	100,00
0,00	0,00	0,00	100,00	100,00
0,00	0,00	0,00	100,00	100,00
0,00	0,00	0,00	100,00	100,00
0,00	0,00	0,00	100,00	100,00
0,00	0,00	0,00	100,00	100,00
0,00	0,49	0,00	100,00	88,73
0,00	0,00	0,00	100,00	100,00
0,82	0,00	0,00	100,00	99,59
0,00	0,00	0,00	100,00	95,86
1,07	0,00	2,14	100,00	16,04
0,00	0,00	0,00	100,00	100,00
0,00	0,00	0,00	100,00	100,00
0,00	0,00	0,00	100,00	97,30
0,00	0,00	0,00	100,00	99,35
3,74	0,00	1,87	100,00	37,38
0,00	0,00	0,00	99,63	97,79
0,73	0,00	0,73	100,00	94,16
0,00	0,00	0,00	100,00	93,26
0,00	0,00	0,00	100,00	100,00
0,00	0,00	0,00	100,00	67,90
0,00	0,00	0,00	100,00	33,70
0,00	0,00	0,00	100,00	100,00
0,00	0,00	1,01	100,00	77,78
0,00	0,00	0,00	100,00	0,00
0,00	0,00	0,00	100,00	96,00
0,00	0,00	0,00	100,00	100,00
6,15	0,00	0,00	100,00	72,31
0,00	0,00	0,00	100,00	100,00

0,00	0,00	0,00	100,00	100,00
0,00	0,00	0,00	98,84	69,77
0,00	0,00	0,00	98,96	98,96
0,00	1,08	0,00	100,00	100,00
0,00	0,00	0,00	100,00	36,36
0,00	0,00	0,00	100,00	98,91
0,00	0,00	0,00	100,00	95,35
1,75	0,00	0,00	98,25	59,65
0,00	0,00	0,00	100,00	100,00
0,00	0,00	0,00	100,00	80,57
0,00	0,00	0,00	100,00	94,23
0,00	0,00	0,00	100,00	100,00
0,00	0,00	0,00	100,00	66,15
0,00	0,00	0,00	100,00	100,00
0,00	0,00	0,00	100,00	12,10
0,00	0,00	0,00	99,24	14,39
0,00	0,00	1,30	100,00	36,36
0,00	0,00	0,00	100,00	14,61
1,39	0,00	0,00	100,00	13,89
0,00	0,00	0,00	100,00	100,00
0,00	0,00	0,00	100,00	98,71
0,00	0,00	0,00	100,00	100,00
0,00	0,00	0,00	100,00	100,00
0,00	0,00	0,00	100,00	100,00
0,00	0,00	0,00	100,00	100,00
0,00	0,00	0,00	100,00	100,00
0,00	0,00	0,00	100,00	100,00
0,00	0,00	0,33	100,00	96,41
0,00	0,00	0,00	100,00	99,15
0,00	0,00	0,00	100,00	69,95
0,00	0,00	0,00	100,00	78,67
0,67	0,00	0,00	100,00	100,00
0,00	0,00	0,00	100,00	99,34
0,00	0,00	0,00	100,00	100,00
0,00	0,00	0,00	100,00	100,00
0,00	0,00	0,00	100,00	100,00
0,00	0,00	0,00	100,00	100,00
0,00	0,00	0,00	100,00	100,00
0,00	0,00	0,00	98,74	84,28
0,85	0,00	0,00	99,57	77,78
0,00	0,00	0,00	100,00	84,76
0,00	1,14	0,00	99,43	98,86
0,00	0,00	0,36	99,64	96,06
0,00	0,00	0,00	100,00	80,77
0,00	0,00	0,00	100,00	92,22
0,00	0,00	0,00	100,00	100,00
0,00	0,00	0,00	100,00	100,00
0,00	0,00	0,00	100,00	100,00
0,00	0,00	0,00	100,00	90,67
0,00	0,00	0,00	100,00	94,61

0,00	0,00	0,00	100,00	98,05
0,00	0,00	0,00	100,00	99,41
0,00	0,00	0,00	100,00	88,05
0,00	0,00	0,00	100,00	100,00
0,00	0,00	0,00	100,00	100,00
3,41	0,00	0,00	100,00	54,55
0,00	0,00	0,00	100,00	100,00
0,00	0,00	0,00	100,00	3,23
3,57	0,00	3,57	100,00	7,14
0,00	0,00	0,00	100,00	100,00
0,00	0,00	0,00	100,00	85,19
0,00	0,00	0,00	100,00	50,00
0,00	0,00	0,00	100,00	100,00
0,00	0,00	0,00	100,00	91,30
0,00	0,00	0,00	100,00	100,00
4,44	0,00	2,22	100,00	40,00
2,00	0,00	0,00	100,00	100,00
0,00	0,00	0,00	100,00	100,00
0,00	0,00	0,00	100,00	100,00
0,00	0,00	0,00	100,00	98,84
0,00	0,00	0,00	100,00	100,00
0,00	0,00	0,00	100,00	100,00
0,00	0,00	0,00	100,00	96,55
0,00	0,00	0,00	100,00	100,00
0,00	0,00	0,00	100,00	100,00
0,00	0,00	0,00	100,00	44,83
1,39	0,00	0,00	100,00	80,56
0,00	0,00	0,00	100,00	100,00
0,00	0,00	0,00	100,00	100,00
0,00	0,00	0,00	100,00	58,62
0,00	0,00	0,00	99,05	88,57
0,00	0,00	0,00	100,00	100,00
0,00	0,00	0,00	100,00	87,50
0,00	0,00	0,00	100,00	100,00
0,00	0,00	0,00	100,00	89,74
0,00	0,00	0,00	100,00	43,33
0,00	0,00	10,34	100,00	51,72

DOMBHFOS	DOMBHFRU	RSRATMS	RSRMS_1	RSR_2
0,00	0,00	0,00	1,92	3,36
0,00	0,00	0,45	4,95	7,21
0,00	0,00	0,15	0,45	2,27
0,00	0,00	0,00	0,56	1,12
0,00	0,00	0,15	1,76	4,26
0,00	0,00	0,38	10,14	12,42
0,00	0,00	0,82	14,72	12,88
0,00	0,00	0,00	0,49	0,24
6,03	0,00	0,14	0,14	0,85
0,00	0,00	0,00	0,17	0,17
0,00	0,00	0,00	1,91	6,31
0,00	0,00	0,16	1,09	5,15
0,00	0,00	0,00	2,67	4,93
0,00	0,00	0,14	1,10	2,62
0,00	0,00	0,00	0,48	2,24
0,00	0,00	0,13	0,88	1,88
0,00	0,00	0,00	0,23	1,14
1,06	0,00	0,00	0,16	1,41
0,00	0,00	0,00	0,23	0,46
0,00	0,00	0,00	0,19	1,15
0,00	0,00	0,11	0,00	0,44
0,00	0,00	0,23	2,34	3,04
0,00	0,00	0,00	0,50	1,25
0,00	0,00	0,00	0,62	0,92
0,00	0,00	0,00	0,20	1,61
0,00	0,00	0,00	0,16	0,32
0,00	0,00	0,00	0,60	0,20
0,00	0,00	0,29	3,73	8,93
0,73	0,00	0,00	2,10	6,31
0,00	0,94	0,10	5,77	6,80
0,00	0,00	0,00	0,00	0,00
0,00	0,00	0,20	7,39	7,98
0,00	0,00	0,55	3,60	6,36
0,00	0,00	0,16	5,31	5,31
0,00	0,00	0,40	7,22	9,89
1,37	0,00	0,49	4,85	8,25
0,00	0,00	0,00	1,53	3,82
0,00	0,00	0,00	3,50	6,67
0,36	0,00	0,22	5,02	6,88
0,53	0,00	0,17	8,04	8,04
0,00	0,00	0,62	6,79	10,58
0,32	0,97	0,31	7,29	10,16
0,44	0,00	0,64	6,38	10,08
0,39	0,00	0,58	6,24	9,60
0,00	0,00	0,66	13,25	8,33
0,00	0,00	0,00	3,35	7,39
0,00	0,00	0,00	6,87	8,27
0,00	0,00	0,11	1,14	2,63
0,00	0,00	0,15	0,61	3,82

0,00	0,00	0,00	1,73	3,26
0,00	0,00	0,14	0,55	2,34
0,00	0,00	0,24	0,60	1,92
0,00	0,00	0,00	1,03	1,03
0,00	1,36	0,15	2,00	4,31
0,00	0,73	0,24	3,65	4,87
0,00	1,14	0,20	3,97	4,76
0,37	0,00	0,55	6,42	9,29
0,00	0,00	0,37	5,73	8,84
0,00	0,00	0,00	4,73	6,71
0,00	0,00	0,36	3,78	10,27
0,00	0,00	0,00	3,12	9,01
0,00	0,00	0,15	1,64	5,52
0,00	0,00	0,10	2,16	3,54
0,95	0,00	0,30	3,77	5,45
0,00	0,00	0,20	3,19	5,78
0,00	0,00	0,00	6,24	7,85
0,00	0,00	0,76	3,78	4,03
0,00	0,00	0,18	4,17	8,71
0,00	0,00	0,00	3,18	5,94
0,00	0,00	0,00	0,59	2,35
0,00	0,00	0,00	0,17	0,17
0,00	0,00	0,00	0,49	1,22
0,00	0,00	0,00	0,93	1,71
0,00	0,00	0,00	0,75	1,62
0,00	0,00	0,00	0,00	1,38
0,00	0,00	0,00	0,29	0,78
0,00	0,00	0,00	0,73	1,64
0,00	0,00	0,13	0,77	3,99
0,00	0,00	0,00	1,19	2,62
0,00	0,00	0,00	0,68	1,09
0,00	0,00	0,21	0,42	1,67
0,00	0,00	0,00	0,70	1,93
0,00	0,00	0,00	0,68	1,91
0,00	0,00	0,00	0,00	1,28
0,00	0,00	0,00	0,71	0,59
0,00	0,00	0,00	0,74	2,08
0,00	0,00	0,00	0,46	0,77
0,00	0,00	0,00	0,33	0,99
0,00	0,00	0,00	1,92	3,28
0,00	0,00	0,00	1,05	3,76
0,00	0,30	0,10	2,44	3,16
0,00	0,00	0,00	0,67	2,45
0,00	0,00	0,00	0,49	2,43
0,00	0,00	0,31	1,69	3,85
0,00	0,00	0,00	1,23	2,33
0,00	0,00	0,00	0,50	2,00
0,42	0,00	0,16	0,64	2,08
0,00	0,00	0,15	0,29	2,47
0,00	0,00	0,00	0,41	0,82

0,00	0,00	0,00	0,00	0,26
0,00	0,00	0,12	0,24	1,70
0,34	0,00	0,37	0,50	2,49
0,00	0,00	0,16	0,16	2,95
0,00	0,00	0,26	0,92	2,89
0,00	0,00	0,00	0,42	1,83
0,00	0,00	0,13	2,18	3,59
0,00	0,00	0,00	1,11	1,00
0,00	0,00	0,15	0,30	1,22
0,00	0,00	0,00	0,54	0,81
0,00	0,00	0,00	0,61	1,83
0,00	0,00	0,00	0,50	1,74
0,00	0,00	0,00	1,86	2,41
0,00	0,00	0,29	0,29	4,58
0,00	0,00	0,00	1,35	2,54
0,00	0,00	0,00	0,12	1,78
0,00	0,00	0,00	0,25	1,27
0,00	0,00	0,00	2,16	2,16
0,00	0,00	0,00	1,42	0,57
0,00	0,00	0,00	0,23	0,94
0,00	0,00	0,00	0,00	0,00
0,00	0,00	0,46	5,50	2,75
0,00	0,00	0,00	1,91	2,75
0,00	0,00	0,00	4,05	4,86
0,00	0,00	0,00	0,00	0,00
0,00	0,00	0,00	1,04	3,11
0,00	0,00	0,00	1,74	3,47
0,00	0,00	0,00	1,06	0,85
0,00	0,00	0,00	0,36	1,96
0,00	0,00	0,00	1,05	2,74
0,00	0,00	0,00	3,01	4,37
0,00	0,00	0,00	0,33	0,17
0,00	0,00	0,00	0,00	3,26
0,00	0,00	0,00	0,93	2,06
0,00	0,00	0,00	1,01	1,81
0,00	0,00	0,00	0,35	2,82
0,00	0,00	0,00	0,29	2,58
0,00	0,00	0,15	1,06	2,26
0,39	0,00	0,26	0,66	0,79
0,00	0,00	0,00	0,73	2,02
0,00	0,00	0,00	0,44	3,11
0,00	0,00	0,00	1,28	2,14
0,00	0,00	0,00	0,68	1,80
0,00	0,00	0,00	0,66	1,53
0,00	0,00	0,00	0,00	1,53
0,00	0,00	0,00	0,86	2,15
0,00	0,00	0,00	1,02	1,76
0,00	0,00	0,28	1,39	3,48
0,00	0,87	0,76	9,67	8,91
0,00	0,00	0,63	13,00	8,38

0,00	0,00	0,47	15,35	8,53
0,00	0,00	0,24	9,98	11,30
0,00	0,00	0,00	10,24	11,02
0,00	0,00	0,53	17,44	7,32
0,00	0,00	0,62	14,78	9,24
0,00	0,53	1,32	11,26	8,92
0,00	0,00	0,00	0,72	0,54
2,08	0,00	3,16	10,13	8,86
0,00	0,00	0,36	14,06	7,47
0,00	0,00	0,78	11,14	9,13
0,00	0,00	4,27	12,18	8,55
0,00	0,00	1,33	10,80	6,67
0,00	0,41	2,87	11,27	8,29
0,00	0,00	1,29	13,32	7,88
0,00	0,00	0,63	11,69	9,00
0,00	0,00	0,47	15,53	7,29
0,00	0,00	0,96	12,05	8,41
0,00	0,00	1,83	7,33	7,07
0,00	0,00	1,26	11,08	13,60
0,00	0,00	0,71	12,56	8,06
0,00	0,00	0,68	13,18	10,10
0,00	0,00	0,82	18,25	4,99
0,00	0,00	1,61	11,58	12,22
0,00	1,32	0,36	16,55	8,63
0,00	0,00	0,96	11,47	6,88
0,00	0,00	0,43	11,03	9,60
0,00	0,00	0,50	11,75	11,75
0,00	0,00	0,00	13,23	9,28
0,00	0,00	0,29	9,10	12,64
0,00	0,00	0,92	11,71	11,71
0,00	0,00	0,17	12,87	9,22
0,00	0,52	0,87	13,93	10,16
0,00	0,00	1,11	14,76	8,12
0,00	0,00	1,45	13,56	4,84
0,00	0,00	0,91	11,26	9,28
0,00	0,00	0,87	16,18	8,38
0,00	0,00	1,13	12,43	12,29
0,00	0,00	0,31	13,66	10,87
0,00	0,00	1,39	10,79	12,82
0,00	0,00	0,61	10,94	12,31
0,00	0,00	0,49	14,63	9,76
0,74	0,00	1,12	11,45	9,30
1,06	0,70	0,41	12,93	9,20
25,72	62,38	0,51	15,09	9,80
0,00	0,00	0,30	1,81	2,71
0,00	0,00	0,00	0,49	0,74
0,00	0,00	0,00	0,71	0,95
0,00	0,00	0,00	0,15	0,77
0,00	0,00	0,17	1,34	2,68
0,00	0,00	0,16	0,81	2,28

0,00	0,00	0,00	0,33	0,82
0,00	0,00	0,14	0,41	1,10
0,00	0,00	0,13	1,02	0,64
0,00	0,00	0,00	0,00	0,66
0,00	0,00	0,00	1,22	2,72
0,00	0,00	0,00	1,61	3,08
0,00	0,00	0,09	0,69	1,20
0,00	0,00	0,00	1,49	1,99
0,00	0,00	0,00	0,90	2,03
0,00	0,00	0,00	0,91	0,73
0,00	0,00	0,24	1,20	2,17
0,00	0,00	0,00	0,64	2,30
0,00	0,00	0,00	1,20	3,25
0,00	0,00	0,00	1,00	3,00
0,00	0,00	0,00	2,15	2,73
0,00	0,00	0,52	1,05	2,36
0,00	0,00	0,23	0,93	1,75
0,00	0,00	0,00	0,00	0,00
0,00	0,00	0,00	1,93	2,31
0,00	0,00	0,00	2,04	2,72
0,00	0,00	0,00	1,71	2,13
0,00	0,00	0,35	1,76	5,63
0,00	0,00	0,00	2,47	4,27
0,00	0,00	0,00	6,41	2,56
0,00	0,00	0,31	4,69	12,19
0,00	0,00	0,00	6,56	10,66
0,00	0,00	0,00	2,72	10,66
0,00	0,00	0,00	3,49	6,43
0,00	0,00	0,00	2,60	7,55
0,00	0,00	0,00	2,31	4,47
0,00	0,00	0,29	1,76	5,14
0,00	0,00	0,27	1,64	3,82
0,00	0,00	0,17	2,78	4,17
0,00	0,00	0,00	1,55	5,32
0,00	0,00	0,14	2,43	6,71
0,00	0,00	0,18	4,32	7,91
0,00	2,27	0,77	2,31	6,15
0,00	0,00	0,18	0,89	3,73
0,00	0,00	0,18	1,25	3,75
0,00	0,55	0,00	1,79	6,12
0,00	0,00	0,19	1,69	5,07
0,00	0,00	0,00	0,54	1,44
0,00	0,00	0,00	0,14	0,55
0,00	0,00	0,00	0,20	0,61
0,00	0,00	0,00	0,57	1,86
0,00	0,00	0,13	0,13	1,53
0,00	0,00	0,00	0,79	2,75
0,00	0,00	0,17	0,87	3,65
0,00	0,00	0,13	1,03	3,08
0,00	0,00	0,00	1,61	2,49

0,00	0,00	0,00	0,61	2,43
0,00	0,00	0,00	0,57	1,29
0,00	0,00	0,00	0,31	1,09
0,00	0,00	0,00	0,32	1,27
0,00	0,00	0,12	0,50	1,36
0,00	0,00	0,00	0,47	0,71
0,00	0,00	0,00	0,50	0,50
0,00	0,00	0,22	3,02	3,23
0,00	0,00	0,00	2,48	6,77
0,00	0,00	0,00	2,10	5,26
0,00	0,00	0,00	0,73	2,19
0,83	0,00	0,18	1,63	3,81
0,00	0,00	0,00	1,00	3,67
0,00	0,00	0,31	5,64	7,21
0,00	0,00	0,23	0,90	3,39
0,00	0,00	0,00	1,72	3,10
0,00	0,00	0,00	2,60	5,52
0,00	0,00	0,19	2,43	5,24
0,54	0,00	0,24	6,11	9,78
0,00	0,00	0,00	2,43	6,48
0,00	0,00	0,46	5,36	9,95
0,00	0,00	0,16	0,32	1,46
0,00	0,00	0,00	0,00	0,95
0,00	0,00	0,00	0,95	1,08
0,00	0,00	0,00	0,70	1,40
0,00	0,00	0,00	0,65	0,78
0,00	0,00	0,00	0,97	1,10
0,00	0,00	0,00	0,25	1,65
0,00	0,00	0,00	0,52	1,20
0,00	0,00	0,00	0,47	1,72
0,00	0,00	0,00	0,52	0,90
0,00	0,00	0,17	1,00	2,00
0,00	0,00	0,00	1,06	3,18
0,81	0,00	0,15	1,68	3,36
0,32	0,00	0,00	1,29	2,59
0,00	0,00	0,13	0,66	1,98
0,00	0,00	0,00	1,08	3,36
0,00	0,00	0,34	0,34	1,54
0,00	0,00	0,00	0,00	0,45
0,00	0,00	0,12	0,50	0,75
0,00	0,00	0,00	0,28	0,70
0,00	0,00	0,00	0,00	0,00
0,00	0,00	0,18	0,72	0,54
0,00	0,00	0,29	0,15	1,17
0,00	0,00	0,25	1,25	3,24
0,00	0,00	0,00	0,00	0,79
0,00	0,00	0,00	0,33	1,34
0,00	0,00	0,35	2,95	5,72
0,00	0,00	0,00	5,11	14,96
0,00	0,00	0,20	3,01	10,24

0,00	0,00	0,16	3,76	10,03
0,00	0,00	0,00	1,98	8,32
0,00	0,00	0,00	5,63	10,74
0,40	0,00	0,00	0,12	0,36
0,00	0,00	0,00	0,00	0,66
0,00	0,00	0,00	0,00	1,22
0,00	0,00	0,00	0,45	0,60
0,00	0,00	0,00	0,18	0,53
0,49	0,00	0,00	0,00	0,32
0,00	0,00	0,00	0,00	0,98
0,00	0,00	0,00	0,00	0,00
0,00	0,00	0,09	0,47	1,13
0,00	0,00	0,00	0,85	1,70
0,00	0,00	0,00	0,79	2,54
0,00	0,00	0,00	0,00	1,51
0,00	0,00	0,00	0,39	3,15
0,00	0,00	0,20	0,40	1,81
0,64	0,00	0,00	0,65	0,91
0,00	0,00	0,16	0,66	1,98
0,00	0,00	0,32	0,80	1,92
0,00	0,00	0,13	0,39	1,54
0,00	0,00	0,30	0,00	0,00
0,00	0,00	0,00	0,20	1,21
0,00	0,00	0,00	0,00	0,29
0,00	0,00	0,12	0,12	1,90
0,00	0,00	0,00	1,45	2,41
0,00	0,00	0,00	0,00	0,00
0,00	0,00	0,00	2,11	3,02
0,00	0,00	0,27	4,89	11,14
0,00	0,00	0,25	3,93	8,60
0,00	0,00	0,00	5,86	17,58
0,00	0,00	0,00	5,84	15,56
0,00	0,00	0,36	2,17	6,86
0,00	0,44	0,00	3,08	6,83
0,00	0,00	0,00	4,60	14,00
0,00	0,00	0,00	1,40	6,21
0,00	0,00	0,00	3,42	7,55
0,00	0,00	0,00	0,26	0,66
0,00	0,00	1,82	13,80	7,91
0,00	0,00	0,40	12,35	11,13
4,68	0,36	1,71	12,52	7,40
0,00	0,00	1,57	13,17	9,09
0,00	0,00	0,86	9,13	9,27
0,67	0,00	0,75	15,07	6,21
0,42	0,00	0,67	11,27	8,26
0,00	0,00	0,00	17,57	8,26
1,03	0,00	0,28	13,77	7,44
2,42	0,00	0,89	12,42	9,58
0,00	0,00	1,07	11,95	9,11
0,00	0,00	1,05	15,04	9,36

0,00	0,00	0,51	11,02	10,41
0,00	0,00	0,29	9,52	12,24
0,00	0,00	0,61	10,33	5,83
0,00	0,00	0,37	7,37	9,61
0,00	0,00	1,01	11,05	10,95
0,00	0,00	0,55	7,65	14,21
0,96	0,96	0,97	10,72	8,88
0,00	0,00	0,28	0,85	1,13
0,00	0,00	0,00	0,37	0,55
0,00	0,00	0,00	0,38	1,78
0,00	0,00	0,00	0,49	0,99
0,00	0,00	0,11	0,23	0,79
0,00	0,00	0,00	0,69	0,17
0,32	0,00	0,00	0,58	0,58
0,00	0,00	0,00	0,34	1,91
0,00	0,00	0,43	1,28	0,85
0,00	0,00	0,00	1,25	1,81
0,00	0,00	0,00	1,06	1,24
0,00	0,00	0,19	1,17	1,55
0,00	0,00	0,00	0,65	2,37
0,00	0,00	0,00	0,62	1,66
0,00	0,00	0,16	0,82	2,45
0,00	0,00	0,00	0,69	1,04
0,00	0,00	0,00	0,86	1,72
0,00	0,00	0,19	0,93	1,68
0,00	0,00	0,00	2,39	6,63
0,00	0,00	0,00	3,29	9,21
0,00	0,00	0,00	2,78	6,08
0,00	0,00	0,34	4,03	4,36
0,00	0,00	0,18	5,89	12,32
0,00	0,00	0,00	3,18	7,60
0,00	0,00	0,00	0,00	0,00
0,70	0,00	0,00	0,51	1,22
0,00	0,00	0,00	0,40	0,93
0,00	0,00	0,00	0,60	1,19
0,00	0,00	0,24	0,24	0,48
0,00	0,00	0,00	0,00	0,00
0,00	0,00	0,11	1,01	2,70
0,00	0,00	0,00	0,27	0,94
0,00	0,00	0,00	1,79	2,38
0,00	0,00	0,11	0,11	0,34
0,00	0,00	0,00	1,26	3,06
0,00	0,00	0,17	0,17	0,69
0,53	0,00	0,00	0,82	1,63
0,00	0,00	0,43	0,86	1,72
0,00	0,00	0,20	0,60	1,81
0,00	0,00	0,00	1,14	2,86
0,00	0,00	0,00	0,56	2,59
0,00	0,00	0,00	2,24	3,25
0,00	0,00	0,41	0,00	2,27

0,00	0,00	0,00	0,73	0,73
0,00	0,00	0,18	2,00	2,00
0,00	0,00	0,00	0,12	0,72
0,00	0,00	0,26	0,52	1,05
0,00	0,00	0,00	0,00	0,00
0,00	0,00	0,16	1,59	3,19
0,00	0,00	0,00	0,00	0,00
0,00	0,00	0,00	0,00	0,20
0,00	0,00	0,00	0,36	0,18
0,00	0,00	0,00	0,26	0,00
0,00	0,00	0,12	0,12	0,24
0,00	0,00	0,00	0,13	0,63
0,41	0,00	0,00	0,77	1,39
0,00	0,00	0,00	0,66	1,31
0,00	0,00	0,00	0,35	0,35
0,00	0,00	0,00	0,46	0,92
0,00	0,00	0,00	0,00	0,00
0,00	0,00	0,00	0,39	0,91
0,00	0,00	0,00	0,29	1,02
0,00	0,00	0,19	0,47	0,84
0,00	0,00	0,09	0,44	0,89
0,43	0,00	0,00	0,34	0,51
0,00	0,00	0,00	0,27	0,82
0,00	0,00	0,00	0,00	0,28
0,00	0,00	0,00	0,68	1,71
0,00	0,00	0,00	0,65	2,20
0,00	0,00	0,00	0,00	0,70
0,00	0,00	0,72	9,99	12,33
0,00	0,00	0,00	0,34	3,38
0,00	0,00	0,28	1,12	1,68
0,00	0,00	0,29	0,88	2,05
0,00	0,00	0,00	0,30	0,91
0,00	0,00	0,00	0,17	1,40
0,00	0,00	0,00	2,32	4,64
0,00	0,00	0,00	0,38	0,57
0,00	0,00	0,00	1,36	1,91
0,00	0,00	0,00	3,46	2,77
0,00	0,00	0,00	2,26	4,07
0,58	0,00	0,23	0,91	1,14
0,00	0,00	0,00	2,68	2,50
0,00	0,00	0,35	1,38	1,38
0,00	0,00	0,00	0,19	2,23
0,00	0,00	0,00	1,13	8,17
0,00	0,00	0,21	1,07	2,15
0,00	0,00	0,00	0,78	1,04
0,00	0,00	0,00	0,82	1,91
0,00	0,00	0,00	0,68	1,71
0,00	0,00	0,00	3,35	6,10
0,00	0,00	0,00	0,52	1,73
0,00	0,00	0,00	0,49	0,98

0,00	0,00	0,00	0,93	3,94
0,00	0,00	0,31	1,25	3,43
0,38	0,00	0,00	1,37	1,51
0,00	0,00	0,00	0,34	0,57
0,00	0,00	0,00	0,18	0,18
0,00	0,00	0,00	0,00	0,19
0,00	0,00	0,00	0,00	1,33
0,00	0,00	0,00	0,49	0,98
0,00	0,00	0,00	0,59	1,77
0,00	0,00	0,00	0,78	3,11
0,00	0,00	0,00	0,33	1,34
0,00	0,00	0,00	0,00	0,92
0,00	0,00	0,63	1,88	0,63
0,00	0,00	0,00	1,59	3,90
0,00	0,00	0,00	0,85	1,41
0,00	0,00	0,00	0,00	0,66
0,32	0,00	0,00	0,22	0,32
0,00	0,00	0,00	1,85	8,15
0,00	0,00	0,00	0,75	1,25
0,00	0,00	0,00	0,83	0,83
0,00	0,00	0,00	0,00	2,88
0,00	0,00	0,00	0,76	1,08
0,00	0,00	0,00	0,30	3,04
0,00	0,00	0,00	0,79	1,42
0,00	0,00	0,00	0,49	0,97
0,57	0,00	0,00	1,28	1,71
0,00	0,00	0,00	0,58	2,91
0,00	0,00	0,00	0,24	1,20
0,00	0,00	0,00	0,46	0,93
0,00	0,00	0,00	1,88	2,50
0,00	0,00	0,00	0,00	2,56
0,00	0,00	0,00	2,47	4,94
0,00	0,00	0,00	0,70	0,00
0,00	0,00	0,00	0,00	3,16
0,00	0,00	0,00	0,00	0,20
0,00	0,00	0,00	0,23	0,94
0,00	0,00	0,00	0,00	0,00
0,00	0,00	0,00	2,55	6,37
0,00	0,00	0,00	0,00	0,33
0,00	0,00	0,00	1,25	1,25
0,00	0,00	0,00	2,20	1,57
0,00	0,00	0,00	0,79	1,18
0,00	0,00	0,00	3,75	3,13
0,00	0,00	0,00	0,00	2,56
0,00	0,00	0,00	0,00	1,96
0,00	0,00	0,00	0,25	1,02
0,00	0,00	0,00	0,00	0,68
0,00	0,00	0,00	0,99	0,66
0,00	0,00	0,00	0,00	0,00
0,00	0,00	0,00	0,00	1,42

0,00	0,00	0,62	13,17	8,85
0,00	0,00	0,00	2,65	8,61
0,00	0,00	0,00	0,00	0,90
0,00	0,00	0,00	0,00	0,00
0,00	0,00	0,93	10,22	11,76
0,00	0,00	0,00	1,07	2,68
0,00	0,00	0,84	8,40	15,13
0,00	0,00	0,50	14,07	5,53
0,00	0,00	0,19	2,29	5,15
0,00	0,00	1,59	10,76	11,16
0,86	0,00	0,22	7,51	5,96
0,00	0,00	0,00	0,14	0,84
0,00	0,00	0,00	0,00	0,00
0,00	0,00	1,54	12,18	10,12
0,00	0,00	1,20	9,37	7,50
0,00	0,00	0,00	0,69	0,35
3,45	0,00	0,00	0,00	1,86
0,00	0,00	0,00	1,17	2,73
0,00	0,00	1,89	11,79	8,96
0,00	0,00	0,29	10,29	12,86
0,88	0,88	0,25	13,58	8,64
0,85	0,00	0,62	11,00	9,49
0,00	0,00	0,00	0,37	0,74
0,00	0,00	0,00	2,41	5,42
0,00	0,00	0,00	0,00	0,00
0,00	0,00	0,00	1,24	2,24
0,00	0,00	0,00	0,52	0,00
0,00	0,00	0,00	0,00	0,00
0,00	0,00	0,24	2,63	5,26
0,00	0,00	0,00	7,19	11,51
0,00	0,00	0,00	11,11	9,72
0,00	0,00	0,00	11,11	9,15
0,00	0,00	0,00	15,00	8,33
0,00	0,00	0,00	0,00	0,00
0,00	0,00	0,18	0,18	0,92
0,00	0,00	0,00	1,09	1,63
0,00	0,00	0,00	0,69	0,92
0,00	0,00	0,00	1,69	3,38
0,00	0,00	0,00	0,89	2,46
0,00	0,00	0,00	0,00	0,90
0,54	0,00	0,00	0,00	1,07
0,00	0,00	0,00	3,31	5,73
0,00	0,00	0,12	3,75	5,51
0,00	0,00	0,74	9,51	8,17
0,00	0,00	0,12	3,22	6,79
0,00	0,00	0,22	3,55	7,10
0,00	0,00	0,13	4,24	7,33
0,50	0,00	0,16	4,60	6,40
0,00	0,00	0,64	9,41	6,22
0,00	0,75	0,22	3,45	5,17

0,00	0,00	0,41	4,23	5,74
0,00	0,00	1,12	7,87	12,36
0,00	0,00	0,00	0,00	0,00
0,00	1,02	0,94	7,09	9,13
0,00	0,00	0,00	8,63	7,49
1,03	0,00	0,15	8,18	8,91
0,32	0,00	0,39	8,01	9,85
0,66	0,99	0,42	10,59	7,27
0,00	0,00	0,97	10,30	11,27
0,00	0,00	0,74	6,80	7,79
0,00	0,00	0,00	4,90	9,31
0,00	0,00	0,24	6,20	8,34
0,00	0,00	0,00	4,98	7,32
0,50	0,00	0,00	3,13	5,64
0,85	0,00	0,39	6,18	10,00
0,00	0,00	0,00	4,77	6,52
0,00	0,00	0,14	0,82	5,05
0,00	0,00	0,00	2,30	6,44
0,00	0,00	0,00	2,92	5,63
0,94	0,00	0,32	4,63	7,89
0,00	0,00	0,00	2,56	4,01
0,00	0,00	0,19	4,75	6,65
0,00	0,00	0,14	4,32	6,47
0,57	0,00	0,00	3,56	5,97
0,00	0,00	0,47	4,12	6,71
0,00	0,00	0,65	4,20	9,53
0,00	0,00	0,00	9,64	7,37
0,00	0,00	0,00	2,22	9,31
0,00	0,00	0,15	2,17	5,57
0,00	0,00	0,23	7,27	11,72
0,00	0,43	0,00	1,59	15,46
0,00	0,00	0,00	6,64	11,78
0,00	0,00	0,48	9,84	8,57
0,00	0,00	0,19	6,69	9,94
0,00	0,00	0,00	4,97	11,14
0,00	0,00	0,54	9,21	11,79
0,00	0,35	0,32	9,44	12,62
0,00	0,00	0,45	9,73	9,58
1,03	0,00	0,00	8,93	10,18
0,00	0,00	0,19	7,57	11,84
0,00	0,00	0,00	9,12	5,40
0,39	0,00	0,24	8,46	7,64
0,68	2,04	1,00	10,44	10,24
0,00	0,00	0,58	5,24	9,71
0,00	0,36	0,12	3,67	5,13
0,00	0,00	0,44	3,29	6,36
0,00	0,00	0,12	2,94	3,68
0,00	0,00	0,00	1,80	4,90
0,00	0,00	0,00	3,21	6,43
0,00	0,00	0,00	3,96	6,55

0,00	0,00	0,00	3,67	6,88
0,00	0,00	0,00	1,40	6,87
0,00	0,00	0,13	4,01	7,49
0,00	0,00	0,29	5,48	7,07
0,00	0,00	0,22	2,97	6,82
0,00	0,00	0,00	2,03	5,38
0,00	0,00	0,39	1,95	4,29
0,00	0,00	0,80	1,86	5,84
0,00	0,00	0,43	4,39	8,99
14,29	0,00	0,00	8,29	8,29
0,00	0,79	0,72	3,37	9,04
0,00	0,00	0,00	3,02	5,41
0,00	0,00	0,00	2,37	7,79
0,00	0,00	0,21	7,30	7,30
0,00	0,00	0,56	6,07	9,00
0,00	0,00	0,41	3,12	10,18
0,00	0,00	0,54	8,03	8,30
0,67	0,00	0,65	8,84	6,03
2,15	0,00	0,17	7,55	12,69
0,00	0,00	1,68	10,16	7,12
0,00	0,00	0,39	10,85	10,47
0,00	0,00	0,49	10,07	11,04
0,00	0,00	0,77	10,36	8,31
0,00	0,00	0,54	9,26	9,81
0,00	0,00	0,58	9,39	10,16
0,00	0,00	0,70	8,93	8,58
0,00	0,00	0,00	4,45	11,46
0,00	0,00	0,40	5,45	7,88
0,00	0,00	0,00	3,52	7,48
0,00	0,00	0,20	5,77	8,55
0,00	0,00	0,45	10,54	10,76
0,00	0,00	0,17	2,60	7,27
0,00	0,00	0,27	2,47	6,18
0,00	0,00	0,21	3,72	6,20
0,00	0,00	0,30	3,47	5,14
0,00	0,00	0,27	2,82	4,70
0,36	0,00	0,00	3,58	5,18
0,00	0,00	0,00	2,80	3,53
0,00	0,00	0,19	1,70	4,72
0,00	0,00	0,13	2,92	4,83
0,00	0,00	0,17	2,32	6,30
0,00	0,00	0,17	1,65	7,11
0,00	0,35	0,24	4,03	6,52
0,00	0,33	0,00	5,31	5,77
0,00	0,00	0,00	6,12	6,96
0,00	0,00	0,36	4,67	6,58
0,00	0,00	0,27	3,47	6,67
0,00	0,00	0,34	11,71	10,19
1,69	0,85	0,60	9,47	10,19
1,06	0,00	2,43	7,99	7,42

0,00	0,00	0,78	8,26	11,53
0,00	1,19	0,00	13,11	9,05
0,00	0,00	0,84	10,51	8,72
0,00	0,00	0,10	12,15	11,95
0,38	0,00	0,13	8,48	13,67
0,00	0,00	0,00	10,56	9,88
0,00	0,00	0,27	5,68	9,88
0,00	0,00	0,00	5,84	7,79
0,00	0,00	0,00	5,02	7,79
0,00	0,00	0,17	2,17	5,83
0,00	0,00	0,00	1,27	3,60
0,00	0,00	0,00	3,39	5,31
0,00	0,00	0,13	12,63	11,84
0,00	0,39	0,00	2,39	5,34
0,00	0,00	0,00	3,69	6,50
0,00	0,00	0,00	2,65	4,34
0,00	0,00	0,13	3,99	6,78
0,00	0,00	0,00	3,62	4,43
0,00	0,00	0,00	3,78	6,29
0,00	0,00	0,00	2,86	6,06
0,00	0,00	0,11	4,09	5,48
0,00	0,00	0,00	2,45	3,48
0,00	0,00	0,28	3,38	6,19
0,00	0,00	0,17	2,15	5,61
0,00	0,00	0,09	3,47	7,31
0,39	0,00	0,12	4,62	10,34
0,00	0,00	0,00	6,63	12,54
0,00	0,00	0,43	5,26	7,84
0,00	0,00	0,10	8,11	9,97
0,00	0,00	0,24	7,26	9,49
0,00	0,00	0,55	6,87	11,12
0,37	0,00	0,34	9,29	8,83
0,00	0,00	0,15	9,04	10,72
0,00	0,00	0,00	10,53	10,15
18,58	0,00	1,50	8,77	12,03
0,00	0,00	0,72	14,49	8,77
0,33	0,00	1,29	9,22	10,41
0,00	0,00	0,51	12,90	8,68
0,40	0,79	0,83	8,17	10,41
0,00	0,00	1,32	9,96	8,88
0,63	0,00	1,23	11,97	10,04
0,00	0,00	1,23	11,93	7,61
0,00	0,00	0,95	8,59	15,87
0,00	0,00	0,35	8,30	12,80
0,79	1,98	0,56	8,74	10,20
0,00	0,00	0,26	10,00	14,21
0,00	0,00	0,12	5,24	8,61
0,00	0,00	0,14	6,95	8,37
0,00	0,00	0,14	3,03	6,61
0,00	0,00	0,00	4,17	6,42

0,00	0,00	0,00	4,66	9,31
0,00	0,00	0,00	3,40	6,47
0,00	0,00	0,30	3,78	7,10
0,00	0,00	0,41	4,50	5,86
0,00	0,00	0,00	5,47	8,43
0,00	0,00	0,10	5,27	7,22
0,00	0,00	0,00	2,50	3,49
0,00	0,00	1,50	11,71	10,11
0,00	0,00	0,00	9,28	6,80
0,00	0,00	1,58	12,08	10,50
0,39	0,39	0,00	2,42	3,77
0,00	0,00	0,39	2,54	4,88
0,00	0,00	0,00	3,37	4,60
0,00	0,00	0,00	1,22	6,91
0,00	0,00	0,46	2,55	4,63
0,00	0,00	0,00	2,21	4,04
0,00	0,00	0,00	1,42	5,29
0,38	0,00	0,00	4,96	6,23
0,00	0,00	0,51	2,53	6,84
0,00	0,00	0,00	3,04	6,07
0,00	0,00	0,19	1,96	3,91
0,67	0,00	0,00	3,19	3,99
0,40	0,00	0,00	0,91	1,94
0,00	0,00	0,41	2,45	4,76
0,00	0,00	0,36	0,54	4,11
0,00	0,00	0,00	8,26	8,94
0,00	0,00	0,25	5,93	8,64
0,00	0,00	0,00	5,82	8,67
0,00	0,00	0,09	9,57	12,23
0,00	0,66	0,00	1,52	6,08
0,00	0,00	0,00	0,00	0,00
1,94	27,91	0,17	16,54	13,33
0,00	0,32	0,86	10,12	11,27
0,00	0,00	0,29	7,05	10,30
0,00	0,00	0,37	4,24	7,93
0,00	0,36	0,35	6,35	9,28
0,00	0,00	0,12	3,61	5,54
0,00	0,00	0,00	6,30	10,17
0,00	0,41	0,17	2,94	6,74
0,00	0,00	0,16	2,19	5,95
0,00	0,00	0,13	3,03	4,87
0,00	0,00	0,12	1,19	3,34
0,00	0,00	0,00	1,98	6,81
0,00	0,00	0,22	1,57	6,97
0,00	0,00	0,91	2,36	4,53
0,00	0,00	0,00	4,48	6,37
0,00	0,00	0,00	2,64	4,52
0,00	0,00	0,00	4,51	6,14
0,00	0,31	0,34	1,95	6,06
0,00	0,00	0,19	3,33	6,48

0,00	0,00	0,16	5,01	8,72
0,50	0,00	0,31	8,84	11,01
0,00	0,00	0,00	15,71	8,43
0,00	0,00	0,32	4,19	8,14
0,00	0,00	0,73	9,25	12,41
6,18	0,00	0,00	2,16	4,91
1,17	0,00	0,36	5,39	9,34
0,31	0,00	0,00	0,79	3,71
0,00	0,00	0,12	0,59	3,90
0,00	0,00	0,00	1,60	6,23
0,41	0,00	0,00	3,62	6,52
0,00	0,29	1,19	17,28	7,08
0,00	0,00	0,00	1,93	1,93
0,00	0,00	0,15	1,55	3,87
0,00	0,00	0,00	1,41	5,11
0,00	0,00	0,39	2,52	4,26
0,00	0,00	0,00	0,73	2,43
0,00	0,00	0,00	2,29	4,23
0,00	0,00	0,20	3,36	6,13
0,00	0,00	0,14	2,87	4,31
0,00	0,00	0,15	2,71	3,76
0,00	0,00	0,16	0,97	2,92
0,00	0,00	0,16	1,56	5,31
0,00	0,00	0,00	3,59	5,30
0,76	0,00	0,00	4,92	16,24
0,20	1,19	1,71	10,73	6,24
0,00	0,00	0,10	10,43	6,61
1,38	0,35	0,18	9,93	8,65
13,89	63,89	0,00	4,50	18,02
0,71	3,53	1,25	9,48	11,59
0,44	0,44	1,41	11,82	5,97
0,00	0,00	0,67	6,99	4,84
1,28	0,00	0,89	10,73	9,12
0,00	5,91	0,82	12,04	10,26
1,30	2,27	2,23	10,09	6,70
0,00	4,19	1,63	15,10	5,88
0,00	0,00	2,33	9,18	7,14
0,00	0,00	0,84	13,28	7,06
2,54	21,32	1,40	8,79	8,92
0,00	0,00	1,18	13,90	7,66
0,00	0,00	0,51	18,31	5,97
0,00	0,97	0,47	7,34	3,91
0,00	0,00	1,10	14,13	6,06
0,00	0,00	0,83	16,01	5,41
0,00	0,00	0,39	5,21	8,30
0,00	0,00	1,06	2,13	6,38
0,00	0,00	0,00	5,41	10,81
0,00	0,00	0,00	6,12	5,81
0,50	0,00	0,20	3,07	8,38
0,00	0,00	0,00	2,51	3,56

0,00	0,00	0,00	2,65	7,96
0,00	0,00	0,00	2,82	5,63
0,00	0,00	0,00	1,88	3,54
0,00	0,00	0,00	2,73	4,71
0,00	0,00	0,00	0,93	3,02
0,00	0,00	0,00	4,34	5,79
0,00	0,00	0,48	3,84	7,91
0,00	0,00	0,00	0,80	4,23
0,00	0,00	0,18	0,54	5,56
0,00	0,00	0,00	3,94	6,40
0,00	0,00	0,00	6,10	9,86
0,47	0,00	0,00	3,79	4,99
0,00	0,00	0,00	4,20	4,20
0,00	0,00	0,00	3,48	6,76
0,00	0,00	0,41	2,28	5,18
0,00	0,00	0,32	2,25	4,82
0,00	0,00	0,48	8,47	7,99
0,00	0,00	0,33	3,27	3,92
0,00	0,00	0,74	2,94	9,56
0,00	0,00	0,00	1,65	9,09
0,00	0,00	0,00	4,07	8,14
0,00	0,00	0,00	2,68	6,50
0,00	0,00	0,00	0,00	4,65
0,00	0,00	0,00	3,54	4,50
0,45	0,00	0,16	3,46	5,82
0,00	0,00	0,00	1,96	5,69
0,00	0,00	0,00	0,00	3,37
0,00	0,00	0,40	10,74	7,55
0,00	0,00	0,00	5,08	10,17
0,00	0,00	0,00	0,00	7,38
0,00	0,00	0,00	6,15	13,41
0,00	0,00	0,70	10,49	11,89
0,00	0,00	0,45	2,46	6,71
0,00	0,00	0,00	18,75	12,50
0,00	0,00	0,00	2,38	5,71
0,00	0,00	0,00	0,00	0,00
3,74	0,93	0,00	12,40	7,24
1,75	0,00	0,00	0,42	13,98
0,00	0,00	0,38	11,88	4,98
0,00	0,00	0,00	5,01	9,23
0,00	0,00	0,00	3,20	6,13
0,00	0,00	0,00	0,00	0,00
0,00	0,00	0,00	7,38	7,38
0,00	0,00	0,00	9,36	7,43
0,00	0,00	0,51	6,67	16,41
0,00	0,00	0,00	4,88	9,96
0,52	0,00	0,17	4,32	9,47
0,00	0,00	0,17	8,68	7,35
0,00	0,00	0,00	8,59	5,54
0,00	0,00	0,00	5,50	7,97

0,94	1,42	0,84	17,28	6,04
0,00	0,00	0,21	1,27	19,87
0,00	0,00	35,71	71,43	7,14
0,00	0,00	0,00	0,00	12,50
0,00	0,00	0,00	4,46	5,36
0,00	1,72	0,52	3,65	6,77
0,00	0,00	0,63	7,85	7,69
0,00	0,00	0,00	5,25	13,13
0,00	0,00	0,94	7,49	7,87
0,00	0,00	0,27	13,24	6,49
0,00	0,00	0,00	0,00	0,00
0,00	0,00	0,00	7,38	9,29
0,00	0,00	0,00	12,61	6,76
0,00	0,00	0,35	8,57	8,57
0,00	0,00	0,00	10,00	2,50
0,00	0,00	0,13	2,68	5,90
0,00	0,00	0,00	4,27	13,41
0,00	0,00	0,00	0,00	0,00
0,79	0,00	1,75	9,08	7,33
0,00	0,00	0,83	9,68	11,52
0,00	0,00	0,54	9,43	11,05
0,00	0,00	0,61	11,29	10,55
0,00	0,00	0,75	8,21	6,72
0,00	0,00	0,00	7,11	7,11
0,00	0,00	0,48	14,25	6,52
0,00	0,00	0,00	9,30	16,28
0,00	0,00	0,18	1,48	3,60
0,00	0,00	0,35	6,01	7,07
0,00	0,00	0,39	4,33	7,09
0,00	0,00	0,26	7,79	9,61
1,89	0,00	0,00	9,34	7,14
0,00	0,00	1,92	3,85	11,54
0,00	0,00	0,33	8,82	7,35
0,00	0,00	0,00	4,84	12,52
0,92	1,83	0,00	7,52	10,58
0,00	0,00	0,96	8,23	10,01
0,00	0,00	1,43	10,32	8,73
0,00	0,00	0,22	9,33	7,38
0,00	0,00	0,00	9,49	8,23
0,00	0,00	0,00	1,89	5,86
0,00	0,00	0,57	3,61	6,08
0,00	0,00	0,00	12,89	5,30
0,00	0,00	0,00	10,75	13,98
0,00	0,00	0,61	9,84	10,45
0,00	0,00	0,00	3,49	8,14
0,00	0,00	0,00	8,26	9,17
0,00	0,41	0,00	5,24	11,33
0,00	0,00	0,00	6,07	5,67
0,00	0,00	11,11	46,03	45,24
0,00	0,00	0,00	2,72	6,52

0,00	0,00	0,00	1,07	4,27
0,00	0,00	0,00	1,11	4,02
0,43	0,00	0,00	2,66	4,59
0,00	0,00	0,00	6,30	10,69
75,00	12,50	0,00	0,00	1,61
0,00	0,00	0,00	0,00	0,00
0,00	0,00	0,45	17,41	3,68
0,00	0,00	0,00	5,59	6,83
0,00	0,00	0,18	5,81	5,63
0,00	0,00	0,00	13,01	11,05
0,00	0,00	0,59	11,89	11,29
0,00	0,00	0,00	9,74	9,90
0,00	0,00	0,00	0,25	2,02
0,00	0,00	0,46	15,08	2,55
0,00	0,00	1,30	15,58	5,63
0,00	0,00	1,90	12,06	8,89
0,00	0,00	1,54	1,54	10,77
0,00	0,00	0,00	4,55	14,55
0,00	0,92	2,59	10,15	7,13
1,52	0,51	1,40	11,56	7,62
0,00	0,00	1,18	13,24	7,65
0,00	0,00	0,44	8,12	6,20
0,00	0,00	0,63	15,46	13,41
0,00	0,72	0,49	6,33	9,59
1,25	0,63	0,77	7,53	8,88
0,00	0,00	0,00	5,81	10,97
0,00	0,00	0,00	2,02	3,64
0,49	0,00	0,17	5,65	6,31
0,00	0,00	0,29	5,15	6,76
0,00	0,34	0,11	5,01	10,58
1,30	0,00	0,58	5,35	7,37
0,00	0,00	0,00	4,67	6,88
0,00	0,00	0,13	2,83	4,58
0,00	0,00	0,65	3,59	6,21
0,00	0,00	0,14	2,56	4,40
0,43	0,00	0,00	3,02	4,17
0,00	1,74	2,99	13,12	4,82
0,00	0,45	0,00	1,19	2,54
0,00	0,00	0,00	1,59	2,23
0,00	0,00	0,00	0,00	0,00
0,00	0,00	0,00	1,48	4,03
0,00	0,00	0,69	9,79	8,76
0,00	0,00	0,00	2,13	8,88
0,00	0,00	0,00	2,84	6,56
0,00	0,00	0,16	2,81	9,84
2,61	0,00	0,21	9,02	11,71
0,34	0,34	0,50	8,27	9,49
0,00	0,00	0,22	7,34	7,78
0,00	0,00	0,30	11,24	10,34
0,00	4,21	0,47	9,18	12,34

0,00	0,00	0,16	7,87	11,64
0,65	7,79	0,76	13,80	10,21
0,00	0,00	0,00	0,00	0,00
1,20	0,00	0,00	3,13	8,85
0,37	0,00	0,00	1,14	2,92
0,00	0,36	0,00	1,07	2,14
10,26	0,00	0,35	1,77	2,30
10,43	0,00	0,33	4,10	3,93
0,95	0,00	0,28	9,97	8,45
1,05	0,00	0,00	5,74	7,18
1,00	0,00	0,00	4,83	7,25
1,16	0,00	0,00	6,05	7,47
0,00	0,00	0,23	0,34	7,75
0,00	0,00	0,00	3,45	8,09
0,00	0,00	0,00	0,00	3,13
0,00	0,00	0,28	4,58	7,57
0,00	0,00	0,16	2,96	11,70
0,00	0,00	0,13	4,47	10,86
9,52	0,00	0,00	2,38	13,10
0,36	0,00	0,11	1,02	11,11
0,33	0,33	0,42	5,27	10,55
0,00	0,00	0,20	6,48	10,32
0,00	0,00	0,38	4,55	9,60
11,45	0,00	1,25	10,34	9,59
0,00	0,00	0,35	8,48	10,25
0,00	0,00	0,22	7,95	14,13
0,00	0,00	0,00	12,99	9,04
0,00	0,00	0,49	9,62	9,62
0,00	0,00	0,32	4,17	8,35
0,33	0,00	1,11	9,08	14,63
0,00	0,00	0,00	6,70	15,38
0,95	9,52	0,14	5,21	14,33
1,95	0,33	0,19	3,94	11,43
0,00	0,00	1,18	12,94	10,08
0,00	0,00	0,53	8,18	9,89
1,07	0,00	0,00	8,84	8,84
0,00	0,00	0,00	4,07	7,78
0,00	0,00	1,64	11,66	10,02
0,00	0,00	1,85	14,33	11,30
0,00	0,00	0,00	10,79	6,61
0,00	1,15	0,52	6,45	11,85
0,00	0,00	0,32	11,37	5,12
0,00	0,00	0,22	9,10	11,21
0,33	0,33	0,86	7,55	7,17
0,35	0,00	0,33	12,21	9,66
0,83	0,83	0,94	8,49	7,55
0,00	0,00	1,47	13,45	7,14
0,00	0,00	0,00	0,63	3,15
0,00	8,89	0,00	9,83	4,62
0,00	0,00	0,00	0,00	0,00

0,00	0,00	0,19	14,59	9,76
0,00	0,00	0,77	12,31	3,85
0,00	0,00	0,88	10,99	12,53
0,00	0,00	1,05	11,85	9,58
0,00	0,00	1,14	17,49	10,65
0,00	0,00	0,90	11,09	11,09
0,00	0,00	0,32	12,32	11,67
0,00	0,00	0,13	4,60	7,02
0,00	0,00	0,00	3,82	7,52
0,00	0,00	0,00	4,47	5,32
0,00	0,00	0,30	10,94	11,25
0,30	0,00	0,22	4,76	8,19
0,00	0,00	0,12	3,01	7,71
0,00	0,00	0,00	0,00	0,19
0,00	0,00	0,33	6,68	7,65
0,00	0,00	0,00	5,34	5,51
0,00	0,00	0,19	5,29	6,24
0,00	0,00	0,34	3,74	7,94
0,00	0,00	0,66	5,27	7,47
0,00	0,00	0,37	7,82	11,41
0,00	0,00	0,00	1,35	4,40
0,00	0,00	0,00	4,24	6,05
0,00	0,00	0,00	5,12	7,97
0,00	0,00	0,00	3,14	5,10
0,00	0,00	0,00	4,29	7,63
0,00	0,00	3,63	6,30	8,12
0,00	0,00	0,43	10,31	9,23
0,00	0,00	0,22	7,02	10,15
0,00	0,00	0,21	4,53	8,34
0,00	0,00	0,40	5,15	7,58
0,00	0,00	0,18	1,05	2,11
0,00	0,00	0,00	1,71	2,62
0,00	0,00	0,00	7,14	9,18
0,00	0,00	0,00	3,70	6,00
0,00	0,00	0,28	3,13	6,41
0,80	0,00	0,27	5,35	5,35
0,00	0,00	0,39	7,21	9,35
0,00	0,00	0,55	8,06	10,35
0,76	0,00	0,12	10,40	6,74
0,00	0,00	0,00	3,05	6,47
0,00	0,00	0,00	1,86	6,40
0,00	0,00	0,00	2,66	7,32
0,00	0,00	0,00	0,97	1,34
0,00	0,00	0,00	1,04	2,66
0,00	0,00	0,00	0,74	2,85
0,00	0,00	0,19	0,75	1,51
0,00	0,00	0,12	0,23	1,88
0,00	0,00	0,00	0,67	1,11
0,00	0,00	0,00	1,92	4,34
0,00	0,00	0,33	2,48	7,26

0,00	0,34	0,22	3,68	6,58
0,00	0,33	0,10	3,04	7,90
0,00	0,00	0,72	5,30	7,83
0,00	0,00	0,33	5,81	8,77
0,00	0,00	0,00	3,33	10,74
0,00	0,00	0,50	8,66	8,79
0,00	0,00	1,87	10,30	8,05
0,00	0,00	0,20	7,69	12,55
0,79	0,00	0,61	7,39	11,52
0,00	0,00	0,83	13,81	8,82
0,38	0,38	0,12	5,40	9,96
0,00	2,36	0,00	6,18	8,98
0,00	0,00	0,13	2,66	2,54
0,00	0,00	0,00	2,00	3,38
0,00	0,00	0,00	1,37	1,16
0,00	0,00	0,00	0,76	0,88
0,00	0,00	0,27	3,28	5,60
0,38	0,00	0,14	1,85	2,84
0,45	0,45	0,00	2,05	2,37
0,00	0,34	0,11	1,26	4,01
0,00	0,50	0,17	1,16	1,32
0,45	1,35	0,00	0,87	1,89
0,00	0,00	0,00	0,20	2,00
0,00	0,00	0,10	2,89	6,49
0,00	0,00	0,00	1,46	4,25
0,00	0,00	0,43	3,47	5,21
0,00	0,00	0,57	5,13	9,68
0,00	0,00	0,33	9,45	13,19
0,00	0,00	0,00	6,85	6,85
0,00	0,00	0,68	7,86	7,18
0,00	0,00	0,24	4,79	10,18
0,00	0,00	0,72	9,89	9,17
0,00	0,00	0,29	7,46	9,50
0,00	0,69	0,42	6,35	8,78
0,00	0,00	0,16	0,41	1,32
0,19	0,19	0,06	0,76	3,10
0,00	0,00	0,38	8,44	8,82
0,00	0,00	0,21	5,97	5,04
0,00	0,00	0,00	5,67	4,99
0,00	0,00	0,00	1,41	2,54
0,00	0,00	0,21	4,91	5,85
0,00	0,00	0,20	0,79	1,78
0,00	0,00	0,00	0,79	2,17
0,00	0,00	0,00	0,24	2,14
0,00	0,00	0,22	3,97	4,86
0,00	0,00	0,23	3,50	6,42
0,00	0,00	0,00	1,50	3,00
0,38	0,00	0,26	5,32	10,52
0,00	0,42	0,13	4,52	9,97
0,00	0,00	0,39	8,90	11,23

0,00	0,00	0,68	6,49	10,93
0,00	0,00	0,14	8,80	9,22
0,00	0,00	1,25	5,88	16,75
0,00	0,00	1,05	12,43	9,18
0,00	0,00	0,00	5,84	9,73
0,00	0,00	0,20	5,49	6,71
0,00	2,05	0,56	5,82	12,42
0,65	0,00	0,00	7,19	7,78
0,00	1,42	0,73	6,89	13,54
0,00	0,00	0,42	7,66	10,03
0,00	0,48	0,14	5,08	9,04
0,00	0,00	0,31	6,85	7,63
0,00	5,13	0,00	4,51	7,52
0,00	0,00	0,12	4,37	8,40
0,33	0,00	0,58	5,91	6,87
0,00	0,00	0,35	7,77	9,05
0,00	0,00	0,23	1,14	5,71
0,00	0,00	0,64	8,34	15,72
0,00	0,00	0,53	9,03	8,18
1,60	0,32	0,53	7,16	10,47
0,00	0,60	0,89	7,84	8,20
0,00	0,00	0,62	5,71	9,55
0,00	0,00	0,42	6,40	12,10
0,00	0,00	0,47	3,38	5,36
0,00	0,00	0,30	10,24	9,64
0,00	0,00	0,00	1,40	5,29
0,00	0,00	0,16	1,97	6,72
3,92	0,00	0,00	7,32	8,54
0,00	0,00	0,56	14,04	9,55
1,05	0,00	0,96	8,08	9,03
0,00	0,00	1,79	4,25	5,92
0,00	0,34	0,20	7,33	6,53
0,00	0,00	0,46	2,28	5,48
0,00	0,00	0,17	5,75	8,54
0,00	0,00	0,26	4,92	10,10
0,00	0,00	0,23	7,52	11,16
0,00	0,34	0,00	1,88	4,47
0,00	1,33	0,45	4,48	7,17
0,00	0,00	0,32	5,08	8,25
1,00	0,00	0,17	2,54	4,75
0,00	0,00	0,00	0,00	0,00
0,00	0,00	0,25	11,88	5,06
0,00	0,00	0,00	0,00	0,00
0,41	0,00	0,40	5,79	11,04
0,00	0,00	0,71	6,39	7,28
0,00	0,00	0,43	6,43	11,15
0,93	0,00	0,00	0,89	2,33
0,00	0,00	0,00	2,66	2,66
0,00	0,00	0,00	0,56	3,97
0,00	0,00	0,00	3,10	2,33

0,00	0,00	0,00	5,08	6,26
0,00	0,32	0,19	4,98	8,80
0,00	2,27	0,00	7,01	7,64
0,00	0,00	0,47	5,85	10,30
0,00	0,35	0,29	5,37	9,14
0,00	0,00	0,00	3,70	6,34
0,00	0,00	0,21	5,38	9,32
0,00	0,00	0,37	2,61	9,55
0,00	0,00	0,98	8,85	10,57
0,00	0,00	0,43	9,17	9,17
0,00	0,00	0,73	8,06	9,28
0,00	0,00	0,42	6,63	9,59
0,00	0,00	0,42	0,70	1,82
0,00	0,00	0,00	0,61	1,21
0,00	0,00	0,00	6,02	5,50
0,00	0,00	0,00	2,72	4,35
0,00	0,00	0,14	4,60	6,04
0,00	0,00	0,00	1,71	10,26
0,00	0,00	0,00	8,52	6,25
0,00	0,00	0,00	1,06	4,03
0,00	0,00	0,00	1,51	3,40
0,00	0,00	0,23	1,35	2,48
0,00	0,00	0,00	1,85	5,93
0,00	0,00	0,00	2,81	6,74
0,00	0,00	0,00	0,81	3,26
0,00	0,00	0,00	0,94	1,87
3,50	0,00	0,71	11,98	9,99
0,00	0,00	0,42	6,95	7,16
0,00	0,00	0,00	5,06	8,86
0,00	0,00	0,46	4,57	9,59
0,00	0,00	0,54	10,75	17,20
0,35	0,00	0,00	2,35	5,61
0,00	0,00	1,10	4,76	6,59
0,00	0,00	0,00	0,00	0,00
0,00	0,00	0,31	0,94	6,27
0,00	0,00	0,00	4,96	7,71
0,00	0,00	0,00	0,00	5,63
0,00	0,00	0,37	5,49	6,96
0,00	0,00	0,13	0,75	2,51
0,00	0,00	0,00	4,59	8,26
0,00	0,00	0,00	6,56	8,52
0,00	0,00	0,00	4,96	7,44
0,00	0,00	0,00	4,63	2,78
0,00	1,31	0,00	1,37	3,66
0,00	0,00	0,36	2,50	5,36
0,00	0,00	1,36	14,04	8,77
0,00	0,00	0,00	1,00	4,16
0,00	0,00	0,00	0,00	2,36
0,00	0,00	0,00	2,46	5,26
0,00	0,00	0,00	4,46	4,46

0,00	0,00	0,00	0,66	1,32
0,00	0,00	0,19	6,32	6,32
0,00	0,00	0,20	8,59	15,34
0,00	0,00	0,00	5,33	10,00
0,00	0,00	0,70	9,15	11,27
0,00	0,00	0,00	4,23	9,86
0,00	0,00	0,61	3,07	8,59
0,00	0,00	0,65	9,96	9,96
0,00	0,00	0,00	2,21	9,73
0,00	0,00	0,00	4,44	4,44
0,00	0,00	0,00	0,00	20,51
0,00	0,00	0,00	0,00	0,00
0,00	0,00	0,92	8,08	7,85
0,00	0,00	0,37	6,22	7,13
0,00	0,00	0,00	3,13	6,25
0,00	0,00	0,31	5,35	9,61
1,15	0,00	0,40	1,19	3,57
0,00	0,71	2,38	13,85	8,23
0,00	0,00	0,00	0,36	5,02
0,00	0,00	0,00	1,08	3,01
0,00	0,42	0,64	9,40	11,45
0,00	0,00	0,77	7,69	12,69
0,00	0,00	0,18	0,88	1,58
0,00	0,00	0,00	0,00	0,00
0,00	0,00	0,00	0,00	7,50
1,12	0,00	0,00	3,48	3,48
0,00	0,00	0,62	11,23	10,50
0,00	0,00	0,55	10,03	9,75
0,00	0,00	0,68	6,12	12,24
0,00	0,00	0,00	2,74	4,11
0,00	0,00	0,00	3,43	9,07
0,00	0,00	0,16	4,84	14,22
0,00	0,00	0,39	4,27	7,51
0,00	0,00	0,25	5,45	5,45
0,00	0,00	0,00	4,31	4,46
0,00	0,75	0,13	5,01	6,46
0,00	0,00	0,00	3,14	7,47
0,00	0,00	0,00	1,79	6,06
0,00	0,00	0,00	1,85	6,02
0,00	0,00	0,00	0,00	0,00
0,30	0,30	0,21	2,88	4,49
0,00	0,00	0,14	2,31	5,78
0,00	0,00	0,00	2,25	1,95
0,00	0,00	0,39	5,68	5,68
0,00	0,00	0,00	5,26	8,41
0,80	0,40	0,13	5,13	8,38
0,48	0,48	0,16	5,13	6,41
0,00	0,00	0,67	13,39	10,94
0,00	0,00	0,79	9,66	8,60
0,26	0,52	0,00	9,32	8,28

0,00	0,00	0,00	0,00	0,00
0,00	2,78	0,00	4,31	11,21
0,00	0,00	0,21	5,44	7,74
0,52	1,04	0,54	5,94	7,01
0,72	0,00	0,36	7,05	10,27
0,00	0,00	0,57	9,03	8,46
0,42	0,00	0,00	11,72	13,90
0,00	0,00	0,11	5,16	9,77
0,00	0,00	0,17	4,75	7,63
0,00	0,00	0,25	8,49	10,99
0,00	0,00	0,67	8,54	12,06
0,00	0,00	0,34	7,11	13,76
0,00	3,60	0,28	10,42	11,36
0,71	0,00	0,79	9,47	9,70
0,00	0,93	0,30	9,79	13,25
0,32	6,41	0,57	7,82	11,06
0,00	0,56	0,36	3,91	12,99
0,00	0,00	0,41	6,94	12,65
0,40	1,20	0,13	6,17	10,47
1,16	0,00	0,26	7,97	8,76
0,00	0,00	0,50	4,96	4,79
0,00	0,00	0,00	1,60	4,97
0,00	0,00	0,00	1,27	2,69
0,00	1,98	0,12	6,26	5,90
0,00	0,00	0,00	2,72	6,58
0,00	0,00	0,00	2,03	4,93
0,00	0,00	0,00	1,87	5,74
2,23	0,00	0,00	1,57	4,72
0,68	0,00	0,45	2,56	5,35
0,00	0,00	0,40	4,39	5,19
1,93	0,00	0,00	3,24	8,43
0,00	0,00	0,00	4,45	5,26
0,00	0,00	0,00	4,45	5,04
0,00	0,00	0,24	4,51	5,48
0,00	0,00	0,00	0,42	1,47
0,00	0,00	0,18	5,01	6,08
0,00	0,00	0,36	5,10	9,11
0,58	0,00	0,00	4,44	9,48
0,00	0,00	0,00	6,67	7,49
0,00	0,00	0,20	5,53	11,89
0,00	0,00	0,00	2,14	6,95
0,00	0,00	0,19	2,84	7,37
0,00	0,00	0,40	5,53	9,88
0,00	0,00	0,23	3,70	7,39
0,00	0,00	0,54	8,70	4,89
0,00	0,59	0,00	4,13	4,53
0,00	0,00	0,00	1,25	3,27
0,00	0,00	0,00	1,17	2,47
0,00	0,00	0,11	1,49	1,28
0,34	0,00	0,12	1,99	4,73

0,00	0,00	0,00	3,10	3,59
0,00	0,00	0,00	1,52	3,23
0,00	0,00	0,00	2,69	5,75
0,68	0,00	0,61	4,24	7,07
0,00	0,00	0,68	5,81	6,67
1,05	0,00	0,17	5,33	6,17
0,00	0,00	0,31	13,13	9,38
1,28	0,00	0,41	8,13	7,72
0,00	0,00	0,00	5,62	13,41
0,00	0,00	0,10	5,32	6,47
0,00	0,00	0,33	6,07	8,83
0,43	0,00	0,26	8,07	7,94
0,00	0,00	0,14	4,22	11,67
0,00	0,00	0,00	5,68	8,47
0,38	0,00	0,00	1,15	3,44
0,00	0,00	0,10	7,02	10,64
0,00	1,14	0,10	7,55	12,23
0,00	0,00	1,29	9,47	11,86
0,61	0,00	0,48	5,96	10,66
0,00	0,00	0,00	7,34	9,63
2,66	0,00	0,25	7,74	11,11
0,00	0,00	0,76	9,58	10,55
0,00	0,00	0,30	9,47	9,26
0,00	0,00	0,24	6,83	9,02
4,96	0,00	0,12	7,07	10,17
1,29	0,77	0,65	7,43	10,73
0,00	0,83	0,25	7,62	9,61
1,38	0,00	0,67	4,69	14,06
0,00	0,00	0,00	8,09	7,45
0,00	0,00	0,51	8,32	9,85
0,52	0,52	0,15	7,25	11,42
0,38	0,38	0,46	5,86	9,99
0,00	0,00	0,38	4,84	8,63
0,00	0,00	0,00	7,50	10,93
2,88	6,47	0,84	10,88	8,16
0,00	0,75	0,85	11,97	10,04
0,40	0,00	0,52	7,05	6,01
0,00	0,00	0,24	4,31	5,74
0,68	0,00	0,24	4,28	7,36
0,00	0,00	0,00	2,69	7,47
0,00	0,32	0,11	3,68	7,68
0,00	0,00	0,30	2,22	5,91
0,00	0,00	0,12	2,71	7,27
0,00	0,00	0,29	2,57	8,43
0,00	0,00	0,00	2,92	6,40
0,00	0,00	0,00	6,52	5,39
0,00	0,38	0,12	3,78	7,20
0,48	0,00	0,32	4,13	6,19
0,00	0,00	0,56	4,52	5,08
0,00	0,00	0,28	2,62	4,82

0,00	0,00	0,00	2,71	6,32
0,47	0,00	0,33	5,22	8,32
0,00	0,00	0,00	3,39	4,64
0,00	0,00	0,38	2,90	4,66
0,00	0,00	0,98	10,78	9,80
0,00	0,00	0,00	0,00	8,33
0,00	0,00	0,00	6,74	14,61
0,00	0,00	0,46	5,05	14,68
0,00	0,00	0,00	4,69	7,81
0,00	0,00	0,00	0,00	0,00
0,00	0,00	0,00	0,00	0,00
0,00	0,00	0,69	9,66	8,28
0,00	0,00	0,00	0,00	0,00
0,00	0,00	0,98	8,78	7,32
0,00	0,00	1,50	9,30	8,14
0,00	0,00	0,00	12,20	4,88
0,00	55,00	1,45	10,14	11,59
0,00	0,00	0,00	0,00	0,00
0,00	0,00	1,48	10,74	9,63
1,62	0,54	0,58	2,32	9,27
0,00	13,39	1,24	11,69	9,45
0,00	0,00	0,48	1,92	11,06
0,00	0,00	0,20	2,96	5,33
0,00	0,00	0,00	11,71	11,22
0,00	0,00	0,38	10,38	15,00
2,50	1,25	0,70	9,41	9,41
0,00	0,00	0,00	1,44	6,25
0,00	0,00	0,73	1,95	1,46
0,00	0,00	0,25	6,99	12,15
0,00	0,00	0,00	7,27	11,31
0,00	0,00	0,00	4,04	7,07
0,00	0,00	0,00	0,00	2,67
0,00	1,04	0,33	11,15	11,15
0,00	0,00	0,40	8,06	10,08
0,33	0,00	0,42	8,64	8,54
0,00	0,00	0,25	7,63	10,81
0,00	0,00	0,41	7,45	10,21
0,00	0,00	0,00	12,12	17,17
0,00	0,00	0,19	9,11	8,18
0,00	0,00	0,47	7,01	7,48
0,00	0,00	0,44	11,57	7,86
0,00	0,00	0,63	9,49	10,76
0,00	0,00	0,00	0,00	0,00
0,00	0,00	0,88	13,24	5,88
1,45	0,00	0,00	11,11	8,76
0,00	0,61	0,18	8,76	8,93
0,30	0,00	0,09	1,04	6,65
0,00	0,00	0,15	4,58	7,24
0,00	0,00	0,10	5,21	7,61
0,00	0,00	0,00	9,14	8,06

1,23	0,62	0,32	4,65	9,93
0,00	0,00	0,00	0,00	0,00
0,00	0,00	0,50	9,36	11,61
0,00	0,49	0,15	8,36	11,30
0,33	0,00	0,21	8,11	10,12
0,00	0,00	0,10	6,23	10,42
0,00	0,00	0,47	8,41	10,63
0,00	0,00	0,10	7,61	7,91
0,34	0,34	0,86	9,09	9,95
0,00	0,00	0,76	8,71	10,35
0,00	0,00	0,00	7,41	10,24
0,00	0,36	0,12	9,67	9,91
0,00	0,00	0,00	6,08	9,79
1,58	0,79	0,25	9,68	9,93
0,00	7,57	0,21	6,79	8,39
0,00	0,00	0,10	5,35	8,02
0,00	0,00	0,27	8,94	8,27
0,00	0,00	0,33	7,33	9,00
0,00	0,00	0,00	1,72	6,69
0,00	0,00	0,15	4,24	7,72
0,00	0,00	0,00	5,80	7,25
0,00	0,00	0,52	5,08	7,17
0,00	0,00	0,14	6,28	6,42
0,00	0,00	0,00	2,72	7,14
0,00	0,00	0,00	1,77	3,85
0,00	0,00	0,00	1,36	4,48
0,00	0,00	0,00	2,73	1,99
18,18	0,00	0,00	11,11	7,41
2,26	0,00	0,34	4,77	4,60
0,00	0,00	0,00	2,34	5,72
0,00	0,00	0,15	5,35	7,28
0,00	0,00	0,17	3,27	8,78
0,00	0,00	0,00	5,00	10,00
0,00	0,00	0,00	4,30	6,79
0,00	1,15	0,00	5,93	6,67
0,68	0,00	0,47	4,57	7,18
0,00	0,00	0,00	2,97	10,89
0,00	0,00	0,00	8,53	12,13
0,00	0,00	0,67	8,51	9,18
1,21	0,00	0,60	4,22	8,63
0,78	0,78	0,00	4,05	7,49
0,00	0,00	0,00	6,93	12,77
0,23	0,23	0,23	1,31	4,17
0,00	0,00	0,00	6,74	11,51
0,00	0,00	1,05	7,06	8,86
0,00	0,00	0,31	1,85	5,65
0,00	0,00	0,29	1,46	10,06
0,00	0,00	0,17	6,51	10,02
0,34	0,00	0,25	4,57	11,68
0,00	0,52	0,20	2,36	4,32

0,29	0,00	0,00	3,65	7,40
1,04	0,00	0,00	7,08	11,09
0,00	0,27	0,32	8,31	9,44
0,00	0,00	1,07	8,40	7,93
0,00	0,00	0,09	4,18	8,17
0,00	0,00	0,30	7,34	10,87
0,00	0,42	0,13	2,48	5,10
0,00	1,58	0,24	4,77	8,47
0,00	0,00	0,11	3,57	4,86
0,88	0,00	0,09	7,19	9,79
0,00	0,00	0,55	4,48	8,52
0,00	0,00	0,12	5,84	11,68
0,00	0,00	1,20	11,30	10,58
0,00	0,00	0,54	11,40	10,99
0,00	0,00	1,44	13,35	9,65
0,00	0,34	1,92	9,98	13,41
0,00	0,00	1,72	10,75	11,61
0,00	0,00	0,41	5,30	12,91
0,00	9,39	0,00	2,31	10,00
0,00	0,00	0,46	9,38	8,47
0,00	0,00	1,16	7,76	10,87
0,00	0,39	0,51	11,04	10,66
0,44	0,00	0,26	8,08	10,95
0,00	0,48	0,15	12,08	11,78
0,00	0,00	0,00	14,61	7,31
0,00	0,00	0,39	16,34	9,53
0,00	2,56	0,18	7,07	10,69
0,00	0,00	1,09	11,35	10,26
0,00	0,37	0,47	12,12	10,61
0,56	0,00	0,18	10,10	11,60
0,00	0,00	0,69	7,87	12,50
1,37	0,00	0,83	8,56	12,35
1,15	0,00	0,24	7,75	12,75
0,00	0,00	0,40	5,24	9,51
0,00	0,38	0,34	7,22	10,82
0,62	0,00	0,00	8,89	10,28
0,00	0,00	0,71	11,44	10,88
0,39	0,00	0,52	8,72	13,54
0,00	0,00	0,12	5,95	10,39
1,17	0,70	0,29	7,70	10,53
1,94	0,00	0,00	8,86	10,92
0,78	0,39	0,13	3,54	8,86
0,00	0,00	0,43	10,39	11,69
0,00	0,00	0,22	7,03	10,60
0,00	0,00	0,42	8,90	9,11
0,25	0,00	0,14	6,29	10,40
0,28	0,83	0,33	6,94	9,80
0,00	0,48	0,48	7,00	10,70
0,00	0,95	0,10	5,20	10,99
0,00	0,44	0,40	6,99	11,21

0,00	0,00	0,17	7,61	11,00
0,00	0,00	0,65	5,93	10,90
0,00	0,44	0,63	7,74	11,68
0,47	0,00	0,00	7,23	11,06
0,38	0,00	0,13	6,99	14,36
0,39	0,39	0,26	8,00	10,97
0,35	0,35	0,11	5,93	9,81
0,30	0,00	0,18	8,13	8,13
0,33	0,00	0,11	7,17	11,06
0,68	0,00	0,00	7,09	13,18
0,37	0,00	0,99	7,18	8,40
0,30	0,00	0,20	8,88	10,37
0,00	0,00	0,32	4,52	5,81
0,33	0,33	0,00	1,54	8,18
0,00	0,00	0,11	2,76	4,86
0,00	0,00	0,09	2,08	5,09
0,34	0,00	0,11	2,69	5,84
0,00	0,00	0,00	3,12	6,86
0,00	0,00	0,38	3,03	6,06
0,00	0,00	0,00	2,00	6,17
0,00	0,00	0,00	1,46	4,48
0,00	0,00	0,00	1,95	3,91
0,00	0,00	0,00	2,41	6,94
0,00	0,00	0,00	2,56	4,23
0,64	0,00	0,00	5,23	10,23
0,00	0,00	0,31	7,39	8,49
0,00	0,00	0,00	4,45	6,68
0,00	0,00	0,34	10,44	10,77
0,00	0,63	0,00	8,56	9,32
1,62	0,00	0,16	9,80	11,04
0,00	0,00	2,63	9,21	14,47
0,00	0,00	0,00	0,00	0,00
2,94	0,00	2,61	3,48	3,48
0,00	0,00	0,93	9,77	8,84
0,00	0,00	1,53	12,21	10,69
0,00	0,00	4,17	16,67	0,00
0,00	0,00	1,76	11,62	5,28
0,98	2,62	0,62	6,67	11,50
0,34	0,00	0,89	9,98	10,42
1,41	0,00	0,33	12,27	11,17
0,78	1,56	0,49	8,15	10,95
0,00	1,38	0,66	8,51	10,51
0,00	0,40	0,25	9,85	9,85
0,49	0,00	0,30	7,70	10,67
0,00	2,52	0,95	12,17	8,59
0,30	0,30	1,75	8,67	9,78
0,00	0,00	0,72	8,99	12,95
0,00	0,00	0,66	12,46	10,82
0,00	3,40	0,39	11,50	9,75
4,35	0,72	0,67	12,95	10,94

0,00	0,00	0,24	10,58	9,98
0,00	2,70	0,80	5,45	8,81
0,00	0,00	0,00	0,00	0,00
0,54	0,00	0,17	3,89	5,50
0,00	0,00	0,00	5,71	8,79
0,30	0,00	0,18	5,45	7,45
10,81	0,00	0,00	4,08	15,41
1,28	0,00	0,22	8,79	13,85
0,00	0,00	0,00	9,52	13,90
2,26	0,00	0,00	9,35	9,93
0,00	1,53	0,12	7,20	8,50
0,00	0,00	0,14	7,30	8,92
0,00	0,00	0,00	15,07	5,78
0,00	0,00	0,29	12,08	9,90
0,00	0,00	0,78	11,18	10,08
4,29	0,00	1,51	8,69	11,94
0,00	0,00	0,12	4,30	9,71
0,00	0,00	0,18	6,09	11,62
0,00	0,00	0,11	2,07	7,10
0,00	0,00	0,12	6,99	10,43
0,00	1,32	0,00	6,43	7,88
0,00	0,00	0,00	4,63	6,53
0,40	0,00	0,00	4,56	6,96
0,00	0,00	1,82	13,01	8,77
0,00	0,00	1,43	15,14	8,57
0,85	0,43	1,36	14,93	9,23
0,34	0,00	1,77	11,21	7,93
0,00	7,63	0,12	8,53	8,41
0,00	0,00	0,00	7,99	7,43
0,00	0,00	0,68	5,71	9,02
0,00	0,00	0,29	8,12	11,16
0,00	0,00	0,15	7,25	11,33
0,00	0,87	0,43	8,42	10,70
0,00	0,00	0,25	8,59	8,84
0,00	1,07	0,73	6,92	9,47
1,98	0,00	0,00	6,51	10,85
0,00	0,00	0,68	8,19	11,26
0,00	0,00	0,67	7,83	10,26
0,00	0,00	0,00	6,99	10,12
0,00	0,00	0,98	6,86	11,76
0,00	0,00	0,00	10,26	8,33
0,00	0,00	0,50	6,72	10,32
0,00	0,00	0,49	8,67	11,11
0,37	1,48	0,60	10,01	8,58
0,00	0,48	0,16	8,29	11,16
0,00	0,00	1,20	10,79	6,24
0,00	0,00	0,00	14,29	10,20
0,00	0,00	2,11	12,41	9,19
0,00	0,40	0,56	13,90	8,74
0,00	0,00	0,00	12,32	11,48

0,00	0,00	0,14	7,97	12,23
0,00	0,00	0,63	5,78	12,03
0,00	0,00	0,27	8,18	9,65
0,00	0,00	0,00	6,34	9,61
0,00	0,00	0,41	11,39	3,93
0,48	0,00	0,67	11,87	4,53
0,00	0,43	0,26	8,17	5,62
0,57	0,00	0,51	11,97	7,69
0,00	0,00	1,96	11,63	8,32
0,00	0,00	0,40	6,45	10,08
0,76	0,00	1,14	11,64	11,42
0,00	0,00	0,22	8,67	10,00
0,00	0,00	0,26	4,48	8,56
0,67	0,00	0,11	6,49	8,69
0,00	0,00	0,14	6,12	11,13
0,00	0,00	0,26	6,20	8,27
0,00	0,00	0,25	7,25	9,00
0,46	0,00	0,00	4,92	10,63
0,00	0,00	0,17	6,23	11,59
0,00	0,00	0,00	7,00	11,98
0,00	0,00	0,43	8,62	5,17
0,00	0,00	0,19	7,75	11,91
0,00	0,00	0,18	2,87	8,62
0,00	0,00	0,00	2,70	7,19
0,00	0,00	0,27	5,61	7,08
0,00	1,97	0,45	3,56	5,79
0,41	0,00	0,49	3,25	5,85
0,00	0,47	0,00	2,77	9,78
0,68	0,00	0,00	7,32	11,36
0,00	0,00	0,00	6,04	5,45
0,00	0,00	0,31	5,19	7,33
0,00	0,00	0,44	5,16	6,34
0,00	0,00	0,00	5,46	4,64
0,00	0,00	0,00	2,73	4,75
0,00	0,00	0,16	6,26	9,95
0,00	0,00	0,07	8,99	8,84
0,00	0,00	0,00	5,39	4,55
0,00	0,00	0,00	2,99	6,13
0,00	0,00	0,40	2,28	4,44
0,00	0,00	0,00	2,33	6,20
0,00	0,00	0,00	3,70	11,11
0,00	0,00	0,00	7,69	8,79
0,00	0,00	0,00	0,00	7,50
0,00	0,00	0,26	1,53	2,04
0,00	0,00	0,18	2,84	3,19
0,00	0,00	0,31	4,37	8,89
0,00	0,00	0,37	3,55	5,42
0,00	0,00	0,00	3,94	5,07
0,00	0,00	0,17	4,19	8,04
0,00	0,00	0,00	0,38	9,23

0,00	0,00	0,00	2,26	9,02
0,00	0,00	0,34	2,07	3,10
0,00	0,00	0,00	0,00	0,00
0,00	0,00	0,00	3,76	5,26
0,00	0,00	0,00	6,74	8,99
1,02	22,45	0,70	5,57	8,01
0,00	0,00	0,00	3,96	7,26
0,00	0,00	0,00	8,33	6,94
0,00	0,00	3,13	3,13	15,63
0,00	0,00	0,00	4,17	20,83
0,00	0,00	0,00	9,66	11,03
0,00	0,00	0,68	9,32	9,77
0,00	0,00	0,00	2,31	6,76
0,00	0,00	0,00	2,63	5,26
0,00	0,00	0,56	6,42	5,31
0,00	0,00	0,00	2,11	3,38
0,00	0,00	0,70	8,45	4,93
0,00	0,00	0,00	6,85	5,24
0,00	0,00	0,37	4,43	4,06
0,00	0,00	0,00	1,47	20,59
0,00	0,00	0,00	13,89	5,56
0,00	0,00	0,00	3,13	4,17
0,00	0,00	0,00	4,43	10,76
1,56	0,00	0,55	12,57	12,02
0,00	0,00	0,36	2,50	7,32
0,00	0,00	0,00	1,18	4,71
0,00	0,00	0,00	6,83	4,68
0,00	0,00	0,00	11,27	11,27
0,00	0,00	0,00	14,29	10,79
0,00	0,00	0,00	11,54	5,13
0,00	0,00	0,00	3,51	5,51
0,00	0,46	0,14	6,34	11,96
0,00	0,00	0,00	6,33	10,76
0,00	0,00	0,00	6,58	11,84
0,00	0,00	0,00	7,81	6,25
0,00	0,00	0,00	7,45	11,70
0,00	0,00	0,00	12,90	9,68
0,00	0,00	0,00	5,38	6,45
0,00	0,00	0,00	8,13	10,57
0,00	0,00	0,49	8,37	10,84
0,00	0,00	0,00	7,43	8,78
0,00	0,00	0,00	1,20	4,35
1,00	0,00	0,16	2,79	6,82
0,00	0,00	0,28	5,34	4,49
0,69	0,00	1,04	9,75	8,09
0,00	0,00	0,23	7,98	12,21
0,00	0,00	0,00	9,09	13,64
0,00	0,00	0,00	3,28	13,11
0,00	0,00	0,22	7,11	10,89
0,00	0,00	0,33	8,36	6,89

0,00	0,00	1,37	4,11	10,96
0,00	0,00	1,10	8,79	7,69
0,00	0,69	0,94	6,56	7,26
0,00	0,00	0,80	4,00	5,60
0,00	0,00	0,19	4,06	8,12
0,00	0,00	0,00	1,67	4,18
0,00	0,00	0,00	1,42	2,83
0,00	0,00	0,00	4,48	2,99
0,00	0,00	0,00	6,22	12,03
0,00	0,00	0,00	0,00	16,67
0,00	0,39	0,49	9,56	11,52
0,00	0,00	0,73	6,00	7,64
0,00	0,00	0,85	6,81	11,91
0,00	0,00	0,21	2,31	7,98
0,00	0,00	0,21	6,40	5,76
0,00	0,00	0,00	6,47	9,48
0,42	0,00	0,26	6,09	8,34
0,00	0,00	0,00	6,35	7,14
0,00	0,00	0,84	11,11	10,48
0,00	0,00	0,00	13,33	4,44
0,00	0,00	0,62	2,47	12,73
0,00	0,00	0,18	3,70	10,23
0,00	0,00	0,00	0,00	0,00
0,00	0,00	0,13	3,88	8,56
0,00	0,00	0,00	3,00	5,88
0,00	0,00	0,00	1,36	7,80
5,88	0,00	0,00	12,50	8,93
0,00	0,53	0,43	5,67	7,66
0,00	0,00	0,40	1,21	3,64
0,00	0,00	0,00	0,00	0,00
0,00	0,00	0,34	13,76	9,73
0,00	0,00	0,66	7,28	14,57
0,00	0,00	0,00	0,00	0,00
0,00	0,00	0,52	8,90	13,61
0,00	0,00	0,91	5,34	10,21
0,00	0,00	2,68	8,12	6,92
0,00	0,00	0,00	3,70	14,81
0,00	0,00	0,35	7,69	16,78
2,41	0,00	0,68	7,43	7,43
3,92	0,00	0,00	8,84	10,88
0,00	0,00	0,00	8,57	12,14
1,52	0,00	2,19	12,72	8,33
0,00	4,55	0,00	15,58	7,79
0,00	0,00	1,85	10,19	12,04
0,00	0,00	0,00	16,35	5,77
0,00	0,00	0,59	6,49	8,55
1,10	0,00	0,77	12,74	10,42
0,00	0,00	0,00	11,76	10,59
0,00	0,00	0,00	6,54	14,95
0,00	0,00	0,00	3,18	10,19

0,00	0,00	0,00	0,00	26,67
0,00	0,00	0,00	10,86	7,43
0,00	0,00	0,28	1,70	4,83
0,00	0,00	0,00	2,70	1,80
0,00	0,00	0,00	8,41	14,95
0,00	0,00	0,00	9,03	6,94
0,00	0,00	0,00	5,54	12,62
0,87	0,00	0,28	6,91	6,35
0,00	1,69	0,26	7,46	12,34
0,00	0,50	0,45	6,90	10,34
0,00	0,00	1,04	12,50	5,21
0,00	0,00	0,00	6,98	6,98
0,00	0,00	0,00	5,36	10,71
0,00	0,00	2,78	12,50	15,28
0,00	0,00	1,84	8,59	4,60
1,56	0,00	0,88	9,25	11,89
0,00	0,00	0,00	6,90	17,24
0,00	0,00	0,47	15,64	8,06
0,00	0,00	1,65	9,89	7,14
0,00	0,00	0,00	4,65	9,30
9,71	0,97	0,28	7,87	6,46
3,57	8,93	0,00	10,34	9,85
0,00	0,00	0,00	0,00	0,00
0,00	0,00	0,00	2,07	1,38
0,00	0,00	0,00	10,34	9,36
0,00	0,00	1,19	7,14	13,10
0,93	0,00	1,30	7,82	10,42
0,00	0,00	0,56	8,43	13,20
0,00	0,00	0,00	6,03	12,06
0,00	0,00	0,00	13,33	0,00
0,00	0,00	0,44	10,18	7,52
0,00	5,00	0,00	10,08	10,08
0,00	0,00	0,00	0,00	0,00
0,00	0,00	0,00	9,16	14,50
0,00	0,00	1,72	13,79	1,72
0,00	0,00	0,00	16,82	6,54
0,00	0,00	0,00	3,03	12,12
0,00	0,00	0,49	5,88	8,33
0,00	0,00	0,00	3,21	5,77
0,00	1,97	0,23	3,04	7,71
0,56	0,00	0,19	1,91	3,63
0,00	0,00	0,24	1,68	4,56
0,00	0,00	0,00	2,62	2,33
0,00	0,00	0,00	3,57	14,29
0,00	0,00	0,00	10,26	10,26
0,00	0,00	0,21	2,29	7,48
0,00	5,68	0,27	5,62	7,13
2,63	0,00	0,88	6,19	11,95
0,00	0,00	0,25	7,70	5,73
0,00	0,00	0,53	6,10	7,49

0,00	0,00	0,00	0,00	0,00
0,00	0,00	0,00	5,61	7,14
0,98	0,00	0,32	9,89	7,13
0,00	0,00	0,69	7,61	8,82
0,00	0,00	0,11	7,99	12,63
0,00	0,00	0,00	7,02	11,18
0,00	0,00	0,32	6,35	9,84
0,00	0,00	0,26	8,66	7,49
0,00	0,00	0,22	6,18	9,60
0,00	0,00	0,25	4,46	10,64
0,00	0,00	0,08	2,00	4,55
0,00	0,00	0,28	4,25	6,37
1,82	0,52	0,08	5,73	8,34
0,00	0,00	0,14	6,82	10,37
0,00	0,00	0,29	5,23	8,82
0,00	0,00	0,46	5,56	13,43
0,00	0,00	0,00	3,89	5,70
0,79	0,26	0,00	2,62	8,82
0,00	0,28	0,17	6,95	8,39
1,71	0,00	0,10	0,59	4,59
0,00	0,00	0,55	4,25	8,29
0,35	0,00	0,00	4,51	9,99
0,00	0,00	0,51	3,81	6,85
0,00	0,00	0,19	4,29	6,90
0,27	0,00	1,17	8,22	7,59
0,00	0,00	0,39	10,07	13,56
0,00	0,00	0,99	11,30	11,44
0,00	0,00	0,33	10,29	13,27
1,15	0,00	0,24	12,21	11,84
1,63	0,00	0,54	6,57	11,30
0,42	5,83	0,89	8,17	9,07
6,19	0,00	1,01	8,94	10,96
0,00	0,00	0,45	10,24	4,90
0,00	0,00	0,26	6,16	6,68
0,00	0,00	0,00	4,43	7,49
0,51	0,00	0,16	4,87	5,19
0,87	0,00	0,16	4,60	8,10
0,00	0,00	0,26	7,91	8,67
0,00	0,00	0,98	16,12	5,70
0,00	0,00	0,00	8,46	10,45
0,00	0,00	0,62	15,79	6,97
0,00	0,00	1,60	11,57	6,58
0,00	0,00	0,28	13,60	7,37
0,00	0,00	0,65	12,84	8,12
0,00	0,00	1,81	16,27	4,82
0,32	0,32	0,60	12,31	8,46
0,00	0,00	0,32	6,66	11,53
0,00	1,39	0,38	8,86	7,26
0,00	0,00	0,00	1,30	3,90
0,00	0,00	0,19	1,14	2,10

0,00	0,00	0,00	1,44	6,14
0,00	0,00	0,00	0,97	2,92
1,12	0,56	0,00	5,32	6,97
0,00	0,00	0,10	3,10	5,88
0,00	0,00	0,00	2,03	5,95
0,00	0,00	0,00	2,14	6,09
0,00	0,00	0,00	2,25	6,93
0,00	0,00	0,28	5,96	8,73
2,35	0,00	0,14	5,56	9,60
0,00	0,00	0,00	7,84	7,25
0,00	0,00	0,00	3,61	9,27
0,00	0,00	0,00	3,38	5,81
0,00	0,00	0,27	4,81	6,95
0,48	0,00	0,82	8,24	8,38
0,00	0,00	0,71	13,10	10,97
0,00	0,00	0,58	13,01	11,95
0,00	0,57	0,18	11,35	11,89
0,45	0,00	0,62	9,67	13,88
0,00	0,32	0,20	10,96	12,52
0,00	0,00	1,14	10,77	9,01
0,00	0,00	0,42	10,57	11,42
0,00	0,00	1,30	11,26	13,03
0,00	0,30	0,25	14,13	8,95
0,40	0,40	0,12	3,48	6,95
2,11	2,11	0,00	4,40	5,66
0,00	0,00	0,23	3,74	6,78
0,00	0,00	0,79	4,87	8,29
0,00	0,00	0,78	3,12	6,23
0,00	0,00	0,29	3,98	5,31
0,00	0,00	0,13	5,92	7,30
1,17	0,00	0,11	4,99	7,77
0,00	0,00	0,00	2,16	4,20
0,00	0,00	0,32	2,53	6,33
0,00	1,54	0,00	0,76	4,35
0,00	0,00	0,52	5,37	7,22
0,00	0,51	0,63	3,65	7,14
0,00	0,54	0,00	9,29	9,64
0,00	0,00	0,27	11,32	10,24
1,09	0,00	0,00	1,22	4,43
0,00	0,00	0,00	2,14	4,05
0,00	0,00	0,13	1,80	5,53
0,00	0,00	0,10	1,75	3,70
0,00	0,00	0,00	0,49	0,33
0,00	0,00	0,00	3,79	4,11
0,00	0,00	0,00	0,00	2,79
0,00	0,00	0,00	0,15	0,91
2,66	0,33	0,00	0,22	1,33
0,00	0,00	0,00	0,00	1,19
0,00	0,00	0,10	0,41	1,13
0,00	0,00	0,00	0,86	3,00

0,70	0,00	0,00	0,33	0,66
1,12	0,00	0,17	7,02	10,90
0,00	0,46	0,14	8,34	9,99
0,32	0,00	0,31	6,59	9,17
0,00	0,00	0,51	8,19	8,58
0,49	0,00	0,00	5,63	9,59
0,87	2,17	0,14	4,73	11,82
0,00	2,30	0,00	7,52	6,24
0,53	0,00	0,34	4,63	6,99
0,00	0,00	0,00	7,27	12,21
0,00	0,00	0,00	7,65	10,87
0,00	0,00	0,97	8,16	7,77
0,00	0,00	0,20	6,20	14,33
0,00	0,31	1,01	12,18	8,74
0,00	0,00	0,59	11,29	11,88
0,00	0,00	1,17	9,96	9,96
0,00	0,00	0,51	18,00	7,47
0,00	0,00	0,28	9,80	14,01
0,00	0,00	0,00	9,09	9,09
0,00	0,00	0,22	17,07	7,22
0,00	0,00	0,95	9,46	14,83
0,00	0,00	0,36	14,08	8,30
0,00	0,00	0,13	11,23	12,14
0,00	0,00	0,81	11,05	11,99
0,00	0,00	0,00	14,22	12,80
0,43	0,00	3,30	10,58	8,93
0,00	0,00	0,99	14,00	9,27
0,68	0,00	1,15	8,80	12,24
0,00	0,00	0,91	16,55	7,48
0,70	0,00	0,44	13,75	10,42
0,36	0,00	0,45	16,91	8,46
0,00	0,00	0,60	16,67	12,50
0,98	0,98	0,63	10,09	11,67
0,00	0,00	1,40	12,29	9,50
3,11	2,48	0,55	14,05	8,87
0,61	1,21	1,14	10,23	10,80
0,00	0,00	0,44	11,40	10,75
0,00	0,00	0,49	9,91	10,40
0,98	0,00	0,30	11,82	10,61
0,31	0,00	0,00	5,61	8,92
0,00	0,00	0,74	4,71	11,66
0,00	0,53	0,21	3,98	10,06
0,00	0,00	0,30	3,50	9,51
0,00	0,00	0,00	2,38	5,71
0,00	0,00	0,00	4,66	7,25
0,00	0,00	0,13	2,15	5,23
0,00	0,00	0,21	0,64	3,95
0,00	0,00	0,00	2,09	8,94
1,09	0,54	0,15	7,87	7,87
0,39	0,00	0,00	3,23	4,49

1,27	0,00	0,17	3,65	4,49
0,00	0,00	0,92	16,54	7,66
0,00	0,46	0,24	4,47	5,10
0,00	0,00	0,00	1,89	4,98
0,00	0,00	0,34	5,15	6,52
0,68	0,00	0,00	1,70	4,83
0,83	0,00	0,00	3,23	3,23
0,00	0,00	0,00	0,57	3,40
0,00	0,00	0,21	5,12	7,25
0,00	0,00	0,00	1,93	2,79
0,00	0,00	0,84	15,63	5,65
2,50	3,33	1,07	8,18	10,86
0,42	0,00	0,51	8,93	8,80
0,00	0,00	0,00	14,38	9,38
0,00	0,00	0,39	10,79	7,67
0,90	0,00	2,11	12,44	8,69
0,00	8,08	0,00	6,21	16,46
0,00	0,00	2,43	8,80	11,42
0,00	0,00	0,40	14,52	14,11
0,00	0,00	0,00	0,00	0,00
0,00	0,77	0,00	0,73	1,79
0,17	0,00	0,12	1,11	2,85
0,00	0,00	0,17	2,34	2,59
0,00	0,00	0,12	0,46	2,88
0,00	0,00	0,10	1,83	2,12
0,00	0,00	0,10	1,23	2,46
0,19	0,00	0,27	0,81	1,29
0,00	0,00	0,31	1,09	1,87
0,00	0,00	0,00	0,60	1,81
0,35	2,12	0,00	6,11	14,07
0,00	0,00	0,00	0,00	3,19
0,00	0,00	0,69	10,84	11,30
0,00	0,00	0,00	6,17	10,80
0,00	0,00	0,62	9,81	12,60
0,00	0,00	0,49	11,36	12,99
0,00	0,00	0,00	6,61	10,96
0,49	0,00	0,57	7,01	11,55
0,00	0,00	0,00	0,00	0,00
0,33	0,00	0,53	13,16	11,02
0,60	0,00	0,39	13,10	10,79
0,30	0,00	0,66	10,66	12,45
0,00	0,00	0,00	4,63	10,98
0,00	0,00	0,00	5,78	8,23
0,00	0,00	0,19	3,27	8,08
0,43	0,00	0,54	8,48	14,00
0,00	0,79	1,42	4,83	12,78
0,37	0,00	0,37	3,21	8,15
0,00	0,00	0,28	4,05	5,73
0,34	0,00	0,00	2,10	4,78
0,00	0,00	0,00	3,09	4,17

0,33	0,00	0,11	3,67	6,42
0,00	0,35	0,14	2,34	3,31
0,00	0,00	0,40	2,37	4,15
0,00	0,00	0,30	1,05	4,20
0,00	0,00	0,37	2,20	4,40
0,00	0,00	0,00	2,01	7,37
0,24	0,00	0,36	3,96	5,05
0,00	0,00	0,00	5,15	6,60
0,00	0,00	0,00	7,69	9,62
0,00	0,00	1,12	10,49	5,62
0,00	0,00	0,42	12,41	9,03
0,00	0,00	0,39	11,58	11,90
0,00	0,00	0,00	5,97	8,18
0,00	0,36	0,44	4,98	9,41
0,00	0,00	0,00	5,66	12,26
0,00	0,00	0,58	10,66	8,53
0,00	0,00	1,76	15,85	9,86
0,00	0,00	1,05	9,90	12,34
0,00	4,76	2,88	6,47	9,35
0,00	0,00	0,00	2,41	5,72
0,00	0,00	0,26	2,42	4,59
0,00	0,00	0,00	3,28	4,63
0,00	0,00	0,12	6,24	7,43
0,29	0,00	0,10	3,46	6,12
0,00	0,00	0,23	2,63	5,14
0,00	0,00	0,12	1,75	3,97
0,00	0,27	0,10	2,31	4,14
0,00	0,00	0,00	1,50	6,00
0,00	0,00	0,39	2,89	6,82
0,00	0,00	0,46	1,99	5,82
0,00	0,00	0,74	1,70	2,77
0,00	0,00	0,00	1,54	6,48
0,00	0,00	0,12	5,66	10,74
0,00	0,00	0,34	11,13	10,46
0,00	0,00	0,52	11,69	9,35
0,00	0,00	0,36	13,26	12,19
0,00	0,00	0,54	10,57	5,69
0,00	0,00	0,71	7,33	7,57
0,00	0,00	0,00	2,62	3,77
0,00	0,00	0,00	3,10	6,90
0,00	0,00	0,00	2,08	4,30
0,30	0,00	0,00	2,19	4,18
0,56	0,00	0,00	1,87	2,27
0,00	0,00	0,11	0,78	2,68
0,00	0,00	0,00	3,05	4,26
0,00	0,00	0,13	3,50	5,25
0,00	0,39	0,00	2,18	3,19
0,28	0,00	0,00	2,16	4,31
0,41	0,00	0,15	1,51	2,26
0,00	0,00	0,28	1,42	3,68

0,00	0,00	0,00	1,06	1,73
0,00	0,39	0,00	0,82	4,50
0,00	0,00	0,14	1,26	2,65
0,00	0,31	0,00	0,86	2,78
0,00	0,00	0,00	1,87	2,41
0,00	0,00	1,71	8,29	8,00
0,00	0,00	0,30	12,22	10,26
0,00	0,00	0,00	9,95	11,54
0,00	0,00	0,20	11,07	13,11
0,00	0,00	0,38	14,25	9,71
0,00	0,00	1,64	14,75	9,51
0,00	0,00	1,52	13,01	8,45
0,00	0,42	0,17	3,72	5,91
0,00	0,00	0,00	2,51	6,28
0,83	0,00	0,21	2,18	3,53
0,00	0,00	0,00	2,90	3,26
0,00	0,00	0,00	2,81	4,14
0,00	0,00	0,00	1,37	2,52
0,00	0,00	0,00	0,90	3,59
0,00	0,00	0,00	0,62	2,47
0,00	0,00	0,00	0,24	0,86
0,00	0,00	0,21	3,01	7,95
0,00	0,00	0,00	1,86	4,41
0,00	0,00	0,00	0,72	3,28
0,47	0,00	0,00	1,65	2,97
0,00	0,00	0,00	0,97	3,87
0,00	0,00	0,00	2,43	2,43
0,00	0,00	0,18	1,66	3,50
0,34	0,00	0,13	1,82	2,46
0,00	0,00	0,00	0,95	1,58
0,00	0,00	0,15	0,73	3,22
0,00	0,00	0,00	0,90	1,43
0,00	0,00	0,00	0,35	1,06
0,00	0,00	0,26	1,71	1,58
0,00	0,00	0,46	0,00	1,38
0,00	0,00	0,00	12,61	10,08
0,00	0,00	0,45	0,67	2,01
0,00	3,08	0,16	1,11	1,35
0,00	0,00	0,00	7,66	8,72
0,00	0,00	0,21	12,89	9,15
0,00	0,00	0,00	3,09	5,93
1,27	0,00	0,00	2,02	7,32
0,00	0,00	0,14	1,50	4,51
0,00	0,00	0,00	2,89	4,93
0,00	0,00	0,00	2,13	2,76
0,00	0,65	0,33	0,77	3,31
0,00	0,00	0,57	1,14	3,71
0,00	0,00	0,00	1,74	4,35
0,00	0,00	0,00	1,00	2,67
0,00	0,00	0,00	1,30	2,04

0,00	0,00	0,15	1,22	2,45
0,00	0,00	0,00	2,26	3,06
0,00	0,00	0,00	0,59	2,34
0,00	0,00	0,13	0,79	2,36
0,00	0,00	0,13	2,30	2,68
0,00	0,00	0,00	0,31	0,61
0,00	0,00	0,00	0,90	2,02
0,00	0,00	0,00	0,52	1,86
0,00	0,28	0,28	0,85	1,70
0,00	0,00	0,00	1,06	2,13
0,00	0,00	0,00	1,37	2,47
0,00	0,00	0,00	0,75	0,75
0,00	0,00	0,00	1,52	3,27
0,00	0,00	0,34	5,90	11,13
0,00	0,00	0,00	8,94	13,01
0,00	0,00	0,25	3,93	10,32
0,00	0,00	0,54	11,83	8,06
0,98	0,00	1,23	11,11	11,11
0,00	0,00	0,40	8,57	11,75
1,20	0,00	0,77	8,08	9,23
0,00	2,27	0,00	12,69	9,60
0,00	0,00	2,78	1,39	5,56
0,00	0,00	0,49	12,68	9,27
0,00	1,09	1,07	10,19	9,38
1,00	0,00	0,42	5,02	9,21
0,00	0,00	1,06	6,38	12,77
0,00	0,00	0,00	0,00	0,00
0,00	0,00	0,12	3,64	9,10
0,00	0,00	0,86	8,93	9,51
0,00	0,00	0,24	9,84	10,40
0,00	0,00	0,40	6,16	9,63
0,00	0,00	0,00	6,67	9,26
0,00	0,00	0,00	9,07	7,14
0,00	0,00	0,00	1,85	6,79
0,00	1,39	0,00	2,88	5,76
0,00	0,00	0,94	11,79	9,91
0,00	0,00	1,06	7,45	14,89
0,00	0,00	0,29	10,59	11,73
0,00	0,00	0,24	8,79	7,13
0,65	1,29	0,69	11,74	8,46
0,00	0,00	0,00	8,75	8,75
0,00	0,00	0,00	11,36	4,55
0,00	0,00	0,00	12,94	8,24
0,00	0,00	0,00	1,42	3,13
0,00	1,33	0,00	5,64	11,69
0,00	0,00	0,49	7,77	5,83
0,00	0,00	0,34	3,03	9,09
0,00	0,00	0,35	3,32	15,18
0,48	0,48	0,00	4,63	9,39
0,00	0,00	0,23	2,89	7,34

0,93	0,00	0,24	3,03	5,33
0,00	4,00	0,00	4,35	1,09
0,00	0,00	0,00	15,38	12,82
0,00	0,00	0,41	1,65	4,94
0,00	0,00	0,00	1,48	3,85
0,00	0,00	0,00	1,01	1,69
0,00	0,00	0,00	0,00	0,00
0,00	0,00	0,19	8,83	9,21
0,00	0,00	0,00	11,58	7,37
0,00	0,00	0,63	1,90	1,90
0,00	0,00	0,37	11,44	12,55
0,43	6,81	0,14	5,92	7,95
0,00	17,14	0,84	8,40	7,56
3,70	0,00	0,00	5,26	13,68
0,00	0,00	0,47	8,14	8,84
1,46	1,95	0,00	0,30	1,67
0,00	0,00	0,00	0,77	0,77
0,00	0,00	1,36	16,33	8,84
1,33	0,00	0,47	5,87	7,51
0,00	0,00	0,00	4,65	8,91
0,00	0,00	0,00	4,08	10,88
0,00	0,00	0,00	17,95	2,56
0,00	0,00	1,69	11,44	11,44
0,00	0,00	0,00	4,21	1,05
0,00	0,00	0,00	0,00	0,00
0,00	0,00	2,54	10,17	7,63
1,44	3,60	0,25	3,50	7,00
0,00	0,00	0,00	11,11	7,94
0,00	0,00	2,78	16,67	2,78
0,00	0,00	1,26	7,04	12,31
0,00	4,92	0,65	11,75	12,08
0,00	0,00	0,00	4,92	14,75
0,00	0,00	0,00	6,25	0,00
0,42	0,00	0,00	0,66	1,48
0,00	0,00	0,18	0,91	1,81
0,00	0,00	0,26	1,03	2,06
0,00	0,00	0,23	0,58	3,27
0,00	0,00	0,00	0,46	1,15
0,00	0,00	0,00	0,39	2,08
0,00	0,00	0,00	0,17	0,52
0,00	0,45	0,00	0,32	3,08
0,00	0,00	0,00	1,11	1,75
0,00	0,00	0,00	0,68	1,35
0,00	0,00	0,00	1,00	1,20
0,00	0,00	0,00	0,60	2,07
0,00	0,00	0,16	0,81	2,10
0,00	0,00	0,31	6,22	4,98
0,00	1,56	0,00	8,70	11,59
2,13	2,13	0,34	6,38	10,40
0,00	0,00	0,00	3,61	9,44

0,50	0,50	0,85	14,49	9,80
0,00	0,00	0,00	0,00	0,00
0,00	0,00	0,00	7,86	10,71
0,00	0,00	0,00	0,00	0,00
0,00	0,00	0,27	13,40	10,99
0,00	0,00	0,60	10,71	9,52
0,00	0,00	0,00	18,18	6,25
0,00	0,00	0,00	10,81	10,81
0,00	0,00	0,48	6,30	10,41
0,00	0,00	0,00	9,38	10,42
0,00	0,00	1,58	10,56	11,97
0,00	0,00	1,49	2,99	10,45
0,00	0,00	0,36	11,31	11,68
0,00	0,00	0,34	7,76	11,55
0,00	0,00	0,00	5,71	6,12
0,00	0,00	0,00	5,31	7,96
0,00	0,44	0,12	10,99	8,30
0,00	0,00	0,00	0,00	0,00
0,00	0,00	0,00	7,59	10,13
0,00	0,00	0,00	4,74	6,03
0,00	0,00	0,00	3,40	6,80
0,00	0,00	0,00	5,14	10,29
0,00	0,00	0,00	3,56	3,16
2,22	0,00	0,76	3,42	6,08
0,00	2,04	0,00	5,13	8,33
0,00	0,93	0,00	2,69	3,59
0,00	0,00	0,18	0,53	3,69
0,00	0,00	0,15	2,12	5,16
0,00	0,00	0,53	19,05	6,88
0,00	0,00	1,21	14,57	4,86
0,58	0,58	0,21	3,64	6,00
0,00	0,00	1,85	9,26	13,89
0,00	0,00	0,00	0,00	0,00
0,00	0,00	0,00	0,00	0,00
0,00	0,00	0,00	10,00	12,50
1,39	0,46	0,99	10,30	9,55
0,00	0,00	0,00	0,00	0,00
0,00	4,18	0,55	11,20	11,31
0,00	6,67	0,00	12,31	6,15
0,00	0,00	0,81	13,28	7,86
0,19	0,00	0,07	0,30	1,71
15,96	0,00	0,00	0,58	1,46
0,00	0,00	0,00	0,00	0,00
0,00	0,00	0,00	0,00	0,00
0,00	0,00	2,05	5,64	8,21
0,36	0,00	0,23	13,94	11,15
0,52	0,00	0,30	7,72	7,12
0,00	0,00	0,42	7,81	12,03
0,00	0,00	0,55	14,75	9,84
5,63	0,00	0,38	5,32	12,93

0,00	0,00	0,41	11,89	13,52
0,00	0,00	0,00	8,14	16,28
0,00	0,00	0,44	2,75	6,48
0,00	0,00	0,51	11,99	11,48
0,00	0,00	0,00	5,00	10,00
0,00	0,00	0,00	9,55	10,45
0,00	0,00	0,00	2,70	8,11
0,00	0,00	0,00	7,89	11,84
0,00	0,00	0,00	1,38	9,69
0,00	1,56	1,04	5,21	11,46
0,00	0,00	0,00	0,00	0,00
0,00	0,00	0,00	0,00	0,00
0,00	0,00	0,39	4,71	4,31
0,00	0,00	0,00	0,00	0,00
0,00	0,00	0,00	1,80	3,60
0,00	0,00	0,00	2,34	6,54
2,22	0,00	0,48	6,28	3,38
0,00	0,00	0,00	1,35	18,92
0,00	0,00	0,00	15,58	7,54
0,00	0,00	0,71	10,71	13,57
0,00	0,00	1,11	16,45	5,36
0,00	0,00	0,94	12,54	13,17
0,00	0,00	3,20	12,00	4,80
0,00	0,00	0,00	4,00	16,00
0,00	0,00	0,00	0,00	0,00
0,00	0,00	1,69	5,06	7,17
0,00	0,00	0,00	0,00	0,00
0,00	1,47	0,42	5,93	4,66
0,00	0,00	0,15	3,81	12,79
0,00	0,00	0,85	8,96	11,95
0,35	0,00	0,00	2,64	5,14
0,00	0,00	0,20	1,20	2,81
0,00	0,00	0,23	0,35	2,54
1,28	0,96	0,00	3,56	6,00
0,00	0,00	0,25	3,40	5,16
3,73	0,00	0,00	1,02	1,42
1,11	0,74	0,10	0,52	1,34
0,00	0,00	0,00	0,71	0,71
0,00	0,00	0,00	0,00	0,00
0,00	2,06	0,00	5,59	9,71
0,76	0,76	1,00	10,16	11,16
0,00	0,00	0,22	8,63	9,29
1,01	4,53	0,43	6,61	11,01
0,00	0,00	0,00	1,28	2,55
0,00	0,00	0,00	0,00	0,00
0,00	0,00	0,30	0,89	2,66
5,29	0,88	0,00	3,13	12,37
0,00	0,00	0,36	7,12	11,27
0,31	0,00	0,27	5,99	7,08
0,00	0,00	0,25	5,95	10,51

0,00	0,00	0,00	5,63	10,61
0,00	0,00	0,38	6,91	8,70
0,00	0,00	2,54	4,24	5,08
25,45	10,43	0,07	0,93	2,01
7,96	0,64	0,11	2,28	3,80
0,00	0,00	0,25	0,74	1,61
0,43	0,00	0,00	1,38	1,79
0,00	0,00	0,97	13,27	8,74
0,00	0,00	0,00	3,56	6,44
0,83	0,83	0,15	2,68	4,92
0,00	0,00	0,00	1,01	3,52
0,00	0,00	0,00	2,84	3,84
0,00	0,00	0,00	2,56	3,01
2,33	0,00	0,00	2,72	5,90
1,58	0,00	0,08	1,20	7,76
1,08	0,54	0,19	3,89	4,28
0,00	0,00	0,00	3,64	5,45
0,00	0,00	0,00	3,33	4,86
0,34	0,00	0,00	2,77	4,84
0,00	0,00	0,00	3,42	4,37
1,14	6,29	0,36	6,62	9,12
0,00	0,00	0,00	3,65	9,74
0,90	0,45	0,00	3,66	9,70
6,04	0,00	0,24	4,67	8,01
0,63	0,00	0,54	8,50	10,13
1,34	0,00	0,58	4,82	13,87
5,85	1,06	0,15	8,75	8,01
0,00	0,00	0,00	0,00	0,00
0,00	0,00	0,00	0,89	6,21
0,00	0,00	0,00	2,27	7,89
0,00	0,00	0,19	1,12	4,28
0,00	0,84	0,15	1,04	3,71
1,35	0,00	0,00	0,93	3,25
0,00	0,00	0,59	5,48	7,26
0,00	0,00	0,13	3,56	6,49
0,00	0,00	0,00	3,78	7,68
0,00	0,00	0,29	2,80	15,93
1,44	0,00	0,00	2,45	10,33
0,00	0,72	0,11	5,69	10,92
0,00	0,00	0,22	7,83	10,22
1,64	0,00	0,13	4,27	13,17
0,45	0,00	0,40	8,46	10,07
0,00	0,00	0,61	8,50	9,59
0,00	0,00	0,11	2,79	7,37
0,00	0,00	0,11	3,52	7,48
0,00	0,00	0,58	2,89	6,94
0,00	0,00	0,13	10,75	9,00
0,00	0,00	0,13	4,71	9,28
0,00	0,00	0,19	12,48	7,60
0,00	0,00	0,32	5,99	12,14

0,00	0,00	0,76	4,04	8,84
0,00	0,00	1,30	11,16	9,13
0,52	1,04	0,53	9,70	10,45
3,53	0,32	0,29	9,11	12,40
0,00	0,00	0,55	7,03	10,91
0,00	0,00	0,14	6,34	9,54
0,00	0,00	0,00	0,00	0,00
13,28	0,83	0,00	3,37	4,46
25,11	16,02	0,39	5,48	10,04
5,43	49,44	2,29	8,38	5,84
7,45	25,00	0,15	2,56	5,86
18,13	23,98	0,17	2,15	2,98
50,00	1,19	0,31	1,54	1,85
22,17	3,45	0,00	0,61	2,89
94,57	1,63	0,00	5,60	6,96
89,17	0,00	0,71	5,67	4,96
1,35	0,00	1,40	1,00	1,60
0,00	0,00	0,00	0,00	1,59
0,46	0,00	0,27	12,42	9,61
0,00	0,00	0,00	2,65	3,19
0,00	0,00	0,00	1,18	2,02
0,00	0,00	0,15	2,68	5,21
0,00	0,00	0,00	0,82	2,95
0,00	0,00	0,00	1,15	2,29
0,00	0,00	0,48	1,93	3,49
0,00	0,00	0,12	2,22	4,45
0,00	0,00	0,15	2,20	3,23
0,00	0,00	0,00	1,98	2,90
0,00	0,00	0,00	0,95	2,30
1,10	0,00	0,46	6,97	9,44
0,00	0,00	0,94	8,08	9,59
0,00	2,38	0,50	6,37	11,74
0,00	0,00	0,00	0,00	0,00
0,00	1,63	0,21	2,76	3,51
0,00	0,00	0,48	7,66	7,66
0,00	0,00	0,13	6,15	10,54
3,30	0,00	0,28	2,42	7,69
0,00	0,00	0,11	7,76	10,99
0,00	0,00	0,38	4,92	10,23
1,89	0,00	0,97	13,59	9,53
5,41	0,00	0,86	9,87	8,58
0,00	0,00	1,46	18,89	1,17
0,00	0,00	1,83	10,09	10,09
1,32	0,00	0,00	5,52	5,17
4,42	6,63	0,93	6,89	11,17
3,72	0,74	0,00	5,51	10,79
0,00	0,00	0,75	9,38	11,22
0,00	0,00	0,00	0,00	7,69
0,00	0,00	0,00	4,20	3,15
0,00	0,00	0,00	1,71	3,81

0,48	0,00	0,00	1,09	2,18
0,00	0,00	0,00	7,81	7,81
1,20	2,40	0,26	5,01	5,54
0,72	2,90	1,77	10,22	8,25
0,43	0,00	0,00	0,58	0,81
2,53	24,05	0,37	11,85	10,37
0,95	0,00	0,00	1,55	1,94
0,00	0,00	0,00	1,40	1,57
0,70	1,40	0,23	1,15	1,83
0,00	0,00	0,19	0,56	2,61
0,00	0,00	0,00	0,29	1,17
0,00	0,00	0,00	0,41	1,62
0,00	0,00	0,00	1,13	2,09
0,00	0,00	0,19	1,12	1,87
0,00	0,00	0,00	3,90	2,81
0,00	0,00	0,33	0,49	3,42
0,58	0,00	0,21	0,21	2,95
0,00	0,00	0,00	1,72	2,46
0,00	0,00	0,00	0,83	1,52
0,00	0,00	0,16	0,95	0,64
0,00	0,00	0,00	1,00	1,99
0,36	0,00	0,15	2,29	8,09
0,43	0,43	0,15	0,74	5,79
0,00	0,40	0,00	1,66	3,45
0,00	0,00	0,28	1,41	2,53
0,00	0,00	0,29	0,87	9,88
0,00	0,00	0,00	2,11	3,17
0,00	0,00	0,00	1,07	1,79
0,00	0,28	0,54	4,45	5,63
0,00	0,00	0,00	2,21	6,99
0,00	0,00	0,00	0,00	0,00
0,54	0,00	0,00	2,83	4,83
0,00	0,00	0,00	0,00	0,00
0,00	0,00	0,22	1,73	2,81
0,00	0,00	0,00	3,06	2,04
0,00	0,00	0,00	4,80	5,81
0,00	0,00	0,00	0,87	4,86
0,00	0,00	0,00	0,85	2,27
0,00	0,44	0,17	2,36	2,87
0,00	3,23	0,00	3,26	4,35
0,00	0,00	0,45	1,34	5,59
0,00	0,00	0,00	1,40	4,34
3,76	0,00	0,00	3,12	11,03
0,00	1,18	0,00	3,10	5,29
0,00	0,00	0,37	5,19	6,67
0,00	0,00	0,00	4,46	7,04
0,00	0,00	0,00	7,14	7,14
0,29	0,88	0,00	2,72	7,17
3,13	0,00	0,00	3,88	5,83
0,75	0,38	0,00	0,85	2,42

0,00	0,00	0,00	5,88	6,54
0,00	0,00	0,16	3,08	5,68
0,00	0,00	0,00	3,73	6,00
0,00	0,00	0,49	0,98	8,82
0,00	0,00	0,00	2,27	2,42
1,79	0,00	0,00	1,64	4,38
0,00	0,00	0,90	16,12	7,76
0,00	0,00	0,14	3,05	5,69
0,95	0,00	0,87	3,47	7,28
0,00	0,00	0,00	1,99	4,71
0,00	0,00	0,00	0,00	0,00
0,93	0,00	0,00	3,26	5,59
0,00	5,26	0,00	8,20	13,11
2,38	2,38	0,00	8,13	10,00
0,00	0,00	0,62	6,17	12,35
0,00	0,00	0,00	9,71	9,71
0,00	0,00	0,30	5,86	7,37
0,00	0,00	0,73	8,06	5,86
0,00	0,00	1,22	13,65	4,55
0,00	0,00	0,72	11,96	10,40
0,00	0,00	0,57	15,38	8,83
0,00	0,00	0,89	9,52	4,02
0,00	0,00	0,15	1,68	3,36
0,60	0,00	0,00	1,59	4,08
0,00	0,00	0,00	0,22	2,20
0,00	0,00	0,00	1,29	3,86
0,00	0,00	0,58	2,92	4,67
0,00	0,00	0,23	0,92	4,60
0,66	0,00	0,00	1,45	2,03
0,00	0,00	0,00	0,95	1,90
0,00	0,39	0,14	0,72	5,22
0,00	0,00	0,00	1,43	2,39
0,00	0,00	0,00	1,23	2,86
0,00	0,00	0,00	1,95	5,53
0,00	0,00	0,34	7,69	10,60
0,00	0,38	0,24	4,13	8,98
0,54	0,00	0,51	10,10	10,62
0,00	0,00	0,42	8,71	11,24
0,00	0,00	0,17	12,85	17,36
0,00	0,43	0,48	8,08	13,63
0,00	0,00	0,56	13,48	7,30
0,00	66,67	0,00	0,00	5,00
0,00	1,45	0,00	7,93	11,45
38,99	52,20	0,56	5,44	5,63
0,00	0,00	0,00	0,00	0,00
21,05	0,00	0,00	11,48	8,20
5,06	0,00	0,67	9,67	12,00
0,00	0,00	0,00	0,00	0,00
2,50	14,17	0,26	2,81	6,89
80,21	4,28	0,15	3,55	6,66

0,00	0,00	0,00	0,00	0,00
5,77	17,31	0,00	3,80	5,98
89,13	0,00	0,00	1,74	5,23
0,00	0,00	0,00	0,00	5,56
0,00	2,76	0,12	5,99	5,40
0,00	0,00	0,00	2,01	2,81
0,43	0,00	0,26	14,49	10,22
0,00	0,00	0,28	2,69	4,11
0,00	0,00	0,00	1,63	2,72
0,00	0,55	0,34	2,05	2,56
0,00	3,24	0,14	1,92	3,70
0,00	0,00	0,00	1,12	2,47
0,00	0,00	0,00	1,81	2,27
0,00	0,00	0,00	2,04	2,04
0,00	0,00	0,00	0,57	3,70
0,00	0,00	0,00	1,90	5,49
0,00	0,00	0,00	1,43	3,57
0,00	0,00	0,00	12,00	10,00
0,00	0,00	0,18	10,27	9,38
0,00	0,00	0,00	8,33	8,33
0,00	0,00	0,00	2,12	5,82
0,00	0,00	2,63	2,63	2,63
0,63	0,00	0,00	2,16	2,80
0,00	0,00	0,00	0,00	0,00
0,00	0,00	0,78	21,71	3,88
0,00	0,00	0,00	14,49	7,25
0,00	3,03	0,61	8,70	10,53
5,36	9,82	0,00	3,67	5,62
0,00	0,00	0,00	0,00	0,00
0,00	0,00	0,00	0,00	0,00
0,97	0,00	0,00	2,00	7,71
0,00	0,00	0,00	0,00	0,00
0,00	0,00	0,52	5,56	8,16
10,31	0,31	0,27	6,09	8,73
1,69	2,82	0,17	5,72	10,75
2,79	0,00	0,00	4,38	5,95
0,81	0,00	0,67	4,77	8,59
0,40	0,00	0,74	7,85	7,98
1,29	0,00	0,39	6,15	11,26
0,35	0,00	0,11	7,02	11,55
0,00	0,00	0,45	6,13	10,93
0,00	0,00	0,23	8,29	9,56
0,54	0,00	0,48	6,94	9,68
0,40	0,00	0,24	4,51	10,00
0,00	0,00	0,00	5,67	12,75
0,00	0,00	1,32	9,91	12,26
0,00	0,00	0,16	7,88	13,78
0,00	0,00	0,78	15,85	6,99
0,28	0,00	0,80	10,26	11,61
0,00	0,00	0,28	5,74	11,69

0,00	0,00	0,26	14,77	8,55
0,00	0,00	0,00	0,87	13,06
1,25	1,25	0,13	3,91	10,55
0,00	0,00	0,43	3,41	9,17
0,00	0,00	0,28	5,42	9,46
0,00	0,00	0,00	6,52	10,91
0,00	0,00	0,00	9,22	8,44
0,00	0,00	0,20	6,37	10,39
0,00	0,00	0,53	3,40	10,73
0,35	0,00	0,23	3,72	9,53
0,00	0,00	0,47	5,92	8,83
2,16	0,22	1,41	10,39	10,56
1,18	0,00	0,85	6,78	11,62
0,34	0,34	0,83	8,67	10,42
0,00	0,00	0,21	2,32	9,18
0,00	0,00	0,28	4,28	7,91
0,27	0,27	1,10	7,95	11,80
0,00	5,56	0,00	6,96	13,04
0,00	0,00	0,86	11,37	8,05
0,53	1,58	0,40	11,55	9,16
4,55	0,00	0,66	9,87	7,89
1,64	6,01	0,32	8,93	6,82
0,00	0,57	0,98	7,82	8,89
1,11	0,00	0,23	5,30	8,12
1,19	0,00	0,12	4,11	7,49
0,00	0,00	0,30	4,51	5,26
0,00	0,00	0,00	8,54	7,87
0,00	0,71	0,43	6,81	12,13
0,00	0,00	0,13	4,38	11,45
0,00	0,00	0,00	6,62	10,73
0,00	0,86	0,40	6,34	10,93
0,00	0,00	0,00	0,00	0,00
0,00	0,00	1,36	5,08	7,68
0,00	0,00	0,00	2,93	11,82
0,00	0,00	0,00	3,88	8,37
0,00	0,00	0,12	4,01	12,97
0,35	0,00	0,89	4,13	9,04
0,00	0,00	0,00	3,13	8,67
0,00	0,00	0,14	1,79	7,57
0,00	0,00	0,62	7,66	9,26
0,18	0,00	0,00	8,32	15,35
0,46	0,46	0,00	6,44	9,90
0,00	0,00	0,29	9,73	13,86
1,57	0,00	0,50	11,06	11,06
0,00	0,00	0,00	4,96	11,57
0,00	0,00	0,86	11,58	10,96
0,00	0,00	0,56	7,28	12,87
0,00	0,00	0,17	9,39	2,78
0,00	0,00	0,46	6,93	13,63
0,00	0,00	0,00	10,99	9,95

0,32	0,00	0,30	6,01	10,92
0,00	0,30	0,18	3,58	7,08
0,00	0,00	0,00	7,18	7,18
0,00	0,00	0,13	4,60	9,33
0,00	0,00	0,13	5,52	12,30
0,00	0,00	0,10	5,60	11,11
1,94	0,00	0,33	4,10	5,82
0,00	0,00	0,00	14,27	12,67
0,67	0,00	0,31	7,32	12,37
0,00	0,37	0,11	1,19	19,61
0,44	0,00	0,29	6,17	11,48
0,00	0,36	0,43	6,62	15,74
0,00	0,00	0,49	10,00	11,48
0,00	3,89	0,55	7,55	7,55
0,00	0,00	0,00	0,00	0,00
0,00	0,78	0,83	10,61	11,44
0,00	0,00	1,11	9,64	11,62
0,00	0,00	0,52	10,60	9,65
0,00	0,00	0,00	0,00	0,00
0,73	0,00	0,00	9,88	14,15
0,00	0,00	0,22	7,33	11,42
0,50	0,00	0,08	1,90	6,19
0,00	0,00	0,73	6,05	6,30
0,00	0,00	0,00	3,10	6,40
0,00	0,00	0,30	2,39	6,27
0,00	0,00	0,00	4,18	7,32
0,00	0,00	0,00	2,87	5,57
0,00	0,00	0,10	3,94	6,50
0,00	0,00	0,09	4,60	8,73
0,00	0,37	0,00	6,55	8,62
0,00	0,00	0,17	6,60	10,94
0,00	0,00	0,00	2,54	6,73
0,00	0,00	0,41	3,73	7,05
0,00	0,00	0,27	4,10	7,79
1,06	0,00	0,70	6,60	8,34
0,67	0,89	0,14	5,84	10,76
0,00	0,00	0,18	6,70	9,06
0,00	0,00	0,50	7,92	10,82
0,60	0,60	0,47	7,44	8,76
0,00	0,00	0,48	3,81	10,48
0,19	50,94	0,11	11,44	10,96
0,00	0,00	1,30	7,15	11,44
0,00	2,00	0,55	5,67	15,49
0,35	2,11	0,63	6,70	12,88
0,00	0,00	0,00	5,88	23,53
11,76	67,65	0,85	6,84	11,97
0,00	6,96	0,19	10,19	14,26
4,91	1,23	0,00	7,90	10,31
0,00	0,00	0,49	10,87	10,38
0,00	0,24	0,53	7,25	13,12

0,00	0,00	0,30	7,88	9,70
0,00	0,00	0,70	6,32	7,28
0,00	0,00	0,46	7,54	13,37
0,00	0,00	0,29	6,28	12,37
0,00	0,00	1,50	9,24	7,63
0,37	0,00	0,36	8,09	13,65
0,30	2,13	0,59	6,72	11,17
0,00	0,00	0,00	8,75	15,97
0,00	0,00	0,71	7,48	8,67
0,00	0,00	0,00	4,34	9,52
0,00	0,00	0,30	4,74	7,26
0,00	0,00	0,00	5,26	6,51
0,00	0,00	0,37	7,89	7,40
0,00	0,00	0,11	3,59	6,23
0,00	0,00	0,00	4,21	6,52
0,00	0,00	0,09	2,26	7,45
0,00	0,00	0,00	2,71	7,76
0,00	0,00	0,00	0,00	0,00
0,00	0,00	0,14	4,55	5,38
0,00	0,00	0,13	3,64	5,77
0,00	0,00	0,00	5,16	5,97
0,00	0,00	0,00	4,24	6,63
0,00	0,00	0,00	2,51	4,68
0,00	0,00	0,00	2,74	5,11
0,00	0,00	0,12	4,50	4,74
0,00	6,99	0,91	6,15	9,57
0,00	0,00	0,36	5,40	8,27
0,00	0,58	0,09	7,31	12,36
0,00	0,25	0,24	6,32	10,21
1,34	0,00	0,22	5,84	9,74
0,00	0,00	0,31	10,80	9,55
0,37	0,00	0,35	9,09	11,22
0,41	0,00	0,12	3,97	7,23
0,00	0,00	0,55	10,86	9,31
0,00	0,00	1,65	6,96	7,60
0,00	7,27	0,00	5,95	13,51
2,35	0,39	0,75	7,20	10,93
0,65	0,00	0,54	8,84	11,25
0,59	0,59	0,54	7,00	11,12
0,00	0,00	0,47	7,42	9,78
0,00	0,00	0,37	6,22	9,01
0,00	0,00	0,91	1,83	7,62
0,26	2,05	0,31	2,47	10,33
0,00	0,00	0,11	4,73	10,45
0,00	0,00	0,11	4,19	11,89
0,00	0,00	0,31	5,82	10,21
0,00	0,00	0,17	13,76	10,28
0,00	0,00	0,14	8,65	9,05
0,00	0,00	0,61	6,56	11,68
0,00	0,00	0,82	5,77	9,89

0,00	0,00	0,00	0,00	14,21
0,00	0,00	0,76	9,45	13,52
0,00	1,72	0,55	7,28	11,13
0,00	0,00	0,00	0,00	0,00
0,00	0,00	0,00	5,83	10,83
0,00	0,00	0,00	5,76	11,02
0,00	0,00	0,00	4,49	9,55
0,00	0,70	0,24	6,92	10,07
0,51	0,00	0,00	3,73	11,04
0,00	0,60	1,36	10,56	10,56
0,00	0,00	0,34	5,06	8,62
0,00	0,00	0,22	2,71	9,01
0,45	0,00	0,41	3,81	8,44
0,00	0,00	0,16	6,91	7,07
0,00	0,00	0,23	9,14	8,56
0,00	0,00	0,00	8,81	6,26
0,00	7,04	0,76	12,25	11,50
0,82	0,00	0,69	9,86	5,05
0,90	0,00	0,54	13,70	9,40
0,00	0,58	0,74	13,47	9,67
0,00	0,00	0,00	8,50	9,80
61,54	18,27	1,30	9,64	5,21
2,53	16,46	0,83	6,61	13,64
0,61	22,56	0,19	7,41	13,89
0,00	0,00	0,46	8,04	11,37
0,00	0,54	1,29	8,72	9,53
0,00	0,00	0,24	5,55	11,92
0,00	0,00	0,53	7,98	11,97
0,00	0,00	0,00	2,44	9,76
0,30	0,00	0,37	7,56	9,95
0,00	0,00	0,19	8,60	10,28
0,00	0,00	0,27	8,29	10,83
0,00	0,00	0,97	9,16	8,77
0,00	0,00	0,00	9,17	12,84
0,00	0,71	0,11	8,66	10,65
7,84	0,00	0,57	10,68	8,48
0,00	0,35	0,21	6,83	9,88
0,00	0,00	0,45	7,27	13,03
0,00	0,46	0,27	7,03	8,36
0,60	25,75	0,43	6,29	11,28
4,26	0,00	0,45	6,64	9,45
0,00	0,76	0,00	4,37	12,86
0,00	0,00	1,00	7,24	9,47
0,00	2,32	1,12	7,73	12,84
0,00	0,98	0,30	5,41	10,93
0,00	0,00	0,41	7,51	6,56
0,74	1,12	0,13	4,70	16,26
0,59	0,00	0,19	5,91	11,34
1,43	0,00	0,65	9,57	12,17
2,51	0,00	0,19	12,80	0,84

0,35	0,00	0,33	7,86	12,29
0,00	0,00	0,49	7,44	15,96
0,00	0,64	0,38	7,09	8,24
0,00	1,49	0,00	8,02	12,69
0,00	0,00	0,00	7,16	7,58
0,00	0,00	0,62	5,72	11,64
0,00	0,00	0,19	8,63	13,88
0,00	0,00	0,45	8,12	10,53
0,00	0,00	0,70	5,14	10,05
0,00	0,00	0,23	7,71	12,85
0,00	0,00	0,67	9,18	12,19
0,00	0,00	0,00	7,00	13,12
0,00	0,00	0,00	0,00	0,00
6,90	27,59	0,93	9,35	11,21
2,92	2,27	1,20	6,11	10,37
0,00	6,67	0,23	5,86	9,68
0,00	2,47	0,00	5,09	10,93
0,78	0,39	0,36	7,84	12,59
0,00	0,00	0,92	9,40	11,09
0,98	5,88	0,60	6,32	13,53
1,43	11,43	0,59	8,50	10,67
0,00	0,00	0,66	6,29	8,11
0,00	0,00	0,75	6,26	12,82
0,00	0,00	0,67	10,15	12,31
0,00	0,00	0,28	8,26	11,90
0,00	0,00	0,53	7,53	7,71
0,00	0,00	0,13	8,20	13,54
0,00	0,00	0,12	6,08	10,55
0,00	0,00	0,40	8,80	9,60
0,00	0,00	1,31	9,51	7,87
0,00	0,00	0,78	12,95	7,48
0,00	0,00	0,48	14,00	8,19
0,57	0,28	0,27	8,58	10,88
0,00	0,00	0,00	7,93	11,99
0,00	0,00	0,30	10,63	10,32
0,00	0,00	0,00	8,45	10,50
0,00	0,00	0,27	2,93	6,65
0,00	0,00	0,31	6,41	10,00
0,00	0,00	0,64	4,28	13,06
0,00	0,65	1,27	8,33	4,61
0,00	0,00	0,00	1,15	2,68
0,00	0,00	0,00	0,00	0,00
0,00	0,00	0,30	5,51	9,69
0,00	0,00	0,00	8,95	12,23
0,00	0,00	0,00	8,13	6,33
2,39	0,68	0,00	5,19	23,28
0,00	0,00	0,53	10,00	11,32
0,00	0,00	0,82	15,80	5,18
0,00	0,00	0,51	6,67	7,69
0,00	0,00	2,08	11,61	10,12

0,00	0,00	0,30	9,89	11,19
2,02	2,02	0,31	1,24	10,87
6,05	0,00	0,00	7,56	16,41
3,03	0,00	0,00	0,78	5,43
0,00	0,00	1,07	11,86	9,43
0,00	0,00	0,00	2,00	12,14
0,00	0,00	0,00	5,67	6,70
0,00	0,00	0,29	5,24	10,49
0,00	0,00	0,80	10,77	10,31
0,00	0,00	0,42	7,31	9,70
0,00	0,00	0,94	12,20	11,63
0,00	0,00	0,00	2,58	8,76
0,00	0,00	0,83	5,83	8,33
0,00	0,00	0,62	9,97	10,90
0,00	2,58	0,37	9,38	9,01
0,00	0,00	0,00	7,63	13,89
0,00	0,00	0,00	2,83	5,80
0,00	0,00	0,00	3,45	6,37
0,00	0,00	0,27	4,54	7,74
0,34	0,34	0,43	2,91	9,70
0,00	0,00	0,00	8,58	11,66
0,00	0,00	4,42	10,77	6,15
0,00	0,00	1,88	14,06	7,50
0,38	0,00	0,35	1,84	5,76
0,96	4,31	0,47	4,08	10,99
0,00	4,87	0,12	3,63	12,82
28,89	0,00	0,52	8,85	6,25
0,00	0,00	0,00	8,84	8,84
0,00	0,00	0,65	9,87	10,42
0,00	0,00	0,26	6,65	10,49
0,00	0,25	0,37	3,45	8,61
0,00	0,00	0,00	2,27	11,36
0,00	0,00	1,04	9,38	9,38
0,00	0,00	0,55	9,94	11,60
0,00	0,00	0,00	11,73	7,65
0,24	0,48	0,29	5,22	10,14
0,00	0,40	0,24	9,82	12,81
0,00	0,00	0,17	5,50	9,00
2,86	2,86	0,80	7,20	12,80
0,00	0,00	0,32	4,46	15,61
0,00	0,00	1,30	10,05	9,72
0,00	0,00	0,19	3,01	14,10
2,08	0,00	0,00	8,39	17,11
0,00	0,00	0,51	11,21	11,38
0,00	6,67	0,00	5,45	10,91
0,00	0,00	0,00	0,00	0,00
0,00	22,73	3,11	11,49	8,54
1,90	0,00	1,01	4,61	13,40
0,00	0,50	0,15	5,21	10,87
0,00	0,00	0,00	4,17	8,33

0,97	0,00	0,00	3,25	7,10
0,00	0,00	0,26	5,79	7,11
0,00	0,00	0,42	8,47	11,02
0,00	1,88	0,20	8,81	11,41
0,00	0,00	0,00	5,44	11,82
0,00	0,00	1,26	9,66	9,24
0,00	1,56	0,00	14,11	20,86
1,20	3,20	0,63	3,76	15,02
0,00	0,00	0,28	16,06	17,46
0,00	0,00	0,00	2,45	5,71
0,00	0,00	0,00	12,35	12,35
0,00	0,00	1,91	10,35	8,72
0,00	0,00	0,55	5,52	12,15
0,50	0,50	0,00	7,84	11,92
0,00	0,00	0,73	8,23	8,96
0,00	0,00	0,51	9,20	7,16
0,67	0,00	0,73	11,36	8,97
0,44	0,00	0,80	5,46	12,68
0,57	2,85	1,09	9,61	10,44
0,00	5,41	0,93	8,36	8,92
0,00	0,00	1,00	9,89	10,69
0,00	0,00	2,23	11,68	12,03
0,00	0,44	0,78	5,81	11,37
0,00	0,00	0,50	8,34	12,96
0,00	0,00	0,41	6,39	9,28
0,00	0,00	0,00	10,34	5,75
0,00	0,00	0,00	6,70	13,41
0,00	0,00	0,00	3,31	10,14
0,37	0,00	0,11	3,85	5,78
0,00	0,00	0,19	4,10	9,87
0,00	0,00	0,60	8,33	8,93
0,00	0,00	1,26	10,06	8,18
0,00	0,00	0,00	5,65	9,68
0,00	0,00	0,00	3,95	11,84
0,00	0,00	0,63	7,14	10,29
10,12	1,79	0,17	4,95	9,73
0,00	0,71	0,23	6,09	8,80
0,00	0,00	0,13	6,65	11,42
0,00	0,00	0,96	10,10	12,98
0,00	0,00	0,25	6,13	7,79
0,00	0,00	0,93	11,16	10,23
0,00	0,00	1,10	14,84	8,24
6,06	0,00	0,00	4,85	11,65
0,00	0,00	0,00	8,44	12,99
0,00	0,00	0,00	0,51	16,11
0,00	0,00	0,00	2,00	11,00
1,30	0,00	0,82	5,33	11,89
0,00	0,00	0,00	6,80	13,60
0,00	0,00	0,59	7,13	11,09
0,00	0,00	0,33	7,68	12,19

0,00	0,00	0,48	2,22	8,73
0,00	0,00	1,43	7,62	9,21
0,00	0,00	0,00	1,84	11,95
0,00	0,00	0,37	9,52	8,79
0,00	0,00	0,00	3,85	3,85
0,00	0,00	0,46	11,87	10,50
0,00	0,00	0,00	0,00	0,00
0,00	0,00	1,12	7,30	7,30
0,00	5,61	0,00	5,34	9,67
0,00	0,00	0,00	0,00	0,00
2,63	5,26	0,00	4,83	10,41
0,00	2,12	0,31	11,31	8,56
0,54	0,00	0,00	1,96	10,87
0,00	0,00	0,60	4,37	10,93
0,00	6,15	0,43	5,15	14,16
0,00	1,28	0,22	10,65	10,54
11,11	0,00	0,00	9,52	14,29
0,00	0,00	0,66	8,06	10,70
0,70	0,00	0,35	6,34	14,06
0,00	0,00	0,35	8,19	9,59
0,00	0,00	0,46	8,97	11,95
0,00	0,00	0,88	11,21	10,41
0,00	0,79	0,49	6,59	8,78
0,00	2,40	0,84	8,21	13,23
0,00	0,46	0,13	3,27	7,71
0,00	0,00	0,76	11,45	9,92
0,00	0,00	0,00	14,40	4,80
0,00	1,83	0,00	5,65	7,74
0,45	0,00	1,30	6,36	7,52
0,00	4,26	0,00	3,61	11,45
0,00	0,00	1,17	11,20	10,87
0,00	0,00	0,00	12,77	10,11
0,00	2,15	0,65	8,44	13,64
0,00	0,00	1,20	10,55	6,47
0,00	0,00	0,62	8,39	8,70
0,39	10,59	0,92	10,74	10,39
0,38	2,29	0,00	2,84	6,12
0,00	0,00	0,00	0,00	0,00
0,00	2,08	0,60	11,16	13,94
0,00	0,00	0,00	4,23	7,04
0,00	0,00	0,00	4,39	6,14
0,00	0,00	0,00	11,07	14,12
0,00	0,00	0,00	4,96	11,07
0,00	0,00	0,00	7,02	12,37
0,00	0,00	1,00	14,93	7,96
1,54	0,00	0,50	13,43	7,96
0,00	0,00	0,72	12,95	11,27
0,00	0,00	0,00	0,00	0,00
0,00	0,00	0,81	8,13	17,07
0,00	0,00	0,53	11,76	11,23

0,00	0,00	0,00	0,00	0,00
0,00	0,26	0,47	8,05	13,53
0,00	2,93	0,09	6,24	10,77
0,83	0,00	0,25	7,12	14,11
0,91	0,00	0,74	7,43	9,65
0,47	0,00	0,00	9,67	14,05
0,00	0,00	0,00	4,63	6,00
0,00	0,66	0,00	5,68	11,57
1,41	16,90	0,43	8,19	12,50
0,30	0,00	0,09	5,11	14,52
0,00	0,00	0,14	5,69	9,85
0,00	0,00	0,51	4,77	6,13
0,00	0,00	0,42	9,79	7,69
0,00	0,00	0,70	7,89	11,48
0,00	0,00	0,00	0,00	0,00
0,29	0,29	1,22	9,34	9,96
0,00	0,00	1,02	9,46	12,53
0,46	2,31	0,29	9,47	12,48
0,43	2,16	0,76	6,68	11,71
0,00	0,00	0,28	8,81	12,87
0,00	0,00	0,95	11,64	9,07
0,00	0,00	0,78	14,03	8,42
0,00	0,00	0,88	13,53	9,79
0,00	0,00	0,53	14,93	8,74
1,71	0,00	0,00	6,15	7,06
0,00	0,61	1,12	9,67	6,69
0,00	3,13	0,18	5,99	9,94
0,23	0,00	2,40	14,38	8,56
0,00	0,00	0,93	6,37	9,28
0,00	0,00	0,62	9,94	9,01
0,00	0,38	1,35	8,75	12,29
0,00	4,67	0,44	8,47	11,46
0,00	0,94	1,23	10,37	10,10
6,54	21,50	0,26	9,04	9,30
0,00	0,00	1,42	7,99	9,06
0,00	0,47	0,89	10,42	10,17
0,99	0,00	0,49	11,50	8,06
0,56	2,26	0,10	6,13	14,92
0,00	4,88	2,01	11,53	12,53
0,00	0,28	0,53	11,40	9,74
0,00	0,00	0,28	9,19	10,21
0,00	0,00	0,08	4,65	11,04
0,78	0,00	0,30	6,28	7,83
0,00	3,27	0,47	10,17	11,58
0,71	0,00	0,21	8,81	10,90
0,00	1,14	0,23	8,69	12,19
0,00	0,34	0,11	9,97	13,04
0,00	3,42	0,70	8,46	14,14
0,29	2,62	0,36	6,04	11,35
0,00	0,00	0,88	7,13	11,64

0,00	0,39	0,46	10,16	10,51
0,00	0,00	0,83	7,20	9,93
0,00	0,00	0,52	8,77	10,73
0,00	0,00	0,12	11,38	9,30
0,00	0,00	0,19	5,34	7,18
0,00	0,00	0,47	9,88	12,35
0,00	0,00	0,15	10,80	13,72
0,00	0,00	0,11	5,93	10,36
0,00	0,00	0,35	8,82	11,11
0,00	0,00	1,67	14,05	11,19
0,83	3,03	0,75	8,51	13,18
0,00	1,16	0,67	7,69	10,03
0,46	5,94	0,69	9,54	11,48
0,00	7,10	0,33	9,03	8,05
0,00	0,91	1,17	9,51	13,15
0,00	0,00	1,71	11,45	8,95
0,00	0,00	1,25	13,73	7,02
0,48	9,62	0,45	12,22	12,97
0,23	0,94	0,59	7,74	10,47
0,00	0,00	0,00	9,61	13,18
0,00	0,00	0,27	10,24	12,53
0,00	0,00	0,42	8,81	12,73
0,00	0,00	0,29	11,29	9,71
0,00	0,00	0,00	9,90	8,38
0,00	0,00	0,26	8,61	10,15
0,00	0,00	0,00	9,26	11,33
0,00	0,00	0,74	8,24	11,83
0,00	0,71	0,11	4,47	7,88
0,00	0,00	0,20	10,26	11,05
0,00	0,00	0,22	5,92	12,40
0,00	0,00	0,00	3,71	8,58
0,00	0,48	0,00	7,32	8,18
0,00	0,00	0,44	7,26	9,20
0,00	0,00	0,25	6,73	8,48
0,00	0,00	0,28	3,85	7,55
0,00	0,00	0,22	3,46	8,11
0,00	0,00	0,00	3,75	11,88
0,00	0,35	0,12	5,26	11,35
0,00	0,00	0,67	4,86	9,21
0,00	0,00	0,00	3,14	7,44
3,60	72,07	1,26	7,56	12,59
0,00	0,00	0,62	6,99	9,47
0,00	0,00	0,40	6,76	8,62
0,00	0,00	1,60	4,93	7,84
0,00	0,00	0,29	9,26	13,53
0,57	0,00	0,36	9,44	11,25
0,00	0,00	0,00	6,62	13,88
0,00	0,32	0,39	7,10	10,02
0,00	0,00	0,31	5,34	11,30
0,00	1,72	0,55	4,79	11,08

0,00	0,00	0,92	8,26	12,84
0,00	0,00	1,03	10,54	12,78
0,00	0,00	0,90	10,41	10,75
2,01	18,62	1,91	8,03	10,10
0,00	0,00	0,28	5,60	9,14
4,27	0,00	0,00	7,69	8,74
0,46	0,00	0,42	8,45	8,31
0,00	0,00	0,11	8,88	10,00
0,30	0,00	0,27	9,38	9,11
0,00	0,00	0,00	3,55	4,73
0,00	0,00	0,00	10,26	8,16
0,00	0,00	0,00	0,00	20,41
0,00	1,82	0,63	8,81	7,55
0,00	0,00	0,00	11,31	6,93
0,00	0,00	0,45	5,37	3,58
0,00	1,86	0,94	7,89	12,97
7,69	15,38	1,03	2,06	9,28
0,00	0,00	0,74	8,92	14,13
0,00	0,52	0,80	11,74	9,65
1,82	0,00	0,00	6,96	10,58
0,99	1,48	0,69	12,95	9,50
0,00	0,00	0,00	5,19	12,99
0,00	0,60	0,09	4,91	9,82
0,42	0,84	0,54	6,36	8,93
0,00	0,00	0,53	9,36	7,77
0,00	0,00	0,00	12,72	7,10
0,00	0,00	1,43	9,52	7,62
0,00	0,00	0,00	0,00	0,00
0,00	0,00	0,16	9,56	11,53
0,00	0,00	0,00	5,88	17,65
0,00	0,00	0,00	9,18	8,44
0,00	0,00	0,00	4,04	11,66
0,00	0,00	0,37	7,73	11,97
0,00	0,00	0,00	4,38	11,48
0,00	0,00	0,23	8,39	10,66
0,84	1,05	0,13	6,91	11,48
0,00	0,00	0,00	8,85	14,58
0,00	0,00	0,00	10,13	7,49
0,00	0,00	0,00	4,72	5,66
0,00	0,00	3,57	0,00	10,71
0,36	0,00	0,00	7,51	11,27
1,10	0,00	0,00	4,26	10,39
0,00	0,00	0,41	8,98	16,33
0,56	0,00	0,46	9,95	10,41
28,89	0,00	0,69	4,17	17,36
0,00	10,87	0,62	9,88	12,35
2,47	13,58	1,16	8,53	12,79
0,00	0,00	0,36	8,96	13,26
0,00	0,00	0,58	7,80	11,85
0,00	0,00	0,82	10,14	7,40

0,00	0,00	0,46	6,22	14,72
0,00	0,00	0,62	5,59	3,11
0,00	0,00	0,00	7,64	4,87
0,00	0,00	2,25	13,48	5,62
0,00	0,00	0,00	0,00	0,00
0,00	0,00	0,00	0,00	0,00
0,00	0,00	0,00	6,93	7,92
0,00	0,00	0,00	10,47	9,08
0,00	0,63	0,00	13,50	8,79
0,00	0,65	0,00	6,13	8,36
0,00	0,00	0,51	7,99	13,95
0,00	2,33	2,67	7,33	12,67
0,00	4,10	0,45	6,90	11,09
0,65	5,88	0,77	7,87	11,71
0,00	0,00	1,16	9,62	10,31
0,00	0,00	1,77	10,28	10,28
0,49	7,88	1,58	13,16	7,76
0,28	0,42	0,19	6,23	10,89
0,00	0,00	0,00	0,00	0,00
0,52	0,00	0,00	12,34	12,34
0,00	0,00	0,26	8,59	10,94
0,00	0,00	0,38	9,16	8,53
0,00	0,00	1,15	7,11	12,39
0,00	0,00	0,31	7,98	10,64
0,00	0,00	0,85	8,97	10,26
0,00	0,00	0,00	5,79	10,85
0,70	0,00	0,51	6,46	11,01
0,00	0,99	0,63	8,86	12,18
0,74	0,00	0,00	10,56	8,31
0,00	0,00	0,00	7,04	10,56
0,00	0,00	0,36	9,16	12,39
0,00	0,00	2,06	12,37	5,67
0,00	0,00	1,14	11,16	7,58
0,00	0,00	0,00	0,00	0,00
0,00	0,46	1,21	11,64	8,61
0,00	0,00	0,98	8,33	7,84
0,00	0,00	0,52	7,95	13,17
0,00	0,00	0,22	6,89	8,89
0,00	0,00	1,04	12,08	10,00
0,00	0,00	0,16	6,69	11,82
0,00	0,00	0,62	9,08	12,15
0,00	0,00	0,63	3,77	8,18
0,00	0,00	0,00	2,75	8,94
0,00	0,00	0,00	2,34	10,33
0,00	0,00	0,00	3,56	17,53
0,00	0,00	0,00	6,43	18,42
0,00	0,00	0,00	0,00	0,00
0,00	0,00	0,32	15,56	9,21
0,00	0,00	0,00	10,02	8,24
0,00	0,00	0,97	10,68	11,12

0,00	0,00	0,13	9,66	10,41
0,44	0,44	0,82	8,19	10,91
0,00	0,36	0,22	7,31	8,40
0,00	0,00	0,00	8,92	11,47
0,61	0,00	0,00	3,15	8,16
1,05	0,00	0,78	6,88	7,66
0,00	0,00	0,24	9,75	8,93
1,62	1,21	0,00	5,17	11,67
0,00	0,00	0,37	9,89	9,52
0,00	0,00	0,81	5,69	8,54
0,00	0,00	1,54	6,65	11,66
0,00	0,00	0,07	10,08	11,32
0,28	0,00	0,69	12,55	9,24
0,00	0,00	1,22	9,65	10,13
0,00	0,00	1,43	10,41	9,80
0,00	0,00	1,01	12,08	6,92
0,99	4,28	0,50	9,78	11,08
0,00	0,00	0,31	8,29	10,95
0,00	0,00	0,16	9,82	10,97
0,00	0,00	0,00	11,59	8,41
0,00	0,00	0,35	6,43	8,63
1,53	0,00	0,23	6,46	8,19
0,00	0,00	0,74	3,27	10,25
0,90	2,70	0,55	8,17	9,00
1,80	0,00	0,00	5,39	7,29
0,00	0,00	0,21	3,53	6,85
0,00	0,00	0,00	6,79	11,25
1,36	0,00	0,25	8,11	9,52
0,68	0,00	0,14	7,52	11,39
0,00	2,05	2,16	7,20	7,80
0,00	0,00	0,00	11,69	9,25
0,42	0,00	0,12	6,50	10,11
0,25	0,00	0,80	8,88	9,80
10,22	4,84	0,00	8,56	9,63
0,00	0,00	1,38	10,61	9,96
0,00	0,00	1,70	10,87	5,77
0,36	0,00	0,80	10,05	10,15
0,60	0,00	0,34	10,53	11,38
0,00	0,00	0,00	0,00	0,00
0,26	0,00	0,39	8,81	11,24
0,00	0,00	0,11	6,35	11,82
0,00	0,00	0,66	8,60	10,36
0,00	0,00	0,30	7,40	12,37
0,00	0,00	0,37	4,71	9,52
0,00	0,00	0,22	7,62	12,44
0,70	0,00	0,33	7,38	12,11
0,00	0,00	0,11	6,04	7,98
0,00	0,00	0,49	7,41	10,49
0,00	0,00	0,44	8,93	6,10
0,00	0,00	0,00	3,17	5,83

0,00	0,00	0,06	1,77	5,31
0,00	0,00	0,00	1,17	4,02
0,00	0,00	0,00	2,73	7,71
0,00	1,43	0,42	5,83	10,83
0,83	0,00	0,51	4,57	9,14
0,00	6,20	0,00	3,73	6,84
5,31	0,41	0,00	4,78	8,78
0,00	0,00	0,48	4,05	14,05
0,46	0,00	0,71	8,42	11,27
0,00	0,00	0,00	11,36	18,69
0,00	2,27	0,74	14,18	7,73
0,46	3,21	0,51	8,71	8,32
4,41	5,88	0,00	9,44	7,73
3,94	45,52	0,61	5,17	6,89
0,00	0,00	0,14	6,42	12,69
1,26	0,42	0,13	7,30	9,43
0,30	0,00	0,26	7,58	10,93
0,00	0,95	0,00	10,05	7,07
0,00	0,00	0,44	8,41	9,93
0,00	0,00	0,17	7,98	9,89
0,00	0,00	0,98	9,12	10,91
0,00	0,00	0,16	7,61	11,57
0,00	0,45	0,40	7,21	11,08
0,00	0,63	0,09	5,91	9,97
0,25	0,00	0,42	8,98	14,02
0,00	0,00	0,00	9,49	7,84
0,00	0,00	0,16	3,99	7,35
0,00	0,00	0,40	3,32	8,76
0,00	0,00	0,16	2,49	6,07
0,00	0,00	0,00	4,40	13,00
0,55	0,00	1,71	10,54	7,91
0,00	0,00	0,10	7,76	14,88
0,00	0,00	0,47	5,68	14,35
0,00	0,00	0,30	8,76	9,97
0,84	0,00	0,89	12,35	6,12
1,05	0,00	0,00	9,23	11,85
8,00	0,00	0,79	7,31	10,65
0,00	0,00	0,72	7,16	8,77
0,00	0,00	0,61	9,91	10,31
0,60	0,00	0,18	7,72	12,13
0,00	0,00	0,28	7,09	11,06
0,00	0,00	0,24	5,60	12,65
0,00	1,91	0,29	7,23	11,93
0,62	0,00	0,00	2,63	9,69
0,00	0,00	1,44	9,95	10,56
0,79	2,78	1,63	9,43	7,80
0,00	0,00	2,07	9,14	10,34
1,57	0,31	0,71	6,81	8,32
2,09	0,00	0,15	7,68	9,37
0,00	0,65	0,40	9,83	10,92

0,30	0,30	0,39	9,32	11,26
0,00	0,84	0,34	7,58	10,14
2,89	0,00	0,29	6,50	10,80
0,57	0,00	1,46	6,86	7,89
0,22	0,00	0,92	9,42	8,70
0,72	0,00	0,69	7,82	13,45
0,00	0,00	0,25	2,17	6,37
0,00	0,00	0,17	1,49	5,64
0,00	0,00	0,00	0,00	0,00
0,56	0,00	1,16	11,88	9,24
0,00	0,00	1,13	9,06	11,65
0,00	0,00	0,09	7,32	8,99
0,00	0,00	0,61	5,80	11,29
0,00	0,00	0,96	9,03	10,81
0,00	0,00	0,45	9,24	9,92
2,12	1,69	0,51	8,41	9,04
0,75	0,37	0,72	6,76	12,44
0,00	0,00	1,04	7,66	12,42
0,00	0,00	0,20	8,01	12,40
0,00	0,27	0,42	8,74	10,40
0,30	0,00	0,65	8,26	11,69
0,00	0,00	0,22	14,21	8,20
0,00	0,00	1,14	7,22	7,41
0,00	0,00	0,81	10,03	12,74
0,00	0,00	0,65	14,47	11,37
0,00	0,00	0,50	10,41	10,54
0,00	0,00	1,28	8,49	13,33
0,00	0,00	1,46	11,17	8,38
0,00	0,93	0,14	14,51	7,75
0,00	0,00	0,71	11,71	11,09
0,85	0,00	0,00	18,69	1,09
0,00	0,00	0,65	9,68	9,03
0,00	0,00	0,00	7,67	11,58
0,00	0,00	1,04	7,01	12,09
0,00	0,00	0,00	4,22	8,18
0,78	0,00	0,35	7,42	8,69
0,00	1,98	0,44	10,07	10,96
0,00	0,68	0,31	5,97	8,74
0,00	0,36	1,02	9,94	9,60
0,00	1,53	0,52	9,02	10,32
0,35	0,69	0,61	9,18	9,08
0,00	0,00	0,92	10,38	9,77
0,00	0,00	1,19	19,05	10,71
0,00	0,00	0,58	6,81	10,26
0,00	0,00	0,40	8,10	7,51
0,00	0,00	0,00	3,47	6,94
0,00	2,50	0,00	3,33	10,00
0,47	0,00	0,71	7,22	11,05
0,98	1,46	1,10	9,62	11,51
0,63	0,00	0,61	10,93	9,11

0,00	0,43	0,50	7,47	11,96
0,00	0,00	0,65	15,62	12,15
0,00	0,00	1,31	14,10	10,49
0,00	0,00	2,66	10,64	9,04
0,37	0,74	1,71	12,31	8,46
0,00	0,00	2,74	13,25	9,43
0,00	0,00	0,26	12,04	13,61
0,00	0,00	0,00	1,76	17,59
0,00	0,00	0,43	6,35	8,07
0,33	0,33	0,20	6,14	7,85
0,39	1,57	0,14	3,22	9,79
0,00	0,00	0,00	3,89	5,49
1,93	0,00	0,00	5,36	7,59
0,00	0,00	0,00	3,66	6,36
0,00	0,00	0,00	2,19	7,37
0,00	0,32	0,17	4,56	10,03
0,00	0,00	0,00	0,71	2,12
1,03	0,00	0,46	6,79	6,64
0,00	1,04	0,11	2,29	5,88
0,30	0,00	0,10	1,82	5,27
0,00	0,00	0,00	3,29	4,94
0,00	0,00	0,00	6,54	6,02
0,00	0,00	1,44	8,42	6,74
0,37	0,00	0,59	4,96	8,15
0,94	0,00	0,00	3,24	6,32
0,40	0,00	0,13	6,65	7,69
0,00	0,00	0,14	2,92	5,43
2,07	0,69	0,70	6,31	11,21
0,00	0,00	0,24	2,51	6,83
0,45	0,00	0,38	3,43	9,00
0,23	0,00	0,21	8,98	17,90
0,65	34,84	1,88	12,67	7,53
0,00	0,00	0,69	11,66	11,15
0,00	0,00	0,00	4,84	7,50
1,70	0,00	0,10	3,77	7,73
1,56	9,73	0,23	7,31	7,99
6,97	0,00	1,31	6,54	9,51
0,95	3,82	0,27	9,26	8,72
0,00	0,00	1,42	7,98	9,29
0,00	0,00	0,00	2,71	9,30
0,00	0,00	1,18	13,37	8,97
0,31	5,25	0,46	5,68	9,53
0,00	2,37	0,08	4,96	8,55
0,96	1,92	0,50	4,90	11,30
0,59	0,00	0,75	9,77	9,40
0,00	0,00	0,61	9,60	15,55
0,34	0,00	0,21	8,75	9,89
0,32	3,86	0,21	8,06	12,91
0,00	0,28	0,16	11,61	11,28
0,50	0,00	0,22	9,67	8,56

0,43	33,62	0,36	6,94	11,36
0,29	0,00	0,00	8,48	7,86
6,07	0,40	0,94	4,82	8,46
0,00	0,00	0,91	10,75	8,22
0,00	1,18	2,83	10,49	7,89
20,93	11,63	2,05	6,85	9,59
2,31	2,31	0,70	7,48	12,00
0,00	0,00	0,00	0,00	0,00
1,86	0,00	0,75	9,45	13,19
0,71	0,00	0,32	12,54	12,43
1,28	0,32	0,38	4,22	9,31
0,90	73,87	2,41	10,72	6,97
0,00	0,00	0,54	6,15	5,42
0,40	0,00	0,00	5,12	12,68
0,00	1,29	0,21	5,82	13,72
0,00	0,00	0,55	4,42	6,08
1,41	0,00	0,80	1,20	5,60
0,00	0,00	0,00	7,36	6,35
0,00	0,56	0,00	5,80	11,92
0,00	0,00	0,82	7,38	12,30
0,00	0,00	0,00	2,51	6,15
0,00	0,00	0,00	4,08	14,80
0,60	0,00	0,00	3,66	9,35
0,00	0,00	1,17	7,33	11,44
0,00	0,00	0,00	8,20	7,10
0,00	0,00	0,00	14,19	7,10
0,75	0,00	0,20	7,46	8,47
0,00	0,00	0,54	5,95	9,19
2,27	2,27	0,00	8,23	12,66
1,49	2,99	1,14	5,95	7,09
0,00	0,00	0,37	13,74	1,83
2,67	0,00	0,00	4,42	9,64
0,00	4,05	0,40	9,56	11,16
0,00	0,00	0,21	12,58	8,39
0,00	0,00	0,00	6,01	5,58
0,00	0,00	1,09	10,04	10,92
3,21	0,80	0,69	8,79	10,29
0,63	0,00	0,33	8,28	11,26
0,00	4,48	0,69	8,78	12,70
0,00	0,00	2,00	13,75	9,87
1,44	1,44	0,00	10,05	10,27
0,00	0,00	0,00	12,12	9,09
0,00	0,00	0,00	10,58	7,69
0,00	0,00	0,00	7,38	9,02
0,00	0,00	0,00	10,23	12,50
0,00	0,00	0,00	0,00	0,00
0,00	0,00	0,00	13,33	14,44
0,00	0,00	0,79	6,35	10,32
0,00	0,00	0,26	2,36	8,64
0,00	0,00	0,00	9,52	15,48

0,00	0,00	0,73	11,69	9,26
0,00	0,00	0,84	10,82	10,20
1,20	0,00	0,82	12,82	6,16
0,00	0,00	0,25	7,01	8,49
0,51	0,51	0,97	8,87	10,16
0,29	0,00	0,27	9,63	9,46
0,00	0,62	0,09	8,52	11,46
0,00	0,00	0,00	8,57	10,77
5,88	8,82	2,75	0,92	11,01
0,00	0,00	0,73	2,92	3,65
0,00	0,00	0,64	10,23	9,59
0,00	0,00	1,04	15,03	6,22
0,00	0,00	0,60	9,37	10,27
0,00	0,00	0,00	6,87	10,69
0,00	0,00	1,71	8,57	8,00
0,00	0,00	0,35	11,75	10,35
0,00	0,00	0,23	6,86	8,01
0,00	0,00	0,82	8,70	10,05
0,00	0,00	3,17	13,89	8,73
0,00	0,00	0,00	18,71	5,85
0,00	0,00	0,00	10,43	11,30
0,00	0,00	1,68	10,06	7,82
0,00	0,00	1,08	13,36	6,86
0,00	0,00	0,93	8,80	12,04
0,00	0,00	3,39	13,56	3,95
0,00	0,00	1,62	9,06	12,94
0,00	0,00	0,75	14,34	6,79
0,00	0,00	1,33	8,85	11,50
0,00	0,00	0,33	13,40	11,11
0,00	0,00	0,00	9,69	11,76
0,00	0,00	0,00	5,13	10,26
37,80	12,20	0,00	7,51	10,04
0,00	0,00	1,03	4,62	12,82
9,80	0,00	0,14	7,87	11,66
2,25	0,00	0,49	14,08	7,59
0,00	8,53	0,22	6,99	8,52
0,00	0,00	1,54	14,29	8,49
0,00	0,00	0,82	8,82	8,66
0,00	15,56	0,67	10,89	13,33
0,00	0,00	0,70	11,27	8,63
0,00	0,00	0,00	4,41	9,56
0,94	0,00	0,00	6,12	7,58
0,00	0,00	0,00	1,89	4,25
0,00	2,63	0,47	3,76	11,27
0,40	0,00	0,45	7,76	11,70
0,00	0,00	0,51	10,53	13,24
0,00	0,00	0,22	11,58	10,47
0,00	0,00	0,00	8,92	13,73
0,49	0,00	0,33	10,44	9,62
0,00	0,00	0,00	3,65	12,34

0,00	0,00	0,68	12,68	7,02
3,42	0,68	0,00	6,30	8,73
0,00	0,48	0,00	7,99	7,99
0,00	0,00	0,78	6,20	15,50
0,00	0,00	0,22	5,22	18,48
0,00	0,00	0,00	4,37	16,09
1,00	0,00	0,35	5,58	9,25
0,22	3,45	0,46	6,11	13,00
0,00	0,00	0,17	5,49	5,99
0,00	0,00	1,48	11,32	9,10
0,00	0,00	0,90	9,14	6,82
0,00	1,90	1,98	10,54	10,72
0,00	0,00	0,35	8,85	14,76
0,00	0,00	0,48	8,70	9,34
0,00	3,70	1,28	10,26	14,10
0,00	0,00	0,00	13,41	4,88
0,00	0,00	0,00	5,42	9,93
0,00	0,00	0,32	5,47	12,22
0,36	0,00	0,15	6,71	17,14
0,00	0,00	0,00	0,78	2,91
0,00	0,00	0,24	11,67	10,24
0,00	0,00	0,16	8,23	11,39
0,41	0,00	1,18	7,73	12,19
0,00	0,00	0,16	6,23	11,99
0,00	2,86	0,90	6,68	9,57
0,00	0,81	0,62	10,07	11,82
0,00	0,00	0,17	7,23	13,61
0,00	0,00	3,07	12,03	10,22
0,00	1,30	0,41	15,64	6,58
0,00	0,00	0,00	0,60	4,17
0,00	0,00	0,28	4,46	11,30
0,00	0,00	0,00	3,63	4,79
0,00	0,00	0,00	3,03	7,44
0,00	2,08	0,63	8,75	10,63
0,00	0,00	3,21	5,54	8,45
26,60	0,00	0,27	5,76	9,38
0,00	1,32	0,73	9,89	5,13
98,84	0,00	0,73	22,34	8,06
96,20	1,27	1,54	13,51	14,67
1,84	0,37	0,43	10,40	12,11
0,00	0,00	0,00	8,82	8,82
0,00	0,00	0,56	9,04	14,12
0,00	38,10	0,00	12,50	6,25
0,00	0,00	0,00	14,29	4,76
0,00	9,09	0,00	9,09	6,82
0,00	0,00	0,85	4,24	11,02
0,00	0,00	0,00	1,33	10,67
0,00	0,00	1,75	13,16	8,77
0,00	0,00	0,00	0,00	0,00
0,00	0,00	0,00	11,28	11,28

0,00	1,19	1,32	8,70	7,37
0,00	0,00	0,00	0,00	0,00
0,00	0,00	0,00	2,09	2,61
0,00	0,00	0,00	0,98	3,10
0,58	0,00	0,18	1,43	3,75
0,00	0,40	0,26	1,92	3,21
0,00	0,00	0,00	2,76	5,01
0,66	0,00	0,00	2,31	4,41
0,00	0,00	0,00	2,47	5,59
0,00	0,00	0,00	2,41	5,00
0,00	0,00	0,00	2,69	4,60
0,00	0,00	0,16	0,63	2,54
0,00	1,40	0,11	2,51	4,45
0,38	0,00	0,24	2,05	5,43
2,19	0,00	0,00	3,51	3,75
0,00	0,00	0,00	5,04	7,66
0,99	0,33	0,21	1,25	6,68
0,94	0,47	0,60	2,84	3,89
1,42	0,00	0,24	2,23	3,29
4,52	0,50	0,17	1,55	6,01
1,73	2,31	0,09	3,15	4,50
0,00	0,00	0,30	5,02	7,60
0,00	0,00	0,00	3,98	4,34
0,48	0,00	0,17	2,48	4,46
0,52	0,00	0,32	7,47	8,44
1,08	0,00	0,32	8,04	12,86
0,43	0,00	0,00	3,13	6,74
0,79	0,00	0,12	1,84	4,53
0,00	0,00	0,00	1,94	2,97
0,00	0,75	0,00	1,58	4,74
0,44	0,44	0,00	2,74	5,02
0,00	0,37	0,00	2,97	3,66
0,00	0,00	0,00	1,84	4,29
0,38	0,00	0,00	1,65	4,25
0,00	0,00	0,12	2,15	5,73
0,00	0,00	1,56	14,84	3,13
0,00	0,00	0,00	1,74	3,26
0,00	0,00	0,25	4,70	5,69
0,00	0,00	0,00	2,04	5,83
0,00	0,00	0,00	0,00	0,00
0,00	0,00	0,00	1,78	2,82
0,42	0,00	0,17	2,01	5,20
0,00	0,00	0,00	4,23	4,93
0,00	0,00	0,29	2,65	7,08
0,00	0,00	0,36	3,76	5,01
0,00	0,00	0,00	1,21	2,41
0,67	0,00	0,00	2,28	6,16
4,76	0,00	0,75	5,97	8,21
0,32	0,00	0,11	1,55	3,11
3,28	0,00	1,75	3,51	7,60

0,00	0,00	0,00	4,08	2,86
0,65	0,00	0,22	2,18	4,46
0,00	0,00	0,09	2,00	5,38
1,81	0,00	0,00	2,96	5,66
1,94	0,00	0,00	1,03	3,35
0,00	0,00	0,81	7,66	14,52
0,00	0,00	1,55	10,53	11,46
0,00	0,67	0,00	2,21	4,66
0,00	0,00	0,00	0,00	0,00
0,00	2,38	0,36	7,01	10,25
0,00	0,00	0,00	5,76	7,54
0,00	0,49	0,00	3,48	8,70
0,00	0,00	0,00	6,06	7,07
0,70	0,00	0,00	1,97	6,76
0,41	0,00	0,33	3,13	9,54
0,00	0,00	0,45	12,27	7,73
0,00	0,00	0,39	9,38	9,77
0,00	0,00	0,39	6,10	12,60
0,00	0,69	0,41	7,82	10,70
0,00	0,00	0,26	5,42	7,27
0,00	0,00	0,58	7,87	8,64
0,00	0,85	0,00	6,85	7,83
0,00	0,00	0,26	7,54	12,70
0,00	0,00	0,00	7,41	7,78
0,00	0,00	0,00	2,56	7,33
0,00	0,00	0,66	8,08	10,30
0,00	0,00	0,54	9,38	9,38
0,00	0,00	0,37	10,66	10,66
0,00	0,00	0,44	7,49	11,31
0,31	0,00	0,00	7,62	11,48
0,00	0,00	0,00	6,87	8,35
0,28	0,00	0,36	4,84	10,95
3,94	0,00	0,00	8,05	7,82
0,00	0,43	0,64	5,15	8,75
0,00	0,00	0,00	2,15	9,54
0,00	0,00	0,36	5,39	9,46
0,00	0,00	0,13	5,74	8,88
1,06	0,00	0,00	7,94	12,06
0,33	0,00	0,10	5,91	7,09
0,00	0,00	0,00	6,29	9,38
0,51	0,00	0,08	3,89	7,69
1,31	1,75	0,39	4,94	6,49
0,00	0,00	0,00	2,04	8,62
0,95	1,42	0,15	5,38	8,14
0,00	0,33	1,03	9,05	9,61
0,00	0,00	0,67	10,37	11,20
0,00	0,42	0,37	8,01	8,38
0,00	0,00	0,33	7,89	12,01
1,08	1,08	0,73	7,39	9,05
0,00	0,00	0,31	3,37	8,28

0,00	0,00	0,30	9,08	11,31
0,00	0,00	0,75	9,39	10,73
0,00	0,00	0,00	4,42	8,99
1,07	0,53	0,46	8,59	11,35
0,00	0,00	0,28	10,81	10,25
0,00	0,00	0,36	6,77	13,10
0,00	0,55	0,16	10,68	11,30
0,00	0,00	0,58	8,41	14,70
1,23	0,61	0,57	8,00	11,81
0,46	0,91	0,84	9,34	11,16
0,59	0,30	0,27	6,06	9,62
0,00	0,00	0,00	8,99	14,39
0,00	0,39	0,82	7,97	10,90
0,00	0,00	0,33	9,16	7,95
0,00	0,00	0,63	6,13	10,06
0,00	0,00	0,00	5,49	10,82
0,00	0,00	0,29	6,01	10,44
0,00	0,00	0,12	6,37	10,99
0,00	0,00	0,14	4,77	8,38
0,00	0,00	0,47	4,30	7,33
0,00	0,00	0,07	2,79	5,37
0,00	0,00	0,00	2,68	8,04
0,66	0,33	0,10	2,85	6,61
0,00	0,00	0,17	9,06	13,68
0,54	0,00	1,21	5,43	8,65
0,34	0,34	0,21	7,22	8,58
0,00	0,00	0,09	4,42	6,26
0,00	0,00	0,00	3,13	7,34
0,00	0,00	0,09	6,15	8,52
0,00	0,00	1,52	7,71	7,23
0,00	0,00	0,18	5,77	10,29
0,00	0,00	1,10	6,59	12,09
0,00	0,00	0,00	4,80	14,40
0,00	0,00	0,25	7,00	11,88
0,00	0,00	0,17	6,78	9,57
0,00	0,00	0,49	8,35	12,77
0,00	0,00	2,27	12,40	8,67
0,79	0,39	1,92	11,51	10,38
0,00	0,00	0,80	12,40	7,60
1,35	0,68	0,00	8,35	13,19
0,56	0,56	1,63	10,91	9,61
0,00	0,00	0,93	6,95	8,51
0,00	0,00	0,72	6,52	13,04
0,00	0,00	0,50	5,74	9,48
0,00	0,00	0,00	7,01	11,21
0,00	0,00	2,22	11,41	7,73
0,00	0,00	0,77	5,01	14,25
0,00	0,00	0,29	7,71	8,98
0,00	0,00	0,47	7,25	11,81
0,61	0,00	0,09	6,87	9,98

0,00	0,00	5,13	14,62	13,59
0,00	0,00	0,77	10,40	11,39
0,00	0,00	0,52	11,46	9,20
0,00	0,99	0,45	7,46	8,66
0,00	0,00	0,40	6,18	6,97
0,00	0,00	1,07	5,81	7,34
0,32	0,00	0,79	6,72	7,21
0,00	0,00	0,34	8,85	11,12
0,00	0,00	0,00	7,64	10,47
0,00	0,00	0,47	10,26	10,26
0,00	0,00	0,17	8,36	10,00
1,04	0,52	0,25	6,87	12,57
0,00	0,00	0,38	10,59	13,11
0,00	0,00	0,00	7,98	13,48
0,00	0,00	0,00	8,14	9,30
0,00	0,48	0,44	7,76	9,08
0,53	0,00	0,16	8,00	5,28
1,93	0,00	0,68	7,25	11,66
0,00	0,00	2,91	6,80	10,68
0,98	0,49	0,54	9,15	9,42
0,98	0,49	0,26	9,02	7,84
0,00	0,00	0,41	7,86	9,21
0,00	0,00	0,00	8,08	12,12
0,00	0,00	0,66	3,95	13,67
0,00	0,28	0,39	5,02	10,83
0,00	0,00	0,36	7,10	12,84
1,08	0,00	0,50	9,14	10,30
0,00	0,00	0,97	10,56	8,98
0,00	0,00	0,00	9,30	8,45
0,00	0,00	0,16	8,32	9,42
0,00	0,00	0,19	6,89	5,21
0,00	0,00	0,14	2,37	10,06
0,00	0,00	0,21	13,25	14,10
1,32	0,00	0,22	7,38	12,05
0,00	0,00	0,47	11,23	8,89
0,00	0,00	0,16	11,82	8,40
0,00	0,00	0,92	10,18	10,34
0,30	0,60	0,10	4,21	9,00
1,71	0,00	0,41	3,16	8,38
0,00	0,00	0,67	13,47	11,95
0,00	0,00	0,38	9,40	11,28
0,00	0,00	1,20	10,61	7,47
0,00	0,00	0,00	2,23	7,23
0,00	0,00	0,23	6,10	7,46
0,00	0,00	0,00	2,48	9,59
0,00	0,00	0,00	5,68	9,35
0,26	0,26	0,41	5,60	7,95
0,00	0,00	0,18	7,92	11,52
0,00	0,48	0,38	5,07	9,45
1,43	0,57	0,09	3,90	4,86

0,00	0,00	0,30	4,97	9,03
0,00	0,00	0,00	4,91	6,23
3,21	0,71	0,00	2,87	5,64
7,55	5,66	0,00	5,88	5,88
0,99	0,00	2,66	13,02	4,73
0,00	0,00	0,00	4,19	8,38
0,53	0,00	0,15	1,95	12,14
0,00	0,00	0,17	7,10	8,91
0,27	0,00	0,41	5,82	8,86
0,00	0,00	0,15	6,59	12,45
3,05	0,00	0,12	6,32	7,84
0,00	0,00	0,38	4,69	9,07
0,00	0,00	0,26	2,81	6,12
0,00	0,00	1,34	11,58	11,80
0,00	0,00	1,40	14,39	9,36
2,36	1,89	1,27	6,92	10,88
0,00	0,00	1,22	15,14	7,13
0,00	1,47	0,44	6,21	12,20
5,65	0,71	0,32	4,98	9,75
1,75	0,00	0,09	6,38	9,43
0,00	0,95	0,99	9,65	5,20
0,00	0,00	0,57	7,79	6,94
0,00	0,00	0,12	5,88	10,29
0,00	0,00	0,29	7,31	11,03
0,00	1,27	0,26	7,77	11,07
0,00	0,00	0,16	8,77	9,84
0,00	0,00	0,63	8,02	10,22
0,00	5,65	0,21	6,64	12,85
0,00	0,00	0,46	7,37	11,21
0,00	0,00	0,47	6,10	9,58
0,00	0,00	0,49	7,82	8,44
0,34	6,48	0,52	8,44	11,25
0,00	1,98	0,46	6,54	13,31
0,00	0,00	0,92	8,85	10,83
0,94	1,89	1,23	9,57	7,72
0,00	0,00	0,00	6,21	4,73
0,51	0,00	1,06	7,26	11,35
0,00	0,00	1,08	11,89	9,01
0,00	0,00	1,35	9,37	10,09
0,00	0,00	0,80	7,91	7,91
0,00	0,00	0,38	9,67	11,58
0,00	0,00	0,24	9,45	10,55
0,40	0,00	0,36	11,41	10,92
0,39	0,00	0,47	7,01	12,97
3,28	0,73	0,00	12,70	8,84
2,68	0,00	0,47	7,22	11,97
2,02	0,00	1,09	9,90	10,63
0,00	0,37	0,74	13,74	9,41
3,26	3,99	0,74	8,11	10,64
0,00	2,26	0,00	0,68	13,48

0,00	3,57	1,61	8,94	7,87
0,90	8,36	0,52	2,66	15,46
0,28	1,65	0,00	0,65	12,74
1,69	0,00	0,95	6,17	10,92
1,05	0,35	0,00	3,34	6,10
0,00	0,00	0,20	9,92	10,12
0,00	0,00	0,93	7,70	12,60
0,00	2,99	1,02	8,80	12,76
2,94	2,94	0,22	10,31	10,76
0,00	10,10	1,69	11,75	12,06
0,81	0,40	0,71	10,34	12,81
3,07	2,13	0,86	9,54	11,27
5,61	0,00	1,53	10,03	9,75
0,00	0,00	0,14	7,91	9,18
1,30	0,00	1,85	9,59	5,17
0,00	1,18	1,07	10,34	9,80
0,00	0,00	0,25	8,31	9,41
0,00	0,00	1,04	7,88	7,77
0,00	0,00	0,09	5,43	11,70
0,76	0,00	0,24	8,43	13,01
0,00	0,00	1,04	7,25	7,54
0,00	0,47	0,59	5,86	13,32
0,00	0,00	0,20	3,94	7,97
0,00	0,00	0,34	11,60	10,34
0,91	0,00	0,90	7,85	10,83
0,00	0,00	0,48	11,61	8,27
6,97	0,50	0,33	5,71	10,11
0,00	0,00	0,00	5,00	0,00
0,00	0,00	0,00	4,94	5,74
0,00	0,00	0,30	4,27	5,77
0,00	0,32	0,64	9,63	9,52
0,73	0,00	0,66	7,66	8,53
0,00	0,00	0,00	2,84	14,20
0,00	0,00	0,34	5,11	9,42
2,63	0,00	0,45	6,47	10,98
0,00	0,00	0,36	7,01	10,76
0,00	0,00	0,79	6,30	7,09
0,00	0,00	0,26	6,44	13,38
0,00	0,28	0,20	6,15	10,12
0,00	0,00	0,00	1,39	4,54
2,56	0,00	0,16	7,33	7,65
0,00	0,00	0,00	0,00	0,00
0,00	0,00	0,00	4,38	13,92
0,00	0,00	0,00	1,94	7,10
0,00	0,00	0,00	2,23	7,18
0,00	0,00	0,00	2,56	4,75
8,99	0,56	0,50	7,10	10,40
0,00	0,47	0,00	3,82	9,38
3,01	1,00	0,41	2,26	6,28
0,00	1,27	0,25	6,90	8,91

0,84	0,00	0,13	3,30	6,73
0,00	0,00	0,00	6,69	13,58
0,00	0,00	0,38	7,20	12,50
0,00	0,00	0,19	2,88	10,58
0,00	0,00	0,00	8,62	8,62
0,00	0,00	0,00	9,30	8,84
0,00	0,00	0,29	2,21	7,95
0,00	2,81	0,74	13,13	8,16
0,00	0,00	0,00	13,41	10,50
0,00	0,00	0,78	8,07	11,46
0,00	0,00	0,00	7,41	11,11
0,00	0,00	0,00	8,59	9,82
0,36	0,00	0,22	6,17	9,32
0,00	0,00	0,36	6,57	6,20
0,00	0,00	0,00	8,24	12,64
0,36	0,00	0,11	10,59	9,03
0,90	0,00	0,00	1,02	10,77
0,00	0,00	1,48	6,80	8,88
0,00	0,00	0,93	7,41	11,11
0,00	0,00	0,00	6,16	7,58
0,00	0,00	0,44	8,13	9,45
0,00	0,00	2,06	11,34	12,37
0,00	0,00	0,00	2,12	5,82
0,00	0,00	2,35	15,29	4,71
0,00	0,00	0,63	11,95	12,58
0,00	0,00	0,23	4,39	8,78
0,00	0,00	0,85	8,49	10,33
0,49	0,00	0,16	6,70	9,81
0,00	0,00	0,00	7,39	11,70
0,41	0,00	1,47	9,71	7,86
0,00	0,00	0,20	5,09	5,30
0,53	0,00	0,98	9,14	6,85
0,00	0,00	0,00	10,40	12,14
0,00	0,00	0,00	12,50	10,71
2,70	0,00	1,65	7,44	9,92
0,65	0,00	0,20	5,50	9,98
2,80	0,00	0,56	14,44	9,44
0,37	0,00	0,00	6,96	9,69
0,00	0,00	1,98	7,92	6,93
5,62	1,12	0,29	8,45	7,29
0,00	0,00	0,42	4,17	9,58
0,00	0,00	0,00	7,60	14,00
0,00	0,00	0,00	8,96	11,34
0,00	0,00	0,74	10,29	7,84
0,00	0,00	0,61	10,03	9,42
0,00	0,00	0,00	8,06	12,10
4,00	0,00	0,00	8,05	9,20
0,00	0,00	0,68	12,27	13,12
3,08	0,00	0,44	9,78	11,11
0,00	0,00	0,00	7,04	14,79

0,00	0,00	1,66	10,90	7,82
0,00	0,00	0,30	9,45	8,84
0,00	0,00	2,19	11,48	8,47
0,00	0,00	1,46	9,04	10,20
0,00	0,00	0,00	10,67	10,67
0,00	1,09	0,49	9,02	11,80
2,33	2,33	0,00	6,08	10,14
0,00	0,00	0,49	10,78	9,31
0,00	0,00	0,85	2,97	11,02
0,00	0,00	0,48	10,24	9,76
0,00	5,77	0,64	14,10	8,97
0,00	0,00	0,63	9,43	9,43
0,00	0,00	0,50	9,90	10,89
0,00	0,00	0,00	1,69	6,18
0,00	0,00	0,59	13,41	12,82
1,52	0,00	1,94	13,17	8,21
0,00	1,30	0,37	8,96	9,33
0,00	1,12	2,33	13,67	9,33
0,00	0,00	0,74	9,23	7,01
0,00	0,00	0,11	5,93	15,47
0,86	0,00	1,03	4,84	9,53
0,00	0,00	0,00	0,88	12,28
0,00	0,00	0,00	6,32	12,63
0,00	0,00	0,00	4,97	11,65
0,00	0,00	0,00	3,61	12,47
0,00	0,00	0,94	11,25	8,59
0,00	0,00	0,00	7,68	10,24
0,33	0,00	0,64	6,94	10,03
0,00	0,85	0,64	8,43	12,77
0,55	0,00	0,16	10,51	10,34
0,00	0,00	0,27	8,69	11,63
0,00	0,00	0,00	5,73	11,46
0,66	0,00	0,00	6,35	11,89
0,00	0,00	0,00	8,33	9,43
0,00	0,00	0,00	10,58	10,10
0,00	0,00	0,13	6,95	3,93
0,00	0,00	0,22	3,46	10,59
0,00	0,00	0,33	8,53	11,20
3,77	4,40	1,33	11,55	11,93
0,00	1,28	0,13	10,21	11,11
15,24	0,00	0,29	7,66	12,86
0,57	0,00	0,36	9,68	11,47
0,36	2,15	0,54	7,99	13,17
0,00	4,90	0,53	11,11	12,59
5,39	0,60	0,55	9,52	12,64
0,00	0,00	0,13	4,17	9,15
0,00	0,00	0,27	4,92	10,66
0,00	0,00	0,28	11,13	9,86
9,33	0,00	0,62	2,47	3,40
0,00	0,00	0,20	2,20	7,60

0,97	0,00	0,20	4,31	7,52
0,00	0,00	0,19	10,02	11,32
6,19	0,44	1,00	11,07	8,83
0,00	0,00	0,00	2,16	18,11
0,00	0,00	0,36	7,17	8,00
43,18	0,00	0,00	7,14	9,71
0,00	0,00	0,00	10,97	7,74
0,00	0,00	0,84	7,56	8,40
0,00	0,00	2,02	17,17	7,07
0,00	0,00	1,61	8,06	1,61
14,81	0,00	0,00	7,00	10,00
5,56	0,00	0,00	7,58	9,09
0,00	0,00	0,00	7,14	17,86
4,35	4,35	0,00	10,45	11,94
0,00	0,00	0,00	13,51	13,51
0,00	0,00	1,94	10,32	8,39
0,00	0,00	0,00	12,34	14,94
0,00	0,00	0,00	2,86	8,57
0,00	0,00	0,00	9,68	16,13
0,00	0,00	0,00	7,86	16,16
0,00	0,00	0,80	17,60	5,60
0,00	0,00	0,00	5,93	13,33
0,00	3,45	0,00	12,50	11,36
0,00	0,00	0,00	5,41	2,70
0,00	0,00	1,19	5,95	14,29
0,00	0,00	0,71	6,74	13,83
1,39	0,00	0,45	5,43	13,12
0,00	0,00	0,00	9,68	9,68
0,00	0,00	0,00	6,98	11,63
0,00	0,00	0,00	4,71	14,12
0,00	0,00	0,00	4,75	8,10
0,00	0,00	0,78	6,25	12,50
3,13	6,25	0,00	10,28	9,35
0,00	0,00	0,00	16,67	8,33
0,00	0,00	1,49	9,29	12,64
13,33	0,00	1,19	5,95	14,29
0,00	0,00	1,11	7,78	5,56

RSR_3	RSR_5	RSR_10	RSR_15	RSR_20
4,80	9,92	12,00	2,72	2,56
5,41	9,23	7,43	1,35	0,90
2,11	4,98	13,90	4,68	3,93
1,40	5,60	12,04	5,04	5,04
3,97	8,53	11,32	4,71	3,68
3,03	1,90	0,66	0,19	0,09
2,25	0,41	0,61	0,00	0,00
1,46	4,14	6,57	2,43	8,27
1,28	3,13	6,11	3,55	4,40
1,49	3,97	7,93	4,13	5,95
4,41	8,66	8,66	1,32	2,35
2,34	8,11	8,58	4,21	2,18
4,11	5,54	13,14	2,05	2,67
3,03	6,34	11,17	3,72	3,86
3,21	4,33	12,02	4,33	4,33
1,76	3,89	7,16	3,02	5,78
1,60	2,75	8,92	3,43	2,29
0,78	2,82	8,15	2,19	4,70
0,92	1,84	5,30	2,76	8,29
1,73	3,46	8,65	3,65	7,69
1,00	0,78	4,33	3,22	7,56
3,97	7,71	14,95	5,14	3,50
2,74	3,49	8,73	3,49	6,73
1,39	2,93	7,55	3,08	5,39
1,81	3,23	10,69	5,65	4,84
0,32	1,58	5,22	1,74	6,65
0,10	0,90	1,61	0,90	2,21
4,12	5,79	4,81	0,98	0,88
5,49	6,66	6,54	1,05	1,75
4,85	5,46	4,54	1,03	0,72
0,00	0,00	0,00	0,00	0,00
4,99	4,19	4,39	0,40	0,70
3,04	4,01	4,29	0,83	0,55
3,86	6,92	5,64	0,97	0,64
4,01	4,68	3,34	0,53	0,53
7,28	7,28	3,40	1,94	0,49
2,87	4,21	7,46	3,25	1,53
3,50	5,14	8,64	1,42	0,77
5,02	4,80	5,68	0,44	0,44
4,52	4,19	4,19	0,17	0,34
3,97	3,88	2,12	0,18	0,26
4,21	3,59	2,36	0,21	0,31
5,48	2,30	2,30	0,51	0,26
4,97	2,20	2,08	0,12	0,12
1,80	0,47	0,28	0,00	0,09
3,07	6,00	6,00	1,53	0,70
3,35	4,58	5,81	1,23	0,53
2,75	5,84	11,33	3,09	3,78
3,82	7,80	9,94	2,29	1,83

4,03	9,60	10,94	2,88	2,88
3,45	6,62	9,24	4,69	3,31
2,75	7,31	12,10	3,83	4,67
3,77	7,19	9,25	3,08	4,79
4,77	5,38	10,46	3,08	0,92
5,11	6,33	8,52	1,46	1,70
5,36	7,74	7,34	2,98	1,39
4,64	4,78	5,60	0,82	1,23
4,98	4,11	5,35	0,87	0,37
4,54	6,51	6,11	1,38	0,20
4,14	7,21	3,78	0,54	1,08
9,01	8,67	5,72	1,21	0,17
4,33	5,07	7,61	1,79	1,94
2,95	3,63	9,53	3,14	3,73
2,58	5,75	6,94	2,68	1,59
4,38	7,37	8,37	2,79	0,60
3,00	2,31	5,54	1,39	0,92
4,03	4,28	5,54	1,76	0,76
8,17	5,81	6,35	1,45	1,09
4,56	9,01	9,33	1,48	0,74
2,35	3,91	13,89	3,33	6,46
1,19	2,56	4,26	2,73	5,45
0,73	3,40	8,63	3,89	6,93
2,34	4,67	10,59	4,83	3,27
1,75	4,00	8,86	4,49	7,24
1,15	3,68	8,17	4,37	4,49
1,37	2,93	7,90	2,63	6,73
2,00	4,73	12,55	4,18	6,00
1,80	3,35	7,35	2,84	3,87
3,57	4,76	9,52	4,29	4,29
2,19	5,06	12,31	6,16	6,29
1,25	1,88	9,81	5,22	7,31
0,53	1,23	5,95	2,45	7,71
2,86	5,32	13,37	4,91	5,73
3,21	1,92	14,74	9,62	7,05
1,54	4,50	9,47	3,55	5,21
0,89	3,12	9,81	4,75	5,05
1,23	2,47	8,17	5,08	6,78
1,81	5,11	13,34	3,46	5,11
4,24	5,34	9,71	3,01	3,56
4,06	9,47	12,78	3,01	2,41
3,16	4,07	8,86	3,87	3,16
3,56	5,35	12,03	3,12	3,12
3,89	5,60	10,71	2,92	4,14
4,31	7,69	9,38	1,69	2,62
2,60	4,79	11,10	5,07	5,62
1,12	4,37	10,61	3,62	6,37
2,40	4,80	12,16	4,48	4,80
4,22	5,81	12,06	4,51	3,05
0,95	3,26	11,01	5,57	4,89

1,05	3,68	9,47	2,89	7,63
0,61	1,09	4,98	4,25	7,04
1,74	3,86	10,59	3,86	6,10
2,64	4,35	10,58	4,51	5,44
3,55	6,04	13,01	4,34	2,89
2,53	5,20	10,55	3,38	5,06
3,33	7,95	9,74	4,62	4,10
3,10	2,88	9,75	3,99	5,09
1,37	1,82	8,05	3,50	8,51
2,71	7,86	13,28	5,15	4,61
1,43	6,11	11,20	3,67	5,30
2,74	6,22	12,44	3,48	5,72
3,15	5,01	12,43	4,45	4,45
2,58	6,59	11,46	4,30	4,87
1,86	4,91	10,15	5,08	5,41
2,26	3,45	11,30	6,06	6,78
1,77	2,03	11,65	5,57	7,34
2,59	6,03	12,93	6,90	4,31
2,83	3,97	19,83	3,97	6,80
1,64	2,35	9,62	7,28	10,80
0,00	0,00	0,00	0,00	0,00
2,29	5,05	12,39	3,21	4,13
3,81	3,60	11,65	2,33	4,24
2,97	6,49	7,03	1,89	1,62
0,00	0,00	0,00	0,00	0,00
3,11	7,44	12,11	6,23	5,36
4,34	8,46	11,93	3,25	4,34
1,59	3,18	7,64	3,92	6,36
2,67	3,02	9,43	3,56	3,56
1,69	4,43	15,40	3,59	4,85
6,15	6,56	7,79	2,60	1,37
1,16	3,64	10,41	3,47	4,30
3,07	5,36	9,77	3,45	4,79
2,79	5,26	13,62	6,09	5,47
5,84	6,44	9,26	3,42	1,61
3,17	4,23	10,92	4,23	5,63
2,29	6,88	13,75	4,58	3,44
2,56	5,88	13,42	3,32	3,32
1,19	2,12	7,41	4,50	6,61
0,92	5,87	11,56	6,06	5,87
3,11	4,44	13,33	4,00	8,44
2,99	7,05	12,61	5,98	5,56
2,25	4,50	16,67	4,50	9,23
0,66	3,06	9,41	4,60	5,91
1,53	3,93	11,14	9,17	7,21
2,15	3,65	9,23	4,08	6,44
1,76	4,39	10,83	6,59	6,59
4,04	7,52	12,95	5,43	5,57
2,54	0,51	1,78	0,00	0,25
2,38	1,13	0,25	0,00	0,00

0,62	1,09	0,93	0,00	0,00
2,28	0,60	0,36	0,12	0,00
0,00	0,00	0,00	0,00	0,00
2,00	0,53	0,93	0,00	0,00
2,67	1,23	0,21	0,00	0,21
0,29	1,46	0,44	0,15	0,00
1,08	1,98	5,41	3,60	6,13
4,43	2,53	1,27	0,00	0,00
0,71	0,18	0,18	0,00	0,00
1,67	0,22	0,33	0,11	0,11
1,28	0,21	0,21	0,21	0,21
1,33	0,40	0,00	0,00	0,40
0,99	0,22	0,22	0,00	0,11
1,86	0,14	0,00	0,00	0,14
0,95	0,79	0,32	0,00	0,00
0,94	0,59	0,00	0,00	0,00
1,91	0,57	0,38	0,00	0,00
1,83	0,26	0,00	0,00	0,00
2,52	0,00	0,25	0,00	0,00
0,24	0,00	0,00	0,00	0,00
1,71	0,51	0,17	0,34	0,00
0,51	0,10	0,00	0,00	0,10
1,61	0,64	0,00	0,00	0,00
0,72	0,00	0,00	0,00	0,00
2,29	0,57	0,19	0,00	0,19
1,29	1,00	0,14	0,14	0,14
1,50	0,00	0,25	0,00	0,00
1,03	0,69	0,17	0,00	0,00
2,68	1,44	1,05	0,00	0,00
2,76	1,38	0,57	0,00	0,00
2,09	0,87	2,09	0,52	0,17
0,73	0,58	0,00	0,00	0,00
2,58	0,74	0,00	0,00	0,00
1,94	1,21	0,48	0,73	0,00
1,67	0,61	0,15	0,00	0,00
1,73	1,16	0,00	0,00	0,87
2,12	1,55	0,00	0,00	0,14
1,55	0,31	0,62	0,00	0,31
2,99	0,64	0,43	0,00	0,00
1,67	0,76	0,46	0,00	0,15
1,46	1,22	0,00	0,24	0,00
2,15	0,51	0,41	0,10	0,00
2,17	1,03	0,62	0,10	0,00
0,26	0,09	0,09	0,00	0,00
0,30	1,51	5,12	1,81	6,63
0,86	1,35	5,64	2,70	5,51
1,18	2,13	10,19	3,79	8,53
1,38	1,84	7,81	3,22	7,04
4,01	9,03	18,56	4,52	7,86
1,30	4,72	11,07	4,72	5,86

5,43	5,26	11,02	6,25	2,14
0,69	2,62	7,17	3,45	4,41
0,64	3,45	8,18	4,22	7,42
0,33	3,32	5,65	2,33	3,99
4,08	6,79	12,64	5,30	3,53
2,14	4,55	9,77	3,88	4,28
1,38	2,75	8,61	4,82	5,59
2,99	6,97	14,43	2,99	6,47
0,90	2,93	9,91	2,93	5,41
0,91	2,93	10,05	3,66	5,85
2,89	5,30	12,29	4,10	3,61
1,92	3,45	8,81	3,32	6,13
3,77	4,97	11,13	2,91	5,14
4,25	7,25	9,50	4,00	6,00
1,76	5,27	8,79	2,54	4,30
0,26	2,89	9,19	3,67	9,71
1,16	2,68	7,33	4,07	6,40
2,13	0,00	17,02	8,51	8,51
0,96	3,08	10,98	4,62	4,05
4,08	7,48	9,52	4,76	4,76
1,49	4,05	12,79	6,18	5,76
9,15	11,97	14,44	3,17	2,82
5,17	5,62	14,38	2,70	2,25
3,85	2,56	2,56	0,00	1,28
7,81	7,19	7,81	1,88	0,31
10,25	9,84	8,20	2,46	0,82
11,56	13,83	12,02	1,36	0,68
5,33	11,21	11,40	1,65	1,29
6,25	10,94	13,02	2,08	0,26
4,78	7,70	10,48	1,39	1,69
4,41	7,64	10,13	3,08	2,35
2,59	5,46	9,41	3,55	1,09
4,00	6,78	10,26	4,70	1,39
3,99	9,98	10,20	4,21	1,11
7,43	6,14	7,43	0,57	1,14
5,94	7,37	7,91	0,54	0,54
3,85	6,15	12,31	0,00	3,08
5,15	7,28	14,03	2,66	2,66
2,86	7,32	11,25	3,04	5,00
4,34	8,93	15,56	3,06	1,02
4,50	9,01	10,88	2,44	1,50
0,72	2,88	10,09	4,50	4,86
0,28	1,10	6,75	3,86	5,92
2,66	1,84	11,89	5,94	6,35
1,29	2,71	7,00	4,14	4,86
0,51	3,58	13,41	3,19	6,51
1,77	5,30	8,45	3,54	5,11
3,82	6,25	9,90	3,30	4,17
2,57	5,40	9,38	2,70	5,01
2,05	6,16	10,70	4,11	4,69

2,73	6,53	8,65	3,64	3,03
1,29	3,16	8,91	6,03	5,60
2,02	4,03	10,70	2,48	8,37
1,27	2,86	6,84	3,66	5,72
1,36	3,22	8,04	5,57	7,05
1,18	3,31	6,39	3,91	5,56
1,17	1,68	4,86	2,35	7,20
3,88	7,54	9,91	2,37	1,29
4,29	8,80	14,90	4,97	2,26
4,50	8,86	15,32	5,11	3,90
4,01	9,12	15,69	4,01	4,38
3,45	7,62	11,80	4,36	2,00
5,68	9,02	15,36	3,67	2,17
4,39	11,91	8,46	4,08	1,88
4,75	7,24	11,09	2,26	3,39
2,75	6,02	12,22	2,93	4,30
5,52	6,17	13,96	2,27	1,95
7,68	6,93	7,87	2,25	2,06
8,31	7,58	7,09	1,22	0,49
7,09	8,91	9,72	2,23	1,62
9,34	10,72	13,32	2,45	1,68
0,97	3,57	9,40	4,21	6,32
1,77	1,36	10,49	3,41	9,13
1,49	3,65	9,73	6,62	5,95
1,97	3,51	9,13	6,46	5,90
1,56	3,90	6,23	5,45	4,94
1,52	4,28	9,67	4,70	8,15
2,16	4,06	10,03	4,19	5,46
1,20	2,75	12,71	4,30	6,36
2,50	3,76	11,11	2,97	5,16
2,45	5,93	12,89	4,77	4,64
1,83	5,32	12,81	3,66	5,66
2,47	4,24	11,48	3,89	4,24
2,91	5,81	11,93	3,36	2,91
2,46	6,73	9,70	4,66	4,66
1,98	4,89	12,70	4,63	5,69
1,95	4,99	12,04	3,80	5,53
1,72	4,29	12,35	4,29	5,83
0,91	3,40	9,30	6,80	4,54
0,62	1,62	8,21	3,98	5,47
0,56	1,81	2,79	2,79	4,04
1,32	1,32	6,98	4,15	7,17
0,90	4,31	8,62	4,49	5,39
2,05	3,51	10,98	8,05	2,49
4,49	7,73	10,47	6,98	7,48
1,11	3,48	8,07	5,54	6,65
3,01	4,35	10,70	3,34	6,35
6,24	7,28	8,15	2,77	2,08
8,76	10,95	12,77	1,82	0,73
9,64	11,24	12,25	1,81	0,40

5,96	11,13	7,99	1,10	0,94
6,93	12,67	11,09	1,78	0,40
4,60	11,51	10,74	1,53	0,51
0,71	2,01	6,98	3,67	5,09
0,83	1,99	7,81	3,99	6,31
0,82	3,95	9,12	4,49	8,16
2,26	4,52	11,45	4,37	5,72
1,05	2,02	6,57	4,03	9,55
0,64	2,88	8,63	3,83	7,03
1,57	4,89	12,13	6,07	13,31
0,00	0,00	0,00	0,00	0,00
1,60	4,24	9,90	6,60	4,81
1,85	5,68	14,49	3,69	4,26
3,33	7,62	15,24	5,71	4,44
1,05	6,29	11,76	6,17	4,42
3,54	8,66	11,02	3,94	6,30
2,82	5,44	14,11	5,65	4,84
2,08	5,19	11,95	4,55	5,97
1,98	5,60	14,00	4,28	3,46
2,08	5,43	11,50	3,99	3,67
1,29	4,63	8,49	4,12	5,15
0,75	1,50	6,89	4,34	9,73
0,91	2,92	6,14	2,52	5,44
0,14	1,01	5,64	3,47	7,66
1,90	4,04	8,68	4,76	7,49
0,72	2,41	7,71	3,37	5,30
2,38	3,06	9,18	5,78	5,78
4,53	6,95	12,69	4,23	6,65
10,05	12,50	13,32	1,90	1,90
8,60	12,53	13,51	1,97	1,97
9,77	16,80	9,38	1,56	1,17
16,73	13,62	11,28	0,78	1,17
9,03	9,39	13,72	1,44	2,17
5,73	9,25	15,20	2,20	1,98
13,13	11,82	10,50	1,53	0,66
4,41	9,42	15,03	2,61	2,61
5,76	8,63	7,73	1,98	1,26
0,66	2,24	7,76	5,13	7,24
0,96	0,64	0,11	0,11	0,00
0,61	0,61	0,20	0,40	0,00
1,14	0,19	0,47	0,19	0,00
0,63	0,31	0,31	0,31	0,00
1,57	0,57	0,57	0,57	1,00
0,19	0,00	0,38	0,00	0,19
1,56	1,34	0,11	0,33	0,33
0,60	0,30	0,15	0,00	0,00
1,93	0,55	0,00	0,28	0,00
0,71	0,44	1,15	0,62	0,98
1,54	0,71	0,36	0,24	0,59
0,97	0,75	0,52	0,00	0,07

1,63	1,02	0,31	0,00	0,10
1,55	0,78	0,97	0,19	0,58
1,58	0,73	0,73	0,24	0,73
1,62	1,87	2,37	0,37	1,00
2,23	0,71	0,41	0,10	0,41
2,73	0,55	1,09	0,00	0,00
1,93	1,14	0,79	0,09	0,00
1,98	4,25	9,07	3,97	5,38
2,03	2,40	13,47	7,01	6,64
2,54	5,58	13,58	5,96	4,95
1,48	4,44	13,83	7,16	2,72
1,24	3,16	12,98	5,64	5,53
1,20	3,77	14,07	6,52	6,69
1,62	3,70	10,39	5,54	5,43
1,80	5,06	11,91	5,28	7,75
2,77	4,26	11,49	6,17	6,38
1,47	4,98	11,10	4,64	5,44
3,01	9,03	18,94	6,73	5,49
5,24	6,80	14,95	4,85	2,91
5,81	6,88	10,32	5,16	5,59
1,87	3,73	10,58	6,02	5,39
1,31	2,45	7,99	4,08	4,89
2,77	3,46	9,34	7,61	6,57
1,58	4,16	9,61	3,30	5,16
1,87	6,34	8,96	4,10	3,92
5,04	12,20	15,92	4,24	1,59
6,25	7,57	11,18	4,28	2,63
7,34	9,37	9,87	1,77	2,53
3,36	4,03	11,07	3,69	2,01
11,07	10,18	6,96	0,54	1,07
6,36	5,65	4,06	0,53	0,71
0,00	0,00	0,00	0,00	0,00
1,22	3,97	7,03	2,65	5,19
1,07	3,46	9,32	4,66	4,26
1,59	4,37	10,93	5,37	4,77
1,20	3,71	10,65	4,67	5,02
0,33	0,65	3,92	2,61	8,17
1,69	3,82	8,66	2,36	2,92
0,94	2,15	5,92	3,50	7,27
1,49	3,57	8,93	3,57	3,27
0,91	2,28	7,76	5,59	6,39
2,70	6,85	9,19	3,96	5,95
1,37	3,44	10,65	3,95	6,36
2,65	4,69	9,59	3,67	5,71
2,15	5,58	13,73	5,58	2,15
0,60	4,23	10,66	5,03	6,44
2,86	7,24	12,00	3,05	3,81
4,81	5,00	11,67	4,07	1,48
5,89	5,89	11,59	4,67	2,64
3,30	4,33	9,48	1,86	5,57

1,28	2,56	6,78	3,48	7,33
2,36	3,99	11,07	3,45	5,44
1,08	2,64	8,51	2,28	6,12
0,79	1,05	10,47	8,12	4,45
0,00	0,00	0,00	0,00	0,00
3,03	6,22	10,53	3,03	3,35
0,00	0,00	0,00	0,00	0,00
0,40	1,41	3,03	2,42	3,94
0,36	0,18	5,01	2,15	4,29
0,17	0,69	3,02	1,81	6,12
0,71	1,66	6,04	6,64	7,82
0,50	1,51	8,68	4,78	9,31
1,55	5,88	11,61	4,02	4,18
1,31	2,79	9,36	3,12	4,11
0,52	1,04	4,68	1,91	6,24
1,10	1,29	5,06	2,48	5,24
0,00	0,00	0,00	0,00	0,00
1,17	3,39	9,64	6,38	5,47
2,47	4,51	10,32	5,67	6,40
0,93	2,52	7,75	4,39	7,19
0,98	2,49	8,35	4,62	4,62
1,52	4,21	12,29	5,22	5,89
0,55	2,47	8,22	2,74	8,22
1,10	3,87	14,09	4,70	3,59
3,08	3,93	9,74	5,30	4,79
2,07	5,05	13,34	5,05	6,22
1,53	2,23	8,62	4,31	4,45
3,42	0,99	0,27	0,09	0,00
2,03	6,08	7,09	2,03	6,42
1,40	4,75	11,73	8,10	3,91
3,80	3,80	10,23	4,97	7,89
0,46	3,04	6,23	2,13	8,36
1,05	2,09	12,22	8,03	6,46
4,64	6,19	10,82	4,12	2,58
1,91	3,44	7,07	5,16	4,97
0,82	4,09	14,17	6,54	6,27
3,93	6,00	9,93	3,93	5,54
2,26	7,92	9,95	2,71	3,39
0,91	2,95	12,95	4,32	8,64
1,61	1,43	5,18	3,75	4,46
0,69	3,46	9,17	5,88	7,61
1,30	2,97	10,95	3,90	5,75
4,79	3,38	8,45	1,97	3,10
1,07	3,22	9,01	4,51	5,58
1,57	4,44	10,70	3,92	12,27
1,64	3,01	9,29	3,28	7,92
0,68	4,44	15,02	5,46	4,78
11,28	11,59	11,28	1,52	0,91
1,91	3,47	7,80	2,77	4,51
1,15	1,64	8,03	3,28	6,56

3,02	3,94	10,67	5,10	6,73
6,54	7,48	9,66	4,05	5,92
1,78	4,66	9,74	3,43	4,12
0,34	1,72	5,40	3,22	7,36
0,35	1,41	5,11	2,46	7,75
0,38	0,94	2,25	2,44	3,75
2,21	2,43	7,74	3,54	7,96
0,98	1,95	11,22	1,95	6,34
2,95	7,08	12,98	4,72	4,42
1,95	6,61	9,73	5,06	5,84
1,51	3,18	7,02	4,18	7,53
1,39	3,08	7,86	5,08	8,78
3,13	8,13	20,00	6,25	3,75
3,90	6,06	8,66	3,03	2,31
1,83	4,23	9,30	4,65	4,65
0,22	1,10	2,87	3,75	7,28
0,32	1,29	4,31	1,94	6,03
2,96	8,89	10,74	5,56	2,59
2,74	3,74	8,73	3,99	5,99
1,67	6,11	10,56	1,94	4,17
2,88	0,96	13,46	0,96	4,81
1,30	4,00	9,96	3,46	5,63
3,34	6,38	9,42	6,08	2,74
3,30	6,29	13,52	5,66	4,56
2,43	3,40	4,85	0,97	4,85
2,35	3,84	11,94	3,20	6,82
1,74	5,23	9,30	1,74	6,40
1,20	1,20	7,45	3,37	5,77
0,62	2,47	7,25	2,31	7,10
3,13	7,50	10,42	4,79	5,63
0,00	7,69	10,26	5,13	2,56
0,00	4,94	9,88	7,41	4,94
2,10	2,80	9,09	5,59	4,20
0,00	4,21	9,47	4,21	10,53
0,40	1,39	5,75	1,98	8,93
0,70	2,11	5,16	6,34	8,22
0,00	0,00	0,00	0,00	0,00
4,46	8,92	10,19	3,82	3,82
3,99	6,98	8,64	2,33	2,66
1,25	1,88	11,88	5,00	4,38
2,52	4,40	11,01	1,57	1,26
1,18	4,72	11,20	3,93	6,48
4,38	5,00	10,00	5,63	3,75
0,00	3,85	4,49	1,92	5,13
1,96	13,73	31,37	17,65	11,76
1,27	3,30	11,93	4,82	6,85
0,00	4,05	10,14	3,38	1,35
0,99	0,33	2,97	1,65	6,60
0,00	0,00	0,00	0,00	0,00
2,84	1,42	7,09	2,13	4,96

1,44	0,21	0,41	0,00	0,21
7,28	8,61	9,27	1,99	1,32
0,00	0,90	5,41	2,70	5,41
0,00	0,00	0,00	0,00	0,00
0,62	0,31	0,00	0,00	0,00
4,83	6,97	15,01	2,68	2,14
4,20	0,84	0,84	0,00	0,00
0,50	0,00	0,00	0,00	0,50
4,58	6,11	8,40	2,67	3,44
1,20	0,80	0,00	0,40	0,00
1,10	1,10	0,22	0,00	0,00
0,56	2,38	3,78	3,08	4,76
0,00	0,00	0,00	0,00	0,00
1,89	0,86	0,00	0,00	0,00
0,94	0,27	0,54	0,13	0,00
0,35	1,04	2,42	1,38	5,88
0,31	0,93	6,19	1,24	4,95
6,64	8,20	18,36	2,34	2,34
0,47	0,00	0,00	0,00	0,00
2,86	0,86	0,57	0,00	0,00
1,98	0,49	0,74	0,00	0,00
2,48	2,13	0,98	0,18	0,44
1,85	7,75	11,81	5,90	4,06
4,02	8,03	12,25	4,22	1,61
0,00	0,00	0,00	0,00	0,00
1,99	3,48	10,70	7,46	3,48
0,52	3,13	5,73	2,08	7,29
2,22	15,56	10,00	4,44	5,56
5,50	7,89	7,66	2,15	0,96
0,72	4,32	0,00	0,00	0,00
0,00	1,39	0,00	0,00	1,39
3,27	1,31	1,31	0,00	0,65
1,67	0,00	0,00	0,00	0,00
0,00	0,00	0,00	0,00	0,00
1,85	4,07	12,01	6,10	5,73
0,41	4,08	9,25	3,81	7,21
1,38	3,68	8,74	4,37	5,75
4,59	6,28	8,70	2,90	2,90
3,13	5,36	16,07	4,24	5,80
0,45	1,79	5,38	1,35	8,52
1,07	4,11	7,69	4,65	5,90
4,45	6,36	6,74	2,29	0,89
5,04	6,10	7,50	2,70	1,29
3,42	2,53	1,78	0,00	0,15
5,87	7,48	7,36	1,15	0,23
5,99	7,32	4,88	1,55	1,55
5,01	4,11	7,71	1,54	1,03
5,42	4,76	5,58	0,99	1,31
3,03	2,23	1,44	0,00	0,32
4,09	3,88	3,45	0,86	0,43

3,01	4,51	3,96	0,68	0,55
4,49	3,37	1,12	0,00	0,00
0,00	0,00	0,00	0,00	0,00
4,41	4,88	2,68	0,31	0,00
2,95	2,27	1,70	0,34	0,00
3,07	2,04	1,31	0,58	0,15
3,28	3,67	1,54	0,39	0,00
5,19	2,70	1,97	0,21	0,21
2,90	1,18	0,86	0,00	0,21
4,08	4,08	1,98	0,00	0,37
4,74	5,72	3,43	0,98	0,33
4,89	4,41	3,81	0,48	0,12
3,66	2,93	3,22	0,29	0,29
2,66	1,88	2,98	0,16	0,00
4,74	3,42	3,68	0,92	0,13
7,79	6,36	5,88	0,48	0,16
3,42	7,10	10,52	2,05	1,09
5,52	6,60	8,44	4,29	1,69
5,63	5,63	7,92	3,13	1,88
3,36	6,94	5,89	1,37	0,84
3,21	8,33	8,65	2,08	1,60
5,89	5,32	7,03	2,28	0,57
6,04	7,91	7,19	1,58	1,73
4,40	8,28	9,96	1,26	1,05
3,53	6,24	2,82	0,94	0,47
6,62	3,07	3,55	0,48	0,16
3,59	2,84	2,27	0,76	0,57
7,32	9,98	7,54	0,22	0,22
3,87	6,81	7,89	2,17	1,08
4,92	4,81	2,11	0,82	0,47
4,91	6,21	2,46	0,00	0,29
5,35	5,14	2,78	0,21	0,43
2,86	3,02	1,75	0,16	0,16
4,78	4,78	4,40	0,57	0,19
5,91	3,89	2,01	0,27	0,13
2,71	2,03	0,54	0,14	0,27
2,44	1,70	1,59	0,11	0,11
1,65	1,05	0,90	0,00	0,00
2,39	1,04	1,97	0,62	1,14
3,50	3,88	3,50	0,39	0,19
1,30	3,17	0,74	0,74	0,56
3,53	3,06	2,70	0,71	0,35
1,81	1,41	1,61	0,20	0,40
6,80	4,27	3,69	1,17	0,19
3,67	6,72	9,05	1,59	2,20
5,70	6,14	5,48	1,32	1,32
3,93	6,01	9,20	2,70	2,33
5,15	9,02	17,01	3,09	2,06
4,15	5,22	11,38	3,21	2,54
5,64	9,30	9,60	1,83	0,30

8,03	11,24	9,63	3,44	1,61
5,01	4,89	9,55	2,10	2,79
5,04	7,24	7,11	2,07	1,29
7,50	5,48	6,06	1,01	0,43
6,05	6,49	8,58	1,10	0,88
4,30	6,09	8,84	1,79	1,43
5,65	7,02	8,58	2,14	1,56
3,45	7,16	8,49	1,33	1,59
5,25	4,39	2,89	0,64	0,11
2,76	5,07	3,23	0,00	1,38
4,22	4,34	4,46	0,60	0,96
5,53	5,28	5,41	2,39	0,50
8,58	12,23	6,11	0,49	0,39
5,21	5,11	2,61	0,42	0,31
6,19	3,94	3,26	0,34	0,34
4,48	5,83	4,07	0,41	0,54
4,60	3,52	2,80	0,36	0,18
3,02	3,23	2,80	0,22	1,29
3,77	3,43	2,06	0,51	0,34
1,26	0,84	0,21	0,00	0,00
2,13	2,13	0,97	0,00	0,58
2,67	1,58	0,73	0,12	0,00
2,69	2,17	1,28	0,51	0,13
3,91	1,63	0,54	0,18	0,18
3,68	1,74	1,74	0,29	0,00
3,85	2,28	1,40	0,35	0,35
6,74	4,58	3,23	0,40	0,40
5,05	4,85	3,23	0,81	0,20
4,55	5,57	7,33	0,59	1,32
3,58	4,77	5,17	0,99	0,60
2,91	1,35	0,45	0,00	0,22
5,36	7,79	5,36	1,38	0,87
6,18	7,01	9,62	2,88	1,37
4,34	5,58	6,82	1,45	2,89
5,59	6,34	9,52	2,57	2,42
4,44	7,53	8,06	2,42	2,02
4,19	6,54	8,63	1,73	1,11
4,50	6,45	11,31	3,41	2,68
5,47	7,92	11,70	2,83	2,26
4,19	7,62	9,40	3,43	1,65
5,31	7,63	11,28	2,16	2,16
4,13	5,62	11,74	1,65	1,16
5,81	4,74	7,24	2,14	1,19
7,97	6,47	4,85	1,62	1,27
8,02	4,22	5,27	0,84	1,27
5,62	5,26	5,74	0,84	0,60
5,33	7,20	7,20	1,33	1,33
2,55	1,53	1,36	0,17	0,00
2,88	1,92	0,84	0,12	0,12
2,43	0,43	0,29	0,14	0,00

2,49	1,40	1,09	0,16	0,62
2,78	1,16	0,23	0,00	0,00
2,27	0,60	0,36	0,00	0,12
2,91	1,51	0,60	0,00	0,00
4,18	2,53	1,27	0,25	0,00
2,56	4,09	2,90	0,00	0,17
5,41	4,60	4,47	0,54	0,54
4,93	3,67	4,93	1,15	0,46
4,50	5,36	4,33	1,38	0,87
5,33	9,33	7,17	1,50	0,83
4,03	5,93	10,38	3,18	2,97
3,13	5,48	10,44	3,05	1,22
1,71	1,84	0,39	0,00	0,00
3,80	8,02	8,86	2,67	1,69
5,27	8,08	8,08	1,58	1,76
4,46	6,51	11,08	2,65	0,84
4,12	5,72	8,78	1,60	1,46
6,58	6,44	9,13	3,22	1,48
3,06	5,58	5,58	2,34	0,36
5,39	6,23	11,11	3,03	1,35
5,27	6,02	6,77	1,61	1,40
3,27	7,57	9,82	3,07	2,66
6,57	6,75	6,10	1,88	1,50
4,79	5,94	9,90	1,16	1,82
5,66	7,12	5,75	0,82	0,73
4,62	4,50	3,16	0,61	0,36
3,58	4,66	3,58	0,00	0,18
3,65	3,65	3,22	1,29	0,54
4,20	3,71	2,64	0,78	0,00
4,15	3,27	2,07	0,32	0,32
4,91	3,16	1,53	0,44	0,22
5,28	3,10	1,49	0,46	0,00
2,76	2,30	1,68	0,15	0,31
2,41	3,55	2,28	0,25	0,13
3,51	1,50	0,50	0,00	0,00
1,97	0,36	0,18	0,00	0,18
1,75	1,20	0,65	0,00	0,09
2,17	1,28	0,64	0,13	0,26
4,02	2,49	1,78	0,24	0,24
3,00	0,96	0,48	0,12	0,00
1,41	0,88	0,18	0,00	0,00
1,65	1,65	0,41	0,00	0,00
2,15	0,60	0,00	0,00	0,00
4,84	1,38	0,35	0,17	0,00
3,36	2,02	0,56	0,00	0,00
1,84	0,79	1,05	0,00	0,00
4,42	5,82	3,73	0,70	0,81
4,40	3,55	1,56	0,28	0,28
5,10	6,47	8,54	2,20	0,55
3,82	5,73	8,16	1,22	0,69

3,77	5,76	4,88	0,67	0,44
5,50	5,50	7,93	1,13	0,49
6,95	7,70	8,01	1,81	0,30
6,27	5,72	7,22	0,54	0,27
5,24	6,83	6,38	0,91	0,68
4,00	6,15	8,10	1,66	0,78
3,33	6,82	12,98	3,00	3,49
2,90	1,70	0,60	0,10	0,10
4,12	5,36	6,80	1,86	1,24
1,78	0,59	0,40	0,00	0,00
4,31	5,65	8,75	3,10	2,83
3,91	7,23	9,77	3,32	2,15
6,13	9,82	9,82	3,68	0,61
3,25	6,50	8,94	1,63	1,22
5,56	6,02	9,26	1,62	2,31
4,17	6,25	9,24	3,65	2,08
6,97	9,16	7,74	2,32	2,06
4,96	6,80	7,51	1,98	0,85
4,30	7,09	8,61	2,78	1,52
3,04	5,67	8,10	2,23	2,02
2,51	5,77	10,61	5,03	2,33
3,53	4,56	8,20	4,78	2,51
1,81	3,63	8,16	3,76	2,98
4,63	6,12	8,30	3,13	1,36
4,64	6,43	10,36	2,68	1,61
5,50	3,21	2,52	0,46	0,23
4,94	4,32	3,46	0,49	0,12
4,06	6,26	4,50	1,10	0,55
3,70	1,99	1,14	0,19	0,19
4,37	7,60	5,32	1,14	1,52
0,00	0,00	0,00	0,00	0,00
1,21	0,35	0,09	0,00	0,00
2,10	1,53	0,67	0,00	0,00
3,34	3,43	3,05	0,95	0,00
4,98	4,61	7,56	0,55	0,55
4,70	3,76	1,53	0,59	0,00
3,98	6,27	7,47	2,77	1,81
7,26	12,35	11,62	0,48	0,73
5,70	8,81	11,92	2,07	1,38
5,32	6,89	9,86	1,72	2,66
4,61	6,46	10,01	3,03	0,92
4,29	5,60	11,08	3,46	1,91
5,93	8,57	11,65	1,32	1,32
5,17	8,31	9,21	2,70	2,70
4,35	6,16	9,06	2,36	1,63
3,77	5,42	4,95	0,94	0,94
3,20	8,10	9,23	2,26	1,69
4,87	4,51	5,42	1,81	1,62
4,69	8,12	9,04	2,17	1,49
6,67	6,11	6,85	1,11	0,37

5,25	5,74	5,01	1,13	0,73
4,34	2,48	1,71	0,16	0,00
3,07	2,30	1,15	0,00	0,00
5,16	4,19	4,03	0,48	0,08
5,11	1,46	0,73	0,24	0,00
3,93	6,29	11,20	2,55	1,96
3,59	3,59	4,67	0,36	0,72
4,16	6,52	11,12	3,03	1,57
4,13	8,38	10,74	3,19	1,06
6,94	7,47	6,05	0,71	0,89
6,23	7,25	6,67	1,59	1,59
1,65	0,55	0,46	0,00	0,00
2,38	6,25	10,71	2,98	4,02
5,42	9,29	11,76	3,10	0,46
5,28	6,51	11,62	2,11	2,11
1,94	6,98	10,27	3,68	2,33
3,89	6,08	11,44	4,38	2,43
3,54	8,00	12,34	4,11	2,40
6,52	9,49	8,30	1,58	1,58
3,30	7,76	7,61	3,16	1,72
5,71	5,41	8,42	2,86	2,11
4,22	6,33	8,77	3,25	1,95
3,91	7,19	9,53	2,97	1,09
4,21	6,40	11,70	1,56	1,72
2,95	2,09	2,95	0,86	0,00
2,15	0,54	0,20	0,05	0,00
2,17	0,93	0,52	0,00	0,10
2,73	1,73	0,36	0,18	0,00
2,70	0,90	3,60	0,00	0,00
1,82	0,67	0,10	0,00	0,00
1,30	0,65	0,11	0,00	0,00
1,34	0,67	0,27	0,13	0,00
2,24	0,81	0,54	0,00	0,00
1,50	0,27	0,27	0,00	0,00
0,89	0,45	0,00	0,00	0,00
0,81	0,45	0,18	0,00	0,00
1,90	0,73	0,15	0,00	0,00
1,01	0,84	0,50	0,00	0,00
1,02	0,38	0,13	0,00	0,13
1,53	0,35	0,35	0,00	0,00
0,72	0,82	0,10	0,00	0,00
0,47	0,24	0,12	0,00	0,00
0,55	0,37	0,18	0,00	0,00
0,62	0,21	0,00	0,00	0,00
5,02	6,76	3,47	0,39	0,19
5,85	6,38	7,45	0,00	0,00
2,70	2,70	8,11	2,70	2,70
6,42	6,12	5,81	0,61	0,92
6,34	6,95	8,59	1,02	1,84
3,14	6,07	10,04	2,72	2,09

7,08	16,81	8,85	1,77	0,88
1,88	8,45	10,33	4,23	1,88
3,96	6,04	11,67	2,50	2,50
4,71	5,46	9,68	2,98	0,74
5,34	6,03	10,90	2,55	1,39
5,37	6,40	5,99	2,27	0,83
5,76	4,32	9,35	1,68	1,20
2,01	5,63	6,84	2,21	1,81
4,12	8,06	5,02	1,08	0,90
5,17	5,67	7,39	1,23	0,74
3,76	7,04	12,21	2,35	2,82
5,16	6,88	9,98	2,07	2,07
5,51	8,14	9,97	3,41	1,84
6,56	5,74	10,66	2,46	1,02
3,73	7,25	8,49	3,31	2,48
3,69	4,49	4,98	1,44	0,96
3,39	4,60	3,39	0,97	0,24
5,23	7,19	12,42	1,63	0,98
2,94	8,09	8,09	1,47	0,74
0,83	5,79	10,74	2,48	0,83
5,08	8,47	8,14	2,37	1,02
4,38	7,20	10,17	1,84	1,41
6,98	6,20	5,43	1,55	0,78
5,47	9,00	15,43	5,14	2,25
4,72	7,39	7,86	1,26	0,79
6,27	6,27	7,25	1,57	1,76
6,74	6,74	12,36	0,00	2,25
3,18	2,39	1,19	0,00	0,00
10,17	6,78	0,00	0,00	0,00
6,15	2,87	2,87	0,41	0,00
4,47	8,94	2,23	0,56	0,00
4,20	2,80	0,70	0,00	0,00
1,57	6,49	8,95	2,46	1,12
6,25	0,00	0,00	0,00	0,00
2,62	6,67	10,71	2,14	3,57
0,00	0,00	0,00	0,00	0,00
1,81	1,55	0,26	0,26	0,00
3,39	1,69	0,00	0,00	0,00
1,53	3,07	0,00	0,00	0,00
4,49	1,85	2,90	0,53	0,53
5,33	6,40	5,60	0,80	0,00
0,00	0,00	0,00	0,00	0,00
4,10	1,64	0,82	0,00	0,00
2,82	2,23	1,93	0,15	0,30
2,05	2,56	2,05	0,00	0,00
4,10	4,49	0,98	0,20	0,00
5,81	6,31	4,15	0,33	0,33
5,51	3,01	1,67	0,33	0,17
3,32	3,88	4,71	0,28	0,28
5,50	7,21	7,59	1,14	0,76

0,84	0,28	0,42	0,00	0,00
1,69	0,42	0,21	0,42	0,00
7,14	0,00	0,00	0,00	0,00
6,25	4,17	0,00	0,00	0,00
3,57	6,25	8,04	0,00	0,00
8,33	5,73	2,08	1,56	0,52
6,59	2,98	1,88	0,00	0,16
6,21	5,73	3,10	0,24	0,48
5,62	2,62	3,18	0,37	0,00
0,00	0,81	0,54	0,00	0,00
0,00	0,00	0,00	0,00	0,00
5,19	2,46	2,19	0,55	0,00
3,15	1,35	1,35	0,00	0,00
3,85	4,20	4,20	0,35	0,17
7,50	2,50	2,50	0,00	0,00
5,50	5,36	6,03	1,61	2,01
4,27	4,47	2,03	0,00	0,41
0,00	0,00	0,00	0,00	0,00
1,05	0,35	1,05	0,00	0,00
1,84	1,67	0,67	0,00	0,00
2,70	2,16	1,35	0,00	0,00
2,33	0,98	0,37	0,00	0,00
4,10	4,10	3,36	0,37	0,00
4,06	6,09	3,55	1,02	0,00
0,48	0,72	0,48	0,00	0,00
2,33	2,33	2,33	0,00	0,00
6,00	10,25	11,27	3,42	1,75
3,89	2,83	4,24	0,00	0,00
3,54	6,69	6,69	1,57	0,39
4,94	5,19	2,34	0,26	0,26
1,10	1,10	0,55	0,00	0,55
9,62	5,77	3,85	0,00	0,00
1,47	0,65	0,00	0,00	0,00
3,67	2,50	1,67	0,33	0,33
5,01	3,62	1,95	0,84	0,00
3,02	2,19	3,43	0,27	0,14
2,38	1,11	0,32	0,00	0,00
2,60	1,08	0,22	0,22	0,00
1,90	2,53	1,27	0,00	0,63
3,40	5,48	10,59	1,32	1,32
5,89	5,51	5,70	1,14	0,38
2,15	1,58	1,00	0,00	0,00
4,30	3,23	0,54	0,00	0,00
1,84	1,64	1,02	0,00	0,00
4,07	6,40	4,07	1,16	1,16
3,67	3,67	0,00	0,00	0,00
5,24	4,14	2,56	0,12	0,12
8,91	9,72	7,29	2,43	2,43
10,32	7,94	7,14	0,00	0,79
5,98	13,04	13,59	2,72	3,80

1,87	8,13	14,80	3,20	4,67
4,44	6,66	10,26	2,77	2,36
3,85	6,36	7,84	2,37	1,78
8,21	3,24	1,34	0,19	0,00
1,61	1,61	9,68	1,61	4,84
0,00	0,00	0,00	0,00	0,00
0,67	0,22	0,11	0,00	0,00
7,45	4,35	3,11	0,62	0,62
6,35	4,72	4,72	1,09	1,63
2,32	2,14	0,53	0,00	0,18
4,01	1,63	0,74	0,00	0,00
1,32	1,32	0,99	0,17	0,00
5,05	6,06	12,63	5,05	1,77
2,32	0,70	0,70	0,00	0,00
0,43	0,43	0,00	0,00	0,00
0,95	0,00	0,32	0,00	0,00
3,08	3,08	0,00	0,00	0,00
2,73	1,82	1,82	0,00	0,00
0,86	0,00	0,00	0,00	0,00
1,02	1,02	0,13	0,00	0,00
1,76	0,00	0,29	0,00	0,00
5,17	4,58	3,84	0,00	0,74
1,42	1,10	0,47	0,47	0,47
3,17	2,18	2,67	0,30	0,49
3,47	2,51	1,93	0,39	0,19
4,52	3,23	1,94	0,00	1,29
3,64	7,15	9,85	1,21	1,89
6,81	6,15	5,15	1,00	0,83
3,97	4,71	3,38	0,15	0,29
5,46	2,84	4,10	0,46	0,11
3,47	1,73	1,45	0,00	0,00
3,69	6,14	5,90	0,98	1,97
4,44	6,19	7,94	1,88	2,56
6,21	5,88	10,13	1,96	1,31
4,26	5,54	8,66	2,41	2,84
2,73	7,19	9,21	2,16	2,16
1,50	0,50	0,66	0,00	0,00
3,39	6,27	10,68	4,41	2,20
2,71	7,18	8,93	3,67	2,23
0,00	0,00	0,00	0,00	0,00
5,08	5,93	10,38	3,60	3,18
5,67	2,23	1,72	0,17	0,17
6,75	9,95	6,22	1,24	0,36
6,35	6,78	7,22	0,98	0,33
4,38	6,88	6,72	1,25	0,16
4,35	2,18	1,97	0,41	0,10
3,73	2,72	2,02	0,00	0,00
4,32	5,51	1,84	0,54	0,43
4,20	1,50	0,90	0,15	0,15
4,91	2,06	1,27	0,32	0,16

3,61	3,61	2,46	0,49	0,33
0,76	1,13	0,00	0,19	0,00
0,00	0,00	0,00	0,00	0,00
3,13	6,77	9,38	2,08	4,17
3,55	4,82	10,52	4,06	3,04
2,14	3,45	9,64	2,98	4,88
2,66	4,96	10,46	2,66	2,13
2,95	5,74	8,03	2,46	1,64
2,49	2,35	2,63	0,28	0,42
8,13	8,61	7,66	0,96	0,48
3,93	6,95	4,83	0,00	0,30
4,27	4,98	4,98	1,42	0,71
6,16	4,22	1,37	0,34	0,11
6,55	6,00	3,64	0,18	0,18
6,25	6,25	6,25	0,00	0,00
5,79	3,64	2,43	0,28	0,09
8,42	6,71	1,72	0,00	0,00
7,79	6,13	2,81	0,38	0,00
2,38	2,38	0,00	0,00	0,00
6,46	4,76	3,06	0,34	0,11
3,69	3,27	2,11	0,32	0,11
6,48	4,45	2,63	0,00	0,00
4,42	5,30	3,54	0,25	0,00
2,49	1,00	0,12	0,00	0,00
5,12	2,83	0,35	0,18	0,00
4,42	2,87	0,66	0,00	0,00
3,39	1,13	0,56	0,00	0,00
2,12	1,14	0,16	0,00	0,00
4,33	2,41	1,44	0,00	0,00
3,23	0,40	0,00	0,00	0,00
1,32	0,11	0,00	0,00	0,00
5,64	3,04	1,01	0,00	0,14
6,72	3,84	1,06	0,00	0,10
1,01	0,00	0,17	0,00	0,00
5,01	1,72	0,92	0,00	0,00
4,98	3,22	2,09	0,16	0,00
5,74	6,30	3,33	0,93	0,56
2,00	1,64	0,00	0,18	0,00
1,01	0,17	0,00	0,00	0,00
0,66	0,00	0,22	0,00	0,00
2,79	2,61	2,09	0,17	0,00
1,28	0,40	0,32	0,16	0,16
4,44	2,66	1,44	0,11	0,11
3,15	2,87	0,96	0,19	0,00
3,33	2,11	0,89	0,22	0,22
0,75	0,38	0,19	0,00	0,00
1,05	0,21	0,00	0,00	0,00
3,47	8,52	8,52	3,15	3,15
1,16	0,58	1,16	0,00	0,00
0,00	0,00	0,00	0,00	0,00

0,46	0,37	0,09	0,00	0,00
2,05	0,51	0,51	0,00	0,00
1,43	0,11	0,33	0,00	0,00
1,57	0,35	0,17	0,00	0,00
0,76	0,00	0,00	0,00	0,00
1,81	0,45	0,23	0,00	0,00
2,43	0,81	0,00	0,00	0,00
4,47	6,64	6,51	1,53	1,02
5,10	4,71	7,77	1,78	1,91
4,26	4,04	9,15	2,34	1,06
3,34	4,26	3,95	0,00	0,00
5,54	6,64	7,97	0,89	0,89
3,73	4,94	7,35	2,17	1,45
1,17	5,44	12,62	7,38	4,47
4,40	5,37	4,89	0,49	0,33
5,68	4,34	6,51	1,67	1,50
4,35	4,91	6,81	1,89	1,51
4,54	7,71	6,69	1,81	0,57
5,05	5,71	5,49	1,43	0,77
4,34	3,72	2,11	0,25	0,12
5,53	6,43	8,35	2,71	2,14
4,84	6,51	6,20	1,97	1,21
5,12	6,45	5,50	0,95	1,71
6,27	7,06	5,49	1,57	1,96
7,31	5,25	4,61	0,64	1,11
3,63	3,42	2,46	0,32	0,43
3,69	2,17	2,17	0,11	0,11
5,18	4,75	2,92	0,76	0,65
4,74	6,08	6,08	1,96	0,51
5,96	4,34	3,43	0,81	0,10
1,40	5,61	12,98	3,68	4,21
3,53	5,93	10,83	3,31	2,74
6,12	2,04	4,08	0,00	1,02
4,41	7,23	7,58	2,12	1,59
6,98	6,70	5,56	1,71	1,71
5,35	5,08	5,61	1,87	0,00
4,67	2,92	1,95	0,78	0,19
4,40	3,66	2,66	0,46	0,09
2,36	2,36	1,30	0,35	0,00
5,18	7,49	8,60	2,50	0,55
7,64	10,74	11,16	1,24	0,21
6,43	10,42	11,09	1,77	2,22
2,07	4,87	8,64	3,28	4,14
3,01	6,94	11,33	2,54	2,77
2,73	5,82	11,65	3,35	3,72
2,08	6,98	13,40	1,51	2,26
1,99	5,63	10,20	3,40	5,86
1,55	4,21	10,42	4,88	3,55
3,70	6,00	10,73	1,66	2,04
7,43	5,94	6,44	1,65	0,50

5,13	7,03	6,36	0,56	0,45
3,85	5,98	5,78	0,91	1,11
4,46	6,14	5,30	0,72	0,48
5,81	3,95	2,96	0,55	0,22
5,00	5,56	4,63	1,30	0,56
5,20	4,21	3,71	0,50	0,00
5,06	2,25	0,19	0,00	0,00
4,86	1,82	1,72	0,51	0,00
3,15	3,27	2,55	0,12	0,00
3,83	2,50	1,83	0,00	0,17
4,32	4,32	2,52	0,96	0,36
4,32	3,50	3,73	0,70	0,35
6,47	9,26	10,91	2,28	0,89
2,88	4,88	10,76	4,26	2,63
0,53	2,73	8,94	4,52	4,00
0,50	2,27	7,30	4,03	6,17
8,06	9,97	10,11	0,82	0,55
2,70	6,69	14,37	3,13	2,42
3,00	7,11	9,00	3,48	3,32
3,33	6,77	9,17	3,21	2,64
1,66	4,64	10,10	4,14	5,63
2,91	6,40	10,33	3,35	2,18
1,30	3,59	9,38	4,09	6,39
3,81	5,98	8,14	2,78	1,86
3,52	4,73	11,04	4,13	3,76
5,86	6,29	9,76	1,52	1,08
4,67	3,76	4,33	0,91	0,34
3,91	3,75	2,12	0,00	0,00
4,36	3,43	2,49	0,31	0,00
5,47	3,76	2,05	0,51	0,34
5,99	5,75	4,19	1,44	0,24
2,01	1,15	0,72	0,14	0,14
5,33	4,07	2,23	0,29	0,00
3,28	5,40	2,75	0,53	0,32
1,65	5,52	11,21	2,47	3,87
3,73	6,83	9,68	2,40	3,29
3,79	2,84	3,22	0,66	0,09
3,40	3,09	3,29	0,72	0,31
2,83	2,95	4,99	0,68	0,79
2,07	4,60	7,42	2,54	3,76
3,76	4,81	6,79	1,88	1,25
2,57	3,95	10,37	3,65	4,74
2,07	4,94	8,79	3,65	3,46
2,26	3,93	10,12	3,57	3,81
4,64	7,06	8,61	0,44	1,10
4,90	6,30	5,72	2,68	1,52
2,57	4,71	6,64	1,07	0,43
5,19	4,29	5,06	0,52	0,26
5,32	4,92	4,12	0,53	0,80
4,52	4,13	2,06	0,39	0,00

4,78	3,08	1,71	0,00	0,23
4,47	3,49	3,63	0,14	0,42
1,88	0,88	0,13	0,00	0,00
1,39	0,00	0,46	0,00	0,12
3,70	4,86	4,28	0,58	0,78
6,71	4,47	5,49	0,61	0,20
5,37	3,36	2,46	0,78	0,00
3,79	3,79	3,99	0,40	0,20
4,96	4,84	1,69	0,36	0,12
3,76	3,34	2,92	0,56	0,42
4,94	4,94	4,38	0,28	0,14
4,36	3,27	2,02	0,93	0,47
9,77	2,26	3,01	0,00	0,00
4,14	4,26	5,52	1,04	0,69
5,42	3,10	4,45	0,68	0,29
3,94	2,90	2,67	0,35	0,12
5,82	8,11	9,70	2,85	1,14
2,89	1,60	1,07	0,21	0,43
2,87	2,02	0,85	0,21	0,00
6,62	4,38	2,24	0,32	0,21
3,57	2,67	2,85	0,36	0,36
3,63	4,05	4,47	1,04	0,52
3,76	3,62	2,64	0,70	0,56
3,49	4,54	6,75	3,03	1,98
6,63	6,33	3,31	0,60	0,00
6,07	5,75	9,02	2,49	1,40
12,46	17,05	7,38	0,33	0,66
4,88	3,66	4,27	0,61	0,61
0,84	0,56	0,00	0,00	0,00
4,14	2,98	1,49	0,00	0,00
3,35	3,80	4,25	1,79	1,01
4,62	4,22	3,71	0,80	0,30
5,63	6,70	9,89	3,20	1,22
4,53	7,84	4,53	0,87	0,52
5,18	5,83	5,18	0,91	0,39
5,24	5,01	4,78	0,46	0,23
5,41	7,76	9,53	2,94	1,06
7,62	7,62	3,14	1,35	0,90
6,67	7,62	5,71	1,27	0,32
3,39	5,42	10,34	1,86	1,53
0,00	0,00	0,00	0,00	0,00
1,01	0,38	0,25	0,13	0,25
0,00	0,00	0,00	0,00	0,00
4,17	4,44	3,23	0,54	0,81
6,22	4,80	2,66	0,71	0,89
5,47	4,82	2,68	0,54	0,21
3,77	7,31	14,40	2,77	3,10
3,29	4,18	7,72	1,90	1,52
6,61	9,88	11,83	1,67	0,70
6,20	8,53	7,75	3,88	0,78

6,09	4,91	6,60	1,18	0,85
4,40	5,07	4,02	0,38	0,19
3,18	1,27	4,46	0,00	0,00
5,97	3,63	3,98	0,47	0,23
5,37	6,97	9,00	2,32	0,73
3,83	7,13	7,27	1,59	1,59
6,00	6,42	5,80	1,66	1,66
6,95	6,82	9,18	1,36	0,99
3,19	4,42	1,97	0,49	0,25
3,58	2,01	1,72	0,57	0,00
3,17	4,03	2,20	0,00	0,00
4,51	3,81	2,82	0,42	0,14
4,07	6,73	10,80	2,24	2,66
2,02	5,47	17,61	2,83	5,26
6,02	3,40	8,12	2,62	1,31
3,26	7,07	8,15	4,35	3,26
4,60	5,76	7,77	1,29	1,15
2,14	4,70	6,41	1,71	1,28
4,55	6,25	5,68	0,57	0,57
3,39	3,18	6,57	1,91	1,91
3,40	8,87	11,51	4,15	2,64
2,03	5,19	15,35	2,48	1,35
3,70	5,93	8,89	4,44	1,48
4,49	7,30	8,43	3,93	1,69
3,46	3,67	9,98	4,07	3,26
3,56	3,93	8,80	3,56	3,56
1,00	0,29	0,43	0,00	0,00
7,79	6,95	4,21	0,42	0,00
1,27	3,80	1,27	0,00	0,00
5,02	3,65	6,39	0,91	0,46
2,15	0,54	0,54	0,00	0,00
6,40	7,05	9,92	1,96	0,91
4,76	4,40	4,40	1,28	0,00
0,00	0,00	0,00	0,00	0,00
7,84	10,03	8,78	2,82	0,94
7,71	7,44	5,51	1,10	1,38
0,70	9,86	10,56	6,34	2,82
4,03	4,76	9,16	1,10	0,37
2,26	4,14	10,28	6,02	4,76
4,59	2,75	3,67	0,00	0,92
8,85	5,90	5,90	0,98	0,66
4,13	8,26	4,13	0,00	0,00
5,56	4,63	7,41	2,78	0,93
1,83	7,09	13,96	1,83	1,60
5,71	4,29	7,86	1,43	2,14
2,14	0,19	0,78	0,00	0,19
3,16	5,82	10,82	3,00	2,83
1,97	5,91	9,06	5,12	3,54
1,40	5,26	5,26	3,16	3,51
1,98	4,46	9,41	3,96	2,48

1,49	4,46	10,58	3,31	3,64
2,42	4,46	5,95	1,49	0,56
3,89	4,09	3,27	0,61	0,41
2,67	5,33	4,00	0,00	0,00
2,11	0,70	1,41	0,70	0,00
7,75	4,23	4,93	0,70	0,00
4,91	6,75	6,13	0,00	1,23
4,11	3,46	2,81	0,22	0,43
7,08	7,52	6,64	0,44	0,00
0,00	11,11	6,67	2,22	0,00
7,69	0,00	0,00	2,56	0,00
0,00	0,00	0,00	0,00	0,00
4,62	4,62	3,46	0,92	0,23
2,38	4,57	6,76	1,46	1,28
0,00	9,38	9,38	0,00	3,13
5,98	7,09	5,51	0,94	0,63
2,38	5,56	7,54	6,35	3,57
2,38	0,65	0,22	0,00	0,00
3,94	11,47	9,32	3,94	1,08
2,37	5,59	10,32	3,66	2,37
3,60	2,96	1,16	0,13	0,00
4,04	1,73	0,77	0,00	0,19
1,94	3,70	15,14	2,11	3,87
0,00	0,00	0,00	0,00	0,00
5,00	5,00	5,00	5,00	0,00
4,11	7,59	6,65	0,32	0,00
2,16	1,03	0,00	0,00	0,00
4,12	2,47	0,41	0,00	0,00
4,08	2,04	1,36	0,00	0,00
8,22	8,22	6,85	1,37	0,00
6,05	7,46	7,46	1,01	0,60
6,56	4,69	5,00	1,41	0,63
6,61	6,74	8,55	1,04	0,65
3,84	4,95	6,19	0,99	1,36
3,57	6,54	9,66	1,93	1,49
3,43	7,39	6,33	2,24	1,72
4,91	4,72	10,81	3,14	2,36
4,41	7,85	9,78	1,24	1,10
4,40	7,87	9,38	1,74	1,39
0,00	0,00	0,00	0,00	0,00
5,45	7,26	8,97	1,71	0,96
4,19	5,64	9,39	3,03	1,88
3,30	5,26	7,96	2,70	4,05
3,52	5,68	3,91	1,37	0,00
4,80	4,95	4,35	1,20	0,75
5,13	5,00	4,88	0,63	0,50
5,29	5,29	7,85	1,44	0,32
3,13	1,79	0,45	0,00	0,00
3,17	2,12	0,13	0,00	0,00
4,27	3,66	3,40	0,52	0,35

0,00	0,00	0,00	0,00	0,00
8,33	6,61	7,18	0,86	0,57
5,65	5,86	6,28	1,05	0,21
5,94	7,37	4,68	1,08	0,36
6,09	4,06	2,39	0,84	0,24
3,52	1,71	2,00	0,10	0,10
3,00	0,82	0,68	0,27	0,14
4,30	4,94	3,97	0,54	0,32
6,61	4,92	5,59	1,02	0,17
5,74	2,87	2,62	0,37	0,12
5,53	2,18	2,18	0,17	0,34
4,24	1,95	0,57	0,11	0,00
3,94	2,72	1,41	0,19	0,00
5,19	2,37	0,79	0,45	0,11
2,26	2,41	1,36	0,00	0,15
3,53	1,43	2,00	0,29	0,10
4,27	4,09	1,60	0,53	0,18
6,33	3,06	2,86	0,41	0,20
6,58	4,43	2,68	0,67	0,40
3,14	3,92	3,79	0,52	0,13
3,31	3,64	5,29	1,32	0,33
5,86	9,77	10,83	1,95	2,31
3,16	7,12	11,23	2,69	2,22
3,90	3,42	3,19	0,47	0,12
3,86	6,29	8,87	1,86	1,86
4,64	5,51	6,38	2,61	2,32
4,24	6,11	9,10	1,87	1,87
4,52	7,47	11,39	2,55	1,77
4,46	6,35	8,92	2,23	1,11
5,33	4,53	7,59	1,73	2,00
5,35	4,54	5,67	1,46	1,46
3,85	6,07	6,68	1,21	1,01
3,40	6,21	7,62	2,23	1,88
3,41	6,21	7,31	1,71	2,56
2,11	2,53	7,58	3,37	5,68
6,44	6,26	5,55	0,54	1,97
5,83	4,25	6,44	0,61	0,49
5,65	4,84	4,64	1,01	0,40
4,68	5,15	5,04	1,41	0,23
3,28	5,94	3,89	1,23	0,82
7,22	6,95	8,82	1,07	0,80
5,48	3,78	9,26	1,51	0,38
6,72	4,94	4,74	0,59	0,20
5,43	5,43	5,77	1,96	1,27
6,52	5,98	10,33	0,00	0,54
4,72	4,72	6,89	2,76	2,56
3,12	6,54	9,03	3,12	2,80
2,73	4,81	9,22	4,42	3,38
2,34	3,94	9,36	5,11	3,19
3,36	6,97	9,46	3,11	2,62

4,25	5,72	7,03	1,47	2,61
4,36	6,64	11,95	3,04	3,42
7,00	9,52	9,87	1,44	1,97
4,65	5,25	5,05	0,81	0,40
5,30	4,44	6,84	1,03	0,85
4,33	4,83	5,00	0,50	0,17
2,50	0,94	0,00	0,00	0,00
4,47	3,66	2,85	0,41	0,81
5,11	3,96	4,60	0,51	0,26
3,86	5,01	4,59	1,04	0,42
5,30	4,97	4,86	0,33	0,33
4,43	4,82	2,34	0,26	0,00
5,91	3,80	3,23	0,84	0,28
5,68	4,28	4,56	0,65	0,19
3,67	4,25	8,61	2,64	1,26
5,17	4,13	3,31	0,72	0,10
5,25	3,82	3,06	0,10	0,29
1,75	1,75	0,64	0,00	0,00
5,19	4,23	2,40	0,29	0,00
6,88	4,36	2,29	0,23	0,23
4,74	3,25	2,25	0,50	0,12
2,29	0,87	0,44	0,00	0,00
1,91	0,91	0,10	0,00	0,10
4,02	2,93	1,95	0,24	0,24
5,21	3,47	1,99	0,62	0,25
4,20	2,99	2,91	0,81	0,32
6,24	2,87	2,12	0,00	0,12
4,69	3,13	2,90	0,00	0,00
6,17	4,47	2,13	0,21	0,85
3,06	4,58	1,19	0,51	0,00
4,63	3,86	0,62	0,15	0,15
5,97	3,33	1,72	0,34	0,34
6,93	5,79	2,85	0,66	0,76
6,23	3,56	2,80	0,89	0,25
3,97	2,51	0,63	0,00	0,21
1,50	1,28	0,43	0,21	0,00
4,05	5,74	6,66	0,78	0,26
5,26	6,22	7,06	1,56	0,24
4,75	7,01	6,06	0,83	0,71
5,53	9,12	5,98	1,05	0,60
4,76	7,89	6,70	0,97	0,76
5,17	8,71	11,08	1,77	1,48
5,18	10,23	7,03	1,23	0,99
7,71	9,29	9,57	1,43	1,00
5,01	5,29	6,26	2,36	0,83
4,27	6,07	4,94	1,35	0,45
2,72	5,79	5,90	1,30	1,77
4,29	5,40	6,03	2,54	1,59
3,77	5,08	4,71	1,32	1,69
3,86	6,47	6,75	1,93	2,48

5,96	5,05	9,57	2,17	2,35
3,43	6,69	5,38	1,96	1,14
5,89	5,18	8,04	3,04	1,61
3,78	8,19	10,71	4,16	2,27
8,82	2,94	2,94	0,00	0,00
25,00	8,33	8,33	0,00	0,00
4,49	0,00	2,25	0,00	0,00
4,59	5,96	3,21	0,00	0,46
4,69	4,69	4,69	1,56	0,00
0,00	0,00	0,00	0,00	0,00
0,00	0,00	0,00	0,00	0,00
4,83	2,07	0,00	0,00	0,00
0,00	0,00	0,00	0,00	0,00
2,93	1,46	1,95	0,49	0,00
1,83	0,00	0,33	0,00	0,00
4,88	4,88	4,88	0,00	0,00
2,90	2,90	0,00	0,00	0,00
0,00	0,00	0,00	0,00	0,00
1,85	1,48	0,74	0,00	0,00
6,76	8,88	5,60	0,39	0,39
1,24	1,74	0,25	0,00	0,00
3,85	7,69	4,33	0,96	1,44
3,94	7,69	10,65	3,16	2,17
3,41	0,98	1,46	0,98	0,00
2,69	1,54	1,54	0,00	0,00
3,14	0,00	0,00	0,00	0,00
2,40	8,65	9,62	1,92	1,92
2,44	5,85	8,54	3,17	1,71
4,54	4,54	1,72	0,00	0,12
7,07	3,84	2,63	0,61	0,00
1,01	7,07	7,07	1,01	1,01
0,00	1,33	6,67	8,00	6,67
4,26	1,31	0,00	0,00	0,00
2,82	4,03	3,23	0,81	0,40
2,32	2,42	0,63	0,21	0,11
4,96	2,54	2,29	0,38	0,13
5,38	3,72	2,21	0,28	0,00
1,01	0,51	0,00	0,00	0,00
3,72	2,23	2,42	0,56	0,00
4,21	1,87	1,40	0,00	0,00
3,49	1,31	0,44	0,00	0,00
1,90	1,90	1,90	0,00	0,00
0,00	0,00	0,00	0,00	0,00
1,47	0,29	0,00	0,00	0,00
2,78	2,78	1,71	0,00	0,00
5,25	1,58	0,88	0,00	0,00
5,41	6,65	7,69	1,33	1,04
5,47	4,73	6,35	1,48	0,59
5,32	5,11	6,57	1,56	0,83
2,69	6,72	3,76	0,27	0,00

4,96	4,75	3,27	0,74	0,42
0,00	0,00	0,00	0,00	0,00
3,25	1,75	1,50	0,00	0,00
5,26	3,87	1,08	0,00	0,15
4,00	4,00	2,63	0,42	0,21
6,13	4,09	2,82	0,29	0,10
3,50	2,34	2,69	0,47	0,12
4,73	3,29	2,57	0,62	0,31
4,60	2,25	1,28	0,21	0,11
4,92	3,03	2,27	0,38	0,00
4,58	4,14	1,96	0,00	0,22
4,31	4,08	2,68	0,35	0,23
5,82	6,61	3,17	0,53	0,00
3,23	3,72	2,48	0,00	0,00
4,99	4,99	4,35	1,06	0,85
5,35	6,44	5,74	0,79	0,40
6,50	4,74	2,17	0,54	0,14
4,00	3,33	4,00	0,67	0,00
4,63	6,69	7,72	1,89	2,06
7,26	5,60	5,90	2,27	0,61
5,43	6,34	7,07	1,09	0,54
6,78	6,00	4,95	0,65	1,56
5,16	7,11	5,86	0,84	0,42
6,80	7,48	5,44	1,70	1,02
3,69	6,10	8,83	3,05	1,61
2,17	7,46	8,68	2,58	2,85
3,47	2,98	8,44	1,49	1,49
22,22	0,00	0,00	0,00	0,00
2,56	3,07	2,04	0,68	0,00
5,26	7,94	8,88	0,82	1,05
5,05	5,65	6,39	1,49	0,45
7,06	7,40	7,57	0,34	0,52
5,00	5,00	2,50	0,83	0,00
5,20	7,24	2,94	1,58	1,13
3,70	4,44	4,44	0,86	0,12
3,55	6,15	5,05	1,10	0,24
0,99	1,98	0,00	0,00	0,00
6,59	3,74	2,84	0,30	0,15
6,51	5,18	2,17	0,00	0,17
6,43	7,43	4,22	1,00	0,20
2,83	4,86	4,86	0,81	0,61
6,25	5,16	3,13	0,41	0,00
5,02	8,73	9,03	1,47	0,93
5,00	3,37	1,40	0,58	0,23
5,11	3,00	2,40	0,30	0,00
5,03	9,45	8,32	1,23	0,62
8,75	7,73	4,81	0,44	0,29
5,18	3,01	2,67	0,17	0,17
7,87	7,11	4,31	0,13	0,25
5,70	11,00	10,02	1,96	0,98

7,62	9,94	6,63	0,88	0,44
2,87	3,59	1,95	0,51	0,10
2,99	3,71	1,53	0,16	0,00
5,33	4,50	3,31	0,24	0,00
5,13	5,22	4,46	1,04	0,19
5,53	2,52	1,91	0,80	0,50
5,75	5,36	5,75	0,78	0,78
5,13	6,68	2,98	0,72	0,00
5,73	8,00	7,24	1,51	0,32
3,64	2,95	1,73	0,35	0,09
6,12	7,76	4,26	0,33	0,11
6,93	5,60	3,16	0,00	0,24
0,96	0,48	0,00	0,00	0,00
2,44	0,27	0,00	0,00	0,00
2,26	0,82	0,21	0,00	0,00
2,12	0,81	0,10	0,00	0,00
2,37	0,43	0,43	0,00	0,00
4,89	4,76	2,85	0,41	0,14
5,08	8,15	4,77	0,62	0,15
5,26	2,75	0,92	0,92	0,23
3,62	2,33	1,42	0,13	0,26
4,19	2,28	1,40	0,00	0,13
3,26	2,87	1,96	0,00	0,13
4,08	1,66	0,30	0,00	0,00
1,37	0,00	0,46	0,00	0,00
2,14	0,00	0,00	0,00	0,00
3,44	2,36	1,27	0,18	0,00
0,87	1,53	0,22	0,00	0,00
3,15	2,21	1,05	0,00	0,00
3,63	2,66	1,24	0,18	0,00
4,72	2,66	1,28	0,20	0,10
4,07	1,73	0,90	0,21	0,00
4,77	2,26	1,79	0,72	0,00
4,92	3,71	2,42	0,16	0,08
4,62	2,82	1,80	0,23	0,00
6,13	3,56	0,79	0,59	0,00
4,10	2,12	1,27	0,00	0,00
4,95	2,86	1,30	0,13	0,00
5,60	4,20	2,68	0,47	0,35
4,79	3,70	1,96	0,36	0,15
4,11	3,64	2,69	0,16	0,00
7,34	6,33	4,56	0,76	0,25
1,62	1,95	0,76	0,11	0,11
4,58	3,46	2,46	0,45	0,11
4,24	3,81	2,97	0,85	0,42
4,24	3,75	2,97	0,07	0,14
4,82	3,76	2,45	0,16	0,33
4,25	2,61	1,58	0,14	0,27
5,69	4,12	3,14	0,39	0,39
3,30	3,56	2,51	0,13	0,13

4,06	4,40	1,52	0,17	0,17
6,04	3,78	1,94	0,32	0,11
2,79	2,41	1,14	0,51	0,38
6,05	4,72	1,47	0,15	0,00
6,47	3,10	1,55	0,00	0,13
4,39	3,87	1,94	0,52	0,26
5,70	5,47	3,42	0,57	0,23
3,51	2,40	2,12	0,09	0,09
4,85	4,53	2,85	0,32	0,42
4,73	3,72	1,58	0,68	0,11
2,43	3,20	2,32	0,55	0,11
4,84	4,24	2,17	0,30	0,20
5,81	5,65	5,16	0,65	0,32
7,58	7,82	7,70	1,18	0,47
5,52	6,30	7,40	1,22	0,99
4,15	5,85	6,70	2,17	1,32
5,72	7,41	6,29	2,47	1,01
5,61	7,11	4,99	1,25	0,37
5,68	6,06	6,63	1,70	0,38
5,50	6,33	7,83	1,17	0,83
3,23	6,04	8,54	2,40	1,35
3,91	7,16	9,24	1,82	0,26
5,28	6,49	6,18	1,06	1,06
3,83	6,88	6,88	1,77	1,18
6,14	6,14	5,00	0,45	0,45
4,40	3,93	3,14	0,63	0,63
3,64	5,67	4,86	1,01	1,62
3,70	3,37	1,68	0,00	0,34
5,70	3,61	0,95	0,57	0,00
4,51	1,71	0,16	0,16	0,00
1,32	0,00	2,63	0,00	0,00
0,00	0,00	0,00	0,00	0,00
1,74	3,48	3,48	0,87	0,00
1,86	0,47	0,47	0,00	0,00
4,20	0,00	0,76	0,00	0,00
4,17	0,00	0,00	0,00	0,00
2,11	0,70	1,41	0,00	0,00
3,39	3,08	1,54	0,21	0,00
4,55	2,66	1,66	0,33	0,00
2,41	1,64	1,20	0,11	0,11
4,62	2,55	1,70	0,12	0,12
4,26	1,73	1,86	0,13	0,27
3,49	2,12	1,25	0,12	0,00
4,15	3,85	1,33	0,00	0,00
1,91	1,67	1,19	0,24	0,00
2,77	0,74	0,74	0,00	0,00
2,88	1,08	1,80	0,00	0,00
1,97	0,33	0,66	0,00	0,00
2,53	1,36	0,78	0,00	0,19
1,12	2,46	0,22	0,00	0,00

4,62	3,41	1,09	0,12	0,12
6,57	2,72	2,40	0,00	0,16
0,00	0,00	0,00	0,00	0,00
3,72	4,82	6,68	2,28	2,37
4,18	2,42	2,20	0,44	0,00
6,45	4,72	3,54	0,73	0,36
4,08	3,93	1,81	0,00	0,00
4,40	3,74	1,98	0,00	0,00
3,81	2,10	1,33	0,19	0,00
3,15	3,04	1,52	0,12	0,00
3,90	3,42	3,90	0,24	0,94
1,49	0,41	0,00	0,00	0,00
0,76	0,15	0,00	0,00	0,00
2,04	0,44	0,15	0,00	0,00
2,88	1,00	0,55	0,00	0,00
3,36	1,51	1,16	0,12	0,00
4,55	6,27	6,02	0,86	0,49
8,86	10,33	6,09	0,55	0,37
8,84	9,06	6,00	1,53	0,33
4,27	4,03	2,96	0,59	0,00
6,59	7,23	4,18	0,32	0,48
4,49	6,80	5,17	0,82	0,68
4,68	5,32	6,33	1,27	1,01
2,27	0,30	0,30	0,00	0,00
2,00	0,57	0,29	0,00	0,00
2,31	0,41	0,68	0,00	0,00
1,35	0,84	0,00	0,00	0,00
3,58	3,21	2,10	0,74	0,12
4,46	5,95	2,97	0,37	0,93
4,11	4,57	3,08	1,14	0,23
5,07	2,32	2,03	0,43	0,29
4,83	4,23	3,17	0,60	0,45
5,14	2,71	2,14	0,14	0,00
4,42	4,55	2,53	0,76	0,76
6,01	6,38	3,10	0,00	0,36
4,51	6,01	3,67	0,50	0,17
4,78	5,46	4,10	0,34	0,34
4,86	3,37	2,83	0,67	0,27
5,51	4,91	3,27	0,15	0,15
2,45	2,94	1,96	0,00	0,00
4,49	5,77	1,92	0,64	0,00
3,73	3,36	2,11	0,12	0,00
2,81	2,81	1,34	0,49	0,24
3,46	2,74	2,38	0,36	0,00
3,67	3,03	1,44	0,16	0,16
0,24	0,24	0,00	0,00	0,00
0,00	6,12	4,08	0,00	0,00
1,65	1,38	0,37	0,00	0,00
2,47	0,56	0,22	0,00	0,00
1,68	0,84	0,28	0,00	0,00

7,97	6,26	2,99	0,57	0,28
8,59	8,28	5,16	0,47	0,47
4,96	7,10	5,76	0,94	0,27
6,54	6,13	2,04	0,82	0,61
3,11	0,83	0,62	0,00	0,00
0,53	1,60	1,87	0,00	0,27
1,40	1,92	0,38	0,00	0,13
2,56	1,37	0,85	0,00	0,17
0,98	0,49	0,00	0,12	0,00
5,85	3,83	4,23	0,40	0,20
1,60	1,14	0,91	0,00	0,23
6,44	3,78	3,78	0,89	0,44
5,14	3,29	4,61	1,32	0,13
4,95	4,95	3,96	0,77	0,77
5,29	5,98	4,45	1,11	1,11
4,65	3,88	6,46	1,81	0,52
5,50	5,75	4,00	0,50	1,25
6,89	9,84	6,30	1,38	0,59
6,06	3,46	3,29	0,69	0,17
7,00	6,84	3,73	0,31	0,47
6,03	4,31	3,02	0,86	0,43
5,67	5,10	5,48	0,57	0,00
6,10	10,59	9,16	1,80	0,54
6,29	8,54	8,09	1,57	0,90
5,47	7,08	4,14	1,07	0,27
6,68	5,57	8,69	0,45	1,56
6,99	6,83	6,99	1,30	1,14
7,56	6,64	7,20	1,85	1,11
7,83	4,04	2,78	0,76	0,00
3,98	3,98	6,19	0,74	0,59
5,19	5,65	3,66	1,37	0,92
5,75	6,34	5,01	0,44	0,74
5,18	7,91	8,32	1,77	1,09
7,34	7,34	6,04	0,58	1,01
5,30	4,65	3,37	0,80	0,32
2,23	0,00	0,07	0,00	0,00
5,39	7,41	9,09	1,35	1,68
4,93	7,03	8,22	2,39	1,35
5,65	8,60	11,56	1,88	1,75
5,62	6,01	7,95	1,94	2,13
7,41	7,41	11,11	0,00	0,00
3,30	1,10	0,00	1,10	1,10
7,50	2,50	12,50	2,50	2,50
2,55	6,63	12,24	3,06	2,30
4,08	4,61	9,22	1,60	2,30
4,52	5,46	5,15	0,62	0,94
4,30	7,10	7,29	2,43	1,68
6,00	10,32	6,75	1,69	0,38
6,70	7,20	10,05	1,68	0,17
5,77	8,46	6,54	0,77	2,31

9,77	7,52	3,01	3,76	0,75
2,76	6,55	8,62	2,76	3,45
0,00	0,00	0,00	0,00	0,00
4,51	6,02	6,02	3,38	0,38
2,25	4,49	1,12	1,12	0,00
4,88	4,53	5,23	0,35	0,35
4,29	9,90	7,92	0,66	0,33
2,08	0,69	2,78	0,00	0,00
6,25	0,00	0,00	0,00	0,00
2,08	4,17	2,08	0,00	0,00
3,45	1,38	0,00	0,69	0,00
2,95	2,73	1,36	0,45	0,00
6,76	7,65	11,74	1,07	0,53
5,26	2,63	18,42	2,63	2,63
4,47	5,31	8,10	1,12	1,12
3,38	3,59	10,55	4,01	2,95
4,93	3,52	0,70	0,00	0,70
4,84	4,23	5,44	0,40	0,20
1,85	4,06	11,07	3,69	0,74
2,94	3,68	1,47	0,74	0,00
2,78	4,17	5,56	0,00	1,39
2,08	5,21	9,38	1,04	0,00
5,06	5,70	3,16	0,00	0,00
3,28	3,28	1,09	0,55	0,00
4,64	5,00	6,96	2,86	1,25
1,76	4,71	7,65	4,12	1,76
3,96	4,68	3,60	0,36	0,36
6,34	3,52	1,41	0,70	0,70
2,86	0,63	0,32	0,00	0,00
2,56	0,00	0,00	0,00	0,00
5,76	3,01	4,51	1,75	0,50
5,19	3,89	2,74	0,29	0,43
3,80	3,16	2,53	0,63	0,00
1,32	6,58	3,95	0,00	0,00
6,25	5,21	1,56	0,00	0,00
2,13	2,13	0,00	0,00	0,00
3,23	1,61	3,23	0,00	0,00
5,38	4,30	5,38	0,00	1,08
4,67	3,66	1,02	0,61	0,00
4,43	2,96	1,97	0,49	0,49
2,03	2,03	0,68	0,00	0,00
3,90	5,71	11,41	2,40	1,50
4,34	5,58	6,36	1,71	1,24
5,34	5,90	10,11	3,09	0,84
4,15	2,28	1,04	0,00	0,00
5,16	2,58	1,41	0,00	0,00
7,58	0,00	0,00	0,00	0,00
4,92	4,92	3,28	0,00	0,00
6,00	4,22	1,78	0,67	0,00
4,59	5,57	2,79	0,66	0,49

8,22	4,11	1,37	2,74	1,37
5,49	6,59	2,20	3,30	0,00
4,45	6,79	3,98	0,94	0,94
1,60	6,40	2,40	0,80	0,00
6,38	5,80	6,00	1,35	0,77
2,51	5,22	7,31	2,71	3,34
4,25	8,02	12,26	3,30	1,89
4,48	1,49	2,99	0,00	2,99
6,64	3,32	1,24	0,00	0,00
8,33	8,33	8,33	0,00	0,00
3,80	2,08	1,35	0,25	0,12
3,27	3,82	5,45	1,09	0,73
5,11	6,38	2,98	0,00	0,00
5,04	6,72	8,19	2,31	1,05
4,48	5,97	2,56	0,64	0,21
3,45	6,03	1,72	2,16	0,86
4,24	4,64	1,19	0,26	0,13
3,57	3,57	0,40	0,00	0,00
2,10	1,05	0,63	0,00	0,00
8,89	0,00	2,22	0,00	0,00
9,39	8,78	2,47	0,00	0,00
9,52	5,47	3,88	0,35	0,18
0,00	0,00	0,00	0,00	0,00
4,81	4,14	4,14	1,47	0,67
6,83	8,63	7,31	1,08	1,56
7,29	8,64	7,12	1,02	1,02
5,36	1,79	1,79	0,00	0,00
2,98	3,55	3,26	0,57	0,14
3,64	5,67	8,91	1,62	0,40
0,00	0,00	0,00	0,00	0,00
2,01	0,34	0,67	0,34	0,34
1,99	2,65	1,99	0,00	0,00
0,00	0,00	0,00	0,00	0,00
2,09	0,52	0,00	0,52	0,00
8,54	8,84	2,59	0,61	0,15
3,51	2,68	0,92	0,18	0,09
7,41	0,00	0,00	0,00	0,00
3,50	1,75	0,70	0,00	0,35
5,07	1,69	1,69	0,34	0,34
4,08	5,44	2,72	0,00	0,00
5,00	3,57	0,71	0,71	0,00
3,07	1,32	0,88	0,00	0,00
1,30	1,30	0,00	0,00	0,00
3,70	0,93	0,93	0,00	0,00
2,88	5,77	1,92	0,00	0,00
3,24	6,19	1,47	0,88	0,29
5,41	3,86	1,54	0,00	0,00
3,53	3,14	1,18	0,00	0,00
4,67	2,80	2,80	0,00	0,00
5,10	4,46	3,18	0,64	0,00

6,67	0,00	0,00	0,00	0,00
4,57	2,29	2,29	0,00	0,57
7,10	12,78	12,50	1,14	0,28
2,70	6,31	10,81	1,80	0,90
2,80	2,80	2,80	0,00	0,00
4,86	3,13	3,13	0,35	0,35
3,08	3,69	3,38	0,31	0,31
3,59	4,97	3,31	0,83	0,83
5,14	2,06	1,03	0,00	0,00
3,00	2,25	1,80	0,15	0,45
3,13	1,04	0,00	0,00	0,00
0,00	0,00	4,65	0,00	0,00
2,98	2,38	1,79	1,19	0,00
0,00	1,39	2,78	0,00	0,00
2,45	0,00	1,23	0,61	0,00
3,52	0,44	0,88	0,44	0,00
5,17	0,00	3,45	0,00	0,00
2,84	0,95	0,47	0,00	0,47
1,65	1,10	0,55	0,00	0,00
2,33	2,33	4,65	0,00	0,00
3,37	2,25	1,12	0,84	0,00
2,46	1,97	0,49	0,00	0,00
0,00	0,00	0,00	0,00	0,00
4,14	3,45	8,97	4,83	3,45
5,67	6,16	2,46	0,25	0,25
3,57	1,19	2,38	0,00	0,00
7,82	2,93	2,28	0,00	0,00
3,93	1,97	1,12	0,00	0,00
3,52	4,52	2,51	0,50	0,50
6,67	20,00	13,33	0,00	0,00
3,54	3,98	1,77	0,00	0,44
4,20	5,88	2,52	0,00	0,00
0,00	0,00	0,00	0,00	0,00
6,11	3,82	2,29	0,00	0,00
1,72	3,45	0,00	0,00	0,00
1,87	0,00	0,93	0,00	0,00
6,06	12,12	3,03	0,00	0,00
5,39	11,27	11,76	0,98	0,00
5,13	5,13	8,97	1,92	1,28
3,97	5,14	7,94	1,40	1,64
4,40	4,97	9,37	1,72	2,29
5,76	7,43	12,23	2,40	1,20
4,37	10,20	12,54	4,08	2,33
3,57	1,79	0,00	0,00	0,00
3,85	3,85	2,56	0,00	0,00
3,22	6,76	5,51	1,14	0,21
4,66	6,17	4,80	0,27	0,00
6,19	3,54	1,33	0,00	0,00
5,98	2,78	1,97	0,16	0,16
4,92	5,13	5,03	0,64	0,53

0,00	0,00	0,00	0,00	0,00
3,91	4,42	5,10	1,19	1,36
5,51	4,70	1,46	0,00	0,00
3,46	1,73	0,69	0,35	0,17
5,29	3,56	2,38	0,54	0,00
5,46	4,16	3,51	0,26	0,26
4,66	2,43	1,69	0,00	0,00
2,71	3,75	3,88	0,26	0,26
4,86	3,09	1,99	0,33	0,22
4,21	6,19	3,22	0,25	0,25
4,79	6,31	9,03	2,16	1,68
6,09	5,10	5,81	0,14	0,42
4,72	4,13	4,04	0,93	0,76
5,05	3,96	1,91	0,41	0,00
4,55	3,97	2,33	0,19	0,29
6,94	2,31	1,39	0,00	0,00
3,76	5,05	5,57	1,42	1,55
3,35	2,19	1,31	0,07	0,22
4,75	3,56	2,54	0,34	0,17
6,54	8,49	10,63	0,78	0,68
3,93	5,23	3,16	1,09	0,22
4,19	4,94	3,97	0,86	0,21
4,57	4,31	5,71	1,65	0,38
4,10	3,45	3,54	0,56	0,56
1,49	0,94	0,47	0,23	0,08
3,95	2,09	0,39	0,15	0,00
3,25	1,41	0,85	0,00	0,14
4,65	2,32	1,11	0,00	0,11
2,93	2,08	0,85	0,00	0,00
5,06	3,34	1,29	0,43	0,00
6,00	2,17	1,79	0,13	0,13
1,76	1,01	0,50	0,13	0,13
1,56	2,23	1,34	0,45	0,22
2,75	4,19	3,28	0,66	0,13
4,43	6,13	6,92	0,68	0,45
4,22	4,87	4,55	1,30	0,97
5,87	6,98	7,30	1,11	1,11
2,81	1,79	0,51	0,00	0,00
0,81	0,16	0,00	0,49	0,00
1,99	0,00	0,00	0,00	0,00
2,01	0,31	0,15	0,00	0,00
1,78	0,71	0,00	0,00	0,18
0,85	0,85	0,00	0,00	0,00
1,30	0,24	0,24	0,08	0,00
0,00	0,30	0,60	0,00	0,30
1,71	0,09	0,34	0,51	0,17
3,73	3,08	1,95	0,32	0,16
3,02	4,15	5,09	1,04	0,66
2,86	6,49	14,29	3,38	2,34
2,29	6,10	11,81	5,14	4,95

8,84	11,91	14,08	1,62	0,72
6,43	8,19	17,74	3,31	1,75
4,59	4,77	6,61	1,10	1,10
5,68	7,12	7,64	1,65	0,72
6,89	8,51	6,35	1,76	0,41
7,07	8,72	7,07	1,48	0,33
6,37	9,18	7,87	0,75	0,19
4,16	3,47	3,19	0,55	0,07
4,17	5,42	1,95	0,56	0,00
6,66	5,77	1,63	0,89	0,15
7,71	5,37	4,59	0,59	0,20
4,65	6,24	5,92	1,27	0,53
4,68	5,35	3,34	0,40	0,40
3,16	1,65	1,51	0,27	0,14
1,42	0,71	0,18	0,00	0,00
3,18	0,77	0,77	0,00	0,10
2,41	1,70	0,18	0,09	0,00
4,68	2,03	0,94	0,00	0,00
2,84	1,66	0,10	0,00	0,00
2,90	0,93	0,00	0,00	0,00
3,59	3,17	2,33	0,00	0,00
2,37	2,25	1,42	0,12	0,00
2,09	0,25	0,25	0,00	0,00
4,92	4,08	5,16	1,32	1,32
4,40	5,35	4,72	0,31	3,46
5,61	10,05	8,64	1,40	0,47
4,61	5,13	4,21	1,58	0,92
3,90	3,64	5,97	0,26	0,52
3,69	3,10	3,98	0,88	0,88
6,05	5,92	6,42	1,13	0,50
3,00	2,77	2,55	0,89	0,44
4,20	6,82	11,02	2,05	0,91
4,43	5,70	8,86	2,22	0,95
4,16	9,07	12,67	2,65	2,08
3,61	3,92	3,72	1,24	0,62
2,22	4,13	4,44	1,11	0,16
5,51	2,75	2,41	0,17	0,17
4,85	1,62	0,27	0,27	0,00
1,38	1,99	6,57	2,14	4,13
4,17	7,02	12,74	3,81	2,62
7,08	9,14	6,31	0,77	0,39
1,75	5,06	11,09	3,89	3,50
0,65	2,28	9,62	7,18	4,40
4,11	7,11	8,69	2,05	2,53
2,23	3,63	10,89	5,03	6,42
0,61	3,05	8,84	6,25	7,16
1,00	3,33	10,65	4,66	6,10
1,02	5,46	15,70	5,12	3,58
1,34	3,39	13,46	6,27	5,04
1,71	6,64	13,92	4,07	2,36

1,77	3,97	6,84	5,63	5,74
4,06	3,47	2,11	0,59	0,42
3,83	3,28	2,33	0,41	0,14
5,15	6,80	2,47	0,21	0,10
4,74	3,71	3,33	1,02	0,13
5,02	3,96	3,20	0,30	0,30
5,98	4,59	3,48	0,70	0,14
6,97	5,50	3,67	0,55	0,00
4,88	3,96	4,63	0,93	0,25
5,09	2,91	1,74	0,15	0,15
5,63	4,02	2,41	0,60	0,00
1,55	0,78	0,39	0,10	0,00
4,17	2,34	2,64	0,20	0,20
1,76	0,59	0,25	0,25	0,00
2,38	0,20	0,40	0,00	0,00
3,81	1,61	0,44	0,00	0,15
0,85	0,17	0,00	0,00	0,00
1,96	0,84	1,40	0,00	0,00
2,27	0,00	2,27	0,00	0,00
0,66	0,22	0,00	0,00	0,00
2,84	1,58	0,32	0,00	0,00
2,17	0,00	0,36	0,00	0,00
1,96	0,78	0,78	0,00	0,00
3,50	1,75	0,27	0,00	0,00
2,84	0,47	0,47	0,00	0,00
1,92	0,55	0,41	0,14	0,14
1,97	0,79	0,39	0,00	0,20
1,53	1,34	0,19	0,00	0,38
1,59	0,23	0,23	0,00	0,00
2,00	0,44	0,22	0,00	0,00
1,69	0,68	0,23	0,11	0,00
0,60	2,98	0,60	0,00	0,00
1,26	1,58	0,32	0,00	0,32
1,40	1,12	0,28	0,00	0,00
2,03	0,92	0,74	0,18	0,00
5,30	0,76	0,95	0,00	0,00
3,29	1,75	0,66	0,22	0,00
2,82	1,59	1,35	0,49	0,00
2,12	1,52	0,91	0,00	0,00
5,41	6,31	3,71	0,50	0,10
6,45	4,22	2,23	0,99	0,25
8,60	10,48	4,40	0,42	0,00
8,41	5,31	4,20	1,10	0,60
5,48	2,62	2,14	0,95	0,00
5,70	5,18	6,22	0,52	0,00
6,58	9,13	9,13	0,81	0,67
4,59	7,05	11,75	2,24	0,75
6,71	10,43	8,35	0,89	0,15
3,94	3,50	1,75	0,29	0,15
5,20	6,88	8,15	2,39	1,40

5,32	6,98	8,47	2,49	1,83
1,99	0,31	0,61	0,00	0,00
3,99	7,89	7,74	1,28	1,04
4,81	8,08	8,76	3,61	1,72
6,00	7,03	3,95	0,69	0,69
5,11	7,81	11,22	2,41	1,56
3,81	9,38	8,21	2,64	2,05
3,96	5,28	16,04	1,51	0,75
1,07	4,69	2,56	1,28	0,64
3,22	5,36	12,02	2,79	4,08
1,32	0,24	0,24	0,00	0,00
2,95	1,47	0,40	0,00	0,13
3,83	2,81	3,19	0,77	0,26
0,83	0,21	0,21	0,00	0,00
1,04	0,78	0,26	0,13	0,00
0,23	0,23	0,70	0,00	0,00
4,35	2,02	1,09	0,00	0,00
0,94	0,94	0,56	0,19	0,19
0,40	0,40	0,40	0,00	0,00
0,00	0,00	0,00	0,00	0,00
2,18	4,75	12,53	4,47	4,92
1,79	3,28	8,66	5,07	6,00
3,17	5,68	11,19	2,92	3,92
2,31	6,34	7,73	2,88	3,46
1,83	3,75	13,27	5,29	6,25
1,74	3,18	7,89	5,74	4,41
1,83	3,87	10,99	3,53	7,33
1,40	4,06	13,42	5,62	4,52
1,81	4,82	11,93	5,42	4,82
4,84	2,88	2,54	0,69	0,00
2,13	6,91	19,68	3,72	5,32
3,46	2,54	1,27	0,23	0,00
6,94	4,11	2,57	0,26	0,00
3,62	2,07	1,34	0,21	0,21
3,25	1,27	0,42	0,00	0,07
8,26	5,41	1,50	0,15	0,00
8,33	3,60	2,65	0,38	0,19
0,00	0,00	0,00	0,00	0,00
2,78	1,93	0,53	0,00	0,00
3,08	1,54	0,58	0,19	0,19
3,96	1,13	0,47	0,00	0,00
7,32	4,39	1,46	0,24	0,49
3,85	6,83	5,95	1,58	0,70
4,04	5,00	7,69	0,96	0,58
3,23	0,94	0,54	0,00	0,00
5,68	5,97	2,56	0,28	0,00
2,84	7,28	6,42	1,11	0,74
4,61	7,26	6,56	2,09	1,82
4,78	7,11	9,91	2,10	1,63
3,09	7,10	8,49	1,70	1,54

3,90	5,39	6,19	1,03	1,38
5,37	9,64	10,74	3,17	1,93
3,46	5,43	7,91	3,66	2,08
2,70	7,51	7,96	3,30	1,35
4,03	4,65	8,44	1,10	1,47
6,17	9,52	8,45	0,94	0,27
4,50	7,39	9,64	2,61	1,71
4,12	6,19	5,98	1,24	0,00
5,77	3,85	0,00	0,00	0,00
0,75	0,75	0,00	0,00	0,00
2,26	0,42	0,00	0,00	0,00
1,83	0,46	0,13	0,00	0,00
5,97	6,92	0,31	0,94	0,00
5,65	4,54	2,33	0,55	0,11
8,49	1,89	0,00	0,00	0,00
2,52	0,97	0,19	0,00	0,00
2,46	1,06	0,00	0,00	0,00
2,33	1,05	0,47	0,00	0,00
1,44	3,60	1,44	0,00	0,00
3,77	6,63	8,28	3,31	1,36
4,08	5,61	9,44	3,32	2,81
4,78	6,57	7,31	1,64	1,79
7,79	4,80	2,88	0,12	0,36
6,12	6,91	7,60	1,28	1,68
4,34	7,09	9,83	1,60	1,26
4,32	8,75	10,97	2,92	1,63
3,95	5,68	9,91	2,31	1,64
7,00	8,50	7,00	2,00	0,00
3,67	3,81	8,14	3,81	0,26
3,37	6,13	9,65	1,99	1,38
4,47	7,45	9,47	4,15	0,32
4,83	8,67	9,99	2,52	1,65
5,43	7,16	4,16	0,69	0,46
1,52	1,18	0,17	0,00	0,00
4,42	1,30	0,52	0,00	0,00
1,08	1,08	0,36	0,00	0,00
1,36	0,81	0,00	0,00	0,00
0,95	1,42	0,00	0,00	0,00
3,28	7,54	9,84	3,44	1,48
4,14	6,90	9,31	2,07	0,34
2,64	5,13	10,12	3,47	3,33
3,66	5,96	8,88	2,51	1,99
2,86	4,54	11,54	2,66	4,44
3,46	6,80	10,70	3,12	2,01
3,45	6,60	12,89	2,44	1,42
3,77	9,15	7,40	3,23	1,08
4,64	6,82	9,87	2,90	1,74
2,57	5,03	11,91	3,59	3,80
4,52	9,20	10,26	3,17	2,56
3,54	6,66	11,33	2,41	2,97

2,53	5,32	7,85	3,99	3,06
3,00	4,23	12,28	3,82	3,96
3,07	5,59	11,17	3,35	3,49
2,78	4,50	10,81	4,39	4,07
2,63	2,63	8,12	4,28	5,93
3,43	0,86	0,57	0,00	0,00
1,06	1,06	0,75	0,00	0,00
1,81	1,58	1,58	0,23	0,00
0,82	1,64	0,82	0,00	0,00
0,63	0,63	0,00	0,00	0,00
2,30	0,00	0,00	0,00	0,00
0,84	0,51	0,17	0,00	0,00
4,39	9,12	7,09	2,53	2,87
3,45	8,79	9,26	1,41	1,57
4,25	8,51	9,34	2,49	4,36
4,35	9,42	9,06	1,45	0,36
4,43	5,61	8,57	3,84	4,14
2,40	4,92	10,07	3,78	2,52
3,59	6,73	11,21	3,59	2,24
2,77	6,01	9,71	2,16	2,62
2,20	4,65	10,64	4,03	4,52
8,59	10,63	5,59	0,75	0,00
5,80	7,89	14,62	2,32	1,62
4,20	6,04	10,75	3,48	3,79
4,12	6,59	11,20	2,64	3,29
2,90	5,12	11,34	2,49	5,12
3,24	4,59	10,27	4,05	4,19
3,13	7,55	9,76	3,68	3,13
3,76	7,13	12,84	2,98	2,20
1,79	4,01	11,60	5,17	4,64
3,51	6,15	8,64	3,22	4,39
1,25	3,94	8,96	2,51	2,87
2,00	5,52	9,98	3,99	4,58
1,45	2,77	9,35	3,82	4,87
0,46	2,75	16,06	4,59	8,72
1,68	0,84	0,00	0,00	0,00
1,34	2,23	6,92	4,91	6,25
1,43	1,66	6,73	5,38	3,56
1,06	0,43	0,00	0,00	0,00
0,83	0,21	0,00	0,00	0,00
4,90	7,22	11,34	2,32	1,55
4,80	7,32	10,61	1,77	2,78
4,23	6,97	9,56	3,01	3,14
4,08	6,46	8,16	2,55	3,91
3,19	7,12	9,14	3,51	3,83
2,76	5,41	10,04	3,42	3,64
4,57	7,57	10,57	2,57	1,43
4,35	5,07	9,13	2,17	3,04
4,17	5,34	10,85	2,67	3,51
2,79	8,55	9,67	3,72	3,35

1,68	6,27	12,08	4,43	3,21
1,94	8,39	11,45	2,74	2,74
3,66	5,12	10,83	3,37	2,34
3,40	5,76	10,73	2,62	2,23
3,32	4,34	8,67	2,17	2,81
0,92	2,91	5,97	2,76	4,75
3,36	5,49	10,43	4,37	5,04
3,00	4,55	11,07	3,83	4,76
1,89	4,17	9,85	3,88	4,73
1,73	4,12	9,71	2,39	3,32
2,19	6,30	9,59	4,38	5,21
0,85	2,91	9,48	4,60	5,07
4,56	10,75	9,11	1,40	0,70
6,75	3,88	5,23	0,51	0,34
1,63	4,07	0,81	0,81	0,81
4,67	4,91	4,42	0,00	0,00
3,76	2,15	0,54	0,00	0,00
2,78	1,54	0,31	0,31	0,00
2,99	3,19	1,59	0,00	0,20
4,62	2,31	3,85	1,15	0,38
3,72	0,31	0,00	0,00	0,00
5,56	6,94	8,33	0,00	1,39
2,44	0,98	1,95	0,49	0,00
2,14	0,27	0,00	0,00	0,00
7,53	8,79	7,53	0,42	0,42
1,06	1,06	1,06	0,00	0,00
0,00	0,00	0,00	0,00	0,00
5,22	5,95	6,19	1,09	0,36
1,44	0,29	0,29	0,00	0,00
3,10	1,03	0,79	0,00	0,00
5,86	4,77	2,68	0,40	0,00
4,07	4,07	2,22	1,48	0,00
4,63	3,47	4,25	0,19	0,58
1,85	2,47	2,47	0,00	0,00
2,66	6,87	5,54	1,11	0,00
4,72	0,47	0,00	0,00	0,00
3,19	2,13	0,00	0,00	0,00
4,15	2,29	1,57	0,00	0,00
4,99	4,04	4,28	0,24	0,24
2,59	1,21	0,52	0,00	0,00
0,00	1,25	1,25	0,00	0,00
9,09	0,00	0,00	0,00	0,00
2,35	0,00	1,18	0,00	0,00
4,83	8,24	13,64	4,55	1,42
3,97	4,18	2,92	0,42	0,00
4,85	1,94	2,43	0,49	0,00
6,73	8,75	1,35	0,34	0,34
5,06	1,92	2,09	0,17	0,17
4,76	3,95	2,99	0,54	0,54
8,67	8,83	5,39	0,70	0,08

4,85	8,61	10,91	1,94	1,45
1,09	3,26	6,52	4,35	3,26
5,13	0,00	2,56	0,00	0,00
4,94	6,17	11,52	2,88	2,06
2,37	6,21	10,36	2,66	0,89
1,01	2,36	10,81	4,73	5,07
0,00	6,90	3,45	0,00	10,34
0,38	0,56	0,38	0,00	0,00
3,16	1,05	0,00	0,00	0,00
3,16	6,33	12,03	3,16	5,06
3,69	1,11	0,37	0,00	0,00
6,21	4,91	1,45	0,14	0,14
4,20	2,52	4,20	0,00	0,00
4,21	3,16	0,00	1,05	1,05
2,79	2,09	1,40	0,47	0,23
1,67	2,28	11,70	3,50	4,41
0,19	3,26	5,76	2,50	4,22
3,40	0,68	0,00	0,00	0,00
5,63	7,04	5,16	0,70	0,47
5,43	4,39	4,26	0,78	0,52
4,08	4,08	3,40	1,36	0,00
2,56	2,56	0,00	0,00	0,00
2,12	0,85	0,00	0,00	0,00
4,21	7,37	10,53	2,11	0,00
0,00	0,00	0,00	0,00	0,00
2,54	0,85	0,00	0,85	0,00
4,75	4,75	6,00	2,00	0,25
0,00	3,17	0,00	0,00	0,00
5,56	0,00	0,00	0,00	0,00
3,02	4,02	1,01	0,25	0,25
1,74	0,54	0,22	0,00	0,00
4,92	2,46	0,41	0,41	0,00
6,25	6,25	12,50	0,00	0,00
2,96	7,22	13,46	3,61	5,91
2,72	6,53	19,60	5,63	3,63
0,77	3,87	10,57	5,41	6,44
2,69	7,01	11,80	5,14	2,80
2,53	4,15	9,45	5,07	4,84
1,69	7,40	14,03	8,18	4,94
1,22	2,27	12,06	10,14	1,40
1,30	2,59	11,35	5,67	3,89
2,06	3,97	11,90	5,08	6,35
1,35	5,68	13,51	5,81	3,24
1,20	5,20	8,60	5,20	6,00
1,72	3,10	11,81	5,69	4,83
0,65	5,18	8,58	3,56	3,24
3,11	3,73	6,69	1,87	2,02
2,90	2,42	2,42	0,48	0,00
5,37	2,68	2,35	0,00	0,00
6,02	4,62	3,82	1,00	0,00

0,99	0,14	0,14	0,00	0,00
0,00	0,00	0,00	0,00	0,00
2,86	2,14	0,71	0,71	0,71
0,00	0,00	0,00	0,00	0,00
2,41	2,41	0,54	0,27	0,00
1,19	1,19	0,60	0,00	0,00
0,57	0,57	0,00	0,00	0,00
5,41	2,70	0,00	0,00	0,00
3,87	1,45	0,73	0,00	0,00
1,04	2,08	0,00	1,04	0,00
2,29	0,53	0,18	0,00	0,00
10,45	1,49	4,48	0,00	0,00
1,09	1,09	0,00	0,00	0,00
4,31	2,76	1,90	0,34	0,17
4,49	5,31	6,53	1,22	0,00
3,54	3,54	3,54	2,65	0,88
3,05	2,69	1,59	0,49	0,00
0,00	0,00	0,00	0,00	0,00
1,27	1,27	0,00	0,00	0,00
2,16	4,74	5,17	1,29	0,43
5,83	7,28	7,28	0,97	0,00
5,14	3,43	6,86	1,71	0,57
4,74	8,70	11,46	2,77	0,79
3,80	5,70	8,37	2,66	1,14
7,05	1,28	2,56	1,92	0,00
2,69	6,29	5,99	2,10	0,60
7,91	11,07	5,98	0,35	0,18
4,40	8,19	6,53	1,52	1,37
1,06	1,06	0,00	0,00	0,00
1,21	0,40	0,00	0,40	0,00
4,50	6,00	7,71	2,36	1,28
2,78	0,93	0,00	0,93	0,93
0,00	0,00	0,00	0,00	0,00
0,00	0,00	0,00	0,00	0,00
2,50	0,00	0,00	0,00	0,00
1,24	0,25	0,12	0,00	0,00
0,00	0,00	0,00	0,00	0,00
0,89	0,22	0,22	0,11	0,00
1,54	1,54	1,54	0,00	0,00
0,27	0,00	0,27	0,00	0,00
2,08	5,50	12,86	4,31	5,58
1,75	2,05	5,56	2,63	6,43
0,00	0,00	0,00	0,00	0,00
0,00	0,00	0,00	0,00	0,00
2,05	3,59	6,15	0,51	0,51
2,79	2,21	0,93	0,00	0,00
4,75	2,37	1,63	0,74	0,00
4,64	1,90	0,42	0,21	0,00
1,64	0,00	0,55	0,00	0,00
4,18	0,76	0,76	0,00	0,00

1,23	1,23	0,41	0,00	0,00
3,49	0,00	1,16	1,16	0,00
9,56	7,25	3,74	0,99	0,33
4,21	1,02	0,38	0,26	0,00
5,00	5,00	0,00	0,00	0,00
4,09	2,27	0,00	0,00	0,00
8,11	2,70	5,41	2,70	0,00
1,32	1,32	0,00	0,00	0,00
4,50	3,46	0,69	0,35	0,00
7,81	3,65	2,08	0,52	0,00
0,00	0,00	0,00	0,00	0,00
0,00	0,00	0,00	0,00	0,00
3,92	5,10	9,41	1,96	1,18
0,00	0,00	0,00	0,00	0,00
2,70	7,21	10,81	2,25	3,15
5,61	9,81	9,81	2,34	0,00
0,97	0,97	0,48	0,00	0,00
0,00	0,00	0,00	0,00	0,00
1,01	0,00	0,00	0,00	0,00
0,71	1,07	0,00	0,00	0,00
0,37	0,37	0,00	0,00	0,00
1,57	0,31	0,00	0,00	0,00
0,80	0,00	0,00	0,00	0,00
4,00	4,00	0,00	0,00	0,00
0,00	0,00	0,00	0,00	0,00
9,28	7,17	4,64	0,00	0,00
3,57	10,71	7,14	0,00	10,71
4,66	3,39	5,51	0,42	0,85
5,94	5,18	2,59	1,07	0,46
2,70	1,14	0,00	0,00	0,00
4,22	7,64	10,14	4,22	1,32
2,61	6,81	11,82	2,81	2,81
2,20	3,70	11,21	4,86	3,35
4,07	5,69	5,89	2,64	1,83
6,16	5,41	8,55	1,89	0,88
0,41	1,83	6,71	4,27	4,67
1,03	3,21	8,69	2,28	3,72
0,94	2,59	3,76	3,76	3,29
0,00	0,00	0,00	0,00	0,00
4,39	3,32	3,06	0,93	0,40
0,60	0,80	0,00	0,00	0,00
3,54	0,88	0,44	0,00	0,00
3,20	1,99	1,14	0,07	0,07
1,66	7,14	12,88	3,83	2,55
0,00	0,00	0,00	0,00	0,00
3,25	6,20	9,31	1,92	2,51
7,45	5,96	2,09	0,15	0,15
3,68	2,02	2,14	0,24	0,00
2,82	2,63	2,54	0,36	0,00
4,94	4,05	2,66	0,38	0,63

5,63	2,25	1,13	0,00	0,00
4,73	4,60	2,81	0,38	0,26
8,47	3,39	0,00	0,00	0,00
1,00	2,72	6,95	3,87	4,37
3,04	4,46	7,17	2,28	5,11
0,99	3,35	5,46	3,10	2,23
1,79	5,24	14,48	3,17	1,38
1,13	0,65	0,32	0,16	0,00
4,75	5,42	9,83	2,37	0,85
4,62	8,05	10,88	0,75	0,75
4,02	7,54	9,55	3,52	2,51
2,84	5,55	7,11	1,28	2,56
3,46	4,22	8,89	3,31	2,56
6,05	5,75	7,11	1,06	0,91
7,20	6,00	9,60	2,08	0,48
4,47	8,75	6,23	1,75	1,56
3,64	12,73	7,27	3,64	0,00
3,33	5,00	7,78	2,64	1,53
5,31	6,00	7,84	2,65	2,19
5,13	6,08	7,22	1,33	0,76
3,58	3,76	4,11	1,07	0,36
9,22	8,52	5,04	0,87	0,70
5,41	5,56	5,41	0,64	0,32
6,58	6,34	6,70	1,20	0,12
3,62	2,35	0,90	0,00	0,18
3,08	2,50	1,16	0,00	0,00
3,71	2,37	2,08	0,45	0,15
0,00	0,00	0,00	0,00	0,00
7,40	7,40	8,88	1,18	0,30
7,09	9,63	6,95	0,80	0,53
6,51	8,18	10,78	1,67	0,93
3,86	5,04	9,20	3,71	2,23
3,25	8,82	11,60	2,09	1,86
5,63	2,81	4,00	1,19	0,74
4,33	5,09	3,18	0,51	0,25
6,19	4,01	3,56	0,69	0,57
4,28	4,87	2,06	0,15	0,15
9,11	7,53	5,25	0,53	0,35
5,35	5,01	2,39	0,00	0,23
5,00	3,26	1,85	0,22	0,22
5,27	3,26	2,51	0,38	0,00
3,76	3,49	1,34	0,13	0,00
4,85	2,55	1,33	0,12	0,12
7,03	5,92	4,46	0,56	0,22
5,72	8,14	6,60	0,99	0,33
2,31	4,62	5,78	1,16	0,00
3,88	2,50	1,63	0,25	0,00
8,50	7,32	3,27	0,78	0,13
1,56	1,36	0,39	0,19	0,00
7,28	3,72	2,27	0,32	0,00

5,30	3,79	3,28	0,00	0,25
2,17	0,72	0,29	0,00	0,00
2,48	1,73	0,68	0,30	0,00
4,07	1,74	1,26	0,29	0,00
5,86	2,90	1,36	0,12	0,06
2,45	0,82	0,27	0,00	0,00
0,00	0,00	0,00	0,00	0,00
3,13	3,37	7,59	2,05	2,65
6,00	2,22	3,13	0,26	0,00
1,37	1,17	0,51	0,05	0,05
4,21	5,41	5,56	1,20	1,65
2,81	4,13	6,94	2,81	2,64
1,54	1,23	8,62	2,46	2,46
0,91	3,34	9,27	3,19	5,02
4,58	4,41	4,41	0,68	1,19
3,07	1,89	4,49	1,18	0,95
1,20	1,60	5,38	2,49	4,49
0,00	0,00	4,76	4,76	14,29
3,34	0,53	0,13	0,00	0,00
2,65	5,84	6,55	2,12	2,65
2,86	6,05	8,74	3,36	2,18
4,17	5,51	7,74	1,04	1,04
2,95	4,92	9,34	2,62	2,79
0,69	5,73	9,86	3,21	4,36
4,82	4,34	9,04	3,25	1,57
3,28	5,62	7,73	2,46	1,17
2,35	6,46	9,25	3,67	2,20
3,21	5,34	8,55	2,75	2,60
2,57	4,34	10,57	4,34	1,90
3,10	3,56	1,55	0,00	0,15
4,32	1,69	0,75	0,19	0,00
5,12	2,25	1,50	0,00	0,00
0,00	0,00	0,00	0,00	0,00
3,72	6,70	8,29	1,06	2,02
0,96	3,35	1,91	0,48	0,00
5,90	3,26	2,89	0,75	0,25
3,56	4,13	5,70	2,28	1,14
4,63	2,80	1,83	0,32	0,00
9,47	9,09	4,17	0,00	0,76
1,32	0,26	0,00	0,00	0,00
4,72	1,29	1,72	0,00	0,00
0,29	0,44	0,00	0,00	0,00
0,00	0,92	0,00	0,00	0,00
7,24	2,41	1,72	0,00	0,34
4,10	5,40	2,98	0,19	0,00
6,10	5,16	2,11	0,35	0,35
2,26	1,76	0,92	0,00	0,00
15,38	0,00	7,69	0,00	0,00
2,10	2,45	8,04	2,10	2,10
3,05	6,48	7,81	3,43	3,62

2,18	3,73	12,44	4,04	0,93
6,25	0,00	4,69	0,00	0,00
4,88	5,15	7,12	1,58	1,32
2,16	0,98	0,79	0,00	0,00
0,35	1,04	7,18	4,40	6,13
3,33	0,74	0,00	0,00	0,00
1,94	7,96	14,17	5,24	3,30
3,32	6,46	13,09	4,71	2,97
1,61	4,59	9,86	4,13	2,98
1,68	4,29	9,70	5,60	5,04
1,60	4,66	12,68	3,64	3,64
2,23	5,48	12,78	3,04	3,45
3,06	7,09	11,92	5,15	2,42
3,00	4,12	10,30	5,24	3,75
2,60	5,41	11,26	2,16	3,03
3,26	5,70	11,56	3,26	2,61
3,59	7,38	12,87	2,74	4,22
1,48	5,17	11,82	1,72	2,71
2,07	4,70	12,31	4,29	3,18
1,43	3,18	7,63	2,86	4,29
0,50	4,65	13,95	3,99	4,82
7,94	10,84	9,01	1,07	0,61
3,26	8,46	9,20	2,37	1,63
4,01	5,11	11,74	2,21	3,04
2,53	6,05	9,99	3,80	2,95
8,43	11,92	6,40	0,87	0,29
5,99	8,10	16,90	2,82	1,76
3,93	7,86	12,86	6,43	5,71
4,63	6,26	6,26	1,63	0,73
5,51	7,35	8,09	4,04	1,47
0,00	0,00	0,00	0,00	0,00
4,38	6,11	8,30	2,92	2,01
0,00	0,00	0,00	0,00	0,00
2,16	8,01	8,01	3,03	4,76
6,12	10,20	12,24	5,10	1,02
4,55	2,27	4,80	2,53	1,77
3,47	4,86	10,24	2,95	2,95
2,55	6,52	13,88	3,40	4,25
4,73	7,94	10,30	4,05	3,04
4,35	2,17	5,43	3,26	5,43
4,25	8,50	8,28	1,79	2,24
4,50	9,30	13,49	3,10	0,62
4,08	5,76	4,32	1,20	0,24
3,28	4,01	4,93	0,91	1,09
6,67	5,56	1,48	0,74	1,11
3,76	4,46	3,99	0,23	0,00
2,86	2,86	4,29	4,29	1,43
6,92	9,15	8,03	1,61	1,36
3,88	2,91	5,83	0,97	0,00
2,54	3,99	9,54	4,35	3,99

3,27	6,21	5,56	1,63	0,98
5,19	5,52	7,63	2,76	1,62
4,86	6,00	8,43	1,94	1,46
2,45	10,29	4,90	1,47	1,47
2,72	4,08	10,88	2,72	2,42
1,37	3,84	3,56	1,92	3,29
1,19	0,60	0,90	0,00	0,00
4,44	7,21	9,29	1,80	0,42
4,68	8,67	8,84	0,87	0,17
5,21	7,69	13,40	2,23	0,50
0,00	0,00	0,00	0,00	0,00
6,21	7,30	5,43	1,86	1,55
0,00	4,92	3,28	0,00	0,00
5,63	0,00	0,00	0,00	0,00
2,16	1,54	0,62	0,31	0,00
4,85	0,00	0,00	0,97	0,00
4,81	6,17	5,11	1,50	0,30
4,03	2,56	6,59	1,10	0,00
0,89	0,22	0,11	0,00	0,00
1,17	0,26	0,26	0,00	0,00
1,42	1,14	0,00	0,00	0,00
0,89	0,00	0,00	0,00	0,00
3,82	9,33	15,44	3,06	1,07
4,31	7,48	12,24	2,72	3,40
4,40	6,15	13,41	6,15	3,30
6,43	9,25	11,31	3,34	1,54
2,92	6,03	12,65	2,92	1,36
2,99	9,89	14,02	3,91	0,92
3,19	8,99	18,26	4,93	2,32
2,48	4,76	12,95	4,00	3,62
3,19	7,25	13,19	3,04	1,16
3,82	8,11	16,71	3,10	2,15
1,64	5,32	11,86	4,29	2,45
4,39	5,20	6,83	2,11	1,95
3,93	2,56	1,54	0,17	0,00
4,85	5,70	5,34	1,21	0,36
4,97	2,74	0,68	0,00	0,17
4,49	3,51	1,54	0,14	0,14
0,00	0,00	0,00	0,00	0,00
2,90	0,60	0,00	0,00	0,00
3,37	2,25	2,25	0,00	0,00
5,00	5,00	5,00	0,00	5,00
2,20	1,76	1,76	0,88	0,00
2,25	3,38	4,13	1,50	1,50
0,00	0,00	0,00	0,00	0,00
4,92	3,28	3,28	0,00	0,00
1,33	1,00	0,33	0,00	0,00
0,00	0,00	0,00	0,00	0,00
4,34	5,87	5,87	1,53	0,77
1,78	2,07	5,47	0,15	1,04

0,00	0,00	0,00	0,00	0,00
3,80	4,89	5,43	2,17	0,00
5,23	6,98	2,33	0,58	2,33
5,56	0,00	5,56	2,78	2,78
3,52	4,81	5,52	1,41	0,35
2,81	5,22	5,62	3,61	2,01
1,68	0,39	0,13	0,00	0,00
3,26	5,95	11,90	2,27	1,70
1,91	4,63	10,90	1,63	2,45
5,13	4,62	6,67	3,59	2,56
2,47	4,52	6,44	1,78	1,23
4,93	7,17	9,87	2,24	1,57
4,23	4,83	12,54	3,63	1,81
2,04	11,22	9,18	3,06	0,00
3,84	5,97	12,94	3,70	1,71
4,01	8,86	11,81	2,74	1,48
2,68	7,32	12,50	2,32	0,71
6,00	2,00	0,00	0,00	0,00
2,65	2,12	0,35	0,18	0,00
3,33	0,00	0,00	0,00	0,00
5,82	7,05	9,35	1,59	0,88
7,89	5,26	0,00	0,00	2,63
6,25	6,25	10,56	2,16	1,72
0,00	0,00	0,00	0,00	0,00
0,00	0,00	0,00	0,00	0,00
5,80	4,35	0,00	0,00	0,00
1,62	0,81	1,82	0,20	0,00
3,91	5,13	6,60	0,24	0,00
0,00	0,00	0,00	0,00	0,00
0,00	0,00	0,00	0,00	0,00
4,29	8,86	4,86	0,57	0,00
0,00	0,00	0,00	0,00	0,00
6,42	4,69	2,78	0,17	0,17
4,82	4,45	2,73	0,45	0,27
4,94	4,59	2,17	0,17	0,17
3,68	6,65	5,78	1,40	0,70
4,04	3,14	1,12	0,11	0,17
5,03	2,82	1,60	0,25	0,00
6,15	4,45	1,31	0,13	0,00
5,78	4,87	1,36	0,11	0,00
5,46	5,24	0,67	0,22	0,00
5,41	3,80	1,27	0,00	0,00
5,48	3,06	1,13	0,00	0,00
6,59	4,02	3,05	0,24	0,12
7,67	2,95	0,24	0,00	0,12
3,40	1,13	0,00	0,00	0,00
2,05	0,16	0,00	0,00	0,00
0,47	0,39	0,08	0,00	0,00
1,83	0,88	0,48	0,00	0,00
4,41	0,91	0,28	0,07	0,07

2,07	1,04	0,00	0,00	0,00
3,92	1,31	0,00	0,00	0,00
2,99	2,60	0,39	0,00	0,00
6,82	6,82	2,56	0,21	0,21
7,23	4,87	2,23	0,56	0,42
8,03	2,88	1,52	0,76	0,00
5,47	3,91	1,56	0,00	0,16
5,39	3,92	2,55	0,20	0,20
5,74	4,25	0,96	0,11	0,11
8,02	6,28	2,67	0,47	0,58
5,54	3,94	2,63	0,19	0,09
1,00	0,47	0,12	0,00	0,00
4,36	3,27	1,33	0,12	0,00
2,95	1,48	0,00	0,00	0,00
7,07	5,27	2,22	0,11	0,11
5,68	5,87	2,33	0,19	0,28
2,36	1,57	0,55	0,00	0,00
5,22	2,61	0,00	0,00	0,00
1,18	1,07	0,11	0,00	0,00
1,06	0,66	0,13	0,00	0,00
2,30	3,62	1,64	0,33	0,00
3,08	0,49	0,49	0,16	0,00
2,22	0,98	0,00	0,00	0,00
4,74	5,19	2,93	0,68	0,23
3,86	3,74	2,90	0,12	0,12
3,16	3,91	2,86	0,15	0,15
11,56	0,50	0,00	0,00	0,00
3,19	2,77	1,49	0,43	0,00
5,66	5,28	3,09	1,03	0,26
5,01	4,29	1,97	0,18	0,72
5,53	3,64	2,16	0,40	0,13
0,00	0,00	0,00	0,00	0,00
5,08	3,50	3,84	0,23	0,11
7,01	5,65	1,57	0,21	0,00
7,75	4,50	3,10	0,31	0,00
8,14	5,07	1,53	0,12	0,00
7,59	6,03	2,46	0,22	0,00
4,94	7,35	4,58	0,36	0,36
7,15	6,05	4,54	0,69	0,00
4,99	2,94	1,25	0,18	0,09
5,80	1,93	0,18	0,00	0,00
4,83	3,84	0,99	0,00	0,00
2,36	1,47	0,29	0,00	0,00
3,39	2,39	0,00	0,13	0,00
5,79	2,07	0,00	0,00	0,00
3,69	1,48	0,25	0,00	0,00
4,29	3,36	1,31	0,19	0,00
1,22	0,00	0,17	0,00	0,00
6,00	2,08	1,15	0,00	0,00
4,19	5,24	0,00	0,00	0,00

6,11	4,61	1,20	0,30	0,10
4,75	4,57	3,41	0,36	0,00
2,44	2,44	1,01	0,00	0,00
5,78	3,42	3,29	0,26	0,00
4,89	3,89	1,38	0,25	0,00
6,18	3,48	1,74	0,00	0,19
3,20	2,13	1,64	0,00	0,00
2,00	0,53	0,40	0,00	0,13
4,85	2,68	1,86	0,21	0,00
3,45	0,97	0,65	0,11	0,00
3,44	1,87	0,86	0,00	0,00
3,26	1,30	0,43	0,11	0,00
2,59	1,11	0,12	0,00	0,00
2,74	2,08	0,66	0,00	0,00
0,00	0,00	0,00	0,00	0,00
3,10	1,43	0,12	0,00	0,00
2,84	1,11	0,12	0,00	0,00
3,25	1,26	0,00	0,00	0,10
0,00	0,00	0,00	0,00	0,00
5,49	1,59	0,61	0,12	0,00
4,09	5,17	1,94	0,22	0,00
8,00	8,25	6,77	0,58	0,08
5,33	4,60	3,63	0,97	0,24
5,14	5,63	3,98	0,97	0,39
4,48	8,66	5,37	0,30	0,00
3,35	4,39	7,11	0,63	0,21
5,10	6,85	7,01	1,75	0,64
6,69	6,50	3,35	0,69	0,10
6,95	5,73	3,19	0,47	0,28
6,31	6,07	2,79	0,12	0,61
4,86	3,65	1,74	0,17	0,00
5,97	7,75	6,73	0,76	0,64
6,85	5,19	4,36	0,62	0,21
4,92	6,28	3,96	0,55	0,00
6,37	4,52	2,43	0,12	0,12
5,77	4,92	2,57	0,07	0,07
5,43	3,17	1,00	0,00	0,00
4,81	3,11	1,90	0,20	0,20
6,21	4,05	1,60	0,19	0,09
4,76	3,81	0,48	0,00	0,00
3,39	0,53	0,42	0,00	0,00
2,08	0,26	0,13	0,00	0,00
2,40	0,76	0,44	0,00	0,11
3,98	1,88	1,05	0,00	0,10
0,00	0,00	0,00	0,00	0,00
4,27	2,56	1,71	0,00	0,00
2,04	0,19	0,00	0,00	0,00
4,64	2,41	0,34	0,00	0,00
2,97	0,82	0,16	0,00	0,00
5,11	2,82	0,61	0,08	0,00

1,52	0,76	0,30	0,00	0,00
2,46	0,79	0,53	0,00	0,00
5,14	2,06	1,03	0,11	0,11
4,44	2,42	0,58	0,00	0,10
2,04	0,75	0,00	0,21	0,00
4,83	2,90	1,21	0,00	0,00
2,47	1,58	0,49	0,00	0,00
4,94	0,00	0,00	0,00	0,00
3,92	2,85	1,31	0,00	0,12
5,18	6,01	3,84	0,50	0,50
3,70	4,15	1,63	0,00	0,00
5,40	6,79	3,88	1,11	0,55
4,44	3,21	1,60	0,12	0,00
5,07	4,96	4,54	0,42	0,42
5,03	7,10	5,94	0,66	0,41
6,98	6,04	4,06	0,75	0,47
6,18	6,78	5,05	1,21	0,38
0,00	0,00	0,00	0,00	0,00
4,55	6,21	6,34	2,21	1,52
6,27	6,65	6,15	1,25	0,25
5,81	6,94	5,48	1,13	0,65
5,52	5,89	4,24	1,66	0,74
5,18	5,52	8,86	1,51	2,51
4,74	6,86	8,98	0,75	1,37
4,03	5,33	3,79	0,83	0,24
5,01	5,24	0,68	0,23	0,00
4,14	4,32	2,88	0,36	0,00
4,51	3,88	1,62	0,18	0,18
5,02	4,94	2,92	0,57	0,08
6,93	4,65	2,06	0,43	0,22
3,29	2,50	1,25	0,16	0,00
4,72	3,66	0,59	0,12	0,00
3,62	2,45	1,28	0,35	0,00
2,65	1,09	0,27	0,00	0,00
2,47	1,47	0,27	0,18	0,00
3,78	3,24	0,00	0,00	0,00
5,22	2,86	1,37	0,00	0,00
3,39	0,80	0,18	0,00	0,00
3,95	3,86	1,43	0,09	0,00
5,65	3,18	1,77	0,12	0,35
4,83	2,97	2,88	0,28	0,19
6,40	7,01	2,13	0,00	0,00
6,86	3,39	4,63	0,31	0,15
8,14	7,92	2,53	0,00	0,00
7,25	6,00	4,19	0,11	0,00
1,94	0,72	0,10	0,00	0,00
2,16	0,00	0,00	0,00	0,00
3,78	2,57	1,08	0,14	0,00
3,69	4,30	1,84	0,00	0,00
3,85	1,92	1,65	0,00	0,00

4,06	1,02	0,00	0,00	0,00
3,40	1,32	0,28	0,00	0,00
5,91	3,85	1,65	0,00	0,00
0,00	0,00	0,00	0,00	0,00
5,69	2,92	3,47	0,28	0,00
4,58	5,42	2,71	0,51	0,17
5,79	4,63	1,59	0,58	0,14
7,28	4,00	3,03	0,24	0,00
7,79	5,19	1,62	0,16	0,16
1,19	0,68	0,00	0,00	0,00
5,98	6,44	4,25	0,69	0,23
6,95	7,06	3,15	0,43	0,11
5,71	5,99	4,22	0,00	0,41
3,62	2,80	1,15	0,00	0,00
4,40	4,17	1,27	0,00	0,12
3,13	1,57	0,00	0,20	0,00
2,12	0,45	0,15	0,00	0,00
0,92	0,00	0,23	0,00	0,00
1,52	0,81	0,09	0,00	0,00
0,99	0,58	0,08	0,00	0,00
6,54	1,31	0,65	0,00	0,00
1,30	0,00	0,00	0,00	0,00
4,13	2,07	0,83	0,00	0,00
4,07	1,11	0,56	0,00	0,19
4,48	2,76	0,80	0,11	0,00
2,91	2,42	0,32	0,16	0,00
7,44	4,25	0,71	0,12	0,12
4,92	1,86	1,33	0,00	0,00
2,79	1,74	0,00	0,00	0,35
5,16	3,32	1,66	0,00	0,18
0,93	1,12	0,19	0,00	0,00
6,82	2,94	0,94	0,00	0,00
4,87	3,31	0,78	0,00	0,00
4,89	4,59	2,75	0,00	0,00
4,99	3,22	1,44	0,55	0,33
5,72	2,86	1,43	0,38	0,19
4,84	4,73	1,16	0,21	0,11
6,06	3,33	0,45	0,00	0,00
5,04	3,05	2,25	0,66	0,40
4,91	2,84	1,03	0,09	0,00
3,49	3,26	1,69	0,00	0,11
5,10	4,61	2,91	0,24	0,24
4,23	3,34	3,12	0,22	0,00
5,74	2,87	1,00	0,00	0,12
3,84	1,77	0,79	0,00	0,00
6,56	4,64	2,05	0,27	0,00
7,12	4,70	1,48	0,13	0,00
7,63	3,15	1,91	0,19	0,00
1,12	0,46	0,00	0,00	0,09
0,19	0,09	0,09	0,00	0,00

4,76	3,21	1,00	0,00	0,00
5,00	2,94	1,37	0,00	0,10
3,45	1,92	1,53	0,00	0,00
2,90	3,12	0,89	0,00	0,00
7,02	6,18	0,28	0,00	0,00
4,89	3,53	0,42	0,00	0,00
6,38	2,44	0,94	0,19	0,00
3,46	2,41	1,20	0,75	0,00
4,67	2,80	1,87	0,00	0,23
6,54	3,74	2,57	0,23	0,00
4,34	2,50	1,67	0,17	0,33
5,10	1,46	0,44	0,15	0,00
0,00	0,00	0,00	0,00	0,00
2,80	0,93	0,93	0,00	0,00
4,26	2,87	1,39	0,00	0,00
4,05	1,58	0,68	0,45	0,00
4,84	5,71	1,74	0,12	0,00
5,23	2,38	1,54	0,12	0,00
2,77	1,69	0,77	0,00	0,00
4,51	3,31	1,35	0,15	0,15
3,75	2,37	0,40	0,00	0,00
4,64	3,15	0,66	0,00	0,17
5,37	3,43	1,19	0,30	0,30
4,66	1,83	0,50	0,00	0,17
6,30	1,54	1,26	0,00	0,00
4,20	1,40	0,35	0,00	0,00
4,95	3,13	1,17	0,00	0,00
5,83	4,09	2,11	0,00	0,00
4,40	3,20	0,80	0,00	0,00
2,13	1,31	0,33	0,16	0,00
3,13	1,67	0,45	0,00	0,11
1,90	1,90	0,57	0,10	0,00
5,31	2,92	1,41	0,09	0,00
5,10	2,74	1,42	0,09	0,09
3,54	2,53	0,40	0,00	0,00
3,35	1,31	0,15	0,15	0,00
5,59	6,12	4,52	0,53	0,00
5,78	3,91	1,56	0,16	0,16
4,50	3,64	1,07	0,00	0,21
4,12	4,71	2,55	0,69	0,10
6,51	6,51	4,60	3,45	1,53
0,00	0,00	0,00	0,00	0,00
6,56	5,37	2,24	0,15	0,30
4,15	3,49	1,09	0,22	0,00
5,12	3,31	3,31	0,00	0,30
7,99	2,38	0,14	0,00	0,00
0,79	0,00	0,00	0,00	0,00
0,00	0,27	0,00	0,00	0,00
1,03	0,51	0,51	0,00	0,00
0,30	0,00	0,00	0,00	0,00

3,40	1,50	0,30	0,10	0,10
3,73	2,17	0,31	0,00	0,00
3,14	0,43	0,00	0,00	0,00
6,98	3,88	0,00	0,78	0,78
2,82	1,26	0,29	0,00	0,00
10,45	6,30	2,30	0,00	0,00
5,15	6,19	4,12	0,00	0,00
5,72	5,15	3,05	0,10	0,00
2,06	0,11	0,00	0,00	0,00
3,94	4,36	1,41	0,14	0,00
1,69	0,56	0,38	0,19	0,00
6,70	4,12	5,15	0,00	0,00
5,00	2,50	0,83	0,00	0,00
3,43	0,31	0,00	0,00	0,00
3,13	1,47	0,74	0,00	0,00
3,52	0,59	0,00	0,00	0,00
6,25	6,40	4,32	0,30	1,04
5,57	7,69	3,71	0,27	0,27
5,07	4,81	3,20	0,80	0,00
3,56	6,14	4,53	0,43	0,32
2,92	4,29	0,86	0,17	0,00
0,96	1,35	0,00	0,00	0,00
2,50	0,94	0,94	0,00	0,00
5,41	6,22	6,22	0,92	0,92
6,44	6,59	2,35	0,16	0,16
7,74	4,84	1,57	0,36	0,00
0,52	0,00	0,00	0,00	0,00
3,31	6,08	2,21	0,00	0,00
3,14	1,29	0,18	0,00	0,00
6,65	2,81	0,77	0,00	0,00
6,59	5,54	3,67	0,15	0,15
15,91	2,27	0,00	2,27	0,00
5,21	0,00	0,00	0,00	0,00
3,31	1,10	0,00	0,00	0,00
4,08	1,53	0,26	0,00	0,00
6,17	4,78	1,98	0,07	0,07
2,63	1,44	0,60	0,00	0,00
6,17	2,50	1,67	0,50	0,17
0,80	2,40	0,80	0,00	0,00
2,55	0,64	0,32	0,64	0,00
1,94	0,49	0,16	0,00	0,00
4,32	2,07	0,56	0,19	0,00
1,68	0,00	0,00	0,00	0,00
2,55	0,85	0,34	0,00	0,00
5,45	1,82	3,64	0,00	0,00
0,00	0,00	0,00	0,00	0,00
0,62	0,16	0,00	0,00	0,00
4,47	1,30	0,29	0,00	0,00
2,60	1,07	0,61	0,15	0,00
12,50	8,33	8,33	0,00	0,00

6,80	4,73	4,44	0,89	0,30
4,47	7,11	3,42	0,26	0,26
5,08	3,81	0,42	0,00	0,00
4,30	2,90	1,20	0,30	0,00
11,11	4,96	2,36	0,00	0,00
1,16	1,26	0,32	0,00	0,11
1,84	0,61	0,00	0,00	0,00
12,05	6,10	0,94	0,00	0,00
0,28	0,28	0,00	0,00	0,00
4,08	1,22	2,04	0,00	0,00
1,23	6,17	0,00	0,00	0,00
1,63	1,91	0,27	0,00	0,00
2,76	2,76	0,55	0,00	0,00
4,52	1,51	1,36	0,15	0,00
2,38	1,28	0,73	0,18	0,00
1,02	0,51	0,00	0,00	0,00
1,28	0,92	0,73	0,00	0,00
6,90	5,62	2,57	0,00	0,00
2,76	1,75	0,50	0,08	0,08
4,65	1,49	0,56	0,00	0,19
1,70	0,50	0,10	0,10	0,00
1,55	0,86	0,00	0,00	0,00
5,56	2,20	1,68	0,00	0,00
3,22	2,11	0,60	0,10	0,00
4,33	4,12	2,06	0,00	0,00
2,30	0,00	0,00	0,00	0,00
3,35	2,79	1,12	0,00	0,00
4,55	4,97	2,90	1,24	0,00
3,85	4,65	6,69	1,36	1,70
6,52	4,66	3,91	0,74	0,19
5,95	2,98	0,00	0,00	0,00
6,92	6,92	1,26	0,00	0,00
5,65	6,45	3,23	0,00	0,00
4,82	2,19	1,75	0,00	0,00
2,31	2,94	1,26	0,21	0,00
6,83	2,90	0,34	0,00	0,00
4,74	5,87	3,39	0,23	0,23
4,89	4,14	1,63	0,13	0,13
1,44	0,96	0,00	0,00	0,00
6,05	4,56	3,31	0,58	0,17
2,79	1,40	0,47	0,00	0,00
1,65	0,55	0,00	0,00	0,00
5,83	6,31	1,94	0,00	0,00
3,90	1,95	0,00	0,00	0,00
1,28	0,00	0,00	0,00	0,00
10,00	2,00	0,00	0,00	0,00
1,64	1,64	2,05	0,00	0,00
3,60	1,60	1,20	0,00	0,00
4,16	1,58	0,99	0,00	0,00
4,84	3,17	0,83	0,00	0,17

3,65	2,22	1,75	0,00	0,16
1,75	1,11	0,32	0,00	0,00
8,05	2,53	0,69	0,00	0,00
5,13	0,00	0,37	0,37	0,37
3,85	7,69	3,85	0,00	0,00
2,28	1,37	0,00	0,00	0,00
0,00	0,00	0,00	0,00	0,00
2,81	2,11	1,83	0,14	0,00
3,31	4,07	1,53	0,00	0,00
0,00	0,00	0,00	0,00	0,00
5,20	4,83	1,49	0,37	0,00
2,75	0,92	0,15	0,15	0,00
8,73	5,53	2,50	0,00	0,00
5,96	3,38	2,19	0,00	0,00
3,00	2,15	1,72	0,00	0,00
2,13	1,12	0,22	0,00	0,00
1,59	0,00	1,59	0,00	0,00
1,59	0,53	0,26	0,00	0,00
4,72	3,69	1,38	0,12	0,00
3,16	1,99	0,12	0,12	0,00
2,53	1,15	0,46	0,00	0,00
3,62	1,24	0,26	0,09	0,00
7,32	3,41	1,46	0,00	0,24
1,34	0,67	0,34	0,00	0,00
3,92	3,92	1,83	0,26	0,00
6,11	3,05	0,76	0,00	0,00
4,80	4,00	0,80	0,80	0,00
5,48	5,97	3,39	0,48	0,16
4,41	3,63	2,33	0,26	0,00
3,01	1,81	0,00	0,00	0,00
1,67	0,84	0,00	0,00	0,00
2,13	0,00	0,00	0,00	0,00
1,95	3,90	0,32	0,00	0,65
1,44	0,72	0,24	0,00	0,00
6,52	2,17	0,93	0,00	0,00
2,19	1,04	0,12	0,00	0,00
5,90	6,23	5,25	0,33	0,55
0,00	0,00	0,00	0,00	0,00
1,00	0,20	0,00	0,00	0,00
2,82	5,63	2,82	0,00	0,00
10,53	4,39	3,51	0,00	0,00
1,53	0,38	0,00	0,00	0,00
6,87	1,91	1,15	0,00	0,00
0,67	0,00	0,00	0,00	0,00
0,00	0,00	0,00	0,00	0,00
4,48	0,50	0,50	0,00	0,00
1,92	0,72	0,24	0,00	0,00
0,00	0,00	0,00	0,00	0,00
2,44	0,00	0,81	0,81	0,00
3,21	0,00	1,07	0,00	0,00

0,00	0,00	0,00	0,00	0,00
4,53	1,49	0,47	0,00	0,00
4,07	0,90	0,63	0,09	0,00
3,68	2,21	0,74	0,00	0,00
2,72	4,21	0,99	0,25	0,00
3,17	2,57	1,06	0,00	0,00
1,54	2,23	1,54	0,00	0,00
5,88	4,49	1,10	0,10	0,00
3,45	3,45	0,43	0,00	0,00
4,84	2,33	0,72	0,09	0,09
3,74	2,36	1,39	0,00	0,00
1,02	2,21	0,17	0,17	0,17
2,10	1,68	0,56	0,00	0,00
5,57	2,09	1,04	0,00	0,12
0,00	0,00	0,00	0,00	0,00
4,37	1,75	0,79	0,00	0,00
1,79	1,28	0,51	0,00	0,26
4,02	2,44	1,15	0,00	0,00
5,42	2,14	0,88	0,13	0,00
4,90	1,68	0,70	0,00	0,14
2,17	1,08	0,14	0,00	0,14
2,02	0,91	0,33	0,00	0,00
1,10	0,55	0,22	0,00	0,00
1,07	0,75	0,00	0,00	0,00
2,96	1,59	0,46	0,00	0,00
2,42	1,12	1,12	0,00	0,00
7,09	5,06	2,21	0,09	0,18
1,03	0,34	0,00	0,00	0,00
3,05	2,39	1,59	0,13	0,00
2,80	0,31	0,31	0,00	0,00
2,08	0,83	0,10	0,00	0,00
3,26	2,03	0,44	0,09	0,00
2,32	0,55	0,00	0,00	0,00
3,36	1,81	0,26	0,00	0,00
3,20	1,24	0,36	0,18	0,00
2,16	0,38	0,13	0,00	0,00
4,13	2,06	1,67	0,00	0,00
6,52	3,16	2,08	0,20	0,00
2,51	2,26	1,00	0,00	0,00
4,04	2,63	1,58	0,26	0,00
3,62	2,14	1,76	0,19	0,00
5,60	4,81	3,00	0,39	0,00
2,66	1,99	0,96	0,30	0,07
4,52	1,69	0,66	0,00	0,19
3,56	2,10	0,42	0,00	0,00
3,16	2,37	0,45	0,23	0,00
3,82	1,27	1,38	0,00	0,11
3,82	1,51	0,46	0,00	0,00
2,79	1,62	0,81	0,00	0,00
5,88	2,75	1,75	0,25	0,13

3,81	2,19	0,58	0,00	0,12
2,90	2,57	1,24	0,00	0,25
3,80	4,19	1,70	0,13	0,00
2,33	1,10	0,98	0,49	0,12
4,27	1,75	0,87	0,00	0,00
2,59	2,12	0,35	0,12	0,12
3,36	1,90	1,17	0,00	0,00
4,31	4,53	3,13	0,65	0,32
3,70	2,12	1,59	0,18	0,18
1,43	0,24	0,00	0,00	0,00
3,17	1,75	1,33	0,08	0,00
5,69	2,34	0,50	0,00	0,00
4,98	1,52	0,55	0,00	0,00
4,43	1,81	0,49	0,16	0,16
2,47	0,65	0,00	0,00	0,00
1,84	0,53	0,13	0,00	0,00
0,62	0,31	0,00	0,00	0,00
2,41	0,90	0,00	0,00	0,00
3,71	2,41	0,72	0,00	0,00
5,58	2,02	0,78	0,00	0,00
3,77	1,21	1,08	0,00	0,13
3,78	2,66	0,84	0,00	0,00
3,14	2,29	1,14	0,00	0,00
2,54	2,03	1,52	0,00	0,00
3,86	2,44	1,29	0,26	0,13
3,59	2,51	2,18	0,00	0,11
3,80	2,11	2,32	0,42	0,11
3,73	2,88	0,85	0,64	0,00
3,55	2,96	0,39	0,20	0,00
3,80	3,35	2,12	0,11	0,00
4,24	2,54	1,27	0,11	0,11
3,73	2,44	1,00	0,14	0,00
4,78	3,36	2,39	0,27	0,09
4,36	4,49	3,49	0,50	0,12
7,55	5,27	4,70	1,85	0,28
5,84	6,59	6,27	0,32	0,22
8,75	4,38	1,25	0,16	0,00
6,20	5,38	2,57	0,58	0,00
6,87	5,36	2,35	0,34	0,00
5,56	5,77	3,88	0,42	0,00
2,43	1,26	0,25	0,00	0,00
3,11	1,86	0,93	0,00	0,00
3,05	1,59	1,72	0,00	0,13
2,47	2,76	1,60	0,00	0,15
1,47	1,62	1,32	0,00	0,00
4,54	2,36	1,81	0,36	0,00
4,42	2,21	2,21	0,95	0,00
5,74	2,92	1,85	0,58	0,00
4,89	4,12	1,98	0,31	0,00
5,88	5,20	2,19	0,27	0,14

5,05	1,38	0,92	0,00	0,00
1,59	0,75	0,00	0,09	0,00
3,05	0,90	0,11	0,00	0,00
2,46	1,67	0,87	0,32	0,08
4,29	5,04	2,05	0,56	0,47
3,67	2,27	0,87	0,52	0,00
4,57	3,60	2,35	0,00	0,28
4,83	3,26	1,91	0,45	0,22
4,38	2,50	2,14	0,09	0,18
6,27	5,68	6,98	0,12	1,54
3,95	1,05	1,32	0,26	0,26
6,12	0,00	2,04	0,00	0,00
3,77	0,63	0,63	0,00	0,00
1,82	1,09	0,00	0,00	0,00
0,67	0,67	0,22	0,00	0,00
3,95	2,26	0,75	0,00	0,00
4,12	2,06	3,09	0,00	0,00
3,35	2,23	1,12	0,00	0,00
2,41	3,22	0,48	0,00	0,48
5,01	3,62	1,39	0,00	0,28
1,65	0,41	0,28	0,00	0,00
7,79	2,60	3,90	0,00	0,00
5,43	4,73	2,45	0,35	0,00
5,95	2,30	2,30	0,41	0,00
4,06	2,12	1,06	0,00	0,00
1,92	2,07	0,44	0,00	0,15
2,86	2,38	1,43	0,00	0,00
0,00	0,00	0,00	0,00	0,00
3,29	2,64	0,99	0,16	0,00
5,88	0,00	0,00	0,00	0,00
2,48	1,24	0,74	0,00	0,00
7,17	3,59	0,90	0,00	0,45
3,87	2,03	1,10	0,37	0,00
3,55	6,26	2,30	0,21	0,21
1,59	1,36	1,13	0,00	0,23
6,24	2,62	1,21	0,20	0,07
5,21	1,04	1,04	0,00	0,00
2,20	0,00	0,00	0,00	0,00
0,94	3,77	0,94	0,00	0,00
3,57	7,14	0,00	0,00	0,00
4,18	3,97	1,07	0,00	0,11
3,07	1,53	1,19	0,00	0,00
2,04	2,04	0,00	0,00	0,00
2,45	2,30	0,31	0,00	0,00
4,86	3,47	0,00	0,00	0,00
3,09	1,85	0,00	0,00	0,00
5,04	0,00	1,16	0,00	0,00
2,15	0,72	0,36	0,00	0,00
1,16	0,58	0,00	0,00	0,00
1,10	0,00	0,00	0,00	0,27

7,44	5,01	2,12	0,15	0,30
3,11	4,35	0,62	0,00	0,00
1,32	1,19	0,53	0,00	0,00
3,37	1,12	0,00	0,00	0,00
0,00	0,00	0,00	0,00	0,00
0,00	0,00	0,00	0,00	0,00
3,96	2,97	2,97	0,00	0,00
3,14	2,27	1,22	0,00	0,00
3,89	2,25	1,23	0,20	0,00
5,20	4,46	1,86	0,93	0,37
1,87	1,53	0,34	0,00	0,00
3,33	0,67	0,67	0,00	0,00
3,90	3,15	2,40	0,00	0,00
3,84	2,30	1,15	0,00	0,19
2,78	0,93	0,23	0,12	0,00
2,13	0,00	0,35	0,00	0,00
0,79	0,00	0,00	0,00	0,00
3,39	1,63	1,79	0,19	0,08
0,00	0,00	0,00	0,00	0,00
4,90	2,36	0,73	0,00	0,00
4,95	2,34	0,78	0,00	0,26
2,76	1,00	1,25	0,00	0,00
3,67	2,29	2,52	0,23	0,23
3,76	2,97	1,56	0,31	0,31
3,42	2,99	0,85	0,43	0,00
3,91	4,49	2,60	0,00	0,00
3,13	2,42	1,21	0,51	0,51
3,64	1,11	1,27	0,00	0,00
3,60	3,82	2,02	0,22	0,00
4,93	2,11	6,34	0,00	0,70
3,77	2,33	1,08	0,18	0,36
1,55	0,00	0,52	0,00	0,00
1,86	0,72	0,72	0,14	0,00
0,00	0,00	0,00	0,00	0,00
1,70	0,61	0,61	0,00	0,00
1,96	3,92	0,98	0,00	0,00
3,39	1,69	1,96	0,13	0,00
2,67	1,78	1,56	0,00	0,22
1,67	1,25	0,21	0,00	0,00
3,58	3,27	1,40	0,16	0,00
3,23	2,15	0,62	0,15	0,00
5,03	5,03	1,89	0,00	0,00
9,40	3,21	4,36	0,69	0,23
4,87	5,65	3,12	0,19	0,00
9,78	4,45	1,02	0,13	0,00
7,79	4,08	0,99	0,12	0,12
0,00	0,00	0,00	0,00	0,00
0,95	0,32	0,32	0,00	0,00
5,57	4,01	2,23	0,00	0,00
2,29	0,71	0,09	0,09	0,00

5,02	2,01	1,25	0,00	0,13
5,87	2,46	1,50	0,00	0,00
5,56	3,49	2,94	0,22	0,11
4,25	3,82	1,13	0,42	0,00
3,90	2,97	2,60	0,56	0,00
5,16	4,69	1,09	0,00	0,00
2,94	3,17	1,18	0,24	0,00
8,09	3,85	1,86	0,13	0,13
4,76	2,56	1,83	0,00	0,00
5,28	3,25	1,63	0,00	0,00
2,60	0,48	0,29	0,10	0,00
2,63	0,95	0,22	0,00	0,07
2,31	0,62	0,31	0,08	0,00
2,44	0,24	0,24	0,12	0,00
1,73	0,71	0,20	0,00	0,00
2,52	0,25	0,38	0,00	0,00
3,59	2,20	1,00	0,10	0,00
3,44	2,50	1,25	0,16	0,16
4,58	2,29	1,15	0,00	0,16
5,22	3,04	1,30	0,00	0,00
5,11	3,87	3,08	0,88	0,09
5,07	4,50	3,23	0,58	0,23
7,43	4,75	4,16	0,30	0,30
4,57	3,88	2,49	0,55	0,00
5,39	5,39	5,25	1,02	0,73
5,92	7,37	6,85	1,14	0,10
3,56	2,78	2,12	0,11	0,00
3,81	2,73	2,65	0,58	0,17
4,42	3,04	1,73	0,21	0,14
2,16	1,44	0,48	0,00	0,00
3,32	1,92	1,75	0,17	0,00
4,93	3,01	1,93	0,12	0,00
2,28	0,68	0,31	0,00	0,00
3,82	1,68	1,38	0,00	0,15
2,49	0,65	0,09	0,00	0,00
1,19	0,42	0,51	0,00	0,00
2,19	1,09	0,20	0,00	0,00
1,02	1,02	0,34	0,00	0,00
0,00	0,00	0,00	0,00	0,00
4,56	1,97	1,26	0,00	0,08
5,80	2,52	1,75	0,44	0,11
6,06	3,86	1,87	0,11	0,11
4,46	2,84	1,52	0,30	0,30
5,36	4,90	4,07	0,46	0,28
5,38	2,58	2,13	0,22	0,34
3,85	2,97	2,09	0,33	0,11
5,07	4,21	3,78	1,19	0,22
4,81	3,70	0,99	0,12	0,25
6,75	3,05	1,96	0,65	0,22
4,19	7,67	7,16	0,51	0,61

5,48	7,30	8,74	1,55	0,72
7,04	9,88	9,21	0,50	0,34
8,78	8,54	4,63	0,95	0,83
5,00	2,50	2,92	1,25	0,00
5,08	4,31	4,31	0,51	0,00
4,23	7,09	5,72	0,75	0,62
8,01	7,55	3,85	0,15	0,62
7,86	4,29	3,33	0,48	0,24
4,28	2,28	1,57	0,29	0,29
1,01	0,76	0,00	0,00	0,00
1,29	0,83	0,09	0,00	0,00
1,66	1,54	0,64	0,00	0,00
3,86	0,86	0,00	0,00	0,43
3,24	0,81	0,41	0,00	0,00
5,16	2,79	1,53	0,00	0,00
3,90	4,53	1,64	0,25	0,13
4,85	3,08	1,67	0,00	0,00
2,72	1,90	2,72	0,54	0,00
5,57	3,38	1,42	0,55	0,33
4,08	2,34	0,61	0,00	0,00
3,26	1,95	0,65	0,16	0,00
4,44	3,33	1,11	0,00	0,00
4,14	1,87	1,47	0,40	0,00
4,34	3,69	2,86	0,37	0,28
3,86	2,60	2,02	0,08	0,00
3,68	4,55	2,13	0,19	0,29
5,59	5,91	5,11	0,80	0,00
5,84	7,04	6,37	0,66	0,53
5,45	3,27	3,74	0,31	0,00
7,33	4,21	1,83	0,37	0,00
1,24	0,47	0,31	0,00	0,00
6,08	2,62	2,10	0,00	0,00
4,73	2,37	1,42	0,00	0,00
6,34	2,11	1,81	0,30	0,00
0,89	0,11	0,00	0,00	0,00
3,69	2,46	0,15	0,00	0,00
3,26	1,67	0,70	0,00	0,00
1,43	0,54	0,00	0,00	0,00
2,63	2,53	1,52	0,20	0,10
5,15	3,13	0,55	0,00	0,18
5,96	2,55	1,13	0,43	0,43
3,41	2,19	0,97	0,24	0,00
4,99	2,46	1,01	0,14	0,14
3,69	2,30	0,66	0,08	0,16
1,95	0,72	0,10	0,00	0,00
1,30	0,98	0,11	0,00	0,00
1,85	0,44	0,11	0,00	0,00
1,50	0,71	0,27	0,00	0,00
4,76	3,69	1,08	0,15	0,00
3,77	2,98	1,09	0,00	0,00

5,53	3,20	1,55	0,00	0,00
5,20	2,90	2,21	0,26	0,17
5,26	3,06	1,91	0,10	0,00
0,94	0,60	0,09	0,00	0,00
0,53	0,33	0,26	0,00	0,07
3,91	2,30	1,15	0,23	0,11
6,11	9,81	6,37	1,27	0,25
5,39	8,96	6,55	0,83	0,33
0,00	0,00	0,00	0,00	0,00
3,14	1,32	0,83	0,00	0,00
3,88	1,29	1,46	0,00	0,00
4,59	4,32	2,12	0,35	0,09
4,37	4,07	2,03	0,41	0,00
3,83	2,46	1,37	0,27	0,00
3,27	3,38	1,92	0,68	0,00
4,33	3,69	2,17	0,00	0,00
4,59	4,59	1,57	0,12	0,12
3,31	2,28	1,66	0,00	0,00
5,18	3,03	2,15	0,29	0,20
3,66	2,91	1,00	0,17	0,08
2,50	2,88	0,83	0,19	0,00
3,50	1,97	0,77	0,33	0,00
2,00	1,43	1,33	0,10	0,00
2,44	1,36	0,81	0,54	0,00
1,68	0,90	0,26	0,00	0,00
2,51	0,63	0,25	0,00	0,00
2,96	2,86	1,18	0,00	0,20
2,39	1,86	0,13	0,00	0,00
2,11	0,70	0,28	0,00	0,00
2,31	0,98	0,71	0,00	0,00
0,36	0,00	0,24	0,00	0,00
4,52	1,94	2,58	0,65	0,00
4,60	3,92	0,51	0,00	0,00
5,07	4,03	1,34	0,30	0,15
4,48	5,63	3,96	1,02	0,13
2,55	2,20	0,93	0,12	0,23
3,85	1,48	0,59	0,15	0,15
4,84	4,12	3,09	0,21	0,10
3,95	2,26	0,90	0,00	0,11
3,82	2,52	0,78	0,00	0,09
3,98	2,96	1,63	0,31	0,00
2,90	1,98	0,61	0,31	0,15
3,57	0,00	1,19	0,00	0,00
3,55	2,49	1,92	0,00	0,10
6,13	3,36	0,99	0,40	0,00
6,94	7,90	8,67	1,35	0,39
4,17	10,00	2,50	1,67	0,83
3,54	4,11	1,70	0,42	0,28
4,26	3,00	0,95	0,16	0,00
5,26	1,82	3,04	0,20	0,20

3,24	2,74	0,75	0,12	0,25
1,30	1,52	0,00	0,00	0,22
2,30	2,62	0,00	0,00	0,33
2,39	0,27	0,80	0,00	0,00
2,25	0,96	0,43	0,00	0,00
1,31	0,12	0,12	0,00	0,00
3,66	1,83	1,05	0,26	0,00
6,28	2,26	1,01	0,25	0,25
5,75	5,32	4,03	0,52	0,43
4,23	5,44	3,63	0,91	0,30
5,31	5,45	7,13	1,40	0,56
6,41	8,24	6,18	1,14	0,23
3,72	4,91	4,91	1,19	0,30
4,13	7,15	5,09	1,27	0,79
5,49	5,17	8,15	1,88	0,63
4,06	2,24	2,74	0,41	0,00
3,07	4,25	8,96	4,01	4,95
4,01	2,47	1,70	0,00	0,00
5,34	5,88	7,08	1,42	1,63
4,12	6,90	7,47	2,11	1,15
3,59	6,44	7,49	0,60	2,10
4,19	6,54	6,28	1,05	0,26
3,49	2,77	3,73	0,48	0,24
3,19	3,31	3,42	0,24	0,71
5,02	7,62	8,43	0,81	0,16
5,48	5,74	4,43	0,26	0,39
5,71	5,15	7,80	1,81	0,84
6,31	6,54	2,34	0,00	0,00
7,19	7,54	5,15	0,96	0,36
5,03	8,09	5,34	0,69	0,46
0,97	0,14	0,14	0,00	0,00
0,86	0,34	0,00	0,00	0,17
2,40	2,92	1,03	0,00	0,00
6,41	4,84	2,78	0,12	0,12
7,04	5,65	2,48	0,10	0,20
3,77	3,08	2,85	0,11	0,11
3,57	1,66	0,48	0,12	0,00
3,83	3,09	0,94	0,27	0,07
3,06	2,30	1,31	0,11	0,00
8,53	6,46	3,36	0,65	0,26
1,52	0,85	0,34	0,00	0,00
5,13	3,39	2,02	0,37	0,37
3,28	5,11	4,66	0,76	0,31
7,70	3,60	1,90	0,20	0,10
3,20	0,75	0,38	0,00	0,00
3,16	1,34	0,49	0,00	0,00
4,84	2,99	1,03	0,31	0,00
4,75	2,89	0,83	0,10	0,10
2,94	1,31	0,57	0,00	0,00
3,72	2,90	0,97	0,00	0,00

3,23	2,87	0,48	0,12	0,24
2,74	2,47	1,33	0,09	0,09
3,88	2,59	2,12	0,35	0,00
2,03	1,01	0,10	0,00	0,00
0,92	0,38	0,00	0,00	0,00
2,05	0,00	0,68	0,00	0,00
4,70	2,61	1,22	0,35	0,00
0,00	0,00	0,00	0,00	0,00
1,68	1,50	0,47	0,00	0,00
2,16	1,08	0,32	0,11	0,00
1,63	0,10	0,10	0,00	0,00
1,34	0,54	0,27	0,00	0,00
0,36	0,18	0,00	0,00	0,00
8,90	1,34	0,85	0,12	0,00
4,57	3,95	1,46	0,83	0,21
3,59	3,87	4,70	1,10	0,00
2,80	6,40	7,20	0,00	1,60
2,68	3,68	5,02	0,33	0,00
3,22	3,86	2,42	0,48	0,16
0,82	2,46	0,00	0,00	0,00
5,59	6,98	4,19	0,28	0,28
5,10	8,67	3,06	0,51	0,00
7,52	7,42	3,66	0,10	0,10
3,52	1,17	0,88	0,00	0,00
4,92	2,19	1,09	0,55	0,00
1,09	0,66	0,22	0,00	0,00
3,63	3,43	1,01	0,20	0,20
4,32	3,24	2,70	0,00	0,00
3,16	2,53	0,63	0,00	0,00
3,89	2,29	1,83	0,23	0,00
1,28	0,00	0,18	0,00	0,00
6,02	4,42	4,42	0,40	0,40
3,59	1,20	1,59	0,00	0,00
1,89	1,26	0,21	0,00	0,00
6,01	3,86	2,58	0,00	0,00
3,49	1,53	0,44	0,00	0,00
3,58	1,97	0,35	0,12	0,00
2,81	0,99	0,50	0,00	0,00
3,70	3,70	0,46	0,23	0,00
1,06	0,24	0,00	0,00	0,00
5,25	2,28	1,14	0,00	0,00
3,03	1,52	0,00	0,00	0,00
1,92	2,88	0,96	0,00	0,00
8,20	1,64	1,64	0,00	0,82
1,14	1,14	1,14	0,00	0,00
0,00	0,00	0,00	0,00	0,00
2,22	3,33	2,22	0,00	0,00
4,76	3,17	1,59	0,00	0,00
6,28	6,54	3,66	2,88	0,52
4,76	2,38	1,19	0,00	0,00

3,29	1,58	0,37	0,00	0,00
2,03	1,12	0,07	0,07	0,00
1,64	1,56	0,08	0,08	0,00
2,09	0,49	0,37	0,00	0,00
3,23	1,45	0,16	0,00	0,00
4,10	2,50	1,61	0,00	0,00
4,26	2,84	1,23	0,09	0,09
4,84	2,86	1,98	0,00	0,00
5,50	4,59	0,00	0,00	0,00
8,76	3,65	0,73	1,46	0,00
1,92	0,64	0,00	0,00	0,00
2,59	2,07	0,52	0,00	0,00
3,47	2,42	1,51	0,15	0,15
2,29	6,11	3,82	0,00	0,76
5,14	3,43	2,86	0,57	0,00
2,11	1,05	0,35	0,00	0,18
4,35	3,66	1,60	0,23	0,23
4,89	4,35	1,63	0,27	0,54
3,57	1,98	0,00	0,00	0,00
1,17	1,75	1,75	0,00	0,00
2,61	4,78	1,74	0,00	0,43
2,23	3,35	1,12	0,56	0,00
1,81	1,44	0,72	0,00	0,00
1,85	3,24	0,93	0,00	0,00
0,00	0,00	0,00	0,00	0,00
1,94	0,97	0,00	0,00	0,00
1,89	0,75	0,38	0,00	0,00
3,10	2,21	1,77	0,00	0,00
2,61	1,96	0,33	0,00	0,00
4,15	3,11	1,38	0,00	0,00
5,13	3,42	3,42	0,00	0,00
3,64	2,21	1,21	0,00	0,00
7,69	2,56	1,03	0,00	0,51
2,67	3,23	1,26	0,00	0,00
1,59	0,12	0,49	0,12	0,00
3,93	1,75	0,22	0,00	0,00
1,93	0,77	0,39	0,00	0,00
1,63	0,82	0,16	0,00	0,00
2,22	1,33	0,22	0,00	0,00
2,46	2,11	0,88	0,00	0,18
6,62	4,41	4,41	0,74	0,00
3,21	6,71	3,50	0,58	0,58
2,83	2,83	4,25	1,89	0,94
12,21	5,16	1,41	0,00	0,00
3,37	2,02	0,34	0,00	0,11
3,90	2,89	1,36	0,00	0,00
3,34	1,56	0,67	0,00	0,00
3,86	3,61	1,45	0,48	0,00
4,57	3,43	2,12	0,49	0,16
5,89	5,05	4,21	0,14	0,28

1,81	0,68	0,11	0,00	0,00
6,79	4,85	3,88	0,24	0,24
4,79	3,67	2,40	0,00	0,48
8,53	5,43	0,00	0,00	0,00
7,83	3,04	1,09	0,22	0,00
12,64	5,06	0,46	0,00	0,00
7,68	6,98	2,97	0,00	0,00
4,88	2,86	1,43	0,00	0,00
4,16	1,50	1,33	0,17	0,00
1,48	0,63	0,21	0,00	0,11
1,16	0,64	0,51	0,00	0,00
2,26	0,94	0,19	0,00	0,00
3,13	3,13	1,39	0,00	0,00
1,45	1,13	0,32	0,32	0,00
0,00	2,56	1,28	0,00	0,00
1,22	0,00	0,00	0,00	0,00
8,12	6,86	4,33	0,00	0,18
3,05	4,66	2,89	0,00	0,16
9,99	3,13	1,64	0,00	0,00
3,88	4,85	6,60	0,97	1,17
3,33	1,19	3,10	0,00	0,24
4,43	3,01	1,90	0,00	0,16
3,54	2,23	1,31	0,13	0,00
6,07	2,80	2,18	0,31	0,00
2,89	3,43	1,81	0,36	0,00
2,24	2,74	1,24	0,00	0,00
5,21	3,19	1,18	0,00	0,17
1,36	0,90	0,18	0,00	0,00
3,70	0,41	1,65	0,00	0,00
5,36	7,54	8,33	2,18	0,40
7,81	4,18	2,51	0,42	0,00
4,06	4,93	9,72	1,60	1,16
7,28	7,59	5,31	0,61	0,15
3,44	3,44	0,00	0,00	0,00
1,46	0,29	0,00	0,00	0,00
2,28	0,13	0,27	0,00	0,13
1,10	0,73	0,73	0,37	0,00
0,00	0,00	0,00	0,00	0,00
0,39	0,00	0,00	0,00	0,00
3,11	1,50	0,54	0,11	0,00
3,92	2,94	0,00	0,00	0,00
1,69	2,26	1,13	0,00	0,00
5,00	0,00	1,25	0,00	0,00
4,76	0,00	0,00	0,00	0,00
6,82	2,27	0,00	0,00	0,00
2,54	0,85	0,00	0,00	0,00
8,00	2,67	2,67	1,33	0,00
1,75	1,75	0,88	0,00	0,00
0,00	0,00	0,00	0,00	0,00
4,51	3,01	0,00	0,00	0,00

2,65	1,70	0,57	0,38	0,00
0,00	0,00	0,00	0,00	0,00
2,09	4,70	6,27	1,83	1,83
3,26	7,01	9,79	2,45	2,61
4,29	5,99	8,04	2,23	1,79
4,10	7,82	7,82	2,18	1,41
3,58	5,92	8,78	1,63	2,25
3,61	4,91	8,93	0,90	0,50
4,44	7,89	7,40	0,82	0,66
5,56	7,41	6,67	1,48	0,74
5,49	6,28	7,40	1,12	1,57
3,49	4,92	8,73	3,02	3,33
3,20	5,94	6,39	1,37	1,48
4,22	5,43	7,36	1,45	0,48
2,58	5,85	10,77	1,87	1,41
3,83	5,24	4,84	1,01	0,81
4,28	6,58	8,35	2,51	0,42
4,04	6,28	6,58	2,09	0,90
4,94	8,70	7,76	2,00	1,29
7,90	7,90	5,50	2,23	0,86
3,42	5,68	8,65	1,53	1,35
4,56	3,80	5,02	0,91	0,30
3,62	6,03	7,12	1,81	1,45
5,61	9,74	7,92	1,16	0,83
4,55	4,55	2,92	0,65	0,49
1,93	2,25	2,89	0,00	0,32
5,26	6,25	8,72	1,32	0,82
2,94	2,94	7,83	3,30	4,04
3,35	4,65	6,45	3,10	1,55
2,71	4,29	5,87	0,68	1,35
5,78	6,23	8,21	1,82	1,82
3,78	5,72	8,47	2,06	1,03
5,76	5,39	7,72	2,33	1,84
3,42	6,25	9,20	3,42	1,30
4,30	5,61	7,52	1,31	0,72
0,78	0,00	0,00	0,00	0,00
3,92	7,51	9,79	2,50	1,96
3,47	3,96	8,17	1,24	1,49
3,79	5,98	9,04	0,73	1,17
0,00	0,00	0,00	0,00	0,00
4,30	5,79	8,01	2,23	0,89
4,70	8,72	12,58	2,18	0,34
3,87	3,17	5,63	1,06	0,35
6,19	5,60	7,67	0,88	0,00
3,94	6,26	9,12	1,61	1,07
4,83	6,64	10,06	3,02	0,40
6,85	7,08	6,85	1,60	0,23
2,99	2,99	4,48	2,24	0,00
3,88	5,77	10,21	3,33	2,22
8,77	5,85	3,51	1,17	0,00

2,04	3,27	2,86	0,82	0,00
5,01	6,09	8,38	2,18	2,39
3,91	5,90	4,25	1,39	2,43
5,27	6,81	9,25	2,70	0,90
3,09	7,35	10,44	2,58	2,32
0,81	1,61	0,00	0,00	0,00
3,41	0,62	0,00	0,00	0,00
7,11	8,82	10,05	1,23	0,98
0,00	0,00	0,00	0,00	0,00
4,50	4,14	1,80	1,08	0,36
3,55	4,43	4,66	0,22	0,67
8,32	9,86	6,38	0,77	0,39
2,02	7,07	5,05	0,00	2,02
5,07	9,86	7,32	0,28	0,56
8,39	8,06	5,26	1,15	0,66
2,73	1,36	0,91	0,00	0,00
2,73	1,95	0,00	0,00	0,00
4,94	3,90	1,69	0,26	0,26
4,53	3,29	1,23	0,00	0,21
2,64	1,45	0,40	0,00	0,00
5,47	3,65	1,82	0,29	0,00
5,14	1,71	2,69	0,61	0,37
5,56	2,51	1,72	0,13	0,13
4,07	4,81	2,22	0,00	0,00
4,21	8,24	4,58	0,73	0,92
3,88	3,43	1,55	0,00	0,11
4,09	2,05	1,83	0,11	0,00
4,12	2,25	0,75	0,00	0,00
4,11	2,06	0,88	0,29	0,00
5,53	2,61	0,94	0,00	0,00
4,26	4,09	1,15	0,00	0,49
4,38	2,55	1,19	0,36	0,18
4,60	3,68	2,76	0,00	0,46
3,22	2,06	2,06	0,39	0,13
3,69	5,54	4,62	0,00	0,00
2,28	3,23	1,92	0,36	0,12
5,74	4,31	2,48	0,13	0,13
3,53	1,47	0,59	0,00	0,00
5,81	3,54	4,63	0,79	0,30
5,65	4,05	2,13	0,21	0,21
4,20	5,79	5,23	0,63	1,03
6,10	5,71	4,42	0,65	0,13
4,99	7,03	4,31	0,23	0,23
5,09	4,80	2,76	0,58	0,00
3,64	2,15	1,03	0,09	0,00
4,35	1,84	0,67	0,17	0,00
3,70	2,34	1,11	0,00	0,12
6,09	5,59	1,15	0,00	0,00
3,95	2,81	1,66	0,21	0,00
3,07	0,61	0,31	0,00	0,00

4,32	2,83	1,34	0,00	0,00
5,66	1,04	1,64	0,00	0,00
5,34	5,64	3,96	0,30	0,00
3,53	1,38	1,23	0,15	0,00
4,07	2,95	1,40	0,00	0,00
4,55	2,94	0,89	0,00	0,18
2,35	2,04	0,63	0,16	0,00
3,58	1,26	0,77	0,29	0,00
4,76	2,86	1,14	0,38	0,00
4,60	2,65	0,84	0,00	0,14
5,52	3,83	1,78	0,45	0,18
2,88	0,36	1,80	0,00	0,00
4,34	2,58	1,88	0,00	0,12
4,30	4,30	1,77	0,33	0,00
3,93	3,62	1,57	0,47	0,00
5,33	7,75	1,62	0,00	0,16
7,01	4,01	2,72	0,14	0,00
5,62	3,50	1,75	0,12	0,12
4,77	5,64	4,91	0,29	0,72
2,67	4,77	2,67	0,70	0,47
6,30	6,24	7,43	1,00	0,80
3,57	7,05	6,55	0,89	0,79
4,57	6,71	5,39	1,12	0,20
4,62	2,39	1,03	0,00	0,17
8,05	5,23	2,82	0,00	0,20
3,97	3,35	2,51	0,21	0,21
6,54	5,43	4,70	1,20	0,18
5,63	7,19	7,50	0,63	0,16
6,24	6,24	4,92	0,35	0,35
2,66	3,24	2,00	0,19	0,10
7,28	4,08	2,22	0,09	0,00
2,20	1,10	1,10	0,00	0,00
4,00	3,20	0,00	0,00	0,00
6,75	3,13	1,38	0,38	0,13
6,09	4,52	3,13	0,17	0,00
3,11	3,11	1,31	0,16	0,00
1,60	0,80	0,00	0,00	0,00
1,92	0,56	0,23	0,00	0,00
3,60	2,00	1,20	0,00	0,40
4,40	3,96	1,54	0,00	0,00
2,44	1,30	0,33	0,16	0,00
3,53	3,53	1,45	0,21	0,21
2,90	2,17	0,00	0,00	0,00
5,99	2,24	1,87	0,12	0,12
6,54	4,21	0,93	0,00	0,00
1,23	0,61	0,15	0,08	0,00
5,39	3,47	0,90	0,13	0,00
4,10	3,02	2,54	0,39	0,00
5,85	2,46	1,29	0,12	0,00
5,49	3,94	2,38	0,00	0,27

1,79	0,26	0,26	0,00	0,00
1,99	1,22	0,33	0,11	0,11
0,87	0,87	0,35	0,00	0,00
4,18	1,49	1,19	0,00	0,00
0,80	0,80	0,20	0,00	0,00
1,68	0,61	0,00	0,00	0,00
4,35	1,78	0,89	0,00	0,00
5,33	2,72	1,59	0,23	0,11
3,98	3,66	2,72	0,00	0,10
3,61	2,45	0,82	0,12	0,00
3,45	3,45	1,64	0,09	0,00
5,78	4,02	1,59	0,08	0,08
2,02	1,77	0,50	0,13	0,13
3,58	2,61	0,96	0,14	0,00
1,74	5,81	0,00	1,16	0,00
3,07	1,90	0,59	0,00	0,00
1,28	1,28	0,32	0,00	0,00
4,53	2,04	1,36	0,11	0,23
3,88	1,94	0,00	0,00	0,00
3,90	2,02	1,21	0,13	0,00
4,05	1,96	0,52	0,00	0,13
3,79	1,63	0,95	0,14	0,00
5,15	3,34	0,70	0,00	0,00
8,90	6,92	2,80	0,16	0,00
5,41	5,31	2,07	0,20	0,00
4,01	3,19	1,91	0,00	0,00
3,99	3,16	2,16	0,17	0,33
3,61	2,46	0,79	0,00	0,09
4,51	2,54	1,13	0,00	0,28
3,77	2,83	2,35	0,31	0,47
2,23	2,98	2,61	0,00	0,37
7,40	7,82	5,45	0,84	0,28
6,62	5,98	1,50	0,00	0,00
3,69	2,06	1,74	0,11	0,22
0,78	0,16	0,00	0,00	0,00
1,09	0,93	0,62	0,00	0,00
2,92	0,33	0,17	0,00	0,00
4,79	4,89	3,72	0,29	0,20
4,95	6,04	4,67	1,37	0,69
0,84	1,01	0,00	0,00	0,00
2,26	0,75	0,00	0,00	0,00
0,90	0,30	0,00	0,00	0,00
7,36	7,88	7,23	0,13	0,13
3,73	3,05	1,36	0,45	0,45
9,92	10,74	5,79	1,16	0,33
4,67	5,18	3,84	0,33	1,00
5,84	4,71	3,81	0,24	0,24
4,77	3,33	2,79	0,45	0,00
5,23	5,46	4,15	0,61	0,31
6,07	6,33	6,24	0,95	0,35

3,85	5,98	4,97	0,81	0,20
6,42	5,66	6,98	0,57	0,75
5,74	7,79	3,28	0,51	0,31
6,68	5,88	2,67	0,00	0,00
1,18	0,89	0,00	0,00	0,00
3,59	4,19	0,60	0,00	0,60
5,10	4,65	2,10	0,30	0,00
5,94	4,95	1,49	0,83	0,00
4,02	4,10	3,45	0,90	0,08
6,59	3,81	1,17	0,44	0,15
3,04	3,39	2,11	0,35	0,00
4,61	5,67	4,23	0,38	0,38
5,61	7,91	7,14	0,77	0,38
2,23	0,67	0,00	0,00	0,00
1,12	0,28	0,00	0,00	0,00
2,82	2,12	1,27	0,00	0,28
1,22	0,33	0,11	0,00	0,00
2,66	3,33	2,11	0,44	0,11
5,83	4,03	2,65	0,21	0,11
5,84	4,22	2,70	0,18	0,00
0,99	0,62	0,00	0,00	0,00
3,12	2,69	1,42	0,57	0,00
6,25	4,04	1,84	0,12	0,12
5,73	3,01	1,00	0,14	0,29
3,82	3,16	2,24	0,13	0,26
4,02	2,54	1,72	0,16	0,00
5,19	3,14	1,26	0,47	0,00
5,25	2,46	1,07	0,11	0,00
3,84	2,46	0,92	0,00	0,00
3,47	2,44	1,41	0,28	0,00
3,30	1,22	0,49	0,00	0,00
4,06	1,88	0,63	0,21	0,00
5,10	3,27	1,22	0,15	0,08
3,43	1,59	0,92	0,00	0,00
4,32	2,78	0,31	0,00	0,00
1,78	0,59	0,30	0,00	0,00
4,08	2,27	1,21	0,30	0,00
3,24	1,98	1,26	0,54	0,00
2,54	1,67	0,40	0,00	0,00
1,87	1,24	0,36	0,00	0,00
4,07	3,31	1,02	0,00	0,00
3,15	2,06	0,73	0,00	0,00
3,28	2,67	0,61	0,00	0,00
4,09	2,92	0,70	0,23	0,00
2,34	0,41	0,30	0,00	0,00
3,99	2,28	0,38	0,00	0,09
3,62	1,69	1,09	0,00	0,00
2,01	0,53	0,32	0,11	0,11
3,79	1,79	0,63	0,11	0,00
5,12	0,34	0,00	0,00	0,00

2,86	0,72	0,89	0,00	0,00
1,55	0,09	0,09	0,00	0,00
3,71	0,65	0,08	0,08	0,00
3,64	3,16	0,95	0,32	0,00
3,74	2,20	0,08	0,00	0,08
4,17	1,79	0,00	0,00	0,00
4,55	2,45	0,58	0,00	0,00
3,32	1,28	0,38	0,00	0,00
2,91	1,35	0,67	0,00	0,00
2,96	0,53	0,11	0,00	0,00
1,88	1,29	0,82	0,00	0,00
2,25	1,46	0,40	0,07	0,00
2,23	0,97	0,84	0,00	0,14
3,53	2,12	1,84	0,14	0,00
1,66	0,74	0,37	0,00	0,00
2,32	0,89	0,36	0,00	0,00
3,65	2,71	0,85	0,25	0,00
4,56	1,24	2,49	0,10	0,00
6,09	4,59	2,53	0,19	0,00
5,30	2,17	1,20	0,24	0,00
3,25	1,04	1,33	0,15	0,00
5,56	3,51	1,17	0,00	0,00
6,40	5,22	3,54	0,39	0,10
4,45	2,44	0,84	0,00	0,08
3,43	1,81	0,72	0,18	0,00
0,95	0,00	0,32	0,00	0,00
6,85	5,87	1,31	0,16	0,00
15,00	5,00	5,00	0,00	0,00
5,10	1,75	1,44	0,00	0,16
3,08	4,97	4,37	1,69	0,50
2,57	2,78	1,60	0,32	0,00
5,91	1,75	1,53	0,00	0,00
7,71	3,25	2,23	0,61	0,20
5,45	5,33	2,84	0,23	0,11
1,65	3,46	2,11	0,45	0,00
4,23	3,99	2,18	0,36	0,00
3,94	2,36	3,94	0,00	0,00
4,89	1,29	0,51	0,00	0,00
7,14	5,36	3,37	0,30	0,20
5,67	7,44	6,18	1,01	0,13
4,72	3,42	3,42	0,65	0,49
0,00	0,00	0,00	0,00	0,00
6,57	6,19	2,58	0,26	0,00
9,29	6,84	6,71	0,52	0,26
6,70	10,37	5,74	0,80	0,16
3,47	4,75	3,47	0,55	0,00
2,97	2,81	2,64	0,99	0,00
6,94	7,81	5,90	0,35	0,35
5,25	7,00	6,48	1,34	0,82
3,64	4,02	3,01	0,50	0,13

4,70	6,47	5,96	1,27	0,38
4,92	2,36	2,17	0,20	0,00
4,92	4,92	1,89	0,38	0,00
7,50	10,96	4,04	0,19	0,00
4,14	5,52	1,72	0,69	0,00
4,65	4,65	0,93	0,00	0,00
6,77	6,63	2,06	0,59	0,29
2,15	0,89	0,30	0,00	0,00
2,04	0,29	0,29	0,29	0,00
6,77	2,86	1,56	0,00	0,00
11,11	0,00	0,00	0,00	0,00
6,13	1,23	0,61	0,00	0,00
5,27	5,72	2,81	0,67	0,11
1,46	1,46	0,73	0,00	0,00
1,10	1,65	0,55	0,00	0,00
4,46	2,68	1,56	0,11	0,00
5,09	5,53	3,35	0,15	0,00
4,14	3,55	1,48	0,00	0,00
4,63	3,70	0,00	0,00	0,00
6,16	2,84	2,37	0,47	0,00
0,88	1,54	0,88	0,22	0,00
1,03	1,03	0,00	0,00	0,00
4,23	6,35	6,35	0,00	0,00
3,53	0,00	0,00	0,00	0,00
2,52	0,63	0,16	0,00	0,00
6,70	5,08	3,12	0,46	0,23
3,39	2,55	0,42	0,00	0,00
5,30	5,30	1,40	0,31	0,16
4,72	2,46	0,21	0,21	0,00
1,72	1,35	0,25	0,00	0,00
2,65	1,83	1,63	0,20	0,20
2,28	1,31	0,16	0,00	0,00
2,89	2,89	0,00	0,00	0,00
1,79	1,79	1,79	0,00	0,00
1,65	2,48	2,48	0,00	0,00
5,30	5,30	3,67	0,20	0,20
2,22	1,67	0,28	0,00	0,00
5,93	4,45	1,60	0,68	0,34
1,98	1,58	0,40	0,00	0,00
0,87	1,46	0,00	0,00	0,29
2,08	2,50	0,00	0,00	0,00
3,60	4,40	0,40	0,40	0,00
1,79	0,30	0,00	0,00	0,00
2,70	0,74	0,25	0,00	0,00
4,26	2,13	0,91	0,00	0,30
3,23	1,61	0,00	0,00	0,00
5,75	3,45	0,00	0,00	0,00
1,02	0,85	0,51	0,00	0,17
2,22	0,89	0,00	0,00	0,00
5,63	6,34	0,70	0,00	0,00

4,27	1,18	0,24	0,00	0,00
1,83	0,91	0,61	0,00	0,00
1,64	0,27	0,00	0,00	0,00
2,04	0,29	0,29	0,00	0,00
5,33	1,33	0,00	0,00	0,00
2,95	3,77	1,15	0,00	0,00
5,41	2,70	0,00	0,00	0,00
3,43	0,49	0,00	0,00	0,00
3,81	2,12	0,42	0,00	0,00
2,56	1,28	1,12	0,16	0,00
1,92	1,92	0,00	0,00	0,00
3,77	1,89	0,63	0,00	0,00
3,47	3,47	2,97	0,00	0,00
5,62	4,49	2,81	1,12	0,00
1,18	0,39	0,20	0,00	0,00
1,94	0,43	0,00	0,00	0,00
2,61	0,75	0,00	0,00	0,00
2,00	0,33	0,00	0,00	0,00
2,95	0,00	0,00	0,00	0,00
6,67	4,98	1,59	0,00	0,00
4,40	3,81	1,76	0,29	0,29
7,89	7,02	3,51	0,00	0,00
2,11	3,16	2,11	1,05	0,00
7,67	4,97	1,85	0,43	0,00
11,27	8,42	2,19	0,11	0,22
3,91	2,81	0,16	0,00	0,00
6,02	3,84	1,54	0,00	0,00
5,23	5,02	2,13	0,21	0,00
3,70	2,81	0,38	0,13	0,00
2,46	2,46	1,31	0,00	0,16
2,94	2,67	0,53	0,00	0,00
4,15	3,56	3,75	0,20	0,00
3,89	4,10	2,66	0,61	0,00
3,95	1,97	1,97	0,00	0,00
2,88	2,40	1,92	0,48	0,00
5,24	6,03	5,50	1,44	1,05
8,65	8,11	4,54	0,43	0,32
3,51	3,34	1,34	0,17	0,00
1,89	0,95	0,19	0,00	0,00
4,26	2,20	0,90	0,00	0,00
4,48	2,46	1,01	0,00	0,00
3,94	2,69	1,43	0,18	0,00
3,24	2,59	1,40	0,11	0,00
2,96	1,06	0,42	0,11	0,00
3,48	1,10	0,73	0,18	0,00
5,79	4,44	1,88	0,00	0,00
3,83	4,37	3,69	1,09	0,14
2,82	2,96	0,70	0,00	0,00
1,85	0,93	0,93	0,31	0,00
5,20	5,60	9,00	1,80	0,60

7,01	5,81	3,11	0,40	0,80
3,53	2,60	0,19	0,00	0,00
2,49	1,49	0,50	0,00	0,00
6,08	2,43	1,35	0,14	0,00
4,42	3,35	1,08	0,00	0,00
0,86	1,14	0,00	0,00	0,00
3,87	3,87	1,29	0,00	0,00
3,36	1,68	0,00	0,00	0,00
1,01	0,00	0,00	0,00	0,00
8,06	1,61	3,23	0,00	0,00
4,00	2,00	1,00	1,00	0,00
3,03	3,03	0,00	0,00	0,00
1,79	1,79	1,79	0,00	0,00
8,96	1,49	1,49	0,00	0,00
1,35	1,35	0,00	0,00	0,00
2,58	2,58	1,94	0,00	0,00
1,95	2,60	0,65	0,00	0,00
8,57	7,14	2,86	0,00	0,00
0,00	0,00	0,00	0,00	0,00
6,55	2,18	1,75	0,44	0,00
0,80	0,80	0,00	0,00	0,00
2,96	2,22	1,48	0,00	0,00
3,41	3,41	1,14	0,00	0,00
8,11	5,41	0,00	0,00	0,00
2,38	0,00	1,19	0,00	0,00
3,90	2,48	1,06	0,00	0,00
7,69	3,17	0,45	0,45	0,00
6,45	0,00	0,00	0,00	0,00
2,33	2,33	0,00	0,00	0,00
9,41	3,53	1,18	0,00	0,00
4,75	1,68	0,28	0,00	0,00
3,91	1,56	0,78	0,00	0,00
4,67	1,87	0,93	0,00	0,00
0,00	2,78	5,56	0,00	0,00
1,49	2,23	1,49	0,00	0,00
2,38	2,38	0,00	0,00	0,00
2,22	1,11	1,11	0,00	0,00

RSRM20	RSRSREN
2,24	7,84
0,68	2,93
3,32	2,27
5,60	0,84
1,91	2,35
0,00	1,23
0,00	0,20
11,44	1,46
7,24	1,42
7,11	1,82
0,73	1,17
1,87	7,64
2,67	0,82
5,38	0,69
4,97	2,08
7,04	4,15
5,26	8,92
5,80	3,45
8,99	1,84
7,88	2,31
7,78	7,44
2,34	4,21
3,99	6,98
9,71	0,92
6,25	1,21
15,35	1,42
4,22	23,09
0,79	0,98
0,93	1,40
0,93	2,68
0,00	0,00
0,90	0,90
0,55	6,50
1,61	1,13
0,27	1,60
0,00	1,46
2,68	4,59
0,77	2,41
0,22	1,97
0,17	1,51
0,00	1,94
0,51	2,87
0,26	1,02
0,00	3,82
0,00	2,46
0,28	2,51
0,18	1,94
2,75	1,14
1,38	4,89

1,54	3,07
3,17	1,24
3,47	1,32
2,05	1,03
1,85	0,92
0,24	1,22
0,40	0,79
0,55	2,73
0,37	4,98
0,79	6,11
0,00	3,60
0,17	3,81
1,34	1,94
2,75	0,98
1,29	0,89
0,60	1,20
0,46	7,62
0,25	1,76
0,54	2,18
0,53	1,27
7,63	0,98
10,56	5,11
8,51	2,07
3,74	3,12
7,12	1,00
9,21	2,19
11,02	1,76
3,82	2,91
6,57	5,03
5,24	3,81
5,20	1,92
11,27	1,46
12,43	0,35
7,09	4,77
7,05	6,41
9,11	5,56
8,02	4,01
8,63	0,62
9,23	1,98
2,46	2,87
1,80	2,41
1,83	2,85
2,23	4,01
5,60	2,19
1,08	5,38
4,38	1,10
5,74	1,37
3,52	2,88
3,63	1,45
5,43	1,90

11,58	1,32
10,32	1,94
5,98	1,12
3,27	2,80
4,34	0,92
4,64	1,97
3,21	0,51
6,87	0,89
8,05	0,76
2,44	3,79
3,67	8,35
6,72	4,48
3,15	4,45
3,72	5,73
4,91	5,58
10,70	0,71
10,13	1,77
5,60	4,31
5,10	2,55
4,23	5,16
0,00	0,00
7,80	1,38
3,60	5,30
1,08	13,51
0,00	0,00
3,63	5,88
2,60	7,16
7,00	3,61
3,56	9,61
2,74	1,90
1,09	3,01
6,45	2,98
3,07	1,92
3,72	1,14
1,41	7,65
5,28	1,41
4,87	2,87
3,92	6,18
7,67	2,78
6,06	2,57
6,67	2,67
5,98	4,27
8,56	0,68
13,35	2,19
11,79	1,75
5,58	4,51
8,64	3,07
4,60	1,67
0,25	4,58
0,00	0,38

0,00	0,78
0,00	3,49
0,00	0,79
0,00	2,40
0,00	2,05
0,00	3,80
11,89	1,08
0,00	0,00
0,53	3,74
0,00	3,23
0,00	2,35
0,00	7,47
0,00	2,76
0,14	1,29
0,00	3,48
0,00	4,59
0,00	1,91
0,00	2,88
0,00	1,26
0,00	2,84
0,17	2,91
0,00	2,65
0,00	2,25
0,00	1,08
0,00	4,59
0,14	2,44
0,00	0,75
0,00	1,72
0,10	1,72
0,00	1,26
0,17	0,52
0,00	1,60
0,37	2,21
0,24	2,91
0,00	1,52
0,00	3,47
0,00	1,98
0,00	2,80
0,00	0,85
0,15	1,98
0,00	0,49
0,00	2,76
0,31	2,59
0,00	0,68
7,53	0,60
10,54	0,37
8,06	0,95
9,49	3,37
2,01	1,67
6,84	1,14

5,59	4,77
7,72	6,21
8,31	0,26
2,99	18,27
3,26	3,13
5,09	3,21
9,81	1,03
4,48	3,98
10,14	2,25
8,78	1,46
2,89	2,89
7,79	0,89
3,77	2,74
2,50	1,00
6,84	1,56
16,54	1,05
11,41	1,28
6,38	0,00
5,78	13,68
2,04	6,12
7,89	1,07
0,35	6,69
1,12	4,72
1,28	3,85
0,63	2,81
0,41	1,64
0,23	1,36
2,94	4,60
1,56	4,43
0,62	4,93
1,32	3,82
2,46	6,41
2,61	3,65
0,89	4,66
0,86	1,29
1,08	2,88
0,00	0,00
2,66	3,55
1,96	4,82
1,53	4,08
2,25	2,25
8,47	1,62
13,22	2,89
5,74	1,43
6,43	5,57
6,39	0,77
5,70	1,57
2,78	2,60
3,34	3,98
4,99	2,93

2,73	5,31
9,05	1,01
6,82	2,17
11,92	2,54
11,39	1,73
12,54	2,13
12,23	1,34
1,94	9,48
2,71	3,61
4,35	2,40
4,74	3,65
2,36	6,72
2,17	1,50
0,63	3,45
2,49	3,62
3,44	3,27
1,95	0,97
0,75	4,87
0,49	3,91
1,21	1,42
1,07	2,45
9,56	4,86
7,22	0,54
5,68	2,57
5,90	3,09
7,14	3,38
6,91	1,93
4,82	1,65
6,53	1,55
7,04	1,88
3,48	1,42
3,99	0,33
3,18	1,94
2,45	3,06
5,30	3,10
5,95	0,53
4,66	2,06
8,58	2,40
13,61	2,72
11,19	2,74
15,48	3,07
12,83	0,57
9,87	3,59
9,37	2,64
3,99	5,24
9,65	3,80
6,35	7,02
0,69	1,04
1,09	0,00
0,80	0,80

0,63	2,19
1,19	1,98
0,77	2,81
9,23	1,07
8,80	1,66
8,16	1,09
4,67	2,41
8,94	0,88
9,11	0,00
4,70	0,98
0,00	0,00
7,26	0,57
4,97	3,41
2,38	2,70
6,05	1,28
3,54	0,39
4,23	1,21
6,62	2,47
4,61	1,32
5,91	1,76
9,01	1,16
11,53	0,90
14,30	1,51
15,32	1,45
12,96	1,90
10,12	1,93
10,88	0,34
3,93	1,81
1,63	2,99
1,47	3,19
0,39	3,52
0,39	2,33
2,17	3,25
0,44	5,73
0,44	3,72
1,00	2,20
1,44	5,94
8,29	0,79
0,00	1,82
0,20	1,82
0,09	2,94
0,00	1,57
0,57	2,57
0,19	5,27
0,00	2,68
0,00	1,35
0,28	2,20
0,53	1,95
0,83	2,01
0,07	3,52

0,00	2,14
0,78	2,53
0,24	4,98
0,62	4,37
0,20	1,52
0,00	2,19
0,09	2,72
5,67	0,85
4,24	0,92
3,43	1,14
4,69	2,47
5,87	1,13
5,66	0,69
6,58	2,08
2,58	1,12
8,51	1,49
4,98	1,02
6,02	0,88
3,69	2,14
3,44	2,58
8,51	1,66
8,48	1,31
6,57	2,42
5,31	3,44
7,28	3,92
1,59	5,31
0,33	3,29
0,51	0,25
3,36	9,40
0,89	2,68
0,53	4,95
0,00	0,00
6,21	1,53
6,66	2,13
4,17	0,60
7,18	0,84
14,05	0,33
4,84	4,50
10,50	3,23
4,46	5,95
8,45	2,17
3,42	2,70
7,56	1,37
6,94	2,65
1,72	5,58
5,63	2,82
2,86	4,57
3,70	4,07
4,27	3,66
6,80	1,65

10,99	1,10
3,81	0,73
8,39	2,40
3,14	1,83
0,00	0,00
2,55	4,47
0,00	0,00
16,77	1,41
17,53	0,72
17,84	0,78
14,93	2,13
15,09	0,75
4,95	3,10
5,75	3,94
8,84	2,25
7,08	4,32
0,00	0,00
6,25	2,60
4,22	2,47
11,58	1,12
8,08	1,15
5,89	2,86
9,32	0,82
3,04	5,80
5,64	1,20
3,76	0,91
8,48	2,64
0,00	1,26
6,42	3,04
7,54	1,96
8,77	0,29
12,31	1,22
6,81	4,89
1,29	1,29
9,75	1,53
6,81	7,63
6,47	3,46
2,94	1,36
6,36	0,45
6,96	3,04
8,48	0,87
8,16	2,78
2,82	4,79
7,51	0,86
9,14	1,83
4,92	1,64
6,48	3,07
1,22	4,27
8,15	2,25
10,49	4,10

8,82	3,71
1,25	3,74
5,76	3,43
14,25	2,87
19,19	0,53
21,01	2,25
11,50	7,08
6,83	2,44
5,90	0,59
5,06	1,95
10,87	1,00
15,72	2,47
6,25	1,25
1,88	3,46
3,94	3,24
15,67	1,55
17,46	1,72
4,07	1,11
6,73	1,25
4,44	6,39
7,69	4,81
8,66	1,19
4,56	1,22
4,56	4,72
4,85	8,25
5,12	1,28
6,98	0,58
8,89	1,44
11,11	2,78
5,21	3,96
0,00	0,00
3,70	0,00
14,69	3,50
6,32	2,11
12,10	0,79
9,62	2,35
0,00	0,00
2,55	2,55
3,65	5,98
3,13	1,88
3,77	4,72
9,23	1,96
3,13	3,75
9,62	1,92
5,88	0,00
5,58	1,27
5,41	6,08
11,55	0,99
0,00	0,00
9,93	1,42

0,00	2,06
1,32	2,65
8,11	2,70
0,00	0,00
0,00	3,72
3,22	1,61
0,00	0,00
0,00	6,03
2,29	2,10
0,00	3,19
0,44	9,05
12,31	1,82
0,00	0,00
0,00	2,06
0,00	4,82
13,84	2,08
7,43	4,02
3,91	5,08
0,00	5,66
0,00	1,43
0,25	2,22
0,09	3,82
3,69	2,21
3,01	4,82
0,00	0,00
7,46	1,00
6,77	4,69
2,22	3,33
0,48	1,20
0,00	2,88
0,00	6,94
0,00	3,27
1,67	3,33
0,00	0,00
4,62	0,37
8,16	2,99
5,98	0,92
3,38	1,69
2,90	1,34
10,31	1,35
8,41	0,00
0,76	2,16
1,41	0,23
0,00	2,23
0,92	2,07
1,55	0,89
0,64	1,16
0,99	2,63
0,16	5,74
0,22	7,11

0,41	9,02
2,25	1,12
0,00	0,00
0,16	1,26
0,57	4,88
0,58	3,50
0,39	2,51
0,31	2,70
0,21	1,29
0,37	4,70
0,33	1,63
0,24	2,98
0,15	5,86
0,00	14,89
0,39	1,18
0,48	1,43
2,19	1,91
1,23	1,69
1,67	2,92
0,63	1,58
1,60	0,96
0,57	1,52
0,29	1,87
0,52	1,68
0,00	6,35
0,16	1,29
0,19	3,59
0,22	1,55
0,62	1,55
0,59	1,99
0,58	2,46
0,00	1,07
0,16	3,02
0,00	1,91
0,67	1,88
0,00	0,81
0,00	1,70
0,00	6,14
1,25	2,80
0,19	1,36
0,93	6,15
0,24	3,53
0,00	2,41
0,00	0,58
1,10	0,49
0,88	2,19
1,72	4,91
2,06	2,58
1,07	1,47
0,61	1,83

0,92	1,83
1,63	1,16
0,78	0,90
0,43	1,44
1,10	1,32
1,31	5,14
0,97	1,17
2,39	0,80
0,11	1,39
0,92	2,30
0,48	2,41
0,88	2,39
0,49	2,47
0,42	2,82
0,34	1,80
0,54	2,99
0,00	1,99
1,51	4,74
0,17	1,20
0,00	5,55
0,19	0,97
0,24	1,70
0,00	4,48
0,09	2,45
0,00	1,94
0,00	7,71
0,40	0,94
0,40	1,62
0,59	1,03
0,40	0,60
0,00	1,79
0,52	0,52
1,51	1,92
1,03	4,96
1,36	1,06
1,48	0,27
1,36	1,85
2,55	0,97
1,32	1,70
1,27	2,67
0,00	3,15
1,32	3,64
0,59	1,07
1,39	0,69
1,48	0,63
0,72	3,23
0,00	1,07
0,51	2,72
0,00	2,16
0,00	5,85

0,16	2,49
0,00	2,90
0,12	3,35
0,00	2,71
0,25	2,15
0,00	1,53
0,41	1,08
0,46	1,03
0,35	1,73
0,67	8,00
2,54	5,72
1,22	2,52
0,13	1,71
0,98	2,39
0,88	1,76
1,57	1,45
0,93	2,39
0,81	1,61
1,08	6,47
1,18	0,84
0,54	2,26
1,23	3,48
0,56	0,94
0,83	1,49
0,27	1,28
0,12	2,55
0,54	1,08
0,32	2,47
0,20	1,08
0,24	2,07
0,11	1,42
0,00	2,29
0,00	2,76
0,25	2,79
0,00	0,50
0,18	1,43
0,00	3,04
0,13	1,53
0,12	1,66
0,24	3,36
0,00	2,11
0,00	2,47
0,00	1,55
0,00	0,87
0,00	2,91
0,00	2,37
0,00	1,05
0,00	5,96
0,55	0,55
0,69	1,74

0,22	2,44
0,65	5,34
0,45	4,08
0,00	4,09
0,68	1,14
0,98	1,56
2,66	1,83
0,10	1,10
0,82	1,86
0,00	2,57
1,75	2,02
3,32	3,32
0,31	2,45
1,63	1,63
1,39	1,39
0,78	1,95
0,77	0,00
1,70	2,12
1,01	5,32
1,01	3,85
1,77	2,23
1,48	1,82
3,50	5,57
0,82	1,77
2,14	2,50
0,00	1,83
0,12	1,73
0,44	1,32
0,00	1,99
0,19	0,95
0,00	0,00
0,00	3,98
0,00	3,15
0,10	1,24
0,37	0,92
0,24	5,76
0,96	2,29
0,48	1,94
0,86	1,90
0,63	2,97
1,45	1,71
1,31	1,55
0,00	2,42
1,35	1,35
1,09	1,09
0,94	6,13
0,19	5,08
0,18	3,43
0,92	1,60
0,93	0,00

0,40	1,29
0,00	2,02
0,00	1,15
0,24	1,53
0,00	1,46
0,98	0,98
0,36	2,33
1,12	4,16
1,30	3,90
0,53	9,25
1,16	0,87
0,00	3,31
2,38	1,19
0,93	4,64
2,64	1,41
2,71	3,88
1,70	1,46
0,91	1,14
0,40	1,38
1,58	3,02
2,11	1,35
1,79	2,44
2,03	1,41
1,40	0,78
0,25	2,21
0,00	3,02
0,00	6,92
0,00	2,55
0,90	1,80
0,00	2,20
0,00	3,25
0,00	7,26
0,00	3,76
0,00	2,74
0,00	7,23
0,00	3,98
0,00	6,85
0,00	4,37
0,00	4,33
0,00	3,42
0,00	2,37
0,00	11,95
0,00	2,20
0,00	3,53
0,00	1,54
0,53	4,79
0,00	0,00
0,00	2,75
0,82	3,89
0,84	6,07

0,00	0,00
1,41	0,94
0,42	1,04
0,74	0,99
1,39	6,50
0,41	0,83
1,20	1,68
1,41	8,65
0,18	11,11
0,00	3,20
1,88	2,35
0,34	1,20
0,79	0,26
1,84	1,43
1,45	0,83
0,64	11,24
0,24	4,36
0,65	2,94
0,74	1,47
0,83	0,00
1,02	1,36
0,28	1,84
0,00	12,40
0,00	0,64
0,31	2,83
0,39	1,18
1,12	0,00
0,00	4,57
0,00	0,00
0,00	13,11
0,00	1,68
0,70	2,80
1,34	1,57
0,00	0,00
1,19	0,95
0,00	0,00
0,00	4,65
0,00	4,66
0,00	4,21
0,00	1,58
0,27	1,87
0,00	0,00
0,00	4,10
0,00	5,79
0,00	0,51
0,20	8,01
0,00	1,33
0,00	2,50
0,00	6,37
0,57	1,14

0,00	4,07
0,00	3,17
0,00	42,86
0,00	4,17
0,00	1,79
0,00	1,04
0,00	2,51
0,00	1,91
0,00	2,43
0,00	7,57
0,00	0,00
0,00	1,64
0,00	2,70
0,00	2,10
0,00	2,50
1,74	1,47
0,00	1,63
0,00	0,00
0,00	1,40
0,17	2,34
0,00	3,77
0,00	3,31
0,00	6,72
0,00	0,51
0,00	3,62
0,00	6,98
0,92	2,68
0,35	6,71
0,39	4,33
0,00	0,78
0,00	9,34
0,00	0,00
0,00	8,17
0,00	0,83
0,00	0,84
0,00	2,06
0,00	4,44
0,00	5,86
0,00	5,06
0,57	4,54
0,95	2,47
0,00	4,15
0,00	0,54
0,00	2,66
0,58	1,74
0,00	1,83
0,00	1,10
0,81	2,02
0,00	15,87
0,54	5,98

2,53	2,67
0,83	0,97
1,48	3,55
0,00	1,91
4,84	0,00
0,00	0,00
0,00	5,25
0,00	0,62
0,36	0,91
0,00	2,85
0,00	1,78
0,00	4,95
0,76	1,52
0,00	3,94
0,00	2,60
0,00	3,49
0,00	9,23
0,00	0,00
0,00	2,81
0,00	2,29
0,00	0,88
0,30	5,47
0,00	1,26
0,20	1,98
0,00	5,41
0,00	3,87
0,13	1,89
0,50	1,66
0,15	8,38
0,00	4,78
0,14	13,15
0,49	1,47
0,94	0,94
0,98	0,98
1,85	0,99
2,01	0,72
0,00	4,98
1,36	5,42
2,07	2,39
0,00	0,00
1,91	3,18
0,17	1,03
0,18	1,07
0,11	0,88
0,16	1,09
0,00	1,76
0,20	2,32
0,22	2,38
0,00	1,65
0,16	3,01

0,00	1,64
0,00	2,27
0,00	0,00
1,56	4,17
3,30	0,63
3,93	2,62
1,95	5,32
2,46	2,95
0,00	2,22
0,00	6,70
0,30	1,81
0,36	0,53
0,00	10,49
0,00	2,36
0,00	3,13
0,00	6,54
0,16	2,34
0,13	1,53
0,00	4,76
0,23	4,31
0,11	5,70
0,00	2,83
0,00	1,77
0,12	3,36
0,00	2,12
0,00	1,77
0,00	1,13
0,00	2,61
0,00	9,95
0,00	2,22
0,00	2,86
0,00	0,87
0,19	2,02
0,00	2,35
0,13	4,62
0,00	1,93
0,00	1,48
0,00	1,82
0,00	0,84
0,00	7,71
0,35	3,48
0,00	9,29
0,00	1,33
0,10	6,31
0,00	2,66
0,00	4,53
0,00	2,94
2,84	0,95
0,00	8,67
0,00	0,00

0,00	1,86
0,00	7,18
0,00	1,65
0,00	1,39
0,00	1,52
0,00	1,81
0,00	0,81
0,00	2,30
0,64	1,27
0,43	2,13
1,22	0,91
0,66	1,66
1,45	1,33
5,24	0,97
0,00	4,72
0,33	1,17
0,76	2,65
0,79	0,91
0,66	2,86
0,12	4,47
1,58	0,90
0,15	1,21
0,00	2,85
0,98	1,18
0,64	0,64
0,11	2,03
0,11	3,37
0,22	1,19
0,62	2,37
0,10	5,15
3,68	1,40
2,74	3,08
0,00	2,04
0,00	0,88
0,71	1,42
0,53	4,01
0,29	3,12
0,27	2,01
0,00	7,45
0,18	1,20
0,62	1,65
0,22	1,33
4,38	0,97
1,85	0,58
2,48	1,73
1,89	1,32
4,92	0,59
3,55	0,89
1,53	1,79
0,33	1,65

0,45	1,90
0,61	1,62
0,24	1,45
0,11	6,25
0,37	1,30
0,37	1,24
0,19	2,62
0,10	1,62
0,00	2,18
0,17	1,83
0,24	3,72
0,23	1,63
0,38	2,41
2,75	3,50
7,15	0,84
7,56	1,13
0,27	2,19
1,99	1,71
1,90	3,00
2,06	1,49
3,48	1,16
2,47	2,18
3,79	1,00
1,75	1,34
0,97	1,21
1,52	2,60
0,23	1,59
0,00	1,30
0,00	6,23
0,34	2,05
0,36	1,32
0,00	1,86
0,10	1,26
0,00	2,75
3,54	2,31
1,27	2,97
0,19	3,32
0,21	8,13
0,34	6,92
2,82	2,91
1,15	1,46
2,67	1,09
4,05	1,58
5,71	1,19
1,32	1,55
0,58	1,40
0,64	11,35
0,52	2,08
0,27	1,33
0,00	1,29

0,23	2,16
0,00	1,68
0,00	3,63
0,00	4,53
0,39	1,17
0,41	1,63
0,11	2,01
0,40	3,19
0,00	1,21
0,00	1,39
0,28	0,71
0,00	3,43
0,00	3,01
0,46	1,15
0,39	1,45
0,00	5,10
1,03	1,48
0,32	5,45
0,11	5,53
0,00	1,39
0,36	2,85
0,10	0,93
0,56	1,53
2,68	2,91
0,30	0,90
0,31	1,09
0,16	1,31
0,00	1,22
0,00	2,25
0,00	4,04
0,56	5,36
0,10	2,11
0,91	4,11
0,70	1,57
0,39	1,30
0,23	0,91
0,35	1,29
0,00	0,90
0,00	0,00
1,19	2,88
0,00	0,00
0,00	7,96
0,00	0,00
0,00	2,02
0,00	1,60
0,11	0,96
0,89	0,22
0,25	8,99
0,28	1,60
1,55	0,78

0,00	0,34
0,00	1,63
0,00	4,46
0,00	2,58
0,58	1,31
1,45	1,85
0,62	1,66
0,12	0,62
0,00	1,72
0,00	3,44
0,12	2,56
0,14	3,95
1,40	3,51
2,83	0,81
0,52	0,52
2,72	1,09
0,58	1,29
0,43	7,26
0,00	1,70
0,42	13,56
1,32	2,45
0,45	1,58
1,48	1,48
0,56	4,49
2,24	1,22
3,56	3,75
0,00	4,14
0,00	0,21
0,00	13,92
0,46	2,74
0,00	2,15
0,26	3,13
0,18	1,10
0,00	0,00
0,94	1,88
0,55	3,58
2,11	3,52
1,10	2,56
4,39	1,50
0,00	6,42
0,33	2,95
0,83	1,65
1,85	1,85
0,69	2,97
1,07	2,50
0,00	2,92
1,00	3,16
1,57	1,57
4,21	2,81
1,49	0,50

3,47	2,48
0,56	4,83
0,00	2,25
0,67	4,00
0,00	2,11
0,70	0,70
1,23	3,07
0,00	2,81
0,00	0,00
2,22	2,22
0,00	0,00
0,00	0,00
0,23	2,54
1,28	1,46
0,00	0,00
0,31	1,42
1,59	2,38
0,00	2,81
1,43	2,15
0,86	1,29
0,00	1,16
0,00	1,35
1,41	0,88
0,00	0,00
0,00	2,50
0,63	1,90
0,00	1,85
0,00	2,47
0,00	5,44
1,37	1,37
0,00	1,21
0,31	1,56
0,65	1,94
0,74	2,85
0,59	1,04
0,40	2,11
1,18	1,38
0,96	0,83
0,58	2,78
0,00	0,00
0,32	3,74
1,73	1,59
2,85	1,50
0,39	3,72
0,30	1,50
0,13	1,25
0,64	0,64
0,00	1,12
0,00	5,82
0,17	3,48

0,00	0,00
0,86	1,72
0,21	1,67
0,36	1,44
0,12	2,03
0,10	5,42
0,14	2,04
0,32	1,72
0,17	0,85
0,12	1,00
0,17	0,34
0,00	5,50
0,09	0,85
0,11	2,71
0,00	2,86
0,19	2,76
0,36	3,56
0,00	1,43
0,13	1,74
0,00	5,23
0,00	5,79
0,71	1,78
1,27	1,90
0,24	6,14
1,57	0,86
0,87	2,03
1,12	1,87
0,79	0,39
0,67	0,78
1,20	2,00
0,81	2,59
0,81	1,62
1,06	1,29
0,85	1,58
5,47	2,11
0,18	1,07
0,12	1,58
0,60	3,63
0,35	1,64
0,82	1,02
0,00	1,07
0,19	2,27
0,40	6,52
0,58	1,96
0,54	2,72
1,57	1,57
2,18	2,18
2,34	3,38
2,77	4,47
1,99	2,49

1,47	5,88
1,14	4,17
0,90	2,33
0,20	1,41
0,34	1,71
0,50	4,83
0,00	3,44
0,81	2,44
0,26	1,28
0,73	3,75
0,33	2,10
0,00	2,08
0,14	0,70
0,09	1,96
1,03	3,79
0,10	1,24
0,10	1,05
0,00	2,57
0,00	2,21
0,00	1,38
0,37	2,50
0,00	2,94
0,00	7,55
0,00	3,29
0,12	0,99
0,08	1,29
0,00	1,12
0,00	2,23
0,00	1,28
0,17	1,53
0,00	1,23
0,11	1,72
0,00	2,28
0,13	1,27
0,00	2,09
0,00	2,14
0,13	1,57
0,12	4,78
0,36	3,44
0,00	1,20
0,65	1,08
0,74	1,33
0,49	3,33
0,14	2,86
1,11	6,12
0,22	5,17
0,83	1,77
0,48	2,38
1,13	4,14
2,89	1,65

1,44	0,72
0,65	1,63
0,71	2,86
1,13	1,64
0,00	0,98
0,00	8,33
0,00	0,00
0,00	1,83
0,00	3,13
0,00	0,00
0,00	0,00
0,00	2,07
0,00	0,00
0,00	8,29
0,00	9,14
0,00	4,88
0,00	0,00
0,00	0,00
0,00	1,11
0,00	1,54
0,00	2,24
0,96	2,40
0,99	0,79
0,00	0,98
0,00	0,77
0,00	5,23
0,48	4,81
2,20	6,59
0,00	1,35
0,00	0,81
1,01	2,02
4,00	4,00
0,00	3,28
0,00	1,81
0,11	8,96
0,13	1,40
0,00	2,76
0,00	2,53
0,19	2,60
0,00	7,48
0,00	5,02
0,63	4,43
0,00	0,00
0,00	4,12
0,00	2,35
0,00	2,98
0,47	1,04
1,18	0,74
0,73	0,83
0,00	2,42

0,00	5,28
0,00	0,00
0,00	3,12
0,00	1,55
0,11	1,90
0,00	1,56
0,12	4,44
0,00	3,91
0,11	3,10
0,38	0,51
0,22	2,18
0,00	0,93
1,06	0,53
0,12	2,11
0,32	1,70
0,50	0,99
0,27	4,61
1,33	3,33
1,03	1,54
0,76	3,48
0,36	2,54
0,39	0,78
0,56	0,98
0,34	1,36
1,93	5,78
1,76	4,34
1,24	8,19
0,00	0,00
0,00	12,10
0,12	3,15
0,45	2,23
0,69	2,93
0,00	0,00
0,45	1,36
0,25	5,68
0,16	6,39
0,99	10,89
0,00	1,50
0,00	1,17
0,00	0,40
0,00	0,40
0,00	1,49
0,46	2,70
0,00	1,74
0,30	4,65
0,21	2,05
0,00	1,17
0,00	5,18
0,13	0,89
0,20	1,18

0,22	1,88
0,10	2,26
0,16	3,15
0,00	2,25
0,19	0,85
0,30	1,31
0,39	4,44
0,12	1,07
0,43	1,62
0,09	3,55
0,00	1,75
0,00	1,95
0,00	1,68
0,00	1,63
0,00	2,26
0,00	1,31
0,00	1,72
0,00	0,82
0,31	1,38
0,00	3,20
0,00	2,07
0,00	2,54
0,00	1,96
0,00	1,66
0,00	4,57
0,00	1,56
0,00	3,26
0,00	1,97
0,00	1,75
0,00	2,21
0,00	1,28
0,07	1,45
0,00	0,72
0,08	5,40
0,00	1,58
0,00	1,58
0,00	2,12
0,00	1,43
0,00	1,87
0,15	1,53
0,00	2,22
0,13	0,51
0,00	4,00
0,22	1,34
0,21	1,48
0,21	0,57
0,08	0,90
0,00	1,37
0,00	1,08
0,13	1,32

0,00	1,52
0,00	1,51
0,00	1,78
0,15	0,88
0,00	1,55
0,13	2,45
0,00	1,03
0,09	5,54
0,00	1,05
0,11	2,03
0,11	4,53
0,00	1,78
0,32	1,61
0,00	1,54
0,44	0,33
0,38	0,57
0,22	0,90
0,62	0,62
0,76	0,76
0,00	0,83
1,25	0,63
0,65	1,04
0,60	1,51
0,79	1,67
0,68	1,59
0,31	2,04
0,40	1,42
0,00	3,37
0,00	1,33
0,00	1,24
0,00	5,26
0,00	0,00
0,87	9,57
0,00	1,40
0,00	3,44
0,00	4,17
0,00	3,87
0,31	4,00
0,11	2,22
0,00	1,86
0,12	2,43
0,00	1,06
0,00	4,49
0,00	2,37
0,00	1,67
0,00	5,81
0,00	1,44
0,00	2,62
0,00	2,14
0,00	2,46

0,12	0,85
0,16	2,56
0,00	0,00
1,35	0,51
0,44	5,27
0,27	1,18
0,15	4,08
0,22	1,10
0,00	0,57
0,00	3,97
0,12	2,60
0,00	9,05
0,00	9,28
0,00	2,04
0,00	3,32
0,00	4,17
0,25	2,21
0,18	1,29
0,00	1,09
0,12	2,49
0,32	3,22
0,27	3,27
0,25	1,52
0,00	2,42
0,00	2,29
0,00	2,85
0,00	2,02
0,49	1,85
0,19	2,79
0,34	4,34
0,00	1,59
0,15	4,08
0,00	3,00
0,38	2,15
0,00	1,09
0,17	1,34
0,00	0,00
0,13	2,83
0,15	1,34
0,00	3,43
0,64	2,56
0,00	4,48
0,00	5,13
0,12	4,05
0,00	5,10
0,00	12,23
0,00	0,00
0,09	1,93
0,00	1,91
0,00	3,92

0,00	0,85
0,00	1,72
0,13	2,55
0,20	1,84
0,00	3,52
0,13	6,40
0,00	11,75
0,00	4,79
0,00	5,51
0,00	3,23
0,00	1,83
0,44	0,67
0,66	5,14
0,22	1,76
0,42	1,81
0,78	1,29
0,75	1,25
0,20	1,97
0,00	1,56
0,16	1,24
1,29	0,86
0,19	2,27
0,54	2,33
0,00	2,25
0,27	5,87
0,22	1,56
0,65	6,02
0,92	1,29
0,00	3,28
0,29	6,63
0,76	5,19
0,44	5,01
0,82	1,09
1,15	4,03
0,64	3,05
0,00	1,56
0,84	1,52
1,35	2,99
0,13	1,48
1,36	1,16
0,00	0,00
3,30	2,20
0,00	0,00
2,55	0,51
1,77	2,84
0,00	5,77
0,37	1,31
0,38	1,69
0,34	1,01
0,77	3,08

0,00	2,26
4,14	0,34
0,00	0,00
0,38	4,89
0,00	3,37
0,35	4,53
0,00	0,66
0,00	10,42
0,00	0,00
0,00	2,08
0,00	2,76
0,00	3,86
1,07	1,42
2,63	0,00
0,56	2,23
3,59	1,27
0,00	4,93
0,60	3,83
0,00	1,11
0,00	1,47
0,00	0,00
0,00	2,08
1,27	0,63
0,00	1,64
0,89	1,43
0,59	10,00
0,36	4,32
0,70	0,00
0,00	2,22
0,00	8,97
0,25	5,76
0,14	0,72
0,00	3,16
0,00	0,00
0,00	1,56
0,00	3,19
0,00	0,00
0,00	3,23
0,41	2,03
0,00	2,96
0,00	10,81
0,60	3,60
0,47	1,71
0,28	4,49
0,00	3,53
0,00	1,88
0,00	0,00
0,00	1,64
0,00	1,56
0,00	1,80

0,00	0,00
2,20	2,20
0,00	2,34
1,60	5,60
0,19	4,26
1,04	2,30
2,83	0,94
0,00	7,46
0,00	2,90
0,00	0,00
0,12	2,21
0,18	2,73
0,00	2,98
0,42	0,84
0,21	4,05
0,43	3,88
0,13	6,23
0,00	7,54
0,00	5,45
0,00	0,00
0,00	0,87
0,00	2,12
0,00	0,00
0,53	1,07
0,24	2,64
0,51	0,68
0,00	0,00
0,28	2,27
2,02	1,21
0,00	0,00
0,00	1,01
0,00	3,97
0,00	0,00
0,00	1,57
0,00	1,22
0,00	5,35
0,00	7,41
0,00	0,35
0,00	3,38
0,00	2,72
0,00	2,14
0,00	0,44
0,00	3,90
0,00	1,85
0,00	0,96
0,29	4,72
0,00	0,39
0,00	1,96
0,00	0,93
0,00	3,18

0,00	0,00
0,00	2,29
0,85	1,14
0,00	0,00
0,00	1,87
0,00	3,47
0,62	1,85
0,00	4,70
0,00	2,06
0,30	4,65
0,00	1,04
0,00	9,30
0,00	5,36
0,00	1,39
0,00	7,98
0,00	0,88
0,00	0,00
0,00	2,37
0,00	4,40
0,00	9,30
0,00	6,74
0,00	2,46
0,00	0,00
1,38	1,38
0,00	0,25
0,00	0,60
0,00	2,28
0,00	1,69
0,00	0,50
0,00	0,00
0,00	1,33
0,00	0,84
0,00	0,00
0,76	1,53
0,00	6,90
0,00	4,67
0,00	0,00
0,00	2,45
0,64	0,64
2,10	2,34
2,29	3,06
0,96	0,96
1,17	2,33
0,00	3,57
0,00	1,28
1,04	3,95
0,14	2,33
0,00	3,54
0,00	4,91
1,07	0,43

0,00	0,00
0,51	0,34
0,16	3,89
0,00	8,13
0,11	1,51
0,13	1,69
0,00	2,43
0,13	3,75
0,22	4,75
0,00	0,74
1,28	1,68
0,28	2,69
0,67	3,12
0,14	2,32
0,39	2,71
0,00	2,31
0,91	4,79
0,15	9,11
0,34	3,47
0,49	1,37
0,22	2,18
0,54	1,18
0,25	1,90
0,65	4,66
0,16	8,14
0,15	1,55
0,14	2,97
0,00	1,66
0,00	1,71
0,22	4,20
0,13	2,17
0,38	1,64
0,22	6,68
0,26	7,99
1,02	3,97
0,49	5,19
0,32	1,11
0,00	5,87
0,00	4,40
0,00	1,00
0,00	1,70
0,18	2,14
0,00	0,85
0,32	3,25
0,30	3,92
0,09	2,22
0,32	0,81
0,66	2,92
1,82	1,56
2,29	0,57

0,00	1,62
1,17	1,95
0,73	1,65
0,62	1,65
0,00	1,49
0,66	0,82
0,19	1,69
0,14	7,69
0,14	2,09
0,15	0,74
0,00	1,56
0,00	1,06
0,00	1,74
0,14	4,12
0,00	5,49
0,00	2,22
0,00	3,49
0,16	2,34
0,00	2,25
0,00	8,07
0,00	1,48
0,00	3,67
0,00	1,76
0,48	1,80
0,94	0,63
0,47	2,34
0,39	1,18
1,30	4,68
0,44	8,26
0,50	1,13
0,55	5,55
0,68	1,14
0,00	0,95
0,76	0,38
0,62	5,68
0,95	6,83
0,52	1,20
0,00	2,96
2,75	3,52
2,26	1,67
0,77	2,06
1,95	3,31
7,34	0,16
0,95	3,00
3,07	4,75
5,03	1,22
5,11	1,00
1,88	1,19
4,42	1,64
4,71	1,71

6,62	0,22
0,25	1,18
0,00	1,23
0,00	1,03
0,13	1,28
0,00	3,35
0,14	0,42
0,18	1,28
0,08	5,22
0,00	1,89
0,00	2,82
0,00	9,81
0,10	2,44
0,00	2,02
0,20	1,19
0,00	2,20
0,00	1,70
0,00	0,56
0,00	6,82
0,00	1,75
0,00	0,95
0,00	2,17
0,00	2,22
0,00	2,02
0,00	5,69
0,14	5,91
0,20	2,76
0,19	2,10
0,00	2,04
0,22	3,99
0,23	2,25
0,00	4,17
0,32	5,99
0,00	3,63
0,37	2,03
0,19	1,89
0,00	1,97
0,12	1,35
0,00	3,64
0,20	1,30
0,74	0,74
0,00	1,05
0,40	2,00
0,00	16,43
0,00	0,52
0,67	1,48
0,85	1,18
0,15	1,64
0,00	1,46
1,40	2,53

0,33	5,81
0,00	2,45
1,20	1,52
1,03	1,37
0,17	6,00
0,71	6,53
0,88	1,76
0,38	0,94
0,21	6,61
0,86	0,43
0,00	3,25
0,00	7,10
0,13	1,40
0,00	2,71
0,00	10,14
0,00	1,64
0,00	0,62
0,00	3,75
0,00	2,02
0,00	0,00
3,08	1,68
5,07	1,61
3,26	4,01
4,61	4,84
4,62	0,87
5,53	2,25
4,61	2,17
3,28	1,56
3,37	2,17
0,00	1,50
0,53	0,53
0,00	1,96
0,26	1,03
0,00	2,07
0,00	2,05
0,00	3,15
0,00	4,73
0,00	0,00
0,00	2,57
0,00	2,50
0,00	1,70
0,00	1,95
0,18	2,28
0,19	3,27
0,00	3,77
0,00	2,84
1,11	2,35
0,56	1,40
1,28	0,93
0,77	3,09

0,11	6,54
1,65	1,38
0,69	4,25
0,45	6,76
0,98	6,85
0,27	4,96
1,17	1,35
1,03	5,36
0,00	0,00
0,00	3,37
0,00	1,41
0,07	1,70
0,00	0,94
0,00	2,77
0,00	0,00
0,00	6,40
0,00	1,41
0,00	3,61
0,00	5,04
0,90	2,56
1,28	0,26
0,75	1,94
0,12	4,20
0,79	0,20
0,46	0,91
0,58	0,58
0,77	4,52
0,50	2,50
2,23	1,97
1,07	0,46
2,98	1,17
1,21	0,99
0,35	1,73
0,00	1,35
0,00	0,26
0,00	1,08
0,00	6,78
0,00	6,38
1,80	3,11
0,34	1,72
2,36	2,22
1,67	4,28
3,06	1,97
2,34	1,67
0,71	2,44
1,08	2,56
1,16	4,35
2,36	1,23
1,36	1,81
2,41	2,97

3,32	5,19
1,64	1,09
3,77	0,98
3,43	1,18
4,17	1,21
0,00	0,29
0,00	1,21
0,00	1,36
0,00	2,66
0,00	1,13
0,00	4,59
0,00	4,39
2,36	1,86
0,78	1,26
1,35	1,24
0,36	3,62
1,62	2,51
2,29	4,46
2,02	1,35
2,77	5,70
4,89	1,96
0,21	1,72
1,39	1,86
2,97	0,41
0,49	2,47
2,90	1,52
2,30	3,78
0,74	2,21
2,20	2,08
2,85	0,42
4,25	0,88
3,23	11,47
4,46	1,41
6,19	2,11
2,29	4,59
0,00	2,52
6,70	1,79
7,76	1,66
0,00	11,06
0,00	2,91
1,80	2,06
2,02	1,01
1,37	1,91
1,53	1,70
2,98	0,85
3,09	1,43
2,29	1,43
2,03	0,87
2,84	1,84
2,23	2,60

2,91	2,75
2,90	2,10
3,07	5,42
4,06	5,10
4,21	5,10
9,80	3,37
4,15	1,79
3,93	1,24
4,45	1,89
5,98	3,19
4,25	1,10
7,42	1,50
0,70	0,82
0,00	2,53
0,81	4,07
0,00	10,07
0,00	2,69
0,00	3,09
0,00	3,39
0,77	1,15
0,00	0,93
0,00	1,39
0,00	3,41
0,00	1,61
0,00	2,51
0,00	0,00
0,00	0,00
0,49	3,16
0,00	3,75
0,00	5,16
0,10	2,09
0,37	1,48
0,00	1,74
0,00	14,81
0,00	7,10
0,00	3,30
0,00	0,00
0,00	0,72
0,24	1,90
0,00	1,55
0,00	6,25
0,00	2,27
0,00	0,00
0,00	1,99
0,00	2,51
0,00	3,88
0,00	0,67
0,17	1,22
0,00	1,77
0,16	1,72

0,61	2,30
2,17	1,09
0,00	0,00
2,47	2,06
2,07	2,07
6,08	2,03
6,90	6,90
0,00	7,89
0,00	1,05
1,27	0,00
0,00	1,11
0,00	7,08
0,84	1,68
0,00	0,00
0,00	7,21
4,26	1,37
4,61	9,21
0,00	2,72
0,23	2,11
0,52	1,29
0,00	2,72
0,00	0,00
0,00	2,12
1,05	6,32
0,00	0,00
0,00	3,39
0,25	6,00
0,00	1,59
0,00	5,56
0,00	1,76
0,00	1,74
0,00	4,10
0,00	0,00
2,30	1,64
0,91	2,72
4,64	0,90
4,32	1,64
4,15	2,30
5,71	2,60
5,59	2,27
5,51	3,24
4,44	0,79
3,92	1,22
5,40	1,40
4,14	1,98
2,91	8,09
1,09	1,71
0,00	2,42
0,34	3,69
0,00	2,01

0,00	1,99
0,00	0,00
0,00	3,57
0,00	0,00
0,00	1,61
0,00	2,38
0,00	1,14
0,00	2,70
0,00	7,99
0,00	6,25
0,00	1,76
0,00	1,49
0,00	4,38
0,00	0,86
0,00	3,27
0,00	2,65
0,00	0,73
0,00	0,00
0,00	8,86
0,43	8,62
0,97	2,91
0,57	2,29
0,79	1,58
1,14	1,14
1,28	3,85
0,30	8,08
0,18	4,39
1,06	5,16
0,00	2,12
0,00	2,02
0,43	4,93
0,00	0,00
0,00	0,00
0,00	0,00
0,00	2,50
0,12	4,22
0,00	0,00
0,00	2,11
0,00	0,00
0,00	2,71
3,57	3,79
6,14	0,88
0,00	0,00
0,00	0,00
0,51	5,13
0,00	1,16
0,00	3,71
0,00	3,16
0,00	3,83
0,00	2,66

0,00	1,64
0,00	3,49
0,11	1,32
0,00	2,68
0,00	10,00
0,00	3,18
0,00	0,00
0,00	5,26
0,00	9,00
0,00	1,56
0,00	0,00
0,00	0,00
0,39	5,10
0,00	0,00
0,45	1,80
0,47	6,07
0,00	9,18
0,00	9,46
0,00	7,54
0,00	0,71
0,00	3,33
0,00	1,57
0,00	5,60
0,00	0,00
0,00	0,00
0,00	6,33
3,57	0,00
0,42	2,54
0,46	1,83
0,00	1,85
0,79	1,98
1,00	2,40
2,08	1,27
1,12	0,91
0,38	2,52
6,30	0,81
6,72	0,52
4,24	12,24
0,00	0,00
0,80	4,12
0,00	2,59
0,00	4,87
0,00	3,84
1,66	1,28
0,00	0,00
1,48	5,76
0,15	2,38
0,00	1,54
0,09	7,63
0,00	1,52

0,16	6,43
0,13	2,05
0,00	4,24
5,59	0,65
3,91	1,96
5,33	5,83
1,66	0,83
0,00	1,62
0,85	1,02
0,60	2,53
1,01	0,00
2,84	2,42
2,11	2,41
1,06	1,97
0,40	0,64
0,58	4,28
1,82	0,00
1,25	1,11
0,92	1,73
0,95	2,66
0,36	1,97
0,17	3,13
0,00	4,61
0,48	1,32
0,36	1,99
0,00	2,70
0,30	1,93
0,00	0,00
0,59	0,89
0,00	1,07
1,12	1,86
1,19	5,19
1,16	1,39
0,59	2,96
0,00	8,65
0,00	5,96
0,15	4,57
0,18	0,70
0,00	1,93
0,00	1,85
0,00	1,76
0,00	2,42
0,00	2,91
0,22	1,67
0,00	1,10
0,00	5,20
0,00	2,38
0,00	1,57
0,00	8,97
0,32	1,46

0,25	5,81
0,00	7,68
0,00	2,93
0,00	1,26
0,00	1,42
0,00	7,09
0,00	0,00
2,05	0,36
0,39	2,22
0,05	7,47
0,90	0,75
2,31	1,32
5,23	0,62
4,26	1,37
0,85	2,55
2,60	2,84
9,47	1,00
4,76	0,00
0,00	2,67
3,01	1,06
1,68	1,85
0,74	2,83
1,48	1,80
2,52	0,46
2,05	1,57
0,94	3,51
1,62	1,47
2,29	2,44
1,49	1,49
0,00	2,94
0,00	5,83
0,00	4,00
0,00	0,00
0,64	3,61
0,00	4,31
0,25	2,13
0,43	2,71
0,22	2,37
0,00	2,27
0,00	2,38
0,00	4,72
0,00	6,00
0,00	3,67
0,00	3,79
0,00	2,42
0,00	1,52
0,00	1,84
0,00	7,69
5,24	2,80
1,90	0,57

1,87	4,20
0,00	3,13
0,53	1,58
0,00	2,95
6,02	0,69
0,00	2,59
3,11	1,94
1,22	0,87
1,83	4,59
2,61	2,43
2,19	1,31
2,84	2,23
1,29	1,93
1,50	2,06
1,52	0,65
1,47	1,14
1,48	0,42
2,46	3,20
2,07	0,83
3,50	8,11
1,99	1,00
0,15	1,98
0,59	2,23
1,93	1,38
1,55	1,69
0,29	2,91
1,76	2,82
1,07	1,07
0,54	1,18
0,37	0,00
0,00	0,00
0,55	1,64
0,00	0,00
1,73	0,87
2,04	3,06
2,02	1,52
3,47	0,69
1,70	1,13
1,01	2,20
3,26	2,17
0,67	5,82
0,31	1,71
0,24	1,92
0,36	8,03
0,00	1,85
0,00	1,17
1,43	4,29
1,48	3,83
2,91	5,83
3,50	0,97

0,65	2,29
0,81	2,27
0,32	2,43
0,98	0,98
3,78	1,36
2,74	7,95
0,00	5,67
0,28	6,38
0,17	1,56
0,00	3,47
0,00	0,00
0,78	1,24
0,00	1,64
0,00	2,50
0,00	1,85
0,00	0,97
0,00	0,90
0,00	1,10
0,00	5,66
0,00	1,37
0,00	4,84
0,00	11,61
0,46	0,76
1,13	0,68
0,66	1,10
0,77	0,77
0,78	3,70
0,46	2,30
1,45	1,45
3,05	0,95
1,59	1,45
0,48	1,19
1,64	1,84
0,81	1,63
0,00	4,27
0,12	0,73
0,00	2,05
0,00	1,12
0,00	1,22
0,12	2,17
0,00	2,25
10,00	0,00
0,44	3,96
1,31	4,13
0,00	0,00
0,00	1,64
0,00	1,33
0,00	0,00
1,02	1,28
1,04	5,92

0,00	0,00
1,09	1,09
1,74	0,58
5,56	0,00
1,06	5,87
1,20	2,01
0,13	2,46
1,56	1,42
0,82	3,27
2,74	0,85
0,96	6,44
1,57	1,57
1,66	0,30
1,02	0,00
1,14	0,57
0,63	1,05
0,36	4,46
0,00	0,00
0,00	3,54
0,00	3,33
1,41	0,71
0,00	10,53
0,86	1,72
0,00	0,00
0,00	5,81
0,00	1,45
0,00	2,43
0,98	1,22
0,00	0,00
0,00	0,00
0,00	1,14
0,00	0,00
0,17	1,74
0,18	1,09
0,17	1,91
0,35	2,45
0,06	5,16
0,12	4,54
0,00	0,52
0,00	1,47
0,00	2,34
0,00	4,72
0,00	3,06
0,00	1,71
0,00	0,83
0,00	0,85
0,00	2,21
0,00	2,95
0,00	2,63
0,00	1,68

0,00	2,59
0,00	10,45
0,13	10,55
0,00	1,71
0,14	2,09
0,00	1,82
0,31	1,41
0,20	1,18
0,00	2,98
0,00	1,40
0,19	2,25
0,00	3,29
0,12	2,30
0,00	2,77
0,00	0,84
0,00	1,68
0,00	3,30
0,00	3,48
0,00	4,18
0,00	2,26
0,00	2,63
0,00	9,42
0,00	10,13
0,56	2,48
0,12	8,09
0,00	7,67
0,00	2,18
0,21	2,34
0,00	0,90
0,18	1,43
0,13	1,75
0,00	0,00
0,11	4,63
0,10	0,84
0,00	1,40
0,12	1,30
0,22	1,12
0,00	1,57
0,00	3,03
0,00	1,60
0,00	1,70
0,12	0,62
0,00	2,51
0,00	3,52
0,00	4,96
0,00	1,85
0,00	0,37
0,00	13,04
0,00	1,39
0,00	1,57

0,00	1,60
0,00	5,91
0,00	9,34
0,00	1,97
0,00	1,38
0,00	1,16
0,00	12,39
0,00	2,00
0,10	1,13
0,00	2,91
0,00	8,61
0,00	2,39
0,00	3,46
0,22	9,63
0,00	0,00
0,00	3,10
0,00	2,22
0,00	3,78
0,00	0,00
0,00	1,83
0,43	1,72
0,25	0,99
0,00	1,69
0,10	3,78
0,00	0,90
0,21	3,14
0,80	1,27
0,10	1,08
0,19	0,56
0,00	1,46
0,17	3,13
0,13	2,03
0,00	1,24
0,00	3,96
0,12	3,48
0,00	1,85
0,00	4,35
0,10	1,80
0,09	2,35
0,00	2,86
0,00	1,22
0,00	3,90
0,00	1,85
0,10	2,51
0,00	0,00
0,00	0,85
0,00	2,41
0,00	2,41
0,00	3,79
0,00	1,75

0,00	9,09
0,00	11,05
0,00	0,80
0,00	2,51
0,00	9,24
0,24	1,57
0,00	9,39
0,00	2,28
0,12	6,77
0,17	1,00
0,30	9,33
0,14	1,25
0,00	6,66
0,32	4,01
0,33	0,83
0,00	1,51
0,53	0,83
0,00	0,00
0,41	2,76
0,13	1,00
0,16	1,13
1,10	0,92
1,84	0,50
0,62	1,87
0,47	7,11
0,00	4,78
0,00	5,76
0,00	1,08
0,08	1,62
0,11	2,16
0,00	3,91
0,00	2,36
0,00	9,57
0,00	3,38
0,00	8,70
0,00	3,24
0,00	3,35
0,00	2,59
0,00	2,24
0,00	1,30
0,09	2,69
0,00	7,62
0,15	1,54
0,00	1,98
0,00	1,70
0,00	9,91
0,00	2,16
0,00	9,05
0,00	0,82
0,00	9,07

0,00	9,64
0,00	0,76
0,14	1,51
0,00	0,00
0,00	1,11
0,00	1,19
0,00	3,04
0,00	2,67
0,00	2,11
0,00	4,09
0,11	0,57
0,00	0,87
0,00	1,36
0,00	8,06
0,00	4,75
0,00	8,81
0,00	2,87
0,00	11,47
0,00	3,67
0,00	2,81
0,00	0,65
0,00	9,64
0,00	4,55
0,19	2,78
0,00	3,21
0,00	4,68
0,00	1,18
0,00	1,60
0,00	9,06
0,00	2,12
0,00	9,91
0,00	1,47
0,00	5,46
0,00	0,61
0,00	1,33
0,00	3,91
0,00	2,00
0,00	1,21
0,00	1,59
0,00	1,89
0,00	3,94
0,49	0,97
0,00	1,78
0,12	0,87
0,00	7,09
0,00	2,19
0,00	1,61
0,10	1,72
0,00	1,95
0,00	11,87

0,00	2,33
0,00	1,47
0,00	7,28
0,22	2,00
0,00	2,81
0,00	2,60
0,00	0,94
0,00	7,22
0,00	5,61
0,00	1,17
0,00	2,84
0,00	3,64
0,00	0,00
0,00	0,93
0,00	2,31
0,00	7,88
0,00	1,74
0,00	0,71
0,00	2,00
0,00	0,75
0,00	1,58
0,00	7,12
0,00	1,64
0,00	1,83
0,14	1,54
0,00	8,41
0,13	1,04
0,00	1,49
0,00	2,80
0,00	5,90
0,11	4,91
0,19	4,38
0,00	1,59
0,09	1,79
0,10	2,63
0,00	8,60
0,53	1,06
0,00	1,25
0,00	0,86
0,10	3,53
0,77	3,07
0,00	0,00
0,15	1,49
0,00	2,18
0,00	2,71
0,00	2,10
0,00	1,58
0,00	4,09
0,00	11,79
0,00	2,98

0,10	1,80
0,00	12,11
0,00	3,14
0,00	6,98
0,00	2,43
0,00	1,84
0,00	2,06
0,10	1,14
0,00	3,89
0,00	2,81
0,00	2,63
0,00	0,52
0,00	3,33
0,00	3,12
0,00	4,41
0,00	4,70
0,15	1,34
0,00	4,51
0,00	0,93
0,11	3,99
0,00	1,37
0,00	4,23
0,00	3,75
1,04	1,96
0,00	1,57
0,12	1,09
0,00	7,29
0,00	3,31
0,00	2,12
0,00	2,30
0,15	1,20
0,00	6,82
0,00	5,21
0,00	3,31
0,00	2,30
0,00	1,76
0,00	2,51
0,17	1,67
0,00	3,20
0,00	3,82
0,00	4,38
0,19	3,76
0,00	5,03
0,00	3,23
0,00	0,00
0,00	0,00
0,00	3,42
0,00	5,33
0,00	9,95
0,00	0,00

0,30	2,66
0,00	2,63
0,00	2,97
0,00	2,80
0,00	0,47
0,00	4,20
0,00	1,84
0,00	0,63
0,00	3,10
0,00	13,47
0,00	0,00
0,00	4,36
0,00	5,52
0,00	2,87
0,00	9,32
0,00	8,69
0,00	3,48
0,00	2,09
0,00	3,01
0,00	2,42
0,00	3,20
0,00	1,03
0,00	1,68
0,10	1,41
0,00	4,54
0,00	5,75
0,00	4,47
0,00	3,31
0,91	1,81
0,00	1,86
0,60	4,17
0,00	1,26
0,00	1,61
0,00	6,14
0,00	7,35
0,00	3,75
0,23	2,03
0,00	1,63
0,00	3,37
0,00	1,16
0,00	3,72
0,00	4,95
0,00	1,46
0,00	1,95
0,00	7,93
0,00	6,00
0,00	8,20
0,00	2,00
0,00	7,72
0,00	2,50

0,00	10,16
0,00	9,21
0,00	5,52
0,00	7,69
0,00	1,92
0,00	0,46
0,00	0,00
0,00	5,48
0,00	3,31
0,00	0,00
0,00	1,12
0,00	4,74
0,00	3,39
0,00	1,19
0,00	1,29
0,00	1,35
0,00	1,59
0,00	10,17
0,00	2,19
0,00	2,57
0,00	2,53
0,00	2,21
0,00	2,68
0,00	3,35
0,00	7,58
0,00	2,29
0,00	2,40
0,16	6,13
0,00	2,85
0,60	7,83
0,00	4,18
0,00	3,72
0,00	0,65
0,00	6,95
0,00	1,55
0,00	4,04
0,00	1,42
0,00	0,00
0,00	1,79
0,00	0,00
0,00	0,88
0,00	0,76
0,00	2,29
0,00	1,00
0,00	2,99
0,00	4,98
0,00	3,12
0,00	0,00
0,00	1,63
0,00	1,07

0,00	0,00
0,00	1,25
0,00	4,98
0,00	1,72
0,00	1,49
0,00	1,81
0,00	13,72
0,00	1,40
0,00	2,16
0,00	1,97
0,28	7,63
0,00	16,70
0,00	10,21
0,00	1,62
0,00	0,00
0,00	2,18
0,00	3,84
0,00	1,15
0,00	1,51
0,00	2,38
0,00	4,47
0,00	1,96
0,00	2,86
0,00	3,73
0,00	8,43
0,00	8,36
0,18	1,47
0,00	3,90
0,00	7,03
0,00	7,14
0,00	1,98
0,00	2,12
0,00	4,37
0,00	3,62
0,18	3,73
0,00	3,18
0,00	1,97
0,10	1,88
0,00	5,26
0,00	1,40
0,00	2,79
0,00	0,71
0,00	8,27
0,00	2,35
0,00	3,35
0,00	2,37
0,00	1,17
0,00	1,39
0,00	7,93
0,00	1,50

0,00	1,85
0,08	5,63
0,00	2,88
0,00	4,77
0,00	10,49
0,00	2,35
0,00	1,75
0,00	2,27
0,00	1,41
0,00	2,62
0,08	1,42
0,00	1,84
0,00	1,52
0,00	3,28
0,00	1,69
0,00	2,50
0,00	5,15
0,00	2,41
0,00	2,15
0,00	1,24
0,00	2,02
0,00	1,82
0,14	2,00
0,00	5,33
0,13	3,08
0,00	2,18
0,11	1,37
0,11	9,16
0,00	3,35
0,11	0,67
0,00	9,53
0,00	7,17
0,18	1,59
0,25	2,99
0,14	0,85
0,11	0,43
0,00	2,81
0,00	1,99
0,00	2,68
0,21	1,15
0,00	2,60
0,00	7,92
0,00	7,16
0,15	6,24
0,00	2,94
0,00	1,63
0,32	0,95
0,00	2,04
0,00	1,22
0,27	1,50

0,00	0,00
0,00	1,87
0,00	2,60
0,08	2,23
0,19	3,36
0,00	4,90
0,00	2,35
0,00	2,02
0,00	1,34
0,00	1,42
0,00	5,53
0,00	0,00
0,00	12,58
0,00	8,39
0,00	16,78
0,00	1,50
0,00	5,15
0,00	2,60
0,00	2,25
0,00	2,79
0,00	2,48
0,00	2,60
0,00	1,23
0,00	5,28
0,00	5,65
0,15	6,07
0,00	5,24
0,00	0,00
0,00	1,98
0,00	0,00
0,00	8,44
0,45	0,45
0,00	2,03
0,00	1,46
0,00	7,71
0,00	3,02
0,00	3,13
0,00	9,25
0,00	9,43
0,00	7,14
0,11	1,82
0,00	10,56
0,00	1,63
0,00	1,68
0,00	0,69
0,00	1,23
0,00	2,71
0,00	5,02
0,00	2,02
0,00	6,85

0,00	1,82
0,00	17,39
0,13	17,13
0,00	1,12
0,00	0,00
0,00	0,00
0,99	1,98
0,00	5,24
0,00	2,86
0,00	1,49
0,00	0,85
0,00	1,33
0,00	1,35
0,00	1,54
0,23	2,55
0,00	2,48
0,00	3,42
0,00	3,58
0,00	0,00
0,00	2,54
0,00	1,82
0,00	8,16
0,23	4,13
0,00	2,50
0,00	0,85
0,00	3,04
0,20	2,93
0,00	4,27
0,00	2,02
0,00	0,70
0,00	0,72
0,00	6,19
0,00	5,15
0,00	0,00
0,00	2,18
0,00	2,94
0,00	1,96
0,00	6,67
0,00	2,08
0,16	1,71
0,00	1,85
0,00	7,55
0,00	0,46
0,00	1,95
0,00	0,89
0,00	1,73
0,00	0,00
0,00	3,17
0,00	3,12
0,00	2,21

0,00	2,38
0,14	1,23
0,00	1,96
0,00	1,27
0,00	8,91
0,00	3,44
0,00	2,94
0,13	1,72
0,00	2,20
0,00	2,44
0,00	3,28
0,00	2,78
0,00	2,08
0,00	4,03
0,00	2,24
0,00	3,40
0,00	2,10
0,16	2,35
0,00	0,98
0,00	1,74
0,26	2,29
0,00	1,61
0,15	5,35
0,14	1,39
0,00	2,04
0,52	0,83
0,00	2,67
0,00	2,57
0,00	1,86
0,00	8,04
0,00	1,40
0,12	1,81
0,00	2,34
0,15	3,06
0,00	2,03
0,00	5,35
0,00	3,48
0,00	3,74
0,00	0,00
0,08	1,89
0,11	0,98
0,00	1,32
0,00	1,32
0,18	1,29
0,11	1,57
0,00	2,20
0,00	1,51
0,12	1,73
0,22	1,96
0,61	2,97

0,44	1,66
0,34	1,84
0,36	1,42
0,00	0,42
0,00	2,03
0,25	2,86
0,00	4,01
0,00	0,71
0,00	1,85
0,00	1,52
0,09	3,50
0,00	6,53
0,00	6,87
0,00	11,14
0,00	1,67
0,00	2,77
0,09	0,97
0,00	3,53
0,11	2,18
0,00	4,51
0,00	3,26
0,00	2,38
0,13	3,20
0,28	1,57
0,08	1,18
0,10	2,71
0,32	0,96
0,27	1,46
0,00	9,81
0,00	1,47
0,00	5,89
0,00	1,89
0,00	3,00
0,00	1,21
0,00	6,12
0,00	2,00
0,00	4,23
0,00	11,27
0,00	1,52
0,00	1,47
0,14	1,56
0,00	1,95
0,00	2,10
0,00	7,47
0,00	2,15
0,00	6,07
0,00	2,39
0,00	9,91
0,00	2,46
0,10	1,69

0,10	1,36
0,00	1,53
0,10	1,72
0,09	11,92
0,00	10,28
0,00	2,30
0,25	2,04
0,08	3,32
0,00	0,00
0,00	1,65
0,00	2,91
0,09	2,03
0,00	1,32
0,00	0,96
0,00	0,90
0,00	1,91
0,00	1,45
0,00	2,48
0,10	1,76
0,00	3,49
0,00	3,62
0,11	1,53
0,00	8,65
0,00	1,36
0,00	2,07
0,13	2,63
0,00	1,97
0,00	5,59
0,00	4,79
0,00	2,84
0,00	8,25
0,00	2,58
0,00	1,53
0,00	1,19
0,51	2,17
0,00	7,30
0,00	2,22
0,21	2,57
0,00	3,39
0,17	1,04
0,00	1,63
0,00	3,51
0,00	2,38
0,00	6,14
0,00	1,38
0,77	1,93
0,83	0,00
0,14	1,27
0,00	1,74
0,20	1,01

0,12	1,87
0,00	1,95
0,00	1,97
0,00	2,39
0,00	2,78
0,12	3,34
0,00	0,79
0,25	0,50
0,34	0,69
0,20	2,01
0,70	1,96
0,92	0,92
0,30	2,53
1,11	2,07
0,63	1,72
0,00	1,41
2,36	0,24
0,15	7,87
0,87	1,09
1,82	0,77
0,60	2,84
1,05	0,79
0,36	1,68
0,12	7,44
0,49	2,43
0,00	1,83
0,42	1,25
0,47	0,00
0,24	0,84
0,15	1,68
0,00	1,60
0,00	3,08
0,00	1,89
0,12	2,30
0,00	2,08
0,00	3,88
0,00	5,95
0,00	1,68
0,00	3,28
0,13	0,90
0,00	2,71
0,09	2,66
0,23	1,07
0,00	1,00
0,00	7,71
0,00	0,61
0,00	1,85
0,10	2,17
0,00	1,72
0,00	3,72

0,12	2,39
0,00	7,60
0,00	5,99
0,00	3,35
0,00	3,52
0,68	7,53
0,00	1,04
0,00	0,00
0,00	3,09
0,00	1,51
0,00	14,20
0,00	7,51
0,00	13,56
0,00	1,46
0,00	1,46
0,55	4,70
0,40	2,40
0,00	1,67
0,00	0,64
0,00	0,00
0,28	3,07
0,00	4,59
0,00	2,34
0,00	2,64
0,00	5,46
0,00	1,64
0,00	2,42
0,00	4,86
0,00	0,63
0,00	8,24
0,00	7,69
0,00	0,40
0,00	1,99
0,00	4,19
0,00	4,72
0,00	4,15
0,00	3,01
0,00	2,32
0,00	0,69
0,00	3,06
0,00	2,74
0,00	1,52
0,00	1,92
0,00	0,82
0,00	7,95
0,00	0,00
0,00	0,00
0,00	0,79
0,52	1,57
0,00	4,76

0,12	4,14
0,00	3,42
0,08	4,11
0,00	8,49
0,00	6,94
0,00	3,30
0,00	1,99
0,00	1,32
0,92	5,50
0,00	7,30
0,00	5,33
0,00	2,07
0,00	1,51
0,76	1,53
0,57	1,14
0,00	1,40
0,00	3,20
0,00	1,36
0,00	2,78
0,00	1,17
0,00	3,04
0,00	2,79
0,00	5,42
0,00	1,39
0,00	5,08
0,00	2,91
0,38	4,15
0,00	4,42
0,00	0,98
0,00	2,08
0,00	0,85
0,00	2,54
0,00	2,05
0,00	1,97
0,00	2,69
0,22	6,33
0,00	3,47
0,00	7,03
0,00	1,33
0,00	2,82
0,00	1,47
0,00	2,92
0,94	8,02
0,00	1,41
0,00	2,59
0,00	0,68
0,00	5,35
0,00	0,48
0,00	2,28
0,14	3,23

0,00	3,28
0,12	4,24
0,32	5,59
0,00	1,55
0,00	3,26
0,00	0,92
0,00	2,27
0,00	1,50
0,00	11,65
0,00	2,96
0,00	8,11
0,00	3,01
0,00	1,56
0,00	7,89
0,00	5,13
0,00	6,10
0,00	1,08
0,00	1,45
0,00	2,09
0,39	10,68
0,00	3,33
0,00	1,90
0,00	3,67
0,00	2,96
0,00	5,96
0,00	1,87
0,00	2,86
0,00	2,71
0,00	3,29
0,99	6,94
0,00	2,23
0,58	2,61
0,30	3,19
0,00	3,13
0,00	4,37
0,00	8,98
0,00	9,16
0,00	1,47
0,00	0,39
0,00	0,96
0,00	0,98
0,00	1,13
0,00	1,25
0,00	4,76
0,00	0,00
0,00	2,54
0,00	1,33
0,88	2,63
0,00	0,00
0,00	3,76

0,19	9,26
0,00	0,00
1,04	5,74
1,31	0,65
1,43	1,52
0,77	2,82
0,51	1,23
0,20	4,61
0,49	1,81
0,74	1,85
1,12	1,91
2,06	3,49
1,37	5,82
0,48	4,83
0,70	1,64
0,40	1,21
0,21	1,25
0,45	4,04
0,59	2,12
0,52	1,55
0,36	2,52
0,30	1,67
0,60	0,72
0,00	1,82
0,16	1,79
0,00	1,29
0,16	5,76
1,22	2,20
1,94	5,81
0,45	8,35
0,91	1,82
1,26	1,83
0,98	0,86
1,53	0,24
0,24	6,09
0,00	5,08
0,98	1,85
0,50	1,49
1,02	4,81
0,00	0,00
0,89	1,63
0,67	3,19
1,76	5,28
0,00	2,95
0,36	1,25
1,41	4,43
0,68	2,51
2,24	4,48
1,55	3,22
1,17	2,34

0,00	24,08
0,98	1,41
1,65	0,78
0,77	1,54
1,29	1,80
0,00	1,21
0,00	1,55
0,98	0,74
0,00	0,00
0,00	0,72
0,67	1,77
0,58	0,77
1,01	3,03
0,28	7,89
0,16	3,45
0,00	5,91
0,00	6,25
0,13	2,86
0,00	1,65
0,00	13,61
0,10	2,21
0,12	3,55
0,13	1,72
0,00	3,33
0,00	1,28
0,00	3,54
0,00	4,09
0,00	3,37
0,00	2,35
0,00	5,53
0,16	4,09
0,09	7,21
0,23	1,61
0,13	7,72
0,00	1,23
0,00	5,75
0,00	1,17
0,00	2,06
0,10	1,28
0,00	1,39
0,40	2,46
0,00	0,91
0,00	2,04
0,00	4,36
0,00	1,59
0,00	1,17
0,00	5,55
0,00	0,16
0,00	3,23
0,31	13,50

0,00	2,53
0,00	1,49
0,00	0,76
0,00	1,99
0,00	0,98
0,00	2,23
0,00	1,41
0,00	2,13
0,19	1,33
0,00	0,98
0,09	2,32
0,00	1,80
0,00	1,64
0,11	2,76
0,16	1,89
0,00	0,48
0,00	2,00
0,00	3,37
0,00	2,46
0,35	5,70
0,27	1,99
0,70	1,09
0,61	2,54
0,00	2,05
0,00	5,43
0,21	4,08
0,28	1,20
0,16	1,56
0,18	1,14
0,00	5,61
0,00	1,33
0,00	5,49
0,00	5,60
0,00	2,63
0,35	3,65
0,00	1,80
0,00	3,07
0,00	2,14
0,40	2,80
0,00	1,10
0,00	2,61
0,10	5,60
0,00	5,07
0,00	4,11
0,00	3,74
0,00	6,51
0,00	0,90
0,10	1,66
0,00	1,17
0,00	0,92

0,00	3,08
0,00	1,66
0,00	6,94
0,00	6,87
0,00	15,54
0,00	13,91
0,00	9,29
0,00	2,61
0,21	1,68
0,12	2,68
0,17	3,10
0,00	0,92
0,00	2,40
0,00	1,79
0,00	1,16
0,00	7,61
0,00	14,08
0,11	1,36
0,00	4,85
0,00	1,08
0,00	2,88
0,00	3,12
0,00	1,81
0,00	2,80
0,00	5,71
0,00	1,37
0,00	1,16
0,00	2,11
0,28	2,25
0,00	2,35
0,19	10,43
0,70	1,54
0,00	4,70
0,11	5,21
0,00	8,11
0,00	3,42
0,00	3,75
0,20	4,99
0,69	1,92
0,00	3,20
0,00	5,64
0,00	8,52
0,00	0,92
0,00	7,46
0,00	1,49
0,00	1,84
0,08	2,03
0,00	2,07
0,31	0,77
0,09	1,39

0,30	1,42
0,00	1,89
0,10	2,46
0,00	1,34
0,00	7,40
0,00	7,19
0,15	1,50
0,00	0,99
0,08	2,38
0,00	1,17
0,00	7,72
0,15	2,42
0,13	3,19
0,00	1,56
0,00	3,77
0,14	4,38
0,00	3,90
0,11	2,55
0,21	1,91
0,27	1,62
0,00	8,66
0,00	6,23
0,00	1,96
0,00	1,43
0,13	2,24
0,08	1,56
0,00	1,73
0,00	1,71
0,00	5,38
0,00	6,10
0,00	8,80
0,10	3,54
0,08	0,76
0,00	4,36
0,62	6,17
0,00	12,72
0,00	2,12
0,00	1,26
0,00	3,49
0,00	9,87
0,00	1,02
0,12	1,94
0,00	1,21
0,00	1,75
0,00	3,25
0,00	1,99
0,00	1,81
0,00	1,37
0,00	3,27
0,00	10,58

0,00	7,16
0,00	8,42
0,00	11,37
0,00	2,06
0,00	7,89
0,00	3,57
0,00	1,52
0,00	2,30
0,22	4,04
0,00	1,27
0,00	1,18
0,07	2,12
0,14	1,67
0,00	3,25
0,00	9,04
0,00	5,53
0,00	4,58
0,00	6,53
0,00	0,94
0,00	0,96
0,00	9,62
0,00	1,02
0,10	4,72
0,00	1,93
0,00	4,06
0,00	7,31
0,00	2,45
0,00	0,00
0,00	12,76
0,10	1,79
0,00	6,63
0,00	3,94
0,00	1,42
0,00	2,84
0,00	3,01
0,00	1,09
0,00	4,72
0,00	5,53
0,30	2,18
0,25	4,29
0,65	3,26
0,00	0,00
0,00	2,58
0,13	1,03
0,32	2,71
0,18	12,07
0,00	1,98
0,52	1,74
0,10	0,93
0,13	2,13

0,63	0,63
0,00	1,57
0,00	0,38
0,00	1,54
0,00	1,03
0,00	1,86
0,00	1,03
0,00	3,64
0,00	2,62
0,00	0,78
0,00	0,00
0,00	1,84
0,11	1,12
0,00	9,85
0,00	7,69
0,00	2,56
0,00	6,40
0,00	2,66
0,00	1,85
0,00	1,90
0,00	8,35
0,00	1,03
0,00	7,94
0,00	1,18
0,00	1,10
0,35	1,50
0,00	3,54
0,00	2,65
0,00	1,85
0,00	7,62
0,20	12,22
0,00	9,79
0,00	2,89
0,00	1,79
0,00	4,96
0,00	0,81
0,00	1,11
0,00	1,25
0,00	6,53
0,00	7,29
0,00	8,75
0,00	2,00
0,00	5,07
0,00	5,88
0,00	2,43
0,00	1,61
0,00	2,30
0,00	1,19
0,00	4,44
0,00	0,70

0,00	5,92
0,00	4,27
0,00	2,19
0,00	3,79
0,00	1,33
0,00	1,15
0,00	4,73
0,00	3,43
0,00	7,63
0,00	2,40
0,00	5,77
0,00	1,26
0,00	1,49
0,00	2,25
0,20	2,17
0,00	2,81
0,00	6,72
0,00	2,00
0,00	6,64
0,00	2,33
0,00	8,21
0,00	0,88
0,00	2,11
0,00	4,97
0,00	0,98
0,00	2,03
0,00	4,10
0,11	2,35
0,00	1,15
0,00	2,63
0,00	3,34
0,20	0,59
0,00	1,64
0,00	4,17
0,72	0,96
0,13	0,92
0,22	1,84
0,00	3,34
0,00	2,27
0,00	1,42
0,00	1,59
0,00	1,79
0,00	1,08
0,00	1,48
0,00	2,38
0,00	8,61
0,27	1,50
0,00	2,82
0,00	12,65
0,20	1,00

0,20	1,50
0,00	3,53
0,00	2,86
0,00	0,95
0,12	4,54
0,00	6,29
0,00	1,94
0,00	4,20
0,00	1,01
0,00	4,84
0,00	2,00
0,00	4,55
0,00	0,00
0,00	0,00
0,00	4,05
0,00	1,29
0,00	0,00
0,00	1,43
0,00	0,00
0,44	2,18
0,00	0,80
0,00	6,67
0,00	1,14
0,00	8,11
0,00	3,57
0,00	2,13
0,45	1,36
0,00	6,45
0,00	2,33
0,00	1,18
0,00	9,78
0,00	0,00
0,00	2,80
0,00	19,44
0,00	0,37
0,00	9,52
0,00	13,33
